# Microsomal membrane proteome of low grade diffuse astrocytomas: Differentially expressed proteins and candidate surveillance biomarkers

**DOI:** 10.1038/srep26882

**Published:** 2016-06-01

**Authors:** Ravindra Varma Polisetty, Poonam Gautam, Manoj Kumar Gupta, Rakesh Sharma, Harsha Gowda, Durairaj Renu, Bhadravathi Marigowda Shivakumar, Akhila Lakshmikantha, Kiran Mariswamappa, Praveen Ankathi, Aniruddh K. Purohit, Megha S. Uppin, Challa Sundaram, Ravi Sirdeshmukh

**Affiliations:** 1 Centre for Cellular and Molecular Biology (CSIR), Hyderabad, India; 2 Institute of Bioinformatics, Bangalore, India; 3 Manipal University, Madhav Nagar, Manipal, India; 4 Strand Life Sciences, Bangalore, India; 5 Neuro-Oncology, Mazumdar Shaw Center for Translational Research, Narayana Health, Bangalore, India; 6 Mazumdar Shaw Medical Center, Narayana Health, Bangalore, India; 7 Nizam’s Institute of Medical Sciences (NIMS), Hyderabad, India

## Abstract

Diffuse astrocytoma (DA; WHO grade II) is a low-grade, primary brain neoplasm with high potential of recurrence as higher grade malignant form. We have analyzed differentially expressed membrane proteins from these tumors, using high-resolution mass spectrometry. A total of 2803 proteins were identified, 340 of them differentially expressed with minimum of 2 fold change and based on ≥2 unique peptides. Bioinformatics analysis of this dataset also revealed important molecular networks and pathways relevant to tumorigenesis, mTOR signaling pathway being a major pathway identified. Comparison of 340 differentially expressed proteins with the transcript data from Grade II diffuse astrocytomas reported earlier, revealed about 190 of the proteins correlate in their trends in expression. Considering progressive and recurrent nature of these tumors, we have mapped the differentially expressed proteins for their secretory potential, integrated the resulting list with similar list of proteins from anaplastic astrocytoma (WHO Grade III) tumors and provide a panel of proteins along with their proteotypic peptides, as a resource that would be useful for investigation as circulatory plasma markers for post-treatment surveillance of DA patients.

Diffuse astrocytoma (WHO grade II) is low-grade primary brain tumor of astrocytes. It is characterized by slow growth with low probability of infiltration into neighboring brain tissue. Though relatively rare^1^, it represents 10% of all astrocytic brain tumors with the mean survival time of 6–8 years^2^,^3^,^4^. It typically affects young adults, the standard method for diagnosis is based on histology and treatment includes surgery followed by radiotherapy. The tumors have an inherent potential of progression to malignant anaplastic astrocytoma (WHO Grade III) or secondary glioblastoma (GBM) over time^5^. The most common genetic alteration in diffuse astrocytoma is mutations of the TP53 and IDH1/2 genes in 32% cases, 1p/19q loss and IDH1/2 mutation in 37% cases and only IDH1/2 mutation in 17% cases^6^. Promoter hypermethylation of the DNA repair gene O-6-methylguanine-DNAmethyltransferase (MGMT) and the protocadherin-gamma subfamily A11 (PCDH-gamma-A11) are some of the epigenetic alterations^7^,^8^ reported for these tumors. Several differential gene expression studies have been carried out to understand pathogenesis or to distinguish primary and recurrent grade II tumors or to differentiate them from higher grade tumors^9^,^10^,^11^. Malzkorn *et al*. studied profiling of 157 microRNAs in four patients with grade II gliomas that spontaneously progressed to WHO grade IV secondary glioblastomas and showed possible role of 20 microRNAs (18-overexpressed and 2 repressed) in glioma progression^12^. Proteomics studies on these tumors have been, however, on the lower side. Earlier studies on differential protein expression of low grade and high grade gliomas were carried out using 2D-MS approach^13^,^14^. Iwadate *et al*. tried to classify the tumors for survival prediction based on expression patterns^13^. Recently, Gimenez *et al*. performed high throughput quantitative proteomic analysis of low grade and high grade astrocytomas and oligodendrogliomas^15^. They identified RNA binding protein NOVA-1 (NOVA1) to be a marker distinguishing astrocytoma with oligodendrogliomas and heat shock protein beta 1 (HSPB1) as a predictive marker for poor prognosis for GBM^15^. Using protein arrays, Jiang *et al*. studied the expression and phosphorylation status of 46 proteins involved in signaling pathways associated with cell proliferation, cell survival, apoptosis, angiogenesis, and cell invasion in lower grades of glioma^16^. The Cancer Genome Atlas (TCGA) group has recently carried out a large scale molecular profiling of diffuse gliomas using 1,122 samples. Some major pathways implicated include PI3K/mToR pathway along with Ras-Raf MEK-ERK, p53/apoptosis pathway and others. Similarly, they confirmed cohesin complex pathway, involved in cell division and telomere length regulation, to play a major role in gliomagenesis. Further, based on unsupervised clustering of protein profiles, TCGA analysis also revealed two macro clusters, one cluster (LGG cluster) with majorly lower grade (Gr II and Gr III) glioma samples while other cluster, GBM-like cluster, with mostly GBM samples. The LGG class showed increased activity of PKC, PTEN, BRAF, and phosphoP70S6K^17^.

In the present study, we have analyzed protein expression changes in the microsomal fraction of clinical tissue samples with diffuse astrocytoma in comparison to control, using iTRAQ and high-resolution mass spectrometry, followed by extensive bioinformatics analysis to get further insights into molecular changes in these tumors and to generate a resource which could be useful for developing circulatory biomarkers for clinical applications such as post-treatment surveillance.

## Experimental procedures

### Sample collection and processing

All the samples were collected at the time of surgery with informed consent from patients and approval of the Institutional Ethics Committee, Nizam’s Institute of Medical Sciences (NIMS), Hyderabad, India and all the experiments were performed in accordance with recommended guidelines and regulations. Tumor tissue specimens were snap frozen in liquid nitrogen and stored at −80 °C until use. Multiple sections from the temporal neocortex were studied and the tumor grade was assigned on the basis of clinical evaluation and histopathology as per WHO guidelines. Out of forty-five astrocytoma specimens collected over the period of 2 years, nine of them were grouped as diffuse astrocytomas. Six age matched samples (20–40 years) of either sex were selected for present study. Brain tissue obtained from temporal lobe epilepsy surgeries were collected as experimental controls. A large amount of temporal cortical tissue needs to be removed in these surgeries to reach hippocampus which is usually the most likely seizure focus. The temporal cortex used as control did not show any histological abnormalities by light microscopy. Further, immunohistochemistry (IHC) with antibodies directed against phosphorylated neurofilament and synaptophysin proteins did not reveal any abnormal neurons in the cortex. These control subjects were in the 20–30 year old age group.

### Sub cellular fractionation for enrichment of microsomal proteins

Tissues from tumor patients (n = 6; 4 males and 2 females) or control subjects (n = 3; 2 males and 1 female) were pooled separately and microsomal fraction was prepared according to the procedure of Cox *et al*.^18^ and described by us earlier^19^ for microsomal protein enrichment. The procedure yields a preparation, which consists of membrane proteins of ER, golgi, intracellular vesicles and plasma membrane. Protein amount in the preparation was estimated using Bradford method.

### Sample processing for iTRAQ labeling and SCX fractionation

Microsomal protein fraction from tumor or control tissues was subjected to trypsin digestion and the peptides were labelled with iTRAQ reagents according to the manufacturer’s instructions (iTRAQ Reagents Multiplex kit; Applied Biosystems/MDS Sciex, Foster City, CA) and as described previously^19^. Tumor tissue samples were labelled with 116 and 117 tags and control samples with 114 and 115 tags. All the four labelled peptide samples were pooled, vacuum-dried and subjected to strong cation exchange (SCX) chromatography as also described previously^19^. Peptides eluting from the column were collected and consecutive fractions were pooled to obtain a total of eight fractions. These fractions were desalted using C18 cartridge (Pierce, Rockford, USA) as per the manufacturer’s instructions for LC-MS/MS analysis.

### LC-MS/MS analysis

Nanoflow electrospray ionization tandem mass spectrometric analysis was carried out using LTQ Orbitrap Velos (Thermo Scientific, Bremen, Germany) interfaced with Agilent’s 1200 Series nanoflow LC system. Peptides from each SCX fraction were enriched using a C18 trap column (75 μm × 2 cm) at a flow rate of 3 μl/min and fractionated on an analytical column (75 μm × 10 cm) at a flow rate of 350 nl/min using a linear gradient of 7–30% acetonitrile (ACN) over 65 min. Mass spectrometric analysis was performed in a data dependent manner using the Orbitrap mass analyzer at a mass resolution of 60,000 at m/z 400. For each MS cycle, twenty top most intense precursor ions were selected and subjected to MS/MS fragmentation and detected at a mass resolution of 15,000 at m/z 400. The fragmentation was carried out using higher-energy collision dissociation (HCD) mode. Collision energy (CE) between 39–42% was used for optimization and normalized CE of 40% was used to obtain release of reporter ions from all peptides detected in the full scan. The ions selected for fragmentation were excluded for next 30 sec. The automatic gain control for full FT MS and FT MS/MS was set to 1 million ions and 0.1 million ions respectively with a maximum time of accumulation of 500 ms. The lock mass option was enabled for accurate mass measurements.

### Bioinformatics analysis

Protein identification, quantification and annotations of differentially expressed proteins were carried out as follows. The MS/MS data was analyzed using Proteome Discoverer (Thermo Fisher Scientific, version 1.4) in Sequest mode using NCBI RefSeq database (release 52) containing 33,985 proteins. Search parameters included trypsin as the enzyme with 1 missed cleavage allowed; precursor and fragment mass tolerance were set to 20 ppm (around 97% of the peptides detected conformed to <10 ppm mass error) and 0.1Da, respectively; Methionine oxidation was set as a dynamic modification while methylthio modification at cysteine and iTRAQ modification at N-terminus of the peptide and lysines were set as static modifications. The peptide and protein information were extracted using high peptide confidence and top one peptide rank filters. The FDR was calculated by enabling the peptide sequence analysis using a decoy database. High confidence peptide identifications were obtained by setting a target FDR threshold of 1% at the peptide level. Mass spectrometric analysis resulted in identification of a total of 20,783 peptides. After removing peptides not labelled with all the four labels (n = 212) and those (n = 1968) shared between multiple proteins, 18,603 peptides were considered for identification of proteins. The labelling efficiency was thus 99%.

Relative quantitation of proteins was carried out based on the intensities of reporter ions released during MS/MS fragmentation of peptides. The average relative intensities of the two reporter ions for each of the unique peptide identifiers for a protein were used to determine relative quantity of a protein and percentage variability. Appropriate filters at the level of peptides/peptide spectral matches (PSMs) and then at the protein level were applied to the quantification values as described in earlier publication^20^. In brief, Only PSMs that are ‘unique’ for a protein were included for fold change calculation. Next, PSMs with more than 30% co-efficient of variation (% CV) between the replicate label measurements (i.e., 114 and 115 for control) and (i.e., 116 and 117 for tumor) were removed programmatically. We then extracted PSMs corresponding to proteins with 1.5 fold change, applied 1.5 fold cut off to these subset of PSMs and recomputed fold change for proteins. Further filters were applied at protein level to select proteins with minimum 2 unique peptides and 2-fold expression change, with PSM quant ratio variability (% CV) of less than 40%. The median pair-wise quant ratio for 116/114, 116/115, 117/114, and 117/115 was used to compute the statistical significance (p-value < 0.05). The Benjamini Hochberg FDR corrected p-value is included in Supplementary Table S1 for proteins that were differential at 2-fold-change or above.

Gene Ontology annotations of the proteins identified were carried out based on Human Protein Reference Database (HPRD, http://www.hprd.org)^21^. Mapping of molecular functions and pathways was done using the Ingenuity Pathway Knowledge Base (Ingenuity Systems, Redwood City, CA) tool. Proteins containing signal peptide and transmembrane domains were identified using SignalP 4.1 and TMHMM 2.0 software tools. Exocarta database was used to map the human exosomal proteins^22^.

### Immunohistochemistry (IHC)

The expression level of four of the select proteins, epidermal growth factor receptor (EGFR), brevican core protein (BCAN), ectonucleotide pyrophosphatase/phosphodiesterase family member 6 (ENPP6) and heterogeneous nuclear ribonucleoprotein (HNRNP) K were studied by immunohistochemistry using commercially available Tissue microarray containing 13 Diffuse Astrocytomas cases and 4 control tissue cores (US BioMax). In brief, after deparaffinization and rehydration of formalin-fixed paraffin-embedded tumor tissue sections, antigen retrieval was performed by immersing the slide in antigen retrieval buffer (10 mM sodium citrate, 0.05% Tween 20, pH 6.0) at 95 °C for 5 min. Endogenous peroxidases were blocked with 0.03% hydrogen peroxide, and nonspecific binding was blocked with 2% fetal calf serum in Tris-buffered saline with 0.1% Triton X-100 (TBST, pH 7.6). Sections were then incubated for 1 h at RT with EGFR (dilution 1:100; Cat No. HPA018530), BCAN (dilution-1:200; Cat No. HPA007865), ENPP6 (dilution-1:10; Cat No. HPA042740) and HNRNP K (dilution-1:250; Cat No. HPA007644) primary antibodies (Atlas Antibodies, Sigma) followed by peroxidase-labelled polymer conjugate to anti-rabbit or anti-mouse immunoglobulins compatible with the primary antibody, for 1 h and developed with DAB system (DAKO, Denmark). Sections were counter stained with the Mayer’s hematoxylin, dehydrated and images were taken under microscope.

## Results and Discussion

### Identification of differentially expressed proteins

DAs are low incidence tumors, yet important as they mostly occur in younger age group individuals with a high chance of recurrence and significantly long median survival time. Presently the general treatment modality is surgery followed by radiation, with mixed outcome. Better treatment strategies as well as post treatment surveillance are important unmet clinical needs. With this focus, we have studied differentially regulated proteins from the microsomal fraction from clinical tissues to understand molecular changes underlying DA and to identify proteins that may have strong secretory potential for application as post treatment surveillance markers. Considering low incidence of these tumors and sample paucity, our experimental approach has been to carry out quantitative LC-MS/MS analysis using iTRAQ, on microsomal fraction purified from pooled tissue biopsies from patients diagnosed with DA, followed by cross-comparison with transcript data from individual patient samples and/or verification of the functionally significant members by immunohistochemistry on tissue microarrays with individual samples. We also screened the proteins from the dataset applying bioinformatics for their secretory potential and identified a set of proteins that may serve as candidates for investigation towards application for post-treatment surveillance. Thus the study represents discovery-stage findings that could be used by us and others for clinical validations.

**Figure 1:**
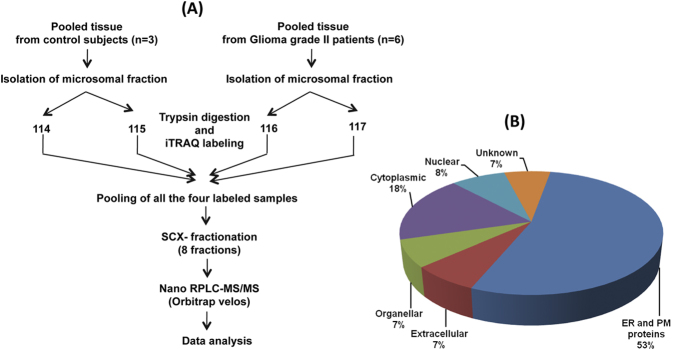
(**A**) Overall workflow for quantitative proteomic analysis of the tumor samples. Details of preparation of microsomal membrane proteins, iTRAQ labeling, LC-MS/MS analysis and protein identifications are provided under Methods. (**B**) Subcellular classification of differentially expressed proteins. Subcellular classification of differentially expressed proteins (n = 340) was carried out using Human Protein Reference Database and shows the enrichment of the membrane proteins.

A pool of biopsies from six male and female patients between 20–40 years of age group was used to prepare the microsomal fraction containing endoplasmic reticulum, golgi, intracellular vesicles, and plasma membrane proteins. This was analyzed to identify differentially expressed proteins using iTRAQ labeling of tryptic peptides followed by LC-MS/MS analysis using LTQ Orbitrap Velos mass spectrometer. Microsomal fraction from a pool of temporal lobe epilepsy surgery specimens was used as control. The workflow of the analysis is given in Fig. 1A.

A total of 18,603 iTRAQ labelled peptides was identified which mapped to 2803 proteins, majority of them with multiple peptides. A total of 340 proteins were found to be differentially expressed with at least 2-fold change (218 upregulated and 122 down regulated proteins). The altered levels of each of the identified proteins were based on at least two peptides with two reporter ions for each peptide. We have identified and quantified 84 proteins with 2 peptides, 73 with 3 peptides and remaining 183 proteins with 4 or more peptides. For averaging the quantities of the proteins, we used only unique peptides identifying a protein with variability of less than 40% in the peptide ratio. Subcellular classification of the 340 differentially expressed proteins using Gene Ontology information from Human Protein Reference Database (HPRD) revealed majority (53%) of them as proteins known to be associated with the endoplasmic reticulum and plasma membrane (Fig. 1B). Supplementary Table S1 provides the list of these proteins along with their peptide information, quantitative levels, molecular or biological functions and cellular localizations.

**Figure 2:**
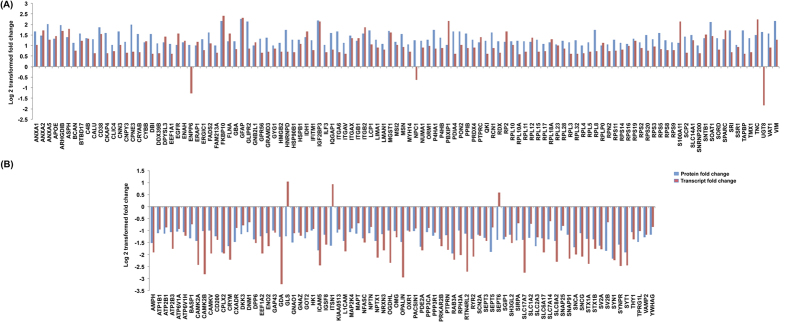
Comparison of differentially expressed proteins observed DA with differential expression reported at transcript levels. The total number of differentially expressed proteins observed in the present study was compared with differentially expressed transcript data available in Oncomine resource (www.oncomine.org, ref. 11). (**A**) shows profiles of upregulated entities and (**B**) represents downregulated entities. The fold change values of these entities are given in Supplementary Table S2.

Comparison of 340 differentially expressed proteins with the differentially expressed transcript data (≥1.5 fold change) by Sun *et al*.^11^ and accessed using Oncomine data resource (www.oncomine.org) in DA tumors revealed a total of 195 proteins (57.4%) to be common (Supplementary Table S2). Of these, 189 proteins showed positive correlation in expression supporting our observations and the proteomic data. The comparative differential protein and transcript expression in fold changes are shown in Fig. 2.

**Figure 3:**
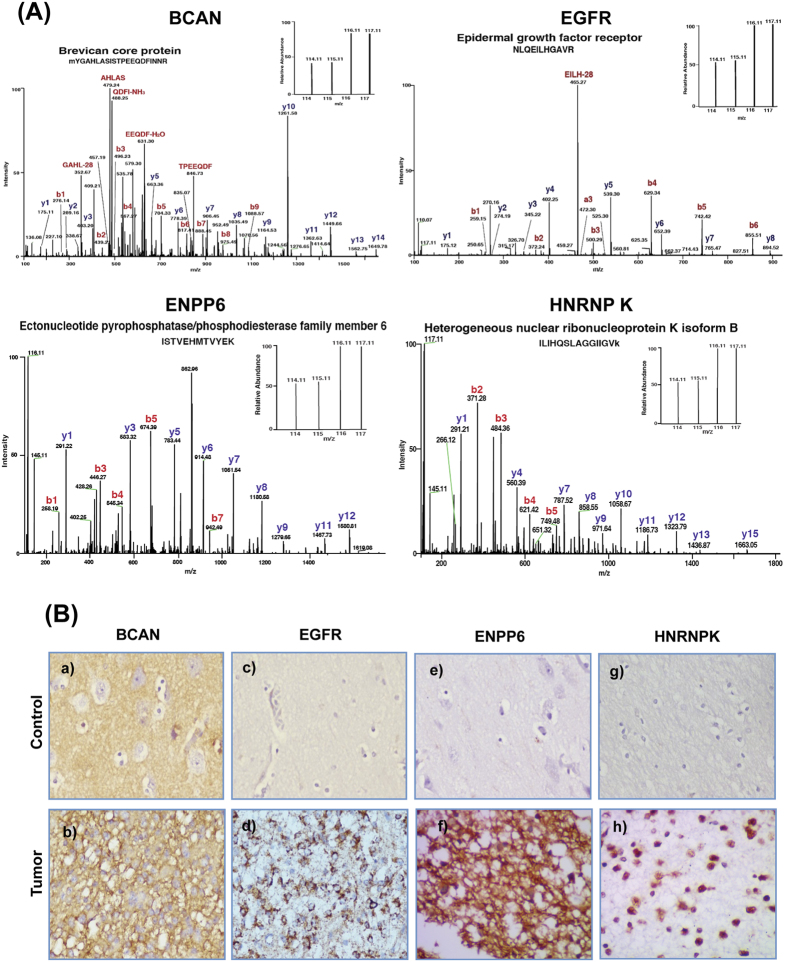
Verification of differential expression of the representative proteins observed in LC-MS/MS analysis by immunohistochemistry on tissue sections. (**A**) shows MS/MS spectra of peptides with their reporter ion intensities for representative differentially expressed proteins - BCAN, EGFR, ENPP6 and HNRNP K. (**B**) immunohistochemistry (IHC) images acquired for the above proteins. IHC protocol is described under Methods and the staining and scoring details for each protein are shown in Supplementary Table S3. For BCAN, normal brain tissue shows low staining with pyramidal cells negative (a), Grade II tumor cells show strong cytoplasmic positivity (b). For EGFR, normal brain tissue shows negative staining (c) and Grade II tumor cells show medium intensity cytoplasmic staining (d). ENPP6 shows medium intensity staining of neurophils in normal brain with no staining of normal glial and neuronal cells (e), while Grade II tumor cells show low to medium intensity staining for ENPP6 in neurophil as well as in tumor cells (f). For HNRNP K, normal brain tissue scored negative (g) whereas Grade II tumor cells showed strong positivity (h).

Changes at the chromosome levels such as mutations, copy number variations are important factors that may affect downstream events relevant to tumor development. We also mapped differentially expressed proteins to the chromosome 12 which is implicated in glial tumors^23^, and found that three of the over expressed proteins, CNPY2, MYL6, LIMA1, mapped to the regions on the chromosome that have been described as amplicons^24^,^25^. This provides a rationale and biological basis for their overexpression and confirms mass spectrometry results. To further confirm the quantitative differences observed by iTRAQ analysis, verification of the expression levels of EGFR, BCAN, ENPP6 and HNRNPK was carried out using immunohistochemistry (IHC) in tissue microarrays with DA tumor tissue sections. EGFR is well known for its involvement in tumorigenesis in general, BCAN is a brain-specific protein involved in brain development, ENPP6 is a protein implicated in the development of myelin sheath and HNRNP K is an important protein involved in post transcriptional regulation of gene expression. EGFR and BCAN are found to be over expressed at both protein and transcript level whereas over expression of the other two was observed only at protein level and not supported at the transcript level. MS/MS spectra of the peptide of representative overexpressed proteins, BCAN, EGFR, ENPP6, and HNRNP K and the corresponding IHC images are given in Fig. 3. We found that EGFR protein was overexpressed in 85% of DAs and BCAN showed overexpression in 77% of DAs in consistence with earlier observations^26^,^27^. ENPP6 was observed to be overexpressed in 30% cases of DA, while HNRNPK showed strong overexpression in all the DA cases (Fig. 3, Supplementary Table S3).

### Altered processes, enriched pathways and key molecular entities

**Figure 4:**
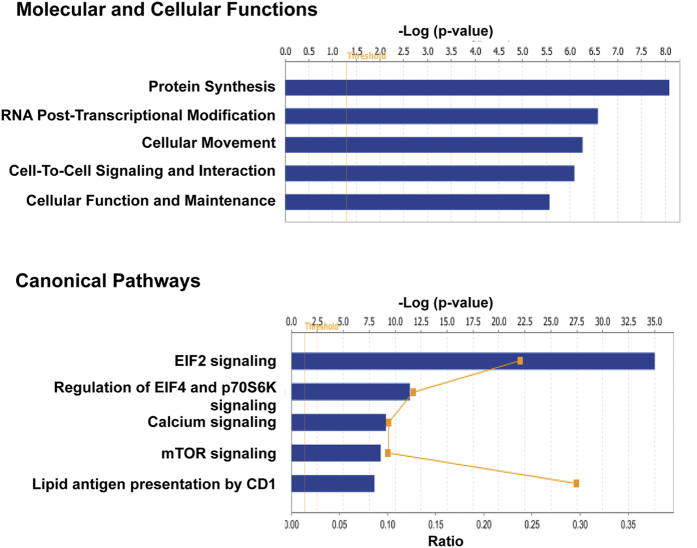
Ingenuity Pathway Analysis (IPA) to identify altered molecular and cellular functions and canonical pathways in diffuse astrocytomas. The differentially expressed proteins (n = 340) as listed in Supplementary Table S1 were used for these analyses. Top 5 molecular and cellular functions and canonical pathways are shown in the figure. Threshold criteria considered for the analysis are -log p-value > 1.3 or p-value < 0.05. The list of proteins under each category is provided in Supplementary Table S4.

Ingenuity Pathway Knowledge Base classification of the protein differentials observed by us indicated following molecular and cellular functions, networks and canonical pathways. The top network identified includes molecules associated with cell-to-cell signaling and interactions, tissue development and cellular movement. Major molecular and cellular functions and canonical pathways enriched in the dataset are shown in Fig. 4. Protein synthesis, cell-to-cell signaling and interactions, RNA post transcriptional modification are the molecular and cellular functions identified. In a recent study by TCGA group, genomic alterations including mutation, copy number variations and fusion transcript profiles, showed PI3K/AKT/mTOR signaling to be one of the major drivers for diffuse glioma^17^. It is interesting to note that among the canonical signaling pathways, we observed mTOR signaling and the downstream pathways i.e. eIF2, eIF4 and p70S6K signaling as most enriched pathways. The protein IDs and *P*-values associated with these molecular and cellular functions, networks and canonical pathways are shown in Supplementary Table S4A–C.

PI3K/AKT/mTOR signaling is known to play important role in cell proliferation and cell growth and mTOR is a master regulator of cell growth through its ability to stimulate ribosome biogenesis and mRNA translation^28^. DA are low grade tumors which represent an early stage of uncontrolled cell proliferation and growth with higher demands on increased protein synthesis. Consistent with this, protein synthesis is the major cellular process enriched in these tumors. The dataset also showed over expression of 43 ribosomal proteins of both small and large subunit (Supplementary Table S4C) suggesting increased ribosome biogenesis. Thus the increase in ribosome biogenesis which may be linked to mTOR activation is reflected by the enrichment of eIF2 pathway to provide the machinery required to promote cell growth and proliferation. Some of the ribosomal proteins include those with extra ribosomal functions which include tumor suppressor and proto-oncogene regulation (RPL5, RPL11, RPL23, RPL7A)^29^. mTOR is also implicated in early stage tumors of other tissues as well as low grade pediatric gliomas and is considered to be a potential therapeutic target^30^,^31^. However, inhibitors of mTOR have not been as successful, presumably because mTOR has multifunctional roles. Targeting multiples kinases or other molecules may be one possibility. On the other hand, it may be useful to view and integrate the mutational or the fusion transcript profiles discussed in the context of deregulated mToR cascades downstream^17^ and explore other possible targets.

RTK signalling is one of the most frequently observed pathway in human cancers and EGFR is one of the best known oncogenic RTK for several cancers including gliomas^32^. It is linked to the malignant transformation of these tumours through mutations and copy number variations as well as overexpression at RNA and protein level^33^,^34^. EGFR is often used to evaluate primary GBMs^35^. EGFRvIII being the most common mutation observed^33^, is viewed for targeted therapy for gliomas. TERT promoter mutation along with wildtype IDH status has been associated with glioma prognosis and in some conditions it is implicated with alterations in chromosome 7, which harbours EGFR gene^17^. A survey through literature until year 2012^33^, indicated several reports of overexpression of EGFR in Gr II tumors as has been also observed in our dataset. EGFR, and its mutant, EGFRvIII, have been also shown in exosomes and micro vesicles isolated from sera of patients with brain tumors^36^. Our observations thus further support EGFR having some potentially interesting features in the context of DA.

Brevican core protein (BCAN) is a brain-specific chondroitin sulfate proteoglycan has been observed to be highly expressed during development, in response to injury and in primary brain tumors^37^. This protein is reported to be overexpressed at gene and protein level in astrocytomas, including DAs. Functional studies showed that BCAN is upregulated during glial cell adhesion, motility and tumor growth^37^,^38^,^39^. We also observed BCAN to be overexpressed in our study. In view of being a brain-specific protein and its functional relevance to cancer progression, we believe BCAN may be considered as a candidate with significant biological and clinical implication. In addition, it should be noted that BCAN occurs both as soluble isoforms secreted into the extracellular space and membrane-bound isoforms which are anchored to the cell surface, raising its circulatory potential.

Ectonucleotide pyrophosphatase/phosphodiesterase family member 6 (ENPP6) was observed to be overexpressed in proteomic data. It is a glycosylphosphatidylinositol (GPI)-anchored alkaline lysophospholipase C predominantly expressed in brain myelin and kidney^40^,^41^. Other ENPP family proteins, ENPP 1, has been reported to be associated with maintenance of stem cell characteristics in glioblastoma, ENPP3 has been shown to have a role in cell invasion in human colon cancer^42^, however, the role of ENPP6 is not yet shown in cancer. Heterogeneous nuclear ribonucleoprotein (HNRNP) are important regulatory proteins involved in post-transcriptional regulation of gene expression^43^. We have earlier reported a large group of HNRNPs were found to be elevated in Gr III tumors. In the present analysis we identified 7 HNRNPs which include an important member HNRNP K. It was observed to be overexpressed in DA in our proteomic data. HNRNPs are generally localised in the nuclei or cytoplasm of the cell, interact with different classes of proteins or mRNAs to form complexes and regulate post transcriptional events such as splicing, stability or translation of mRNA in the gene expression cascade. Over expression of HNRNP K even in early stage, low grade tumors suggest the possible significance of alterations in these regulatory mechanisms as initial events. Interestingly, the localization of HNRNP K on the cell surface and their role in cell adhesion^44^ has also been been demonstrated.

### Differential proteins as potential surveillance markers for targeted investigation for recurrence

DAs have a long median survival but invariably recur. After treatment, they may recur as Gr II or higher grade anaplastic astrocytomas (WHO grade III) or secondary glioblastomas (WHO Grade IV)^5^,^45^. Some of the grade II tumor progress rapidly while others take more indolent course^5^. Histology at times helps in prediction of progression. For example, gemistocytic Grade II astrocytomas have higher chance of progression into malignant astrocytoma than fibrillary or protoplasmic type. IHC for proliferating antigen (Ki67/PCNA) or p53 and micro vessel density are sometimes used^46^,^47^, but they are not definitive for distinguishing the two tumor types. Alternative methods to monitor the recurrence post-treatment would therefore be useful^48^. Thus, we looked at the circulatory potential of the differentially expressed proteins in Gr II and III to explore them as circulatory protein markers for predicting recurrence. Although blood brain barrier may be a factor challenging appearance of tumor proteins in the plasma of DA patients, it is to be noted that the barrier is usually breached significantly as the tumor progresses to higher grades - a frequent feature of these tumors^49^,^50^.

On mapping of the differentially expressed protein dataset (n = 340) to SignalP 4.1 and TMHMM 2.0 software tools, or Exocarta database, we identified 84 signal sequence containing proteins, 106 with transmembrane domain containing proteins and 157 as exosomal proteins (Supplementary Table S5). Taking proteins that meet at least two of the above criteria namely, Signal Sequence/transmembrane domain/presence in exosomes, we arrived at a subset of 81 proteins which may be considered to have strong secretory potential. Comparison of this subset (n = 81) with the proteins experimentally detected in blood plasma or cerebrospinal fluid leaves a filtered list of 43 proteins with secretory character^51^,^52^. We generated a similar list of proteins (n = 40) from our dataset on anaplastic astrocytoma (WHO grade III)^20^ using the same criteria as above. Integration of the two resulted in a non-redundant list of 64 potential secretory proteins representing both grade II and III tumors (Supplementary Table S6). In view of their potential for clinical applications, their validation in specific sample cohorts is required. For this purpose, we have extracted their proteotypic peptides using GPMdb’s MRM database (Supplementary Table S6) for targeted analysis by mass spectrometry. Representative members under this category are shown in Table 1 which include EGFR, BCAN (discussed above) and others such as PDIA4, SPARC, ITGB1, SERPINA1 which are known to play important role in cancer. PDIA4 is reported to be associated with chemo resistance^53^ SPARC is reported to have role in cancer progression;^54^ Protein S100A10 is reported to have role in cell proliferation;^55^ SERPINA1 and ITGB1 are reported to play role in invasion and migration^56^,^57^. We believe this high confidence list of proteins with their proteotypic peptides would serve as a protein/peptide resource for further investigation by us and others in the community for tumor recurrence in a follow-up study cohort of patients diagnosed and treated for grade II tumors.

## Conclusions

We have identified 340 high confidence differentially expressed proteins with high resolution mass spectrometry, from the microsomal fraction of low-grade (Grade II) glioma - diffuse astrocytoma. mTOR activation, increase in ribosome biogenesis and protein translation were found to be major processes altered in these tumors; PI3Kc/mTOR pathway is also implicated in the large study carried out by TCGA. Differentially expressed proteins in this early stage, low-grade gliomas, include important regulatory proteins such as EGFR, HNRNP K and BCAN. Though not specific to DA they may be promising candidates for confirmatory diagnosis of these early stage tumors. We also provide a catalogue of differentially expressed proteins in Gr II and Gr III with secretory potential along with their proteotypic peptides which may be a useful resource for targeted investigation as surveillance markers for tumor recurrence - an important unmet clinical need.

## Additional Information

**How to cite this article**: Polisetty, R. V. *et al*. Microsomal membrane proteome of low grade diffuse astrocytomas: Differentially expressed proteins and candidate surveillance biomarkers. *Sci. Rep.* **6**, 26882; doi: 10.1038/srep26882 (2016).

## Supplementary Material

Supplementary Information

## Figures and Tables

**Table 1:** A list of Candidate proteins with secretory potential observed in DA, for post-treatment surveillance.

Gene symbol	Protein name	Peptides	Fold change	Signal peptide	TM domain	Exocarta database	CSF	Plasma
ANXA5	Annexin A5	10	4.01	+	−	+	+	+
APOE	Apolipoprotein E	7	2.52	+	−	+	+	+
BCAN	brevican core protein isoform 1 precursor	6	2.19	+	+		+	
CALR	Calreticulin	11	2.66	+	−	+	+	+
CANX	Calnexin	11	2.18	+	+	+	−	+
CD14	CD14 antigen	3	3.76	+	−	+	+	+
CP	Ceruloplasmin	6	2.25	+	−	+	+	+
EGFR	Epidermal growth factor receptor isoform a	7	2.04	+	+	+	+	+
ERAP1	Endoplasmic reticulum aminopeptidase 1 isoform b	6	2.01	+	−	+	+	+
FN1	Fibronectin 1 isoform 6	4	2.01	+	−	+	+	−
GSN	Gelsolin isoform b	10	2.77	+	−	+	+	+
HP	Haptoglobin isoform 1	10	2.91	+	−	+	+	+
HPX	Hemopexin	6	2.29	+	−	+	+	+
HSP90B1	Endoplasmin	14	2.43	+	−	+	+	+
HSPA5	78 kda glucose-regulated protein	18	2.36	+	−	+	+	+
ITGA6	Integrin alpha-6 isoform b	3	3.20	+	+	+	−	+
ITGB1	Integrin beta-1 isoform 1A	3	2.34	+	+	+	+	+
ITIH2	Inter-alpha globulin inhibitor H2 polypeptide	2	4.62	+	−	+	+	+
LGALS3BP	Galectin-3-binding protein	4	3.13	+	−	+	+	+
LMAN2	Vesicular integral-membrane protein VIP36	4	2.37	+	+	+	+	+
NUCB2	Nucleobindin-2	4	2.24	+	+	+	−	+
PDIA4	Protein disulfide-isomerase A4	9	3.15	+	−	+	−	+
PPIB	Peptidylprolyl isomerase B	7	2.97	+	−	+	+	+
S100A10	Protein S100-A10	2	2.75	−	+	+	−	+
SERPINA1	Serine proteinase inhibitor, clade A, member 1	11	2.47	+	−	+	+	+
SPARC	Sparc	5	2.45	+	−	+	+	+
TMED4	Transmembrane emp24 domain-containing protein 4	3	2.17	+	+	−	+	−
TNC	Tenascin	9	2.81	+	−	+	−	+
TPP1	Tripeptidyl-peptidase 1	4	2.22	+	−	+	+	+

The proteins were selected from Supplementary Table S6 on the basis of their upregulated expression and relevance to the cancer. The proteotypic peptides of these proteins are given in Supplementary Table S6 and may be useful for their targeted analysis in patient samples.
