# Supplementary material for: Microsomal membrane proteome of low grade diffuse astrocytomas: Differentially expressed proteins and candidate surveillance biomarkers
Source: Sci Rep. 2016 Jun 1;6:26882. doi: 10.1038/srep26882 (PMC4887981; doi:10.1038/srep26882)
Supplement: Supplementary Information [file srep26882-s1.pdf]

Microsomal membrane proteome of low grade diffuse astrocytomas: Differentially expressed proteins and candidate surveillance biomarkers

Ravindra Varma Polisetty[1,2*], Poonam Gautam[1,3*], Manoj Kumar Gupta[1,4,5], Rakesh Sharma[4], Harsha Gowda[4], Durairaj Renu[6], Bhadravathi Marigowda Shivakumar[7], Akhila Lakshmikantha[8], Kiran Mariswamappa[8], Praveen Ankathi[9], Aniruddh K Purohit[9], Megha S Uppin[9], Challa Sundaram[9], Ravi Sirdeshmukh[1,4,7#]

* Both the authors contributed equally to this work

[1]Centre for Cellular and Molecular Biology (CSIR), Hyderabad, India; [4]Institute of Bioinformatics, Bangalore, India; [5]Manipal University, Madhav Nagar, Manipal, India; [6]Strand Life Sciences, Bangalore, India; [7]Neuro-Oncology, Mazumdar Shaw Center for Translational Research, Narayana Health, Bangalore, India; [8]Mazumdar Shaw Medical Center, Narayana Health, Bangalore, India; [9]Nizam's Institute of Medical Sciences (NIMS), Hyderabad, India;

Present Address:

[2]Department of Biochemistry, Sri Venkateswara College, University of Delhi, New Delhi, India; [3]National Institute of Pathology (ICMR), New Delhi, India

Supplementary Table legends:

Supplementary Table S1: Differentially expressed proteins identified in diffuse astrocytoma. The list includes 340 proteins identified with ≥2 unique peptides and ≥2.0 fold change along with their peptide information, molecular function, biological process and cellular localization.

Supplementary Table S2: Comparison of 340 differentially expressed proteins with differential transcript data in Grade II diffuse astrocytoma. Out of 340 differentially expressed proteins listed in Supplementary Table S1, a total of 195 proteins were found to be common with the transcript data (Oncomine data resource; www.oncomine.org, Ref. 11) and are listed in the Table along with their fold change values.

Supplementary Table S3: Immunohistochemistry scoring details for four differentially expressed proteins - BCAN, EGFR, ENPP6 and HNRNP K. **Immunohistochemistry** was performed using tissue microarrays consisting of 4 control subjects and 13 diffuse astrocytoma samples. Scoring was based on both staining intensities and distributions. Scores +1, +2 and +3 indicate low, medium and strong staining, respectively.

Supplementary Table S4: Ingenuity Pathway Analysis of the differentially expressed proteins associated with major molecular and cellular functions (A), networks and processes (B) and those associated canonical pathways (C). Differentially expressed proteins from Supplementary Table S1 were used for the analysis. The Table includes only top 5 molecular and cellular functions, networks and processes or pathways and proteins from the dataset mapping to these respective groups are shown in bold.

Supplementary Table S5: List of proteins with secretory potential. Proteins with secretory potential were derived from the differentially expressed protein dataset (n=340) on the basis of mapping to Signal peptide, transmembrane domain or Exocarta database or detectability in cerebrospinal fluid (CSF) or plasma.

Supplementary Table S6: List of proteins with secretory potential and their proteotypic peptides. Proteins listed in **Supplementary Table S5** were first screened for mapping to at least two of the three criteria namely Signal peptide, transmembrane domain or Exocarta database. The filtered list was further screened for experimental detection in cerebrospinal fluid (CSF) or plasma. The proteotypic peptides for these proteins were derived from the GPMdb's MRM database. The same strategy was also applied to differentially expressed proteins from Grade III – anaplastic astrocytoma and proteins along with their proteotypic peptides are included in the Table.

Supplementary Table S1: Differentially expressed proteins identified in diffuse astrocytoma. The list includes 340 proteins identified with ≥2 unique peptides and ≥2.0 fold change along with their peptide information, molecular function, biological process and cellular localization.

Protein Accession	Protein Accession	Gene Symbol	Protein name	Unique peptides	PSMs	Protein fold change (116+117) /(114+115)	% variability (RSD) [Avg. Ratio]	Score	Coverage	p value (corr)	# AAs	MW [kDa]	calc. pI	Molecular class	Biological process	Molecular function	Primary Localization	Alternate Localization																								
**Accession**			**Sequence**	**PSM Ambiguity**	**Modifications**	**Activation Type**	**116+117 vs 114+115**	**# Missed Cleavages**	**# Protein Groups**	**# Proteins**	**116 vs 114**	**116 vs 115**	**117 vs 114**	**117 vs 115**	**% variability (RSD) [Between the four ratio's]**	**114**	**115**	**116**	**117**	**% variability (RSD) [Normal-Between 114 and 115]**	**% variability (RSD) [Tumor-Between 116 and 117]**	**Charge**	**Confidence Level**	**First Scan**	**Quan Info**	**Quan Usage**	**Quan Result ID**	**Rank**	**RT [min]**	**Search Engine Rank**	**XCorr**	**ΔM [ppm]**	**ΔScore**	**Ion Inject Time [ms]**	**Ions Matched**	**Isolation Interference [%]**	**Last Scan**	**MH+ [Da]**	**MS Order**	**m/z [Da]**	**Peptides Matched**	**Spectrum File**
4502101	NP_000691.1	ANXA1	annexin A1 [Homo sapiens]	4	5	3.201819897	29.89453293	31.659422	19.65	2.96E-04	346	38.69	7.02	Calcium binding protein	Cell communication,Signal transduction	Calcium ion binding	Plasma membrane	Mitochondrion, Cytoplasm, Nucleus																								
4502101			gVDEATIIDILTk	Unambiguous	N-Term(iTRAQ4plex); K13(iTRAQ4plex)	HCD	2.6463041	0	1	1	2.70424	2.6323	2.6607	2.589914	1.817119591	44937.047	46165.504	121520.37	119564.69	1.906972452	1.147208865	2	High	7874	Unique	Used	55160	1	70.093	1	5.7661	2.2616	1	73	20/24	32.59505	7874	1675.98	MS2	838.492	202	Glioma_grade2_2.raw
4502101			aAYLQETGkPLDETLk	Unambiguous	N-Term(iTRAQ4plex); K9(iTRAQ4plex); K16(iTRAQ4plex)	HCD	4.100691	0	1	1	3.7727	4.2587	3.9607	4.470933	7.532031215	25262.238	22379.412	95306.82	100056.85	8.557494602	3.438488255	3	High	3085	Unique	Used	80413	1	35.517	1	4.2926	2.2851	1	22	28/60	12.82002	3085	2209.25	MS2	737.088	83	Glioma_grade2_6.raw
4502101			aLTGHLEEVVLALLk	Unambiguous	N-Term(iTRAQ4plex); K15(iTRAQ4plex)	HCD	5.04951	0	1	1	4.81088	6.364	4.0558	5.365181	18.87035364	13807.609	10437.95	66426.695	56001.492	19.65480548	12.04254007	3	High	7451	Unique	Used	84030	1	76.034	1	5.4529	0.0792	1	7	24/56	2.030732	7451	1894.16	MS2	632.059	33	Glioma_grade2_6.raw
4502101			gGPGSAVSPYPTFNPSSDVAALHk	Unambiguous	N-Term(iTRAQ4plex); K24(iTRAQ4plex)	HCD	3.20182	0	1	1	2.99342	2.8335	3.5909	3.3990903	10.95124443	5192.0967	5485.139	15542.1045	18644.482	3.881382847	12.83375963	3	High	4694	Unique	Used	75146	1	48.411	1	7.5615	1.3038	1	10	32/92	4.152109	4694	2644.36	MS2	882.126	164	Glioma_grade2_5.raw
4502101			gVDEATIIDILTk	Unambiguous	N-Term(iTRAQ4plex); K13(iTRAQ4plex)	HCD	2.582681	0	1	1	2.5217	2.551	2.614	2.6443732	2.179177927	3475.6504	3435.6648	8764.541	9085.18	0.818197061	2.540387171	3	High	7865	Unique	Used	55152	1	70.016	1	4.3217	0.724	1	67	19/48	27.38943	7865	1675.97	MS2	559.329	189	Glioma_grade2_2.raw
4502107	NP_001145.1	ANXA5	annexin A5 [Homo sapiens]	10	29	4.00873661	34.24569015	195.71695	42.5	1.49E-04	320	35.914	5.0464	Calcium binding protein	Cell communication,Signal transduction	Calcium ion binding	Cytoplasm	Caveola, Endoplasmic reticulum, Nucleus, Extracellular, Plasma membrane																								
4502107			nFATSLYSMIk	Unambiguous	N-Term(iTRAQ4plex); K11(iTRAQ4plex)	HCD	5.1494384	0	1	1	4.7714	5.2762	5.0348	5.567472	6.580121555	244789.45	221370.6	1167987.8	1232474.6	7.104696184	3.799189154	2	High	6964	Unique	Used	54329	1	62.846	1	4.8149	-1.9915	1	8	18/20	12.32393	6964	1562.85	MS2	781.927	227	Glioma_grade2_2.raw
4502107			lYDAYELk	Unambiguous	N-Term(iTRAQ4plex); K8(iTRAQ4plex)	HCD	4.61685	0	1	1	4.57686	4.6176	4.6161	4.6571937	0.710458895	237592.78	235494.16	1087429.5	1096741.9	0.62734703	0.602961946	2	High	3837	Unique	Used	51389	1	39.889	1	3.4867	1.8142	1	10	14/14	35.28328	3837	1302.72	MS2	651.864	215	Glioma_grade2_2.raw
4502107			nFATSLYSmIk	Unambiguous	N-Term(iTRAQ4plex); M9(Oxidation); K11(iTRAQ4plex)	HCD	2.5439389	0	1	1	2.4485	2.7231	2.3829	2.6500847	6.328621695	196806.2	176961.84	481879.25	468963.88	7.508443753	1.920936361	2	High	5566	Unique	Used	53017	1	52.527	1	3.8459	-1.6886	1	14	17/20	29.67301	5566	1578.84	MS2	789.924	208	Glioma_grade2_2.raw
4502107			qEISAAFk	Unambiguous	N-Term(iTRAQ4plex); K8(iTRAQ4plex)	HCD	4.1157703	0	1	1	4.48774	4.1506	4.0782	3.771747	7.130641386	189603	205005.72	850889.2	773229.75	5.520084686	6.762253925	2	High	1588	Unique	Used	49268	1	23.938	1	3.1698	-0.9906	1	5	14/14	6.260353	1588	1181.68	MS2	591.342	238	Glioma_grade2_2.raw
4502107			lYDAYELk	Unambiguous	N-Term(iTRAQ4plex); K8(iTRAQ4plex)	HCD	4.050477	0	1	1	3.34639	4.2313	3.9075	4.940751	16.20769775	95387.125	75438.266	319202.66	372721.7	16.51507822	10.93867431	2	High	3450	Unique	Used	80747	1	38.338	1	3.4513	1.252	1	33	13/14	9.433839	3450	1302.72	MS2	651.864	228	Glioma_grade2_6.raw
4502107			sEIDLFNIR	Unambiguous	N-Term(iTRAQ4plex)	HCD	3.6726139	0	1	1	3.42846	3.9071	3.4668	3.9508512	7.562232376	86934.086	76283.625	298050.38	301385.25	9.228181366	0.786776452	2	High	4246	Unique	Used	86858	1	57.105	1	3.863	0.2276	1	32	15/16	25.19465	4246	1250.69	MS2	625.847	322	Glioma_grade2_1rep.raw
4502107			lYDAYELk	Unambiguous	N-Term(iTRAQ4plex); K8(iTRAQ4plex)	HCD	4.181157	0	1	1	4.0217	4.5058	3.8914	4.359813	6.826667099	83255.664	74310.234	334829.03	323978.72	8.028861948	2.32915529	2	High	3793	Unique	Used	59376	1	39.952	1	3.1792	0.315	1	30	14/14	15.51158	3793	1302.72	MS2	651.863	236	Glioma_grade2_3.raw
4502107			nFATSLYSmIk	Unambiguous	N-Term(iTRAQ4plex); M9(Oxidation); K11(iTRAQ4plex)	HCD	3.4474475	0	1	1	3.19876	3.382	3.5093	3.7103808	6.240322729	75847.31	71737.484	242617.44	266173.38	3.938191402	6.547510001	2	High	5701	Unique	Used	53144	1	53.501	1	4.3819	-0.6835	1	33	17/20	18.72362	5701	1578.84	MS2	789.925	234	Glioma_grade2_2.raw
4502107			mLVVLLQANR	Unambiguous	N-Term(iTRAQ4plex)	HCD	4.556553	0	1	1	4.29151	4.655	4.4658	4.8440466	5.225033972	65712.72	60581.17	282006.78	293458	5.746206413	2.814154957	2	High	4473	Unique	Used	87009	1	59.806	1	3.1414	-0.2601	1	33	14/18	38.97628	4473	1300.79	MS2	650.898	181	Glioma_grade2_1rep.raw
4502107			kNFATSLYSmIk	Unambiguous	N-Term(iTRAQ4plex); K1(iTRAQ4plex); M10(Oxidation); K12(iTRAQ4plex)	HCD	1.586069	1	1	1	1.54908	1.7838	1.4144	1.6286637	9.685939489	37354.953	32439.75	57865.77	52833.445	9.95941876	6.428936524	3	High	4061	Unique	Used	91504	1	46.017	1	5.7704	-0.036	1	25	20/44	52.53526	4061	1851.04	MS2	617.685	192	Glioma_grade2_7rep.raw
4502107			kNFATSLYSMIk	Unambiguous	N-Term(iTRAQ4plex); K1(iTRAQ4plex); K12(iTRAQ4plex)	HCD	3.798566	1	1	1	3.36696	4.004	3.6258	4.3118353	10.86261051	34841.305	29298.191	117309.13	126328.98	12.22202774	5.235631733	2	High	4884	Unique	Used	92126	1	54.446	1	5.3075	2.6215	1	100	18/22	20.34062	4884	1835.05	MS2	918.029	196	Glioma_grade2_7rep.raw
4502107			yMTISGFQIEETIDR	Unambiguous	N-Term(iTRAQ4plex)	HCD	1.9368309	0	1	1	1.8797	2.0184	1.8609	1.9981786	4.148058441	17955.947	16722.438	33751.75	33414.414	5.030352933	0.710276005	2	High	4852	Unique	Used	87288	1	63.937	1	4.8922	-0.5277	1	100	22/28	51.49546	4852	1946.96	MS2	973.986	171	Glioma_grade2_1rep.raw
4502107			eTSGNLEQLLLAVVk	Unambiguous	N-Term(iTRAQ4plex); K15(iTRAQ4plex)	HCD	5.8851485	0	1	1	6.29621	5.8989	5.8704	5.5000267	5.522285324	16269.718	17365.436	102437.516	95510.35	4.607022926	4.94902638	3	High	8577	Unique	Used	55796	1	75.906	1	5.8402	0.908	1	16	23/56	10.76001	8577	1902.12	MS2	634.71	110	Glioma_grade2_2.raw
4502107			kNFATSLYSMIk	Unambiguous	N-Term(iTRAQ4plex); K1(iTRAQ4plex); K12(iTRAQ4plex)	HCD	3.5391364	1	1	1	3.11501	3.4196	3.648	4.004731	10.5814058	14297.984	13024.42	44538.41	52159.3	6.591994911	11.14562692	3	High	4878	Unique	Used	92121	1	54.395	1	6.3152	0.8181	1	30	21/44	45.40845	4878	1835.05	MS2	612.354	191	Glioma_grade2_7rep.raw
4502107			nFATSLYSMIk	Unambiguous	N-Term(iTRAQ4plex); K11(iTRAQ4plex)	HCD	6.0360336	0	1	1	5.92587	6.2309	5.8508	6.1518683	2.98909106	13214.57	12567.753	78307.81	77315.164	3.547924575	0.902060538	3	High	6970	Unique	Used	54335	1	62.879	1	4.1613	-3.6404	1	18	20/40	11.74458	6970	1562.84	MS2	521.619	247	Glioma_grade2_2.raw
4502107			nFATSLYSmIk	Unambiguous	N-Term(iTRAQ4plex); M9(Oxidation); K11(iTRAQ4plex)	HCD	2.9910831	0	1	1	3.35815	2.7047	3.3466	2.695442	12.44619417	11545.05	14334.136	38769.97	38636.832	15.24145021	0.243241628	3	High	5703	Unique	Used	53146	1	53.513	1	4.5077	-1.1173	1	28	17/40	14.85111	5703	1578.84	MS2	526.952	224	Glioma_grade2_2.raw
4502107			sIPAYLAETLYYAmk	Unambiguous	N-Term(iTRAQ4plex); M14(Oxidation); K15(iTRAQ4plex)	HCD	6.001161	0	1	1	5.59047	6.7388	5.3893	6.4962096	10.96237054	11406.89	9463.1455	63769.867	61474.58	13.17137114	2.591752435	2	High	8424	Unique	Used	55655	1	74.703	1	4.8501	-0.6501	1	59	20/28	13.41487	8424	2038.08	MS2	1019.54	218	Glioma_grade2_2.raw
4502107			eTSGNLEQLLLAVVk	Unambiguous	N-Term(iTRAQ4plex); K15(iTRAQ4plex)	HCD	2.677374	0	1	1	2.94135	2.2682	3.208	2.4738128	15.75136058	11255.566	14595.96	33106.527	36107.67	18.27370074	6.132061509	3	High	5975	Unique	Used	88133	1	76.052	1	4.705	1.6781	1	13	22/56	15.19012	5975	1902.12	MS2	634.711	118	Glioma_grade2_1rep.raw
4502107			sIPAYLAETLYYAmk	Unambiguous	N-Term(iTRAQ4plex); M14(Oxidation); K15(iTRAQ4plex)	HCD	4.181609	0	1	1	3.70545	4.0526	4.2996	4.702373	10.01438023	10035.341	9175.727	37185.49	43147.69	6.328007257	10.49606663	3	High	8377	Unique	Used	55612	1	74.318	1	5.0824	-2.2139	1	6	24/56	5.476365	8377	2038.08	MS2	680.03	192	Glioma_grade2_2.raw
4502107			sIPAYLAETLYYAMk	Unambiguous	N-Term(iTRAQ4plex); K15(iTRAQ4plex)	HCD	2.9106615	0	1	1	2.87365	3.0315	2.7961	2.9497066	3.467293784	9754.794	9246.867	28031.857	27275.545	3.78028664	1.933894287	3	High	6094	Unique	Used	88240	1	76.996	1	4.8277	-0.3694	1	16	22/56	28.81751	6094	2022.09	MS2	674.7	242	Glioma_grade2_1rep.raw
4502107			sIPAYLAETLYYAmk	Unambiguous	N-Term(iTRAQ4plex); M14(Oxidation); K15(iTRAQ4plex)	HCD	5.036475	0	1	1	4.52242	4.9593	5.1069	5.6001806	8.804892557	9251.975	8437.012	41841.355	47248.79	6.515532673	8.583741686	2	High	8341	Unique	Used	55580	1	74.003	1	5.1227	0.6077	1	100	20/28	37.43287	8341	2038.08	MS2	1019.54	242	Glioma_grade2_2.raw
4502107			sIPAYLAETLYYAmk	Unambiguous	N-Term(iTRAQ4plex); M14(Oxidation); K15(iTRAQ4plex)	HCD	1.7515465	0	1	1	1.55459	1.8798	1.6455	1.9897002	11.41876396	7241.7505	5989.0376	11257.95	11916.39	13.39000791	4.018128577	3	High	8299	Unique	Used	55542	1	73.651	1	6.3854	-0.8663	1	68	21/56	38.31666	8299	2038.08	MS2	680.031	198	Glioma_grade2_2.raw
4502107			sIPAYLAETLYYAmk	Unambiguous	N-Term(iTRAQ4plex); M14(Oxidation); K15(iTRAQ4plex)	HCD	4.467264	0	1	1	3.25883	4.4149	4.5059	6.1043906	25.58051754	5850.492	4318.5205	19065.732	26361.936	21.30526315	22.71389025	3	High	8044	Unique	Used	63290	1	73.857	1	5.5064	-1.7647	1	13	22/56	2.311425	8044	2038.08	MS2	680.031	188	Glioma_grade2_3.raw
4502107			sIPAYLAETLYYAmk	Unambiguous	N-Term(iTRAQ4plex); M14(Oxidation); K15(iTRAQ4plex)	HCD	5.870147	0	1	1	6.27919	5.4945	6.2994	5.5122213	7.697975956	5722.088	6539.27	35930.074	36045.902	9.425300748	0.227583616	2	High	7994	Unique	Used	63244	1	73.443	1	5.0133	-0.7099	1	100	20/28	25.73871	7994	2038.08	MS2	1019.54	221	Glioma_grade2_3.raw
4502107			dPDAGIDEAQVEQDAQALFQAGELk	Unambiguous	N-Term(iTRAQ4plex); K25(iTRAQ4plex)	HCD	2.7945337	0	1	1	2.97558	2.8162	2.7717	2.623184	5.186325995	5081.443	5369.071	15120.254	14084.061	3.892319732	5.017745473	3	High	5774	Unique	Used	87978	1	73.978	1	7.8472	-3.4902	1	32	26/96	28.22181	5774	2946.45	MS2	982.821	112	Glioma_grade2_1rep.raw
4502107			sIPAYLAETLYYAMk	Unambiguous	N-Term(iTRAQ4plex); K15(iTRAQ4plex)	HCD	2.0299962	0	1	1	1.89859	2.2365	1.8547	2.1847858	9.533297659	4317.027	3664.7432	8196.283	8006.6787	11.55719312	1.65488864	3	High	6898	Unique	Used	93525	1	77.025	1	4.6419	-2.0899	1	89	21/56	0	6898	2022.08	MS2	674.699	238	Glioma_grade2_7rep.raw
4502107			sIPAYLAETLYYAMk	Unambiguous	N-Term(iTRAQ4plex); K15(iTRAQ4plex)	HCD	4.0087366	0	1	1	3.69586	3.1204	5.0609	4.2728963	20.52646447	4195.708	4969.469	15506.755	21234.025	11.93935807	22.04521218	3	High	7552	Unique	Used	84120	1	76.834	1	6.4783	-0.6411	1	89	21/56	16.03208	7552	2022.08	MS2	674.7	241	Glioma_grade2_6.raw
4502107			sIPAYLAETLYYAMk	Unambiguous	N-Term(iTRAQ4plex); K15(iTRAQ4plex)	HCD	6.1481557	0	1	1	5.86153	5.3867	6.9767	6.4115644	11.15957657	3580.7454	3896.3564	20988.648	24981.74	5.969443356	12.28417925	3	High	8725	Unique	Used	55936	1	76.935	1	5.8475	1.2605	1	33	23/56	14.07269	8725	2022.09	MS2	674.701	254	Glioma_grade2_2.raw
4502107			sIPAYLAETLYYAmk	Unambiguous	N-Term(iTRAQ4plex); M14(Oxidation); K15(iTRAQ4plex)	HCD	3.5990605	0	1	1	4.68535	3.2659	4.0769	2.8418548	22.16349632	3000.0254	4303.862	14056.181	12230.951	25.24550697	9.819500356	3	High	8461	Unique	Used	55689	1	75.004	1	5.569	1.9188	1	23	22/56	4.250354	8461	2038.08	MS2	680.033	194	Glioma_grade2_2.raw
4502163	NP_001638.1	APOD	apolipoprotein D precursor [Homo sapiens]	4	6	2.610782862	10.10105977	40.351877	25.93	1.47E-04	189	21.262	5.1479	Transport/cargo protein	Transport	Transporter activity	Extracellular	-																								
4502163			nILTSNNIDVk	Unambiguous	N-Term(iTRAQ4plex); K11(iTRAQ4plex)	HCD	2.1676707	0	1	1	2.08326	2.1132	2.2214	2.253293	3.794949303	118105.17	116431.09	246044.02	262353.38	1.009441628	4.536789155	2	High	2890	Unique	Used	50499	1	33.025	1	4.704	-0.1099	1	24	19/20	11.63018	2890	1518.87	MS2	759.94	244	Glioma_grade2_2.raw
4502163			mTVTDQVNcPk	Unambiguous	N-Term(iTRAQ4plex); M1(Oxidation); C9(Methylthio); K11(iTRAQ4plex)	HCD	2.2935903	0	1	1	2.12932	2.3635	2.2306	2.4759274	6.590893971	109830.836	98948.63	233864.94	244989.61	7.371301215	3.285477645	2	High	1845	Unique	Used	49512	1	25.694	1	4.4121	-0.1227	1	28	19/20	0	1845	1585.76	MS2	793.385	100	Glioma_grade2_2.raw
4502163			nPNLPPETVDSLk	Unambiguous	N-Term(iTRAQ4plex); K13(iTRAQ4plex)	HCD	2.740175	0	1	1	2.6085	2.5239	2.9637	2.8675828	7.603400542	55072.195	56917.844	143655.67	163216.62	2.330691078	9.014616726	2	High	3139	Unique	Used	50730	1	34.89	1	4.1027	-0.2737	1	70	15/24	0	3139	1711.95	MS2	856.477	185	Glioma_grade2_2.raw
4502163			cPNPPVQENFDVNk	Unambiguous	N-Term(iTRAQ4plex); C1(Methylthio); K14(iTRAQ4plex)	HCD	2.6168954	0	1	1	2.39284	2.5583	2.6717	2.856446	7.440435859	37632.383	35198.43	90048.2	100542.41	4.726199257	7.786878616	2	High	4396	Unique	Used	51916	1	43.993	1	4.3365	0.2707	1	48	18/26	5.81731	4396	1934.93	MS2	967.971	94	Glioma_grade2_2.raw
4502163			cPNPPVQENFDVNk	Unambiguous	N-Term(iTRAQ4plex); C1(Methylthio); K14(iTRAQ4plex)	HCD	2.6046703	0	1	1	2.45388	2.5684	2.6394	2.7624986	4.961999698	22926.02	21904.15	56257.62	60510.188	3.223593426	5.150425827	2	High	4187	Unique	Used	59744	1	42.968	1	4.293	2.9834	1	97	18/26	0	4187	1934.94	MS2	967.974	103	Glioma_grade2_3.raw
4502163			cPNPPVQENFDVNk	Unambiguous	N-Term(iTRAQ4plex); C1(Methylthio); K14(iTRAQ4plex)	HCD	2.8226104	0	1	1	2.75422	2.886	2.7621	2.8942723	2.702337144	17739.977	16930.127	48859.797	49000.395	3.303424915	0.203183332	2	High	4307	Unique	Used	51833	1	43.322	1	4.5357	1.5324	1	100	18/26	0	4307	1934.94	MS2	967.972	93	Glioma_grade2_2.raw
4502277	NP_001668.1	ATP1B1	sodium/potassium-transporting ATPase subunit beta-1 [Homo sapiens]	9	35	0.464111835	19.50419629	243.4818	38.94	6.25E-05	303	35.039	8.5288	ATPase	Transport	ATPase activity	Plasma membrane	-																								
4502277			fIWNSEk	Unambiguous	N-Term(iTRAQ4plex); K7(iTRAQ4plex)	HCD	0.44985414	0	1	1	0.4136	0.4339	0.465	0.48789346	0.073064686	1.12E+07	1.07E+07	4645108	5222684.5	3.393900052	8.277601293	2	High	3713	Unique	Used	66975	1	38.695	1	2.9925	0.2015	1	1	12-Dec	6.917296	3713	1211.67	MS2	606.337	243	Glioma_grade2_4.raw
4502277			fIWNSEk	Unambiguous	N-Term(iTRAQ4plex); K7(iTRAQ4plex)	HCD	0.5816051	0	1	1	0.55892	0.5924	0.5714	0.6056506	0.035952819	7783132.5	7342691.5	4350150.5	4447105.5	4.117974899	1.558611866	2	High	3291	Unique	Used	58911	1	36.053	1	3.1996	1.0075	1	1	12-Dec	16.74347	3291	1211.67	MS2	606.337	262	Glioma_grade2_3.raw

4502277			fIWNSEk	Unambiguous	N-Term(iTRAQ4plex); K7(iTRAQ4plex)	HCD	0.4839184	0	1	1	0.48874	0.467	0.5016	0.4793105	0.030285781	7519154.5	7869456	3674919	3771913	3.219271371	1.841994425	2	High	3628	Unique	Used	66897	1	38.01	1	3.0104	0.806	1	1	12-Dec	7.82231	3628	1211.67	MS2	606.337	248	Glioma_grade2_4.raw
4502277			aYGENIGYSEk	Unambiguous	N-Term(iTRAQ4plex); K11(iTRAQ4plex)	HCD	0.35844907	0	1	1	0.3567	0.3611	0.3559	0.3602208	0.007146966	6433470	6355729.5	2294811	2289465.8	0.859644651	0.164895242	2	High	1569	Unique	Used	49250	1	23.809	1	5.3255	0.3232	1	1	18/20	4.853107	1569	1518.77	MS2	759.888	116	Glioma_grade2_2.raw
4502277			yNPNVLPVQcTGk	Unambiguous	N-Term(iTRAQ4plex); C10(Methylthio); K13(iTRAQ4plex)	HCD	0.43105575	0	1	1	0.39556	0.4135	0.4478	0.46816346	0.076023158	6189407	5920443	2448285.5	2771735	3.141017738	8.762928606	2	High	5072	Unique	Used	52551	1	48.954	1	4.2155	0.5955	1	1	18/24	6.590322	5072	1766.92	MS2	883.963	145	Glioma_grade2_2.raw
4502277			vAPPGLTQIPQIQk	Unambiguous	N-Term(iTRAQ4plex); K14(iTRAQ4plex)	HCD	0.40586686	0	1	1	0.37861	0.4067	0.4051	0.43514556	0.056834797	5414660	5041132.5	2050033.2	2193626.2	5.05220103	4.785284325	2	High	5064	Unique	Used	68226	1	49.24	1	5.3383	1.6022	1	1	19/26	7.638177	5064	1778.08	MS2	889.544	68	Glioma_grade2_4.raw
4502277			tEISFRPNDPk	Unambiguous	N-Term(iTRAQ4plex); K11(iTRAQ4plex)	HCD	0.35430562	0	1	1	0.32531	0.3505	0.3579	0.38554037	0.06984686	2916518	2707079	948783.8	1043688.3	5.266939901	6.736112575	2	High	1300	Unique	Used	72073	1	21.605	1	2.9322	2.566	1	2	15/20	7.217797	1300	1591.87	MS2	796.44	217	Glioma_grade2_5.raw
4502277			vAPPGLTQIPQIQk	Unambiguous	N-Term(iTRAQ4plex); K14(iTRAQ4plex)	HCD	0.42894045	0	1	1	0.39774	0.4396	0.4193	0.463426	0.065298296	2587814.2	2341311.5	1029276.75	1085024.6	7.072399503	3.728861335	2	High	4910	Unique	Used	60412	1	48.671	1	5.6342	-0.2515	1	2	18/26	10.13506	4910	1778.08	MS2	889.542	66	Glioma_grade2_3.raw
4502277			vAPPGLTQIPQIQk	Unambiguous	N-Term(iTRAQ4plex); K14(iTRAQ4plex)	HCD	0.38964728	0	1	1	0.34786	0.3987	0.3818	0.43753436	0.095232537	2409826.5	2102714	838289.5	920009.6	9.624792568	6.572810834	2	High	5122	Unique	Used	52597	1	49.34	1	5.0964	1.1902	1	1	18/26	11.16934	5122	1778.08	MS2	889.544	76	Glioma_grade2_2.raw
4502277			vAPPGLTQIPQIQk	Unambiguous	N-Term(iTRAQ4plex); K14(iTRAQ4plex)	HCD	0.4488833	0	1	1	0.40898	0.4492	0.4486	0.49270424	0.07601903	1376634	1253498.2	563018.5	617603.9	6.62097207	6.538535351	2	High	5033	Unique	Used	52515	1	48.653	1	4.7175	0.5724	1	5	15/26	33.24094	5033	1778.08	MS2	889.543	75	Glioma_grade2_2.raw
4502277			vAPPGLTQIPQIQk	Unambiguous	N-Term(iTRAQ4plex); K14(iTRAQ4plex)	HCD	0.40898806	0	1	1	0.33469	0.3427	0.4738	0.48505726	0.199102106	851991.1	832136.94	285154.2	403634.06	1.66721423	24.32617317	3	High	5029	Unique	Used	68194	1	48.964	1	3.7416	1.423	1	0	21/52	9.510057	5029	1778.08	MS2	593.365	58	Glioma_grade2_4.raw
4502277			vAPPGLTQIPQIQk	Unambiguous	N-Term(iTRAQ4plex); K14(iTRAQ4plex)	HCD	0.41522723	0	1	1	0.3837	0.4305	0.4016	0.45060182	0.071420461	731390.56	651892.06	280632.84	293743.75	8.127612916	3.228130648	2	High	5003	Unique	Used	60497	1	49.433	1	5.6754	2.426	1	8	21/26	4.63307	5003	1778.08	MS2	889.545	61	Glioma_grade2_3.raw
4502277			fIWNSEk	Unambiguous	N-Term(iTRAQ4plex); K7(iTRAQ4plex)	HCD	0.61346376	0	1	1	0.54574	0.6787	0.561	0.69768673	0.12641369	720638.1	579453.75	393282.06	404277.2	15.35774742	1.949632697	2	High	3309	Unique	Used	50891	1	36.087	1	2.9518	-3.6268	1	13	12-Dec	68.56671	3309	1211.66	MS2	606.335	244	Glioma_grade2_2.raw
4502277			vAPPGLTQIPQIQk	Unambiguous	N-Term(iTRAQ4plex); K14(iTRAQ4plex)	HCD	0.46411183	0	1	1	0.43191	0.4645	0.4638	0.49874005	0.0587291	703024.4	653729.3	303644.47	326040.97	5.138279629	5.030040722	2	High	4972	Unique	Used	68141	1	48.526	1	5.5756	1.9454	1	9	18/26	11.68508	4972	1778.08	MS2	889.544	68	Glioma_grade2_4.raw
4502277			vAPPGLTQIPQIQk	Unambiguous	N-Term(iTRAQ4plex); K14(iTRAQ4plex)	HCD	0.3860902	0	1	1	0.33829	0.4287	0.3525	0.44666424	0.138198986	621564.56	490467.8	210270.34	219074.44	16.67207022	2.899971819	2	High	5150	Unique	Used	68303	1	49.965	1	5.4146	2.5633	1	8	19/26	8.647473	5150	1778.08	MS2	889.545	62	Glioma_grade2_4.raw
4502277			aYGENIGYSEk	Unambiguous	N-Term(iTRAQ4plex); K11(iTRAQ4plex)	HCD	0.30759776	0	1	1	0.26735	0.2926	0.3213	0.35164234	0.11803655	608494.1	555996.4	162682.8	195511.88	6.375574497	12.96147954	2	High	1486	Unique	Used	78968	1	22.838	1	5.5118	-7.7143	1	26	18/20	10.09104	1486	1518.76	MS2	759.882	97	Glioma_grade2_6.raw
4502277			vAPPGLTQIPQIQk	Unambiguous	N-Term(iTRAQ4plex); K14(iTRAQ4plex)	HCD	0.45408225	0	1	1	0.44903	0.4656	0.443	0.4593216	0.022358145	562831.1	542787.4	252727.78	249313.95	2.563820376	0.961650078	3	High	5120	Unique	Used	68276	1	49.717	1	4.0892	1.9379	1	0	21/52	8.835566	5120	1778.08	MS2	593.365	54	Glioma_grade2_4.raw
4502277			kFIWNSEk	Unambiguous	N-Term(iTRAQ4plex); K1(iTRAQ4plex); K8(iTRAQ4plex)	HCD	0.60906214	1	1	1	0.56887	0.6127	0.6056	0.6523505	0.056095118	471465.5	437706.4	268204.16	285538	5.251215647	4.426925271	2	High	1969	Unique	Used	89803	1	26.33	1	3.6465	-7.6584	1	37	14/14	39.69008	1969	1483.85	MS2	742.43	194	Glioma_grade2_7rep.raw
4502277			vAPPGLTQIPQIQk	Unambiguous	N-Term(iTRAQ4plex); K14(iTRAQ4plex)	HCD	0.49464813	0	1	1	0.54362	0.4524	0.5454	0.45389745	0.105787476	329531.94	395989.12	179139.19	179738.47	12.9540892	0.236155659	2	High	4634	Unique	Used	75097	1	47.842	1	5.3987	-0.0455	1	12	20/26	1.713779	4634	1778.08	MS2	889.543	71	Glioma_grade2_5.raw
4502277			sYEAYVLNIVR	Unambiguous	N-Term(iTRAQ4plex)	HCD	0.53424793	0	1	1	0.51976	0.5324	0.5361	0.5490872	0.022582001	310538.25	303169.8	161405.6	166466.66	1.697967287	2.182990318	2	High	4739	Unique	Used	87209	1	62.643	1	4.4593	0.3975	1	29	18/20	29.10339	4739	1470.81	MS2	735.908	227	Glioma_grade2_1rep.raw
4502277			yNPNVLPVQcTGk	Unambiguous	N-Term(iTRAQ4plex); C10(Methylthio); K13(iTRAQ4plex)	HCD	0.49087834	0	1	1	0.47866	0.4665	0.5158	0.50279045	0.045646562	270343.28	277362.22	129401.68	139455.08	1.812338956	5.288189379	2	High	4990	Unique	Used	52475	1	48.326	1	4.4607	4.2571	1	51	16/24	46.41596	4990	1766.92	MS2	883.966	206	Glioma_grade2_2.raw
4502277			kFIWNSEk	Unambiguous	N-Term(iTRAQ4plex); K1(iTRAQ4plex); K8(iTRAQ4plex)	HCD	0.6256053	1	1	1	0.59247	0.5992	0.6517	0.6591122	0.055360382	183583.52	181521.47	108768.586	119643.02	0.798723424	6.732920588	3	High	1955	Unique	Used	89790	1	26.23	1	4.7504	-5.4257	1	11	15/28	44.41162	1955	1483.86	MS2	495.29	179	Glioma_grade2_7rep.raw
4502277			yNPNVLPVQcTGk	Unambiguous	N-Term(iTRAQ4plex); C10(Methylthio); K13(iTRAQ4plex)	HCD	0.37393382	0	1	1	0.30898	0.3531	0.3921	0.4481769	0.157387235	158260.16	138468.19	48898.516	62058.24	9.432894565	16.77289498	2	High	4359	Unique	Used	91739	1	48.911	1	4.5975	-0.8553	1	76	16/24	12.65441	4359	1766.92	MS2	883.962	196	Glioma_grade2_7rep.raw
4502277			yNPNVLPVQcTGk	Unambiguous	N-Term(iTRAQ4plex); C10(Methylthio); K13(iTRAQ4plex)	HCD	0.5615499	0	1	1	0.67707	0.4825	0.6725	0.47922918	0.193802216	127236.13	178547.33	86147.58	85565.086	23.7308437	0.479738003	2	High	4911	Unique	Used	60413	1	48.676	1	4.4063	-2.0989	1	39	17/24	15.64045	4911	1766.91	MS2	883.96	145	Glioma_grade2_3.raw
4502277			yNPNVLPVQcTGk	Unambiguous	N-Term(iTRAQ4plex); C10(Methylthio); K13(iTRAQ4plex)	HCD	0.6152352	0	1	1	0.56234	0.6294	0.6026	0.674438	0.076281983	108967.63	97352.99	61277.16	65658.555	7.961192347	4.881390735	3	High	5123	Unique	Used	52598	1	49.346	1	3.7762	######	1	3	19/48	10.87289	5123	1766.92	MS2	589.644	195	Glioma_grade2_2.raw
4502277			yNPNVLPVQcTGk	Unambiguous	N-Term(iTRAQ4plex); C10(Methylthio); K13(iTRAQ4plex)	HCD	0.650151	0	1	1	0.59843	0.6274	0.6719	0.7043728	0.072130974	108089.36	103102.41	64684	72622.53	3.339435209	8.176433263	2	High	3808	Unique	Used	86572	1	51.826	1	4.9601	0.8028	1	100	16/24	17.67562	3808	1766.92	MS2	883.963	236	Glioma_grade2_1rep.raw
4502277			vAPPGLTQIPQIQk	Unambiguous	N-Term(iTRAQ4plex); K14(iTRAQ4plex)	HCD	0.45026562	0	1	1	0.54736	0.5177	0.379	0.35843158	0.212443117	107796.125	113970.54	59003.26	40850.64	3.937445437	25.7092426	3	High	5002	Unique	Used	60496	1	49.43	1	3.8694	1.217	1	2	21/52	8.320319	5002	1778.08	MS2	593.365	56	Glioma_grade2_3.raw
4502277			vAPPGLTQIPQIQk	Unambiguous	N-Term(iTRAQ4plex); K14(iTRAQ4plex)	HCD	0.5461032	0	1	1	0.54099	0.5331	0.5593	0.551144	0.021000528	73142.64	74224.45	39569.28	40908.363	1.038162845	2.353137181	2	High	4815	Unique	Used	60325	1	47.913	1	5.4654	4.0737	1	100	18/26	7.895434	4815	1778.09	MS2	889.546	64	Glioma_grade2_3.raw
4502277			sYEAYVLNIVR	Unambiguous	N-Term(iTRAQ4plex)	HCD	0.43547073	0	1	1	0.4125	0.4356	0.4353	0.45972583	0.04424601	55645.234	52689.086	22953.895	24222.533	3.85900294	3.803011676	2	High	6109	Unique	Used	76414	1	59.884	1	3.9644	0.0655	1	100	18/20	43.57193	6109	1470.81	MS2	735.907	270	Glioma_grade2_5.raw
4502277			dDMIFEDcGDVPSEPk	Unambiguous	N-Term(iTRAQ4plex); C8(Methylthio); K16(iTRAQ4plex)	HCD	0.5002013	0	1	1	0.48911	0.5206	0.481	0.512009	0.037262286	50968.973	47886.6	24929.31	24518.371	4.409598335	1.175293756	2	High	4510	Unique	Used	87038	1	60.172	1	5.5725	1.1435	1	100	19/30	21.19293	4510	2130.93	MS2	1065.97	29	Glioma_grade2_1rep.raw
4502277			vGNVEYFGLGNSPGFPLQYYPYYGk	Unambiguous	N-Term(iTRAQ4plex); K25(iTRAQ4plex)	HCD	0.662208	0	1	1	0.6063	0.658	0.6661	0.72287714	0.071953879	30525.73	28127.943	18507.885	20333.047	5.781364954	6.645486401	2	High	8142	Unique	Used	55402	1	72.316	1	3.8227	-0.8554	1	9	15/48	11.03254	8142	3117.56	MS2	1559.28	63	Glioma_grade2_2.raw
4502277			vAPPGLTQIPQIQk	Unambiguous	N-Term(iTRAQ4plex); K14(iTRAQ4plex)	HCD	0.4993696	0	1	1	0.49409	0.4491	0.5547	0.5041645	0.086636604	18502.434	20357.963	9141.938	10263.763	6.752669761	8.175433238	2	High	4552	Unique	Used	75030	1	47.052	1	3.0068	0.3664	1	100	15/26	26.58753	4552	1778.08	MS2	889.543	77	Glioma_grade2_5.raw
4502277			vGNVEYFGLGNSPGFPLQYYPYYGk	Unambiguous	N-Term(iTRAQ4plex); K25(iTRAQ4plex)	HCD	0.5296538	0	1	1	0.56331	0.5493	0.5095	0.49683332	0.059715825	11985.815	12291.483	6751.7446	6106.8184	1.780592845	7.093042813	2	High	8230	Unique	Used	55480	1	73.07	1	4.1347	-0.8554	1	19	18/48	6.127344	8230	3117.56	MS2	1559.28	71	Glioma_grade2_2.raw
4502277			vGNVEYFGLGNSPGFPLQYYPYYGk	Unambiguous	N-Term(iTRAQ4plex); K25(iTRAQ4plex)	HCD	0.45706686	0	1	1	0.36544	0.3621	0.5529	0.54785293	0.235778017	7487.692	7556.8296	2736.321	4140.0312	0.649906552	28.86917285	2	High	7031	Unique	Used	83713	1	71.578	1	3.8929	-0.1506	1	61	17/48	12.55918	7031	3117.56	MS2	1559.28	89	Glioma_grade2_6.raw
4502277			vGNVEYFGLGNSPGFPLQYYPYYGk	Unambiguous	N-Term(iTRAQ4plex); K25(iTRAQ4plex)	HCD	0.6576657	0	1	1	0.7401	0.7624	0.556	0.5727463	0.164951221	7433.9097	7216.2446	5501.827	4133.077	2.101171975	20.09054593	3	High	5643	Unique	Used	87876	1	72.6	1	7.9959	-0.4094	1	41	26/96	8.609827	5643	3117.56	MS2	1039.86	124	Glioma_grade2_1rep.raw
4502389	NP_003851.1	BANF1	barrier-to-autointegration factor [Homo sapiens]	2	2	2.791496754	24.54157644	10.622501	40.45	2.65E-05	89	10.052	6.0874	DNA binding protein	Biological_process unknown	DNA binding	Nucleus	Nucleolus																								
4502389			dFVAEPMGEkPVGSLAGIGEVLGk	Unambiguous	N-Term(iTRAQ4plex); K10(iTRAQ4plex); K24(iTRAQ4plex)	HCD	3.2759194	0	1	1	3.21835	3.1039	3.4542	3.331439	4.586262126	22364.135	23188.455	71975.69	77250.93	2.559161891	4.999319795	3	High	5912	Unique	Used	82853	1	60.037	1	6.0752	-0.6595	1	11	33/92	10.27413	5912	2832.55	MS2	944.856	71	Glioma_grade2_6.raw
4502389			aYVVLGQFLVLk	Unambiguous	N-Term(iTRAQ4plex); K12(iTRAQ4plex)	HCD	2.3070738	0	1	1	2.38722	2.3766	2.2372	2.2272813	3.754689589	3912.259	3929.6875	9339.43	8752.52	0.314304887	4.587764624	3	High	7665	Unique	Used	62951	1	70.58	1	4.5473	-3.7437	1	45	22/44	12.33012	7665	1638.02	MS2	546.677	53	Glioma_grade2_3.raw
4502551	NP_001210.1	CALU	calumenin isoform a precursor [Homo sapiens]	3	5	2.462156057	19.21377574	133.09362	54.29	1.49E-04	315	37.084	4.6401	Calcium binding protein	Cell communication,Signal transduction	Calcium ion binding	Endoplasmic reticulum	Mitochondrion, Nucleus, Extracellular, Golgi apparatus																								
4502551			dGFVTVDELk	Unambiguous	N-Term(iTRAQ4plex); K10(iTRAQ4plex)	HCD	2.462156	0	1	3	2.28853	2.4863	2.4399	2.6507916	6.047391571	75691.266	69669.07	173221.67	184678.19	5.85900631	4.526955099	2	High	4448	Unique	Used	51965	1	44.381	1	4.1417	1.5439	1	50	17/18	27.12616	4448	1410.77	MS2	705.891	299	Glioma_grade2_2.raw
4502551			iDGDkDGFVTVDELk	Unambiguous	N-Term(iTRAQ4plex); K5(iTRAQ4plex); K15(iTRAQ4plex)	HCD	2.7922459	1	1	3	2.61905	2.8504	2.7388	2.9807358	5.525090636	64793.5	59535.004	169697.66	177458.11	5.981441199	3.161385922	2	High	4068	Unique	Used	81271	1	43.897	1	5.272	-2.3079	1	29	20/28	2.540046	4068	2083.12	MS2	1042.07	209	Glioma_grade2_6.raw
4502551			iDGDkDGFVTVDELk	Unambiguous	N-Term(iTRAQ4plex); K5(iTRAQ4plex); K15(iTRAQ4plex)	HCD	2.2072575	1	1	3	2.11456	2.0898	2.3261	2.2988687	5.541318857	53908.406	54545.96	113992.734	125393.99	0.831351975	6.735465774	3	High	4025	Unique	Used	81235	1	43.506	1	4.5967	0.0297	1	16	23/56	13.49527	4025	2083.13	MS2	695.048	182	Glioma_grade2_6.raw
4502551			gHDLNEDGLVSWEEYk	Unambiguous	N-Term(iTRAQ4plex); K16(iTRAQ4plex)	HCD	1.6390983	0	1	3	1.38216	1.4959	1.7714	1.9171706	14.97617781	35833.914	33109.938	49528.348	63477.402	5.587566826	17.45658194	3	High	4710	Unique	Used	75160	1	48.55	1	5.5209	-1.3384	1	7	24/60	10.21404	4710	2179.05	MS2	727.022	111	Glioma_grade2_5.raw
4502551			gHDLNEDGLVSWEEYk	Unambiguous	N-Term(iTRAQ4plex); K16(iTRAQ4plex)	HCD	2.6313553	0	1	3	2.4864	2.6455	2.6181	2.785587	4.657736167	29001.664	27257.523	72109.7	75928.2	4.384329011	3.647832405	2	High	4704	Unique	Used	75155	1	48.492	1	6.4537	-0.744	1	65	22/30	29.6022	4704	2179.05	MS2	1090.03	106	Glioma_grade2_5.raw
4502673	NP_001768.1	CD47	leukocyte surface antigen CD47 isoform 1 precursor [Homo sapiens]	4	7	0.369160503	14.9419826	30.669699	8.67	5.00E-04	323	35.191	7.2104	Unclassified	Immune response	Molecular function unknown	Cell surface, Plasma membrane	-																								
4502673			eGETIIELk	Unambiguous	N-Term(iTRAQ4plex); K9(iTRAQ4plex)	HCD	0.36207968	0	1	2	0.32986	0.3417	0.3818	0.39544827	0.086654549	1217004.5	1174919.4	401447.16	464619.8	2.488261403	10.31555392	2	High	3245	Unique	Used	50830	1	35.649	1	4.1177	-2.5417	1	9	16/16	41.09034	3245	1319.76	MS2	660.385	301	Glioma_grade2_2.raw
4502673			aVEEPLNAFk	Unambiguous	N-Term(iTRAQ4plex); K10(iTRAQ4plex)	HCD	0.35064816	0	1	1	0.32584	0.3352	0.3656	0.37617165	0.068447204	924020.1	898122.25	301082.72	337848.12	2.010001617	8.137676889	2	High	3543	Unique	Used	51113	1	37.755	1	4.4694	-4.2342	1	10	17/18	17.59392	3543	1405.79	MS2	703.397	234	Glioma_grade2_2.raw
4502673			iEVSQLLk	Unambiguous	N-Term(iTRAQ4plex); K8(iTRAQ4plex)	HCD	0.45188978	0	1	2	0.44114	0.4233	0.4817	0.46220306	0.056066937	430755.75	448916.44	190024.33	207490.55	2.919621012	6.213846689	2	High	4528	Unique	Used	60058	1	45.683	1	3.6893	-8.1013	1	24	14/14	54.28724	4528	1217.76	MS2	609.384	189	Glioma_grade2_3.raw
4502673			iEVSQLLk	Unambiguous	N-Term(iTRAQ4plex); K8(iTRAQ4plex)	HCD	0.42166367	0	1	2	0.41446	0.4084	0.4351	0.42876124	0.029331003	413547.3	419676.62	171399.19	179941.06	1.040316686	3.438270566	2	High	4650	Unique	Used	52153	1	45.908	1	3.7528	-6.0965	1	24	14/14	25.82325	4650	1217.76	MS2	609.385	192	Glioma_grade2_2.raw
4502673			aVEEPLNAFk	Unambiguous	N-Term(iTRAQ4plex); K10(iTRAQ4plex)	HCD	0.3393134	0	1	1	0.28983	0.343	0.3362	0.39786756	0.129519851	349014.3	294953.8	101154.445	117352.55	11.87218626	10.48368258	2	High	3646	Unique	Used	51208	1	38.532	1	4.0734	-0.0662	1	19	17/18	28.85686	3646	1405.79	MS2	703.4	238	Glioma_grade2_2.raw
4502673			kAVEEPLNAFk	Unambiguous	N-Term(iTRAQ4plex); K1(iTRAQ4plex); K11(iTRAQ4plex)	HCD	0.4983092	1	1	1	0.48343	0.477	0.5199	0.512996	0.042635059	102791.87	104168.016	49692.24	53437.773	0.940358238	5.136219237	3	High	2348	Unique	Used	90124	1	29.679	1	5.6839	-9.273	1	18	21/40	51.23443	2348	1677.97	MS2	559.996	149	Glioma_grade2_7rep.raw
4502673			kAVEEPLNAFk	Unambiguous	N-Term(iTRAQ4plex); K1(iTRAQ4plex); K11(iTRAQ4plex)	HCD	0.3691605	1	1	1	0.31336	0.449	0.3134	0.44911918	0.205496453	82166.766	57339.51	25747.932	25752.275	25.16807355	0.011926029	3	High	2098	Unique	Used	79537	1	27.372	1	4.8831	13.642	1	23	19/40	55.4027	2098	1678.01	MS2	560.009	90	Glioma_grade2_6.raw
4502847	NP_001271.1	CIRBP	cold-inducible RNA-binding protein [Homo sapiens]	2	7	2.179893732	23.89707946	41.114061	20.35	2.67E-03	172	18.637	9.5103	RNA binding protein	Cell communication,Signal transduction	RNA binding	Nucleus	-																								
4502847			gFGFVTFENIDDAk	Unambiguous	N-Term(iTRAQ4plex); K14(iTRAQ4plex)	HCD	2.5421493	0	1	1	2.30397	2.801	2.3292	2.8317144	11.26027707	72375.59	59532.637	166751.02	168579.4	13.7691778	0.771096101	2	High	6823	Unique	Used	54196	1	61.81	1	6.1423	1.6313	1	30	22/26	17.05599	6823	1847.94	MS2	924.476	166	Glioma_grade2_2.raw
4502847			gFGFVTFENIDDAk	Unambiguous	N-Term(iTRAQ4plex); K14(iTRAQ4plex)	HCD	2.7408023	0	1	1	2.62618	2.8747	2.6185	2.866273	5.218926656	47683.914	43562.016	125226.414	124860.63	6.388497607	0.20684949	2	High	6576	Unique	Used	61962	1	61.652	1	6.4328	-0.6807	1	71	21/26	17.37192	6576	1847.94	MS2	924.474	174	Glioma_grade2_3.raw
4502847			gFGFVTFENIDDAk	Unambiguous	N-Term(iTRAQ4plex); K14(iTRAQ4plex)	HCD	1.6884136	0	1	1	1.63128	1.3249	2.1359	1.7348157	19.62105875	13102.547	16131.909	21373.963	27985.889	14.65450503	18.94388869	3	High	6842	Unique	Used	54214	1	61.951	1	4.4891	-1.5468	1	12	18/52	4.845573	6842	1847.94	MS2	616.651	164	Glioma_grade2_2.raw
4502847			gFGFVTFENIDDAk	Unambiguous	N-Term(iTRAQ4plex); K14(iTRAQ4plex)	HCD	1.8139656	0	1	1	1.87567	1.6611	1.9866	1.7593228	7.752789422	8122.572	9171.746	15235.234	16136.063	8.579442682	4.060922003	3	High	6582	Unique	Used	61968	1	61.692	1	5.4566	-1.3486	1	62	18/52	3.751555	6582	1847.94	MS2	616.651	155	Glioma_grade2_3.raw
4502847			lFVGGLSFDTNEQSLEQVFSk	Unambiguous	N-Term(iTRAQ4plex); K21(iTRAQ4plex)	HCD	2.811462	0	1	1	2.55175	2.7726	2.8473	3.0936513	7.933937087	6651.0684	6121.333	16971.848	18937.27	5.865451325	7.74044756	2	High	8169	Unique	Used	63394	1	75.058	1	5.9507	0.3585	1	100	23/40	0	8169	2633.37	MS2	1317.19	155	Glioma_grade2_3.raw
4502847			lFVGGLSFDTNEQSLEQVFSk	Unambiguous	N-Term(iTRAQ4plex); K21(iTRAQ4plex)	HCD	2.1798937	0	1	1	2.37525	1.5725	3.0974	2.0505612	28.16222175	4371.235	6602.8022	10382.785	13539.449	28.75799072	18.66128824	3	High	8175	Unique	Used	63399	1	75.114	1	5.7387	1.7672	1	14	27/80	11.12515	8175	2633.37	MS2	878.463	156	Glioma_grade2_3.raw
4502847			lFVGGLSFDTNEQSLEQVFSk	Unambiguous	N-Term(iTRAQ4plex); K21(iTRAQ4plex)	HCD	1.5325377	0	1	1	1.55907	1.5466	1.5184	1.5062157	1.595845329	3833.0974	3864.0603	5976.0767	5820.1084	0.568887306	1.869862869	3	High	8494	Unique	Used	55719	1	75.277	1	6.9039	-2.8219	1	62	26/80	32.15222	8494	2633.36	MS2	878.459	155	Glioma_grade2_2.raw

4502923	NP_001830.1	CNN3	calponin-3 [Homo sapiens]	2	3	3.197804213	17.38372233	13.346586	6.69	7.27E-05	329	36.391	6.0493	Cytoskeletal associated protein	Cell growth and/or maintenance	Cytoskeletal protein binding	Cytoplasm	Nucleus																								
4502923			gPSYGLSAEVk	Unambiguous	N-Term(iTRAQ4plex); K11(iTRAQ4plex)	HCD	2.5188894	0	1	3	2.40994	2.6007	2.4431	2.6364627	4.465510186	77580.82	71891.56	186964.98	189539.42	5.382819657	0.967002766	2	High	1867	Unique	Used	49533	1	25.842	1	3.8326	0.6316	1	64	19/20	24.12273	1867	1395.77	MS2	698.39	260	Glioma_grade 2_2.raw
4502923			dGIILcELINk	Unambiguous	N-Term(iTRAQ4plex); C6(Methylthio); K11(iTRAQ4plex)	HCD	3.5832782	0	1	1	3.65894	3.6186	3.5475	3.5084455	1.896494185	21485.527	21724.791	78614.35	76220.25	0.783077768	2.186700317	2	High	7903	Unique	Used	70780	1	72.855	1	4.7883	0.3525	1	73	18/20	18.68065	7903	1564.87	MS2	782.938	266	Glioma_grade 2_4.raw
4502923			dGIILcELINk	Unambiguous	N-Term(iTRAQ4plex); C6(Methylthio); K11(iTRAQ4plex)	HCD	3.1978042	0	1	1	3.10959	3.0565	3.3416	3.2845163	4.270087178	16784.184	17075.793	52191.85	56085.72	1.217949447	5.0857844	2	High	7918	Unique	Used	63178	1	72.747	1	4.7258	0.8206	1	100	18/20	13.83937	7918	1564.87	MS2	782.938	255	Glioma_grade 2_3.raw
4503015	NP_003900.1	CPNE3	copine-3 [Homo sapiens]	3	6	3.975733519	26.11190217	31.896209	8.57	2.65E-05	537	60.092	5.8462	Transport/cargo protein	Transport	Transporter activity	Cytoplasm	-																								
4503015			qTSDGNWLMVHR	Unambiguous	N-Term(iTRAQ4plex)	HCD	2.862294	0	1	1	2.58566	2.8027	2.9172	3.1621459	8.374108063	27599.111	25461.658	71362.04	80513.48	5.696892598	8.521511935	2	High	2577	Unique	Used	65921	1	29.933	1	4.0738	12.854	1	92	18/22	16.58322	2577	1587.8	MS2	794.405	152	Glioma_grade 2_4.raw
4503015			sDPYLEFHk	Unambiguous	N-Term(iTRAQ4plex); K9(iTRAQ4plex)	HCD	4.374232	0	1	1	3.73595	4.6129	4.1809	5.162354	13.77338895	23829.602	19299.176	89026.08	99629.18	14.85548673	7.948385761	3	High	1949	Unique	Used	72660	1	26.66	1	4.5602	8.5299	1	17	23/32	9.016302	1949	1423.76	MS2	475.258	216	Glioma_grade 2_5.raw
4503015			lYGPTNFSPIINHVAR	Unambiguous	N-Term(iTRAQ4plex)	HCD	2.659668	0	1	1	2.45639	3.0955	2.3138	2.9158454	13.73998798	19909.574	15798.69	48905.594	46066.54	16.28101525	4.227586032	3	High	4905	Unique	Used	75337	1	50.08	1	5.2058	0.6182	1	23	23/60	48.83559	4905	1943.06	MS2	648.359	173	Glioma_grade 2_5.raw
4503015			lYGPTNFSPIINHVAR	Unambiguous	N-Term(iTRAQ4plex)	HCD	5.222053	0	1	1	4.76966	5.7424	4.7898	5.7667127	10.68796314	15138.573	12574.145	72205.81	72511.48	13.08658666	0.298708371	2	High	4930	Unique	Used	75360	1	50.274	1	5.4165	-0.914	1	68	24/30	0	4930	1943.06	MS2	972.034	184	Glioma_grade 2_5.raw
4503015			sDPYLEFHk	Unambiguous	N-Term(iTRAQ4plex); K9(iTRAQ4plex)	HCD	3.5772352	0	1	1	3.69579	3.7139	3.4412	3.4580967	4.128488136	11373.805	11318.284	42035.22	39139.723	0.346017289	5.044476751	3	High	2024	Unique	Used	72729	1	27.228	1	4.3226	-2.9162	1	21	19/32	16.83888	2024	1423.74	MS2	475.252	201	Glioma_grade 2_5.raw
4503015			sDPYLEFHk	Unambiguous	N-Term(iTRAQ4plex); K9(iTRAQ4plex)	HCD	4.5569763	0	1	1	4.82356	3.9778	5.2594	4.3371387	12.17676601	7513.135	9110.678	36240.043	39514.273	13.59054615	6.112470836	3	High	1877	Unique	Used	72603	1	25.946	1	4.6042	-0.9227	1	49	23/32	8.990518	1877	1423.74	MS2	475.253	207	Glioma_grade 2_5.raw
4503057	NP_001876.1	CRYAB	alpha-crystallin B chain [Homo sapiens]	5	17	2.923062325	39.67830829	102.687	30.86	5.13E-05	175	20.146	7.3276	Heat shock protein	Protein metabolism	Heat shock protein activity	Nucleus	Golgi membrane																								
4503057			fSVNLDVk	Unambiguous	N-Term(iTRAQ4plex); K8(iTRAQ4plex)	HCD	6.043528	0	1	1	5.31625	6.5175	5.6569	6.9351387	12.26263355	2870666	2341576.2	1.53E+07	1.62E+07	14.35554877	4.390655697	2	High	4038	Unique	Used	51578	1	41.385	1	3.263	1.2111	1	1	14/14	5.484411	4038	1209.71	MS2	605.358	208	Glioma_grade 2_2.raw
4503057			fSVNLDVk	Unambiguous	N-Term(iTRAQ4plex); K8(iTRAQ4plex)	HCD	3.279556	0	1	1	3.17175	3.3321	3.2296	3.3928137	3.031731015	489692.56	466128.9	1553181.4	1581488.6	3.486430149	1.277085823	2	High	3945	Unique	Used	51491	1	40.665	1	3.594	-2.018	1	8	14/14	18.98923	3945	1209.71	MS2	605.357	232	Glioma_grade 2_2.raw
4503057			vLGDVIEVHGk	Unambiguous	N-Term(iTRAQ4plex); K11(iTRAQ4plex)	HCD	2.978486	0	1	1	2.87878	2.9361	3.0201	3.0801766	2.990945185	326796.62	320421.34	940775.4	986954.3	1.393040366	3.387758495	2	High	2441	Unique	Used	79858	1	29.911	1	4.7135	6.0485	1	9	18/20	9.703692	2441	1453.87	MS2	727.439	157	Glioma_grade 2_6.raw
4503057			vLGDVIEVHGk	Unambiguous	N-Term(iTRAQ4plex); K11(iTRAQ4plex)	HCD	5.4773626	0	1	1	4.45249	6.1537	4.988	6.893821	19.67326732	318890.34	230732.61	1419855	1590629.2	22.68352466	8.022337063	2	High	2545	Unique	Used	73217	1	31.027	1	4.3447	12.597	1	5	18/20	3.79921	2545	1453.88	MS2	727.444	132	Glioma_grade 2_5.raw
4503057			vLGDVIEVHGk	Unambiguous	N-Term(iTRAQ4plex); K11(iTRAQ4plex)	HCD	4.1129107	0	1	1	4.00906	3.9578	4.2701	4.2154293	3.715384461	291205.62	294979.66	1167461.9	1243465.9	0.910513917	4.458278991	3	High	2440	Unique	Used	79857	1	29.908	1	3.8886	6.2701	1	1	27/40	11.36327	2440	1453.87	MS2	485.295	152	Glioma_grade 2_6.raw
4503057			vLGDVIEVHGk	Unambiguous	N-Term(iTRAQ4plex); K11(iTRAQ4plex)	HCD	5.45485	0	1	1	5.49361	5.2825	5.6341	5.4175787	2.691037589	276716.53	287774.4	1520172.9	1559040.6	2.770317271	1.785106115	3	High	2543	Unique	Used	73215	1	31.018	1	3.7351	12.315	1	1	27/40	4.656571	2543	1453.88	MS2	485.298	77	Glioma_grade 2_5.raw
4503057			fSVNLDVk	Unambiguous	N-Term(iTRAQ4plex); K8(iTRAQ4plex)	HCD	2.4147642	0	1	1	2.32072	2.5039	2.3321	2.516233	4.394173602	240384.48	222794.92	557865.1	560603.94	5.370574406	0.346304147	2	High	4144	Unique	Used	51678	1	42.159	1	3.0448	-1.1098	1	13	14/14	18.84889	4144	1209.71	MS2	605.357	246	Glioma_grade 2_2.raw
4503057			rPFFPFHSPSR	Unambiguous	N-Term(iTRAQ4plex)	HCD	4.475066	0	1	1	4.60999	4.3166	4.6443	4.348733	3.818944866	82192.43	87778.44	378905.88	381724.97	4.647744111	0.524143257	2	High	2539	Unique	Used	85795	1	35.567	1	3.8461	-5.8955	1	7	14/20	5.465343	2539	1518.8	MS2	759.904	182	Glioma_grade 2_1rep.raw
4503057			fSVNLDVk	Unambiguous	N-Term(iTRAQ4plex); K8(iTRAQ4plex)	HCD	2.3785744	0	1	1	2.46146	2.3143	2.4469	2.3006432	3.573890625	79236.414	84273.9	195037.28	193884.17	4.35696125	0.419299013	2	High	3878	Unique	Used	59456	1	40.574	1	3.2818	-0.5044	1	41	14/14	30.82713	3878	1209.71	MS2	605.357	240	Glioma_grade 2_3.raw
4503057			aPSWFDTGLSEMR	Unambiguous	N-Term(iTRAQ4plex)	HCD	2.5949507	0	1	1	2.25871	2.8504	2.3926	3.019264	13.7839064	72116.35	57147.11	162890.11	172542.2	16.37717436	4.069410184	2	High	4631	Unique	Used	87126	1	61.514	1	4.5544	0.8635	1	38	17/24	32.28549	4631	1640.79	MS2	820.897	144	Glioma_grade 2_1rep.raw
4503057			vLGDVIEVHGk	Unambiguous	N-Term(iTRAQ4plex); K11(iTRAQ4plex)	HCD	2.3887112	0	1	1	2.38038	2.4053	2.3723	2.39713	0.633549176	69618.23	68896.01	165717.83	165152.69	0.737377846	0.241553298	2	High	2596	Unique	Used	80003	1	31.053	1	4.6072	4.1173	1	37	18/20	28.09154	2596	1453.87	MS2	727.438	175	Glioma_grade 2_6.raw
4503057			vLGDVIEVHGk	Unambiguous	N-Term(iTRAQ4plex); K11(iTRAQ4plex)	HCD	3.5620687	0	1	1	4.13123	2.8544	4.5862	3.1688101	21.97612884	60593.324	87696.83	250325.12	277894.6	25.848072	7.381233802	3	High	2585	Unique	Used	79992	1	30.988	1	4.1758	5.7034	1	6	27/40	14.80593	2585	1453.87	MS2	485.295	97	Glioma_grade 2_6.raw
4503057			rPFFPFHSPSR	Unambiguous	N-Term(iTRAQ4plex)	HCD	2.6469572	0	1	1	2.49906	2.6004	2.6917	2.8008523	4.862254721	26145.99	25127.479	65340.324	70378.35	2.809234674	5.249715818	2	High	2751	Unique	Used	95323	1	38.86	1	3.8289	0.8558	1	41	15/20	3.133906	2751	1518.81	MS2	759.909	203	Glioma_grade 2_8rep.raw
4503057			rPFFPFHSPSR	Unambiguous	N-Term(iTRAQ4plex)	HCD	1.6124451	0	1	1	1.61383	1.6819	1.5458	1.6110066	3.44473665	16045.043	15395.762	25893.898	24802.674	2.920478645	3.044039704	2	High	2652	Unique	Used	95266	1	37.515	1	3.4321	1.338	1	100	14/20	24.39606	2652	1518.81	MS2	759.909	277	Glioma_grade 2_8rep.raw
4503057			eEkPAVTAAPk	Unambiguous	N-Term(iTRAQ4plex); K3(iTRAQ4plex); K11(iTRAQ4plex)	HCD	2.8472123	0	1	1	2.84138	2.7602	2.9368	2.8528817	2.537145467	4871.591	5014.875	13842.022	14306.845	2.049611824	2.33529467	3	High	923	Unique	Used	78466	1	18.282	1	4.967	-1.1687	1	100	24/40	37.19212	923	1572.93	MS2	524.982	136	Glioma_grade 2_6.raw
4503057			rPFFPFHSPSR	Unambiguous	N-Term(iTRAQ4plex)	HCD	1.7046356	0	1	1	1.40831	1.8807	1.5728	2.1003542	17.78983727	2496.8467	1869.6876	3516.3435	3927.0063	20.3121479	7.802466859	4	High	2662	Unique	Used	85879	1	37.011	1	3.6814	0.597	1	62	19/60	8.095489	2662	1518.81	MS2	380.458	244	Glioma_grade 2_1rep.raw
4503057			rPFFPFHSPSR	Unambiguous	N-Term(iTRAQ4plex)	HCD	2.9230623	0	1	1	3.15095	3.5674	2.3539	2.6650484	18.21623417	2116.7722	1869.6455	6669.852	4982.696	8.767017334	20.47619883	4	High	2466	Unique	Used	85747	1	34.676	1	4.536	4.6156	1	26	19/60	7.616902	2466	1518.82	MS2	380.459	221	Glioma_grade 2_1rep.raw
4503065	NP_001879.1	CRYM	thiomorpholine-carboxylate dehydrogenase isoform 1 [Homo sapiens]	2	6	0.319060564	37.09263714	45.004388	18.79	8.95E-05	314	33.754	5.1353	Enzyme: Deaminase	Hormone metabolism,Osmoregulation	Hormone binding	Cytoplasm	-																								
4503065			vPAFLSAAEVEEHLR	Unambiguous	N-Term(iTRAQ4plex)	HCD	0.34475204	0	1	1	0.41759	0.3656	0.3209	0.28097558	0.169773775	311475.12	355741.1	130069.586	99954.56	9.382498115	18.51504676	3	High	6333	Unique	Used	69393	1	59.268	1	6.2993	1.1981	1	1	31/56	8.729092	6333	1811.98	MS2	604.665	190	Glioma_grade 2_4.raw
4503065			lIYDSWSSGk	Unambiguous	N-Term(iTRAQ4plex); K10(iTRAQ4plex)	HCD	0.29336908	0	1	2	0.2745	0.2779	0.3086	0.31246972	0.067940665	225068.19	222297.06	61782.03	69461.1	0.876011185	8.27460069	2	High	4199	Unique	Used	59755	1	43.065	1	4.1806	-1.3271	1	44	14/18	39.31303	4199	1443.77	MS2	722.389	197	Glioma_grade 2_3.raw
4503065			vPAFLSAAEVEEHLR	Unambiguous	N-Term(iTRAQ4plex)	HCD	0.2761298	0	1	1	0.2461	0.2549	0.2967	0.30722675	0.109441323	131281.64	126762.734	32308.842	38944.902	2.476588834	13.17096552	2	High	6323	Unique	Used	69384	1	59.18	1	4.5047	2.4519	1	41	19/28	9.329379	6323	1811.98	MS2	906.495	164	Glioma_grade 2_4.raw
4503065			vPAFLSAAEVEEHLR	Unambiguous	N-Term(iTRAQ4plex)	HCD	0.2849069	0	1	1	0.26907	0.2843	0.2855	0.30163693	0.046666186	106667.45	100954.03	28701.328	30451.465	3.891695624	4.184193773	3	High	5864	Unique	Used	76197	1	57.858	1	6.649	-0.4187	1	4	28/56	7.062255	5864	1811.98	MS2	604.664	192	Glioma_grade 2_5.raw
4503065			vPAFLSAAEVEEHLR	Unambiguous	N-Term(iTRAQ4plex)	HCD	0.63753057	0	1	1	0.53372	0.5981	0.6728	0.7538594	0.148574968	20465.182	18263.398	10922.619	13768.033	8.040038634	16.29775945	3	High	6248	Unique	Used	69314	1	58.609	1	6.7314	-1.025	1	38	29/56	31.97081	6248	1811.98	MS2	604.663	185	Glioma_grade 2_4.raw
4503065			vPAFLSAAEVEEHLR	Unambiguous	N-Term(iTRAQ4plex)	HCD	0.45243406	0	1	1	0.46986	0.4439	0.4615	0.4359686	0.034460829	16117.883	17062.348	7573.218	7438.648	4.025515109	1.267735264	3	High	5775	Unique	Used	76116	1	57.155	1	5.4733	-2.2377	1	46	28/56	50.91911	5775	1811.97	MS2	604.663	180	Glioma_grade 2_5.raw
4503183	NP_001905.1	CYB5A	cytochrome b5 isoform 2 [Homo sapiens]	2	7	2.188870907	16.1944673	37.320946	36.73	1.90E-04	98	11.261	5.1353	Enzyme: Oxidoreductase	Metabolism,Energy pathways	Oxidoreductase activity	Mitochondrion	-																								
4503183			fLEEHPGGEEVLR	Unambiguous	N-Term(iTRAQ4plex)	HCD	2.188871	0	1	3	2.21317	2.1965	2.1812	2.1647544	0.947472198	155252.28	156428.22	343599.7	338628.66	0.533568926	1.0304632	3	High	3234	Unique	Used	66533	1	34.913	1	3.356	-2.1609	1	3	20/48	11.50883	3234	1655.85	MS2	552.621	172	Glioma_grade 2_4.raw
4503183			fLEEHPGGEEVLR	Unambiguous	N-Term(iTRAQ4plex)	HCD	2.1008885	0	1	3	1.97821	1.9306	2.2753	2.2206206	8.188136108	113048.91	115834.27	223634.05	257223.97	1.721006274	9.878866203	3	High	2975	Unique	Used	58614	1	33.715	1	3.2961	-1.8291	1	5	20/48	23.69159	2975	1655.85	MS2	552.621	179	Glioma_grade 2_3.raw
4503183			fLEEHPGGEEVLR	Unambiguous	N-Term(iTRAQ4plex)	HCD	2.9792266	0	1	3	2.574	3.0222	2.9427	3.4550047	12.05968248	94881.39	80811.234	244224.95	279203.2	11.32557815	9.450526407	2	High	2961	Unique	Used	58601	1	33.608	1	4.2563	-2.5951	1	17	16/24	23.45152	2961	1655.85	MS2	828.427	197	Glioma_grade 2_3.raw
4503183			tFIIGELHPDDRPk	Unambiguous	N-Term(iTRAQ4plex); K14(iTRAQ4plex)	HCD	2.1861258	0	1	2	1.87565	2.2626	2.1227	2.5606637	12.95953467	86145.984	71412.15	161579.39	182862.5	13.22482524	8.738444331	3	High	2904	Unique	Used	90597	1	34.584	1	5.7583	-0.553	1	14	25/52	22.49787	2904	1926.07	MS2	642.694	134	Glioma_grade 2_7rep.raw
4503183			fLEEHPGGEEVLR	Unambiguous	N-Term(iTRAQ4plex)	HCD	1.9119745	0	1	3	1.87743	1.8865	1.9374	1.9466869	1.835312635	56841.69	56569.55	106716.21	110123.2	0.339352677	2.222015133	2	High	3264	Unique	Used	66562	1	35.115	1	3.8582	-0.5309	1	37	16/24	28.12859	3264	1655.85	MS2	828.429	197	Glioma_grade 2_4.raw
4503183			tFIIGELHPDDRPk	Unambiguous	N-Term(iTRAQ4plex); K14(iTRAQ4plex)	HCD	2.6958501	0	1	2	2.42805	2.8651	2.5525	3.0118496	9.962905925	24910.947	21111.316	60485.02	63584.11	11.67584847	3.532526672	2	High	2945	Unique	Used	90632	1	34.937	1	5.0501	-5.4579	1	78	21/26	12.38386	2945	1926.06	MS2	963.533	125	Glioma_grade 2_7rep.raw
4503183			tFIIGELHPDDRPk	Unambiguous	N-Term(iTRAQ4plex); K14(iTRAQ4plex)	HCD	2.6367087	0	1	2	2.29285	2.3857	2.878	2.9944875	13.26840211	10270.455	9870.86	23548.613	29558.166	2.805738694	16.00321374	4	High	2895	Unique	Used	90590	1	34.492	1	4.0315	-0.7818	1	32	23/78	39.57166	2895	1926.07	MS2	482.272	136	Glioma_grade 2_7rep.raw
4503471	NP_001393.1	EEF1A1	elongation factor 1-alpha 1 [Homo sapiens]	3	9	2.091904402	16.45514066	164.54001	27.49	1.05E-04	462	50.109	9.0122	Transcription regulatory protein	Regulation of cell cycle	Transcription regulator activity	Cytoplasm	Mitochondrion, Ribosome, Nucleolus, Nucleus																								
4503471			yYVTIIDAPGHR	Unambiguous	N-Term(iTRAQ4plex)	HCD	1.7150261	0	1	1	1.65089	1.6635	1.7662	1.7796489	3.920869224	187231.2	185815.66	309098.47	330686.62	0.536628526	4.771954676	2	High	3625	Unique	Used	51188	1	38.386	1	3.8856	-2.3484	1	21	14/22	33.542	3625	1548.83	MS2	774.916	292	Glioma_grade 2_2.raw
4503471			yYVTIIDAPGHR	Unambiguous	N-Term(iTRAQ4plex)	HCD	2.0919044	0	1	1	1.96316	2.1584	2.0314	2.2334585	5.815913668	179035.22	162839.72	351473.97	363695.75	6.699495362	2.416797936	2	High	3593	Unique	Used	59191	1	38.393	1	3.918	1.0406	1	18	15/22	22.96909	3593	1548.83	MS2	774.919	307	Glioma_grade 2_3.raw
4503471			vETGVLkPGMVVTFAPVNVTTEVk	Unambiguous	N-Term(iTRAQ4plex); K7(iTRAQ4plex); K24(iTRAQ4plex)	HCD	2.552279	0	1	1	2.41184	2.9999	2.1924	2.7269666	13.71548137	150718.33	121171.45	363508.1	330430.53	15.36858003	6.741049724	3	High	5559	Unique	Used	82543	1	57.085	1	8.155	1.0117	1	2	29/92	3.847005	5559	2947.69	MS2	983.236	37	Glioma_grade 2_6.raw
4503471			yYVTIIDAPGHR	Unambiguous	N-Term(iTRAQ4plex)	HCD	1.6073768	0	1	1	1.62821	1.5437	1.6745	1.5876224	3.476000481	94503.84	99676.14	153872.33	158248.08	3.766988136	1.982646696	3	High	3825	Unique	Used	67079	1	39.573	1	3.5397	0.3226	1	10	19/44	31.32184	3825	1548.83	MS2	516.948	233	Glioma_grade 2_4.raw
4503471			yYVTIIDAPGHR	Unambiguous	N-Term(iTRAQ4plex)	HCD	1.863594	0	1	1	1.92654	1.9085	1.8183	1.8012421	3.381254882	65960.33	66585.37	127075	119936.37	0.666894547	4.087078001	3	High	3591	Unique	Used	59189	1	38.384	1	4.7102	-0.2685	1	3	22/44	6.057091	3591	1548.83	MS2	516.948	220	Glioma_grade 2_3.raw
4503471			yYVTIIDAPGHR	Unambiguous	N-Term(iTRAQ4plex)	HCD	1.7541072	0	1	1	1.64222	1.689	1.8174	1.869184	6.069202446	59221.496	57582.004	97254.51	107631.36	1.985036255	7.162564214	3	High	3622	Unique	Used	51185	1	38.369	1	5.3154	-3.5787	1	5	22/44	10.20515	3622	1548.82	MS2	516.946	227	Glioma_grade 2_2.raw
4503471			yYVTIIDAPGHR	Unambiguous	N-Term(iTRAQ4plex)	HCD	2.2642837	0	1	1	2.31181	2.2118	2.3192	2.2188127	2.559860119	38055.867	39776.92	87977.98	88257.53	3.127135219	0.224326755	2	High	3818	Unique	Used	67073	1	39.503	1	3.9451	1.1983	1	100	16/22	24.7732	3818	1548.83	MS2	774.919	234	Glioma_grade 2_4.raw
4503471			vETGVLkPGMVVTFAPVNVTTEVk	Unambiguous	N-Term(iTRAQ4plex); K7(iTRAQ4plex); K24(iTRAQ4plex)	HCD	2.466667	0	1	1	2.60947	2.5724	2.3594	2.3258927	5.869138859	12691.787	12874.816	33118.855	29945.441	1.012422707	7.116364413	2	High	5551	Unique	Used	82537	1	56.997	1	4.868	-0.7023	1	37	21/46	6.794603	5551	2947.69	MS2	1474.35	35	Glioma_grade 2_6.raw
4503471			dGNASGTTLLEALDcILPPTRPTDkPLR	Unambiguous	N-Term(iTRAQ4plex); C15(Methylthio); K25(iTRAQ4plex)	HCD	2.1202419	0	1	1	2.17028	1.9078	2.362	2.0762556	8.899848036	6289.683	7155.2134	13650.38	14856.053	9.10415963	5.9813836	3	High	6502	Unique	Used	93252	1	72.56	1	5.4175	1.5881	1	31	25/108	34.42066	6502	3298.74	MS2	1100.25	37	Glioma_grade 2_7rep.raw
4503475	NP_001949.1	EEF1A2	elongation factor 1-alpha 2 [Homo sapiens]	2	5	0.424885482	10.48394702	142.30488	27.43	1.26E-04	463	50.438	9.0269	Translation regulatory protein	Protein metabolism	Translation regulator activity	Cytoplasm	Nucleolus																								
4503475			yYITIIDAPGHR	Unambiguous	N-Term(iTRAQ4plex)	HCD	0.40906465	0	1	1	0.39136	0.4233	0.3959	0.42820936	0.045766732	176774.16	163432.9	69183.18	69983.5	5.545855172	0.813286196	2	High	4226	Unique	Used	59780	1	43.279	1	4.1664	2.1869	1	58	16/22	47.17227	4226	1562.85	MS2	781.928	238	Glioma_grade 2_3.raw
4503475			yYITIIDAPGHR	Unambiguous	N-Term(iTRAQ4plex)	HCD	0.42488548	0	1	1	0.38375	0.4008	0.448	0.46784553	0.092633034	123195.586	117961.92	47276.37	55187.957	3.069164863	10.91957949	2	High	4542	Unique	Used	67747	1	45.09	1	3.9927	1.3277	1	78	15/22	60.73923	4542	1562.85	MS2	781.927	242	Glioma_grade 2_4.raw
4503475			yYITIIDAPGHR	Unambiguous	N-Term(iTRAQ4plex)	HCD	0.46048054	0	1	1	0.44057	0.5071	0.42	0.48340327	0.085700249	91146.805	79184.516	40156.203	38278.055	9.931955698	3.386405942	3	High	4536	Unique	Used	67741	1	45.053	1	3.9499	0.7724	1	15	16/44	49.96581	4536	1562.85	MS2	521.62	230	Glioma_grade 2_4.raw
4503475			yYITIIDAPGHR	Unambiguous	N-Term(iTRAQ4plex)	HCD	0.50688195	0	1	1	0.50574	0.4972	0.5167	0.508009	0.01582373	40257.023	40947.543	20359.406	20801.72	1.202571231	1.519702006	3	High	4203	Unique	Used	59759	1	43.091	1	5.161	1.944	1	58	17/44	52.34008	4203	1562.85	MS2	521.621	223	Glioma_grade 2_3.raw
4503475			vETGILRPGMVVTFAPVNITTEVk	Unambiguous	N-Term(iTRAQ4plex); K24(iTRAQ4plex)	HCD	0.39114612	0	1	1	0.47119	0.4613	0.3195	0.31277832	0.22194788	28775.217	29389.32	13558.491	9192.343	1.4931311	27.14039282	3	High	6472	Unique	Used	76731	1	62.999	1	7.323	-5.4111	1	11	29/92	11.40017	6472	2859.61	MS2	953.875	39	Glioma_grade 2_5.raw

4503841	NP_001460.1	XRCC6	X-ray repair cross-complementing protein 6 [Homo sapiens]	8	10	2.062315941	16.45008062	72.761587	21.02	1.71E-04	609	69.799	6.6392	DNA binding protein	Regulation of nucleobase, nucleoside, nucleotide and nucleic acid metabolism	DNA binding	Nucleus	Cytoplasm, Nucleolus																								
4503841			lGSLVDEFk	Unambiguous	N-Term(iTRAQ4plex); K9(iTRAQ4plex)	HCD	2.1583636	0	1	1	2.02667	2.2835	2.0473	2.3067546	6.906556311	261490.2	232074.89	529953.1	535339.8	8.428377774	0.715103254	2	High	5796	Unique	Used	61232	1	55.612	1	3.9714	-0.3186	1	16	16/16	10.96429	5796	1295.74	MS2	648.376	282	Glioma_grade2_3.raw
4503841			lGSLVDEFk	Unambiguous	N-Term(iTRAQ4plex); K9(iTRAQ4plex)	HCD	2.04929	0	1	1	2.03859	2.1127	1.9881	2.0603778	2.517615534	166295.38	160465.1	339008.25	330618.72	2.523334844	1.771820378	2	High	5986	Unique	Used	53412	1	55.621	1	3.8708	-1.4491	1	24	16/16	17.82007	5986	1295.74	MS2	648.375	282	Glioma_grade2_2.raw
4503841			kQELLEALTk	Unambiguous	N-Term(iTRAQ4plex); K1(iTRAQ4plex); K10(iTRAQ4plex)	HCD	2.147558	1	1	1	1.98017	2.0846	2.2074	2.3237782	6.93377632	116624.25	110781.99	230935.34	257432.77	3.633235098	7.67311053	2	High	3316	Unique	Used	90937	1	38.37	1	4.7437	1.9253	1	60	17/18	26.79235	3316	1605	MS2	803.003	78	Glioma_grade2_7rep.raw
4503841			iMATPEQVGk	Unambiguous	N-Term(iTRAQ4plex); K10(iTRAQ4plex)	HCD	1.5432694	0	1	1	1.47628	1.5272	1.5588	1.6125687	3.700056607	87177.84	84270.31	128699.02	135891.67	2.398315968	3.844407065	2	High	1757	Unique	Used	57479	1	24.663	1	3.6165	0.7479	1	52	14/18	0	1757	1361.77	MS2	681.389	293	Glioma_grade2_3.raw
4503841			kPGGFDISLFYR	Unambiguous	N-Term(iTRAQ4plex); K1(iTRAQ4plex)	HCD	2.0770752	0	1	1	1.8774	2.2881	1.9039	2.3204336	11.41419004	68416.15	56135.57	128444.44	130258.87	13.94389639	0.991866518	3	High	4855	Unique	Used	92105	1	54.142	1	5.3979	-1.2858	1	15	23/44	24.20068	4855	1687.94	MS2	563.318	162	Glioma_grade2_7rep.raw
4503841			eLVYPPDYNPEGk	Unambiguous	N-Term(iTRAQ4plex); K13(iTRAQ4plex)	HCD	2.075342	0	1	1	1.98224	2.0287	2.1209	2.1706305	4.125468727	28314.38	27665.627	56125.8	60051.85	1.638933863	4.779123314	2	High	3773	Unique	Used	51329	1	39.428	1	3.5265	0.2182	1	100	13/24	17.57329	3773	1808.93	MS2	904.969	219	Glioma_grade2_2.raw
4503841			dTGIFLDLmHLk	Unambiguous	N-Term(iTRAQ4plex); M9(Oxidation); K12(iTRAQ4plex)	HCD	1.5345312	0	1	1	1.44278	1.7087	1.3875	1.6431906	10.00354161	21303.559	17988.025	30736.41	29557.754	11.93353047	2.76456491	3	High	5294	Unique	Used	82331	1	54.516	1	4.2744	1.1722	1	36	18/44	35.49743	5294	1706.94	MS2	569.652	237	Glioma_grade2_6.raw
4503841			iQVTPPGFQLVFLPFADDk	Unambiguous	N-Term(iTRAQ4plex); K19(iTRAQ4plex)	HCD	2.5361574	0	1	1	2.63396	2.543	2.5291	2.4417362	3.101547512	20062.146	20780.016	52842.83	50739.32	2.485719267	2.871935339	2	High	8300	Unique	Used	71119	1	76.433	1	3.9904	-0.6713	1	29	18/36	0	8300	2420.35	MS2	1210.68	121	Glioma_grade2_4.raw
4503841			dTGIFLDLMHLk	Unambiguous	N-Term(iTRAQ4plex); K12(iTRAQ4plex)	HCD	1.9451116	0	1	1	1.75173	1.9514	1.9395	2.1605315	8.564578938	13764.286	12355.91	24111.365	26695.334	7.625304344	7.192523971	3	High	6374	Unique	Used	83210	1	64.758	1	5.5346	-3.8453	1	100	27/44	10.38712	6374	1690.94	MS2	564.317	212	Glioma_grade2_6.raw
4503841			nIPPYFVALVPQEEELDDQk	Unambiguous	N-Term(iTRAQ4plex); K20(iTRAQ4plex)	HCD	1.5805707	0	1	1	1.39514	1.6054	1.559	1.7939509	10.32941068	3969.2925	3449.3855	5537.717	6188.0283	9.910923895	7.843246094	3	High	7536	Unique	Used	54852	1	67.371	1	4.602	3.8124	1	43	23/76	23.17902	7536	2632.39	MS2	878.133	119	Glioma_grade2_2.raw
4503911	NP_002036.1	GAP43	neuromodulin isoform 2 [Homo sapiens]	3	3	0.497784734	16.50632927	45.46987	42.86	4.24E-04	238	24.788	4.7163	Growth factor	Cell communication,Signal transduction	Growth factor activity	Cytoplasm	-																								
4503911			kDDVQAAEAEANk	Unambiguous	N-Term(iTRAQ4plex); K1(iTRAQ4plex); K13(iTRAQ4plex)	HCD	0.3857967	1	1	1	0.35272	0.4052	0.3689	0.4237923	0.084085292	119152.26	103713.07	42027.902	43952.805	9.797087726	3.166086933	3	High	1036	Unique	Used	78555	1	19.425	1	6.5195	-5.2682	1	26	25/48	54.56086	1036	1820.96	MS2	607.659	173	Glioma_grade2_6.raw
4503911			aTAQPPTETGESSQAEENIEAVDETkPk	Unambiguous	N-Term(iTRAQ4plex); K26(iTRAQ4plex); K28(iTRAQ4plex)	HCD	0.49778473	0	1	2	0.50825	0.5025	0.493	0.48743987	0.018786843	8077.5894	8169.1733	4105.4097	3981.9807	0.797200008	2.158359584	3	High	3073	Unique	Used	73706	1	35.009	1	5.5251	0.6038	1	25	29/108	5.784709	3073	3389.69	MS2	1130.57	89	Glioma_grade2_5.raw
4503911			qDEGkEEEPEADQEHA	Unambiguous	N-Term(iTRAQ4plex); K5(iTRAQ4plex)	HCD	0.53612715	1	1	2	0.41909	0.5831	0.5024	0.6989535	0.216526491	2389.2	1717.2491	1001.30145	1200.2773	23.14121161	12.78147991	3	High	923	Unique	Used	64394	1	17.499	1	3.8479	-0.0979	1	100	21/60	11.83447	923	2128.95	MS2	710.322	16	Glioma_grade2_4.raw
4503979	NP_002046.1	GFAP	glial fibrillary acidic protein isoform 1 [Homo sapiens]	15	46	4.7569499	39.24283965	492.6367	45.37	6.56E-05	432	49.85	5.5161	Structural protein	Cell growth and/or maintenance	Structural molecule activity	Cytoplasm	-																								
4503979			fASYIEk	Unambiguous	N-Term(iTRAQ4plex); K7(iTRAQ4plex)	HCD	9.721589	0	1	3	8.86056	10.196	9.309	10.712435	8.583505375	1.06E+07	9240526	9.42E+07	9.90E+07	9.913253687	3.490340375	2	High	2772	Unique	Used	50388	1	32.182	1	2.9604	-0.4616	1	0	12-Dec	6.899364	2772	1145.64	MS2	573.326	170	Glioma_grade2_2.raw
4503979			lEEEGQSLk	Unambiguous	N-Term(iTRAQ4plex); K9(iTRAQ4plex)	HCD	4.2671185	0	1	3	3.91557	4.3178	4.2212	4.654781	7.118577798	237554.92	215425.05	930162.9	1002756.3	6.908994737	5.311270684	2	High	1430	Unique	Used	49118	1	22.869	1	3.8954	-1.3365	1	9	16/16	35.49528	1430	1320.72	MS2	660.865	269	Glioma_grade2_2.raw
4503979			kIESLEEEIR	Unambiguous	N-Term(iTRAQ4plex); K1(iTRAQ4plex)	HCD	2.405935	1	1	3	2.30417	2.4765	2.3403	2.515318	4.258963809	86724.53	80688.45	199827.69	202957.11	5.098951228	1.098767433	2	High	2171	Unique	Used	79605	1	27.914	1	3.6273	4.1585	1	49	16/18	6.702128	2171	1533.88	MS2	767.443	260	Glioma_grade2_6.raw
4503979			lADVYQAELR	Unambiguous	N-Term(iTRAQ4plex)	HCD	5.9194794	0	1	3	5.39839	5.3376	6.508	6.434709	10.78082927	60452.254	61140.383	326344.56	393420.6	0.800345636	13.17927718	2	High	3839	Unique	Used	74385	1	41.335	1	3.3624	1.1385	1	21	17/18	29.98204	3839	1321.72	MS2	661.366	337	Glioma_grade2_5.raw
4503979			eAASYQEALAR	Unambiguous	N-Term(iTRAQ4plex)	HCD	5.5418267	0	1	3	5.22182	5.1178	5.9745	5.855458	7.849354604	59834.074	61050.44	312442.75	357478.28	1.423012127	9.50706941	2	High	1502	Unique	Used	72255	1	23.186	1	3.7139	4.6706	1	16	18/20	14.24496	1502	1352.7	MS2	676.853	229	Glioma_grade2_5.raw
4503979			tVEMRDGEVIk	Unambiguous	N-Term(iTRAQ4plex); K11(iTRAQ4plex)	HCD	7.324713	1	1	1	7.28219	6.9549	7.7119	7.365327	4.243376086	55818.97	58445.887	406484.25	430473.06	3.251237297	4.053408703	2	High	1310	Unique	Used	64746	1	20.523	1	3.6431	1.5589	1	6	16/20	8.656992	1310	1564.86	MS2	782.935	304	Glioma_grade2_4.raw
4503979			kIESLEEEIR	Unambiguous	N-Term(iTRAQ4plex); K1(iTRAQ4plex)	HCD	3.7663476	1	1	3	3.55476	4.1532	3.4352	4.013563	9.181838835	51468.88	44052.18	182959.3	176806.22	10.98061279	2.418733509	2	High	2880	Unique	Used	80229	1	33.854	1	3.7269	0.816	1	61	17/18	40.67247	2880	1533.87	MS2	767.441	292	Glioma_grade2_6.raw
4503979			kIESLEEEIR	Unambiguous	N-Term(iTRAQ4plex); K1(iTRAQ4plex)	HCD	3.6915703	1	1	3	3.27449	3.6661	3.7143	4.158529	9.768497656	49522.664	44232.793	162161.52	183943.34	7.979276674	8.900234818	2	High	2799	Unique	Used	73456	1	32.859	1	3.3945	0.4181	1	25	17/18	21.47136	2799	1533.87	MS2	767.44	243	Glioma_grade2_5.raw
4503979			lEAENNLAAYR	Unambiguous	N-Term(iTRAQ4plex)	HCD	7.725389	0	1	3	6.31996	7.786	7.6762	9.456838	16.44899978	49326.43	40038.73	311740.88	378639.78	14.6978882	13.70393714	2	High	2940	Unique	Used	66260	1	32.654	1	4.1364	-0.9492	1	24	19/20	14.78204	2940	1407.73	MS2	704.37	199	Glioma_grade2_4.raw
4503979			hLQEYQDLLNVk	Unambiguous	N-Term(iTRAQ4plex); K12(iTRAQ4plex)	HCD	3.62461	0	1	3	3.09134	4.0706	3.2859	4.326805	16.1838941	39225.31	29788.844	121258.85	128890.52	19.33687139	4.314546632	3	High	3594	Unique	Used	86456	1	48.877	1	4.9827	0.8745	1	11	23/44	19.93388	3594	1787.99	MS2	596.669	216	Glioma_grade2_1rep.raw
4503979			eAASYQEALAR	Unambiguous	N-Term(iTRAQ4plex)	HCD	7.4211383	0	1	3	6.73957	6.918	7.9113	8.120754	9.358169094	38054.99	37073.484	256474.14	301064.66	1.847580581	11.31051653	2	High	1594	Unique	Used	65016	1	22.431	1	3.6122	1.1512	1	19	18/20	8.274342	1594	1352.69	MS2	676.851	205	Glioma_grade2_4.raw
4503979			iTIPVQTFSNLQIR	Unambiguous	N-Term(iTRAQ4plex)	HCD	3.2575169	0	1	3	2.99244	3.204	3.3075	3.5413313	6.994622223	37835.63	35337.055	113221.03	125140.23	4.829013247	7.071742402	2	High	4837	Unique	Used	87278	1	63.757	1	4.5407	1.8325	1	65	17/26	27.4061	4837	1774.04	MS2	887.522	115	Glioma_grade2_1rep.raw
4503979			vDFSLAGALNAGFk	Unambiguous	N-Term(iTRAQ4plex); K14(iTRAQ4plex)	HCD	3.464032	0	1	3	3.27185	3.4628	3.4652	3.6674306	4.659365934	36865.918	34833.215	120619.69	127748.41	4.00935971	4.059109244	2	High	7533	Unique	Used	54850	1	67.335	1	6.7152	3.6863	1	75	21/26	35.89856	7533	1697.95	MS2	849.48	222	Glioma_grade2_2.raw
4503979			hLQEYQDLLNVk	Unambiguous	N-Term(iTRAQ4plex); K12(iTRAQ4plex)	HCD	4.2667065	0	1	3	3.68272	4.4431	4.1205	4.9712687	12.61293565	31946.139	26478.953	117648.73	131634	13.23364384	7.93402676	2	High	3592	Unique	Used	86455	1	48.845	1	5.7251	1.0869	1	100	19/22	58.723	3592	1787.99	MS2	894.499	218	Glioma_grade2_1rep.raw
4503979			qLQSLTcDLESLR	Unambiguous	N-Term(iTRAQ4plex); C7(Methylthio)	HCD	6.9196854	0	1	3	6.68866	6.6388	7.2027	7.148986	4.294980233	27352.87	27558.504	182954.08	197015.36	0.529599554	5.23348743	2	High	5018	Unique	Used	87419	1	65.623	1	4.7221	1.6812	1	61	19/24	24.20417	5018	1695.86	MS2	848.431	182	Glioma_grade2_1rep.raw
4503979			tQYEAMASSNMHEAEEWYR	Unambiguous	N-Term(iTRAQ4plex)	HCD	3.6552055	0	1	3	3.0155	4.2283	3.2465	4.55219	19.80880994	26665.857	19017.484	80410.79	86571.2	23.67697412	5.217410196	3	High	4898	Unique	Used	52388	1	47.662	1	5.8912	1.4124	1	12	34/72	35.10242	4898	2477.07	MS2	826.36	32	Glioma_grade2_2.raw
4503979			lEEEGQSLkDEMAR	Unambiguous	N-Term(iTRAQ4plex); K9(iTRAQ4plex)	HCD	5.8367944	1	1	3	5.53025	5.3933	6.2916	6.135749	7.576797406	26556.584	27231.139	146864.45	167083.42	1.773573552	9.107862905	3	High	4012	Unique	Used	67253	1	41.009	1	3.6455	-1.9083	1	11	22/52	6.144714	4012	1922.97	MS2	641.661	136	Glioma_grade2_4.raw
4503979			kIESLEEEIR	Unambiguous	N-Term(iTRAQ4plex); K1(iTRAQ4plex)	HCD	4.559722	1	1	3	4.53737	4.3103	4.8223	4.5809584	4.598398232	23943.422	25204.76	108640.09	115461.95	3.629434973	4.304988447	3	High	2760	Unique	Used	73420	1	32.571	1	3.4744	10.357	1	7	23/36	15.93006	2760	1533.89	MS2	511.968	159	Glioma_grade2_5.raw
4503979			tQYEAmASSNmHEAEEWYR	Unambiguous	N-Term(iTRAQ4plex); M6(Oxidation); M11(Oxidation)	HCD	6.3497043	0	1	3	5.9169	6.2319	6.4616	6.805551	5.898756023	23574.604	22383.023	139488.52	152328.81	3.666747221	6.222698379	3	High	2886	Unique	Used	50495	1	33.002	1	6.6499	0.2906	1	7	29/72	10.34616	2886	2509.05	MS2	837.022	15	Glioma_grade2_2.raw
4503979			hLQEYQDLLNVk	Unambiguous	N-Term(iTRAQ4plex); K12(iTRAQ4plex)	HCD	7.27605	0	1	3	6.22156	7.3687	7.1978	8.524967	12.88676485	21682.648	18307.145	134899.94	156067.81	11.93725139	10.28838723	2	High	3684	Unique	Used	95905	1	50.731	1	6.0228	1.7013	1	100	21/22	5.098254	3684	1787.99	MS2	894.5	198	Glioma_grade2_8rep.raw
4503979			tQYEAMASSNmHEAEEWYR	Unambiguous	N-Term(iTRAQ4plex); M11(Oxidation)	HCD	4.4829817	0	1	3	4.2281	4.5987	4.3766	4.76021	5.242577443	21437.65	19709.887	90640.445	93823.2	5.938206866	2.440098853	3	High	4061	Unique	Used	51600	1	41.549	1	7.2242	0.4806	1	16	30/72	83.63573	4061	2493.06	MS2	831.691	14	Glioma_grade2_2.raw
4503979			hLQEYQDLLNVk	Unambiguous	N-Term(iTRAQ4plex); K12(iTRAQ4plex)	HCD	9.895156	0	1	3	8.19854	11.041	9.0443	12.180011	17.99494519	19972.545	14830.6045	163745.67	180636.92	20.89409176	6.93642348	2	High	3933	Unique	Used	74469	1	42.096	1	5.8623	0.4724	1	72	21/22	0	3933	1787.99	MS2	894.499	188	Glioma_grade2_5.raw
4503979			tQYEAmASSNmHEAEEWYR	Unambiguous	N-Term(iTRAQ4plex); M6(Oxidation); M11(Oxidation)	HCD	7.570424	0	1	3	6.33103	7.9379	7.2774	9.124383	15.30948284	19829.531	15815.482	125541.36	144306.53	15.92571319	9.834413719	3	High	2646	Unique	Used	50271	1	31.24	1	6.7226	0.6555	1	7	31/72	1.604863	2646	2509.05	MS2	837.023	15	Glioma_grade2_2.raw
4503979			tQYEAMASSNMHEAEEWYR	Unambiguous	N-Term(iTRAQ4plex)	HCD	3.8550515	0	1	3	3.24743	4.0861	3.6714	4.6195893	15.00333011	18588.877	14773.622	60366.09	68248.06	16.17260555	8.666844878	3	High	5088	Unique	Used	52566	1	49.072	1	8.0395	-2.0619	1	24	34/72	20.44705	5088	2477.06	MS2	826.357	20	Glioma_grade2_2.raw
4503979			tQYEAMASSNmHEAEEWYR	Unambiguous	N-Term(iTRAQ4plex); M11(Oxidation)	HCD	8.268175	0	1	3	8.19946	6.1953	11.012	8.320097	23.44550554	18131.354	23996.719	148667.28	199655.03	19.68967043	20.70139221	3	High	4161	Unique	Used	51694	1	42.281	1	5.6288	-0.6945	1	5	31/72	3.460679	4161	2493.06	MS2	831.69	13	Glioma_grade2_2.raw
4503979			hLQEYQDLLNVk	Unambiguous	N-Term(iTRAQ4plex); K12(iTRAQ4plex)	HCD	4.8865232	0	1	3	4.80125	4.745	5.0297	4.9707956	2.768354486	17967.748	18180.72	86267.695	90372.65	0.833196834	3.286498922	2	High	3927	Unique	Used	81156	1	42.546	1	5.6064	1.1551	1	100	19/22	29.40492	3927	1787.99	MS2	894.499	220	Glioma_grade2_6.raw
4503979			hLQEYQDLLNVk	Unambiguous	N-Term(iTRAQ4plex); K12(iTRAQ4plex)	HCD	7.878153	0	1	3	7.53116	6.6192	9.3105	8.183128	14.31185335	17590.668	20014.162	132478.16	163778.47	9.114090087	14.94154676	3	High	3939	Unique	Used	74474	1	42.158	1	4.8518	0.7721	1	5	24/44	9.13591	3939	1787.99	MS2	596.668	215	Glioma_grade2_5.raw
4503979			tQYEAMASSNMHEAEEWYR	Unambiguous	N-Term(iTRAQ4plex)	HCD	2.735391	0	1	3	2.43049	2.9695	2.5438	3.1079128	11.82540489	17099.705	13995.707	41560.67	43497.438	14.11692525	3.22015577	3	High	4036	Unique	Used	86719	1	54.603	1	8.5056	0.5992	1	66	31/72	57.64356	4036	2477.06	MS2	826.359	30	Glioma_grade2_1rep.raw
4503979			qQVHVELDVAkPDLTAALk	Unambiguous	N-Term(iTRAQ4plex); K11(iTRAQ4plex); K19(iTRAQ4plex)	HCD	9.24202	0	1	3	8.63271	9.3038	9.1847	9.8987	5.610696961	16781.285	15570.869	144867.88	154131.36	5.291105882	4.381462324	3	High	4288	Unique	Used	91681	1	48.259	1	6.6082	1.738	1	15	31/72	8.240958	4288	2507.46	MS2	836.492	47	Glioma_grade2_7rep.raw
4503979			hLQEYQDLLNVk	Unambiguous	N-Term(iTRAQ4plex); K12(iTRAQ4plex)	HCD	3.8479419	0	1	3	3.56852	3.5817	4.1132	4.1283875	8.190892323	16073.42	16014.481	57358.4	66113.984	0.259762498	10.02836848	3	High	3674	Unique	Used	95898	1	50.613	1	5.3365	-0.1496	1	31	23/44	27.08387	3674	1787.99	MS2	596.668	208	Glioma_grade2_8rep.raw
4503979			lEVERDNLAQDLATVR	Unambiguous	N-Term(iTRAQ4plex)	HCD	6.644529	1	1	3	6.2703	6.4138	6.8701	7.0273185	5.430511287	15303.543	14961.187	95957.85	105137.02	1.599764803	6.455314701	3	High	5276	Unique	Used	68421	1	50.901	1	4.1525	-1.086	1	14	26/60	8.755244	5276	1986.07	MS2	662.695	162	Glioma_grade2_4.raw
4503979			lEEEGQSLkDEmAR	Unambiguous	N-Term(iTRAQ4plex); K9(iTRAQ4plex); M12(Oxidation)	HCD	9.317812	1	1	3	8.93515	8.9524	9.6825	9.701213	4.636771442	12999	12973.979	116148.016	125863.33	0.136237886	5.677225076	2	High	1776	Unique	Used	65175	1	23.997	1	3.5338	0.5165	1	63	15/26	13.91633	1776	1938.97	MS2	969.988	138	Glioma_grade2_4.raw
4503979			tQYEAMASSNMHEAEEWYR	Unambiguous	N-Term(iTRAQ4plex)	HCD	6.294637	0	1	3	6.16305	6.5688	6.0374	6.4348817	3.867048956	12206.701	11452.769	75230.56	73697.21	4.506528927	1.456064484	3	High	5784	Unique	Used	53221	1	54.142	1	7.476	-2.2097	1	14	37/72	4.863477	5784	2477.06	MS2	826.357	21	Glioma_grade2_2.raw
4503979			hLQEYQDLLNVk	Unambiguous	N-Term(iTRAQ4plex); K12(iTRAQ4plex)	HCD	5.493904	0	1	3	5.35053	5.337	5.6512	5.636913	3.158656528	11885.999	11916.046	63596.445	67169.71	0.178526151	3.864424877	3	High	4036	Unique	Used	74563	1	42.917	1	4.6849	0.6697	1	8	26/44	8.824677	4036	1787.99	MS2	596.668	211	Glioma_grade2_5.raw
4503979			lEEEGQSLkDEmAR	Unambiguous	N-Term(iTRAQ4plex); K9(iTRAQ4plex); M12(Oxidation)	HCD	8.963389	1	1	3	8.0676	9.842	8.2432	10.056201	11.50837554	11275.6045	9242.716	90967.086	92946.6	14.01156828	1.522157272	2	High	1873	Unique	Used	65264	1	24.741	1	2.9399	0.5165	1	36	16/26	8.123863	1873	1938.97	MS2	969.988	137	Glioma_grade2_4.raw
4503979			tQYEAmASSNMHEAEEWYR	Unambiguous	N-Term(iTRAQ4plex); M6(Oxidation)	HCD	4.158696	0	1	3	3.67049	3.865	4.4376	4.672775	11.32768133	9609.14	9125.556	35270.24	42641.664	3.650398444	13.38019899	3	High	4057	Unique	Used	81262	1	43.794	1	8.2001	0.4806	1	43	31/72	33.09181	4057	2493.06	MS2	831.691	18	Glioma_grade2_6.raw
4503979			tQYEAMASSNMHEAEEWYR	Unambiguous	N-Term(iTRAQ4plex)	HCD	4.6273766	0	1	3	4.70001	5.1522	4.1486	4.547752	8.942757751	7253.641	6617.01	34092.203	30092.52	6.490915202	8.812698224	3	High	5480	Unique	Used	60945	1	53.047	1	7.1193	-2.1358	1	37	37/72	12.82452	5480	2477.06	MS2	826.357	20	Glioma_grade2_3.raw
4503979			tQYEAMASSNMHEAEEWYR	Unambiguous	N-Term(iTRAQ4plex)	HCD	3.5212955	0	1	3	3.83316	3.3114	3.7643	3.2518804	8.498043364	6304.1226	7297.469	24164.727	23730.498	10.32823211	1.282158171	3	High	4962	Unique	Used	60459	1	49.102	1	7.4723	3.4082	1	56	34/72	6.215134	4962	2477.07	MS2	826.362	30	Glioma_grade2_3.raw
4503979			vDFSLAGALNAGFk	Unambiguous	N-Term(iTRAQ4plex); K14(iTRAQ4plex)	HCD	3.2813663	0	1	3	2.97157	3.4474	3.1382	3.6407692	9.124135642	5515.956	4754.573	16391.064	17310.305	10.48396012	3.857419232	3	High	7390	Unique	Used	54720	1	66.172	1	5.2137	1.3778	1	36	24/52	45.37598	7390	1697.95	MS2	566.655	199	Glioma_grade2_2.raw
4503979			lEVERDNLAQDLATVR	Unambiguous	N-Term(iTRAQ4plex)	HCD	2.5959418	1	1	3	2.5422	2.5826	2.6091	2.65054	1.754158732	5016.6333	4938.215	12753.27	13088.938	1.114032279	1.836938779	2	High	5311	Unique	Used	68453	1	51.182	1	4.8499	-3.1076	1	100	22/30	21.18683	5311	1986.07	MS2	993.537	145	Glioma_grade2_4.raw
4503979			qQVHVELDVAkPDLTAALk	Unambiguous	N-Term(iTRAQ4plex); K11(iTRAQ4plex); K19(iTRAQ4plex)	HCD	3.8163562	0	1	3	4.17952	3.6042	4.0623	3.5031788	8.691571436	4897.083	5678.727	20467.46	19893.596	10.45226366	2.010765654	4	High	4106	Unique	Used	91540	1	46.444	1	5.3103	-3.16	1	22	30/108	20.76206	4106	2507.45	MS2	627.617	38	Glioma_grade2_7rep.raw
4503979			vDFSLAGALNAGFk	Unambiguous	N-Term(iTRAQ4plex); K14(iTRAQ4plex)	HCD	2.8588998	0	1	3	3.19288	2.8513	2.8674	2.56065	9.009070119	4798.6562	5373.517	15321.526	13759.698	7.992155893	7.595135403	3	High	6975	Unique	Used	62334	1	64.755	1	4.4688	-1.3182	1	25	22/52	38.78706	6975	1697.94	MS2	566.653	195	Glioma_grade2_3.raw
4503979			hLQEYQDLLNVk	Unambiguous	N-Term(iTRAQ4plex); K12(iTRAQ4plex)	HCD	5.533657	0	1	3	3.9855	6.1134	5.1557	7.908404	28.63864002	4574.8193	2982.4453	18232.93	23586.383	29.79857166	18.10389816	4	High	3849	Unique	Used	81095	1	41.755	1	3.7136	0.4574	1	41	24/66	0	3849	1787.99	MS2	447.753	212	Glioma_grade2_6.raw
4503979			vDFSLAGALNAGFk	Unambiguous	N-Term(iTRAQ4plex); K14(iTRAQ4plex)	HCD	3.7977087	0	1	3	2.95565	4.0498	3.6137	4.9514937	21.50150408	3648.7917	2662.9866	10784.534	13185.762	22.08789466	14.16690559	3	High	7240	Unique	Used	62574	1	66.933	1	4.3583	-0.2398	1	41	20/52	38.54767	7240	1697.95	MS2	566.654	199	Glioma_grade2_3.raw

4503979			vDFSLAGALNAGFk	Unambiguous	N-Term(iTRAQ4plex); K14(iTRAQ4plex)	HCD	3.1241024	0	1	3	3.43379	4.0288	2.353	2.760749	23.51197415	2479.6648	2113.4397	8514.651	5834.677	11.27604441	26.41277402	3	High	7513	Unique	Used	54832	1	67.172	1	3.479	-0.8869	1	70	18/52	13.9478	7513	1697.95	MS2	566.653	219	Glioma_grade 2_2.raw
4503979			hLQEYQDLLNVk	Unambiguous	N-Term(iTRAQ4plex); K12(iTRAQ4plex)	HCD	6.7785254	0	1	3	6.22748	6.5347	7.0109	7.356754	7.378933628	957.70264	912.6828	5964.0723	6714.383	3.403986523	8.369312687	4	High	3960	Unique	Used	74493	1	42.321	1	4.3448	-1.4543	1	45	24/66	4.800938	3960	1787.99	MS2	447.752	222	Glioma_grade 2_5.raw
4503987	NP_003869.1	GGH	gamma-glutamyl hydrolase precursor [Homo sapiens]	3	6	2.098605633	18.30017517	27.657675	10.38	1.67E-04	318	35.941	7.1079	Enzyme: Hydrolase	Metabolism,Energy pathways	Hydrolase activity	Lysosome	Extracellular, Plasma membrane																								
4503987			iEFISTMEGYk	Unambiguous	N-Term(iTRAQ4plex); K11(iTRAQ4plex)	HCD	1.5181483	0	1	1	1.39078	1.5674	1.4744	1.661691	7.677388336	83243.61	73862.87	115773.664	122737.27	8.444190038	4.128959789	2	High	6245	Unique	Used	53658	1	57.475	1	3.4708	1.4308	1	46	15/20	19.07347	6245	1605.85	MS2	803.427	260	Glioma_grade 2_2.raw
4503987			yPVYGVQWHPEk	Unambiguous	N-Term(iTRAQ4plex); K12(iTRAQ4plex)	HCD	1.9794563	0	1	1	1.79006	1.8803	2.0738	2.1783984	8.945493153	48206.434	45892.52	86292.57	99972.19	3.477582292	10.38623953	3	High	3264	Unique	Used	80578	1	36.88	1	5.3041	-2.7408	1	32	24/44	21.82506	3264	1790.94	MS2	597.652	186	Glioma_grade 2_6.raw
4503987			yPVYGVQWHPEk	Unambiguous	N-Term(iTRAQ4plex); K12(iTRAQ4plex)	HCD	2.1797113	0	1	1	2.229	2.061	2.3081	2.1341422	4.95047186	39462	42678.688	87960.61	91082.39	5.538161301	2.465811908	3	High	3320	Unique	Used	73935	1	36.877	1	4.4049	0.2242	1	22	21/44	30.40019	3320	1790.95	MS2	597.654	191	Glioma_grade 2_5.raw
4503987			kPIIGILMQk	Unambiguous	N-Term(iTRAQ4plex); K1(iTRAQ4plex); K10(iTRAQ4plex)	HCD	2.7051222	0	1	1	2.71031	2.7706	2.6411	2.6998239	1.960489615	21774.53	21300.814	59015.63	57508.445	1.555264636	1.829219814	3	High	3771	Unique	Used	86551	1	51.334	1	5.9853	2.3042	1	49	23/36	30.79349	3771	1573.03	MS2	525.014	21	Glioma_grade 2_1rep.raw
4503987			kPIIGILMQk	Unambiguous	N-Term(iTRAQ4plex); K1(iTRAQ4plex); K10(iTRAQ4plex)	HCD	2.0260823	0	1	1	1.97357	2.0713	1.983	2.08119	2.802701788	20938.818	19951.15	41324.305	41522.137	3.415931949	0.337705145	2	High	3895	Unique	Used	96018	1	53.694	1	3.3019	1.9635	1	100	15/18	42.9	3895	1573.03	MS2	787.017	21	Glioma_grade 2_8rep.raw
4503987			kPIIGILMQk	Unambiguous	N-Term(iTRAQ4plex); K1(iTRAQ4plex); K10(iTRAQ4plex)	HCD	2.1711287	0	1	1	1.87836	2.1055	2.2297	2.4992912	11.8817146	8364.195	7462.0044	15710.994	18649.723	8.061886181	12.09517953	3	High	3881	Unique	Used	96012	1	53.476	1	5.1907	-2.4684	1	100	22/36	28.55231	3881	1573.02	MS2	525.011	21	Glioma_grade 2_8rep.raw
4504051	NP_002064.1	GNAZ	guanine nucleotide-binding protein G(z) subunit alpha [Homo sapiens]	4	6	0.473659992	17.52483206	25.641288	12.68	2.61E-04	355	40.898	7.606	G protein	Cell communication,Signal transduction	GTPase activity	Plasma membrane	Cytoplasm, Nucleus, Endoplasmic reticulum, Golgi apparatus																								
4504051			aYDAVQLFALTGPAESk	Unambiguous	N-Term(iTRAQ4plex); K17(iTRAQ4plex)	HCD	0.43923348	0	1	1	0.42811	0.438	0.4404	0.45061466	0.021040767	82662.28	80797.6	35388.47	36408.582	1.613274001	2.00935301	2	High	6824	Unique	Used	54197	1	61.817	1	4.964	0.1049	1	24	22/32	10.90386	6824	2069.12	MS2	1035.06	187	Glioma_grade 2_2.raw
4504051			iPLTIcFPEYk	Unambiguous	N-Term(iTRAQ4plex); C6(Methylthio); K11(iTRAQ4plex)	HCD	0.49675378	0	1	1	0.46801	0.5057	0.4885	0.52780867	0.051078882	60134.95	55651.78	28144.006	29373.49	5.475722318	3.022998337	2	High	7694	Unique	Used	62978	1	70.809	1	3.6266	2.3465	1	100	14/20	18.29545	7694	1657.9	MS2	829.452	208	Glioma_grade 2_3.raw
4504051			rIPLTIcFPEYk	Unambiguous	N-Term(iTRAQ4plex); C7(Methylthio); K12(iTRAQ4plex)	HCD	0.65151286	1	1	1	0.56645	0.6348	0.6665	0.74683654	0.114515705	47942.832	42782.477	27157.188	31951.518	8.043889966	11.47074089	2	High	5373	Unique	Used	92499	1	59.415	1	3.6952	0.7062	1	100	15/22	52.9684	5373	1814	MS2	907.502	233	Glioma_grade 2_7rep.raw
4504051			iPLTIcFPEYk	Unambiguous	N-Term(iTRAQ4plex); C6(Methylthio); K11(iTRAQ4plex)	HCD	0.4384706	0	1	1	0.43306	0.4324	0.4445	0.44387627	0.015080804	45098.66	45162.22	19530.303	20046.438	0.099586237	1.844328509	2	High	7751	Unique	Used	70652	1	71.425	1	3.6964	0.8739	1	100	15/20	36.23534	7751	1657.9	MS2	829.451	232	Glioma_grade 2_4.raw
4504051			eYkPLIIYNAIDSLTR	Unambiguous	N-Term(iTRAQ4plex); K3(iTRAQ4plex)	HCD	0.45056617	0	1	1	0.39099	0.3769	0.5269	0.50799984	0.17235489	11953.328	12399.177	4673.6353	6298.78	2.589161577	20.94617833	3	High	6977	Unique	Used	77161	1	67.54	1	5.0786	-2.8438	1	53	20/60	34.50043	6977	2197.24	MS2	733.085	98	Glioma_grade 2_5.raw
4504051			rIPLTIcFPEYk	Unambiguous	N-Term(iTRAQ4plex); C7(Methylthio); K12(iTRAQ4plex)	HCD	0.5889355	1	1	1	0.52548	0.5881	0.5897	0.6599468	0.092998203	9335.825	8342.206	4905.8076	5505.412	7.948789464	8.14475832	3	High	5334	Unique	Used	92470	1	59.01	1	4.5804	1.2035	1	43	16/44	38.9544	5334	1814	MS2	605.337	155	Glioma_grade 2_7rep.raw
4504327	NP_000174.1	HADHB	trifunctional enzyme subunit beta, mitochondrial precursor [Homo sapiens]	8	14	2.25451088	18.48025396	82.646789	27.85	2.08E-04	474	51.262	9.4077	Enzyme: Dehydrogenase	Metabolism,Energy pathways	Catalytic activity	Mitochondrion	-																								
4504327			aALTGLLHR	Unambiguous	N-Term(iTRAQ4plex)	HCD	1.6791816	0	1	1	1.55659	1.8205	1.5583	1.8225672	9.025345204	141891.61	121319.12	220866.38	221112.23	11.05346061	0.078665437	2	High	1864	Unique	Used	72592	1	25.827	1	3.4247	0.8259	1	25	15/16	39.49096	1864	1095.68	MS2	548.342	207	Glioma_grade 2_5.raw
4504327			dLMPHDLAR	Unambiguous	N-Term(iTRAQ4plex)	HCD	2.603167	0	1	1	2.40542	2.908	2.3511	2.8422272	11.0012115	106394.23	88007.875	255922.77	250138.38	13.37548922	1.616477137	3	High	1833	Unique	Used	65227	1	24.44	1	3.5396	0.8515	1	5	17/32	6.350413	1833	1211.63	MS2	404.549	230	Glioma_grade 2_4.raw
4504327			dLMPHDLAR	Unambiguous	N-Term(iTRAQ4plex)	HCD	2.1386452	0	1	1	1.96844	2.2682	2.0262	2.3347752	8.340697783	100457.57	87179.555	197744.58	203544.66	10.00758719	2.044049773	2	High	1774	Unique	Used	65173	1	23.987	1	2.9841	0.107	1	30	16-Dec	8.953625	1774	1211.63	MS2	606.32	261	Glioma_grade 2_4.raw
4504327			kLMLDLNk	Unambiguous	N-Term(iTRAQ4plex); K1(iTRAQ4plex); K8(iTRAQ4plex)	HCD	1.8991653	1	1	1	1.71914	1.946	1.8578	2.1029515	8.437934033	89682.484	79226.46	154176.61	166609.42	8.754451126	5.481114162	2	High	2440	Unique	Used	90202	1	30.498	1	3.1583	8.122	1	54	14/14	48.93055	2440	1406.89	MS2	703.948	91	Glioma_grade 2_7rep.raw
4504327			dFmYVSQDPk	Unambiguous	N-Term(iTRAQ4plex); M3(Oxidation); K10(iTRAQ4plex)	HCD	1.6314874	0	1	1	1.59407	1.5964	1.6665	1.6689595	2.566142283	86748.13	86619.85	138282.66	144565.02	0.104641766	3.141124833	2	High	1921	Unique	Used	49584	1	26.22	1	3.2981	1.2011	1	44	13/18	35.37853	1921	1533.75	MS2	767.379	130	Glioma_grade 2_2.raw
4504327			dQLLLGPTYATPk	Unambiguous	N-Term(iTRAQ4plex); K13(iTRAQ4plex)	HCD	1.6006827	0	1	1	1.50862	1.6413	1.5634	1.7008381	5.280659844	48292.617	44390.08	72855.25	75500.336	5.954747684	2.521453082	2	High	4228	Unique	Used	51758	1	42.76	1	4.5764	3.6381	1	100	15/24	23.33496	4228	1704.98	MS2	852.996	183	Glioma_grade 2_2.raw
4504327			kAQDEGLLSDVVPFk	Unambiguous	N-Term(iTRAQ4plex); K1(iTRAQ4plex); K15(iTRAQ4plex)	HCD	2.683904	1	1	1	2.6483	2.8228	2.5536	2.721857	4.241301774	41795.035	39211.26	110685.664	106727.45	4.510772462	2.574714569	2	High	4708	Unique	Used	91999	1	52.561	1	5.0775	-3.8776	1	58	18/28	23.25584	4708	2078.18	MS2	1039.59	144	Glioma_grade 2_7rep.raw
4504327			fNNWGGSLSLGHPFGATGcR	Unambiguous	N-Term(iTRAQ4plex); C19(Methylthio)	HCD	2.0255237	0	1	1	1.7812	2.2422	1.8534	2.3330743	13.42995344	18502.701	14698.654	32957.082	34293.055	16.20335935	2.809438344	3	High	5908	Unique	Used	76237	1	58.203	1	6.3344	-0.7186	1	31	32/76	32.79622	5908	2268.05	MS2	756.69	63	Glioma_grade 2_5.raw
4504327			aALTGLLHR	Unambiguous	N-Term(iTRAQ4plex)	HCD	2.3703768	0	1	1	2.21038	2.2556	2.4828	2.533651	6.804409563	15301.226	14994.14	33821.51	37989.918	1.433503678	8.209026462	3	High	1853	Unique	Used	72583	1	25.723	1	4.6227	1.7606	1	33	18/32	37.83576	1853	1095.68	MS2	365.897	75	Glioma_grade 2_5.raw
4504327			dLMPHDLAR	Unambiguous	N-Term(iTRAQ4plex)	HCD	2.4587438	0	1	1	2.23534	2.4678	2.4505	2.7053833	7.795193392	14898.339	13494.829	33302.84	36508.684	6.990635483	6.49426886	3	High	1668	Unique	Used	57395	1	24.03	1	3.8212	0.4737	1	79	17/32	29.33723	1668	1211.63	MS2	404.549	233	Glioma_grade 2_3.raw
4504327			aALTGLLHR	Unambiguous	N-Term(iTRAQ4plex)	HCD	2.7558498	0	1	1	2.54062	2.6821	2.8257	2.9830701	6.88751842	6018.3584	5700.7847	15290.358	17005.842	3.832336799	7.511907713	3	High	1927	Unique	Used	72643	1	26.433	1	4.5684	0.5908	1	61	18/32	52.79989	1927	1095.68	MS2	365.897	62	Glioma_grade 2_5.raw
4504327			fNFLAPELPAVSEFSTSETMGHSADR	Unambiguous	N-Term(iTRAQ4plex)	HCD	2.5067792	0	1	1	2.24781	2.587	2.4371	2.804825	9.353610295	4973.55	4321.484	11179.592	12121.006	9.921002772	5.713846686	3	High	7458	Unique	Used	62763	1	68.858	1	7.705	3.9071	1	46	32/100	24.98478	7458	2984.43	MS2	995.483	108	Glioma_grade 2_3.raw
4504327			aALTGLLHR	Unambiguous	N-Term(iTRAQ4plex)	HCD	2.442888	0	1	1	2.22465	2.5803	2.3244	2.6960127	8.916005445	3948.7085	3404.4473	8784.503	9178.433	10.46763582	3.101392493	3	High	2332	Unique	Used	65693	1	28.102	1	3.8244	13.208	1	100	19/32	42.7853	2332	1095.69	MS2	365.902	72	Glioma_grade 2_4.raw
4504327			fNFLAPELPAVSEFSTSETmGHSADR	Unambiguous	N-Term(iTRAQ4plex); M20(Oxidation)	HCD	1.883677	0	1	1	1.93966	1.8688	1.8991	1.8297411	2.470387343	1826.5127	1895.7637	3542.8098	3468.7566	2.631070154	1.493632575	3	High	7163	Unique	Used	62508	1	66.23	1	6.4693	-0.8471	1	100	37/100	7.703476	7163	3000.41	MS2	1000.81	88	Glioma_grade 2_3.raw
4504517	NP_001531.1	HSPB1	heat shock protein beta-1 [Homo sapiens]	2	3	2.400006056	15.40979815	32.848706	32.2	4.06E-04	205	22.768	6.4048	Chaperone	Protein metabolism	Chaperone activity	Cytoplasm	Microtubule, Nucleus, Plasma membrane																								
4504517			vSLDVNHFAPDELTVk	Unambiguous	N-Term(iTRAQ4plex); K16(iTRAQ4plex)	HCD	2.7582335	0	1	1	2.50776	2.7089	2.8039	3.0287914	7.831203885	60942.64	56417.703	152829.78	170877.45	5.452631707	7.884673964	3	High	5102	Unique	Used	75514	1	51.659	1	4.3854	0.1105	1	4	27/60	8.611927	5102	2072.13	MS2	691.381	152	Glioma_grade 2_5.raw
4504517			lFDQAFGLPR	Unambiguous	N-Term(iTRAQ4plex)	HCD	2.0208144	0	1	1	1.78284	2.1799	1.8907	2.3117952	12.06217948	58993.34	48246.84	105175.68	111536.82	14.17178342	4.151126705	2	High	4516	Unique	Used	87042	1	60.243	1	3.7651	-0.3323	1	75	16/18	18.17256	4516	1307.72	MS2	654.365	331	Glioma_grade 2_1rep.raw
4504517			vSLDVNHFAPDELTVk	Unambiguous	N-Term(iTRAQ4plex); K16(iTRAQ4plex)	HCD	2.400006	0	1	1	2.15096	2.5004	2.3136	2.689511	9.646618768	40490.086	34831.36	87092.6	93679.32	10.62465935	5.152917973	2	High	5120	Unique	Used	75531	1	51.785	1	6.1277	-1.1496	1	44	20/30	29.17478	5120	2072.12	MS2	1036.57	183	Glioma_grade 2_5.raw
4505257	NP_002435.1	MSN	moesin [Homo sapiens]	5	10	2.918308973	24.47093161	144.10842	24.78	3.28E-04	577	67.778	6.4048	Cytoskeletal protein	Cell growth and/or maintenance	Structural constituent of cytoskeleton	Cytoplasm	Mitochondrion, Cytoskeleton, Nucleus, Plasma membrane																								
4505257			aQmVQEDLEk	Unambiguous	N-Term(iTRAQ4plex); M3(Oxidation); K10(iTRAQ4plex)	HCD	2.9610345	0	1	1	2.51964	3.327	2.6839	3.5438635	16.36402009	414901.78	314219.03	1045402.44	1113549.4	19.52857587	4.463941867	2	High	1410	Unique	Used	49099	1	22.738	1	3.2693	-0.4796	1	4	14/18	29.01153	1410	1494.77	MS2	747.889	149	Glioma_grade 2_2.raw
4505257			aQMVQEDLEk	Unambiguous	N-Term(iTRAQ4plex); K10(iTRAQ4plex)	HCD	3.700258	0	1	1	3.6921	3.6068	3.796	3.708226	2.094674501	272431.16	278876.56	1005843.5	1034137.3	1.653372838	1.961463348	2	High	1890	Unique	Used	49554	1	26.018	1	4.5629	-0.7866	1	8	18/18	4.319439	1890	1478.78	MS2	739.891	184	Glioma_grade 2_2.raw
4505257			kESPLLFk	Unambiguous	N-Term(iTRAQ4plex); K1(iTRAQ4plex); K8(iTRAQ4plex)	HCD	1.8261737	1	1	1	1.76065	1.8912	1.7656	1.896553	4.131854004	269106.16	250526.56	473802.66	475136.9	5.056556543	0.198843044	2	High	1898	Unique	Used	89740	1	25.754	1	3.4221	-2.506	1	21	13/14	31.5539	1898	1393.87	MS2	697.441	86	Glioma_grade 2_7rep.raw
4505257			gFSTWLk	Unambiguous	N-Term(iTRAQ4plex); K7(iTRAQ4plex)	HCD	3.6095107	0	1	1	3.4632	3.4608	3.7583	3.755725	4.718894622	177906.92	178029.16	616126.56	668628.56	0.048568683	5.779236782	2	High	4584	Unique	Used	67786	1	45.42	1	3.22	0.0201	1	18	12-Dec	13.75089	4584	1126.65	MS2	563.829	193	Glioma_grade 2_4.raw
4505257			kESPLLFk	Unambiguous	N-Term(iTRAQ4plex); K1(iTRAQ4plex); K8(iTRAQ4plex)	HCD	2.8755834	1	1	1	2.54955	3.129	2.6691	3.2757165	12.07853178	161628.03	131698.3	412078.03	431406.28	14.43001386	3.240638025	3	High	1896	Unique	Used	89738	1	25.743	1	4.2026	-1.9902	1	4	20/28	18.21926	1896	1393.88	MS2	465.297	83	Glioma_grade 2_7rep.raw
4505257			gFSTWLk	Unambiguous	N-Term(iTRAQ4plex); K7(iTRAQ4plex)	HCD	2.5549662	0	1	1	2.42217	2.5939	2.5186	2.697179	4.551286704	42825.824	39990.535	103731.36	107861.64	4.841681288	2.760534608	2	High	4243	Unique	Used	59796	1	43.405	1	2.9757	0.5618	1	82	12-Dec	44.93705	4243	1126.65	MS2	563.829	184	Glioma_grade 2_3.raw
4505257			kTQEQLALEMAELTAR	Unambiguous	N-Term(iTRAQ4plex); K1(iTRAQ4plex)	HCD	3.4505558	1	1	1	3.49621	3.3432	3.5628	3.406898	2.803289658	18940.64	19807.379	66220.484	67481.72	3.163397976	1.334052097	2	High	4838	Unique	Used	81930	1	50.697	1	6.0707	-2.0945	1	75	24/30	20.38604	4838	2120.16	MS2	1060.58	162	Glioma_grade 2_6.raw
4505257			kTQEQLALEMAELTAR	Unambiguous	N-Term(iTRAQ4plex); K1(iTRAQ4plex)	HCD	2.2423925	1	1	1	2.62481	1.9959	2.5665	1.9516006	15.77037324	16116.771	21195.098	42303.492	41364.363	19.248135	1.587382596	3	High	5011	Unique	Used	75431	1	50.952	1	5.6223	-1.3812	1	6	28/60	11.17258	5011	2120.16	MS2	707.391	149	Glioma_grade 2_5.raw
4505257			kTANDMIHAENMR	Unambiguous	N-Term(iTRAQ4plex); K1(iTRAQ4plex)	HCD	1.8277422	1	1	1	1.62333	2.1686	1.5726	2.1008189	16.70692935	13353.533	9995.99	21677.146	20999.764	20.33567387	2.244686439	4	High	1166	Unique	Used	89085	1	19.986	1	3.4721	-1.026	1	15	27/72	44.43849	1166	1818.92	MS2	455.485	145	Glioma_grade 2_7rep.raw
4505257			kTQEQLALEMAELTAR	Unambiguous	N-Term(iTRAQ4plex); K1(iTRAQ4plex)	HCD	3.0888627	1	1	1	2.95973	2.8759	3.308	3.214334	6.627922665	9623.33	9903.809	28482.5	31834.15	2.031312451	7.858441884	2	High	5067	Unique	Used	75482	1	51.387	1	5.717	-0.5975	1	76	24/30	15.6416	5067	2120.16	MS2	1060.58	121	Glioma_grade 2_5.raw
4505343	NP_002477.1	NCBP1	nuclear cap-binding protein subunit 1 [Homo sapiens]	2	2	2.834197044	37.65273207	14.270037	4.43	1.48E-04	790	91.781	6.4302	RNA binding protein	Regulation of nucleobase, nucleoside, nucleotide and nucleic acid metabolism	RNA binding	Nucleus	Cytoplasm																								
4505343			lFVWEILHSTIR	Unambiguous	N-Term(iTRAQ4plex)	HCD	3.588788	0	1	1	3.70059	3.1923	4.0484	3.4923444	9.974556438	3978.6782	4612.1157	14723.441	16107.098	10.42762652	6.34690978	3	High	7059	Unique	Used	83737	1	71.834	1	4.7201	-2.2452	1	67	28/44	4.138608	7059	1657.95	MS2	553.322	134	Glioma_grade 2_6.raw
4505343			fIMILTEHLVR	Unambiguous	N-Term(iTRAQ4plex)	HCD	2.0796063	0	1	1	1.66678	2.2143	1.9782	2.6280499	19.09583093	3748.3887	2821.4873	6247.7466	7415.0093	19.95222636	12.08217986	3	High	6706	Unique	Used	76935	1	65.019	1	5.0394	-4.3558	1	100	22/40	11.9843	6706	1515.88	MS2	505.964	164	Glioma_grade 2_5.raw
4506025	NP_000936.1	PPP3R1	calcineurin subunit B type 1 [Homo sapiens]	2	4	0.429428309	31.3001687	29.564661	34.12	3.16E-04	170	19.288	4.8052	Regulatory/other subunit	Cell communication,Signal transduction	Phosphatase regulator activity	Cytoplasm	Nucleus, Plasma membrane																								
4506025			eFIEGVSQFSVk	Unambiguous	N-Term(iTRAQ4plex); K12(iTRAQ4plex)	HCD	0.59283346	0	1	1	0.56641	0.6162	0.5713	0.6215845	0.048896281	492479.8	452667.5	278943.8	281371.1	5.957070883	0.612641317	2	High	5565	Unique	Used	53016	1	52.521	1	4.7761	-1.9258	1	9	19/22	23.02208	5565	1657.9	MS2	829.454	242	Glioma_grade 2_2.raw
4506025			iSFEEFcAVVGGLDIHk	Unambiguous	N-Term(iTRAQ4plex); C7(Methylthio); K17(iTRAQ4plex)	HCD	0.27884382	0	1	1	0.22496	0.2413	0.3138	0.33664918	0.194830365	85286.625	79503.77	19185.9	26764.88	4.962784372	23.32560254	3	High	7489	Unique	Used	84064	1	76.335	1	7.0246	2.3368	1	7	29/64	8.219891	7489	2198.13	MS2	733.381	149	Glioma_grade 2_6.raw
4506025			iSFEEFcAVVGGLDIHk	Unambiguous	N-Term(iTRAQ4plex); C7(Methylthio); K17(iTRAQ4plex)	HCD	0.48270038	0	1	1	0.48857	0.4821	0.4833	0.4769082	0.009911496	5685.2974	5761.3154	2777.6655	2747.6187	0.93919213	0.76905713	3	High	7618	Unique	Used	84176	1	77.431	1	6.2704	1.4205	1	100	29/64	26.24768	7618	2198.13	MS2	733.38	143	Glioma_grade 2_6.raw
4506025			iSFEEFcAVVGGLDIHk	Unambiguous	N-Term(iTRAQ4plex); C7(Methylthio); K17(iTRAQ4plex)	HCD	0.37615624	0	1	1	0.31913	0.4472	0.3255	0.45607275	0.19332826	2751.8257	1963.7124	878.1833	895.59576	23.63591374	1.388275329	3	High	7690	Unique	Used	84214	1	78.453	1	5.221	1.3372	1	100	28/64	39.28558	7690	2198.13	MS2	733.38	147	Glioma_grade 2_6.raw

4506341	NP_002849.1	ABCD3	ATP-binding cassette sub-family D member 3 isoform a [Homo sapiens]	7	8	2.093060255	20.41999851	69.494407	10.93	1.63E-04	659	75.428	9.3638	Integral membrane protein	Transport	ATP binding	Peroxisome	Mitochondrion																								
4506341			iTELMQVLk	Unambiguous	N-Term(iTRAQ4plex); K9(iTRAQ4plex)	HCD	2.212802	0	1	1	2.04846	2.2362	2.1914	2.392209	6.385670133	157813.03	144564.56	323273.06	345828.62	6.196281264	4.767344009	2	High	6483	Unique	Used	61876	1	60.916	1	4.2427	0.4659	1	30	15/16	26.35702	6483	1362.83	MS2	681.917	191	Glioma_grade 2_3.raw
4506341			vLGELWPLFGGR	Unambiguous	N-Term(iTRAQ4plex)	HCD	2.2902374	0	1	1	2.14329	2.3053	2.2762	2.4482987	5.45599224	118893.61	110536.13	254823.1	270625.47	5.151582163	4.253113863	2	High	8614	Unique	Used	55831	1	76.164	1	3.9228	-0.8242	1	24	18/22	4.344161	8614	1487.85	MS2	744.428	243	Glioma_grade 2_2.raw
4506341			iTELMQVLk	Unambiguous	N-Term(iTRAQ4plex); K9(iTRAQ4plex)	HCD	1.9733187	0	1	1	1.85481	2.0211	1.9295	2.102455	5.453327729	90655.086	83197.375	168147.58	174918.72	6.066520991	2.791249977	2	High	6641	Unique	Used	54029	1	60.388	1	3.9129	0.3763	1	49	15/16	34.02827	6641	1362.83	MS2	681.917	192	Glioma_grade 2_2.raw
4506341			kGISDLVLk	Unambiguous	N-Term(iTRAQ4plex); K1(iTRAQ4plex); K9(iTRAQ4plex)	HCD	1.9209688	1	1	1	1.77979	2.0538	1.8058	2.0838904	8.296749787	79847.94	69194.18	142112.17	144193.08	10.10901608	1.027871876	2	High	2399	Unique	Used	90166	1	30.156	1	3.2598	-4.1448	1	53	15/16	16.56186	2399	1404.91	MS2	702.958	50	Glioma_grade 2_7rep.raw
4506341			sTHSELLEDYYQSGR	Unambiguous	N-Term(iTRAQ4plex)	HCD	2.327282	0	1	1	2.34267	2.31	2.3448	2.3121114	0.81309368	23792.432	24129.15	55737.754	55789.28	0.993688318	0.065337314	2	High	2922	Unique	Used	66243	1	32.525	1	5.3879	0.89	1	66	24/28	0	2922	1928.91	MS2	964.96	111	Glioma_grade 2_4.raw
4506341			eYLDNVQLGHILER	Unambiguous	N-Term(iTRAQ4plex)	HCD	3.0498013	0	1	1	2.69847	3.3784	2.7874	3.4896567	13.05551564	18990.441	15168.657	51245.06	52933.406	15.82248678	2.291914925	2	High	5567	Unique	Used	61021	1	53.8	1	5.5411	1.8522	1	57	23/26	15.36654	5567	1842.99	MS2	921.997	198	Glioma_grade 2_3.raw
4506341			lVEHLHNFILFR	Unambiguous	N-Term(iTRAQ4plex)	HCD	1.6986209	0	1	1	1.71718	1.4729	1.9618	1.6827008	11.72704625	16111.384	18784.035	27666.178	31607.908	10.83150568	9.404528018	3	High	4893	Unique	Used	92132	1	54.552	1	4.2783	-1.4871	1	41	23/44	76.97	4893	1681.96	MS2	561.326	222	Glioma_grade 2_7rep.raw
4506341			kLVEHLHNFILFR	Unambiguous	N-Term(iTRAQ4plex); K1(iTRAQ4plex)	HCD	1.7091198	1	1	1	1.83705	1.8045	1.612	1.5834603	7.604132241	1976.7161	2012.3723	3631.3184	3186.512	1.264085339	9.226560454	4	High	4028	Unique	Used	96093	1	55.489	1	4.24	0.5813	1	100	26/72	19.71243	4028	1954.16	MS2	489.296	65	Glioma_grade 2_8rep.raw
4506367	NP_002857.1	RAB3A	ras-related protein Rab-3A [Homo sapiens]	3	6	0.258286923	17.10124162	80.29177	22.73	2.65E-05	220	24.968	5.0337	GTPase	Cell communication,Signal transduction	GTPase activity	Golgi apparatus	Cytoplasm, Plasma membrane, Endoplasmic reticulum																								
4506367			qLADHLGFEFFEASAk	Unambiguous	N-Term(iTRAQ4plex); K16(iTRAQ4plex)	HCD	0.25486907	0	1	1	0.28117	0.2671	0.242	0.22988144	0.091456843	1290788.1	1358723.6	362933.25	312345.34	3.626151395	10.59445827	3	High	5598	Unique	Used	82578	1	57.4	1	6.9924	1.937	1	1	24/60	5.881489	5598	2098.09	MS2	700.035	176	Glioma_grade 2_6.raw
4506367			iLIIGNSSVGk	Unambiguous	N-Term(iTRAQ4plex); K11(iTRAQ4plex)	HCD	0.33023134	0	1	1	0.31362	0.3336	0.327	0.3479075	0.043139221	461328.34	433640.3	144679.97	150866.73	4.375214944	2.960411975	2	High	3734	Unique	Used	59321	1	39.496	1	4.3267	-0.2188	1	35	16/20	33.80009	3734	1388.87	MS2	694.939	135	Glioma_grade 2_3.raw
4506367			qLADHLGFEFFEASAk	Unambiguous	N-Term(iTRAQ4plex); K16(iTRAQ4plex)	HCD	0.26170477	0	1	1	0.24879	0.2487	0.2747	0.27461785	0.057158269	402011.88	402133.03	100015.65	110432.91	0.021306107	7.000394954	2	High	5591	Unique	Used	82572	1	57.336	1	7.783	-1.6638	1	24	21/30	0	5591	2098.08	MS2	1049.54	185	Glioma_grade 2_6.raw
4506367			tYSWDNAQVLLVGNk	Unambiguous	N-Term(iTRAQ4plex); K15(iTRAQ4plex)	HCD	0.21396126	0	1	1	0.19354	0.1959	0.2318	0.23463102	0.104066609	210949.11	208391.17	40827.54	48895.03	0.862658231	12.71603541	2	High	6151	Unique	Used	53569	1	56.787	1	6.4672	-1.0301	1	24	21/28	6.666463	6151	1996.07	MS2	998.54	200	Glioma_grade 2_2.raw
4506367			tYSWDNAQVLLVGNk	Unambiguous	N-Term(iTRAQ4plex); K15(iTRAQ4plex)	HCD	0.27320218	0	1	1	0.24467	0.2642	0.2815	0.30401558	0.092267585	94287.13	87303.62	23069.33	26541.658	5.438699138	9.898237363	2	High	5938	Unique	Used	53367	1	55.261	1	6.2048	-1.6416	1	54	21/28	32.47205	5938	1996.07	MS2	998.539	200	Glioma_grade 2_2.raw
4506367			qLADHLGFEFFEASAk	Unambiguous	N-Term(iTRAQ4plex); K16(iTRAQ4plex)	HCD	0.21027982	0	1	1	0.21944	0.2408	0.1825	0.20022766	0.119068571	10498.114	9567.057	2303.7112	1915.5894	6.562183979	13.00895967	4	High	5630	Unique	Used	82607	1	57.649	1	5.1348	-0.9203	1	50	19/90	21.65565	5630	2098.08	MS2	525.276	198	Glioma_grade 2_6.raw
4506455	NP_002892.1	RCN1	reticulocalbin-1 precursor [Homo sapiens]	3	5	3.079221725	20.78754161	50.174009	21.45	7.87E-05	331	38.866	4.9956	Calcium binding protein	Cell communication,Signal transduction	Calcium ion binding	Endoplasmic reticulum	-																								
4506455			hWILPQDYDHAQAEAR	Unambiguous	N-Term(iTRAQ4plex)	HCD	3.0792217	0	1	1	3.06202	2.8593	3.3147	3.0952814	6.048413762	47272.234	50623.3	144748.7	156693.36	4.841000189	5.60382986	3	High	2352	Unique	Used	79774	1	29.27	1	6.2964	3.7717	1	31	24/60	5.822925	2352	2094.04	MS2	698.683	124	Glioma_grade 2_6.raw
4506455			hWILPQDYDHAQAEAR	Unambiguous	N-Term(iTRAQ4plex)	HCD	3.648336	0	1	1	3.52731	3.6822	3.6159	3.7746754	2.864632439	41129.867	39399.625	145077.84	148720.8	3.038553506	1.753555918	4	High	2384	Unique	Used	79804	1	29.506	1	4.2533	3.8819	1	4	26/90	18.7611	2384	2094.04	MS2	524.264	154	Glioma_grade 2_6.raw
4506455			iDNDGDGFVTTEELk	Unambiguous	N-Term(iTRAQ4plex); K15(iTRAQ4plex)	HCD	3.9792378	0	1	1	3.66153	4.1399	3.8371	4.3384595	7.581432051	25421.678	22483.947	93082.18	97545.695	8.672424215	3.311353835	2	High	4553	Unique	Used	52064	1	45.155	1	6.5256	-1.4638	1	77	24/28	0	4553	1940.97	MS2	970.987	173	Glioma_grade 2_2.raw
4506455			eEFTAFLHPEEFEHMk	Unambiguous	N-Term(iTRAQ4plex); K16(iTRAQ4plex)	HCD	2.934872	0	1	1	2.66576	3.0069	2.871	3.238421	8.161705195	24368.01	21603.107	64959.383	69959.95	8.505695699	5.241553982	3	High	5104	Unique	Used	92293	1	56.705	1	5.3966	0.0217	1	26	30/60	24.1234	5104	2309.12	MS2	770.377	68	Glioma_grade 2_7rep.raw
4506455			eEFTAFLHPEEFEHmk	Unambiguous	N-Term(iTRAQ4plex); M15(Oxidation); K16(iTRAQ4plex)	HCD	2.2729154	0	1	1	2.09749	2.114	2.4306	2.4497213	8.506890414	18840.96	18693.957	39518.707	45794.99	0.553867313	10.40396191	3	High	4714	Unique	Used	92003	1	52.63	1	5.631	-1.9127	1	25	23/60	42.52776	4714	2325.11	MS2	775.707	75	Glioma_grade 2_7rep.raw
4506457	NP_002893.1	RCN2	reticulocalbin-2 precursor [Homo sapiens]	4	5	2.181978941	37.38734761	31.029608	22.08	9.35E-05	317	36.854	4.3989	Calcium binding protein	Cell communication,Signal transduction	Calcium ion binding	Endoplasmic reticulum	-																								
4506457			qQFVEYDk	Unambiguous	N-Term(iTRAQ4plex); K8(iTRAQ4plex)	HCD	2.1241705	0	1	1	1.96251	2.1369	2.1125	2.300204	6.498050971	823820.2	756576.44	1616751.8	1740280.2	6.017289266	5.203868733	2	High	1550	Unique	Used	49232	1	23.675	1	3.8804	-1.1075	1	5	14/14	14.39937	1550	1344.7	MS2	672.855	182	Glioma_grade 2_2.raw
4506457			aEELHYPLGER	Unambiguous	N-Term(iTRAQ4plex)	HCD	2.181979	0	1	1	2.22222	2.0668	2.3058	2.1445506	4.695341568	180729.03	194316.28	401619.9	416721.1	5.12345381	2.609709381	2	High	1775	Unique	Used	57496	1	24.79	1	4.125	-0.4313	1	10	17/20	7.808459	1775	1457.75	MS2	729.379	161	Glioma_grade 2_3.raw
4506457			wDPTANEDPEWILVEk	Unambiguous	N-Term(iTRAQ4plex); K16(iTRAQ4plex)	HCD	2.1407561	0	1	1	1.98704	2.1331	2.1478	2.3057706	6.079102312	66668.86	62102.582	132473.94	143194.3	5.01484815	5.4996827	2	High	7010	Unique	Used	54373	1	63.165	1	5.9318	-2.6752	1	38	18/30	7.055596	7010	2230.12	MS2	1115.56	166	Glioma_grade 2_2.raw
4506457			qLHDDYFYHDEL	Unambiguous	N-Term(iTRAQ4plex)	HCD	2.8099008	0	1	1	2.43781	3.0817	2.5949	3.2802694	13.95012885	46733.77	36969.62	113927.9	121270.32	16.49705389	4.414893082	3	High	4326	Unique	Used	67545	1	43.438	1	4.9548	0.0574	1	7	23/44	11.45633	4326	1738.78	MS2	580.266	25	Glioma_grade 2_4.raw
4506457			qLHDDYFYHDEL	Unambiguous	N-Term(iTRAQ4plex)	HCD	4.5278983	0	1	1	4.60435	4.4138	4.6469	4.454609	2.496317194	17291.598	18037.908	79616.58	80351.83	2.987422818	0.650003661	2	High	4317	Unique	Used	67537	1	43.359	1	4.0594	2.9084	1	83	22-Nov	0	4317	1738.79	MS2	869.898	28	Glioma_grade 2_4.raw
4506467	NP_002897.1	RDX	radixin [Homo sapiens]	5	7	2.29739809	21.12627439	135.65818	28.13	1.49E-04	583	68.521	6.3667	Cytoskeletal associated protein	Cell growth and/or maintenance	Cytoskeletal protein binding	Plasma membrane	Cytoplasm																								
4506467			aFAAQEDLEk	Unambiguous	N-Term(iTRAQ4plex); K10(iTRAQ4plex)	HCD	2.7483757	0	1	1	2.81759	2.6652	2.8363	2.6829097	3.23227559	450261.2	476007.2	1268649.2	1277084.2	3.930863061	0.468583686	2	High	1971	Unique	Used	49631	1	26.582	1	4.391	-0.3498	1	5	18/18	17.89735	1971	1409.75	MS2	705.379	247	Glioma_grade 2_2.raw
4506467			iALLEEAk	Unambiguous	N-Term(iTRAQ4plex); K8(iTRAQ4plex)	HCD	1.8667403	0	1	1	1.86356	1.9187	1.8163	1.8700187	2.243540735	268864.84	261139.39	501044.75	488335.56	2.061386598	1.816643073	2	High	3872	Unique	Used	51422	1	40.145	1	3.1558	-0.644	1	13	14/14	30.37067	3872	1174.73	MS2	587.868	219	Glioma_grade 2_2.raw
4506467			iALLEEAk	Unambiguous	N-Term(iTRAQ4plex); K8(iTRAQ4plex)	HCD	2.400435	0	1	1	2.31151	2.3486	2.4514	2.4907842	3.514791206	162979.23	160405	376728.6	399534.22	1.125754023	4.154780607	2	High	3794	Unique	Used	59377	1	39.956	1	3.3331	-0.2283	1	28	14/14	45.37353	3794	1174.73	MS2	587.868	236	Glioma_grade 2_3.raw
4506467			nQEQLAAELAEFTAk	Unambiguous	N-Term(iTRAQ4plex); K15(iTRAQ4plex)	HCD	3.1780448	0	1	1	2.99179	3.4519	2.9407	3.3929384	8.304825711	20809.336	18035.729	62257.242	61194.113	10.09773735	1.217881691	2	High	6643	Unique	Used	54031	1	60.4	1	6.197	-1.7642	1	75	23/28	15.81237	6643	1951.03	MS2	976.021	206	Glioma_grade 2_2.raw
4506467			kTQNDVLHAENVk	Unambiguous	N-Term(iTRAQ4plex); K1(iTRAQ4plex); K13(iTRAQ4plex)	HCD	2.297398	1	1	1	2.14618	2.3064	2.2891	2.4599	5.577624312	17470.12	16256.854	37494.05	39990.234	5.08737378	4.555939714	2	High	1053	Unique	Used	88983	1	19.124	1	4.2283	-0.6349	1	93	18/24	26.56206	1053	1928.09	MS2	964.549	155	Glioma_grade 2_7rep.raw
4506467			nQEQLAAELAEFTAk	Unambiguous	N-Term(iTRAQ4plex); K15(iTRAQ4plex)	HCD	2.110131	0	1	1	1.91938	2.3867	1.8877	2.347332	12.56994735	10176.666	8183.8804	19532.871	19210.283	15.34934942	1.177519839	3	High	6690	Unique	Used	54073	1	60.785	1	5.7727	0.7941	1	29	27/56	20.71005	6690	1951.04	MS2	651.018	176	Glioma_grade 2_2.raw
4506467			gTELWLGVDALGLNIYEHDDk	Unambiguous	N-Term(iTRAQ4plex); K21(iTRAQ4plex)	HCD	1.7788078	0	1	1	1.62048	1.7073	1.8466	1.9456228	8.115312434	1781.6235	1690.9926	2887.0828	3290.0337	3.690919015	9.225317796	3	High	7314	Unique	Used	77447	1	70.602	1	7.4485	-2.4893	1	100	37/80	35.91682	7314	2646.36	MS2	882.791	152	Glioma_grade 2_5.raw
4506597	NP_000967.1	RPL12	60S ribosomal protein L12 [Homo sapiens]	4	19	2.259622574	21.41309905	93.624828	35.15	2.72E-04	165	17.808	9.4224	Ribosomal subunit	Protein metabolism	Structural constituent of ribosome	Nucleus	Mitochondrion, Ribosome, Nucleolus																								
4506597			iGPLGLSPk	Unambiguous	N-Term(iTRAQ4plex); K9(iTRAQ4plex)	HCD	3.1345198	0	1	1	3.07507	3.1752	3.0951	3.1959054	1.887431755	1232098.5	1193247	3788789	3813504.5	2.265422317	0.459769085	2	High	3542	Unique	Used	66817	1	37.344	1	3.3154	1.2729	1	2	15/16	5.484012	3542	1169.75	MS2	585.379	65	Glioma_grade 2_4.raw
4506597			iGPLGLSPk	Unambiguous	N-Term(iTRAQ4plex); K9(iTRAQ4plex)	HCD	2.5366952	0	1	1	2.3525	2.5152	2.5568	2.73363	6.165478732	837719.06	783533.06	1970733.4	2141889.2	4.726629199	5.885559585	2	High	3019	Unique	Used	73655	1	34.621	1	3.3162	-0.5012	1	3	14/16	18.05797	3019	1169.75	MS2	585.378	64	Glioma_grade 2_5.raw
4506597			iGPLGLSPk	Unambiguous	N-Term(iTRAQ4plex); K9(iTRAQ4plex)	HCD	2.2596226	0	1	1	2.0536	2.4309	2.115	2.503492	9.862478877	329910.03	278710.34	677503.1	697749.2	11.89695573	2.081967738	2	High	3641	Unique	Used	66909	1	38.112	1	3.2984	-0.5012	1	10	14/16	29.2183	3641	1169.75	MS2	585.378	67	Glioma_grade 2_4.raw
4506597			iGPLGLSPk	Unambiguous	N-Term(iTRAQ4plex); K9(iTRAQ4plex)	HCD	2.2475283	0	1	1	2.00618	2.1945	2.296	2.5115309	9.350206153	311856.38	285092.47	625640.9	716018.6	6.340557404	9.526513178	2	High	3117	Unique	Used	73747	1	35.341	1	3.1691	0.0206	1	8	14/16	27.01007	3117	1169.75	MS2	585.379	67	Glioma_grade 2_5.raw
4506597			iGPLGLSPk	Unambiguous	N-Term(iTRAQ4plex); K9(iTRAQ4plex)	HCD	1.6126122	0	1	1	1.46276	1.7255	1.5169	1.7893844	9.746702732	231980.33	196653.55	339331.47	351888.8	11.65554421	2.569187734	2	High	3256	Unique	Used	58878	1	35.791	1	3.0518	-0.9186	1	16	14/16	27.71402	3256	1169.75	MS2	585.378	113	Glioma_grade 2_3.raw
4506597			cTGGEVGATSALAPk	Unambiguous	N-Term(iTRAQ4plex); C1(Methylthio); K15(iTRAQ4plex)	HCD	2.262056	0	1	1	2.08543	2.3142	2.2151	2.4580548	6.942463627	142677.55	128573.086	297544.47	316039.7	7.353613829	4.262855266	2	High	2566	Unique	Used	50195	1	30.691	1	5.9864	1.6116	1	21	23/28	4.409869	2566	1695.87	MS2	848.437	173	Glioma_grade 2_2.raw
4506597			hSGNITFDEIVNIAR	Unambiguous	N-Term(iTRAQ4plex)	HCD	3.1744251	0	1	1	2.96963	3.022	3.3242	3.3828294	6.582994738	108116.03	106241.84	321064.97	359398.06	1.236485937	7.966806921	2	High	5845	Unique	Used	68942	1	55.413	1	5.3823	1.1339	1	22	24/28	0	5845	1829.96	MS2	915.486	144	Glioma_grade 2_4.raw
4506597			hSGNITFDEIVNIAR	Unambiguous	N-Term(iTRAQ4plex)	HCD	2.5804296	0	1	1	2.57518	2.6262	2.5355	2.5857792	1.445102713	59268.047	58115.82	152626.12	150274.67	1.388176324	1.09786854	2	High	5932	Unique	Used	69022	1	56.117	1	4.7404	1.8677	1	28	23/28	5.950813	5932	1829.97	MS2	915.487	147	Glioma_grade 2_4.raw
4506597			iGPLGLSPk	Unambiguous	N-Term(iTRAQ4plex); K9(iTRAQ4plex)	HCD	2.026046	0	1	1	2.0103	2.0052	2.047	2.0417488	1.054378785	51355.777	51487.477	103240.68	105124.49	0.181102716	1.278577245	2	High	3461	Unique	Used	66744	1	36.655	1	3.2768	-0.5012	1	70	14/16	29.85461	3461	1169.75	MS2	585.378	67	Glioma_grade 2_4.raw
4506597			hSGNITFDEIVNIAR	Unambiguous	N-Term(iTRAQ4plex)	HCD	3.1738424	0	1	1	3.20205	2.9884	3.3726	3.1475155	4.986731095	37414.35	40089.668	119802.69	126182.85	4.881644974	3.668064717	2	High	5390	Unique	Used	75770	1	54.022	1	5.1424	1.7342	1	53	23/28	16.3521	5390	1829.97	MS2	915.487	162	Glioma_grade 2_5.raw
4506597			hSGNITFDEIVNIAR	Unambiguous	N-Term(iTRAQ4plex)	HCD	2.2021923	0	1	1	2.06043	2.2152	2.1901	2.354599	5.467398532	22050.984	20510.658	45434.547	48294.375	5.118105922	4.315004864	2	High	5725	Unique	Used	61167	1	55.032	1	5.0632	0.7337	1	100	24/28	17.15972	5725	1829.96	MS2	915.486	161	Glioma_grade 2_3.raw
4506597			hSGNITFDEIVNIAR	Unambiguous	N-Term(iTRAQ4plex)	HCD	2.1439943	0	1	1	2.09311	2.1575	2.1309	2.1964383	2.030810185	20804.291	20183.854	43545.758	44332.586	2.140693169	1.266230994	3	High	5721	Unique	Used	61163	1	55.006	1	3.6589	0.3594	1	32	25/56	38.58752	5721	1829.96	MS2	610.659	160	Glioma_grade 2_3.raw
4506597			hPHDIIDDINSGAVEcPAS	Unambiguous	N-Term(iTRAQ4plex); C16(Methylthio)	HCD	2.2555943	0	1	1	2.95167	2.2905	2.2106	1.7154331	22.16397421	18498.406	23837.732	54601.113	40892.035	17.8357016	20.30257086	3	High	5295	Unique	Used	75684	1	53.275	1	4.8092	-1.4759	1	3	24/72	7.846462	5295	2179.98	MS2	727.333	26	Glioma_grade 2_5.raw
4506597			iGPLGLSPk	Unambiguous	N-Term(iTRAQ4plex); K9(iTRAQ4plex)	HCD	2.6553159	0	1	1	2.40076	2.7093	2.6075	2.9425855	8.450272818	17868.633	15833.674	42898.336	46591.938	8.539078991	5.836994132	3	High	3557	Unique	Used	66831	1	37.453	1	3.3642	-0.2257	1	26	16/32	7.380979	3557	1169.75	MS2	390.588	65	Glioma_grade 2_4.raw
4506597			hSGNITFDEIVNIAR	Unambiguous	N-Term(iTRAQ4plex)	HCD	1.5642858	0	1	1	1.69653	1.4685	1.6749	1.4498177	8.352037057	14970.037	17294.55	25397.09	25073.945	10.18874908	0.905461997	3	High	5384	Unique	Used	75764	1	53.986	1	5.7872	0.6596	1	14	27/56	8.936837	5384	1829.96	MS2	610.66	222	Glioma_grade 2_5.raw
4506597			hPHDIIDDINSGAVEcPAS	Unambiguous	N-Term(iTRAQ4plex); C16(Methylthio)	HCD	3.0672114	0	1	1	2.66951	3.306	2.8744	3.5597386	13.02701678	14152.627	11427.768	37780.61	40679.867	15.06439816	5.225775737	2	High	5284	Unique	Used	75674	1	53.184	1	3.7284	-0.6018	1	17	13/36	0	5284	2179.99	MS2	1090.5	21	Glioma_grade 2_5.raw
4506597			hSGNITFDEIVNIAR	Unambiguous	N-Term(iTRAQ4plex)	HCD	2.311309	0	1	1	2.129	2.3865	2.2443	2.515664	7.255210067	13169.676	11748.908	28038.242	29556.307	8.063336884	3.727554341	2	High	6083	Unique	Used	69164	1	57.261	1	4.7242	3.4686	1	100	22/28	18.21268	6083	1829.97	MS2	915.488	161	Glioma_grade 2_4.raw
4506597			hSGNITFDEIVNIAR	Unambiguous	N-Term(iTRAQ4plex)	HCD	1.9453169	0	1	1	1.79929	1.9363	1.9537	2.1024566	6.366652454	11480.57	10668.487	20656.904	22430.031	5.18513629	5.819815801	2	High	6166	Unique	Used	69239	1	57.945	1	4.1721	3.5353	1	100	20/28	24.02559	6166	1829.97	MS2	915.488	219	Glioma_grade 2_4.raw
4506597			hSGNITFDEIVNIAR	Unambiguous	N-Term(iTRAQ4plex)	HCD	1.7140061	0	1	1	1.83372	1.6663	1.7665	1.6052217	5.929750701	9583.951	10546.782	17574.275	16929.924	6.764029201	2.640982691	3	High	6045	Unique	Used	69128	1	56.976	1	5.9774	0.0592	1	46	26/56	12.16483	6045	1829.96	MS2	610.659	172	Glioma_grade 2_4.raw
4506605	NP_000969.1	RPL23	60S ribosomal protein L23 [Homo sapiens]	4	10	2.056071758	13.90503498	53.275831	37.14	1.05E-04	140	14.856	10.506	Ribosomal subunit	Protein metabolism	Structural constituent of ribosome	Ribosome	-																								
4506605			nLYIISVk	Unambiguous	N-Term(iTRAQ4plex); K8(iTRAQ4plex)	HCD	2.0850146	0	1	1	2.02967	2.1073	2.0635	2.1424756	2.368644468	451796.88	435147.8	916999	932293.56	2.65465877	1.169624247	2	High	4183	Unique	Used	51715	1	42.436	1	3.686	-0.725	1	10	13/14	31.19766	4183	1237.77	MS2	619.391	118	Glioma_grade 2_2.raw
4506605			lPAAGVGDMVMATVk	Unambiguous	N-Term(iTRAQ4plex); K15(iTRAQ4plex)	HCD	2.5627158	0	1	1	2.40897	2.5515	2.5733	2.725563	5.05246975	87059.56	82196.37	209723.48	224031.38	4.063425875	4.664945133	2	High	6274	Unique	Used	61680	1	59.294	1	6.1152	1.9281	1	25	22/28	14.87778	6274	1747.97	MS2	874.49	204	Glioma_grade 2_3.raw
4506605			lPAAGVGDMVMATVk	Unambiguous	N-Term(iTRAQ4plex); K15(iTRAQ4plex)	HCD	2.1734016	0	1	1	2.10372	2.1525	2.1939	2.2446935	2.759462586	76124.14	74400.09	160144.19	167005.4	1.619788982	2.965987589	2	High	6414	Unique	Used	53816	1	58.724	1	5.7966	0.3219	1	56	21/28	22.54514	6414	1747.97	MS2	874.488	212	Glioma_grade 2_2.raw
4506605			lPAAGVGDMVmATVk	Unambiguous	N-Term(iTRAQ4plex); M11(Oxidation); K15(iTRAQ4plex)	HCD	2.0534844	0	1	1	1.87333	2.0927	2.0184	2.2547388	7.705577854	42848.836	38356.582	80269.98	86484.08	7.823377662	5.270075282	2	High	5280	Unique	Used	52747	1	50.464	1	5.4033	0.0184	1	57	20/28	23.66981	5280	1763.96	MS2	882.486	239	Glioma_grade 2_2.raw

4506605			kVHPAVVIR	Unambiguous	N-Term(iTRAQ4plex); K1(iTRAQ4plex)	HCD	1.8730742	1	1	1	1.74437	1.9431	1.8102	2.0164344	6.580461046	26230.572	23548.379	45755.91	47483.76	7.620075637	2.620718095	3	High	1010	Unique	Used	94081	1	18.845	1	3.6573	-0.547	1	100	18/32	57.31432	1010	1306.86	MS2	436.29	37	Glioma_grade2_8rep.raw
4506605			kVHPAVVIR	Unambiguous	N-Term(iTRAQ4plex); K1(iTRAQ4plex)	HCD	2.0136187	1	1	1	1.88118	2.0199	2.0078	2.1558228	5.568165148	25473.82	23724.627	47920.83	51146.086	5.028070223	4.604161471	2	High	1019	Unique	Used	94089	1	18.924	1	3.0971	-0.7002	1	100	15/16	32.27338	1019	1306.86	MS2	653.931	41	Glioma_grade2_8rep.raw
4506605			lPAAGVGDMVmATVk	Unambiguous	N-Term(iTRAQ4plex); M11(Oxidation); K15(iTRAQ4plex)	HCD	1.5027043	0	1	1	1.45467	1.4726	1.5325	1.551335	3.089457364	17512.16	17299.186	25474.328	26836.832	0.865208485	3.683480993	2	High	5160	Unique	Used	60646	1	50.597	1	4.2635	0.918	1	100	19/28	15.60177	5160	1763.97	MS2	882.486	289	Glioma_grade2_3.raw
4506605			lPAAGVGDMVMATVk	Unambiguous	N-Term(iTRAQ4plex); K15(iTRAQ4plex)	HCD	2.0586588	0	1	1	2.39667	1.9463	2.197	1.7841692	12.99424886	11469.334	14123.491	27488.197	25198.697	14.66639508	6.145440935	3	High	6273	Unique	Used	61679	1	59.288	1	4.6527	-0.0349	1	16	25/56	12.93838	6273	1747.97	MS2	583.328	247	Glioma_grade2_3.raw
4506605			iSLGLPVGAVINcADNTGAk	Unambiguous	N-Term(iTRAQ4plex); C13(Methylthio); K20(iTRAQ4plex)	HCD	2.2029588	0	1	1	2.19846	2.0587	2.357	2.2071743	5.526429876	7634.0576	8152.325	16783.148	17993.604	4.642867239	4.922378294	2	High	7541	Unique	Used	62839	1	69.541	1	5.6266	-0.0966	1	100	24/38	14.6894	7541	2247.21	MS2	1124.11	166	Glioma_grade2_3.raw
4506605			iSLGLPVGAVINcADNTGAk	Unambiguous	N-Term(iTRAQ4plex); C13(Methylthio); K20(iTRAQ4plex)	HCD	1.7573695	0	1	1	1.55245	1.5649	1.9484	1.9639249	13.06663515	4383.4385	4348.6895	6805.076	8540.5	0.562778135	15.99327492	3	High	7858	Unique	Used	55145	1	69.971	1	5.6724	2.3419	1	26	28/76	6.668609	7858	2247.21	MS2	749.743	136	Glioma_grade2_2.raw
4506607	NP_000970.1	RPL18	60S ribosomal protein L18 [Homo sapiens]	3	7	2.471544266	36.24124103	39.89743	20.21	9.51E-05	188	21.621	11.722	Ribosomal subunit	Protein metabolism	Structural constituent of ribosome	Ribosome	Mitochondrion, Nucleolus																								
4506607			tNSTFNQVVLk	Unambiguous	N-Term(iTRAQ4plex); K11(iTRAQ4plex)	HCD	2.4715443	0	1	1	2.4533	2.3838	2.5619	2.4892704	3.000938496	279553.8	287707.88	685829.75	716182.7	2.032855546	3.061709869	2	High	2520	Unique	Used	50151	1	30.385	1	4.9289	-0.4746	1	12	19/20	11.32822	2520	1538.88	MS2	769.942	223	Glioma_grade2_2.raw
4506607			iLTFDQLALDSPk	Unambiguous	N-Term(iTRAQ4plex); K13(iTRAQ4plex)	HCD	2.1411736	0	1	1	2.37033	2.1176	2.1675	1.9364486	8.305863318	108780.8	121762.2	257846.34	235786.25	7.963144376	6.32002001	2	High	6650	Unique	Used	54037	1	60.464	1	5.8916	0.7634	1	19	22/24	12.87277	6650	1749.01	MS2	875.006	213	Glioma_grade2_2.raw
4506607			iLTFDQLALDSPk	Unambiguous	N-Term(iTRAQ4plex); K13(iTRAQ4plex)	HCD	2.8356042	0	1	1	2.67585	2.9346	2.7454	3.010806	5.527060383	91989.78	83880.13	246150.67	252546.83	6.521170686	1.813832277	2	High	6439	Unique	Used	61835	1	60.568	1	6.1112	2.0197	1	25	21/24	32.69098	6439	1749.01	MS2	875.007	210	Glioma_grade2_3.raw
4506607			aPGTPHSHTkPYVR	Unambiguous	N-Term(iTRAQ4plex); K10(iTRAQ4plex)	HCD	2.2160397	0	1	1	1.90592	2.1648	2.2612	2.5682797	12.29676597	10850.628	9553.072	20680.45	24534.96	8.99357123	12.05584627	3	High	832	Unique	Used	93936	1	17.087	1	4.3002	0.7802	1	100	18/52	27.30231	832	1836.01	MS2	612.676	138	Glioma_grade2_8rep.raw
4506607			iLTFDQLALDSPk	Unambiguous	N-Term(iTRAQ4plex); K13(iTRAQ4plex)	HCD	1.5849202	0	1	1	1.42001	1.5412	1.6252	1.7638993	9.108933929	7425.85	6842.0317	10544.798	12068.655	5.78672977	9.529987466	3	High	6633	Unique	Used	54022	1	60.319	1	4.6184	-1.0938	1	30	19/48	41.29067	6633	1749	MS2	583.672	205	Glioma_grade2_2.raw
4506607			iLTFDQLALDSPk	Unambiguous	N-Term(iTRAQ4plex); K13(iTRAQ4plex)	HCD	2.5682628	0	1	1	2.1609	2.6032	2.5393	3.059006	14.21519617	7086.664	5882.643	15313.55	17995.041	13.12901937	11.3850536	3	High	6442	Unique	Used	61838	1	60.587	1	4.6675	0.5812	1	34	20/48	13.90055	6442	1749	MS2	583.673	195	Glioma_grade2_3.raw
4506607			aPGTPHSHTkPYVR	Unambiguous	N-Term(iTRAQ4plex); K10(iTRAQ4plex)	HCD	4.600235	0	1	1	4.95847	4.2884	4.9608	4.2904086	8.367558422	2831.53	3273.9634	14040.063	14046.641	10.24807127	0.033121355	5	High	831	Unique	Used	93935	1	17.082	1	4.6607	0.1838	1	82	20/104	4.532337	831	1836.01	MS2	368.008	230	Glioma_grade2_8rep.raw
4506623	NP_000979.1	RPL27	60S ribosomal protein L27 [Homo sapiens]	2	3	2.182480097	17.85200377	13.659549	23.53	2.65E-05	136	15.788	10.565	Ribosomal subunit	Protein metabolism	Structural constituent of ribosome	Cytoplasm	Nucleolus																								
4506623			vYNYNHLMPTR	Unambiguous	N-Term(iTRAQ4plex)	HCD	2.18248	0	1	1	2.18487	2.158	2.2073	2.1801157	0.925856763	48001.758	48599.57	104877.8	105952.68	0.875178277	0.721010489	2	High	2564	Unique	Used	65909	1	29.835	1	3.2019	14.514	1	70	17/20	41.82664	2564	1551.81	MS2	776.408	233	Glioma_grade2_4.raw
4506623			vYNYNHLMPTR	Unambiguous	N-Term(iTRAQ4plex)	HCD	1.9093506	0	1	1	1.83837	1.9078	1.9109	1.9830142	3.092724317	30515.104	29405.168	56097.945	58310.867	2.619625199	2.735404948	2	High	2658	Unique	Used	65996	1	30.555	1	3.2413	13.727	1	100	17/20	0	2658	1551.81	MS2	776.407	215	Glioma_grade2_4.raw
4506623			nIDDGTSDRPYSHALVAGIDR	Unambiguous	N-Term(iTRAQ4plex)	HCD	2.7053523	0	1	1	2.41901	2.9615	2.4961	3.0559053	11.78387091	19672.824	16069.106	47588.85	49105.664	14.25895823	2.218428783	3	High	2609	Unique	Used	73278	1	31.472	1	3.355	11.922	1	18	22/80	3.77176	2609	2416.23	MS2	806.08	101	Glioma_grade2_5.raw
4506635	NP_000985.1	RPL32	60S ribosomal protein L32 [Homo sapiens]	3	7	2.362955809	22.24526481	31.146775	27.41	1.01E-03	135	15.85	11.327	Ribosomal subunit	Protein metabolism	Structural constituent of ribosome	Ribosome	-																								
4506635			sYcAEIAHNVSSk	Unambiguous	N-Term(iTRAQ4plex); C3(Methylthio); K13(iTRAQ4plex)	HCD	2.3629558	0	1	1	2.0916	2.6552	2.1328	2.707434	13.75631651	91984.73	72460.445	192394.81	196181.86	16.79070769	1.378285905	3	High	3423	Unique	Used	74032	1	37.631	1	3.8671	1.5215	1	4	26/48	7.356153	3423	1742.85	MS2	581.621	77	Glioma_grade2_5.raw
4506635			gQILmPNIGYGSNk	Unambiguous	N-Term(iTRAQ4plex); M5(Oxidation); K14(iTRAQ4plex)	HCD	2.0058413	0	1	1	1.89101	1.9459	2.0641	2.1240113	5.316979616	62108.812	60356.664	117448.07	128198.23	2.023355109	6.188991033	2	High	2866	Unique	Used	50477	1	32.847	1	3.629	0.9188	1	40	15/26	0	2866	1795.96	MS2	898.485	237	Glioma_grade2_2.raw
4506635			sYcAEIAHNVSSk	Unambiguous	N-Term(iTRAQ4plex); C3(Methylthio); K13(iTRAQ4plex)	HCD	1.8779063	0	1	1	1.91273	1.9028	1.8529	1.8432591	1.860148274	56961.3	57258.242	108951.83	105541.77	0.367659856	2.248343587	2	High	3432	Unique	Used	74041	1	37.683	1	5.4125	4.4709	1	43	22/24	10.35443	3432	1742.85	MS2	871.93	121	Glioma_grade2_5.raw
4506635			eLEVLLMcNk	Unambiguous	N-Term(iTRAQ4plex); C8(Methylthio); K10(iTRAQ4plex)	HCD	2.0203574	0	1	1	1.95937	1.9827	2.0576	2.0820723	2.904968081	52425.805	51809.414	102721.445	107870.95	0.836289807	3.458101994	2	High	7102	Unique	Used	62453	1	65.714	1	4.7886	1.7862	1	62	15/18	30.72894	7102	1525.81	MS2	763.407	231	Glioma_grade2_3.raw
4506635			gQILMPNIGYGSNk	Unambiguous	N-Term(iTRAQ4plex); K14(iTRAQ4plex)	HCD	2.620491	0	1	1	2.58422	2.6961	2.548	2.6583369	2.578650232	42346.945	40589.645	109433.63	107900.95	2.996503103	0.997330459	2	High	4467	Unique	Used	51983	1	44.517	1	4.6242	1.705	1	50	18/26	22.22582	4467	1779.97	MS2	890.488	242	Glioma_grade2_2.raw
4506635			gQILMPNIGYGSNk	Unambiguous	N-Term(iTRAQ4plex); K14(iTRAQ4plex)	HCD	2.9902725	0	1	1	2.80003	3.1532	2.8456	3.2045114	6.91333985	22069.738	19597.902	61795.902	62801.703	8.38949361	1.141608954	2	High	4344	Unique	Used	59891	1	44.174	1	4.7016	-0.6268	1	77	17/26	7.619229	4344	1779.97	MS2	890.486	255	Glioma_grade2_3.raw
4506635			gQILmPNIGYGSNk	Unambiguous	N-Term(iTRAQ4plex); M5(Oxidation); K14(iTRAQ4plex)	HCD	3.3186088	0	1	1	3.23212	3.0389	3.6161	3.3999255	7.389188403	18296.014	19459.062	59134.97	66159.36	4.356495681	7.928521271	2	High	2921	Unique	Used	58564	1	33.305	1	4.1238	-0.8484	1	44	18/26	0	2921	1795.96	MS2	898.484	241	Glioma_grade2_3.raw
4506645	NP_000990.1	RPL38	60S ribosomal protein L38 [Homo sapiens]	2	6	2.433260441	23.94861075	23.86949	28.57	1.05E-04	70	8.2127	10.096	Ribosomal subunit	Protein metabolism	Structural constituent of ribosome	Ribosome	-																								
4506645			qSLPPGLAVk	Unambiguous	N-Term(iTRAQ4plex); K10(iTRAQ4plex)	HCD	2.8466733	0	1	1	2.74796	2.8523	2.8413	2.9491363	2.888655282	239422.94	230667.86	657924	680270.94	2.633864112	2.361639899	2	High	2254	Unique	Used	57941	1	28.36	1	3.2462	12.163	1	11	16/18	10.0408	2254	1297.82	MS2	649.416	68	Glioma_grade2_3.raw
4506645			qSLPPGLAVk	Unambiguous	N-Term(iTRAQ4plex); K10(iTRAQ4plex)	HCD	2.476761	0	1	1	2.37297	2.5134	2.4422	2.586697	3.710681359	138082.19	130366.99	327664.6	337219.9	4.064434273	2.03241839	2	High	2328	Unique	Used	58011	1	28.886	1	3.2272	-2.3223	1	22	14/18	44.48817	2328	1297.81	MS2	649.406	132	Glioma_grade2_3.raw
4506645			qSLPPGLAVk	Unambiguous	N-Term(iTRAQ4plex); K10(iTRAQ4plex)	HCD	2.3897598	0	1	1	2.21464	2.4245	2.358	2.5814738	6.357948498	88695.57	81017.516	196428.9	209144.6	6.398097139	4.433898022	2	High	2147	Unique	Used	57843	1	27.518	1	3.437	11.316	1	47	15/18	29.27835	2147	1297.82	MS2	649.415	69	Glioma_grade2_3.raw
4506645			qSLPPGLAVk	Unambiguous	N-Term(iTRAQ4plex); K10(iTRAQ4plex)	HCD	2.3766768	0	1	1	2.23839	2.5735	2.2055	2.535659	8.086030008	60300.88	52449.69	134977.14	132994.53	9.847630375	1.046317303	2	High	2414	Unique	Used	58091	1	29.524	1	3.2566	-0.0649	1	60	16/18	20.00249	2414	1297.81	MS2	649.408	149	Glioma_grade2_3.raw
4506645			yLYTLVITDk	Unambiguous	N-Term(iTRAQ4plex); K10(iTRAQ4plex)	HCD	2.2469857	0	1	1	2.29501	2.3056	2.1886	2.19874	2.752590514	50066.055	49835.816	114902.055	109576.01	0.325926945	3.355412509	2	High	6307	Unique	Used	53717	1	57.914	1	4.2408	-2.411	1	33	15/18	24.47123	6307	1516.88	MS2	758.945	193	Glioma_grade2_2.raw
4506645			yLYTLVITDk	Unambiguous	N-Term(iTRAQ4plex); K10(iTRAQ4plex)	HCD	3.983236	0	1	1	4.27664	3.8029	4.1861	3.7223384	6.883156512	10142.067	11405.579	43373.938	42455.426	8.292672929	1.513435574	3	High	6318	Unique	Used	53728	1	57.982	1	3.4991	-4.3513	1	34	15/36	21.04688	6318	1516.88	MS2	506.298	184	Glioma_grade2_2.raw
4506661	NP_000963.1	RPL7A	60S ribosomal protein L7a [Homo sapiens]	4	9	2.093409538	24.8023343	56.229189	21.8	7.61E-05	266	29.977	10.609	Ribosomal subunit	Protein metabolism	Structural constituent of ribosome	Cytoplasm	Ribosome, Nucleolus, Nucleus, Extracellular																								
4506661			vAPAPAVVk	Unambiguous	N-Term(iTRAQ4plex); K9(iTRAQ4plex)	HCD	3.3007922	0	1	1	3.61181	3.0661	3.5773	3.036765	9.452540954	1155319.6	1360947	4172797.2	4132876.5	11.55684608	0.679732884	2	High	1262	Unique	Used	64700	1	20.216	1	3.3536	0.611	1	1	15/16	7.608314	1262	1139.74	MS2	570.374	70	Glioma_grade2_4.raw
4506661			kVVNPLFEk	Unambiguous	N-Term(iTRAQ4plex); K1(iTRAQ4plex); K9(iTRAQ4plex)	HCD	1.9811051	1	1	1	1.83044	2.0811	1.8932	2.1523993	7.651616751	366156.5	322056.3	670228.1	693193.8	9.062182648	2.382124301	3	High	2092	Unique	Used	89911	1	27.347	1	4.3248	-2.8217	1	2	21/32	12.27305	2092	1505.94	MS2	502.651	80	Glioma_grade2_7rep.raw
4506661			nFGIGQDIQPk	Unambiguous	N-Term(iTRAQ4plex); K11(iTRAQ4plex)	HCD	2.974862	0	1	1	2.86092	2.8708	3.0785	3.089195	4.234969913	251463.6	250594.28	719418.4	774134.6	0.244872988	5.180960577	2	High	2914	Unique	Used	58557	1	33.267	1	4.3789	-0.661	1	7	19/20	4.31124	2914	1504.84	MS2	752.921	255	Glioma_grade2_3.raw
4506661			nFGIGQDIQPk	Unambiguous	N-Term(iTRAQ4plex); K11(iTRAQ4plex)	HCD	2.148896	0	1	1	2.0169	2.0717	2.224	2.284486	5.848514346	250154.8	243532.12	504536.06	556345.75	1.897130245	6.906515464	2	High	2777	Unique	Used	50393	1	32.208	1	4.584	0.6369	1	18	19/20	15.60253	2777	1504.84	MS2	752.922	270	Glioma_grade2_2.raw
4506661			kVVNPLFEk	Unambiguous	N-Term(iTRAQ4plex); K1(iTRAQ4plex); K9(iTRAQ4plex)	HCD	2.0309517	1	1	1	1.88365	2.113	1.9578	2.196184	6.991684222	134052.78	119504.516	252508.73	262453.9	8.114281301	2.731187367	2	High	2071	Unique	Used	89893	1	27.165	1	4.0446	-3.1775	1	58	16/16	25.2758	2071	1505.94	MS2	753.472	81	Glioma_grade2_7rep.raw
4506661			aGVNTVTTLVENk	Unambiguous	N-Term(iTRAQ4plex); K13(iTRAQ4plex)	HCD	1.6457069	0	1	1	1.38437	1.7092	1.5943	1.9683566	14.62656018	72329.49	58585.055	100130.68	115316.28	14.84752239	9.967966813	2	High	4408	Unique	Used	51927	1	44.088	1	5.4797	1.9238	1	53	20/24	8.623209	4408	1633.94	MS2	817.473	206	Glioma_grade2_2.raw
4506661			aGVNTVTTLVENk	Unambiguous	N-Term(iTRAQ4plex); K13(iTRAQ4plex)	HCD	2.0934095	0	1	1	2.11324	2.0584	2.1294	2.07409	1.581006543	47782.348	49056.07	100975.76	101746.71	1.860124282	0.537822939	2	High	4318	Unique	Used	51843	1	43.408	1	5.5628	3.1939	1	49	22/24	28.1029	4318	1633.94	MS2	817.474	199	Glioma_grade2_2.raw
4506661			nFGIGQDIQPk	Unambiguous	N-Term(iTRAQ4plex); K11(iTRAQ4plex)	HCD	2.2084894	0	1	1	2.26834	2.1082	2.3164	2.1528668	4.393933321	36127.027	38870.496	81948.234	83683.01	5.173305614	1.481208308	2	High	2821	Unique	Used	58471	1	32.533	1	4.5729	-0.3365	1	94	19/20	19.54926	2821	1504.84	MS2	752.922	276	Glioma_grade2_3.raw
4506661			aGVNTVTTLVENk	Unambiguous	N-Term(iTRAQ4plex); K13(iTRAQ4plex)	HCD	1.7001557	0	1	1	1.48576	1.6087	1.7846	1.932295	11.51377356	22772.594	21032.096	33834.582	40640.215	5.619114932	12.92332289	3	High	4395	Unique	Used	51915	1	43.987	1	4.6616	-0.6992	1	22	19/48	12.85503	4395	1633.94	MS2	545.317	203	Glioma_grade2_2.raw
4506675	NP_002941.1	RPN1	dolichyl-diphosphooligosaccharide--protein glycosyltransferase subunit 1 precursor [Homo sapiens]	12	25	2.3173666	25.27592396	160.50302	37.07	4.86E-04	607	68.527	6.3794	Ubiquitin proteasome system protein	Protein metabolism	Ubiquitin-specific protease activity	Cytosol, Nucleus, Endoplasmic reticulum	Mitochondrion, Cytoplasm, Nucleus																								
4506675			fPLFGGWk	Unambiguous	N-Term(iTRAQ4plex); K8(iTRAQ4plex)	HCD	2.6909814	0	1	1	2.63251	2.8362	2.5562	2.7539737	4.623673534	331687.47	307869.88	873171.3	847865.56	5.266636182	2.079427905	2	High	6244	Unique	Used	76530	1	61.071	1	3.0123	-0.6401	1	10	14-Dec	11.32327	6244	1239.71	MS2	620.36	199	Glioma_grade2_5.raw
4506675			lAHLGVQVk	Unambiguous	N-Term(iTRAQ4plex); K9(iTRAQ4plex)	HCD	2.536238	0	1	1	2.45846	2.5938	2.4817	2.6182952	3.140179177	294991.8	279601.47	725226.1	732079.2	3.787933927	0.66504575	2	High	1437	Unique	Used	78921	1	22.505	1	3.781	-8.0683	1	11	16/16	16.67281	1437	1252.79	MS2	626.898	95	Glioma_grade2_6.raw
4506675			tVDLSSHLAk	Unambiguous	N-Term(iTRAQ4plex); K10(iTRAQ4plex)	HCD	2.6613624	0	1	1	2.49968	2.6636	2.6593	2.83364	5.119331054	145264.98	136326.75	363116.66	386300.94	4.488969221	4.375067147	2	High	1357	Unique	Used	72127	1	22.002	1	4.4465	-1.5889	1	20	18/18	5.00254	1357	1358.79	MS2	679.897	201	Glioma_grade2_5.raw
4506675			lAHLGVQVk	Unambiguous	N-Term(iTRAQ4plex); K9(iTRAQ4plex)	HCD	3.50666	0	1	1	3.23214	3.5218	3.4927	3.8057795	6.677346635	61448.336	56394.094	198609.84	214623.48	6.06553818	5.480368057	3	High	1415	Unique	Used	78901	1	22.332	1	4.6477	-9.3701	1	47	18/32	18.56321	1415	1252.79	MS2	418.267	125	Glioma_grade2_6.raw
4506675			aTSFLLALEPELEAR	Unambiguous	N-Term(iTRAQ4plex)	HCD	1.5754752	0	1	1	1.48459	1.6872	1.4772	1.6787556	7.380005446	59654.43	52492.312	88562.65	88121.766	9.031706343	0.352891413	2	High	5669	Unique	Used	87897	1	72.862	1	5.9397	0.7431	1	39	20/28	18.21207	5669	1804	MS2	902.503	209	Glioma_grade2_1rep.raw
4506675			vTAEVVLAHLGGGSTSR	Unambiguous	N-Term(iTRAQ4plex)	HCD	2.3173666	0	1	1	2.00651	2.6057	2.0953	2.7210565	15.21163083	50127.676	38600.64	100581.52	105034.52	18.37259105	3.062744032	3	High	4953	Unique	Used	60451	1	49.03	1	5.1412	1.0453	1	7	31/64	5.23548	4953	1798	MS2	600.004	141	Glioma_grade2_3.raw
4506675			fPLFGGWk	Unambiguous	N-Term(iTRAQ4plex); K8(iTRAQ4plex)	HCD	2.4430032	0	1	1	2.3282	2.4751	2.4128	2.5650482	4.089433707	46559.69	43795.754	108400.43	112338.21	4.326021326	2.522830566	2	High	6166	Unique	Used	76464	1	60.37	1	2.9809	-1.034	1	93	13/14	72.75671	6166	1239.71	MS2	620.359	243	Glioma_grade2_5.raw
4506675			tVDLSSHLAk	Unambiguous	N-Term(iTRAQ4plex); K10(iTRAQ4plex)	HCD	3.6126885	0	1	1	3.7386	3.4039	3.842	3.4980454	5.636165607	32785.195	36008.992	122570.89	125961.08	6.627213197	1.929109031	3	High	1347	Unique	Used	72118	1	21.924	1	4.3771	1.815	1	42	24/36	0	1347	1358.79	MS2	453.602	186	Glioma_grade2_5.raw
4506675			vTAEVVLAHLGGGSTSR	Unambiguous	N-Term(iTRAQ4plex)	HCD	1.9183738	0	1	1	2.12515	1.7643	2.104	1.7467067	10.72917693	31200.111	37581.777	66305.03	65644.34	13.12124292	0.708117635	3	High	4981	Unique	Used	52466	1	48.272	1	5.2232	0.8416	1	11	29/64	5.748227	4981	1798	MS2	600.003	177	Glioma_grade2_2.raw
4506675			vTAEVVLAHLGGGSTSR	Unambiguous	N-Term(iTRAQ4plex)	HCD	1.8759335	0	1	1	1.94108	1.6712	2.1138	1.8198472	9.938374266	25229.434	29304.408	48972.312	53329.547	10.5675362	6.023410417	3	High	5141	Unique	Used	68295	1	49.883	1	5.4243	0.638	1	15	30/64	6.250648	5141	1798	MS2	600.003	154	Glioma_grade2_4.raw
4506675			qFVVFEGNHYFYSPYPTk	Unambiguous	N-Term(iTRAQ4plex); K18(iTRAQ4plex)	HCD	3.5893493	0	1	1	3.17808	3.6879	3.5044	4.0665965	10.27080134	24713.926	21297.363	78542.805	86607.78	10.50122663	6.906180214	3	High	5538	Unique	Used	75903	1	55.223	1	5.9579	-0.0144	1	16	28/68	23.31114	5538	2511.26	MS2	837.758	128	Glioma_grade2_5.raw
4506675			vTAEVVLAHLGGGSTSR	Unambiguous	N-Term(iTRAQ4plex)	HCD	2.133247	0	1	1	1.95525	2.1869	2.0852	2.3323402	7.453930342	21459.723	19186.207	41959.04	44748.766	7.910354521	4.550072856	2	High	4994	Unique	Used	52479	1	48.353	1	5.8361	4.2437	1	100	20/32	15.3099	4994	1798	MS2	899.504	253	Glioma_grade2_2.raw
4506675			fVDHVFDEQVIDSLTVk	Unambiguous	N-Term(iTRAQ4plex); K17(iTRAQ4plex)	HCD	2.089875	0	1	1	2.07744	2.4056	1.8172	2.104273	11.45949457	20811.701	17972.352	43235.09	37818.734	10.35334256	9.450367343	3	High	6450	Unique	Used	76713	1	62.789	1	5.5816	-3.6001	1	7	27/64	7.397533	6450	2279.21	MS2	760.408	157	Glioma_grade2_5.raw
4506675			vTAEVVLAHLGGGSTSR	Unambiguous	N-Term(iTRAQ4plex)	HCD	1.7671227	0	1	1	1.73098	1.9387	1.6139	1.8076048	7.688395317	19931.584	17795.863	34501.145	32167.889	8.005751366	4.949407666	3	High	4866	Unique	Used	60372	1	48.314	1	5.7064	-0.7878	1	29	30/64	31.87798	4866	1797.99	MS2	600.002	182	Glioma_grade2_3.raw
4506675			vTAEVVLAHLGGGSTSR	Unambiguous	N-Term(iTRAQ4plex)	HCD	2.428142	0	1	1	2.51132	2.2571	2.6184	2.3533845	6.611522102	16892.63	18794.945	42422.773	44231.734	7.538421069	2.95224942	2	High	4930	Unique	Used	60431	1	48.82	1	6.0516	-0.0335	1	83	22/32	0	4930	1797.99	MS2	899.501	202	Glioma_grade2_3.raw

4506675			fVDHVFDEQVIDSLTVk	Unambiguous	N-Term(iTRAQ4plex); K17(iTRAQ4plex)	HCD	2.8424168	0	1	1	2.58544	2.8738	2.8142	3.1280487	7.822481452	16319.3125	14682.073	42192.613	45926.24	7.468718796	5.992072934	2	High	6446	Unique	Used	76710	1	62.746	1	6.3808	-5.6294	1	84	24/32	29.11727	6446	2279.2	MS2	1140.11	156	Glioma_grade 2_5.raw
4506675			nLVEQHIQDIVVHYTFNk	Unambiguous	N-Term(iTRAQ4plex); K18(iTRAQ4plex)	HCD	1.8095353	0	1	1	1.44183	2.1594	1.5759	2.3602395	23.59578313	15575.021	10399.376	22456.572	24545.018	28.17954678	6.283848392	4	High	5562	Unique	Used	92628	1	61.57	1	4.5843	0.3606	1	10	29/102	7.971156	5562	2485.35	MS2	622.092	97	Glioma_grade 2_7rep.raw
4506675			vTAEVVLAHLGGGSTSR	Unambiguous	N-Term(iTRAQ4plex)	HCD	1.9364178	0	1	1	1.89081	1.9559	1.9175	1.9835948	2.11654626	14588.006	14102.199	27583.178	27973.049	2.394666919	0.992437546	2	High	5062	Unique	Used	68225	1	49.208	1	5.9381	1.3922	1	100	22/32	40.90273	5062	1798	MS2	899.502	195	Glioma_grade 2_4.raw
4506675			vTAEVVLAHLGGGSTSR	Unambiguous	N-Term(iTRAQ4plex)	HCD	1.733516	0	1	1	2.07781	1.5949	1.9141	1.4692355	15.91592183	14499.037	18888.918	30126.283	27752.271	18.59421833	5.800697729	3	High	5053	Unique	Used	68216	1	49.149	1	5.4952	-0.1768	1	20	27/64	13.73645	5053	1797.99	MS2	600.003	143	Glioma_grade 2_4.raw
4506675			vHYENNSPFLTITSMTR	Unambiguous	N-Term(iTRAQ4plex)	HCD	1.9515975	0	1	1	2.05593	1.7992	2.1258	1.860298	7.929479156	13639.044	15585.388	28040.861	28993.465	9.418646979	2.362060869	3	High	5546	Unique	Used	68673	1	52.956	1	5.329	-0.5118	1	14	24/64	15.13869	5546	2154.08	MS2	718.697	134	Glioma_grade 2_4.raw
4506675			aPDELHYTYLDTFGRPVIVAYk	Unambiguous	N-Term(iTRAQ4plex); K22(iTRAQ4plex)	HCD	2.2925532	0	1	1	2.01397	2.6919	1.9938	2.664916	16.64519188	6109.154	4570.6084	12303.635	12180.289	20.37341256	0.712457636	4	High	5046	Unique	Used	92248	1	56.124	1	4.3351	-0.5235	1	25	26/126	45.67255	5046	2856.52	MS2	714.884	83	Glioma_grade 2_7rep.raw
4506675			iSVIVETVYTHVLHPYPTQITQSEk	Unambiguous	N-Term(iTRAQ4plex); K25(iTRAQ4plex)	HCD	2.829444	0	1	1	3.01358	2.6918	2.9836	2.6649694	6.538304954	5045.0435	5648.1484	15203.6455	15052.144	7.976281891	0.708147034	3	High	6916	Unique	Used	83630	1	70.292	1	9.0174	-1.0184	1	54	31/96	19.24446	6916	3170.73	MS2	1057.58	54	Glioma_grade 2_6.raw
4506675			vAcITEQVLTLVNk	Unambiguous	N-Term(iTRAQ4plex); C3(Methylthio); K14(iTRAQ4plex)	HCD	1.7300241	0	1	1	1.39036	1.8561	1.6356	2.1834588	19.07253107	4829.5537	3617.7827	6714.8164	7899.2793	20.28690349	11.46210844	3	High	8271	Unique	Used	55517	1	73.41	1	5.5885	-1.4193	1	58	22/52	6.598087	8271	1865.05	MS2	622.353	221	Glioma_grade 2_2.raw
4506675			aPDELHYTYLDTFGRPVIVAYk	Unambiguous	N-Term(iTRAQ4plex); K22(iTRAQ4plex)	HCD	2.8446207	0	1	1	2.44171	3.5455	2.362	3.429669	21.37635102	3488.0642	2402.1782	8516.83	8238.676	26.07150273	2.347700865	4	High	5335	Unique	Used	82360	1	54.976	1	5.2328	-0.1816	1	21	28/126	8.477724	5335	2856.52	MS2	714.885	74	Glioma_grade 2_6.raw
4506675			iSVIVETVYTHVLHPYPTQITQSEk	Unambiguous	N-Term(iTRAQ4plex); K25(iTRAQ4plex)	HCD	3.197457	0	1	1	2.86355	2.4858	4.0173	3.4873085	21.05333088	1293.3613	1489.9269	3703.6099	5195.8345	9.98767348	23.71298895	4	High	6915	Unique	Used	83629	1	70.289	1	6.7877	0.3825	1	28	26/144	16.16664	6915	3170.74	MS2	793.439	67	Glioma_grade 2_6.raw
4506681	NP_001006.1	RPS11	40S ribosomal protein S11 [Homo sapiens]	5	6	2.430689812	19.73216477	24.66854	39.87	7.24E-05	158	18.419	10.301	Ribosomal subunit	Protein metabolism	Structural constituent of ribosome	Ribosome	Nucleolus																								
4506681			nIGLGFk	Unambiguous	N-Term(iTRAQ4plex); K7(iTRAQ4plex)	HCD	2.4714723	0	1	1	2.25923	2.473	2.4701	2.7037969	7.332455938	1084497.9	990768.3	2450126.5	2678836.2	6.387309325	6.306233414	2	High	2376	Unique	Used	58056	1	29.23	1	3.0266	-0.0362	1	5	12-Dec	10.47943	2376	1036.64	MS2	518.823	168	Glioma_grade 2_3.raw
4506681			nIGLGFk	Unambiguous	N-Term(iTRAQ4plex); K7(iTRAQ4plex)	HCD	2.723341	0	1	1	2.80764	2.3892	3.116	2.6516066	11.08186722	662721.3	778788.75	1860682.1	2065041.4	11.38695925	7.361896314	2	High	2696	Unique	Used	66030	1	30.862	1	3.0262	-3.8044	1	5	12-Dec	19.73058	2696	1036.64	MS2	518.821	254	Glioma_grade 2_4.raw
4506681			qPTIFQNk	Unambiguous	N-Term(iTRAQ4plex); K8(iTRAQ4plex)	HCD	2.7887688	0	1	1	2.67664	2.9353	2.6551	2.9117332	5.343363854	296637.75	270494.1	793992.94	787606.6	6.519243171	0.571045224	2	High	1572	Unique	Used	57306	1	23.308	1	3.2994	-0.2417	1	9	13/14	3.876981	1572	1263.73	MS2	632.368	237	Glioma_grade 2_3.raw
4506681			iLSGVVTk	Unambiguous	N-Term(iTRAQ4plex); K8(iTRAQ4plex)	HCD	1.5627422	0	1	1	1.51689	1.5668	1.5588	1.6100972	2.443127535	283660.6	274629.16	430283.3	442179.66	2.287769873	1.928333285	2	High	2466	Unique	Used	50100	1	30.013	1	2.9899	-0.3562	1	24	13/14	54.05079	2466	1104.72	MS2	552.865	81	Glioma_grade 2_2.raw
4506681			dVQIGDIVTVGEcRPLSk	Unambiguous	N-Term(iTRAQ4plex); C13(Methylthio); K18(iTRAQ4plex)	HCD	2.3899074	0	1	1	2.43664	2.3164	2.4673	2.3454783	3.009397625	26857.697	28252.145	65442.63	66264.79	3.578393989	0.882797509	3	High	5837	Unique	Used	76172	1	57.651	1	4.6187	0.3403	1	15	21/68	32.17603	5837	2263.2	MS2	755.073	136	Glioma_grade 2_5.raw
4506681			nMSVHLSPcFR	Unambiguous	N-Term(iTRAQ4plex); C9(Methylthio)	HCD	2.0472488	0	1	1	1.77364	1.9207	2.1642	2.3435323	12.34710783	7221.915	6669.1406	12809.1	15629.348	5.627657652	14.02479126	3	High	3582	Unique	Used	74176	1	38.889	1	3.7684	1.1656	1	87	17/40	14.90684	3582	1480.7	MS2	494.238	70	Glioma_grade 2_5.raw
4506687	NP_001009.1	RPS15	40S ribosomal protein S15 [Homo sapiens]	3	11	2.700737476	20.12947999	52.982762	42.76	1.19E-04	145	17.029	10.389	Ribosomal subunit	Regulation of nucleobase, nucleoside, nucleotide and nucleic acid metabolism	Structural constituent of ribosome	Ribosome	-																								
4506687			eAPPmEkPEVVk	Unambiguous	N-Term(iTRAQ4plex); M5(Oxidation); K7(iTRAQ4plex); K12(iTRAQ4plex)	HCD	3.2883737	0	1	1	2.95002	3.2166	3.3542	3.6573029	8.934346205	293223.78	268920.72	865016.8	983524.44	6.114035992	9.066344213	3	High	1030	Unique	Used	78549	1	19.392	1	3.2458	-1.6717	1	2	17/44	7.083499	1030	1802.01	MS2	601.34	172	Glioma_grade 2_6.raw
4506687			eAPPMEkPEVVk	Unambiguous	N-Term(iTRAQ4plex); K7(iTRAQ4plex); K12(iTRAQ4plex)	HCD	2.7007375	0	1	1	2.52355	2.8215	2.5928	2.8988378	6.623259985	106689.016	95423.96	269235.53	276618.6	7.882321705	1.912825635	3	High	1348	Unique	Used	78837	1	21.87	1	3.6643	-9.3183	1	11	18/44	10.62203	1348	1786	MS2	596.004	194	Glioma_grade 2_6.raw
4506687			eAPPMEkPEVVk	Unambiguous	N-Term(iTRAQ4plex); K7(iTRAQ4plex); K12(iTRAQ4plex)	HCD	2.853433	0	1	1	2.67099	2.9114	2.8002	3.0522945	5.673226155	97579.16	89521.13	260633.27	273244.88	6.090730972	3.340745431	2	High	1371	Unique	Used	78859	1	22.024	1	5.0529	-7.9096	1	39	18/22	0	1371	1786	MS2	893.504	194	Glioma_grade 2_6.raw
4506687			eAPPmEkPEVVk	Unambiguous	N-Term(iTRAQ4plex); M5(Oxidation); K7(iTRAQ4plex); K12(iTRAQ4plex)	HCD	2.9410305	0	1	1	2.67972	3.1122	2.7936	3.2445095	8.952526564	68665.96	59124.03	184005.81	191828.48	10.55976827	2.943564838	2	High	1035	Unique	Used	78554	1	19.418	1	3.3786	-0.8175	1	53	13/22	13.36196	1035	1802.01	MS2	901.508	176	Glioma_grade 2_6.raw
4506687			kEAPPMEkPEVVk	Unambiguous	N-Term(iTRAQ4plex); K1(iTRAQ4plex); K8(iTRAQ4plex); K13(iTRAQ4plex)	HCD	2.441453	1	1	1	2.29298	2.4947	2.3925	2.6029868	5.449645901	25494.17	23432.527	58457.676	60994.56	5.959125938	3.003456343	3	High	1506	Unique	Used	85045	1	24.16	1	3.555	-4.3253	1	24	23/48	8.247334	1506	2058.2	MS2	686.739	58	Glioma_grade 2_1rep.raw
4506687			eAPPmEkPEVVk	Unambiguous	N-Term(iTRAQ4plex); M5(Oxidation); K7(iTRAQ4plex); K12(iTRAQ4plex)	HCD	3.7209325	0	1	1	3.58743	3.5991	3.8424	3.8548684	3.966750095	15082.651	15033.797	54107.957	57953.31	0.229409489	4.852836765	4	High	1027	Unique	Used	78547	1	19.352	1	5.2238	-3.0679	1	100	23/66	43.17554	1027	1802	MS2	451.256	206	Glioma_grade 2_6.raw
4506687			kEAPPMEkPEVVk	Unambiguous	N-Term(iTRAQ4plex); K1(iTRAQ4plex); K8(iTRAQ4plex); K13(iTRAQ4plex)	HCD	2.348625	1	1	1	2.16507	2.5182	2.2029	2.5621104	8.763168229	13663.721	11747.847	29582.959	30099.281	10.66228969	1.22346208	4	High	1655	Unique	Used	94587	1	25.591	1	3.4121	-0.3582	1	20	26/72	14.69731	1655	2058.21	MS2	515.308	57	Glioma_grade 2_8rep.raw
4506687			kEAPPmEkPEVVk	Unambiguous	K1(iTRAQ4plex); N-Term(iTRAQ4plex); M6(Oxidation); K8(iTRAQ4plex); K13(iTRAQ4plex)	HCD	2.0844455	1	1	1	1.78851	2.3394	1.8896	2.4715297	15.73784651	9263.391	7082.154	16567.643	17503.754	18.87202261	3.885549137	4	High	1113	Unique	Used	94165	1	19.871	1	3.5605	-1.1995	1	46	26/72	22.6399	1113	2074.2	MS2	519.306	58	Glioma_grade 2_8rep.raw
4506687			tFNQVEIkPEMIGHYLGEFSITYkPVk	Unambiguous	N-Term(iTRAQ4plex); K8(iTRAQ4plex); K24(iTRAQ4plex); K27(iTRAQ4plex)	HCD	3.0952988	0	1	1	2.65376	3.3912	2.8638	3.659529	14.76549018	2188.2954	1712.4473	5807.2134	6266.7505	17.25186428	5.382520642	4	High	5157	Unique	Used	87524	1	67.106	1	3.3088	9.1136	1	33	32/156	12.83224	5157	3745.09	MS2	937.027	21	Glioma_grade 2_1rep.raw
4506687			tFNQVEIkPEmIGHYLGEFSITYkPVk	Unambiguous	N-Term(iTRAQ4plex); K8(iTRAQ4plex); M11(Oxidation); K24(iTRAQ4plex); K27(iTRAQ4plex)	HCD	1.8609152	0	1	1	1.60365	1.5154	2.2265	2.1040256	19.06741231	1226.9224	1298.355	1967.5548	2731.7722	4.000390282	22.99832746	4	High	4791	Unique	Used	87250	1	63.16	1	4.5139	3.4812	1	65	23/156	27.91236	4791	3761.06	MS2	941.021	19	Glioma_grade 2_1rep.raw
4506687			tFNQVEIkPEMIGHYLGEFSITYkPVk	Unambiguous	N-Term(iTRAQ4plex); K8(iTRAQ4plex); K24(iTRAQ4plex); K27(iTRAQ4plex)	HCD	2.4371288	0	1	1	2.80384	2.9144	1.978	2.0559647	20.07069992	1083.9163	1042.804	3039.1233	2143.9683	2.733860783	24.4244254	4	High	5040	Unique	Used	96673	1	69.088	1	4.9836	2.9858	1	88	31/156	19.27116	5040	3745.06	MS2	937.021	26	Glioma_grade 2_8rep.raw
4506691	NP_001011.1	RPS16	40S ribosomal protein S16 [Homo sapiens]	3	5	2.110394716	15.53313805	26.620862	28.08	1.21E-03	146	16.435	10.213	Ribosomal subunit	Protein metabolism	Structural constituent of ribosome	Ribosome	Nucleolus																								
4506691			lLEPVLLLGk	Unambiguous	N-Term(iTRAQ4plex); K10(iTRAQ4plex)	HCD	2.7262223	0	1	1	2.70848	2.7334	2.7191	2.7441263	0.5751159	344248.53	341109.62	932390.9	936047.9	0.647703554	0.27679681	2	High	7134	Unique	Used	70112	1	65.986	1	3.9477	-0.54	1	7	16/18	25.22708	7134	1382.92	MS2	691.965	10	Glioma_grade 2_4.raw
4506691			lLEPVLLLGk	Unambiguous	N-Term(iTRAQ4plex); K10(iTRAQ4plex)	HCD	2.369312	0	1	1	2.19201	2.4646	2.2845	2.5686703	7.17062585	130653.39	116201.04	286393.03	298482.16	8.279660761	2.923121359	2	High	7261	Unique	Used	54609	1	65.023	1	4.1844	1.5785	1	23	16/18	44.59613	7261	1382.92	MS2	691.966	10	Glioma_grade 2_2.raw
4506691			gGGHVAQIYAIR	Unambiguous	N-Term(iTRAQ4plex)	HCD	1.9185691	0	1	1	1.89331	1.9034	1.9337	1.9439645	1.255391924	115080.04	114469.484	217882.02	222524.61	0.376152632	1.490807198	2	High	1435	Unique	Used	64865	1	21.349	1	3.4809	1.5718	1	25	18/22	22.7767	1435	1385.78	MS2	693.393	181	Glioma_grade 2_4.raw
4506691			eIkDILIQYDR	Unambiguous	N-Term(iTRAQ4plex); K3(iTRAQ4plex)	HCD	1.9198661	1	1	1	1.75435	1.7504	2.0898	2.085005	10.07585423	16851.902	16890.307	29564.146	35216.375	0.160964185	12.33929395	3	High	4843	Unique	Used	75280	1	49.605	1	4.6335	0.4407	1	33	22/40	31.008	4843	1693.97	MS2	565.329	144	Glioma_grade 2_5.raw
4506691			lLEPVLLLGk	Unambiguous	N-Term(iTRAQ4plex); K10(iTRAQ4plex)	HCD	2.1103947	0	1	1	1.60269	2.2405	2.0174	2.8201416	23.38727148	4579.731	3276.0542	7339.8716	9238.937	23.46904052	16.19949967	3	High	7119	Unique	Used	70099	1	65.852	1	3.6456	-1.6752	1	74	19/36	58.71531	7119	1382.92	MS2	461.645	9	Glioma_grade 2_4.raw
4506695	NP_001013.1	RPS19	40S ribosomal protein S19 [Homo sapiens]	4	15	2.490714788	16.10970144	59.361795	26.21	1.34E-04	145	16.051	10.316	Ribosomal subunit	Protein metabolism	Structural constituent of ribosome	Nucleolus	Mitochondrion																								
4506695			aLAAFLk	Unambiguous	N-Term(iTRAQ4plex); K7(iTRAQ4plex)	HCD	2.9389274	0	1	1	2.60142	2.9177	2.9579	3.3174632	9.938586058	1476640.4	1316584.2	3841365.2	4367719.5	8.103668025	9.067726999	2	High	4521	Unique	Used	67727	1	44.939	1	3.3315	0.5232	1	2	12-Dec	6.253683	4521	1021.67	MS2	511.336	153	Glioma_grade 2_4.raw
4506695			vLQALEGLk	Unambiguous	N-Term(iTRAQ4plex); K9(iTRAQ4plex)	HCD	1.6978192	0	1	1	1.5363	1.7601	1.6435	1.882865	8.755668892	1404485	1225924.1	2157709.5	2308249.5	9.600151037	4.76707712	2	High	6130	Unique	Used	69206	1	57.662	1	4.1379	0.9312	1	2	16/16	12.1203	6130	1258.8	MS2	629.903	110	Glioma_grade 2_4.raw
4506695			vLQALEGLk	Unambiguous	N-Term(iTRAQ4plex); K9(iTRAQ4plex)	HCD	2.3053484	0	1	1	2.12541	2.4146	2.2092	2.5097694	7.687324409	1056286.5	929785.56	2245040.5	2333547.2	9.007696579	2.733755116	2	High	6055	Unique	Used	61475	1	57.606	1	4.1995	0.9312	1	3	16/16	6.054983	6055	1258.8	MS2	629.903	108	Glioma_grade 2_3.raw
4506695			vPEWVDTVk	Unambiguous	N-Term(iTRAQ4plex); K9(iTRAQ4plex)	HCD	2.7902973	0	1	1	2.73772	2.8049	2.776	2.8441598	1.613269846	569871.2	556222.9	1560150.4	1581986.8	1.714031799	0.982813005	2	High	4504	Unique	Used	60036	1	45.494	1	3.2181	1.6255	1	4	13/16	10.36576	4504	1360.77	MS2	680.891	215	Glioma_grade 2_3.raw
4506695			aLAAFLk	Unambiguous	N-Term(iTRAQ4plex); K7(iTRAQ4plex)	HCD	2.9449837	0	1	1	2.79455	2.9328	2.9566	3.1028645	4.284927956	527581.6	502706.78	1474351.5	1559831.1	3.41441372	3.984150777	2	High	4124	Unique	Used	59684	1	42.503	1	3.4417	-1.1495	1	7	12-Dec	9.035557	4124	1021.66	MS2	511.335	154	Glioma_grade 2_3.raw
4506695			vLQALEGLk	Unambiguous	N-Term(iTRAQ4plex); K9(iTRAQ4plex)	HCD	2.4146814	0	1	1	2.0991	2.4425	2.3907	2.7818975	11.52495835	331643.06	285011.44	696151.5	792872.56	10.69433036	9.186166866	2	High	6224	Unique	Used	53638	1	57.328	1	4.1221	-0.9113	1	8	16/16	7.46304	6224	1258.8	MS2	629.902	138	Glioma_grade 2_2.raw
4506695			aLAAFLk	Unambiguous	N-Term(iTRAQ4plex); K7(iTRAQ4plex)	HCD	2.7285151	0	1	1	2.51129	2.8572	2.6154	2.9756687	7.802828156	299191.47	262967.25	751355.2	782503.4	9.112868197	2.871855781	2	High	4223	Unique	Used	59777	1	43.26	1	3.3953	-1.4482	1	13	12-Dec	10.34563	4223	1021.66	MS2	511.335	185	Glioma_grade 2_3.raw
4506695			aLAAFLk	Unambiguous	N-Term(iTRAQ4plex); K7(iTRAQ4plex)	HCD	2.4907148	0	1	1	2.4779	2.4427	2.5394	2.503346	1.638590511	207314.05	210299.94	513703.78	526453.5	1.011145755	1.733471205	2	High	4433	Unique	Used	67646	1	44.241	1	3.3105	2.7336	1	30	12-Dec	7.278488	4433	1021.67	MS2	511.337	155	Glioma_grade 2_4.raw
4506695			vLQALEGLk	Unambiguous	N-Term(iTRAQ4plex); K9(iTRAQ4plex)	HCD	3.1436145	0	1	1	3.0633	3.0329	3.2554	3.2231328	3.557742633	203178.7	205213.95	622397.2	661431.8	0.704782065	4.29989202	2	High	6133	Unique	Used	53552	1	56.655	1	4.1705	0.4463	1	24	16/16	26.22764	6133	1258.8	MS2	629.903	112	Glioma_grade 2_2.raw
4506695			aLAAFLk	Unambiguous	N-Term(iTRAQ4plex); K7(iTRAQ4plex)	HCD	2.2173772	0	1	1	2.03445	2.3459	2.1059	2.4283118	8.449498801	194237.86	168451.42	395166.3	409052.56	10.05475904	2.441889665	2	High	4236	Unique	Used	51765	1	42.825	1	3.4178	0.105	1	21	12-Dec	4.632766	4236	1021.67	MS2	511.336	163	Glioma_grade 2_2.raw
4506695			vLQALEGLk	Unambiguous	N-Term(iTRAQ4plex); K9(iTRAQ4plex)	HCD	2.4494464	0	1	1	2.41819	2.2903	2.6174	2.4790478	5.542773681	148482.39	156771.56	359059.03	388644.2	3.84029646	5.595769415	2	High	5969	Unique	Used	61395	1	56.925	1	3.8912	-0.0385	1	44	15/16	19.53388	5969	1258.8	MS2	629.902	113	Glioma_grade 2_3.raw
4506695			vLQALEGLk	Unambiguous	N-Term(iTRAQ4plex); K9(iTRAQ4plex)	HCD	3.1608365	0	1	1	3.34429	2.9822	3.3611	2.9972405	6.614745304	95805.68	107437.31	320402.44	322015.44	8.093567654	0.355084525	2	High	6042	Unique	Used	69125	1	56.958	1	4.2389	0.2524	1	41	16/16	9.983746	6042	1258.8	MS2	629.902	111	Glioma_grade 2_4.raw
4506695			eLAPYDENWFYTR	Unambiguous	N-Term(iTRAQ4plex)	HCD	2.086467	0	1	1	1.88988	2.1902	1.9969	2.3142915	9.078255174	62664.734	54071.89	118429	125138.13	10.40985777	3.895491055	2	High	4546	Unique	Used	87065	1	60.556	1	4.5121	1.4499	1	61	17/24	25.18239	4546	1847.87	MS2	924.441	101	Glioma_grade 2_1rep.raw
4506695			vLQALEGLk	Unambiguous	N-Term(iTRAQ4plex); K9(iTRAQ4plex)	HCD	2.843517	0	1	1	2.46926	3.3902	2.4454	3.3573587	18.15708215	4870.024	3547.1194	12025.338	11908.952	22.22689502	0.687693931	3	High	6118	Unique	Used	69196	1	57.544	1	3.6657	-2.4736	1	100	19/32	43.32537	6118	1258.79	MS2	420.27	129	Glioma_grade 2_4.raw
4506695			vLQALEGLk	Unambiguous	N-Term(iTRAQ4plex); K9(iTRAQ4plex)	HCD	2.323271	0	1	1	2.37523	2.0842	2.5958	2.2776828	9.119384355	2952.1543	3364.4468	7012.0347	7663.142	9.230749828	6.274573677	3	High	5985	Unique	Used	61410	1	57.051	1	3.2486	-2.9099	1	100	19/32	49.25714	5985	1258.79	MS2	420.269	128	Glioma_grade 2_3.raw
4506697	NP_001014.1	RPS20	40S ribosomal protein S20 isoform 2 [Homo sapiens]	2	11	2.800516844	22.47361608	63.975324	19.33	1.05E-04	119	13.364	9.9351	Ribosomal subunit	Protein metabolism	Structural constituent of ribosome	Ribosome, Nucleus	-																								

4506697			tPVEPEVAIHR	Unambiguous	N-Term(iTRAQ4plex)	HCD	2.3509703	0	1	2	2.29171	2.5205	2.1968	2.416152	6.009618281	185171.98	168362.38	424359.72	406789.1	6.724201941	2.989670262	2	High	1431	Unique	Used	49119	1	22.875	1	3.2722	-0.6798	1	8	15/20	8.909453	1431	1391.78	MS2	696.391	313	Glioma_grade2_2.raw
4506697			tPVEPEVAIHR	Unambiguous	N-Term(iTRAQ4plex)	HCD	3.9400432	0	1	2	3.61053	4.0064	3.8803	4.30568	7.303724051	178486.39	160852.1	644430.75	692577.56	7.349196397	5.092703701	2	High	1449	Unique	Used	57189	1	22.48	1	4.1046	-0.1535	1	20	14/20	12.2142	1449	1391.78	MS2	696.392	216	Glioma_grade2_3.raw
4506697			tPVEPEVAIHR	Unambiguous	N-Term(iTRAQ4plex)	HCD	3.116131	0	1	2	2.87742	3.1699	3.0673	3.3791122	6.695563256	127683.5	115901.33	367398.66	391643.62	6.840534613	4.517212302	3	High	1425	Unique	Used	49113	1	22.844	1	6.1373	-0.3824	1	3	24/40	4.151303	1425	1391.78	MS2	464.597	204	Glioma_grade2_2.raw
4506697			tPVEPEVAIHR	Unambiguous	N-Term(iTRAQ4plex)	HCD	3.7527354	0	1	2	3.4409	3.7084	3.7939	4.0888147	7.102700638	83260.875	77254.86	286492.28	315880.8	5.291560898	6.899651552	3	High	1445	Unique	Used	57185	1	22.458	1	6.1085	-0.1193	1	7	24/40	11.47448	1445	1391.78	MS2	464.597	203	Glioma_grade2_3.raw
4506697			tPVEPEVAIHR	Unambiguous	N-Term(iTRAQ4plex)	HCD	3.1102636	0	1	2	3.03002	3.1335	3.0878	3.193246	2.224273281	81626.78	78931.36	247330.88	252047.23	2.374155256	1.335646477	2	High	1412	Unique	Used	64843	1	21.194	1	3.0468	1.6884	1	17	14/20	14.85816	1412	1391.78	MS2	696.393	207	Glioma_grade2_4.raw
4506697			lIDLHSPSEIVk	Unambiguous	N-Term(iTRAQ4plex); K12(iTRAQ4plex)	HCD	2.7544234	0	1	2	2.58642	2.792	2.7196	2.9357808	5.281448049	75077.72	69549.79	194182.48	204182.92	5.405384894	3.550197351	2	High	3871	Unique	Used	74412	1	41.619	1	4.6874	1.737	1	32	18/22	43.37185	3871	1638.97	MS2	819.989	148	Glioma_grade2_5.raw
4506697			lIDLHSPSEIVk	Unambiguous	N-Term(iTRAQ4plex); K12(iTRAQ4plex)	HCD	2.2815423	0	1	2	2.31138	2.2521	2.3118	2.252468	1.500911293	71880.08	73773.27	166142.34	166171.95	1.838182901	0.012600982	2	High	3620	Unique	Used	80893	1	39.823	1	4.7828	1.8859	1	55	17/22	15.0628	3620	1638.97	MS2	819.989	150	Glioma_grade2_6.raw
4506697			lIDLHSPSEIVk	Unambiguous	N-Term(iTRAQ4plex); K12(iTRAQ4plex)	HCD	2.818471	0	1	2	2.28082	3.4131	2.4211	3.6230311	23.22888237	71272.58	47628.617	162559.62	172559.97	28.12218378	4.220174236	3	High	3854	Unique	Used	74397	1	41.481	1	4.4515	0.1275	1	4	24/44	10.42685	3854	1638.97	MS2	546.994	122	Glioma_grade2_5.raw
4506697			lIDLHSPSEIVk	Unambiguous	N-Term(iTRAQ4plex); K12(iTRAQ4plex)	HCD	2.8005168	0	1	2	2.72726	2.843	2.7598	2.8768826	2.494335445	53827.29	51636.695	146800.94	148552.7	2.937466433	0.838778472	2	High	3790	Unique	Used	74343	1	40.907	1	4.8884	0.6943	1	30	18/22	16.75069	3790	1638.97	MS2	819.988	151	Glioma_grade2_5.raw
4506697			lIDLHSPSEIVk	Unambiguous	N-Term(iTRAQ4plex); K12(iTRAQ4plex)	HCD	2.3358393	0	1	2	2.07731	2.3297	2.3413	2.625777	9.564739633	45172.902	40279.25	93838.164	105764.33	8.098882083	8.449867217	3	High	3769	Unique	Used	74325	1	40.727	1	4.5835	0.5743	1	8	23/44	13.02703	3769	1638.97	MS2	546.994	126	Glioma_grade2_5.raw
4506697			lIDLHSPSEIVk	Unambiguous	N-Term(iTRAQ4plex); K12(iTRAQ4plex)	HCD	1.8256766	0	1	2	1.6866	2.0511	1.6403	1.994817	11.37627827	25460.578	20935.797	42941.69	41763.082	13.79204013	1.96777983	2	High	3714	Unique	Used	74279	1	40.213	1	4.4524	1.0667	1	100	17/22	43.58419	3714	1638.97	MS2	819.988	160	Glioma_grade2_5.raw
4506707	NP_001019.1	RPS25	40S ribosomal protein S25 [Homo sapiens]	3	5	2.558540821	17.71723404	25.090611	16	1.23E-04	125	13.734	10.111	Ribosomal subunit	Protein metabolism	Structural constituent of ribosome	Ribosome	Nucleolus, Nucleus																								
4506707			aALQELLSk	Unambiguous	N-Term(iTRAQ4plex); K9(iTRAQ4plex)	HCD	1.8613895	0	1	1	1.66228	1.8453	1.8759	2.0824175	9.221011772	582560.56	524783.8	968380.3	1092819	7.37879566	8.537888463	2	High	4859	Unique	Used	60365	1	48.272	1	3.8584	0.6298	1	8	16/16	23.70017	4859	1260.78	MS2	630.892	187	Glioma_grade2_3.raw
4506707			lNNLVLFDk	Unambiguous	N-Term(iTRAQ4plex); K9(iTRAQ4plex)	HCD	2.834404	0	1	1	2.8325	2.7388	2.9332	2.8362424	2.799694159	396653.25	410217.03	1123521.4	1163475	2.377343931	2.47061705	2	High	5678	Unique	Used	61122	1	54.698	1	3.7854	1.4144	1	8	16/16	17.46385	5678	1363.82	MS2	682.414	197	Glioma_grade2_3.raw
4506707			lNNLVLFDk	Unambiguous	N-Term(iTRAQ4plex); K9(iTRAQ4plex)	HCD	2.6837695	0	1	1	2.63719	2.6586	2.7088	2.7307312	1.614969815	226341.61	224521.36	596904.75	613107.5	0.57095446	1.893712134	2	High	5862	Unique	Used	53294	1	54.728	1	3.666	1.7724	1	13	16/16	16.92793	5862	1363.82	MS2	682.414	191	Glioma_grade2_2.raw
4506707			dkLNNLVLFDk	Unambiguous	N-Term(iTRAQ4plex); K2(iTRAQ4plex); K11(iTRAQ4plex)	HCD	2.5585408	1	1	1	2.39061	2.6084	2.5129	2.7417738	5.79734485	82015.31	75167.49	196066.34	206092.25	6.161157529	3.525668293	2	High	4195	Unique	Used	91608	1	47.345	1	5.0536	-0.144	1	54	17/20	41.15937	4195	1751.04	MS2	876.025	111	Glioma_grade2_7rep.raw
4506707			dkLNNLVLFDk	Unambiguous	N-Term(iTRAQ4plex); K2(iTRAQ4plex); K11(iTRAQ4plex)	HCD	2.0321023	1	1	1	1.97525	2.1908	1.889	2.0951638	6.508956549	79848.92	71991.42	157721.31	150833.83	7.318333894	3.156767256	3	High	4193	Unique	Used	91606	1	47.333	1	4.7472	-1.9294	1	9	18/40	38.17225	4193	1751.04	MS2	584.351	121	Glioma_grade2_7rep.raw
4506723	NP_000997.1	RPS3A	40S ribosomal protein S3a [Homo sapiens]	4	10	2.206995487	23.82491185	67.410528	24.24	3.52E-04	264	29.926	9.73	Ribosomal subunit	Protein metabolism	Structural constituent of ribosome	Cytoplasm	Cytosol, Ribosome, Nucleolus, Nucleus																								
4506723			lIPDSIGk	Unambiguous	N-Term(iTRAQ4plex); K8(iTRAQ4plex)	HCD	1.6610006	0	1	1	1.56405	1.7111	1.6152	1.7670717	5.508505327	1363910.2	1246681.8	2133219	2202976	6.350513339	2.275065938	2	High	3162	Unique	Used	50751	1	35.068	1	2.9258	-0.4753	1	5	14-Oct	57.52566	3162	1130.7	MS2	565.855	142	Glioma_grade2_2.raw
4506723			kVVDPFSk	Unambiguous	N-Term(iTRAQ4plex); K1(iTRAQ4plex); K8(iTRAQ4plex)	HCD	2.2133327	1	1	1	2.06326	2.3041	2.1321	2.380917	6.643666225	186435.5	166949.17	384665.72	397492.12	7.798253435	2.319131499	2	High	1712	Unique	Used	89573	1	24.286	1	3.2842	-1.3546	1	30	14-Dec	24.03494	1712	1351.83	MS2	676.418	144	Glioma_grade2_7rep.raw
4506723			acQSIYPLHDVFVR	Unambiguous	N-Term(iTRAQ4plex); C2(Methylthio)	HCD	3.0213242	0	1	1	2.42956	2.69	3.3206	3.676519	18.85428289	70727.91	63880.504	171837.77	234857.88	7.193974095	21.91414987	3	High	5986	Unique	Used	69073	1	56.527	1	5.5007	0.4531	1	3	30/52	4.662883	5986	1837.92	MS2	613.312	170	Glioma_grade2_4.raw
4506723			acQSIYPLHDVFVR	Unambiguous	N-Term(iTRAQ4plex); C2(Methylthio)	HCD	2.200658	0	1	1	2.13135	2.4843	1.9573	2.2814448	10.11241921	59964.418	51445.21	127805.086	117369.41	10.81412775	6.019498265	3	High	5480	Unique	Used	75852	1	54.726	1	4.6718	-0.5431	1	5	27/52	31.82304	5480	1837.92	MS2	613.312	152	Glioma_grade2_5.raw
4506723			ncLTNFHGMDLTR	Unambiguous	N-Term(iTRAQ4plex); C2(Methylthio)	HCD	2.1616867	0	1	1	1.80952	2.1974	2.1323	2.5893295	14.66841186	45860.61	37766.203	82985.83	97789.14	13.68845679	11.58071926	3	High	5165	Unique	Used	68317	1	50.077	1	4.9407	0.9967	1	17	22/48	19.70529	5165	1711.79	MS2	571.267	60	Glioma_grade2_4.raw
4506723			acQSIYPLHDVFVR	Unambiguous	N-Term(iTRAQ4plex); C2(Methylthio)	HCD	3.433136	0	1	1	3.17781	3.6097	3.2777	3.7231643	7.562340108	41010.03	36103.312	130322.02	134418.56	8.998633651	2.188324293	2	High	5992	Unique	Used	69079	1	56.559	1	5.3862	1.6228	1	31	19/26	2.293376	5992	1837.92	MS2	919.466	179	Glioma_grade2_4.raw
4506723			acQSIYPLHDVFVR	Unambiguous	N-Term(iTRAQ4plex); C2(Methylthio)	HCD	2.9039447	0	1	1	2.71678	2.8302	2.9747	3.0989282	5.74224911	31446.03	30185.582	85431.836	93542.95	2.892253826	6.409197452	2	High	5482	Unique	Used	75854	1	54.737	1	5.3953	0.0287	1	53	19/26	32.09225	5482	1837.92	MS2	919.464	206	Glioma_grade2_5.raw
4506723			ncLTNFHGmDLTR	Unambiguous	N-Term(iTRAQ4plex); C2(Methylthio); M9(Oxidation)	HCD	2.1539671	0	1	1	2.10842	2.1764	2.1323	2.2009845	1.943149944	30521.45	29568.334	64351.97	65079.445	2.243159293	0.794864996	3	High	3506	Unique	Used	66784	1	37.044	1	4.669	0.292	1	28	18/48	13.43098	3506	1727.78	MS2	576.598	48	Glioma_grade2_4.raw
4506723			ncLTNFHGMDLTR	Unambiguous	N-Term(iTRAQ4plex); C2(Methylthio)	HCD	1.7214177	0	1	1	1.48298	1.7714	1.6796	2.00623	12.51626821	21600.03	18082.93	32032.43	36278.52	12.53417215	8.790505863	2	High	5176	Unique	Used	68328	1	50.149	1	5.3209	2.5378	1	100	17/24	44.36277	5176	1711.79	MS2	856.398	71	Glioma_grade2_4.raw
4506723			acQSIYPLHDVFVR	Unambiguous	N-Term(iTRAQ4plex); C2(Methylthio)	HCD	2.3723905	0	1	1	2.08667	2.5119	2.2565	2.7163475	11.60106132	10588.676	8796.022	22095.023	23893.053	13.07833426	5.529255913	3	High	5564	Unique	Used	75927	1	55.421	1	5.4951	-2.436	1	22	27/52	19.19754	5564	1837.92	MS2	613.31	170	Glioma_grade2_5.raw
4506725	NP_000998.1	RPS4X	40S ribosomal protein S4, X isoform X isoform [Homo sapiens]	6	15	2.188516855	25.78680862	68.831572	20.53	1.49E-04	263	29.579	10.155	Ribosomal subunit	Protein metabolism	Structural constituent of ribosome	Ribosome	Cytosol, Nucleolus																								
4506725			lSNIFVIGk	Unambiguous	N-Term(iTRAQ4plex); K9(iTRAQ4plex)	HCD	2.8518934	0	1	1	2.59476	2.8767	2.8295	3.1369648	7.774519054	1021504.7	921398.44	2650558	2890394.8	7.286602593	6.121338108	2	High	5663	Unique	Used	61108	1	54.584	1	3.9876	0.5715	1	2	15/16	5.635829	5663	1278.8	MS2	639.905	143	Glioma_grade2_3.raw
4506725			lSNIFVIGk	Unambiguous	N-Term(iTRAQ4plex); K9(iTRAQ4plex)	HCD	2.7654672	0	1	1	2.71482	2.7851	2.7463	2.8174307	1.619210455	500077.6	487456.12	1357618.2	1373373.9	1.807479363	0.815891215	2	High	5817	Unique	Used	68916	1	55.196	1	3.952	0.667	1	5	15/16	25.89381	5817	1278.8	MS2	639.905	152	Glioma_grade2_4.raw
4506725			lSNIFVIGk	Unambiguous	N-Term(iTRAQ4plex); K9(iTRAQ4plex)	HCD	2.1885169	0	1	1	2.0619	2.2027	2.1753	2.3237753	4.907407412	430507.8	402993.4	887665.3	936466.1	4.668408113	3.783431019	2	High	5815	Unique	Used	53250	1	54.378	1	3.8445	-1.3376	1	9	15/16	20.34836	5815	1278.8	MS2	639.904	179	Glioma_grade2_2.raw
4506725			hPGSFDVVHVk	Unambiguous	N-Term(iTRAQ4plex); K11(iTRAQ4plex)	HCD	3.0050552	0	1	2	2.89736	3.0644	2.949	3.1189606	3.391911395	371061.78	350836.84	1075099	1094246.2	3.962105433	1.248221349	2	High	1343	Unique	Used	89242	1	21.4	1	3.7883	-1.6547	1	7	15/20	4.7258	1343	1509.84	MS2	755.423	236	Glioma_grade2_7rep.raw
4506725			yALTGDEVk	Unambiguous	N-Term(iTRAQ4plex); K9(iTRAQ4plex)	HCD	1.7019612	0	1	3	1.64151	1.6193	1.7858	1.7615973	4.923683438	320367.88	324763.25	525886.06	572102.06	0.963523782	5.952641273	2	High	1723	Unique	Used	49396	1	24.864	1	3.5661	-0.7791	1	22	16-Dec	43.16437	1723	1283.71	MS2	642.357	238	Glioma_grade2_2.raw
4506725			hPGSFDVVHVk	Unambiguous	N-Term(iTRAQ4plex); K11(iTRAQ4plex)	HCD	2.0422556	0	1	2	1.97184	2.2731	1.842	2.1234357	9.09407255	296303.78	257030.27	584262.3	545787.25	10.0375407	4.815004574	3	High	1329	Unique	Used	89229	1	21.296	1	5.1284	-2.1487	1	6	20/40	32.21383	1329	1509.84	MS2	503.951	284	Glioma_grade2_7rep.raw
4506725			ecLPLIIFLR	Unambiguous	N-Term(iTRAQ4plex); C2(Methylthio)	HCD	2.8747885	0	1	1	2.776	2.8909	2.8593	2.9776654	2.897792431	207273.3	199034.66	575390.9	592658.6	2.867577692	2.090683274	2	High	8746	Unique	Used	55955	1	77.102	1	3.5126	-0.3654	1	22	16/18	10.08458	8746	1406.8	MS2	703.904	205	Glioma_grade2_2.raw
4506725			hPGSFDVVHVk	Unambiguous	N-Term(iTRAQ4plex); K11(iTRAQ4plex)	HCD	3.2308524	0	1	2	2.73227	3.2687	3.1992	3.82733	13.77816944	178887.16	149527.86	488768	572292.5	12.64263743	11.13239826	4	High	1328	Unique	Used	89228	1	21.291	1	6.1223	-2.7236	1	4	24/60	7.7956	1328	1509.84	MS2	378.215	253	Glioma_grade2_7rep.raw
4506725			ecLPLIIFLR	Unambiguous	N-Term(iTRAQ4plex); C2(Methylthio)	HCD	2.3928154	0	1	1	2.25575	2.48	2.3136	2.5435095	5.659166906	163611.1	148819.06	369065.03	378522.72	6.695609535	1.789113514	2	High	6101	Unique	Used	88246	1	77.054	1	3.992	-0.3654	1	15	16/18	14.87029	6101	1406.8	MS2	703.904	210	Glioma_grade2_1rep.raw
4506725			vNDTIQIDLETGk	Unambiguous	N-Term(iTRAQ4plex); K13(iTRAQ4plex)	HCD	1.5184909	0	1	1	1.42401	1.586	1.4579	1.6237229	6.360847231	66621.27	59817.188	94869.11	97126.53	7.61036254	1.662784624	2	High	4550	Unique	Used	52061	1	45.136	1	5.6648	1.2862	1	57	19/24	26.52826	4550	1733.95	MS2	867.481	219	Glioma_grade2_2.raw
4506725			lSNIFVIGk	Unambiguous	N-Term(iTRAQ4plex); K9(iTRAQ4plex)	HCD	1.9117988	0	1	1	1.82111	1.8935	1.9294	2.0060935	4.022830974	56796.96	54625.4	103433.47	109583.66	2.756223799	4.083093037	2	High	5577	Unique	Used	61030	1	53.879	1	3.8112	0.9534	1	76	15/16	10.28562	5577	1278.8	MS2	639.905	150	Glioma_grade2_3.raw
4506725			lSNIFVIGk	Unambiguous	N-Term(iTRAQ4plex); K9(iTRAQ4plex)	HCD	2.5324895	0	1	1	2.68005	2.442	2.6318	2.3980362	5.468967388	49211.805	54009.85	131890.19	129517.58	6.573679052	1.283579765	2	High	5729	Unique	Used	68835	1	54.494	1	3.9615	0.1897	1	69	15/16	27.78083	5729	1278.8	MS2	639.905	182	Glioma_grade2_4.raw
4506725			eRHPGSFDVVHVk	Unambiguous	N-Term(iTRAQ4plex); K13(iTRAQ4plex)	HCD	1.6273946	1	1	2	1.32998	1.6747	1.5898	2.0018892	16.80709796	6144.5015	4879.799	8172.07	9768.817	16.2237906	12.58656422	4	High	1365	Unique	Used	84939	1	22.66	1	3.2536	1.6293	1	75	20/72	22.67993	1365	1794.99	MS2	449.503	160	Glioma_grade2_1rep.raw
4506725			hPGSFDVVHVk	Unambiguous	N-Term(iTRAQ4plex); K11(iTRAQ4plex)	HCD	1.5740988	0	1	2	1.55999	1.4641	1.6913	1.5873445	5.929122241	4147.185	4418.6875	6469.551	7013.9795	4.48246828	5.710211939	4	High	1808	Unique	Used	94692	1	27.397	1	5.3013	-0.2981	1	100	21/60	26.23714	1808	1509.84	MS2	378.216	228	Glioma_grade2_8rep.raw
4506725			eRHPGSFDVVHVk	Unambiguous	N-Term(iTRAQ4plex); K13(iTRAQ4plex)	HCD	1.7236674	1	1	2	1.85537	1.5065	1.9911	1.6167281	12.66220058	1234.107	1519.865	2289.7234	2457.2085	14.67418112	4.989742952	5	High	1495	Unique	Used	94467	1	23.832	1	4.5311	-0.9624	1	100	24/96	31.65729	1495	1794.98	MS2	359.803	236	Glioma_grade2_8rep.raw
4506741	NP_001002.1	RPS7	40S ribosomal protein S7 [Homo sapiens]	4	9	2.153954029	14.20610683	61.450244	26.29	1.33E-04	194	22.113	10.096	Ribosomal subunit	Protein metabolism	Structural constituent of ribosome	Ribosome	-																								
4506741			eLNITAAk	Unambiguous	N-Term(iTRAQ4plex); K8(iTRAQ4plex)	HCD	1.8641225	0	1	1	1.73354	1.8608	1.8673	2.0042884	5.925686594	652384.3	607779.7	1130934.4	1218165.8	5.005731815	5.251535415	2	High	1530	Unique	Used	49213	1	23.539	1	3.1049	-1.9989	1	4	14/14	15.66385	1530	1147.69	MS2	574.349	363	Glioma_grade2_2.raw
4506741			vETFSGVYk	Unambiguous	N-Term(iTRAQ4plex); K9(iTRAQ4plex)	HCD	2.6523485	0	1	1	2.77204	2.4514	2.8796	2.5465038	7.422711339	266065.56	300869.16	737544.1	766164.44	8.681726732	2.691696689	2	High	2498	Unique	Used	58169	1	30.163	1	3.9715	-1.854	1	13	16/16	6.065121	2498	1317.73	MS2	659.367	228	Glioma_grade2_3.raw
4506741			aIIIFVPVPQLk	Unambiguous	N-Term(iTRAQ4plex); K12(iTRAQ4plex)	HCD	1.956381	0	1	1	1.95642	1.9453	1.9675	1.9563433	0.462691335	65341.3	65713.9	127834.95	128558.95	0.402071778	0.399340065	2	High	6887	Unique	Used	77088	1	66.667	1	3.8876	-0.7368	1	32	15/22	48.09407	6887	1626.06	MS2	813.533	14	Glioma_grade2_5.raw
4506741			aIIIFVPVPQLk	Unambiguous	N-Term(iTRAQ4plex); K12(iTRAQ4plex)	HCD	1.8400067	0	1	1	1.72904	1.7277	1.9524	1.9508866	7.005720156	64909.195	64959.348	112230.664	126728.33	0.054614498	8.580047788	2	High	7355	Unique	Used	70309	1	67.874	1	3.864	1.14	1	26	16/22	24.06112	7355	1626.06	MS2	813.534	15	Glioma_grade2_4.raw
4506741			tLTAVHDAILEDLVFPSEIVGk	Unambiguous	N-Term(iTRAQ4plex); K22(iTRAQ4plex)	HCD	2.6420243	0	1	1	2.15275	2.9029	2.4486	3.301799	18.70498128	45686.344	33880.258	98351.086	111865.81	20.98408945	9.0918981	2	High	7807	Unique	Used	77838	1	75.509	1	7.082	-2.4584	1	6	28/42	2.98545	7807	2655.48	MS2	1328.24	61	Glioma_grade2_5.raw
4506741			aIIIFVPVPQLk	Unambiguous	N-Term(iTRAQ4plex); K12(iTRAQ4plex)	HCD	1.961413	0	1	1	1.85983	2.1305	1.8138	2.077772	7.963936661	37430.36	32675.84	69614.27	67892.94	9.59102999	1.770327702	2	High	7277	Unique	Used	70240	1	67.194	1	3.89	-0.7368	1	60	16/22	34.34541	7277	1626.06	MS2	813.533	14	Glioma_grade2_4.raw
4506741			tLTAVHDAILEDLVFPSEIVGk	Unambiguous	N-Term(iTRAQ4plex); K22(iTRAQ4plex)	HCD	2.291713	0	1	1	2.89711	2.4201	2.1381	1.7860003	20.31677751	28079.309	33614.39	81348.945	60035.31	12.68814603	21.319228	3	High	7806	Unique	Used	77837	1	75.506	1	6.7171	-0.6936	1	1	33/84	2.599528	7806	2655.48	MS2	885.833	66	Glioma_grade2_5.raw
4506741			aIIIFVPVPQLk	Unambiguous	N-Term(iTRAQ4plex); K12(iTRAQ4plex)	HCD	2.2137306	0	1	1	1.80879	2.557	1.9709	2.786174	20.41697242	6077.1123	4298.9062	10992.209	11977.502	24.23630204	6.066313692	3	High	7345	Unique	Used	70300	1	67.795	1	3.537	-1.0453	1	16	17/44	7.465778	7345	1626.06	MS2	542.691	13	Glioma_grade2_4.raw
4506741			aIIIFVPVPQLk	Unambiguous	N-Term(iTRAQ4plex); K12(iTRAQ4plex)	HCD	2.153954	0	1	1	2.11861	2.1473	2.1605	2.1897807	1.371822749	3943.016	3890.2744	8353.7	8518.848	0.952190998	1.384228045	3	High	6878	Unique	Used	77080	1	66.591	1	3.4724	-1.721	1	72	19/44	11.78366	6878	1626.06	MS2	542.691	12	Glioma_grade2_5.raw
4506743	NP_001003.1	RPS8	40S ribosomal protein S8 [Homo sapiens]	3	7	2.358191252	9.974004594	40.588751	17.79	1.52E-04	208	24.19	10.316	Ribosomal subunit	Protein metabolism	Structural constituent of ribosome	Cytoplasm	Nucleolus																								
4506743			lTPEEEEILNk	Unambiguous	N-Term(iTRAQ4plex); K11(iTRAQ4plex)	HCD	2.8237834	0	1	1	2.91093	2.7261	2.9281	2.742167	3.802258818	404366.03	431788.9	1177083.1	1184037.2	4.638111106	0.416521875	2	High	4612	Unique	Used	52118	1	45.62	1	4.5123	-1.8697	1	6	18/20	5.271058	4612	1602.88	MS2	801.944	196	Glioma_grade2_2.raw
4506743			iSSLLEEQFQQGk	Unambiguous	N-Term(iTRAQ4plex); K13(iTRAQ4plex)	HCD	2.3581913	0	1	1	2.19849	2.525	2.2129	2.5416114	7.990979787	216554.69	188551.38	476093.28	479224.28	9.77587445	0.463500604	2	High	5701	Unique	Used	61144	1	54.858	1	6.0203	1.5748	1	9	21/24	10.06175	5701	1794.99	MS2	897.997	229	Glioma_grade2_3.raw
4506743			iSSLLEEQFQQGk	Unambiguous	N-Term(iTRAQ4plex); K13(iTRAQ4plex)	HCD	2.2898822	0	1	1	2.14305	2.3436	2.2408	2.4504504	5.768199376	194391.06	177759.12	416590.16	435589.94	6.32032883	3.153059612	2	High	5885	Unique	Used	53316	1	54.889	1	6.3502	-0.3294	1	14	21/24	7.485066	5885	1794.98	MS2	897.995	229	Glioma_grade2_2.raw
4506743			lTPEEEEILNk	Unambiguous	N-Term(iTRAQ4plex); K11(iTRAQ4plex)	HCD	2.1271386	0	1	1	2.03644	2.2292	2.0339	2.2264178	5.217312346	107679.2	98370.086	219282.48	219012.9	6.389284587	0.086983279	2	High	4521	Unique	Used	52034	1	44.917	1	4.3908	-0.4227	1	39	17/20	32.11197	4521	1602.88	MS2	801.945	188	Glioma_grade2_2.raw
4506743			iSSLLEEQFQQGk	Unambiguous	N-Term(iTRAQ4plex); K13(iTRAQ4plex)	HCD	2.5522292	0	1	1	2.08382	2.1591	2.9316	3.03757	19.62883328	48164.984	46484.664	100367.17	141200.42	2.510660508	23.90508426	3	High	5699	Unique	Used	61142	1	54.847	1	4.83	0.1732	1	3	26/48	8.731863	5699	1794.98	MS2	599	206	Glioma_grade2_3.raw
4506743			qWYESHYALPLGR	Unambiguous	N-Term(iTRAQ4plex)	HCD	2.3205585	0	1	1	2.38485	2.2706	2.3731	2.2593462	2.848699351	37166.855	39037.316	88637.43	88198.81	3.471242163	0.350777845	3	High	4450	Unique	Used	74939	1	46.216	1	4.5041	-0.1444	1	9	21/48	21.08222	4450	1763.9	MS2	588.638	150	Glioma_grade2_5.raw
4506743			qWYESHYALPLGR	Unambiguous	N-Term(iTRAQ4plex)	HCD	2.6805475	0	1	1	2.45632	2.7891	2.5849	2.9351592	7.89857948	33576.934	29570.348	82475.59	86793.68	8.972940846	3.607684633	2	High	4966	Unique	Used	68136	1	48.468	1	5.1723	2.147	1	100	20/24	0	4966	1763.9	MS2	882.455	173	Glioma_grade2_4.raw

4506787	NP_003861.1	IQGAP1	ras GTPase-activating-like protein IQGAP1 [Homo sapiens]	3	4	3.012497425	23.72038932	37.081304	5.01	2.41E-04	1657	189.13	6.481	GTPase activating protein	Cytoskeleton organization and biogenesis	GTPase activator activity	Cytoplasm, Nucleus	Mitochondrion, Nucleus, Golgi apparatus, Plasma membrane																								
4506787			lPYDVTPEQALAHEEVk	Unambiguous	N-Term(iTRAQ4plex); K17(iTRAQ4plex)	HCD	4.1180706	0	1	1	3.88193	4.1995	4.0428	4.3735323	5.108331796	22107.523	20435.514	85819.84	89375.38	5.558084168	2.870108494	3	High	5167	Unique	Used	68319	1	50.089	1	6.1906	1.6045	1	24	28/64	13.72004	5167	2227.19	MS2	743.068	171	Glioma_grade 2_4.raw
4506787			lPYDVTPEQALAHEEVk	Unambiguous	N-Term(iTRAQ4plex); K17(iTRAQ4plex)	HCD	3.2760458	0	1	1	2.80018	3.4695	3.1199	3.86565	13.83042587	20681.16	16691.639	57910.97	64524.03	15.09663407	7.638566701	3	High	4753	Unique	Used	75199	1	48.886	1	6.548	2.4266	1	25	27/64	10.45873	4753	2227.19	MS2	743.068	165	Glioma_grade 2_5.raw
4506787			iTLQDVVSHSk	Unambiguous	N-Term(iTRAQ4plex); K11(iTRAQ4plex)	HCD	2.4091318	0	1	1	2.2725	2.5798	2.2588	2.5642388	7.321066734	14936.1	13156.97	33942.293	33737.61	8.956193735	0.427697827	3	High	3139	Unique	Used	73768	1	35.496	1	4.3484	-2.8279	1	35	25/40	36.24202	3139	1514.87	MS2	505.629	188	Glioma_grade 2_5.raw
4506787			lEGVLAEVAQHYQDTLIR	Unambiguous	N-Term(iTRAQ4plex)	HCD	2.748949	0	1	1	2.62433	2.8202	2.6827	2.882864	4.343540282	7558.2104	7033.312	19835.268	20276.082	5.087326845	1.554186377	3	High	7956	Unique	Used	70820	1	73.426	1	7.02	0.9371	1	30	35/68	30.17479	7956	2199.19	MS2	733.735	115	Glioma_grade 2_4.raw
4506889	NP_003001.1	MAP2K4	dual specificity mitogen-activated protein kinase kinase 4 [Homo sapiens]	3	4	0.476647168	13.90522243	26.361253	11.03	1.21E-03	399	44.259	8.0747	Dual specificity kinase	Cell communication,Signal transduction	Protein threonine/tyrosine kinase activity	Cytoplasm, Plasma membrane	Cytosol, Nucleus																								
4506889			eFSPSFINFVNLcLTk	Unambiguous	N-Term(iTRAQ4plex); C13(Methylthio); K16(iTRAQ4plex)	HCD	0.53097564	0	1	1	0.51109	0.498	0.5648	0.5503555	0.059584029	31325.71	32148.906	16010.147	17693.326	1.834079544	7.062698167	2	High	8386	Unique	Used	71198	1	77.095	1	6.5073	-3.6044	1	81	21/30	7.008943	8386	2193.12	MS2	1097.07	191	Glioma_grade 2_4.raw
4506889			dIkPSNILLDR	Unambiguous	N-Term(iTRAQ4plex); K3(iTRAQ4plex)	HCD	0.39525896	0	1	1	0.36768	0.3506	0.4421	0.4215572	0.109661691	21561.807	22613.018	7927.8145	9532.68	3.365348596	12.99861557	3	High	2703	Unique	Used	73367	1	32.143	1	3.7631	11.48	1	48	19/40	24.30058	2703	1571.95	MS2	524.656	88	Glioma_grade 2_5.raw
4506889			fTLNPNPTGVQNPHIER	Unambiguous	N-Term(iTRAQ4plex)	HCD	0.4353273	0	1	1	0.33522	0.3657	0.4991	0.54454416	0.232573136	20367.922	18669.926	6827.6416	10166.599	6.15128419	27.78587729	2	High	3649	Unique	Used	66917	1	38.159	1	4.931	-1.4738	1	100	22/32	10.80664	3649	2078.09	MS2	1039.55	252	Glioma_grade 2_4.raw
4506889			fTLNPNPTGVQNPHIER	Unambiguous	N-Term(iTRAQ4plex)	HCD	0.51796705	0	1	1	0.47778	0.5367	0.5013	0.56311053	0.072611646	18371.178	16353.052	8777.432	9208.575	8.219220872	3.390014682	3	High	3616	Unique	Used	66886	1	37.91	1	5.5998	1.0163	1	38	24/64	13.91655	3616	2078.09	MS2	693.369	227	Glioma_grade 2_4.raw
4506931	NP_003017.1	SH3GL2	endophilin-A1 [Homo sapiens]	4	10	0.445738971	19.21365706	81.690231	17.61	6.41E-05	352	39.937	5.4526	Unclassified	Cell communication,Signal transduction	Molecular function unknown	Cytoplasm	Nucleus																								
4506931			lSMINTMSk	Unambiguous	N-Term(iTRAQ4plex); K9(iTRAQ4plex)	HCD	0.37483707	0	1	1	0.36145	0.3722	0.3774	0.38862145	0.030115268	522100.28	506993.22	188713.97	197028.44	2.076061032	3.048261206	2	High	3171	Unique	Used	58798	1	35.159	1	3.6928	-2.8801	1	24	14/16	44.71981	3171	1312.72	MS2	656.862	186	Glioma_grade 2_3.raw
4506931			aLYDFEPENEGELGFk	Unambiguous	N-Term(iTRAQ4plex); K16(iTRAQ4plex)	HCD	0.43572745	0	1	1	0.43122	0.41	0.4628	0.44001755	0.050162988	196883.27	207082.25	84899.04	91119.83	3.570486867	4.998058212	2	High	6479	Unique	Used	53877	1	59.198	1	5.4646	-1.7969	1	28	20/30	3.965802	6479	2146.05	MS2	1073.53	133	Glioma_grade 2_2.raw
4506931			qNFIDPLQNLHDk	Unambiguous	N-Term(iTRAQ4plex); K13(iTRAQ4plex)	HCD	0.48835117	0	1	1	0.46728	0.485	0.4916	0.51021916	0.036309629	108454.5	104495.97	50678.766	53315.844	2.628877416	3.586139198	3	High	4073	Unique	Used	81275	1	43.948	1	6.9418	-0.995	1	17	26/48	36.32349	4073	1870	MS2	624.006	169	Glioma_grade 2_6.raw
4506931			qNFIDPLQNLHDk	Unambiguous	N-Term(iTRAQ4plex); K13(iTRAQ4plex)	HCD	0.5805846	0	1	1	0.56238	0.5964	0.5656	0.5998965	0.034105598	76733.27	72351.03	43152.92	43403.13	4.156992548	0.408810679	2	High	4020	Unique	Used	81231	1	43.458	1	4.061	1.9497	1	94	13/24	36.60947	4020	1870.01	MS2	935.509	190	Glioma_grade 2_6.raw
4506931			gPGYPQAEALLAEAMLk	Unambiguous	N-Term(iTRAQ4plex); K17(iTRAQ4plex)	HCD	0.38649923	0	1	1	0.35262	0.3856	0.3873	0.42354584	0.074824276	73890.79	67567.61	26055.584	28617.977	6.321524147	6.62801337	2	High	7838	Unique	Used	55127	1	69.806	1	6.6019	-1.2582	1	80	21/32	54.98927	7838	2047.11	MS2	1024.06	197	Glioma_grade 2_2.raw
4506931			gPGYPQAEALLAEAMLk	Unambiguous	N-Term(iTRAQ4plex); K17(iTRAQ4plex)	HCD	0.39937398	0	1	1	0.38977	0.3894	0.4093	0.40896863	0.028262193	44515.37	44554.31	17350.797	18221.314	0.06182741	3.460848718	2	High	7510	Unique	Used	62811	1	69.277	1	7.1802	1.7233	1	100	21/32	47.67123	7510	2047.12	MS2	1024.06	189	Glioma_grade 2_3.raw
4506931			qNFIDPLQNLHDk	Unambiguous	N-Term(iTRAQ4plex); K13(iTRAQ4plex)	HCD	0.45575052	0	1	1	0.46872	0.4927	0.4206	0.44211525	0.068889806	33222.332	31602.506	15572.004	13971.949	3.533799649	7.65916288	3	High	4115	Unique	Used	74634	1	43.556	1	6.5527	1.2571	1	40	26/48	26.13752	4115	1870.01	MS2	624.008	209	Glioma_grade 2_5.raw
4506931			gPGYPQAEALLAEAMLk	Unambiguous	N-Term(iTRAQ4plex); K17(iTRAQ4plex)	HCD	0.43132222	0	1	1	0.43304	0.4413	0.4215	0.4295671	0.019040474	31635.742	31042.262	13699.682	13334.734	1.339078164	1.909101388	2	High	7920	Unique	Used	55203	1	70.455	1	6.3712	3.7507	1	96	21/32	28.84061	7920	2047.12	MS2	1024.06	176	Glioma_grade 2_2.raw
4506931			gPGYPQAEALLAEAMLk	Unambiguous	N-Term(iTRAQ4plex); K17(iTRAQ4plex)	HCD	0.51463145	0	1	1	0.50949	0.5861	0.4525	0.5205467	0.10592384	14389.466	12508.666	7331.28	6511.344	9.888615567	8.376768823	3	High	7817	Unique	Used	55107	1	69.653	1	6.5424	1.2099	1	46	24/64	44.17152	7817	2047.12	MS2	683.044	200	Glioma_grade 2_2.raw
4506931			gPGYPQAEALLAEAMLk	Unambiguous	N-Term(iTRAQ4plex); K17(iTRAQ4plex)	HCD	0.65932083	0	1	1	0.65786	0.712	0.6106	0.6609021	0.062721189	10101.083	9332.876	6645.097	6168.1177	5.590259597	5.264491471	3	High	7484	Unique	Used	62787	1	69.068	1	6.6502	-0.3107	1	49	24/64	25.56938	7484	2047.11	MS2	683.043	206	Glioma_grade 2_3.raw
4507171	NP_003109.1	SPARC	SPARC precursor [Homo sapiens]	5	8	2.446489096	24.42099875	65.815431	35.97	3.09E-04	303	34.61	4.8433	Secreted polypeptide	Cell growth and/or maintenance	Extracellular matrix structural constituent	Extracellular	Cytoplasm																								
4507171			fFETcDLDNDk	Unambiguous	N-Term(iTRAQ4plex); C5(Methylthio); K11(iTRAQ4plex)	HCD	2.2126188	0	1	1	2.01595	2.1188	2.3019	2.4193149	8.170954949	197641.44	188051.42	398435.62	454955.56	3.516356602	9.366310265	2	High	6076	Unique	Used	53498	1	56.243	1	4.5942	0.8767	1	40	16/20	58.0634	6076	1680.75	MS2	840.879	32	Glioma_grade 2_2.raw
4507171			lHLDYIGPck	Unambiguous	N-Term(iTRAQ4plex); C9(Methylthio); K10(iTRAQ4plex)	HCD	2.6803594	0	1	1	2.39526	2.8756	2.5177	3.022626	10.91235684	145068.92	120837.41	347478.2	365246.28	12.88744427	3.525606376	2	High	4259	Unique	Used	81438	1	45.527	1	3.8104	-0.5737	1	33	15/18	43.15639	4259	1492.79	MS2	746.898	202	Glioma_grade 2_6.raw
4507171			aPLIPmEHcTTR	Unambiguous	N-Term(iTRAQ4plex); M6(Oxidation); C9(Methylthio)	HCD	3.3540921	0	1	1	3.33062	3.3489	3.3593	3.3776963	0.586838299	52130.84	51846.555	173627.86	175121.9	0.38666068	0.60584748	3	High	1759	Unique	Used	72495	1	25.052	1	4.063	3.2558	1	14	15/44	63.52871	1759	1574.77	MS2	525.594	131	Glioma_grade 2_5.raw
4507171			aPLIPMEHcTTR	Unambiguous	N-Term(iTRAQ4plex); C9(Methylthio)	HCD	2.862859	0	1	1	2.67592	2.7936	2.9292	3.0580177	5.779759417	33704.477	32284.318	90190.6	98726.02	3.043559317	6.389541971	2	High	2852	Unique	Used	73506	1	33.244	1	3.3712	0.8188	1	58	14/22	0	2852	1558.77	MS2	779.887	160	Glioma_grade 2_5.raw
4507171			lEAGDHPVELLAR	Unambiguous	N-Term(iTRAQ4plex)	HCD	2.0337665	0	1	1	2.00262	2.0365	2.0311	2.0654442	1.265756667	30825.555	30312.887	61731.73	62609.58	1.18586934	0.998435175	2	High	3077	Unique	Used	50671	1	34.451	1	4.0177	3.5505	1	100	18/24	0	3077	1563.87	MS2	782.437	327	Glioma_grade 2_2.raw
4507171			lEAGDHPVELLAR	Unambiguous	N-Term(iTRAQ4plex)	HCD	1.7496194	0	1	1	1.83646	1.6652	1.8427	1.6708771	5.649620777	27333.889	30144.453	50197.543	50367.676	6.915192033	0.239252098	3	High	3384	Unique	Used	66674	1	36.031	1	3.411	-1.9221	1	23	21/48	25.63747	3384	1563.86	MS2	521.958	213	Glioma_grade 2_4.raw
4507171			yIPPcLDSELTEFPLR	Unambiguous	N-Term(iTRAQ4plex); C5(Methylthio)	HCD	3.025442	0	1	1	2.96113	2.8705	3.1852	3.0877817	4.576811748	27141.576	27998.19	80369.86	86452.3	2.197026256	5.156310852	2	High	5959	Unique	Used	88120	1	75.905	1	4.1989	1.3157	1	45	15/30	20.33119	5959	2083.04	MS2	1042.02	210	Glioma_grade 2_1rep.raw
4507171			lEAGDHPVELLAR	Unambiguous	N-Term(iTRAQ4plex)	HCD	1.7853619	0	1	1	1.72319	1.7513	1.8189	1.8485482	3.256220901	11717.406	11529.293	20191.318	21312.453	1.144385944	3.820193404	3	High	3284	Unique	Used	66581	1	35.261	1	3.8771	0.4196	1	100	22/48	65.49074	3284	1563.86	MS2	521.959	257	Glioma_grade 2_4.raw
4507677	NP_003290.1	HSP90B1	endoplasmin precursor [Homo sapiens]	14	58	2.431212902	26.2020789	336.17924	26.03	6.25E-05	803	92.411	4.8433	Heat shock protein	Protein metabolism	Heat shock protein activity	Endoplasmic reticulum	Mitochondrion, Cytosol																								
4507677			lIINSLYk	Unambiguous	N-Term(iTRAQ4plex); K8(iTRAQ4plex)	HCD	2.8370605	0	1	1	2.54665	3.1138	2.6107	3.192141	11.65901076	887781.44	726080.25	2260872.5	2317750.5	14.16974065	1.756808521	2	High	4643	Unique	Used	60165	1	46.579	1	3.3281	0.9209	1	3	13/14	7.486902	4643	1251.79	MS2	626.4	60	Glioma_grade 2_3.raw
4507677			lSLNIDPDAk	Unambiguous	N-Term(iTRAQ4plex); K10(iTRAQ4plex)	HCD	2.785452	0	1	1	2.73021	3.0079	2.5835	2.8463135	6.435663583	766143.5	695406.44	2091732.5	1979344.8	6.844604271	3.904131459	2	High	4040	Unique	Used	51580	1	41.394	1	4.0443	1.0605	1	4	16/18	3.638558	4040	1373.79	MS2	687.398	259	Glioma_grade 2_2.raw
4507677			lIINSLYk	Unambiguous	N-Term(iTRAQ4plex); K8(iTRAQ4plex)	HCD	2.7171283	0	1	1	2.53032	2.9263	2.5363	2.933175	8.380244563	731967.94	632923.94	1852109.5	1856476.8	10.26230503	0.166540412	2	High	4758	Unique	Used	52255	1	46.69	1	3.2282	-0.8343	1	4	14/14	10.60235	4758	1251.79	MS2	626.399	79	Glioma_grade 2_2.raw
4507677			gLFDEYGSk	Unambiguous	N-Term(iTRAQ4plex); K9(iTRAQ4plex)	HCD	2.168906	0	1	1	2.11786	2.2446	2.0975	2.223011	3.4011462	615658.8	580892.9	1303876.8	1291331.4	4.109008185	0.683639749	2	High	3051	Unique	Used	50646	1	34.274	1	3.7987	2.0802	1	5	16/16	25.79217	3051	1303.68	MS2	652.344	145	Glioma_grade 2_2.raw
4507677			eFEPLLNWMk	Unambiguous	N-Term(iTRAQ4plex); K10(iTRAQ4plex)	HCD	2.6784823	0	1	1	2.56858	2.7799	2.5848	2.7974236	4.576314947	311760.8	288063.62	800783.2	805836	5.587113867	0.444768635	2	High	7293	Unique	Used	62620	1	67.406	1	5.0287	0.8145	1	7	17/18	16.67983	7293	1594.86	MS2	797.931	242	Glioma_grade 2_3.raw
4507677			eFEPLLNWmk	Unambiguous	N-Term(iTRAQ4plex); M9(Oxidation); K10(iTRAQ4plex)	HCD	2.441069	0	1	1	2.45425	2.4273	2.455	2.4280322	0.636858951	303182.84	306544.6	744086.56	744300.1	0.779733086	0.020289833	2	High	6243	Unique	Used	61651	1	59.056	1	3.8979	-1.1141	1	10	14/18	25.94105	6243	1610.85	MS2	805.927	252	Glioma_grade 2_3.raw
4507677			gLFDEYGSk	Unambiguous	N-Term(iTRAQ4plex); K9(iTRAQ4plex)	HCD	2.4016814	0	1	1	2.35077	2.3818	2.4213	2.4532633	1.867466465	284168.47	280470.12	668015.06	688067	0.926301677	2.091151143	2	High	3031	Unique	Used	58667	1	34.117	1	3.9283	-2.7888	1	14	15/16	29.05039	3031	1303.67	MS2	652.34	133	Glioma_grade 2_3.raw
4507677			lIINSLYk	Unambiguous	N-Term(iTRAQ4plex); K8(iTRAQ4plex)	HCD	2.708596	0	1	1	2.77422	2.6498	2.7702	2.6459188	2.650151405	236540.55	247647.48	656214.56	655255.1	3.24410561	0.103462656	2	High	4556	Unique	Used	60084	1	45.9	1	3.3152	0.0433	1	14	13/14	25.50485	4556	1251.79	MS2	626.399	59	Glioma_grade 2_3.raw
4507677			nLLHVTDTGVGmTR	Unambiguous	N-Term(iTRAQ4plex); M12(Oxidation)	HCD	1.944408	0	1	1	1.90374	2.0012	1.8904	1.9871626	2.90964927	232994.62	221650.25	443560.06	440455.1	3.528767829	0.496719597	3	High	1827	Unique	Used	57545	1	25.159	1	4.1994	0.0027	1	1	18/52	6.040396	1827	1673.88	MS2	558.63	159	Glioma_grade 2_3.raw
4507677			nLLHVTDTGVGMTR	Unambiguous	N-Term(iTRAQ4plex)	HCD	2.4606335	0	1	1	2.30989	2.5268	2.4002	2.625534	5.631890912	191451.16	175018.7	442230.44	459517.56	6.341314889	2.711143197	3	High	3201	Unique	Used	66502	1	34.669	1	4.3109	0.6171	1	1	23/52	13.28764	3201	1657.88	MS2	553.299	203	Glioma_grade 2_4.raw
4507677			lSLNIDPDAk	Unambiguous	N-Term(iTRAQ4plex); K10(iTRAQ4plex)	HCD	1.8577203	0	1	1	1.7765	1.9402	1.7822	1.9464267	5.088263384	187291.47	171492.03	332722.72	333796.7	6.227650471	0.227875893	2	High	4145	Unique	Used	51679	1	42.165	1	3.7397	0.2608	1	15	16/18	18.75118	4145	1373.79	MS2	687.398	284	Glioma_grade 2_2.raw
4507677			gLFDEYGSk	Unambiguous	N-Term(iTRAQ4plex); K9(iTRAQ4plex)	HCD	2.0519924	0	1	1	1.90308	2.0064	2.0953	2.2089841	6.334841995	181086.53	171763.05	344622.78	379421.84	3.736830823	6.79699859	2	High	3130	Unique	Used	58759	1	34.86	1	4.0684	-2.0397	1	22	16/16	26.38763	3130	1303.67	MS2	652.341	198	Glioma_grade 2_3.raw
4507677			kTLDMIk	Unambiguous	N-Term(iTRAQ4plex); K1(iTRAQ4plex); K7(iTRAQ4plex)	HCD	2.0800853	1	1	1	2.02868	1.9682	2.1954	2.1299634	4.880415709	174494.55	179852.95	353992.84	383080.22	2.138556629	5.580961986	3	High	1374	Unique	Used	89271	1	21.618	1	3.2232	-3.6897	1	8	16/24	31.84868	1374	1280.79	MS2	427.602	129	Glioma_grade 2_7rep.raw
4507677			eFEPLLNWMk	Unambiguous	N-Term(iTRAQ4plex); K10(iTRAQ4plex)	HCD	1.767532	0	1	1	1.62436	1.8531	1.6925	1.9308641	7.958788437	173515.12	152097.8	281851.1	293680.2	9.30204625	2.906683555	2	High	7215	Unique	Used	62552	1	66.717	1	4.179	1.5799	1	34	16/18	54.36144	7215	1594.86	MS2	797.932	260	Glioma_grade 2_3.raw
4507677			eFEPLLNWMk	Unambiguous	N-Term(iTRAQ4plex); K10(iTRAQ4plex)	HCD	2.2572613	0	1	1	2.07922	2.3694	2.1588	2.4601512	7.839749561	166947.6	146500.98	347120.78	360414.6	9.225081609	2.657153419	2	High	7579	Unique	Used	54890	1	67.739	1	5.032	1.1207	1	28	16/18	41.17769	7579	1594.86	MS2	797.932	263	Glioma_grade 2_2.raw
4507677			nLLHVTDTGVGMTR	Unambiguous	N-Term(iTRAQ4plex)	HCD	2.5759935	0	1	1	2.44139	2.4998	2.6504	2.713814	4.932883895	164034.44	160201.94	400472.56	434758.25	1.671611766	5.80525613	2	High	2915	Unique	Used	58558	1	33.27	1	4.0501	-0.4425	1	8	18/26	1.90011	2915	1657.88	MS2	829.444	215	Glioma_grade 2_3.raw
4507677			nLLHVTDTGVGMTR	Unambiguous	N-Term(iTRAQ4plex)	HCD	1.7524757	0	1	1	1.37275	1.6617	1.8275	2.2121158	19.81222384	156482.12	129274.05	214811.58	285969.16	13.46533361	20.09502496	3	High	2881	Unique	Used	58526	1	33.015	1	4.275	0.5066	1	2	24/52	5.550142	2881	1657.88	MS2	553.299	202	Glioma_grade 2_3.raw
4507677			iADDkYNDTFWk	Unambiguous	N-Term(iTRAQ4plex); K5(iTRAQ4plex); K12(iTRAQ4plex)	HCD	1.9545898	1	1	1	1.97016	1.8053	2.1175	1.9403186	6.539582051	126647.07	138210.62	249515.52	268172.66	6.174383398	5.09673225	3	High	3487	Unique	Used	91078	1	39.957	1	3.9201	-1.8835	1	11	21/44	16.67666	3487	1948.01	MS2	650.01	155	Glioma_grade 2_7rep.raw
4507677			nLLHVTDTGVGMTR	Unambiguous	N-Term(iTRAQ4plex)	HCD	2.5924401	0	1	1	2.51719	2.5446	2.6398	2.6685076	2.814808796	111916.05	110710.36	281714.2	295431.44	0.765903358	3.361215177	2	High	3141	Unique	Used	66447	1	34.195	1	3.949	0.4411	1	16	18/26	4.465806	3141	1657.88	MS2	829.445	219	Glioma_grade 2_4.raw
4507677			eFEPLLNWMk	Unambiguous	N-Term(iTRAQ4plex); K10(iTRAQ4plex)	HCD	1.7243325	0	1	1	1.77696	1.7071	1.7423	1.6737708	2.580773143	108195.15	112625.34	192258.94	188509.02	2.837252459	1.39276102	2	High	7364	Unique	Used	70317	1	67.948	1	4.2762	1.0441	1	40	16/18	56.10872	7364	1594.86	MS2	797.932	261	Glioma_grade 2_4.raw
4507677			eFEPLLNWMk	Unambiguous	N-Term(iTRAQ4plex); K10(iTRAQ4plex)	HCD	2.707449	0	1	1	2.57961	2.8347	2.5916	2.847936	5.447407802	101956.375	92780.664	263007.2	264233.4	6.663557692	0.328902719	2	High	7663	Unique	Used	54965	1	68.435	1	4.9047	-0.104	1	27	17/18	15.47668	7663	1594.85	MS2	797.931	242	Glioma_grade 2_2.raw
4507677			nLLHVTDTGVGmTR	Unambiguous	N-Term(iTRAQ4plex); M12(Oxidation)	HCD	2.4213572	0	1	1	2.15063	2.4719	2.3774	2.7325222	9.899516365	99437.266	86514.06	213852.98	236401.6	9.828471562	7.082340887	2	High	1810	Unique	Used	57529	1	25.031	1	3.2096	0.0472	1	18	17/26	3.781152	1810	1673.88	MS2	837.442	198	Glioma_grade 2_3.raw
4507677			lIINSLYk	Unambiguous	N-Term(iTRAQ4plex); K8(iTRAQ4plex)	HCD	2.9871948	0	1	1	3.13321	2.7705	3.2323	2.858087	7.31938407	80375.81	90898.9	251833.94	259796.95	8.688904857	2.201078348	2	High	4794	Unique	Used	67978	1	47.106	1	3.4583	1.0185	1	90	13/14	49.86227	4794	1251.79	MS2	626.4	62	Glioma_grade 2_4.raw
4507677			fQSSHHPTDITSLDQYVER	Unambiguous	N-Term(iTRAQ4plex)	HCD	2.7733943	0	1	1	2.42396	2.7144	2.8261	3.1646972	11.00323445	66036.57	58971.055	160069.92	186625.53	7.993230123	10.83236132	3	High	3526	Unique	Used	80814	1	38.975	1	5.7793	2.5337	1	6	27/72	18.95782	3526	2404.17	MS2	802.062	106	Glioma_grade 2_6.raw
4507677			lIINSLYk	Unambiguous	N-Term(iTRAQ4plex); K8(iTRAQ4plex)	HCD	1.7539699	0	1	1	1.78848	1.7497	1.7584	1.7202046	1.601885795	64417.56	65846.3	115209.77	113269.1	1.551115931	1.201214727	2	High	4679	Unique	Used	52181	1	46.105	1	3.3278	-1.907	1	78	14-Dec	37.61361	4679	1251.79	MS2	626.398	76	Glioma_grade 2_2.raw
4507677			iADDkYNDTFWk	Unambiguous	N-Term(iTRAQ4plex); K5(iTRAQ4plex); K12(iTRAQ4plex)	HCD	2.1940618	1	1	1	2.08287	2.1251	2.2617	2.3075109	4.892175366	54154.734	53079.48	112797	122481.48	1.418053747	5.821154132	2	High	3476	Unique	Used	91069	1	39.849	1	5.447	-5.2918	1	67	18/22	0	3476	1948.01	MS2	974.507	188	Glioma_grade 2_7rep.raw
4507677			vFITDDFHDMMPk	Unambiguous	N-Term(iTRAQ4plex); K13(iTRAQ4plex)	HCD	2.043998	0	1	1	1.90643	2.0842	2.0072	2.1943913	5.943259578	53101.75	48571.926	101234.98	106585.81	6.300685475	3.641222014	2	High	5282	Unique	Used	82321	1	54.408	1	5.2671	-0.1623	1	71	18/24	19.72888	5282	1883.93	MS2	942.467	158	Glioma_grade 2_6.raw
4507677			vFITDDFHDmMPk	Unambiguous	N-Term(iTRAQ4plex); M10(Oxidation); K13(iTRAQ4plex)	HCD	2.277562	0	1	1	2.54255	2.2702	2.2858	2.040954	8.973320187	50832.31	56930.17	129243.86	116191.88	8.002485015	7.520622131	3	High	4217	Unique	Used	81401	1	45.173	1	4.2875	0.7095	1	21	22/48	31.97339	4217	1899.92	MS2	633.979	141	Glioma_grade 2_6.raw

4507677			kGYEVIYLTEPVDEYcIQALPEFDGk	Unambiguous	N-Term(iTRAQ4plex); K1(iTRAQ4plex); C16(Methylthio); K26(iTRAQ4plex)	HCD	1.8081143	1	1	1	2.06403	1.6567	1.9968	1.6027087	12.78785089	49394.934	61540.97	101952.54	98632.25	15.4837958	2.34095474	3	High	7428	Unique	Used	84010	1	75.846	1	5.1767	0.2376	1	4	26/100	40.90886	7428	3497.76	MS2	1166.59	96	Glioma_grade2_6.raw
4507677			vFITDDFHDMMPk	Unambiguous	N-Term(iTRAQ4plex); K13(iTRAQ4plex)	HCD	2.6284163	0	1	1	2.4319	2.6163	2.6396	2.8398368	6.340323617	47886.984	44511.133	116456.36	126404.35	5.166960566	5.792860597	3	High	5563	Unique	Used	75926	1	55.415	1	4.9682	-1.0765	1	16	23/48	29.12091	5563	1883.93	MS2	628.647	119	Glioma_grade2_5.raw
4507677			vFITDDFHDmMPk	Unambiguous	N-Term(iTRAQ4plex); M10(Oxidation); K13(iTRAQ4plex)	HCD	2.051093	0	1	1	2.08864	1.9589	2.1493	2.015874	4.053093297	45110.176	48096.797	94219.11	96957.08	4.531549291	2.025395687	3	High	4314	Unique	Used	74815	1	45.144	1	4.006	-1.0253	1	25	21/48	56.36316	4314	1899.92	MS2	633.978	99	Glioma_grade2_5.raw
4507677			vFITDDFHDMmPk	Unambiguous	N-Term(iTRAQ4plex); M11(Oxidation); K13(iTRAQ4plex)	HCD	2.1197448	0	1	1	1.87517	2.23	2.027	2.410601	10.95255346	41673.9	35042.914	78145.586	84474.484	12.22369627	5.503879925	3	High	4472	Unique	Used	74960	1	46.367	1	4.8509	-0.9289	1	25	23/48	43.35617	4472	1899.92	MS2	633.978	102	Glioma_grade2_5.raw
4507677			eFEPLLNWMk	Unambiguous	N-Term(iTRAQ4plex); K10(iTRAQ4plex)	HCD	2.4514177	0	1	1	2.4155	2.4573	2.4457	2.4879534	1.22182779	34536.082	33949.156	83422.016	84463.914	1.211996532	0.877659183	2	High	7371	Unique	Used	62684	1	68.15	1	5.1163	0.5849	1	86	17/18	51.02791	7371	1594.86	MS2	797.931	246	Glioma_grade2_3.raw
4507677			vFITDDFHDMMPk	Unambiguous	N-Term(iTRAQ4plex); K13(iTRAQ4plex)	HCD	2.32685	0	1	1	2.08554	2.3557	2.3013	2.599415	9.039291435	33373.9	29546.992	69602.57	76804.9	8.601380279	6.95704445	2	High	5566	Unique	Used	75929	1	55.434	1	5.6524	-1.7822	1	67	23/24	15.70264	5566	1883.92	MS2	942.466	143	Glioma_grade2_5.raw
4507677			lSLNIDPDAk	Unambiguous	N-Term(iTRAQ4plex); K10(iTRAQ4plex)	HCD	1.6230172	0	1	1	1.73289	1.5336	1.7241	1.5257831	7.051975216	31406.07	35487.844	54423.223	54146.75	8.629335322	0.360128915	2	High	3931	Unique	Used	59505	1	40.997	1	4.3094	-0.9832	1	100	16/18	65.07306	3931	1373.79	MS2	687.397	307	Glioma_grade2_3.raw
4507677			nLLHVTDTGVGMTR	Unambiguous	N-Term(iTRAQ4plex)	HCD	2.6777112	0	1	1	2.48064	2.6978	2.6592	2.8920298	6.290711493	30739.092	28264.852	76252.73	81742.79	5.930287922	4.914137635	2	High	2816	Unique	Used	58467	1	32.482	1	4.0047	1.5456	1	58	20/26	13.77126	2816	1657.88	MS2	829.446	218	Glioma_grade2_3.raw
4507677			vFITDDFHDmMPk	Unambiguous	N-Term(iTRAQ4plex); M10(Oxidation); K13(iTRAQ4plex)	HCD	2.1095006	0	1	1	2.1021	2.0744	2.1451	2.1168075	1.39885007	29987.402	30388.498	63036.4	64326.6	0.939506331	1.432612563	2	High	4229	Unique	Used	81412	1	45.266	1	3.8633	0.3953	1	100	15/24	0	4229	1899.92	MS2	950.465	130	Glioma_grade2_6.raw
4507677			ySQFINFPIYVWSSk	Unambiguous	N-Term(iTRAQ4plex); K15(iTRAQ4plex)	HCD	2.45931	0	1	1	2.80325	2.3212	2.6261	2.1745107	11.50231632	27891.646	33683.84	78187.34	73245.87	13.30302015	4.614769701	2	High	8594	Unique	Used	55812	1	76.028	1	4.9608	-0.9637	1	21	18/28	15.85963	8594	2167.14	MS2	1084.08	174	Glioma_grade2_2.raw
4507677			vFITDDFHDmMPk	Unambiguous	N-Term(iTRAQ4plex); M10(Oxidation); K13(iTRAQ4plex)	HCD	2.4708576	0	1	1	2.2629	2.547	2.4032	2.7049294	7.656449345	25714.227	22845.662	58188.67	61795.902	8.354144977	4.251710309	2	High	4328	Unique	Used	74828	1	45.248	1	5.094	0.9735	1	94	18/24	14.30328	4328	1899.92	MS2	950.466	123	Glioma_grade2_5.raw
4507677			vFITDDFHDMmPk	Unambiguous	N-Term(iTRAQ4plex); M11(Oxidation); K13(iTRAQ4plex)	HCD	1.9317862	0	1	1	1.91039	1.8433	2.0234	1.9524286	3.907630125	25162.896	26078.166	48071.02	50915.758	2.5260742	4.064246905	2	High	4447	Unique	Used	81591	1	47.331	1	3.8741	1.616	1	100	16/24	22.60047	4447	1899.93	MS2	950.466	133	Glioma_grade2_6.raw
4507677			vFITDDFHDmmPk	Unambiguous	N-Term(iTRAQ4plex); M10(Oxidation); M11(Oxidation); K13(iTRAQ4plex)	HCD	1.6071303	0	1	1	1.66463	1.5239	1.698	1.5544872	5.222701925	24860.012	27155.213	41382.812	42212.43	6.240296726	1.403497375	3	High	2967	Unique	Used	80305	1	34.594	1	4.0127	2.4656	1	52	Dec-48	61.76327	2967	1915.92	MS2	639.312	112	Glioma_grade2_6.raw
4507677			vFITDDFHDMmPk	Unambiguous	N-Term(iTRAQ4plex); M11(Oxidation); K13(iTRAQ4plex)	HCD	2.4935832	0	1	1	2.22277	2.6604	2.3542	2.8177114	10.87075738	20239.598	16910.215	44988.04	47648.105	12.67424574	4.060941871	2	High	4506	Unique	Used	74992	1	46.614	1	4.6103	-0.44	1	88	19/24	9.932618	4506	1899.92	MS2	950.464	122	Glioma_grade2_5.raw
4507677			tETVEEPMEEEEAAkEEk	Unambiguous	N-Term(iTRAQ4plex); K15(iTRAQ4plex); K18(iTRAQ4plex)	HCD	4.8696713	1	1	1	4.58658	4.3795	5.383	5.139988	9.604239378	17410.576	18233.674	79854.92	93720.86	3.265705842	11.29731372	3	High	2648	Unique	Used	65987	1	30.475	1	5.3333	13.508	1	9	31/68	6.780256	2648	2540.26	MS2	847.426	103	Glioma_grade2_4.raw
4507677			vFITDDFHDMmPk	Unambiguous	N-Term(iTRAQ4plex); M11(Oxidation); K13(iTRAQ4plex)	HCD	1.8371722	0	1	1	1.63632	1.8244	1.8486	2.0611112	9.434666929	15783.89	14156.649	25827.494	29178.43	7.686121788	8.615325029	3	High	4389	Unique	Used	81545	1	46.752	1	4.7032	-1.9891	1	70	23/48	27.28202	4389	1899.92	MS2	633.978	104	Glioma_grade2_6.raw
4507677			tETVEEPMEEEEAAkEEk	Unambiguous	N-Term(iTRAQ4plex); K15(iTRAQ4plex); K18(iTRAQ4plex)	HCD	5.051945	1	1	1	5.33064	4.7624	5.376	4.8029575	6.519211455	15356.844	17189.123	81861.82	82558.625	7.961766236	0.599336099	3	High	2325	Unique	Used	73010	1	29.439	1	4.7079	7.453	1	7	32/68	7.827706	2325	2540.25	MS2	847.421	96	Glioma_grade2_5.raw
4507677			eFEPLLNWmk	Unambiguous	N-Term(iTRAQ4plex); M9(Oxidation); K10(iTRAQ4plex)	HCD	2.4011555	0	1	1	2.33627	2.3751	2.4268	2.4671204	2.392564607	14955.103	14710.901	34939.125	36293.562	1.16413987	2.689022772	3	High	6229	Unique	Used	61638	1	58.939	1	4.1112	-1.274	1	36	16/36	26.14195	6229	1610.85	MS2	537.621	264	Glioma_grade2_3.raw
4507677			eFEPLLNWMk	Unambiguous	N-Term(iTRAQ4plex); K10(iTRAQ4plex)	HCD	2.6411645	0	1	1	2.29736	2.8852	2.4469	3.0729313	13.59922655	14540.93	11578.408	33405.82	35579.652	16.04037128	4.45639148	3	High	7303	Unique	Used	62627	1	67.523	1	3.7756	-0.4185	1	29	18/36	15.76853	7303	1594.85	MS2	532.289	254	Glioma_grade2_3.raw
4507677			kYSQFINFPIYVWSSk	Unambiguous	N-Term(iTRAQ4plex); K1(iTRAQ4plex); K16(iTRAQ4plex)	HCD	2.8779757	1	1	1	3.05247	2.5763	3.2354	2.7307036	10.3334016	14320.387	16967.098	43712.504	46332.113	11.96329648	4.114278787	2	High	6036	Unique	Used	92967	1	66.707	1	5.7963	0.3401	1	100	19/30	46.06417	6036	2439.34	MS2	1220.18	144	Glioma_grade2_7rep.raw
4507677			eFEPLLNWmk	Unambiguous	N-Term(iTRAQ4plex); M9(Oxidation); K10(iTRAQ4plex)	HCD	2.917118	0	1	1	2.62031	3.0365	2.8141	3.2610784	9.446070246	13483.399	11635.198	35330.625	37943.293	10.40564057	5.042545315	3	High	6411	Unique	Used	53813	1	58.706	1	4.2512	-4.4568	1	28	17/36	14.93909	6411	1610.84	MS2	537.619	258	Glioma_grade2_2.raw
4507677			eFEPLLNWMk	Unambiguous	N-Term(iTRAQ4plex); K10(iTRAQ4plex)	HCD	2.778396	0	1	1	2.63961	2.8228	2.7368	2.9268138	4.400715557	13275.246	12413.609	35041.484	36332.324	4.743452876	2.557693762	3	High	7593	Unique	Used	54903	1	67.844	1	3.9875	-1.9111	1	31	19/36	3.894143	7593	1594.85	MS2	532.289	252	Glioma_grade2_2.raw
4507677			fQSSHHPTDITSLDQYVER	Unambiguous	N-Term(iTRAQ4plex)	HCD	3.7471855	0	1	1	3.71754	3.2761	4.2818	3.7733085	10.94175329	9875.375	11206.076	36712.125	42283.984	8.926783084	9.97491986	2	High	3532	Unique	Used	80819	1	39.034	1	5.7812	0.4321	1	34	21/36	0	3532	2404.17	MS2	1202.59	149	Glioma_grade2_6.raw
4507677			eFEPLLNWMk	Unambiguous	N-Term(iTRAQ4plex); K10(iTRAQ4plex)	HCD	2.7104316	0	1	1	2.44946	2.869	2.5751	3.0161004	9.557366632	9757.232	8330.501	23899.938	25125.627	11.15508688	3.535677778	3	High	7226	Unique	Used	62562	1	66.806	1	3.9286	-0.5334	1	48	17/36	0	7226	1594.85	MS2	532.289	245	Glioma_grade2_3.raw
4507677			tETVEEPMEEEEAAkEEk	Unambiguous	N-Term(iTRAQ4plex); K15(iTRAQ4plex); K18(iTRAQ4plex)	HCD	2.6420481	1	1	1	2.81123	2.5954	2.6926	2.4858599	5.239912561	7933.511	8593.361	22302.9	21361.89	5.646372884	3.047739619	3	High	2439	Unique	Used	58114	1	29.721	1	5.1647	3.5606	1	28	30/68	11.95966	2439	2540.24	MS2	847.417	97	Glioma_grade2_3.raw
4507677			fQSSHHPTDITSLDQYVER	Unambiguous	N-Term(iTRAQ4plex)	HCD	2.222493	0	1	1	2.00768	2.2495	2.1984	2.4631824	8.397774257	7578.504	6763.7715	15215.203	16660.404	8.033632817	6.411871167	3	High	3777	Unique	Used	74332	1	40.791	1	5.3314	0.5535	1	28	24/72	28.6333	3777	2404.17	MS2	802.06	107	Glioma_grade2_5.raw
4507677			eEkEESDDEAAVEEEEEEk	Unambiguous	N-Term(iTRAQ4plex); K3(iTRAQ4plex); K19(iTRAQ4plex)	HCD	3.0439308	1	1	1	3.41027	2.8208	3.3136	2.7409086	11.0492993	4368.871	5281.769	14899.035	14476.846	13.37769032	2.032502139	3	High	1742	Unique	Used	72479	1	24.934	1	7.0539	7.5443	1	33	25/72	5.793573	1742	2685.23	MS2	895.749	56	Glioma_grade2_5.raw
4507677			eFEPLLNWMk	Unambiguous	N-Term(iTRAQ4plex); K10(iTRAQ4plex)	HCD	2.6550584	0	1	1	2.49957	2.5946	2.7133	2.8164573	5.202500714	3395.5615	3271.2388	8487.44	9213.304	2.637229863	5.799342182	3	High	7373	Unique	Used	70326	1	68.009	1	3.7454	-1.4518	1	100	18/36	32.86567	7373	1594.85	MS2	532.289	250	Glioma_grade2_4.raw
4507677			ySQFINFPIYVWSSk	Unambiguous	N-Term(iTRAQ4plex); K15(iTRAQ4plex)	HCD	1.794735	0	1	1	1.47096	1.5281	2.0514	2.1310732	19.15895352	2437.7095	2346.6199	3585.7844	5000.819	2.692543446	23.30561957	3	High	8599	Unique	Used	55817	1	76.057	1	3.2157	-0.9699	1	17	17/56	5.831816	8599	2167.14	MS2	723.053	96	Glioma_grade2_2.raw
4507677			eEkEESDDEAAVEEEEEEk	Unambiguous	N-Term(iTRAQ4plex); K3(iTRAQ4plex); K19(iTRAQ4plex)	HCD	1.9998527	1	1	1	2.87773	1.9945	2.0076	1.3914121	29.5815805	2044.3851	2949.7153	5883.196	4104.27	25.63685438	25.18938513	3	High	1987	Unique	Used	65370	1	25.594	1	5.2257	2.2255	1	32	22/72	0	1987	2685.22	MS2	895.744	52	Glioma_grade2_4.raw
4557287	NP_000020.1	AGT	angiotensinogen preproprotein [Homo sapiens]	2	2	3.165401459	7.963457628	10.320197	7.42	9.95E-05	485	53.121	6.3159	Peptide hormone	Cell communication,Signal transduction	Peptide hormone	Extracellular	-																								
4557287			dPTFIPAPIQAk	Unambiguous	N-Term(iTRAQ4plex); K12(iTRAQ4plex)	HCD	3.3436458	0	1	1	3.28473	3.2959	3.3913	3.4027576	1.852915359	49770.69	49602.582	163483.45	168785.56	0.239239998	2.256700338	2	High	4286	Unique	Used	59836	1	43.74	1	3.778	1.6806	1	64	15/22	24.56988	4286	1585.92	MS2	793.465	175	Glioma_grade2_3.raw
4557287			aDSQAQLLLSTVVGVFTAPGLHLk	Unambiguous	N-Term(iTRAQ4plex); K24(iTRAQ4plex)	HCD	2.9871573	0	1	1	3.40135	3.2866	2.6773	2.5869434	13.89818638	3821.7656	3955.2463	12999.16	10231.998	2.427284652	16.84529901	3	High	7562	Unique	Used	84128	1	76.935	1	6.5422	0.1334	1	11	35/92	4.043294	7562	2753.58	MS2	918.532	46	Glioma_grade2_6.raw
4557303	NP_000373.1	ALDH3A2	fatty aldehyde dehydrogenase isoform 2 [Homo sapiens]	5	13	2.443501949	32.09019398	79.360867	13.61	2.14E-04	485	54.813	7.8843	Enzyme: Dehydrogenase	Metabolism	Catalytic activity	Endoplasmic reticulum	Microsome																								
4557303			iLSLLEGQk	Unambiguous	N-Term(iTRAQ4plex); K9(iTRAQ4plex)	HCD	3.1830308	0	1	2	3.08392	3.0933	3.2725	3.2824466	3.430401665	186345.22	185780.94	574672.94	609816	0.214446743	4.195884858	2	High	6008	Unique	Used	69094	1	56.681	1	3.7697	-0.9381	1	21	14/16	40.82752	6008	1288.81	MS2	644.907	157	Glioma_grade2_4.raw
4557303			iLSLLEGQk	Unambiguous	N-Term(iTRAQ4plex); K9(iTRAQ4plex)	HCD	2.443502	0	1	2	2.31371	2.3531	2.5324	2.5755022	5.300706499	109831.34	107992.01	254118.17	278133.66	1.194181171	6.381007965	2	High	5899	Unique	Used	61329	1	56.389	1	3.8011	-1.2223	1	57	14/16	39.25983	5899	1288.81	MS2	644.907	159	Glioma_grade2_3.raw
4557303			iLSLLEGQk	Unambiguous	N-Term(iTRAQ4plex); K9(iTRAQ4plex)	HCD	1.7783535	0	1	2	1.54046	1.8736	1.7001	2.0676904	12.63297544	96581.16	79408.98	148779.48	164193.19	13.7991423	6.964914134	2	High	6136	Unique	Used	53555	1	56.673	1	3.4389	0.1037	1	44	14/16	50.83831	6136	1288.81	MS2	644.908	173	Glioma_grade2_2.raw
4557303			hLTPVTLELGGk	Unambiguous	N-Term(iTRAQ4plex); K12(iTRAQ4plex)	HCD	3.2225711	0	1	6	3.11401	2.9769	3.4795	3.3263535	6.909844786	96438.28	100878.8	300309.78	335558.56	3.182615315	7.83956357	2	High	2558	Unique	Used	79967	1	30.783	1	4.2	6.1017	1	52	15/22	9.129482	2558	1552.94	MS2	776.973	120	Glioma_grade2_6.raw
4557303			iLSLLEGQk	Unambiguous	N-Term(iTRAQ4plex); K9(iTRAQ4plex)	HCD	1.8104312	0	1	2	1.65017	1.6877	1.9304	1.9743385	9.131218766	68409.29	66887.62	112886.94	132058.8	1.590550997	11.06902469	2	High	5998	Unique	Used	61422	1	57.151	1	3.5772	0.4826	1	47	14/16	25.18674	5998	1288.81	MS2	644.908	126	Glioma_grade2_3.raw
4557303			fDHIFYTGNTAVGk	Unambiguous	N-Term(iTRAQ4plex); K14(iTRAQ4plex)	HCD	1.5449748	0	1	2	1.44455	1.6032	1.4926	1.6564258	6.299254503	42620.42	38403.867	61567.332	63613.152	7.359653087	2.311243972	2	High	3748	Unique	Used	81009	1	40.852	1	4.8177	0.397	1	94	17/26	9.70862	3748	1857.97	MS2	929.491	204	Glioma_grade2_6.raw
4557303			vMQEEIFGPILPIVPVk	Unambiguous	N-Term(iTRAQ4plex); K17(iTRAQ4plex)	HCD	3.271696	0	1	2	2.91699	3.2879	3.2573	3.6715076	9.3968117	7398.8604	6564.234	21582.367	24100.635	8.453283639	7.79582909	2	High	7476	Unique	Used	77578	1	72.171	1	4.7572	-4.5073	1	99	20/32	21.44174	7476	2197.28	MS2	1099.14	75	Glioma_grade2_5.raw
4557303			ekDILTAIAADLck	Unambiguous	N-Term(iTRAQ4plex); K2(iTRAQ4plex); C13(Methylthio); K14(iTRAQ4plex)	HCD	2.0563264	1	1	2	1.94788	2.3334	1.825	2.1862414	11.06337524	7328.273	6117.3813	14274.581	13374.072	12.73615569	4.606054555	3	High	6691	Unique	Used	93370	1	74.906	1	6.1498	2.1525	1	87	25/52	26.27676	6691	1982.11	MS2	661.374	143	Glioma_grade2_7rep.raw
4557303			vMQEEIFGPILPIVPVk	Unambiguous	N-Term(iTRAQ4plex); K17(iTRAQ4plex)	HCD	4.394053	0	1	2	4.51797	4.2258	4.5739	4.2781477	3.923504073	6085.0845	6505.8164	27492.244	27832.844	4.725672641	0.870637819	2	High	7888	Unique	Used	70768	1	72.7	1	5.0502	-0.7296	1	72	19/32	4.086361	7888	2197.29	MS2	1099.15	70	Glioma_grade2_4.raw
4557303			vmQEEIFGPILPIVPVk	Unambiguous	N-Term(iTRAQ4plex); M2(Oxidation); K17(iTRAQ4plex)	HCD	3.0166805	0	1	2	2.79265	3.0924	2.9483	3.2647545	6.664602913	5119.7197	4623.45	14297.601	15094.43	7.203316394	3.833986085	2	High	7462	Unique	Used	62767	1	68.885	1	4.2993	-3.17	1	100	19/32	0	7462	2213.28	MS2	1107.14	92	Glioma_grade2_3.raw
4557303			vMQEEIFGPILPIVPVk	Unambiguous	N-Term(iTRAQ4plex); K17(iTRAQ4plex)	HCD	3.5661998	0	1	2	3.14763	3.8996	3.2971	4.084772	12.61213501	4657.336	3759.2444	14659.551	15355.657	15.09037234	3.279812507	2	High	7957	Unique	Used	63212	1	73.101	1	4.2184	0.0482	1	100	18/32	6.937497	7957	2197.29	MS2	1099.15	68	Glioma_grade2_3.raw
4557303			vmQEEIFGPILPIVPVk	Unambiguous	N-Term(iTRAQ4plex); M2(Oxidation); K17(iTRAQ4plex)	HCD	2.3383815	0	1	2	1.99789	2.6155	2.1267	2.7841356	15.88084333	4396.8423	3358.5925	8784.389	9350.777	18.93261933	4.41679768	2	High	7502	Unique	Used	70439	1	69.136	1	4.0969	2.566	1	100	17/32	15.61215	7502	2213.29	MS2	1107.15	95	Glioma_grade2_4.raw
4557303			vMQEEIFGPILPIVPVk	Unambiguous	N-Term(iTRAQ4plex); K17(iTRAQ4plex)	HCD	1.918827	0	1	2	2.07827	2.0187	1.816	1.763958	7.956131847	1822.9044	1876.6986	3788.4807	3310.4175	2.056341916	9.523780199	3	High	7873	Unique	Used	70757	1	72.531	1	3.7642	0.6254	1	37	26/64	19.4211	7873	2197.29	MS2	733.102	55	Glioma_grade2_4.raw
4557325	NP_000032.1	APOE	apolipoprotein E precursor [Homo sapiens]	2	3	2.515723228	20.67118323	10.949355	6.31	2.65E-05	317	36.132	5.7319	Transport/cargo protein	Transport	Transporter activity	Extracellular	Cytoplasm																								
4557325			qWAGLVEk	Unambiguous	N-Term(iTRAQ4plex); K8(iTRAQ4plex)	HCD	2.5157232	0	1	1	2.50779	2.4784	2.5535	2.5235672	1.245371573	556930.1	563535.4	1396661.8	1422119.4	0.833698569	1.27723582	2	High	2959	Unique	Used	58599	1	33.596	1	3.2883	-3.3839	1	12	13/14	45.92665	2959	1218.7	MS2	609.856	278	Glioma_grade2_3.raw
4557325			qWAGLVEk	Unambiguous	N-Term(iTRAQ4plex); K8(iTRAQ4plex)	HCD	2.0175767	0	1	1	1.8721	2.0953	1.9482	2.1803918	6.89118407	457727.38	408976.03	856912.7	891728	7.954834325	2.815688176	2	High	2887	Unique	Used	50496	1	33.008	1	3.0855	-0.5793	1	12	13/14	55.14743	2887	1218.71	MS2	609.858	291	Glioma_grade2_2.raw
4557325			sWFEPLVEDMQR	Unambiguous	N-Term(iTRAQ4plex)	HCD	3.0641644	0	1	1	2.95447	3.1409	2.992	3.1807775	3.606507033	34528.65	32478.88	102013.984	103308.09	4.326099669	0.891351924	2	High	5281	Unique	Used	87614	1	68.491	1	4.5755	1.9026	1	64	17/22	8.622189	5281	1680.82	MS2	840.914	115	Glioma_grade2_1rep.raw
4557469	NP_001273.1	AP2B1	AP-2 complex subunit beta isoform b [Homo sapiens]	14	39	0.355501801	29.67558297	330.48577	36.18	1.34E-04	937	104.49	5.3765	Unclassified,Transport/cargo protein	Biological_process unknown,Transport	Transporter activity,Molecular function unknown	Plasma membrane	Mitochondrion																								
4557469			qVFLATWk	Unambiguous	N-Term(iTRAQ4plex); K8(iTRAQ4plex)	HCD	0.3555018	0	1	2	0.32646	0.3596	0.3518	0.38749653	0.070534883	1743391.2	1582723.4	569140	613299.75	6.831351565	5.28156444	2	High	4664	Unique	Used	60185	1	46.728	1	3.9621	-0.6685	1	11	13/14	39.92056	4664	1280.76	MS2	640.884	225	Glioma_grade2_3.raw
4557469			qVFLATWk	Unambiguous	N-Term(iTRAQ4plex); K8(iTRAQ4plex)	HCD	0.31803602	0	1	2	0.27849	0.3104	0.3249	0.36211723	0.10871918	1423249.5	1276804.9	396361.53	462353.03	7.670361733	10.86811365	2	High	4803	Unique	Used	52298	1	46.996	1	4.1384	-5.9106	1	9	13/14	44.13733	4803	1280.75	MS2	640.88	226	Glioma_grade2_2.raw

4557469			aVWLPAVk	Unambiguous	N-Term(iTRAQ4plex); K8(iTRAQ4plex)	HCD	0.30908495	0	1	2	0.29967	0.3026	0.3155	0.31859323	0.030253435	1056920.4	1046677.06	316726.06	333464.22	0.6886427	3.640677754	2	High	4564	Unique	Used	67767	1	45.276	1	3.2736	-5.6163	1	9	14-Dec	60.0366	4564	1171.74	MS2	586.372	115	Glioma_grade2_4.raw
4557469			fLELLPk	Unambiguous	N-Term(iTRAQ4plex); K7(iTRAQ4plex)	HCD	0.25833353	0	1	2	0.22813	0.2613	0.2558	0.29291955	0.102375297	1010556.06	882439.94	230540.45	258483.9	9.571259235	8.080989417	2	High	5990	Unique	Used	61414	1	57.103	1	3.2276	-4.3429	1	13	12-Dec	51.29679	5990	1147.73	MS2	574.368	103	Glioma_grade2_3.raw
4557469			fLELLPk	Unambiguous	N-Term(iTRAQ4plex); K7(iTRAQ4plex)	HCD	0.2863246	0	1	2	0.27544	0.2729	0.2999	0.29710475	0.049311443	897105.06	905448.25	247102.38	269012.97	0.654574397	6.003745778	2	High	6006	Unique	Used	69092	1	56.669	1	3.1475	-5.7255	1	17	12-Dec	48.88002	6006	1147.73	MS2	574.367	196	Glioma_grade2_4.raw
4557469			fLELLPk	Unambiguous	N-Term(iTRAQ4plex); K7(iTRAQ4plex)	HCD	0.29428726	0	1	2	0.26945	0.2886	0.2996	0.32088932	0.07290876	730204	681731.44	196754.25	218760.34	4.855076926	7.489823867	2	High	5896	Unique	Used	61326	1	56.37	1	3.1141	-5.5128	1	23	12-Dec	55.05181	5896	1147.73	MS2	574.367	198	Glioma_grade2_3.raw
4557469			lLSTDPVTAk	Unambiguous	N-Term(iTRAQ4plex); K10(iTRAQ4plex)	HCD	0.40208036	0	1	2	0.40735	0.4	0.4042	0.39690414	0.011459092	673902.9	686349.6	274515.8	272415	1.294045918	0.543209461	2	High	2477	Unique	Used	50110	1	30.096	1	4.0498	-0.6954	1	9	15/18	18.92532	2477	1332.8	MS2	666.902	230	Glioma_grade2_2.raw
4557469			qVFLATWk	Unambiguous	N-Term(iTRAQ4plex); K8(iTRAQ4plex)	HCD	0.29541117	0	1	2	0.27914	0.2876	0.303	0.31218073	0.050374816	671093.25	651344.94	187325.67	203337.33	2.111881527	5.796276261	2	High	4880	Unique	Used	68057	1	47.786	1	3.9261	-2.8606	1	21	13/14	26.11221	4880	1280.76	MS2	640.882	237	Glioma_grade2_4.raw
4557469			qVFLATWk	Unambiguous	N-Term(iTRAQ4plex); K8(iTRAQ4plex)	HCD	0.35962817	0	1	2	0.37215	0.3401	0.381	0.34818605	0.05375825	529519.5	579463.8	197060.44	201761.22	6.369068535	1.666887107	2	High	4853	Unique	Used	68032	1	47.578	1	3.8547	9.1485	1	33	13/14	63.17513	4853	1280.77	MS2	640.89	193	Glioma_grade2_4.raw
4557469			fLELLPk	Unambiguous	N-Term(iTRAQ4plex); K7(iTRAQ4plex)	HCD	0.27773356	0	1	2	0.25432	0.2968	0.2614	0.30505288	0.090368504	513161.3	439752.1	130508.41	134147.64	10.8946192	1.944655496	2	High	6010	Unique	Used	61433	1	57.249	1	3.2512	9.6963	1	25	12-Dec	48.27381	6010	1147.74	MS2	574.376	84	Glioma_grade2_3.raw
4557469			iQPGNPNYTLSLk	Unambiguous	N-Term(iTRAQ4plex); K13(iTRAQ4plex)	HCD	0.3103557	0	1	2	0.29362	0.3003	0.3202	0.32747006	0.051706444	490159.84	479333.6	143920.4	156967.39	1.579238683	6.132262863	2	High	3851	Unique	Used	59431	1	40.373	1	5.4351	2.1495	1	27	18/24	7.617186	3851	1732.99	MS2	866.997	178	Glioma_grade2_3.raw
4557469			fLELLPk	Unambiguous	N-Term(iTRAQ4plex); K7(iTRAQ4plex)	HCD	0.29728398	0	1	2	0.26324	0.2762	0.3174	0.33300352	0.111217547	423895.6	404009.66	111586.34	134536.64	3.396882156	13.1871577	2	High	5881	Unique	Used	61312	1	56.255	1	3.1902	10.76	1	33	12-Dec	59.05522	5881	1147.75	MS2	574.376	74	Glioma_grade2_3.raw
4557469			fLELLPk	Unambiguous	N-Term(iTRAQ4plex); K7(iTRAQ4plex)	HCD	0.29741815	0	1	2	0.27611	0.3037	0.2917	0.3208602	0.063474173	266622.84	242395.86	73616.22	77775.18	6.731014733	3.885067208	2	High	6104	Unique	Used	69183	1	57.435	1	3.255	9.5899	1	43	12-Dec	59.47582	6104	1147.74	MS2	574.376	85	Glioma_grade2_4.raw
4557469			lLSTDPVTAk	Unambiguous	N-Term(iTRAQ4plex); K10(iTRAQ4plex)	HCD	0.6099606	0	1	2	0.55344	0.6234	0.5981	0.67362565	0.08195697	243410.94	216108.67	134712.53	145576.34	8.402522912	5.481397617	2	High	2595	Unique	Used	50223	1	30.882	1	4.0403	8.1888	1	33	16/18	28.61631	2595	1332.81	MS2	666.908	193	Glioma_grade2_2.raw
4557469			iQPGNPNYTLSLk	Unambiguous	N-Term(iTRAQ4plex); K13(iTRAQ4plex)	HCD	0.34021145	0	1	2	0.32574	0.3316	0.3487	0.35493907	0.040613529	240206.84	235982.19	78245.664	83759.3	1.254660849	4.813098696	2	High	3951	Unique	Used	51497	1	40.702	1	5.5454	1.5859	1	56	18/24	11.778	3951	1732.99	MS2	866.997	179	Glioma_grade2_2.raw
4557469			lLSTDPVTAk	Unambiguous	N-Term(iTRAQ4plex); K10(iTRAQ4plex)	HCD	0.58514327	0	1	2	0.62683	0.5161	0.669	0.5508207	0.118017194	238139.5	289211.72	149272.22	159303.81	13.69619021	4.597508961	2	High	2531	Unique	Used	58200	1	30.4	1	3.9058	-2.344	1	29	16/18	31.6495	2531	1332.79	MS2	666.901	225	Glioma_grade2_3.raw
4557469			fLELLPk	Unambiguous	N-Term(iTRAQ4plex); K7(iTRAQ4plex)	HCD	0.25488105	0	1	2	0.23687	0.2489	0.2606	0.2738015	0.062042203	182014.77	173241.69	43114.305	47433.84	3.492409039	6.74640544	2	High	6078	Unique	Used	53500	1	56.255	1	2.9559	-6.6828	1	38	12-Dec	49.88767	6078	1147.73	MS2	574.366	202	Glioma_grade2_2.raw
4557469			gLEISGTFTHR	Unambiguous	N-Term(iTRAQ4plex)	HCD	0.41552413	0	1	2	0.40085	0.4013	0.4297	0.43021074	0.040113134	174527	174321.19	69959.99	74994.85	0.083434371	4.912128009	2	High	2458	Unique	Used	65809	1	29.067	1	3.6908	-1.3495	1	39	17/20	46.51813	2458	1361.73	MS2	681.367	268	Glioma_grade2_4.raw
4557469			kVIAAMTVGk	Unambiguous	N-Term(iTRAQ4plex); K1(iTRAQ4plex); K10(iTRAQ4plex)	HCD	0.32246473	1	1	2	0.29981	0.3146	0.33	0.34623528	0.0619127	167235.92	159389.42	50138.91	55186.234	3.397356346	6.777103532	2	High	1601	Unique	Used	89475	1	23.379	1	4.6766	-6.4134	1	45	17/18	57.23926	1601	1449.91	MS2	725.458	89	Glioma_grade2_7rep.raw
4557469			aVWLPAVk	Unambiguous	N-Term(iTRAQ4plex); K8(iTRAQ4plex)	HCD	0.46626198	0	1	2	0.465	0.4574	0.4752	0.46750283	0.015722585	165982.4	168724.47	77181.93	78879.17	1.158587687	1.538025701	2	High	4255	Unique	Used	59807	1	43.5	1	3.1644	7.6143	1	37	14-Dec	63.11758	4255	1171.75	MS2	586.38	90	Glioma_grade2_3.raw
4557469			iQPGNPNYTLSLk	Unambiguous	N-Term(iTRAQ4plex); K13(iTRAQ4plex)	HCD	0.31204203	0	1	2	0.29912	0.3141	0.3101	0.32560894	0.035034938	163253.73	155465.03	48832.85	50620.805	3.455988964	2.542440708	2	High	3941	Unique	Used	59514	1	41.08	1	5.3532	0.7407	1	45	19/24	4.584525	3941	1732.98	MS2	866.996	176	Glioma_grade2_3.raw
4557469			gLEISGTFTHR	Unambiguous	N-Term(iTRAQ4plex)	HCD	0.49846268	0	1	2	0.47895	0.4762	0.5209	0.51785684	0.048565585	152695.55	153594.28	73134.2	79539.85	0.414965183	5.933527738	2	High	2361	Unique	Used	65719	1	28.335	1	4.025	0.7123	1	36	19/20	17.73204	2361	1361.73	MS2	681.369	265	Glioma_grade2_4.raw
4557469			aPEVSQYIYQVYDSILk	Unambiguous	N-Term(iTRAQ4plex); K17(iTRAQ4plex)	HCD	0.20415117	0	1	2	0.15494	0.189	0.2166	0.26416892	0.223897813	132313.77	108485.23	20500.97	28658.428	13.99451179	23.46730881	2	High	8649	Unique	Used	55864	1	76.409	1	5.3714	-1.6898	1	15	20/32	7.074802	8649	2304.23	MS2	1152.62	163	Glioma_grade2_2.raw
4557469			gLEISGTFTHR	Unambiguous	N-Term(iTRAQ4plex)	HCD	0.653079	0	1	2	0.65873	0.6778	0.629	0.6472687	0.031334947	127606.086	124009.305	84057.375	80267.34	2.021583994	3.261784996	2	High	2329	Unique	Used	65690	1	28.082	1	3.1631	13.531	1	38	17/20	23.19665	2329	1361.75	MS2	681.378	307	Glioma_grade2_4.raw
4557469			gLEISGTFTHR	Unambiguous	N-Term(iTRAQ4plex)	HCD	0.41392347	0	1	2	0.34878	0.4314	0.3998	0.49449864	0.145645731	122454.164	99007.914	42709.08	48959.28	14.97231717	9.64249563	3	High	2374	Unique	Used	65731	1	28.432	1	5.714	-2.4127	1	8	20/40	10.4954	2374	1361.73	MS2	454.58	227	Glioma_grade2_4.raw
4557469			lAPPLVTLLSGEPEVQYVALR	Unambiguous	N-Term(iTRAQ4plex)	HCD	0.3278029	0	1	2	0.28709	0.3179	0.3367	0.3728861	0.109202125	121975.15	110146.85	35018.03	41072.23	7.206444146	11.25233604	2	High	6032	Unique	Used	88184	1	76.504	1	5.0278	1.5342	1	15	22/40	5.576763	6032	2409.39	MS2	1205.2	43	Glioma_grade2_1rep.raw
4557469			aLQHMTDFAIQFNk	Unambiguous	N-Term(iTRAQ4plex); K14(iTRAQ4plex)	HCD	0.3163763	0	1	2	0.30267	0.2725	0.3651	0.32871392	0.123872497	121252.76	134669.47	36699.996	44267.73	7.414007468	13.21809638	3	High	4999	Unique	Used	82069	1	52.088	1	6.4714	0.2605	1	8	25/52	10.51085	4999	1952.03	MS2	651.348	183	Glioma_grade2_6.raw
4557469			lHDINAQMVEDQGFLDSLR	Unambiguous	N-Term(iTRAQ4plex)	HCD	0.21360184	0	1	2	0.20255	0.2422	0.1897	0.22681822	0.109653013	102848.68	86021.37	20831.775	19511.213	12.59988549	4.629197744	2	High	6696	Unique	Used	69720	1	62.287	1	6.5506	0.7534	1	30	28/36	4.179307	6696	2345.17	MS2	1173.09	148	Glioma_grade2_4.raw
4557469			aLQHMTDFAIQFNk	Unambiguous	N-Term(iTRAQ4plex); K14(iTRAQ4plex)	HCD	0.36437955	0	1	2	0.33952	0.3456	0.3829	0.38967726	0.070020856	101544.28	99768.32	34476.754	38877.445	1.247605325	8.484199924	2	High	4995	Unique	Used	82066	1	52.045	1	6.452	3.957	1	100	21/26	0	4995	1952.04	MS2	976.523	184	Glioma_grade2_6.raw
4557469			kVIAAMTVGk	Unambiguous	N-Term(iTRAQ4plex); K1(iTRAQ4plex); K10(iTRAQ4plex)	HCD	0.44787413	1	1	2	0.39451	0.4489	0.447	0.5085935	0.103674324	86470.02	75992.25	34113.48	38649.168	9.120766586	8.815555317	3	High	1593	Unique	Used	89468	1	23.314	1	5.6236	-9.0116	1	21	19/36	61.59419	1593	1449.91	MS2	483.973	112	Glioma_grade2_7rep.raw
4557469			dIPNENELQFQIk	Unambiguous	N-Term(iTRAQ4plex); K13(iTRAQ4plex)	HCD	0.45307562	0	1	2	0.47389	0.4194	0.4911	0.4346534	0.073372216	69776.01	78838.12	33066.184	34267.254	8.623513031	2.522624024	2	High	5338	Unique	Used	52801	1	50.902	1	6.2298	-0.9582	1	88	20/24	27.3246	5338	1876	MS2	938.506	234	Glioma_grade2_2.raw
4557469			qGHIYMEMNFTNk	Unambiguous	N-Term(iTRAQ4plex); K13(iTRAQ4plex)	HCD	0.39514697	0	1	2	0.36178	0.3839	0.4057	0.43056267	0.074497269	64559.223	60831.547	23355.986	26191.793	4.204240835	8.094079696	2	High	2947	Unique	Used	80288	1	34.418	1	5.1135	1.8156	1	100	19/24	0	2947	1900.93	MS2	950.97	116	Glioma_grade2_6.raw
4557469			qGHIYMEMNFTNk	Unambiguous	N-Term(iTRAQ4plex); K13(iTRAQ4plex)	HCD	0.55318415	0	1	2	0.50518	0.5753	0.5338	0.6078469	0.08140012	56961.582	50021.555	28775.928	30405.447	9.17404423	3.893941075	3	High	2942	Unique	Used	80283	1	34.386	1	5.4532	1.4232	1	28	20/48	35.1336	2942	1900.93	MS2	634.315	102	Glioma_grade2_6.raw
4557469			sQPDMAIMAVNSFVk	Unambiguous	N-Term(iTRAQ4plex); K15(iTRAQ4plex)	HCD	0.44470644	0	1	2	0.42429	0.4523	0.4375	0.46646887	0.040991374	50017.684	46916.637	21222.168	21885.15	4.524241445	2.175032406	2	High	6682	Unique	Used	54066	1	60.718	1	5.9261	1.5659	1	73	21/28	0	6682	1926.01	MS2	963.509	213	Glioma_grade2_2.raw
4557469			lHDINAQmVEDQGFLDSLR	Unambiguous	N-Term(iTRAQ4plex); M8(Oxidation)	HCD	0.45980763	0	1	2	0.45683	0.5297	0.3996	0.46326536	0.115179278	49091.918	42340.348	22426.44	19614.816	10.44288005	9.45794006	2	High	5911	Unique	Used	69003	1	55.942	1	4.4565	1.2475	1	61	19/36	8.630671	5911	2361.17	MS2	1181.09	151	Glioma_grade2_4.raw
4557469			lHDINAQmVEDQGFLDSLR	Unambiguous	N-Term(iTRAQ4plex); M8(Oxidation)	HCD	0.39605087	0	1	2	0.40291	0.3988	0.3933	0.3892578	0.015151283	44628.773	45094.414	17981.594	17553.352	0.733941626	1.704310017	3	High	5907	Unique	Used	68999	1	55.921	1	7.3916	0.4405	1	6	30/72	10.32664	5907	2361.16	MS2	787.726	130	Glioma_grade2_4.raw
4557469			aPEVSQYIYQVYDSILk	Unambiguous	N-Term(iTRAQ4plex); K17(iTRAQ4plex)	HCD	0.29003587	0	1	2	0.23061	0.3429	0.2545	0.37839323	0.233370611	40769.453	27419.188	9401.918	10375.234	27.68808052	6.959933805	3	High	8644	Unique	Used	55859	1	76.383	1	5.2666	-0.5037	1	5	24/64	5.327924	8644	2304.24	MS2	768.75	165	Glioma_grade2_2.raw
4557469			sQPDMAIMAVNSFVk	Unambiguous	N-Term(iTRAQ4plex); K15(iTRAQ4plex)	HCD	0.53509307	0	1	2	0.50183	0.5332	0.5369	0.5704295	0.052379314	17985.656	16928.896	9025.793	9656.741	4.280405271	4.776093108	3	High	6719	Unique	Used	54100	1	61.004	1	5.2634	1.9709	1	29	24/56	18.14872	6719	1926.01	MS2	642.675	227	Glioma_grade2_2.raw
4557469			lHDINAQMVEDQGFLDSLR	Unambiguous	N-Term(iTRAQ4plex)	HCD	0.5942156	0	1	2	0.62513	0.6911	0.5066	0.5600354	0.134256168	17135.537	15499.373	10711.975	8680.197	7.090215107	14.81715407	3	High	6657	Unique	Used	69685	1	61.956	1	7.3249	2.049	1	21	32/72	20.70632	6657	2345.17	MS2	782.396	150	Glioma_grade2_4.raw
4757900	NP_004334.1	CALR	calreticulin precursor [Homo sapiens]	11	28	2.655634403	29.28660329	205.09767	53.24	1.19E-04	417	48.112	4.437	Chaperone	Protein metabolism	Chaperone activity	Endoplasmic reticulum	Cytoplasm, Sarcoplasmic reticulum, Nucleus, Mitochondrion, Endoplasmic reticulum lumen, Extracellular, Plasma membrane																								
4757900			vHVIFNYk	Unambiguous	N-Term(iTRAQ4plex); K8(iTRAQ4plex)	HCD	2.439059	0	1	1	2.26031	2.3867	2.4887	2.6278038	6.380737264	1111088	1052260.4	2511400.5	2765134	3.845649169	6.80054981	2	High	2217	Unique	Used	79647	1	28.282	1	3.3015	3.7204	1	4	14-Dec	13.64494	2217	1307.78	MS2	654.392	220	Glioma_grade2_6.raw
4757900			vHVIFNYk	Unambiguous	N-Term(iTRAQ4plex); K8(iTRAQ4plex)	HCD	2.548931	0	1	1	2.45601	2.426	2.6734	2.6407168	4.944394099	683732.94	692187.75	1679254.9	1827871.9	0.86901135	5.992830855	3	High	2216	Unique	Used	79646	1	28.279	1	3.9344	3.8734	1	2	15/28	42.98087	2216	1307.78	MS2	436.597	225	Glioma_grade2_6.raw
4757900			ikDPDASkPEDWDER	Unambiguous	N-Term(iTRAQ4plex); K2(iTRAQ4plex); K8(iTRAQ4plex)	HCD	2.0317514	1	1	1	1.93413	2.5138	1.6609	2.1586287	17.45731146	244609.75	188207.31	473106.94	406269.72	18.42928641	10.74876186	3	High	1750	Unique	Used	89607	1	24.596	1	3.7326	-1.9486	1	2	21/56	7.595127	1750	2233.14	MS2	745.052	144	Glioma_grade2_7rep.raw
4757900			hEQNIDcGGGYVk	Unambiguous	N-Term(iTRAQ4plex); C7(Methylthio); K13(iTRAQ4plex)	HCD	2.1659877	0	1	1	1.94858	2.3033	2.0498	2.4229727	10.07001036	161392.1	136537.44	314485.22	330826.5	11.79802354	3.58122425	3	High	1478	Unique	Used	72232	1	23.027	1	5.8347	4.1941	1	1	27/48	6.24972	1478	1753.83	MS2	585.282	90	Glioma_grade2_5.raw
4757900			hEQNIDcGGGYVk	Unambiguous	N-Term(iTRAQ4plex); C7(Methylthio); K13(iTRAQ4plex)	HCD	2.8048868	0	1	1	2.81987	2.7226	2.8901	2.7904162	2.475071702	131722.39	136428.89	371440.6	380693.4	2.482179511	1.739774462	2	High	1427	Unique	Used	72186	1	22.632	1	5.634	2.4269	1	25	23/24	4.466251	1427	1753.83	MS2	877.418	63	Glioma_grade2_5.raw
4757900			fYALSASFEPFSNk	Unambiguous	N-Term(iTRAQ4plex); K14(iTRAQ4plex)	HCD	2.3880384	0	1	1	2.31008	2.3234	2.4523	2.466445	3.465082432	123374.26	122668.64	285004.44	302555.44	0.40557861	4.224397049	2	High	7004	Unique	Used	54367	1	63.132	1	4.8703	-3.6162	1	9	23/26	24.22349	7004	1895.97	MS2	948.489	142	Glioma_grade2_2.raw
4757900			kVHVIFNYk	Unambiguous	N-Term(iTRAQ4plex); K1(iTRAQ4plex); K9(iTRAQ4plex)	HCD	2.7336514	1	1	1	2.6095	3.0313	2.4774	2.8778663	9.143111255	120582.914	103803.836	314661.6	298733.56	10.57513408	3.672290174	3	High	2027	Unique	Used	85433	1	29.742	1	4.1563	-6.0919	1	6	19/32	9.851522	2027	1579.96	MS2	527.324	71	Glioma_grade2_1rep.raw
4757900			kVHVIFNYk	Unambiguous	N-Term(iTRAQ4plex); K1(iTRAQ4plex); K9(iTRAQ4plex)	HCD	3.0480306	1	1	1	2.7204	3.2013	2.9177	3.4335816	10.21720335	112675.18	95747.805	306521.72	328757.9	11.48574055	4.950057635	2	High	2028	Unique	Used	85434	1	29.745	1	4.9256	-4.8085	1	42	15/16	12.6924	2028	1579.96	MS2	790.484	93	Glioma_grade2_1rep.raw
4757900			fYALSASFEPFSNk	Unambiguous	N-Term(iTRAQ4plex); K14(iTRAQ4plex)	HCD	3.1609137	0	1	1	2.97884	3.0948	3.2245	3.3500748	5.077808759	52377.734	50414.863	156024.97	168893.56	2.70050458	5.601076216	2	High	6724	Unique	Used	62101	1	62.78	1	5.8385	2.5646	1	100	21/26	28.52736	6724	1895.98	MS2	948.495	171	Glioma_grade2_3.raw
4757900			fYALSASFEPFSNk	Unambiguous	N-Term(iTRAQ4plex); K14(iTRAQ4plex)	HCD	1.8600723	0	1	1	1.8641	2.492	1.3874	1.8546876	23.86505327	32672.113	24440.324	60904.098	45329.168	20.38348958	20.73387938	3	High	7024	Unique	Used	54386	1	63.277	1	3.4966	0.948	1	4	20/52	23.72777	7024	1895.98	MS2	632.665	147	Glioma_grade2_2.raw
4757900			vHVIFNYk	Unambiguous	N-Term(iTRAQ4plex); K8(iTRAQ4plex)	HCD	1.772263	0	1	1	1.50348	1.8569	1.7037	2.1042223	14.14331937	28890.365	23391.809	43436.2	49221.566	14.87339159	8.830067261	3	High	2847	Unique	Used	95388	1	39.998	1	3.9852	0.8632	1	100	14/28	45.10765	2847	1307.77	MS2	436.596	247	Glioma_grade2_8rep.raw
4757900			kPEDWDEEMDGEWEPPVIQNPEYk	Unambiguous	N-Term(iTRAQ4plex); K1(iTRAQ4plex); K24(iTRAQ4plex)	HCD	3.272681	0	1	1	3.39585	3.2191	3.3292	3.1559227	3.290877643	28809.832	30391.705	97833.88	95913.875	3.778797585	1.401459909	3	High	5450	Unique	Used	82451	1	56.103	1	5.563	-0.5668	1	3	30/92	3.126203	5450	3392.6	MS2	1131.54	47	Glioma_grade2_6.raw
4757900			kVHVIFNYk	Unambiguous	N-Term(iTRAQ4plex); K1(iTRAQ4plex); K9(iTRAQ4plex)	HCD	2.6965623	1	1	1	2.61598	2.7583	2.638	2.7815332	3.096587707	28122.84	26671.453	73568.69	74187.53	3.745957959	0.592308006	2	High	2195	Unique	Used	94969	1	31.836	1	4.5398	0.2135	1	100	16/16	17.02693	2195	1579.97	MS2	790.488	90	Glioma_grade2_8rep.raw
4757900			kVHVIFNYk	Unambiguous	N-Term(iTRAQ4plex); K1(iTRAQ4plex); K9(iTRAQ4plex)	HCD	1.7093874	1	1	1	1.54721	1.7857	1.6432	1.8965659	8.968326253	18634.99	16145.819	28832.27	30621.611	10.12115442	4.256257569	3	High	2187	Unique	Used	94963	1	31.748	1	3.6156	-0.1813	1	100	19/32	78.3725	2187	1579.97	MS2	527.328	72	Glioma_grade2_8rep.raw
4757900			kVHVIFNYk	Unambiguous	N-Term(iTRAQ4plex); K1(iTRAQ4plex); K9(iTRAQ4plex)	HCD	2.2948883	1	1	1	2.19938	2.3927	2.205	2.3987913	4.863092267	17112.36	15729.824	37636.58	37732.566	5.953322598	0.180106463	4	High	2014	Unique	Used	85422	1	29.622	1	3.598	-4.8255	1	81	17/48	40.55297	2014	1579.96	MS2	395.746	91	Glioma_grade2_1rep.raw
4757900			ikDPDASkPEDWDER	Unambiguous	N-Term(iTRAQ4plex); K2(iTRAQ4plex); K8(iTRAQ4plex)	HCD	2.5051007	1	1	1	2.3274	2.389	2.6182	2.6875107	6.955788064	12982.462	12647.633	30215.32	33990.65	1.847514466	8.315617517	3	High	1931	Unique	Used	85361	1	28.713	1	3.5549	2.2331	1	41	22/56	17.57334	1931	2233.15	MS2	745.055	142	Glioma_grade2_1rep.raw
4757900			iDNSQVESGSLEDDWDFLPPkk	Unambiguous	N-Term(iTRAQ4plex); K21(iTRAQ4plex); K22(iTRAQ4plex)	HCD	1.6021572	1	1	1	1.74026	1.3712	1.8952	1.4933413	14.56487714	12274.417	15577.838	21360.662	23263.027	16.77330177	6.028973498	4	High	5432	Unique	Used	82436	1	55.939	1	4.2913	-0.9274	1	7	29/126	35.86255	5432	2951.5	MS2	738.63	86	Glioma_grade2_6.raw
4757900			dPDASkPEDWDER	Unambiguous	N-Term(iTRAQ4plex); K6(iTRAQ4plex)	HCD	3.1343708	0	1	1	2.99266	2.7477	3.5555	3.2644837	11.08881031	10060.814	10957.676	30108.586	35771.156	6.034469669	12.15560816	2	High	1315	Unique	Used	72088	1	21.693	1	3.4662	3.221	1	48	17/24	0	1315	1847.87	MS2	924.439	84	Glioma_grade2_5.raw
4757900			ckDDEFTHLYTLIVRPDNTYEVk	Unambiguous	N-Term(iTRAQ4plex); C1(Methylthio); K2(iTRAQ4plex); K23(iTRAQ4plex)	HCD	2.696154	1	1	1	2.22506	2.5986	2.7797	3.2463324	15.64265065	9739.445	8339.528	21670.82	27072.88	10.95074155	15.67313626	3	High	4707	Unique	Used	87185	1	62.302	1	4.8569	1.2888	1	23	24/88	5.677636	4707	3277.66	MS2	1093.23	62	Glioma_grade2_1rep.raw

4757900			dPDASkPEDWDER	Unambiguous	N-Term(iTRAQ4plex); K6(iTRAQ4plex)	HCD	4.8281074	0	1	1	4.25329	5.2477	4.4881	5.5373144	12.48088289	9437.511	7649.217	40140.438	42356.117	14.80113471	3.798271687	2	High	1442	Unique	Used	64872	1	21.391	1	3.3853	1.5695	1	39	16/24	0	1442	1847.87	MS2	924.438	77	Glioma_grade 2_4.raw
4757900			ckDDEFTHLYTLIVRPDNTY EVk	Unambiguous	N-Term(iTRAQ4plex); C1(Methylthio); K2(iTRAQ4plex); K23(iTRAQ4plex)	HCD	2.9041517	1	1	1	3.03998	2.3839	3.5677	2.797643	16.77481212	8084.817	10310.047	24577.643	28843.832	17.10776685	11.29377716	4	High	4680	Unique	Used	87166	1	61.99	1	3.5141	2.011	1	4	26/132	15.075	4680	3277.67	MS2	820.172	57	Glioma_grade 2_1rep.raw
4757900			kPEDWDEEmDGEWEPPVI QNPEYk	Unambiguous	N-Term(iTRAQ4plex); K1(iTRAQ4plex); M9(Oxidation); K24(iTRAQ4plex)	HCD	4.3863735	0	1	1	3.63645	4.52	4.2789	5.3185053	15.66260284	7781.781	6260.647	28298.047	33297.285	15.31934743	11.47812659	3	High	4956	Unique	Used	82032	1	51.715	1	6.0428	-2.2597	1	9	26/92	0.997018	4956	3408.59	MS2	1136.87	54	Glioma_grade 2_6.raw
4757900			lFPNSLDQTDMHGDSEYNI MFGPDIcGPGTk	Unambiguous	N-Term(iTRAQ4plex); C26(Methylthio); K31(iTRAQ4plex)	HCD	3.3935626	0	1	1	3.03875	2.7499	4.1049	3.7146482	18.19106717	7245.591	8006.73	22017.521	29742.186	7.057372426	21.10584978	3	High	6928	Unique	Used	83638	1	70.438	1	7.4533	1.1565	1	43	35/120	7.86486	6928	3733.69	MS2	1245.24	44	Glioma_grade 2_6.raw
4757900			kVHVIFNYk	Unambiguous	N-Term(iTRAQ4plex); K1(iTRAQ4plex); K9(iTRAQ4plex)	HCD	2.615115	1	1	1	2.60347	2.5298	2.703	2.6264284	2.726922318	6892.624	7093.4487	17944.752	18630.436	2.030655928	2.651260774	4	High	2190	Unique	Used	94965	1	31.788	1	4.1628	1.1236	1	93	15/48	23.01382	2190	1579.97	MS2	395.748	79	Glioma_grade 2_8rep.raw
4757900			lFPNSLDQTDMHGDSEYNI MFGPDIcGPGTk	Unambiguous	N-Term(iTRAQ4plex); C26(Methylthio); K31(iTRAQ4plex)	HCD	3.0068614	0	1	1	2.41131	3.5426	2.6422	3.881811	22.58748681	6683.2134	4549.0435	16115.313	17658.525	26.87057502	6.461899119	3	High	7305	Unique	Used	77439	1	70.525	1	5.9981	-4.2381	1	26	31/120	4.384965	7305	3733.67	MS2	1245.23	27	Glioma_grade 2_5.raw
4757900			iDNSQVESGSLEDDWDFLP Pkk	Unambiguous	N-Term(iTRAQ4plex); K21(iTRAQ4plex); K22(iTRAQ4plex)	HCD	1.5614989	1	1	1	1.6017	1.5673	1.5555	1.5221647	2.101960379	5703.8066	5828.8164	9135.76	8872.418	1.532960495	2.068070562	3	High	5496	Unique	Used	82490	1	56.509	1	6.8115	-0.3231	1	94	24/84	36.59914	5496	2951.5	MS2	984.505	137	Glioma_grade 2_6.raw
4757900			lFPNSLDQTDmHGDSEYNI MFGPDIcGPGTk	Unambiguous	N-Term(iTRAQ4plex); M11(Oxidation); C26(Methylthio); K31(iTRAQ4plex)	HCD	4.1752763	0	1	1	4.02425	4.0128	4.3383	4.3258753	4.338996803	3908.3962	3919.5815	15728.35	16955.621	0.202075217	5.310319523	3	High	6800	Unique	Used	83545	1	69.03	1	6.7424	-0.6827	1	59	28/120	8.612128	6800	3749.68	MS2	1250.57	39	Glioma_grade 2_6.raw
4757900			iDDPTDSkPEDWDkPEHIPD PDAk	Unambiguous	N-Term(iTRAQ4plex); K8(iTRAQ4plex); K14(iTRAQ4plex); K24(iTRAQ4plex)	HCD	2.5789816	0	1	1	2.40204	3.0362	2.2173	2.8026323	14.24550804	1529.3617	1209.9304	3673.594	3390.99	16.49127074	5.657267428	4	High	3141	Unique	Used	95579	1	43.653	1	3.5119	0.0343	1	56	20/138	3.775685	3141	3336.67	MS2	834.923	49	Glioma_grade 2_8rep.raw
4758304	NP_004902.1	PDIA4	protein disulfide-isomerase A4 precursor [Homo sapiens]	13	28	3.151153564	33.64851642	159.28684	31.94	1.22E-04	645	72.887	5.0718	Chaperone	Protein metabolism	Chaperone activity	Endoplasmic reticulum	Mitochondrion, Plasma membrane																								
4758304			fDVSGYPTLk	Unambiguous	N-Term(iTRAQ4plex); K10(iTRAQ4plex)	HCD	1.7863287	0	1	1	1.58356	1.8495	1.7323	2.0231526	10.34293496	141623.05	121260.58	224267.9	245328.64	10.95423144	6.342547614	2	High	4366	Unique	Used	51888	1	43.767	1	3.1018	1.009	1	18	15/18	35.16555	4366	1414.78	MS2	707.895	231	Glioma_grade 2_2.raw
4758304			qLEPVYNSLAk	Unambiguous	N-Term(iTRAQ4plex); K11(iTRAQ4plex)	HCD	3.424119	0	1	1	3.38694	3.4497	3.399	3.4619825	1.07982396	111190.79	109167.55	376597	377936.12	1.298472955	0.250989866	2	High	3205	Unique	Used	50792	1	35.363	1	4.8354	0.3233	1	21	18/20	23.09669	3205	1549.88	MS2	775.445	214	Glioma_grade 2_2.raw
4758304			fDVSGYPTIk	Unambiguous	N-Term(iTRAQ4plex); K10(iTRAQ4plex)	HCD	3.6246486	0	1	1	3.388	3.6307	3.619	3.878253	5.51835153	107495.48	100309.91	364194.28	389027.22	4.89011885	4.662517007	2	High	4251	Unique	Used	51779	1	42.94	1	3.3828	3.5975	1	12	16/18	17.50468	4251	1414.79	MS2	707.897	236	Glioma_grade 2_2.raw
4758304			dGDDVIIIGVFk	Unambiguous	N-Term(iTRAQ4plex); K12(iTRAQ4plex)	HCD	2.5409105	0	1	1	2.42885	2.728	2.3743	2.6667676	6.82561099	75396.88	67129.22	183128.05	179018.02	8.203575977	1.605004347	2	High	6808	Unique	Used	54182	1	61.7	1	4.4833	1.9605	1	23	18/22	16.95582	6808	1578.9	MS2	789.954	223	Glioma_grade 2_2.raw
4758304			yALPLVGHR	Unambiguous	N-Term(iTRAQ4plex)	HCD	2.8477635	0	1	1	2.77753	2.7736	2.9221	2.9178944	2.929148115	66066.305	66160.57	183501.31	193049.56	0.100819778	3.586039954	2	High	2828	Unique	Used	66154	1	31.835	1	2.9726	1.1782	1	51	13/16	23.24379	2828	1169.69	MS2	585.35	248	Glioma_grade 2_4.raw
4758304			tFDSIVMDPk	Unambiguous	N-Term(iTRAQ4plex); K10(iTRAQ4plex)	HCD	1.6285546	0	1	1	1.51144	1.6756	1.5862	1.7583855	6.56767313	64307.08	58008.53	97196.28	102001.37	7.282385979	3.411397397	2	High	4397	Unique	Used	51917	1	43.999	1	3.0109	2.0964	1	66	14/18	32.46885	4397	1440.77	MS2	720.888	262	Glioma_grade 2_2.raw
4758304			fHHTFSTEIAk	Unambiguous	N-Term(iTRAQ4plex); K11(iTRAQ4plex)	HCD	3.064127	0	1	1	2.8797	2.9031	3.2238	3.2500508	6.52756255	46930.816	46552.473	135146.77	151297.89	0.572356629	7.974012485	3	High	1677	Unique	Used	85178	1	25.905	1	5.3939	-3.8838	1	17	16/40	11.28761	1677	1605.86	MS2	535.957	248	Glioma_grade 2_1rep.raw
4758304			sHMMDVQGSTQDSAIk	Unambiguous	N-Term(iTRAQ4plex); K16(iTRAQ4plex)	HCD	3.281643	0	1	1	2.87426	3.6507	2.9911	3.7990825	13.9345497	44498.973	35034.598	127901.445	133099.33	16.82892811	2.816435876	3	High	1279	Unique	Used	72053	1	21.464	1	6.3473	2.4156	1	3	26/60	14.70296	1279	2022.99	MS2	675.001	132	Glioma_grade 2_5.raw
4758304			yGIVDYMIEQSGPPSk	Unambiguous	N-Term(iTRAQ4plex); K16(iTRAQ4plex)	HCD	3.251147	0	1	1	2.85819	3.596	2.977	3.745553	13.411282	43758.555	34779.867	125070.12	130269.84	16.16760564	2.87989179	2	High	6562	Unique	Used	53956	1	59.781	1	6.242	-1.9936	1	22	22/30	0	6562	2072.06	MS2	1036.53	180	Glioma_grade 2_2.raw
4758304			vEGFPTIYFAPSGDk	Unambiguous	N-Term(iTRAQ4plex); K15(iTRAQ4plex)	HCD	4.6221056	0	1	1	3.89466	5.0521	4.2907	5.5657353	15.96464334	39530.895	30474.578	153959.25	169613.44	18.29509291	6.841853002	2	High	6407	Unique	Used	53809	1	58.684	1	4.9269	-2.6786	1	24	21/28	29.55529	6407	1916	MS2	958.503	235	Glioma_grade 2_2.raw
4758304			rFDVSGYPTLk	Unambiguous	N-Term(iTRAQ4plex); K11(iTRAQ4plex)	HCD	1.947646	1	1	1	1.85744	1.8939	2.0004	2.039626	4.423251293	34945.77	34273.3	64909.53	69904.7	1.373922236	5.239978866	2	High	2599	Unique	Used	80006	1	31.072	1	3.5328	5.7752	1	81	15/20	14.76517	2599	1570.89	MS2	785.95	250	Glioma_grade 2_6.raw
4758304			fDVSGYPTIk	Unambiguous	N-Term(iTRAQ4plex); K10(iTRAQ4plex)	HCD	2.4878147	0	1	1	2.9607	2.3363	2.6798	2.1146588	14.79188851	29718.957	37661.973	87989.05	79642.23	16.67106844	7.041756197	2	High	4585	Unique	Used	52093	1	45.412	1	3.0114	0.5776	1	18	16/18	26.42486	4585	1414.78	MS2	707.895	220	Glioma_grade 2_2.raw
4758304			yALPLVGHR	Unambiguous	N-Term(iTRAQ4plex)	HCD	4.664617	0	1	1	4.07962	4.5197	4.7954	5.312727	11.03998235	25622.072	23127.158	104528.2	122868.28	7.237737326	11.40597685	3	High	2911	Unique	Used	66232	1	32.459	1	3.9007	1.3233	1	9	18/32	3.258072	2911	1169.69	MS2	390.569	136	Glioma_grade 2_4.raw
4758304			fHHTFSTEIAk	Unambiguous	N-Term(iTRAQ4plex); K11(iTRAQ4plex)	HCD	2.7073977	0	1	1	2.5501	2.8754	2.5584	2.884764	6.925294619	24568.934	21789.62	62653.133	62857.91	8.478572375	0.230735403	2	High	1674	Unique	Used	85176	1	25.865	1	4.1643	3.8402	1	100	15/20	25.01046	1674	1605.87	MS2	803.438	250	Glioma_grade 2_1rep.raw
4758304			yALPLVGHR	Unambiguous	N-Term(iTRAQ4plex)	HCD	4.6224504	0	1	1	4.94744	4.5935	4.6536	4.320711	5.55451469	20777.744	22378.762	102796.66	96692.16	5.246442725	4.327594244	3	High	2355	Unique	Used	73039	1	29.634	1	3.6399	11.342	1	14	17/32	4.030191	2355	1169.7	MS2	390.573	130	Glioma_grade 2_5.raw
4758304			fHHTFSTEIAk	Unambiguous	N-Term(iTRAQ4plex); K11(iTRAQ4plex)	HCD	1.8117646	0	1	1	1.79582	1.759	1.8656	1.827384	2.504377895	18319.586	18702.727	32898.652	34177.062	1.463558472	2.695379076	3	High	1822	Unique	Used	94703	1	27.545	1	5.0918	-0.007	1	90	16/40	37.38667	1822	1605.86	MS2	535.959	257	Glioma_grade 2_8rep.raw
4758304			yALPLVGHR	Unambiguous	N-Term(iTRAQ4plex)	HCD	5.404762	0	1	1	5.05195	5.1826	5.6213	5.766699	6.333938221	16462.375	16047.258	83167.11	92539.7	1.805815807	7.543728039	3	High	2809	Unique	Used	66136	1	31.698	1	3.5412	-0.79	1	16	17/32	0	2809	1169.69	MS2	390.568	136	Glioma_grade 2_4.raw
4758304			vEGFPTIYFAPSGDk	Unambiguous	N-Term(iTRAQ4plex); K15(iTRAQ4plex)	HCD	4.9291058	0	1	1	4.93548	4.7595	5.105	4.922959	2.862241703	16354.754	16959.393	80718.555	83490.39	2.566743414	2.387182166	2	High	6231	Unique	Used	61640	1	58.952	1	5.1669	-0.8947	1	73	20/28	4.652942	6231	1916	MS2	958.505	230	Glioma_grade 2_3.raw
4758304			rFDVSGYPTLk	Unambiguous	N-Term(iTRAQ4plex); K11(iTRAQ4plex)	HCD	2.3247745	1	1	1	2.11774	2.5019	2.1749	2.569365	9.724807731	12487.851	10570.463	26446	27159.379	11.7597328	1.882031758	2	High	2703	Unique	Used	80091	1	32.056	1	3.4542	-3.3166	1	100	18/20	25.51463	2703	1570.88	MS2	785.943	264	Glioma_grade 2_6.raw
4758304			kFAMEPEEFDSDTLR	Unambiguous	N-Term(iTRAQ4plex); K1(iTRAQ4plex)	HCD	1.9917922	1	1	1	1.73404	1.9535	2.0258	2.282165	11.30424312	12050.868	10696.975	20896.713	24412.262	8.417034717	10.97296303	3	High	4161	Unique	Used	81352	1	44.693	1	6.0803	1.4647	1	59	23/56	25.82978	4161	2103.03	MS2	701.683	129	Glioma_grade 2_6.raw
4758304			rFDVSGYPTLk	Unambiguous	N-Term(iTRAQ4plex); K11(iTRAQ4plex)	HCD	2.0289135	1	1	1	2.59995	1.8809	2.2335	1.6157992	20.54159724	11963.34	16536.725	31104.143	26720.025	22.69378366	10.72229718	3	High	2659	Unique	Used	80054	1	31.659	1	4.1707	-6.045	1	18	21/40	3.661153	2659	1570.87	MS2	524.296	251	Glioma_grade 2_6.raw
4758304			yALPLVGHR	Unambiguous	N-Term(iTRAQ4plex)	HCD	4.4840035	0	1	1	4.74811	4.8498	4.1259	4.214239	8.188488032	10346.973	10130.104	49128.6	42690.68	1.497767875	9.915775616	3	High	2287	Unique	Used	72975	1	29.146	1	3.8781	-1.8075	1	44	18/32	14.35562	2287	1169.69	MS2	390.568	133	Glioma_grade 2_5.raw
4758304			fHHTFSTEIAk	Unambiguous	N-Term(iTRAQ4plex); K11(iTRAQ4plex)	HCD	2.9027956	0	1	1	2.93594	2.7903	3.0212	2.8712943	3.369598163	10277.183	10813.574	30173.201	31048.953	3.596700806	2.022961093	4	High	1694	Unique	Used	85193	1	26.043	1	4.055	-2.7139	1	66	15/60	54.32539	1694	1605.86	MS2	402.22	257	Glioma_grade 2_1rep.raw
4758304			dLGLSESGEDVNAAILDES Gkk	Unambiguous	N-Term(iTRAQ4plex); K21(iTRAQ4plex); K22(iTRAQ4plex)	HCD	4.8247566	1	1	1	4.54254	5.4938	4.2716	5.1660686	11.51006756	9995.581	8264.906	45405.336	42697.07	13.40349826	4.347289344	3	High	4894	Unique	Used	75327	1	49.992	1	7.6742	0.0729	1	13	39/84	4.633718	4894	2679.41	MS2	893.807	103	Glioma_grade 2_5.raw
4758304			fHHTFSTEIAk	Unambiguous	N-Term(iTRAQ4plex); K11(iTRAQ4plex)	HCD	3.23818	0	1	1	2.96839	3.4448	3.0601	3.5512724	8.757411171	9572.322	8248.398	28414.393	29292.309	10.50637279	2.151501768	2	High	1868	Unique	Used	94739	1	28.019	1	3.4373	0.5715	1	100	14/20	34.00007	1868	1605.86	MS2	803.436	261	Glioma_grade 2_8rep.raw
4758304			yALPLVGHR	Unambiguous	N-Term(iTRAQ4plex)	HCD	3.532231	0	1	1	2.95527	4.4465	2.9246	4.4003406	23.27240327	8424.629	5599.1875	24897.027	24638.334	28.49279787	0.738557551	3	High	2730	Unique	Used	66063	1	31.083	1	3.9024	-2.4337	1	87	18/32	37.02247	2730	1169.69	MS2	390.568	129	Glioma_grade 2_4.raw
4758304			fHHTFSTEIAk	Unambiguous	N-Term(iTRAQ4plex); K11(iTRAQ4plex)	HCD	2.9138	0	1	1	2.7295	3.0227	2.8155	3.1178968	6.152168555	6419.1245	5796.5405	17520.986	18073.016	7.207685677	2.193314235	4	High	1853	Unique	Used	94728	1	27.853	1	3.6184	0.2507	1	100	13/60	45.70781	1853	1605.86	MS2	402.221	308	Glioma_grade 2_8rep.raw
4758304			yALPLVGHR	Unambiguous	N-Term(iTRAQ4plex)	HCD	3.341979	0	1	1	3.17283	3.7827	2.9723	3.5436373	10.80733774	5119.153	4293.8506	16242.211	15215.848	12.39937746	4.614068765	3	High	2448	Unique	Used	73126	1	30.316	1	3.7291	10.872	1	48	17/32	0	2448	1169.7	MS2	390.573	162	Glioma_grade 2_5.raw
4758334	NP_004256.1	FADS2	fatty acid desaturase 2 [Homo sapiens]	2	3	3.05711174	18.4167414	30.008151	15.77	6.82E-05	444	52.226	8.8218	Enzyme: Oxidase	Metabolism,Energy pathways	Catalytic activity	Endoplasmic reticulum	-																								
4758334			aFHPDLEFVGk	Unambiguous	N-Term(iTRAQ4plex); K11(iTRAQ4plex)	HCD	3.964726	0	1	1	3.53865	4.3452	3.6549	4.4879103	11.96076193	47829.215	38951.402	169250.97	174810.39	14.46765877	2.285117737	2	High	3643	Unique	Used	80914	1	40.004	1	4.2878	3.016	1	50	15/20	6.177916	3643	1547.85	MS2	774.429	235	Glioma_grade 2_6.raw
4758334			aFHPDLEFVGk	Unambiguous	N-Term(iTRAQ4plex); K11(iTRAQ4plex)	HCD	3.0571117	0	1	1	2.99614	3.0664	3.048	3.1195104	1.665563638	28039.857	27397.08	84011.43	85465.48	1.639744185	1.213343594	3	High	3617	Unique	Used	80890	1	39.805	1	5.0313	0.878	1	21	18/40	44.48986	3617	1547.85	MS2	516.621	235	Glioma_grade 2_6.raw
4758334			fLkPLLIGELAPEEPSQDHGk	Unambiguous	N-Term(iTRAQ4plex); K3(iTRAQ4plex); K21(iTRAQ4plex)	HCD	2.8200524	0	1	1	3.34653	2.4869	3.2684	2.428809	17.07171389	1094.6311	1473.0026	3663.2195	3577.6418	20.84012634	1.671419172	4	High	4419	Unique	Used	96319	1	60.703	1	6.6562	1.0391	1	56	31/120	15.76049	4419	2750.55	MS2	688.393	54	Glioma_grade 2_8rep.raw
4758426	NP_004284.1	GDA	guanine deaminase isoform b [Homo sapiens]	3	5	0.419755995	18.28119931	32.200757	12.33	1.52E-04	454	50.971	5.6812	Enzyme: Deaminase	Metabolism,Energy pathways	Deaminase activity	Cytoplasm	Plasma membrane																								
4758426			iVFLEEASQQEk	Unambiguous	N-Term(iTRAQ4plex); K12(iTRAQ4plex)	HCD	0.3823318	0	1	2	0.35088	0.3905	0.375	0.4173325	0.072682275	605090.2	543700.94	212315.31	226904.08	7.557294016	4.697341889	2	High	5175	Unique	Used	52647	1	49.717	1	5.3668	-0.6819	1	13	18/22	9.196624	5175	1708.93	MS2	854.971	226	Glioma_grade 2_2.raw
4758426			fVSEMLQk	Unambiguous	N-Term(iTRAQ4plex); K8(iTRAQ4plex)	HCD	0.419756	0	1	3	0.39748	0.4145	0.4248	0.442981	0.045365095	331987.2	318373.53	131959.38	141033.42	2.960301239	4.700721203	2	High	4276	Unique	Used	51803	1	43.11	1	3.1799	-0.1622	1	28	14/14	11.43817	4276	1269.71	MS2	635.359	222	Glioma_grade 2_2.raw
4758426			fVSEMLQk	Unambiguous	N-Term(iTRAQ4plex); K8(iTRAQ4plex)	HCD	0.40930307	0	1	3	0.36582	0.4074	0.411	0.45773876	0.091558983	261097.25	234421.78	95513.53	107303.94	7.613191255	8.221263054	2	High	4165	Unique	Used	59723	1	42.804	1	3.463	-1.7966	1	37	14/14	17.39228	4165	1269.71	MS2	635.358	244	Glioma_grade 2_3.raw
4758426			vcMDLNDTFPEYk	Unambiguous	N-Term(iTRAQ4plex); C2(Methylthio); K13(iTRAQ4plex)	HCD	0.5609568	0	1	3	0.51751	0.5529	0.5685	0.60737455	0.06632787	86347.195	80819.19	44685.594	49087.52	4.676645753	6.63864426	2	High	6546	Unique	Used	53941	1	59.664	1	4.5164	1.7304	1	45	20/24	19.49864	6546	1908.88	MS2	954.945	57	Glioma_grade 2_2.raw
4758426			iVFLEEASQQEk	Unambiguous	N-Term(iTRAQ4plex); K12(iTRAQ4plex)	HCD	0.55286026	0	1	2	0.53408	0.5238	0.5824	0.5712825	0.051261219	23117.652	23569.549	12346.628	13464.871	1.368852389	6.126860035	3	High	5162	Unique	Used	52635	1	49.622	1	3.2746	-2.9398	1	16	17/44	25.69439	5162	1708.93	MS2	570.315	223	Glioma_grade 2_2.raw
4758950	NP_000933.1	PPIB	peptidyl-prolyl cis-trans isomerase B precursor [Homo sapiens]	6	12	2.970469475	21.87781162	56.253423	27.78	1.44E-04	216	23.728	9.4077	Chaperone	Protein metabolism	Chaperone activity	Endoplasmic reticulum	Nucleolus, Golgi apparatus, Cytosol, Nucleus, Mitochondrion, Extracellular, Plasma membrane																								
4758950			vIFGLFGk	Unambiguous	N-Term(iTRAQ4plex); K8(iTRAQ4plex)	HCD	3.6321497	0	1	1	3.59515	3.6382	3.6262	3.6695902	0.847426534	626311.7	618904.75	2251685.8	2271126.8	0.841219946	0.607890008	2	High	6120	Unique	Used	76423	1	59.991	1	3.4802	-0.2734	1	4	13/14	8.833798	6120	1168.73	MS2	584.87	105	Glioma_grade 2_5.raw
4758950			vIFGLFGk	Unambiguous	N-Term(iTRAQ4plex); K8(iTRAQ4plex)	HCD	3.3055754	0	1	1	3.08944	3.3446	3.2695	3.5395625	5.628634436	305262.97	281972.78	943091.75	998060.25	5.608872172	4.004694027	2	High	6710	Unique	Used	69733	1	62.402	1	3.3348	0.5622	1	7	13/14	2.76119	6710	1168.73	MS2	584.871	108	Glioma_grade 2_4.raw
4758950			tVDNFVALATGEk	Unambiguous	N-Term(iTRAQ4plex); K13(iTRAQ4plex)	HCD	3.0771928	0	1	1	2.7465	2.937	3.2083	3.430814	9.760117694	271469.88	253865.33	745592.94	870964.75	4.739182316	10.96790514	2	High	6087	Unique	Used	53508	1	56.334	1	6.212	-1.0955	1	7	21/24	8.980614	6087	1652.91	MS2	826.958	251	Glioma_grade 2_2.raw
4758950			vIFGLFGk	Unambiguous	N-Term(iTRAQ4plex); K8(iTRAQ4plex)	HCD	3.665145	0	1	1	3.57755	3.6117	3.7181	3.7535772	2.291265297	154425.38	152966.25	552464.2	574170.6	0.671300463	2.724705936	2	High	6628	Unique	Used	69659	1	61.716	1	3.1404	0.2488	1	19	13/14	24.01908	6628	1168.73	MS2	584.87	130	Glioma_grade 2_4.raw
4758950			hYGPGWVSMANAGk	Unambiguous	N-Term(iTRAQ4plex); K14(iTRAQ4plex)	HCD	3.4123735	0	1	1	3.20574	3.259	3.5632	3.6224437	6.172164922	134552.97	132353	431341.6	479441.3	1.165664227	7.4686567	2	High	2261	Unique	Used	79688	1	28.607	1	4.9503	5.6423	1	28	19/26	12.84274	2261	1762.9	MS2	881.956	161	Glioma_grade 2_6.raw
4758950			hYGPGWVSMANAGk	Unambiguous	N-Term(iTRAQ4plex); K14(iTRAQ4plex)	HCD	2.827714	0	1	1	2.70132	3.217	2.5009	2.9782333	11.0072518	124095.27	104204.51	335221.16	310345.3	12.32142342	5.44944336	3	High	2256	Unique	Used	79683	1	28.581	1	5.1002	4.0767	1	3	23/52	8.846018	2256	1762.9	MS2	588.305	170	Glioma_grade 2_6.raw
4758950			dVIIADcGk	Unambiguous	N-Term(iTRAQ4plex); C7(Methylthio); K9(iTRAQ4plex)	HCD	1.8853942	0	1	1	1.81543	1.9123	1.8599	1.959092	3.309393559	92459.164	87775.984	167853.08	171961.22	3.674653221	1.709694767	2	High	3770	Unique	Used	51326	1	39.408	1	3.4492	0.7633	1	64	14/16	65.51657	3770	1267.66	MS2	634.336	197	Glioma_grade 2_2.raw
4758950			iEVEkPFAIAk	Unambiguous	N-Term(iTRAQ4plex); K5(iTRAQ4plex); K11(iTRAQ4plex)	HCD	2.6514642	0	1	1	2.60869	2.6199	2.6829	2.6944175	1.638354773	88878.086	88498.71	231855.75	238452.48	0.302473997	1.983632103	2	High	3430	Unique	Used	91033	1	39.395	1	5.2555	-4.8678	1	57	18/20	53.07673	3430	1677.02	MS2	839.015	94	Glioma_grade 2_7rep.raw

4758950			vIFGLFGk	Unambiguous	N-Term(iTRAQ4plex); K8(iTRAQ4plex)	HCD	3.3357408	0	1	1	2.84104	3.6683	3.0782	3.9744973	15.39829859	65596.74	50803.258	186362.81	201917.4	17.97349075	5.665370413	2	High	6580	Unique	Used	61966	1	61.678	1	3.2115	-2.1535	1	83	13/14	35.42965	6580	1168.73	MS2	584.869	122	Glioma_grade 2_3.raw
4758950			vIFGLFGk	Unambiguous	N-Term(iTRAQ4plex); K8(iTRAQ4plex)	HCD	2.8637464	0	1	1	2.70922	2.8925	2.8369	3.028725	4.619126541	60366.746	56542.473	163546.75	171251.6	4.626101166	3.254586937	2	High	6204	Unique	Used	76496	1	60.709	1	2.9845	-1.4223	1	40	13/14	4.582429	6204	1168.73	MS2	584.869	121	Glioma_grade 2_5.raw
4758950			tVDNFVALATGEk	Unambiguous	N-Term(iTRAQ4plex); K13(iTRAQ4plex)	HCD	1.8506603	0	1	1	1.84873	1.7065	2.0069	1.8524432	6.619306309	25296.941	27405.797	46767.188	50767.684	5.658857337	5.800546598	3	High	6077	Unique	Used	53499	1	56.249	1	5.139	-0.2912	1	24	21/48	32.66742	6077	1652.91	MS2	551.641	253	Glioma_grade 2_2.raw
4758950			dkPLkDVIIADcGk	Unambiguous	N-Term(iTRAQ4plex); K2(iTRAQ4plex); K5(iTRAQ4plex); C12(Methylthio); K14(iTRAQ4plex)	HCD	2.233345	1	1	1	1.90249	2.5406	2.0033	2.675182	16.85502467	7703.426	5768.5425	14655.652	15431.902	20.31134861	3.64862919	4	High	3925	Unique	Used	86646	1	53.279	1	5.8442	0.8425	1	23	23/78	13.42737	3925	2137.22	MS2	535.061	90	Glioma_grade 2_1rep.raw
4759182	NP_004594.1	STX1A	syntaxin-1A isoform 1 [Homo sapiens]	6	19	0.393559098	23.48822664	136.34344	35.42	3.71E-04	288	33.003	5.2368	Membrane transport protein	Cell communication,Signal transduction	Auxiliary transport protein activity	Plasma membrane	-																								
4759182			iAENVEEVk	Unambiguous	N-Term(iTRAQ4plex); K9(iTRAQ4plex)	HCD	0.37250558	0	1	2	0.36955	0.361	0.3843	0.37539205	0.026396932	2965137.2	3035716.2	1095767.8	1139583.6	1.663326403	2.772042848	2	High	1682	Unique	Used	49357	1	24.593	1	4.3419	-0.5889	1	3	16/16	17.78914	1682	1318.74	MS2	659.876	288	Glioma_grade 2_2.raw
4759182			fMDEFFEQVEEIR	Unambiguous	N-Term(iTRAQ4plex)	HCD	0.26026484	0	1	2	0.24388	0.2531	0.2672	0.27726632	0.056877287	1568345.6	1511499.1	382487.5	419087.78	2.610296918	6.457361355	2	High	5993	Unique	Used	88148	1	76.215	1	5.3077	2.0128	1	4	20/24	10.82775	5993	1862.88	MS2	931.943	108	Glioma_grade 2_1rep.raw
4759182			fmDEFFEQVEEIR	Unambiguous	N-Term(iTRAQ4plex); M2(Oxidation)	HCD	0.3326835	0	1	2	0.31546	0.3262	0.3389	0.35049093	0.045728274	232295.4	224641.77	73280.56	78734.9	2.368786796	5.074221794	2	High	5152	Unique	Used	87520	1	67.054	1	4.5248	2.2982	1	31	18/24	8.583629	5152	1878.87	MS2	939.941	98	Glioma_grade 2_1rep.raw
4759182			tkEELEELmSDIk	Unambiguous	N-Term(iTRAQ4plex); K2(iTRAQ4plex); M9(Oxidation); K13(iTRAQ4plex)	HCD	0.3721142	1	1	2	0.34982	0.3631	0.3808	0.3952606	0.053471471	205387	197857.62	71847.73	78205.33	2.640618321	5.991883234	3	High	4089	Unique	Used	81288	1	44.1	1	5.6403	0.2083	1	6	21/48	19.31578	4089	2013.08	MS2	671.698	199	Glioma_grade 2_6.raw
4759182			fmDEFFEQVEEIR	Unambiguous	N-Term(iTRAQ4plex); M2(Oxidation)	HCD	0.3649477	0	1	2	0.33968	0.3598	0.3698	0.3917104	0.059223905	145671.56	137516.84	49482.18	53866.777	4.072382775	5.999824984	2	High	5143	Unique	Used	87512	1	66.976	1	4.1055	13.798	1	69	18/24	35.01508	5143	1878.9	MS2	939.952	136	Glioma_grade 2_1rep.raw
4759182			kHSAILASPNPDEk	Unambiguous	N-Term(iTRAQ4plex); K1(iTRAQ4plex); K14(iTRAQ4plex)	HCD	0.28428075	1	1	2	0.26527	0.2836	0.2849	0.30460024	0.056463185	144947.34	135587.56	38450.67	41300	4.718388984	5.052698779	2	High	1044	Unique	Used	84678	1	19.58	1	6.0977	-3.149	1	94	21/26	10.61166	1044	1939.09	MS2	970.049	161	Glioma_grade 2_1rep.raw
4759182			kFVEVMSEYNATQSDYR	Unambiguous	N-Term(iTRAQ4plex); K1(iTRAQ4plex)	HCD	0.3186383	1	1	2	0.32384	0.321	0.3162	0.3134857	0.014605582	143666.94	144928.81	46524.547	45433.11	0.61835757	1.678517111	2	High	4205	Unique	Used	81390	1	45.08	1	6.466	-0.6634	1	62	25/32	0	4205	2355.15	MS2	1178.08	110	Glioma_grade 2_6.raw
4759182			iEYNVEHAVDYVER	Unambiguous	N-Term(iTRAQ4plex)	HCD	0.5334129	0	1	1	0.53773	0.4978	0.5719	0.52941495	0.056987907	128122.164	138399.78	68895.336	73270.914	5.453488639	4.352651738	2	High	5175	Unique	Used	60660	1	50.707	1	5.7947	0.848	1	51	22/26	34.24626	5175	1879.93	MS2	940.47	174	Glioma_grade 2_3.raw
4759182			tkEELEELmSDIk	Unambiguous	N-Term(iTRAQ4plex); K2(iTRAQ4plex); M9(Oxidation); K13(iTRAQ4plex)	HCD	0.40694523	1	1	2	0.34621	0.3681	0.4435	0.47152045	0.146664999	107908.46	101498.24	37358.562	47858.496	4.329097427	17.42509002	3	High	3981	Unique	Used	81200	1	43.07	1	4.4079	-18.438	1	30	19/48	60.89411	3981	2013.04	MS2	671.685	230	Glioma_grade 2_6.raw
4759182			kHSAILASPNPDEk	Unambiguous	N-Term(iTRAQ4plex); K1(iTRAQ4plex); K14(iTRAQ4plex)	HCD	0.40494904	1	1	2	0.36892	0.3617	0.4491	0.44027418	0.113728302	81491.71	83120.09	30063.76	36595.633	1.398974485	13.85770702	4	High	1032	Unique	Used	84669	1	19.457	1	5.5142	-3.4147	1	20	18/78	35.33107	1032	1939.09	MS2	485.528	165	Glioma_grade 2_1rep.raw
4759182			tkEELEELMSDIk	Unambiguous	N-Term(iTRAQ4plex); K2(iTRAQ4plex); K13(iTRAQ4plex)	HCD	0.43934408	1	1	2	0.43109	0.463	0.4174	0.44820616	0.045199048	70659.84	65797.01	30460.885	29490.623	5.039747098	2.288779257	3	High	5582	Unique	Used	82564	1	57.262	1	5.3729	-2.7641	1	88	20/48	52.39848	5582	1997.08	MS2	666.364	170	Glioma_grade 2_6.raw
4759182			iEYNVEHAVDYVER	Unambiguous	N-Term(iTRAQ4plex)	HCD	0.5115384	0	1	1	0.53043	0.5232	0.4997	0.4929064	0.035362341	57875.17	58669.516	30698.5	28918.58	0.963900565	4.222258125	2	High	5355	Unique	Used	52817	1	51.028	1	5.2081	-0.2558	1	71	22/26	41.77247	5355	1879.93	MS2	940.469	194	Glioma_grade 2_2.raw
4759182			iEYNVEHAVDYVER	Unambiguous	N-Term(iTRAQ4plex)	HCD	0.59468275	0	1	1	0.61692	0.5529	0.6413	0.5747502	0.06709152	57101.83	63717.53	35227.527	36621.664	7.743802537	2.744091375	3	High	5344	Unique	Used	52806	1	50.964	1	5.316	0.4513	1	10	25/52	25.24925	5344	1879.93	MS2	627.315	165	Glioma_grade 2_2.raw
4759182			kFVEVMSEYNATQSDYR	Unambiguous	N-Term(iTRAQ4plex); K1(iTRAQ4plex)	HCD	0.5133548	1	1	2	0.45904	0.4898	0.5354	0.5713167	0.096313207	40849.055	38281.715	18751.176	21870.984	4.588312545	10.86125106	3	High	4190	Unique	Used	81376	1	44.967	1	7.137	2.7517	1	18	25/64	15.68247	4190	2355.16	MS2	785.725	144	Glioma_grade 2_6.raw
4759182			kFVEVMSEYNATQSDYR	Unambiguous	N-Term(iTRAQ4plex); K1(iTRAQ4plex)	HCD	0.54357785	1	1	2	0.46053	0.7069	0.4372	0.67105526	0.245611353	34662.285	22581.662	15962.998	15153.544	29.8452182	3.678881879	3	High	4166	Unique	Used	91585	1	47.065	1	6.963	2.8294	1	19	25/64	12.2696	4166	2355.16	MS2	785.725	131	Glioma_grade 2_7rep.raw
4759182			kHSAILASPNPDEk	Unambiguous	N-Term(iTRAQ4plex); K1(iTRAQ4plex); K14(iTRAQ4plex)	HCD	0.33636153	1	1	2	0.30661	0.2905	0.3848	0.36454445	0.13432872	30099.486	31773.045	9228.851	11582.688	3.8252352	15.99510532	3	High	1106	Unique	Used	94159	1	19.81	1	7.3725	4.7131	1	57	25/52	26.73502	1106	1939.11	MS2	647.04	103	Glioma_grade 2_8rep.raw
4759182			kFVEVmSEYNATQSDYR	Unambiguous	N-Term(iTRAQ4plex); K1(iTRAQ4plex); M6(Oxidation)	HCD	0.3935591	1	1	2	0.32413	0.3883	0.398	0.47673136	0.157688474	26322.656	21973.428	8531.941	10475.422	12.73547815	14.46017098	2	High	2793	Unique	Used	80155	1	33.064	1	3.1408	0.7648	1	100	17/32	29.5726	2793	2371.15	MS2	1186.08	124	Glioma_grade 2_6.raw
4759182			kFVEVmSEYNATQSDYR	Unambiguous	N-Term(iTRAQ4plex); K1(iTRAQ4plex); M6(Oxidation)	HCD	0.30593306	1	1	2	0.35022	0.3785	0.2388	0.2580678	0.223192438	23876.572	22091.799	8362.062	5701.182	5.490841045	26.75806936	3	High	2723	Unique	Used	80104	1	32.304	1	6.103	2.6125	1	21	25/64	10.04213	2723	2371.15	MS2	791.056	126	Glioma_grade 2_6.raw
4759182			fMDEFFEQVEEIR	Unambiguous	N-Term(iTRAQ4plex)	HCD	0.45580238	0	1	2	0.42971	0.445	0.4662	0.48282406	0.051219574	11672.043	11270.797	5015.5894	5441.8115	2.473309909	5.764042903	3	High	5997	Unique	Used	88152	1	76.236	1	4.4117	0.0397	1	40	17/48	10.77235	5997	1862.88	MS2	621.63	89	Glioma_grade 2_1rep.raw
4826762	NP_005134.1	HP	haptoglobin isoform 1 preproprotein [Homo sapiens]	3	5	2.908862829	30.56051989	34.900466	16.26	6.25E-05	406	45.177	6.5806	Transport/cargo protein	Immune response	Transporter activity	Extracellular	-																								
4826762			gSFPWQAk	Unambiguous	N-Term(iTRAQ4plex); K8(iTRAQ4plex)	HCD	2.1622617	0	1	3	2.15315	1.967	2.376	2.1705823	7.716818353	387632.38	424312.6	834632.2	921005.44	6.388815237	6.957597892	2	High	2955	Unique	Used	58595	1	33.573	1	3.2599	-3.8161	1	7	14/14	14.77936	2955	1208.66	MS2	604.835	317	Glioma_grade 2_3.raw
4826762			gSFPWQAk	Unambiguous	N-Term(iTRAQ4plex); K8(iTRAQ4plex)	HCD	3.1414266	0	1	3	2.92551	3.2004	3.0875	3.377635	6.045374433	321356.66	293755.84	940130.75	992200	6.345742279	3.810788579	2	High	3235	Unique	Used	66534	1	34.916	1	3.3402	-2.4022	1	6	14/14	7.842739	3235	1208.66	MS2	604.836	281	Glioma_grade 2_4.raw
4826762			scAVAEYGVYVk	Unambiguous	N-Term(iTRAQ4plex); C2(Methylthio); K12(iTRAQ4plex)	HCD	1.5634346	0	1	3	1.44204	1.5963	1.5338	1.6978168	6.860039794	56362.805	50917.316	81277.17	86448.28	7.178482207	4.360133715	2	High	4921	Unique	Used	52410	1	47.819	1	5.3772	0.0748	1	61	19/22	26.65351	4921	1622.82	MS2	811.912	164	Glioma_grade 2_2.raw
4826762			sPVGVQPILNEHTFcAGMSk	Unambiguous	N-Term(iTRAQ4plex); C15(Methylthio); K20(iTRAQ4plex)	HCD	2.9088628	0	1	2	3.00501	2.8328	2.9895	2.8182244	3.419271834	18730.29	19868.932	56284.734	55995.11	4.171801595	0.364794049	3	High	5593	Unique	Used	75953	1	55.658	1	7.5435	-1.29	1	34	27/76	35.5781	5593	2449.22	MS2	817.08	108	Glioma_grade 2_5.raw
4826762			sPVGVQPILNEHTFcAGMSk	Unambiguous	N-Term(iTRAQ4plex); C15(Methylthio); K20(iTRAQ4plex)	HCD	3.632021	0	1	2	3.96767	3.1936	4.1766	3.361849	12.83141144	17572.584	21831.574	69722.27	73394.46	15.28549708	3.628688904	3	High	5298	Unique	Used	82334	1	54.557	1	7.7004	0.4295	1	27	29/76	34.30924	5298	2449.23	MS2	817.081	130	Glioma_grade 2_6.raw
4826870	NP_005004.1	NUCB2	nucleobindin-2 precursor [Homo sapiens]	4	4	2.2363379	23.75355886	21.57558	14.05	8.99E-04	420	50.191	5.1226	Calcium binding protein	Cell communication,Signal transduction	Calcium ion binding	Nucleus, Golgi apparatus	Cytoplasm, Extracellular, Plasma membrane																								
4826870			lVTLEEFLk	Unambiguous	N-Term(iTRAQ4plex); K9(iTRAQ4plex)	HCD	2.546443	0	1	1	2.42335	2.5756	2.5191	2.6772702	4.167182797	166576.81	156732.6	403673.5	419615.5	4.306034672	2.73845425	2	High	7073	Unique	Used	62426	1	65.492	1	3.995	0.1229	1	18	15/16	43.86016	7073	1379.84	MS2	690.423	158	Glioma_grade 2_3.raw
4826870			lEYHQVIQQMEQk	Unambiguous	N-Term(iTRAQ4plex); K13(iTRAQ4plex)	HCD	1.9262327	0	1	1	1.80289	2.3774	1.5841	2.0888846	17.5641552	23132.576	17542.652	41705.375	36644.58	19.43528462	9.134714788	3	High	4080	Unique	Used	74602	1	43.285	1	5.098	0.3383	1	18	22/48	27.62173	4080	1962.04	MS2	654.684	228	Glioma_grade 2_5.raw
4826870			lDSLQDIGMDHQALLk	Unambiguous	N-Term(iTRAQ4plex); K16(iTRAQ4plex)	HCD	1.5259587	0	1	1	1.56803	1.4326	1.6281	1.4875228	5.644721256	9408.472	10297.596	14752.736	15317.909	6.380832642	2.657991944	3	High	5315	Unique	Used	75703	1	53.412	1	5.384	-0.4919	1	38	32/60	32.73902	5315	2085.12	MS2	695.713	156	Glioma_grade 2_5.raw
4826870			lHDVNSDGFLDEQELEALFTk	Unambiguous	N-Term(iTRAQ4plex); K21(iTRAQ4plex)	HCD	2.5757918	0	1	1	1.90912	2.2175	2.8842	3.3501527	25.08664782	5212.8867	4487.928	9952.035	15035.244	10.56866312	28.76961156	3	High	7198	Unique	Used	77350	1	69.524	1	7.0986	0.4173	1	25	31/80	20.72624	7198	2708.37	MS2	903.461	134	Glioma_grade 2_5.raw
4885375	NP_005310.1	HIST1H1C	histone H1.2 [Homo sapiens]	4	7	2.037414551	14.93629387	34.830223	15.49	2.55E-04	213	21.352	10.931	DNA binding protein	Regulation of nucleobase, nucleoside, nucleotide and nucleic acid metabolism	DNA binding	Nucleus	Mitochondrion, Nucleolus																								
4885375			sGVSLAALk	Unambiguous	N-Term(iTRAQ4plex); K9(iTRAQ4plex)	HCD	2.0039062	0	1	3	1.92289	1.8732	2.1381	2.0828247	6.304835927	818045.5	839767.9	1573013.1	1749089.2	1.853050089	7.495531027	2	High	2512	Unique	Used	58182	1	30.27	1	3.1777	1.9709	1	4	16/16	56.36148	2512	1133.72	MS2	567.361	160	Glioma_grade 2_3.raw
4885375			sGVSLAALk	Unambiguous	N-Term(iTRAQ4plex); K9(iTRAQ4plex)	HCD	2.0374146	0	1	3	1.90388	2.2271	1.8752	2.1936204	9.078512902	677634.94	579277.4	1290136.8	1270714.6	11.06668799	1.072578387	2	High	2476	Unique	Used	50109	1	30.09	1	3.2298	-0.2903	1	7	16/16	46.57673	2476	1133.71	MS2	567.36	153	Glioma_grade 2_2.raw
4885375			aLAAAGYDVEk	Unambiguous	N-Term(iTRAQ4plex); K11(iTRAQ4plex)	HCD	2.6164038	0	1	5	2.45604	2.67	2.5671	2.7907436	5.455583235	403976.97	371599.47	992182.44	1037038.8	5.903828076	3.126148663	2	High	2124	Unique	Used	49775	1	27.662	1	4.4372	-0.2501	1	7	20/20	9.790433	2124	1395.77	MS2	698.39	279	Glioma_grade 2_2.raw
4885375			aSGPPVSELITk	Unambiguous	N-Term(iTRAQ4plex); K12(iTRAQ4plex)	HCD	1.8529228	0	1	3	1.7377	1.8913	1.8177	1.9783326	5.535184342	207376.86	190538.94	360357.88	376949.4	5.984284823	3.182384501	2	High	3203	Unique	Used	50790	1	35.351	1	4.8304	0.2655	1	21	20/22	25.9791	3203	1486.87	MS2	743.94	168	Glioma_grade 2_2.raw
4885375			aSGPPVSELITk	Unambiguous	N-Term(iTRAQ4plex); K12(iTRAQ4plex)	HCD	2.389978	0	1	3	2.25121	2.3574	2.421	2.5352983	4.971196512	121006.72	115553.62	272411.1	292962.9	3.259991923	5.140780137	2	High	3294	Unique	Used	58914	1	36.068	1	4.7622	0.2655	1	19	21/22	10.7509	3294	1486.87	MS2	743.94	159	Glioma_grade 2_3.raw
4885375			aSGPPVSELITk	Unambiguous	N-Term(iTRAQ4plex); K12(iTRAQ4plex)	HCD	2.677915	0	1	3	2.69427	2.6285	2.7285	2.6619632	1.601912099	107756.02	110450.836	290323.38	294016.06	1.746528686	0.893699412	2	High	3187	Unique	Used	58813	1	35.277	1	4.8713	1.1686	1	25	21/22	3.260815	3187	1486.87	MS2	743.941	164	Glioma_grade 2_3.raw
4885375			kASGPPVSELITk	Unambiguous	N-Term(iTRAQ4plex); K1(iTRAQ4plex); K13(iTRAQ4plex)	HCD	1.9921002	1	1	3	1.84257	2.0221	1.9647	2.1561978	6.522179616	40583.266	36979.438	74777.7	79734.984	6.570918974	4.537270394	2	High	2125	Unique	Used	89940	1	27.62	1	4.2633	-2.0722	1	100	20/24	0	2125	1759.07	MS2	880.036	63	Glioma_grade 2_7rep.raw
4885381	NP_005313.1	HIST1H1B	histone H1.5 [Homo sapiens]	2	2	2.127483845	26.78854795	17.645803	10.62	7.70E-05	226	22.566	10.917	DNA binding protein	Regulation of nucleobase, nucleoside, nucleotide and nucleic acid metabolism	DNA binding	Nucleus	Mitochondrion, Nucleolus																								
4885381			aLAAGGYDVEk	Unambiguous	N-Term(iTRAQ4plex); K11(iTRAQ4plex)	HCD	1.724488	0	1	1	1.77942	1.7261	1.7228	1.671203	2.562584637	99784.19	102867.36	177557.9	171912.25	2.151604974	2.284645712	2	High	1860	Unique	Used	49526	1	25.799	1	4.263	0.0305	1	23	19/20	13.74488	1860	1381.76	MS2	691.382	253	Glioma_grade 2_2.raw
4885381			aTGPPVSELITk	Unambiguous	N-Term(iTRAQ4plex); K12(iTRAQ4plex)	HCD	2.5304794	0	1	1	2.32979	2.5279	2.5328	2.7482407	6.7430988	35600.934	32810.35	82942.62	90170.75	5.768758469	5.904870015	2	High	3416	Unique	Used	59029	1	36.975	1	3.9731	0.7343	1	100	20/22	37.3812	3416	1500.89	MS2	750.948	156	Glioma_grade 2_3.raw
5031631	NP_005497.1	SCARB2	lysosome membrane protein 2 isoform 1 precursor [Homo sapiens]	8	9	2.402140141	18.65086852	95.563388	28.24	8.90E-05	478	54.255	5.1353	Cell surface receptor	Cell communication,Signal transduction	Receptor activity	Plasma membrane	-																								
5031631			tSLDWWITDk	Unambiguous	N-Term(iTRAQ4plex); K10(iTRAQ4plex)	HCD	2.7490385	0	1	1	2.48291	2.7718	2.7286	3.04614	8.368934071	391451.34	350647.2	971937	1068120.4	7.776025026	6.667649094	2	High	6386	Unique	Used	53790	1	58.512	1	4.6568	-2.2066	1	5	17/18	7.378596	6386	1552.82	MS2	776.914	223	Glioma_grade 2_2.raw
5031631			kLDDFVETGDIR	Unambiguous	N-Term(iTRAQ4plex); K1(iTRAQ4plex)	HCD	2.405257	1	1	2	2.24767	2.5626	2.2672	2.5849307	7.577321907	326104.9	286024.03	732975.1	739352.3	9.259962594	0.612548726	2	High	3218	Unique	Used	80535	1	36.539	1	4.8322	1.6187	1	11	19/22	6.981573	3218	1695.92	MS2	848.463	193	Glioma_grade 2_6.raw
5031631			eIIEAMLk	Unambiguous	N-Term(iTRAQ4plex); K8(iTRAQ4plex)	HCD	1.941269	0	1	1	1.86641	1.9385	1.9439	2.0190158	3.210394162	259579.47	249925.58	484482.72	504603.66	2.679593101	2.876928326	2	High	5143	Unique	Used	52617	1	49.49	1	3.6524	-3.2513	1	12	14/14	22.97276	5143	1234.73	MS2	617.868	217	Glioma_grade 2_2.raw
5031631			tSLDWWITDk	Unambiguous	N-Term(iTRAQ4plex); K10(iTRAQ4plex)	HCD	2.9101079	0	1	1	2.84288	2.8163	3.0048	2.9767046	3.24374328	92527.164	93401.52	263043.78	278028.75	0.66505398	3.916655648	2	High	6192	Unique	Used	61603	1	58.663	1	4.6091	-0.9488	1	48	16/18	28.78504	6192	1552.82	MS2	776.915	237	Glioma_grade 2_3.raw
5031631			dEVLYVFPSDFcR	Unambiguous	N-Term(iTRAQ4plex); C12(Methylthio)	HCD	2.4021401	0	1	2	2.33655	2.3345	2.4699	2.4676707	3.203184252	24472.947	24494.725	57182.305	60444.91	0.062896073	3.922578835	2	High	5820	Unique	Used	88015	1	74.438	1	4.4099	-0.3121	1	75	17/24	17.66017	5820	1779.82	MS2	890.414	79	Glioma_grade 2_1rep.raw
5031631			tLNIPVLTVIEWSQVHFLR	Unambiguous	N-Term(iTRAQ4plex)	HCD	1.9492342	0	1	1	1.83309	1.9778	1.9227	2.0745456	5.180480188	19416.447	17995.65	35592.15	37332.797	5.3707505	3.375589145	3	High	7971	Unique	Used	77985	1	76.826	1	7.6623	-1.2233	1	12	33/72	7.334313	7971	2409.37	MS2	803.796	78	Glioma_grade 2_5.raw

5031631			nGAPIIMSFPHFYQADER	Unambiguous	N-Term(iTRAQ4plex)	HCD	2.333143	0	1	2	2.30902	2.8391	1.9216	2.3628075	15.94793379	7375.851	5998.694	17030.967	14173.759	14.56194665	12.94900748	3	High	6289	Unique	Used	69352	1	58.925	1	5.1009	0.7266	1	47	27/68	15.05258	6289	2237.09	MS2	746.37	130	Glioma_grade 2_4.raw
5031631			tMVFPVMYLNESVHIDk	Unambiguous	N-Term(iTRAQ4plex); K17(iTRAQ4plex)	HCD	1.573445	0	1	2	1.55507	1.6792	1.4755	1.593291	5.370519468	7295.5327	6756.2227	11345.034	10764.629	5.427788163	3.712479121	3	High	6570	Unique	Used	76817	1	63.839	1	6.9829	-2.5897	1	100	33/64	33.36177	6570	2311.2	MS2	771.072	194	Glioma_grade 2_5.raw
5031631			fVSAIEGMHPNQEDHETFV DINPLTGIILk	Unambiguous	N-Term(iTRAQ4plex); K30(iTRAQ4plex)	HCD	2.6674566	0	1	2	2.56427	2.0038	3.5167	2.748087	23.08039409	5009.3574	6410.432	12845.367	17616.426	17.35074643	22.15003019	3	High	6294	Unique	Used	93125	1	69.944	1	9.0445	1.2205	1	28	32/116	15.39071	6294	3652.9	MS2	1218.3	82	Glioma_grade 2_7rep.raw
5031863	NP_005558.1	LGALS3BP	galectin-3-binding protein precursor [Homo sapiens]	2	2	3.132508993	3.033800161	19.625144	7.18	1.34E-04	585	65.289	5.2749	Extracellular matrix protein	Immune response	Extracellular matrix structural constituent	Extracellular	-																								
5031863			aSHEEVEGLVEk	Unambiguous	N-Term(iTRAQ4plex); K12(iTRAQ4plex)	HCD	3.1997082	0	1	1	3.16937	3.165	3.2345	3.230005	1.177391974	27972.234	28011.275	88654.33	90476.56	0.098622456	1.438625342	2	High	1325	Unique	Used	72097	1	21.768	1	5.5579	2.8331	1	31	22/22	0	1325	1614.86	MS2	807.935	240	Glioma_grade 2_5.raw
5031863			tLQALEFHTVPFQLLAR	Unambiguous	N-Term(iTRAQ4plex)	HCD	3.0653098	0	1	1	2.67183	3.4225	2.7865	3.5693474	14.43147228	4328.3784	3379.0154	11564.67	12060.88	17.41966305	2.970288149	3	High	7220	Unique	Used	70189	1	66.71	1	6.1094	1.5116	1	100	25/64	43.18256	7220	2128.21	MS2	710.074	104	Glioma_grade 2_4.raw
5031873	NP_005561.1	LMAN1	protein ERGIC-53 precursor [Homo sapiens]	6	8	2.11824894	25.46384584	81.24847	21.76	3.28E-04	510	57.513	6.771	Chaperone	Protein metabolism	Chaperone activity	Endoplasmic reticulum	Golgi apparatus																								
5031873			lVSGMQHPGSAGGVYETT QHFIDlk	Unambiguous	N-Term(iTRAQ4plex); K25(iTRAQ4plex)	HCD	3.0625691	0	1	1	2.96333	3.0739	3.0517	3.165506	2.710521781	18972.295	18289.922	56221.258	57896.855	2.58981142	2.076490699	3	High	4698	Unique	Used	91991	1	52.465	1	8.3961	-5.4735	1	15	35/96	6.79637	4698	2960.5	MS2	987.505	127	Glioma_grade 2_7rep.raw
5031873			yQEEFEHFQQELDkk	Unambiguous	N-Term(iTRAQ4plex); K14(iTRAQ4plex); K15(iTRAQ4plex)	HCD	1.836453	1	1	1	1.967	2.0406	1.6396	1.7010175	10.69554014	18254.684	17595.879	35906.973	29930.896	2.598818228	12.83675986	4	High	4653	Unique	Used	91955	1	52.036	1	3.4082	0.0688	1	9	23/84	19.93065	4653	2430.23	MS2	608.313	41	Glioma_grade 2_7rep.raw
5031873			ekYQEEFEHFQQELDk	Unambiguous	N-Term(iTRAQ4plex); K2(iTRAQ4plex); K16(iTRAQ4plex)	HCD	1.9799098	1	1	1	2.00309	1.9333	2.0282	1.9575363	2.168503297	15906.214	16480.09	31861.584	32260.375	2.505945792	0.879535887	3	High	4584	Unique	Used	91904	1	51.309	1	5.4063	-1.8602	1	35	25/60	16.88544	4584	2559.27	MS2	853.761	121	Glioma_grade 2_7rep.raw
5031873			gHPDLQGQPAEEIFESVGD R	Unambiguous	N-Term(iTRAQ4plex)	HCD	1.9472402	0	1	1	1.7294	1.7576	2.1338	2.168635	12.12418184	10251.759	10087.14	17729.398	21875.324	1.144636307	14.80435787	3	High	5458	Unique	Used	60925	1	52.869	1	5.2851	0.9289	1	59	27/76	33.33909	5458	2325.13	MS2	775.713	91	Glioma_grade 2_3.raw
5031873			gAGmPGQHGQITQQELDT VVk	Unambiguous	N-Term(iTRAQ4plex); M4(Oxidation); K21(iTRAQ4plex)	HCD	3.1633134	0	1	1	3.13131	2.8528	3.5042	3.1924686	8.431107204	9238.997	10141.1	28930.178	32375.143	6.582868482	7.946971234	3	High	2607	Unique	Used	73276	1	31.461	1	6.318	12.871	1	12	26/80	4.518153	2607	2498.32	MS2	833.446	149	Glioma_grade 2_5.raw
5031873			gPHLVQSDGTVPFWAHAG NAIPSSDQIR	Unambiguous	N-Term(iTRAQ4plex)	HCD	2.6404548	0	1	1	2.59892	2.0538	3.3829	2.6732805	20.37684517	4373.733	5534.71	11366.963	14795.832	16.57040787	18.53453747	3	High	4863	Unique	Used	81951	1	50.923	1	7.8152	1.1819	1	43	33/108	0	4863	3101.57	MS2	1034.53	103	Glioma_grade 2_6.raw
5031873			gPHLVQSDGTVPFWAHAG NAIPSSDQIR	Unambiguous	N-Term(iTRAQ4plex)	HCD	2.256588	0	1	1	2.2503	1.9237	2.646	2.261965	13.00918748	3441.2896	4025.5237	7743.927	9105.594	11.06538699	11.42873996	4	High	4707	Unique	Used	91998	1	52.554	1	6.5394	-1.8726	1	30	30/162	33.65416	4707	3101.56	MS2	776.146	67	Glioma_grade 2_7rep.raw
5031873			lVSGMQHPGSAGGVYETT QHFIDlk	Unambiguous	N-Term(iTRAQ4plex); K25(iTRAQ4plex)	HCD	1.571765	0	1	1	1.70795	1.8041	1.3518	1.4279168	13.81026995	3338.584	3160.711	5702.132	4513.2324	3.870426084	16.45910878	4	High	4679	Unique	Used	91976	1	52.28	1	3.8862	-1.0661	1	31	20/144	26.88395	4679	2960.52	MS2	740.884	77	Glioma_grade 2_7rep.raw
5031973	NP_005733.1	PDIA6	protein disulfide-isomerase A6 precursor [Homo sapiens]	5	9	2.488211632	32.41113527	72.573635	24.32	4.16E-05	440	48.091	5.0845	Enzyme: Isomerase	Protein metabolism	Isomerase activity	Endoplasmic reticulum	Mitochondrion, Endoplasmic reticulum lumen, Extracellular																								
5031973			kDVIELTDDSFDk	Unambiguous	N-Term(iTRAQ4plex); K1(iTRAQ4plex); K13(iTRAQ4plex)	HCD	3.750731	1	1	1	3.3753	4.0362	3.512	4.1996765	10.55073103	205199.27	171599.27	692608.44	720661.44	12.61087044	2.807173182	2	High	3559	Unique	Used	80841	1	39.284	1	5.4042	-0.4062	1	11	21/24	8.15138	3559	1957.05	MS2	979.028	201	Glioma_grade 2_6.raw
5031973			nLEPEWAAAASEVk	Unambiguous	N-Term(iTRAQ4plex); K14(iTRAQ4plex)	HCD	2.4882116	0	1	1	2.43306	2.4982	2.4785	2.544845	1.862238658	58180.688	56663.402	141556.81	144199.58	1.868416946	1.307911671	2	High	5432	Unique	Used	52890	1	51.572	1	5.7155	-0.9884	1	65	20/26	35.42424	5432	1802.95	MS2	901.979	212	Glioma_grade 2_2.raw
5031973			hHSLGGQYGVQGFPTlk	Unambiguous	N-Term(iTRAQ4plex); K17(iTRAQ4plex)	HCD	3.5221567	0	1	1	3.09161	3.7571	3.3288	4.045387	12.01113922	45640.85	37556.605	141103.48	151930.98	13.7418253	5.225459609	3	High	2782	Unique	Used	90495	1	33.481	1	7.0075	-2.0466	1	5	26/64	3.722866	2782	2114.13	MS2	705.383	180	Glioma_grade 2_7rep.raw
5031973			hHSLGGQYGVQGFPTlk	Unambiguous	N-Term(iTRAQ4plex); K17(iTRAQ4plex)	HCD	2.240525	0	1	1	2.03533	2.1459	2.3302	2.456866	8.379397049	40560.316	38469.723	82553.82	94514.95	3.741039498	9.553120105	3	High	2765	Unique	Used	90480	1	33.335	1	5.9211	8.0867	1	10	27/64	10.68931	2765	2114.16	MS2	705.39	172	Glioma_grade 2_7rep.raw
5031973			hHSLGGQYGVQGFPTlk	Unambiguous	N-Term(iTRAQ4plex); K17(iTRAQ4plex)	HCD	4.1803727	0	1	1	4.16806	4.0025	4.3657	4.1921988	3.553678562	22777.682	23720.107	94938.695	99439.4	2.86635181	3.27452435	2	High	2775	Unique	Used	90489	1	33.414	1	6.2854	-0.6256	1	62	22/32	12.42204	2775	2114.14	MS2	1057.57	185	Glioma_grade 2_7rep.raw
5031973			tGEAIVDAALSALR	Unambiguous	N-Term(iTRAQ4plex)	HCD	2.9713788	0	1	1	2.82241	2.817	3.126	3.12006	5.895026515	21688.514	21730.096	61213.934	67799.2	0.135439224	7.218623562	2	High	5735	Unique	Used	87948	1	73.56	1	4.997	1.9289	1	100	22/26	30.88036	5735	1530.86	MS2	765.936	228	Glioma_grade 2_1rep.raw
5031973			hHSLGGQYGVQGFPTlk	Unambiguous	N-Term(iTRAQ4plex); K17(iTRAQ4plex)	HCD	2.129317	0	1	1	2.34842	2.2201	2.0333	1.9221876	8.918457015	13705.726	14497.902	32186.775	27867.69	3.97220543	10.1709483	3	High	2901	Unique	Used	86037	1	39.918	1	6.0719	-7.2433	1	69	23/64	47.56974	2901	2114.12	MS2	705.379	175	Glioma_grade 2_1rep.raw
5031973			hHSLGGQYGVQGFPTlk	Unambiguous	N-Term(iTRAQ4plex); K17(iTRAQ4plex)	HCD	1.9861399	0	1	1	1.61386	2.2231	1.8141	2.4989445	19.5647602	7042.476	5112.573	11365.592	12776.037	22.45400242	8.262368906	3	High	3035	Unique	Used	95509	1	42.35	1	4.0418	2.9767	1	100	19/64	39.92198	3035	2114.14	MS2	705.387	170	Glioma_grade 2_8rep.raw
5031973			dGELPVEDDIDLSDVELDDL GkDEL	Unambiguous	N-Term(iTRAQ4plex); K22(iTRAQ4plex)	HCD	1.563071	1	1	1	1.62534	1.5686	1.5574	1.5029727	3.208762331	1833.8322	1900.2161	2980.6096	2855.973	2.514188467	3.019965315	3	High	5806	Unique	Used	88003	1	74.309	1	4.2146	-1.6761	1	77	18/96	11.81182	5806	3046.47	MS2	1016.16	112	Glioma_grade 2_1rep.raw
5032057	NP_005611.1	S100A11	protein S100-A11 [Homo sapiens]	2	2	2.174885511	18.75831152	20.645764	34.29	1.52E-04	105	11.733	7.1226	Calcium binding protein	Cell communication,Signal transduction	Calcium ion binding	Cytoplasm	Endosome, Nucleus, Plasma membrane																								
5032057			dGYNYTLSk	Unambiguous	N-Term(iTRAQ4plex); K9(iTRAQ4plex)	HCD	2.463365	0	1	1	2.35727	2.4984	2.4303	2.5758142	3.790943423	294970.66	278306.16	695325.1	716864.94	4.110956712	2.157070436	2	High	1755	Unique	Used	49426	1	25.093	1	3.5109	-0.7621	1	11	15/16	29.69741	1755	1348.7	MS2	674.853	175	Glioma_grade 2_2.raw
5032057			tEFLSFMNTELAAFTk	Unambiguous	N-Term(iTRAQ4plex); K16(iTRAQ4plex)	HCD	1.8864058	0	1	1	1.75433	1.9991	1.7875	2.036906	7.606801712	48601.64	42651.625	85263.34	86877.35	9.221140646	1.325982156	2	High	8712	Unique	Used	55924	1	76.837	1	3.969	-1.5197	1	30	19/30	51.06159	8712	2138.11	MS2	1069.56	233	Glioma_grade 2_2.raw
5174447	NP_006089.1	GNB2L1	guanine nucleotide-binding protein subunit beta-2-like 1 [Homo sapiens]	6	10	1.996773958	14.69402323	81.312551	33.75	9.95E-05	317	35.055	7.6938	Adapter molecule	Cell communication,Signal transduction	Receptor signaling complex scaffold activity	Cytoplasm	Cytoskeleton, Nucleus, Plasma membrane																								
5174447			iIVDELk	Unambiguous	N-Term(iTRAQ4plex); K7(iTRAQ4plex)	HCD	1.9779356	0	1	1	1.86058	1.9465	2.008	2.1007104	5.114367242	1151036.5	1100220	2141597	2311243.8	3.192234359	5.387949315	2	High	3465	Unique	Used	51039	1	37.194	1	2.9456	-2.9832	1	4	12-Dec	14.84903	3465	1117.7	MS2	559.356	235	Glioma_grade 2_2.raw
5174447			iIVDELk	Unambiguous	N-Term(iTRAQ4plex); K7(iTRAQ4plex)	HCD	2.0063288	0	1	1	1.93869	1.9516	2.0607	2.074421	3.543656316	475872.44	472725.84	922567.7	980632.3	0.469109473	4.314614587	2	High	3374	Unique	Used	58989	1	36.669	1	2.9719	-1.5634	1	13	12-Dec	26.80587	3374	1117.71	MS2	559.356	192	Glioma_grade 2_3.raw
5174447			lWNTLGVck	Unambiguous	N-Term(iTRAQ4plex); C8(Methylthio); K9(iTRAQ4plex)	HCD	1.987219	0	1	1	1.90339	1.9368	2.0368	2.072515	4.03550087	175240.31	172216.6	333551.47	356921.47	1.230705612	4.786599016	2	High	6344	Unique	Used	69403	1	59.355	1	3.6916	0.5798	1	31	15/16	15.90035	6344	1367.74	MS2	684.375	262	Glioma_grade 2_4.raw
5174447			dGQAmLWDLNEGk	Unambiguous	N-Term(iTRAQ4plex); M5(Oxidation); K13(iTRAQ4plex)	HCD	1.755381	0	1	1	1.58909	2.0548	1.5238	1.9704078	14.95863583	81453.29	62991.88	129436.6	124119.7	18.07493903	2.96551074	2	High	4188	Unique	Used	51720	1	42.465	1	4.2765	1.6984	1	38	14/24	13.69045	4188	1780.88	MS2	890.944	200	Glioma_grade 2_2.raw
5174447			lWNTLGVck	Unambiguous	N-Term(iTRAQ4plex); C8(Methylthio); K9(iTRAQ4plex)	HCD	1.7590835	0	1	1	1.78149	1.7045	1.8161	1.7376484	2.780001709	61034.67	63789.625	108732.42	110843.93	3.121263152	1.359948865	2	High	5900	Unique	Used	76230	1	58.134	1	3.5166	-2.1869	1	48	15/16	14.6699	5900	1367.74	MS2	684.373	305	Glioma_grade 2_5.raw
5174447			dGQAMLWDLNEGk	Unambiguous	N-Term(iTRAQ4plex); K13(iTRAQ4plex)	HCD	2.434922	0	1	1	2.49917	2.2941	2.5884	2.3759449	5.340725913	35925.156	39137.285	89783.164	92988.03	6.051810114	2.479802678	2	High	5203	Unique	Used	60686	1	50.921	1	5.5907	-2.8966	1	66	18/24	16.64212	5203	1764.88	MS2	882.943	168	Glioma_grade 2_3.raw
5174447			yTVQDESHSEWVScVR	Unambiguous	N-Term(iTRAQ4plex); C14(Methylthio)	HCD	1.8288509	0	1	1	1.69738	1.9357	1.7351	1.9787891	7.681753646	28759.438	25218.168	48815.555	49901.438	9.278129271	1.555629299	2	High	4854	Unique	Used	52347	1	47.337	1	5.6896	-0.2255	1	73	24/30	0	4854	2114.94	MS2	1057.97	44	Glioma_grade 2_2.raw
5174447			hLYTLDGGDIINALcFSPNR	Unambiguous	N-Term(iTRAQ4plex); C15(Methylthio)	HCD	2.0716121	0	1	1	2.08385	1.8736	2.2919	2.060613	8.237844563	24710.299	27483.51	51492.457	56632.87	7.514133731	6.723347788	2	High	7621	Unique	Used	77692	1	73.633	1	5.6008	-5.4869	1	54	24/38	7.702936	7621	2409.17	MS2	1205.09	89	Glioma_grade 2_5.raw
5174447			hLYTLDGGDIINALcFSPNR	Unambiguous	N-Term(iTRAQ4plex); C15(Methylthio)	HCD	2.7124805	0	1	1	3.01837	2.7507	2.6705	2.4337118	8.868979652	3743.921	4108.19	11300.548	9998.15	6.56070909	8.647800514	3	High	7603	Unique	Used	77677	1	73.468	1	5.7029	-0.907	1	23	27/76	8.930424	7603	2409.18	MS2	803.731	150	Glioma_grade 2_5.raw
5174447			tNHIGHTGYLNTVTVSPDGS LcASGGk	Unambiguous	N-Term(iTRAQ4plex); C22(Methylthio); K27(iTRAQ4plex)	HCD	2.1967561	0	1	1	2.51025	2.449	1.9382	1.8909172	14.91746908	3228.639	3309.425	8104.6836	6257.8486	1.747438643	18.18494864	3	High	3580	Unique	Used	80858	1	39.487	1	7.4226	3.2639	1	38	28/104	5.289191	3580	3020.49	MS2	1007.5	105	Glioma_grade 2_6.raw
5453549	NP_006397.1	PRDX4	peroxiredoxin-4 precursor [Homo sapiens]	4	8	2.424170971	25.52248148	62.264608	28.78	7.32E-05	271	30.521	6.2905	Enzyme: Peroxidase	Metabolism,Energy pathways	Peroxidase activity	Cytoplasm	Mitochondrion, Nucleus, Golgi apparatus, Endoplasmic reticulum																								
5453549			lVQAFQYTDk	Unambiguous	N-Term(iTRAQ4plex); K10(iTRAQ4plex)	HCD	2.609941	0	1	1	2.33119	2.7618	2.4817	2.9401834	10.4146836	216217.83	182503.78	504045.3	536594.56	11.95793395	4.423394366	2	High	3928	Unique	Used	51475	1	40.545	1	4.0036	3.2857	1	16	15/18	15.06305	3928	1500.84	MS2	750.921	234	Glioma_grade 2_2.raw
5453549			vSVADHSLHLSk	Unambiguous	N-Term(iTRAQ4plex); K12(iTRAQ4plex)	HCD	1.9185497	0	1	1	1.87347	2.0796	1.7735	1.9685848	6.804606474	133282.53	120072.27	249701.31	236372.45	7.373899707	3.877982342	3	High	1629	Unique	Used	89499	1	23.622	1	5.1234	-3.2955	1	6	26/44	28.51209	1629	1580.89	MS2	527.636	167	Glioma_grade 2_7rep.raw
5453549			vSVADHSLHLSk	Unambiguous	N-Term(iTRAQ4plex); K12(iTRAQ4plex)	HCD	2.2384012	0	1	1	2.26094	2.0314	2.4688	2.218154	7.996046371	72665.91	80875.68	164292.94	179394.7	7.561708901	6.214105869	2	High	1643	Unique	Used	89512	1	23.727	1	5.716	-0.8161	1	40	19/22	43.89094	1643	1580.9	MS2	790.953	220	Glioma_grade 2_7rep.raw
5453549			lVQAFQYTDk	Unambiguous	N-Term(iTRAQ4plex); K10(iTRAQ4plex)	HCD	3.2759423	0	1	1	3.08118	3.2843	3.2681	3.4835463	5.014441898	70419.73	66064.445	216975.72	230138.53	4.512833165	4.163370866	2	High	3810	Unique	Used	59392	1	40.079	1	4.514	0.6016	1	100	15/18	47.21559	3810	1500.83	MS2	750.919	260	Glioma_grade 2_3.raw
5453549			iPLLSDLTHQISk	Unambiguous	N-Term(iTRAQ4plex); K13(iTRAQ4plex)	HCD	2.0567882	0	1	1	1.90066	2.1974	1.9352	2.2372892	8.425183642	62854.84	54367.473	119465.695	121635.76	10.23947507	1.272881308	2	High	5712	Unique	Used	82674	1	58.422	1	4.2844	0.5302	1	64	15/24	50.25523	5712	1753.05	MS2	877.027	102	Glioma_grade 2_6.raw
5453549			dYGVYLEDSGHTLR	Unambiguous	N-Term(iTRAQ4plex)	HCD	1.6989753	0	1	1	1.36337	1.9739	1.5091	2.1848645	21.94748546	53249.59	36779.594	72598.94	80358.44	25.87171257	7.174279618	3	High	3802	Unique	Used	51356	1	39.64	1	5.8469	1.8941	1	10	26/52	29.31518	3802	1768.87	MS2	590.293	150	Glioma_grade 2_2.raw
5453549			vSVADHSLHLSk	Unambiguous	N-Term(iTRAQ4plex); K12(iTRAQ4plex)	HCD	3.4734087	0	1	1	3.43315	3.2182	3.7456	3.5111444	6.261336521	17606.89	18782.627	60447.145	65948.516	4.569291786	6.155364368	4	High	1631	Unique	Used	89501	1	23.633	1	5.5054	-2.8407	1	13	26/66	10.5642	1631	1580.9	MS2	395.979	225	Glioma_grade 2_7rep.raw
5453549			iPLLSDLTHQISk	Unambiguous	N-Term(iTRAQ4plex); K13(iTRAQ4plex)	HCD	2.6330228	0	1	1	2.6216	2.6232	2.6428	2.644454	0.467027726	17583.426	17572.625	46096.688	46470	0.043448909	0.570337888	3	High	5655	Unique	Used	82626	1	57.908	1	5.1263	0.1047	1	43	28/48	17.69511	5655	1753.05	MS2	585.02	90	Glioma_grade 2_6.raw
5453990	NP_006254.1	PSME1	proteasome activator complex subunit 1 isoform 1 [Homo sapiens]	8	12	2.03614831	24.88487926	65.098485	36.55	1.73E-04	249	28.705	6.0239	Ubiquitin proteasome system protein	Protein metabolism	Ubiquitin-specific protease activity	Nucleus	Cytoplasm																								
5453990			iSELDAFLk	Unambiguous	N-Term(iTRAQ4plex); K9(iTRAQ4plex)	HCD	2.0574722	0	1	2	1.98332	2.1291	1.9908	2.137075	4.098202337	105574.14	98346.81	209387.16	210174.53	5.012230526	0.265398238	2	High	6256	Unique	Used	61663	1	59.155	1	4.468	1.3102	1	43	15/16	48.63406	6256	1323.78	MS2	662.393	280	Glioma_grade 2_3.raw
5453990			nAYAVLYDIILk	Unambiguous	N-Term(iTRAQ4plex); K12(iTRAQ4plex)	HCD	3.6673582	0	1	1	3.45407	3.5687	3.7628	3.8877223	5.288274634	74000.74	71623.78	255603.9	278453.34	2.308353751	6.050660027	2	High	8580	Unique	Used	55799	1	75.923	1	5.2526	1.9334	1	38	19/22	15.60123	8580	1684	MS2	842.501	119	Glioma_grade 2_2.raw
5453990			lEGFHTQISk	Unambiguous	N-Term(iTRAQ4plex); K10(iTRAQ4plex)	HCD	2.0148244	0	1	2	1.88689	2.089	1.9478	2.156466	6.150311145	68928.47	62260.266	130060.11	134262.14	7.18831877	2.248228371	2	High	1781	Unique	Used	79241	1	25.027	1	4.2671	3.8629	1	69	17/18	12.21027	1781	1447.82	MS2	724.414	315	Glioma_grade 2_6.raw
5453990			aPLDIPVPDPVk	Unambiguous	N-Term(iTRAQ4plex); K12(iTRAQ4plex)	HCD	1.7028022	0	1	2	1.70018	1.6732	1.7329	1.705385	1.437878306	61407.25	62398.598	104403.234	106413.63	1.132400294	1.348625169	2	High	5415	Unique	Used	68550	1	51.982	1	3.4572	2.5093	1	100	16/22	67.0661	5415	1548.93	MS2	774.968	174	Glioma_grade 2_4.raw
5453990			iEDGNNFGVAVQEk	Unambiguous	N-Term(iTRAQ4plex); K14(iTRAQ4plex)	HCD	1.7428762	0	1	2	1.75121	1.7241	1.762	1.734676	0.96878489	49760.348	50543.625	87140.586	87676.82	1.104363988	0.433795131	2	High	3320	Unique	Used	50902	1	36.154	1	6.2755	4.0519	1	80	21/26	15.63811	3320	1807.95	MS2	904.479	212	Glioma_grade 2_2.raw
5453990			aPLDIPVPDPVk	Unambiguous	N-Term(iTRAQ4plex); K12(iTRAQ4plex)	HCD	1.9743015	0	1	2	1.80796	2.0289	1.9256	2.1609697	7.583160851	46105.26	41084.395	83356.54	88782.13	8.143827817	4.457419685	2	High	5293	Unique	Used	60771	1	51.595	1	3.9226	1.5636	1	70	22-Nov	45.76968	5293	1548.93	MS2	774.967	194	Glioma_grade 2_3.raw
5453990			tENLLGSYFPk	Unambiguous	N-Term(iTRAQ4plex); K11(iTRAQ4plex)	HCD	2.2209902	0	1	2	2.02453	2.2175	2.2242	2.4361794	7.556152346	40181.418	36685.14	81348.29	89371.58	6.432555189	6.646353194	2	High	5730	Unique	Used	53171	1	53.724	1	4.7249	1.929	1	82	19/20	38.87801	5730	1556.86	MS2	778.933	225	Glioma_grade 2_2.raw
5453990			qLVHELDEAEYR	Unambiguous	N-Term(iTRAQ4plex)	HCD	2.2909973	0	1	2	2.09591	2.3429	2.2446	2.509074	7.547623332	37539.9	33582.94	78680.16	84262.086	7.868058275	4.844683099	2	High	2651	Unique	Used	58314	1	31.218	1	4.3081	0.2866	1	56	19/22	11.91174	2651	1645.83	MS2	823.419	186	Glioma_grade 2_3.raw

5453990			vFELMTSLHTk	Unambiguous	N-Term(iTRAQ4plex); K11(iTRAQ4plex)	HCD	2.822902	0	1	2	2.7474	2.7648	2.8807	2.898878	2.758024928	23697.555	23548.576	65106.75	68264.45	0.445937303	3.348295708	3	High	4661	Unique	Used	81775	1	49.209	1	4.9883	1.7315	1	28	25/40	28.77017	4661	1593.89	MS2	531.97	192	Glioma_grade2_6.raw
5453990			qLVHELDEAEYR	Unambiguous	N-Term(iTRAQ4plex)	HCD	2.357807	0	1	2	2.36221	2.2958	2.4216	2.3535235	2.181900547	22581.393	23234.277	53342.086	54682.418	2.015287362	1.754708999	2	High	2966	Unique	Used	66285	1	32.837	1	4.655	-0.01	1	100	18/22	0	2966	1645.83	MS2	823.419	184	Glioma_grade2_4.raw
5453990			qLVHELDEAEYR	Unambiguous	N-Term(iTRAQ4plex)	HCD	1.8666513	0	1	2	1.79739	2.0562	1.7009	1.9458876	8.386967119	20908.57	18276.492	37580.848	35564	9.499335244	3.899459597	3	High	2975	Unique	Used	66293	1	32.914	1	3.8728	-3.3188	1	22	21/44	34.3807	2975	1645.82	MS2	549.28	133	Glioma_grade2_4.raw
5453990			qLVHELDEAEYR	Unambiguous	N-Term(iTRAQ4plex)	HCD	1.8798841	0	1	2	1.77796	1.9857	1.7852	1.9937116	6.376497888	18910.977	16932.934	33623.01	33759.387	7.804324806	0.286226391	2	High	3054	Unique	Used	66366	1	33.524	1	4.0347	-0.7517	1	100	17/22	0	3054	1645.83	MS2	823.418	184	Glioma_grade2_4.raw
5454088	NP_006392.1	ANP32B	acidic leucine-rich nuclear phosphoprotein 32 family member B [Homo sapiens]	2	2	2.919318199	16.93053122	8.9303012	9.56	1.19E-04	251	28.77	4.0562	Unclassified	Biological_process unknown	Molecular function unknown	Nucleus	Cytoplasm, Nucleolus																								
5454088			lPNLTHLNLSGNk	Unambiguous	N-Term(iTRAQ4plex); K13(iTRAQ4plex)	HCD	2.5698264	0	1	1	2.42341	2.9876	2.2309	2.7503288	12.96213572	30995.684	25142.148	75115.31	69149.18	14.74611631	5.848550784	3	High	3812	Unique	Used	81063	1	41.429	1	4.4778	-1.1552	1	29	18/48	22.93583	3812	1708.99	MS2	570.336	159	Glioma_grade2_6.raw
5454088			iFGGLDMLAEk	Unambiguous	N-Term(iTRAQ4plex); K11(iTRAQ4plex)	HCD	3.26881	0	1	1	3.1629	3.2527	3.2845	3.3777256	2.711719411	25979.191	25261.793	82169.63	85327.41	1.979965844	2.666181625	2	High	6677	Unique	Used	69703	1	62.123	1	4.4525	0.9109	1	100	19/20	9.186368	6677	1481.83	MS2	741.418	298	Glioma_grade2_4.raw
5729770	NP_000382.3	TPP1	tripeptidyl-peptidase 1 preproprotein [Homo sapiens]	4	10	2.222961903	14.47404572	58.40701	12.26	1.75E-04	563	61.21	6.481	Serine protease	Protein metabolism	Serine-type peptidase activity	Lysosome	Nucleus																								
5729770			lFGGNFAHQASVAR	Unambiguous	N-Term(iTRAQ4plex)	HCD	2.374877	0	1	1	2.15691	2.3843	2.3663	2.6158175	7.879465867	130898.47	118414.71	282336.8	309751.28	7.081335492	6.548000328	2	High	2491	Unique	Used	73166	1	30.634	1	4.8732	13.12	1	22	22/26	22.50154	2491	1618.88	MS2	809.943	197	Glioma_grade2_5.raw
5729770			lYQQHGAGLFDVTR	Unambiguous	N-Term(iTRAQ4plex)	HCD	2.3367531	0	1	1	1.91374	2.3327	2.3401	2.852375	16.27955324	108055.766	88647.766	206790.94	252856.67	13.95351499	14.1732011	3	High	3346	Unique	Used	73959	1	37.078	1	4.0984	0.6595	1	4	22/52	11.36624	3346	1748.92	MS2	583.645	160	Glioma_grade2_5.raw
5729770			lYQQHGAGLFDVTR	Unambiguous	N-Term(iTRAQ4plex)	HCD	2.101276	0	1	1	1.78019	2.2881	1.9559	2.5139737	15.42004147	104628.79	81403.63	186258.86	204646.6	17.65570553	6.65229677	2	High	3366	Unique	Used	73978	1	37.219	1	5.1353	1.6095	1	21	22/26	22.85567	3366	1748.92	MS2	874.965	295	Glioma_grade2_5.raw
5729770			lYQQHGAGLFDVTR	Unambiguous	N-Term(iTRAQ4plex)	HCD	1.9462365	0	1	1	1.70071	2.0706	1.8441	2.2451599	12.25875018	100990.4	82950.484	171755.36	186237.1	13.86983324	5.72086717	3	High	3944	Unique	Used	67190	1	40.488	1	4.1419	-0.2827	1	5	21/52	8.083447	3944	1748.92	MS2	583.645	184	Glioma_grade2_4.raw
5729770			lFGGNFAHQASVAR	Unambiguous	N-Term(iTRAQ4plex)	HCD	2.273958	0	1	1	2.2975	2.258	2.2902	2.2508192	1.018725484	100969.21	102737.15	231976.94	231242.77	1.22737686	0.224142701	3	High	2489	Unique	Used	73164	1	30.623	1	4.4908	11.716	1	5	19/52	40.15115	2489	1618.88	MS2	540.297	190	Glioma_grade2_5.raw
5729770			vPIPWVSGTSASTPVFGGILSLINEHR	Unambiguous	N-Term(iTRAQ4plex)	HCD	2.9221106	0	1	1	2.74291	3.0374	2.818	3.1205544	6.086368049	93528.14	84460.5	256538.86	263563.6	7.204717934	1.910101056	3	High	7497	Unique	Used	84071	1	76.401	1	8.9128	0.7072	1	2	35/104	6.203377	7497	2978.62	MS2	993.546	92	Glioma_grade2_6.raw
5729770			iLSGRPPLGFLNPR	Unambiguous	N-Term(iTRAQ4plex)	HCD	1.7077639	0	1	1	1.7131	1.6213	1.7991	1.702712	4.253682409	40405.668	42692.48	69219.016	72693	3.891832276	3.461972726	2	High	4912	Unique	Used	81996	1	51.295	1	3.9612	-2.4422	1	39	15/26	10.79553	4912	1681	MS2	841.003	103	Glioma_grade2_6.raw
5729770			lYQQHGAGLFDVTR	Unambiguous	N-Term(iTRAQ4plex)	HCD	2.1719656	0	1	1	2.10929	2.2888	2.0643	2.2399788	4.873446261	33761.902	31114.568	71213.49	69695.97	5.770806653	1.52303285	2	High	3934	Unique	Used	67181	1	40.398	1	5.3932	3.0054	1	81	23/26	5.722895	3934	1748.93	MS2	874.966	240	Glioma_grade2_4.raw
5729770			iLSGRPPLGFLNPR	Unambiguous	N-Term(iTRAQ4plex)	HCD	2.0386071	0	1	1	1.83936	1.9371	2.135	2.24844	9.097124246	32888.266	31228.977	60493.387	70216.484	3.659840782	10.51989076	3	High	4879	Unique	Used	81966	1	51.04	1	3.3271	-0.8889	1	69	21/52	37.95393	4879	1681	MS2	561.005	81	Glioma_grade2_6.raw
5729770			vPIPWVSGTSASTPVFGGILSLINEHR	Unambiguous	N-Term(iTRAQ4plex)	HCD	2.3065202	0	1	1	2.30889	1.9995	2.6611	2.3044708	11.66416613	9956.509	11497.14	22988.453	26494.822	10.1557607	10.02107196	3	High	7928	Unique	Used	77946	1	76.491	1	8.244	-2.7353	1	15	32/104	18.85448	7928	2978.61	MS2	993.543	76	Glioma_grade2_5.raw
5729997	NP_004154.2	RAB27B	ras-related protein Rab-27B [Homo sapiens]	2	6	0.278001666	17.73723908	28.060048	10.09	1.00E-04	218	24.592	5.5161	GTPase	Cell communication,Signal transduction	GTPase activity	Plasma membrane	Cytoplasm, Zymogen granule																								
5729997			lLALGDSGVGk	Unambiguous	N-Term(iTRAQ4plex); K11(iTRAQ4plex)	HCD	0.31854686	0	1	1	0.29041	0.3203	0.317	0.34958017	0.075811348	371200.03	336585.9	107799.24	117663.76	6.916183272	6.187506584	2	High	3735	Unique	Used	59322	1	39.503	1	4.13	9.4058	1	34	19/20	57.84973	3735	1317.81	MS2	659.409	167	Glioma_grade2_3.raw
5729997			lLALGDSGVGk	Unambiguous	N-Term(iTRAQ4plex); K11(iTRAQ4plex)	HCD	0.4000841	0	1	1	0.36219	0.4237	0.3799	0.4444146	0.094474919	283708.66	242543.7	102755.25	107789.95	11.06238169	3.381763641	2	High	3750	Unique	Used	59336	1	39.613	1	4.2647	-2.5436	1	43	18/20	28.59948	3750	1317.79	MS2	659.401	224	Glioma_grade2_3.raw
5729997			aVETLLDLIMk	Unambiguous	N-Term(iTRAQ4plex); K11(iTRAQ4plex)	HCD	0.2596807	0	1	1	0.22899	0.2502	0.2684	0.29321083	0.104790273	208457.38	190788.84	47735.18	55941.36	6.258566179	11.19374841	2	High	8408	Unique	Used	63617	1	76.815	1	5.1395	2.5884	1	22	19/20	22.81924	8408	1533.92	MS2	767.464	160	Glioma_grade2_3.raw
5729997			aVETLLDLIMk	Unambiguous	N-Term(iTRAQ4plex); K11(iTRAQ4plex)	HCD	0.26587954	0	1	1	0.23839	0.2619	0.2695	0.29607427	0.089205325	130425.055	118727.016	31092.424	35152.016	6.639930923	8.66658404	2	High	8714	Unique	Used	55926	1	76.85	1	5.2553	3.2251	1	38	19/20	11.34696	8714	1533.92	MS2	767.464	158	Glioma_grade2_2.raw
5729997			aVETLLDLImk	Unambiguous	N-Term(iTRAQ4plex); M10(Oxidation); K11(iTRAQ4plex)	HCD	0.27342018	0	1	1	0.25172	0.2611	0.2853	0.29593232	0.075183082	122919.94	118485.91	30941.42	35063.81	2.597561477	8.832542281	2	High	7867	Unique	Used	63134	1	72.275	1	4.5654	5.1337	1	43	19/20	18.91139	7867	1549.92	MS2	775.463	180	Glioma_grade2_3.raw
5729997			aVETLLDLImk	Unambiguous	N-Term(iTRAQ4plex); M10(Oxidation); K11(iTRAQ4plex)	HCD	0.28258312	0	1	1	0.26336	0.2798	0.2852	0.3030084	0.057762568	60328.023	56777.625	15887.982	17204.098	4.287599351	5.624515282	2	High	8242	Unique	Used	55491	1	73.165	1	4.7051	5.37	1	100	19/20	11.63882	8242	1549.92	MS2	775.464	183	Glioma_grade2_2.raw
5803011	NP_001966.1	ENO2	gamma-enolase [Homo sapiens]	6	15	0.465863615	25.91994765	203.99486	41.47	2.14E-04	434	47.239	5.0337	Enzyme: Hydratase	Metabolism,Energy pathways	Catalytic activity	Cytoplasm	-																								
5803011			lGAEVYHTLk	Unambiguous	N-Term(iTRAQ4plex); K10(iTRAQ4plex)	HCD	0.46997556	0	1	1	0.45483	0.4292	0.5132	0.48426938	0.077246705	1366906.6	1448485.6	621708.4	701457.25	4.097835044	8.523642165	2	High	2286	Unique	Used	79711	1	28.803	1	4.9248	4.4127	1	7	17/18	20.95214	2286	1418.83	MS2	709.919	226	Glioma_grade2_6.raw
5803011			lGAEVYHTLk	Unambiguous	N-Term(iTRAQ4plex); K10(iTRAQ4plex)	HCD	0.5549807	0	1	1	0.57771	0.5854	0.525	0.53195065	0.055746929	339391.8	334940.72	196069.61	178171.94	0.933482743	6.763313066	3	High	2277	Unique	Used	79703	1	28.726	1	3.9183	2.8115	1	14	17/36	22.00593	2277	1418.83	MS2	473.614	221	Glioma_grade2_6.raw
5803011			vNQIGSVTEAIQAck	Unambiguous	N-Term(iTRAQ4plex); C14(Methylthio); K15(iTRAQ4plex)	HCD	0.24211074	0	1	1	0.21303	0.2545	0.2318	0.27685294	0.113413303	254405.83	212975.08	54195.164	58962.777	12.5362263	5.958417858	2	High	6622	Unique	Used	54012	1	60.232	1	5.5731	0.288	1	14	24/28	12.62485	6622	1895	MS2	948.002	174	Glioma_grade2_2.raw
5803011			lGAEVYHTLk	Unambiguous	N-Term(iTRAQ4plex); K10(iTRAQ4plex)	HCD	0.5624376	0	1	1	0.47747	0.6223	0.5165	0.67317796	0.158780823	232926.48	178723.62	111214.69	120312.81	18.62125619	5.557300063	2	High	2382	Unique	Used	73064	1	29.835	1	3.911	13.36	1	39	18/18	11.76828	2382	1418.84	MS2	709.925	204	Glioma_grade2_5.raw
5803011			lDNLMLELDGTENk	Unambiguous	N-Term(iTRAQ4plex); K14(iTRAQ4plex)	HCD	0.36315703	0	1	1	0.36192	0.3461	0.381	0.36434197	0.039234137	196179.58	205127.4	71000.93	74736.52	3.153229081	3.624958473	2	High	6733	Unique	Used	54113	1	61.113	1	5.3577	-0.0575	1	21	22/26	41.70315	6733	1892.99	MS2	946.998	184	Glioma_grade2_2.raw
5803011			lGAEVYHTLk	Unambiguous	N-Term(iTRAQ4plex); K10(iTRAQ4plex)	HCD	0.4879089	0	1	1	0.41614	0.4836	0.4917	0.57130694	0.129480827	174986.62	150589.25	72818.69	86032.69	10.59755796	11.76408918	2	High	2760	Unique	Used	85937	1	38.301	1	4.8949	-4.1049	1	89	15/18	43.25015	2760	1418.82	MS2	709.913	270	Glioma_grade2_1rep.raw
5803011			lGAEVYHTLk	Unambiguous	N-Term(iTRAQ4plex); K10(iTRAQ4plex)	HCD	0.441991	0	1	1	0.38285	0.395	0.4876	0.50300485	0.140103406	148989.55	144418.72	57040.5	72643.31	2.203117784	17.01500404	3	High	2755	Unique	Used	85933	1	38.255	1	3.6822	-1.6408	1	23	16/36	42.91198	2755	1418.82	MS2	473.612	237	Glioma_grade2_1rep.raw
5803011			yITGDQLGALYQDFVR	Unambiguous	N-Term(iTRAQ4plex)	HCD	0.46586362	0	1	1	0.42719	0.4391	0.4919	0.505615	0.082806062	101235.96	98483.05	43247.312	49794.504	1.949340054	9.951576743	2	High	5753	Unique	Used	87961	1	73.767	1	5.2096	-0.3988	1	56	26/30	17.02817	5753	2003.03	MS2	1002.02	201	Glioma_grade2_1rep.raw
5803011			vNQIGSVTEAIQAck	Unambiguous	N-Term(iTRAQ4plex); C14(Methylthio); K15(iTRAQ4plex)	HCD	0.26359782	0	1	1	0.18087	0.2627	0.2642	0.38375282	0.306144262	77834.6	53590.81	14077.826	20565.625	26.08772278	26.48446697	3	High	6597	Unique	Used	53989	1	60.035	1	6.293	-0.3954	1	6	27/56	28.92281	6597	1895	MS2	632.337	162	Glioma_grade2_2.raw
5803011			lDNLMLELDGTENk	Unambiguous	N-Term(iTRAQ4plex); K14(iTRAQ4plex)	HCD	0.32187647	0	1	1	0.32155	0.3073	0.3372	0.322193	0.037903788	73783.96	77211.52	23724.879	24877.012	3.210229762	3.3524665	3	High	6732	Unique	Used	54112	1	61.107	1	5.1291	0.806	1	8	23/52	7.166037	6732	1892.99	MS2	631.668	179	Glioma_grade2_2.raw
5803011			acNcLLLk	Unambiguous	N-Term(iTRAQ4plex); C2(Methylthio); C4(Methylthio); K8(iTRAQ4plex)	HCD	0.31913868	0	1	3	0.30462	0.3373	0.3027	0.33521885	0.058964308	61273.016	55330.086	18664.873	18547.688	7.207846157	0.445345904	2	High	6293	Unique	Used	69356	1	58.952	1	3.3868	-2.5454	1	95	14/14	40.47897	6293	1257.64	MS2	629.324	188	Glioma_grade2_4.raw
5803011			lGAEVYHTLk	Unambiguous	N-Term(iTRAQ4plex); K10(iTRAQ4plex)	HCD	0.46059236	0	1	1	0.44038	0.4465	0.4745	0.48108926	0.043786399	58188.355	57389.8	25624.799	27609.617	0.977111385	5.272823007	3	High	2910	Unique	Used	95428	1	40.798	1	4.0363	6.2315	1	84	17/36	48.01344	2910	1418.83	MS2	473.616	228	Glioma_grade2_8rep.raw
5803011			yITGDQLGALYQDFVR	Unambiguous	N-Term(iTRAQ4plex)	HCD	0.57769996	0	1	1	0.63178	0.6806	0.4821	0.51943463	0.161062042	7576.146	7032.3	4786.4785	3652.8198	5.264861088	18.99726081	3	High	5762	Unique	Used	87969	1	73.843	1	5.6493	3.4034	1	60	26/60	15.82385	5762	2003.04	MS2	668.352	200	Glioma_grade2_1rep.raw
5803011			aVDHINSTIAPALISSGLSVVEQEk	Unambiguous	N-Term(iTRAQ4plex); K25(iTRAQ4plex)	HCD	0.4912447	0	1	1	0.55879	0.5149	0.4656	0.42900756	0.115254439	5188.652	5631.23	2899.3694	2415.8403	5.784719371	12.86521981	2	High	6966	Unique	Used	77152	1	67.433	1	5.3952	-3.681	1	56	24/48	0	6966	2866.57	MS2	1433.79	74	Glioma_grade2_5.raw
5803011			aVDHINSTIAPALISSGLSVVEQEk	Unambiguous	N-Term(iTRAQ4plex); K25(iTRAQ4plex)	HCD	0.59590954	0	1	1	0.53305	0.5617	0.6284	0.66214967	0.099517899	2981.842	2829.8145	1589.461	1873.7606	3.699450447	11.60943181	3	High	6840	Unique	Used	69851	1	63.471	1	3.9951	-1.5352	1	100	20/96	10.30014	6840	2866.57	MS2	956.196	100	Glioma_grade2_4.raw
5803023	NP_006807.1	LMAN2	vesicular integral-membrane protein VIP36 precursor [Homo sapiens]	4	8	2.369429111	16.48156489	78.50899	31.18	6.25E-05	356	40.203	6.9468	Transport/cargo protein	Transport	Transporter activity	Golgi apparatus	Mitochondrion, Cytoplasm																								
5803023			iEPSVNFLk	Unambiguous	N-Term(iTRAQ4plex); K9(iTRAQ4plex)	HCD	2.2045748	0	1	1	2.00347	2.3051	2.1172	2.4359627	8.692288274	347967.72	302434.7	697141.1	736719.6	9.900549635	3.903618495	2	High	4726	Unique	Used	52225	1	46.447	1	3.6407	1.3078	1	12	16/16	18.09662	4726	1334.79	MS2	667.901	246	Glioma_grade2_2.raw
5803023			lVPGPVFGSk	Unambiguous	N-Term(iTRAQ4plex); K10(iTRAQ4plex)	HCD	2.296323	0	1	1	2.21145	2.233	2.3591	2.3820179	3.771210167	272486.44	269862.03	602591.06	642816.25	0.684334228	4.567743323	2	High	4066	Unique	Used	67304	1	41.409	1	3.2675	0.6735	1	13	18-Dec	10.04602	4066	1288.79	MS2	644.897	179	Glioma_grade2_4.raw
5803023			iEPSVNFLk	Unambiguous	N-Term(iTRAQ4plex); K9(iTRAQ4plex)	HCD	2.4425352	0	1	1	2.40673	2.5383	2.3517	2.4802938	3.35092087	128780.164	122103.266	309939.6	302851.97	3.763723936	1.635698492	2	High	4559	Unique	Used	60087	1	45.918	1	3.9788	0.4847	1	36	16/16	22.33747	4559	1334.79	MS2	667.9	250	Glioma_grade2_3.raw
5803023			lVPGPVFGSk	Unambiguous	N-Term(iTRAQ4plex); K10(iTRAQ4plex)	HCD	1.7952595	0	1	1	1.55729	1.9261	1.6894	2.0895865	13.10826648	107134.016	86618.36	166839.23	180996.55	14.97453583	5.756013355	2	High	3830	Unique	Used	59411	1	40.226	1	3.1645	-0.5579	1	48	18-Dec	33.05913	3830	1288.79	MS2	644.897	184	Glioma_grade2_3.raw
5803023			lPTGYYFGASAGTGDLSDNHDIISMk	Unambiguous	N-Term(iTRAQ4plex); K26(iTRAQ4plex)	HCD	2.5552042	0	1	1	2.51796	2.4081	2.709	2.5908191	4.94558983	13640.8955	14263.307	34347.28	36953.65	3.15444523	5.169587273	3	High	5947	Unique	Used	76270	1	58.555	1	8.6345	-1.619	1	23	34/100	6.476918	5947	3018.47	MS2	1006.83	80	Glioma_grade2_5.raw
5803023			lPTGYYFGASAGTGDLSDNHDIISMk	Unambiguous	N-Term(iTRAQ4plex); K26(iTRAQ4plex)	HCD	2.8069434	0	1	1	2.83534	2.5009	3.154	2.781893	9.500876231	9719.872	11019.861	27559.18	30656.076	8.864444276	7.523238109	3	High	6372	Unique	Used	69428	1	59.583	1	8.6611	-0.8304	1	33	36/100	12.21964	6372	3018.47	MS2	1006.83	82	Glioma_grade2_4.raw
5803023			lFQLMVEHTPDEESIDWTk	Unambiguous	N-Term(iTRAQ4plex); K19(iTRAQ4plex)	HCD	1.7260127	0	1	1	1.7428	1.5768	1.8909	1.7108203	7.452454292	7766.1064	8583.599	13534.804	14684.995	7.071131215	5.764093896	3	High	6648	Unique	Used	76885	1	64.508	1	6.1032	-2.2045	1	41	31/72	15.53601	6648	2606.3	MS2	869.438	115	Glioma_grade2_5.raw
5803023			lPTGYYFGASAGTGDLSDNHDIISmk	Unambiguous	N-Term(iTRAQ4plex); M25(Oxidation); K26(iTRAQ4plex)	HCD	2.5626283	0	1	1	2.53989	2.6167	2.5101	2.5860565	1.849574946	5946.897	5772.333	15104.452	14927.579	2.10654434	0.832898033	3	High	5833	Unique	Used	68931	1	55.316	1	8.8876	0.6486	1	72	32/100	5.683687	5833	3034.47	MS2	1012.16	67	Glioma_grade2_4.raw
5902090	NP_008862.1	SLC2A3	solute carrier family 2, facilitated glucose transporter member 3 [Homo sapiens]	3	3	0.317240238	35.50887242	19.888799	5.85	6.25E-05	496	53.889	7.1958	Membrane transport protein	Transport	Auxiliary transport protein activity	Plasma membrane	-																								
5902090			lWGTQDVSQDIQEMk	Unambiguous	N-Term(iTRAQ4plex); K15(iTRAQ4plex)	HCD	0.31724024	0	1	2	0.30803	0.3309	0.3045	0.3271322	0.041835791	281420.56	261979.23	86686.445	85701.84	5.059661977	0.807735714	2	High	6484	Unique	Used	53882	1	59.228	1	7.5728	-2.1555	1	51	20/28	9.99294	6484	2066.04	MS2	1033.52	172	Glioma_grade2_2.raw
5902090			qVTVLELFR	Unambiguous	N-Term(iTRAQ4plex)	HCD	0.32321957	0	1	2	0.30995	0.3208	0.3255	0.33695364	0.034607855	123576.664	119383.71	38302.77	40226.773	2.440617091	3.464875807	2	High	4767	Unique	Used	87231	1	62.915	1	3.9123	0.3328	1	71	14/16	20.6504	4767	1248.74	MS2	624.876	299	Glioma_grade2_1rep.raw
5902090			lWGTQDVSQDIQEMkDESAR	Unambiguous	N-Term(iTRAQ4plex); K15(iTRAQ4plex)	HCD	0.15685417	1	1	2	0.14954	0.1319	0.1852	0.16330358	0.142960164	68040.48	77157.9	10174.808	12600.161	8.880249916	15.06024929	3	High	6190	Unique	Used	76484	1	60.59	1	4.716	0.642	1	20	29/76	8.273731	6190	2624.29	MS2	875.434	75	Glioma_grade2_5.raw
6005721	NP_009106.1	ERLIN2	erlin-2 isoform 1 [Homo sapiens]	5	7	2.252240181	15.41489391	52.78867	28.61	1.00E-04	339	37.815	5.6177	Unclassified	Biological_process unknown	Molecular function unknown	Endoplasmic reticulum lumen	-																								
6005721			vTkPNIPEAIR	Unambiguous	N-Term(iTRAQ4plex); K3(iTRAQ4plex)	HCD	2.1783202	0	1	1	2.27756	2.2466	2.1091	2.0804276	4.504023639	115060.766	116647.48	262058.1	242676.64	0.968438757	5.430480888	3	High	1952	Unique	Used	79401	1	26.295	1	3.9407	2.7981	1	8	19/40	18.46378	1952	1525.93	MS2	509.316	83	Glioma_grade2_6.raw
6005721			nYELMESEk	Unambiguous	N-Term(iTRAQ4plex); K9(iTRAQ4plex)	HCD	2.2522402	0	1	1	2.26919	2.3859	2.1251	2.2344155	4.766096995	104518.34	99406.67	237172.3	222115.81	3.544927148	4.636107902	2	High	1857	Unique	Used	49523	1	25.781	1	3.4442	-0.6113	1	23	15/16	17.06718	1857	1430.71	MS2	715.857	127	Glioma_grade2_2.raw
6005721			iSEIEDAAFLAR	Unambiguous	N-Term(iTRAQ4plex)	HCD	2.3713899	0	1	2	2.34763	2.3457	2.3971	2.3951335	1.205489034	52721.277	52765.05	123769.88	126379.34	0.058684734	1.475250702	2	High	4350	Unique	Used	86930	1	58.302	1	4.2439	-0.4314	1	60	20/22	39.81702	4350	1478.8	MS2	739.902	269	Glioma_grade2_1rep.raw
6005721			lTPEYLQLMk	Unambiguous	N-Term(iTRAQ4plex); K10(iTRAQ4plex)	HCD	2.5013745	0	1	1	2.32544	2.5153	2.4885	2.691677	5.986149692	44263.04	40921.51	102930.97	110147.5	5.547528331	4.789650779	2	High	5974	Unique	Used	53401	1	55.521	1	4.2638	-2.7485	1	83	16/18	30.61045	5974	1523.87	MS2	762.439	254	Glioma_grade2_2.raw
6005721			vTkPNIPEAIR	Unambiguous	N-Term(iTRAQ4plex); K3(iTRAQ4plex)	HCD	2.6865084	0	1	1	2.41568	2.7646	2.6182	2.9964643	9.066024029	43396.99	37919.152	104833.1	113623.38	9.526808087	5.690530761	2	High	1946	Unique	Used	79396	1	26.233	1	3.5797	2.067	1	96	14/20	23.1432	1946	1525.93	MS2	763.47	106	Glioma_grade2_6.raw
6005721			lSFGLEDEPLETATk	Unambiguous	N-Term(iTRAQ4plex); K15(iTRAQ4plex)	HCD	1.9461721	0	1	1	1.77802	2.1603	1.7699	2.1504815	11.21239101	17014.158	14003.154	30251.508	30113.523	13.72847103	0.323267056	2	High	4275	Unique	Used	86879	1	57.422	1	4.7512	3.0162	1	100	19/28	51.65438	4275	1938.04	MS2	969.522	240	Glioma_grade2_1rep.raw

6005721			lTPEYLQLMk	Unambiguous	N-Term(iTRAQ4plex); K10(iTRAQ4plex)	HCD	1.6623747	0	1	1	1.4275	1.6045	1.7138	1.9263766	12.51443973	5587.7026	4971.2046	7976.449	9576.412	8.257102901	12.8907155	3	High	5793	Unique	Used	61230	1	55.572	1	3.4092	0.0263	1	100	17/36	37.16258	5793	1523.87	MS2	508.63	232	Glioma_grade 2_3.raw
6466450	NP_009294.1	COMT	catechol O-methyltransferase isoform S-COMT [Homo sapiens]	8	10	2.093437672	27.7920853	106.54109	59.28	1.07E-04	221	24.433	5.3257	Enzyme: Acyltransferase	Metabolism,Energy pathways	Acyltransferase activity	Cytoplasm, Endoplasmic reticulum	-																								
6466450			mVDFAGVk	Unambiguous	N-Term(iTRAQ4plex); K8(iTRAQ4plex)	HCD	1.572337	0	1	2	1.50667	1.5731	1.5716	1.6409018	3.484284699	475152.7	455078.12	715897.9	746738.5	3.051903107	2.981957429	2	High	3184	Unique	Used	58810	1	35.26	1	2.9738	0.8489	1	13	14/14	46.76691	3184	1154.65	MS2	577.828	223	Glioma_grade 2_3.raw
6466450			eWAMNVGDk	Unambiguous	N-Term(iTRAQ4plex); K9(iTRAQ4plex)	HCD	1.784942	0	1	2	1.77706	1.6947	1.8796	1.7924583	4.2417173	436395.1	457606.03	775500.44	820239.7	3.355340823	3.964985694	2	High	2547	Unique	Used	58215	1	30.514	1	3.2073	-9.8035	1	12	15/16	36.41949	2547	1337.66	MS2	669.335	123	Glioma_grade 2_3.raw
6466450			yLPDTLLLEEcGLLR	Unambiguous	N-Term(iTRAQ4plex); C11(Methylthio)	HCD	2.6786788	0	1	2	2.54311	2.7218	2.6384	2.8237758	4.458100807	119775.914	111911.375	304603.2	316012.62	4.800495428	2.599894489	2	High	6083	Unique	Used	88230	1	76.906	1	4.8233	-0.2119	1	21	20/28	6.569861	6083	1938.02	MS2	969.513	194	Glioma_grade 2_1rep.raw
6466450			vTLVVGASQDIIPQLk	Unambiguous	N-Term(iTRAQ4plex); K16(iTRAQ4plex)	HCD	1.6923705	0	1	2	1.68027	1.6132	1.7748	1.7039853	3.939864581	25664.693	26731.83	43123.684	45550.645	2.880266727	3.87061419	2	High	6832	Unique	Used	54205	1	61.867	1	5.87	-1.5529	1	60	22/30	37.99858	6832	1969.19	MS2	985.099	46	Glioma_grade 2_2.raw
6466450			yDVDTLDMVFLDHWk	Unambiguous	N-Term(iTRAQ4plex); K15(iTRAQ4plex)	HCD	2.9228783	0	1	2	2.84333	2.6829	3.1772	2.99794	7.229759349	17847.748	18914.855	50747.01	56705.6	4.105033563	7.84226534	3	High	7304	Unique	Used	77438	1	70.519	1	7.4041	-1.1189	1	20	33/56	13.72242	7304	2185.09	MS2	729.033	113	Glioma_grade 2_5.raw
6466450			yDVDTLDmVFLDHWk	Unambiguous	N-Term(iTRAQ4plex); M8(Oxidation); K15(iTRAQ4plex)	HCD	2.232752	0	1	2	2.16612	2.245	2.221	2.3018115	2.518962062	13902.893	13414.558	30115.309	30877.783	2.528090853	1.767906883	3	High	7122	Unique	Used	77283	1	68.873	1	5.6034	0.0902	1	30	28/56	35.37189	7122	2201.08	MS2	734.366	125	Glioma_grade 2_5.raw
6466450			yDVDTLDMVFLDHWk	Unambiguous	N-Term(iTRAQ4plex); K15(iTRAQ4plex)	HCD	2.7414062	0	1	2	2.6936	2.616	2.8705	2.7878308	4.041430878	10970.305	11295.75	29549.66	31490.643	2.067042109	4.496970605	2	High	7316	Unique	Used	77448	1	70.634	1	6.5198	-2.5373	1	100	21/28	29.79538	7316	2185.08	MS2	1093.04	148	Glioma_grade 2_5.raw
6466450			iLNHVLQHAEPGNAQSVLEAIDTYcEQk	Unambiguous	N-Term(iTRAQ4plex); C25(Methylthio); K28(iTRAQ4plex)	HCD	3.5336196	0	1	2	2.71733	3.6311	3.4606	4.624423	21.75481652	6419.952	4804.306	17445.117	22217.145	20.35652143	17.01533492	3	High	6586	Unique	Used	93305	1	73.591	1	9.6721	1.4196	1	21	30/108	8.66202	6586	3454.74	MS2	1152.25	101	Glioma_grade 2_7rep.raw
6466450			gTVLLADNVIcPGAPDFLAHVR	Unambiguous	N-Term(iTRAQ4plex); C11(Methylthio)	HCD	1.9131162	0	1	2	2.22115	2.0691	1.7457	1.626171	14.44104794	686.04816	736.4689	1523.8162	1197.6244	5.012642473	16.9507601	4	High	7247	Unique	Used	77391	1	69.978	1	4.2755	-3.1639	1	77	24/126	15.97628	7247	2468.28	MS2	617.826	118	Glioma_grade 2_5.raw
6466450			kYDVDTLDMVFLDHWk	Unambiguous	N-Term(iTRAQ4plex); K1(iTRAQ4plex); K16(iTRAQ4plex)	HCD	1.9541235	1	1	2	2.32309	1.8418	2.0958	1.6615995	14.62192909	668.7595	843.51953	1553.5884	1401.5917	16.34275155	7.273864445	4	High	4955	Unique	Used	96626	1	67.913	1	5.1031	0.1623	1	100	30/90	33.23071	4955	2457.29	MS2	615.077	125	Glioma_grade 2_8rep.raw
6806898	NP_009292.1	SNCA	alpha-synuclein isoform NACP112 [Homo sapiens]	2	5	0.30733189	26.98253351	26.47375	25.89	1.19E-04	112	11.365	8.2944	Chaperone	Protein metabolism	Chaperone activity	Cytoplasm	Nucleus																								
6806898			eGVVHGVATVAEk	Unambiguous	N-Term(iTRAQ4plex); K13(iTRAQ4plex)	HCD	0.3073319	0	1	2	0.37924	0.3105	0.3035	0.24846275	0.172712479	2588622.2	3162151.2	981719.25	785676.75	14.10405929	15.68669174	3	High	1259	Unique	Used	72034	1	21.332	1	4.6696	1.9931	1	0	22/48	9.973667	1259	1583.9	MS2	528.639	172	Glioma_grade 2_5.raw
6806898			eGVVHGVATVAEk	Unambiguous	N-Term(iTRAQ4plex); K13(iTRAQ4plex)	HCD	0.280619	0	1	2	0.21664	0.2511	0.3061	0.3547706	0.21564388	1819249	1569739.9	394117.03	556897.56	10.41193003	24.20640394	3	High	1388	Unique	Used	64820	1	21.039	1	4.3413	1.8775	1	0	24/48	9.348939	1388	1583.9	MS2	528.639	188	Glioma_grade 2_4.raw
6806898			eGVVHGVATVAEk	Unambiguous	N-Term(iTRAQ4plex); K13(iTRAQ4plex)	HCD	0.21573447	0	1	2	0.20712	0.2411	0.1939	0.22576445	0.095431775	1577053.6	1354809.8	326636.12	305867.88	10.72015143	4.643563784	2	High	1389	Unique	Used	64821	1	21.044	1	4.4865	2.8493	1	3	21/24	15.60866	1389	1583.9	MS2	792.456	180	Glioma_grade 2_4.raw
6806898			tVEGAGSIAAATGFVk	Unambiguous	N-Term(iTRAQ4plex); K16(iTRAQ4plex)	HCD	0.3428598	0	1	2	0.32402	0.3327	0.3527	0.36220276	0.051300889	531389.6	517468.66	172182.77	187428.58	1.877010736	5.995592539	2	High	4862	Unique	Used	52354	1	47.403	1	6.9299	3.7509	1	18	24/30	8.884917	4862	1767	MS2	884.002	215	Glioma_grade 2_2.raw
6806898			tVEGAGSIAAATGFVk	Unambiguous	N-Term(iTRAQ4plex); K16(iTRAQ4plex)	HCD	0.4486093	0	1	2	0.41928	0.4268	0.47	0.47846562	0.066693949	145732.78	143163.45	61102.742	68498.79	1.257746192	8.070576966	2	High	4972	Unique	Used	52458	1	48.194	1	6.0465	2.3002	1	83	21/30	37.86326	4972	1766.99	MS2	884	221	Glioma_grade 2_2.raw
6996021	NP_000388.2	CYBB	cytochrome b-245 heavy chain [Homo sapiens]	3	4	2.20909071	15.56685008	35.539596	10.18	3.11E-04	570	65.294	8.6313	Enzyme: Oxidase	Metabolism,Energy pathways	Catalytic activity	Plasma membrane	-																								
6996021			gVHFIFNk	Unambiguous	N-Term(iTRAQ4plex); K8(iTRAQ4plex)	HCD	2.2298598	0	1	1	2.14247	2.3535	2.1173	2.325854	5.462969748	126069.945	114765.32	270101.53	266927.38	6.63821139	0.835881625	2	High	2620	Unique	Used	90357	1	32.057	1	3.907	-1.8859	1	26	14/14	28.76192	2620	1249.73	MS2	625.367	342	Glioma_grade 2_7rep.raw
6996021			iGVFLcGPEALAETLSk	Unambiguous	N-Term(iTRAQ4plex); C6(Methylthio); K17(iTRAQ4plex)	HCD	2.1883218	0	1	1	1.9623	2.2723	2.1158	2.450044	9.510110375	117750.81	101687.22	231062.89	249138.16	10.35251129	5.323247831	2	High	8321	Unique	Used	71138	1	76.599	1	6.1583	-2.3233	1	11	24/32	25.42316	8321	2082.12	MS2	1041.56	225	Glioma_grade 2_4.raw
6996021			kLLGSALALAR	Unambiguous	N-Term(iTRAQ4plex); K1(iTRAQ4plex)	HCD	1.797412	1	1	1	1.57192	2.0353	1.6137	2.0893683	14.91117123	9512.101	7346.506	14952.306	15349.556	18.16647022	1.853999393	3	High	3277	Unique	Used	80590	1	36.979	1	4.8954	-0.504	1	88	26/40	39.12244	3277	1400.92	MS2	467.644	30	Glioma_grade 2_6.raw
6996021			iGVFLcGPEALAETLSk	Unambiguous	N-Term(iTRAQ4plex); C6(Methylthio); K17(iTRAQ4plex)	HCD	2.6404998	0	1	1	2.08607	3.0289	2.373	3.445513	22.58573882	5574.9326	3839.5503	11629.72	13229.221	26.06835883	9.099486608	3	High	8376	Unique	Used	63587	1	76.584	1	6.2378	0.5723	1	47	22/64	29.64359	8376	2082.12	MS2	694.713	208	Glioma_grade 2_3.raw
7019485	NP_037364.1	PDCD6	programmed cell death protein 6 [Homo sapiens]	2	8	2.116760731	9.390846165	41.764472	12.57	8.40E-05	191	21.855	5.4019	Calcium binding protein	Cell communication,Signal transduction	Calcium ion binding	Cytoplasm	Nucleoplasm, Perinuclear region, Nucleolus, Nucleus																								
7019485			lSDQFHDILIR	Unambiguous	N-Term(iTRAQ4plex)	HCD	1.9216288	0	1	1	1.84086	1.9759	1.8711	2.0083213	4.191279983	124185.08	115700.34	228607.27	232363.48	5.002069063	1.152368805	2	High	4760	Unique	Used	75205	1	48.946	1	4.0992	1.8739	1	28	15/20	45.98308	4760	1500.83	MS2	750.92	294	Glioma_grade 2_5.raw
7019485			lSDQFHDILIR	Unambiguous	N-Term(iTRAQ4plex)	HCD	2.158949	0	1	1	2.09451	2.0168	2.3066	2.220993	5.978500293	117384.41	121909.17	245863.19	270759.44	2.674111424	6.8151514	2	High	5193	Unique	Used	68343	1	50.292	1	4.8639	1.4673	1	23	18/20	14.82396	5193	1500.83	MS2	750.919	224	Glioma_grade 2_4.raw
7019485			aGVNFSEFTGVWk	Unambiguous	N-Term(iTRAQ4plex); K13(iTRAQ4plex)	HCD	2.1152468	0	1	1	1.99612	2.1281	2.1032	2.242253	4.770772002	98098.4	92015.42	195815.8	206321.88	4.524990774	3.69471491	2	High	6545	Unique	Used	61933	1	61.414	1	4.7213	3.0883	1	53	21/24	56.97256	6545	1729.92	MS2	865.464	213	Glioma_grade 2_3.raw
7019485			lSDQFHDILIR	Unambiguous	N-Term(iTRAQ4plex)	HCD	2.118275	0	1	1	2.40319	1.8044	2.5364	1.9043523	16.73202661	57999.83	77248.63	139384.81	147108.62	20.12733751	3.812693665	3	High	5192	Unique	Used	68342	1	50.286	1	3.9155	-0.0464	1	7	20/40	6.923824	5192	1500.83	MS2	500.948	202	Glioma_grade 2_4.raw
7019485			aGVNFSEFTGVWk	Unambiguous	N-Term(iTRAQ4plex); K13(iTRAQ4plex)	HCD	2.1811411	0	1	1	2.21754	2.0917	2.276	2.1468122	3.692544811	40008.55	42416.133	88720.39	91059.46	4.13084577	1.839997373	2	High	6659	Unique	Used	69687	1	61.968	1	5.3481	2.3121	1	61	22/24	38.76498	6659	1729.92	MS2	865.463	215	Glioma_grade 2_4.raw
7019485			lSDQFHDILIR	Unambiguous	N-Term(iTRAQ4plex)	HCD	2.5249493	0	1	1	2.51256	2.3026	2.7675	2.5363016	7.516586497	26591.127	29015.553	66811.86	73592.19	6.16590692	6.829457301	3	High	4750	Unique	Used	75196	1	48.868	1	3.3396	0.3806	1	12	19/40	26.27551	4750	1500.83	MS2	500.948	215	Glioma_grade 2_5.raw
7019485			aGVNFSEFTGVWk	Unambiguous	N-Term(iTRAQ4plex); K13(iTRAQ4plex)	HCD	2.0611436	0	1	1	1.85206	2.0547	2.0669	2.2931132	8.721964794	9000.578	8112.743	16669.574	18603.44	7.336906134	7.7535181	3	High	6572	Unique	Used	61958	1	61.627	1	5.1006	-1.365	1	27	18/48	31.97118	6572	1729.91	MS2	577.309	201	Glioma_grade 2_3.raw
7019485			aGVNFSEFTGVWk	Unambiguous	N-Term(iTRAQ4plex); K13(iTRAQ4plex)	HCD	1.8651522	0	1	1	2.04365	2.2506	1.5151	1.668582	18.04717137	5416.6284	4918.5444	11069.676	8206.994	6.815533341	21.0017794	3	High	6661	Unique	Used	69689	1	61.982	1	5.0586	0.5402	1	61	23/48	17.19302	6661	1729.92	MS2	577.31	204	Glioma_grade 2_4.raw
7262378	NP_004205.2	FIBP	acidic fibroblast growth factor intracellular-binding protein isoform b [Homo sapiens]	2	2	0.449292183	22.65727648	9.3859799	8.12	4.50E-04	357	41.184	6.7563	Unclassified	Cell communication,Signal transduction	Molecular function unknown	Nucleus	Mitochondrion, Endoplasmic reticulum																								
7262378			gSLVDNIQQHFLLSDR	Unambiguous	N-Term(iTRAQ4plex)	HCD	0.5212738	0	1	2	0.47106	0.5055	0.536	0.575157	0.08490135	13752.601	12816.584	6478.2715	7371.548	4.982192476	9.121301121	3	High	6424	Unique	Used	69473	1	60.05	1	5.9215	-0.2095	1	31	31/60	14.52699	6424	1986.05	MS2	662.689	173	Glioma_grade 2_4.raw
7262378			vLVADkDLLDLHk	Unambiguous	N-Term(iTRAQ4plex); K6(iTRAQ4plex); K13(iTRAQ4plex)	HCD	0.37731057	1	1	2	0.39888	0.5027	0.2778	0.35013056	0.246839886	5477.411	4346.043	2184.8108	1521.6825	16.28750916	25.30167896	4	High	3991	Unique	Used	91450	1	45.312	1	3.4645	6.5101	1	100	22/72	35.20479	3991	1911.18	MS2	478.55	46	Glioma_grade 2_7rep.raw
7330335	NP_039234.1	CLIC4	chloride intracellular channel protein 4 [Homo sapiens]	3	4	2.02753067	10.97466141	35.541291	25.3	1.49E-04	253	28.754	5.5923	Intracellular ligand gated channel	Transport	Intracellular ligand-gated ion channel activity	Cytoplasm	Plasma membrane																								
7330335			hPESNTAGMDIFAk	Unambiguous	N-Term(iTRAQ4plex); K14(iTRAQ4plex)	HCD	2.2861958	0	1	1	2.09861	2.2887	2.2839	2.4907799	6.993685771	47486.312	43542	99655	108453.55	6.127873188	5.97910501	2	High	2282	Unique	Used	72970	1	29.113	1	4.5954	-0.493	1	45	18/26	0	2282	1805.91	MS2	903.458	115	Glioma_grade 2_5.raw
7330335			eEDkEPLIELFVk	Unambiguous	N-Term(iTRAQ4plex); K4(iTRAQ4plex); K13(iTRAQ4plex)	HCD	2.124344	1	1	1	1.99008	2.1661	2.086	2.2704828	5.595735452	36009.55	33082.906	71661.984	75114.17	5.990378511	3.32624076	2	High	5581	Unique	Used	82563	1	57.255	1	5.1844	-0.4913	1	79	18/24	0	5581	2021.15	MS2	1011.08	160	Glioma_grade 2_6.raw
7330335			iEEFLEEVLcPPk	Unambiguous	N-Term(iTRAQ4plex); C10(Methylthio); K13(iTRAQ4plex)	HCD	1.7761998	0	1	1	1.92001	1.688	1.8765	1.6497674	7.542490343	8336.4375	9482.238	16006.043	15643.487	9.093866752	1.620029152	2	High	8467	Unique	Used	63673	1	77.246	1	3.1669	-2.6787	1	50	17/24	33.86707	8467	1879.97	MS2	940.491	224	Glioma_grade 2_3.raw
7330335			iEEFLEEVLcPPk	Unambiguous	N-Term(iTRAQ4plex); C10(Methylthio); K13(iTRAQ4plex)	HCD	1.9307173	0	1	1	1.75743	1.8815	1.9767	2.116231	7.843874047	4889.4316	4567.1084	8592.85	9665.057	4.820302573	8.305057535	3	High	8446	Unique	Used	63653	1	77.094	1	4.6884	0.853	1	46	23/48	0	8446	1879.98	MS2	627.332	194	Glioma_grade 2_3.raw
7656991	NP_055140.1	CORO1C	coronin-1C isoform 1 [Homo sapiens]	3	5	2.344576359	20.59207553	41.687518	18.78	1.16E-03	474	53.215	7.0786	Cytoskeletal associated protein	Cell growth and/or maintenance	Cytoskeletal protein binding	Cytoplasm	-																								
7656991			kcEPIIMTVPR	Unambiguous	N-Term(iTRAQ4plex); K1(iTRAQ4plex); C2(Methylthio)	HCD	1.7121078	1	1	1	1.59298	1.7213	1.7036	1.8408283	5.91792879	64362.49	59563.71	102528.445	109646.56	5.476242924	4.744448938	2	High	3250	Unique	Used	80565	1	36.774	1	3.7269	2.5709	1	82	16/20	23.7173	3250	1620.89	MS2	810.95	236	Glioma_grade 2_6.raw
7656991			kcEPIIMTVPR	Unambiguous	N-Term(iTRAQ4plex); K1(iTRAQ4plex); C2(Methylthio)	HCD	1.7504022	1	1	1	1.41687	1.86	1.6669	2.188248	18.25072153	38078.26	29006.656	53951.836	63473.754	19.12380036	11.46770953	3	High	3237	Unique	Used	80553	1	36.675	1	4.165	-2.2573	1	23	18/40	23.0005	3237	1620.88	MS2	540.966	181	Glioma_grade 2_6.raw
7656991			yFEITDESPYVHYLNTFSSk	Unambiguous	N-Term(iTRAQ4plex); K20(iTRAQ4plex)	HCD	2.5585968	0	1	1	2.15129	2.6252	2.504	3.05562	14.44108092	30075.574	24646.508	64701.39	75310.36	14.03064081	10.71577868	3	High	7105	Unique	Used	70086	1	65.746	1	7.0049	2.0012	1	13	31/76	19.4816	7105	2728.34	MS2	910.12	84	Glioma_grade 2_4.raw
7656991			yFEITDESPYVHYLNTFSSk	Unambiguous	N-Term(iTRAQ4plex); K20(iTRAQ4plex)	HCD	2.6901367	0	1	1	2.32254	2.7641	2.628	3.1276214	12.31336491	21632.986	18176.934	50243.555	56850.566	12.2773309	8.724778238	3	High	6700	Unique	Used	76929	1	64.979	1	7.7584	-0.9517	1	18	34/76	3.265429	6700	2728.34	MS2	910.117	73	Glioma_grade 2_5.raw
7656991			kSDLFQDDLYPDTAGPEAALEAEEWFEGk	Unambiguous	N-Term(iTRAQ4plex); K1(iTRAQ4plex); K29(iTRAQ4plex)	HCD	2.3445764	1	1	1	2.13384	2.7667	2.019	2.6178071	15.25454698	1005.8089	775.7493	2146.2373	2030.7621	18.26229457	3.909662854	3	High	7250	Unique	Used	83877	1	73.946	1	8.5856	0.8954	1	75	39/112	15.41515	7250	3703.81	MS2	1235.27	91	Glioma_grade 2_6.raw
7657176	NP_055070.1	CNPY2	protein canopy homolog 2 isoform 1 precursor [Homo sapiens]	3	5	2.01738143	23.46114611	45.604908	31.32	2.42E-03	182	20.639	4.9194	Integral membrane protein	Cell growth and/or maintenance	Protein binding	Integral to membrane	Cytoplasm																								
7657176			iDSDISGTLk	Unambiguous	N-Term(iTRAQ4plex); K10(iTRAQ4plex)	HCD	1.55597	0	1	1	1.41884	1.6524	1.4732	1.7156649	9.045290321	302879.38	260076.64	429738.28	446204.34	10.75256561	2.658453287	2	High	2649	Unique	Used	50274	1	31.257	1	4.2439	-0.1708	1	14	16/18	30.26787	2649	1336.76	MS2	668.882	317	Glioma_grade 2_2.raw
7657176			tDLcDHALHISHDEL	Unambiguous	N-Term(iTRAQ4plex); C4(Methylthio)	HCD	1.9692278	0	1	1	2.28946	1.7634	2.2365	1.7225796	15.05025223	23431.043	30421.254	53644.406	52403.03	18.35697222	1.655458011	3	High	4660	Unique	Used	81774	1	49.203	1	6.0147	-0.0079	1	23	21/56	49.89585	4660	1908.87	MS2	636.962	51	Glioma_grade 2_6.raw
7657176			sEAHLTELLEEIcDR	Unambiguous	N-Term(iTRAQ4plex); C13(Methylthio)	HCD	2.920471	0	1	1	3.11394	2.4338	3.5432	2.7692616	16.02176407	9142.199	11697.211	28468.254	32392.637	17.33893917	9.119018094	2	High	7701	Unique	Used	77753	1	74.469	1	5.3269	-1.886	1	67	23/28	15.86271	7701	1947.92	MS2	974.465	97	Glioma_grade 2_5.raw
7657176			tDLcDHALHISHDEL	Unambiguous	N-Term(iTRAQ4plex); C4(Methylthio)	HCD	2.1582928	0	1	1	2.59091	2.0176	2.3389	1.8213943	15.54656284	4211.092	5407.5854	10910.575	9849.346	17.59178651	7.22933601	4	High	4664	Unique	Used	81778	1	49.228	1	5.7517	-0.8782	1	45	21/84	6.489103	4664	1908.87	MS2	477.973	47	Glioma_grade 2_6.raw
7657176			sEAHLTELLEEIcDR	Unambiguous	N-Term(iTRAQ4plex); C13(Methylthio)	HCD	2.0173814	0	1	1	2.22307	1.743	2.3673	1.8561069	14.44553466	3797.5188	4843.3545	8442.152	8989.784	17.11673091	4.442814622	3	High	7682	Unique	Used	77739	1	74.265	1	5.3354	-0.6082	1	32	23/56	13.11048	7682	1947.93	MS2	649.98	81	Glioma_grade 2_5.raw
7662164	NP_055547.1	KIAA0513	uncharacterized protein KIAA0513 [Homo sapiens]	2	2	0.469408333	20.12083884	10.906427	5.11	3.99E-04	411	46.609	5.0591	Unclassified	Biological_process unknown	Molecular function unknown	Cytoplasm	Perinuclear region																								
7662164			gFFGGLETk	Unambiguous	N-Term(iTRAQ4plex); K9(iTRAQ4plex)	HCD	0.5361938	0	1	1	0.47426	0.5408	0.5322	0.60681075	0.100791863	185652.6	162821.08	88047.84	98801.57	9.265734283	8.139212648	2	High	4247	Unique	Used	51776	1	42.895	1	3.2762	9.0421	1	43	16/16	60.51501	4247	1243.7	MS2	622.356	244	Glioma_grade 2_2.raw
7662164			ekPAGSIDSYLk	Unambiguous	N-Term(iTRAQ4plex); K2(iTRAQ4plex); K12(iTRAQ4plex)	HCD	0.40262288	0	1	1	0.37676	0.4151	0.3913	0.43112025	0.06006776	150781.45	136842.73	56808.402	58995.676	6.853501283	2.671125757	2	High	1995	Unique	Used	79442	1	26.59	1	3.7777	17.471	1	77	20/22	22.51677	1995	1740.02	MS2	870.514	151	Glioma_grade 2_6.raw

7662228	NP_055656.1	SNAP91	clathrin coat assembly protein AP180 isoform a [Homo sapiens]	7	16	0.440889895	20.30924969	110.11686	13.12	1.05E-04	907	92.444	4.856	Adapter molecule	Cell communication,Signal transduction	Receptor signaling complex scaffold activity	Cytoplasm	Plasma membrane																								
7662228			lTGGANWQPk	Unambiguous	N-Term(iTRAQ4plex); K10(iTRAQ4plex)	HCD	0.3638106	0	1	5	0.32594	0.351	0.3757	0.4045886	0.092422113	241878.05	224608.06	78838.734	90873.86	5.235623003	10.02885998	2	High	1880	Unique	Used	57596	1	25.508	1	3.9064	6.8443	1	31	17/18	31.55972	1880	1359.77	MS2	680.389	282	Glioma_grade2_3.raw
7662228			nTLFNLSNFLDk	Unambiguous	N-Term(iTRAQ4plex); K12(iTRAQ4plex)	HCD	0.45190883	0	1	8	0.4089	0.4573	0.4471	0.5000145	0.082623526	228563.27	204367.22	93458.54	102186.57	7.903897475	6.309024743	2	High	7830	Unique	Used	55119	1	69.755	1	5.2624	-2.127	1	40	18/22	34.20416	7830	1713.94	MS2	857.473	229	Glioma_grade2_2.raw
7662228			nTLFNLSNFLDk	Unambiguous	N-Term(iTRAQ4plex); K12(iTRAQ4plex)	HCD	0.45944065	0	1	8	0.43916	0.4282	0.4915	0.47921768	0.066617885	120424.445	123518.78	52885.055	59192.383	1.793880737	7.958701554	2	High	7485	Unique	Used	62788	1	69.074	1	6.0716	2.8585	1	94	20/22	47.86949	7485	1713.95	MS2	857.477	223	Glioma_grade2_3.raw
7662228			sGSHGYDMSTFIR	Unambiguous	N-Term(iTRAQ4plex)	HCD	0.3539174	0	1	4	0.34493	0.3111	0.4014	0.36202383	0.105857086	119847.26	132886.34	41338.79	48108.023	7.296237531	10.70260728	2	High	3244	Unique	Used	66543	1	34.97	1	4.6055	-0.8251	1	65	19/24	21.18911	3244	1601.75	MS2	801.378	94	Glioma_grade2_4.raw
7662228			kPGNNEGSGAPSPLSk	Unambiguous	N-Term(iTRAQ4plex); K1(iTRAQ4plex); K16(iTRAQ4plex)	HCD	0.4098433	0	1	2	0.36696	0.4282	0.3941	0.45987746	0.09803783	103263.63	88500.69	37893.848	40699.47	10.88729643	5.048455498	2	High	1021	Unique	Used	88955	1	18.862	1	5.5145	-1.7257	1	100	23/30	0	1021	1972.08	MS2	986.543	168	Glioma_grade2_7rep.raw
7662228			sGSHGYDMSTFIR	Unambiguous	N-Term(iTRAQ4plex)	HCD	0.5843282	0	1	4	0.59956	0.5496	0.6223	0.57036686	0.054650607	60468.19	65970.695	36254.31	37627.496	6.154528489	2.628493225	2	High	2937	Unique	Used	58579	1	33.425	1	4.5236	0.9278	1	99	18/24	0	2937	1601.75	MS2	801.379	111	Glioma_grade2_3.raw
7662228			aLVTTHHLMVHGNER	Unambiguous	N-Term(iTRAQ4plex)	HCD	0.39559194	0	1	4	0.36868	0.3624	0.4293	0.42204627	0.088305738	57143.79	58129.207	21067.855	24533.215	1.208947561	10.74702657	3	High	1121	Unique	Used	84741	1	20.308	1	5.7412	-0.1389	1	29	25/56	17.91333	1121	1858.98	MS2	620.332	177	Glioma_grade2_1rep.raw
7662228			aLVTTHHLMVHGNER	Unambiguous	N-Term(iTRAQ4plex)	HCD	0.55166423	0	1	4	0.58044	0.5948	0.5095	0.52217144	0.076457391	25380.248	24765.54	14731.779	12931.858	1.733602014	9.201511318	4	High	1118	Unique	Used	84739	1	20.27	1	5.465	1.1671	1	44	26/84	37.24201	1118	1858.98	MS2	465.502	169	Glioma_grade2_1rep.raw
7662228			aLVTTHHLmVHGNER	Unambiguous	N-Term(iTRAQ4plex); M9(Oxidation)	HCD	0.44792032	0	1	4	0.406	0.4661	0.4321	0.4960442	0.087342909	16353.094	14244.756	6639.371	7066.0283	9.744606872	4.402531637	3	High	1050	Unique	Used	94114	1	19.237	1	4.9834	2.1511	1	100	19/56	38.35348	1050	1874.98	MS2	625.665	161	Glioma_grade2_8rep.raw
7662228			nTLFNLSNFLDk	Unambiguous	N-Term(iTRAQ4plex); K12(iTRAQ4plex)	HCD	0.43385944	0	1	8	0.38026	0.3826	0.4848	0.4877885	0.139545164	15673.895	15576.842	5960.222	7598.2046	0.439201382	17.08500017	3	High	7506	Unique	Used	62807	1	69.251	1	4.7768	2.9219	1	47	20/44	52.51254	7506	1713.95	MS2	571.987	221	Glioma_grade2_3.raw
7662228			aLVTTHHLMVHGNER	Unambiguous	N-Term(iTRAQ4plex)	HCD	0.45343223	0	1	4	0.46806	0.4736	0.4335	0.43862697	0.044826958	15243.822	15064.711	7135.077	6607.7886	0.835742216	5.426076542	3	High	1231	Unique	Used	94260	1	21.071	1	5.1154	-0.7299	1	100	24/56	12.34824	1231	1858.98	MS2	620.332	202	Glioma_grade2_8rep.raw
7662228			aLVTTHHLMVHGNER	Unambiguous	N-Term(iTRAQ4plex)	HCD	0.48577723	0	1	4	0.52714	0.5506	0.4237	0.44257018	0.128136147	11190.654	10713.619	5899.0693	4741.5283	3.07989846	15.38456995	2	High	1139	Unique	Used	84757	1	20.459	1	3.9883	1.8383	1	100	20/28	0	1139	1858.99	MS2	929.997	148	Glioma_grade2_1rep.raw
7662228			gDIPDLTQAPSSLMETLEQHLNTLEGk	Unambiguous	N-Term(iTRAQ4plex); K27(iTRAQ4plex)	HCD	0.42205915	0	1	4	0.52459	0.4326	0.4093	0.33751616	0.181175317	7880.014	9556.228	4133.744	3225.3813	13.59538697	17.45613504	3	High	7915	Unique	Used	77935	1	76.374	1	5.8486	-3.5606	1	20	32/104	10.62738	7915	3225.64	MS2	1075.89	74	Glioma_grade2_5.raw
7662228			hLDYLIQATNETNVNIPQMADTLFER	Unambiguous	N-Term(iTRAQ4plex)	HCD	0.639958	0	1	3	0.6119	0.7765	0.5324	0.67556226	0.158949268	2209.6724	1741.3322	1352.0988	1176.3783	16.76366215	9.828299979	3	High	7964	Unique	Used	63218	1	73.166	1	6.5111	0.5351	1	100	29/100	5.210578	7964	3190.6	MS2	1064.2	113	Glioma_grade2_3.raw
7662228			hLDYLIQATNETNVNIPQMADTLFER	Unambiguous	N-Term(iTRAQ4plex)	HCD	0.3754816	0	1	3	0.39487	0.4715	0.295	0.35232463	0.196426653	1945.1599	1628.8667	768.0914	573.8899	12.51546738	20.46544129	3	High	7893	Unique	Used	70772	1	72.754	1	8.0859	-1.4162	1	87	31/100	20.0496	7893	3190.59	MS2	1064.2	107	Glioma_grade2_4.raw
7662228			hLDYLIQATNETNVNIPQMADTLFER	Unambiguous	N-Term(iTRAQ4plex)	HCD	0.27978855	0	1	3	0.27538	0.2347	0.3328	0.2835441	0.142978421	1358.3066	1594.0385	374.05215	451.98016	11.29187946	13.34171161	3	High	8087	Unique	Used	70932	1	74.634	1	7.3696	-2.6787	1	100	29/100	5.93754	8087	3190.59	MS2	1064.2	107	Glioma_grade2_4.raw
7662270	NP_055663.1	SV2B	synaptic vesicle glycoprotein 2B isoform 1 [Homo sapiens]	6	16	0.279870093	28.19484604	64.012682	13.47	6.41E-05	683	77.393	5.4399	Membrane transport protein	Transport	Auxiliary transport protein activity	Cytoplasmic vesicle	-																								
7662270			fLLEMGk	Unambiguous	N-Term(iTRAQ4plex); K7(iTRAQ4plex)	HCD	0.17571448	0	1	2	0.16316	0.1777	0.1739	0.18938717	0.061465742	2952007.8	2710943.2	481644.56	513417.9	6.020126728	4.51572541	2	High	4759	Unique	Used	52256	1	46.695	1	2.934	-6.9944	1	6	12-Dec	58.77502	4759	1125.65	MS2	563.329	202	Glioma_grade2_2.raw
7662270			yFQDEEYk	Unambiguous	N-Term(iTRAQ4plex); K8(iTRAQ4plex)	HCD	0.27983227	0	1	2	0.26557	0.287	0.2732	0.29524747	0.047628973	2885201.5	2670186.8	766211.1	788365.9	5.473545474	2.015443341	2	High	2351	Unique	Used	49990	1	29.247	1	3.1608	0.735	1	5	13/14	35.82342	2351	1409.68	MS2	705.346	93	Glioma_grade2_2.raw
7662270			vFTVSNIk	Unambiguous	N-Term(iTRAQ4plex); K8(iTRAQ4plex)	HCD	0.2799079	0	1	2	0.24734	0.2883	0.2727	0.31787148	0.104843238	2585470.2	2217893	639494.5	705004.9	10.82226431	6.890720528	2	High	2867	Unique	Used	58513	1	32.908	1	3.6127	-0.2878	1	5	14/14	34.26907	2867	1195.73	MS2	598.368	208	Glioma_grade2_3.raw
7662270			hDEAWMILk	Unambiguous	N-Term(iTRAQ4plex); K9(iTRAQ4plex)	HCD	0.24589707	0	1	3	0.23086	0.234	0.2577	0.26113513	0.063816232	712353.75	702877	164455.2	183545.88	0.946993865	7.758107696	2	High	3259	Unique	Used	80573	1	36.85	1	4.508	4.8324	1	33	15/16	6.619371	3259	1430.78	MS2	715.892	215	Glioma_grade2_6.raw
7662270			hDEAWMILk	Unambiguous	N-Term(iTRAQ4plex); K9(iTRAQ4plex)	HCD	0.22996555	0	1	3	0.21677	0.2376	0.223	0.2444237	0.055363016	529393.94	483035.56	114758.57	118065.34	6.475576791	2.008590519	3	High	3293	Unique	Used	80604	1	37.124	1	3.5549	3.6925	1	7	18/32	18.48349	3293	1430.78	MS2	477.597	221	Glioma_grade2_6.raw
7662270			vFTVSNIk	Unambiguous	N-Term(iTRAQ4plex); K8(iTRAQ4plex)	HCD	0.28649086	0	1	2	0.2395	0.2722	0.2991	0.33989403	0.147757809	434134.1	382042.84	103973.15	129854.08	9.026004382	15.6530795	2	High	2804	Unique	Used	50418	1	32.411	1	3.2031	-8.0466	1	9	14/14	60.17055	2804	1195.72	MS2	598.363	259	Glioma_grade2_2.raw
7662270			vFTVSNIk	Unambiguous	N-Term(iTRAQ4plex); K8(iTRAQ4plex)	HCD	0.26944962	0	1	2	0.27046	0.2525	0.2876	0.2685049	0.053253696	421206.97	451187.2	113920.32	121145.96	4.860010455	4.34711354	2	High	2710	Unique	Used	50331	1	31.7	1	3.3506	-4.6776	1	11	14/14	33.42567	2710	1195.72	MS2	598.365	232	Glioma_grade2_2.raw
7662270			fLLEMGk	Unambiguous	N-Term(iTRAQ4plex); K7(iTRAQ4plex)	HCD	0.2689426	0	1	2	0.24659	0.285	0.255	0.29478452	0.085747884	338681.78	292998.56	83514.31	86371.445	10.22761438	2.378421349	2	High	4711	Unique	Used	60229	1	47.082	1	2.9619	-6.669	1	39	12-Dec	59.20254	4711	1125.65	MS2	563.329	205	Glioma_grade2_3.raw
7662270			vFTVSNIk	Unambiguous	N-Term(iTRAQ4plex); K8(iTRAQ4plex)	HCD	0.41005188	0	1	2	0.34017	0.398	0.4204	0.49181154	0.151979952	313715.72	268149.94	106715.89	131879.23	11.07467728	14.91494742	2	High	2819	Unique	Used	50432	1	32.524	1	3.2286	7.8793	1	21	14/14	63.22633	2819	1195.74	MS2	598.373	164	Glioma_grade2_2.raw
7662270			fLLEMGk	Unambiguous	N-Term(iTRAQ4plex); K7(iTRAQ4plex)	HCD	0.2861521	0	1	2	0.2875	0.2865	0.2858	0.28481263	0.003957291	267616.4	268544.8	76938.71	76484.95	0.244880807	0.418262441	2	High	4549	Unique	Used	60078	1	45.836	1	2.9457	10.14	1	99	12-Dec	61.42268	4549	1125.67	MS2	563.338	179	Glioma_grade2_3.raw
7662270			vFTVSNIk	Unambiguous	N-Term(iTRAQ4plex); K8(iTRAQ4plex)	HCD	0.31624487	0	1	2	0.3093	0.2953	0.3382	0.32287636	0.058156414	179826.88	188377.66	55620.105	60822.695	3.284215085	6.318615953	2	High	2745	Unique	Used	58402	1	31.925	1	3.5084	-7.434	1	40	14/14	35.42705	2745	1195.72	MS2	598.364	220	Glioma_grade2_3.raw
7662270			vFTVSNIk	Unambiguous	N-Term(iTRAQ4plex); K8(iTRAQ4plex)	HCD	0.27239883	0	1	2	0.25389	0.2547	0.2901	0.29096636	0.07682324	104962.96	104639.14	26648.895	30446.47	0.21848571	9.406336345	3	High	2807	Unique	Used	58459	1	32.406	1	3.5235	-2.5705	1	77	28-Dec	18.49788	2807	1195.73	MS2	399.247	222	Glioma_grade2_3.raw
7662270			vFTVSNIk	Unambiguous	N-Term(iTRAQ4plex); K8(iTRAQ4plex)	HCD	0.29674387	0	1	2	0.28502	0.3277	0.2698	0.3102242	0.08637991	48761.902	42415.418	13898.022	13158.289	9.843767887	3.86653022	3	High	2879	Unique	Used	58525	1	32.982	1	3.4569	8.7613	1	99	28-Nov	60.34654	2879	1195.74	MS2	399.251	144	Glioma_grade2_3.raw
7662270			fGAILGNTIFASFVGITk	Unambiguous	N-Term(iTRAQ4plex); K18(iTRAQ4plex)	HCD	0.27217618	0	1	2	0.3526	0.2502	0.3032	0.21511148	0.21512409	7291.4214	10275.789	2570.936	2210.4402	24.02506169	10.66257973	3	High	8368	Unique	Used	71181	1	76.962	1	5.1942	-2.5587	1	21	22/68	17.62159	8368	2144.23	MS2	715.415	104	Glioma_grade2_4.raw
7662270			fGAILGNTIFASFVGITk	Unambiguous	N-Term(iTRAQ4plex); K18(iTRAQ4plex)	HCD	0.51675403	0	1	2	0.59695	0.5628	0.4679	0.44114572	0.144068618	4748.5625	5036.4707	2834.6377	2221.8174	4.161086353	17.13965144	3	High	8450	Unique	Used	63657	1	77.122	1	5.0815	2.9919	1	71	20/68	51.82924	8450	2144.24	MS2	715.419	84	Glioma_grade2_3.raw
7662270			lEDEEQLAHQYETIMDEcGHGR	Unambiguous	N-Term(iTRAQ4plex); C18(Methylthio)	HCD	0.45122567	0	1	1	0.33697	0.3958	0.4984	0.5854198	0.242320533	2927.5312	2492.576	986.4882	1459.2032	11.34884459	27.33460011	4	High	6796	Unique	Used	77010	1	65.854	1	5.8408	0.1485	1	39	25/126	40.85279	6796	2793.2	MS2	699.056	14	Glioma_grade2_5.raw
7705696	NP_056997.1	TXNDC12	thioredoxin domain-containing protein 12 precursor [Homo sapiens]	3	7	2.152822971	13.86215292	39.610908	27.33	1.80E-04	172	19.194	5.4019	Enzyme: Reductase	Protein metabolism	Catalytic activity	Endoplasmic reticulum	Mitochondrion, Golgi apparatus																								
7705696			iLFLDPSGk	Unambiguous	N-Term(iTRAQ4plex); K9(iTRAQ4plex)	HCD	2.296513	0	1	1	2.14687	2.4181	2.1885	2.4650595	6.950951509	133551.55	118569.88	286718.3	292281.8	8.403601749	1.358890466	2	High	5413	Unique	Used	60883	1	52.519	1	3.302	0.4873	1	25	13/16	4.297534	5413	1277.77	MS2	639.389	199	Glioma_grade2_3.raw
7705696			iLFLDPSGk	Unambiguous	N-Term(iTRAQ4plex); K9(iTRAQ4plex)	HCD	2.0244157	0	1	1	1.8318	2.1758	1.897	2.2532094	10.11695174	108417.13	91275.1	198598.19	205661.92	12.13992718	2.471087926	2	High	5665	Unique	Used	53111	1	53.217	1	3.4699	-1.9011	1	25	13/16	28.80377	5665	1277.77	MS2	639.388	220	Glioma_grade2_2.raw
7705696			iLFLDPSGk	Unambiguous	N-Term(iTRAQ4plex); K9(iTRAQ4plex)	HCD	2.2450693	0	1	1	2.25362	2.2124	2.2783	2.2366788	1.236695467	82244.18	83774.94	185346.78	187377.62	1.303959178	0.770553651	2	High	5657	Unique	Used	68772	1	53.874	1	3.1659	0.3917	1	30	13/16	17.5646	5657	1277.77	MS2	639.389	215	Glioma_grade2_4.raw
7705696			eAAASGLPLMVIIHk	Unambiguous	N-Term(iTRAQ4plex); K15(iTRAQ4plex)	HCD	1.8263751	0	1	1	1.60494	1.8378	1.8164	2.0799367	10.59072493	32168.295	28092.85	51628.1	58431.35	9.564288219	8.74186489	3	High	5179	Unique	Used	82229	1	53.569	1	6.1027	-0.7666	1	20	26/56	33.16021	5179	1838.08	MS2	613.365	130	Glioma_grade2_6.raw
7705696			yFYVSAEQVVQGMk	Unambiguous	N-Term(iTRAQ4plex); K14(iTRAQ4plex)	HCD	2.152823	0	1	1	1.97277	2.0721	2.2296	2.341949	7.608835859	32040.809	30504.28	63209.04	71439.46	3.474261825	8.644412369	2	High	6940	Unique	Used	54307	1	62.651	1	5.624	-1.6556	1	88	21/26	39.1135	6940	1937	MS2	969.006	205	Glioma_grade2_2.raw
7705696			iLFLDPSGk	Unambiguous	N-Term(iTRAQ4plex); K9(iTRAQ4plex)	HCD	2.3405793	0	1	1	2.32299	2.2601	2.4233	2.3576937	2.909872344	31654.42	32535.242	73532.86	76708.13	1.940609094	2.988871345	2	High	5587	Unique	Used	68712	1	53.249	1	3.5564	-1.2324	1	100	13/16	49.30232	5587	1277.77	MS2	639.388	222	Glioma_grade2_4.raw
7705696			yFYVSAEQVVQGMk	Unambiguous	N-Term(iTRAQ4plex); K14(iTRAQ4plex)	HCD	1.5528456	0	1	1	1.46924	1.7908	1.3576	1.6547474	12.27530016	9256.346	7594.386	13599.795	12566.79	13.94815591	5.583035314	3	High	6937	Unique	Used	54304	1	62.632	1	5.1741	2.6228	1	44	25/52	27.56083	6937	1937.01	MS2	646.343	235	Glioma_grade2_2.raw
7705813	NP_057177.1	RPL26L1	60S ribosomal protein L26-like 1 [Homo sapiens]	2	2	2.576769352	7.442887185	6.8134134	11.03	7.32E-05	145	17.246	10.55	Unclassified	Biological_process unknown	Molecular function unknown	Ribosome	Nucleolus																								
7705813			iMSSPLSk	Unambiguous	N-Term(iTRAQ4plex); K8(iTRAQ4plex)	HCD	2.4411561	0	1	2	2.37366	2.4223	2.4596	2.5100324	2.364136081	652632.94	639522.7	1549131	1605222.8	1.434864241	2.51480301	2	High	1946	Unique	Used	57658	1	25.985	1	3.0617	-0.5941	1	5	14-Dec	28.91484	1946	1150.67	MS2	575.841	302	Glioma_grade2_3.raw
7705813			ykEELIEk	Unambiguous	N-Term(iTRAQ4plex); K2(iTRAQ4plex); K8(iTRAQ4plex)	HCD	2.7123826	1	1	1	2.56097	2.7074	2.7171	2.8724535	4.687812107	243052.89	229905.84	622451.2	660393.8	3.931154081	4.182807706	2	High	1504	Unique	Used	78985	1	22.963	1	3.7517	-7.3309	1	28	14/14	13.22958	1504	1483.86	MS2	742.435	187	Glioma_grade2_6.raw
8400711	NP_058518.1	MAPT	microtubule-associated protein tau isoform 3 [Homo sapiens]	3	6	0.456835687	18.98392553	159.32099	44.39	1.00E-04	383	39.982	9.437	Structural protein	Cell growth and/or maintenance	Structural molecule activity	Cytoplasm	Nucleus, Plasma membrane																								
8400711			kLDLSNVQSk	Unambiguous	N-Term(iTRAQ4plex); K1(iTRAQ4plex); K10(iTRAQ4plex)	HCD	0.45215264	1	1	5	0.42521	0.4449	0.4591	0.48034865	0.051392036	796799.94	761522.06	338803.34	365796.1	3.201549895	5.417762932	2	High	1455	Unique	Used	89345	1	22.225	1	4.0654	-5.704	1	22	17/18	40.90572	1455	1563.93	MS2	782.471	170	Glioma_grade2_7rep.raw
8400711			lDLSNVQSk	Unambiguous	N-Term(iTRAQ4plex); K9(iTRAQ4plex)	HCD	0.64557195	0	1	5	0.60234	0.6997	0.599	0.69578445	0.086378336	346675.53	298450.1	208818.08	207656.94	10.5717482	0.394285337	2	High	2190	Unique	Used	49838	1	28.11	1	3.6112	3.876	1	23	15/16	51.27892	2190	1291.75	MS2	646.379	280	Glioma_grade2_2.raw
8400711			hVPGGGSVQIVYkPVDLSk	Unambiguous	N-Term(iTRAQ4plex); K13(iTRAQ4plex); K19(iTRAQ4plex)	HCD	0.3892635	0	1	5	0.36249	0.3468	0.4336	0.4148768	0.106307743	58579.3	61226.832	21234.564	25401.592	3.125195346	12.6362634	4	High	3185	Unique	Used	86216	1	43.488	1	5.8039	2.8517	1	11	24/108	14.32399	3185	2412.4	MS2	603.857	45	Glioma_grade2_1rep.raw
8400711			hVPGGGSVQIVYkPVDLSk	Unambiguous	N-Term(iTRAQ4plex); K13(iTRAQ4plex); K19(iTRAQ4plex)	HCD	0.42083564	0	1	5	0.44874	0.469	0.3747	0.39166898	0.106894558	42059.86	40241.01	18873.984	15761.156	3.125413301	12.71022313	3	High	3258	Unique	Used	95657	1	45.063	1	4.9735	-0.1029	1	100	26/72	18.7256	3258	2412.4	MS2	804.804	35	Glioma_grade2_8rep.raw
8400711			hVPGGGSVQIVYkPVDLSk	Unambiguous	N-Term(iTRAQ4plex); K13(iTRAQ4plex); K19(iTRAQ4plex)	HCD	0.46345308	0	1	5	0.49654	0.4509	0.4773	0.4334094	0.060140797	41292.49	45472.31	20503.287	19708.125	6.81285283	2.79654165	3	High	3170	Unique	Used	86207	1	43.29	1	4.8858	-0.5583	1	100	23/72	45.0593	3170	2412.4	MS2	804.804	31	Glioma_grade2_1rep.raw
8400711			hVPGGGSVQIVYkPVDLSk	Unambiguous	N-Term(iTRAQ4plex); K13(iTRAQ4plex); K19(iTRAQ4plex)	HCD	0.46151873	0	1	5	0.50051	0.4636	0.4593	0.42540935	0.066464073	18305.375	19764.602	9161.962	8408.046	5.420698347	6.068285411	4	High	3257	Unique	Used	95656	1	45.059	1	5.3375	-0.8928	1	26	24/108	23.36874	3257	2412.4	MS2	603.854	54	Glioma_grade2_8rep.raw
8922720	NP_060717.1	TMEM30A	cell cycle control protein 50A isoform 1 [Homo sapiens]	4	5	0.441521883	18.3831397	39.413816	18.84	9.90E-05	361	40.658	8.5874	Integral membrane protein	Biological_process unknown	Molecular function unknown	Integral to membrane	-																								
8922720			gIAWWTDk	Unambiguous	N-Term(iTRAQ4plex); K8(iTRAQ4plex)	HCD	0.4374485	0	1	2	0.41724	0.432	0.4427	0.45837316	0.039644186	289692.9	279773.62	120871.56	128240.73	2.463354703	4.183486956	2	High	4522	Unique	Used	75006	1	46.755	1	3.1785	-4.7855	1	26	13/14	23.00355	4522	1264.69	MS2	632.847	238	Glioma_grade2_5.raw
8922720			sDLHPTLPAGR	Unambiguous	N-Term(iTRAQ4plex)	HCD	0.37122077	0	1	2	0.39928	0.4076	0.3356	0.3425748	0.100800833	231577.47	226851.44	92464.75	77713.586	1.457939405	12.25849111	3	High	1221	Unique	Used	64661	1	19.945	1	4.1371	1.0122	1	7	19/40	7.502496	1221	1307.72	MS2	436.578	252	Glioma_grade2_4.raw
8922720			kSDLHPTLPAGR	Unambiguous	N-Term(iTRAQ4plex); K1(iTRAQ4plex)	HCD	0.6004178	1	1	2	0.62782	0.6926	0.5168	0.5701898	0.125596635	128680.08	116640.34	80787.61	66507.13	6.940622226	13.71104528	3	High	1035	Unique	Used	88966	1	19	1	3.2483	-3.3031	1	10	14/44	40.47982	1035	1579.91	MS2	527.308	244	Glioma_grade2_7rep.raw
8922720			dEVDGGPPcAPGGTAk	Unambiguous	N-Term(iTRAQ4plex); C9(Methylthio); K16(iTRAQ4plex)	HCD	0.44152188	0	1	2	0.43885	0.4618	0.4222	0.44433168	0.036929615	86156.31	81871.734	37809.85	36378.203	3.606127492	2.729084431	2	High	1946	Unique	Used	49608	1	26.394	1	5.7868	1.295	1	75	22/30	0	1946	1804.85	MS2	902.927	85	Glioma_grade2_2.raw
8922720			kSDLHPTLPAGR	Unambiguous	N-Term(iTRAQ4plex); K1(iTRAQ4plex)	HCD	0.44849885	1	1	2	0.41222	0.3762	0.5277	0.48160633	0.151501591	28105.902	30796.434	11585.871	14831.758	6.459823333	17.37619003	4	High	1031	Unique	Used	88963	1	18.956	1	3.9439	3.7194	1	21	19/66	31.62701	1031	1579.92	MS2	395.736	208	Glioma_grade2_7rep.raw
8923579	NP_060377.1	LAMTOR1	ragulator complex protein LAMTOR1 [Homo sapiens]	2	5	2.915744781	19.89494917	37.497407	34.16	2.54E-04	161	17.734	5.1479	Unclassified	Biological_process unknown	Molecular function unknown	-	-																								

8923579			lLLDPSSPPTk	Unambiguous	N-Term(iTRAQ4plex); K11(iTRAQ4plex)	HCD	2.0974839	0	1	1	1.91926	2.1889	2.0173	2.300742	8.10832225	255121.3	223695.89	489644.88	514666.5	9.281671994	3.523400716	2	High	3470	Unique	Used	51044	1	37.223	1	4.1976	-1.6005	1	12	16/20	20.92387	3470	1455.86	MS2	728.436	163	Glioma_grade 2_2.raw
8923579			lLLDPSSPPTk	Unambiguous	N-Term(iTRAQ4plex); K11(iTRAQ4plex)	HCD	3.3402412	0	1	1	3.10326	3.2158	3.4603	3.5858135	6.610907889	220541.94	212825.39	684399.3	763152.2	2.518152364	7.693917574	2	High	3406	Unique	Used	59019	1	36.915	1	4.2559	0.9987	1	8	17/20	5.884743	3406	1455.87	MS2	728.437	156	Glioma_grade 2_3.raw
8923579			lAVLSSSLTHWk	Unambiguous	N-Term(iTRAQ4plex); K12(iTRAQ4plex)	HCD	2.9157448	0	1	1	2.65048	2.7128	3.114	3.1872435	9.382492617	79350.05	77527.375	210315.89	247098.61	1.643099193	11.37231581	3	High	4902	Unique	Used	81987	1	51.219	1	5.3805	-2.1271	1	8	31/44	12.41288	4902	1629.95	MS2	543.989	131	Glioma_grade 2_6.raw
8923579			lLLDPSSPPTk	Unambiguous	N-Term(iTRAQ4plex); K11(iTRAQ4plex)	HCD	2.2613637	0	1	1	2.04906	2.2869	2.2385	2.498309	8.137658871	70164.68	62866.73	143771.89	157060.52	7.758212792	6.246986743	2	High	3380	Unique	Used	50959	1	36.573	1	4.2148	-0.259	1	51	14/20	18.5308	3380	1455.87	MS2	728.437	174	Glioma_grade 2_2.raw
8923579			lLLDPSSPPTk	Unambiguous	N-Term(iTRAQ4plex); K11(iTRAQ4plex)	HCD	3.149277	0	1	1	3.10103	3.0803	3.2187	3.1971972	2.184947196	59904.176	60307.48	185764.88	192814.9	0.474461467	2.633588592	2	High	3317	Unique	Used	58936	1	36.227	1	4.3269	0.4956	1	54	16/20	5.802151	3317	1455.87	MS2	728.437	163	Glioma_grade 2_3.raw
9789987	NP_036413.1	KCND2	potassium voltage-gated channel subfamily D member 2 precursor [Homo sapiens]	2	2	2.066504002	11.99546201	9.8544974	3.33	1.08E-04	630	70.491	7.9722	Voltage gated channel	Transport	Voltage-gated ion channel activity	Plasma membrane	-																								
9789987			dFFYHPETQQYFFDR	Unambiguous	N-Term(iTRAQ4plex)	HCD	1.8912215	0	1	1	1.80341	1.7125	2.0794	1.9746103	8.735135257	9775.991	10294.656	17630.107	20327.936	3.654606039	10.05137794	3	High	6216	Unique	Used	69284	1	58.365	1	5.6498	0.556	1	69	21/56	46.70011	6216	2184	MS2	728.67	55	Glioma_grade 2_4.raw
9789987			dFFYHPETQQYFFDRDPDIFR	Unambiguous	N-Term(iTRAQ4plex)	HCD	2.2417862	1	1	1	2.16181	2.3109	2.1772	2.3272767	3.869683764	1844.503	1725.5322	3987.4646	4015.7913	4.712842016	0.500546325	4	High	6172	Unique	Used	83041	1	62.915	1	4.2047	-1.1708	1	52	25/120	24.25511	6172	2927.35	MS2	732.593	44	Glioma_grade 2_6.raw
9845502	NP_002286.2	RPSA	40S ribosomal protein SA [Homo sapiens]	6	11	2.367699623	20.28632691	110.51266	39.32	1.85E-04	295	32.833	4.8687	Cell surface receptor,Ribosomal subunit	Cell adhesion,Cell communication,Signal transduction	Cell adhesion molecule activity,Structural constituent of ribosome	Cytoplasm	Cytosol, Ribosome, Nucleolus																								
9845502			fTPGTFTNQIQAAFR	Unambiguous	N-Term(iTRAQ4plex)	HCD	2.4767122	0	1	1	2.35743	2.4247	2.5273	2.5993993	4.332220582	102173.84	99340.78	240867.36	258226.38	1.988218957	4.918787704	2	High	5084	Unique	Used	87469	1	66.335	1	5.0146	2.0519	1	29	19/28	17.2804	5084	1842.97	MS2	921.987	261	Glioma_grade 2_1rep.raw
9845502			dPEEIEkEEQAAAEk	Unambiguous	N-Term(iTRAQ4plex); K7(iTRAQ4plex); K15(iTRAQ4plex)	HCD	2.7553823	1	1	1	2.76843	2.6536	2.8616	2.7428808	3.104570027	73716.84	76907.805	204079.56	210948.94	2.995994434	2.340747771	3	High	2180	Unique	Used	72873	1	28.404	1	3.9797	1.2527	1	5	27/56	7.03496	2180	2148.11	MS2	716.707	149	Glioma_grade 2_5.raw
9845502			kSDGIYIINLk	Unambiguous	N-Term(iTRAQ4plex); K1(iTRAQ4plex); K11(iTRAQ4plex)	HCD	1.7407631	1	1	1	1.67563	1.729	1.7522	1.8079745	3.150675204	66354.86	64307.12	111185.914	116265.63	2.216361393	3.158385395	3	High	3387	Unique	Used	90997	1	39.003	1	4.8684	-1.0017	1	9	20/40	15.18978	3387	1696.04	MS2	566.017	73	Glioma_grade 2_7rep.raw
9845502			kSDGIYIINLk	Unambiguous	N-Term(iTRAQ4plex); K1(iTRAQ4plex); K11(iTRAQ4plex)	HCD	2.0652928	1	1	1	1.99304	2.116	2.0176	2.1420066	3.526234417	63546.203	59854.15	126649.89	128207.98	4.231228922	0.864588568	2	High	3391	Unique	Used	91001	1	39.025	1	4.9438	-0.382	1	94	16/20	12.84898	3391	1696.04	MS2	848.522	71	Glioma_grade 2_7rep.raw
9845502			sDGIYIINLk	Unambiguous	N-Term(iTRAQ4plex); K10(iTRAQ4plex)	HCD	1.606322	0	1	1	1.52584	1.6581	1.5586	1.6937861	4.952823544	60987.37	56121.44	93056.74	95057.72	5.876128533	1.504303845	2	High	5414	Unique	Used	52873	1	51.442	1	3.7204	-0.2754	1	43	18/18	21.84775	5414	1423.84	MS2	712.423	234	Glioma_grade 2_2.raw
9845502			eHPWEVMPDLYFYR	Unambiguous	N-Term(iTRAQ4plex)	HCD	3.0064774	0	1	1	2.77309	3.0933	2.9286	3.2668223	7.048810195	52359.855	46938.97	145198.42	153341.27	7.720422761	3.857352738	2	High	6457	Unique	Used	76719	1	62.849	1	4.7648	-6.0761	1	27	20/26	73.91631	6457	2025.95	MS2	1013.48	80	Glioma_grade 2_5.raw
9845502			fLAAGTHLGGTNLDFQMEQYIYk	Unambiguous	N-Term(iTRAQ4plex); K23(iTRAQ4plex)	HCD	2.5138333	0	1	1	2.02259	2.9142	2.236	3.221627	21.66700341	23127.273	16051.55	46776.92	51712.105	25.54079644	7.086480509	3	High	6560	Unique	Used	76807	1	63.778	1	8.2354	-3.5036	1	9	33/88	4.496211	6560	2905.47	MS2	969.161	156	Glioma_grade 2_5.raw
9845502			eHPWEVmPDLYFYR	Unambiguous	N-Term(iTRAQ4plex); M7(Oxidation)	HCD	1.6549144	0	1	1	1.57954	1.524	1.7906	1.7276365	7.521568939	13345.751	13832.056	21080.18	23896.762	2.530517368	8.856201171	3	High	5608	Unique	Used	75967	1	55.769	1	5.3245	-1.2433	1	31	20/52	15.60732	5608	2041.96	MS2	681.324	85	Glioma_grade 2_5.raw
9845502			eHPWEVMPDLYFYR	Unambiguous	N-Term(iTRAQ4plex)	HCD	2.0942392	0	1	1	1.93777	2.9671	1.5242	2.333818	28.03331803	11828.856	7725.295	22921.64	18029.432	29.67815693	16.89486152	3	High	6504	Unique	Used	76760	1	63.253	1	4.5425	-3.7329	1	17	23/52	22.50985	6504	2025.96	MS2	675.991	79	Glioma_grade 2_5.raw
9845502			eHPWEVMPDLYFYR	Unambiguous	N-Term(iTRAQ4plex)	HCD	2.4075935	0	1	1	2.61145	2.1671	2.6974	2.238425	10.90145691	6125.498	7381.6226	15996.446	16523.207	13.15179229	2.290776443	3	High	6416	Unique	Used	76683	1	62.504	1	5.0033	-0.9311	1	35	23/52	27.3364	6416	2025.96	MS2	675.993	79	Glioma_grade 2_5.raw
9845502			fLAAGTHLGGTNLDFQmEQYIYk	Unambiguous	N-Term(iTRAQ4plex); M17(Oxidation); K23(iTRAQ4plex)	HCD	2.3676996	0	1	1	2.29686	2.1393	2.6129	2.43368	8.492756036	3252.0596	3491.5342	7469.527	8497.277	5.022073352	9.102998876	3	High	5747	Unique	Used	76091	1	56.922	1	7.2374	-2.4542	1	39	32/88	4.07811	5747	2921.47	MS2	974.494	137	Glioma_grade 2_5.raw
9910352	NP_064530.1	SH3GLB2	endophilin-B2 [Homo sapiens]	4	6	0.491710305	14.25087437	56.074657	12.91	9.35E-05	395	43.947	5.9858	Unclassified	Biological_process unknown	Molecular function unknown	Cytoplasm	-																								
9910352			tELDAHFENLLAR	Unambiguous	N-Term(iTRAQ4plex)	HCD	0.42712876	0	1	1	0.46105	0.4906	0.3674	0.3910328	0.135372545	139910.94	131470.77	64505.55	51409.375	4.398307787	15.9779151	3	High	5187	Unique	Used	60671	1	50.805	1	3.887	-0.126	1	8	24/48	14.51353	5187	1672.88	MS2	558.297	198	Glioma_grade 2_3.raw
9910352			tELDAHFENLLAR	Unambiguous	N-Term(iTRAQ4plex)	HCD	0.50608844	0	1	1	0.50723	0.5179	0.4945	0.50491816	0.018990313	98157.15	96131.08	49788.703	48538.324	1.474765441	1.798389511	2	High	5195	Unique	Used	60679	1	50.853	1	4.5982	2.1441	1	50	22/24	7.676855	5195	1672.88	MS2	836.944	280	Glioma_grade 2_3.raw
9910352			lLLEGISSTHVNHLR	Unambiguous	N-Term(iTRAQ4plex)	HCD	0.61410934	0	1	1	0.61339	0.6267	0.6018	0.6148472	0.01660046	85051.18	83243.31	52169.31	51181.914	1.519190719	1.351110089	3	High	3300	Unique	Used	90923	1	38.234	1	6.2905	-0.9293	1	21	29/56	36.96544	3300	1833.04	MS2	611.686	115	Glioma_grade 2_7rep.raw
9910352			rLDLDAck	Unambiguous	N-Term(iTRAQ4plex); C7(Methylthio); K8(iTRAQ4plex)	HCD	0.47887674	1	1	1	0.44508	0.4764	0.4812	0.5150554	0.059749572	66839.516	62446.152	29748.674	32163.227	4.805756933	5.515407449	3	High	2354	Unique	Used	79776	1	29.282	1	3.8049	0.6327	1	26	16/28	49.07874	2354	1267.67	MS2	423.23	168	Glioma_grade 2_6.raw
9910352			tELDAHFENLLAR	Unambiguous	N-Term(iTRAQ4plex)	HCD	0.42170987	0	1	1	0.43764	0.4416	0.402	0.40563744	0.049327347	51525.387	51059.945	22549.42	20711.826	0.641645717	6.007109358	3	High	5373	Unique	Used	52834	1	51.157	1	3.7034	-0.6733	1	19	24/48	54.2785	5373	1672.88	MS2	558.297	195	Glioma_grade 2_2.raw
9910352			dFIHTASISFLTPLR	Unambiguous	N-Term(iTRAQ4plex)	HCD	0.50454384	0	1	1	0.48292	0.4962	0.5127	0.52675736	0.037900827	31575.56	30732.607	15248.575	16188.628	1.913257318	4.22886127	2	High	6848	Unique	Used	69858	1	63.539	1	5.1639	2.3107	1	100	21/28	8.598763	6848	1862.03	MS2	931.52	192	Glioma_grade 2_4.raw
9945306	NP_064696.1	MGST1	microsomal glutathione S-transferase 1 [Homo sapiens]	2	4	3.231176853	11.68086423	25.307788	9.03	1.76E-04	155	17.587	9.3931	Enzyme: Glutathione transferase	Xenobiotic metabolism,Metabolism,Energy pathways	Glutathione transferase activity	Microsome	Nuclear membrane, Endoplasmic reticulum																								
9945306			vFANPEDcVAFGk	Unambiguous	N-Term(iTRAQ4plex); C8(Methylthio); K13(iTRAQ4plex)	HCD	3.1137311	0	1	1	2.83698	3.2202	3.02	3.427863	8.150283235	121061.484	106655.65	343448.97	365600.94	8.946593285	4.418252646	2	High	6362	Unique	Used	53768	1	58.326	1	5.6068	-1.088	1	22	20/24	10.39899	6362	1730.85	MS2	865.927	136	Glioma_grade 2_2.raw
9945306			kVFANPEDcVAFGk	Unambiguous	N-Term(iTRAQ4plex); K1(iTRAQ4plex); C9(Methylthio); K14(iTRAQ4plex)	HCD	2.7567987	1	1	1	2.39084	2.6817	2.8238	3.167267	11.66349059	95185.37	84862.96	227573.34	268783.66	8.107874268	11.7415879	3	High	4476	Unique	Used	91822	1	50.213	1	5.3767	-1.3182	1	4	26/52	21.53663	4476	2003.04	MS2	668.353	168	Glioma_grade 2_7rep.raw
9945306			kVFANPEDcVAFGk	Unambiguous	N-Term(iTRAQ4plex); K1(iTRAQ4plex); C9(Methylthio); K14(iTRAQ4plex)	HCD	3.3486226	1	1	1	3.03972	3.5628	3.1659	3.710682	9.445870715	72993.45	62277.465	221879.39	231091.86	11.20321491	2.876209035	2	High	4468	Unique	Used	91816	1	50.128	1	5.8674	-2.2866	1	37	20/26	4.733947	4468	2003.04	MS2	1002.02	183	Glioma_grade 2_7rep.raw
9945306			vFANPEDcVAFGk	Unambiguous	N-Term(iTRAQ4plex); C8(Methylthio); K13(iTRAQ4plex)	HCD	3.6455164	0	1	1	3.49871	3.6407	3.6502	3.7982817	3.355507976	59600.914	57276.54	208526.23	217552.42	2.812485319	2.995916438	2	High	6173	Unique	Used	61585	1	58.519	1	5.1695	0.3931	1	59	19/24	22.46953	6173	1730.85	MS2	865.928	134	Glioma_grade 2_3.raw
9945322	NP_064705.1	SLC17A7	vesicular glutamate transporter 1 [Homo sapiens]	3	4	0.380355358	30.84561216	62.29386	22.68	6.25E-05	560	61.573	7.4741	Transport/cargo protein	Transport	Transporter activity	Plasma membrane	Cytoplasmic vesicle																								
9945322			lMNPLTk	Unambiguous	N-Term(iTRAQ4plex); K7(iTRAQ4plex)	HCD	0.23072143	0	1	1	0.20852	0.2376	0.2246	0.25601736	0.086798197	560001.25	491385.72	116774.234	125803.27	9.229428924	5.263878536	2	High	3083	Unique	Used	66392	1	33.77	1	3.1094	7.1044	1	23	12-Nov	60.84133	3083	1104.68	MS2	552.842	353	Glioma_grade 2_4.raw
9945322			qEGAETLELSADGRPVTTQTR	Unambiguous	N-Term(iTRAQ4plex)	HCD	0.3149248	0	1	1	0.3449	0.3539	0.277	0.2841666	0.12705584	181294.19	176691.17	62528.74	50209.73	1.818413276	15.45320867	3	High	2270	Unique	Used	49913	1	28.687	1	3.4078	-0.8945	1	5	25/80	5.514724	2270	2403.22	MS2	801.745	109	Glioma_grade 2_2.raw
9945322			kYIEDAIGESAk	Unambiguous	N-Term(iTRAQ4plex); K1(iTRAQ4plex); K12(iTRAQ4plex)	HCD	0.44578594	1	1	1	0.39147	0.4217	0.4682	0.5042919	0.111672941	33922.62	31493.65	13279.661	15881.992	5.251113089	12.62017552	3	High	2138	Unique	Used	79574	1	27.672	1	3.9556	-0.4494	1	29	17/44	51.79155	2138	1755.98	MS2	586	154	Glioma_grade 2_6.raw
9945322			kYIEDAIGESAk	Unambiguous	N-Term(iTRAQ4plex); K1(iTRAQ4plex); K12(iTRAQ4plex)	HCD	0.47008067	1	1	1	0.47123	0.4937	0.4475	0.46888125	0.04018412	15419.8125	14716.833	7266.2075	6900.4473	3.29885137	3.651271544	3	High	2565	Unique	Used	95211	1	36.411	1	5.5741	-4.7247	1	100	24/44	61.39925	2565	1755.98	MS2	585.997	157	Glioma_grade 2_8rep.raw
9945439	NP_002679.2	SEPT5	septin-5 isoform 1 [Homo sapiens]	5	8	0.270779669	28.86839007	71.445773	20.33	9.77E-05	369	42.75	6.6685	GTPase	Signal transduction	GTP binding	Cytoplasm	Nucleus, Membrane fraction, Synaptic vesicle																								
9945439			vNIVPLIAk	Unambiguous	N-Term(iTRAQ4plex); K9(iTRAQ4plex)	HCD	0.21546674	0	1	2	0.20515	0.2209	0.2104	0.22657782	0.045184197	366353.56	340185.9	75157.17	77078.58	5.237734304	1.78491851	2	High	4821	Unique	Used	60330	1	47.971	1	3.4066	8.6462	1	17	14/16	58.84181	4821	1254.85	MS2	627.928	17	Glioma_grade 2_3.raw
9945439			vNIVPLIAk	Unambiguous	N-Term(iTRAQ4plex); K9(iTRAQ4plex)	HCD	0.23873878	0	1	2	0.2151	0.2367	0.2406	0.2647469	0.085028479	244979.11	222658.23	52695.09	58948.08	6.75020759	7.920827815	2	High	4940	Unique	Used	52427	1	47.971	1	3.2076	-9.5451	1	25	14/16	58.43877	4940	1254.83	MS2	627.917	46	Glioma_grade 2_2.raw
9945439			qYVGFATLPNQVHR	Unambiguous	N-Term(iTRAQ4plex)	HCD	0.30839726	0	1	2	0.30226	0.3098	0.3071	0.31468254	0.016818978	78512.125	76612.234	23731.393	24108.531	1.732062995	1.114871492	2	High	3734	Unique	Used	66995	1	38.846	1	5.1496	0.4092	1	75	20/26	18.34758	3734	1773.95	MS2	887.48	204	Glioma_grade 2_4.raw
9945439			lYPWGIVEVENQAHcDFVk	Unambiguous	N-Term(iTRAQ4plex); C15(Methylthio); K19(iTRAQ4plex)	HCD	0.204065	0	1	1	0.19524	0.2064	0.2018	0.21339905	0.03746588	67694.414	64021.023	13216.484	13662.025	3.944077847	2.344215316	3	High	6777	Unique	Used	83528	1	68.778	1	8.1916	1.3565	1	48	33/72	25.1445	6777	2581.29	MS2	861.1	157	Glioma_grade 2_6.raw
9945439			sTLVHSLFLTDLYk	Unambiguous	N-Term(iTRAQ4plex); K14(iTRAQ4plex)	HCD	0.2304506	0	1	2	0.18839	0.1788	0.2849	0.2703681	0.237354734	63476.516	66877.38	11958.625	18081.51	3.689608167	28.82499366	3	High	5838	Unique	Used	76173	1	57.657	1	5.7337	-1.0095	1	10	26/52	45.26907	5838	1925.1	MS2	642.37	139	Glioma_grade 2_5.raw
9945439			sTLVHSLFLTDLYk	Unambiguous	N-Term(iTRAQ4plex); K14(iTRAQ4plex)	HCD	0.42319393	0	1	2	0.40239	0.3958	0.4511	0.44365966	0.066600249	52476.777	53356.402	21115.875	23672.082	1.175413624	8.071416626	3	High	5512	Unique	Used	82503	1	56.661	1	5.8244	-0.6291	1	32	21/52	53.32655	5512	1925.1	MS2	642.371	145	Glioma_grade 2_6.raw
9945439			qYVGFATLPNQVHR	Unambiguous	N-Term(iTRAQ4plex)	HCD	0.39850664	0	1	2	0.39405	0.4106	0.3869	0.4031525	0.026005147	48737.133	46772.047	19204.773	18856.268	2.909721633	1.294921223	2	High	3418	Unique	Used	59031	1	36.989	1	4.3936	1.3726	1	87	20/26	8.020684	3418	1773.95	MS2	887.481	224	Glioma_grade 2_3.raw
9945439			mESPIPILPLPTPDAETEk	Unambiguous	N-Term(iTRAQ4plex); M1(Oxidation); K19(iTRAQ4plex)	HCD	0.30282056	0	1	1	0.29306	0.3217	0.2856	0.3135358	0.055749017	41661.535	37955.656	12209.266	11900.457	6.582629048	1.811393171	2	High	6379	Unique	Used	61779	1	60.091	1	4.3626	1.5514	1	45	20/36	0	6379	2382.28	MS2	1191.64	149	Glioma_grade 2_3.raw
10567816	NP_066268.1	GNAO1	guanine nucleotide-binding protein G(o) subunit alpha isoform a [Homo sapiens]	2	6	0.355933428	33.16829186	216.6299	48.87	2.61E-04	354	40.025	5.5288	G protein	Cell communication,Signal transduction	GTPase activity	Plasma membrane	Cytoplasm																								
10567816			mHESLMLFDSIcNNk	Unambiguous	N-Term(iTRAQ4plex); C12(Methylthio); K15(iTRAQ4plex)	HCD	0.37057507	0	1	1	0.37025	0.3566	0.3851	0.3708898	0.031337843	139113.58	144430.36	51506.566	53567.75	2.651815583	2.774183529	2	High	6251	Unique	Used	83105	1	63.669	1	6.2343	0.5021	1	55	20/28	11.0441	6251	2115.99	MS2	1058.5	71	Glioma_grade 2_6.raw
10567816			mHESLMLFDSIcNNk	Unambiguous	N-Term(iTRAQ4plex); M1(Oxidation); C12(Methylthio); K15(iTRAQ4plex)	HCD	0.55740595	0	1	1	0.5307	0.6343	0.4931	0.58932495	0.111201809	68416.234	57239.723	36308.582	33732.797	12.57876969	5.200797204	3	High	5754	Unique	Used	82711	1	58.781	1	6.0876	0.8158	1	13	21/56	16.93277	5754	2131.99	MS2	711.335	62	Glioma_grade 2_6.raw
10567816			mHESLMLFDSIcNNk	Unambiguous	N-Term(iTRAQ4plex); C12(Methylthio); K15(iTRAQ4plex)	HCD	0.58736324	0	1	1	0.62631	0.5811	0.5941	0.55122185	0.052900518	43137.145	46490.7	27017.412	25626.69	5.291483872	3.735989103	3	High	6236	Unique	Used	83092	1	63.542	1	5.7871	0.1784	1	18	19/56	26.37337	6236	2115.99	MS2	706.003	83	Glioma_grade 2_6.raw
10567816			fFIDTSIILFLNk	Unambiguous	N-Term(iTRAQ4plex); K13(iTRAQ4plex)	HCD	0.34129176	0	1	1	0.2785	0.3071	0.3723	0.4105366	0.175858058	27207.682	24670.387	7577.4614	10128.096	6.916751278	20.37293694	3	High	8356	Unique	Used	71170	1	76.87	1	4.3468	-0.887	1	32	19/48	16.91078	8356	1859.09	MS2	620.368	83	Glioma_grade 2_4.raw
10567816			fFIDTSIILFLNk	Unambiguous	N-Term(iTRAQ4plex); K13(iTRAQ4plex)	HCD	0.28849828	0	1	1	0.27692	0.2867	0.2902	0.30048445	0.033706758	18056.969	17440.598	5000.359	5240.6284	2.455605556	3.317963697	2	High	8358	Unique	Used	71172	1	76.882	1	3.811	0.3021	1	64	16/24	44.56022	8358	1859.09	MS2	930.05	96	Glioma_grade 2_4.raw

10567816			mHESLMLFDSIcNNk	Unambiguous	N-Term(iTRAQ4plex); C12(Methylthio); K15(iTRAQ4plex)	HCD	0.28330705	0	1	1	0.25112	0.2738	0.292	0.31839842	0.100320448	13436.782	12323.727	3374.279	3923.8547	6.110506111	10.64953645	4	High	6277	Unique	Used	83126	1	63.918	1	5.7466	0.6624	1	55	20/84	31.36107	6277	2116	MS2	529.754	79	Glioma_grade2_6.raw
11038643	NP_067586.1	AP2S1	AP-2 complex subunit sigma isoform AP17delta [Homo sapiens]	2	10	0.47632134	16.95101573	48.208934	21.15	3.54E-04	104	12.409	5.8462	Adapter molecule	Cell communication,Signal transduction	Receptor signaling complex scaffold activity	Plasma membrane	-																								
11038643			wYmQFDDDEk	Unambiguous	N-Term(iTRAQ4plex); M3(Oxidation); K10(iTRAQ4plex)	HCD	0.5336844	0	1	2	0.49451	0.5412	0.5268	0.5765631	0.063620525	177545	162225.47	87796.97	93533.23	6.376389065	4.473770331	2	High	4154	Unique	Used	51688	1	42.22	1	3.6052	1.8685	1	49	13/18	37.85875	4154	1680.75	MS2	840.878	34	Glioma_grade2_2.raw
11038643			wYMQFDDDEk	Unambiguous	N-Term(iTRAQ4plex); K10(iTRAQ4plex)	HCD	0.40399706	0	1	2	0.39798	0.3744	0.4354	0.40965903	0.062705784	156555.28	166399.17	62305.723	68166.92	4.310627317	6.353043901	2	High	5410	Unique	Used	52869	1	51.418	1	4.3162	-1.0214	1	37	14/18	21.49338	5410	1664.75	MS2	832.878	50	Glioma_grade2_2.raw
11038643			IIEEVHAVVTVR	Unambiguous	N-Term(iTRAQ4plex)	HCD	0.5526204	0	1	2	0.53442	0.5599	0.5457	0.5716892	0.02944183	134039.1	127945.73	71632.97	73145.19	3.289246364	1.477157904	2	High	5040	Unique	Used	60532	1	49.708	1	4.884	2.6	1	52	19/22	3.278808	5040	1508.9	MS2	754.952	224	Glioma_grade2_3.raw
11038643			IIEEVHAVVTVR	Unambiguous	N-Term(iTRAQ4plex)	HCD	0.63109344	0	1	2	0.57736	0.5992	0.6618	0.6868581	0.081553596	99398.25	95769.09	57389.043	65779.78	2.629747012	9.634170218	2	High	4946	Unique	Used	60445	1	48.961	1	4.7528	-1.6877	1	68	19/22	33.60381	4946	1508.89	MS2	754.948	238	Glioma_grade2_3.raw
11038643			IIEEVHAVVTVR	Unambiguous	N-Term(iTRAQ4plex)	HCD	0.43036637	0	1	2	0.40727	0.4281	0.4326	0.45463714	0.045111138	58980.434	56116.176	24021.21	25512.498	3.519367347	4.257706116	2	High	5104	Unique	Used	68262	1	49.57	1	4.9745	1.7101	1	84	19/22	14.74219	5104	1508.89	MS2	754.951	233	Glioma_grade2_4.raw
11038643			IIEEVHAVVTVR	Unambiguous	N-Term(iTRAQ4plex)	HCD	0.44023532	0	1	2	0.40442	0.4415	0.4391	0.47933716	0.069410139	54301.84	49742.84	21960.55	23843.592	6.196760498	5.813936074	3	High	5168	Unique	Used	68320	1	50.095	1	3.8655	0.063	1	29	19/44	11.43387	5168	1508.89	MS2	503.635	183	Glioma_grade2_4.raw
11038643			IIEEVHAVVTVR	Unambiguous	N-Term(iTRAQ4plex)	HCD	0.48029366	0	1	2	0.41984	0.5615	0.4196	0.5611498	0.166670675	49849.656	37274.145	20928.63	20916.377	20.41285849	0.041410816	3	High	5054	Unique	Used	60545	1	49.82	1	4.2584	0.427	1	26	21/44	8.089904	5054	1508.89	MS2	503.636	201	Glioma_grade2_3.raw
11038643			IIEEVHAVVTVR	Unambiguous	N-Term(iTRAQ4plex)	HCD	0.34542853	0	1	2	0.36673	0.34	0.3513	0.32568043	0.05026099	35279.355	38054.61	12938.001	12393.643	5.351958346	3.039038707	3	High	4937	Unique	Used	60437	1	48.885	1	4.7788	0.245	1	56	20/44	10.37108	4937	1508.89	MS2	503.636	180	Glioma_grade2_3.raw
11038643			IIEEVHAVVTVR	Unambiguous	N-Term(iTRAQ4plex)	HCD	0.48265097	0	1	2	0.49936	0.512	0.454	0.46551955	0.05674303	24675.264	24067.172	12321.845	11203.739	1.764318783	6.721366277	3	High	5076	Unique	Used	68237	1	49.334	1	4.7112	0.3057	1	72	20/44	7.744321	5076	1508.89	MS2	503.636	177	Glioma_grade2_4.raw
11038643			IIEEVHAVVTVR	Unambiguous	N-Term(iTRAQ4plex)	HCD	0.472349	0	1	2	0.44382	0.5019	0.4462	0.5046163	0.071022249	15901.337	14060.227	7057.2944	7095.02	8.690209669	0.376984666	3	High	5093	Unique	Used	52571	1	49.105	1	3.7759	-6.5507	1	56	23/44	56.60235	5093	1508.88	MS2	503.632	222	Glioma_grade2_2.raw
11321585	NP_002065.1	GNB1	guanine nucleotide-binding protein G(I)/G(S)/G(T) subunit beta-1 [Homo sapiens]	3	17	0.498079091	15.34384675	253.10217	39.12	1.97E-04	340	37.353	5.9985	G protein	Cell communication,Signal transduction	GTPase activity	Plasma membrane	Mitochondrion																								
11321585			lFVSGAcDASAk	Unambiguous	N-Term(iTRAQ4plex); C7(Methylthio); K12(iTRAQ4plex)	HCD	0.4305992	0	1	1	0.41077	0.4189	0.4421	0.45082033	0.043885861	676604	663503.8	277928.38	299121	1.382461956	5.193817317	2	High	4365	Unique	Used	59910	1	44.346	1	4.8558	0.9709	1	16	20/22	14.23178	4365	1502.76	MS2	751.884	122	Glioma_grade2_3.raw
11321585			lFVSGAcDASAk	Unambiguous	N-Term(iTRAQ4plex); C7(Methylthio); K12(iTRAQ4plex)	HCD	0.44162667	0	1	1	0.41423	0.4385	0.4446	0.4706265	0.05241677	649195.44	613328.7	268915.62	288648.72	4.017604776	5.005129569	2	High	4615	Unique	Used	52121	1	45.637	1	5.2169	0.4023	1	16	20/22	13.20728	4615	1502.76	MS2	751.883	127	Glioma_grade2_2.raw
11321585			lFVSGAcDASAk	Unambiguous	N-Term(iTRAQ4plex); C7(Methylthio); K12(iTRAQ4plex)	HCD	0.46604553	0	1	1	0.45619	0.4825	0.4505	0.47647166	0.033153223	589389.44	557263.25	268872.22	265520.12	3.962254133	0.887098285	2	High	4572	Unique	Used	60099	1	46.019	1	5.1419	-0.8974	1	21	20/22	17.85053	4572	1502.76	MS2	751.882	120	Glioma_grade2_3.raw
11321585			lFVSGAcDASAk	Unambiguous	N-Term(iTRAQ4plex); C7(Methylthio); K12(iTRAQ4plex)	HCD	0.45966566	0	1	1	0.43784	0.4613	0.4581	0.48265874	0.039882374	564033.44	535358.4	246957.25	258395.4	3.68864212	3.200930451	2	High	4469	Unique	Used	60005	1	45.192	1	4.8188	2.758	1	27	20/22	9.459979	4469	1502.76	MS2	751.885	136	Glioma_grade2_3.raw
11321585			lFVSGAcDASAk	Unambiguous	N-Term(iTRAQ4plex); C7(Methylthio); K12(iTRAQ4plex)	HCD	0.5894692	0	1	1	0.56417	0.5989	0.5805	0.61632645	0.038272433	443507.03	417757.62	250213.89	257475.08	4.228103968	2.022670175	2	High	4727	Unique	Used	52226	1	46.453	1	5.1208	1.5395	1	25	20/22	35.48549	4727	1502.76	MS2	751.884	127	Glioma_grade2_2.raw
11321585			lFVSGAcDASAk	Unambiguous	N-Term(iTRAQ4plex); C7(Methylthio); K12(iTRAQ4plex)	HCD	0.47947723	0	1	1	0.47016	0.4662	0.4928	0.4887173	0.027607256	365489.06	368562.56	171838.14	180122.9	0.592136202	3.328896844	2	High	4264	Unique	Used	59815	1	43.578	1	5.1202	-0.7349	1	34	20/22	14.55029	4264	1502.76	MS2	751.882	125	Glioma_grade2_3.raw
11321585			lFVSGAcDASAk	Unambiguous	N-Term(iTRAQ4plex); C7(Methylthio); K12(iTRAQ4plex)	HCD	0.49948356	0	1	1	0.46689	0.4905	0.508	0.5337229	0.056443612	225054.14	214215.89	105076.24	114331.92	3.489334372	5.965825603	2	High	4827	Unique	Used	60336	1	48.008	1	4.9388	3.9764	1	52	20/22	41.56934	4827	1502.76	MS2	751.886	132	Glioma_grade2_3.raw
11321585			lFVSGAcDASAk	Unambiguous	N-Term(iTRAQ4plex); C7(Methylthio); K12(iTRAQ4plex)	HCD	0.4370027	0	1	1	0.43038	0.4551	0.4199	0.4440013	0.03527746	204482.42	193368.75	88006.055	85855.98	3.950498082	1.748895453	2	High	4440	Unique	Used	51957	1	44.331	1	4.5024	1.4583	1	29	19/22	15.86491	4440	1502.76	MS2	751.884	134	Glioma_grade2_2.raw
11321585			lFVSGAcDASAk	Unambiguous	N-Term(iTRAQ4plex); C7(Methylthio); K12(iTRAQ4plex)	HCD	0.4980791	0	1	1	0.4677	0.491	0.5048	0.52996826	0.052267425	196047.72	186754.14	91691.836	98973.77	3.433396818	5.401189045	2	High	4925	Unique	Used	60426	1	48.787	1	5.0719	0.3211	1	59	20/22	28.21245	4925	1502.76	MS2	751.883	130	Glioma_grade2_3.raw
11321585			lFVSGAcDASAk	Unambiguous	N-Term(iTRAQ4plex); C7(Methylthio); K12(iTRAQ4plex)	HCD	0.5904099	0	1	1	0.56334	0.598	0.5833	0.61914474	0.039865007	123627.836	116470.99	69644.32	72112.41	4.215476121	2.462250894	2	High	5223	Unique	Used	60705	1	51.078	1	4.9011	8.2004	1	100	19/22	47.30175	5223	1502.77	MS2	751.889	160	Glioma_grade2_3.raw
11321585			lFVSGAcDASAk	Unambiguous	N-Term(iTRAQ4plex); C7(Methylthio); K12(iTRAQ4plex)	HCD	0.6069459	0	1	1	0.55182	0.6118	0.6025	0.66806465	0.078273025	122407.69	110400.2	67547.31	73754.48	7.294063448	6.212422361	2	High	4867	Unique	Used	52359	1	47.433	1	4.2276	3.2454	1	50	19/22	45.8656	4867	1502.76	MS2	751.885	140	Glioma_grade2_2.raw
11321585			eLAGHTGYLSccR	Unambiguous	N-Term(iTRAQ4plex); C11(Methylthio); C12(Methylthio)	HCD	0.64725876	0	1	1	0.63116	0.6088	0.6872	0.6627913	0.053310815	70680.61	73279.73	44610.42	48569.164	2.553280129	6.008300547	2	High	4131	Unique	Used	74649	1	43.68	1	4.7142	0.5818	1	62	20/24	20.70043	4131	1645.71	MS2	823.358	35	Glioma_grade2_5.raw
11321585			eLAGHTGYLSccR	Unambiguous	N-Term(iTRAQ4plex); C11(Methylthio); C12(Methylthio)	HCD	0.5646832	0	1	1	0.53421	0.5248	0.6052	0.5946183	0.072706152	55563.73	56556.605	29682.877	33629.594	1.252348462	8.815799826	2	High	4626	Unique	Used	67826	1	45.727	1	4.734	3.0295	1	100	20/24	25.78615	4626	1645.71	MS2	823.36	37	Glioma_grade2_4.raw
11321585			eLAGHTGYLSccR	Unambiguous	N-Term(iTRAQ4plex); C11(Methylthio); C12(Methylthio)	HCD	0.64856213	0	1	1	0.66823	0.6182	0.6814	0.6303695	0.046351291	41109.125	44439.4	27470.295	28013.242	5.505320017	1.383911431	3	High	4114	Unique	Used	74633	1	43.55	1	3.8427	-0.4278	1	18	18/48	19.88124	4114	1645.71	MS2	549.241	22	Glioma_grade2_5.raw
11321585			lFVSGAcDASAk	Unambiguous	N-Term(iTRAQ4plex); C7(Methylthio); K12(iTRAQ4plex)	HCD	0.48700923	0	1	1	0.37357	0.4849	0.4886	0.63426757	0.215809101	35984.633	27720.775	13442.732	17582.389	18.34516162	18.86973808	3	High	4578	Unique	Used	60105	1	46.055	1	4.3404	-4.2977	1	36	16/44	10.00715	4578	1502.75	MS2	501.589	125	Glioma_grade2_3.raw
11321585			lFVSGAcDASAk	Unambiguous	N-Term(iTRAQ4plex); C7(Methylthio); K12(iTRAQ4plex)	HCD	0.52583766	0	1	1	0.47032	0.5112	0.5393	0.58617866	0.092455065	9858.899	9070.526	4636.8555	5316.949	5.889918943	9.66261143	2	High	8596	Unique	Used	63763	1	78.738	1	2.9871	-0.7349	1	100	14/22	43.98504	8596	1502.76	MS2	751.882	114	Glioma_grade2_3.raw
11321585			aDQELMTYSHDNIIcGITSVSFSk	Unambiguous	N-Term(iTRAQ4plex); C15(Methylthio); K24(iTRAQ4plex)	HCD	0.3891802	0	1	1	0.42652	0.4039	0.3736	0.35381567	0.082587765	4549.16	4803.868	1940.3296	1699.684	3.851282259	9.349532959	3	High	7477	Unique	Used	70417	1	68.917	1	6.0474	-0.4267	1	59	28/92	5.52157	7477	2993.43	MS2	998.481	57	Glioma_grade2_4.raw
11321640	NP_066301.1	SNTB1	beta-1-syntrophin [Homo sapiens]	2	3	2.560838223	26.57233887	30.74003	11.15	8.89E-04	538	58.025	8.6313	Adapter molecule	Cell communication,Signal transduction	Receptor signaling complex scaffold activity	Cytoplasm	-																								
11321640			dSATAQAWFSAIHSNVNDLLTR	Unambiguous	N-Term(iTRAQ4plex)	HCD	2.5608382	0	1	1	2.29547	2.7185	2.4277	2.8751175	10.26886869	3130.144	2643.0186	7185.1465	7598.9893	11.93278962	3.958717022	3	High	8398	Unique	Used	55632	1	74.476	1	7.3835	1.2946	1	100	28/84	38.33324	8398	2561.29	MS2	854.435	155	Glioma_grade2_2.raw
11321640			gAGAGHPGAGGAQPPDSPAGVR	Unambiguous	N-Term(iTRAQ4plex)	HCD	1.5705425	0	1	1	1.61091	1.5325	1.6105	1.5321407	2.87924128	2492.059	2619.5142	4014.478	4013.4644	3.526289566	0.017855719	3	High	1011	Unique	Used	64472	1	18.242	1	6.1539	0.9469	1	100	29/84	22.83511	1011	2028.01	MS2	676.676	123	Glioma_grade2_4.raw
11321640			gAGAGHPGAGGAQPPDSPAGVR	Unambiguous	N-Term(iTRAQ4plex)	HCD	2.6563523	0	1	1	2.32462	3.3627	2.1681	3.1362092	21.46862907	2118.6926	1464.6633	4925.1655	4593.49	25.81203576	4.927796687	2	High	1036	Unique	Used	64494	1	18.469	1	5.1293	-4.3434	1	100	26/42	15.40607	1036	2028	MS2	1014.51	139	Glioma_grade2_4.raw
11415026	NP_000971.1	RPL18A	60S ribosomal protein L18a [Homo sapiens]	3	7	2.21247673	17.1344216	26.103558	15.34	9.69E-05	176	20.749	10.711	RNA binding protein	Regulation of nucleobase, nucleoside, nucleotide and nucleic acid metabolism	RNA binding	Cytoplasm	Mitochondrion, Nucleolus																								
11415026			iFAPNHVVAk	Unambiguous	N-Term(iTRAQ4plex); K10(iTRAQ4plex)	HCD	2.2467082	0	1	1	2.08514	2.3059	2.1932	2.4253802	6.498441454	453023	409646.1	944618.06	993547.56	7.110976883	3.570219273	2	High	1457	Unique	Used	78940	1	22.651	1	4.2055	-7.4884	1	10	16/18	37.30491	1457	1383.82	MS2	692.416	160	Glioma_grade2_6.raw
11415026			iFAPNHVVAk	Unambiguous	N-Term(iTRAQ4plex); K10(iTRAQ4plex)	HCD	2.1261172	0	1	1	1.97924	2.045	2.2046	2.2778735	6.500701807	66495.984	64357.19	131611.64	146597.55	2.311530847	7.617748776	3	High	1444	Unique	Used	78928	1	22.546	1	3.6226	-8.9316	1	24	15/36	58.672	1444	1383.82	MS2	461.946	151	Glioma_grade2_6.raw
11415026			fWYFVSQLk	Unambiguous	N-Term(iTRAQ4plex); K9(iTRAQ4plex)	HCD	2.2124767	0	1	1	2.15672	2.2605	2.1667	2.2709193	2.724945996	51378.754	49020.832	110809.39	111322.36	3.321333687	0.32658507	2	High	7237	Unique	Used	77382	1	69.898	1	3.9235	-1.2918	1	61	15/16	27.22016	7237	1505.84	MS2	753.422	239	Glioma_grade2_5.raw
11415026			ikFPLPHR	Unambiguous	N-Term(iTRAQ4plex); K2(iTRAQ4plex)	HCD	1.6016266	1	1	1	1.53194	1.5426	1.6602	1.6717988	4.65768727	24569.105	24398.979	37638.406	40790.184	0.491329202	5.683242799	3	High	2306	Unique	Used	85631	1	32.916	1	3.671	0.2101	1	100	18/28	38.76444	2306	1295.82	MS2	432.611	89	Glioma_grade2_1rep.raw
11415026			fWYFVSQLk	Unambiguous	N-Term(iTRAQ4plex); K9(iTRAQ4plex)	HCD	1.5555295	0	1	1	1.4921	1.5139	1.5966	1.619882	3.994606227	22025.105	21708.303	32863.72	35164.887	1.024447226	4.783783946	2	High	7756	Unique	Used	70656	1	71.476	1	3.152	2.8425	1	100	16-Dec	67.47713	7756	1505.84	MS2	753.426	287	Glioma_grade2_4.raw
11415026			fWYFVSQLk	Unambiguous	N-Term(iTRAQ4plex); K9(iTRAQ4plex)	HCD	2.448821	0	1	1	2.28809	2.4042	2.4913	2.6177013	5.681745101	15116.145	14386.422	34587.152	37659.355	3.497947021	6.013787143	3	High	7676	Unique	Used	70587	1	70.75	1	3.9146	-0.8752	1	42	17/32	6.393466	7676	1505.84	MS2	502.618	259	Glioma_grade2_4.raw
11415026			fWYFVSQLk	Unambiguous	N-Term(iTRAQ4plex); K9(iTRAQ4plex)	HCD	2.3497555	0	1	1	2.30192	2.163	2.5485	2.3947089	6.885082897	4188.064	4457.048	9640.566	10673.333	4.400183836	7.189920055	3	High	7220	Unique	Used	77369	1	69.716	1	3.6144	-3.8543	1	100	16/32	0	7220	1505.83	MS2	502.616	256	Glioma_grade2_5.raw
11641247	NP_071738.1	GLIPR2	Golgi-associated plant pathogenesis-related protein 1 [Homo sapiens]	2	2	4.412676811	15.04657715	16.132192	38.96	9.35E-05	154	17.208	9.4077	Unclassified	Biological_process unknown	Molecular function unknown	-	-																								
11641247			gQcGENLAWASYDQTGk	Unambiguous	N-Term(iTRAQ4plex); C3(Methylthio); K17(iTRAQ4plex)	HCD	4.882165	0	1	1	4.68986	5.0407	4.7347	5.0888486	4.198883494	12206.053	11356.609	57244.734	57792.07	5.098300121	0.672871609	2	High	5024	Unique	Used	52507	1	48.576	1	6.9546	-0.8966	1	90	22/32	0	5024	2161.99	MS2	1081.5	68	Glioma_grade2_2.raw
11641247			nYNFQQPGFTSGTGHFTAMVWk	Unambiguous	N-Term(iTRAQ4plex); K22(iTRAQ4plex)	HCD	3.9431882	0	1	1	4.02833	3.1729	4.9211	3.8761296	17.96409072	11908.139	15118.571	47969.867	58601.54	16.79907201	14.10833972	3	High	5715	Unique	Used	76062	1	56.671	1	5.7432	-0.2727	1	17	26/84	28.86558	5715	2806.36	MS2	936.126	89	Glioma_grade2_5.raw
12667788	NP_002464.1	MYH9	myosin-9 [Homo sapiens]	39	79	2.785342932	29.35304789	728.18553	37.14	1.00E-04	1960	226.39	5.605	Structural protein	Cell growth and/or maintenance	Structural molecule activity	Cytoplasm	Cytosol, Mitochondrion, Nucleus																								
12667788			eQEVNILk	Unambiguous	N-Term(iTRAQ4plex); K8(iTRAQ4plex)	HCD	1.9260225	0	1	1	1.81935	1.9917	1.8661	2.0427954	5.422550221	1397143.9	1276266.4	2541897.8	2607151	6.394327122	1.792213747	2	High	1706	Unique	Used	49380	1	24.747	1	3.3461	-0.5217	1	2	14/14	23.41347	1706	1260.74	MS2	630.873	304	Glioma_grade2_2.raw
12667788			lQEMEGTVk	Unambiguous	N-Term(iTRAQ4plex); K9(iTRAQ4plex)	HCD	2.785343	0	1	1	2.81006	2.7428	2.8289	2.7612183	1.451002505	709954.9	727364.1	1995015.2	2008411.1	1.712934063	0.473211246	2	High	1765	Unique	Used	49435	1	25.173	1	3.866	-0.116	1	4	15/16	11.12257	1765	1322.72	MS2	661.865	261	Glioma_grade2_2.raw
12667788			lVWVPSDk	Unambiguous	N-Term(iTRAQ4plex); K8(iTRAQ4plex)	HCD	2.6423497	0	1	1	2.60005	2.5352	2.7522	2.6835966	3.592627952	548414.44	562440.6	1425903.8	1509363.8	1.785650241	4.021107442	2	High	3861	Unique	Used	59440	1	40.454	1	3.0681	0.7036	1	4	14-Dec	17.12281	3861	1231.73	MS2	616.369	220	Glioma_grade2_3.raw
12667788			dLGEELEALk	Unambiguous	N-Term(iTRAQ4plex); K10(iTRAQ4plex)	HCD	2.9968402	0	1	5	2.87511	3.0105	2.9838	3.124301	3.412568331	485609	463772.28	1396179.8	1448964.2	3.25283279	2.623713048	2	High	6024	Unique	Used	53448	1	55.893	1	4.7877	-2.3241	1	6	18/18	22.03769	6024	1404.78	MS2	702.893	274	Glioma_grade2_2.raw
12667788			sGFEPASLk	Unambiguous	N-Term(iTRAQ4plex); K9(iTRAQ4plex)	HCD	1.7179412	0	1	1	1.69294	1.7085	1.7273	1.7431724	1.275038921	440212.47	436210.7	745253	760390.44	0.645733431	1.421822218	2	High	1851	Unique	Used	49517	1	25.746	1	3.3845	0.082	1	5	14/16	30.6343	1851	1223.69	MS2	612.347	307	Glioma_grade2_2.raw

12667788			fVSELWk	Unambiguous	N-Term(iTRAQ4plex); K7(iTRAQ4plex)	HCD	3.2976294	0	1	1	3.36949	3.1187	3.4909	3.2311149	4.909733726	316912.9	342397.16	1067835.6	1106324.6	5.466348582	2.503571991	2	High	5275	Unique	Used	68420	1	50.896	1	3.0225	-1.4091	1	11	12-Dec	13.25539	5275	1196.69	MS2	598.849	218	Glioma_grade2_4.raw
12667788			hEAMITDLEER	Unambiguous	N-Term(iTRAQ4plex)	HCD	2.7665431	0	1	1	2.49811	3.0117	2.5632	3.0901654	10.86557315	314978.03	261267.86	786848.25	807360.94	13.18146509	1.819668622	2	High	2050	Unique	Used	65428	1	26.072	1	3.485	1.3617	1	9	18/20	12.30268	2050	1487.73	MS2	744.369	111	Glioma_grade2_4.raw
12667788			aLEQQVEEMk	Unambiguous	N-Term(iTRAQ4plex); K10(iTRAQ4plex)	HCD	2.6841848	0	1	1	2.62525	2.6309	2.7374	2.7432435	2.417065619	252079.83	251538.97	661773.4	690032.6	0.15187907	2.956381604	2	High	3328	Unique	Used	50909	1	36.221	1	4.8934	1.5754	1	11	17/18	8.622993	3328	1492.79	MS2	746.901	211	Glioma_grade2_2.raw
12667788			aLEQQVEEMk	Unambiguous	N-Term(iTRAQ4plex); K10(iTRAQ4plex)	HCD	2.1457489	0	1	1	2.01399	2.1703	2.123	2.2877364	5.279482574	207275.95	192346.69	417451.34	440038.5	5.283274733	3.725183264	2	High	3428	Unique	Used	51004	1	36.924	1	4.4069	2.3114	1	14	17/18	11.89995	3428	1492.8	MS2	746.901	249	Glioma_grade2_2.raw
12667788			tEMEDLMSSk	Unambiguous	N-Term(iTRAQ4plex); K10(iTRAQ4plex)	HCD	2.107086	0	1	1	2.01306	2.05	2.1631	2.2028434	4.280073963	201837.8	198196.84	406310.78	436596.6	1.287162285	5.081295811	2	High	3584	Unique	Used	51150	1	38.08	1	4.448	-2.2026	1	17	17/18	9.001565	3584	1458.7	MS2	729.855	87	Glioma_grade2_2.raw
12667788			kLEMDLk	Unambiguous	N-Term(iTRAQ4plex); K1(iTRAQ4plex); K7(iTRAQ4plex)	HCD	2.2564626	1	1	1	2.03975	2.3195	2.2011	2.5028934	8.617788859	191946.16	168799.42	391521.94	422487	9.074105255	5.379696175	3	High	1560	Unique	Used	89439	1	23.049	1	3.4296	-3.2735	1	6	15/24	33.13493	1560	1308.79	MS2	436.934	195	Glioma_grade2_7rep.raw
12667788			dLEAHIDSANk	Unambiguous	N-Term(iTRAQ4plex); K11(iTRAQ4plex)	HCD	2.382872	0	1	1	2.12592	2.7566	2.0946	2.7160482	14.94032812	160652.5	123896.94	341534.88	336510.06	18.26755008	1.048037995	3	High	1265	Unique	Used	64703	1	20.232	1	3.4391	0.7452	1	2	21/40	9.304198	1265	1500.79	MS2	500.935	207	Glioma_grade2_4.raw
12667788			hEAMITDLEER	Unambiguous	N-Term(iTRAQ4plex)	HCD	3.0718455	0	1	1	2.81097	3.0961	3.0498	3.359185	7.298217735	156954.39	142499.34	441194	478681.66	6.826606486	5.763339492	2	High	1839	Unique	Used	57557	1	25.226	1	3.8037	0.5412	1	40	16/20	52.00626	1839	1487.73	MS2	744.368	120	Glioma_grade2_3.raw
12667788			fVSELWk	Unambiguous	N-Term(iTRAQ4plex); K7(iTRAQ4plex)	HCD	3.027741	0	1	1	2.86207	3.0381	3.018	3.2036057	4.609941675	127438.2	120055.055	364736.44	384609.06	4.218839738	3.750490091	2	High	5233	Unique	Used	52702	1	50.126	1	3.088	-2.3271	1	30	12-Dec	19.26477	5233	1196.69	MS2	598.848	216	Glioma_grade2_2.raw
12667788			aLEEAMEQk	Unambiguous	N-Term(iTRAQ4plex); K9(iTRAQ4plex)	HCD	1.7027569	0	1	1	1.69821	1.7042	1.7014	1.7073245	0.228609455	126988.46	126545.34	215652.45	216053.97	0.24717269	0.131532681	2	High	2208	Unique	Used	49855	1	28.234	1	4.084	-0.4052	1	27	15/16	17.42543	2208	1336.7	MS2	668.854	210	Glioma_grade2_2.raw
12667788			lVWVPSDk	Unambiguous	N-Term(iTRAQ4plex); K8(iTRAQ4plex)	HCD	2.5661023	0	1	1	2.58689	2.4345	2.7059	2.5465357	4.361061748	126250.11	134150.47	326595.53	341618.94	4.290618807	3.179564515	2	High	4084	Unique	Used	67321	1	41.547	1	2.9832	0.7036	1	24	14-Dec	15.65193	4084	1231.73	MS2	616.369	232	Glioma_grade2_4.raw
12667788			kVEAQLQELQVk	Unambiguous	N-Term(iTRAQ4plex); K1(iTRAQ4plex); K12(iTRAQ4plex)	HCD	2.5817058	1	1	1	2.36337	2.923	2.3057	2.8517447	12.30845654	119145.07	96332.664	281583.94	274716.16	14.97213348	1.745911536	3	High	2383	Unique	Used	79803	1	29.5	1	5.998	4.051	1	3	23/44	10.89694	2383	1845.12	MS2	615.713	72	Glioma_grade2_6.raw
12667788			lkDVLLQVDDER	Unambiguous	N-Term(iTRAQ4plex); K2(iTRAQ4plex)	HCD	1.6520374	1	1	1	1.55384	1.627	1.6759	1.7548652	5.110807151	118329.9	113006.25	183865.23	198310.73	3.254475373	5.345449257	2	High	4445	Unique	Used	81589	1	47.319	1	4.6887	0.9434	1	44	19/22	42.0292	4445	1730.99	MS2	865.999	254	Glioma_grade2_6.raw
12667788			qIATLHAQVADMk	Unambiguous	N-Term(iTRAQ4plex); K13(iTRAQ4plex)	HCD	3.2742012	0	1	1	3.00645	3.1365	3.4062	3.5535386	7.603645002	110245.836	105675.15	331448.03	375520.72	2.993653489	8.816258983	2	High	2183	Unique	Used	72876	1	28.421	1	6.0033	1.6643	1	16	22/24	0	2183	1713.96	MS2	857.483	173	Glioma_grade2_5.raw
12667788			aLEQQVEEmk	Unambiguous	N-Term(iTRAQ4plex); M9(Oxidation); K10(iTRAQ4plex)	HCD	2.3470066	0	1	1	2.15564	2.4088	2.2917	2.5608418	7.316568146	99887.36	89389.81	215321.6	228913.17	7.843406356	4.32685236	2	High	1555	Unique	Used	49237	1	23.701	1	3.6489	-1.139	1	24	17/18	6.181643	1555	1508.79	MS2	754.896	188	Glioma_grade2_2.raw
12667788			lVWVPSDk	Unambiguous	N-Term(iTRAQ4plex); K8(iTRAQ4plex)	HCD	2.823535	0	1	1	2.64375	2.8947	2.7586	3.0203795	5.779387083	95278.35	87019.45	251892.47	262831.78	6.407015548	3.005593882	2	High	4195	Unique	Used	67424	1	42.4	1	3.0963	-0.3866	1	31	14-Dec	15.16382	4195	1231.73	MS2	616.368	215	Glioma_grade2_4.raw
12667788			tDLLLEPYNk	Unambiguous	N-Term(iTRAQ4plex); K10(iTRAQ4plex)	HCD	1.5154432	0	1	1	1.39924	1.5865	1.4528	1.6471988	7.560780329	95236.09	83994.84	133258.1	138356.2	8.869857568	2.654426576	2	High	4115	Unique	Used	51651	1	41.942	1	3.9847	-2.7869	1	37	15/18	31.79191	4115	1493.84	MS2	747.424	269	Glioma_grade2_2.raw
12667788			eQADFAIEALAk	Unambiguous	N-Term(iTRAQ4plex); K12(iTRAQ4plex)	HCD	2.4586842	0	1	1	2.23021	2.5018	2.4202	2.7149842	8.140259499	89095.26	79421.984	198701.25	215629.44	8.117909946	5.778011734	2	High	5375	Unique	Used	52836	1	51.169	1	6.0284	0.4727	1	31	20/22	23.97271	5375	1593.87	MS2	797.44	249	Glioma_grade2_2.raw
12667788			qIATLHAQVADmk	Unambiguous	N-Term(iTRAQ4plex); M12(Oxidation); K13(iTRAQ4plex)	HCD	4.1128364	0	1	1	4.36084	3.7903	4.4839	3.8972812	8.241414804	88847.17	102221.19	387448.2	398384.72	9.898928565	1.968175997	3	High	1261	Unique	Used	72036	1	21.34	1	4.3673	0.9466	1	3	23/48	30.46012	1261	1729.95	MS2	577.322	149	Glioma_grade2_5.raw
12667788			kQELEEIcHDLEAR	Unambiguous	N-Term(iTRAQ4plex); K1(iTRAQ4plex); C8(Methylthio)	HCD	2.5552027	1	1	1	2.57992	2.6328	2.4792	2.529975	2.580584975	80286.03	78674	207131.88	199043.25	1.434168507	2.816285243	3	High	5117	Unique	Used	92303	1	56.837	1	5.3755	######	1	4	29/52	12.53364	5117	2047.02	MS2	683.011	127	Glioma_grade2_7rep.raw
12667788			iAEFTTNLTEEEEk	Unambiguous	N-Term(iTRAQ4plex); K14(iTRAQ4plex)	HCD	4.2033396	0	1	1	3.94278	4.0702	4.3323	4.4723253	5.737802672	79873.77	77372.81	314924.44	346036.38	2.249264532	6.656813259	2	High	5150	Unique	Used	52624	1	49.531	1	5.8475	-1.9681	1	28	22/26	0	5150	1941.99	MS2	971.497	185	Glioma_grade2_2.raw
12667788			kVEAQLQELQVk	Unambiguous	N-Term(iTRAQ4plex); K1(iTRAQ4plex); K12(iTRAQ4plex)	HCD	3.6327302	1	1	1	3.38623	3.6259	3.6391	3.896679	5.733426128	79684.09	74416.63	269828.62	289977.72	4.834054877	5.090176451	2	High	2379	Unique	Used	79800	1	29.457	1	5.2759	5.0175	1	100	19/22	25.21482	2379	1845.13	MS2	923.067	70	Glioma_grade2_6.raw
12667788			nkHEAMITDLEER	Unambiguous	N-Term(iTRAQ4plex); K2(iTRAQ4plex)	HCD	3.1307652	1	1	1	2.59011	3.1885	3.0838	3.7963314	15.65340674	70016.586	56875.93	181350.81	215919.88	14.64522457	12.30597899	3	High	1476	Unique	Used	89364	1	22.391	1	4.4665	-0.7355	1	9	24/48	26.00558	1476	1873.97	MS2	625.327	166	Glioma_grade2_7rep.raw
12667788			lQQELDDLLVDLDHQR	Unambiguous	N-Term(iTRAQ4plex)	HCD	3.204664	0	1	1	2.9272	3.2867	3.1316	3.5162075	7.736755992	68786.64	61262.164	201352.27	215410.48	8.182478948	4.770414641	2	High	8254	Unique	Used	63471	1	75.739	1	6.4253	-3.2979	1	26	22/30	12.86435	8254	2094.09	MS2	1047.55	203	Glioma_grade2_3.raw
12667788			iAQLEEQLDNETk	Unambiguous	N-Term(iTRAQ4plex); K13(iTRAQ4plex)	HCD	2.056457	0	1	1	1.9386	2.3172	1.8383	2.1973286	10.72356296	66031.016	55242.457	128007.875	121385.83	12.58092646	3.755101136	2	High	5273	Unique	Used	52740	1	50.42	1	5.6199	-0.1957	1	35	21/24	8.443954	5273	1818.97	MS2	909.988	242	Glioma_grade2_2.raw
12667788			iAQLEEQLDNETk	Unambiguous	N-Term(iTRAQ4plex); K13(iTRAQ4plex)	HCD	3.9484377	0	1	1	3.81243	3.7527	4.1473	4.0823135	4.943066055	61959.29	62945.816	236215.5	256964.55	1.116974713	5.94987326	2	High	5189	Unique	Used	52661	1	49.803	1	6.2023	-1.2694	1	98	20/24	19.51181	5189	1818.97	MS2	909.987	234	Glioma_grade2_2.raw
12667788			vSHLLGINVTDFTR	Unambiguous	N-Term(iTRAQ4plex)	HCD	3.2888691	0	1	1	3.03727	3.3242	3.2566	3.5642307	6.58373073	61892.48	56550.973	187984.33	201560.7	6.377753647	4.928797726	2	High	5456	Unique	Used	75831	1	54.524	1	5.6433	0.1178	1	62	21/26	27.0072	5456	1715.96	MS2	858.482	175	Glioma_grade2_5.raw
12667788			kVEAQLQELQVk	Unambiguous	N-Term(iTRAQ4plex); K1(iTRAQ4plex); K12(iTRAQ4plex)	HCD	2.0455792	1	1	1	1.69422	2.1297	1.9786	2.4872582	15.93540692	61865.195	49214.47	104813.15	122409.09	16.10630251	10.9515763	3	High	2713	Unique	Used	90435	1	32.896	1	5.8984	-1.8041	1	18	21/44	51.59319	2713	1845.11	MS2	615.709	79	Glioma_grade2_7rep.raw
12667788			nkHEAMITDLEER	Unambiguous	N-Term(iTRAQ4plex); K2(iTRAQ4plex)	HCD	3.563122	1	1	1	3.25064	3.8032	3.3579	3.9287245	9.23892521	60687.89	51870.38	197274.42	203784.42	11.078566	2.295556006	2	High	1480	Unique	Used	89368	1	22.412	1	4.7022	0.1836	1	32	20/24	0	1480	1873.97	MS2	937.488	165	Glioma_grade2_7rep.raw
12667788			nFINNPLAQADWAAk	Unambiguous	N-Term(iTRAQ4plex); K15(iTRAQ4plex)	HCD	3.7579038	0	1	1	3.76174	3.5051	4.0292	3.7543268	5.68843856	59059.773	63383.633	222167.67	237962.88	4.994030837	4.854665747	2	High	6205	Unique	Used	53620	1	57.188	1	5.8053	-1.4316	1	17	23/28	7.955737	6205	1961.05	MS2	981.026	207	Glioma_grade2_2.raw
12667788			lQQELDDLLVDLDHQR	Unambiguous	N-Term(iTRAQ4plex)	HCD	4.5410643	0	1	1	4.85401	4.2941	4.8202	4.2642117	7.078545911	56404.65	63758.883	273788.97	271881.38	8.655251547	0.494390001	3	High	8247	Unique	Used	63464	1	75.702	1	6.4264	-1.4098	1	1	32/60	3.448761	8247	2094.09	MS2	698.702	167	Glioma_grade2_3.raw
12667788			hSQAVEELAEQLEQTk	Unambiguous	N-Term(iTRAQ4plex); K16(iTRAQ4plex)	HCD	3.2258382	0	1	1	3.17572	3.0391	3.421	3.2738044	4.989088285	53683.5	56097.375	170483.52	183651.83	3.109589683	5.258668076	2	High	4893	Unique	Used	75326	1	49.989	1	5.8562	-2.0184	1	17	25/30	7.09297	4893	2128.11	MS2	1064.56	176	Glioma_grade2_5.raw
12667788			kQELEEIcHDLEAR	Unambiguous	N-Term(iTRAQ4plex); K1(iTRAQ4plex); C8(Methylthio)	HCD	2.9153843	1	1	1	2.91529	2.8363	2.9967	2.915475	2.245919793	53272.457	54756.45	155304.73	159641.05	1.942705046	1.947155017	2	High	5120	Unique	Used	92306	1	56.852	1	5.3162	-2.1701	1	51	22/26	25.60533	5120	2047.01	MS2	1024.01	156	Glioma_grade2_7rep.raw
12667788			hEMPPHIYAITDTAYR	Unambiguous	N-Term(iTRAQ4plex)	HCD	1.8069133	0	1	1	1.75752	1.964	1.6663	1.8621109	7.108677416	48040.01	42989.27	84431.23	80050.78	7.846733499	3.766303561	2	High	2331	Unique	Used	79754	1	29.119	1	5.0865	3.9284	1	49	17/30	21.94796	2331	2059.03	MS2	1030.02	167	Glioma_grade2_6.raw
12667788			hEMPPHIYAITDTAYR	Unambiguous	N-Term(iTRAQ4plex)	HCD	2.384274	0	1	1	2.41478	2.4806	2.2905	2.352936	3.421478214	47166.277	45915.375	113896.21	108035.94	1.900527693	3.734327502	3	High	2326	Unique	Used	79749	1	29.089	1	6.1867	4.248	1	16	20/60	40.24929	2326	2059.03	MS2	687.014	151	Glioma_grade2_6.raw
12667788			nFINNPLAQADWAAk	Unambiguous	N-Term(iTRAQ4plex); K15(iTRAQ4plex)	HCD	3.9412076	0	1	1	3.37069	4.1533	3.7691	4.644197	13.6463374	45843.66	37205.19	154524.55	172788.23	14.71018736	7.891150463	2	High	6028	Unique	Used	61450	1	57.388	1	6.6313	-0.3734	1	39	22/28	9.878302	6028	1961.05	MS2	981.027	215	Glioma_grade2_3.raw
12667788			vISGVLQLGNIVFk	Unambiguous	N-Term(iTRAQ4plex); K14(iTRAQ4plex)	HCD	3.718839	0	1	1	3.33116	4.1626	3.3637	4.203285	12.82428261	45024.62	36031.17	149984.14	151449.3	15.69124052	0.687398566	2	High	8126	Unique	Used	63357	1	74.672	1	5.8345	1.7552	1	27	24/26	23.65504	8126	1775.11	MS2	888.057	52	Glioma_grade2_3.raw
12667788			qIATLHAQVADMk	Unambiguous	N-Term(iTRAQ4plex); K13(iTRAQ4plex)	HCD	1.6253239	0	1	1	1.53193	1.9711	1.3566	1.7454915	16.10793423	40356.91	31365.232	61823.96	54747.75	17.72974513	8.584649013	3	High	2165	Unique	Used	72859	1	28.294	1	4.1607	0.4813	1	12	20/48	33.21738	2165	1713.96	MS2	571.991	153	Glioma_grade2_5.raw
12667788			nMDPLNDNIATLLHQSSDk	Unambiguous	N-Term(iTRAQ4plex); K19(iTRAQ4plex)	HCD	3.4077172	0	1	1	3.13311	3.2711	3.5386	3.6944203	7.446869802	38385.23	36765.758	120265.164	135828.16	3.047570401	8.594288859	3	High	6230	Unique	Used	69297	1	58.482	1	6.5212	0.8467	1	6	34/72	12.36769	6230	2414.22	MS2	805.413	146	Glioma_grade2_4.raw
12667788			vSHLLGINVTDFTR	Unambiguous	N-Term(iTRAQ4plex)	HCD	3.206098	0	1	1	2.83805	2.6737	3.7712	3.5528436	16.669223	37566.508	39875.027	106615.44	141669.73	4.2157466	19.96665864	3	High	5460	Unique	Used	75834	1	54.567	1	4.8357	-0.9216	1	3	22/52	2.411485	5460	1715.95	MS2	572.656	174	Glioma_grade2_5.raw
12667788			lQQELDDLLVDLDHQR	Unambiguous	N-Term(iTRAQ4plex)	HCD	2.823121	0	1	1	2.63184	2.9615	2.7002	3.038367	6.964995779	37082.586	32954.78	97595.24	100128.72	8.334978257	1.812062522	2	High	8200	Unique	Used	71028	1	75.66	1	6.4896	0.3163	1	100	22/30	31.93719	8200	2094.1	MS2	1047.55	213	Glioma_grade2_4.raw
12667788			rQLEEAEEEAQR	Unambiguous	N-Term(iTRAQ4plex)	HCD	1.503451	1	1	1	1.5589	1.6287	1.3835	1.4455217	7.332871453	31531.143	30179.334	49153.797	43624.883	3.097928771	8.427652952	2	High	1278	Unique	Used	64716	1	20.306	1	4.2469	1.9328	1	22	20/22	3.774377	1278	1631.81	MS2	816.411	136	Glioma_grade2_4.raw
12667788			vSHLLGINVTDFTR	Unambiguous	N-Term(iTRAQ4plex)	HCD	2.2429352	0	1	1	2.0942	2.4076	2.0997	2.4139283	8.040809287	31060.2	27016.703	65046.344	65216.383	9.846200471	0.184604964	2	High	5973	Unique	Used	69061	1	56.414	1	5.2286	1.256	1	67	21/26	9.46785	5973	1715.96	MS2	858.483	201	Glioma_grade2_4.raw
12667788			lTEMETLQSQLMAEk	Unambiguous	N-Term(iTRAQ4plex); K15(iTRAQ4plex)	HCD	3.5196383	0	1	1	3.60031	3.3723	3.6769	3.444077	3.966850014	30669.953	32743.51	110421.29	112771.16	4.624337314	1.488949837	2	High	6760	Unique	Used	54138	1	61.318	1	6.4572	0.6585	1	32	22/28	4.124568	6760	2040.06	MS2	1020.53	207	Glioma_grade2_2.raw
12667788			kVEAQLQELQVk	Unambiguous	N-Term(iTRAQ4plex); K1(iTRAQ4plex); K12(iTRAQ4plex)	HCD	2.2566407	1	1	1	2.09151	2.282	2.2334	2.436805	6.29834473	28728.635	26330.756	60086.348	64162.914	6.159009283	4.639975186	2	High	2726	Unique	Used	90446	1	33.009	1	5.1582	-2.1937	1	100	20/22	23.37149	2726	1845.11	MS2	923.06	86	Glioma_grade2_7rep.raw
12667788			lQQELDDLLVDLDHQR	Unambiguous	N-Term(iTRAQ4plex)	HCD	4.022499	0	1	1	4.17633	3.7288	4.3514	3.8851485	6.954903858	26015.484	29137.791	108649.37	113204.65	8.006068371	2.903773732	3	High	8193	Unique	Used	71021	1	75.621	1	7.3681	0.5139	1	5	30/60	2.26305	8193	2094.1	MS2	698.704	170	Glioma_grade2_4.raw
12667788			hSQAVEELAEQLEQTk	Unambiguous	N-Term(iTRAQ4plex); K16(iTRAQ4plex)	HCD	3.9084108	0	1	1	4.39555	3.8173	4.0133	3.4853597	9.684043524	24505.6	28217.682	107715.484	98348.766	9.957037024	6.428354084	3	High	4853	Unique	Used	75289	1	49.691	1	5.626	-2.2255	1	1	28/60	2.545346	4853	2128.11	MS2	710.041	181	Glioma_grade2_5.raw
12667788			kMEDSVGcLETAEEVk	Unambiguous	N-Term(iTRAQ4plex); K1(iTRAQ4plex); C8(Methylthio); K16(iTRAQ4plex)	HCD	3.0531504	1	1	1	2.77824	2.8664	3.2341	3.336787	8.940251379	23270.088	22554.059	64649.918	75258.09	2.209791102	10.72291781	3	High	4460	Unique	Used	81602	1	47.453	1	7.0399	1.8492	1	7	27/60	10.35512	4460	2246.11	MS2	749.376	191	Glioma_grade2_6.raw
12667788			hEAMITDLEER	Unambiguous	N-Term(iTRAQ4plex)	HCD	1.5954013	0	1	1	1.38286	1.5028	1.6806	1.82637	12.21504363	22889.625	21063.002	31653.24	38468.836	5.877316548	13.74561172	3	High	2030	Unique	Used	65410	1	25.908	1	3.3527	-0.2884	1	29	18/40	57.10827	2030	1487.73	MS2	496.581	106	Glioma_grade2_4.raw
12667788			tFHIFYYLLSGAGEHLk	Unambiguous	N-Term(iTRAQ4plex); K17(iTRAQ4plex)	HCD	2.8076649	0	1	1	2.47717	2.6672	2.9381	3.1635067	10.72072738	20052.45	18623.936	49673.383	58916.95	5.223403947	12.03825189	3	High	5433	Unique	Used	87723	1	70.232	1	7.7791	0.6252	1	18	32/64	7.822915	5433	2284.24	MS2	762.084	192	Glioma_grade2_1rep.raw
12667788			iIGLDQVAGMSETALPGAFk	Unambiguous	N-Term(iTRAQ4plex); K20(iTRAQ4plex)	HCD	3.7530234	0	1	1	3.01271	4.5398	3.2309	4.868593	23.70789377	18241.672	12105.553	54956.832	58937.016	28.59497931	4.942172288	2	High	7494	Unique	Used	62796	1	69.153	1	7.07	-0.2308	1	42	26/38	16.84917	7494	2306.27	MS2	1153.64	152	Glioma_grade2_3.raw
12667788			kLEEEQIILEDQNck	Unambiguous	N-Term(iTRAQ4plex); K1(iTRAQ4plex); C14(Methylthio); K15(iTRAQ4plex)	HCD	3.5808914	1	1	1	3.85684	3.1253	4.1431	3.3572855	12.791873	17935.1	22133.22	69172.76	74307.55	14.81728767	5.061105359	3	High	3977	Unique	Used	81196	1	43.05	1	5.8047	0.7496	1	6	25/56	7.56383	3977	2310.21	MS2	770.74	116	Glioma_grade2_6.raw
12667788			vIQYLAYVASSHk	Unambiguous	N-Term(iTRAQ4plex); K13(iTRAQ4plex)	HCD	2.4822598	0	1	1	2.15705	2.4264	2.5319	2.8480818	11.4700406	17673.707	15711.743	38123.023	44748.332	8.310914179	11.30620085	3	High	5492	Unique	Used	82486	1	56.482	1	7.0994	0.426	1	40	29/48	27.00525	5492	1767.01	MS2	589.673	192	Glioma_grade2_6.raw
12667788			vISGVLQLGNIVFk	Unambiguous	N-Term(iTRAQ4plex); K14(iTRAQ4plex)	HCD	3.7635658	0	1	1	3.5154	3.8957	3.6444	4.038581	6.280171824	16667.994	15041.015	58594.582	60744.355	7.256284066	2.547565957	2	High	8058	Unique	Used	70908	1	74.346	1	5.8016	1.8239	1	63	24/26	20.66182	8058	1775.11	MS2	888.057	53	Glioma_grade2_4.raw
12667788			iIGLDQVAGMSETALPGAFk	Unambiguous	N-Term(iTRAQ4plex); K20(iTRAQ4plex)	HCD	1.6396708	0	1	1	1.4684	1.5498	1.7248	1.8204429	9.784613405	15679.275	14855.589	23023.377	27043.752	3.814878338	11.35609124	3	High	7491	Unique	Used	62793	1	69.137	1	5.7895	0.7426	1	5	32/76	5.213469	7491	2306.27	MS2	769.428	135	Glioma_grade2_3.raw
12667788			nFINNPLAQADWAAk	Unambiguous	N-Term(iTRAQ4plex); K15(iTRAQ4plex)	HCD	2.3023899	0	1	1	2.10218	2.3322	2.2756	2.5245078	7.538940541	15528.855	13997.498	32644.385	35336.793	7.334687891	5.601020784	3	High	6042	Unique	Used	61463	1	57.498	1	6.4638	0.7402	1	18	25/56	11.43979	6042	1961.05	MS2	654.355	189	Glioma_grade2_3.raw
12667788			vSHLLGINVTDFTR	Unambiguous	N-Term(iTRAQ4plex)	HCD	1.9363664	0	1	1	1.65362	1.8725	1.9927	2.2565413	12.92871838	15441.451	13636.151	25534.355	30770.537	8.780227971	13.15175172	3	High	5955	Unique	Used	69044	1	56.282	1	4.8814	-0.2813	1	37	22/52	30.70916	5955	1715.96	MS2	572.657	185	Glioma_grade2_4.raw
12667788			kLEEEQIILEDQNck	Unambiguous	N-Term(iTRAQ4plex); K1(iTRAQ4plex); C14(Methylthio); K15(iTRAQ4plex)	HCD	4.429329	1	1	1	4.23682	4.4308	4.4279	4.6306496	3.628597597	15124.837	14462.593	64081.266	66971.195	3.165379509	3.118580723	2	High	3964	Unique	Used	81186	1	42.912	1	6.759	-1.4639	1	85	22/28	0	3964	2310.2	MS2	1155.6	156	Glioma_grade2_6.raw
12667788			iIGLDQVAGMSETALPGAFk	Unambiguous	N-Term(iTRAQ4plex); K20(iTRAQ4plex)	HCD	3.3189971	0	1	1	2.91086	3.6138	3.0815	3.8257008	12.87258208	14326.318	11539.631	41701.836	44147.176	15.23613361	4.028273491	2	High	7816	Unique	Used	55106	1	69.646	1	6.5792	1.0395	1	51	27/38	5.266559	7816	2306.27	MS2	1153.64	154	Glioma_grade2_2.raw
12667788			nmDPLNDNIATLLHQSSDk	Unambiguous	N-Term(iTRAQ4plex); M2(Oxidation); K19(iTRAQ4plex)	HCD	2.5688303	0	1	1	2.83277	2.3099	2.8863	2.3536026	11.78978241	14078.577	17265.229	39881.426	40635.484	14.37798102	1.324441102	3	High	5697	Unique	Used	68806	1	54.233	1	5.9485	-0.1054	1	21	30/72	23.27679	5697	2430.22	MS2	810.744	137	Glioma_grade2_4.raw

12667788			sMEAEMIQLQEELAAAER	Unambiguous	N-Term(iTRAQ4plex)	HCD	2.9280832	0	1	1	2.80171	3.1481	2.7323	3.070076	6.877327516	13764.886	12250.33	38565.273	37609.445	8.233280232	1.774532229	2	High	5862	Unique	Used	88046	1	74.904	1	6.3645	-0.852	1	77	24/34	24.11811	5862	2193.06	MS2	1097.04	124	Glioma_grade 2_1rep.raw
12667788			nmDPLNDNIATLLHQSSDk	Unambiguous	N-Term(iTRAQ4plex); M2(Oxidation); K19(iTRAQ4plex)	HCD	2.5532122	0	1	1	2.20418	2.6873	2.4432	2.9787536	12.87224148	12109.845	9932.705	26692.227	29587.08	13.96817027	7.274326199	3	High	5232	Unique	Used	75628	1	52.751	1	6.1925	-0.1054	1	29	30/72	30.22641	5232	2430.22	MS2	810.744	136	Glioma_grade 2_5.raw
12667788			iIGLDQVAGmSETALPGAFk	Unambiguous	N-Term(iTRAQ4plex); M10(Oxidation); K20(iTRAQ4plex)	HCD	3.2728124	0	1	1	3.67392	2.9753	3.6402	2.9479797	12.14430784	9338.603	11531.43	34309.28	33994.42	14.8592275	0.651910925	2	High	6579	Unique	Used	61965	1	61.671	1	6.0901	-1.4037	1	73	24/38	5.24253	6579	2322.26	MS2	1161.63	135	Glioma_grade 2_3.raw
12667788			lQVELDNVTGLLSQSDSk	Unambiguous	N-Term(iTRAQ4plex); K18(iTRAQ4plex)	HCD	2.4288476	0	1	1	2.19228	2.6763	2.2261	2.717643	11.5140372	8762.855	7178.065	19210.646	19507.416	14.05961206	1.083980285	3	High	7245	Unique	Used	54594	1	64.902	1	5.8836	######	1	9	30/68	5.312748	7245	2234.21	MS2	745.409	176	Glioma_grade 2_2.raw
12667788			iIGLDQVAGMSETALPGAFk	Unambiguous	N-Term(iTRAQ4plex); K20(iTRAQ4plex)	HCD	1.6485418	0	1	1	1.34712	1.4481	1.835	1.972559	18.20063106	6771.711	6299.567	9122.28	12426.267	5.108241506	21.68379717	3	High	7788	Unique	Used	55079	1	69.445	1	5.6137	-3.1478	1	24	30/76	13.5087	7788	2306.26	MS2	769.425	140	Glioma_grade 2_2.raw
12667788			qELEEIcHDLEAR	Unambiguous	N-Term(iTRAQ4plex); C7(Methylthio)	HCD	3.330212	0	1	1	3.11007	3.7111	3.011	3.5928984	10.34645455	6449.0825	5404.5737	20057.104	19418.086	12.46162775	2.289306074	3	High	6904	Unique	Used	62269	1	64.184	1	5.1848	2.2417	1	73	25/48	31.55514	6904	1774.83	MS2	592.28	55	Glioma_grade 2_3.raw
12667788			vISGVLQLGNIVFk	Unambiguous	N-Term(iTRAQ4plex); K14(iTRAQ4plex)	HCD	5.019847	0	1	1	4.59797	5.7839	4.4124	5.55054	13.40575708	6371.1733	5064.811	29294.457	28112.438	16.15493021	2.911892902	3	High	8134	Unique	Used	63364	1	74.739	1	4.6734	0.3722	1	10	25/52	4.478929	8134	1775.1	MS2	592.373	56	Glioma_grade 2_3.raw
12667788			vISGVLQLGNIVFk	Unambiguous	N-Term(iTRAQ4plex); K14(iTRAQ4plex)	HCD	1.9820161	0	1	1	1.44033	2.1204	1.888	2.7794776	27.13699431	4957.2524	3367.2732	7140.083	9359.261	27.01139088	19.02131154	3	High	8064	Unique	Used	63308	1	74.028	1	5.0638	-1.3813	1	40	24/52	11.23213	8064	1775.1	MS2	592.372	57	Glioma_grade 2_3.raw
12667788			vISGVLQLGNIVFk	Unambiguous	N-Term(iTRAQ4plex); K14(iTRAQ4plex)	HCD	1.6723005	0	1	1	1.44323	2.2108	1.3208	2.0231948	24.81243569	4842.5645	3161.288	6988.9453	6395.9014	29.7067447	6.265971851	3	High	5863	Unique	Used	88047	1	74.909	1	4.9267	-1.5877	1	100	25/52	18.7745	5863	1775.1	MS2	592.372	57	Glioma_grade 2_1rep.raw
12667788			qELEEIcHDLEAR	Unambiguous	N-Term(iTRAQ4plex); C7(Methylthio)	HCD	1.8226031	0	1	1	1.73583	1.7245	1.9213	1.9088073	5.869140339	4382.6445	4411.407	7607.54	8420.525	0.462543545	7.173257739	3	High	7023	Unique	Used	70016	1	64.995	1	4.4383	0.7974	1	97	26/48	67.77914	7023	1774.82	MS2	592.279	39	Glioma_grade 2_4.raw
12667788			tFHIFYYLLSGAGEHLk	Unambiguous	N-Term(iTRAQ4plex); K17(iTRAQ4plex)	HCD	2.8282034	0	1	1	2.34675	2.4401	3.2015	3.3288043	17.93419665	3569.4507	3432.9365	8376.602	11427.574	2.757063093	21.78695029	4	High	5429	Unique	Used	87722	1	70.16	1	6.8076	0.3521	1	32	29/96	14.77086	5429	2284.24	MS2	571.815	213	Glioma_grade 2_1rep.raw
12667788			tFHIFYYLLSGAGEHLk	Unambiguous	N-Term(iTRAQ4plex); K17(iTRAQ4plex)	HCD	2.0465057	0	1	1	1.75593	1.8504	2.2326	2.3527238	14.13155334	3554.7095	3373.1619	6241.8096	7936.118	3.706002368	16.90031143	3	High	5302	Unique	Used	96805	1	72.876	1	8.3718	2.6292	1	100	32/64	18.90207	5302	2284.24	MS2	762.086	191	Glioma_grade 2_8rep.raw
12667788			dFSALESQLQDTQELLQEENR	Unambiguous	N-Term(iTRAQ4plex)	HCD	2.447145	0	1	1	2.59533	2.4792	2.4136	2.3055933	4.954015479	3133.8345	3280.6746	8133.3335	7563.9014	3.237399116	5.130193972	2	High	5583	Unique	Used	87830	1	71.957	1	5.4331	1.3098	1	100	24/40	28.1238	5583	2637.28	MS2	1319.14	162	Glioma_grade 2_1rep.raw
12667788			tFHIFYYLLSGAGEHLk	Unambiguous	N-Term(iTRAQ4plex); K17(iTRAQ4plex)	HCD	4.2296934	0	1	1	3.69698	3.9038	4.5383	4.7922044	12.21235395	1663.8197	1575.6798	6151.11	7550.9795	3.847774694	14.44826669	4	High	5310	Unique	Used	96810	1	72.975	1	7.0911	0.0315	1	59	31/96	7.331355	5310	2284.24	MS2	571.815	215	Glioma_grade 2_8rep.raw
13236587	NP_077310.1	TMEM43	transmembrane protein 43 [Homo sapiens]	8	10	2.286777496	26.32181953	65.744658	28.25	1.04E-04	400	44.847	8.1333	Integral membrane protein	Biological_process unknown	Molecular function unknown	Integral to membrane	-																								
13236587			fFLSSGLIDk	Unambiguous	N-Term(iTRAQ4plex); K10(iTRAQ4plex)	HCD	3.6179795	0	1	1	3.34595	3.7008	3.5431	3.9188528	6.68998401	197868.3	178896.33	662058.4	701068.4	7.121267535	4.047200236	2	High	6091	Unique	Used	61507	1	57.913	1	3.7884	1.2516	1	15	18/18	7.135516	6091	1414.82	MS2	707.914	174	Glioma_grade 2_3.raw
13236587			lVHIIGALR	Unambiguous	N-Term(iTRAQ4plex)	HCD	2.2469862	0	1	1	2.06319	2.3256	2.1772	2.4541605	7.572751045	91755.42	81402.2	189308.7	199774.08	8.455685715	3.803890352	2	High	3112	Unique	Used	80439	1	35.702	1	3.3066	1.1712	1	32	16-Dec	35.18773	3112	1135.74	MS2	568.376	124	Glioma_grade 2_6.raw
13236587			lEDPHVDIIR	Unambiguous	N-Term(iTRAQ4plex)	HCD	3.0240054	0	1	1	2.9401	3.0823	2.9684	3.1119676	2.78140578	85857.14	81897.29	252428.6	254861.7	3.338256745	0.678294661	2	High	2938	Unique	Used	66258	1	32.642	1	3.3787	-0.6554	1	21	17/18	8.138971	2938	1350.75	MS2	675.878	301	Glioma_grade 2_4.raw
13236587			lEDPHVDIIR	Unambiguous	N-Term(iTRAQ4plex)	HCD	2.7564635	0	1	1	2.55892	2.7254	2.7856	2.9668615	6.103135316	77009.85	72305.04	197062.02	214519.05	4.456090153	5.998324603	2	High	2639	Unique	Used	58303	1	31.126	1	3.3557	-0.9266	1	36	17/18	12.29553	2639	1350.75	MS2	675.878	363	Glioma_grade 2_3.raw
13236587			gDQLVPFSTk	Unambiguous	N-Term(iTRAQ4plex); K10(iTRAQ4plex)	HCD	1.9177958	0	1	1	1.78342	1.8954	1.9389	2.0606031	5.968959665	68133.016	64108.64	121510.02	132102.47	4.303732494	5.906643813	2	High	2951	Unique	Used	50556	1	33.481	1	3.644	-0.4844	1	49	18-Dec	8.563479	2951	1379.78	MS2	690.392	276	Glioma_grade 2_2.raw
13236587			lLSDPNYGVHLPAVk	Unambiguous	N-Term(iTRAQ4plex); K15(iTRAQ4plex)	HCD	1.6459575	0	1	1	1.58457	1.6415	1.6503	1.7095501	3.106969347	39884.332	38501.99	63199.477	65821.086	2.493964195	2.87358458	3	High	4615	Unique	Used	81735	1	48.816	1	6.2583	0.1037	1	27	25/56	22.67095	4615	1911.09	MS2	637.703	118	Glioma_grade 2_6.raw
13236587			sGDTLLLLHHGDFSAEEVFHR	Unambiguous	N-Term(iTRAQ4plex)	HCD	2.326569	0	1	1	2.35801	2.2899	2.3643	2.296033	1.698041867	15828.909	16299.44	37324.773	37424.05	2.071165629	0.187827546	3	High	4674	Unique	Used	91972	1	52.234	1	6.4955	-0.8778	1	26	33/80	15.84466	4674	2524.27	MS2	842.094	116	Glioma_grade 2_7rep.raw
13236587			sGDTLLLLHHGDFSAEEVFHR	Unambiguous	N-Term(iTRAQ4plex)	HCD	2.026875	0	1	1	1.88466	1.5883	2.5472	2.146729	19.93035993	5836.89	6925.796	11000.563	14867.807	12.06599953	21.14207008	4	High	4662	Unique	Used	91962	1	52.125	1	8.9558	0.8578	1	13	36/120	21.75454	4662	2524.27	MS2	631.824	145	Glioma_grade 2_7rep.raw
13236587			rGDFFYHSENPk	Unambiguous	N-Term(iTRAQ4plex); K12(iTRAQ4plex)	HCD	1.9932834	1	1	1	2.02545	1.8167	2.1902	1.9644322	7.731652932	4021.7717	4483.918	8145.8965	8808.354	7.683957308	5.525790603	4	High	1667	Unique	Used	94593	1	25.763	1	5.1892	-1.8368	1	100	24/66	38.18784	1667	1784.89	MS2	446.979	178	Glioma_grade 2_8rep.raw
13236587			vSFSYAGLSGDDPDLGPAHVVTVIAR	Unambiguous	N-Term(iTRAQ4plex)	HCD	3.2856133	0	1	1	3.05212	2.8968	3.6953	3.5072196	11.41617121	791.18054	833.611	2414.78	2923.6567	3.693134197	13.48073174	2	High	6699	Unique	Used	62078	1	62.579	1	4.9468	0.319	1	100	23/50	20.71131	6699	2787.44	MS2	1394.23	114	Glioma_grade 2_3.raw
13904870	NP_001000.2	RPS5	40S ribosomal protein S5 [Homo sapiens]	3	8	3.023864746	12.26702983	58.555118	14.22	8.40E-05	204	22.862	9.7153	Ribosomal subunit	Protein metabolism	Structural constituent of ribosome	Ribosome	Nucleolus																								
13904870			tIAEcLADELINAAk	Unambiguous	N-Term(iTRAQ4plex); C5(Methylthio); K15(iTRAQ4plex)	HCD	2.7302687	0	1	1	2.41861	3.0047	2.5094	3.1174474	12.66060801	298350.6	240155.36	721594.3	748671.7	15.28311733	2.60451009	2	High	8686	Unique	Used	55899	1	76.665	1	6.7877	-0.3082	1	3	25/28	7.429575	8686	1909	MS2	955.004	205	Glioma_grade 2_2.raw
13904870			vNQAIWLLcTGAR	Unambiguous	N-Term(iTRAQ4plex); C9(Methylthio)	HCD	3.1632407	0	1	1	2.88101	3.1584	3.1676	3.4726472	7.624602859	119308.945	108829.055	343730.47	377924.9	6.496419961	6.701013901	2	High	5881	Unique	Used	88059	1	75.134	1	5.0415	-0.9369	1	16	18/24	29.96634	5881	1634.86	MS2	817.934	192	Glioma_grade 2_1rep.raw
13904870			tIAEcLADELINAAk	Unambiguous	N-Term(iTRAQ4plex); C5(Methylthio); K15(iTRAQ4plex)	HCD	2.7137144	0	1	1	2.58541	2.7447	2.6845	2.8499222	4.077997208	94565.984	89076.83	244492.11	253862.03	4.227138467	2.658966161	2	High	6049	Unique	Used	88200	1	76.625	1	7.0484	-3.8251	1	24	23/28	31.38431	6049	1908.99	MS2	955.001	224	Glioma_grade 2_1rep.raw
13904870			rVNQAIWLLcTGAR	Unambiguous	N-Term(iTRAQ4plex); C10(Methylthio)	HCD	2.2545047	1	1	1	2.15907	2.2764	2.2338	2.3551285	3.630884673	52675.566	49961.52	113729.99	117665.81	3.739623572	2.405438246	2	High	5884	Unique	Used	82827	1	59.827	1	5.2619	0.4143	1	19	18/26	4.910862	5884	1790.96	MS2	895.986	168	Glioma_grade 2_6.raw
13904870			vNQAIWLLcTGAR	Unambiguous	N-Term(iTRAQ4plex); C9(Methylthio)	HCD	3.29969	0	1	1	3.33725	3.0192	3.6097	3.2657113	7.343785154	27155.502	30015.777	90624.63	98022.87	7.075300339	5.546159555	2	High	8411	Unique	Used	55643	1	74.599	1	4.6264	0.5565	1	62	18/24	12.32407	8411	1634.86	MS2	817.936	221	Glioma_grade 2_2.raw
13904870			tIAEcLADELINAAk	Unambiguous	N-Term(iTRAQ4plex); C5(Methylthio); K15(iTRAQ4plex)	HCD	2.8844888	0	1	1	2.78276	3.0316	2.7495	2.9953132	4.98958414	25854.506	23732.678	71946.88	71086.805	6.051398149	0.850379916	3	High	6042	Unique	Used	88193	1	76.584	1	5.1175	-3.5444	1	10	26/56	7.252918	6042	1908.99	MS2	637.003	207	Glioma_grade 2_1rep.raw
13904870			tIAEcLADELINAAk	Unambiguous	N-Term(iTRAQ4plex); C5(Methylthio); K15(iTRAQ4plex)	HCD	3.2592337	0	1	1	3.78918	2.975	3.6213	2.8431597	14.14696067	18455.69	23506.832	69931.99	66833.67	17.02327025	3.203791189	2	High	8390	Unique	Used	63600	1	76.686	1	6.3057	-4.3367	1	27	23/28	12.25738	8390	1908.99	MS2	955	216	Glioma_grade 2_3.raw
13904870			tIAEcLADELINAAk	Unambiguous	N-Term(iTRAQ4plex); C5(Methylthio); K15(iTRAQ4plex)	HCD	3.1825051	0	1	1	3.34616	3.646	2.7571	3.004189	12.20427539	8347.87	7661.3193	27933.275	23016.05	6.064824976	13.64886833	3	High	8384	Unique	Used	63594	1	76.651	1	5.2244	-3.1607	1	10	27/56	8.296182	8384	1909	MS2	637.003	196	Glioma_grade 2_3.raw
14141161	NP_004492.2	HNRNPU	heterogeneous nuclear ribonucleoprotein U isoform b [Homo sapiens]	7	8	2.05106616	23.36619224	41.109358	10.67	2.65E-05	806	88.924	5.7827	Ribonucleoprotein	Regulation of nucleobase, nucleoside, nucleotide and nucleic acid metabolism	RNA binding	Cytoplasm, Nucleus	Mitochondrion, Cytoplasm																								
14141161			vSELkEELk	Unambiguous	N-Term(iTRAQ4plex); K5(iTRAQ4plex); K9(iTRAQ4plex)	HCD	1.6904203	1	1	2	1.54849	1.7266	1.658	1.8486761	7.41773865	231969.1	208041.53	359201.5	384601.4	7.690426481	4.82935507	2	High	2015	Unique	Used	79460	1	26.748	1	4.3857	3.4605	1	24	16/16	0	2015	1506.92	MS2	753.961	139	Glioma_grade 2_6.raw
14141161			lLEQYkEESk	Unambiguous	N-Term(iTRAQ4plex); K6(iTRAQ4plex); K10(iTRAQ4plex)	HCD	2.2343159	1	1	2	2.21033	2.2476	2.2213	2.2587056	1.006368328	180317.3	177327.58	398560.88	400530.78	1.182206934	0.348628253	2	High	1957	Unique	Used	89792	1	26.242	1	4.6489	-1.7524	1	26	17/18	5.011631	1957	1698.96	MS2	849.984	191	Glioma_grade 2_7rep.raw
14141161			yNILGTNTIMDk	Unambiguous	N-Term(iTRAQ4plex); K12(iTRAQ4plex)	HCD	1.5263369	0	1	2	1.50449	1.4725	1.5814	1.5477202	3.133603458	87992.49	89905.664	132383.72	139148.81	1.520890778	3.52343862	2	High	5332	Unique	Used	52795	1	50.863	1	4.5138	-1.0059	1	47	19/22	26.86464	5332	1670.9	MS2	835.954	251	Glioma_grade 2_2.raw
14141161			mMVAGFk	Unambiguous	N-Term(iTRAQ4plex); K7(iTRAQ4plex)	HCD	2.0575268	0	1	2	2.13147	1.9069	2.2259	1.9913732	6.891930361	77515.945	86643.67	165222.92	172539.89	7.863415426	3.063616805	2	High	2928	Unique	Used	66249	1	32.565	1	3.044	-1.4211	1	72	12-Nov	60.29924	2928	1071.59	MS2	536.3	222	Glioma_grade 2_4.raw
14141161			kDcEVVMMIGLPGAGk	Unambiguous	N-Term(iTRAQ4plex); K1(iTRAQ4plex); C3(Methylthio); K16(iTRAQ4plex)	HCD	2.0446055	1	1	2	1.96664	2.1928	1.9117	2.1315393	6.489797138	38892.16	34880.336	76486.84	74348.805	7.690638406	2.004591218	3	High	5172	Unique	Used	92348	1	57.347	1	5.3456	2.0619	1	15	21/60	36.15722	5172	2126.13	MS2	709.38	201	Glioma_grade 2_7rep.raw
14141161			gYFEYIEENk	Unambiguous	N-Term(iTRAQ4plex); K10(iTRAQ4plex)	HCD	2.9208605	0	1	2	2.87407	3.1333	2.726	2.9718742	5.844135875	14814.601	13589.115	42578.152	40385.14	6.101662619	3.738264524	3	High	5279	Unique	Used	60758	1	51.487	1	3.8195	0.0614	1	60	18/36	15.4157	5279	1579.79	MS2	527.268	145	Glioma_grade 2_3.raw
14141161			kDcEVVMMIGLPGAGk	Unambiguous	N-Term(iTRAQ4plex); K1(iTRAQ4plex); C3(Methylthio); K16(iTRAQ4plex)	HCD	1.5369786	1	1	2	1.48127	1.5149	1.5586	1.5939485	3.210487172	4978.999	4868.6294	7375.249	7760.3447	1.585012948	3.598190943	4	High	5177	Unique	Used	92353	1	57.377	1	3.3376	-1.7051	1	63	19/90	50.19364	5177	2126.12	MS2	532.285	194	Glioma_grade 2_7rep.raw
14141161			ekPYFPIPEEYTFIQNVPLEDR	Unambiguous	N-Term(iTRAQ4plex); K2(iTRAQ4plex)	HCD	2.4489965	0	1	2	2.59268	2.1312	2.8357	2.3308938	12.41941943	4115.447	5006.677	10670.017	11670.032	13.81684302	6.330490929	3	High	6749	Unique	Used	76972	1	65.4	1	7.4837	-4.5787	1	13	29/84	19.29271	6749	3012.55	MS2	1004.85	117	Glioma_grade 2_5.raw
14141193	NP_001004.2	RPS9	40S ribosomal protein S9 [Homo sapiens]	3	4	2.216852188	13.48115991	21.977853	19.59	8.40E-05	194	22.578	10.653	Ribosomal subunit	Protein metabolism	Structural constituent of ribosome	Ribosome	Nucleolus																								
14141193			lDYILGLk	Unambiguous	N-Term(iTRAQ4plex); K8(iTRAQ4plex)	HCD	2.5770886	0	1	1	2.41662	2.5306	2.6215	2.7451253	5.397808156	437718.2	418004.9	1057798.2	1147475.9	3.257924931	5.750914119	2	High	6209	Unique	Used	61619	1	58.796	1	3.69	-1.7111	1	9	14/14	12.91412	6209	1222.76	MS2	611.885	158	Glioma_grade 2_3.raw
14141193			eLLTLDEk	Unambiguous	N-Term(iTRAQ4plex); K8(iTRAQ4plex)	HCD	2.06131	0	1	1	1.9677	2.1051	2.0204	2.1614518	4.183642382	396480.84	370602.8	780157.2	801040.1	4.770936731	1.86775429	2	High	3085	Unique	Used	50678	1	34.519	1	3.4286	-1.5047	1	9	14/14	70.35798	3085	1248.73	MS2	624.867	372	Glioma_grade 2_2.raw
14141193			lDYILGLk	Unambiguous	N-Term(iTRAQ4plex); K8(iTRAQ4plex)	HCD	2.372394	0	1	1	2.27687	2.3506	2.3935	2.4710124	3.42097594	300960.22	291520.8	685246.9	720351.56	2.253128005	3.531982119	2	High	6376	Unique	Used	53781	1	58.43	1	3.5705	-2.8093	1	13	14/14	30.07263	6376	1222.76	MS2	611.884	154	Glioma_grade 2_2.raw
14141193			kQVVNIPSFIVR	Unambiguous	N-Term(iTRAQ4plex); K1(iTRAQ4plex)	HCD	1.9105988	1	1	1	1.9992	1.8038	2.0289	1.8306578	5.99296358	12378.278	13718.934	24746.627	25114.674	7.265043859	1.043889848	3	High	4146	Unique	Used	81339	1	44.567	1	4.2289	1.4039	1	35	21/44	25.40351	4146	1688.05	MS2	563.354	53	Glioma_grade 2_6.raw
14165435	NP_112552.1	HNRNPK	heterogeneous nuclear ribonucleoprotein K isoform b [Homo sapiens]	6	13	2.063493013	12.0578147	100.37313	27.65	1.49E-04	463	50.944	5.5415	Ribonucleoprotein	Regulation of nucleobase, nucleoside, nucleotide and nucleic acid metabolism	Ribonucleoprotein	Nucleus	Mitochondrion, Cytoplasm, Nucleolus																								
14165435			vVLIGGkPDR	Unambiguous	N-Term(iTRAQ4plex); K7(iTRAQ4plex)	HCD	1.7378869	0	1	2	1.57248	1.7717	1.7079	1.9242537	8.373445077	194416.19	172556.06	305714.6	332041.66	8.42431337	5.837980377	2	High	1274	Unique	Used	78768	1	21.33	1	3.521	0.3127	1	43	13/18	24.57121	1274	1341.85	MS2	671.427	81	Glioma_grade 2_6.raw
14165435			iILDLISESPIk	Unambiguous	N-Term(iTRAQ4plex); K12(iTRAQ4plex)	HCD	2.0493712	0	1	2	1.82901	2.0008	2.0938	2.2904258	9.364348223	50110.715	45808.93	91653.016	104921.95	6.342436619	9.546030607	2	High	7865	Unique	Used	63132	1	72.263	1	4.8561	2.3234	1	47	19/22	16.68263	7865	1629.01	MS2	815.009	72	Glioma_grade 2_3.raw
14165435			lLIHQSLAGGIIGVk	Unambiguous	N-Term(iTRAQ4plex); K15(iTRAQ4plex)	HCD	2.1601186	0	1	2	2.05318	2.1148	2.2041	2.2702703	4.436378111	48156.58	46753.777	98873.89	106143.72	2.090249253	5.01473614	2	High	4655	Unique	Used	81770	1	49.153	1	6.4753	2.2111	1	100	21/28	22.93601	4655	1807.14	MS2	904.076	24	Glioma_grade 2_6.raw
14165435			rPAEDMEEEQAFk	Unambiguous	N-Term(iTRAQ4plex); K13(iTRAQ4plex)	HCD	2.2442589	0	1	2	2.3453	2.1276	2.3729	2.1526005	5.660231311	44625.527	49191.516	104660.055	105889.68	6.882847042	0.825910492	2	High	1440	Unique	Used	78924	1	22.522	1	4.4703	-7.7542	1	39	20/24	10.33531	1440	1867.9	MS2	934.451	68	Glioma_grade 2_6.raw
14165435			iILDLISESPIk	Unambiguous	N-Term(iTRAQ4plex); K12(iTRAQ4plex)	HCD	1.9336623	0	1	2	1.95879	1.8498	2.0225	1.9099352	3.785699757	37681.09	39901.15	73809.24	76208.61	4.046852683	2.261878567	2	High	7782	Unique	Used	63058	1	71.541	1	5.0527	1.724	1	100	19/22	42.46824	7782	1629.01	MS2	815.009	70	Glioma_grade 2_3.raw
14165435			iILDLISESPIk	Unambiguous	N-Term(iTRAQ4plex); K12(iTRAQ4plex)	HCD	1.8566139	0	1	2	1.68056	1.782	1.927	2.043292	8.585411586	35832.83	33793.24	60219.27	69049.45	4.142723896	9.660310951	2	High	8145	Unique	Used	55405	1	72.334	1	4.9682	-0.674	1	100	20/22	8.735788	8145	1629.01	MS2	815.007	68	Glioma_grade 2_2.raw
14165435			iILDLISESPIk	Unambiguous	N-Term(iTRAQ4plex); K12(iTRAQ4plex)	HCD	2.1603522	0	1	2	2.007	2.4136	1.9497	2.3447762	10.75355648	28692.719	23858.652	57586.266	55943.2	13.00899098	2.046734036	2	High	8241	Unique	Used	55490	1	73.158	1	4.873	1.4242	1	90	19/22	6.031224	8241	1629.01	MS2	815.009	72	Glioma_grade 2_2.raw

14165435			gSYGDLGGPIITTQVTIPk	Unambiguous	N-Term(iTRAQ4plex); K19(iTRAQ4plex)	HCD	2.063493	0	1	2	1.90745	1.9601	2.1641	2.2238436	7.447236939	24788.582	24122.67	47282.977	53645.05	1.925409273	8.914600026	2	High	6016	Unique	Used	53441	1	55.823	1	6.26	-3.3727	1	42	24/36	0	6016	2205.23	MS2	1103.12	91	Glioma_grade 2_2.raw
14165435			iLSISADIETIGEILk	Unambiguous	N-Term(iTRAQ4plex); K16(iTRAQ4plex)	HCD	1.9438322	0	1	2	2.21347	1.8053	2.1137	1.7239186	12.03050528	12725.91	15603.6	28168.479	26899.336	14.36547341	3.25932533	3	High	8441	Unique	Used	63648	1	77.065	1	5.2276	0.4536	1	11	25/60	7.447503	8441	2003.19	MS2	668.401	86	Glioma_grade 2_3.raw
14165435			iILDLISESPIk	Unambiguous	N-Term(iTRAQ4plex); K12(iTRAQ4plex)	HCD	2.2622838	0	1	2	2.05553	2.5679	2.0176	2.5205681	12.84179333	7712.682	6173.799	15853.681	15561.481	15.67214336	1.315394149	3	High	8166	Unique	Used	55424	1	72.51	1	4.5272	-1.3941	1	32	19/44	6.244612	8166	1629.01	MS2	543.673	74	Glioma_grade 2_2.raw
14165435			iILDLISESPIk	Unambiguous	N-Term(iTRAQ4plex); K12(iTRAQ4plex)	HCD	2.383478	0	1	2	2.40512	2.6346	2.1542	2.3597748	8.253562487	5286.426	4825.8896	12714.468	11388.014	6.440629907	7.782971217	3	High	7787	Unique	Used	63062	1	71.593	1	4.1983	-1.2817	1	52	19/44	0	7787	1629.01	MS2	543.673	75	Glioma_grade 2_3.raw
14165435			iLSISADIETIGEILk	Unambiguous	N-Term(iTRAQ4plex); K16(iTRAQ4plex)	HCD	2.5596406	0	1	2	2.51433	2.1264	3.072	2.5979578	15.06618211	3348.5232	3959.5007	8419.299	10286.615	11.8233421	14.11737279	3	High	8752	Unique	Used	55961	1	77.138	1	4.891	-1.5574	1	33	20/60	23.04459	8752	2003.18	MS2	668.4	82	Glioma_grade 2_2.raw
14165435			iLSISADIETIGEILk	Unambiguous	N-Term(iTRAQ4plex); K16(iTRAQ4plex)	HCD	1.6904114	0	1	2	2.00038	1.7147	1.662	1.4247061	13.9027837	640.1141	746.7413	1280.4694	1063.8868	10.87306091	13.0651669	3	High	8565	Unique	Used	63747	1	78.29	1	3.2457	0.6364	1	100	19/60	33.43179	8565	2003.19	MS2	668.401	69	Glioma_grade 2_3.raw
14211889	NP_115963.1	DPY30	protein dpy-30 homolog [Homo sapiens]	2	3	2.377270222	20.97577835	23.690784	36.36	3.54E-04	99	11.243	4.8813	Unclassified	Biological_process unknown	Molecular function unknown	-	-																								
14211889			eRPPNPIEFLASYLLk	Unambiguous	N-Term(iTRAQ4plex); K16(iTRAQ4plex)	HCD	2.4449155	0	1	1	2.62385	2.4579	2.4311	2.2773001	5.799477306	32806.363	35021.902	86079	79755.38	4.619379991	5.39269913	3	High	6870	Unique	Used	93502	1	76.767	1	6.0253	-0.8395	1	5	23/60	8.202841	6870	2175.24	MS2	725.751	108	Glioma_grade 2_7rep.raw
14211889			aYLDQTVVPILLQGLAVLAk	Unambiguous	N-Term(iTRAQ4plex); K20(iTRAQ4plex)	HCD	1.6317017	0	1	1	1.36008	1.802	1.5032	1.9915668	17.15629069	3149.1726	2376.9324	4283.1353	4733.8193	19.76279069	7.068499881	3	High	8530	Unique	Used	63728	1	77.81	1	6.5475	1.3117	1	40	28/76	11.08548	8530	2413.47	MS2	805.162	13	Glioma_grade 2_3.raw
14211889			eRPPNPIEFLASYLLk	Unambiguous	N-Term(iTRAQ4plex); K16(iTRAQ4plex)	HCD	2.3772702	0	1	1	2.6286	2.2197	2.5638	2.1650348	9.844677227	3135.5173	3713.0845	8242.0205	8038.9575	11.92657116	1.763864853	4	High	6872	Unique	Used	93504	1	76.777	1	4.3186	-2.9501	1	20	22/90	7.26925	6872	2175.24	MS2	544.564	125	Glioma_grade 2_7rep.raw
14277700	NP_001007.2	RPS12	40S ribosomal protein S12 [Homo sapiens]	4	10	2.52189064	14.115605	42.537287	31.82	1.07E-04	132	14.505	7.2104	Ribosomal subunit	Protein metabolism	Structural constituent of ribosome	Ribosome	Nucleolus																								
14277700			tALIHDGLAR	Unambiguous	N-Term(iTRAQ4plex)	HCD	2.9549758	0	1	1	2.71242	3.0545	2.8666	3.228118	7.558270808	1178094	1046169.3	3195486.5	3377158	8.387932308	3.908963876	2	High	1327	Unique	Used	64762	1	20.636	1	3.6462	0.266	1	3	16/18	17.46166	1327	1210.7	MS2	605.855	156	Glioma_grade 2_4.raw
14277700			lGEWVGLck	Unambiguous	N-Term(iTRAQ4plex); C8(Methylthio); K9(iTRAQ4plex)	HCD	2.6524377	0	1	1	2.67962	2.5835	2.7239	2.6262307	2.311094737	218737.9	226874	586134.25	595823.5	2.582108549	1.159319676	2	High	6844	Unique	Used	69854	1	63.515	1	3.6425	0.8126	1	18	15/16	12.18259	6844	1338.72	MS2	669.862	220	Glioma_grade 2_4.raw
14277700			lGEWVGLck	Unambiguous	N-Term(iTRAQ4plex); C8(Methylthio); K9(iTRAQ4plex)	HCD	2.4519634	0	1	1	2.23417	2.6296	2.3011	2.7082992	9.541574802	182827.23	155335.6	408467.62	420695.25	11.49713468	2.085534797	2	High	6746	Unique	Used	62121	1	62.967	1	3.9131	0.5391	1	21	15/16	12.13793	6746	1338.72	MS2	669.862	237	Glioma_grade 2_3.raw
14277700			lVEALcAEHQINLIk	Unambiguous	N-Term(iTRAQ4plex); C6(Methylthio); K15(iTRAQ4plex)	HCD	2.5655234	0	1	1	2.42554	2.5825	2.5496	2.7145715	4.625533907	179849.72	168916.94	436231.94	458537.1	4.433131811	3.525408052	2	High	5911	Unique	Used	82852	1	60.031	1	6.7968	0.4242	1	7	21/28	5.020681	5911	2028.12	MS2	1014.57	185	Glioma_grade 2_6.raw
14277700			lGEWVGLck	Unambiguous	N-Term(iTRAQ4plex); C8(Methylthio); K9(iTRAQ4plex)	HCD	2.5731716	0	1	1	2.42238	2.5269	2.6175	2.7304657	5.093486014	142124.8	136245.66	344280.88	372014.12	2.986796632	5.475498801	2	High	6370	Unique	Used	76643	1	62.097	1	3.9443	-4.8408	1	25	16/16	21.04745	6370	1338.71	MS2	669.858	217	Glioma_grade 2_5.raw
14277700			lVEALcAEHQINLIk	Unambiguous	N-Term(iTRAQ4plex); C6(Methylthio); K15(iTRAQ4plex)	HCD	2.478258	0	1	1	2.32093	2.5299	2.4309	2.649754	5.647390335	114321.93	104880.61	265332.62	277907.8	6.091189815	3.273686834	3	High	5883	Unique	Used	82826	1	59.821	1	7.1889	0.5379	1	10	26/56	11.02711	5883	2028.12	MS2	676.713	162	Glioma_grade 2_6.raw
14277700			dVIEEYFk	Unambiguous	N-Term(iTRAQ4plex); K8(iTRAQ4plex)	HCD	2.119218	0	1	1	1.91371	2.2748	1.9883	2.3635058	10.19914946	77741.336	65399.957	148774.45	154573.17	12.19308922	2.703376565	2	High	4061	Unique	Used	86736	1	54.894	1	3.0642	0.7242	1	51	13/14	33.44706	4061	1330.71	MS2	665.861	292	Glioma_grade 2_1rep.raw
14277700			lGEWVGLck	Unambiguous	N-Term(iTRAQ4plex); C8(Methylthio); K9(iTRAQ4plex)	HCD	2.0738518	0	1	1	1.92645	2.3012	1.8835	2.2499318	10.31904596	38267.316	32035.23	73719.98	72077.086	12.5365311	1.593586922	2	High	6945	Unique	Used	69945	1	64.354	1	3.5644	-0.5551	1	98	14/16	45.01734	6945	1338.71	MS2	669.861	321	Glioma_grade 2_4.raw
14277700			lGEWVGLck	Unambiguous	N-Term(iTRAQ4plex); C8(Methylthio); K9(iTRAQ4plex)	HCD	1.8473699	0	1	1	1.73202	1.9176	1.7839	1.9750752	6.114928823	25662.174	23178.49	44447.51	45779.26	7.191670444	2.087383724	2	High	6454	Unique	Used	76717	1	62.813	1	3.3953	-4.932	1	100	15/16	29.81771	6454	1338.71	MS2	669.858	277	Glioma_grade 2_5.raw
14277700			lGEWVGLck	Unambiguous	N-Term(iTRAQ4plex); C8(Methylthio); K9(iTRAQ4plex)	HCD	2.8253899	0	1	1	2.50312	3.1107	2.5958	3.2258847	12.67387249	6781.448	5456.955	16974.752	17603.508	15.30523193	2.571544267	3	High	6871	Unique	Used	69879	1	63.725	1	3.3815	-0.132	1	51	16/32	5.087799	6871	1338.72	MS2	446.91	259	Glioma_grade 2_4.raw
14591909	NP_000960.2	RPL5	60S ribosomal protein L5 [Homo sapiens]	7	12	2.210912704	15.41897176	87.941334	30.64	2.57E-04	297	34.341	9.7153	Ribosomal subunit	Protein metabolism	Structural constituent of ribosome	Ribosome	Cytosol, Nucleolus																								
14591909			qFSQYIk	Unambiguous	N-Term(iTRAQ4plex); K7(iTRAQ4plex)	HCD	2.6826997	0	1	1	2.50392	2.6965	2.6699	2.87523	5.658881853	700026.6	650040.5	1752808.8	1869015.9	5.236111638	4.537537581	2	High	2273	Unique	Used	57959	1	28.498	1	2.9604	11.271	1	4	12-Dec	11.33017	2273	1201.7	MS2	601.351	222	Glioma_grade 2_3.raw
14591909			hImGQNVADYMR	Unambiguous	N-Term(iTRAQ4plex); M3(Oxidation)	HCD	2.197809	0	1	1	2.07736	2.1332	2.2607	2.3214996	5.115568334	199292.84	194073.94	414002.44	450542.56	1.876274138	5.977194163	2	High	1614	Unique	Used	72358	1	24.045	1	3.0468	5.3614	1	20	18/22	44.44095	1614	1594.77	MS2	797.887	100	Glioma_grade 2_5.raw
14591909			hIMGQNVADYMR	Unambiguous	N-Term(iTRAQ4plex)	HCD	2.444772	0	1	1	2.28648	2.6565	2.2626	2.6286771	8.664461702	179805.3	154762.55	411121.3	406820.78	10.58553495	0.743555547	2	High	2605	Unique	Used	73274	1	31.449	1	4.356	12.865	1	17	19/22	22.03392	2605	1578.78	MS2	789.896	170	Glioma_grade 2_5.raw
14591909			yLMEEDEDAYkk	Unambiguous	N-Term(iTRAQ4plex); K11(iTRAQ4plex); K12(iTRAQ4plex)	HCD	2.5143805	1	1	1	2.35412	2.6186	2.4207	2.6926444	6.348960273	119111.766	107082.73	280403.3	288335.72	7.520795667	1.972457586	2	High	2262	Unique	Used	79689	1	28.613	1	5.3939	2.7605	1	24	20/22	0	2262	1965.99	MS2	983.498	130	Glioma_grade 2_6.raw
14591909			gAVDGGLSIPHSTk	Unambiguous	N-Term(iTRAQ4plex); K14(iTRAQ4plex)	HCD	2.25187	0	1	1	2.0634	2.3463	2.1688	2.4661803	7.949064158	83601.07	73520.555	172502.5	181314.95	9.073226571	3.522349253	2	High	1370	Unique	Used	72139	1	22.098	1	4.6325	-1.3224	1	22	21/26	4.692359	1370	1626.9	MS2	813.955	195	Glioma_grade 2_5.raw
14591909			hIMGQNVADYMR	Unambiguous	N-Term(iTRAQ4plex)	HCD	2.508234	0	1	1	2.30764	2.5299	2.4884	2.7281547	6.866096994	66526.21	60680.887	153518.34	165546.84	6.498485743	5.331471092	2	High	3147	Unique	Used	66453	1	34.231	1	4.5733	0.4165	1	64	19/22	34.47903	3147	1578.76	MS2	789.886	124	Glioma_grade 2_4.raw
14591909			yLMEEDEDAYkk	Unambiguous	N-Term(iTRAQ4plex); K11(iTRAQ4plex); K12(iTRAQ4plex)	HCD	1.9638032	1	1	1	1.746	2.0621	1.8806	2.2210402	10.50490682	63731.816	53962.72	111275.53	119853.37	11.7385127	5.248542118	3	High	2292	Unique	Used	79717	1	28.835	1	3.7868	4.2749	1	18	23/44	20.78333	2292	1965.99	MS2	656.002	149	Glioma_grade 2_6.raw
14591909			hIMGQNVADYMR	Unambiguous	N-Term(iTRAQ4plex)	HCD	1.6301028	0	1	1	1.50689	1.6953	1.5721	1.7687229	7.223418512	59011.336	52452.13	88923.61	92773.29	8.322115233	2.99634703	3	High	2604	Unique	Used	73273	1	31.444	1	3.841	12.006	1	6	17/44	18.45847	2604	1578.78	MS2	526.932	126	Glioma_grade 2_5.raw
14591909			kQFSQYIk	Unambiguous	N-Term(iTRAQ4plex); K1(iTRAQ4plex); K8(iTRAQ4plex)	HCD	2.0373824	1	1	1	1.70167	2.1161	1.9741	2.454858	15.20568733	43817.184	35235.79	74562.27	86498.86	15.35163469	10.48104373	3	High	1667	Unique	Used	89532	1	23.94	1	4.5326	-2.6706	1	29	18/28	45.14056	1667	1473.88	MS2	491.963	196	Glioma_grade 2_7rep.raw
14591909			gAVDGGLSIPHSTk	Unambiguous	N-Term(iTRAQ4plex); K14(iTRAQ4plex)	HCD	1.6039854	0	1	1	1.64667	1.574	1.6353	1.563181	2.635431429	42227.69	44177.043	69535.23	69056.72	3.19056764	0.48827896	2	High	1486	Unique	Used	72240	1	23.072	1	3.9377	4.7552	1	29	20/26	0	1486	1626.91	MS2	813.96	220	Glioma_grade 2_5.raw
14591909			vGLTNYAAAYcTGLLLAR	Unambiguous	N-Term(iTRAQ4plex); C11(Methylthio)	HCD	2.1814194	0	1	1	1.89373	2.1325	2.2248	2.5053952	11.54919801	36876.613	32746.84	69834.17	82043.77	8.388525324	11.36898612	2	High	6063	Unique	Used	88213	1	76.729	1	5.9661	-5.4905	1	35	23/34	35.46593	6063	2060.07	MS2	1030.54	205	Glioma_grade 2_1rep.raw
14591909			iEGDMIVcAAYAHELPk	Unambiguous	N-Term(iTRAQ4plex); C8(Methylthio); K17(iTRAQ4plex)	HCD	2.2240162	0	1	1	2.13978	2.4177	2.0526	2.3191957	7.440666121	15460.222	13683.109	33081.434	31733.809	8.623644656	2.940403312	3	High	6497	Unique	Used	76754	1	63.193	1	7.7466	-3.3448	1	28	37/64	18.87978	6497	2194.09	MS2	732.034	109	Glioma_grade 2_5.raw
14916501	NP_148982.1	RPS24	40S ribosomal protein S24 isoform a [Homo sapiens]	3	5	2.575587988	31.24334643	26.33071	30	4.69E-04	130	15.059	10.887	Ribosomal subunit	Protein metabolism	Structural constituent of ribosome	Ribosome	-																								
14916501			qMVIDVLHPGk	Unambiguous	N-Term(iTRAQ4plex); K11(iTRAQ4plex)	HCD	2.575588	0	1	5	2.2726	2.6992	2.4715	2.935446	11.03514447	537127.25	452235.94	1220677.8	1327514.2	12.13451674	5.929281853	3	High	3000	Unique	Used	80335	1	34.862	1	4.6145	0.5268	1	1	24/40	11.27679	3000	1524.88	MS2	508.965	212	Glioma_grade 2_6.raw
14916501			qMVIDVLHPGk	Unambiguous	N-Term(iTRAQ4plex); K11(iTRAQ4plex)	HCD	2.8048258	0	1	5	2.66867	2.77	2.8383	2.9461493	4.158418767	166001.38	159927.3	443003.44	471169.7	2.635560122	4.35728257	2	High	2985	Unique	Used	80321	1	34.747	1	4.64	0.6757	1	29	16/20	43.81964	2985	1524.88	MS2	762.945	217	Glioma_grade 2_6.raw
14916501			tTGFGMIYDSLDYAk	Unambiguous	N-Term(iTRAQ4plex); K15(iTRAQ4plex)	HCD	3.171705	0	1	5	3.04651	3.2382	3.1092	3.3047802	3.712789061	59286.97	55777.68	180618.14	184332.97	4.313127891	1.439525137	2	High	6930	Unique	Used	54297	1	62.588	1	6.0358	-1.1804	1	34	24/28	6.338328	6930	1969.98	MS2	985.493	141	Glioma_grade 2_2.raw
14916501			tTPDVIFVFGFR	Unambiguous	N-Term(iTRAQ4plex)	HCD	1.6415143	0	1	5	1.57099	1.6303	1.6523	1.7146996	3.614766516	29529.168	28454.6	46390.094	48791.09	2.620851821	3.567428943	2	High	5582	Unique	Used	87829	1	71.949	1	4.1342	0.8257	1	100	16/22	28.91888	5582	1542.85	MS2	771.926	303	Glioma_grade 2_1rep.raw
14916501			tTGFGMIYDSLDYAk	Unambiguous	N-Term(iTRAQ4plex); K15(iTRAQ4plex)	HCD	1.5103717	0	1	5	1.47979	1.787	1.2813	1.5473052	13.69078266	8316.292	6886.572	12306.347	10655.628	13.29966126	10.16667424	3	High	6972	Unique	Used	54337	1	62.89	1	3.6822	-2.6435	1	24	21/56	29.39365	6972	1969.98	MS2	657.33	143	Glioma_grade 2_2.raw
15055539	NP_002943.2	RPS2	40S ribosomal protein S2 [Homo sapiens]	10	20	2.202753067	16.94948888	114.87629	36.18	3.40E-04	293	31.305	10.243	Ribosomal subunit	Protein metabolism	Structural constituent of ribosome	Ribosome	Mitochondrion, Cytoplasm, Nucleolus																								
15055539			aTFDAISk	Unambiguous	N-Term(iTRAQ4plex); K8(iTRAQ4plex)	HCD	2.4858801	0	1	1	2.3932	2.6263	2.3579	2.5875845	5.429777023	3794996	3458208.8	9082190	8948407	6.566601096	1.049314856	2	High	1704	Unique	Used	49378	1	24.736	1	3.5691	-0.2238	1	1	14/14	6.985296	1704	1140.65	MS2	570.829	183	Glioma_grade 2_2.raw
15055539			aTFDAISk	Unambiguous	N-Term(iTRAQ4plex); K8(iTRAQ4plex)	HCD	2.5646834	0	1	1	2.45729	2.5913	2.5394	2.677937	3.605833149	536119.06	508392.72	1317398.4	1361443.8	3.75399941	2.325243422	2	High	1727	Unique	Used	57450	1	24.462	1	3.3761	-1.7221	1	7	14/14	28.27714	1727	1140.65	MS2	570.828	184	Glioma_grade 2_3.raw
15055539			tYSYLTPDLWk	Unambiguous	N-Term(iTRAQ4plex); K11(iTRAQ4plex)	HCD	2.4636707	0	1	1	2.32645	2.4968	2.4328	2.6109326	4.826482034	462248	430715.4	1075398.9	1124568.9	4.993914709	3.160813575	2	High	6300	Unique	Used	53710	1	57.873	1	4.9706	-1.6933	1	5	17/20	6.516219	6300	1674.9	MS2	837.951	217	Glioma_grade 2_2.raw
15055539			gcTATLGNFAk	Unambiguous	N-Term(iTRAQ4plex); C2(Methylthio); K11(iTRAQ4plex)	HCD	1.5407877	0	1	1	1.63957	1.5095	1.5747	1.4498374	5.307534231	459722.66	499324.4	753748.75	723939.25	5.839684009	2.852902588	2	High	3991	Unique	Used	59560	1	41.477	1	5	1.0629	1	8	20/20	22.76945	3991	1416.72	MS2	708.865	164	Glioma_grade 2_3.raw
15055539			sPYQEFTDHLVk	Unambiguous	N-Term(iTRAQ4plex); K12(iTRAQ4plex)	HCD	2.5325973	0	1	1	2.13403	2.466	2.5902	2.9931633	13.94343748	294791.84	255108.25	629094.8	763580.6	10.20568503	13.65656651	3	High	4264	Unique	Used	74769	1	44.762	1	4.9104	0.1052	1	1	27/44	8.76592	4264	1751.92	MS2	584.645	168	Glioma_grade 2_5.raw
15055539			aEDkEWMPVTk	Unambiguous	N-Term(iTRAQ4plex); K4(iTRAQ4plex); K11(iTRAQ4plex)	HCD	2.538855	1	1	1	2.27335	2.7785	2.3428	2.8633559	11.67774878	209992.53	171813.11	477387.1	491962.1	14.14171188	2.126391879	2	High	1931	Unique	Used	79381	1	26.143	1	4.149	4.0906	1	12	16/20	8.007225	1931	1765.96	MS2	883.483	220	Glioma_grade 2_6.raw
15055539			sPYQEFTDHLVk	Unambiguous	N-Term(iTRAQ4plex); K12(iTRAQ4plex)	HCD	2.4023752	0	1	1	2.31796	3.0185	1.9292	2.5123086	18.53183519	197818.36	151905.72	458534.47	381634.03	18.56614454	12.94426597	3	High	4168	Unique	Used	74682	1	43.986	1	4.5959	-0.2084	1	3	24/44	41.98332	4168	1751.92	MS2	584.645	221	Glioma_grade 2_5.raw
15055539			gTGIVSAPVPk	Unambiguous	N-Term(iTRAQ4plex); K11(iTRAQ4plex)	HCD	2.7764375	0	1	1	2.96744	2.4997	3.1049	2.615538	10.22137656	139284.69	165346.77	413319.5	432470.75	12.09899562	3.202207342	2	High	1573	Unique	Used	57307	1	23.313	1	4.5661	-1.3077	1	13	19/20	8.78832	1573	1313.8	MS2	657.404	146	Glioma_grade 2_3.raw
15055539			aEDkEWMPVTk	Unambiguous	N-Term(iTRAQ4plex); K4(iTRAQ4plex); K11(iTRAQ4plex)	HCD	2.0630054	1	1	1	2.04056	2.2367	1.9045	2.0876093	6.627610071	124751.75	113811.484	254563.33	237593.9	6.485438805	4.87616489	3	High	1896	Unique	Used	79348	1	25.892	1	4.4399	3.288	1	8	23/40	12.83127	1896	1765.96	MS2	589.324	174	Glioma_grade 2_6.raw
15055539			eSEIIDFFLGASLkDEVLk	Unambiguous	N-Term(iTRAQ4plex); K14(iTRAQ4plex); K19(iTRAQ4plex)	HCD	2.2330723	1	1	1	1.86852	2.6988	1.9106	2.7596154	21.03156209	95837.375	66353.336	179073.9	183109.69	25.70845616	1.575849682	3	High	7450	Unique	Used	84029	1	76.03	1	5.9936	1.4685	1	6	33/72	7.584864	7450	2585.45	MS2	862.487	81	Glioma_grade 2_6.raw
15055539			sPYQEFTDHLVk	Unambiguous	N-Term(iTRAQ4plex); K12(iTRAQ4plex)	HCD	1.9521525	0	1	1	1.87295	2.02	1.8893	2.0375736	4.389346255	91542.01	84879.65	171453.44	172948.53	5.340613998	0.613926963	2	High	4173	Unique	Used	74687	1	44.015	1	4.6482	1.5412	1	41	17/22	38.04519	4173	1751.92	MS2	876.465	295	Glioma_grade 2_5.raw
15055539			iGkPHTVPck	Unambiguous	N-Term(iTRAQ4plex); K3(iTRAQ4plex); C9(Methylthio); K10(iTRAQ4plex)	HCD	2.0395744	0	1	1	1.79043	2.0426	2.0369	2.3238115	10.64202282	61103.54	53559.773	109401.53	124462.82	9.304194449	9.107792866	3	High	1480	Unique	Used	85028	1	23.842	1	3.3322	-5.0312	1	27	14/36	25.56813	1480	1557.89	MS2	519.968	179	Glioma_grade 2_1rep.raw
15055539			sPYQEFTDHLVk	Unambiguous	N-Term(iTRAQ4plex); K12(iTRAQ4plex)	HCD	2.939448	0	1	1	2.48529	3.0342	2.8618	3.4939084	14.09399971	59282.617	48557.984	147334.7	169657.14	14.06420337	9.958835973	2	High	4288	Unique	Used	74791	1	44.948	1	4.8594	2.8651	1	32	17/22	25.27795	4288	1751.93	MS2	876.467	250	Glioma_grade 2_5.raw
15055539			aEDkEWmPVTk	Unambiguous	N-Term(iTRAQ4plex); K4(iTRAQ4plex); M7(Oxidation); K11(iTRAQ4plex)	HCD	1.8928794	1	1	1	1.72638	2.2367	1.6275	2.1085873	15.25869486	53076.715	40967.64	91630.766	86383.85	18.20929932	4.16834299	3	High	1270	Unique	Used	78764	1	21.306	1	4.0525	-1.4233	1	16	16/40	16.78778	1270	1781.94	MS2	594.653	196	Glioma_grade 2_6.raw

15055539			iGkPHTVPck	Unambiguous	N-Term(iTRAQ4plex); K3(iTRAQ4plex); C9(Methylthio); K10(iTRAQ4plex)	HCD	2.194323	0	1	1	2.11642	2.3635	2.0428	2.2813256	6.689165955	31422.457	28137.504	66502.984	64190.812	7.799912905	2.501958854	2	High	1494	Unique	Used	85038	1	23.996	1	3.4094	-3.1028	1	100	14/18	0	1494	1557.89	MS2	779.45	233	Glioma_grade 2_1rep.raw
15055539			gcTATLGNFAk	Unambiguous	N-Term(iTRAQ4plex); C2(Methylthio); K11(iTRAQ4plex)	HCD	1.8022234	0	1	1	1.66538	1.721	1.8808	1.9436347	7.266909643	28777.219	27846.666	47925.145	54123.746	2.324108763	8.590142936	3	High	3979	Unique	Used	59549	1	41.378	1	4.2546	0.8165	1	33	19/40	66.13356	3979	1416.72	MS2	472.913	170	Glioma_grade 2_3.raw
15055539			eSEIIDFFLGASLk	Unambiguous	N-Term(iTRAQ4plex); K14(iTRAQ4plex)	HCD	1.8039919	0	1	1	1.74656	1.9039	1.7124	1.8666005	5.105382772	28145.926	25820.354	49158.45	48196.285	6.094278617	1.397679109	2	High	8694	Unique	Used	55907	1	76.711	1	4.3765	1.2988	1	32	21/26	34.25504	8694	1857.03	MS2	929.017	187	Glioma_grade 2_2.raw
15055539			sLEEIYLFSLPIk	Unambiguous	N-Term(iTRAQ4plex); K13(iTRAQ4plex)	HCD	1.75375	0	1	1	1.64565	1.6861	1.8198	1.8645098	5.969220422	17096.404	16686.133	28134.666	31111.457	1.717487388	7.105643359	2	High	8393	Unique	Used	55627	1	74.441	1	4.1015	1.3459	1	72	19/24	5.587674	8393	1840.07	MS2	920.54	123	Glioma_grade 2_2.raw
15055539			sLEEIYLFSLPIk	Unambiguous	N-Term(iTRAQ4plex); K13(iTRAQ4plex)	HCD	2.211183	0	1	1	2.117	2.3015	2.1281	2.3135767	4.830416774	13835.992	12726.984	29290.746	29444.855	5.904361599	0.371057815	2	High	7975	Unique	Used	63227	1	73.277	1	4.7438	1.943	1	100	19/24	23.475	7975	1840.07	MS2	920.541	126	Glioma_grade 2_3.raw
15055539			sLEEIYLFSLPIk	Unambiguous	N-Term(iTRAQ4plex); K13(iTRAQ4plex)	HCD	1.9236915	0	1	1	1.82081	1.8937	1.9526	2.030691	4.625092605	13083.266	12579.899	23822.125	25545.89	2.773891834	4.93795799	2	High	8310	Unique	Used	55552	1	73.743	1	3.9878	-0.6443	1	100	19/24	11.02248	8310	1840.07	MS2	920.539	134	Glioma_grade 2_2.raw
15431288	NP_009035.3	RPL10A	60S ribosomal protein L10a [Homo sapiens]	6	18	2.321801662	22.04666515	88.391596	35.02	7.24E-05	217	24.816	9.9351	Ribosomal subunit	Protein metabolism	Structural constituent of ribosome	Ribosome	Nucleolus																								
15431288			fPSLLTHNENMVAk	Unambiguous	N-Term(iTRAQ4plex); K14(iTRAQ4plex)	HCD	2.9583423	0	1	1	2.92247	3.0382	2.8815	2.9956384	2.384846918	479753	461481.44	1402062.2	1382431.4	2.745319004	0.997026662	3	High	3703	Unique	Used	80968	1	40.496	1	5.1943	2.8407	1	2	26/52	12.37385	3703	1889.02	MS2	630.346	186	Glioma_grade 2_6.raw
15431288			aVDIPHMDIEALk	Unambiguous	N-Term(iTRAQ4plex); K13(iTRAQ4plex)	HCD	2.3597753	0	1	1	2.42607	2.4635	2.2576	2.2924566	4.245671479	211289.98	208080.56	512604.7	477015.66	1.08228997	5.085839487	3	High	5086	Unique	Used	75499	1	51.547	1	5.194	0.1731	1	1	25/48	9.82282	5086	1739.96	MS2	580.658	172	Glioma_grade 2_5.raw
15431288			iLGPGLNk	Unambiguous	N-Term(iTRAQ4plex); K8(iTRAQ4plex)	HCD	1.9725453	0	1	1	2.04623	1.9345	2.0128	1.9028841	3.378335946	138398.89	146394.2	283196.06	278571.2	3.970277452	1.164279267	2	High	2076	Unique	Used	65453	1	26.246	1	3.0846	-0.4222	1	42	13/14	47.26417	2076	1099.71	MS2	550.357	66	Glioma_grade 2_4.raw
15431288			fPSLLTHNENMVAk	Unambiguous	N-Term(iTRAQ4plex); K14(iTRAQ4plex)	HCD	2.6034963	0	1	1	2.53986	2.435	2.7792	2.6645107	5.738379127	87332.65	91091.914	221812.34	242715.39	2.979635771	6.363748576	2	High	3696	Unique	Used	80962	1	40.429	1	5.3704	2.1046	1	44	16/26	38.58283	3696	1889.02	MS2	945.015	168	Glioma_grade 2_6.raw
15431288			aVDIPHMDIEALk	Unambiguous	N-Term(iTRAQ4plex); K13(iTRAQ4plex)	HCD	2.1529808	0	1	1	2.12855	2.0435	2.267	2.1764338	4.332241534	81304.21	84686.98	173060.23	184315.6	2.882056097	4.453993686	2	High	5091	Unique	Used	75504	1	51.573	1	4.7211	1.128	1	20	18/24	19.51117	5091	1739.96	MS2	870.485	182	Glioma_grade 2_5.raw
15431288			aVDIPHmDIEALk	Unambiguous	N-Term(iTRAQ4plex); M7(Oxidation); K13(iTRAQ4plex)	HCD	2.572174	0	1	1	2.58343	2.4306	2.7227	2.5615854	4.644921912	67364.7	71600.414	174031.88	183410.58	4.310581277	3.710662896	3	High	3345	Unique	Used	73958	1	37.075	1	4.359	0.8428	1	3	22/48	12.79326	3345	1755.96	MS2	585.991	198	Glioma_grade 2_5.raw
15431288			kYDAFLASESLIk	Unambiguous	N-Term(iTRAQ4plex); K1(iTRAQ4plex); K13(iTRAQ4plex)	HCD	2.8777273	1	1	1	3.06142	2.7952	2.9681	2.7100093	5.545506446	62585.723	68547	191601.19	185763.02	6.428996966	2.187917925	2	High	4495	Unique	Used	91837	1	50.393	1	6.3219	-1.1513	1	47	22/24	11.15604	4495	1917.1	MS2	959.055	119	Glioma_grade 2_7rep.raw
15431288			fPSLLTHNENmVAk	Unambiguous	N-Term(iTRAQ4plex); M11(Oxidation); K14(iTRAQ4plex)	HCD	2.3192995	0	1	1	2.19068	2.3987	2.2468	2.460137	5.434735274	55560.312	50741.195	121714.75	124830.29	6.411254941	1.787113187	3	High	2876	Unique	Used	80225	1	33.829	1	4.4657	0.4559	1	10	26/52	10.14243	2876	1905.02	MS2	635.677	209	Glioma_grade 2_6.raw
15431288			aVDIPHMDIEALk	Unambiguous	N-Term(iTRAQ4plex); K13(iTRAQ4plex)	HCD	1.8655075	0	1	1	1.87253	1.7573	1.9808	1.8589153	4.897265953	52943.098	56415.26	99137.64	104871.2	4.490172202	3.974568671	2	High	4889	Unique	Used	81975	1	51.118	1	4.5854	-1.538	1	57	17/24	35.54214	4889	1739.96	MS2	870.483	215	Glioma_grade 2_6.raw
15431288			aVDIPHMDIEALk	Unambiguous	N-Term(iTRAQ4plex); K13(iTRAQ4plex)	HCD	2.3572752	0	1	1	2.30965	2.1806	2.5444	2.4022377	6.499416497	44526.594	47162.543	102840.89	113295.63	4.065688638	6.840692678	3	High	4871	Unique	Used	81958	1	50.99	1	5.7774	0.2784	1	17	22/48	38.59005	4871	1739.96	MS2	580.659	177	Glioma_grade 2_6.raw
15431288			aVDIPHMDIEALk	Unambiguous	N-Term(iTRAQ4plex); K13(iTRAQ4plex)	HCD	1.7339301	0	1	1	1.90321	1.7118	1.7585	1.5816718	7.632208935	39464.266	43876.207	75108.8	69397.76	7.48667073	5.589109742	3	High	5174	Unique	Used	75577	1	52.26	1	4.3763	-1.9316	1	11	24/48	10.18433	5174	1739.96	MS2	580.657	166	Glioma_grade 2_5.raw
15431288			fPSLLTHNENmVAk	Unambiguous	N-Term(iTRAQ4plex); M11(Oxidation); K14(iTRAQ4plex)	HCD	2.3243036	0	1	1	2.18881	2.4708	2.1945	2.4772525	6.989497589	24471.463	21678.613	53563.387	53703.402	8.558352857	0.184596848	2	High	2864	Unique	Used	80215	1	33.716	1	3.6012	0.7193	1	86	13/26	0	2864	1905.02	MS2	953.012	203	Glioma_grade 2_6.raw
15431288			fSVcVLGDQQHcDEAk	Unambiguous	N-Term(iTRAQ4plex); C4(Methylthio); C12(Methylthio); K16(iTRAQ4plex)	HCD	2.0862927	0	1	1	2.03738	1.9329	2.2479	2.132699	6.437249284	22569.168	23788.65	45981.945	50734.03	3.720209574	6.948658747	3	High	4895	Unique	Used	75328	1	49.998	1	4.3907	-0.9558	1	19	23/60	50.97626	4895	2158.96	MS2	720.325	39	Glioma_grade 2_5.raw
15431288			iLGPGLNk	Unambiguous	N-Term(iTRAQ4plex); K8(iTRAQ4plex)	HCD	3.2576299	0	1	1	3.0396	3.5031	3.0446	3.5089004	8.180423828	22149.39	19218.877	67325.375	67437.125	10.01823748	0.117271767	3	High	2116	Unique	Used	65491	1	26.532	1	3.4035	-0.6564	1	100	17/28	36.95307	2116	1099.71	MS2	367.241	60	Glioma_grade 2_4.raw
15431288			aVDIPHmDIEALk	Unambiguous	N-Term(iTRAQ4plex); M7(Oxidation); K13(iTRAQ4plex)	HCD	1.5752629	0	1	1	1.57461	1.6348	1.5179	1.5759459	3.028955813	21920.16	21112.93	34515.598	33272.836	2.652832074	2.592670713	2	High	3278	Unique	Used	80591	1	36.986	1	3.0024	0.8157	1	100	14/24	25.3264	3278	1755.96	MS2	878.482	259	Glioma_grade 2_6.raw
15431288			aVDIPHmDIEALk	Unambiguous	N-Term(iTRAQ4plex); M7(Oxidation); K13(iTRAQ4plex)	HCD	1.527877	0	1	1	1.48898	1.6585	1.4106	1.5712074	6.959781183	20821.016	18692.943	31001.977	29370.49	7.616421575	3.821727282	2	High	3373	Unique	Used	73985	1	37.263	1	3.1257	0.8157	1	86	15/24	32.45809	3373	1755.96	MS2	878.482	253	Glioma_grade 2_5.raw
15431288			fPSLLTHNENmVAk	Unambiguous	N-Term(iTRAQ4plex); M11(Oxidation); K14(iTRAQ4plex)	HCD	2.1844661	0	1	1	2.07063	2.1118	2.2558	2.300561	5.06983476	16401.506	16082.044	33961.492	36997.723	1.390819332	6.051193011	3	High	2767	Unique	Used	80136	1	32.78	1	5.0194	4.7812	1	65	22/52	0	2767	1905.02	MS2	635.679	231	Glioma_grade 2_6.raw
15431288			vLcLAVAVGHVk	Unambiguous	N-Term(iTRAQ4plex); C3(Methylthio); K12(iTRAQ4plex)	HCD	3.2080076	0	1	1	2.73322	3.6923	2.8495	3.8494017	17.40588265	8057.222	5964.3545	22022.127	22959.197	21.10862215	2.946149613	3	High	4966	Unique	Used	82041	1	51.792	1	5.454	0.0552	1	80	29/44	63.42978	4966	1542.91	MS2	514.975	167	Glioma_grade 2_6.raw
15431301	NP_000962.2	RPL7	60S ribosomal protein L7 [Homo sapiens]	5	10	2.424465179	24.53283941	47.47169	23.39	5.21E-05	248	29.207	10.653	Ribosomal subunit	Protein metabolism	Structural constituent of ribosome	Ribosome	Cytosol, Cytoplasm, Mitochondrion, Nucleolus, Nucleus																								
15431301			eVPAVPETLk	Unambiguous	N-Term(iTRAQ4plex); K10(iTRAQ4plex)	HCD	2.5167062	0	1	1	2.60012	2.4839	2.5511	2.4370255	2.860059818	614157.75	642901.25	1596881.4	1566766.8	3.233694483	1.346182415	2	High	2748	Unique	Used	50366	1	31.994	1	3.477	-0.5235	1	3	14/18	4.37562	2748	1370.81	MS2	685.91	185	Glioma_grade 2_2.raw
15431301			sVNELIYk	Unambiguous	N-Term(iTRAQ4plex); K8(iTRAQ4plex)	HCD	2.317032	0	1	1	2.19122	2.4087	2.2337	2.4553246	5.569651708	513495.44	467142.34	1125183.2	1146986	6.684749865	1.357021099	2	High	2528	Unique	Used	50159	1	30.432	1	3.4866	-0.0612	1	15	13/14	53.14286	2528	1253.73	MS2	627.371	240	Glioma_grade 2_2.raw
15431301			aGNFYVPAEPk	Unambiguous	N-Term(iTRAQ4plex); K11(iTRAQ4plex)	HCD	1.9866768	0	1	1	1.94247	1.933	2.0407	2.0306716	2.860832561	255243.55	256499.55	495801.8	520866.3	0.347098424	3.48654156	2	High	2542	Unique	Used	50172	1	30.534	1	3.9528	0.1671	1	10	16/20	25.81933	2542	1480.8	MS2	740.906	219	Glioma_grade 2_2.raw
15431301			eANNFLWPFk	Unambiguous	N-Term(iTRAQ4plex); K10(iTRAQ4plex)	HCD	3.2449036	0	1	1	3.03199	3.4931	3.0295	3.4901953	8.15940875	194200.11	168566.53	588812.6	588330.1	9.993023749	0.057967317	2	High	6990	Unique	Used	54354	1	63.021	1	4.2699	1.2438	1	12	14/18	5.409845	6990	1553.84	MS2	777.422	264	Glioma_grade 2_2.raw
15431301			eANNFLWPFk	Unambiguous	N-Term(iTRAQ4plex); K10(iTRAQ4plex)	HCD	3.005431	0	1	1	2.98556	3.0044	3.0064	3.025424	0.542142637	170997.75	169924.5	510524.6	514093.62	0.445205529	0.492608504	2	High	6854	Unique	Used	69863	1	63.598	1	4.0141	1.0081	1	13	14/18	21.92476	6854	1553.84	MS2	777.422	276	Glioma_grade 2_4.raw
15431301			eANNFLWPFk	Unambiguous	N-Term(iTRAQ4plex); K10(iTRAQ4plex)	HCD	2.989811	0	1	1	2.77364	3.265	2.756	3.2442782	9.403564743	168126.06	142822.78	466321.4	463356.88	11.50808016	0.450958625	2	High	6731	Unique	Used	62107	1	62.846	1	4.2536	1.558	1	15	14/18	13.84067	6731	1553.84	MS2	777.423	263	Glioma_grade 2_3.raw
15431301			yGIIcMEDLIHEIYTVGk	Unambiguous	N-Term(iTRAQ4plex); C5(Methylthio); K18(iTRAQ4plex)	HCD	2.332224	0	1	1	2.10114	2.3545	2.3123	2.5911708	8.587442699	44723.375	39910.297	93970.28	103414.39	8.042567483	6.766477076	3	High	7574	Unique	Used	84139	1	77.029	1	7.1966	-2.2927	1	14	29/68	27.21421	7574	2431.23	MS2	811.08	143	Glioma_grade 2_6.raw
15431301			eANNFLWPFk	Unambiguous	N-Term(iTRAQ4plex); K10(iTRAQ4plex)	HCD	1.5787255	0	1	1	1.5538	1.5437	1.614	1.6034893	2.224748179	28843.74	29032.023	44817.41	46552.535	0.460075787	2.685606637	2	High	6838	Unique	Used	62208	1	63.653	1	3.4601	0.6939	1	100	14/18	44.45364	6838	1553.84	MS2	777.422	283	Glioma_grade 2_3.raw
15431301			yGIIcmEDLIHEIYTVGk	Unambiguous	N-Term(iTRAQ4plex); C5(Methylthio); M6(Oxidation); K18(iTRAQ4plex)	HCD	1.5793865	0	1	1	1.71029	1.473	1.703	1.466652	8.612554136	9929.658	11529.569	16982.545	16909.867	10.54379002	0.30326025	3	High	7085	Unique	Used	83756	1	72.128	1	6.0662	0.7479	1	65	25/68	23.05177	7085	2447.23	MS2	816.414	137	Glioma_grade 2_6.raw
15431301			eANNFLWPFk	Unambiguous	N-Term(iTRAQ4plex); K10(iTRAQ4plex)	HCD	2.9528627	0	1	1	2.75233	2.9427	2.9623	3.167275	5.738601408	5982.503	5595.377	16465.793	17722.098	4.728662238	5.196821206	3	High	6744	Unique	Used	62120	1	62.934	1	3.6823	0.2531	1	100	17/36	24.29058	6744	1553.84	MS2	518.617	255	Glioma_grade 2_3.raw
15431306	NP_150644.1	RPL8	60S ribosomal protein L8 [Homo sapiens]	2	6	3.316642523	20.86552183	41.141999	8.95	2.66E-05	257	28.007	11.034	Ribosomal subunit	Protein metabolism	Structural constituent of ribosome	Nucleolus	Mitochondrion																								
15431306			iDkPILk	Unambiguous	N-Term(iTRAQ4plex); K3(iTRAQ4plex); K7(iTRAQ4plex)	HCD	3.2581363	0	1	1	3.13356	3.2145	3.3007	3.3859246	3.341261614	331064.47	322731.4	1037411.8	1092744.2	1.802510715	3.673525813	2	High	1437	Unique	Used	89328	1	22.101	1	3.0005	-2.2522	1	12	12-Dec	10.27841	1437	1258.84	MS2	629.925	33	Glioma_grade 2_7rep.raw
15431306			aSGNYATVISHNPETk	Unambiguous	N-Term(iTRAQ4plex); K16(iTRAQ4plex)	HCD	3.5743654	0	1	1	3.85206	2.6325	4.9526	3.3845868	26.21652984	135332.1	198028.58	521307.9	670244.94	26.59768162	17.67683101	3	High	1318	Unique	Used	72090	1	21.73	1	5.4872	2.1155	1	1	30/60	5.616711	1318	1977.03	MS2	659.682	131	Glioma_grade 2_5.raw
15431306			aSGNYATVISHNPETk	Unambiguous	N-Term(iTRAQ4plex); K16(iTRAQ4plex)	HCD	3.466873	0	1	1	3.30758	3.6694	3.2843	3.6435852	6.001407825	83623.82	75378.945	276592.78	274649.6	7.333214643	0.498523265	2	High	1509	Unique	Used	64935	1	21.858	1	6.1398	0.0539	1	10	22/30	4.033935	1509	1977.03	MS2	989.018	184	Glioma_grade 2_4.raw
15431306			aSGNYATVISHNPETk	Unambiguous	N-Term(iTRAQ4plex); K16(iTRAQ4plex)	HCD	3.3389442	0	1	1	3.83128	3.1836	3.5259	2.929835	11.6974097	49208.195	59218.94	188530.42	173501.73	13.05700031	5.870687789	3	High	1471	Unique	Used	64899	1	21.599	1	6.0706	1.8376	1	3	31/60	3.377697	1471	1977.03	MS2	659.682	139	Glioma_grade 2_4.raw
15431306			aSGNYATVISHNPETk	Unambiguous	N-Term(iTRAQ4plex); K16(iTRAQ4plex)	HCD	3.2943408	0	1	1	3.27172	3.569	3.0426	3.3190224	6.539621754	38508.926	35301.57	125990.23	117166.7	6.145313472	5.131811704	2	High	1312	Unique	Used	72085	1	21.674	1	6.964	1.5357	1	41	22/30	0	1312	1977.03	MS2	989.019	180	Glioma_grade 2_5.raw
15431306			aSGNYATVISHNPETk	Unambiguous	N-Term(iTRAQ4plex); K16(iTRAQ4plex)	HCD	1.8156254	0	1	1	1.80007	1.7361	1.8981	1.830626	3.706333126	8003.416	8298.4	14406.726	15191.269	2.559042339	3.748603076	3	High	1430	Unique	Used	72189	1	22.648	1	6.5519	2.6712	1	55	29/60	28.14175	1430	1977.03	MS2	659.683	134	Glioma_grade 2_5.raw
15718687	NP_000996.2	RPS3	40S ribosomal protein S3 [Homo sapiens]	7	23	2.47321701	19.78059639	127.59173	34.57	4.16E-05	243	26.671	9.6567	Ribosomal subunit	Protein metabolism	Structural constituent of ribosome	Cytoplasm	Ribosome, Nucleus																								
15718687			fVADGIFk	Unambiguous	N-Term(iTRAQ4plex); K8(iTRAQ4plex)	HCD	1.947843	0	1	1	1.84858	2.0363	1.8675	2.0571835	5.611646569	522742.28	474543.2	966333.06	976222.44	6.834932825	0.719963745	2	High	4663	Unique	Used	52165	1	46.006	1	3.4543	-1.4318	1	10	14/14	21.41964	4663	1184.69	MS2	592.849	231	Glioma_grade 2_2.raw
15718687			fVADGIFk	Unambiguous	N-Term(iTRAQ4plex); K8(iTRAQ4plex)	HCD	2.261399	0	1	1	2.14667	2.2304	2.2912	2.3806043	4.362338812	475178.84	457333.62	1020052.4	1088730.4	2.706339404	4.605754516	2	High	4516	Unique	Used	60047	1	45.587	1	3.4346	0.629	1	9	14/14	22.15688	4516	1184.69	MS2	592.85	228	Glioma_grade 2_3.raw
15718687			kFVADGIFk	Unambiguous	N-Term(iTRAQ4plex); K1(iTRAQ4plex); K9(iTRAQ4plex)	HCD	2.7909293	1	1	1	2.3551	2.8994	2.7028	3.3274872	14.37927841	393478.22	319613.28	926678.94	1063509.1	14.64900366	9.723054512	3	High	2721	Unique	Used	90441	1	32.98	1	4.7703	-1.4118	1	3	19/32	44.5729	2721	1456.89	MS2	486.3	97	Glioma_grade 2_7rep.raw
15718687			fIMESGAk	Unambiguous	N-Term(iTRAQ4plex); K8(iTRAQ4plex)	HCD	1.7925698	0	1	1	1.75829	1.7789	1.806	1.8272538	1.687479501	298422.9	294957.94	524713.5	538963.06	0.825809176	1.894553454	2	High	1777	Unique	Used	57498	1	24.802	1	2.9186	-0.6541	1	13	14-Dec	26.25243	1777	1170.64	MS2	585.825	199	Glioma_grade 2_3.raw
15718687			kFVADGIFk	Unambiguous	N-Term(iTRAQ4plex); K1(iTRAQ4plex); K9(iTRAQ4plex)	HCD	3.0508022	1	1	1	2.89702	3.2818	2.8469	3.2250085	7.26041218	247374.78	218374.02	716649.4	704258.06	8.805877355	1.233296437	3	High	2742	Unique	Used	90459	1	33.161	1	4.4667	12.539	1	3	18/32	25.31006	2742	1456.91	MS2	486.307	62	Glioma_grade 2_7rep.raw
15718687			fGFPEGSVELYAEk	Unambiguous	N-Term(iTRAQ4plex); K14(iTRAQ4plex)	HCD	2.622677	0	1	1	2.59432	2.5958	2.6496	2.6510484	1.217152911	235158.34	235027.88	610076.4	623070.25	0.039239411	1.490177904	2	High	6239	Unique	Used	53652	1	57.441	1	5.2129	1.552	1	7	22/26	15.32576	6239	1860.97	MS2	930.986	202	Glioma_grade 2_2.raw
15718687			iMLPWDPTGk	Unambiguous	N-Term(iTRAQ4plex); K10(iTRAQ4plex)	HCD	2.528845	0	1	1	2.39128	2.579	2.4823	2.6772127	4.866707764	96084.16	89089.164	229763.98	238510.64	5.342248008	2.641536541	2	High	6444	Unique	Used	69491	1	60.217	1	3.5842	1.2864	1	30	13/18	20.90553	6444	1445.81	MS2	723.408	265	Glioma_grade 2_4.raw
15718687			kPLPDHVSIVEPk	Unambiguous	N-Term(iTRAQ4plex); K1(iTRAQ4plex); K13(iTRAQ4plex)	HCD	2.8348584	0	1	1	2.71359	2.5243	3.1687	2.9476693	9.866080466	91290.28	98136.22	247724.22	289273.12	5.111017305	10.94214319	3	High	2069	Unique	Used	85465	1	30.181	1	5.902	-8.2088	1	4	23/48	9.869469	2069	1891.12	MS2	631.046	103	Glioma_grade 2_1rep.raw
15718687			kPLPDHVSIVEPk	Unambiguous	N-Term(iTRAQ4plex); K1(iTRAQ4plex); K13(iTRAQ4plex)	HCD	3.1170702	0	1	1	2.7398	3.5823	2.7612	3.6103604	15.3952785	69158.93	52893.156	189481.56	190963.36	18.84709958	0.550823929	3	High	2114	Unique	Used	85495	1	30.724	1	4.617	1.0863	1	6	22/48	5.379607	2114	1891.14	MS2	631.051	76	Glioma_grade 2_1rep.raw
15718687			imLPWDPTGk	Unambiguous	N-Term(iTRAQ4plex); M2(Oxidation); K10(iTRAQ4plex)	HCD	2.4510071	0	1	1	2.42935	2.4911	2.4119	2.4732141	1.506846371	67370.41	65701.16	163666.33	162493.03	1.773989733	0.508738051	2	High	6009	Unique	Used	69095	1	56.687	1	3.657	-0.176	1	35	13/18	10.55068	6009	1461.8	MS2	731.404	276	Glioma_grade 2_4.raw
15718687			iMLPWDPTGk	Unambiguous	N-Term(iTRAQ4plex); K10(iTRAQ4plex)	HCD	1.96265	0	1	1	1.91303	1.9521	1.973	2.013287	2.130024594	66758.984	65422.277	127711.73	131713.83	1.430149141	2.181675583	2	High	6367	Unique	Used	61768	1	59.998	1	3.4508	2.6373	1	100	13/18	10.34454	6367	1445.81	MS2	723.409	272	Glioma_grade 2_3.raw

15718687			iMLPWDPTGk	Unambiguous	N-Term(iTRAQ4plex); K10(iTRAQ4plex)	HCD	1.5314407	0	1	1	1.44693	1.6355	1.4394	1.6269656	7.069752902	49909.004	44155.105	72214.77	71838.836	8.650740531	0.369064667	2	High	6589	Unique	Used	53982	1	59.966	1	3.0554	0.1044	1	51	13/18	11.2532	6589	1445.81	MS2	723.407	251	Glioma_grade2_2.raw
15718687			kPLPDHVSIVEPk	Unambiguous	N-Term(iTRAQ4plex); K1(iTRAQ4plex); K13(iTRAQ4plex)	HCD	2.6451516	0	1	1	2.7499	2.5527	2.7448	2.5479186	4.295961925	43611.41	46980.75	119926.87	119703.12	5.259799881	0.132049534	3	High	2194	Unique	Used	94968	1	31.832	1	4.8743	1.28	1	10	23/48	9.131681	2194	1891.14	MS2	631.052	76	Glioma_grade2_8rep.raw
15718687			kPLPDHVSIVEPk	Unambiguous	N-Term(iTRAQ4plex); K1(iTRAQ4plex); K13(iTRAQ4plex)	HCD	2.4946706	0	1	1	2.51977	2.5023	2.487	2.4697497	0.856051815	37855.094	38119.215	95385.95	94144.914	0.491644485	0.926018014	2	High	2071	Unique	Used	85467	1	30.19	1	4.8176	-10.784	1	100	18/24	23.51158	2071	1891.12	MS2	946.062	105	Glioma_grade2_1rep.raw
15718687			kPLPDHVSIVEPk	Unambiguous	N-Term(iTRAQ4plex); K1(iTRAQ4plex); K13(iTRAQ4plex)	HCD	2.8459582	0	1	1	2.73152	2.8457	2.8462	2.965176	3.350693064	29630.123	28441.717	80935.33	84334.695	2.894104755	2.908832431	2	High	2107	Unique	Used	85491	1	30.629	1	4.8289	-0.0685	1	94	18/24	10.56841	2107	1891.14	MS2	946.072	80	Glioma_grade2_1rep.raw
15718687			kPLPDHVSIVEPk	Unambiguous	N-Term(iTRAQ4plex); K1(iTRAQ4plex); K13(iTRAQ4plex)	HCD	2.5024073	0	1	1	2.5455	2.3801	2.6332	2.4621181	4.34277629	17442.861	18654.88	44400.74	45930.52	4.748368347	2.395002155	2	High	2183	Unique	Used	94960	1	31.706	1	4.5497	0.8998	1	100	19/24	0	2183	1891.14	MS2	946.073	76	Glioma_grade2_8rep.raw
15718687			fVDGLMIHSGDPVNYYVDTAVR	Unambiguous	N-Term(iTRAQ4plex)	HCD	2.159337	0	1	1	2.09483	1.9832	2.3454	2.220406	7.243460699	16945.04	17898.66	35496.996	39742.29	3.870491186	7.979544557	3	High	6928	Unique	Used	54295	1	62.576	1	6.8508	1.2303	1	19	30/84	12.70161	6928	2612.3	MS2	871.437	118	Glioma_grade2_2.raw
15718687			fVDGLMIHSGDPVNYYVDTAVR	Unambiguous	N-Term(iTRAQ4plex)	HCD	2.0458934	0	1	1	2.05356	2.0804	2.0118	2.0381222	1.404059725	16182.43	15973.37	33231.66	32555.68	0.919446841	1.453136856	3	High	6682	Unique	Used	62062	1	62.449	1	6.959	2.2116	1	32	28/84	26.31134	6682	2612.3	MS2	871.438	135	Glioma_grade2_3.raw
15718687			fVDGLmIHSGDPVNYYVDTAVR	Unambiguous	N-Term(iTRAQ4plex); M6(Oxidation)	HCD	1.5731845	0	1	1	1.35508	1.3942	1.7472	1.7975852	14.68845368	12533.754	12182.361	16984.178	21898.834	2.010610269	17.87508943	3	High	6501	Unique	Used	53898	1	59.351	1	5.1629	1.0443	1	36	29/84	19.50337	6501	2628.29	MS2	876.769	149	Glioma_grade2_2.raw
15718687			kPLPDHVSIVEPk	Unambiguous	N-Term(iTRAQ4plex); K1(iTRAQ4plex); K13(iTRAQ4plex)	HCD	2.5999312	0	1	1	2.54071	2.4944	2.7074	2.6580749	3.81855602	10960.961	11164.407	27848.607	29675.828	1.300390088	4.492144459	4	High	2044	Unique	Used	85447	1	29.901	1	6.0774	-6.6667	1	42	26/72	28.99649	2044	1891.13	MS2	473.537	90	Glioma_grade2_1rep.raw
15718687			kPLPDHVSIVEPk	Unambiguous	N-Term(iTRAQ4plex); K1(iTRAQ4plex); K13(iTRAQ4plex)	HCD	2.4024124	0	1	1	2.39685	2.3996	2.4052	2.4079797	0.21224455	5234.2515	5228.31	12545.723	12589.664	0.080310638	0.247228969	4	High	2167	Unique	Used	85533	1	31.327	1	5.5844	-5.4402	1	69	26/72	46.39035	2167	1891.13	MS2	473.537	89	Glioma_grade2_1rep.raw
15718687			kPLPDHVSIVEPk	Unambiguous	N-Term(iTRAQ4plex); K1(iTRAQ4plex); K13(iTRAQ4plex)	HCD	2.473217	0	1	1	2.42569	2.592	2.3621	2.5240026	4.12387777	4774.043	4467.7217	11580.35	11276.541	4.687456898	1.879742998	4	High	2272	Unique	Used	95019	1	32.802	1	5.9074	-1.0509	1	89	25/72	23.92309	2272	1891.14	MS2	473.539	80	Glioma_grade2_8rep.raw
15718687			fVDGLmIHSGDPVNYYVDTAVR	Unambiguous	N-Term(iTRAQ4plex); M6(Oxidation)	HCD	1.5376016	0	1	1	1.42532	1.5497	1.5265	1.6596813	6.244210711	3464.5447	3186.5134	4938.0806	5288.597	5.91177568	4.847175848	3	High	6324	Unique	Used	61728	1	59.655	1	5.5292	0.2779	1	100	27/84	16.50351	6324	2628.29	MS2	876.768	139	Glioma_grade2_3.raw
15812198	NP_036300.2	FBXO2	F-box only protein 2 [Homo sapiens]	4	7	0.502478302	15.61541882	58.181914	26.69	2.17E-03	296	33.307	4.3735	Ubiquitin proteasome system protein	Protein metabolism	Ubiquitin-specific protease activity	Cytoplasm	-																										
15812198			dHWQQFYFLSk	Unambiguous	N-Term(iTRAQ4plex); K11(iTRAQ4plex)	HCD	0.49710825	0	1	1	0.40953	0.5368	0.4668	0.6119092	0.172982047	139615.14	106510.25	57176.363	65174.598	19.0217614	9.244890534	3	High	5014	Unique	Used	82082	1	52.218	1	5.1273	0.3551	1	9	18/40	17.79062	5014	1786.92	MS2	596.31	158	Glioma_grade2_6.raw
15812198			dHWQQFYFLSk	Unambiguous	N-Term(iTRAQ4plex); K11(iTRAQ4plex)	HCD	0.5877564	0	1	1	0.56821	0.5788	0.5965	0.60766673	0.030038631	135468.73	132986.03	76974.81	80811.195	1.307880706	3.438497411	2	High	5008	Unique	Used	82077	1	52.16	1	5.4625	-1.6185	1	92	15/20	37.63636	5008	1786.91	MS2	893.96	174	Glioma_grade2_6.raw
15812198			eLVDGAPLWLLk	Unambiguous	N-Term(iTRAQ4plex); K12(iTRAQ4plex)	HCD	0.52910113	0	1	1	0.49619	0.5442	0.5153	0.5652042	0.057626846	111664.88	101807	55406.477	57541.742	6.530671671	2.673544345	2	High	7666	Unique	Used	54968	1	68.453	1	5.2573	-0.228	1	60	21/22	37.24862	7666	1641.98	MS2	821.494	131	Glioma_grade2_2.raw
15812198			vEELPGDSGVEFTHDESVk	Unambiguous	N-Term(iTRAQ4plex); K19(iTRAQ4plex)	HCD	0.39518493	0	1	1	0.35967	0.3459	0.4464	0.42933676	0.126310684	102496.17	106574.82	36865.22	45756.484	2.758910811	15.21893828	3	High	3968	Unique	Used	67212	1	40.674	1	6.0757	0.8917	1	7	31/72	5.693866	3968	2362.17	MS2	788.061	90	Glioma_grade2_4.raw
15812198			vEELPGDSGVEFTHDESVk	Unambiguous	N-Term(iTRAQ4plex); K19(iTRAQ4plex)	HCD	0.41028327	0	1	1	0.37857	0.369	0.4527	0.4411975	0.103987304	36309.4	37252.254	13745.512	16435.602	1.812624977	12.60510716	3	High	3744	Unique	Used	59331	1	39.559	1	5.6097	0.6591	1	50	30/72	32.43181	3744	2362.17	MS2	788.061	96	Glioma_grade2_3.raw
15812198			nPcGEEDLEGWcDVEHGGDGWR	Unambiguous	N-Term(iTRAQ4plex); C3(Methylthio); C12(Methylthio)	HCD	0.5024783	0	1	1	0.41357	0.4327	0.5692	0.59550273	0.184731135	20359.72	19459.416	8420.116	11588.135	3.197513193	22.39203934	3	High	7232	Unique	Used	62567	1	66.864	1	7.4724	2.3891	1	54	34/84	8.788371	7232	2696.05	MS2	899.353	1	Glioma_grade2_3.raw
15812198			eLVDGAPLWLLk	Unambiguous	N-Term(iTRAQ4plex); K12(iTRAQ4plex)	HCD	0.5971001	0	1	1	0.63721	0.6702	0.5276	0.5549167	0.112554963	9356.62	8895.673	5962.088	4936.357	3.571482766	13.31018041	3	High	7669	Unique	Used	54971	1	68.473	1	3.9735	2.7375	1	100	20/44	42.68946	7669	1641.99	MS2	548	104	Glioma_grade2_2.raw
16418379	NP_443106.1	STX1B	syntaxin-1B [Homo sapiens]	10	32	0.38897115	20.01935747	246.60852	40.28	1.73E-04	288	33.224	5.3765	Membrane transport protein	Transport	Auxiliary transport protein activity	Integral to membrane	-																										
16418379			lAIFTDDIk	Unambiguous	N-Term(iTRAQ4plex); K9(iTRAQ4plex)	HCD	0.30109763	0	1	1	0.26418	0.2807	0.3203	0.34032562	0.116293768	1.27E+07	1.19E+07	3345742.5	4056265.5	4.290713529	13.57511722	2	High	5626	Unique	Used	53074	1	52.939	1	3.8129	-0.8107	1	1	16/16	7.438268	5626	1323.78	MS2	662.391	274	Glioma_grade2_2.raw
16418379			lAIFTDDIk	Unambiguous	N-Term(iTRAQ4plex); K9(iTRAQ4plex)	HCD	0.35215142	0	1	1	0.35954	0.338	0.3672	0.3452068	0.037691787	6776753	7208710.5	2436505	2488496	4.367965029	1.492921064	2	High	5372	Unique	Used	60844	1	52.22	1	4.0129	0.9413	1	1	16/16	17.39243	5372	1323.78	MS2	662.392	288	Glioma_grade2_3.raw
16418379			lAIFTDDIk	Unambiguous	N-Term(iTRAQ4plex); K9(iTRAQ4plex)	HCD	0.29826096	0	1	1	0.2744	0.3157	0.2831	0.32571575	0.082836291	2940510.5	2555691	806873.5	832428.9	9.901692214	2.204644687	2	High	5489	Unique	Used	60953	1	53.126	1	4.0528	2.4168	1	4	16/16	9.828991	5489	1323.78	MS2	662.393	269	Glioma_grade2_3.raw
16418379			tkQELEDLTADIk	Unambiguous	N-Term(iTRAQ4plex); K2(iTRAQ4plex); K13(iTRAQ4plex)	HCD	0.2590423	1	1	1	0.26391	0.2537	0.2646	0.2543638	0.022894868	1487295.1	1547321.8	392511.47	393582.66	2.797406593	0.192711199	2	High	4243	Unique	Used	81424	1	45.393	1	5.3279	-1.6916	1	6	18/24	13.26888	4243	1936.09	MS2	968.55	148	Glioma_grade2_6.raw
16418379			iEYNVEHSVDYVER	Unambiguous	N-Term(iTRAQ4plex)	HCD	0.47219592	0	1	1	0.45594	0.4467	0.4983	0.48811626	0.052559737	424151.3	432960.78	193389.64	211335.2	1.453542236	6.270644109	2	High	4162	Unique	Used	51695	1	42.286	1	5.7688	-1.6253	1	8	22/26	0	4162	1895.92	MS2	948.465	136	Glioma_grade2_2.raw
16418379			dSDDEEEVVHVDR	Unambiguous	N-Term(iTRAQ4plex)	HCD	0.4549437	0	1	1	0.46003	0.4655	0.4445	0.44979987	0.020952226	353941.7	349779.7	162822.75	157330.86	0.836404413	2.42593089	2	High	1317	Unique	Used	49011	1	22.103	1	4.489	2.1881	1	10	21/24	27.94313	1317	1687.76	MS2	844.382	62	Glioma_grade2_2.raw
16418379			dHFMDEFFEQVEEIR	Unambiguous	N-Term(iTRAQ4plex)	HCD	0.26466474	0	1	1	0.256	0.2638	0.2655	0.27359432	0.02725836	271207.34	263173.6	69428.99	72002.8	2.126090812	2.573620124	2	High	7524	Unique	Used	77613	1	72.7	1	5.8304	-4.424	1	13	22/28	10.28522	7524	2114.95	MS2	1057.98	49	Glioma_grade2_5.raw
16418379			kFVEVMTEYNATQSk	Unambiguous	N-Term(iTRAQ4plex); K1(iTRAQ4plex); K15(iTRAQ4plex)	HCD	0.36749095	1	1	1	0.31767	0.3982	0.343	0.4299486	0.137349375	260334.66	207669.95	82700.055	89287.41	15.91419092	5.416631253	2	High	3388	Unique	Used	80690	1	37.874	1	5.291	9.9975	1	38	21/28	9.280796	3388	2207.2	MS2	1104.1	143	Glioma_grade2_6.raw
16418379			dSDDEEEVVHVDR	Unambiguous	N-Term(iTRAQ4plex)	HCD	0.31709084	0	1	1	0.31905	0.2799	0.3595	0.3153759	0.102189695	236209.98	269231.53	75361.74	84909.13	9.239352712	8.424518082	3	High	1310	Unique	Used	49004	1	22.063	1	4.6173	0.9868	1	2	29/48	10.55624	1310	1687.75	MS2	563.256	44	Glioma_grade2_2.raw
16418379			kFVEVMTEYNATQSk	Unambiguous	N-Term(iTRAQ4plex); K1(iTRAQ4plex); K15(iTRAQ4plex)	HCD	0.57423013	1	1	1	0.56649	0.631	0.5233	0.5828547	0.077285375	169152.31	151858.88	95822.64	88511.664	7.618613932	5.608983889	3	High	3310	Unique	Used	80619	1	37.267	1	8.0554	-5.8538	1	7	25/56	19.14814	3310	2207.16	MS2	736.392	170	Glioma_grade2_6.raw
16418379			dHFmDEFFEQVEEIR	Unambiguous	N-Term(iTRAQ4plex); M4(Oxidation)	HCD	0.34011617	0	1	1	0.33395	0.3287	0.3517	0.3461887	0.031224913	127437.29	129460.13	42557.336	44817.633	1.113568117	3.658419235	2	High	6202	Unique	Used	76494	1	60.697	1	4.365	-0.5151	1	20	18/28	0	6202	2130.95	MS2	1065.98	47	Glioma_grade2_5.raw
16418379			dHFMDEFFEQVEEIR	Unambiguous	N-Term(iTRAQ4plex)	HCD	0.41619816	0	1	1	0.37019	0.5519	0.3252	0.48478612	0.239932218	126324.17	84736.664	46763.977	41079.16	27.86571715	9.152160972	3	High	7523	Unique	Used	77612	1	72.697	1	6.1233	-3.074	1	2	26/56	13.24838	7523	2114.95	MS2	705.656	54	Glioma_grade2_5.raw
16418379			kFVEVMTEYNATQSk	Unambiguous	N-Term(iTRAQ4plex); K1(iTRAQ4plex); K15(iTRAQ4plex)	HCD	0.45205826	1	1	1	0.47185	0.4059	0.5057	0.43502998	0.095500087	112945.22	131282.3	53293.33	57111.734	10.6181921	4.89111507	2	High	3424	Unique	Used	91028	1	39.338	1	6.8541	-8.2535	1	76	22/28	0	3424	2207.16	MS2	1104.08	175	Glioma_grade2_7rep.raw
16418379			dHFMDEFFEQVEEIR	Unambiguous	N-Term(iTRAQ4plex)	HCD	0.3792403	0	1	1	0.36173	0.3965	0.3635	0.39843327	0.053064089	111042	101297.266	40167.402	40360.2	6.490149111	0.338588931	2	High	7459	Unique	Used	77564	1	72.01	1	6.1254	-3.6159	1	37	21/28	32.51812	7459	2114.95	MS2	1057.98	66	Glioma_grade2_5.raw
16418379			tkQELEDLTADIk	Unambiguous	N-Term(iTRAQ4plex); K2(iTRAQ4plex); K13(iTRAQ4plex)	HCD	0.38820204	1	1	1	0.34874	0.3893	0.3872	0.4322534	0.087647879	105854.305	94815.82	36916.094	40984.46	7.779322007	7.385747698	3	High	4329	Unique	Used	81496	1	46.18	1	5.6848	-3.464	1	18	22/48	30.82775	4329	1936.09	MS2	646.035	156	Glioma_grade2_6.raw
16418379			iEYNVEHSVDYVER	Unambiguous	N-Term(iTRAQ4plex)	HCD	0.47362566	0	1	1	0.47575	0.4674	0.48	0.4715407	0.011488012	102645.04	104487.664	48833.176	49270.184	1.258064903	0.629970921	2	High	4020	Unique	Used	59587	1	41.695	1	5.8958	0.6926	1	55	22/26	0	4020	1895.93	MS2	948.467	136	Glioma_grade2_3.raw
16418379			dHFMDEFFEQVEEIR	Unambiguous	N-Term(iTRAQ4plex)	HCD	0.3125127	0	1	1	0.30055	0.3367	0.2909	0.325916	0.06812336	91389.15	81580.56	27466.82	26588.412	8.019578113	2.298124457	2	High	7967	Unique	Used	70829	1	73.533	1	6.1099	-0.0374	1	48	23/28	32.72189	7967	2114.96	MS2	1057.98	61	Glioma_grade2_4.raw
16418379			dHFmDEFFEQVEEIR	Unambiguous	N-Term(iTRAQ4plex); M4(Oxidation)	HCD	0.38974023	0	1	1	0.36048	0.4277	0.3577	0.42445377	0.098610745	84693.65	71378.76	30530.71	30296.982	12.06497549	0.543405966	2	High	6671	Unique	Used	69697	1	62.083	1	4.4415	0.4014	1	32	18/28	4.624462	6671	2130.96	MS2	1065.98	48	Glioma_grade2_4.raw
16418379			fVEVMTEYNATQSk	Unambiguous	N-Term(iTRAQ4plex); K14(iTRAQ4plex)	HCD	0.45081392	0	1	1	0.4656	0.4351	0.4677	0.43699616	0.039209248	72289.19	77360.06	33657.914	33806.05	4.792067536	0.310530138	2	High	5047	Unique	Used	52528	1	48.76	1	5.5472	-3.857	1	58	22/26	7.937941	5047	1934.97	MS2	967.989	136	Glioma_grade2_2.raw
16418379			qHSAILAAPNPDEk	Unambiguous	N-Term(iTRAQ4plex); K14(iTRAQ4plex)	HCD	0.47248483	0	1	1	0.44231	0.4735	0.4715	0.50478476	0.05395768	70061.664	65448.37	30989.096	33037.34	4.814538636	4.52415381	2	High	1253	Unique	Used	72029	1	21.272	1	4.6426	-0.3782	1	65	19/26	36.79122	1253	1778.96	MS2	889.985	222	Glioma_grade2_5.raw
16418379			kQHSAILAAPNPDEk	Unambiguous	N-Term(iTRAQ4plex); K1(iTRAQ4plex); K15(iTRAQ4plex)	HCD	0.5450311	1	1	1	0.49956	0.5363	0.5532	0.59385055	0.071691871	55830.785	52006.01	27890.605	30883.799	5.015958308	7.202141173	4	High	1226	Unique	Used	84828	1	21.287	1	5.1187	2.2718	1	14	17/84	41.2622	1226	2051.17	MS2	513.547	132	Glioma_grade2_1rep.raw
16418379			dHFMDEFFEQVEEIR	Unambiguous	N-Term(iTRAQ4plex)	HCD	0.34598592	0	1	1	0.30213	0.327	0.3635	0.39345512	0.115921507	54020.84	49907.023	16321.402	19636.174	5.597936517	13.03701762	2	High	7899	Unique	Used	70777	1	72.814	1	6.1762	-0.2683	1	100	21/28	34.41032	7899	2114.96	MS2	1057.98	66	Glioma_grade2_4.raw
16418379			dHFMDEFFEQVEEIR	Unambiguous	N-Term(iTRAQ4plex)	HCD	0.44389257	0	1	1	0.44804	0.4612	0.4271	0.43961957	0.03229203	53920.812	52384.457	24158.885	23029.232	2.043863016	3.385535798	3	High	7955	Unique	Used	70819	1	73.423	1	5.7528	1.2548	1	4	26/56	22.51763	7955	2114.96	MS2	705.659	67	Glioma_grade2_4.raw
16418379			dHFmDEFFEQVEEIR	Unambiguous	N-Term(iTRAQ4plex); M4(Oxidation)	HCD	0.37562	0	1	1	0.33442	0.3629	0.3874	0.42033097	0.097001172	50231.375	46292.375	16798.13	19458.12	5.771208871	10.37557368	2	High	6589	Unique	Used	69624	1	61.383	1	5.0109	-0.9734	1	81	18/28	4.584469	6589	2130.95	MS2	1065.98	46	Glioma_grade2_4.raw
16418379			iEYNVEHSVDYVER	Unambiguous	N-Term(iTRAQ4plex)	HCD	0.488495	0	1	1	0.43456	0.5522	0.4384	0.55703163	0.137745578	47485.83	37370.137	20635.365	20816.35	16.85886183	0.617468883	2	High	3700	Unique	Used	80966	1	40.455	1	4.9317	3.0105	1	100	21/26	11.54044	3700	1895.93	MS2	948.469	148	Glioma_grade2_6.raw
16418379			dHFmDEFFEQVEEIR	Unambiguous	N-Term(iTRAQ4plex); M4(Oxidation)	HCD	0.34628782	0	1	1	0.37073	0.3747	0.3182	0.32158446	0.088324667	34954.77	34582.95	12958.726	11121.34	0.756183675	10.79090149	2	High	6127	Unique	Used	76430	1	60.03	1	4.5395	0.6306	1	75	19/28	19.08262	6127	2130.96	MS2	1065.98	49	Glioma_grade2_5.raw
16418379			dHFmDEFFEQVEEIR	Unambiguous	N-Term(iTRAQ4plex); M4(Oxidation)	HCD	0.40612668	0	1	1	0.43506	0.412	0.3999	0.37872294	0.057901594	34130.61	36039.402	14848.966	13648.948	3.846998821	5.955108611	3	High	6670	Unique	Used	69696	1	62.08	1	5.5143	0.9393	1	7	27/56	7.267646	6670	2130.96	MS2	710.991	48	Glioma_grade2_4.raw
16418379			kQHSAILAAPNPDEk	Unambiguous	N-Term(iTRAQ4plex); K1(iTRAQ4plex); K15(iTRAQ4plex)	HCD	0.37485352	1	1	1	0.39191	0.4109	0.3404	0.35696906	0.085650642	22428.877	21390.76	8790.105	7635.8394	3.350368103	9.937803431	3	High	1301	Unique	Used	94317	1	21.771	1	6.3271	-2.9588	1	43	24/56	20.46577	1301	2051.16	MS2	684.39	107	Glioma_grade2_8rep.raw
16418379			tkQELEDLTADIk	Unambiguous	N-Term(iTRAQ4plex); K2(iTRAQ4plex); K13(iTRAQ4plex)	HCD	0.3147188	1	1	1	0.33091	0.3595	0.2735	0.29713532	0.119652389	21800.361	20068.396	7213.8564	5963.0293	5.850110125	13.42454271	4	High	4264	Unique	Used	81442	1	45.573	1	4.4651	-7.6953	1	21	18/72	50.69742	4264	1936.08	MS2	484.776	174	Glioma_grade2_6.raw
16418379			kQHSAILAAPNPDEk	Unambiguous	N-Term(iTRAQ4plex); K1(iTRAQ4plex); K15(iTRAQ4plex)	HCD	0.43402198	1	1	1	0.45177	0.3961	0.4773	0.41846162	0.082276864	17663.96	20148.549	7980.0654	8431.394	9.292531899	3.88920334	4	High	1300	Unique	Used	94316	1	21.767	1	5.0836	-6.2981	1	26	20/84	37.23754	1300	2051.15	MS2	513.543	147	Glioma_grade2_8rep.raw
16418379			dHFMDEFFEQVEEIR	Unambiguous	N-Term(iTRAQ4plex)	HCD	0.52455443	0	1	1	0.5304	0.7694	0.3558	0.5160729	0.314043775	8229.446	5672.9995	4364.908	2927.6814	26.00521838	27.87138064	3	High	7887	Unique	Used	70767	1	72.696	1	5.9741	0.3025	1	65	23/56	40.1201	7887	2114.96	MS2	705.659	70	Glioma_grade2_4.raw
16418379			eLHDMFVDMAmLVESQGEMIDR	Unambiguous	N-Term(iTRAQ4plex); M11(Oxidation)	HCD	0.42008165	0	1	1	0.33013	0.3726	0.4622	0.5216011	0.205031696	6394.0474	5665.6543	2110.848	2955.2114	8.541698844	23.570789	2	High	7962	Unique	Used	77977	1	76.753	1	3.4641	-2.4527	1	63	17/42	19.73779	7962	2756.25	MS2	1378.63	63	Glioma_grade2_5.raw
16507237	NP_005338.1	HSPA5	78 kDa glucose-regulated protein precursor [Homo sapiens]	18	39	2.363461256	22.07606362	381.88588	37.77	1.23E-04	654	72.288	5.1606	Chaperone	Protein metabolism	Chaperone activity	Endoplasmic reticulum	Nucleolus, Cytoplasm, Mitochondrion, Cell surface, Endoplasmic reticulum lumen, Plasma membrane																										
16507237			vMEHFIk	Unambiguous	N-Term(iTRAQ4plex); K7(iTRAQ4plex)	HCD	2.5106826	0	1	1	2.42074	2.5789	2.4467	2.6064968	3.703876343	1034913.7	971450.25	2505261.2	2532082	4.473309626	0.752983023	3	High	1557	Unique	Used	79035	1	23.341	1	3.4864	-7.1563	1	1	15/24	8.599871	1557	1191.67	MS2	397.895	283	Glioma_grade2_6.raw

16507237			kSDIDEIVLVGGSTR	Unambiguous	N-Term(iTRAQ4plex); K1(iTRAQ4plex)	HCD	2.54832	1	1	1	2.45616	2.7315	2.3836	2.6508076	6.368535734	276689.2	248800.62	679593.6	659522.6	7.505456161	2.119657757	2	High	3422	Unique	Used	74031	1	37.625	1	4.7161	2.92	1	6	23/28	4.577995	3422	1877.06	MS2	939.036	111	Glioma_grade2_5.raw
16507237			tWNDPSVQQDIk	Unambiguous	N-Term(iTRAQ4plex); K12(iTRAQ4plex)	HCD	2.394086	0	1	1	2.30226	2.3189	2.4687	2.4865806	4.05018589	243027.53	241281.23	559511.56	599965.2	0.509931132	4.934129627	2	High	2707	Unique	Used	50328	1	31.683	1	4.6966	1.9011	1	8	20/22	3.967063	2707	1718.9	MS2	859.953	202	Glioma_grade2_2.raw
16507237			fEELNMDLFR	Unambiguous	N-Term(iTRAQ4plex)	HCD	2.0784242	0	1	1	1.88525	2.1083	2.0517	2.2944562	8.093606799	171894.9	153707.36	324064.84	352674.8	7.899535382	5.978753284	2	High	5012	Unique	Used	87414	1	65.57	1	4.0385	2.1079	1	22	16/18	31.34655	5012	1457.72	MS2	729.366	149	Glioma_grade2_1rep.raw
16507237			iEWLESHQDADIEDFk	Unambiguous	N-Term(iTRAQ4plex); K16(iTRAQ4plex)	HCD	2.7423127	0	1	1	2.61056	2.7629	2.7228	2.8817554	4.078429616	147160.61	139045.66	384171.5	400695.56	4.009790683	2.977389541	2	High	5630	Unique	Used	75987	1	55.947	1	6.7217	-1.2172	1	9	23/30	0.883828	5630	2263.11	MS2	1132.06	134	Glioma_grade2_5.raw
16507237			tkPYIQVDIGGGQTk	Unambiguous	N-Term(iTRAQ4plex); K2(iTRAQ4plex); K15(iTRAQ4plex)	HCD	2.5134969	0	1	1	2.82926	2.5674	2.4541	2.2269583	9.938919916	136532.36	150457.1	386285.44	335061.7	6.861769823	10.04250295	3	High	2135	Unique	Used	79571	1	27.658	1	5.5548	4.1071	1	1	26/56	5.365838	2135	2037.18	MS2	679.731	92	Glioma_grade2_6.raw
16507237			tkPYIQVDIGGGQTk	Unambiguous	N-Term(iTRAQ4plex); K2(iTRAQ4plex); K15(iTRAQ4plex)	HCD	2.936255	0	1	1	2.64464	2.9595	2.9155	3.2625878	8.59214531	119154.086	106477.68	315119.6	347392.78	7.945310895	6.8891043	2	High	2121	Unique	Used	79558	1	27.55	1	6.3209	2.7355	1	28	19/28	0	2121	2037.18	MS2	1019.09	110	Glioma_grade2_6.raw
16507237			nELESYAYSLk	Unambiguous	N-Term(iTRAQ4plex); K11(iTRAQ4plex)	HCD	1.5425131	0	1	1	1.57715	1.4529	1.6398	1.5106071	5.241257882	116701.32	126679.3	184055.16	191362.64	5.797912192	2.752756348	2	High	5429	Unique	Used	52887	1	51.553	1	5.2631	-2.5334	1	35	18/20	40.63874	5429	1604.84	MS2	802.922	196	Glioma_grade2_2.raw
16507237			tWNDPSVQQDIk	Unambiguous	N-Term(iTRAQ4plex); K12(iTRAQ4plex)	HCD	2.53647	0	1	1	2.36151	2.4445	2.6253	2.7175763	6.425848437	97160.24	93860.336	229445.33	255072.62	2.443071364	7.480106998	2	High	2620	Unique	Used	50247	1	31.048	1	4.9998	-0.3714	1	57	20/22	27.03344	2620	1718.89	MS2	859.951	215	Glioma_grade2_2.raw
16507237			tFAPEEISAMVLTk	Unambiguous	N-Term(iTRAQ4plex); K14(iTRAQ4plex)	HCD	2.3954275	0	1	1	2.19682	2.4245	2.3691	2.6146204	7.168628923	92437.26	83756.95	203067.78	218992.64	6.967205181	5.336002128	2	High	6977	Unique	Used	54342	1	62.921	1	6.5269	-2.8853	1	62	22/26	44.43	6977	1825	MS2	913.002	231	Glioma_grade2_2.raw
16507237			eLEEIVQPIISk	Unambiguous	N-Term(iTRAQ4plex); K12(iTRAQ4plex)	HCD	3.0545905	0	1	1	3.40326	2.9329	3.1958	2.7541168	9.313017278	91441.266	106107.29	311198.1	292231.88	10.49913523	4.444970658	2	High	6341	Unique	Used	53749	1	58.157	1	5.0849	3.3472	1	14	17/22	0	6341	1686	MS2	843.503	144	Glioma_grade2_2.raw
16507237			iEWLESHQDADIEDFk	Unambiguous	N-Term(iTRAQ4plex); K16(iTRAQ4plex)	HCD	2.949547	0	1	1	3.0287	3.029	2.8701	2.8703818	3.10558805	60522.504	60515.62	183304.7	173702.94	0.008043289	3.803543032	3	High	5372	Unique	Used	82387	1	55.374	1	6.9305	-0.0095	1	9	27/60	12.36464	5372	2263.11	MS2	755.042	117	Glioma_grade2_6.raw
16507237			eLEEIVQPIISk	Unambiguous	N-Term(iTRAQ4plex); K12(iTRAQ4plex)	HCD	1.8696201	0	1	1	1.71646	1.9794	1.7744	2.0462377	8.436382731	57945.523	50248.727	99461.3	102820.84	10.06052844	2.348752604	2	High	4484	Unique	Used	87018	1	59.908	1	4.8379	1.7544	1	63	16/22	22.53064	4484	1686	MS2	843.502	136	Glioma_grade2_1rep.raw
16507237			kSDIDEIVLVGGSTR	Unambiguous	N-Term(iTRAQ4plex); K1(iTRAQ4plex)	HCD	1.9832753	1	1	1	1.68666	2.3795	1.7024	2.401732	19.68254388	54117.59	38360.242	91277.984	92131.02	24.09686167	0.657751285	3	High	3419	Unique	Used	74028	1	37.611	1	4.7426	2.23	1	1	26/56	16.88588	3419	1877.06	MS2	626.359	154	Glioma_grade2_5.raw
16507237			vTHAVVTVPAYFNDAQR	Unambiguous	N-Term(iTRAQ4plex)	HCD	1.8989259	0	1	1	1.46115	2.1567	1.7243	2.5450902	24.23119159	53841.508	36477.863	78670.336	92839.45	27.18785791	11.68338767	3	High	4060	Unique	Used	51599	1	41.543	1	4.1767	0.9174	1	8	19/64	24.2095	4060	2032.08	MS2	678.03	151	Glioma_grade2_2.raw
16507237			dNHLLGTFDLTGIPPAPR	Unambiguous	N-Term(iTRAQ4plex)	HCD	1.8384709	0	1	1	1.54818	1.8162	1.8575	2.1790092	13.97408251	53584.59	45678.113	82958.57	99533.02	11.26449982	12.84432449	3	High	6058	Unique	Used	69140	1	57.082	1	4.6474	0.7112	1	8	23/68	26.3671	6058	2078.12	MS2	693.377	155	Glioma_grade2_4.raw
16507237			sQIFSTASDNQPTVTIk	Unambiguous	N-Term(iTRAQ4plex); K17(iTRAQ4plex)	HCD	2.8192923	0	1	1	2.99056	2.5836	3.0921	2.671334	8.64969776	46074.895	53332.96	137789.66	142470.16	10.32559646	2.361817894	2	High	4041	Unique	Used	51581	1	41.4	1	6.4128	0.6939	1	30	25/32	0	4041	2125.14	MS2	1063.07	204	Glioma_grade2_2.raw
16507237			kELEEIVQPIISk	Unambiguous	N-Term(iTRAQ4plex); K1(iTRAQ4plex); K13(iTRAQ4plex)	HCD	2.0239499	1	1	1	1.98706	1.987	2.0609	2.0608354	2.10762847	42286.133	42288.46	84025.17	87149.555	0.003891092	2.58130845	2	High	4702	Unique	Used	81813	1	49.516	1	4.8695	-2.485	1	99	15/24	30.90164	4702	1958.18	MS2	979.596	47	Glioma_grade2_6.raw
16507237			vTHAVVTVPAYFNDAQR	Unambiguous	N-Term(iTRAQ4plex)	HCD	2.873366	0	1	1	2.91784	2.9985	2.7516	2.8276663	3.73294336	41923.836	40796.703	122326.94	115359.46	1.926978226	4.145590455	2	High	4008	Unique	Used	59576	1	41.603	1	5.0817	-0.3075	1	25	22/32	1.729818	4008	2032.07	MS2	1016.54	194	Glioma_grade2_3.raw
16507237			mkETAEAYLGk	Unambiguous	N-Term(iTRAQ4plex); K2(iTRAQ4plex); K11(iTRAQ4plex)	HCD	1.9952347	1	1	1	1.92081	1.9258	2.0645	2.0698574	4.165983408	41677.047	41569.098	80053.5	86042.1	0.183387399	5.098966703	2	High	2820	Unique	Used	80177	1	33.322	1	4.7474	1.9804	1	82	18/20	11.59868	2820	1672.93	MS2	836.971	176	Glioma_grade2_6.raw
16507237			tFAPEEISAmVLTk	Unambiguous	N-Term(iTRAQ4plex); M10(Oxidation); K14(iTRAQ4plex)	HCD	2.5130546	0	1	1	2.25585	2.7257	2.3371	2.8238237	11.08189855	40296.7	33350.73	90903.43	94176.58	13.33798746	2.501044344	2	High	5934	Unique	Used	53363	1	55.236	1	3.5832	-1.8221	1	44	16/26	27.99671	5934	1840.99	MS2	921	241	Glioma_grade2_2.raw
16507237			dNHLLGTFDLTGIPPAPR	Unambiguous	N-Term(iTRAQ4plex)	HCD	1.5195037	0	1	1	1.43953	2.0209	1.1624	1.6317767	23.06230547	36296.117	25854.924	52249.273	42189.46	23.75837397	15.06450111	3	High	5617	Unique	Used	75975	1	55.848	1	6.0381	-0.5223	1	2	29/68	5.0308	5617	2078.11	MS2	693.376	133	Glioma_grade2_5.raw
16507237			iEWLESHQDADIEDFk	Unambiguous	N-Term(iTRAQ4plex); K16(iTRAQ4plex)	HCD	2.7320204	0	1	1	2.81243	2.6927	2.7731	2.6550364	2.639604659	34697.07	36239.703	97582.984	96217.734	3.075432414	0.996257953	3	High	5606	Unique	Used	75965	1	55.757	1	5.8073	-0.3332	1	14	28/60	15.07081	5606	2263.11	MS2	755.042	106	Glioma_grade2_5.raw
16507237			vTHAVVTVPAYFNDAQR	Unambiguous	N-Term(iTRAQ4plex)	HCD	2.615733	0	1	1	2.58467	2.7656	2.4757	2.6489697	4.629247421	27487.654	25689.762	71046.516	68051.4	4.781359158	3.045145312	2	High	4315	Unique	Used	67535	1	43.346	1	5.1153	0.7738	1	45	24/32	4.307584	4315	2032.07	MS2	1016.54	198	Glioma_grade2_4.raw
16507237			dNHLLGTFDLTGIPPAPR	Unambiguous	N-Term(iTRAQ4plex)	HCD	2.832609	0	1	1	2.59704	2.8289	2.836	3.0892138	7.084589934	24812.158	22778.443	64438.08	70367.484	6.043435625	6.220398702	2	High	6111	Unique	Used	69189	1	57.497	1	5.6087	0.3946	1	26	22/34	29.39849	6111	2078.12	MS2	1039.56	172	Glioma_grade2_4.raw
16507237			iEWLESHQDADIEDFk	Unambiguous	N-Term(iTRAQ4plex); K16(iTRAQ4plex)	HCD	1.6972454	0	1	1	1.65766	1.582	1.8181	1.7350268	5.974330211	23797.627	24936.453	39448.277	43265.414	3.304757522	6.526424888	3	High	5735	Unique	Used	76080	1	56.832	1	5.9812	-0.6568	1	18	28/60	32.14558	5735	2263.11	MS2	755.042	121	Glioma_grade2_5.raw
16507237			kSDIDEIVLVGGSTR	Unambiguous	N-Term(iTRAQ4plex); K1(iTRAQ4plex)	HCD	1.7330582	1	1	1	1.59082	1.6904	1.7732	1.8842057	7.177050819	18804.625	17696.748	29914.691	33344.312	4.292371903	7.667235179	2	High	3556	Unique	Used	74154	1	38.653	1	4.6563	4.6108	1	100	23/28	11.94637	3556	1877.07	MS2	939.037	132	Glioma_grade2_5.raw
16507237			vTHAVVTVPAYFNDAQR	Unambiguous	N-Term(iTRAQ4plex)	HCD	2.3634613	0	1	1	2.14869	2.3038	2.4192	2.593735	7.935639997	18692.994	17434.785	40165.395	45221.215	4.925230062	8.373689051	2	High	3916	Unique	Used	59491	1	40.878	1	4.9805	-0.7881	1	67	25/32	0	3916	2032.07	MS2	1016.54	213	Glioma_grade2_3.raw
16507237			kELEEIVQPIISk	Unambiguous	N-Term(iTRAQ4plex); K1(iTRAQ4plex); K13(iTRAQ4plex)	HCD	1.6637539	1	1	1	1.51839	1.8658	1.4994	1.8423755	11.87534165	18632.756	15163.631	28291.77	27937.102	14.51659204	0.892026245	3	High	4676	Unique	Used	81789	1	49.319	1	5.1806	-3.1465	1	35	18/48	25.97574	4676	1958.18	MS2	653.399	37	Glioma_grade2_6.raw
16507237			dNHLLGTFDLTGIPPAPR	Unambiguous	N-Term(iTRAQ4plex)	HCD	2.0324707	0	1	1	1.83305	2.0925	1.9799	2.2601123	8.837237972	16330.471	14305.812	29934.604	32332.74	9.346108394	5.446637415	2	High	5669	Unique	Used	76022	1	56.269	1	5.2332	-1.955	1	50	22/34	4.801878	5669	2078.11	MS2	1039.56	163	Glioma_grade2_5.raw
16507237			vTHAVVTVPAYFNDAQR	Unambiguous	N-Term(iTRAQ4plex)	HCD	2.5548127	0	1	1	2.20308	2.635	2.4878	2.9754946	12.48040163	14148.389	11829.426	31170.059	35198.39	12.62426777	8.583778015	2	High	4053	Unique	Used	51593	1	41.477	1	5.0446	-0.9082	1	100	22/32	0	4053	2032.07	MS2	1016.54	224	Glioma_grade2_2.raw
16507237			vTHAVVTVPAYFNDAQR	Unambiguous	N-Term(iTRAQ4plex)	HCD	2.5790014	0	1	1	2.48397	2.4225	2.7395	2.6716797	5.830628856	13832.564	14183.509	34359.695	37893.793	1.771523006	6.917270655	2	High	4227	Unique	Used	67454	1	42.642	1	5.1401	-0.5478	1	81	24/32	0	4227	2032.07	MS2	1016.54	195	Glioma_grade2_4.raw
16507237			dNHLLGTFDLTGIPPAPR	Unambiguous	N-Term(iTRAQ4plex)	HCD	1.9306235	0	1	1	1.93698	1.8889	1.9734	1.924424	1.807030701	13365.853	13706.179	25889.4	26376.5	1.777826078	1.317997827	2	High	5586	Unique	Used	75947	1	55.597	1	5.6391	-3.9522	1	100	22/34	11.56687	5586	2078.11	MS2	1039.56	152	Glioma_grade2_5.raw
16507237			tFAPEEISAmVLTk	Unambiguous	N-Term(iTRAQ4plex); M10(Oxidation); K14(iTRAQ4plex)	HCD	1.7498747	0	1	1	1.79902	1.7161	1.7853	1.7029954	2.760851471	10567.958	11078.875	19011.973	18867.271	3.337882039	0.540241856	3	High	5933	Unique	Used	53362	1	55.23	1	4.5639	-1.1	1	26	21/52	41.67964	5933	1840.99	MS2	614.337	217	Glioma_grade2_2.raw
16507237			lYGSAGPPPTGEEDTAEkDEL	Unambiguous	N-Term(iTRAQ4plex); K18(iTRAQ4plex)	HCD	2.2181213	1	1	1	2.19548	2.3249	2.1173	2.2421021	3.914058378	9989.415	9433.259	21931.516	21150.33	4.049511195	2.564337275	2	High	3989	Unique	Used	51533	1	40.985	1	3.8996	0.6033	1	81	18/40	30.65017	3989	2464.2	MS2	1232.6	129	Glioma_grade2_2.raw
16507237			lYGSAGPPPTGEEDTAEkDEL	Unambiguous	N-Term(iTRAQ4plex); K18(iTRAQ4plex)	HCD	1.5054084	1	1	1	1.29305	1.4195	1.5836	1.7385325	12.85809471	9694.599	8830.875	12535.64	15352.764	6.593570534	14.28556101	3	High	3985	Unique	Used	51529	1	40.96	1	4.2726	-0.1948	1	31	28/80	4.044229	3985	2464.2	MS2	822.07	96	Glioma_grade2_2.raw
16507237			nQLTSNPENTVFDAk	Unambiguous	N-Term(iTRAQ4plex); K15(iTRAQ4plex)	HCD	1.7147665	0	1	1	1.54172	1.4638	1.9791	1.8790699	14.65804392	8656.519	9117.295	13345.904	17132.035	3.666268075	17.56810987	2	High	3698	Unique	Used	51258	1	38.886	1	3.736	-0.1429	1	100	19/28	0	3698	1966.01	MS2	983.51	204	Glioma_grade2_2.raw
16507237			kSQIFSTASDNQPTVTIk	Unambiguous	N-Term(iTRAQ4plex); K1(iTRAQ4plex); K18(iTRAQ4plex)	HCD	3.3549995	1	1	1	3.45697	2.8833	3.9205	3.2699478	12.73648245	6622.7153	7940.3374	22894.549	25964.49	12.79538763	8.885873089	3	High	2590	Unique	Used	79997	1	31.017	1	7.5684	8.4207	1	20	28/68	8.095606	2590	2397.36	MS2	799.79	96	Glioma_grade2_6.raw
16507237			kTkPYIQVDIGGGQTk	Unambiguous	N-Term(iTRAQ4plex); K1(iTRAQ4plex); K3(iTRAQ4plex); K16(iTRAQ4plex)	HCD	1.5153381	1	1	1	1.37502	1.1827	1.902	1.6360341	20.54695581	1192.6931	1386.6201	1639.9738	2268.5579	10.63283798	22.74389023	4	High	2214	Unique	Used	94983	1	32.045	1	5.7929	-2.0966	1	100	31/90	39.6542	2214	2309.36	MS2	578.096	52	Glioma_grade2_8rep.raw
16579885	NP_000959.2	RPL4	60S ribosomal protein L4 [Homo sapiens]	10	27	1.998665571	29.75789475	147.55529	30.44	1.59E-04	427	47.667	11.063	Ribosomal subunit	Protein metabolism	Structural constituent of ribosome	Ribosome	Nucleolus																								
16579885			aAAAAAALQAk	Unambiguous	N-Term(iTRAQ4plex); K11(iTRAQ4plex)	HCD	2.3129873	0	1	1	2.30121	2.3098	2.3162	2.3248093	0.431743021	2130865.8	2122972.5	4903568	4935506.5	0.262417401	0.459066143	2	High	1487	Unique	Used	49172	1	23.261	1	4.9716	-0.2202	1	1	20/20	11.1219	1487	1244.76	MS2	622.882	167	Glioma_grade2_2.raw
16579885			eAVLLLk	Unambiguous	N-Term(iTRAQ4plex); K7(iTRAQ4plex)	HCD	1.959996	0	1	1	1.81362	1.8728	2.0444	2.1111448	7.153133367	1034850.56	1002146.06	1876829.8	2115675.5	2.270555925	8.460322601	2	High	3161	Unique	Used	50750	1	35.062	1	3.0217	-0.6398	1	3	12-Dec	12.61231	3161	1073.72	MS2	537.362	77	Glioma_grade2_2.raw
16579885			aAAAAAALQAk	Unambiguous	N-Term(iTRAQ4plex); K11(iTRAQ4plex)	HCD	1.7971671	0	1	1	1.87865	1.6974	1.9075	1.7235457	5.917333558	752837.06	833206.2	1414315.8	1436068.9	7.166206033	1.079276388	2	High	1546	Unique	Used	57281	1	23.137	1	4.6792	-0.5144	1	3	19/20	10.96623	1546	1244.76	MS2	622.882	174	Glioma_grade2_3.raw
16579885			nVTLPAVFk	Unambiguous	N-Term(iTRAQ4plex); K9(iTRAQ4plex)	HCD	2.3948996	0	1	1	2.25513	2.3816	2.4075	2.5425093	4.916053151	554398.9	524954.6	1250241.2	1334702.1	3.857913871	4.620826703	2	High	4647	Unique	Used	52150	1	45.893	1	3.5356	-1.2245	1	5	14/16	8.799735	4647	1276.79	MS2	638.896	169	Glioma_grade2_2.raw
16579885			nVTLPAVFk	Unambiguous	N-Term(iTRAQ4plex); K9(iTRAQ4plex)	HCD	2.7066197	0	1	1	2.53405	2.6106	2.7998	2.8844104	6.004826359	524607.56	509215.7	1329379.5	1468787	2.105522095	7.045755755	2	High	4570	Unique	Used	60097	1	46.008	1	3.6107	0.114	1	4	15/16	8.276269	4570	1276.79	MS2	638.897	168	Glioma_grade2_3.raw
16579885			nVTLPAVFk	Unambiguous	N-Term(iTRAQ4plex); K9(iTRAQ4plex)	HCD	2.7082512	0	1	1	2.49226	2.8295	2.6015	2.9534714	7.726336617	280707.4	247250.45	699595.5	730247.1	8.96194127	3.031655962	2	High	4478	Unique	Used	60013	1	45.267	1	3.8481	1.0701	1	13	14/16	21.04283	4478	1276.79	MS2	638.898	161	Glioma_grade2_3.raw
16579885			eAVLLLk	Unambiguous	N-Term(iTRAQ4plex); K7(iTRAQ4plex)	HCD	1.8157492	0	1	1	1.77703	1.7664	1.8654	1.8542334	2.821597126	229031.44	230406.77	406996.66	427227.94	0.423345359	3.429693941	2	High	3169	Unique	Used	58796	1	35.147	1	2.9939	-3.3684	1	25	12-Dec	44.30544	3169	1073.71	MS2	537.361	77	Glioma_grade2_3.raw
16579885			kLDELYGTWR	Unambiguous	N-Term(iTRAQ4plex); K1(iTRAQ4plex)	HCD	1.9420513	1	1	1	1.85511	1.9954	1.8925	2.035566	4.361473773	180477.19	167789.56	334805.16	341546.75	5.15209058	1.409628312	2	High	3588	Unique	Used	80864	1	39.568	1	3.9357	-0.426	1	24	17/18	15.11728	3588	1568.87	MS2	784.937	194	Glioma_grade2_6.raw
16579885			aPIRPDIVNFVHTNLR	Unambiguous	N-Term(iTRAQ4plex)	HCD	1.6991297	0	1	1	1.61355	1.7681	1.6361	1.7929084	5.33963139	155727.22	142111.33	251273.75	254792.58	6.465172592	0.983344833	3	High	4389	Unique	Used	91759	1	49.27	1	6.1254	-3.7006	1	5	23/60	28.30344	4389	2006.13	MS2	669.383	116	Glioma_grade2_7rep.raw
16579885			nVTLPAVFk	Unambiguous	N-Term(iTRAQ4plex); K9(iTRAQ4plex)	HCD	1.5273404	0	1	1	1.55827	1.5297	1.525	1.4969808	1.642849006	146729.61	149473.39	228644.02	223758.78	1.310010664	1.527128626	2	High	4561	Unique	Used	52071	1	45.223	1	3.3182	1.6437	1	20	13/16	25.22426	4561	1276.79	MS2	638.898	148	Glioma_grade2_2.raw
16579885			nVTLPAVFk	Unambiguous	N-Term(iTRAQ4plex); K9(iTRAQ4plex)	HCD	1.8366252	0	1	1	1.7816	1.8551	1.8189	1.8939183	2.622539743	130348.664	125182.65	232229.56	237085.7	2.859080927	1.463327455	2	High	4393	Unique	Used	59936	1	44.562	1	3.7447	-2.085	1	32	13/16	42.99923	4393	1276.78	MS2	638.896	185	Glioma_grade2_3.raw
16579885			aAAAAAALQAk	Unambiguous	N-Term(iTRAQ4plex); K11(iTRAQ4plex)	HCD	2.6091738	0	1	1	2.28144	2.6932	2.538	2.9960585	11.37524909	102678.53	86978.84	234255.34	260593.7	11.70675019	7.527157357	3	High	1489	Unique	Used	49174	1	23.269	1	3.7789	-1.7756	1	3	21/40	7.179262	1489	1244.75	MS2	415.59	174	Glioma_grade2_2.raw
16579885			nIPGITLLNVSk	Unambiguous	N-Term(iTRAQ4plex); K12(iTRAQ4plex)	HCD	2.00389	0	1	1	1.85834	1.9758	2.0303	2.158636	6.214005731	73774.2	69388.84	137097.72	149785.27	4.332008868	6.254433309	2	High	5803	Unique	Used	61239	1	55.652	1	4.9555	1.6632	1	54	19/22	34.06762	5803	1556.96	MS2	778.986	65	Glioma_grade2_3.raw
16579885			iEEVPELPLVVEDk	Unambiguous	N-Term(iTRAQ4plex); K14(iTRAQ4plex)	HCD	2.2262046	0	1	1	2.1386	2.1886	2.2629	2.3158555	3.523923343	71842.664	70199.94	153642.98	162572.92	1.635539264	3.993740435	2	High	6542	Unique	Used	53937	1	59.64	1	4.4615	1.1049	1	28	18/26	9.323347	6542	1897.08	MS2	949.043	170	Glioma_grade2_2.raw
16579885			fcIWTESAFR	Unambiguous	N-Term(iTRAQ4plex); C2(Methylthio)	HCD	1.9041891	0	1	1	1.75787	1.9746	1.8415	2.0685537	7.223798672	61659.895	54891.848	108389.83	113546.73	8.21220139	3.286055222	2	High	5351	Unique	Used	87668	1	69.236	1	3.7829	1.476	1	71	14/18	31.12337	5351	1449.68	MS2	725.344	84	Glioma_grade2_1rep.raw
16579885			aAAAAAALQAk	Unambiguous	N-Term(iTRAQ4plex); K11(iTRAQ4plex)	HCD	2.589934	0	1	1	2.76609	2.3776	2.8369	2.4385169	8.842189123	47684.953	55475.54	131900.84	135278.05	10.68001274	1.787602376	3	High	1531	Unique	Used	57267	1	23.033	1	3.7263	-0.6723	1	8	21/40	7.321525	1531	1244.76	MS2	415.59	176	Glioma_grade2_3.raw
16579885			qPYAVSELAGHQTSAESWGTGR	Unambiguous	N-Term(iTRAQ4plex)	HCD	3.9679139	0	1	1	4.3945	3.6446	4.3577	3.6141155	10.78174331	44461.31	53609.023	195385.42	193749.2	13.19136929	0.594643703	3	High	4004	Unique	Used	59572	1	41.582	1	6.7485	0.989	1	6	28/84	2.323943	4004	2476.2	MS2	826.072	90	Glioma_grade2_3.raw
16579885			aPIRPDIVNFVHTNLR	Unambiguous	N-Term(iTRAQ4plex)	HCD	3.9335568	0	1	1	4.20117	3.3529	4.6612	3.719981	14.30075393	38120.137	47764.68	160149.11	177683.7	15.88108817	7.340215117	4	High	4392	Unique	Used	91761	1	49.305	1	5.0284	-4.407	1	2	22/90	24.08369	4392	2006.13	MS2	502.289	146	Glioma_grade2_7rep.raw
16579885			qPYAVSELAGHQTSAESWGTGR	Unambiguous	N-Term(iTRAQ4plex)	HCD	3.3654287	0	1	1	3.41263	3.2464	3.4906	3.320531	3.163887462	32481.477	34144.75	110847.12	113378.7	3.530476421	1.596688454	3	High	4100	Unique	Used	51637	1	41.833	1	6.3366	1.4327	1	14	30/84	4.151562	4100	2476.2	MS2	826.072	95	Glioma_grade2_2.raw
16579885			nIPGITLLNVSk	Unambiguous	N-Term(iTRAQ4plex); K12(iTRAQ4plex)	HCD	1.7046226	0	1	1	1.42041	1.6765	1.7284	2.040072	14.82015588	25492.307	21598.287	36209.633	44062.062	11.69442861	13.83428068	3	High	5827	Unique	Used	61261	1	55.846	1	4.2974	1.6938	1	23	19/44	16.07132	5827	1556.96	MS2	519.66	62	Glioma_grade2_3.raw
16579885			aPIRPDIVNFVHTNLR	Unambiguous	N-Term(iTRAQ4plex)	HCD	1.8689798	0	1	1	1.79816	1.7689	1.9707	1.9386462	5.370598419	20670.824	21012.627	37169.48	40736.05	1.159650717	6.474369233	2	High	4401	Unique	Used	91768	1	49.392	1	4.9197	-2.5073	1	57	19/30	0	4401	2006.14	MS2	1003.57	132	Glioma_grade2_7rep.raw
16579885			yAIcSALAASALPALVmSk	Unambiguous	N-Term(iTRAQ4plex); C4(Methylthio); M17(Oxidation); K19(iTRAQ4plex)	HCD	1.8362824	0	1	1	1.62505	1.9434	1.7467	2.0888872	11.11775605	18260.51	15269.408	29674.324	31896.074	12.61576904	5.103148728	3	High	8631	Unique	Used	55847	1	76.284	1	4.8812	-0.1581	1	14	24/72	26.30626	8631	2230.19	MS2	744.068	163	Glioma_grade2_2.raw

16579885			kLDELYGTWR	Unambiguous	N-Term(iTRAQ4plex); K1(iTRAQ4plex)	HCD	1.9986656	1	1	1	2.00264	1.9212	2.0794	1.9948522	3.233129481	16620.49	17324.742	33284.867	34560.297	2.934028348	2.65859834	3	High	3577	Unique	Used	80856	1	39.448	1	3.997	-2.4187	1	32	21/36	21.89112	3577	1568.86	MS2	523.626	196	Glioma_grade 2_6.raw
16579885			nVTLPAVFk	Unambiguous	N-Term(iTRAQ4plex); K9(iTRAQ4plex)	HCD	2.991139	0	1	1	2.61928	3.2674	2.7696	3.4550223	13.12066858	11979.329	9602.988	31377.168	33178.54	15.57132939	3.946242388	3	High	4544	Unique	Used	60073	1	45.801	1	3.7585	-0.1117	1	52	20/32	6.837997	4544	1276.79	MS2	426.267	165	Glioma_grade 2_3.raw
16579885			aPIRPDIVNFVHTNLR	Unambiguous	N-Term(iTRAQ4plex)	HCD	1.6509099	0	1	1	1.53638	2.0044	1.3799	1.8003248	16.49284046	8485.576	6504.151	13037.105	11709.584	18.69385685	7.586462183	4	High	4496	Unique	Used	91838	1	50.399	1	4.3793	-0.3302	1	36	21/90	49.91182	4496	2006.14	MS2	502.291	136	Glioma_grade 2_7rep.raw
16579885			nVTLPAVFk	Unambiguous	N-Term(iTRAQ4plex); K9(iTRAQ4plex)	HCD	2.9577568	0	1	1	2.77008	3.1677	2.7742	3.1723673	7.732734599	4920.103	4302.525	13629.1	13649.189	9.470046753	0.10414926	3	High	4450	Unique	Used	59989	1	45.012	1	4.2912	-3.5535	1	100	20/32	57.00153	4450	1276.78	MS2	426.266	181	Glioma_grade 2_3.raw
16579885			nVTLPAVFk	Unambiguous	N-Term(iTRAQ4plex); K9(iTRAQ4plex)	HCD	1.504442	0	1	1	1.27063	1.3354	1.6653	1.7501705	15.78918292	4450.9683	4235.061	5655.548	7412.079	3.515288993	19.00964852	3	High	4734	Unique	Used	52233	1	46.498	1	3.4827	-1.5458	1	63	15/32	8.20714	4734	1276.78	MS2	426.266	193	Glioma_grade 2_2.raw
16933546	NP_444505.1	RPLP0	60S acidic ribosomal protein P0 [Homo sapiens]	6	11	1.998790979	13.535609	93.346379	39.75	7.59E-05	317	34.252	5.9731	Ribosomal subunit	Protein metabolism	Structural constituent of ribosome	Ribosome	Cytosol, Cytoplasm, Mitochondrion, Nucleolus, Nucleus																								
16933546			gNVGFVFTk	Unambiguous	N-Term(iTRAQ4plex); K9(iTRAQ4plex)	HCD	2.331636	0	1	1	2.29242	2.3588	2.3052	2.371983	1.678971003	651631.7	633294.75	1493816.2	1502164.4	2.01819827	0.394065892	2	High	3444	Unique	Used	51019	1	37.049	1	3.7128	-2.1358	1	4	16/16	10.55316	3444	1256.72	MS2	628.864	218	Glioma_grade 2_2.raw
16933546			gNVGFVFTk	Unambiguous	N-Term(iTRAQ4plex); K9(iTRAQ4plex)	HCD	2.1325827	0	1	1	2.13411	2.1286	2.1366	2.1310577	0.165171847	294587.3	295356.62	628682.2	629422	0.18442139	0.083159661	2	High	3427	Unique	Used	59039	1	37.065	1	3.9207	0.5839	1	10	15/16	16.35464	3427	1256.72	MS2	628.866	215	Glioma_grade 2_3.raw
16933546			gNVGFVFTk	Unambiguous	N-Term(iTRAQ4plex); K9(iTRAQ4plex)	HCD	1.998791	0	1	1	2.01129	1.9924	2.0053	1.9864082	0.572402099	278745.2	281391.75	560637.75	558958.94	0.668191396	0.212058136	2	High	3328	Unique	Used	58946	1	36.315	1	4.0665	0.9724	1	14	15/16	11.23927	3328	1256.73	MS2	628.866	214	Glioma_grade 2_3.raw
16933546			tSFFQALGITTk	Unambiguous	N-Term(iTRAQ4plex); K12(iTRAQ4plex)	HCD	1.9132338	0	1	1	1.7584	2.0105	1.8282	2.0902653	8.044278207	159755.56	139726.53	280913.72	292065.53	9.458103444	2.752462842	2	High	6844	Unique	Used	54216	1	61.962	1	5.8551	-0.1057	1	21	20/22	8.196064	6844	1601.91	MS2	801.461	165	Glioma_grade 2_2.raw
16933546			iIQLLDDYPk	Unambiguous	N-Term(iTRAQ4plex); K10(iTRAQ4plex)	HCD	1.796906	0	1	1	1.78095	1.7595	1.8347	1.8126698	1.85457335	142284.58	144017.1	253401.75	261055.44	0.855794238	2.103955861	2	High	6211	Unique	Used	61621	1	58.805	1	4.8311	-1.6384	1	27	17/18	17.17232	6211	1505.88	MS2	753.443	196	Glioma_grade 2_3.raw
16933546			aGAIAPcEVTVPAQNTGLGPEk	Unambiguous	N-Term(iTRAQ4plex); C7(Methylthio); K22(iTRAQ4plex)	HCD	1.6063837	0	1	1	1.5112	1.8605	1.4	1.7235677	12.75831365	89758.836	72906.914	135643.61	125660	14.65103542	5.403276542	3	High	5585	Unique	Used	53035	1	52.657	1	4.5728	-0.3105	1	4	24/84	27.99321	5585	2457.27	MS2	819.762	135	Glioma_grade 2_2.raw
16933546			cFIVGADNVGSk	Unambiguous	N-Term(iTRAQ4plex); C1(Methylthio); K12(iTRAQ4plex)	HCD	2.274706	0	1	1	2.10468	2.2761	2.2734	2.4585812	6.344462162	88105.58	81470.66	185433.81	200302.23	5.53331873	5.451168425	2	High	4472	Unique	Used	60008	1	45.21	1	4.576	2.1709	1	83	19/22	24.3684	4472	1543.79	MS2	772.398	225	Glioma_grade 2_3.raw
16933546			vLALSVETDYTFPLAEk	Unambiguous	N-Term(iTRAQ4plex); K17(iTRAQ4plex)	HCD	2.1361961	0	1	1	1.97055	2.0851	2.1845	2.3114665	6.78341878	37821.65	35744.4	74529.53	82621.98	3.993248411	7.282432439	2	High	7484	Unique	Used	54804	1	66.966	1	6.2281	-0.5058	1	52	22/32	19.56527	7484	2184.2	MS2	1092.61	167	Glioma_grade 2_2.raw
16933546			vLALSVETDYTFPLAEk	Unambiguous	N-Term(iTRAQ4plex); K17(iTRAQ4plex)	HCD	1.9970678	0	1	1	1.8937	2.0696	1.9307	2.1100414	5.246006685	18999.441	17384.412	35979.195	36681.83	6.277498745	1.367550136	2	High	7584	Unique	Used	54895	1	67.77	1	5.4061	0.5002	1	54	23/32	7.926195	7584	2184.21	MS2	1092.61	151	Glioma_grade 2_2.raw
16933546			cFIVGADNVGSk	Unambiguous	N-Term(iTRAQ4plex); C1(Methylthio); K12(iTRAQ4plex)	HCD	1.5589895	0	1	1	1.52821	1.3608	1.7816	1.5863978	11.10050818	15637.18	17561.516	23896.87	27859.549	8.197376396	10.82778618	3	High	4603	Unique	Used	52110	1	45.543	1	3.5378	2.439	1	38	16/44	39.28872	4603	1543.79	MS2	515.268	202	Glioma_grade 2_2.raw
16933546			aGAIAPcEVTVPAQNTGLGPEk	Unambiguous	N-Term(iTRAQ4plex); C7(Methylthio); K22(iTRAQ4plex)	HCD	2.3465002	0	1	1	2.99503	2.1797	2.5757	1.874522	20.20982597	15284.3	21001.693	45776.95	39368.137	22.28301902	10.64468965	2	High	5593	Unique	Used	53043	1	52.703	1	4.7889	-2.4163	1	15	23/42	0	5593	2457.27	MS2	1229.14	128	Glioma_grade 2_2.raw
17105394	NP_000975.2	RPL23A	60S ribosomal protein L23a [Homo sapiens]	5	12	3.028947592	26.39955219	68.59277	42.31	2.65E-05	156	17.684	10.448	RNA binding protein	Regulation of nucleobase, nucleoside, nucleotide and nucleic acid metabolism	RNA binding	Cytoplasm	Mitochondrion, Nucleolus																								
17105394			lDHYAIIk	Unambiguous	N-Term(iTRAQ4plex); K8(iTRAQ4plex)	HCD	3.4672554	0	1	1	3.39679	3.5622	3.3767	3.5411496	2.766074941	915618.75	873099.9	3110167	3091777.2	3.361665309	0.419337932	2	High	1893	Unique	Used	79345	1	25.875	1	3.6429	3.947	1	3	14/14	6.865871	1893	1260.76	MS2	630.884	259	Glioma_grade 2_6.raw
17105394			lDHYAIIk	Unambiguous	N-Term(iTRAQ4plex); K8(iTRAQ4plex)	HCD	3.458573	0	1	1	3.20417	3.9771	3.0408	3.7743468	12.79476513	596094.6	480241.75	1909989.1	1812598.9	15.22206991	3.699859928	3	High	1892	Unique	Used	79344	1	25.872	1	3.8027	3.7185	1	2	15/28	8.001315	1892	1260.76	MS2	420.925	259	Glioma_grade 2_6.raw
17105394			fPLTTESAMk	Unambiguous	N-Term(iTRAQ4plex); K10(iTRAQ4plex)	HCD	3.0592632	0	1	1	2.96382	3.1993	2.9295	3.162287	4.463342987	557302.5	516278.88	1651746.2	1632622	5.403983421	0.823467448	2	High	3345	Unique	Used	50925	1	36.342	1	3.1664	1.3936	1	4	16/18	6.693771	3345	1412.77	MS2	706.89	217	Glioma_grade 2_2.raw
17105394			vNTLIRPDGEk	Unambiguous	N-Term(iTRAQ4plex); K11(iTRAQ4plex)	HCD	3.3106883	0	1	1	3.20697	3.2207	3.4003	3.4148526	3.387740532	393030.7	391355.97	1260436.6	1336423	0.301946217	4.13811349	2	High	1281	Unique	Used	78774	1	21.39	1	4.282	1.5607	1	6	17/20	8.12505	1281	1529.89	MS2	765.449	167	Glioma_grade 2_6.raw
17105394			lAPDYDALDVANk	Unambiguous	N-Term(iTRAQ4plex); K13(iTRAQ4plex)	HCD	1.8769137	0	1	1	1.70976	1.8588	1.8936	2.0586338	7.616781446	306952.62	282346.44	524815.56	581247.94	5.90504819	7.215448042	2	High	4761	Unique	Used	52258	1	46.706	1	5.0163	0.0623	1	12	20/24	13.65603	4761	1692.9	MS2	846.956	216	Glioma_grade 2_2.raw
17105394			fPLTTESAmk	Unambiguous	N-Term(iTRAQ4plex); M9(Oxidation); K10(iTRAQ4plex)	HCD	2.4848304	0	1	1	2.40955	2.569	2.4059	2.565095	3.699657401	275482.7	258382.53	663788.8	662775.7	4.529849665	0.108003777	2	High	2181	Unique	Used	49829	1	28.058	1	2.945	0.2379	1	8	15/18	9.946836	2181	1428.77	MS2	714.886	182	Glioma_grade 2_2.raw
17105394			fPLTTESAMk	Unambiguous	N-Term(iTRAQ4plex); K10(iTRAQ4plex)	HCD	2.2486832	0	1	1	2.13746	2.4255	2.0928	2.3748982	7.391551575	272965.47	240545.48	583452.4	571271	8.928493063	1.49188118	2	High	3246	Unique	Used	50831	1	35.654	1	3.4204	1.8256	1	14	15/18	28.70914	3246	1412.77	MS2	706.89	223	Glioma_grade 2_2.raw
17105394			kLYDIDVAk	Unambiguous	N-Term(iTRAQ4plex); K1(iTRAQ4plex); K9(iTRAQ4plex)	HCD	3.4532392	1	1	1	3.08202	3.6217	3.3099	3.889466	10.17221839	188083.94	160055.28	579678.44	622529.6	11.38582177	5.040782429	2	High	2269	Unique	Used	90060	1	28.931	1	3.7981	0.3011	1	26	16/16	10.63622	2269	1496.91	MS2	748.956	118	Glioma_grade 2_7rep.raw
17105394			fPLTTESAMk	Unambiguous	N-Term(iTRAQ4plex); K10(iTRAQ4plex)	HCD	3.107627	0	1	1	3.1338	3.2678	2.9541	3.0803354	4.179397735	187563.31	179874.02	587785.9	554072.25	2.959497393	4.175501227	2	High	3255	Unique	Used	58877	1	35.785	1	3.4512	-0.1617	1	15	16/18	5.752912	3255	1412.77	MS2	706.888	208	Glioma_grade 2_3.raw
17105394			lAPDYDALDVANk	Unambiguous	N-Term(iTRAQ4plex); K13(iTRAQ4plex)	HCD	2.998632	0	1	1	3.01869	2.9244	3.0753	2.979204	2.123854396	150550.03	155406.52	454463.34	462987.7	2.244800454	1.313995516	2	High	4668	Unique	Used	52170	1	46.036	1	5.7128	0.7834	1	32	21/24	22.69496	4668	1692.91	MS2	846.957	209	Glioma_grade 2_2.raw
17105394			lDHYAIIk	Unambiguous	N-Term(iTRAQ4plex); K8(iTRAQ4plex)	HCD	1.691954	0	1	1	1.42523	1.709	1.6777	2.0117848	14.08252417	24817.562	20696.863	35370.684	41637.633	12.8037395	11.50889231	3	High	2517	Unique	Used	95181	1	35.792	1	3.6176	0.1603	1	100	16/28	42.98912	2517	1260.76	MS2	420.923	287	Glioma_grade 2_8rep.raw
17105394			lDHYAIIk	Unambiguous	N-Term(iTRAQ4plex); K8(iTRAQ4plex)	HCD	1.5692714	0	1	1	1.60357	1.4764	1.6701	1.5376893	5.315767965	24583.635	26701.305	39421.676	41058.312	5.839604443	2.875935851	3	High	2373	Unique	Used	85681	1	33.634	1	3.8484	-4.8503	1	56	16/28	30.2318	2373	1260.75	MS2	420.921	282	Glioma_grade 2_1rep.raw
17402888	NP_055108.2	NPTXR	neuronal pentraxin receptor [Homo sapiens]	5	6	0.444683224	23.82681642	63.23117	22.8	1.16E-04	500	52.813	6.2144	Cell surface receptor	Cell communication,Signal transduction	Receptor activity	Plasma membrane	-																								
17402888			vAQLPLSLk	Unambiguous	N-Term(iTRAQ4plex); K9(iTRAQ4plex)	HCD	0.38343275	0	1	1	0.36616	0.3979	0.3701	0.40220135	0.048410331	504522.12	464237.38	184737.23	186716.9	5.880843044	0.753707103	2	High	4306	Unique	Used	59855	1	43.89	1	3.3122	7.4576	1	21	16-Dec	63.8854	4306	1256.83	MS2	628.917	41	Glioma_grade 2_3.raw
17402888			vAQLPLSLk	Unambiguous	N-Term(iTRAQ4plex); K9(iTRAQ4plex)	HCD	0.3418145	0	1	1	0.32721	0.336	0.3475	0.3568146	0.037948951	340128.03	331254.97	111291.836	118196.6	1.869037761	4.255033963	2	High	4587	Unique	Used	67789	1	45.437	1	3.3685	9.8857	1	32	13/16	66.7386	4587	1256.83	MS2	628.919	29	Glioma_grade 2_4.raw
17402888			iDRLEQELPAR	Unambiguous	N-Term(iTRAQ4plex)	HCD	0.491489	1	1	1	0.46606	0.5243	0.4623	0.52010125	0.068104555	44318.348	39392.74	20654.863	20488.213	8.321312986	0.572827103	2	High	3267	Unique	Used	66565	1	35.134	1	2.9589	-1.7045	1	49	14/20	26.67797	3267	1483.83	MS2	742.42	187	Glioma_grade 2_4.raw
17402888			mDQLEGQLLAQVLALEk	Unambiguous	N-Term(iTRAQ4plex); M1(Oxidation); K17(iTRAQ4plex)	HCD	0.39787745	0	1	1	0.41524	0.4381	0.3597	0.3795531	0.088342799	25534.72	24201.309	10603.162	9185.683	3.791472617	10.13004057	3	High	8529	Unique	Used	55751	1	75.558	1	6.7062	-0.8945	1	19	27/64	13.77163	8529	2203.22	MS2	735.079	151	Glioma_grade 2_2.raw
17402888			vAELEHGSSAYSPPDAFk	Unambiguous	N-Term(iTRAQ4plex); K18(iTRAQ4plex)	HCD	0.63730997	0	1	1	0.54976	0.4993	0.7893	0.71682394	0.21409741	19623.938	21606.941	10788.478	15488.373	6.801673418	25.29471759	3	High	3892	Unique	Used	67142	1	40.075	1	7.5548	1.2689	1	40	28/68	18.81866	3892	2193.11	MS2	731.708	158	Glioma_grade 2_4.raw
17402888			rDTMADGPWDSPALILELEDAVR	Unambiguous	N-Term(iTRAQ4plex)	HCD	0.53092253	1	1	1	0.41688	0.4223	0.6382	0.64643705	0.242309683	5764.1743	5690.513	2402.9932	3678.5588	0.9094339	29.66220089	3	High	8220	Unique	Used	71045	1	75.837	1	8.2343	0.0962	1	30	30/88	11.57304	8220	2714.36	MS2	905.458	135	Glioma_grade 2_4.raw
17978471	NP_006086.1	ATP8A1	probable phospholipid-transporting ATPase IA isoform a [Homo sapiens]	9	14	0.36559993	28.85853031	84.374818	10.48	1.80E-04	1164	131.29	6.8442	ATPase	Transport	ATPase activity	Integral to membrane	-																								
17978471			sQDPGAVVLGk	Unambiguous	N-Term(iTRAQ4plex); K11(iTRAQ4plex)	HCD	0.3699668	0	1	2	0.32348	0.3646	0.3747	0.42236432	0.10941043	1311871.8	1163886.6	424364.25	491584.2	8.453275444	10.37868069	2	High	1793	Unique	Used	49462	1	25.358	1	3.9956	-1.6036	1	5	18/20	15.45878	1793	1358.79	MS2	679.897	198	Glioma_grade 2_2.raw
17978471			tIFINQPQLTk	Unambiguous	N-Term(iTRAQ4plex); K11(iTRAQ4plex)	HCD	0.2900174	0	1	2	0.2538	0.2874	0.2924	0.33102173	0.10862288	711041.8	627981.25	180464.55	207875.44	8.772467084	9.982194312	2	High	4164	Unique	Used	51697	1	42.297	1	4.5435	0.0384	1	12	17/20	11.62512	4164	1590.95	MS2	795.977	138	Glioma_grade 2_2.raw
17978471			vAVGEIVk	Unambiguous	N-Term(iTRAQ4plex); K8(iTRAQ4plex)	HCD	0.45471758	0	1	1	0.42387	0.4457	0.4633	0.48715836	0.058942059	647896.4	616124.2	274622.34	300150.06	3.554742395	6.281033647	2	High	1896	Unique	Used	49560	1	26.053	1	2.9451	8.07	1	10	14/14	69.51157	1896	1102.72	MS2	551.862	128	Glioma_grade 2_2.raw
17978471			hLYDFVGNIR	Unambiguous	N-Term(iTRAQ4plex)	HCD	0.3130084	0	1	2	0.29336	0.2968	0.329	0.33288327	0.066532488	474960.3	469479.78	139335.7	156281.97	0.82065828	8.106973059	2	High	3930	Unique	Used	74466	1	42.08	1	3.3895	1.8354	1	17	16/18	19.62149	3930	1377.74	MS2	689.375	290	Glioma_grade 2_5.raw
17978471			hLEQFATEGLR	Unambiguous	N-Term(iTRAQ4plex)	HCD	0.2192347	0	1	2	0.2005	0.2105	0.2276	0.23890297	0.078300576	467531.1	445417.44	93738.46	106411.56	3.425542242	8.954514855	2	High	2226	Unique	Used	72917	1	28.72	1	3.999	0.7485	1	31	16/20	22.86123	2226	1444.77	MS2	722.887	195	Glioma_grade 2_5.raw
17978471			nLQLLGATAIEDk	Unambiguous	N-Term(iTRAQ4plex); K13(iTRAQ4plex)	HCD	0.36123303	0	1	2	0.31771	0.3694	0.3542	0.41183066	0.107231034	287847.56	247588.31	91452.664	101964.46	10.63342606	7.68594019	2	High	5393	Unique	Used	52853	1	51.295	1	5.9209	0.9669	1	23	20/24	18.28668	5393	1673.97	MS2	837.488	169	Glioma_grade 2_2.raw
17978471			hLEQFATEGLR	Unambiguous	N-Term(iTRAQ4plex)	HCD	0.24982394	0	1	2	0.20988	0.2423	0.2563	0.29593444	0.141979123	196639.73	170323.94	41271.594	50404.72	10.14164348	14.08890704	2	High	2650	Unique	Used	65989	1	30.487	1	3.7185	14.267	1	48	16/20	33.14383	2650	1444.79	MS2	722.897	217	Glioma_grade 2_4.raw
17978471			tIFINQPQLTk	Unambiguous	N-Term(iTRAQ4plex); K11(iTRAQ4plex)	HCD	0.3710286	0	1	2	0.37387	0.3318	0.4152	0.36850393	0.091684151	121791.41	137226.4	45534.547	50568.47	8.427363428	7.407719758	2	High	4048	Unique	Used	59614	1	41.896	1	4.3047	-0.7289	1	75	17/20	15.02384	4048	1590.94	MS2	795.976	137	Glioma_grade 2_3.raw
17978471			hLYDFVGNIR	Unambiguous	N-Term(iTRAQ4plex)	HCD	0.41938412	0	1	2	0.39137	0.4097	0.4286	0.44870934	0.058755707	105187.51	100479.41	41167.39	45086.047	3.23739903	6.425040055	2	High	4027	Unique	Used	74555	1	42.838	1	3.0467	1.8354	1	51	16/18	27.82991	4027	1377.74	MS2	689.375	295	Glioma_grade 2_5.raw
17978471			hLYDFVGNIR	Unambiguous	N-Term(iTRAQ4plex)	HCD	0.28098562	0	1	2	0.26868	0.2785	0.2834	0.29373693	0.037099274	61862.355	59689.16	16621.314	17532.912	2.528443881	3.774625884	3	High	3918	Unique	Used	74456	1	41.967	1	3.3687	1.4269	1	52	18/36	5.877202	3918	1377.74	MS2	459.919	207	Glioma_grade 2_5.raw
17978471			lDGHGTVPLGADQILLR	Unambiguous	N-Term(iTRAQ4plex)	HCD	0.2793766	0	1	2	0.27219	0.294	0.2658	0.28714305	0.046648452	53612.65	49626.69	14592.694	14249.96	5.460126625	1.680494004	2	High	5353	Unique	Used	68492	1	51.51	1	6.156	1.279	1	97	22/32	6.214023	5353	1919.09	MS2	960.046	123	Glioma_grade 2_4.raw
17978471			nTQWVHGIVVYTGHDTk	Unambiguous	N-Term(iTRAQ4plex); K17(iTRAQ4plex)	HCD	0.43322638	0	1	2	0.436	0.4289	0.4376	0.4304968	0.009683552	37174.07	37787.535	16207.932	16267.414	1.157353185	0.259028037	3	High	2905	Unique	Used	80251	1	34.063	1	7.0438	2.3485	1	29	34/64	0	2905	2243.19	MS2	748.4	140	Glioma_grade 2_6.raw
17978471			lDGHGTVPLGADQILLR	Unambiguous	N-Term(iTRAQ4plex)	HCD	0.3921224	0	1	2	0.37916	0.373	0.4115	0.40487593	0.048209582	23125.516	23505.814	8768.25	9516.938	1.15335031	5.79050499	3	High	5332	Unique	Used	68472	1	51.36	1	5.0399	1.1448	1	21	25/64	16.92202	5332	1919.09	MS2	640.367	134	Glioma_grade 2_4.raw
17978471			kENDFALIIDGk	Unambiguous	N-Term(iTRAQ4plex); K1(iTRAQ4plex); K12(iTRAQ4plex)	HCD	0.6255585	1	1	2	0.60156	0.6063	0.6446	0.64974815	0.040177882	16462.174	16332.769	9902.97	10612.186	0.558032091	4.888984933	3	High	3794	Unique	Used	91314	1	43.071	1	5.1431	-6.3888	1	69	19/44	38.9419	3794	1795.02	MS2	599.012	158	Glioma_grade 2_7rep.raw
17986258	NP_066299.2	MYL6	myosin light polypeptide 6 isoform 1 [Homo sapiens]	3	8	2.305343866	14.51645084	63.521681	42.38	1.68E-04	151	16.919	4.6528	Cytoskeletal protein	Cell growth and/or maintenance	Structural constituent of cytoskeleton	Cytoplasm	Cytoskeleton																								
17986258			hVLVTLGEk	Unambiguous	N-Term(iTRAQ4plex); K9(iTRAQ4plex)	HCD	2.6085978	0	1	2	2.71913	2.5378	2.6845	2.5054321	4.051459652	108856.875	116634.96	295996.5	292220.97	4.878169223	0.907726479	3	High	1326	Unique	Used	78816	1	21.718	1	4.2165	-8.0361	1	9	19/32	10.31981	1326	1283.78	MS2	428.599	143	Glioma_grade 2_6.raw
17986258			vLDFEHFLPMLQTVAk	Unambiguous	N-Term(iTRAQ4plex); K16(iTRAQ4plex)	HCD	2.679269	0	1	2	2.38574	2.7043	2.6572	3.0119896	9.539740327	103489.664	91298.914	246899.62	274991.38	8.850787948	7.612269225	2	High	7364	Unique	Used	83960	1	75.215	1	4.8833	0.2786	1	10	19/30	3.504591	7364	2176.21	MS2	1088.61	148	Glioma_grade 2_6.raw
17986258			nkDQGTYEDYVEGLR	Unambiguous	N-Term(iTRAQ4plex); K2(iTRAQ4plex)	HCD	1.9807612	1	1	2	2.09971	2.2199	1.7546	1.8550099	10.83027466	48789.18	46148.227	102442.914	85605.414	3.93403577	12.66260706	3	High	3150	Unique	Used	73778	1	35.585	1	5.9515	-1.2363	1	6	25/56	15.39446	3150	2075.03	MS2	692.347	112	Glioma_grade 2_5.raw
17986258			nkDQGTYEDYVEGLR	Unambiguous	N-Term(iTRAQ4plex); K2(iTRAQ4plex)	HCD	2.61221	1	1	2	2.40859	2.6865	2.5456	2.8393273	7.06355863	27583.773	24730.615	66437.87	70218.31	7.712934994	3.912263258	2	High	3142	Unique	Used	73771	1	35.515	1	5.6452	-1.318	1	76	21/28	0	3142	2075.03	MS2	1038.02	120	Glioma_grade 2_5.raw
17986258			vLDFEHFLPmLQTVAk	Unambiguous	N-Term(iTRAQ4plex); M10(Oxidation); K16(iTRAQ4plex)	HCD	2.045233	0	1	2	2.28894	1.9065	2.2118	1.8422498	10.71186573	23834.312	28615.512	54555.195	52716.918	12.89163122	2.423478192	3	High	6694	Unique	Used	83465	1	67.903	1	5.3996	0.6968	1	16	24/60	8.551507	6694	2192.2	MS2	731.406	150	Glioma_grade 2_6.raw

17986258			vLDFEHFLPmLQTVAk	Unambiguous	N-Term(iTRAQ4plex); M10(Oxidation); K16(iTRAQ4plex)	HCD	2.4001646	0	1	2	2.24605	2.5687	2.2528	2.5764215	7.740516508	16744.178	14640.815	37608.234	37720.914	9.4777924	0.211543059	2	High	6724	Unique	Used	83490	1	68.179	1	3.5145	-0.2994	1	68	16/30	0	6724	2192.2	MS2	1096.6	175	Glioma_grade2_6.raw
17986258			vLDFEHFLPMLQTVAk	Unambiguous	N-Term(iTRAQ4plex); K16(iTRAQ4plex)	HCD	2.2105231	0	1	2	2.0005	2.4182	2.0388	2.4643917	10.96937097	5860.123	4847.991	11723.185	11947.349	13.36716065	1.339284399	3	High	7316	Unique	Used	83926	1	74.667	1	6.2472	-2.0274	1	91	24/60	21.73699	7316	2176.2	MS2	726.073	146	Glioma_grade2_6.raw
17986258			vLDFEHFLPmLQTVAk	Unambiguous	N-Term(iTRAQ4plex); M10(Oxidation); K16(iTRAQ4plex)	HCD	1.7962155	0	1	2	1.28284	1.9026	1.7245	2.557595	28.33963518	1600.4673	1079.1481	2053.1428	2760.024	27.51352612	20.76971403	4	High	6713	Unique	Used	83482	1	68.057	1	4.3398	0.1895	1	57	23/90	7.712997	6713	2192.2	MS2	548.806	191	Glioma_grade2_6.raw
18034692	NP_536856.2	SLC44A1	choline transporter-like protein 1 [Homo sapiens]	3	4	2.139163017	8.677582934	34.355107	9.13	4.67E-04	657	73.253	8.6021	Integral membrane protein	Transport	Molecular function unknown	Integral to membrane	Cytoplasm, Plasma membrane																								
18034692			lVSGYDSYGNlcGQk	Unambiguous	N-Term(iTRAQ4plex); C12(Methylthio); K15(iTRAQ4plex)	HCD	2.1878374	0	1	1	2.14384	2.1599	2.2155	2.232162	1.947330642	39983.605	39685.7	85718.58	88584.91	0.52881256	2.325600457	2	High	5695	Unique	Used	53139	1	53.444	1	6.1873	-0.2369	1	94	24/28	11.99844	5695	1937.93	MS2	969.47	91	Glioma_grade2_2.raw
18034692			fAEALITFVSDNSVLHR	Unambiguous	N-Term(iTRAQ4plex)	HCD	1.9783103	0	1	1	1.61087	2.3189	1.7417	2.5072598	21.30239089	31926.654	22178.48	51429.535	55607.21	25.48002169	5.519716285	3	High	8267	Unique	Used	71088	1	76.198	1	5.1827	-0.7406	1	3	26/64	2.077336	8267	2063.1	MS2	688.372	175	Glioma_grade2_4.raw
18034692			lPVPASAPIPFFHR	Unambiguous	N-Term(iTRAQ4plex)	HCD	2.420858	0	1	1	2.04261	2.6513	2.2434	2.9118197	15.93518985	22934.908	17669.744	46846.98	51451.11	18.33796368	6.623956873	3	High	5215	Unique	Used	92382	1	57.756	1	4.887	-1.7713	1	15	24/52	0	5215	1692.97	MS2	564.994	152	Glioma_grade2_7rep.raw
18034692			lVSGYDSYGNlcGQk	Unambiguous	N-Term(iTRAQ4plex); C12(Methylthio); K15(iTRAQ4plex)	HCD	2.0904887	0	1	1	1.98013	2.3927	1.8404	2.223845	11.69046495	17821.6	14748.788	35289.01	32799.02	13.34221872	5.171801311	3	High	5726	Unique	Used	53167	1	53.698	1	3.4642	0.7955	1	24	19/56	15.40147	5726	1937.94	MS2	646.65	77	Glioma_grade2_2.raw
18104948	NP_000973.2	RPL21	60S ribosomal protein L21 [Homo sapiens]	4	8	2.37228322	11.85483179	43.175366	26.25	9.95E-05	160	18.553	10.492	Ribosomal subunit	Protein metabolism	Structural constituent of ribosome	Ribosome	Nucleolus																								
18104948			gTWVQLk	Unambiguous	N-Term(iTRAQ4plex); K7(iTRAQ4plex)	HCD	2.209513	0	1	1	2.13233	2.2618	2.1602	2.2913814	3.484599791	1505063.1	1418892	3209294.2	3251222.8	4.167791027	0.917821202	2	High	2434	Unique	Used	50069	1	29.809	1	2.9149	-0.034	1	2	12-Dec	8.451432	2434	1119.68	MS2	560.342	296	Glioma_grade2_2.raw
18104948			kGDIVDlk	Unambiguous	N-Term(iTRAQ4plex); K1(iTRAQ4plex); K8(iTRAQ4plex)	HCD	2.2442677	1	1	1	2.12405	2.3256	2.17	2.375887	5.373759233	1053024.6	961778	2236680.5	2285075.8	6.404705813	1.513599696	2	High	1285	Unique	Used	89189	1	20.962	1	2.9482	-2.6254	1	4	13/14	13.25172	1285	1319.82	MS2	660.415	159	Glioma_grade2_7rep.raw
18104948			kGDIVDlk	Unambiguous	N-Term(iTRAQ4plex); K1(iTRAQ4plex); K8(iTRAQ4plex)	HCD	2.8823996	1	1	1	2.65346	2.4783	3.3151	3.0962248	13.40122878	622828.7	666861.3	1652649.4	2064752.6	4.828408385	15.67766775	3	High	1284	Unique	Used	89188	1	20.959	1	3.7315	-1.5025	1	1	18/28	11.90825	1284	1319.82	MS2	440.613	164	Glioma_grade2_7rep.raw
18104948			gTWVQLk	Unambiguous	N-Term(iTRAQ4plex); K7(iTRAQ4plex)	HCD	2.4821332	0	1	1	2.52038	2.4259	2.5406	2.445325	2.253832602	444440.5	461754.03	1120156.8	1129138.6	2.701961679	0.564718328	2	High	2377	Unique	Used	58057	1	29.236	1	3.1321	-0.1431	1	7	12-Dec	16.2808	2377	1119.68	MS2	560.342	292	Glioma_grade2_3.raw
18104948			hGVVPLATYMR	Unambiguous	N-Term(iTRAQ4plex)	HCD	2.1651797	0	1	1	2.02877	2.2364	2.1006	2.3155556	5.970164759	172855.86	156806.22	350684.12	363093.5	6.885116589	2.458680828	2	High	2246	Unique	Used	72936	1	28.859	1	3.1593	0.7146	1	22	14/20	23.03079	2246	1387.76	MS2	694.386	248	Glioma_grade2_5.raw
18104948			vYNVTQHAVGIVVNk	Unambiguous	N-Term(iTRAQ4plex); K15(iTRAQ4plex)	HCD	2.2624333	0	1	1	1.79973	2.4543	2.1217	2.893431	20.19233697	92261.84	67654.12	166046.69	195752.52	21.76178748	11.61152001	3	High	3475	Unique	Used	74081	1	38.004	1	4.7996	1.6212	1	4	20/56	34.94261	3475	1929.12	MS2	643.711	106	Glioma_grade2_5.raw
18104948			hGVVPLATYMR	Unambiguous	N-Term(iTRAQ4plex)	HCD	2.5530963	0	1	1	2.48949	2.6197	2.4898	2.6200316	2.942277794	42456.05	40346.184	105693.79	105708.27	3.603527306	0.009686666	3	High	2247	Unique	Used	72937	1	28.865	1	3.7762	-0.6365	1	14	17/40	30.97083	2247	1387.76	MS2	463.259	178	Glioma_grade2_5.raw
18104948			vYNVTQHAVGIVVNk	Unambiguous	N-Term(iTRAQ4plex); K15(iTRAQ4plex)	HCD	2.8749166	0	1	1	2.50285	2.8038	2.9384	3.2917173	11.33966597	37617.54	33579.94	94151.15	110535.67	8.019987055	11.3203236	2	High	3450	Unique	Used	74058	1	37.811	1	6.1856	0.1095	1	45	21/28	23.872	3450	1929.12	MS2	965.062	118	Glioma_grade2_5.raw
18375623	NP_542165.1	DDX39B	spliceosome RNA helicase DDX39B [Homo sapiens]	3	3	2.127487898	9.080426892	13.928139	7.71	1.90E-04	428	48.96	5.6685	RNA binding protein	Regulation of nucleobase, nucleoside, nucleotide and nucleic acid metabolism	RNA binding	Nucleus	Nucleolus																								
18375623			dFLLkPELLR	Unambiguous	N-Term(iTRAQ4plex); K5(iTRAQ4plex)	HCD	1.8217753	0	1	2	1.77357	1.9009	1.748	1.8734361	4.087072391	80481.29	75092.15	142739.5	140680.34	4.898904901	1.027483467	2	High	4907	Unique	Used	81992	1	51.248	1	3.8728	-1.17	1	45	14/18	35.8409	4907	1531.94	MS2	766.475	84	Glioma_grade2_6.raw
18375623			lTLHGLQQYYVk	Unambiguous	N-Term(iTRAQ4plex); K12(iTRAQ4plex)	HCD	2.127488	0	1	2	2.06508	2.2475	2.0173	2.1954048	5.067197805	42528.695	39077.734	87825.26	85791.445	5.98040609	1.656665905	3	High	4245	Unique	Used	81426	1	45.403	1	5.5338	-2.3319	1	17	20/44	14.07307	4245	1751.01	MS2	584.34	190	Glioma_grade2_6.raw
18375623			vAVFFGGLSlk	Unambiguous	N-Term(iTRAQ4plex); K11(iTRAQ4plex)	HCD	2.1543438	0	1	1	2.00414	2.2734	2.0494	2.3247187	7.381209042	29112.535	25665.082	58345.727	59664.098	8.900414175	1.57991773	2	High	6841	Unique	Used	69852	1	63.478	1	4.5215	-1.4204	1	100	18/20	62.74428	6841	1425.87	MS2	713.438	118	Glioma_grade2_4.raw
18379349	NP_006364.2	VAT1	synaptic vesicle membrane protein VAT-1 homolog [Homo sapiens]	4	7	2.967602968	22.49448877	33.348454	19.08	6.25E-05	393	41.893	6.2905	Transport/cargo protein	Transport	Transporter activity	Cytoplasmic vesicle	-																								
18379349			vLLVPGPEk	Unambiguous	N-Term(iTRAQ4plex); K9(iTRAQ4plex)	HCD	3.8098042	0	1	1	3.89333	3.8572	3.762	3.7270577	2.053233626	298138.1	300930.34	1160748.6	1121584.8	0.659160692	2.42672596	2	High	3101	Unique	Used	66409	1	33.906	1	2.9688	6.8132	1	4	16-Dec	15.00062	3101	1239.8	MS2	620.404	70	Glioma_grade2_4.raw
18379349			gYNLLkPMGk	Unambiguous	N-Term(iTRAQ4plex); K6(iTRAQ4plex); K10(iTRAQ4plex)	HCD	3.8205152	0	1	1	3.59349	3.8741	3.7708	4.0652637	5.154342022	128929.78	119591.38	463308.38	486170.5	5.31403118	3.405227947	3	High	2590	Unique	Used	90331	1	31.804	1	3.25	-2.5783	1	6	22/36	18.66221	2590	1552.92	MS2	518.312	127	Glioma_grade2_7rep.raw
18379349			gYNLLkPMGk	Unambiguous	N-Term(iTRAQ4plex); K6(iTRAQ4plex); K10(iTRAQ4plex)	HCD	3.0105772	0	1	1	2.82882	3.0873	2.9403	3.2089381	5.517063352	114906.73	105286.09	325050.97	337856.56	6.178966038	2.731880124	2	High	2564	Unique	Used	90307	1	31.606	1	4.1418	-1.3906	1	26	15/18	16.64735	2564	1552.92	MS2	776.965	159	Glioma_grade2_7rep.raw
18379349			gYNLLkPMGk	Unambiguous	N-Term(iTRAQ4plex); K6(iTRAQ4plex); K10(iTRAQ4plex)	HCD	2.967603	0	1	1	2.88003	2.9973	2.9391	3.0587468	2.585059632	61130.645	58738.504	176057.83	179666.22	2.822242648	1.434548515	2	High	2546	Unique	Used	90291	1	31.46	1	3.7926	11.737	1	69	15/18	18.26027	2546	1552.94	MS2	776.975	120	Glioma_grade2_7rep.raw
18379349			vVTYGMANLLTGPk	Unambiguous	N-Term(iTRAQ4plex); K14(iTRAQ4plex)	HCD	2.737875	0	1	1	2.5694	2.8433	2.6426	2.9243095	6.064115546	36643.855	33114.312	94152.64	96836.5	7.15547411	1.987312583	2	High	5931	Unique	Used	61359	1	56.64	1	6.0916	2.1693	1	71	21/26	25.16634	5931	1752	MS2	876.504	183	Glioma_grade2_3.raw
18379349			gVDIVMDPLGGSDTAk	Unambiguous	N-Term(iTRAQ4plex); K16(iTRAQ4plex)	HCD	1.9990855	0	1	1	1.84642	2.0933	1.916	2.1721606	7.545597483	28324.97	24984.227	52299.895	54269.75	8.862493387	2.614061121	2	High	5448	Unique	Used	52905	1	51.691	1	4.6915	1.0834	1	54	22/30	27.52878	5448	1862.98	MS2	931.993	233	Glioma_grade2_2.raw
18379349			gVDIVmDPLGGSDTAk	Unambiguous	N-Term(iTRAQ4plex); M6(Oxidation); K16(iTRAQ4plex)	HCD	2.4638326	0	1	1	2.33181	2.3674	2.5588	2.5978673	5.430396737	11208.961	11040.409	26137.209	28681.518	1.071349545	6.563808559	2	High	4123	Unique	Used	51659	1	41.995	1	4.5937	-2.1965	1	100	20/30	14.07738	4123	1878.97	MS2	939.988	210	Glioma_grade2_2.raw
18426911	NP_542970.1	SIRPA	tyrosine-protein phosphatase non-receptor type substrate 1 precursor [Homo sapiens]	8	26	0.378529012	38.1884354	184.4599	24.8	2.61E-04	504	54.933	6.9761	Cell surface receptor	Cell communication,Signal transduction	Receptor activity	Plasma membrane	-																								
18426911			vTTVSDLTk	Unambiguous	N-Term(iTRAQ4plex); K9(iTRAQ4plex)	HCD	0.63707906	0	1	8	0.6144	0.6046	0.6701	0.6593911	0.050941331	849218	863029.75	521763.06	569074.1	1.140767402	6.133629919	2	High	1665	Unique	Used	49341	1	24.475	1	3.4561	-2.4968	1	6	16/16	25.77195	1665	1251.74	MS2	626.372	259	Glioma_grade2_2.raw
18426911			lTcQVEHDGQPAVSk	Unambiguous	N-Term(iTRAQ4plex); C3(Methylthio); K15(iTRAQ4plex)	HCD	0.26563814	0	1	4	0.24879	0.2677	0.2638	0.2837604	0.054015165	555379.3	516229.56	138174.67	146485.52	5.166632653	4.128893747	2	High	2345	Unique	Used	73029	1	29.576	1	5.8172	11.936	1	7	21/28	8.154161	2345	1945.99	MS2	973.501	187	Glioma_grade2_5.raw
18426911			qPAPkPEPSFSEYASVQVPR	Unambiguous	N-Term(iTRAQ4plex); K5(iTRAQ4plex)	HCD	0.18423848	0	1	1	0.16639	0.1727	0.1954	0.20276374	0.094959704	236256.47	227634.77	39310.543	46156.08	2.628401664	11.32728886	3	High	3775	Unique	Used	74330	1	40.781	1	5.2133	0.5762	1	6	29/76	6.97941	3775	2502.32	MS2	834.78	80	Glioma_grade2_5.raw
18426911			qPAPkPEPSFSEYASVQVPR	Unambiguous	N-Term(iTRAQ4plex); K5(iTRAQ4plex)	HCD	0.21080922	0	1	1	0.21822	0.2037	0.2184	0.20389317	0.039626373	190729.77	204284.81	41620.434	41652.277	4.852914899	0.054078703	3	High	3550	Unique	Used	80834	1	39.198	1	5.9332	-0.0824	1	10	27/76	7.870466	3550	2502.32	MS2	834.779	105	Glioma_grade2_6.raw
18426911			lTcQVEHDGQPAVSk	Unambiguous	N-Term(iTRAQ4plex); C3(Methylthio); K15(iTRAQ4plex)	HCD	0.350131	0	1	4	0.31811	0.3878	0.3192	0.3891661	0.114033565	130887.13	107362.266	41636.746	41781.75	13.96401514	0.245828723	3	High	2703	Unique	Used	66037	1	30.902	1	6.0347	-1.4004	1	12	26/56	21.67284	2703	1945.97	MS2	649.328	140	Glioma_grade2_4.raw
18426911			lTcQVEHDGQPAVSk	Unambiguous	N-Term(iTRAQ4plex); C3(Methylthio); K15(iTRAQ4plex)	HCD	0.37259877	0	1	4	0.39866	0.4034	0.3421	0.34622526	0.088383808	129904.13	128367.87	51787.63	44444.2	0.841206065	10.79183291	3	High	2264	Unique	Used	72953	1	28.985	1	5.5329	-0.1772	1	13	23/56	32.72316	2264	1945.97	MS2	649.329	140	Glioma_grade2_5.raw
18426911			lTcQVEHDGQPAVSk	Unambiguous	N-Term(iTRAQ4plex); C3(Methylthio); K15(iTRAQ4plex)	HCD	0.32919958	0	1	4	0.31423	0.337	0.3219	0.34524977	0.042700532	124042.445	115667.91	38978.23	39934.316	4.940704783	1.713428164	2	High	2284	Unique	Used	72972	1	29.126	1	6.746	-0.7976	1	43	22/28	5.238815	2284	1945.97	MS2	973.489	145	Glioma_grade2_5.raw
18426911			cTATSLIPVGPIQWFR	Unambiguous	N-Term(iTRAQ4plex); C1(Methylthio)	HCD	0.63550556	0	1	1	0.58819	0.6365	0.6345	0.68671006	0.063226718	122921.9	113584.06	72301.53	77999.32	5.583664772	5.361175199	2	High	5980	Unique	Used	88137	1	76.098	1	5.3951	0.9966	1	27	21/30	22.36383	5980	1979.04	MS2	990.023	204	Glioma_grade2_1rep.raw
18426911			kGSPDDVEFk	Unambiguous	N-Term(iTRAQ4plex); K1(iTRAQ4plex); K10(iTRAQ4plex)	HCD	0.66502815	1	1	3	0.68069	0.6888	0.6416	0.64918166	0.034847218	114289.75	112947.51	77795.72	73323.45	0.835344526	4.185269737	3	High	1114	Unique	Used	89037	1	19.611	1	4.5261	-4.0655	1	9	19/36	50.07946	1114	1553.85	MS2	518.621	244	Glioma_grade2_7rep.raw
18426911			aTPQHTVSFTcESHGFSPR	Unambiguous	N-Term(iTRAQ4plex); C11(Methylthio)	HCD	0.3633157	0	1	4	0.37772	0.4147	0.3166	0.34750047	0.115193302	89254.19	81304.98	33713.305	28253.516	6.591190959	12.46039014	3	High	2509	Unique	Used	79921	1	30.421	1	7.7687	7.5474	1	13	30/72	12.03698	2509	2279.06	MS2	760.359	67	Glioma_grade2_6.raw
18426911			lTcQVEHDGQPAVSk	Unambiguous	N-Term(iTRAQ4plex); C3(Methylthio); K15(iTRAQ4plex)	HCD	0.26312873	0	1	4	0.22944	0.2995	0.2352	0.30710906	0.153716793	60803.527	46573.164	13950.851	14303.041	18.74221692	1.762843415	2	High	2729	Unique	Used	66062	1	31.075	1	6.8001	-2.4913	1	100	20/28	19.81982	2729	1945.97	MS2	973.487	142	Glioma_grade2_4.raw
18426911			lTcQVEHDGQPAVSk	Unambiguous	N-Term(iTRAQ4plex); C3(Methylthio); K15(iTRAQ4plex)	HCD	0.3844593	0	1	4	0.37667	0.4436	0.3343	0.39363652	0.116824251	54510.76	46288.28	20532.371	18220.758	11.53616417	8.435743229	3	High	2811	Unique	Used	66138	1	31.71	1	5.5162	2.2693	1	27	22/56	28.28265	2811	1945.98	MS2	649.33	157	Glioma_grade2_4.raw
18426911			eDVHSQVlcEVAHVTLQGDPLR	Unambiguous	N-Term(iTRAQ4plex); C9(Methylthio)	HCD	0.3853192	0	1	4	0.43862	0.434	0.3361	0.33258212	0.152870531	41066.32	41504.91	18012.49	13803.791	0.751181648	18.7074008	3	High	6130	Unique	Used	76432	1	60.069	1	7.4057	0.424	1	6	35/84	6.178385	6130	2635.31	MS2	879.108	102	Glioma_grade2_5.raw
18426911			aTPQHTVSFTcESHGFSPR	Unambiguous	N-Term(iTRAQ4plex); C11(Methylthio)	HCD	0.51479506	0	1	4	0.51175	0.5987	0.4431	0.51835626	0.12295308	34441.562	29439.188	17625.506	15259.986	11.07442407	10.17272439	4	High	2508	Unique	Used	79920	1	30.418	1	5.5723	5.8275	1	6	29/108	22.0079	2508	2279.06	MS2	570.52	64	Glioma_grade2_6.raw
18426911			akPSAPVVSGPAAR	Unambiguous	N-Term(iTRAQ4plex); K2(iTRAQ4plex)	HCD	0.47998384	0	1	4	0.38315	0.4931	0.4698	0.60460407	0.186994713	34256.09	26618.021	13125.226	16093.364	17.74458894	14.36613134	3	High	1006	Unique	Used	78528	1	19.181	1	5.7236	-5.0348	1	41	23/52	37.44525	1006	1595.94	MS2	532.651	84	Glioma_grade2_6.raw
18426911			aTPQHTVSFTcESHGFSPR	Unambiguous	N-Term(iTRAQ4plex); C11(Methylthio)	HCD	0.38735595	0	1	4	0.38799	0.3348	0.4482	0.38680932	0.11897908	31917.977	36983.387	12383.835	14305.519	10.39685008	10.1826053	2	High	2557	Unique	Used	79966	1	30.776	1	4.218	7.3926	1	36	21/36	0	2557	2279.06	MS2	1140.03	81	Glioma_grade2_6.raw
18426911			qPAPkPEPSFSEYASVQVPR	Unambiguous	N-Term(iTRAQ4plex); K5(iTRAQ4plex)	HCD	0.23469937	0	1	1	0.22599	0.2297	0.2396	0.24355476	0.035129782	29349.424	28877.715	6632.568	7033.3047	1.145680995	4.147025869	2	High	3563	Unique	Used	80845	1	39.308	1	4.0963	1.7036	1	100	19/38	0	3563	2502.33	MS2	1251.67	91	Glioma_grade2_6.raw
18426911			qPAPkPEPSFSEYASVQVPR	Unambiguous	N-Term(iTRAQ4plex); K5(iTRAQ4plex)	HCD	0.27610746	0	1	1	0.24284	0.2783	0.2742	0.3142277	0.105340647	29168.057	25454.594	7083.182	7998.5386	9.614380924	8.583302612	3	High	3701	Unique	Used	74268	1	40.096	1	5.145	3.2836	1	79	30/76	20.51121	3701	2502.33	MS2	834.782	102	Glioma_grade2_5.raw
18426911			qPAPkPEPSFSEYASVQVPR	Unambiguous	N-Term(iTRAQ4plex); K5(iTRAQ4plex)	HCD	0.20548989	0	1	1	0.20599	0.2147	0.1967	0.20496581	0.035899952	18861.56	18096.629	3885.344	3709.1902	2.927025982	3.280241901	2	High	3792	Unique	Used	74345	1	40.919	1	3.2143	1.5084	1	66	17/38	6.732311	3792	2502.33	MS2	1251.67	66	Glioma_grade2_5.raw
18426911			eDVHSQVlcEVAHVTLQGDPLR	Unambiguous	N-Term(iTRAQ4plex); C9(Methylthio)	HCD	0.58144796	0	1	4	0.42656	0.5918	0.574	0.7963289	0.254585558	18548.727	13370.444	7912.0645	10647.271	22.94294563	20.84215854	3	High	6212	Unique	Used	76503	1	60.777	1	7.3947	0.2156	1	20	36/84	3.909982	6212	2635.31	MS2	879.108	110	Glioma_grade2_5.raw
18426911			eDVHSQVlcEVAHVTLQGDPLR	Unambiguous	N-Term(iTRAQ4plex); C9(Methylthio)	HCD	0.27911857	0	1	4	0.2977	0.3118	0.2479	0.2596556	0.10876124	9676.525	9238.476	2880.7058	2398.8218	3.27515096	12.90810353	2	High	6105	Unique	Used	76410	1	59.857	1	6.1154	-0.1267	1	59	23/42	0	6105	2635.31	MS2	1318.16	130	Glioma_grade2_5.raw
18426911			eDVHSQVlcEVAHVTLQGDPLR	Unambiguous	N-Term(iTRAQ4plex); C9(Methylthio)	HCD	0.5103016	0	1	4	0.46463	0.4709	0.5491	0.55659205	0.096559468	9387.991	9262.074	4361.9604	5155.1973	0.954814523	11.78719968	3	High	6058	Unique	Used	76370	1	59.439	1	7.0403	-0.2013	1	62	33/84	28.12347	6058	2635.31	MS2	879.108	105	Glioma_grade2_5.raw
18426911			qPAPkPEPSFSEYASVQVPR	Unambiguous	N-Term(iTRAQ4plex); K5(iTRAQ4plex)	HCD	0.4185435	0	1	1	0.567	0.4285	0.4053	0.30633917	0.251739448	8934.237	11820.747	5065.705	3621.158	19.66824735	23.51709655	4	High	3573	Unique	Used	80853	1	39.407	1	4.4362	0.4733	1	18	22/114	12.92358	3573	2502.32	MS2	626.337	139	Glioma_grade2_6.raw
18426911			aTPQHTVSFTcESHGFSPR	Unambiguous	N-Term(iTRAQ4plex); C11(Methylthio)	HCD	0.6379914	0	1	4	0.56537	0.6516	0.6262	0.7216877	0.100916014	8073.7773	7005.475	4564.6685	5055.7646	10.01911482	7.21916319	4	High	2743	Unique	Used	80118	1	32.528	1	5.2718	1.6497	1	38	28/108	25.22895	2743	2279.05	MS2	570.518	73	Glioma_grade2_6.raw
18426911			qPAPkPEPSFSEYASVQVPR	Unambiguous	N-Term(iTRAQ4plex); K5(iTRAQ4plex)	HCD	0.32166657	0	1	1	0.36032	0.3628	0.2808	0.2827488	0.143224941	5306.4985	5270.391	1912.0352	1490.1967	0.482785759	17.53465799	4	High	3743	Unique	Used	74303	1	40.489	1	4.9986	-1.8683	1	25	20/114	30.41879	3743	2502.32	MS2	626.335	143	Glioma_grade2_5.raw

18426911			eDVHSQVIcEVAHVTLQGDPLR	Unambiguous	N-Term(iTRAQ4plex); C9(Methylthio)	HCD	0.6597261	0	1	4	0.70551	0.8695	0.4895	0.60330015	0.24179891	1781.1621	1445.302	1256.6317	871.9509	14.72131391	25.55789024	4	High	6308	Unique	Used	76587	1	61.598	1	6.5211	0.002	1	55	29/126	13.70551	6308	2635.31	MS2	659.583	103	Glioma_grade2_5.raw
18641362	NP_563578.1	PTPRC	receptor-type tyrosine-protein phosphatase C isoform 2 precursor [Homo sapiens]	4	4	2.222333908	21.06691989	20.438784	5.16	7.59E-05	1143	130.82	6.3286	Receptor tyrosine phosphatase	Cell communication,Signal transduction	Receptor signaling protein tyrosine phosphatase activity	Plasma membrane	Mitochondrion																								
18641362			eLISMIQVVk	Unambiguous	N-Term(iTRAQ4plex); K10(iTRAQ4plex)	HCD	2.743241	0	1	2	2.64981	2.8294	2.6625	2.8430083	3.79511094	41058.86	38452.555	108798.04	109320.93	4.635651219	0.339025134	2	High	6379	Unique	Used	53784	1	58.447	1	4.0445	-2.3683	1	33	18/18	33.07113	6379	1447.88	MS2	724.442	175	Glioma_grade2_2.raw
18641362			fQLHDcTQVEk	Unambiguous	N-Term(iTRAQ4plex); C6(Methylthio); K11(iTRAQ4plex)	HCD	2.49689	0	1	2	2.37236	2.4815	2.5117	2.6271508	4.193658205	32445.67	31019.236	76972.69	81492.21	3.178579211	4.033427263	3	High	2987	Unique	Used	80323	1	34.759	1	5.274	-0.6296	1	23	19/40	16.27217	2987	1681.83	MS2	561.281	130	Glioma_grade2_6.raw
18641362			yHLEVEAGNTLVR	Unambiguous	N-Term(iTRAQ4plex)	HCD	1.947778	0	1	2	1.92049	1.8985	1.9976	1.974751	2.369335975	21327.975	21575.287	40960.207	42605.82	0.815210705	2.784921465	2	High	3096	Unique	Used	58729	1	34.576	1	3.4845	3.9963	1	100	13/24	0	3096	1644.89	MS2	822.948	247	Glioma_grade2_3.raw
18641362			sTPLLIHcR	Unambiguous	N-Term(iTRAQ4plex); C8(Methylthio)	HCD	1.7321739	0	1	2	1.91311	1.5992	1.8913	1.5809302	10.34152921	16132.67	19299.39	30863.502	30510.988	12.63945244	0.812275719	3	High	2368	Unique	Used	73051	1	29.729	1	3.2587	11.365	1	33	19/32	27.74665	2368	1229.68	MS2	410.563	235	Glioma_grade2_5.raw
18765733	NP_003072.2	SNAP25	synaptosomal-associated protein 25 isoform SNAP25A [Homo sapiens]	2	4	0.501970291	11.12837649	101.78795	42.23	9.69E-05	206	23.321	4.856	Membrane transport protein	Transport	Auxiliary transport protein activity	Plasma membrane	Cytoplasm, Nucleus																								
18765733			vEEGMNHINQDMk	Unambiguous	N-Term(iTRAQ4plex); K13(iTRAQ4plex)	HCD	0.43124563	0	1	1	0.36214	0.3631	0.4992	0.5005362	0.183791206	129784.68	129446.734	46999.633	64792.78	0.184363388	22.50896025	3	High	1481	Unique	Used	72235	1	23.043	1	4.0228	5.1762	1	5	22/48	11.15571	1481	1832.9	MS2	611.637	105	Glioma_grade2_5.raw
18765733			vEEGMNHINQDMk	Unambiguous	N-Term(iTRAQ4plex); K13(iTRAQ4plex)	HCD	0.5041202	0	1	1	0.49961	0.5113	0.4971	0.50874007	0.013635133	122522.28	119725.805	61212.83	60909.316	1.632546599	0.351478933	2	High	1457	Unique	Used	72213	1	22.861	1	6.2964	0.5216	1	90	21/24	35.43025	1457	1832.89	MS2	916.948	114	Glioma_grade2_5.raw
18765733			rADQLADESLESTR	Unambiguous	N-Term(iTRAQ4plex)	HCD	0.56761575	1	1	1	0.56686	0.6068	0.531	0.56842273	0.054497192	45759.66	42746.652	25939.41	24298.17	4.81438745	4.620174513	3	High	1577	Unique	Used	57311	1	23.337	1	5.0109	-0.2161	1	16	25/52	26.46866	1577	1734.87	MS2	578.963	177	Glioma_grade2_3.raw
18765733			rADQLADESLESTR	Unambiguous	N-Term(iTRAQ4plex)	HCD	0.49982038	1	1	1	0.47482	0.5302	0.4726	0.52774477	0.063743715	42808.094	38332.934	20325.932	20230.006	7.799792684	0.334500586	2	High	1579	Unique	Used	57313	1	23.348	1	4.2116	0.847	1	30	19/26	9.704196	1579	1734.88	MS2	867.941	122	Glioma_grade2_3.raw
19743823	NP_596867.1	ITGB1	integrin beta-1 isoform 1A precursor [Homo sapiens]	3	5	2.335299253	18.61473703	47.125806	7.77	1.43E-04	798	88.357	5.3892	Cell surface receptor	Immune cell migration,Cell communication	Receptor activity	Plasma membrane	Cell surface																								
19743823			iGFGSFVEk	Unambiguous	N-Term(iTRAQ4plex); K9(iTRAQ4plex)	HCD	1.8233109	0	1	2	1.63113	1.8866	1.7686	2.0455902	9.60348498	312693.06	270356.22	510042.06	553038	10.26900047	5.719742451	2	High	4930	Unique	Used	52418	1	47.892	1	3.102	-2.907	1	22	16/16	33.50738	4930	1271.72	MS2	636.364	201	Glioma_grade2_2.raw
19743823			iGFGSFVEk	Unambiguous	N-Term(iTRAQ4plex); K9(iTRAQ4plex)	HCD	3.0077608	0	1	2	2.91172	2.9903	3.0248	3.1063952	2.683326416	243095.2	236709.6	707824.5	735313.56	1.882140846	2.693810284	2	High	5005	Unique	Used	68172	1	48.771	1	2.9965	1.3165	1	16	16/16	19.94049	5005	1271.73	MS2	636.366	222	Glioma_grade2_4.raw
19743823			iGFGSFVEk	Unambiguous	N-Term(iTRAQ4plex); K9(iTRAQ4plex)	HCD	2.1498034	0	1	2	2.09793	2.1749	2.1256	2.2035823	2.212804632	52089.773	50247.168	109280.54	110723.77	2.546330734	0.927725207	2	High	4792	Unique	Used	60304	1	47.725	1	3.1481	-3.7709	1	100	16/16	15.62554	4792	1271.72	MS2	636.363	200	Glioma_grade2_3.raw
19743823			lkPEDITQIQPQQLVLR	Unambiguous	N-Term(iTRAQ4plex); K2(iTRAQ4plex)	HCD	2.3352993	0	1	2	2.57727	2.0167	2.7424	2.1459546	14.54418461	30350.016	38785.43	78220.18	83231.766	17.25522517	4.389821904	3	High	5166	Unique	Used	82217	1	53.468	1	6.2236	2.7282	1	6	25/64	6.440424	5166	2307.37	MS2	769.795	33	Glioma_grade2_6.raw
19743823			dkLPQPVQPDPVSHck	Unambiguous	N-Term(iTRAQ4plex); K2(iTRAQ4plex); C15(Methylthio); K16(iTRAQ4plex)	HCD	2.5137727	1	1	2	2.36252	2.6056	2.4306	2.6805816	5.882093688	2530.3213	2294.3093	5977.942	6150.0835	6.918071018	2.007291656	4	High	2573	Unique	Used	95216	1	36.517	1	6.4444	-0.0612	1	84	25/90	35.7488	2573	2266.2	MS2	567.307	157	Glioma_grade2_8rep.raw
19913416	NP_570603.2	AP2A1	AP-2 complex subunit alpha-1 isoform 2 [Homo sapiens]	14	25	0.460578591	19.56083398	211.77312	29.74	1.49E-04	955	105.29	7.6646	Transport/cargo protein	Transport	Transporter activity	Plasma membrane	Mitochondrion																								
19913416			tHIDTVINALk	Unambiguous	N-Term(iTRAQ4plex); K11(iTRAQ4plex)	HCD	0.45974416	0	1	2	0.43376	0.4634	0.4563	0.48750225	0.04809217	869241.94	813631.25	377044.06	396647.06	4.673280939	3.583190726	2	High	3331	Unique	Used	73945	1	36.964	1	4.8322	3.3981	1	9	19/20	29.68821	3331	1512.9	MS2	756.956	111	Glioma_grade2_5.raw
19913416			yGGAPQALTLk	Unambiguous	N-Term(iTRAQ4plex); K11(iTRAQ4plex)	HCD	0.32340994	0	1	2	0.28517	0.2984	0.3474	0.36342293	0.116513053	650851.75	622067.5	185601.17	226073.61	3.197930798	13.90337138	2	High	2832	Unique	Used	50444	1	32.624	1	3.1824	0.5386	1	7	16/20	42.17337	2832	1406.83	MS2	703.916	189	Glioma_grade2_2.raw
19913416			lVEcLETVLNk	Unambiguous	N-Term(iTRAQ4plex); C4(Methylthio); K11(iTRAQ4plex)	HCD	0.3420987	0	1	2	0.33022	0.3304	0.3538	0.35398167	0.039844993	485945.47	485730	160469.39	171939.52	0.031360326	4.879897295	2	High	8646	Unique	Used	55861	1	76.392	1	5.1761	-1.2237	1	13	17/20	28.79003	8646	1594.88	MS2	797.942	246	Glioma_grade2_2.raw
19913416			lVEcLETVLNk	Unambiguous	N-Term(iTRAQ4plex); C4(Methylthio); K11(iTRAQ4plex)	HCD	0.34268025	0	1	2	0.30782	0.3499	0.3364	0.38230273	0.089873515	483452.6	425350.22	148816.22	162612.55	9.041474343	6.264982197	2	High	8347	Unique	Used	63559	1	76.393	1	5.0919	0.2305	1	12	17/20	24.67993	8347	1594.88	MS2	797.943	233	Glioma_grade2_3.raw
19913416			iLVAGDSMDSVk	Unambiguous	N-Term(iTRAQ4plex); K12(iTRAQ4plex)	HCD	0.6411027	0	1	2	0.59242	0.6451	0.6374	0.69412124	0.064858645	344934.4	316748.47	204344.8	219861.83	6.024173555	5.173043682	2	High	3599	Unique	Used	51164	1	38.19	1	4.8206	-4.4433	1	26	20/22	30.62574	3599	1522.83	MS2	761.92	271	Glioma_grade2_2.raw
19913416			lVEcLETVLNk	Unambiguous	N-Term(iTRAQ4plex); C4(Methylthio); K11(iTRAQ4plex)	HCD	0.39455333	0	1	2	0.39833	0.3269	0.4771	0.39145264	0.15441482	286548.3	349215.06	114141.41	136701.16	13.93980646	12.71885566	2	High	8270	Unique	Used	71091	1	76.215	1	5.1977	-1.2237	1	18	17/20	7.982626	8270	1594.88	MS2	797.942	246	Glioma_grade2_4.raw
19913416			mYLFYGNk	Unambiguous	N-Term(iTRAQ4plex); K8(iTRAQ4plex)	HCD	0.37594926	0	1	2	0.35998	0.3674	0.3843	0.39225066	0.039534162	279436.9	273766.06	100591.32	107384.91	1.449699191	4.619562816	2	High	4367	Unique	Used	59912	1	44.358	1	3.5933	-0.8239	1	41	13/14	26.28155	4367	1323.7	MS2	662.354	194	Glioma_grade2_3.raw
19913416			iLVAGDSmDSVk	Unambiguous	N-Term(iTRAQ4plex); M8(Oxidation); K12(iTRAQ4plex)	HCD	0.5537476	0	1	2	0.54769	0.5816	0.5275	0.5601782	0.040846716	271847	256011.23	148888.33	143411.92	4.242646876	2.649608851	2	High	1955	Unique	Used	49616	1	26.466	1	3.8609	0.0179	1	23	19/22	14.29162	1955	1538.83	MS2	769.921	242	Glioma_grade2_2.raw
19913416			tHIDTVINALk	Unambiguous	N-Term(iTRAQ4plex); K11(iTRAQ4plex)	HCD	0.4739576	0	1	2	0.45924	0.5546	0.4072	0.49172828	0.129019255	210920.92	174660.33	96863.734	85885.42	13.29945846	8.495623761	3	High	3330	Unique	Used	73944	1	36.958	1	5.4024	3.2076	1	6	18/40	31.59032	3330	1512.9	MS2	504.973	118	Glioma_grade2_5.raw
19913416			qLSLPQQEAQk	Unambiguous	N-Term(iTRAQ4plex); K11(iTRAQ4plex)	HCD	0.5196131	0	1	2	0.50594	0.4994	0.54	0.53311396	0.038386344	190080.14	192551.58	96168.52	102651.93	0.913448568	4.611661603	2	High	1563	Unique	Used	49245	1	23.75	1	4.8178	0.8885	1	55	17/20	0	1563	1557.89	MS2	779.446	217	Glioma_grade2_2.raw
19913416			iLVAGDSMDSVk	Unambiguous	N-Term(iTRAQ4plex); K12(iTRAQ4plex)	HCD	0.6220516	0	1	2	0.61372	0.6126	0.6315	0.6303631	0.016563833	158137.83	158435.36	97053.05	99871.805	0.132914275	2.024285634	2	High	3517	Unique	Used	51089	1	37.545	1	5.0364	-0.5154	1	86	20/22	18.56895	3517	1522.84	MS2	761.923	254	Glioma_grade2_2.raw
19913416			dFLTPPLLSVR	Unambiguous	N-Term(iTRAQ4plex)	HCD	0.47621885	0	1	2	0.43816	0.4837	0.4694	0.5182375	0.069598239	140292.88	127072.22	61470.71	65853.586	6.992998217	4.868138192	2	High	4747	Unique	Used	87215	1	62.722	1	3.5316	1.334	1	46	14/20	39.31718	4747	1401.82	MS2	701.416	188	Glioma_grade2_1rep.raw
19913416			mYLFYGNk	Unambiguous	N-Term(iTRAQ4plex); K8(iTRAQ4plex)	HCD	0.40246382	0	1	2	0.39908	0.3917	0.4135	0.40578347	0.023158099	136613.75	139204.58	54519.984	56486.914	1.328405884	2.50584345	2	High	4526	Unique	Used	52039	1	44.949	1	3.3336	3.6026	1	61	14-Nov	51.7607	4526	1323.71	MS2	662.357	243	Glioma_grade2_2.raw
19913416			fHLcSVATR	Unambiguous	N-Term(iTRAQ4plex); C4(Methylthio)	HCD	0.3403254	0	1	2	0.31541	0.3367	0.3438	0.36691618	0.062320303	134086.27	125622.77	42292.55	46093.027	4.608694593	6.080953815	3	High	2665	Unique	Used	73331	1	31.868	1	3.3768	10.659	1	24	16/32	16.98863	2665	1223.63	MS2	408.547	184	Glioma_grade2_5.raw
19913416			yGGAPQALTLk	Unambiguous	N-Term(iTRAQ4plex); K11(iTRAQ4plex)	HCD	0.36381143	0	1	2	0.36611	0.3423	0.3868	0.3616577	0.050097937	120437.12	128806.22	44093.812	46583.76	4.748650345	3.883339787	2	High	2741	Unique	Used	50360	1	31.928	1	3.6392	1.0592	1	33	16/20	38.22736	2741	1406.83	MS2	703.917	192	Glioma_grade2_2.raw
19913416			aADLLYAMcDR	Unambiguous	N-Term(iTRAQ4plex); C9(Methylthio)	HCD	0.4767678	0	1	2	0.4641	0.4868	0.4672	0.49006045	0.027835407	115407.87	110026.03	53560.22	53919.402	3.376187485	0.472610571	2	High	5424	Unique	Used	87719	1	70.094	1	4.1696	2.8442	1	65	17/20	40.37093	5424	1431.66	MS2	716.333	75	Glioma_grade2_1rep.raw
19913416			fFQPTEMAAQDFFQR	Unambiguous	N-Term(iTRAQ4plex)	HCD	0.43514523	0	1	2	0.40815	0.4454	0.4258	0.46460605	0.055982564	91302.54	83668.25	37264.926	38872.773	6.170467915	2.98648247	2	High	5314	Unique	Used	87638	1	68.864	1	4.7276	-0.455	1	68	18/28	21.8009	5314	2006.95	MS2	1003.98	127	Glioma_grade2_1rep.raw
19913416			fINLFPETk	Unambiguous	N-Term(iTRAQ4plex); K9(iTRAQ4plex)	HCD	0.5584927	0	1	2	0.56193	0.5106	0.6112	0.55537	0.073582559	87423.01	96214.01	49125.562	53434.37	6.770068163	5.941476942	2	High	6115	Unique	Used	69193	1	57.522	1	3.4313	1.8436	1	86	14/16	41.11263	6115	1396.81	MS2	698.909	225	Glioma_grade2_4.raw
19913416			fFQPTEmAAQDFFQR	Unambiguous	N-Term(iTRAQ4plex); M7(Oxidation)	HCD	0.4605786	0	1	2	0.41795	0.4659	0.4558	0.5081039	0.080228206	73772.695	66173.76	30833.197	33623.15	7.679020477	6.121335686	2	High	4983	Unique	Used	87392	1	65.251	1	4.4055	-0.11	1	90	17/28	28.01747	4983	2022.95	MS2	1011.98	108	Glioma_grade2_1rep.raw
19913416			sSPPVQFSLLHSk	Unambiguous	N-Term(iTRAQ4plex); K13(iTRAQ4plex)	HCD	0.5075771	0	1	2	0.51071	0.4986	0.5168	0.504514	0.015495844	48937.906	50130.496	24993.32	25291.535	1.70243682	0.83870123	3	High	2721	Unique	Used	80103	1	32.272	1	6.7423	1.6309	1	37	33/48	0	2721	1714.98	MS2	572.33	163	Glioma_grade2_6.raw
19913416			tVFEALQAPAcHENmVk	Unambiguous	N-Term(iTRAQ4plex); C11(Methylthio); M15(Oxidation); K17(iTRAQ4plex)	HCD	0.47029993	0	1	2	0.4323	0.5207	0.4284	0.5160715	0.107276597	41626.684	34557.254	17995.287	17834.014	13.12308611	0.636558508	3	High	4715	Unique	Used	75165	1	48.579	1	6.9992	-2.4186	1	32	28/64	29.66492	4715	2238.09	MS2	746.702	71	Glioma_grade2_5.raw
19913416			lVEcLETVLNk	Unambiguous	N-Term(iTRAQ4plex); C4(Methylthio); K11(iTRAQ4plex)	HCD	0.3548997	0	1	2	0.38134	0.3811	0.3286	0.32846957	0.08570467	21623.188	21634.883	8245.876	7106.401	0.038233854	10.49656021	3	High	8653	Unique	Used	55868	1	76.433	1	3.6635	-2.4568	1	22	22/40	11.86688	8653	1594.87	MS2	532.296	257	Glioma_grade2_2.raw
19913416			tSVQFQNFSPTVVHPGDLQTQLAVQTk	Unambiguous	N-Term(iTRAQ4plex); K27(iTRAQ4plex)	HCD	0.3858411	0	1	2	0.33167	0.4902	0.3152	0.46590346	0.224736086	16142.137	10921.613	5353.8896	5088.4175	27.2797962	3.595318933	3	High	5540	Unique	Used	75905	1	55.234	1	8.7398	-0.043	1	28	33/104	0	5540	3258.74	MS2	1086.92	75	Glioma_grade2_5.raw
19913416			lVEcLETVLNk	Unambiguous	N-Term(iTRAQ4plex); C4(Methylthio); K11(iTRAQ4plex)	HCD	0.46774986	0	1	2	0.48606	0.4549	0.4815	0.4506175	0.038647635	15274.694	16321.714	7424.366	7354.8496	4.686323471	0.665197926	3	High	8273	Unique	Used	71094	1	76.232	1	3.8188	-3.9493	1	34	21/40	15.93463	8273	1594.87	MS2	532.296	272	Glioma_grade2_4.raw
19913416			lVEcLETVLNk	Unambiguous	N-Term(iTRAQ4plex); C4(Methylthio); K11(iTRAQ4plex)	HCD	0.51103073	0	1	2	0.51269	0.589	0.4431	0.5091213	0.116175928	13533.274	11779.279	6938.411	5997.0825	9.799578562	10.29137026	3	High	8359	Unique	Used	63571	1	76.465	1	3.4648	0.1838	1	35	20/40	13.0554	8359	1594.88	MS2	532.298	238	Glioma_grade2_3.raw
19913424	NP_001681.2	ATP6V1A	V-type proton ATPase catalytic subunit A [Homo sapiens]	13	18	0.485551059	15.97142784	135.90936	31.12	1.40E-04	617	68.26	5.5161	Transport/cargo protein	Metabolism,Energy pathways	Transporter activity	Endosome	Secretory vesicle, Plasma membrane, Lysosome																								
19913424			iTLEVAk	Unambiguous	N-Term(iTRAQ4plex); K7(iTRAQ4plex)	HCD	0.50393844	0	1	1	0.45013	0.5099	0.4987	0.5648928	0.093124105	2967206	2619532.2	1335617	1479755	8.800931521	7.240319022	2	High	2392	Unique	Used	50029	1	29.524	1	3.0431	4.7692	1	4	12-Dec	48.35624	2392	1061.69	MS2	531.347	138	Glioma_grade2_2.raw
19913424			hFTEFVPLR	Unambiguous	N-Term(iTRAQ4plex)	HCD	0.49042144	0	1	1	0.47616	0.4667	0.5146	0.5043997	0.046328245	1537703.2	1568928.1	732191.8	791366.94	1.421432825	5.492816479	2	High	2985	Unique	Used	73624	1	34.353	1	3.3073	1.241	1	5	16-Dec	13.87585	2985	1289.71	MS2	645.361	234	Glioma_grade2_5.raw
19913424			eHMGDILYk	Unambiguous	N-Term(iTRAQ4plex); K9(iTRAQ4plex)	HCD	0.48068064	0	1	1	0.39749	0.4834	0.4784	0.58184844	0.155490944	956118.75	786190.1	380050.7	457443.47	13.79292778	13.06873634	3	High	1709	Unique	Used	72447	1	24.725	1	4.1277	5.5885	1	2	19/32	22.63921	1709	1393.75	MS2	465.254	233	Glioma_grade2_5.raw
19913424			aLDEYYDk	Unambiguous	N-Term(iTRAQ4plex); K8(iTRAQ4plex)	HCD	0.63296044	0	1	1	0.61227	0.6283	0.6375	0.65419555	0.027681871	925558.3	901931.8	566689.25	590039.75	1.828350081	2.854825442	2	High	2141	Unique	Used	49791	1	27.783	1	3.6733	-4.6513	1	14	14/14	44.25914	2141	1304.66	MS2	652.831	136	Glioma_grade2_2.raw
19913424			hFTEFVPLR	Unambiguous	N-Term(iTRAQ4plex)	HCD	0.46008033	0	1	1	0.43096	0.4433	0.4764	0.49002948	0.060080661	849637.6	826014.94	366163.12	404771.66	1.993700087	7.082404674	2	High	2904	Unique	Used	73553	1	33.659	1	3.4665	0.2945	1	10	13/16	8.660983	2904	1289.71	MS2	645.36	188	Glioma_grade2_5.raw
19913424			vGHSELVGEIIR	Unambiguous	N-Term(iTRAQ4plex)	HCD	0.37083387	0	1	1	0.36203	0.3804	0.3617	0.38009053	0.028629376	579884.5	551829.6	209932.72	209745.2	3.505798865	0.063189726	2	High	3217	Unique	Used	66517	1	34.785	1	4.581	-1.0118	1	10	20/22	33.95302	3217	1452.83	MS2	726.918	202	Glioma_grade2_4.raw
19913424			vGHSELVGEIIR	Unambiguous	N-Term(iTRAQ4plex)	HCD	0.44976038	0	1	1	0.43441	0.4359	0.4636	0.46515858	0.03756333	524965.75	523192.44	228052.56	243367.45	0.239261504	4.594316042	2	High	2641	Unique	Used	73308	1	31.706	1	3.892	12.852	1	9	19/22	6.85791	2641	1452.85	MS2	726.928	151	Glioma_grade2_5.raw
19913424			hFTEFVPLR	Unambiguous	N-Term(iTRAQ4plex)	HCD	0.40878507	0	1	1	0.3926	0.4472	0.3751	0.42721933	0.079519805	449597.5	394744.38	176511.92	168642.42	9.187513741	3.224399157	3	High	2966	Unique	Used	73608	1	34.177	1	3.5735	######	1	2	19/32	5.713283	2966	1289.71	MS2	430.576	191	Glioma_grade2_5.raw
19913424			wDFTPck	Unambiguous	N-Term(iTRAQ4plex); C6(Methylthio); K7(iTRAQ4plex)	HCD	0.43826187	0	1	1	0.40968	0.4229	0.4531	0.4677644	0.060978597	254420.5	246460.1	104231.61	115285.26	2.247582686	7.121193803	2	High	5470	Unique	Used	60936	1	52.964	1	2.9321	0.4362	1	40	12-Oct	12.30425	5470	1230.59	MS2	615.798	82	Glioma_grade2_3.raw
19913424			eILQEEEDLAEIVQLVGk	Unambiguous	N-Term(iTRAQ4plex); K18(iTRAQ4plex)	HCD	0.43148062	0	1	1	0.42776	0.4603	0.4047	0.43547976	0.05307658	183496.95	170521.83	78493.44	74258.805	5.183225217	3.920517338	2	High	6043	Unique	Used	88194	1	76.59	1	6.131	-4.161	1	12	23/34	9.257195	6043	2343.28	MS2	1172.14	135	Glioma_grade2_1rep.raw
19913424			dFPELTMEVDGk	Unambiguous	N-Term(iTRAQ4plex); K12(iTRAQ4plex)	HCD	0.467904	0	1	1	0.46879	0.4315	0.5075	0.4670864	0.066199121	94382.414	102539.22	44245.746	47894.67	5.857896584	5.600536695	2	High	5623	Unique	Used	53072	1	52.9	1	5.0747	-0.7832	1	81	18/22	19.94971	5623	1668.84	MS2	834.923	167	Glioma_grade2_2.raw
19913424			lAEMPADSGYPAYLGAR	Unambiguous	N-Term(iTRAQ4plex)	HCD	0.61233956	0	1	1	0.56079	0.6268	0.5994	0.6699644	0.074853963	74664.05	66797.28	41870.57	44751.797	7.864547029	4.703947076	2	High	3968	Unique	Used	86671	1	53.841	1	5.0522	-0.6778	1	100	19/32	62.21845	3968	1925.95	MS2	963.48	205	Glioma_grade2_1rep.raw
19913424			dMGYHVSMMADSTSR	Unambiguous	N-Term(iTRAQ4plex)	HCD	0.59763134	0	1	1	0.656	0.6214	0.5725	0.5423402	0.084486296	37869.9	39977.37	24842.633	21681.338	3.82853844	9.609554317	2	High	2491	Unique	Used	50124	1	30.177	1	5.168	0.8893	1	50	24/28	0	2491	1831.79	MS2	916.399	35	Glioma_grade2_2.raw

19913424			lPANHPLLTGQR	Unambiguous	N-Term(iTRAQ4plex)	HCD	0.65890956	0	1	1	0.67195	0.6406	0.6781	0.64647627	0.028086048	37534.195	39371.46	25221.158	25452.715	3.378535793	0.646232527	3	High	1309	Unique	Used	72082	1	21.656	1	3.5351	1.6701	1	20	19/44	23.23169	1309	1460.85	MS2	487.621	241	Glioma_grade 2_5.raw
19913424			hFTEFVPLR	Unambiguous	N-Term(iTRAQ4plex)	HCD	0.5222592	0	1	1	0.56463	0.4875	0.5625	0.48567185	0.084651805	31715.555	36731.766	17907.654	17839.586	10.3641655	0.269287052	3	High	2877	Unique	Used	73528	1	33.453	1	4.4046	-0.7808	1	33	18/32	13.02336	2877	1289.71	MS2	430.575	180	Glioma_grade 2_5.raw
19913424			dMGYHVSMMADSTSR	Unambiguous	N-Term(iTRAQ4plex)	HCD	0.54878265	0	1	1	0.59498	0.5345	0.5647	0.5072852	0.068823405	29992.031	33386.906	17844.58	16936.682	7.575195317	3.691532713	2	High	2456	Unique	Used	58130	1	29.845	1	5.2704	0.6893	1	87	23/28	0	2456	1831.79	MS2	916.399	35	Glioma_grade 2_3.raw
19913424			vGSHITGGDIYGIVSENSLIk	Unambiguous	N-Term(iTRAQ4plex); K21(iTRAQ4plex)	HCD	0.5402962	0	1	1	0.52934	0.5684	0.5141	0.5520632	0.044425382	26557.578	24731.914	14057.937	13653.58	5.033933245	2.063572173	2	High	5650	Unique	Used	76006	1	56.094	1	7.4346	-4.1149	1	100	26/40	11.26897	5650	2447.33	MS2	1224.17	129	Glioma_grade 2_5.raw
19913424			lIkDDFLQQNGYTPYDR	Unambiguous	N-Term(iTRAQ4plex); K3(iTRAQ4plex)	HCD	0.45800343	1	1	1	0.40847	0.4608	0.4556	0.5138806	0.093811564	20152.004	17865.082	8231.4375	9180.52	8.507217277	7.708526415	2	High	4973	Unique	Used	82047	1	51.854	1	3.8489	-5.1888	1	100	16/32	0	4973	2374.22	MS2	1187.61	190	Glioma_grade 2_6.raw
19920317	NP_006816.2	CKAP4	cytoskeleton-associated protein 4 [Homo sapiens]	13	21	3.029812574	32.92043697	129.2964	27.74	8.61E-05	602	65.983	5.9224	Cytoskeletal associated protein	Cell growth and/or maintenance	Cytoskeletal protein binding	Plasma membrane	Cytoplasm, Cytoskeleton, Golgi apparatus, Endoplasmic reticulum																								
19920317			dLSDGIHVVk	Unambiguous	N-Term(iTRAQ4plex); K10(iTRAQ4plex)	HCD	2.65504	0	1	1	2.58642	2.6032	2.7065	2.72411	2.647271092	197138.11	195867.2	509881.06	533563.75	0.457331775	3.209789447	2	High	1712	Unique	Used	72450	1	24.742	1	4.337	4.6513	1	11	18/18	24.76636	1712	1370.79	MS2	685.901	213	Glioma_grade 2_5.raw
19920317			iETNENNLESAk	Unambiguous	N-Term(iTRAQ4plex); K12(iTRAQ4plex)	HCD	4.137046	0	1	1	4.12955	4.0052	4.273	4.1443186	2.646050664	162751.23	167803.97	672088.9	695433.1	2.161712608	2.414124544	2	High	1473	Unique	Used	49159	1	23.157	1	5.8122	-0.253	1	18	20/22	22.20452	1473	1649.86	MS2	825.433	178	Glioma_grade 2_2.raw
19920317			lALQALTEk	Unambiguous	N-Term(iTRAQ4plex); K9(iTRAQ4plex)	HCD	5.046223	0	1	1	4.92336	4.9674	5.1244	5.1701875	2.36662427	160103.72	158685.08	788247.7	820431.6	0.629338273	2.829333844	2	High	4054	Unique	Used	59619	1	41.955	1	3.9457	1.2736	1	13	14/16	17.88072	4054	1274.79	MS2	637.901	160	Glioma_grade 2_3.raw
19920317			hSEAFEALQQk	Unambiguous	N-Term(iTRAQ4plex); K11(iTRAQ4plex)	HCD	2.7516189	0	1	1	2.52118	2.8056	2.7031	3.0080585	7.364064959	141782.36	127409.17	357458.25	383254.25	7.551040047	4.925129933	2	High	1334	Unique	Used	78824	1	21.764	1	4.934	-7.2852	1	30	18/20	35.17791	1334	1575.83	MS2	788.416	182	Glioma_grade 2_6.raw
19920317			vASLEESEGNkQDLk	Unambiguous	N-Term(iTRAQ4plex); K11(iTRAQ4plex); K15(iTRAQ4plex)	HCD	2.7165823	1	1	1	2.69128	2.646	2.7884	2.7414615	2.268804031	116645.99	118642.45	313926.7	325253.72	1.199982799	2.506150815	3	High	1478	Unique	Used	78960	1	22.792	1	3.4733	-6.7869	1	6	23/56	16.40094	1478	2079.12	MS2	693.71	172	Glioma_grade 2_6.raw
19920317			dLSDGIHVVk	Unambiguous	N-Term(iTRAQ4plex); K10(iTRAQ4plex)	HCD	4.4362097	0	1	1	3.84312	4.961	4.0297	5.2018147	14.91848709	113281.39	87755.664	435353.8	456488.7	17.95630566	3.351405906	3	High	1692	Unique	Used	72431	1	24.611	1	3.3403	5.2648	1	4	19/36	22.67572	1692	1370.8	MS2	457.603	196	Glioma_grade 2_5.raw
19920317			eELGQGLQGVEQk	Unambiguous	N-Term(iTRAQ4plex); K13(iTRAQ4plex)	HCD	1.8086374	0	1	1	1.79103	1.6726	1.9543	1.8250769	6.400164895	85312.54	91353.74	152797.62	166727.61	4.835980569	6.165391316	2	High	2530	Unique	Used	50161	1	30.444	1	5.661	-1.2262	1	35	22/24	20.06499	2530	1702.92	MS2	851.963	215	Glioma_grade 2_2.raw
19920317			aSVSQVEADLk	Unambiguous	N-Term(iTRAQ4plex); K11(iTRAQ4plex)	HCD	2.1182704	0	1	1	2.0371	2.2882	1.967	2.209442	7.00293452	83189.17	74060.12	169464.98	163631.53	8.210165096	2.47668284	2	High	3336	Unique	Used	50917	1	36.267	1	4.5708	0.9146	1	29	19/20	35.69857	3336	1434.81	MS2	717.906	322	Glioma_grade 2_2.raw
19920317			lALQALTEk	Unambiguous	N-Term(iTRAQ4plex); K9(iTRAQ4plex)	HCD	1.5837483	0	1	1	1.49624	1.5626	1.604	1.6751369	4.732178193	61138.715	58543.082	91478.17	98067.68	3.067115872	4.916475044	2	High	4157	Unique	Used	59716	1	42.735	1	3.2727	4.8166	1	79	14/16	48.77924	4157	1274.8	MS2	637.903	173	Glioma_grade 2_3.raw
19920317			sINDNIAIFTEVQk	Unambiguous	N-Term(iTRAQ4plex); K14(iTRAQ4plex)	HCD	2.3784592	0	1	1	2.09222	2.6397	2.1714	2.7396047	13.53366882	38941.344	30864.45	81473.84	84556.39	16.36318761	2.625656796	2	High	6038	Unique	Used	53462	1	55.974	1	5.143	-3.9695	1	52	19/26	17.49778	6038	1880.03	MS2	940.518	226	Glioma_grade 2_2.raw
19920317			sVGELPSTVESLQk	Unambiguous	N-Term(iTRAQ4plex); K14(iTRAQ4plex)	HCD	2.3019037	0	1	1	2.34551	2.1649	2.4504	2.2616577	5.270489851	24903.799	26982.02	58412.06	61024.094	5.66445395	3.092843988	2	High	4305	Unique	Used	51831	1	43.308	1	4.6644	3.1772	1	84	18/26	10.01418	4305	1761.99	MS2	881.498	208	Glioma_grade 2_2.raw
19920317			vQEQVHTLLSQDQAQAAR	Unambiguous	N-Term(iTRAQ4plex)	HCD	3.3470476	0	1	1	3.46857	3.757	2.9686	3.2154236	10.09041839	20724.027	19133.016	71882.67	61520.75	5.645249032	10.98470174	3	High	3324	Unique	Used	58942	1	36.295	1	5.346	1.7597	1	6	28/68	2.678958	3324	2166.14	MS2	722.719	185	Glioma_grade 2_3.raw
19920317			vQEQVHTLLSQDQAQAAR	Unambiguous	N-Term(iTRAQ4plex)	HCD	4.0256004	0	1	1	3.67373	3.9039	4.1402	4.3995113	7.736366494	18671.072	17570.445	68592.5	77301.37	4.29485783	8.441891402	2	High	3330	Unique	Used	58948	1	36.327	1	6.3136	0.6952	1	31	24/34	0	3330	2166.14	MS2	1083.57	191	Glioma_grade 2_3.raw
19920317			sINDNIAIFTEVQk	Unambiguous	N-Term(iTRAQ4plex); K14(iTRAQ4plex)	HCD	1.6968193	0	1	1	1.61108	1.5478	1.8519	1.7791895	8.359512838	18392.107	19144.22	29631.17	34061.2	2.833650733	9.836356392	3	High	6014	Unique	Used	53439	1	55.811	1	4.7018	-0.2757	1	23	21/52	23.04894	6014	1880.04	MS2	627.35	199	Glioma_grade 2_2.raw
19920317			lQHVEDGVLSMQVASAR	Unambiguous	N-Term(iTRAQ4plex)	HCD	3.7806332	0	1	1	3.65962	3.7515	3.8091	3.9046826	2.718121284	16590.426	16184.209	60714.676	63194.2	1.752811559	2.829963908	2	High	4600	Unique	Used	67802	1	45.523	1	5.9019	1.608	1	100	25/32	14.10275	4600	1984.04	MS2	992.525	239	Glioma_grade 2_4.raw
19920317			lQHVEDGVLSMQVASAR	Unambiguous	N-Term(iTRAQ4plex)	HCD	3.5891018	0	1	1	3.20106	3.7357	3.4635	4.0419474	9.999737477	15350.937	13154.071	49139.312	53168.062	10.89926967	5.569014888	2	High	4445	Unique	Used	59984	1	44.977	1	5.2493	-0.73	1	67	25/32	0	4445	1984.04	MS2	992.523	240	Glioma_grade 2_3.raw
19920317			lQHVEDGVLSmQVASAR	Unambiguous	N-Term(iTRAQ4plex); M11(Oxidation)	HCD	3.3915231	0	1	1	3.1334	3.3873	3.3955	3.6705635	6.459533682	13061.47	12082.574	40926.754	44349.855	5.505749192	5.67681562	2	High	3252	Unique	Used	66551	1	35.025	1	4.092	-2.5762	1	100	21/32	0	3252	2000.03	MS2	1000.52	228	Glioma_grade 2_4.raw
19920317			qREELGQGLQGVEQk	Unambiguous	N-Term(iTRAQ4plex); K15(iTRAQ4plex)	HCD	3.2831004	1	1	1	2.78499	3.769	2.9241	3.957205	17.56790221	11594.824	8567.732	32291.42	33904.277	21.23220172	3.445728872	2	High	1597	Unique	Used	72342	1	23.92	1	5.489	3.6633	1	58	24/28	0	1597	1987.09	MS2	994.048	192	Glioma_grade 2_5.raw
19920317			vQEQVHTLLSQDQAQAAR	Unambiguous	N-Term(iTRAQ4plex)	HCD	3.8874378	0	1	1	4.12478	3.5336	4.3004	3.68411	9.234235894	11544.964	13476.328	47620.44	49648.273	10.91614759	2.9483159	2	High	3415	Unique	Used	50992	1	36.823	1	5.573	2.8366	1	29	23/34	3.838467	3415	2166.14	MS2	1083.58	191	Glioma_grade 2_2.raw
19920317			lQHVEDGVLSMQVASAR	Unambiguous	N-Term(iTRAQ4plex)	HCD	3.0298126	0	1	1	3.00223	3.0697	2.9908	3.0580113	1.302846303	6629.02	6483.223	19901.871	19825.77	1.572485308	0.270902232	2	High	4689	Unique	Used	67882	1	46.269	1	4.9291	2.0387	1	100	24/32	45.94796	4689	1984.04	MS2	992.526	256	Glioma_grade 2_4.raw
19920317			eRDFTSLENTVEER	Unambiguous	N-Term(iTRAQ4plex)	HCD	1.5474479	1	1	1	1.49156	1.5335	1.561	1.604905	3.076469528	5315.4844	5170.065	7928.3784	8297.463	1.961309607	3.216871373	2	High	3821	Unique	Used	67076	1	39.524	1	3.9259	1.9423	1	100	19/26	11.66081	3821	1868.91	MS2	934.961	139	Glioma_grade 2_4.raw
19923362	NP_006279.2	THY1	thy-1 membrane glycoprotein preproprotein [Homo sapiens]	4	22	0.480991483	20.6972194	125.69509	24.84	9.95E-05	161	17.923	8.7339	Unclassified, Integral membrane protein	Immune response	Protein binding,Mole cular function unknown	Plasma membrane	-																								
19923362			vLYLSAFTSk	Unambiguous	N-Term(iTRAQ4plex); K10(iTRAQ4plex)	HCD	0.4914629	0	1	1	0.48536	0.4832	0.4998	0.4975401	0.01710892	422799.28	424707.3	205209.14	211308.9	0.318386644	2.071065954	2	High	5719	Unique	Used	61161	1	54.996	1	4.3982	0.2985	1	17	15/18	21.16224	5719	1416.83	MS2	708.921	174	Glioma_grade 2_3.raw
19923362			hVLFGTVGVPEHTYR	Unambiguous	N-Term(iTRAQ4plex)	HCD	0.6237657	0	1	1	0.57606	0.6126	0.6342	0.67450607	0.065924404	275950.28	259470.33	158962.69	175014.31	4.352871063	6.797000602	2	High	2328	Unique	Used	79751	1	29.101	1	4.4887	2.2985	1	47	17/28	44.49829	2328	1856	MS2	928.503	207	Glioma_grade 2_6.raw
19923362			hVLFGTVGVPEHTYR	Unambiguous	N-Term(iTRAQ4plex)	HCD	0.37603277	0	1	1	0.37751	0.4544	0.311	0.37425652	0.154751542	221853.38	184327.53	83751.55	68985.78	13.06549981	13.67180649	4	High	2324	Unique	Used	79747	1	29.08	1	5.4921	4.1256	1	5	23/84	14.92191	2324	1856	MS2	464.756	183	Glioma_grade 2_6.raw
19923362			vLYLSAFTSk	Unambiguous	N-Term(iTRAQ4plex); K10(iTRAQ4plex)	HCD	0.45570442	0	1	1	0.40454	0.426	0.4839	0.5095845	0.107439547	184622.8	175328.6	74686.7	89344.734	3.651599547	12.63757194	2	High	5907	Unique	Used	53337	1	55.046	1	3.9336	-3.9232	1	26	15/18	43.84895	5907	1416.83	MS2	708.918	194	Glioma_grade 2_2.raw
19923362			hVLFGTVGVPEHTYR	Unambiguous	N-Term(iTRAQ4plex)	HCD	0.51396054	0	1	1	0.50273	0.4989	0.5291	0.52510285	0.029813724	92757.086	93460.97	46632.113	49076.62	0.534557347	3.612058006	2	High	2598	Unique	Used	90339	1	31.849	1	5.1392	0.0623	1	100	20/28	0	2598	1855.99	MS2	928.501	174	Glioma_grade 2_7rep.raw
19923362			vTSLTAcLVDQSLR	Unambiguous	N-Term(iTRAQ4plex); C7(Methylthio)	HCD	0.6008928	0	1	1	0.56366	0.618	0.5853	0.6417167	0.057403425	88664.06	80863.6	49976.426	51891.527	6.507207334	2.658698568	2	High	4779	Unique	Used	87240	1	63.045	1	5.4356	3.329	1	91	21/26	45.56937	4779	1695.89	MS2	848.451	277	Glioma_grade 2_1rep.raw
19923362			hVLFGTVGVPEHTYR	Unambiguous	N-Term(iTRAQ4plex)	HCD	0.60343045	0	1	1	0.54471	0.5715	0.6339	0.66503984	0.091672982	85310.36	81312.83	46469.24	54076.27	3.392901758	10.69959762	3	High	2593	Unique	Used	90334	1	31.818	1	5.7835	-1.655	1	18	23/56	11.85614	2593	1855.99	MS2	619.335	199	Glioma_grade 2_7rep.raw
19923362			kHVLFGTVGVPEHTYR	Unambiguous	N-Term(iTRAQ4plex); K1(iTRAQ4plex)	HCD	0.3986211	1	1	1	0.38283	0.3805	0.4169	0.41431782	0.049295698	75826.46	76295.14	29028.445	31610.438	0.435713017	6.021696538	4	High	2311	Unique	Used	85635	1	32.967	1	5.514	-1.7418	1	6	25/90	13.35305	2311	2128.19	MS2	532.802	148	Glioma_grade 2_1rep.raw
19923362			hVLFGTVGVPEHTYR	Unambiguous	N-Term(iTRAQ4plex)	HCD	0.38648874	0	1	1	0.33917	0.3844	0.3883	0.44012475	0.106410648	49002.105	43231.12	16620.016	19027.088	8.848660833	9.549482247	2	High	2791	Unique	Used	85959	1	38.656	1	4.5559	0.7858	1	100	20/28	10.03146	2791	1856	MS2	928.501	166	Glioma_grade 2_1rep.raw
19923362			hVLFGTVGVPEHTYR	Unambiguous	N-Term(iTRAQ4plex)	HCD	0.32112843	0	1	1	0.24697	0.3147	0.3262	0.41564	0.212670907	48263.73	37872.438	11919.473	15741.299	17.06078459	19.53986737	4	High	2786	Unique	Used	85955	1	38.608	1	5.4684	0.9028	1	39	25/84	17.8273	2786	1856	MS2	464.754	213	Glioma_grade 2_1rep.raw
19923362			hVLFGTVGVPEHTYR	Unambiguous	N-Term(iTRAQ4plex)	HCD	0.5820789	0	1	1	0.4877	0.6374	0.5397	0.7054284	0.164584378	37465.598	28666.242	18271.967	20221.98	18.8172121	7.164073955	3	High	2780	Unique	Used	85950	1	38.553	1	5.5177	1.4034	1	59	25/56	43.67992	2780	1856	MS2	619.337	221	Glioma_grade 2_1rep.raw
19923362			kHVLFGTVGVPEHTYR	Unambiguous	N-Term(iTRAQ4plex); K1(iTRAQ4plex)	HCD	0.38790968	1	1	1	0.38294	0.394	0.382	0.393025	0.016452144	31343.352	30465.812	12002.546	11973.826	2.007839759	0.169400998	2	High	2327	Unique	Used	85648	1	33.121	1	5.1882	-1.213	1	100	22/30	0	2327	2128.19	MS2	1064.6	91	Glioma_grade 2_1rep.raw
19923362			vLYLSAFTSk	Unambiguous	N-Term(iTRAQ4plex); K10(iTRAQ4plex)	HCD	0.42849505	0	1	1	0.37716	0.4636	0.3999	0.49159425	0.123489966	28001.912	22781.133	10561.209	11199.074	14.53890067	4.145522069	3	High	5747	Unique	Used	61187	1	55.215	1	3.3569	0.3967	1	30	15/36	31.35132	5747	1416.83	MS2	472.95	167	Glioma_grade 2_3.raw
19923362			hVLFGTVGVPEHTYR	Unambiguous	N-Term(iTRAQ4plex)	HCD	0.41211125	0	1	1	0.38163	0.447	0.3823	0.4478193	0.091107082	26142.492	22319.082	9976.6455	9994.915	11.15753747	0.129368833	2	High	2940	Unique	Used	95448	1	41.156	1	4.6349	-0.3981	1	100	19/28	29.58598	2940	1855.99	MS2	928.5	170	Glioma_grade 2_8rep.raw
19923362			kHVLFGTVGVPEHTYR	Unambiguous	N-Term(iTRAQ4plex); K1(iTRAQ4plex)	HCD	0.5753878	1	1	1	0.56175	0.5745	0.5762	0.58933496	0.019605775	24040.053	23504.984	13504.523	13852.309	1.591547477	1.797882438	3	High	2305	Unique	Used	85630	1	32.909	1	6.7746	3.312	1	82	26/60	40.58355	2305	2128.2	MS2	710.071	95	Glioma_grade 2_1rep.raw
19923362			kHVLFGTVGVPEHTYR	Unambiguous	N-Term(iTRAQ4plex); K1(iTRAQ4plex)	HCD	0.5039779	1	1	1	0.46544	0.5711	0.4493	0.5512613	0.119442718	20104.701	16386.01	9357.539	9032.973	14.41195061	2.495882872	4	High	2462	Unique	Used	95144	1	35.133	1	5.3016	0.8967	1	32	25/90	17.05812	2462	2128.19	MS2	532.804	140	Glioma_grade 2_8rep.raw
19923362			kHVLFGTVGVPEHTYR	Unambiguous	N-Term(iTRAQ4plex); K1(iTRAQ4plex)	HCD	0.4884143	1	1	1	0.46916	0.491	0.4859	0.50856984	0.03320227	18892.572	18051.807	8863.559	9180.604	3.218409127	2.484844206	3	High	2467	Unique	Used	95148	1	35.183	1	6.6493	0.0426	1	69	29/60	10.75222	2467	2128.19	MS2	710.068	92	Glioma_grade 2_8rep.raw
19923362			kHVLFGTVGVPEHTYR	Unambiguous	N-Term(iTRAQ4plex); K1(iTRAQ4plex)	HCD	0.34888682	1	1	1	0.30868	0.3679	0.333	0.39680314	0.110137296	18173.154	15248.784	5609.7085	6050.7656	12.3741589	5.349258763	2	High	2481	Unique	Used	95158	1	35.341	1	4.7547	-1.213	1	100	23/30	0	2481	2128.19	MS2	1064.6	79	Glioma_grade 2_8rep.raw
19923362			hVLFGTVGVPEHTYR	Unambiguous	N-Term(iTRAQ4plex)	HCD	0.5910742	0	1	1	0.5376	0.6087	0.5755	0.6516279	0.081764569	16734.607	14778.756	8996.487	9630.249	8.777200358	4.81176528	3	High	2939	Unique	Used	95447	1	41.151	1	6.1083	-0.0765	1	97	24/56	32.23166	2939	1855.99	MS2	619.336	210	Glioma_grade 2_8rep.raw
19923362			hVLFGTVGVPEHTYR	Unambiguous	N-Term(iTRAQ4plex)	HCD	0.35168123	0	1	1	0.27842	0.3577	0.347	0.4457998	0.192324633	14456.83	11253.532	4025.0293	5016.8228	17.61992879	15.51239506	4	High	2919	Unique	Used	95432	1	40.935	1	5.0695	-1.1361	1	100	22/84	60.67421	2919	1855.99	MS2	464.753	220	Glioma_grade 2_8rep.raw
19923362			kHVLFGTVGVPEHTYR	Unambiguous	N-Term(iTRAQ4plex); K1(iTRAQ4plex)	HCD	0.6239045	1	1	1	0.53252	0.5796	0.6646	0.7233711	0.136548709	8425.648	7740.751	4486.854	5599.4355	5.991381422	15.5996697	5	High	2322	Unique	Used	85644	1	33.075	1	3.6089	-0.1564	1	38	17/120	36.27045	2322	2128.19	MS2	426.444	128	Glioma_grade 2_1rep.raw
19923362			kHVLFGTVGVPEHTYR	Unambiguous	N-Term(iTRAQ4plex); K1(iTRAQ4plex)	HCD	0.4735687	1	1	1	0.47654	0.5667	0.3953	0.4700301	0.14703074	4884.779	4107.9907	2327.815	1930.8792	12.21586436	13.18131722	5	High	2476	Unique	Used	95154	1	35.292	1	4.122	0.8473	1	97	18/120	23.07603	2476	2128.19	MS2	426.444	123	Glioma_grade 2_8rep.raw
19923830	NP_065385.2	PBXIP1	pre-B-cell leukemia transcription factor-interacting protein 1 [Homo sapiens]	6	9	2.036073685	10.06122383	73.593165	13.54	1.72E-04	731	80.594	5.3257	Transcription regulatory protein	Regulation of nucleobase, nucleoside, nucleotide and nucleic acid metabolism	Transcription regulator activity	Cytoplasm	Microtubule, Nucleus																								
19923830			lLQAQLQAQk	Unambiguous	N-Term(iTRAQ4plex); K10(iTRAQ4plex)	HCD	2.28963	0	1	1	2.25885	2.1865	2.3961	2.3194196	3.889870897	318664.72	329206.2	719817.25	763567.25	2.301060832	4.170991631	2	High	2335	Unique	Used	58017	1	28.943	1	4.6941	-0.9217	1	12	17/18	22.22516	2335	1428.88	MS2	714.942	116	Glioma_grade 2_3.raw
19923830			lPWAGQLTk	Unambiguous	N-Term(iTRAQ4plex); K9(iTRAQ4plex)	HCD	2.2604768	0	1	1	1.99557	2.3836	2.1574	2.5768926	11.18427902	244873.36	205010.38	488662.06	528289.75	12.53096343	5.510783903	2	High	4902	Unique	Used	68077	1	47.967	1	3.4517	-1.303	1	16	14/16	7.227962	4902	1301.78	MS2	651.394	155	Glioma_grade 2_4.raw
19923830			lPWAGQLTk	Unambiguous	N-Term(iTRAQ4plex); K9(iTRAQ4plex)	HCD	2.0360737	0	1	1	1.88291	2.038	2.0343	2.2018542	6.387985688	217083.16	200565.8	408747.44	441616.66	5.592992384	5.466375722	2	High	4741	Unique	Used	60256	1	47.338	1	3.6406	0.1035	1	20	14/16	9.862837	4741	1301.78	MS2	651.395	141	Glioma_grade 2_3.raw
19923830			lPWAGQLTk	Unambiguous	N-Term(iTRAQ4plex); K9(iTRAQ4plex)	HCD	1.9936212	0	1	1	1.96208	1.8736	2.1193	2.0237403	5.186796466	147173.73	154125.75	288766.56	311910.47	3.263079302	5.448923427	2	High	4432	Unique	Used	74923	1	46.064	1	3.2992	0.3849	1	20	14/16	24.94347	4432	1301.78	MS2	651.395	154	Glioma_grade 2_5.raw
19923830			eELQSLMHQPk	Unambiguous	N-Term(iTRAQ4plex); K11(iTRAQ4plex)	HCD	1.6656404	0	1	1	1.69682	1.636	1.6964	1.6355816	2.107040505	136915.12	142004.6	232319.84	232260.11	2.580531646	0.018182226	3	High	2158	Unique	Used	72852	1	28.255	1	4.1936	-0.0941	1	3	24/40	9.659237	2158	1627.87	MS2	543.295	206	Glioma_grade 2_5.raw
19923830			qkEQLEAEAQALR	Unambiguous	N-Term(iTRAQ4plex); K2(iTRAQ4plex)	HCD	1.9742527	1	1	1	1.86209	2.0341	1.9194	2.0967832	5.391753606	52138.637	47728.777	97086.62	100076.89	6.244763502	2.144859558	2	High	2596	Unique	Used	73266	1	31.375	1	3.9135	13.846	1	44	21/24	18.211	2596	1802.03	MS2	901.517	118	Glioma_grade 2_5.raw
19923830			lLQAQLQAQk	Unambiguous	N-Term(iTRAQ4plex); K10(iTRAQ4plex)	HCD	2.2627418	0	1	1	2.46146	2.0598	2.5053	2.0964503	10.30894705	49152.504	58737.223	120986.94	123139.66	12.56360543	1.24706256	2	High	2595	Unique	Used	65938	1	30.068	1	4.4106	12.405	1	51	17/18	15.67534	2595	1428.9	MS2	714.951	92	Glioma_grade 2_4.raw
19923830			eLPLSPAFFGEDGIFR	Unambiguous	N-Term(iTRAQ4plex)	HCD	1.9361672	0	1	1	1.87419	1.9952	1.8808	2.0021462	3.615732095	32418.828	30453.18	60759.004	60971.715	4.421436739	0.247118216	2	High	5865	Unique	Used	88048	1	74.941	1	5.2088	-0.6543	1	84	19/30	20.19214	5865	1939.01	MS2	970.007	229	Glioma_grade 2_1rep.raw
19923830			dSHDPLPSWAELLRPk	Unambiguous	N-Term(iTRAQ4plex); K16(iTRAQ4plex)	HCD	2.2400715	0	1	1	1.88762	2.0923	2.3733	2.6307516	14.45696711	14689.087	13251.847	27727.371	34862.316	7.274503781	16.12140349	4	High	4964	Unique	Used	92187	1	55.271	1	4.733	-2.5168	1	6	28/90	3.801434	4964	2149.16	MS2	538.045	134	Glioma_grade 2_7rep.raw
19923985	NP_612462.1	RAB3C	ras-related protein Rab-3C [Homo sapiens]	2	3	0.298628211	19.14816983	17.707561	11.45	1.56E-04	227	25.936	5.2368	GTPase	Transport	GTPase activity	Cytoplasmic vesicle	Microsome, Plasma membrane																								
19923985			lVDIIcDk	Unambiguous	N-Term(iTRAQ4plex); C6(Methylthio); K8(iTRAQ4plex)	HCD	0.21510775	0	1	1	0.20102	0.2098	0.2202	0.22981182	0.058046904	1549528.6	1484515.1	311487.22	341159.1	3.030377362	6.429573543	2	High	6405	Unique	Used	61803	1	60.305	1	3.3448	2.5526	1	8	14/14	20.88697	6405	1252.69	MS2	626.849	272	Glioma_grade 2_3.raw

19923985			gQHLGEQLGFEFFETSAk	Unambiguous	N-Term(iTRAQ4plex); K18(iTRAQ4plex)	HCD	0.2986282	0	1	1	0.28073	0.2678	0.331	0.3157041	0.098697165	62737.75	65770.37	17612.174	20763.975	3.337355129	11.61481763	3	High	5407	Unique	Used	82415	1	55.716	1	7.6853	2.5866	1	10	28/68	17.64053	5407	2313.18	MS2	771.732	168	Glioma_grade 2_6.raw
19923985			gQHLGEQLGFEFFETSAk	Unambiguous	N-Term(iTRAQ4plex); K18(iTRAQ4plex)	HCD	0.312683	0	1	1	0.30804	0.3077	0.3177	0.3173258	0.017790242	45365.484	45414.59	13974.164	14411.222	0.076499575	2.177505535	2	High	5403	Unique	Used	82412	1	55.669	1	6.6774	-1.2071	1	86	22/34	10.10631	5403	2313.17	MS2	1157.09	149	Glioma_grade 2_6.raw
19924099	NP_008881.2	SYN1	synapsin-1 isoform Ia [Homo sapiens]	13	36	0.223543525	39.11775161	270.45823	31.21	2.65E-05	705	74.066	9.8325	Transport/cargo protein	Transport	Transporter activity	Cytoplasm, Postsynaptic density	Postsynaptic density																								
19924099			iHGEIDlk	Unambiguous	N-Term(iTRAQ4plex); K8(iTRAQ4plex)	HCD	0.1155108	0	1	2	0.10977	0.118	0.1132	0.12167677	0.045255515	1165403.2	1084367.4	127930.484	131942.31	5.093938351	2.183213815	3	High	1241	Unique	Used	78737	1	21.094	1	3.7986	-6.6084	1	5	17/28	59.57758	1241	1212.71	MS2	404.909	224	Glioma_grade 2_6.raw
19924099			gSHGQTPSPGALPLGR	Unambiguous	N-Term(iTRAQ4plex)	HCD	0.17386842	0	1	2	0.18622	0.1856	0.1621	0.16155747	0.080049238	828918.7	831599.94	154360.58	134351.17	0.228353111	9.801325716	2	High	1368	Unique	Used	64801	1	20.906	1	4.7547	2.5502	1	3	22/30	6.937934	1368	1675.9	MS2	838.456	203	Glioma_grade 2_4.raw
19924099			qGPPQkPPGPAGPTR	Unambiguous	N-Term(iTRAQ4plex); K6(iTRAQ4plex)	HCD	0.16400385	0	1	2	0.1779	0.1708	0.1569	0.1506646	0.07611618	776965.75	809120.8	138218.44	121905.87	2.867063453	8.868628131	3	High	932	Unique	Used	84592	1	18.427	1	3.9626	-5.6663	1	4	21/56	9.689385	932	1772.99	MS2	591.669	154	Glioma_grade 2_1rep.raw
19924099			iHGEIDlk	Unambiguous	N-Term(iTRAQ4plex); K8(iTRAQ4plex)	HCD	0.14471325	0	1	2	0.14091	0.1511	0.1388	0.14879245	0.041282951	720065.06	671485.25	101463.84	99911.93	4.937099699	1.089869039	3	High	1230	Unique	Used	78727	1	21.009	1	3.8427	7.8109	1	12	16/28	54.91214	1230	1212.73	MS2	404.914	168	Glioma_grade 2_6.raw
19924099			sLkPDFVLIR	Unambiguous	N-Term(iTRAQ4plex); K3(iTRAQ4plex)	HCD	0.20450412	0	1	2	0.19811	0.1998	0.2092	0.21095246	0.031797987	595820	590857.94	118037.586	124642.93	0.591349372	3.84924477	2	High	3714	Unique	Used	80978	1	40.58	1	4.0193	6.7085	1	25	16/18	40.78232	3714	1475.93	MS2	738.468	90	Glioma_grade 2_6.raw
19924099			gSHGQTPSPGALPLGR	Unambiguous	N-Term(iTRAQ4plex)	HCD	0.21140741	0	1	2	0.22841	0.2555	0.172	0.1923818	0.175400396	584447.25	522439.88	133496.22	100507.93	7.922367263	19.93660673	3	High	1366	Unique	Used	64799	1	20.897	1	5.5363	1.4132	1	0	26/60	6.966747	1366	1675.9	MS2	559.306	142	Glioma_grade 2_4.raw
19924099			vLLVIDEPHTDWAk	Unambiguous	N-Term(iTRAQ4plex); K14(iTRAQ4plex)	HCD	0.17529145	0	1	2	0.16453	0.1726	0.1778	0.18658786	0.052768888	404455.8	385465.25	66543.27	71923.13	3.399921216	5.494669447	2	High	5050	Unique	Used	75466	1	51.263	1	5.3848	1.5899	1	12	20/26	6.767928	5050	1924.08	MS2	962.544	189	Glioma_grade 2_5.raw
19924099			sLkPDFVLIR	Unambiguous	N-Term(iTRAQ4plex); K3(iTRAQ4plex)	HCD	0.18233429	0	1	2	0.18553	0.1887	0.1761	0.17908509	0.031622727	324962.16	319550.84	60290.03	57226.79	1.187371261	3.686345115	3	High	3705	Unique	Used	80970	1	40.505	1	3.7554	3.3497	1	20	18/36	47.99431	3705	1475.92	MS2	492.646	86	Glioma_grade 2_6.raw
19924099			qLIVELVVNk	Unambiguous	N-Term(iTRAQ4plex); K10(iTRAQ4plex)	HCD	0.32397962	0	1	2	0.31286	0.3025	0.3462	0.334732	0.061674176	299152.9	309442.9	93592.195	103580.44	2.391120273	7.164032445	2	High	5968	Unique	Used	61394	1	56.919	1	4.4923	2.6138	1	43	16/18	30.04056	5968	1442.92	MS2	721.965	43	Glioma_grade 2_3.raw
19924099			kIHGEIDlk	Unambiguous	N-Term(iTRAQ4plex); K1(iTRAQ4plex); K9(iTRAQ4plex)	HCD	0.24059983	1	1	2	0.23345	0.2437	0.2376	0.24806961	0.026916031	244012.05	233698.4	56963.58	57973.477	3.053251966	1.242601883	2	High	1216	Unique	Used	84820	1	21.188	1	3.2393	-7.7592	1	91	16/16	54.78405	1216	1484.9	MS2	742.956	98	Glioma_grade 2_1rep.raw
19924099			vLLVIDEPHTDWAk	Unambiguous	N-Term(iTRAQ4plex); K14(iTRAQ4plex)	HCD	0.1714968	0	1	2	0.14619	0.1637	0.1785	0.19983363	0.132191755	237188.72	211828.64	34674.55	42330.49	7.987345763	14.06029291	3	High	5022	Unique	Used	75441	1	51.034	1	6.4115	0.4727	1	11	26/52	18.83775	5022	1924.08	MS2	642.031	171	Glioma_grade 2_5.raw
19924099			vDNQHDFQDIASVVALTk	Unambiguous	N-Term(iTRAQ4plex); K18(iTRAQ4plex)	HCD	0.1505122	0	1	2	0.15028	0.1521	0.1489	0.15075141	0.008881514	216257.02	213604.4	32498.227	32201.166	0.872693153	0.649322467	2	High	5851	Unique	Used	76185	1	57.761	1	7.5555	-0.2603	1	11	23/34	8.794409	5851	2288.21	MS2	1144.61	183	Glioma_grade 2_5.raw
19924099			dHIIEVVGSSmPLIGDHQDEDk	Unambiguous	N-Term(iTRAQ4plex); M11(Oxidation); K22(iTRAQ4plex)	HCD	0.15365618	0	1	2	0.14366	0.1456	0.1616	0.1637877	0.068149066	192559.28	189932.75	27663.617	31108.648	0.97112464	8.289640637	3	High	3396	Unique	Used	80697	1	37.944	1	6.3414	1.4287	1	8	28/84	12.82393	3396	2738.36	MS2	913.458	114	Glioma_grade 2_6.raw
19924099			vLLVIDEPHTDWAk	Unambiguous	N-Term(iTRAQ4plex); K14(iTRAQ4plex)	HCD	0.16929172	0	1	2	0.15368	0.1991	0.1463	0.18952435	0.151484551	172087.05	132813.83	26445.738	25171.455	18.21599215	3.491294656	3	High	5126	Unique	Used	75536	1	51.839	1	6.1218	1.5195	1	11	26/52	16.04821	5126	1924.08	MS2	642.032	163	Glioma_grade 2_5.raw
19924099			qLIVELVVNk	Unambiguous	N-Term(iTRAQ4plex); K10(iTRAQ4plex)	HCD	0.2597317	0	1	2	0.24677	0.2544	0.2649	0.27309275	0.044484708	150499.67	145962.64	37139.32	39861.344	2.16429851	4.999337743	2	High	6098	Unique	Used	53519	1	56.398	1	4.7474	-0.178	1	50	17/18	26.58508	6098	1442.92	MS2	721.963	50	Glioma_grade 2_2.raw
19924099			kIHGEIDlk	Unambiguous	N-Term(iTRAQ4plex); K1(iTRAQ4plex); K9(iTRAQ4plex)	HCD	0.1933001	1	1	2	0.18245	0.1878	0.1987	0.20446718	0.051855318	118869.02	115492.086	21687.684	23614.342	2.037755301	6.014534259	3	High	1367	Unique	Used	94372	1	22.416	1	3.8679	11.468	1	32	18/32	58.5749	1367	1484.93	MS2	495.649	55	Glioma_grade 2_8rep.raw
19924099			qGPPQkPPGPAGPTR	Unambiguous	N-Term(iTRAQ4plex); K6(iTRAQ4plex)	HCD	0.21722992	0	1	2	0.21376	0.213	0.2215	0.22069016	0.020535298	109170.14	109551.125	23335.947	24176.855	0.246338258	2.502953832	3	High	956	Unique	Used	94035	1	18.345	1	4.7647	-2.3615	1	47	18/56	21.21586	956	1773	MS2	591.671	150	Glioma_grade 2_8rep.raw
19924099			qLIVELVVNk	Unambiguous	N-Term(iTRAQ4plex); K10(iTRAQ4plex)	HCD	0.3933858	0	1	2	0.34802	0.4037	0.3845	0.446018	0.103171746	104792.516	90328.69	36469.69	40288.22	10.48319622	7.035388163	2	High	6213	Unique	Used	53628	1	57.236	1	4.3714	-6.7768	1	65	16/18	68.603	6213	1442.91	MS2	721.958	63	Glioma_grade 2_2.raw
19924099			dHIIEVVGSSMPLIGDHQDEDk	Unambiguous	N-Term(iTRAQ4plex); K22(iTRAQ4plex)	HCD	0.31722152	0	1	2	0.31694	0.354	0.2843	0.31753913	0.089424403	91679.234	82088.8	29056.553	26066.404	7.805188054	7.671412238	3	High	5000	Unique	Used	82070	1	52.094	1	9.5551	2.3493	1	18	35/84	13.3375	5000	2722.37	MS2	908.127	112	Glioma_grade 2_6.raw
19924099			kIHGEIDlk	Unambiguous	N-Term(iTRAQ4plex); K1(iTRAQ4plex); K9(iTRAQ4plex)	HCD	0.28670827	1	1	2	0.2744	0.3085	0.2674	0.30054423	0.069132066	73357.69	65257.438	20129.355	19612.746	8.264239555	1.838341295	2	High	1336	Unique	Used	94345	1	22.132	1	3.1594	-6.5261	1	100	16/16	49.30691	1336	1484.91	MS2	742.957	94	Glioma_grade 2_8rep.raw
19924099			vLLVIDEPHTDWAk	Unambiguous	N-Term(iTRAQ4plex); K14(iTRAQ4plex)	HCD	0.357343	0	1	2	0.36688	0.3269	0.3915	0.34884593	0.076357625	63178.996	70900.86	23179.018	24733.475	8.144672228	4.588227483	2	High	4880	Unique	Used	81967	1	51.047	1	4.8316	-0.3768	1	100	18/26	22.82118	4880	1924.08	MS2	962.542	207	Glioma_grade 2_6.raw
19924099			gSHGQTPSPGALPLGR	Unambiguous	N-Term(iTRAQ4plex)	HCD	0.45378768	0	1	2	0.55834	0.5255	0.3776	0.3553909	0.225783746	59460.34	63180.414	33199.117	22453.744	4.289747847	27.30542861	3	High	1263	Unique	Used	72038	1	21.352	1	5.611	1.5225	1	7	25/60	28.93246	1263	1675.9	MS2	559.306	159	Glioma_grade 2_5.raw
19924099			kIHGEIDlk	Unambiguous	N-Term(iTRAQ4plex); K1(iTRAQ4plex); K9(iTRAQ4plex)	HCD	0.26450127	1	1	2	0.22988	0.2412	0.2867	0.30082926	0.129979461	53888.08	51350.473	12388.005	15447.725	3.410079418	15.5451196	4	High	1334	Unique	Used	94343	1	22.118	1	3.3198	10.144	1	49	14/48	64.11575	1334	1484.93	MS2	371.988	72	Glioma_grade 2_8rep.raw
19924099			dHIIEVVGSSmPLIGDHQDEDk	Unambiguous	N-Term(iTRAQ4plex); M11(Oxidation); K22(iTRAQ4plex)	HCD	0.22122417	0	1	2	0.20925	0.2152	0.2271	0.23353922	0.049845954	53103.598	51628.836	11111.988	12057.358	1.991387331	5.770361733	3	High	3376	Unique	Used	80680	1	37.764	1	6.739	12.395	1	19	31/84	7.280326	3376	2738.39	MS2	913.468	149	Glioma_grade 2_6.raw
19924099			lGTEEFPLIDQTFYPNHk	Unambiguous	N-Term(iTRAQ4plex); K18(iTRAQ4plex)	HCD	0.31171972	0	1	2	0.31773	0.3251	0.2986	0.3055673	0.038239928	51926.14	50742.125	16498.588	15505.135	1.630932471	4.389972711	2	High	6116	Unique	Used	76420	1	59.944	1	5.4502	-0.4169	1	90	19/34	29.81311	6116	2437.26	MS2	1219.14	149	Glioma_grade 2_5.raw
19924099			dHIIEVVGSSMPLIGDHQDEDk	Unambiguous	N-Term(iTRAQ4plex); K22(iTRAQ4plex)	HCD	0.16441752	0	1	2	0.17385	0.1813	0.1482	0.15458478	0.094982288	43558.6	41769.113	7572.502	6456.869	2.965879075	11.24600183	2	High	5040	Unique	Used	82106	1	52.416	1	5.6396	1.2333	1	46	21/42	9.932335	5040	2722.36	MS2	1361.69	84	Glioma_grade 2_6.raw
19924099			dHIIEVVGSSmPLIGDHQDEDk	Unambiguous	N-Term(iTRAQ4plex); M11(Oxidation); K22(iTRAQ4plex)	HCD	0.28634351	0	1	2	0.27961	0.297	0.2763	0.2934916	0.035534045	36870.766	34709.973	10309.596	10187.085	4.269057303	0.845291575	4	High	3419	Unique	Used	80718	1	38.118	1	7.4503	-1.1617	1	8	20/126	62.78352	3419	2738.35	MS2	685.343	169	Glioma_grade 2_6.raw
19924099			kLGTEEFPLIDQTFYPNHk	Unambiguous	N-Term(iTRAQ4plex); K1(iTRAQ4plex); K19(iTRAQ4plex)	HCD	0.42263338	1	1	2	0.39202	0.4043	0.4404	0.4542079	0.069411994	35026.496	33959.086	13731.124	15424.485	2.188204629	8.213768034	3	High	4188	Unique	Used	86821	1	56.392	1	7.9178	-3.0649	1	35	29/72	87.36947	4188	2709.45	MS2	903.823	122	Glioma_grade 2_1rep.raw
19924099			vDNQHDFQDIASVVALTk	Unambiguous	N-Term(iTRAQ4plex); K18(iTRAQ4plex)	HCD	0.45521614	0	1	2	0.44783	0.5264	0.3947	0.46390343	0.11833488	32027.232	27248.71	14342.597	12640.771	11.40066339	8.919369004	3	High	5884	Unique	Used	76215	1	58.018	1	5.8464	-2.8535	1	13	23/68	10.90389	5884	2288.21	MS2	763.407	155	Glioma_grade 2_5.raw
19924099			vDNQHDFQDIASVVALTk	Unambiguous	N-Term(iTRAQ4plex); K18(iTRAQ4plex)	HCD	0.2869862	0	1	2	0.2404	0.2713	0.3009	0.33956242	0.146889065	28020.756	24829.938	6736.1064	8431.313	8.538200256	15.80614409	3	High	6260	Unique	Used	69325	1	58.703	1	6.8378	3.5481	1	23	24/68	19.97779	6260	2288.22	MS2	763.412	150	Glioma_grade 2_4.raw
19924099			qGPPQkPPGPAGPTR	Unambiguous	N-Term(iTRAQ4plex); K6(iTRAQ4plex)	HCD	0.22586286	0	1	2	0.18589	0.2174	0.2331	0.2726093	0.158661519	21858.242	18691.59	4063.2	5095.501	11.04399694	15.93996872	2	High	981	Unique	Used	94057	1	18.567	1	3.4997	5.2195	1	100	16/28	0	981	1773.01	MS2	887.009	123	Glioma_grade 2_8rep.raw
19924099			qLIVELVVNk	Unambiguous	N-Term(iTRAQ4plex); K10(iTRAQ4plex)	HCD	0.2818399	0	1	2	0.30588	0.3338	0.2342	0.25560912	0.161423304	20554.984	18834.865	6287.268	4814.3633	6.175742432	18.76302452	3	High	5974	Unique	Used	61400	1	56.959	1	4.1542	-2.9368	1	76	19/36	16.04215	5974	1442.91	MS2	481.643	53	Glioma_grade 2_3.raw
19924099			qTSQQPAGPPAQQRPPPQGGPPQPGPGPQR	Unambiguous	N-Term(iTRAQ4plex)	HCD	0.11564535	0	1	2	0.11429	0.1233	0.1086	0.11710822	0.052765487	14930.607	13842.632	1706.4054	1621.0858	5.347430647	3.626159416	3	High	990	Unique	Used	78515	1	19.03	1	5.1853	-0.3109	1	56	25/116	11.10533	990	3182.63	MS2	1061.55	63	Glioma_grade 2_6.raw
19924099			kLGTEEFPLIDQTFYPNHk	Unambiguous	N-Term(iTRAQ4plex); K1(iTRAQ4plex); K19(iTRAQ4plex)	HCD	0.41719922	1	1	2	0.42979	0.4489	0.3869	0.40405285	0.065695247	9751.055	9336.128	4190.869	3772.289	3.074290171	7.433753204	3	High	4249	Unique	Used	96223	1	58.409	1	8.4111	1.3954	1	100	30/72	13.43162	4249	2709.47	MS2	903.827	114	Glioma_grade 2_8rep.raw
19924099			qTSQQPAGPPAQQRPPPQGGPPQPGPGPQR	Unambiguous	N-Term(iTRAQ4plex)	HCD	0.12356674	0	1	2	0.09688	0.106	0.1396	0.15277804	0.215140297	9500.309	8679.529	920.3818	1326.0414	6.384865518	25.53789989	3	High	1129	Unique	Used	78637	1	20.188	1	5.4122	1.8754	1	98	22/116	0	1129	3182.64	MS2	1061.55	63	Glioma_grade 2_6.raw
19924099			qGPPLQQRPPPQGQQHLSGLGPPAGSPLPQR	Unambiguous	N-Term(iTRAQ4plex)	HCD	0.27132624	0	1	2	0.32031	0.2463	0.3039	0.2336645	0.153913708	3522.3298	4580.802	1128.2217	1070.3707	18.47317528	3.721184008	4	High	2843	Unique	Used	85996	1	39.252	1	6.1357	2.4906	1	50	22/180	11.72951	2843	3364.82	MS2	841.96	34	Glioma_grade 2_1rep.raw
19924103	NP_598328.1	SYN2	synapsin-2 isoform IIa [Homo sapiens]	12	31	0.293955654	38.45496905	203.0655	36.43	9.02E-05	582	62.957	8.4116	Unclassified	Cell communication,Signal transduction	Molecular function unknown	Synaptic vesicle	-																								
19924103			vLLVVDEPHADWAk	Unambiguous	N-Term(iTRAQ4plex); K14(iTRAQ4plex)	HCD	0.16310537	0	1	2	0.15028	0.1558	0.1702	0.17640395	0.074665354	1145693.2	1105261.6	172170.28	194972.52	2.540207252	8.783295508	3	High	4675	Unique	Used	75130	1	48.244	1	5.9413	1.6338	1	1	26/52	4.747934	4675	1880.06	MS2	627.357	132	Glioma_grade 2_5.raw
19924103			qLITELVISk	Unambiguous	N-Term(iTRAQ4plex); K10(iTRAQ4plex)	HCD	0.27484137	0	1	2	0.2415	0.2975	0.2564	0.31591436	0.124976695	436734	354478.34	105473.08	111984.8	14.70238317	4.234825953	2	High	5775	Unique	Used	53213	1	54.067	1	4.5765	-1.8	1	26	16/18	29.94043	5775	1431.9	MS2	716.454	101	Glioma_grade 2_2.raw
19924103			qHAFGMAENEDFR	Unambiguous	N-Term(iTRAQ4plex)	HCD	0.18181837	0	1	2	0.15734	0.1815	0.1821	0.21005383	0.117932048	323201	280235.3	50851.3	58864.5	10.06944323	10.32884609	2	High	2122	Unique	Used	65496	1	26.587	1	4.6148	3.1702	1	20	21/24	3.464797	2122	1695.77	MS2	848.39	57	Glioma_grade 2_4.raw
19924103			vLGDYDlk	Unambiguous	N-Term(iTRAQ4plex); K8(iTRAQ4plex)	HCD	0.387068	0	1	2	0.32881	0.382	0.3914	0.4547529	0.132678788	322195.8	277338.2	105940.086	126120.34	10.58125582	12.29817138	2	High	3030	Unique	Used	50627	1	34.111	1	3.1871	-4.1348	1	42	14/14	50.05179	3030	1210.69	MS2	605.847	239	Glioma_grade 2_2.raw
19924103			sFRPDFVLIR	Unambiguous	N-Term(iTRAQ4plex)	HCD	0.25509477	0	1	2	0.20422	0.2304	0.277	0.31248268	0.18826314	219724.81	194782.53	44872.49	60866.17	8.509791564	21.39092662	2	High	4466	Unique	Used	74954	1	46.332	1	3.2922	0.0906	1	12	13/18	13.92298	4466	1393.81	MS2	697.407	165	Glioma_grade 2_5.raw
19924103			eMLTLPTFPVVVk	Unambiguous	N-Term(iTRAQ4plex); K13(iTRAQ4plex)	HCD	0.23540887	0	1	2	0.2343	0.2515	0.2204	0.2366022	0.054096476	210987.28	196531.53	49433.754	46499.793	5.016582598	4.325126681	2	High	7089	Unique	Used	62441	1	65.614	1	4.9126	1.2555	1	27	17/24	22.31336	7089	1762.04	MS2	881.526	137	Glioma_grade 2_3.raw
19924103			emLTLPTFPVVVk	Unambiguous	N-Term(iTRAQ4plex); M2(Oxidation); K13(iTRAQ4plex)	HCD	0.5293773	0	1	2	0.46209	0.51	0.5469	0.60364616	0.112631622	204843.58	185585.3	94656.33	112027.85	6.975744408	11.88626976	2	High	6649	Unique	Used	62031	1	62.196	1	4.696	2.7996	1	29	17/24	36.90781	6649	1778.04	MS2	889.525	170	Glioma_grade 2_3.raw
19924103			emLTLPTFPVVVk	Unambiguous	N-Term(iTRAQ4plex); M2(Oxidation); K13(iTRAQ4plex)	HCD	0.47716883	0	1	2	0.43914	0.4838	0.4711	0.51906306	0.069101278	165624.1	150325.92	72732.87	78028.625	6.84756837	4.967666674	2	High	6900	Unique	Used	54269	1	62.363	1	4.6994	3.0742	1	30	17/24	31.72101	6900	1778.04	MS2	889.525	173	Glioma_grade 2_2.raw
19924103			vLLVVDEPHADWAk	Unambiguous	N-Term(iTRAQ4plex); K14(iTRAQ4plex)	HCD	0.21942723	0	1	2	0.20585	0.2158	0.2229	0.23365502	0.053367707	160953.1	153542.2	33132.92	35875.906	3.332512533	5.62126358	2	High	4638	Unique	Used	75100	1	47.887	1	5.1657	0.245	1	30	21/26	9.491192	4638	1880.05	MS2	940.53	202	Glioma_grade 2_5.raw
19924103			fPLIEQTYYPNHk	Unambiguous	N-Term(iTRAQ4plex); K13(iTRAQ4plex)	HCD	0.24111076	0	1	2	0.23888	0.2488	0.2337	0.24343285	0.02657865	140218.11	134640.08	33495.45	32775.82	2.87003479	1.535673763	3	High	4144	Unique	Used	81337	1	44.558	1	6.9672	0.2064	1	15	24/48	14.5123	4144	1938.04	MS2	646.684	206	Glioma_grade 2_6.raw
19924103			sFRPDFVLIR	Unambiguous	N-Term(iTRAQ4plex)	HCD	0.13964136	0	1	2	0.12856	0.1525	0.1288	0.15278019	0.098345656	137390.72	115825.57	17663.613	17695.854	12.04414124	0.128948944	3	High	4452	Unique	Used	74941	1	46.226	1	3.3559	-0.97	1	16	18/36	3.777597	4452	1393.81	MS2	465.274	173	Glioma_grade 2_5.raw
19924103			qHAFGMAENEDFR	Unambiguous	N-Term(iTRAQ4plex)	HCD	0.30235413	0	1	2	0.29095	0.3256	0.2816	0.31511423	0.067623211	134712.8	120372.766	39195.004	37931.17	7.950222698	2.317411964	2	High	1802	Unique	Used	72536	1	25.337	1	4.116	5.3298	1	34	21/24	0	1802	1695.78	MS2	848.392	67	Glioma_grade 2_5.raw
19924103			kVLGDYDlk	Unambiguous	N-Term(iTRAQ4plex); K1(iTRAQ4plex); K9(iTRAQ4plex)	HCD	0.506457	1	1	2	0.40926	0.5086	0.5048	0.6272404	0.174171643	126990.12	102193.21	51971.785	64099.71	15.30134257	14.77664781	3	High	1932	Unique	Used	89770	1	26.035	1	4.6576	-9.1345	1	25	17/32	66.82311	1932	1482.88	MS2	494.963	172	Glioma_grade 2_7rep.raw
19924103			vLLVVDEPHADWAk	Unambiguous	N-Term(iTRAQ4plex); K14(iTRAQ4plex)	HCD	0.25387284	0	1	2	0.27748	0.231	0.2814	0.23422258	0.105984421	109179.516	131175.22	30295.34	30724.197	12.94196379	0.993936394	3	High	4540	Unique	Used	81670	1	48.163	1	5.9227	-0.9959	1	20	26/52	37.22311	4540	1880.05	MS2	627.355	146	Glioma_grade 2_6.raw
19924103			qLITELVISk	Unambiguous	N-Term(iTRAQ4plex); K10(iTRAQ4plex)	HCD	0.44606653	0	1	2	0.40203	0.4531	0.4398	0.4956979	0.086337361	77445.18	68709.75	31135.545	34059.277	8.452512398	6.342193015	2	High	5677	Unique	Used	53122	1	53.311	1	4.053	1.5248	1	27	16/18	48.93521	5677	1431.9	MS2	716.456	100	Glioma_grade 2_2.raw
19924103			qHAFGmAENEDFR	Unambiguous	N-Term(iTRAQ4plex); M6(Oxidation)	HCD	0.56006503	0	1	2	0.46771	0.5006	0.6156	0.6589162	0.162539332	75716.195	70739.55	35413.31	46611.434	4.805573762	19.30702622	3	High	1319	Unique	Used	64755	1	20.574	1	4.4559	1.6463	1	9	22/48	25.05284	1319	1711.76	MS2	571.26	45	Glioma_grade 2_4.raw
19924103			vLLVVDEPHADWAk	Unambiguous	N-Term(iTRAQ4plex); K14(iTRAQ4plex)	HCD	0.31925827	0	1	2	0.27974	0.3445	0.2987	0.36792567	0.125746379	75393.03	61216.883	21090.582	22523.262	14.67543527	4.64557879	2	High	4722	Unique	Used	75171	1	48.639	1	4.9581	1.2189	1	49	22/26	28.03199	4722	1880.05	MS2	940.531	184	Glioma_grade 2_5.raw
19924103			fPLIEQTYYPNHk	Unambiguous	N-Term(iTRAQ4plex); K13(iTRAQ4plex)	HCD	0.27246544	0	1	2	0.25284	0.3189	0.2357	0.2972196	0.139491361	67504.13	53524.617	17067.584	15908.565	16.33497609	4.970563388	3	High	4227	Unique	Used	74736	1	44.452	1	6.1482	1.7181	1	29	24/48	29.40072	4227	1938.04	MS2	646.685	202	Glioma_grade 2_5.raw
19924103			tPPQRPPPQGGPGQPQGmQPPGk	Unambiguous	N-Term(iTRAQ4plex); M18(Oxidation); K23(iTRAQ4plex)	HCD	0.31023622	0	1	1	0.25208	0.2937	0.3244	0.37799335	0.169905353	50142.016	43034.043	12640.008	16266.581	10.78838483	17.7424902	3	High	988	Unique	Used	88929	1	18.548	1	3.6755	-0.3995	1	32	Nov-88	16.51666	988	2638.38	MS2	880.13	95	Glioma_grade 2_7rep.raw
19924103			eMLTLPTFPVVVk	Unambiguous	N-Term(iTRAQ4plex); K13(iTRAQ4plex)	HCD	0.27723235	0	1	2	0.24798	0.2386	0.3174	0.30537263	0.143539463	43498.184	45211.348	10786.844	13806.309	2.731138029	17.36324071	2	High	7364	Unique	Used	54699	1	65.92	1	4.8766	4.9272	1	100	17/24	28.6284	7364	1762.05	MS2	881.529	124	Glioma_grade 2_2.raw

19924103			dYIFEVMDcSMPLIGEHQVEDR	Unambiguous	N-Term(iTRAQ4plex); C9(Methylthio)	HCD	0.16016245	0	1	2	0.15817	0.1607	0.1596	0.16218504	0.010615945	43295.914	42608.35	6848.201	6910.4375	1.131913934	0.639712297	3	High	8326	Unique	Used	63539	1	76.252	1	6.8635	-1.1579	1	8	38/84	4.83092	8326	2816.25	MS2	939.421	33	Glioma_grade2_3.raw
19924103			tPALSPQRPLTTQQPQSGTLk	Unambiguous	N-Term(iTRAQ4plex); K21(iTRAQ4plex)	HCD	0.19832183	0	1	2	0.21158	0.2237	0.1743	0.18430677	0.116007579	40110.39	37940.473	8486.505	6992.686	3.931700346	13.64786499	3	High	1936	Unique	Used	72650	1	26.523	1	3.7493	10.108	1	35	19/80	14.2854	1936	2537.45	MS2	846.49	34	Glioma_grade2_5.raw
19924103			vLLVVDEPHADWAk	Unambiguous	N-Term(iTRAQ4plex); K14(iTRAQ4plex)	HCD	0.43525457	0	1	2	0.44515	0.4251	0.4459	0.4258053	0.026573124	34912.86	36556.613	15541.415	15566.001	3.252602388	0.111773523	2	High	4532	Unique	Used	81664	1	48.077	1	4.4925	-0.3394	1	100	19/26	0	4532	1880.05	MS2	940.529	216	Glioma_grade2_6.raw
19924103			vLLVVDEPHADWAk	Unambiguous	N-Term(iTRAQ4plex); K14(iTRAQ4plex)	HCD	0.35980353	0	1	2	0.33981	0.3757	0.3455	0.3819089	0.058647291	29089.018	26312.572	9884.683	10049.005	7.087319314	1.165797323	3	High	4588	Unique	Used	75060	1	47.386	1	6.278	0.7572	1	100	25/52	42.02796	4588	1880.05	MS2	627.356	156	Glioma_grade2_5.raw
19924103			sFRPDFVLIR	Unambiguous	N-Term(iTRAQ4plex)	HCD	0.20109542	0	1	2	0.18447	0.2125	0.1912	0.2202417	0.084095087	24173.3	20985.926	4459.3374	4621.976	9.981631526	2.532736222	3	High	4539	Unique	Used	75020	1	46.913	1	3.4431	6.0583	1	64	19/36	9.14598	4539	1393.82	MS2	465.277	191	Glioma_grade2_5.raw
19924103			tPPQRPPPQGGPGQPQGMQPPGk	Unambiguous	N-Term(iTRAQ4plex); K23(iTRAQ4plex)	HCD	0.42130706	0	1	1	0.35308	0.398	0.442	0.49821243	0.14664568	22632.48	20079.3	7991.0176	10003.757	8.453737548	15.81816622	3	High	1339	Unique	Used	89239	1	21.354	1	4.9329	-1.1307	1	47	19/88	0	1339	2622.38	MS2	874.798	113	Glioma_grade2_7rep.raw
19924103			sFRPDFVLIR	Unambiguous	N-Term(iTRAQ4plex)	HCD	0.27129352	0	1	2	0.30332	0.3126	0.2312	0.23828675	0.156746792	11337.712	11001.259	3438.9639	2621.4543	2.129983497	19.07678852	3	High	4559	Unique	Used	75036	1	47.112	1	3.2301	-4.9768	1	100	16/36	13.73524	4559	1393.8	MS2	465.272	182	Glioma_grade2_5.raw
19924103			dGkDYIFEVMDcSMPLIGEHQVEDR	Unambiguous	N-Term(iTRAQ4plex); K3(iTRAQ4plex); C12(Methylthio)	HCD	0.57237095	1	1	2	0.57118	0.5472	0.5987	0.5735124	0.036791206	7692.26	8030.062	4393.66	4605.34	3.038509005	3.326599921	3	High	6575	Unique	Used	93296	1	73.488	1	8.9242	-2.0391	1	60	27/96	45.71467	6575	3260.49	MS2	1087.5	63	Glioma_grade2_7rep.raw
19924103			dYIFEVMDcSMPLIGEHQVEDR	Unambiguous	N-Term(iTRAQ4plex); C9(Methylthio)	HCD	0.49186242	0	1	2	0.41444	0.4161	0.5674	0.56959367	0.179877441	7539.8037	7510.319	3124.7595	4277.83	0.277058622	22.02861498	3	High	8658	Unique	Used	55873	1	76.464	1	4.3542	-1.548	1	36	30/84	22.75354	8658	2816.25	MS2	939.421	20	Glioma_grade2_2.raw
19924103			dYIFEVMDcSmPLIGEHQVEDR	Unambiguous	N-Term(iTRAQ4plex); C9(Methylthio); M11(Oxidation)	HCD	0.29395565	0	1	2	0.36007	0.2687	0.3278	0.2446202	0.176517729	4425.957	5931.3037	1593.6586	1450.9166	20.55448618	6.630405198	3	High	7808	Unique	Used	63080	1	71.791	1	7.9074	-1.1877	1	100	33/84	8.702733	7808	2832.24	MS2	944.753	27	Glioma_grade2_3.raw
19924103			dGkDYIFEVMDcSMPLIGEHQVEDR	Unambiguous	N-Term(iTRAQ4plex); K3(iTRAQ4plex); C12(Methylthio)	HCD	0.39129922	1	1	2	0.54152	0.42	0.3542	0.27477977	0.283700129	1806.6578	2329.157	978.3359	640.0052	17.86650251	29.56557579	4	High	7150	Unique	Used	83804	1	72.836	1	7.8461	-0.2648	1	58	33/144	16.62111	7150	3260.5	MS2	815.88	44	Glioma_grade2_6.raw
20070125	NP_000909.2	P4HB	protein disulfide-isomerase precursor [Homo sapiens]	15	42	2.576843023	21.89103115	236.1208	44.09	1.06E-04	508	57.081	4.8687	Enzyme: Isomerase	Protein metabolism	Isomerase activity	Endoplasmic reticulum	Mitochondrion, Zymogen granule, Nucleolus, Nucleus, Extracellular																								
20070125			qLAPIWDk	Unambiguous	N-Term(iTRAQ4plex); K8(iTRAQ4plex)	HCD	2.604433	0	1	1	2.32497	2.5647	2.6405	2.9127142	9.273951228	862125.1	781543.75	2004411.6	2276413.8	6.933223687	8.985865208	2	High	3726	Unique	Used	59313	1	39.449	1	3.5113	0.951	1	4	13/14	20.26774	3726	1258.74	MS2	629.874	202	Glioma_grade2_3.raw
20070125			ykPESEELTAER	Unambiguous	N-Term(iTRAQ4plex); K2(iTRAQ4plex)	HCD	2.7479186	0	1	1	2.70592	2.8924	2.6128	2.7928154	4.345296995	609193.06	569923.4	1648425	1591690.8	4.709940676	2.476278011	2	High	1239	Unique	Used	72015	1	21.191	1	3.3462	0.4185	1	2	18/22	7.252458	1239	1739.91	MS2	870.457	171	Glioma_grade2_5.raw
20070125			iLEFFGLk	Unambiguous	N-Term(iTRAQ4plex); K8(iTRAQ4plex)	HCD	2.8154967	0	1	1	2.93166	2.6733	2.9714	2.7095706	5.378559036	583401.9	639777.56	1710335.1	1733522.4	6.518031537	0.952182085	2	High	7608	Unique	Used	70529	1	70.124	1	3.5273	-1.7433	1	4	14/14	3.854862	7608	1254.77	MS2	627.888	139	Glioma_grade2_4.raw
20070125			qLAPIWDk	Unambiguous	N-Term(iTRAQ4plex); K8(iTRAQ4plex)	HCD	2.0115519	0	1	1	1.78538	2.052	1.9764	2.271499	9.944591865	420981.94	366289.8	751611.06	832026.94	9.824608482	7.181264161	2	High	3801	Unique	Used	51355	1	39.635	1	3.1124	0.5631	1	15	14-Dec	48.22271	3801	1258.74	MS2	629.874	210	Glioma_grade2_2.raw
20070125			lITLEEEMTk	Unambiguous	N-Term(iTRAQ4plex); K10(iTRAQ4plex)	HCD	2.7895746	0	1	1	2.65315	2.7275	2.85	2.929822	4.42783019	412973.7	401720.78	1095680.6	1176970.4	1.953374237	5.058459818	2	High	5522	Unique	Used	52975	1	52.22	1	4.5855	-0.324	1	8	17/18	8.052874	5522	1494.83	MS2	747.92	254	Glioma_grade2_2.raw
20070125			eNLLDFIk	Unambiguous	N-Term(iTRAQ4plex); K8(iTRAQ4plex)	HCD	2.938736	0	1	1	2.89232	2.8882	2.9894	2.9850886	1.907462716	307505.34	307947.9	889402.75	919251.8	0.101693242	2.333941069	2	High	6473	Unique	Used	53871	1	59.163	1	3.7987	-2.0029	1	9	14/14	15.68283	6473	1279.75	MS2	640.377	273	Glioma_grade2_2.raw
20070125			iLEFFGLk	Unambiguous	N-Term(iTRAQ4plex); K8(iTRAQ4plex)	HCD	2.8624594	0	1	1	2.43927	2.9687	2.7752	3.3775063	13.55173411	270453.47	222219.86	659708.5	750549	13.84540653	9.109532629	2	High	7166	Unique	Used	77322	1	69.249	1	3.5518	-0.1867	1	10	14/14	9.297417	7166	1254.77	MS2	627.889	136	Glioma_grade2_5.raw
20070125			qLAPIWDk	Unambiguous	N-Term(iTRAQ4plex); K8(iTRAQ4plex)	HCD	2.9273572	0	1	1	2.84219	2.8305	3.0246	3.012171	3.597438711	247107.84	248125.31	702328.5	747395.9	0.29055403	4.396347906	2	High	3955	Unique	Used	67200	1	40.577	1	3.5069	0.854	1	15	13/14	32.54012	3955	1258.74	MS2	629.874	196	Glioma_grade2_4.raw
20070125			lITLEEEMTk	Unambiguous	N-Term(iTRAQ4plex); K10(iTRAQ4plex)	HCD	2.9294343	0	1	1	3.23475	2.6112	3.3236	2.6829672	12.41614533	231160.06	286358.5	747746.1	768290.5	15.08397737	1.916455652	2	High	5327	Unique	Used	60802	1	51.875	1	4.2303	1.4726	1	11	17/18	8.79978	5327	1494.84	MS2	747.921	249	Glioma_grade2_3.raw
20070125			lITLEEEmTk	Unambiguous	N-Term(iTRAQ4plex); M8(Oxidation); K10(iTRAQ4plex)	HCD	1.7003105	0	1	1	1.62181	1.6617	1.738	1.7807424	4.233181632	211657.06	206579.38	343267.16	367864.66	1.716953196	4.891655404	2	High	3037	Unique	Used	50633	1	34.171	1	3.2142	1.106	1	14	16/18	17.20158	3037	1510.83	MS2	755.918	265	Glioma_grade2_2.raw
20070125			tHILLFLPk	Unambiguous	N-Term(iTRAQ4plex); K9(iTRAQ4plex)	HCD	2.0470514	0	1	1	1.93951	2.0428	2.0511	2.1603193	4.404514713	192742.22	182994.77	373825.8	395327.12	3.668783313	3.953369683	2	High	4268	Unique	Used	91665	1	48.069	1	4.1123	0.6469	1	18	13/16	53.01927	4268	1369.88	MS2	685.445	39	Glioma_grade2_7rep.raw
20070125			nFEDVAFDEk	Unambiguous	N-Term(iTRAQ4plex); K10(iTRAQ4plex)	HCD	1.9259294	0	1	1	1.78658	2.0812	1.7927	2.088257	8.79738352	165880.03	142398.88	296357.97	297365.44	10.77185617	0.239973313	2	High	4129	Unique	Used	51664	1	42.049	1	3.7724	2.2982	1	19	17/18	17.21211	4129	1501.74	MS2	751.376	127	Glioma_grade2_2.raw
20070125			sNFAEALAAHk	Unambiguous	N-Term(iTRAQ4plex); K11(iTRAQ4plex)	HCD	2.057925	0	1	1	1.92438	2.4538	1.7475	2.228211	15.04492798	151956.58	119172.16	292421.88	265540.7	17.10042668	6.813311625	3	High	2199	Unique	Used	72891	1	28.542	1	4.855	1.0117	1	3	29/40	7.373508	2199	1446.8	MS2	482.937	241	Glioma_grade2_5.raw
20070125			lGETYkDHENIVIAk	Unambiguous	N-Term(iTRAQ4plex); K6(iTRAQ4plex); K15(iTRAQ4plex)	HCD	2.164974	1	1	1	2.37038	1.9913	2.3717	1.9924188	10.03555306	147163.05	175177.66	348832	349027.25	12.29092081	0.039567463	3	High	2335	Unique	Used	90113	1	29.567	1	4.3699	3.3561	1	3	24/56	9.330368	2335	2162.23	MS2	721.413	110	Glioma_grade2_7rep.raw
20070125			sNFAEALAAHk	Unambiguous	N-Term(iTRAQ4plex); K11(iTRAQ4plex)	HCD	2.5838358	0	1	1	2.27019	2.8685	2.3586	2.9801407	13.62590363	127620.57	101002.53	289722.8	301001.75	16.46534981	2.700216888	3	High	2150	Unique	Used	79585	1	27.766	1	4.6198	2.7203	1	9	22/40	52.44706	2150	1446.8	MS2	482.938	261	Glioma_grade2_6.raw
20070125			yQLDkDGVVLFk	Unambiguous	N-Term(iTRAQ4plex); K5(iTRAQ4plex); K12(iTRAQ4plex)	HCD	1.9809406	1	1	1	1.87954	1.9224	2.0382	2.0846536	4.853902255	107276.92	104885.62	201631.2	218650.19	1.593970779	5.726755228	3	High	4184	Unique	Used	81371	1	44.914	1	4.0334	-1.2222	1	14	21/44	19.47437	4184	1857.08	MS2	619.699	106	Glioma_grade2_6.raw
20070125			tHILLFLPk	Unambiguous	N-Term(iTRAQ4plex); K9(iTRAQ4plex)	HCD	1.7698032	0	1	1	1.65131	1.8344	1.7117	1.9014289	6.409545291	101788.445	91630.555	168084.56	174229	7.427101682	2.538476823	2	High	4121	Unique	Used	81317	1	44.356	1	3.8247	-0.7788	1	40	13/16	41.59021	4121	1369.88	MS2	685.444	43	Glioma_grade2_6.raw
20070125			iTEFcHR	Unambiguous	N-Term(iTRAQ4plex); C5(Methylthio)	HCD	3.1866412	0	1	1	2.9217	3.401	3.0025	3.4950397	8.894427401	98393.516	84527.38	287476.7	295426.6	10.7203048	1.9287687	3	High	2276	Unique	Used	72964	1	29.078	1	3.3588	-0.8255	1	8	16/24	7.933425	2276	1095.52	MS2	365.844	81	Glioma_grade2_5.raw
20070125			sNFAEALAAHk	Unambiguous	N-Term(iTRAQ4plex); K11(iTRAQ4plex)	HCD	1.8011664	0	1	1	1.96179	1.8193	1.7816	1.652211	7.061784152	90736.81	97841.695	178006.17	161655.12	5.328191952	6.807922289	3	High	2105	Unique	Used	72803	1	27.859	1	4.4294	-0.3172	1	8	24/40	52.7062	2105	1446.79	MS2	482.936	278	Glioma_grade2_5.raw
20070125			lITLEEEmTk	Unambiguous	N-Term(iTRAQ4plex); M8(Oxidation); K10(iTRAQ4plex)	HCD	2.1053853	0	1	1	2.09087	2.1251	2.086	2.120137	0.947818896	85169.945	83797.266	178079.38	177661.7	1.148898208	0.166044563	2	High	3078	Unique	Used	58712	1	34.443	1	3.4318	1.6716	1	36	16/18	28.75647	3078	1510.83	MS2	755.919	299	Glioma_grade2_3.raw
20070125			yQLDkDGVVLFk	Unambiguous	N-Term(iTRAQ4plex); K5(iTRAQ4plex); K12(iTRAQ4plex)	HCD	2.1807349	1	1	1	2.1012	2.2088	2.154	2.264347	3.220470709	66081.41	62860.887	138850.1	142338.86	3.532205809	1.754639196	2	High	4193	Unique	Used	81379	1	44.985	1	5.1941	0.4612	1	64	18/22	15.65735	4193	1857.09	MS2	929.046	108	Glioma_grade2_6.raw
20070125			nFEDVAFDEk	Unambiguous	N-Term(iTRAQ4plex); K10(iTRAQ4plex)	HCD	1.7593673	0	1	1	1.68653	1.7581	1.7606	1.8352966	3.451489749	58404.5	56028.19	98500.79	102828.35	2.936756822	3.039845123	2	High	4030	Unique	Used	51571	1	41.308	1	4.0754	-0.7094	1	100	17/18	52.32711	4030	1501.74	MS2	751.374	132	Glioma_grade2_2.raw
20070125			iLEFFGLk	Unambiguous	N-Term(iTRAQ4plex); K8(iTRAQ4plex)	HCD	3.0827036	0	1	1	2.99334	2.7832	3.4048	3.1657972	8.536223056	57648.355	62000.953	172560.9	196282.44	5.144620747	9.095277032	2	High	7084	Unique	Used	77251	1	68.521	1	3.6819	-2.6189	1	73	13/14	6.954952	7084	1254.77	MS2	627.887	151	Glioma_grade2_5.raw
20070125			eNLLDFIk	Unambiguous	N-Term(iTRAQ4plex); K8(iTRAQ4plex)	HCD	3.10884	0	1	1	3.21055	2.9033	3.3361	3.0168614	6.212139598	54624.684	60405.402	175375.4	182234.73	7.10698399	2.712607027	2	High	6322	Unique	Used	61726	1	59.641	1	3.9222	0.191	1	100	14/14	40.48616	6322	1279.75	MS2	640.379	277	Glioma_grade2_3.raw
20070125			iLEFFGLk	Unambiguous	N-Term(iTRAQ4plex); K8(iTRAQ4plex)	HCD	2.665339	0	1	1	2.2989	2.7296	2.6112	3.1004202	12.33484556	52915.406	44566.633	121647.38	138175.3	12.11192146	8.996138683	2	High	7530	Unique	Used	62829	1	69.449	1	3.6606	1.078	1	100	14/14	33.44195	7530	1254.77	MS2	627.889	110	Glioma_grade2_3.raw
20070125			lGETYkDHENIVIAk	Unambiguous	N-Term(iTRAQ4plex); K6(iTRAQ4plex); K15(iTRAQ4plex)	HCD	2.9332123	1	1	1	2.48628	3.2645	2.6809	3.520038	16.23060151	50634.145	38563.47	125890.68	135744.88	19.13785732	5.326471404	2	High	2325	Unique	Used	90106	1	29.452	1	4.703	-2.4244	1	30	20/28	0	2325	2162.21	MS2	1081.61	152	Glioma_grade2_7rep.raw
20070125			hNQLPLVIEFTEQTAPk	Unambiguous	N-Term(iTRAQ4plex); K17(iTRAQ4plex)	HCD	2.9730966	0	1	1	2.69859	3.2036	2.779	3.2989714	10.02445147	50057.707	42167.316	135085.12	139108.77	12.09942552	2.075283443	2	High	5159	Unique	Used	82211	1	53.407	1	5.829	-1.7837	1	39	20/32	14.59825	5159	2253.24	MS2	1127.13	143	Glioma_grade2_6.raw
20070125			lGETYkDHENIVIAk	Unambiguous	N-Term(iTRAQ4plex); K6(iTRAQ4plex); K15(iTRAQ4plex)	HCD	1.922046	1	1	1	1.77309	1.5757	2.3118	2.0544202	16.70495536	48789.9	54902.81	86508.7	112793.45	8.337095469	18.6512006	4	High	2328	Unique	Used	90108	1	29.49	1	4.0484	-2.1546	1	2	20/84	13.7647	2328	2162.21	MS2	541.309	146	Glioma_grade2_7rep.raw
20070125			iLFIFIDSDHTDNQR	Unambiguous	N-Term(iTRAQ4plex)	HCD	3.6910825	0	1	1	4.1486	3.7197	3.6592	3.2808664	9.600305621	36690.04	40920.79	152212.33	134255.64	7.709212785	8.864723876	3	High	6597	Unique	Used	76841	1	64.069	1	6.222	-2.4141	1	8	28/56	12.42507	6597	1978.01	MS2	660.008	138	Glioma_grade2_5.raw
20070125			iLEFFGLk	Unambiguous	N-Term(iTRAQ4plex); K8(iTRAQ4plex)	HCD	2.5698502	0	1	1	2.53663	2.4144	2.7332	2.6014664	5.166382762	30838.877	32400.455	78226.92	84288.695	3.492138067	5.274966604	2	High	7535	Unique	Used	70468	1	69.428	1	3.4888	0.8834	1	100	14/14	36.04288	7535	1254.77	MS2	627.889	127	Glioma_grade2_4.raw
20070125			tHILLFLPk	Unambiguous	N-Term(iTRAQ4plex); K9(iTRAQ4plex)	HCD	2.537799	0	1	1	2.4588	2.4162	2.6615	2.615426	4.681618605	26986.041	27461.633	66353.38	71823.87	1.235293645	5.598925403	3	High	4258	Unique	Used	91659	1	47.938	1	3.8459	-0.3208	1	59	17/32	34.92485	4258	1369.88	MS2	457.298	38	Glioma_grade2_7rep.raw
20070125			iLFIFIDSDHTDNQR	Unambiguous	N-Term(iTRAQ4plex)	HCD	2.9214299	0	1	1	2.85415	2.8407	3.0025	2.9883957	2.938359356	22423.898	22530.023	64001.105	67328.625	0.333860564	3.583212966	3	High	7001	Unique	Used	69995	1	64.835	1	6.0775	0.2704	1	20	25/56	42.76541	7001	1978.02	MS2	660.01	130	Glioma_grade2_4.raw
20070125			iLFIFIDSDHTDNQR	Unambiguous	N-Term(iTRAQ4plex)	HCD	3.4420078	0	1	1	3.16503	3.3107	3.5675	3.7317364	7.377412834	20042.934	19160.945	63436.46	71503.59	3.181625996	8.454602363	2	High	6532	Unique	Used	76783	1	63.519	1	4.9244	-4.4748	1	55	20/28	11.88007	6532	1978.01	MS2	989.507	149	Glioma_grade2_5.raw
20070125			iLFIFIDSDHTDNQR	Unambiguous	N-Term(iTRAQ4plex)	HCD	3.0078309	0	1	1	2.84272	2.7548	3.2689	3.1678383	8.254841495	19488.184	20109.965	55399.41	63705.12	2.220636937	9.861965558	2	High	7009	Unique	Used	70003	1	64.886	1	4.8005	-2.9936	1	100	19/28	14.32061	7009	1978.01	MS2	989.508	155	Glioma_grade2_4.raw
20070125			tHILLFLPk	Unambiguous	N-Term(iTRAQ4plex); K9(iTRAQ4plex)	HCD	2.2030191	0	1	1	2.11107	2.3098	2.1054	2.303628	5.193897984	16920.66	15464.608	35720.664	35624.703	6.358349376	0.190214661	3	High	4225	Unique	Used	81408	1	45.24	1	3.3833	0.8153	1	46	16/32	6.044942	4225	1369.88	MS2	457.299	36	Glioma_grade2_6.raw
20070125			tHILLFLPk	Unambiguous	N-Term(iTRAQ4plex); K9(iTRAQ4plex)	HCD	2.8410575	0	1	1	2.41244	2.7455	2.9251	3.3288448	13.38214925	15415.64	13545.754	37189.273	45091.71	9.130838596	13.58240164	3	High	4087	Unique	Used	81287	1	44.068	1	3.6213	1.751	1	57	16/32	11.79667	4087	1369.88	MS2	457.299	34	Glioma_grade2_6.raw
20070125			iLFIFIDSDHTDNQR	Unambiguous	N-Term(iTRAQ4plex)	HCD	2.1100914	0	1	1	2.18459	1.9996	2.2308	2.0419037	5.245745054	14973.771	16358.857	32711.502	33403.215	6.251653727	1.479594787	2	High	6624	Unique	Used	76865	1	64.287	1	4.641	-4.1045	1	100	20/28	16.13371	6624	1978.01	MS2	989.507	145	Glioma_grade2_5.raw
20070125			iLFIFIDSDHTDNQR	Unambiguous	N-Term(iTRAQ4plex)	HCD	2.8043027	0	1	1	3.09514	2.5312	3.1382	2.5664508	11.60086607	12343.622	15093.545	38205.277	38736.844	14.1741252	0.977032152	3	High	6514	Unique	Used	76769	1	63.34	1	6.4274	-3.8027	1	33	26/56	23.11434	6514	1978.01	MS2	660.007	134	Glioma_grade2_5.raw
20070125			ikPHLMSQELPEDWDk	Unambiguous	N-Term(iTRAQ4plex); K2(iTRAQ4plex); K16(iTRAQ4plex)	HCD	1.7706969	0	1	1	1.72095	1.5539	2.0108	1.8156134	10.73859119	12273.67	13592.967	21122.4	24679.572	7.213027771	10.98337182	3	High	3532	Unique	Used	86416	1	48.117	1	6.6803	1.792	1	38	23/60	18.85217	3532	2398.28	MS2	800.1	141	Glioma_grade2_1rep.raw
20070125			iLFIFIDSDHTDNQR	Unambiguous	N-Term(iTRAQ4plex)	HCD	2.3712564	0	1	1	2.27534	2.3917	2.3518	2.472072	3.45381945	8009.1226	7619.6094	18223.51	18836.22	3.52462919	2.338124945	3	High	7098	Unique	Used	70080	1	65.681	1	6.7291	0.6407	1	88	29/56	12.00503	7098	1978.02	MS2	660.01	146	Glioma_grade2_4.raw
20070125			ikPHLMSQELPEDWDk	Unambiguous	N-Term(iTRAQ4plex); K2(iTRAQ4plex); K16(iTRAQ4plex)	HCD	1.6410557	0	1	1	1.35367	1.7076	1.5883	2.0035944	16.25387381	7715.174	6115.9497	10443.761	12253.883	16.35185068	11.27825902	3	High	3614	Unique	Used	95858	1	49.891	1	6.6836	1.8683	1	100	26/60	13.35689	3614	2398.28	MS2	800.1	152	Glioma_grade2_8rep.raw
20070125			kSNFAEALAAHk	Unambiguous	N-Term(iTRAQ4plex); K1(iTRAQ4plex); K12(iTRAQ4plex)	HCD	1.5662893	1	1	1	1.35511	1.5012	1.6251	1.8002276	12.02348837	7664.648	6918.9526	10386.447	12455.689	7.23122209	12.81119288	2	High	2228	Unique	Used	94993	1	32.21	1	3.7095	0.6929	1	100	18/22	26.88289	2228	1718.99	MS2	860	194	Glioma_grade2_8rep.raw
20070197	NP_005207.2	DDOST	dolichyl-diphosphooligosaccharide--protein glycosyltransferase 48 kDa subunit precursor [Homo sapiens]	4	7	2.190453529	15.21999328	42.239362	15.13	1.79E-04	456	50.67	6.4048	Enzyme: Galactosyltransferase	Metabolism,Energy pathways	Galactosyltransferase activity	Endoplasmic reticulum	Mitochondrion																								
20070197			lPDVYGVFQFk	Unambiguous	N-Term(iTRAQ4plex); K11(iTRAQ4plex)	HCD	2.3991215	0	1	1	2.32657	2.4278	2.3717	2.4748275	2.696351114	84944.21	81403.22	197628.78	201458.92	3.010395822	1.357254542	2	High	7076	Unique	Used	62429	1	65.51	1	4.6201	1.2046	1	39	16/20	14.56654	7076	1600.9	MS2	800.954	202	Glioma_grade2_3.raw
20070197			lPDVYGVFQFk	Unambiguous	N-Term(iTRAQ4plex); K11(iTRAQ4plex)	HCD	2.2438977	0	1	1	2.16808	2.2867	2.2033	2.323863	3.212775835	77482.51	73462.78	167988.34	170717.44	3.766104052	1.139493466	2	High	7174	Unique	Used	70148	1	66.317	1	4.4395	1.7384	1	36	17/20	23.36919	7174	1600.9	MS2	800.954	195	Glioma_grade2_4.raw
20070197			eLGSEcGIEFDEEk	Unambiguous	N-Term(iTRAQ4plex); C6(Methylthio); K14(iTRAQ4plex)	HCD	1.7678417	0	1	1	1.72793	1.6881	1.8494	1.8068341	4.147095833	29574.621	30272.15	51102.867	54696.754	1.648301079	4.803914881	2	High	5975	Unique	Used	53402	1	55.528	1	5.8851	-3.9683	1	66	24/26	32.78552	5975	1918.86	MS2	959.933	50	Glioma_grade2_2.raw

20070197			wVPFDGDDIQLEFVR	Unambiguous	N-Term(iTRAQ4plex)	HCD	2.0950656	0	1	1	2.01604	2.0973	2.0929	2.1772804	3.142248456	25934.465	24929.818	52284.805	54279.207	2.793287016	2.646775684	2	High	5610	Unique	Used	87850	1	72.258	1	4.1343	-0.6648	1	100	19/28	29.38284	5610	1980	MS2	990.502	200	Glioma_grade2_1rep.raw
20070197			tAVIDHHNYDISDLGQHTLIVADTENLLk	Unambiguous	N-Term(iTRAQ4plex); K29(iTRAQ4plex)	HCD	1.6538898	0	1	1	1.56988	1.7597	1.5595	1.7480608	6.594108945	11700.397	10438.273	18368.186	18246.738	8.062421447	0.469080337	3	High	5277	Unique	Used	92431	1	58.359	1	8.8682	-2.8851	1	26	34/112	18.78231	5277	3533.84	MS2	1178.62	61	Glioma_grade2_7rep.raw
20070197			lPDVYGVFQFk	Unambiguous	N-Term(iTRAQ4plex); K11(iTRAQ4plex)	HCD	2.5568783	0	1	1	2.88839	2.2559	2.9423	2.2979646	14.23783437	7060.76	9040.422	20394.195	20774.568	17.38794611	1.306642746	3	High	7064	Unique	Used	62418	1	65.416	1	3.7075	-0.405	1	48	19/40	23.30227	7064	1600.9	MS2	534.304	217	Glioma_grade2_3.raw
20070197			lPDVYGVFQFk	Unambiguous	N-Term(iTRAQ4plex); K11(iTRAQ4plex)	HCD	2.1904535	0	1	1	1.5128	2.2611	2.1432	3.2032974	30.59736874	2520.7966	1686.5592	3813.4673	5402.551	28.04112372	24.38475757	3	High	7407	Unique	Used	54735	1	66.317	1	3.7402	0.2812	1	100	19/40	42.7167	7407	1600.9	MS2	534.304	216	Glioma_grade2_2.raw
21070969	NP_004787.2	NRXN3	neurexin-3-beta isoform 1 precursor [Homo sapiens]	2	5	0.44741869	24.09997007	32.521853	4.15	2.45E-04	1061	117.24	6.4683	Adhesion molecule	Cell communication,Signal transduction	Cell adhesion molecule activity	Plasma membrane	Nucleus																								
21070969			lFQGQLSGLYYDGLk	Unambiguous	N-Term(iTRAQ4plex); K15(iTRAQ4plex)	HCD	0.42914194	0	1	3	0.38146	0.4009	0.456	0.4792573	0.106777533	31239.58	29725.66	11916.501	14246.239	3.511847401	12.59327989	2	High	6728	Unique	Used	62105	1	62.81	1	5.8514	0.9027	1	100	23/28	33.6018	6728	1990.09	MS2	995.549	218	Glioma_grade2_3.raw
21070969			lFQGQLSGLYYDGLk	Unambiguous	N-Term(iTRAQ4plex); K15(iTRAQ4plex)	HCD	0.4474187	0	1	3	0.44211	0.466	0.4298	0.45301807	0.034470933	21507.002	20404.65	9508.381	9243.676	3.719636603	1.99631113	2	High	6816	Unique	Used	69829	1	63.276	1	4.9536	-1.7349	1	100	21/28	32.79251	6816	1990.09	MS2	995.546	195	Glioma_grade2_4.raw
21070969			lPDLINDALHR	Unambiguous	N-Term(iTRAQ4plex)	HCD	0.3349755	0	1	1	0.32338	0.3567	0.3152	0.34776562	0.058531427	11132.141	10091.004	3599.9297	3509.3044	6.937661999	1.802775469	3	High	5711	Unique	Used	76059	1	56.627	1	5.471	-1.6202	1	92	23/40	0	5711	1420.8	MS2	474.272	207	Glioma_grade2_5.raw
21070969			lPDLINDALHR	Unambiguous	N-Term(iTRAQ4plex)	HCD	0.48522413	0	1	1	0.47716	0.4055	0.579	0.49207368	0.145841693	9348.625	11000.559	4460.8296	5413.085	11.48049704	13.63889152	3	High	6206	Unique	Used	69275	1	58.283	1	5.6687	-1.1692	1	87	24/40	0	6206	1420.8	MS2	474.272	205	Glioma_grade2_4.raw
21070969			lPDLINDALHR	Unambiguous	N-Term(iTRAQ4plex)	HCD	0.6440515	0	1	1	0.53842	0.7179	0.5887	0.7848936	0.17295316	5204.0156	3903.0967	2801.9338	3063.5156	20.20154239	6.306976738	3	High	6295	Unique	Used	69358	1	58.966	1	4.7972	0.6995	1	95	24/40	0	6295	1420.8	MS2	474.273	234	Glioma_grade2_4.raw
21070982	NP_620177.1	PAM	peptidyl-glycine alpha-amidating monooxygenase isoform d preproprotein [Homo sapiens]	4	4	0.479805648	7.249552011	33.013319	10.26	2.42E-04	887	98.699	6.2144	Enzyme: Oxygenase	Protein metabolism	Catalytic activity	Cytoplasm	Golgi apparatus, Endoplasmic reticulum, Axon, Dendrite, Nucleus, Extracellular																								
21070982			iPVDEEAFVIDFkPR	Unambiguous	N-Term(iTRAQ4plex); K13(iTRAQ4plex)	HCD	0.51218957	0	1	5	0.42082	0.4257	0.5977	0.6046122	0.200654013	41241.27	40770.77	17355.209	24650.504	0.811329021	24.56119512	3	High	6030	Unique	Used	76344	1	59.231	1	6.7418	-0.6516	1	37	28/56	32.32891	6030	2063.14	MS2	688.385	165	Glioma_grade2_5.raw
21070982			dGNYWVTDVALHQVFk	Unambiguous	N-Term(iTRAQ4plex); K16(iTRAQ4plex)	HCD	0.4327346	0	1	5	0.40035	0.3833	0.4843	0.46374583	0.112468965	20729.12	21649.121	8298.842	10039.689	3.070155488	13.42489994	3	High	6205	Unique	Used	76497	1	60.716	1	7.486	0.8377	1	36	34/60	29.90532	6205	2180.14	MS2	727.385	112	Glioma_grade2_5.raw
21070982			yFVLQVHYGDISAFR	Unambiguous	N-Term(iTRAQ4plex)	HCD	0.4932948	0	1	5	0.46025	0.5215	0.4684	0.53074276	0.07277531	14232.708	12560.534	6550.5547	6666.4126	8.826147838	1.239677831	3	High	6733	Unique	Used	69755	1	62.568	1	5.2803	0.0756	1	100	25/56	31.59993	6733	1959.02	MS2	653.68	159	Glioma_grade2_4.raw
21070982			qSPQLPQAFYPVGHPVDVSFGDLLAAR	Unambiguous	N-Term(iTRAQ4plex)	HCD	0.4663165	0	1	5	0.47665	0.4478	0.486	0.45660904	0.037679618	6477.2354	6893.846	3087.3633	3147.7925	4.406347872	1.370612009	3	High	7599	Unique	Used	70522	1	70.028	1	6.8995	-5.2941	1	96	33/104	16.20763	7599	3053.58	MS2	1018.53	85	Glioma_grade2_4.raw
21071030	NP_570602.2	A1BG	alpha-1B-glycoprotein precursor [Homo sapiens]	4	4	2.671071768	17.41954193	24.209723	15.96	6.99E-05	495	54.22	5.8589	Secreted polypeptide	Biological_process unknown	Molecular function unknown	Extracellular	-																								
21071030			lLELTGPk	Unambiguous	N-Term(iTRAQ4plex); K8(iTRAQ4plex)	HCD	2.9891162	0	1	1	2.92607	2.9617	3.0162	3.0529337	1.885516605	121386.86	119925.3	355186.06	366124	0.856549448	2.144512319	2	High	3655	Unique	Used	66922	1	38.218	1	2.9279	-0.9284	1	21	14-Dec	24.81892	3655	1158.73	MS2	579.87	88	Glioma_grade2_4.raw
21071030			cEGPIPDVTFELLR	Unambiguous	N-Term(iTRAQ4plex); C1(Methylthio)	HCD	2.3530273	0	1	1	2.1871	2.3126	2.3913	2.5284698	6.075167996	16474.568	15580.813	36031.58	39395.617	3.943052314	6.307362515	2	High	5772	Unique	Used	87977	1	73.946	1	4.2283	-2.3213	1	100	19/26	69.90825	5772	1778.89	MS2	889.949	179	Glioma_grade2_1rep.raw
21071030			sLPAPWLSMAPVSWITPGLk	Unambiguous	N-Term(iTRAQ4plex); K20(iTRAQ4plex)	HCD	2.273356	0	1	1	2.13906	2.2901	2.2577	2.4171307	5.022579556	14709.596	13739.408	31464.768	33209.945	4.822850837	3.816101947	2	High	7934	Unique	Used	77952	1	76.528	1	5.7767	-1.5816	1	82	21/38	45.85957	7934	2439.37	MS2	1220.19	133	Glioma_grade2_5.raw
21071030			tPGAAANLELIFVGPQHAGNYR	Unambiguous	N-Term(iTRAQ4plex)	HCD	3.233102	0	1	1	3.43408	3.4925	2.9781	3.0287023	8.269904693	13771.448	13541.176	47292.316	41012.19	1.192319659	10.05774197	3	High	6196	Unique	Used	69265	1	58.217	1	7.8323	2.5931	1	18	31/84	5.694581	6196	2440.29	MS2	814.102	92	Glioma_grade2_4.raw
21361657	NP_005304.3	PDIA3	protein disulfide-isomerase A3 precursor [Homo sapiens]	13	44	2.238077641	21.10872128	307.74718	34.65	1.49E-04	505	56.747	6.354	Enzyme: Isomerase	Protein metabolism	Isomerase activity	Endoplasmic reticulum	Mitochondrion, Cytoplasm, Plasma membrane																								
21361657			yGVSGYPTLk	Unambiguous	N-Term(iTRAQ4plex); K10(iTRAQ4plex)	HCD	2.0369406	0	1	1	1.91333	2.0095	2.0631	2.1667697	5.189691327	1230592.6	1171690.4	2354524.5	2538783.5	3.467546917	5.325264152	2	High	3081	Unique	Used	50674	1	34.499	1	3.7738	-0.5899	1	3	16/18	19.99277	3081	1372.77	MS2	686.889	219	Glioma_grade2_2.raw
21361657			gFPTIYFSPANk	Unambiguous	N-Term(iTRAQ4plex); K12(iTRAQ4plex)	HCD	1.5891935	0	1	1	1.52595	1.6263	1.5544	1.6565903	3.826769423	770501.94	722968.4	1175750.8	1197662.5	4.501098893	1.305622721	2	High	5675	Unique	Used	53120	1	53.302	1	3.6844	0.3301	1	6	15/22	42.6565	5675	1629.89	MS2	815.448	278	Glioma_grade2_2.raw
21361657			gFPTIYFSPANk	Unambiguous	N-Term(iTRAQ4plex); K12(iTRAQ4plex)	HCD	1.7474409	0	1	1	1.64959	1.7522	1.743	1.8513764	4.715814895	716709.7	674751.9	1182277.1	1249219.6	4.264385723	3.893527447	2	High	5490	Unique	Used	60954	1	53.129	1	3.7303	0.7795	1	8	16/22	58.68645	5490	1629.89	MS2	815.448	265	Glioma_grade2_3.raw
21361657			eATNPPVIQEEkPk	Unambiguous	N-Term(iTRAQ4plex); K12(iTRAQ4plex); K14(iTRAQ4plex)	HCD	1.8192508	0	1	1	1.87784	1.6341	2.0321	1.7682716	9.224316645	376850.84	433069.72	707663.75	765784.9	9.816456883	5.578458305	3	High	1200	Unique	Used	71978	1	20.917	1	4.4278	1.0661	1	1	27/52	19.85145	1200	2012.14	MS2	671.385	105	Glioma_grade2_5.raw
21361657			fLQDYFDGNLk	Unambiguous	N-Term(iTRAQ4plex); K11(iTRAQ4plex)	HCD	2.4480028	0	1	1	2.26538	2.4047	2.4888	2.6418571	6.429805723	360874.53	339971.72	817516.8	898156.7	4.217906195	6.647071267	2	High	6406	Unique	Used	61804	1	60.308	1	4.6792	0.8834	1	13	20/20	14.4337	6406	1647.86	MS2	824.435	181	Glioma_grade2_3.raw
21361657			dPNIVIAk	Unambiguous	N-Term(iTRAQ4plex); K8(iTRAQ4plex)	HCD	2.190419	0	1	1	2.06246	2.2035	2.1782	2.3271294	4.951157755	316529.12	296266.12	652828.75	689449.6	4.676310706	3.858342998	2	High	1693	Unique	Used	49368	1	24.653	1	3.3146	-1.655	1	14	13/14	27.69501	1693	1157.71	MS2	579.359	112	Glioma_grade2_2.raw
21361657			fLQDYFDGNLk	Unambiguous	N-Term(iTRAQ4plex); K11(iTRAQ4plex)	HCD	2.3528683	0	1	1	1.99205	2.482	2.2492	2.802438	14.47463152	209000.42	167743.02	416338.28	470089.44	15.48713751	8.575501166	2	High	6677	Unique	Used	54061	1	60.687	1	4.3571	-0.8945	1	13	20/20	8.874115	6677	1647.86	MS2	824.434	175	Glioma_grade2_2.raw
21361657			gFPTIYFSPANk	Unambiguous	N-Term(iTRAQ4plex); K12(iTRAQ4plex)	HCD	2.1082222	0	1	1	2.00891	2.1783	2.0436	2.2159152	4.77544053	208643.84	192416.7	419145.62	426379.1	5.72198937	1.209862382	2	High	5573	Unique	Used	61026	1	53.856	1	3.9645	0.7046	1	14	16/22	37.98205	5573	1629.89	MS2	815.448	254	Glioma_grade2_3.raw
21361657			tADGIVSHLk	Unambiguous	N-Term(iTRAQ4plex); K10(iTRAQ4plex)	HCD	2.6352117	0	1	1	2.56454	2.6141	2.6559	2.7072535	2.303921989	199365.73	195586.39	511280.56	529501.94	1.353276414	2.475918141	2	High	1551	Unique	Used	72300	1	23.565	1	4.5677	3.4745	1	14	18/18	13.94694	1551	1328.78	MS2	664.895	178	Glioma_grade2_5.raw
21361657			sEPIPESNDGPVk	Unambiguous	N-Term(iTRAQ4plex); K13(iTRAQ4plex)	HCD	2.4623232	0	1	1	2.74776	2.2965	2.6608	2.223767	10.4973199	182237.12	218047.94	500743.38	484887.8	12.65202037	2.275006791	2	High	1412	Unique	Used	49101	1	22.749	1	4.1426	0.1392	1	6	18/24	2.65024	1412	1656.87	MS2	828.938	188	Glioma_grade2_2.raw
21361657			fLQDYFDGNLk	Unambiguous	N-Term(iTRAQ4plex); K11(iTRAQ4plex)	HCD	1.9821413	0	1	1	1.85447	2.0664	1.9066	2.1244018	6.442673881	177515.75	159312.17	329197.4	338443.06	7.642997564	1.958439991	2	High	6581	Unique	Used	53974	1	59.917	1	4.0703	0.1426	1	24	17/20	24.642	6581	1647.86	MS2	824.435	187	Glioma_grade2_2.raw
21361657			dASIVGFFDDSFSEAHSEFLk	Unambiguous	N-Term(iTRAQ4plex); K21(iTRAQ4plex)	HCD	2.9152892	0	1	1	2.63225	3.258	2.6384	3.2656205	12.26818792	145614.56	117645.98	383293.47	384187.1	15.02448683	0.164666536	3	High	7715	Unique	Used	77764	1	74.618	1	6.4764	1.5306	1	1	35/80	4.175442	7715	2636.28	MS2	879.432	99	Glioma_grade2_5.raw
21361657			fLQDYFDGNLk	Unambiguous	N-Term(iTRAQ4plex); K11(iTRAQ4plex)	HCD	2.6053402	0	1	1	2.9505	2.2451	3.0787	2.342693	15.87104684	117958.26	155017.98	348036.4	363159.56	19.19960457	3.007241208	2	High	6527	Unique	Used	69567	1	60.886	1	4.4081	1.3278	1	21	20/20	18.97518	6527	1647.86	MS2	824.436	188	Glioma_grade2_4.raw
21361657			gFPTIYFSPANk	Unambiguous	N-Term(iTRAQ4plex); K12(iTRAQ4plex)	HCD	2.3308146	0	1	1	2.26219	2.3338	2.3279	2.401615	2.443825709	115438.43	111896.61	261143.27	268732.56	2.203307453	2.025545654	2	High	5656	Unique	Used	68771	1	53.868	1	3.7906	1.1539	1	18	17/22	27.79883	5656	1629.89	MS2	815.449	305	Glioma_grade2_4.raw
21361657			eLSDFISYLQR	Unambiguous	N-Term(iTRAQ4plex)	HCD	2.239591	0	1	1	2.12593	2.2288	2.2499	2.3587503	4.259861206	79576.51	75905.1	169174	179041.17	3.339403171	4.007374416	2	High	5402	Unique	Used	87706	1	69.801	1	4.2322	1.4521	1	40	17/20	30.1102	5402	1514.8	MS2	757.903	302	Glioma_grade2_1rep.raw
21361657			eATNPPVIQEEkPk	Unambiguous	N-Term(iTRAQ4plex); K12(iTRAQ4plex); K14(iTRAQ4plex)	HCD	2.3946612	0	1	1	2.255	2.5336	2.271	2.5515742	6.729727687	67423.55	60010.324	152040.16	153120.78	8.226921478	0.500793929	3	High	1065	Unique	Used	78581	1	19.654	1	5.9898	-0.4809	1	22	28/52	28.54139	1065	2012.14	MS2	671.384	109	Glioma_grade2_6.raw
21361657			eATNPPVIQEEkPk	Unambiguous	N-Term(iTRAQ4plex); K12(iTRAQ4plex); K14(iTRAQ4plex)	HCD	2.7989485	0	1	1	2.74244	2.8345	2.7645	2.8573565	1.961897625	56817.848	54972.25	155819.39	157075.33	2.334795093	0.567656553	2	High	1081	Unique	Used	78596	1	19.768	1	5.2714	-1.0506	1	56	16/26	0	1081	2012.14	MS2	1006.57	167	Glioma_grade2_6.raw
21361657			gFPTIYFSPANk	Unambiguous	N-Term(iTRAQ4plex); K12(iTRAQ4plex)	HCD	1.7752373	0	1	1	1.78026	1.6802	1.876	1.7704998	4.506117409	48237.582	51111.53	85875.3	90492.95	4.091004094	3.702675088	2	High	5562	Unique	Used	68688	1	53.071	1	3.7694	0.4799	1	34	17/22	14.59979	5562	1629.89	MS2	815.448	251	Glioma_grade2_4.raw
21361657			fIQENIFGIcPHMTEDNk	Unambiguous	N-Term(iTRAQ4plex); C10(Methylthio); K18(iTRAQ4plex)	HCD	1.8780559	0	1	1	1.88727	1.8996	1.8567	1.8687791	1.014866386	42865.707	42588.45	80899.29	79588.4	0.458843225	1.155153032	3	High	6379	Unique	Used	83214	1	64.807	1	7.6597	-0.2216	1	21	36/68	11.44057	6379	2470.18	MS2	824.065	95	Glioma_grade2_6.raw
21361657			eATNPPVIQEEkPk	Unambiguous	N-Term(iTRAQ4plex); K12(iTRAQ4plex); K14(iTRAQ4plex)	HCD	2.0114675	0	1	1	1.88495	1.9242	2.0969	2.1406221	6.262001468	40419.55	39594.74	76188.766	84757.375	1.457811459	7.5291293	4	High	1201	Unique	Used	71979	1	20.922	1	5.6921	0.6347	1	2	21/78	13.85188	1201	2012.14	MS2	503.791	150	Glioma_grade2_5.raw
21361657			vVVAENFDEIVNNENk	Unambiguous	N-Term(iTRAQ4plex); K16(iTRAQ4plex)	HCD	2.3041823	0	1	1	2.15786	2.2808	2.3263	2.458844	5.390511476	37888.84	35846.324	81758.74	88140.516	3.91747122	5.312085752	2	High	5680	Unique	Used	53125	1	53.329	1	6.9034	-1.0172	1	47	23/30	9.375999	5680	2121.1	MS2	1061.06	189	Glioma_grade2_2.raw
21361657			fIQENIFGIcPHmTEDNk	Unambiguous	N-Term(iTRAQ4plex); C10(Methylthio); M13(Oxidation); K18(iTRAQ4plex)	HCD	1.5738269	0	1	1	1.39496	1.633	1.5233	1.7832159	10.40880785	36388.926	31084.39	50761.137	55430.18	11.11809408	6.218045053	3	High	5790	Unique	Used	82743	1	59.078	1	7.461	-0.7771	1	27	30/68	16.11542	5790	2486.17	MS2	829.396	76	Glioma_grade2_6.raw
21361657			yGVSGYPTLk	Unambiguous	N-Term(iTRAQ4plex); K10(iTRAQ4plex)	HCD	1.5337399	0	1	1	1.48691	1.4092	1.6651	1.5781263	7.228859493	36325.703	38328.492	54012.918	60487.203	3.793988223	7.996517011	2	High	2797	Unique	Used	80158	1	33.106	1	3.2643	1.6331	1	100	14/18	32.11717	2797	1372.77	MS2	686.891	264	Glioma_grade2_6.raw
21361657			gFPTIYFSPANk	Unambiguous	N-Term(iTRAQ4plex); K12(iTRAQ4plex)	HCD	1.6943821	0	1	1	1.49132	1.5451	1.8384	1.9047765	12.21225321	29413.457	28389.1	43864.73	54074.887	2.50622055	14.74310697	3	High	5553	Unique	Used	61008	1	53.695	1	3.5459	-0.2773	1	9	18/44	23.94227	5553	1629.89	MS2	543.967	229	Glioma_grade2_3.raw
21361657			gFPTIYFSPANk	Unambiguous	N-Term(iTRAQ4plex); K12(iTRAQ4plex)	HCD	1.6327608	0	1	1	1.61808	1.5879	1.6785	1.6471658	2.378006385	26905.117	27416.361	43534.684	45159.293	1.330980351	2.590417252	3	High	5676	Unique	Used	53121	1	53.305	1	3.6538	-1.2884	1	10	17/44	37.25647	5676	1629.89	MS2	543.967	240	Glioma_grade2_2.raw
21361657			dASIVGFFDDSFSEAHSEFLk	Unambiguous	N-Term(iTRAQ4plex); K21(iTRAQ4plex)	HCD	2.8629549	0	1	1	3.12064	2.5283	3.2761	2.6541858	12.43304667	25857.969	31916.494	80693.375	84712.305	14.83016506	3.436172997	3	High	8108	Unique	Used	70949	1	74.847	1	7.9312	1.8779	1	8	35/80	7.195259	8108	2636.28	MS2	879.432	107	Glioma_grade2_4.raw
21361657			eATNPPVIQEEkPk	Unambiguous	N-Term(iTRAQ4plex); K12(iTRAQ4plex); K14(iTRAQ4plex)	HCD	3.0463479	0	1	1	3.21603	2.8846	3.2267	2.8941507	6.276353387	24584.428	27409.22	79064.33	79326.414	7.683360008	0.234005307	4	High	1085	Unique	Used	78599	1	19.812	1	4.7915	-1.1246	1	6	25/78	9.009563	1085	2012.14	MS2	503.79	150	Glioma_grade2_6.raw
21361657			fLQDYFDGNLk	Unambiguous	N-Term(iTRAQ4plex); K11(iTRAQ4plex)	HCD	2.8524418	0	1	1	2.87577	2.7857	2.9214	2.8298423	2.049600571	22630.266	23362.125	65079.49	66111.13	2.250382946	1.112091154	3	High	6407	Unique	Used	61805	1	60.314	1	4.7218	-0.273	1	27	23/40	6.509741	6407	1647.86	MS2	549.959	216	Glioma_grade2_3.raw
21361657			gFPTIYFSPANk	Unambiguous	N-Term(iTRAQ4plex); K12(iTRAQ4plex)	HCD	2.65592	0	1	1	2.49773	2.6657	2.6467	2.824752	5.031086607	22241.832	20840.154	55554.043	58868.27	4.60116216	4.096250678	3	High	5468	Unique	Used	60934	1	52.952	1	3.4735	0.0597	1	13	17/44	54.00592	5468	1629.89	MS2	543.968	232	Glioma_grade2_3.raw
21361657			dASIVGFFDDSFSEAHSEFLk	Unambiguous	N-Term(iTRAQ4plex); K21(iTRAQ4plex)	HCD	2.649696	0	1	1	2.56746	2.3435	2.9852	2.7247624	10.16592661	18771.262	20565.242	48194.383	56035.402	6.44966021	10.63887392	3	High	7646	Unique	Used	77711	1	73.898	1	7.7142	-0.5531	1	13	37/80	8.779999	7646	2636.27	MS2	879.43	101	Glioma_grade2_5.raw
21361657			gFPTIYFSPANk	Unambiguous	N-Term(iTRAQ4plex); K12(iTRAQ4plex)	HCD	2.55428	0	1	1	2.8044	2.4396	2.6861	2.3367007	8.410900447	11189.934	12863.217	31380.996	30057.486	9.838126873	3.046503968	3	High	5646	Unique	Used	68762	1	53.788	1	3.5104	-1.0637	1	18	19/44	4.755254	5646	1629.89	MS2	543.967	230	Glioma_grade2_4.raw
21361657			tFSHELSDFGLESTAGEIPVVAIR	Unambiguous	N-Term(iTRAQ4plex)	HCD	1.7144774	0	1	1	1.73329	2.1219	1.3817	1.6914513	17.52298522	10994.201	8980.817	19056.104	15190.614	14.25458019	15.96248839	2	High	7136	Unique	Used	70114	1	65.998	1	6.5435	-1.7922	1	27	29/46	5.945879	7136	2719.4	MS2	1360.2	139	Glioma_grade2_4.raw
21361657			dASIVGFFDDSFSEAHSEFLk	Unambiguous	N-Term(iTRAQ4plex); K21(iTRAQ4plex)	HCD	2.0352333	0	1	1	2.02367	1.7488	2.3667	2.0452235	12.35555678	10829.71	12532.2295	21915.79	25631.209	10.30619126	11.0509518	3	High	7837	Unique	Used	77865	1	75.749	1	6.9612	-1.1782	1	26	36/80	29.26731	7837	2636.27	MS2	879.429	104	Glioma_grade2_5.raw
21361657			fLQDYFDGNLk	Unambiguous	N-Term(iTRAQ4plex); K11(iTRAQ4plex)	HCD	1.6839993	0	1	1	1.64922	1.6785	1.6894	1.719397	1.721884534	9169.5	9009.716	15122.51	15491.277	1.243005748	1.703530807	3	High	6692	Unique	Used	54075	1	60.797	1	3.5913	0.8382	1	31	21/40	9.257303	6692	1647.86	MS2	549.959	209	Glioma_grade2_2.raw
21361657			dASIVGFFDDSFSEAHSEFLk	Unambiguous	N-Term(iTRAQ4plex); K21(iTRAQ4plex)	HCD	2.9714246	0	1	1	3.04511	2.9551	2.9882	2.8999128	2.045724264	8357.446	8611.953	25449.375	24973.914	2.121037116	1.333517523	2	High	7721	Unique	Used	77769	1	74.669	1	5.8711	-0.8489	1	53	24/40	4.908348	7721	2636.27	MS2	1318.64	119	Glioma_grade2_5.raw
21361657			dASIVGFFDDSFSEAHSEFLk	Unambiguous	N-Term(iTRAQ4plex); K21(iTRAQ4plex)	HCD	2.993911	0	1	1	2.46245	3.4348	2.6778	3.73523	19.66035245	8175.011	5860.7515	20130.568	21891.254	23.31798626	5.925459444	2	High	8123	Unique	Used	70961	1	74.992	1	6.0903	-1.4972	1	66	24/40	5.574625	8123	2636.27	MS2	1318.64	124	Glioma_grade2_4.raw
21361657			eATNPPVIQEEkPk	Unambiguous	N-Term(iTRAQ4plex); K12(iTRAQ4plex); K14(iTRAQ4plex)	HCD	1.7284552	0	1	1	1.68905	1.659	1.7992	1.7671598	3.791071609	7574.3237	7711.6465	12793.402	13627.712	1.270470658	4.465718278	2	High	1222	Unique	Used	78720	1	20.944	1	3.0858	-4.0233	1	100	14/26	0	1222	2012.13	MS2	1006.57	175	Glioma_grade2_6.raw
21361657			fLQDYFDGNLk	Unambiguous	N-Term(iTRAQ4plex); K11(iTRAQ4plex)	HCD	2.754411	0	1	1	2.91587	2.5156	3.0312	2.6151142	8.801307907	7494.481	8687.023	21852.955	22717.559	10.42244942	2.74337133	3	High	6509	Unique	Used	69551	1	60.735	1	4.7476	-0.3841	1	100	21/40	21.0867	6509	1647.86	MS2	549.959	214	Glioma_grade2_4.raw
21361657			dASIVGFFDDSFSEAHSEFLk	Unambiguous	N-Term(iTRAQ4plex); K21(iTRAQ4plex)	HCD	1.6503329	0	1	1	1.47267	1.8154	1.5164	1.8693459	12.15575984	6525.729	5293.6924	9610.239	9895.742	14.74152423	2.069940572	3	High	8276	Unique	Used	71097	1	76.251	1	6.2709	2.5724	1	73	32/80	17.95491	8276	2636.28	MS2	879.432	105	Glioma_grade2_4.raw

21361657			kTFSHELSDFGLESTAGEIPVVAIR	Unambiguous	N-Term(iTRAQ4plex); K1(iTRAQ4plex)	HCD	1.6266044	1	1	1	1.64835	1.8799	1.4045	1.601807	11.95041213	5571.2773	4885.1567	9183.402	7825.079	9.279655549	11.29412326	2	High	5404	Unique	Used	92522	1	59.746	1	4.8305	4.0796	1	89	22/48	0	5404	2991.62	MS2	1496.31	74	Glioma_grade2_7rep.raw
21361657			dASIVGFFDDSFSEAHSEFLk	Unambiguous	N-Term(iTRAQ4plex); K21(iTRAQ4plex)	HCD	1.6784526	0	1	1	1.36226	1.7359	1.6334	2.0813718	17.45634582	4527.3223	3552.8835	6167.371	7394.871	17.05482015	12.7998538	3	High	7269	Unique	Used	77409	1	70.189	1	7.4558	-1.9422	1	75	33/80	18.27026	7269	2636.27	MS2	879.428	95	Glioma_grade2_5.raw
21361657			dASIVGFFDDSFSEAHSEFLk	Unambiguous	N-Term(iTRAQ4plex); K21(iTRAQ4plex)	HCD	2.4393153	0	1	1	2.05896	2.1445	2.7223	2.8354843	16.19544383	3715.8892	3567.5906	7650.8564	10115.847	2.879473784	19.6211028	2	High	7657	Unique	Used	77721	1	73.984	1	5.2254	-2.2381	1	100	24/40	0	7657	2636.27	MS2	1318.64	120	Glioma_grade2_5.raw
21361657			dASIVGFFDDSFSEAHSEFLk	Unambiguous	N-Term(iTRAQ4plex); K21(iTRAQ4plex)	HCD	2.5638404	0	1	1	2.73362	2.3633	2.7957	2.4170544	8.488740709	3493.7932	4041.1829	9550.717	9767.759	10.27376766	1.588861047	3	High	8027	Unique	Used	78034	1	77.308	1	7.2924	-2.7062	1	72	33/80	36.35375	8027	2636.27	MS2	879.428	94	Glioma_grade2_5.raw
21361657			eLSDFISYLQR	Unambiguous	N-Term(iTRAQ4plex)	HCD	2.2365644	0	1	1	2.05392	2.413	2.0864	2.451147	9.327221749	3326.526	2831.4688	6832.4053	6940.3467	11.36923023	1.108363759	3	High	5406	Unique	Used	87709	1	69.842	1	3.5616	-1.9816	1	100	16/40	23.61045	5406	1514.79	MS2	505.603	201	Glioma_grade2_1rep.raw
21464101	NP_036611.2	YWHAG	14-3-3 protein gamma [Homo sapiens]	2	2	0.44528386	11.59713527	33.01119	22.67	6.82E-05	247	28.285	4.894	Adapter molecule	Cell communication,Signal transduction	Receptor signaling complex scaffold activity	Cytoplasm	Nucleus, Golgi apparatus, Endoplasmic reticulum																								
21464101			yLAEVATGEk	Unambiguous	N-Term(iTRAQ4plex); K10(iTRAQ4plex)	HCD	0.40876874	0	1	1	0.38529	0.4055	0.4119	0.43347797	0.048537789	452673.06	430159.3	174409.69	186464.58	3.606490448	4.724135342	2	High	2835	Unique	Used	50447	1	32.641	1	4.4107	-2.0053	1	13	16/18	18.91752	2835	1368.76	MS2	684.883	219	Glioma_grade2_2.raw
21464101			tAFDDAIAELDTLNEDSYk	Unambiguous	N-Term(iTRAQ4plex); K19(iTRAQ4plex)	HCD	0.48179898	0	1	1	0.4758	0.4812	0.4824	0.48786724	0.010284172	100501.85	99383.41	47818.605	48485.91	0.791310483	0.979924754	2	High	5932	Unique	Used	88098	1	75.653	1	6.4099	-0.0937	1	34	23/36	11.99354	5932	2419.18	MS2	1210.09	139	Glioma_grade2_1rep.raw
21536423	NP_647477.1	AMPH	amphiphysin isoform 2 [Homo sapiens]	8	18	0.349061251	28.32360571	100.70268	14.85	6.25E-05	653	71.885	4.729	Adapter molecule	Cell communication,Signal transduction	Receptor signaling complex scaffold activity	Cytoplasm	-																								
21536423			gYLAAIk	Unambiguous	N-Term(iTRAQ4plex); K7(iTRAQ4plex)	HCD	0.30281937	0	1	2	0.28394	0.3018	0.3038	0.32288384	0.05252936	655868.75	617038.3	186229.22	199231.7	4.314105183	4.770466371	2	High	2292	Unique	Used	49934	1	28.833	1	3.3268	-8.7217	1	12	12-Dec	54.70824	2292	1023.64	MS2	512.321	189	Glioma_grade2_2.raw
21536423			gYLAAIk	Unambiguous	N-Term(iTRAQ4plex); K7(iTRAQ4plex)	HCD	0.43095544	0	1	2	0.37306	0.424	0.4371	0.4967563	0.117502768	444812.47	391391.6	165940.44	194426.25	9.034698775	11.17889637	2	High	2326	Unique	Used	58009	1	28.874	1	3.141	-8.2447	1	16	12-Dec	50.47889	2326	1023.64	MS2	512.322	232	Glioma_grade2_3.raw
21536423			cDVLWEDFHQk	Unambiguous	N-Term(iTRAQ4plex); C1(Methylthio); K11(iTRAQ4plex)	HCD	0.2971213	0	1	2	0.30606	0.3093	0.285	0.28808922	0.041552386	432334.03	427733.44	132318.97	123225.38	0.756477486	5.032503481	3	High	5292	Unique	Used	82329	1	54.505	1	5.7627	0.4346	1	7	21/40	31.29141	5292	1753.83	MS2	585.281	71	Glioma_grade2_6.raw
21536423			vGFYVNTFk	Unambiguous	N-Term(iTRAQ4plex); K9(iTRAQ4plex)	HCD	0.3498093	0	1	2	0.29623	0.3389	0.3593	0.41110864	0.135760869	323888.78	283095.97	95945.71	116383.2	9.504315413	13.61235996	2	High	4836	Unique	Used	52330	1	47.21	1	4.2057	-5.1796	1	31	15/16	71.76746	4836	1362.76	MS2	681.883	287	Glioma_grade2_2.raw
21536423			eIAVLcHk	Unambiguous	N-Term(iTRAQ4plex); C6(Methylthio); K8(iTRAQ4plex)	HCD	0.27343383	0	1	2	0.28176	0.2751	0.2718	0.2653012	0.025073206	302402.3	309755.94	85206.15	82178.625	1.698844635	2.557918972	3	High	2412	Unique	Used	79831	1	29.693	1	4.2002	11.008	1	21	19/28	70.36919	2412	1246.7	MS2	416.239	257	Glioma_grade2_6.raw
21536423			vGFYVNTFk	Unambiguous	N-Term(iTRAQ4plex); K9(iTRAQ4plex)	HCD	0.34831318	0	1	2	0.34685	0.3234	0.375	0.34967536	0.060486447	125384.7	134466.14	43489.98	47019.492	4.942487626	5.514874442	2	High	4885	Unique	Used	68062	1	47.817	1	4.3852	-4.0151	1	100	15/16	58.50287	4885	1362.76	MS2	681.884	276	Glioma_grade2_4.raw
21536423			aDETkDEQFEEYVQNFk	Unambiguous	N-Term(iTRAQ4plex); K5(iTRAQ4plex); K17(iTRAQ4plex)	HCD	0.23328245	1	1	2	0.21007	0.251	0.2184	0.26101667	0.104996696	105289.55	88117.37	22118.334	23000.104	12.5564948	2.763861402	2	High	5090	Unique	Used	82148	1	52.867	1	5.5615	1.0586	1	33	19/32	0	5090	2552.25	MS2	1276.63	136	Glioma_grade2_6.raw
21536423			kGPPVPPLPk	Unambiguous	N-Term(iTRAQ4plex); K1(iTRAQ4plex); K10(iTRAQ4plex)	HCD	0.33395702	1	1	2	0.31004	0.3378	0.3304	0.36002025	0.061678246	94115.48	86373.3	29179.355	31096.137	6.066358229	4.497249231	3	High	2116	Unique	Used	94913	1	30.922	1	3.6941	10.691	1	51	14/36	57.20026	2116	1461.97	MS2	487.994	5	Glioma_grade2_8rep.raw
21536423			lTESLHEVYEPDWYGR	Unambiguous	N-Term(iTRAQ4plex)	HCD	0.31243965	0	1	2	0.29742	0.2905	0.3349	0.327114	0.069836837	78016.11	79878.36	23203.162	26129.33	1.66796165	8.388439958	2	High	5554	Unique	Used	61009	1	53.701	1	6.361	-0.6181	1	30	23/30	3.277584	5554	2138.03	MS2	1069.52	141	Glioma_grade2_3.raw
21536423			kGPPVPPLPk	Unambiguous	N-Term(iTRAQ4plex); K1(iTRAQ4plex); K10(iTRAQ4plex)	HCD	0.3772686	1	1	2	0.34619	0.4011	0.3567	0.41328716	0.086656795	68228.375	58880.375	23619.64	24334.502	10.40059664	2.108196484	3	High	2095	Unique	Used	94898	1	30.678	1	3.6715	-5.7159	1	72	15/36	53.51817	2095	1461.94	MS2	487.986	36	Glioma_grade2_8rep.raw
21536423			kGPPVPPLPk	Unambiguous	N-Term(iTRAQ4plex); K1(iTRAQ4plex); K10(iTRAQ4plex)	HCD	0.45945084	1	1	2	0.43016	0.4535	0.4651	0.49033746	0.05442429	62814.2	59577.23	27019.896	29212.947	3.740267497	5.515357755	3	High	1936	Unique	Used	85365	1	28.761	1	3.5227	12.007	1	64	15/36	64.56382	1936	1461.97	MS2	487.995	4	Glioma_grade2_1rep.raw
21536423			vETLHDFEAANSDELTLQR	Unambiguous	N-Term(iTRAQ4plex)	HCD	0.30981424	0	1	2	0.28732	0.2991	0.3201	0.33323342	0.066525855	53281.316	51183.15	15308.643	17055.936	2.84044415	7.635030439	3	High	5267	Unique	Used	60746	1	51.415	1	6.5071	1.6829	1	8	26/72	5.678033	5267	2332.16	MS2	778.057	139	Glioma_grade2_3.raw
21536423			kGPPVPPLPk	Unambiguous	N-Term(iTRAQ4plex); K1(iTRAQ4plex); K10(iTRAQ4plex)	HCD	0.6053887	1	1	2	0.52527	0.6238	0.5899	0.7005327	0.119627474	50486.24	42514.39	26518.81	29782.72	12.12238926	8.198473094	3	High	2045	Unique	Used	85448	1	29.907	1	3.2859	0.3587	1	100	13/36	68.64661	2045	1461.95	MS2	487.989	36	Glioma_grade2_1rep.raw
21536423			vETLHDFEAANSDELTLQR	Unambiguous	N-Term(iTRAQ4plex)	HCD	0.25714505	0	1	2	0.22818	0.2429	0.2705	0.28797868	0.104461152	45747.758	42968.895	10438.923	12374.125	4.429727238	11.99659473	2	High	5257	Unique	Used	60737	1	51.329	1	6.5487	0.6418	1	63	25/36	32.94667	5257	2332.16	MS2	1166.58	169	Glioma_grade2_3.raw
21536423			vETLHDFEAANSDELTLQR	Unambiguous	N-Term(iTRAQ4plex)	HCD	0.46154147	0	1	2	0.49919	0.4752	0.4472	0.42570618	0.06952091	41779.145	43889.746	20855.61	18684.137	3.484159214	7.766682389	3	High	5428	Unique	Used	52886	1	51.547	1	6.5319	-1.9288	1	11	25/72	18.26979	5428	2332.15	MS2	778.055	132	Glioma_grade2_2.raw
21536423			lTESLHEVYEPDWYGR	Unambiguous	N-Term(iTRAQ4plex)	HCD	0.35076207	0	1	2	0.38404	0.3701	0.3307	0.31868988	0.088820374	40488.516	42011.918	15549.249	13388.773	2.611399316	10.55833899	2	High	5729	Unique	Used	53170	1	53.717	1	6.3819	3.2643	1	69	22/30	13.3862	5729	2138.04	MS2	1069.52	156	Glioma_grade2_2.raw
21536423			lTESLHEVYEPDWYGR	Unambiguous	N-Term(iTRAQ4plex)	HCD	0.5702509	0	1	2	0.45399	0.5137	0.6203	0.7017913	0.192697333	20440.012	18065.303	9279.619	12678.071	8.72177172	21.8881718	3	High	5702	Unique	Used	53145	1	53.507	1	4.9069	-1.4523	1	30	22/60	18.6065	5702	2138.03	MS2	713.348	90	Glioma_grade2_2.raw
21536423			lTESLHEVYEPDWYGR	Unambiguous	N-Term(iTRAQ4plex)	HCD	0.5062938	0	1	2	0.51635	0.4456	0.5766	0.49761212	0.106212402	10461.084	12121.522	5401.6167	6031.817	10.3983302	7.795014469	3	High	5677	Unique	Used	68789	1	54.05	1	3.7941	0.4319	1	30	23/60	0	5677	2138.03	MS2	713.349	99	Glioma_grade2_4.raw
21735621	NP_005909.2	MDH2	malate dehydrogenase, mitochondrial precursor [Homo sapiens]	12	25	0.485153228	18.83269456	170.61012	46.75	1.05E-04	338	35.481	8.6753	Enzyme: Dehydrogenase	Metabolism,Energy pathways	Catalytic activity	Mitochondrion	Nucleolus, Extracellular																								
21735621			iQEAGTEVVk	Unambiguous	N-Term(iTRAQ4plex); K10(iTRAQ4plex)	HCD	0.5113125	0	1	1	0.50807	0.5071	0.5155	0.51455116	0.00844032	5446444	5456392.5	2767162.8	2807593	0.129042599	1.025640211	2	High	1405	Unique	Used	49094	1	22.71	1	4.4995	0.6217	1	2	18/18	21.73149	1405	1361.79	MS2	681.398	323	Glioma_grade2_2.raw
21735621			vNVPVIGGHAGk	Unambiguous	N-Term(iTRAQ4plex); K12(iTRAQ4plex)	HCD	0.35406414	0	1	1	0.35203	0.3576	0.3506	0.35612804	0.009353717	2836732	2792619.5	998621.4	994530.1	1.108200399	0.290292632	3	High	1633	Unique	Used	79106	1	23.886	1	5.3921	12.449	1	1	26/44	6.011527	1633	1435.88	MS2	479.298	80	Glioma_grade2_6.raw
21735621			vNVPVIGGHAGk	Unambiguous	N-Term(iTRAQ4plex); K12(iTRAQ4plex)	HCD	0.40500626	0	1	1	0.38604	0.3985	0.4113	0.42458054	0.040925051	1370554	1327696.9	529094.25	563714.25	2.246236333	4.480206141	2	High	1618	Unique	Used	79092	1	23.778	1	3.9941	12.374	1	7	15/22	6.649279	1618	1435.88	MS2	718.444	91	Glioma_grade2_6.raw
21735621			aNTFVAELk	Unambiguous	N-Term(iTRAQ4plex); K9(iTRAQ4plex)	HCD	0.48995298	0	1	1	0.4662	0.5172	0.4654	0.5163052	0.059846679	1290212.4	1163077.5	601493.7	600503	7.328766968	0.116561167	2	High	3386	Unique	Used	50964	1	36.63	1	3.7281	0.7543	1	8	15/16	46.851	3386	1280.75	MS2	640.877	238	Glioma_grade2_2.raw
21735621			mISDAIPELk	Unambiguous	N-Term(iTRAQ4plex); M1(Oxidation); K10(iTRAQ4plex)	HCD	0.5825893	0	1	1	0.53382	0.5762	0.5885	0.63523316	0.071519425	642151.9	594933.06	342791.3	377921.2	5.397973954	6.893342494	2	High	4143	Unique	Used	51677	1	42.154	1	3.4918	0.7985	1	14	16/18	45.59917	4143	1420.8	MS2	710.902	248	Glioma_grade2_2.raw
21735621			vNVPVIGGHAGk	Unambiguous	N-Term(iTRAQ4plex); K12(iTRAQ4plex)	HCD	0.5051735	0	1	1	0.45727	0.4809	0.5282	0.5555494	0.088114955	439660.66	418045.2	201045.52	232244.75	3.564027963	10.18309832	3	High	1562	Unique	Used	79040	1	23.369	1	5.4127	-6.2326	1	7	25/44	31.00611	1562	1435.85	MS2	479.289	127	Glioma_grade2_6.raw
21735621			iFGVTTLDIVR	Unambiguous	N-Term(iTRAQ4plex)	HCD	0.39679512	0	1	1	0.374	0.3991	0.3946	0.42112237	0.048666798	253357.34	237396.36	94755.75	99972.914	4.599503658	3.788956353	2	High	4712	Unique	Used	87189	1	62.351	1	4.3356	1.7116	1	30	16/20	32.96258	4712	1377.82	MS2	689.416	277	Glioma_grade2_1rep.raw
21735621			vNVPVIGGHAGk	Unambiguous	N-Term(iTRAQ4plex); K12(iTRAQ4plex)	HCD	0.5548412	0	1	1	0.49975	0.5285	0.5797	0.6131115	0.091479733	241589.45	228429.47	120733.14	140052.73	3.95963256	10.47680467	2	High	1567	Unique	Used	79045	1	23.397	1	4.9079	-6.6696	1	36	16/22	7.97324	1567	1435.85	MS2	718.43	171	Glioma_grade2_6.raw
21735621			eGVVEcSFVk	Unambiguous	N-Term(iTRAQ4plex); C6(Methylthio); K10(iTRAQ4plex)	HCD	0.4857437	0	1	1	0.49415	0.4853	0.4862	0.4774898	0.014017777	213188.16	217065.45	105346.445	103646.54	1.27443814	1.150291578	2	High	4870	Unique	Used	52362	1	47.452	1	4.1625	2.2292	1	82	18/18	36.60537	4870	1430.73	MS2	715.868	203	Glioma_grade2_2.raw
21735621			aNTFVAELk	Unambiguous	N-Term(iTRAQ4plex); K9(iTRAQ4plex)	HCD	0.4790323	0	1	1	0.47211	0.4473	0.5125	0.4855893	0.056664984	200876.6	212000.94	94836.29	102945.38	3.810377406	5.798305301	2	High	3300	Unique	Used	58920	1	36.103	1	3.9322	-2.2956	1	31	15/16	41.45035	3300	1280.74	MS2	640.875	318	Glioma_grade2_3.raw
21735621			lTLYDIAHTPGVAADLSHIETk	Unambiguous	N-Term(iTRAQ4plex); K22(iTRAQ4plex)	HCD	0.39342484	0	1	1	0.34308	0.3911	0.3955	0.4508157	0.11153381	193014.23	169311.5	66219.664	76328.28	9.25154342	10.02872538	3	High	5697	Unique	Used	82660	1	58.314	1	9.7475	2.2492	1	5	37/84	13.75046	5697	2653.45	MS2	885.155	66	Glioma_grade2_6.raw
21735621			gYLGPEQLPDcLk	Unambiguous	N-Term(iTRAQ4plex); C11(Methylthio); K13(iTRAQ4plex)	HCD	0.34775952	0	1	1	0.35047	0.3715	0.3254	0.34488344	0.054509393	104905.04	98969.41	36766.37	34132.91	4.117361664	5.252909265	2	High	6425	Unique	Used	53826	1	58.809	1	5.2301	-0.654	1	63	19/24	24.5727	6425	1766.9	MS2	883.956	179	Glioma_grade2_2.raw
21735621			lTLYDIAHTPGVAADLSHIETk	Unambiguous	N-Term(iTRAQ4plex); K22(iTRAQ4plex)	HCD	0.48515323	0	1	1	0.45951	0.4816	0.4886	0.5120318	0.044575815	54105.645	51627.535	24861.855	26434.94	3.314547781	4.336875514	3	High	5353	Unique	Used	92483	1	59.228	1	10.047	-1.8912	1	20	37/84	8.218488	5353	2653.44	MS2	885.151	71	Glioma_grade2_7rep.raw
21735621			lTLYDIAHTPGVAADLSHIETk	Unambiguous	N-Term(iTRAQ4plex); K22(iTRAQ4plex)	HCD	0.5318425	0	1	1	0.73207	0.502	0.5754	0.39454305	0.257221807	53114.246	77457.61	38883.305	30560.361	26.3661072	16.94959521	4	High	5365	Unique	Used	92492	1	59.353	1	6.7746	-1.4823	1	3	33/126	17.53092	5365	2653.44	MS2	664.116	79	Glioma_grade2_7rep.raw
21735621			tIIPLISQcTPk	Unambiguous	N-Term(iTRAQ4plex); C9(Methylthio); K12(iTRAQ4plex)	HCD	0.65145105	0	1	1	0.63017	0.6971	0.6102	0.67499495	0.061110437	53091.582	47995.688	33456.562	32396.848	7.12916909	2.275754454	2	High	6905	Unique	Used	54274	1	62.394	1	3.9715	1.4436	1	95	22-Nov	55.88948	6905	1647.94	MS2	824.476	227	Glioma_grade2_2.raw
21735621			gcDVVVIPAGVPR	Unambiguous	N-Term(iTRAQ4plex); C2(Methylthio)	HCD	0.64024186	0	1	1	0.54832	0.7293	0.5733	0.7625097	0.165629836	50303.766	37819.465	27582.473	28837.707	20.03497556	3.146336908	2	High	4134	Unique	Used	86786	1	55.739	1	4.2085	1.4238	1	75	17/24	32.34428	4134	1471.79	MS2	736.399	270	Glioma_grade2_1rep.raw
21735621			lTLYDIAHTPGVAADLSHIETk	Unambiguous	N-Term(iTRAQ4plex); K22(iTRAQ4plex)	HCD	0.63439864	0	1	1	0.53315	0.6578	0.6154	0.7593164	0.146708239	42589.332	34518.4	22706.656	26210.385	14.80269384	10.12943745	3	High	4708	Unique	Used	87186	1	62.306	1	9.8401	3.4913	1	29	38/84	25.23694	4708	2653.45	MS2	885.156	55	Glioma_grade2_1rep.raw
21735621			vAVLGASGGIGQPLSLLLk	Unambiguous	N-Term(iTRAQ4plex); K19(iTRAQ4plex)	HCD	0.36498177	0	1	1	0.38637	0.4066	0.3254	0.34247383	0.103155235	39952.676	37966.1	15436.538	13002.396	3.605604279	12.10452332	3	High	7761	Unique	Used	63038	1	71.385	1	5.7271	1.5874	1	3	28/72	7.747819	7761	2081.3	MS2	694.437	8	Glioma_grade2_3.raw
21735621			sQETEcTYFSTPLLLGk	Unambiguous	N-Term(iTRAQ4plex); C6(Methylthio); K17(iTRAQ4plex)	HCD	0.3749714	0	1	1	0.36525	0.3685	0.3814	0.3847781	0.025430953	35615.043	35297.973	13008.467	13581.888	0.632330592	3.049751518	2	High	7434	Unique	Used	54759	1	66.549	1	6.1234	0.4036	1	98	21/32	28.25561	7434	2251.12	MS2	1126.07	158	Glioma_grade2_2.raw
21735621			lTLYDIAHTPGVAADLSHIETk	Unambiguous	N-Term(iTRAQ4plex); K22(iTRAQ4plex)	HCD	0.5357552	0	1	1	0.42853	0.4672	0.5986	0.6526685	0.197764902	31953.895	29306.348	13693.163	19127.33	6.111952371	23.41546994	4	High	5690	Unique	Used	82654	1	58.253	1	6.3568	1.37	1	7	28/126	13.19497	5690	2653.45	MS2	664.117	72	Glioma_grade2_6.raw
21735621			vAVLGASGGIGQPLSLLLk	Unambiguous	N-Term(iTRAQ4plex); K19(iTRAQ4plex)	HCD	0.45553637	0	1	1	0.47768	0.3995	0.5225	0.43701595	0.11522965	26188.084	31310.016	12509.5	13682.977	12.59781749	6.335968486	2	High	7788	Unique	Used	70684	1	71.759	1	6.9189	-1.1627	1	100	27/36	16.95681	7788	2081.29	MS2	1041.15	10	Glioma_grade2_4.raw
21735621			lTLYDIAHTPGVAADLSHIETk	Unambiguous	N-Term(iTRAQ4plex); K22(iTRAQ4plex)	HCD	0.48307174	0	1	1	0.45848	0.4666	0.4993	0.50809836	0.050246212	19624.07	19284.545	8997.207	9798.446	1.234073379	6.028644285	2	High	5702	Unique	Used	82665	1	58.341	1	6.5868	2.8983	1	100	23/42	0	5702	2653.45	MS2	1327.23	83	Glioma_grade2_6.raw
21735621			lTLYDIAHTPGVAADLSHIETk	Unambiguous	N-Term(iTRAQ4plex); K22(iTRAQ4plex)	HCD	0.46241254	0	1	1	0.42058	0.5443	0.3992	0.5165413	0.151098806	17401.93	13447.703	7318.9624	6946.2944	18.12702748	3.694515614	3	High	4672	Unique	Used	96461	1	64.135	1	9.756	2.7322	1	64	35/84	20.5093	4672	2653.45	MS2	885.155	55	Glioma_grade2_8rep.raw
21735621			lTLYDIAHTPGVAADLSHIETk	Unambiguous	N-Term(iTRAQ4plex); K22(iTRAQ4plex)	HCD	0.4377747	0	1	1	0.50652	0.5061	0.3693	0.36908123	0.18083624	10971.496	10979.482	5557.2617	4052.3208	0.051450598	22.14776585	2	High	5386	Unique	Used	92508	1	59.567	1	6.267	-4.0944	1	90	25/42	35.82138	5386	2653.43	MS2	1327.22	95	Glioma_grade2_7rep.raw
21735621			vAVLGASGGIGQPLSLLLk	Unambiguous	N-Term(iTRAQ4plex); K19(iTRAQ4plex)	HCD	0.6239887	0	1	1	0.653	0.5211	0.7529	0.60083836	0.153773723	5428.446	6802.4507	3544.7678	4087.173	15.88711056	10.05087448	3	High	7780	Unique	Used	70677	1	71.689	1	7.0145	-2.5475	1	75	26/72	18.15984	7780	2081.29	MS2	694.434	9	Glioma_grade2_4.raw
22035577	NP_665798.1	SEPT6	septin-6 isoform A [Homo sapiens]	2	2	0.383820891	3.102549505	33.368869	14.52	2.64E-04	427	48.842	6.8149	GTPase	Biological_process unknown	Molecular function unknown	Cytoplasmic vesicle	-																								
22035577			tVPLAGHVGFDSLPDQLVNk	Unambiguous	N-Term(iTRAQ4plex); K20(iTRAQ4plex)	HCD	0.39224127	0	1	3	0.35024	0.3423	0.4434	0.43329224	0.136142381	41389.47	42350.516	14496.128	18350.15	1.623029036	16.59369193	3	High	5464	Unique	Used	75838	1	54.59	1	6.5622	-1.3398	1	27	23/76	34.37816	5464	2395.32	MS2	799.111	112	Glioma_grade2_5.raw
22035577			kTAAELLQSQGSQAGGSQTLk	Unambiguous	N-Term(iTRAQ4plex); K1(iTRAQ4plex); K21(iTRAQ4plex)	HCD	0.37540048	1	1	3	0.34169	0.4443	0.3224	0.4192361	0.154552473	13850.48	10650.739	4732.595	4465.1743	18.46894686	4.111757628	2	High	2238	Unique	Used	79667	1	28.429	1	4.6885	7.9463	1	100	23/40	0	2238	2535.43	MS2	1268.22	74	Glioma_grade2_6.raw

22748667	NP_689509.1	ATP1A3	sodium/potassium-transporting ATPase subunit alpha-3 isoform 1 [Homo sapiens]	17	89	0.444973201	20.66990138	880.52272	39.49	9.95E-05	1013	111.68	5.3765	Transport/cargo protein	Transport	Transporter activity	Plasma membrane	-																								
22748667			gGQDNIPVLk	Unambiguous	N-Term(iTRAQ4plex); K10(iTRAQ4plex)	HCD	0.29535964	0	1	3	0.28156	0.3031	0.2882	0.3102104	0.044616488	1.23E+07	1.14E+07	3461495.5	3542459	5.213766076	1.634786173	2	High	1870	Unique	Used	49535	1	25.882	1	3.9207	0.6191	1	0	17/18	8.012754	1870	1328.78	MS2	664.893	201	Glioma_grade 2_2.raw
22748667			acVIHGTDLk	Unambiguous	N-Term(iTRAQ4plex); C2(Methylthio); K10(iTRAQ4plex)	HCD	0.38866347	0	1	3	0.39327	0.361	0.4188	0.3844385	0.061268825	5219788	5685945	2052763.9	2185896.2	6.044944907	4.441910882	3	High	1687	Unique	Used	79157	1	24.265	1	3.8895	12.148	1	0	17/36	7.876094	1687	1390.76	MS2	464.258	201	Glioma_grade 2_6.raw
22748667			cIELSSGSVk	Unambiguous	N-Term(iTRAQ4plex); C1(Methylthio); K10(iTRAQ4plex)	HCD	0.33102703	0	1	3	0.30903	0.3315	0.3306	0.35462224	0.056184157	2477982	2309811.2	765778.56	819110.44	4.967412255	4.75886122	2	High	2939	Unique	Used	50544	1	33.411	1	4.125	0.3972	1	4	16/18	19.80236	2939	1356.71	MS2	678.859	189	Glioma_grade 2_2.raw
22748667			iATLASGLEVGk	Unambiguous	N-Term(iTRAQ4plex); K12(iTRAQ4plex)	HCD	0.39724654	0	1	3	0.37656	0.4238	0.3737	0.42052254	0.068230756	2349143.5	2087534.2	884599.7	877855.2	8.338929377	0.541186238	2	High	4757	Unique	Used	52254	1	46.686	1	5.3099	0.3827	1	3	20/22	13.37815	4757	1446.88	MS2	723.942	176	Glioma_grade 2_2.raw
22748667			sPDcTHDNPLETR	Unambiguous	N-Term(iTRAQ4plex); C4(Methylthio)	HCD	0.39024726	0	1	3	0.41111	0.3996	0.3806	0.36997274	0.047374384	2027926.2	2086370	833692.75	771900	2.008898013	5.442734162	3	High	2189	Unique	Used	57881	1	27.858	1	4.3055	12.704	1	0	25/48	10.14285	2189	1674.75	MS2	558.923	53	Glioma_grade 2_3.raw
22748667			sPDcTHDNPLETR	Unambiguous	N-Term(iTRAQ4plex); C4(Methylthio)	HCD	0.3388542	0	1	3	0.30272	0.345	0.3335	0.38002616	0.093822647	1806403.6	1585228.5	546840.44	602428.3	9.222368962	6.840271799	2	High	2190	Unique	Used	57882	1	27.861	1	4.1701	13.732	1	3	20/24	3.35312	2190	1674.76	MS2	837.882	63	Glioma_grade 2_3.raw
22748667			cSTILLQGk	Unambiguous	N-Term(iTRAQ4plex); C1(Methylthio); K9(iTRAQ4plex)	HCD	0.35153514	0	1	3	0.30533	0.3551	0.3484	0.40527156	0.115801474	1564088.4	1344770.8	477568.88	544997.4	10.66266543	9.325393311	2	High	4102	Unique	Used	59664	1	42.325	1	3.8161	1.4578	1	5	15/16	13.81441	4102	1296.73	MS2	648.868	247	Glioma_grade 2_3.raw
22748667			sPDcTHDNPLETR	Unambiguous	N-Term(iTRAQ4plex); C4(Methylthio)	HCD	0.33626157	0	1	3	0.31234	0.3597	0.3159	0.36381325	0.081596182	1345976.1	1168856.1	420396.06	425245.34	9.960326823	0.810972304	2	High	2198	Unique	Used	49845	1	28.178	1	4.3439	1.1953	1	3	20/24	3.821128	2198	1674.74	MS2	837.871	37	Glioma_grade 2_2.raw
22748667			sPDcTHDNPLETR	Unambiguous	N-Term(iTRAQ4plex); C4(Methylthio)	HCD	0.52451247	0	1	3	0.453	0.4895	0.5569	0.60179234	0.127036623	1255746.4	1162094.2	568848.25	699339.4	5.477789205	14.55165993	3	High	2229	Unique	Used	49874	1	28.401	1	4.6114	-0.0883	1	1	24/48	16.87262	2229	1674.73	MS2	558.916	30	Glioma_grade 2_2.raw
22748667			cIELSSGSVk	Unambiguous	N-Term(iTRAQ4plex); C1(Methylthio); K10(iTRAQ4plex)	HCD	0.4319751	0	1	3	0.43006	0.4307	0.4332	0.43389496	0.004334273	1110347.5	1108646.6	477513.97	481036.16	0.108402084	0.519652412	2	High	2834	Unique	Used	50446	1	32.635	1	4.023	-1.2224	1	11	17/18	51.79389	2834	1356.71	MS2	678.858	167	Glioma_grade 2_2.raw
22748667			cSTILLQGk	Unambiguous	N-Term(iTRAQ4plex); C1(Methylthio); K9(iTRAQ4plex)	HCD	0.45152128	0	1	3	0.39601	0.4586	0.4454	0.5157995	0.108487422	788054.2	680529.75	312079.97	351016.94	10.35436452	8.304244858	2	High	4234	Unique	Used	51763	1	42.813	1	3.9687	3.1523	1	14	15/16	28.55013	4234	1296.73	MS2	648.869	289	Glioma_grade 2_2.raw
22748667			gGQDNIPVLk	Unambiguous	N-Term(iTRAQ4plex); K10(iTRAQ4plex)	HCD	0.405151	0	1	3	0.45076	0.3764	0.4395	0.36706126	0.104780114	786132.44	941356.25	354358.38	345535.4	12.70744165	1.782781664	2	High	1831	Unique	Used	57549	1	25.179	1	3.8236	1.2622	1	12	16/18	41.8425	1831	1328.78	MS2	664.893	197	Glioma_grade 2_3.raw
22748667			yNTDcVQGLTHSk	Unambiguous	N-Term(iTRAQ4plex); C5(Methylthio); K13(iTRAQ4plex)	HCD	0.42556986	0	1	3	0.31381	0.4218	0.4283	0.57580495	0.247375145	696410.7	518061.66	218540.38	298302.47	20.76816563	21.82493759	3	High	3324	Unique	Used	66618	1	35.58	1	5.7211	1.2495	1	1	31/48	14.77758	3324	1799.87	MS2	600.628	79	Glioma_grade 2_4.raw
22748667			iATLASGLEVGk	Unambiguous	N-Term(iTRAQ4plex); K12(iTRAQ4plex)	HCD	0.53534585	0	1	3	0.53475	0.4866	0.5889	0.5358902	0.07786732	551748.44	606318.1	295046.16	324919.97	6.663965377	6.814557315	2	High	4630	Unique	Used	60153	1	46.47	1	5.4544	-4.0044	1	23	20/22	38.7648	4630	1446.87	MS2	723.939	174	Glioma_grade 2_3.raw
22748667			sSHTWVALSHIAGLcNR	Unambiguous	N-Term(iTRAQ4plex); C15(Methylthio)	HCD	0.27067053	0	1	3	0.27616	0.2588	0.2833	0.26552424	0.040176453	459689.75	490448.72	126948.48	130226.02	4.578254003	1.802333248	3	High	4971	Unique	Used	92193	1	55.332	1	6.384	-1.8604	1	2	31/64	14.14204	4971	2042.01	MS2	681.343	111	Glioma_grade 2_7rep.raw
22748667			gGQDNIPVLk	Unambiguous	N-Term(iTRAQ4plex); K10(iTRAQ4plex)	HCD	0.62243265	0	1	3	0.57154	0.6211	0.6237	0.67773795	0.069588797	448818.1	413029.66	256516.31	279925.88	5.872556567	6.171425738	2	High	1770	Unique	Used	49440	1	25.199	1	3.9686	-2.6881	1	20	17/18	33.59318	1770	1328.77	MS2	664.891	205	Glioma_grade 2_2.raw
22748667			sPDcTHDNPLETR	Unambiguous	N-Term(iTRAQ4plex); C4(Methylthio)	HCD	0.4956676	0	1	3	0.48471	0.499	0.4924	0.5069452	0.019095046	425056.25	412877.7	206030.36	209306.36	2.055421025	1.115471714	3	High	2372	Unique	Used	65729	1	28.42	1	4.3176	-2.603	1	2	21/48	40.59288	2372	1674.73	MS2	558.915	23	Glioma_grade 2_4.raw
22748667			cSTILLQGk	Unambiguous	N-Term(iTRAQ4plex); C1(Methylthio); K9(iTRAQ4plex)	HCD	0.44284025	0	1	3	0.43344	0.4064	0.4816	0.45165068	0.071280495	416657.97	444331.22	180598.17	200682.5	4.545456078	7.44950744	2	High	4007	Unique	Used	59575	1	41.597	1	3.9889	-2.5901	1	20	15/16	19.3819	4007	1296.72	MS2	648.865	251	Glioma_grade 2_3.raw
22748667			cSTILLQGk	Unambiguous	N-Term(iTRAQ4plex); C1(Methylthio); K9(iTRAQ4plex)	HCD	0.48589832	0	1	3	0.45439	0.4562	0.5155	0.51753205	0.072781897	400884.47	399304	182157.62	206652.62	0.279324458	8.909529032	2	High	4112	Unique	Used	51648	1	41.924	1	4.1288	-6.8263	1	28	15/16	5.51613	4112	1296.72	MS2	648.862	226	Glioma_grade 2_2.raw
22748667			sPDcTHDNPLETR	Unambiguous	N-Term(iTRAQ4plex); C4(Methylthio)	HCD	0.5260273	0	1	3	0.53525	0.5103	0.5426	0.51723766	0.028674041	391325.84	410479.8	209456.08	212315.61	3.378348649	0.958809281	2	High	2375	Unique	Used	65732	1	28.437	1	4.4422	-1.2101	1	16	17/24	44.75745	2375	1674.73	MS2	837.869	30	Glioma_grade 2_4.raw
22748667			yNTDcVQGLTHSk	Unambiguous	N-Term(iTRAQ4plex); C5(Methylthio); K13(iTRAQ4plex)	HCD	0.44229272	0	1	3	0.42567	0.4953	0.3968	0.46163192	0.0963011	373390	320915	158941.45	148144.61	10.68850962	4.972234024	2	High	3326	Unique	Used	66620	1	35.589	1	6.0973	4.1394	1	21	21/24	0	3326	1799.87	MS2	900.44	104	Glioma_grade 2_4.raw
22748667			kYNTDcVQGLTHSk	Unambiguous	N-Term(iTRAQ4plex); K1(iTRAQ4plex); C6(Methylthio); K14(iTRAQ4plex)	HCD	0.37533307	1	1	3	0.34526	0.3463	0.4042	0.4054994	0.090866975	373232.94	372063.78	128862.87	150871.64	0.221850155	11.12665757	3	High	2034	Unique	Used	89859	1	26.887	1	7.1398	-0.6581	1	3	29/52	11.36494	2034	2072.06	MS2	691.359	147	Glioma_grade 2_7rep.raw
22748667			cSTILLQGk	Unambiguous	N-Term(iTRAQ4plex); C1(Methylthio); K9(iTRAQ4plex)	HCD	0.3869076	0	1	3	0.29388	0.3946	0.3812	0.5118233	0.22652711	359831.9	267981.44	105746.625	137159.12	20.69025265	18.28856557	3	High	4160	Unique	Used	51693	1	42.277	1	3.3189	-1.5649	1	5	14/32	14.70265	4160	1296.72	MS2	432.913	219	Glioma_grade 2_2.raw
22748667			yNTDcVQGLTHSk	Unambiguous	N-Term(iTRAQ4plex); C5(Methylthio); K13(iTRAQ4plex)	HCD	0.46955618	0	1	3	0.4772	0.457	0.4827	0.4622379	0.025832859	339936	354967.47	162216.8	164079.42	3.059088011	0.807285621	2	High	2829	Unique	Used	73484	1	33.078	1	5.9542	-0.8794	1	21	21/24	4.664068	2829	1799.86	MS2	900.436	92	Glioma_grade 2_5.raw
22748667			iATLASGLEVGk	Unambiguous	N-Term(iTRAQ4plex); K12(iTRAQ4plex)	HCD	0.45954674	0	1	3	0.42513	0.4652	0.4543	0.497211	0.064653933	321945.34	294189.6	136868.5	146274.31	6.370770653	4.697920483	2	High	4731	Unique	Used	60247	1	47.251	1	5.3623	-2.4858	1	25	21/22	15.4499	4731	1446.87	MS2	723.94	180	Glioma_grade 2_3.raw
22748667			dFTSEQIDEILQNHTEIVFAR	Unambiguous	N-Term(iTRAQ4plex)	HCD	0.41548532	0	1	3	0.40029	0.5324	0.3276	0.43570015	0.200776659	320343.6	240859.44	128229.125	104942.49	20.02975199	14.12362094	3	High	7709	Unique	Used	55007	1	68.809	1	8.3513	-1.2601	1	1	40/80	3.009529	7709	2649.32	MS2	883.78	105	Glioma_grade 2_2.raw
22748667			sPDcTHDNPLETR	Unambiguous	N-Term(iTRAQ4plex); C4(Methylthio)	HCD	0.36972472	0	1	3	0.33325	0.3676	0.3716	0.40996167	0.084667002	317653.06	287934.78	105858.56	118042.23	6.940032783	7.695511639	2	High	2104	Unique	Used	57805	1	27.157	1	3.9564	13.951	1	17	18/24	12.28187	2104	1674.76	MS2	837.882	63	Glioma_grade 2_3.raw
22748667			yQLSIHETEDPNDNR	Unambiguous	N-Term(iTRAQ4plex)	HCD	0.52046573	0	1	3	0.51718	0.4801	0.564	0.5235142	0.065931725	279127.44	300700.9	144359.58	157421.2	5.261812439	6.120973029	2	High	1875	Unique	Used	49540	1	25.907	1	5.5181	-1.4209	1	14	22/28	0	1875	1974.92	MS2	987.966	34	Glioma_grade 2_2.raw
22748667			acVIHGTDLk	Unambiguous	N-Term(iTRAQ4plex); C2(Methylthio); K10(iTRAQ4plex)	HCD	0.4347117	0	1	3	0.4594	0.4285	0.4413	0.41168344	0.046340334	245678.17	263366.53	112864.04	108423.64	4.914130057	2.837787401	2	High	1674	Unique	Used	79145	1	24.165	1	3.4135	9.7881	1	16	15/18	6.613288	1674	1390.76	MS2	695.882	234	Glioma_grade 2_6.raw
22748667			yQLSIHETEDPNDNR	Unambiguous	N-Term(iTRAQ4plex)	HCD	0.53013515	0	1	3	0.44177	0.455	0.6031	0.6211459	0.17912196	221725.31	215284.36	97951.195	133723	2.084365512	21.83625664	3	High	1872	Unique	Used	49537	1	25.89	1	4.0053	-0.2842	1	2	26/56	7.680771	1872	1974.93	MS2	658.98	57	Glioma_grade 2_2.raw
22748667			yQLSIHETEDPNDNR	Unambiguous	N-Term(iTRAQ4plex)	HCD	0.64332616	0	1	3	0.73365	0.6487	0.6372	0.5634596	0.10795304	215984.98	244266.14	158457.48	137634.1	8.689951701	9.945810148	3	High	1969	Unique	Used	49629	1	26.573	1	4.7915	-0.8405	1	2	25/56	4.183539	1969	1974.93	MS2	658.98	47	Glioma_grade 2_2.raw
22748667			eAFQNAYLELGGLGER	Unambiguous	N-Term(iTRAQ4plex)	HCD	0.42669752	0	1	4	0.41736	0.4284	0.425	0.4362868	0.018392713	199975.48	194820.97	83460.84	84997.82	1.84641426	1.290297549	2	High	4685	Unique	Used	87170	1	62.037	1	6.3353	0.5182	1	33	22/30	31.77921	4685	1910.97	MS2	955.991	223	Glioma_grade 2_1rep.raw
22748667			vLGFcHYYLPEEQFPk	Unambiguous	N-Term(iTRAQ4plex); C5(Methylthio); K16(iTRAQ4plex)	HCD	0.46987852	0	1	3	0.45191	0.538	0.4126	0.4912722	0.113392206	186051.62	156275.78	84078.34	76773.95	12.30091332	6.422020727	3	High	6388	Unique	Used	83222	1	64.883	1	5.619	-0.8209	1	4	24/60	13.49884	6388	2304.14	MS2	768.719	167	Glioma_grade 2_6.raw
22748667			kYNTDcVQGLTHSk	Unambiguous	N-Term(iTRAQ4plex); K1(iTRAQ4plex); C6(Methylthio); K14(iTRAQ4plex)	HCD	0.44596797	1	1	3	0.43725	0.4351	0.4569	0.45464167	0.025477799	180070.25	180948.73	78736.07	82266.836	0.344125489	3.101345986	2	High	2031	Unique	Used	89857	1	26.847	1	6.0704	-1.9477	1	73	23/26	9.665175	2031	2072.06	MS2	1036.53	178	Glioma_grade 2_7rep.raw
22748667			yNTDcVQGLTHSk	Unambiguous	N-Term(iTRAQ4plex); C5(Methylthio); K13(iTRAQ4plex)	HCD	0.4881919	0	1	3	0.45676	0.4841	0.4921	0.52150387	0.054526298	173003.84	163246.61	79021.01	85133.74	4.103728931	5.266192827	3	High	2916	Unique	Used	73564	1	33.752	1	4.8714	1.2495	1	6	26/48	33.90569	2916	1799.87	MS2	600.628	97	Glioma_grade 2_5.raw
22748667			sSHTWVALSHIAGLcNR	Unambiguous	N-Term(iTRAQ4plex); C15(Methylthio)	HCD	0.3231328	0	1	3	0.31848	0.3279	0.3185	0.32792535	0.016882833	168457.12	163602.1	53649.95	53649.273	2.067714045	0.000892292	2	High	4965	Unique	Used	92188	1	55.274	1	6.3684	-1.6147	1	19	21/32	0	4965	2042.01	MS2	1021.51	145	Glioma_grade 2_7rep.raw
22748667			vLGFcHYYLPEEQFPk	Unambiguous	N-Term(iTRAQ4plex); C5(Methylthio); K16(iTRAQ4plex)	HCD	0.44753265	0	1	3	0.36205	0.4459	0.4488	0.55282587	0.172650785	148923.6	120905.35	53917.66	66839.61	14.68478054	15.13316502	3	High	6490	Unique	Used	83302	1	65.898	1	5.1357	1.4836	1	4	23/60	11.63865	6490	2304.15	MS2	768.721	166	Glioma_grade 2_6.raw
22748667			cSTILLQGk	Unambiguous	N-Term(iTRAQ4plex); C1(Methylthio); K9(iTRAQ4plex)	HCD	0.41640753	0	1	3	0.38247	0.389	0.4434	0.4509238	0.085706024	131650.17	129443.46	50352.223	58369.137	1.1952644	10.42815184	3	High	4019	Unique	Used	59586	1	41.689	1	3.3897	-4.9539	1	21	15/32	44.13545	4019	1296.72	MS2	432.911	214	Glioma_grade 2_3.raw
22748667			iATLASGLEVGk	Unambiguous	N-Term(iTRAQ4plex); K12(iTRAQ4plex)	HCD	0.39827853	0	1	3	0.41088	0.4422	0.3574	0.38471207	0.090894094	129467.945	120288.81	53196.2	46276.56	5.197560005	9.837717114	3	High	4679	Unique	Used	60199	1	46.846	1	3.9591	1.8921	1	5	21/44	11.61507	4679	1446.88	MS2	482.965	146	Glioma_grade 2_3.raw
22748667			vLGFcHYYLPEEQFPk	Unambiguous	N-Term(iTRAQ4plex); C5(Methylthio); K16(iTRAQ4plex)	HCD	0.51445997	0	1	3	0.50683	0.5116	0.5173	0.52216226	0.012963414	122733.39	121582.5	62204.95	63485.79	0.666188452	1.441141407	2	High	6393	Unique	Used	83227	1	64.91	1	5.4491	-0.6297	1	47	16/30	4.490551	6393	2304.14	MS2	1152.57	159	Glioma_grade 2_6.raw
22748667			rDVAGDASESALLk	Unambiguous	N-Term(iTRAQ4plex); K14(iTRAQ4plex)	HCD	0.3461541	1	1	3	0.30829	0.3402	0.3516	0.3879398	0.094736471	115828.59	104965.98	35708.46	40720.48	6.95762146	9.274061185	2	High	1392	Unique	Used	72157	1	22.307	1	4.656	0.0933	1	32	22/26	0	1392	1719.95	MS2	860.478	216	Glioma_grade 2_5.raw
22748667			yQLSIHETEDPNDNR	Unambiguous	N-Term(iTRAQ4plex)	HCD	0.5232493	0	1	3	0.44484	0.4823	0.561	0.60826087	0.141400847	111198.375	102562.81	49465.445	62384.95	5.713166842	16.3351584	2	High	1982	Unique	Used	49642	1	26.644	1	4.8896	-1.05	1	13	21/28	0	1982	1974.93	MS2	987.966	39	Glioma_grade 2_2.raw
22748667			sPDcTHDNPLETR	Unambiguous	N-Term(iTRAQ4plex); C4(Methylthio)	HCD	0.5098849	0	1	3	0.49493	0.5084	0.5113	0.5252465	0.024385624	93869.66	91384.99	46458.902	47999.645	1.896769669	2.306768118	2	High	2096	Unique	Used	49750	1	27.433	1	4.8464	1.414	1	100	17/24	0	2096	1674.74	MS2	837.871	40	Glioma_grade 2_2.raw
22748667			rDVAGDASESALLk	Unambiguous	N-Term(iTRAQ4plex); K14(iTRAQ4plex)	HCD	0.35124338	1	1	3	0.35643	0.4251	0.2893	0.34505367	0.157595909	93864.06	78693.14	33456.215	27153.357	12.43351238	14.70656692	3	High	1406	Unique	Used	72168	1	22.446	1	6.9716	-1.1211	1	5	27/52	16.40505	1406	1719.95	MS2	573.987	270	Glioma_grade 2_5.raw
22748667			kYNTDcVQGLTHSk	Unambiguous	N-Term(iTRAQ4plex); K1(iTRAQ4plex); C6(Methylthio); K14(iTRAQ4plex)	HCD	0.3224775	1	1	3	0.31265	0.3192	0.3257	0.3325163	0.026435412	85899.68	84127.336	26856.176	27973.71	1.474161566	2.882427549	2	High	2498	Unique	Used	90251	1	31.025	1	3.9371	7.7139	1	100	15/26	0	2498	2072.08	MS2	1036.54	204	Glioma_grade 2_7rep.raw
22748667			yNTDcVQGLTHSk	Unambiguous	N-Term(iTRAQ4plex); C5(Methylthio); K13(iTRAQ4plex)	HCD	0.4561774	0	1	3	0.41149	0.4479	0.4638	0.5048241	0.084628322	79165.58	72728.5	32575.545	36715.1	5.993259144	8.448780962	3	High	2999	Unique	Used	90676	1	35.443	1	5.9954	-3.532	1	26	25/48	33.2733	2999	1799.86	MS2	600.625	73	Glioma_grade 2_7rep.raw
22748667			sPDcTHDNPLETR	Unambiguous	N-Term(iTRAQ4plex); C4(Methylthio)	HCD	0.46876046	0	1	3	0.57076	0.5189	0.4136	0.37602744	0.192574844	76655.31	84312.79	43751.56	31703.92	6.727613775	22.58011729	3	High	2123	Unique	Used	49774	1	27.659	1	3.2403	0.021	1	5	22/48	18.50961	2123	1674.73	MS2	558.916	31	Glioma_grade 2_2.raw
22748667			sPDcTHDNPLETR	Unambiguous	N-Term(iTRAQ4plex); C4(Methylthio)	HCD	0.32635233	0	1	3	0.26993	0.2723	0.38	0.38326514	0.19558702	69472.51	68875.96	18752.59	26397.756	0.609800094	23.9464332	3	High	2103	Unique	Used	57804	1	27.151	1	3.7172	12.594	1	7	22/48	30.3473	2103	1674.75	MS2	558.923	56	Glioma_grade 2_3.raw
22748667			dFTSEQIDEILQNHTEIVFAR	Unambiguous	N-Term(iTRAQ4plex)	HCD	0.58732736	0	1	3	0.67507	0.6259	0.5457	0.50597274	0.12998404	68573.6	73959.95	46292.137	37421.72	5.344320142	14.98517029	3	High	7806	Unique	Used	55096	1	69.588	1	6.6499	4.5455	1	6	27/80	33.76381	7806	2649.34	MS2	883.785	114	Glioma_grade 2_2.raw
22748667			dFTSEQIDEILQNHTEIVFAR	Unambiguous	N-Term(iTRAQ4plex)	HCD	0.32214537	0	1	3	0.25397	0.3385	0.3099	0.4130102	0.201278429	68390.35	51315.156	17368.89	21193.684	20.17281556	14.02674922	3	High	7623	Unique	Used	54929	1	68.113	1	9.2104	2.3339	1	10	43/80	5.242705	7623	2649.33	MS2	883.783	100	Glioma_grade 2_2.raw
22748667			yNTDcVQGLTHSk	Unambiguous	N-Term(iTRAQ4plex); C5(Methylthio); K13(iTRAQ4plex)	HCD	0.5451687	0	1	3	0.52362	0.4968	0.5961	0.56561494	0.080672542	66314.49	69888.336	34723.633	39529.887	3.710775783	9.153868518	3	High	2803	Unique	Used	73460	1	32.883	1	6.7754	-1.7008	1	35	29/48	42.93859	2803	1799.86	MS2	600.626	71	Glioma_grade 2_5.raw
22748667			vLGFcHYYLPEEQFPk	Unambiguous	N-Term(iTRAQ4plex); C5(Methylthio); K16(iTRAQ4plex)	HCD	0.57373124	0	1	3	0.47188	0.4999	0.6434	0.6816283	0.18078546	59798.496	56448.664	28217.623	38477.004	4.075263298	21.75430976	3	High	5888	Unique	Used	92863	1	65.071	1	5.6005	0.4506	1	17	21/60	32.5304	5888	2304.14	MS2	768.72	164	Glioma_grade 2_7rep.raw
22748667			iATLASGLEVGk	Unambiguous	N-Term(iTRAQ4plex); K12(iTRAQ4plex)	HCD	0.4661376	0	1	3	0.46994	0.4276	0.5085	0.46268234	0.071074833	51476.746	56578.246	24190.736	26177.756	6.676795172	5.579024746	3	High	4635	Unique	Used	60158	1	46.503	1	4.0463	-9.4977	1	43	20/44	45.7887	4635	1446.86	MS2	482.959	198	Glioma_grade 2_3.raw

22748667			dFTSEQIDEILQNHTEIVFAR	Unambiguous	N-Term(iTRAQ4plex)	HCD	0.40918592	0	1	3	0.35915	0.3643	0.4534	0.4599399	0.134226084	48688.203	47998.758	17486.4	22076.543	1.008432222	16.40788574	3	High	7923	Unique	Used	55205	1	70.491	1	8.7543	3.025	1	12	38/80	21.17365	7923	2649.34	MS2	883.783	109	Glioma_grade2_2.raw
22748667			kYNTDcVQGLTHSk	Unambiguous	N-Term(iTRAQ4plex); K1(iTRAQ4plex); C6(Methylthio); K14(iTRAQ4plex)	HCD	0.3432108	1	1	3	0.26859	0.3	0.3819	0.42656106	0.211318842	46363.83	41510.3	12452.672	17706.678	7.811090649	24.63675955	4	High	2335	Unique	Used	85654	1	33.21	1	3.8807	1.3973	1	8	28/78	24.37306	2335	2072.07	MS2	518.772	129	Glioma_grade2_1rep.raw
22748667			kYNTDcVQGLTHSk	Unambiguous	N-Term(iTRAQ4plex); K1(iTRAQ4plex); C6(Methylthio); K14(iTRAQ4plex)	HCD	0.48306164	1	1	3	0.45837	0.4435	0.5239	0.5069555	0.079395049	44221.023	45702.004	20269.48	23168.885	2.329129129	9.43953087	3	High	2325	Unique	Used	85646	1	33.111	1	6.7347	-2.1604	1	35	26/52	28.5641	2325	2072.06	MS2	691.358	126	Glioma_grade2_1rep.raw
22748667			vLGFcHYYLPEEQFPk	Unambiguous	N-Term(iTRAQ4plex); C5(Methylthio); K16(iTRAQ4plex)	HCD	0.52359563	0	1	3	0.49625	0.5924	0.4659	0.5562455	0.108359829	42143.438	35301.047	20913.547	19636.049	12.49488863	4.455420462	2	High	6495	Unique	Used	83306	1	65.943	1	3.9048	1.7014	1	55	17/30	11.37259	6495	2304.15	MS2	1152.58	166	Glioma_grade2_6.raw
22748667			mQVNAEEVVVGDLVEIk	Unambiguous	N-Term(iTRAQ4plex); M1(Oxidation); K17(iTRAQ4plex)	HCD	0.65161693	0	1	3	0.66395	0.5793	0.7345	0.6408545	0.097955948	39171.965	44897.457	26008.32	28772.736	9.631407288	7.136544792	2	High	7096	Unique	Used	54454	1	63.804	1	5.2603	4.5314	1	77	19/32	31.73033	7096	2176.19	MS2	1088.6	167	Glioma_grade2_2.raw
22748667			vLGFcHYYLPEEQFPk	Unambiguous	N-Term(iTRAQ4plex); C5(Methylthio); K16(iTRAQ4plex)	HCD	0.4753946	0	1	3	0.42269	0.4383	0.5112	0.53003997	0.111447687	36819.33	35511.844	15563.153	18822.697	2.556386592	13.4057798	3	High	5254	Unique	Used	87595	1	68.183	1	5.5948	-0.0263	1	34	21/60	22.98923	5254	2304.14	MS2	768.719	168	Glioma_grade2_1rep.raw
22748667			dFTSEQIDEILQNHTEIVFAR	Unambiguous	N-Term(iTRAQ4plex)	HCD	0.47256497	0	1	3	0.52243	0.493	0.4509	0.42551073	0.091228599	36680.7	38871.566	19163.084	16540.27	4.100939091	10.38899351	3	High	8515	Unique	Used	55738	1	75.457	1	8.7974	1.5736	1	11	41/80	9.387476	8515	2649.33	MS2	883.782	101	Glioma_grade2_2.raw
22748667			vLGFcHYYLPEEQFPk	Unambiguous	N-Term(iTRAQ4plex); C5(Methylthio); K16(iTRAQ4plex)	HCD	0.31968653	0	1	3	0.25984	0.3033	0.3337	0.38954613	0.169491098	35287.2	30231.045	9168.914	11776.386	10.91372789	17.60548794	3	High	5112	Unique	Used	96709	1	70.119	1	5.8462	-0.9799	1	23	22/60	10.28357	5112	2304.14	MS2	768.719	162	Glioma_grade2_8rep.raw
22748667			dFTSEQIDEILQNHTEIVFAR	Unambiguous	N-Term(iTRAQ4plex)	HCD	0.3386361	0	1	3	0.31602	0.4047	0.287	0.36759305	0.152702043	34330.086	26806.803	10849.164	9853.994	17.40279727	6.797914168	2	High	7649	Unique	Used	54953	1	68.312	1	7.1405	-0.7712	1	28	25/40	0	7649	2649.33	MS2	1325.17	143	Glioma_grade2_2.raw
22748667			dFTSEQIDEILQNHTEIVFAR	Unambiguous	N-Term(iTRAQ4plex)	HCD	0.45200062	0	1	3	0.43101	0.5422	0.3803	0.47840017	0.150554256	32135.604	25547.312	13850.874	12221.84	16.15253275	8.836065077	3	High	8015	Unique	Used	55287	1	71.274	1	9.1141	0.3296	1	15	42/80	14.91024	8015	2649.33	MS2	883.781	110	Glioma_grade2_2.raw
22748667			mQVNAEEVVVGDLVEIk	Unambiguous	N-Term(iTRAQ4plex); M1(Oxidation); K17(iTRAQ4plex)	HCD	0.6392029	0	1	3	0.61978	0.7327	0.5601	0.6621654	0.112847909	31336.852	26506.316	19421.953	17551.566	11.81022714	7.154111196	3	High	5013	Unique	Used	87415	1	65.574	1	5.9088	0.4584	1	23	22/64	21.95414	5013	2176.18	MS2	726.064	150	Glioma_grade2_1rep.raw
22748667			dFTSEQIDEILQNHTEIVFAR	Unambiguous	N-Term(iTRAQ4plex)	HCD	0.4449732	0	1	3	0.37145	0.4965	0.4065	0.54323643	0.174397788	30232.275	22619.996	11229.839	12288.006	20.36882805	6.363143063	3	High	8120	Unique	Used	55382	1	72.137	1	8.0971	2.4721	1	19	42/80	11.48171	8120	2649.33	MS2	883.783	103	Glioma_grade2_2.raw
22748667			yNTDcVQGLTHSk	Unambiguous	N-Term(iTRAQ4plex); C5(Methylthio); K13(iTRAQ4plex)	HCD	0.5444666	0	1	3	0.53355	0.5735	0.5174	0.55619586	0.045286982	25552.748	23772.35	13633.787	13222.082	5.104628475	2.168013236	3	High	3063	Unique	Used	73697	1	34.928	1	5.6789	-0.8869	1	47	29/48	61.07023	3063	1799.86	MS2	600.626	81	Glioma_grade2_5.raw
22748667			dFTSEQIDEILQNHTEIVFAR	Unambiguous	N-Term(iTRAQ4plex)	HCD	0.36399773	0	1	3	0.42448	0.4305	0.2984	0.3026655	0.201468239	24733.203	24388.496	10498.63	7381.5566	0.992411346	24.65414694	3	High	8652	Unique	Used	55867	1	76.427	1	8.0842	-1.2601	1	22	41/80	18.47939	8652	2649.32	MS2	883.78	106	Glioma_grade2_2.raw
22748667			tVNDLEDSYGQQWTYEQR	Unambiguous	N-Term(iTRAQ4plex)	HCD	0.29781142	0	1	3	0.23049	0.2689	0.3226	0.3763516	0.212326901	24044.447	20610.084	5541.991	7756.6387	10.87666273	23.55118447	2	High	5379	Unique	Used	68516	1	51.72	1	5.8372	-0.3584	1	100	23/34	0	5379	2376.09	MS2	1188.55	60	Glioma_grade2_4.raw
22748667			dFTSEQIDEILQNHTEIVFAR	Unambiguous	N-Term(iTRAQ4plex)	HCD	0.46501723	0	1	3	0.38856	0.4901	0.4451	0.56145734	0.155010776	22655.367	17961.629	8802.915	10084.688	16.34278404	9.597300946	3	High	8367	Unique	Used	55603	1	74.235	1	8.5248	2.5412	1	23	43/80	17.34222	8367	2649.33	MS2	883.783	102	Glioma_grade2_2.raw
22748667			vLGFcHYYLPEEQFPk	Unambiguous	N-Term(iTRAQ4plex); C5(Methylthio); K16(iTRAQ4plex)	HCD	0.43750265	0	1	3	0.45043	0.4295	0.4459	0.4251786	0.028080094	22259.717	23344.514	10026.385	9925.588	3.364018198	0.714458086	4	High	6419	Unique	Used	83248	1	65.159	1	4.3106	-2.7075	1	9	20/90	15.50712	6419	2304.14	MS2	576.79	181	Glioma_grade2_6.raw
22748667			dFTSEQIDEILQNHTEIVFAR	Unambiguous	N-Term(iTRAQ4plex)	HCD	0.5932876	0	1	3	0.52955	0.5472	0.6379	0.65915006	0.108798681	21664.309	20964.27	11472.408	13818.6	2.32239655	13.11935272	3	High	5342	Unique	Used	87661	1	69.142	1	8.957	2.403	1	47	39/80	24.39647	5342	2649.33	MS2	883.783	122	Glioma_grade2_1rep.raw
22748667			tVNDLEDSYGQQWTYEQR	Unambiguous	N-Term(iTRAQ4plex)	HCD	0.431627	0	1	3	0.38682	0.481	0.3919	0.487352	0.125597462	20435.744	16432.812	7904.9004	8008.564	15.35454962	0.921247978	2	High	3906	Unique	Used	86634	1	53.041	1	5.194	1.7993	1	100	22/34	0	3906	2376.09	MS2	1188.55	62	Glioma_grade2_1rep.raw
22748667			dFTSEQIDEILQNHTEIVFAR	Unambiguous	N-Term(iTRAQ4plex)	HCD	0.4898428	0	1	3	0.39927	0.4479	0.5273	0.591436	0.172966048	19674.727	17540.318	7855.546	10373.976	8.110994238	19.53752743	3	High	8274	Unique	Used	55519	1	73.45	1	8.862	1.4354	1	23	42/80	39.61456	8274	2649.33	MS2	883.782	108	Glioma_grade2_2.raw
22748667			dFTSEQIDEILQNHTEIVFAR	Unambiguous	N-Term(iTRAQ4plex)	HCD	0.28633294	0	1	3	0.27695	0.2686	0.3046	0.29543212	0.057754665	19344.273	19946.857	5357.4023	5892.943	2.168892738	6.731961562	3	High	7358	Unique	Used	62673	1	68.032	1	8.1606	4.0617	1	24	40/80	9.718121	7358	2649.34	MS2	883.784	111	Glioma_grade2_3.raw
22748667			kYNTDcVQGLTHSk	Unambiguous	N-Term(iTRAQ4plex); K1(iTRAQ4plex); C6(Methylthio); K14(iTRAQ4plex)	HCD	0.47886723	1	1	3	0.5092	0.4666	0.4922	0.4510752	0.054053818	17330.824	18912.312	8824.774	8530.875	6.170994084	2.394816534	2	High	2323	Unique	Used	85645	1	33.079	1	4.1824	0.0553	1	100	20/26	0	2323	2072.06	MS2	1036.54	175	Glioma_grade2_1rep.raw
22748667			kYNTDcVQGLTHSk	Unambiguous	N-Term(iTRAQ4plex); K1(iTRAQ4plex); C6(Methylthio); K14(iTRAQ4plex)	HCD	0.45618877	1	1	3	0.40927	0.3822	0.5355	0.49999845	0.159338418	15633.125	16742.502	6398.1724	8371.225	4.845916958	18.89256326	3	High	2480	Unique	Used	95157	1	35.333	1	7.0143	-2.1604	1	100	26/52	26.35263	2480	2072.06	MS2	691.358	124	Glioma_grade2_8rep.raw
22748667			mTVAHMWFDNQIHEADTTEDQSGTSFDk	Unambiguous	N-Term(iTRAQ4plex); K28(iTRAQ4plex)	HCD	0.29633513	0	1	3	0.33543	0.3079	0.2838	0.26045042	0.108388224	14911.329	16245.97	5001.733	4231.2695	6.057865937	11.80114411	4	High	4796	Unique	Used	92060	1	53.549	1	8.4335	-0.2636	1	9	23/162	9.94276	4796	3529.59	MS2	883.152	36	Glioma_grade2_7rep.raw
22748667			dFTSEQIDEILQNHTEIVFAR	Unambiguous	N-Term(iTRAQ4plex)	HCD	0.46213263	0	1	3	0.50927	0.5158	0.4091	0.41438472	0.126139564	13758.037	13583.321	7006.614	5628.7207	0.903706893	15.42211139	3	High	8792	Unique	Used	55998	1	77.448	1	9.3063	0.7443	1	29	41/80	9.611156	8792	2649.33	MS2	883.781	109	Glioma_grade2_2.raw
22748667			vLGFcHYYLPEEQFPk	Unambiguous	N-Term(iTRAQ4plex); C5(Methylthio); K16(iTRAQ4plex)	HCD	0.33972248	0	1	3	0.28841	0.3556	0.3268	0.40298292	0.140605259	12932.59	10489.158	3729.9426	4226.9517	14.75353024	8.83355972	2	High	5106	Unique	Used	96706	1	70.036	1	4.0768	3.6086	1	100	16/30	18.20356	5106	2304.15	MS2	1152.58	180	Glioma_grade2_8rep.raw
22748667			sSHTWVALSHIAGLcNR	Unambiguous	N-Term(iTRAQ4plex); C15(Methylthio)	HCD	0.35744324	0	1	3	0.31693	0.2937	0.4262	0.39498916	0.175479244	12794.092	13804.933	4054.8428	5452.799	5.374426512	20.79389042	4	High	4943	Unique	Used	92169	1	55.086	1	6.2549	-0.7909	1	30	27/96	4.936557	4943	2042.02	MS2	511.26	129	Glioma_grade2_7rep.raw
22748667			dFTSEQIDEILQNHTEIVFAR	Unambiguous	N-Term(iTRAQ4plex)	HCD	0.3768938	0	1	3	0.31801	0.3618	0.3902	0.44387862	0.139396952	10490.644	9221.716	3336.1433	4093.3225	9.103603969	14.41305637	2	High	7769	Unique	Used	55062	1	69.288	1	6.902	2.4541	1	93	24/40	0	7769	2649.33	MS2	1325.17	136	Glioma_grade2_2.raw
22748667			dFTSEQIDEILQNHTEIVFAR	Unambiguous	N-Term(iTRAQ4plex)	HCD	0.45690614	0	1	3	0.40707	0.4832	0.4347	0.5160633	0.105864351	9184.107	7736.5728	3738.6016	3992.5608	12.09834665	4.645518052	3	High	5144	Unique	Used	96727	1	70.554	1	9.457	2.4721	1	100	39/80	10.07398	5144	2649.33	MS2	883.783	123	Glioma_grade2_8rep.raw
22748667			dFTSEQIDEILQNHTEIVFAR	Unambiguous	N-Term(iTRAQ4plex)	HCD	0.33036724	0	1	3	0.27673	0.3205	0.3389	0.39248657	0.144314334	8175.903	7059.4365	2262.523	2770.734	10.36355026	14.27939978	3	High	8854	Unique	Used	56038	1	78.222	1	8.8793	2.6794	1	59	41/80	8.601005	8854	2649.34	MS2	883.783	108	Glioma_grade2_2.raw
22748667			kYNTDcVQGLTHSk	Unambiguous	N-Term(iTRAQ4plex); K1(iTRAQ4plex); C6(Methylthio); K14(iTRAQ4plex)	HCD	0.54252905	1	1	3	0.43073	0.6154	0.4915	0.7022699	0.218009147	5034.1445	3523.433	2168.3335	2474.4011	24.96581179	9.323060399	4	High	2508	Unique	Used	95174	1	35.697	1	4.3527	-1.9018	1	100	26/78	43.91948	2508	2072.06	MS2	518.77	138	Glioma_grade2_8rep.raw
22748667			mTVAHmWFDNQIHEADTTEDQSGTSFDk	Unambiguous	N-Term(iTRAQ4plex); M6(Oxidation); K28(iTRAQ4plex)	HCD	0.5856458	0	1	3	0.49599	0.6028	0.5715	0.694614	0.139068966	4446.855	3658.9055	2205.5776	2541.5269	13.74736978	10.0082915	4	High	4800	Unique	Used	81899	1	50.334	1	7.0994	1.1717	1	21	22/162	9.828332	4800	3545.59	MS2	887.152	44	Glioma_grade2_6.raw
22748667			dFTSEQIDEILQNHTEIVFAR	Unambiguous	N-Term(iTRAQ4plex)	HCD	0.3605447	0	1	3	0.33316	0.469	0.2835	0.39909264	0.217070263	3698.8103	2627.587	1232.2985	1048.6506	23.946307	11.38637205	3	High	8923	Unique	Used	56081	1	79.116	1	9.0888	1.9192	1	100	39/80	8.089329	8923	2649.33	MS2	883.783	95	Glioma_grade2_2.raw
22748667			yQLSIHETEDPNDNR	Unambiguous	N-Term(iTRAQ4plex)	HCD	0.4631608	0	1	3	0.50362	0.5714	0.3678	0.41726044	0.19446496	3062.106	2699.0806	1542.1362	1126.2196	8.911279565	22.04334581	3	High	856	Unique	Used	56716	1	16.916	1	3.6187	-0.1915	1	100	22/56	25.29618	856	1974.93	MS2	658.981	41	Glioma_grade2_3.raw
22748667			vLGFcHYYLPEEQFPk	Unambiguous	N-Term(iTRAQ4plex); C5(Methylthio); K16(iTRAQ4plex)	HCD	0.338432	0	1	3	0.22802	0.3232	0.3492	0.49492997	0.316623841	2925.821	2064.1873	667.1503	1021.6281	24.41947971	29.68461181	4	High	5120	Unique	Used	96714	1	70.218	1	4.2377	2.2725	1	100	18/90	19.10156	5120	2304.15	MS2	576.793	173	Glioma_grade2_8rep.raw
22748667			dFTSEQIDEILQNHTEIVFAR	Unambiguous	N-Term(iTRAQ4plex)	HCD	0.6033932	0	1	3	0.63408	0.4922	0.7467	0.57957095	0.173720876	1318.9762	1699.3015	836.3424	984.8658	17.82013621	11.53321222	3	High	8992	Unique	Used	56136	1	79.833	1	6.7247	0.8825	1	100	37/80	6.435271	8992	2649.33	MS2	883.782	113	Glioma_grade2_2.raw
22749415	NP_689926.1	STT3A	dolichyl-diphosphooligosaccharide--protein glycosyltransferase subunit STT3A [Homo sapiens]	3	4	2.281242847	23.74681705	16.652716	4.96	1.16E-04	705	80.477	8.0747	Integral membrane protein	Biological_process unknown,Protein modification	Glucosyltransferase activity,Molecular function unknown	Plasma membrane	-																								
22749415			fLAEEGFYk	Unambiguous	N-Term(iTRAQ4plex); K9(iTRAQ4plex)	HCD	2.4432075	0	1	1	2.21855	2.4975	2.3949	2.6961143	8.137522145	169922.39	150941.38	376981.56	406955.2	8.365918586	5.40721272	2	High	4764	Unique	Used	60277	1	47.523	1	3.9678	-0.114	1	25	16/16	18.64523	4764	1391.74	MS2	696.376	185	Glioma_grade2_3.raw
22749415			fLAEEGFYk	Unambiguous	N-Term(iTRAQ4plex); K9(iTRAQ4plex)	HCD	2.1192784	0	1	1	2.09636	2.1152	2.1233	2.1424067	0.900739336	85733.29	84969.34	179727.53	182038.89	0.63290674	0.90355447	2	High	4868	Unique	Used	52360	1	47.439	1	3.8993	1.9033	1	54	16/16	39.63298	4868	1391.75	MS2	696.377	200	Glioma_grade2_2.raw
22749415			fYSLLDPSYAk	Unambiguous	N-Term(iTRAQ4plex); K11(iTRAQ4plex)	HCD	1.8072608	0	1	1	1.79156	1.8208	1.7939	1.8232163	0.938070655	30001.55	29519.604	53749.62	53820.625	1.145096363	0.093349452	2	High	6217	Unique	Used	53632	1	57.264	1	4.1318	-1.7812	1	98	16/20	41.53748	6217	1591.86	MS2	796.433	257	Glioma_grade2_2.raw
22749415			fESVIHEFDPYFNYR	Unambiguous	N-Term(iTRAQ4plex)	HCD	3.1237102	0	1	1	2.92319	3.0553	3.1892	3.3332949	5.636839992	2584.4575	2472.7373	7554.8486	8242.363	3.124186991	6.154834242	3	High	7678	Unique	Used	70589	1	70.763	1	4.6538	1.0045	1	100	21/56	44.28054	7678	2107.01	MS2	703.007	100	Glioma_grade2_4.raw
23308577	NP_006614.2	PHGDH	D-3-phosphoglycerate dehydrogenase [Homo sapiens]	8	16	2.335171223	23.74863036	87.834355	19.51	4.41E-04	533	56.614	6.7124	Enzyme: Dehydrogenase	Metabolism,Energy pathways	Catalytic activity	Extracellular	Cytosol																								
23308577			vTADVINAAEk	Unambiguous	N-Term(iTRAQ4plex); K11(iTRAQ4plex)	HCD	1.9510669	0	1	1	1.83374	1.9945	1.9112	2.078676	5.404933591	160527.88	147590.75	294366.66	306793.34	5.937928747	2.923344765	2	High	2627	Unique	Used	50253	1	31.106	1	4.5487	-0.7698	1	18	19/20	21.866	2627	1418.81	MS2	709.908	251	Glioma_grade2_2.raw
23308577			qHVTEAFQFHF	Unambiguous	N-Term(iTRAQ4plex)	HCD	1.7875376	0	1	1	1.60488	1.809	1.7685	1.9934365	8.896826811	62507.5	55453.06	100316.78	110542.14	8.457474874	6.858065474	2	High	5202	Unique	Used	68352	1	50.346	1	4.4884	1.1557	1	37	17/20	7.907771	5202	1534.76	MS2	767.883	164	Glioma_grade2_4.raw
23308577			iLQDGGLQVVEk	Unambiguous	N-Term(iTRAQ4plex); K12(iTRAQ4plex)	HCD	3.0194304	0	1	1	3.04989	3.0443	2.9945	2.9890215	1.064026193	48602.72	48691.316	148233.17	145539.39	0.128778361	1.296778777	2	High	4184	Unique	Used	59741	1	42.948	1	4.757	5.0498	1	67	21/22	18.3976	4184	1586.94	MS2	793.975	152	Glioma_grade2_3.raw
23308577			nAGNcLSPAVIVGLLk	Unambiguous	N-Term(iTRAQ4plex); C5(Methylthio); K16(iTRAQ4plex)	HCD	2.8691533	0	1	1	2.85174	2.8664	2.8719	2.8866515	0.503020972	41521.61	41310.02	118409	119247.63	0.361255052	0.499040115	2	High	8238	Unique	Used	63456	1	75.623	1	6.5087	-1.3752	1	31	24/30	15.78073	8238	1903.07	MS2	952.04	156	Glioma_grade2_3.raw
23308577			aLVDHENVIScPHLGASTk	Unambiguous	N-Term(iTRAQ4plex); C11(Methylthio); K19(iTRAQ4plex)	HCD	2.450104	0	1	1	2.19057	3.0211	2.0361	2.8080368	18.89131119	28186.951	20438.012	61745.527	57390.69	22.53709608	5.169435208	4	High	3549	Unique	Used	91130	1	40.511	1	5.0926	2.451	1	4	30/108	14.50628	3549	2325.2	MS2	582.055	145	Glioma_grade2_7rep.raw
23308577			qHVTEAFQFHF	Unambiguous	N-Term(iTRAQ4plex)	HCD	2.3386261	0	1	1	2.15211	2.5495	2.1606	2.5595903	9.763275089	27048.201	22831.871	58210.605	58440.234	11.95425514	0.27839015	3	High	5182	Unique	Used	68333	1	50.204	1	5.2399	0.5902	1	35	20/40	24.80133	5182	1534.76	MS2	512.257	103	Glioma_grade2_4.raw
23308577			cGEEIAVQFVDMVk	Unambiguous	N-Term(iTRAQ4plex); C1(Methylthio); K14(iTRAQ4plex)	HCD	2.4465032	0	1	1	2.16097	2.5146	2.388	2.7787585	10.47207364	25168.586	21629.303	54388.58	60102.605	10.69557223	7.058055737	2	High	8517	Unique	Used	55740	1	75.466	1	5.076	-1.5217	1	53	22/26	21.6278	8517	1901.94	MS2	951.473	134	Glioma_grade2_2.raw
23308577			gTIQVITQGTSLk	Unambiguous	N-Term(iTRAQ4plex); K13(iTRAQ4plex)	HCD	2.1894002	0	1	1	2.11489	2.1226	2.2559	2.2641833	3.73171631	23938.787	23851.398	50627.906	54003.938	0.258602701	4.563075694	2	High	3551	Unique	Used	51121	1	37.807	1	4.7732	-0.4687	1	91	17/24	0	3551	1633.97	MS2	817.49	154	Glioma_grade2_2.raw
23308577			qHVTEAFQFHF	Unambiguous	N-Term(iTRAQ4plex)	HCD	2.3293355	0	1	1	2.23502	2.1285	2.5402	2.4191558	7.902051884	21664.238	22748.396	48420.008	55031.914	3.452240521	9.038640324	3	High	4512	Unique	Used	74997	1	46.67	1	5.8123	-3.4662	1	16	21/40	2.192826	4512	1534.75	MS2	512.255	89	Glioma_grade2_5.raw
23308577			nAGNcLSPAVIVGLLk	Unambiguous	N-Term(iTRAQ4plex); C5(Methylthio); K16(iTRAQ4plex)	HCD	3.2426515	0	1	1	2.7038	3.6069	2.9696	3.961482	17.40466261	21063.951	15789.947	56952.754	62551.594	20.23820651	6.625663078	2	High	8564	Unique	Used	55784	1	75.807	1	4.6926	-3.8127	1	53	20/30	42.88056	8564	1903.07	MS2	952.037	171	Glioma_grade2_2.raw
23308577			dLPLLLFR	Unambiguous	N-Term(iTRAQ4plex)	HCD	3.524099	0	1	1	3.49966	3.3625	3.6923	3.5475802	3.859914802	20223.729	21048.78	70776.17	74672.234	2.827059324	3.788193199	2	High	5276	Unique	Used	87611	1	68.427	1	3.2039	-0.7136	1	99	13/14	19.40543	5276	1130.7	MS2	565.856	134	Glioma_grade2_1rep.raw

23308577			aLVDHENVIScPHLGASTk	Unambiguous	N-Term(iTRAQ4plex); C11(Methylthio); K19(iTRAQ4plex)	HCD	1.6659883	0	1	1	1.63231	1.6664	1.6655	1.7003684	1.66771572	20141.422	19728.877	32877.094	33546.36	1.46331166	1.424925982	3	High	3536	Unique	Used	91119	1	40.396	1	7.667	2.168	1	65	36/72	31.11798	3536	2325.2	MS2	775.738	147	Glioma_grade 2_7rep.raw
23308577			aLVDHENVIScPHLGASTk	Unambiguous	N-Term(iTRAQ4plex); C11(Methylthio); K19(iTRAQ4plex)	HCD	3.7116609	0	1	1	3.52191	3.6654	3.7561	3.9091487	4.373389771	10471.2	10061.21	36878.58	39330.766	2.823893632	4.550511034	2	High	3566	Unique	Used	91145	1	40.647	1	6.4849	0.4151	1	69	21/36	0	3566	2325.19	MS2	1163.1	178	Glioma_grade 2_7rep.raw
23308577			cGEEIAVQFVDMVk	Unambiguous	N-Term(iTRAQ4plex); C1(Methylthio); K14(iTRAQ4plex)	HCD	2.1605053	0	1	1	2.19276	2.3979	1.9434	2.12523	8.67043761	9816.25	8976.478	21524.707	19077.078	6.319555903	8.525413667	3	High	8516	Unique	Used	55739	1	75.46	1	4.7771	0.5572	1	15	20/52	13.27548	8516	1901.94	MS2	634.652	162	Glioma_grade 2_2.raw
23308577			nAGNcLSPAVIVGLLk	Unambiguous	N-Term(iTRAQ4plex); C5(Methylthio); K16(iTRAQ4plex)	HCD	2.3317165	0	1	1	2.19073	2.914	1.894	2.5192442	18.42404837	5410.957	4067.9575	11853.963	10248.179	20.03697899	10.27466709	3	High	8258	Unique	Used	63475	1	75.763	1	4.767	-2.601	1	24	25/60	6.032022	8258	1903.07	MS2	635.028	162	Glioma_grade 2_3.raw
23308577			nAGNcLSPAVIVGLLk	Unambiguous	N-Term(iTRAQ4plex); C5(Methylthio); K16(iTRAQ4plex)	HCD	2.2246163	0	1	1	1.58754	2.4326	2.0889	3.2008133	29.12148957	3647.8591	2380.6223	5791.131	7619.9277	29.72794225	19.28489878	3	High	8181	Unique	Used	71011	1	75.507	1	5.483	0.4779	1	87	26/60	25.57487	8181	1903.08	MS2	635.03	162	Glioma_grade 2_4.raw
23308751	NP_689953.1	HIBADH	3-hydroxyisobutyrate dehydrogenase, mitochondrial precursor [Homo sapiens]	2	2	2.18712163	34.67884994	13.992323	7.44	6.41E-05	336	35.306	8.1333	Enzyme: Dehydrogenase	Metabolism,Energy pathways	Catalytic activity	Mitochondrion	-																								
23308751			sPILLGSLAHQIYR	Unambiguous	N-Term(iTRAQ4plex)	HCD	2.72344	0	1	1	2.51609	2.7217	2.725	2.9477398	6.463310021	52675.34	48695.67	132535.69	143542.16	5.551985018	5.638083297	3	High	5285	Unique	Used	75675	1	53.19	1	4.3601	-1.4685	1	16	30/52	44.87667	5285	1712	MS2	571.337	148	Glioma_grade 2_5.raw
23308751			kDFSSVFQFLR	Unambiguous	N-Term(iTRAQ4plex); K1(iTRAQ4plex)	HCD	1.6508033	1	1	1	1.73534	1.6271	1.6761	1.5715402	4.224030781	10985.443	11716.164	19063.467	18412.424	4.552081042	2.456816411	3	High	5502	Unique	Used	92590	1	60.845	1	5.1539	2.8958	1	35	23/40	24.62903	5502	1661.93	MS2	554.648	170	Glioma_grade 2_7rep.raw
23503267	NP_699174.1	ENPP6	ectonucleotide pyrophosphatase/phosphodiesterase family member 6 precursor [Homo sapiens]	9	22	2.048764229	17.70686541	158.63814	25.23	6.25E-05	440	50.208	7.9868	Enzyme: Hydrolase	Metabolism,Energy pathways	Hydrolase activity	-	-																								
23503267			lLVFLLDGFR	Unambiguous	N-Term(iTRAQ4plex)	HCD	2.4310915	0	1	1	2.2301	2.5441	2.332	2.6603801	8.02261679	342939.25	300611.62	764789.9	799741.25	9.301565921	3.15932816	2	High	8476	Unique	Used	63681	1	77.326	1	3.6496	0.8042	1	5	15/18	14.50908	8476	1336.81	MS2	668.91	193	Glioma_grade 2_3.raw
23503267			aDLAAIYHER	Unambiguous	N-Term(iTRAQ4plex)	HCD	1.6542573	0	1	1	1.60874	1.637	1.6712	1.7005728	2.418573546	168024.8	165125.34	270308.38	280807.7	1.230813127	2.694220198	2	High	2192	Unique	Used	65561	1	27.108	1	3.0482	15.09	1	16	16/18	49.06895	2192	1302.71	MS2	651.86	292	Glioma_grade 2_4.raw
23503267			aDLAAIYHER	Unambiguous	N-Term(iTRAQ4plex)	HCD	1.6742924	0	1	1	1.66601	1.715	1.6347	1.6828194	2.000183973	132999.53	129198.23	221578.44	217417.27	2.050303563	1.340510378	2	High	2175	Unique	Used	65545	1	26.991	1	3.7865	2.252	1	24	16/18	57.39418	2175	1302.7	MS2	651.851	254	Glioma_grade 2_4.raw
23503267			lLVFLLDGFR	Unambiguous	N-Term(iTRAQ4plex)	HCD	2.0864694	0	1	1	1.92089	2.2293	1.9634	2.2786384	8.674539323	91092.18	78489.13	174977.89	178848.34	10.51024092	1.546986181	2	High	8783	Unique	Used	55990	1	77.371	1	3.7902	-0.8395	1	25	16/18	15.73885	8783	1336.81	MS2	668.909	190	Glioma_grade 2_2.raw
23503267			aDLAAIYHER	Unambiguous	N-Term(iTRAQ4plex)	HCD	1.6022223	0	1	1	1.49754	1.6151	1.5903	1.7151275	5.573762408	87517.7	81147.61	131060.836	139178.5	5.341150277	4.248127121	3	High	2256	Unique	Used	65621	1	27.564	1	4.4227	0.7659	1	10	22/36	8.331324	2256	1302.69	MS2	434.903	242	Glioma_grade 2_4.raw
23503267			lSTVEHMTVYEk	Unambiguous	N-Term(iTRAQ4plex); K12(iTRAQ4plex)	HCD	2.091951	0	1	1	2.11818	2.0673	2.1172	2.0663562	1.402952133	65453.355	67063.12	138641.66	138576.28	1.717938464	0.033353283	2	High	2802	Unique	Used	73459	1	32.877	1	5.4439	0.7589	1	40	19/22	5.713987	2802	1724.91	MS2	862.961	243	Glioma_grade 2_5.raw
23503267			gPVVSLWPAPGk	Unambiguous	N-Term(iTRAQ4plex); K12(iTRAQ4plex)	HCD	2.0542707	0	1	1	1.9415	2.0428	2.0652	2.1729245	4.618682719	63897.707	60730.43	124057.305	131962.64	3.594056845	4.366781647	2	High	5180	Unique	Used	68331	1	50.191	1	4.9714	0.607	1	36	16/22	37.8995	5180	1495.89	MS2	748.448	180	Glioma_grade 2_4.raw
23503267			gIFLAFGPDFk	Unambiguous	N-Term(iTRAQ4plex); K11(iTRAQ4plex)	HCD	2.32876	0	1	1	2.21776	2.3686	2.2914	2.447315	4.241716336	57555.934	53889.7	127645.055	131885.08	4.652347211	2.310444935	2	High	7198	Unique	Used	70169	1	66.532	1	4.7655	1.4022	1	46	17/20	21.0483	7198	1499.85	MS2	750.43	223	Glioma_grade 2_4.raw
23503267			lSTVEHMTVYEk	Unambiguous	N-Term(iTRAQ4plex); K12(iTRAQ4plex)	HCD	1.9727925	0	1	1	2.03011	1.8768	2.0766	1.9198079	4.716362232	45484.71	49200.14	92338.75	94454.82	5.54936877	1.602075967	2	High	2783	Unique	Used	73441	1	32.748	1	4.233	11.799	1	40	19/22	19.07003	2783	1724.93	MS2	862.97	234	Glioma_grade 2_5.raw
23503267			lSTVEHMTVYEk	Unambiguous	N-Term(iTRAQ4plex); K12(iTRAQ4plex)	HCD	2.2398171	0	1	1	2.07938	2.2491	2.2312	2.4133515	6.088722565	40926.8	37837.992	85102.35	91316.375	5.545922301	4.981307076	3	High	2793	Unique	Used	73450	1	32.824	1	3.9196	-1.0535	1	11	21/44	18.57429	2793	1724.91	MS2	575.642	195	Glioma_grade 2_5.raw
23503267			sDYISDEALESLPGFk	Unambiguous	N-Term(iTRAQ4plex); K16(iTRAQ4plex)	HCD	1.8109735	0	1	1	1.61815	1.8208	1.8023	2.0279374	9.222359692	30918.36	27477.785	50030.64	55723.23	8.332241499	7.612523289	2	High	6889	Unique	Used	54259	1	62.277	1	5.5292	-0.0128	1	45	21/30	8.686241	6889	2059.05	MS2	1030.03	171	Glioma_grade 2_2.raw
23503267			aDLAAIYHER	Unambiguous	N-Term(iTRAQ4plex)	HCD	2.0432575	0	1	1	2.09114	1.8365	2.2787	2.0012026	8.984486355	28432.082	32374.713	59455.582	64788.355	9.169570986	6.070058696	3	High	1916	Unique	Used	57630	1	25.764	1	4.3338	-2.2562	1	43	22/36	34.56047	1916	1302.69	MS2	434.901	218	Glioma_grade 2_3.raw
23503267			gIFLAFGPDFk	Unambiguous	N-Term(iTRAQ4plex); K11(iTRAQ4plex)	HCD	2.5614305	0	1	1	2.63035	2.4856	2.6417	2.4963057	3.277569907	27043.717	28619.11	71134.41	71442.05	4.002567363	0.30514761	2	High	6779	Unique	Used	76996	1	65.688	1	4.7086	-1.2022	1	83	17/20	31.69568	6779	1499.85	MS2	750.428	265	Glioma_grade 2_5.raw
23503267			lSTVEHmTVYEk	Unambiguous	N-Term(iTRAQ4plex); M7(Oxidation); K12(iTRAQ4plex)	HCD	1.8146832	0	1	1	1.74992	1.8031	1.8259	1.8814169	3.002214166	18106.484	17572.549	31684.85	33061.29	2.116363743	3.006480565	2	High	1471	Unique	Used	72226	1	22.963	1	2.9548	2.4107	1	73	17/22	11.21638	1471	1740.91	MS2	870.96	212	Glioma_grade 2_5.raw
23503267			iDVEGHHYGPASPQR	Unambiguous	N-Term(iTRAQ4plex)	HCD	1.8532511	0	1	1	1.9061	1.9565	1.7527	1.7990036	5.071893252	17126.361	16685.19	32644.578	30016.717	1.845257	5.930864765	4	High	1150	Unique	Used	78652	1	20.423	1	5.402	-0.4745	1	12	26/84	13.23893	1150	1806.9	MS2	452.48	157	Glioma_grade 2_6.raw
23503267			lSTVEHMTVYEk	Unambiguous	N-Term(iTRAQ4plex); K12(iTRAQ4plex)	HCD	2.0038302	0	1	1	1.94562	1.6906	2.3643	2.0544145	13.85695206	16836.377	19375.893	32757.125	39806.117	9.917682512	13.73805774	3	High	2774	Unique	Used	73433	1	32.673	1	3.6513	11.579	1	31	21/44	33.16018	2774	1724.93	MS2	575.649	234	Glioma_grade 2_5.raw
23503267			gWHGYDNELMDMR	Unambiguous	N-Term(iTRAQ4plex)	HCD	1.8921502	0	1	1	1.76776	1.8302	1.952	2.0209293	6.061113976	12966.132	12524.026	22921.035	25310.172	2.452838077	7.005319083	3	High	4171	Unique	Used	74685	1	44.003	1	5.6297	0.989	1	25	25/48	20.72678	4171	1767.77	MS2	589.929	22	Glioma_grade 2_5.raw
23503267			lSTVEHmTVYEk	Unambiguous	N-Term(iTRAQ4plex); M7(Oxidation); K12(iTRAQ4plex)	HCD	1.9343569	0	1	1	2.2578	1.7476	2.1756	1.6840011	14.86563925	8213.584	10611.465	18544.645	17869.719	18.01384863	2.621189547	3	High	1429	Unique	Used	72188	1	22.641	1	3.2854	2.9989	1	54	18/44	14.1879	1429	1740.91	MS2	580.976	163	Glioma_grade 2_5.raw
23503267			gIFLAFGPDFk	Unambiguous	N-Term(iTRAQ4plex); K11(iTRAQ4plex)	HCD	2.502826	0	1	1	2.73922	2.2863	2.7622	2.3055198	10.41720662	7061.6606	8460.484	19343.414	19505.816	12.7445986	0.591185748	3	High	7210	Unique	Used	70180	1	66.627	1	4.4689	-0.3769	1	55	19/40	11.90692	7210	1499.85	MS2	500.621	238	Glioma_grade 2_4.raw
23503267			kLLVFLLDGFR	Unambiguous	N-Term(iTRAQ4plex); K1(iTRAQ4plex)	HCD	2.934368	1	1	1	2.56213	3.5523	2.4887	3.450462	18.7768931	5999.7383	4327.3354	15372.126	14931.307	22.90227543	2.057232949	3	High	6665	Unique	Used	93353	1	74.597	1	5.0883	-0.8268	1	69	24/40	8.05421	6665	1609.01	MS2	537.007	30	Glioma_grade 2_7rep.raw
23503267			gIFLAFGPDFk	Unambiguous	N-Term(iTRAQ4plex); K11(iTRAQ4plex)	HCD	2.1016386	0	1	1	2.36823	2.134	2.0657	1.8614181	9.915355065	5906.445	6554.752	13987.799	12201.135	7.35759616	9.648061506	3	High	7105	Unique	Used	62456	1	65.733	1	4.4945	-0.8653	1	89	19/40	29.09961	7105	1499.85	MS2	500.621	229	Glioma_grade 2_3.raw
23503267			gIFLAFGPDFk	Unambiguous	N-Term(iTRAQ4plex); K11(iTRAQ4plex)	HCD	2.9635396	0	1	1	2.94134	2.8454	3.0856	2.9850163	3.362736523	2714.4446	2805.944	7984.101	8375.789	2.34403231	3.385905907	3	High	6793	Unique	Used	77008	1	65.818	1	4.0587	-1.964	1	100	20/40	0	6793	1499.85	MS2	500.621	217	Glioma_grade 2_5.raw
24234747	NP_004506.2	ILF2	interleukin enhancer-binding factor 2 [Homo sapiens]	4	9	2.29758954	23.93545218	63.896808	13.59	9.35E-05	390	43.035	5.2622	Transcription factor	Regulation of nucleobase, nucleoside, nucleotide and nucleic acid metabolism	Transcription factor activity	Nucleus	Cytoplasm, Nucleolus																								
24234747			wFEENASQSTVk	Unambiguous	N-Term(iTRAQ4plex); K12(iTRAQ4plex)	HCD	2.7854674	0	1	1	2.67584	2.7864	2.7845	2.899627	3.279025166	42119.266	40447.61	112704.36	117282.98	2.863228817	2.815438469	2	High	3301	Unique	Used	50884	1	36.022	1	5.0798	1.6572	1	94	18/22	57.08062	3301	1713.87	MS2	857.439	199	Glioma_grade 2_2.raw
24234747			vkPAPDETSFSEALLk	Unambiguous	N-Term(iTRAQ4plex); K2(iTRAQ4plex); K16(iTRAQ4plex)	HCD	2.943109	0	1	1	2.81167	2.873	3.0117	3.0774143	4.15749837	30647.166	29992.594	86169.78	92299.64	1.526563759	4.857376209	2	High	3834	Unique	Used	81082	1	41.625	1	5.1418	1.1667	1	62	21/30	0	3834	2164.23	MS2	1082.62	107	Glioma_grade 2_6.raw
24234747			iLPTLEAVAALGNk	Unambiguous	N-Term(iTRAQ4plex); K14(iTRAQ4plex)	HCD	2.2975895	0	1	1	2.30921	2.1727	2.4303	2.2866592	4.590292295	23143.826	24597.467	53443.875	56246.023	4.306039254	3.612762686	2	High	7302	Unique	Used	70262	1	67.415	1	5.7961	0.7941	1	99	22/26	10.10702	7302	1698.04	MS2	849.525	54	Glioma_grade 2_4.raw
24234747			vkPAPDETSFSEALLk	Unambiguous	N-Term(iTRAQ4plex); K2(iTRAQ4plex); K16(iTRAQ4plex)	HCD	1.696899	0	1	1	1.54907	2.0156	1.452	1.889248	15.57347987	21673.064	16656.984	33573.05	31469.172	18.50717319	4.574463648	3	High	3784	Unique	Used	81039	1	41.184	1	7.1839	0.9066	1	31	26/60	16.67776	3784	2164.22	MS2	722.08	106	Glioma_grade 2_6.raw
24234747			vkPAPDETSFSEALLk	Unambiguous	N-Term(iTRAQ4plex); K2(iTRAQ4plex); K16(iTRAQ4plex)	HCD	1.9238075	0	1	1	2.06939	1.7514	2.1276	1.8005965	9.744514094	21472.594	25371.73	44435.215	45684.246	11.77135359	1.96006119	3	High	3894	Unique	Used	81130	1	42.217	1	6.9613	0.6528	1	13	29/60	13.08332	3894	2164.22	MS2	722.08	118	Glioma_grade 2_6.raw
24234747			kLDPELHLDIk	Unambiguous	N-Term(iTRAQ4plex); K1(iTRAQ4plex); K11(iTRAQ4plex)	HCD	1.9340672	1	1	1	1.83368	1.984	1.8879	2.0426812	4.848536555	10480.599	9686.495	19218.094	19786.422	5.568638926	2.060625917	2	High	3165	Unique	Used	95596	1	43.931	1	3.3084	1.7916	1	100	16/20	15.65006	3165	1753.06	MS2	877.034	63	Glioma_grade 2_8rep.raw
24234747			kLDPELHLDIk	Unambiguous	N-Term(iTRAQ4plex); K1(iTRAQ4plex); K11(iTRAQ4plex)	HCD	2.9777718	1	1	1	2.97167	2.9649	2.9907	2.9838612	0.390441232	4354.178	4364.092	12939.174	13021.844	0.160817608	0.450340719	4	High	3001	Unique	Used	86104	1	41.118	1	3.6303	1.0799	1	82	27/60	39.26257	3001	1753.06	MS2	439.021	64	Glioma_grade 2_1rep.raw
24234747			kLDPELHLDIk	Unambiguous	N-Term(iTRAQ4plex); K1(iTRAQ4plex); K11(iTRAQ4plex)	HCD	1.5475342	1	1	1	1.50004	1.7306	1.3888	1.602329	9.370139817	3727.1792	3230.535	5590.9214	5176.38	10.09470845	5.444726104	4	High	3131	Unique	Used	95573	1	43.521	1	4.04	-1.2876	1	100	25/60	20.95535	3131	1753.06	MS2	439.02	80	Glioma_grade 2_8rep.raw
24234747			iLPTLEAVAALGNk	Unambiguous	N-Term(iTRAQ4plex); K14(iTRAQ4plex)	HCD	2.6534486	0	1	1	2.55367	2.9779	2.3752	2.7698033	9.798512455	2466.243	2114.893	6297.9707	5857.8374	10.84630395	5.120535607	3	High	7290	Unique	Used	70251	1	67.316	1	4.8995	-1.1908	1	57	24/52	3.868754	7290	1698.04	MS2	566.684	43	Glioma_grade 2_4.raw
24234756	NP_703194.1	ILF3	interleukin enhancer-binding factor 3 isoform c [Homo sapiens]	9	19	2.02964282	29.80675625	108.35267	22.9	2.69E-04	690	74.56	8.2358	RNA binding protein,Transcription factor	Regulation of nucleobase, nucleoside, nucleotide and nucleic acid metabolism,Regulation of gene expression, epigenetic	Transcription factor activity,Transcription regulator activity	Nucleus	Mitochondrion, Cytoplasm, Ribosome, Nucleolus																								
24234756			aYAALAALEk	Unambiguous	N-Term(iTRAQ4plex); K10(iTRAQ4plex)	HCD	1.8259662	0	1	5	1.62375	1.7956	1.8534	2.0495837	9.591598142	423318.47	382805.84	687363.1	784592.7	7.107279868	9.341545377	2	High	4741	Unique	Used	52239	1	46.562	1	4.7969	-1.1654	1	14	16/18	52.73346	4741	1308.78	MS2	654.891	236	Glioma_grade 2_2.raw
24234756			lAAFGQLHk	Unambiguous	N-Term(iTRAQ4plex); K9(iTRAQ4plex)	HCD	2.0946188	0	1	5	1.90217	2.0853	2.1031	2.30559	7.858331526	377925.84	344739.9	718880.44	794828.9	6.49428966	7.095638465	2	High	2273	Unique	Used	79699	1	28.703	1	3.9411	3.9904	1	11	16/16	26.91501	2273	1272.77	MS2	636.89	195	Glioma_grade 2_6.raw
24234756			eDITQSAQHALR	Unambiguous	N-Term(iTRAQ4plex)	HCD	1.6949131	0	1	5	1.63513	1.7886	1.6093	1.7603046	5.257330276	198404.17	181380.4	324417.16	319284.75	6.33918498	1.127590849	2	High	1451	Unique	Used	49138	1	23.007	1	4.2835	0.0063	1	16	20/22	28.15586	1451	1512.79	MS2	756.898	233	Glioma_grade 2_2.raw
24234756			ekPTTALLDk	Unambiguous	N-Term(iTRAQ4plex); K2(iTRAQ4plex); K10(iTRAQ4plex)	HCD	2.0296428	0	1	5	1.87002	2.1234	1.9471	2.2108867	7.688820204	187857.23	165444.61	351297.25	365779.3	8.971431106	2.856140188	2	High	1390	Unique	Used	78877	1	22.158	1	3.8337	-7.8687	1	28	18/18	30.11708	1390	1547.92	MS2	774.466	166	Glioma_grade 2_6.raw
24234756			vLGmDPLPSk	Unambiguous	N-Term(iTRAQ4plex); M4(Oxidation); K10(iTRAQ4plex)	HCD	1.5126238	0	1	5	1.35119	1.5248	1.5019	1.6948001	9.267964645	165973.23	147078.9	224260.75	249269.36	8.535516988	7.468905291	2	High	2616	Unique	Used	58281	1	30.975	1	3.2763	1.0218	1	23	15/18	28.94968	2616	1360.78	MS2	680.892	211	Glioma_grade 2_3.raw
24234756			vLGmDPLPSk	Unambiguous	N-Term(iTRAQ4plex); M4(Oxidation); K10(iTRAQ4plex)	HCD	1.6458013	0	1	5	1.59389	1.6132	1.678	1.6983361	3.049341341	127986.48	126456	203996.98	214764.8	0.850654173	3.636434319	2	High	2576	Unique	Used	58243	1	30.705	1	3.8247	-9.5637	1	49	15/18	29.97886	2576	1360.76	MS2	680.885	219	Glioma_grade 2_3.raw
24234756			eATDAIGHLDR	Unambiguous	N-Term(iTRAQ4plex)	HCD	2.3142834	0	1	5	1.96766	1.9784	2.6484	2.6627934	17.03060652	127570.65	126879.086	251015.97	337852.78	0.38436636	20.85452733	3	High	1441	Unique	Used	57181	1	22.435	1	4.6479	-0.5253	1	2	27/40	2.280845	1441	1341.69	MS2	447.901	166	Glioma_grade 2_3.raw
24234756			lNQLkPGLQYk	Unambiguous	N-Term(iTRAQ4plex); K5(iTRAQ4plex); K11(iTRAQ4plex)	HCD	2.774859	0	1	5	2.6496	2.7557	2.7933	2.9051294	3.798793148	79964.38	76885.45	211873.97	223362.17	2.776072224	3.732862865	2	High	2452	Unique	Used	90212	1	30.614	1	4.0327	11.931	1	62	16/20	7.77807	2452	1734.08	MS2	867.546	41	Glioma_grade 2_7rep.raw
24234756			eDITQSAQHALR	Unambiguous	N-Term(iTRAQ4plex)	HCD	1.5584643	0	1	5	1.36203	1.6167	1.5094	1.7916291	11.52583377	78346.54	66004.25	106710.414	118255.13	12.09181738	7.257419803	3	High	1466	Unique	Used	49152	1	23.12	1	5.7862	-1.8988	1	4	28/44	8.638102	1466	1512.79	MS2	504.933	211	Glioma_grade 2_2.raw
24234756			lFPDTPLALDANk	Unambiguous	N-Term(iTRAQ4plex); K13(iTRAQ4plex)	HCD	2.8459554	0	1	5	2.5586	3.033	2.6881	3.1865985	10.20665108	78228.17	65991.414	200154.36	210288.14	11.99933172	3.491677668	2	High	6194	Unique	Used	53610	1	57.096	1	4.8599	0.7781	1	45	19/24	18.80362	6194	1702.96	MS2	851.985	220	Glioma_grade 2_2.raw

24234756			lFPDTPLALDANk	Unambiguous	N-Term(iTRAQ4plex); K13(iTRAQ4plex)	HCD	3.2776475	0	1	5	3.1685	3.2896	3.2661	3.3909726	2.785705828	72677.85	70001.85	230279.42	237374.36	2.65239939	2.145553142	2	High	5996	Unique	Used	61420	1	57.138	1	5.0631	1.6383	1	42	20/24	27.76775	5996	1702.96	MS2	851.986	234	Glioma_grade 2_3.raw
24234756			aYAALAALEk	Unambiguous	N-Term(iTRAQ4plex); K10(iTRAQ4plex)	HCD	1.7626626	0	1	5	1.69159	1.6662	1.8606	1.8326707	5.564204537	62707.87	63664.5	106075.8	116676.06	1.070549773	6.729924256	2	High	4590	Unique	Used	60116	1	46.153	1	3.7368	0.2336	1	57	16/18	53.51265	4590	1308.78	MS2	654.892	245	Glioma_grade 2_3.raw
24234756			eDITQSAQHALR	Unambiguous	N-Term(iTRAQ4plex)	HCD	2.579122	0	1	5	2.90644	2.5412	2.6224	2.2929313	9.759980051	58465.63	66867.58	169926.88	153322.77	9.480449468	7.264279344	3	High	1483	Unique	Used	57221	1	22.716	1	6.0028	-1.1121	1	3	28/44	6.671403	1483	1512.79	MS2	504.934	159	Glioma_grade 2_3.raw
24234756			eDITQSAQHALR	Unambiguous	N-Term(iTRAQ4plex)	HCD	1.8981315	0	1	5	1.96261	1.8054	1.999	1.8388196	4.934149793	52884.707	57490.84	103792.016	105715.29	5.901720024	1.298245979	2	High	1492	Unique	Used	57230	1	22.767	1	3.8432	1.2167	1	21	21/22	10.32384	1492	1512.79	MS2	756.899	237	Glioma_grade 2_3.raw
24234756			ePPLSLTIHLTSPVVR	Unambiguous	N-Term(iTRAQ4plex)	HCD	1.5911726	0	1	5	1.51749	1.7227	1.4753	1.674817	7.491834743	47585.742	41917.76	72210.945	70204.58	8.955780318	1.992359045	3	High	5764	Unique	Used	68867	1	54.775	1	4.8007	0.6052	1	19	20/60	38.49038	5764	1903.11	MS2	635.043	100	Glioma_grade 2_4.raw
24234756			ePPLSLTIHLTSPVVR	Unambiguous	N-Term(iTRAQ4plex)	HCD	1.5673112	0	1	5	1.52823	1.6135	1.5236	1.608578	3.138531635	43751.746	41439.836	66862.51	66659.2	3.837860972	0.215338584	3	High	5681	Unique	Used	61125	1	54.715	1	4.3292	1.0863	1	14	22/60	25.67868	5681	1903.12	MS2	635.043	101	Glioma_grade 2_3.raw
24234756			lFPDTPLALDANk	Unambiguous	N-Term(iTRAQ4plex); K13(iTRAQ4plex)	HCD	3.527231	0	1	5	3.63806	3.2621	3.8229	3.4278584	6.913213187	34444.17	38413.887	125309.83	131677.38	7.705431426	3.504094842	2	High	6065	Unique	Used	69147	1	57.124	1	4.6361	2.5701	1	72	19/24	8.410089	6065	1702.97	MS2	851.987	220	Glioma_grade 2_4.raw
24234756			lAAFGQLHk	Unambiguous	N-Term(iTRAQ4plex); K9(iTRAQ4plex)	HCD	2.8299584	0	1	5	2.70476	2.9207	2.7459	2.9651582	4.518411271	26478.969	24520.82	71619.17	72708.11	5.42990653	1.067014993	3	High	2239	Unique	Used	79668	1	28.436	1	4.6208	2.8289	1	100	20/32	74.42854	2239	1272.77	MS2	424.928	187	Glioma_grade 2_6.raw
24234756			aYAALAALEk	Unambiguous	N-Term(iTRAQ4plex); K10(iTRAQ4plex)	HCD	3.2843375	0	1	5	2.87348	3.0144	3.5416	3.7153509	12.34363622	6065.292	5781.711	17428.47	21481.086	3.385194519	14.72970936	3	High	4622	Unique	Used	60146	1	46.401	1	3.9285	-4.4635	1	69	19/36	17.2013	4622	1308.77	MS2	436.928	252	Glioma_grade 2_3.raw
25121993	NP_733839.1	MSI2	RNA-binding protein Musashi homolog 2 isoform b [Homo sapiens]	2	3	2.244668961	30.46172831	13.547775	9.96	1.05E-04	251	28.402	8.4995	RNA binding protein	Regulation of nucleobase, nucleoside, nucleotide and nucleic acid metabolism	RNA binding	Cytoplasm	-																								
25121993			vEDAMLMFDk	Unambiguous	N-Term(iTRAQ4plex); K10(iTRAQ4plex)	HCD	1.8886676	0	1	2	1.88333	1.9252	1.853	1.894121	1.578234619	40725.242	39840.125	76699.17	75462.02	1.553700445	1.149829538	2	High	6162	Unique	Used	53580	1	56.854	1	3.6153	-0.5601	1	91	17/18	44.67365	6162	1486.75	MS2	743.879	183	Glioma_grade 2_2.raw
25121993			gFGFVTFADPASVDk	Unambiguous	N-Term(iTRAQ4plex); K15(iTRAQ4plex)	HCD	3.3452976	0	1	2	3.17927	3.5455	3.1657	3.5304563	6.29388841	14880.942	13343.691	47310.484	47109.316	7.702495947	0.301308109	2	High	6603	Unique	Used	53995	1	60.071	1	5.592	-2.9234	1	76	20/28	13.06862	6603	1845.96	MS2	923.482	181	Glioma_grade 2_2.raw
25121993			gFGFVTFADPASVDk	Unambiguous	N-Term(iTRAQ4plex); K15(iTRAQ4plex)	HCD	2.244669	0	1	2	2.26037	2.3669	2.1279	2.2282245	4.383281555	7934.349	7577.2847	17934.59	16883.89	3.255396469	4.267602118	2	High	6463	Unique	Used	61858	1	60.747	1	4.3405	2.3669	1	100	22/28	0	6463	1845.97	MS2	923.487	218	Glioma_grade 2_3.raw
25952118	NP_741960.1	CAMK2A	calcium/calmodulin-dependent protein kinase type II subunit alpha isoform 2 [Homo sapiens]	8	17	0.371803552	27.09364275	190.29438	31.8	4.90E-05	478	54.054	7.0786	Serine/threonine kinase	Cell communication,Signal transduction	Protein serine/threonine kinase activity	Centrosome	Cytoplasm, Nucleus																								
25952118			iTAAEALk	Unambiguous	N-Term(iTRAQ4plex); K8(iTRAQ4plex)	HCD	0.26250446	0	1	2	0.25026	0.2637	0.2614	0.2753997	0.039225845	4.16E+07	3.94E+07	1.04E+07	1.09E+07	3.685937967	3.080172726	2	High	1735	Unique	Used	49407	1	24.959	1	3.671	0.3096	1	0	14/14	8.170938	1735	1104.69	MS2	552.847	234	Glioma_grade 2_2.raw
25952118			mLTINPSk	Unambiguous	N-Term(iTRAQ4plex); K8(iTRAQ4plex)	HCD	0.30610436	0	1	2	0.27144	0.2819	0.3294	0.34210664	0.113588983	1.46E+07	1.41E+07	3971088.2	4819633.5	2.668312424	13.65103245	2	High	1922	Unique	Used	57635	1	25.825	1	3.41	0.1948	1	0	14-Dec	10.45067	1922	1191.7	MS2	596.354	287	Glioma_grade 2_3.raw
25952118			iTAAEALk	Unambiguous	N-Term(iTRAQ4plex); K8(iTRAQ4plex)	HCD	0.48154113	0	1	2	0.51807	0.4247	0.5509	0.4515969	0.119823366	1944315.4	2371926.2	1007293.3	1071154.5	14.01063816	4.345231819	2	High	1773	Unique	Used	57494	1	24.779	1	3.3243	-6.3206	1	5	13/14	34.71405	1773	1104.68	MS2	552.844	345	Glioma_grade 2_3.raw
25952118			wQIVHFHR	Unambiguous	N-Term(iTRAQ4plex)	HCD	0.38146612	0	1	2	0.38682	0.4064	0.3578	0.3758378	0.053331394	975862.06	928902.8	377486.44	349116.78	3.486541765	5.521687329	2	High	2174	Unique	Used	89980	1	28.08	1	3.3781	-1.7148	1	11	13/14	25.53005	2174	1266.7	MS2	633.851	354	Glioma_grade 2_7rep.raw
25952118			mLTINPSk	Unambiguous	N-Term(iTRAQ4plex); K8(iTRAQ4plex)	HCD	0.63954675	0	1	2	0.60198	0.6748	0.6081	0.6816555	0.066103076	487970.47	435322.72	293749	296740.16	8.064086564	0.71637878	2	High	2018	Unique	Used	49676	1	26.89	1	3.1957	-6.9756	1	14	14-Dec	44.49035	2018	1191.69	MS2	596.35	290	Glioma_grade 2_2.raw
25952118			fYFENLWSR	Unambiguous	N-Term(iTRAQ4plex)	HCD	0.3517248	0	1	2	0.33714	0.3458	0.3575	0.36668196	0.036870432	354413.28	345534.25	119487.73	126701.17	1.79396942	4.143706186	2	High	5202	Unique	Used	87558	1	67.588	1	4.1066	2.2149	1	36	14/16	38.44195	5202	1405.71	MS2	703.356	180	Glioma_grade 2_1rep.raw
25952118			iTQYLDAGGIPR	Unambiguous	N-Term(iTRAQ4plex)	HCD	0.37180355	0	1	2	0.36363	0.359	0.3847	0.37987307	0.033378951	309476.06	313436	112535.03	119065.89	0.899035548	3.987907641	2	High	3877	Unique	Used	86618	1	52.643	1	4.2732	0.8885	1	23	19/22	29.29859	3877	1447.8	MS2	724.406	297	Glioma_grade 2_1rep.raw
25952118			wQIVHFHR	Unambiguous	N-Term(iTRAQ4plex)	HCD	0.3784705	0	1	2	0.35113	0.4064	0.3543	0.4101141	0.084439333	233484.39	201720.53	81983.8	82728.44	10.32177706	0.639345313	3	High	2267	Unique	Used	90058	1	28.922	1	3.7804	1.1175	1	11	22/28	11.38952	2267	1266.7	MS2	422.905	295	Glioma_grade 2_7rep.raw
25952118			wQIVHFHR	Unambiguous	N-Term(iTRAQ4plex)	HCD	0.40997583	0	1	2	0.40785	0.4305	0.3905	0.4122224	0.040085971	216491.16	205081.48	88295.41	84539.18	3.827507449	3.073523309	3	High	2154	Unique	Used	89963	1	27.904	1	4.1449	-0.3281	1	14	18/28	2.853831	2154	1266.7	MS2	422.904	260	Glioma_grade 2_7rep.raw
25952118			fTEEYQLFEELGk	Unambiguous	N-Term(iTRAQ4plex); K13(iTRAQ4plex)	HCD	0.2143154	0	1	2	0.20362	0.2125	0.216	0.22547671	0.042133943	182775.9	175099.55	37217.355	39480.867	3.033457109	4.173616135	2	High	7410	Unique	Used	54737	1	66.354	1	5.9662	1.1213	1	35	22/24	17.72952	7410	1920.99	MS2	960.996	157	Glioma_grade 2_2.raw
25952118			iTQYLDAGGIPR	Unambiguous	N-Term(iTRAQ4plex)	HCD	0.524711	0	1	2	0.43656	0.5369	0.5148	0.63312584	0.152581821	125220.79	101813.164	54666.676	64460.543	14.58080678	11.62674631	2	High	4818	Unique	Used	75257	1	49.415	1	4.2153	3.7551	1	66	18/22	30.91292	4818	1447.81	MS2	724.408	309	Glioma_grade 2_5.raw
25952118			fTEEYQLFEELGk	Unambiguous	N-Term(iTRAQ4plex); K13(iTRAQ4plex)	HCD	0.3311269	0	1	2	0.29218	0.3231	0.3384	0.37419212	0.1026752	61452.92	55571.305	17955.525	20794.346	7.107810115	10.36054845	2	High	5179	Unique	Used	87541	1	67.333	1	5.4975	0.4858	1	100	18/24	53.71458	5179	1920.98	MS2	960.996	170	Glioma_grade 2_1rep.raw
25952118			fTEEYQLFEELGk	Unambiguous	N-Term(iTRAQ4plex); K13(iTRAQ4plex)	HCD	0.27134997	0	1	2	0.22624	0.2177	0.3271	0.31475654	0.211771873	57021.324	59265.184	12900.234	18654.104	2.728861068	25.78789956	3	High	7476	Unique	Used	54796	1	66.921	1	4.8069	0.9872	1	6	24/48	7.193805	7476	1920.99	MS2	641	146	Glioma_grade 2_2.raw
25952118			mcDPGmTAFEPEALGNLVEGLDFHR	Unambiguous	N-Term(iTRAQ4plex); C2(Methylthio); M6(Oxidation)	HCD	0.33392495	0	1	2	0.2592	0.2961	0.3671	0.4192815	0.213546825	34098.89	29851.357	8838.462	12516.122	9.393112704	24.35541076	3	High	8205	Unique	Used	71031	1	75.728	1	8.5386	-2.8829	1	9	29/96	10.48123	8205	2955.32	MS2	985.778	43	Glioma_grade 2_4.raw
25952118			vTEQLIEAISNGDFESYTk	Unambiguous	N-Term(iTRAQ4plex); K19(iTRAQ4plex)	HCD	0.43417385	0	1	2	0.40882	0.3756	0.4979	0.45747268	0.12358908	10803.007	11757.2705	4416.452	5378.63	5.981896206	13.89192226	3	High	8144	Unique	Used	55404	1	72.328	1	5.3666	-1.8121	1	32	25/72	17.08004	8144	2432.24	MS2	811.418	121	Glioma_grade 2_2.raw
25952118			mcDPGMTAFEPEALGNLVEGLDFHR	Unambiguous	N-Term(iTRAQ4plex); M1(Oxidation); C2(Methylthio)	HCD	0.37937137	0	1	2	0.46068	0.4524	0.305	0.2995355	0.234937855	10313.185	10502.817	4751.029	3145.9668	1.288336474	28.74385132	3	High	8602	Unique	Used	55820	1	76.075	1	4.6705	-2.3253	1	33	29/96	33.40168	8602	2955.32	MS2	985.778	39	Glioma_grade 2_2.raw
25952118			fTEEYQLFEELGk	Unambiguous	N-Term(iTRAQ4plex); K13(iTRAQ4plex)	HCD	0.3460126	0	1	2	0.36527	0.3082	0.3908	0.3297619	0.105293478	8842.075	10477.925	3229.743	3455.2205	11.97433362	4.77000867	3	High	7385	Unique	Used	54716	1	66.124	1	5.3733	-1.3005	1	73	23/48	45.69989	7385	1920.98	MS2	640.999	148	Glioma_grade 2_2.raw
26051210	NP_742077.1	CAMK2B	calcium/calmodulin-dependent protein kinase type II subunit beta isoform 4 [Homo sapiens]	5	11	0.495601445	25.96547441	167.86206	28.82	1.05E-03	517	57.901	7.2837	Serine/threonine kinase	Cell communication,Signal transduction	Protein serine/threonine kinase activity	Cytoplasm	-																								
26051210			fYFENLLAk	Unambiguous	N-Term(iTRAQ4plex); K9(iTRAQ4plex)	HCD	0.33386996	0	1	7	0.32674	0.3412	0.3269	0.34131232	0.024967605	1304150.4	1248947.9	426121.5	426281.28	3.057779803	0.026508952	2	High	6803	Unique	Used	62175	1	63.396	1	4.4466	1.9746	1	11	16/16	17.12293	6803	1432.81	MS2	716.909	236	Glioma_grade 2_3.raw
26051210			fYFENLLAk	Unambiguous	N-Term(iTRAQ4plex); K9(iTRAQ4plex)	HCD	0.3452361	0	1	7	0.33883	0.3438	0.3466	0.35173905	0.015615208	1107674.6	1091529.4	375311.06	383933.5	1.038228414	1.606066376	2	High	7052	Unique	Used	54412	1	63.493	1	4.5636	0.5263	1	13	16/16	24.51332	7052	1432.81	MS2	716.908	241	Glioma_grade 2_2.raw
26051210			qETVEcLk	Unambiguous	N-Term(iTRAQ4plex); C6(Methylthio); K8(iTRAQ4plex)	HCD	0.39114296	0	1	8	0.37302	0.3974	0.3853	0.4104509	0.041033396	733316.56	688322.5	273541.53	282522.6	4.475904726	2.284116222	2	High	1962	Unique	Used	49623	1	26.508	1	3.7672	-8.5866	1	21	14/14	59.54644	1962	1283.65	MS2	642.327	140	Glioma_grade 2_2.raw
26051210			fYFENLLAk	Unambiguous	N-Term(iTRAQ4plex); K9(iTRAQ4plex)	HCD	0.36137396	0	1	7	0.3876	0.4055	0.3192	0.3339392	0.114763361	52316.445	50007.676	20277.75	16699.521	3.190931327	13.68510938	3	High	6820	Unique	Used	62191	1	63.52	1	3.2626	-1.4213	1	29	16/32	31.32841	6820	1432.81	MS2	478.273	226	Glioma_grade 2_3.raw
26051210			gTLPPAALEPQTTVIHNPVDGlk	Unambiguous	N-Term(iTRAQ4plex); K23(iTRAQ4plex)	HCD	0.3422218	0	1	4	0.29357	0.3914	0.3053	0.40708196	0.166501157	35705.38	26780.86	10482.148	10902.005	20.19833042	2.77667048	3	High	5196	Unique	Used	68346	1	50.31	1	6.5036	1.3246	1	23	27/88	15.20143	5196	2656.49	MS2	886.17	68	Glioma_grade 2_4.raw
26051210			wQNVHFHcSGAPVAPLQ	Unambiguous	N-Term(iTRAQ4plex); C8(Methylthio)	HCD	0.6141524	0	1	8	0.62308	0.6005	0.6283	0.60555065	0.021878227	33580.14	34844.176	20923.041	21099.916	2.612546181	0.595243747	2	High	5002	Unique	Used	82072	1	52.106	1	4.5167	-0.0123	1	64	13/32	24.74337	5002	2081	MS2	1041	95	Glioma_grade 2_6.raw
26051210			gTLPPAALEPQTTVIHNPVDGlk	Unambiguous	N-Term(iTRAQ4plex); K23(iTRAQ4plex)	HCD	0.6046797	0	1	4	0.48106	0.6383	0.5793	0.76871425	0.194922288	13567.227	10224.375	6526.6763	7859.6226	19.8704847	13.10323627	3	High	4945	Unique	Used	75373	1	50.402	1	6.5119	1.4625	1	35	28/88	5.184968	4945	2656.5	MS2	886.17	66	Glioma_grade 2_5.raw
26051210			gTLPPAALEPQTTVIHNPVDGlk	Unambiguous	N-Term(iTRAQ4plex); K23(iTRAQ4plex)	HCD	0.5372306	0	1	4	0.64014	0.6304	0.4426	0.43587887	0.210872985	12382.932	12573.635	7926.8496	5480.582	1.080656522	25.80318826	3	High	4743	Unique	Used	75190	1	48.805	1	6.1131	3.5303	1	49	26/88	18.02612	4743	2656.5	MS2	886.172	57	Glioma_grade 2_5.raw
26051210			gTLPPAALEPQTTVIHNPVDGlk	Unambiguous	N-Term(iTRAQ4plex); K23(iTRAQ4plex)	HCD	0.49560145	0	1	4	0.4218	0.5502	0.4537	0.5918803	0.158379863	8564.8	6565.492	3612.609	3885.9856	18.6873359	5.155804734	3	High	5189	Unique	Used	75590	1	52.392	1	6.2008	0.7043	1	60	24/88	14.61551	5189	2656.49	MS2	886.169	77	Glioma_grade 2_5.raw
26051210			gTLPPAALEPQTTVIHNPVDGlk	Unambiguous	N-Term(iTRAQ4plex); K23(iTRAQ4plex)	HCD	0.56933886	0	1	4	0.5576	0.5841	0.5553	0.5816387	0.026892536	4751.159	4535.7466	2649.2307	2638.1655	3.280308326	0.295959586	3	High	5444	Unique	Used	68577	1	52.206	1	5.7576	0.1529	1	100	26/88	13.38229	5444	2656.49	MS2	886.169	79	Glioma_grade 2_4.raw
26051210			icDPGLTSFEPEALGNLVEGmDFHR	Unambiguous	N-Term(iTRAQ4plex); C2(Methylthio); M21(Oxidation)	HCD	0.64641726	0	1	7	0.49283	0.7509	0.5778	0.88042593	0.257243985	1245.619	817.5168	613.88367	719.763	29.34503571	11.22756033	3	High	8075	Unique	Used	70923	1	74.497	1	5.7744	-5.7669	1	100	27/96	7.438884	8075	2953.35	MS2	985.121	39	Glioma_grade 2_4.raw
26667186	NP_742125.1	CAMK2D	calcium/calmodulin-dependent protein kinase type II subunit delta isoform 1 [Homo sapiens]	5	11	0.484251946	14.62795557	123.06871	24.9	1.49E-04	478	54.093	7.2544	Serine/threonine kinase	Regulation of cell growth	Protein serine/threonine kinase activity	Cytoplasm	Nucleus																								
26667186			iPTGQEYAAk	Unambiguous	N-Term(iTRAQ4plex); K10(iTRAQ4plex)	HCD	0.6023267	0	1	5	0.49272	0.5797	0.6216	0.7312732	0.16344747	586353.9	498411.3	288908.2	364474.84	11.46511868	16.35600568	2	High	1515	Unique	Used	49199	1	23.435	1	3.0169	0.8769	1	6	13/18	30.23843	1515	1365.76	MS2	683.385	297	Glioma_grade 2_2.raw
26667186			fYFENALSk	Unambiguous	N-Term(iTRAQ4plex); K9(iTRAQ4plex)	HCD	0.4528637	0	1	5	0.4227	0.4724	0.4354	0.48656988	0.06631654	479382.88	428985.62	202635.62	208731.48	7.846208736	2.095658084	2	High	5479	Unique	Used	52934	1	51.924	1	4.1101	-0.3915	1	13	15/16	46.78579	5479	1406.76	MS2	703.881	214	Glioma_grade 2_2.raw
26667186			lTQYMDGSGmPk	Unambiguous	N-Term(iTRAQ4plex); M10(Oxidation); K12(iTRAQ4plex)	HCD	0.5183607	0	1	5	0.49465	0.5322	0.5055	0.54387105	0.044034907	216413.42	201149.83	107048.8	109399.57	5.169510485	1.535927859	2	High	2320	Unique	Used	49961	1	29.02	1	4.276	-0.7464	1	36	20/22	20.89739	2320	1631.8	MS2	816.404	126	Glioma_grade 2_2.raw
26667186			fTDEYQLFEELGk	Unambiguous	N-Term(iTRAQ4plex); K13(iTRAQ4plex)	HCD	0.45883143	0	1	5	0.44166	0.4613	0.4565	0.4767647	0.031519165	103802.92	99380.164	45845.836	47380.953	3.078367251	2.328711848	2	High	7360	Unique	Used	54696	1	65.875	1	5.7233	2.3589	1	43	21/24	13.73183	7360	1906.97	MS2	953.99	159	Glioma_grade 2_2.raw
26667186			fTDEYQLFEELGk	Unambiguous	N-Term(iTRAQ4plex); K13(iTRAQ4plex)	HCD	0.48425195	0	1	5	0.43587	0.4602	0.507	0.5353411	0.092628221	75497.51	71500.516	32906.918	38277.16	3.845359885	10.66905589	2	High	7285	Unique	Used	54632	1	65.196	1	5.9625	-2.7622	1	100	20/24	30.0524	7285	1906.96	MS2	953.985	147	Glioma_grade 2_2.raw
26667186			lTQYMDGSGMPk	Unambiguous	N-Term(iTRAQ4plex); K12(iTRAQ4plex)	HCD	0.42447802	0	1	5	0.40223	0.4166	0.4321	0.44751918	0.046056174	68092.555	65751.47	27388.805	29425.045	2.473621185	5.068620106	2	High	3886	Unique	Used	51436	1	40.231	1	4.9382	1.2364	1	75	21/22	0	3886	1615.81	MS2	808.408	164	Glioma_grade 2_2.raw
26667186			lTQYMDGSGMPk	Unambiguous	N-Term(iTRAQ4plex); K12(iTRAQ4plex)	HCD	0.5592625	0	1	5	0.57832	0.4878	0.6439	0.54318947	0.116062813	66692.625	79060.88	38569.44	42945.04	12.00064037	7.591329618	2	High	3815	Unique	Used	59397	1	40.116	1	5.6716	-2.692	1	100	21/22	0	3815	1615.8	MS2	808.405	158	Glioma_grade 2_3.raw
26667186			icDPGLTAFEPEALGNLVEGMDFHR	Unambiguous	N-Term(iTRAQ4plex); C2(Methylthio)	HCD	0.46072552	0	1	5	0.47815	0.5635	0.3735	0.44018728	0.170897611	55831.11	47376.082	26695.838	20854.35	11.5856415	17.37345719	3	High	8281	Unique	Used	71101	1	76.301	1	7.9373	-2.69	1	3	32/96	5.569886	8281	2921.37	MS2	974.461	93	Glioma_grade 2_4.raw
26667186			lTQYmDGSGMPk	Unambiguous	N-Term(iTRAQ4plex); M5(Oxidation); K12(iTRAQ4plex)	HCD	0.6269661	0	1	5	0.55137	0.6721	0.59	0.719112	0.120486207	54605.473	44798.61	30107.773	32215.217	13.95214184	4.782145219	2	High	2048	Unique	Used	49705	1	27.084	1	3.3459	-0.1479	1	70	16/22	0	2048	1631.8	MS2	816.404	136	Glioma_grade 2_2.raw
26667186			icDPGLTAFEPEALGNLVEGmDFHR	Unambiguous	N-Term(iTRAQ4plex); C2(Methylthio); M21(Oxidation)	HCD	0.39442205	0	1	5	0.3333	0.5105	0.3187	0.4880418	0.243908171	11766.424	7682.5312	3921.7004	3749.3962	29.69566501	3.176533281	3	High	8136	Unique	Used	70972	1	75.111	1	8.4336	-3.2091	1	22	33/96	6.872916	8136	2937.36	MS2	979.792	73	Glioma_grade 2_4.raw
26667186			icDPGLTAFEPEALGNLVEGMDFHR	Unambiguous	N-Term(iTRAQ4plex); C2(Methylthio)	HCD	0.5257504	0	1	5	0.53226	0.5864	0.4707	0.5185843	0.090273356	9886.097	8973.775	5261.926	4653.659	6.841075834	8.675428035	3	High	8351	Unique	Used	63563	1	76.417	1	4.8388	0.1932	1	12	29/96	12.39069	8351	2921.38	MS2	974.464	102	Glioma_grade 2_3.raw
27436893	NP_003181.3	TAPBP	tapasin isoform 1 precursor [Homo sapiens]	2	2	3.298223495	31.45777443	16.845798	7.81	9.35E-05	448	47.541	6.9907	Chaperone	Protein folding	Chaperone activity	Endoplasmic reticulum	Golgi apparatus																								
27436893			fVPLPASAk	Unambiguous	N-Term(iTRAQ4plex); K9(iTRAQ4plex)	HCD	4.0318804	0	1	2	3.49528	4.4816	3.6811	4.7199016	14.59196217	88432.85	68970.53	309097.5	325534.12	17.48620449	3.662737593	2	High	3204	Unique	Used	66505	1	34.684	1	2.9584	0.8217	1	27	13/16	19.58342	3204	1217.75	MS2	609.379	215	Glioma_grade 2_4.raw
27436893			hHSDGSVSLSGHLQPPPVTTEQHGAR	Unambiguous	N-Term(iTRAQ4plex)	HCD	2.5645664	0	1	3	2.80181	2.0655	3.2415	2.3896682	19.42408498	641.13214	869.6714	1796.33	2078.226	21.39280935	10.28920853	5	High	1795	Unique	Used	94681	1	27.276	1	7.9638	0.946	1	93	34/200	15.09653	1795	2875.43	MS2	575.893	114	Glioma_grade 2_8rep.raw
27477041	NP_036437.1	AP2A2	AP-2 complex subunit alpha-2 isoform 2 [Homo sapiens]	12	21	0.444018006	22.68345131	213.75617	30.56	7.60E-05	939	103.89	6.9614	Transport/cargo protein	Transport	Transporter activity	Plasma membrane	-																								

27477041			lTEcLETILNk	Unambiguous	N-Term(iTRAQ4plex); C4(Methylthio); K11(iTRAQ4plex)	HCD	0.35944006	0	1	2	0.32166	0.3458	0.3721	0.4000506	0.093845021	1302562	1211647.5	418987.28	484720.3	5.113834743	10.28657172	2	High	8270	Unique	Used	63486	1	75.856	1	5.3091	-0.7041	1	5	17/20	11.6614	8270	1610.87	MS2	805.94	256	Glioma_grade 2_3.raw
27477041			vLVAGDTMDSVk	Unambiguous	N-Term(iTRAQ4plex); K12(iTRAQ4plex)	HCD	0.42370915	0	1	2	0.39414	0.4424	0.407	0.4569023	0.069214189	459318.53	409174.5	181035.69	186952.77	8.165220082	2.273988371	2	High	3128	Unique	Used	50719	1	34.82	1	4.9757	-1.8782	1	17	20/22	13.93012	3128	1522.84	MS2	761.922	258	Glioma_grade 2_2.raw
27477041			qLSNPQQEVQNIFk	Unambiguous	N-Term(iTRAQ4plex); K14(iTRAQ4plex)	HCD	0.4470425	0	1	2	0.416	0.4382	0.4554	0.47973904	0.060252684	258180.95	245084.05	107404.46	117576.38	3.680330165	6.393996582	2	High	5576	Unique	Used	53027	1	52.585	1	6.2494	-2.2989	1	28	20/26	4.453231	5576	1961.07	MS2	981.036	214	Glioma_grade 2_2.raw
27477041			tVFEALQAPAcHENLVk	Unambiguous	N-Term(iTRAQ4plex); C11(Methylthio); K17(iTRAQ4plex)	HCD	0.27178395	0	1	2	0.2767	0.2583	0.2862	0.26719776	0.044235638	246382.62	263920.84	68173.23	70519.06	4.860399846	2.391989202	3	High	5574	Unique	Used	75936	1	55.508	1	7.1731	-0.4112	1	4	36/64	5.999711	5574	2204.14	MS2	735.386	146	Glioma_grade 2_5.raw
27477041			vLVAGDTmDSVk	Unambiguous	N-Term(iTRAQ4plex); M8(Oxidation); K12(iTRAQ4plex)	HCD	0.444018	0	1	2	0.43543	0.4414	0.4466	0.45272073	0.01661849	238797.33	235588.48	103980.06	106655.79	0.956605171	1.796490795	2	High	1652	Unique	Used	49329	1	24.378	1	4.4204	1.6045	1	29	19/22	16.88711	1652	1538.84	MS2	769.922	232	Glioma_grade 2_2.raw
27477041			kGPSTVTDLEDTk	Unambiguous	N-Term(iTRAQ4plex); K1(iTRAQ4plex); K13(iTRAQ4plex)	HCD	0.56698406	1	1	2	0.53098	0.5645	0.5693	0.60526377	0.053568302	201944.7	189959.77	107227.82	114975.77	4.324842365	4.931176184	2	High	1397	Unique	Used	78884	1	22.202	1	4.9627	-9.9189	1	73	21/24	7.568727	1397	1822.99	MS2	912.001	212	Glioma_grade 2_6.raw
27477041			tHIETVINALk	Unambiguous	N-Term(iTRAQ4plex); K11(iTRAQ4plex)	HCD	0.32821354	0	1	2	0.30014	0.3371	0.3203	0.3597391	0.076790003	198465.31	176705.69	59568.258	63567.945	8.20232633	4.59362191	3	High	3459	Unique	Used	74066	1	37.885	1	5.3581	1.483	1	7	21/40	19.04009	3459	1526.92	MS2	509.644	114	Glioma_grade 2_5.raw
27477041			fVNLFPEVkPTIQDVLR	Unambiguous	N-Term(iTRAQ4plex); K9(iTRAQ4plex)	HCD	0.36344942	0	1	2	0.38157	0.3947	0.3333	0.34470803	0.080433051	193282.7	186866.94	73750.63	64414.53	2.386758752	9.556127782	3	High	6756	Unique	Used	83512	1	68.559	1	4.9082	-1.4593	1	9	22/64	10.4512	6756	2303.33	MS2	768.449	70	Glioma_grade 2_6.raw
27477041			kGPSTVTDLEDTk	Unambiguous	N-Term(iTRAQ4plex); K1(iTRAQ4plex); K13(iTRAQ4plex)	HCD	0.44873565	1	1	2	0.42806	0.4394	0.4579	0.4699532	0.041658977	189176.11	184311.17	80979.43	86617.625	1.842114711	4.757608561	3	High	1404	Unique	Used	78890	1	22.265	1	6.213	-10.529	1	7	20/48	19.05783	1404	1822.99	MS2	608.336	174	Glioma_grade 2_6.raw
27477041			emAEAFAGEIPk	Unambiguous	N-Term(iTRAQ4plex); M2(Oxidation); K12(iTRAQ4plex)	HCD	0.65012264	0	1	2	0.62624	0.6599	0.6408	0.67528874	0.033027691	130956.38	124274.35	82010.21	83921.07	3.702460691	1.6286044	2	High	3231	Unique	Used	50817	1	35.543	1	4.3709	-4.1779	1	49	18/22	13.67093	3231	1596.81	MS2	798.909	174	Glioma_grade 2_2.raw
27477041			eMAEAFAGEIPk	Unambiguous	N-Term(iTRAQ4plex); K12(iTRAQ4plex)	HCD	0.62743324	0	1	2	0.56597	0.6367	0.6192	0.6965753	0.085493414	108452.336	96409.39	61380.656	67156.4	8.313557587	6.354693154	2	High	5050	Unique	Used	52531	1	48.779	1	5.0239	-4.348	1	73	20/22	32.61985	5050	1580.82	MS2	790.912	229	Glioma_grade 2_2.raw
27477041			aVDLLYAMcDR	Unambiguous	N-Term(iTRAQ4plex); C9(Methylthio)	HCD	0.44818434	0	1	2	0.42274	0.4595	0.4377	0.4758468	0.052209245	71645.54	65908.01	30287.229	31362.115	5.898861019	2.465749448	2	High	5793	Unique	Used	87993	1	74.17	1	4.1526	0.6647	1	100	17/20	46.45333	5793	1459.69	MS2	730.347	96	Glioma_grade 2_1rep.raw
27477041			tHIETVINALk	Unambiguous	N-Term(iTRAQ4plex); K11(iTRAQ4plex)	HCD	0.49947023	0	1	2	0.47771	0.5193	0.4812	0.52312595	0.048354	69756.91	64170.234	33323.504	33569.117	5.899291758	0.519263905	2	High	3522	Unique	Used	74125	1	38.35	1	4.0172	5.3491	1	78	19/20	0	3522	1526.92	MS2	763.965	121	Glioma_grade 2_5.raw
27477041			fHLcSVPTR	Unambiguous	N-Term(iTRAQ4plex); C4(Methylthio)	HCD	0.30634338	0	1	2	0.28995	0.3199	0.2941	0.3244265	0.057237071	69015.2	62560.07	20011.068	20296.145	6.938182527	1.000217405	3	High	3079	Unique	Used	73712	1	35.046	1	3.8241	2.7979	1	42	17/32	0	3079	1249.63	MS2	417.216	156	Glioma_grade 2_5.raw
27477041			nPTFMGLALHcIASVGSR	Unambiguous	N-Term(iTRAQ4plex); C11(Methylthio)	HCD	0.37477314	0	1	2	0.3313	0.3603	0.3881	0.42204982	0.103250943	65435.055	60164.45	21678.928	25392.395	5.934546536	11.15676182	3	High	7913	Unique	Used	77933	1	76.363	1	5.7227	-1.3779	1	12	35/68	15.4829	7913	2064.03	MS2	688.681	104	Glioma_grade 2_5.raw
27477041			tHIETVINALk	Unambiguous	N-Term(iTRAQ4plex); K11(iTRAQ4plex)	HCD	0.5654755	0	1	2	0.58003	0.5879	0.5434	0.55072606	0.038461057	41984.21	41424.094	24352.027	22813.328	0.949693982	4.613659739	3	High	3482	Unique	Used	80777	1	38.575	1	5.1623	0.7635	1	68	18/40	35.10686	3482	1526.92	MS2	509.643	133	Glioma_grade 2_6.raw
27477041			fVNLFPEVkPTIQDVLR	Unambiguous	N-Term(iTRAQ4plex); K9(iTRAQ4plex)	HCD	0.4094897	0	1	2	0.37086	0.438	0.3853	0.4551137	0.098413609	25595.277	21670.338	9492.314	9862.468	11.74363639	2.704638094	2	High	6770	Unique	Used	83523	1	68.699	1	4.336	1.1964	1	100	20/32	19.12007	6770	2303.34	MS2	1152.17	82	Glioma_grade 2_6.raw
27477041			sSPLIQFHLLHSk	Unambiguous	N-Term(iTRAQ4plex); K13(iTRAQ4plex)	HCD	0.43369383	0	1	2	0.43078	0.4866	0.3869	0.43698213	0.093757423	23534.525	20836.004	10138.263	9104.961	8.600945453	7.593892283	4	High	3491	Unique	Used	86396	1	47.516	1	6.2557	2.6513	1	64	27/72	21.50135	3491	1795.05	MS2	449.518	120	Glioma_grade 2_1rep.raw
27477041			sSPLIQFHLLHSk	Unambiguous	N-Term(iTRAQ4plex); K13(iTRAQ4plex)	HCD	0.5015757	0	1	2	0.47919	0.4833	0.5197	0.5241566	0.047119016	18200.816	18046.973	8721.57	9459.438	0.600221043	5.739521884	3	High	3496	Unique	Used	86398	1	47.595	1	4.8792	2.6928	1	84	26/48	59.7242	3496	1795.05	MS2	599.022	94	Glioma_grade 2_1rep.raw
27477041			sSPLIQFHLLHSk	Unambiguous	N-Term(iTRAQ4plex); K13(iTRAQ4plex)	HCD	0.36754718	0	1	2	0.36674	0.3964	0.3409	0.36841735	0.06160196	15725.928	14550.284	5767.3594	5360.577	5.491478555	5.169666381	4	High	3621	Unique	Used	95863	1	49.97	1	6.2957	3.4673	1	100	29/72	17.8681	3621	1795.05	MS2	449.519	123	Glioma_grade 2_8rep.raw
27477041			tVFEALQAPAcHENLVk	Unambiguous	N-Term(iTRAQ4plex); C11(Methylthio); K17(iTRAQ4plex)	HCD	0.49804175	0	1	2	0.51293	0.5258	0.471	0.48278	0.051251998	3733.4548	3642.2795	1915.0038	1758.4198	1.748183172	6.028251587	4	High	5587	Unique	Used	75948	1	55.604	1	5.9061	-1.1096	1	66	25/96	12.69425	5587	2204.14	MS2	551.791	166	Glioma_grade 2_5.raw
27735037	NP_775741.1	MMGT1	membrane magnesium transporter 1 precursor [Homo sapiens]	2	2	2.410765171	12.57990792	28.555912	24.43	5.21E-05	131	14.677	9.1587	Unclassified	Biological_process unknown	Molecular function unknown	-	-																								
27735037			nHPSFYVFNHR	Unambiguous	N-Term(iTRAQ4plex)	HCD	2.1963196	0	1	1	1.88767	2.1508	2.2363	2.547986	12.34014438	9681.571	8497.216	18275.639	21650.787	9.213656025	11.95493951	4	High	1725	Unique	Used	89585	1	24.385	1	5.3851	-1.008	1	36	25/60	24.73623	1725	1561.78	MS2	391.2	173	Glioma_grade 2_7rep.raw
27735037			gLVGIGLFALAHAAFSAAQHR	Unambiguous	N-Term(iTRAQ4plex)	HCD	2.625211	0	1	1	3.05488	2.7502	2.4863	2.2383847	13.3220806	470.91473	523.0762	1438.5901	1170.8458	7.421341189	14.51070786	4	High	6827	Unique	Used	93467	1	76.368	1	6.7871	-1.1595	1	78	39/120	19.5298	6827	2251.26	MS2	563.569	132	Glioma_grade 2_7rep.raw
27764867	NP_003170.1	SYP	synaptophysin [Homo sapiens]	3	14	0.349502832	24.54339193	60.419863	10.22	1.63E-04	313	33.823	4.8052	Transport/cargo protein	Transport	Transporter activity	Cytoplasm	Endosome, Nucleus																								
27764867			mATDPENIIk	Unambiguous	N-Term(iTRAQ4plex); K10(iTRAQ4plex)	HCD	0.26050293	0	1	1	0.23869	0.2627	0.2585	0.2845161	0.07199496	2.63E+07	2.39E+07	6266249.5	6786230	6.77756633	5.633898718	2	High	2871	Unique	Used	50481	1	32.898	1	3.5247	0.6406	1	0	17/18	7.505084	2871	1419.78	MS2	710.392	226	Glioma_grade 2_2.raw
27764867			mATDPENIIk	Unambiguous	N-Term(iTRAQ4plex); M1(Oxidation); K10(iTRAQ4plex)	HCD	0.314577	0	1	1	0.29338	0.3308	0.3002	0.33847597	0.070523051	4181121.2	3707834.5	1226671.4	1255012.9	8.48437354	1.615069801	2	High	1786	Unique	Used	49455	1	25.321	1	3.3786	1.1994	1	1	18/18	7.546511	1786	1435.77	MS2	718.39	199	Glioma_grade 2_2.raw
27764867			lHQVYFDAPTcR	Unambiguous	N-Term(iTRAQ4plex); C11(Methylthio)	HCD	0.30875468	0	1	1	0.27544	0.3002	0.3166	0.34505576	0.09446769	2795678.8	2565352.5	770053.75	885189.7	6.075893965	9.837031647	3	High	3534	Unique	Used	74135	1	38.463	1	4.6164	2.2196	1	0	22/44	7.020886	3534	1639.79	MS2	547.267	80	Glioma_grade 2_5.raw
27764867			lHQVYFDAPTcR	Unambiguous	N-Term(iTRAQ4plex); C11(Methylthio)	HCD	0.2634361	0	1	1	0.24571	0.2675	0.2597	0.28273925	0.058570687	2590797.2	2379526	636577.2	672785.44	6.011331343	3.910771738	2	High	3545	Unique	Used	74144	1	38.564	1	3.7896	1.9672	1	2	17/22	3.692067	3545	1639.79	MS2	820.397	105	Glioma_grade 2_5.raw
27764867			lHQVYFDAPTcR	Unambiguous	N-Term(iTRAQ4plex); C11(Methylthio)	HCD	0.45857382	0	1	1	0.42517	0.4442	0.4724	0.49347308	0.06577392	1833549.9	1755142	779564.8	866115.4	3.089858886	7.437716778	2	High	3458	Unique	Used	74065	1	37.88	1	3.7675	2.7116	1	4	17/22	20.9004	3458	1639.79	MS2	820.398	116	Glioma_grade 2_5.raw
27764867			mATDPENIIk	Unambiguous	N-Term(iTRAQ4plex); M1(Oxidation); K10(iTRAQ4plex)	HCD	0.35076258	0	1	1	0.34506	0.3402	0.3615	0.3563849	0.028107558	396570.12	402266.1	136840.28	143361.56	1.008383441	3.291371184	2	High	1695	Unique	Used	49370	1	24.665	1	3.5096	4.2602	1	21	17/18	27.69069	1695	1435.78	MS2	718.392	200	Glioma_grade 2_2.raw
27764867			mATDPENIIk	Unambiguous	N-Term(iTRAQ4plex); K10(iTRAQ4plex)	HCD	0.3986053	0	1	1	0.37175	0.4122	0.3863	0.42838037	0.063615797	265434.8	239375.48	98676.305	102543.76	7.300454295	2.718122227	2	High	2974	Unique	Used	50577	1	33.657	1	3.46	1.2425	1	18	17/18	10.21803	2974	1419.78	MS2	710.392	239	Glioma_grade 2_2.raw
27764867			lHQVYFDAPTcR	Unambiguous	N-Term(iTRAQ4plex); C11(Methylthio)	HCD	0.30398732	0	1	1	0.29568	0.2936	0.3144	0.31224123	0.035685107	220315.05	221846.33	65141.887	69269.57	0.48976619	4.342952164	3	High	3442	Unique	Used	74050	1	37.765	1	4.8223	1.6612	1	6	22/44	12.81015	3442	1639.79	MS2	547.267	82	Glioma_grade 2_5.raw
27764867			lHQVYFDAPTcR	Unambiguous	N-Term(iTRAQ4plex); C11(Methylthio)	HCD	0.3482431	0	1	1	0.29484	0.3405	0.355	0.4099135	0.135489896	148338.14	128458.8	43735.625	52656.996	10.15677133	13.08888972	3	High	3606	Unique	Used	74193	1	39.155	1	4.2463	0.8796	1	5	21/44	7.118756	3606	1639.79	MS2	547.267	81	Glioma_grade 2_5.raw
27764867			lHQVYFDAPTcR	Unambiguous	N-Term(iTRAQ4plex); C11(Methylthio)	HCD	0.37060815	0	1	1	0.34252	0.3631	0.3777	0.4003806	0.065686176	98369.766	92792.06	33693.99	37152.14	4.126382153	6.903076612	2	High	3619	Unique	Used	74204	1	39.269	1	3.9498	1.7439	1	51	17/22	16.6699	3619	1639.79	MS2	820.397	109	Glioma_grade 2_5.raw
27764867			vVkEPLGFVk	Unambiguous	N-Term(iTRAQ4plex); K3(iTRAQ4plex); K10(iTRAQ4plex)	HCD	0.36983576	1	1	1	0.37719	0.3934	0.3472	0.36216766	0.053634905	63523.98	60900.74	23960.434	22056.277	2.981579211	5.851971155	3	High	2707	Unique	Used	90432	1	32.807	1	3.8504	-7.0583	1	34	18/36	58.2885	2707	1547.98	MS2	516.664	50	Glioma_grade 2_7rep.raw
27764867			vVkEPLGFVk	Unambiguous	N-Term(iTRAQ4plex); K3(iTRAQ4plex); K10(iTRAQ4plex)	HCD	0.34442112	1	1	1	0.29096	0.3262	0.3607	0.40436378	0.140211209	59384.574	52967.133	17278.31	21417.99	8.077876465	15.12907332	3	High	2731	Unique	Used	90450	1	33.058	1	3.7577	7.0178	1	51	19/36	72.3051	2731	1548	MS2	516.671	32	Glioma_grade 2_7rep.raw
27764867			mATDPENIIk	Unambiguous	N-Term(iTRAQ4plex); M1(Oxidation); K10(iTRAQ4plex)	HCD	0.52641535	0	1	1	0.49949	0.5212	0.5314	0.55450827	0.043372622	56916.812	54543.26	28429.6	30244.69	3.011580173	4.374871677	2	High	1765	Unique	Used	57487	1	24.716	1	3.0107	-2.7966	1	100	18/18	0	1765	1435.77	MS2	718.387	203	Glioma_grade 2_3.raw
27764867			lHQVYFDAPTcR	Unambiguous	N-Term(iTRAQ4plex); C11(Methylthio)	HCD	0.5655225	0	1	1	0.5256	0.508	0.6251	0.60410297	0.101781144	2129.8977	2203.9043	1119.4774	1331.385	2.414995826	12.22763881	3	High	896	Unique	Used	78448	1	17.953	1	3.2306	0.0979	1	100	17/44	17.15616	896	1639.78	MS2	547.266	69	Glioma_grade 2_6.raw
28178825	NP_005887.2	IDH1	isocitrate dehydrogenase [NADP] cytoplasmic [Homo sapiens]	5	6	2.621839046	30.96472882	39.84399	20.77	6.25E-05	414	46.63	7.0054	Enzyme: Dehydrogenase	Metabolism,Energy pathways	Catalytic activity	Cytoplasm	Mitochondrion, Peroxisome																								
28178825			iIWELIk	Unambiguous	N-Term(iTRAQ4plex); K7(iTRAQ4plex)	HCD	3.213788	0	1	1	3.34631	3.2156	3.2119	3.0864441	3.300418249	136720.67	142277.86	457509.47	439132.66	2.816879883	2.898451128	2	High	6904	Unique	Used	69908	1	64.021	1	3.1129	-0.6632	1	19	12-Dec	28.02564	6904	1202.77	MS2	601.891	113	Glioma_grade 2_4.raw
28178825			gWPLYLSTk	Unambiguous	N-Term(iTRAQ4plex); K9(iTRAQ4plex)	HCD	2.0298903	0	1	1	1.97694	2.1172	1.9484	2.0865912	4.042705527	98815.305	92271.22	195352.39	192532.3	4.843216318	1.028194623	2	High	5149	Unique	Used	60635	1	50.522	1	3.6013	0.6291	1	35	14/16	22.58751	5149	1352.78	MS2	676.895	285	Glioma_grade 2_3.raw
28178825			aTDFVVPGPGk	Unambiguous	N-Term(iTRAQ4plex); K11(iTRAQ4plex)	HCD	1.5225018	0	1	1	1.47839	1.4756	1.5695	1.5665336	3.454035697	85121.83	85283.65	125842.95	133599.69	0.134296173	4.228174254	2	High	2569	Unique	Used	58236	1	30.662	1	3.7455	-9.6053	1	31	13/20	30.37694	2569	1375.77	MS2	688.388	300	Glioma_grade 2_3.raw
28178825			iIWELIk	Unambiguous	N-Term(iTRAQ4plex); K7(iTRAQ4plex)	HCD	3.234184	0	1	1	2.91784	3.4722	3.0341	3.6106336	10.27085334	63472.76	53338.36	185203.38	192585.27	12.26955612	2.763335784	2	High	6794	Unique	Used	62167	1	63.314	1	3.1373	-0.8662	1	64	12-Dec	20.05071	6794	1202.77	MS2	601.891	104	Glioma_grade 2_3.raw
28178825			lIFPYVELDLHSYDLGIENR	Unambiguous	N-Term(iTRAQ4plex)	HCD	3.2553062	0	1	1	3.60383	3.1038	3.4312	2.955138	9.064120772	16178.703	18784.887	58305.26	55511.934	10.54153981	3.470793273	3	High	8146	Unique	Used	70980	1	75.213	1	7.515	0.8696	1	25	33/76	20.08071	8146	2550.34	MS2	850.784	133	Glioma_grade 2_4.raw
28178825			sIEDFAHSSFQMALSk	Unambiguous	N-Term(iTRAQ4plex); K16(iTRAQ4plex)	HCD	1.9576167	0	1	1	1.76037	1.8859	2.0245	2.1689303	8.990790184	5003.0737	4669.9897	8807.269	10128.882	4.869728345	9.870237245	3	High	6184	Unique	Used	76480	1	60.516	1	5.7824	-1.358	1	100	21/60	51.72254	6184	2086.05	MS2	696.021	131	Glioma_grade 2_5.raw
29550838	NP_057632.2	GOLM1	Golgi membrane protein 1 [Homo sapiens]	2	2	2.545351982	33.44432549	13.715846	8.73	2.56E-04	401	45.306	4.9702	Transport/cargo protein	Transport	Transporter activity	Golgi apparatus	-																								
29550838			kNEFQGELEk	Unambiguous	N-Term(iTRAQ4plex); K1(iTRAQ4plex); K10(iTRAQ4plex)	HCD	1.9434092	1	1	1	1.81771	2.2477	1.6973	2.0988429	12.84386473	139842.78	113090.945	254193.61	237360.11	14.95759724	4.843044215	3	High	1344	Unique	Used	89243	1	21.403	1	4.739	-2.7126	1	7	19/36	20.12052	1344	1653.91	MS2	551.976	254	Glioma_grade 2_7rep.raw
29550838			lQQDVLQFQk	Unambiguous	N-Term(iTRAQ4plex); K10(iTRAQ4plex)	HCD	3.147295	0	1	1	2.91505	3.2267	3.0756	3.4043612	6.627398253	72929.48	65886.41	212593.4	224301.11	7.175262943	3.7897483	2	High	4270	Unique	Used	59821	1	43.615	1	4.352	-2.6786	1	67	16/18	18.2223	4270	1534.88	MS2	767.943	245	Glioma_grade 2_3.raw
29568111	NP_006088.2	MYL9	myosin regulatory light polypeptide 9 isoform a [Homo sapiens]	3	16	2.205278397	15.62895279	128.71813	32.56	4.06E-04	172	19.814	4.9194	Unclassified	Muscle contraction	Molecular function unknown	-	-																								
29568111			aTSNVFAMFDQSQIQEFk	Unambiguous	N-Term(iTRAQ4plex); K18(iTRAQ4plex)	HCD	2.6684217	0	1	4	2.53137	2.9127	2.4561	2.8261178	8.274430194	55419.246	48163.824	140286.69	136116.62	9.905784983	2.133610321	2	High	7647	Unique	Used	54951	1	68.303	1	6.2938	0.5607	1	14	23/34	3.179607	7647	2379.19	MS2	1190.1	151	Glioma_grade 2_2.raw
29568111			dGFIDkEDLHDmLASLGk	Unambiguous	N-Term(iTRAQ4plex); K6(iTRAQ4plex); M12(Oxidation); K18(iTRAQ4plex)	HCD	2.0511756	1	1	3	2.02907	2.1662	1.9434	2.074773	4.521822113	46967.54	43994.43	95300.5	91278.46	4.62238503	3.048587856	3	High	3933	Unique	Used	86651	1	53.378	1	6.6017	1.1202	1	10	25/68	14.26948	3933	2452.28	MS2	818.098	112	Glioma_grade 2_1rep.raw
29568111			dGFIDkEDLHDMLASLGk	Unambiguous	N-Term(iTRAQ4plex); K6(iTRAQ4plex); K18(iTRAQ4plex)	HCD	1.7343111	1	1	3	1.82818	1.8912	1.5826	1.6372011	8.541577094	44850.312	43355.27	81994.625	70981.3	2.397023663	10.18146717	3	High	4921	Unique	Used	87344	1	64.61	1	7.0969	-1.4607	1	17	31/68	23.99649	4921	2436.28	MS2	812.764	115	Glioma_grade 2_1rep.raw
29568111			dGFIDkEDLHDMLASLGk	Unambiguous	N-Term(iTRAQ4plex); K6(iTRAQ4plex); K18(iTRAQ4plex)	HCD	1.7262094	1	1	3	1.42721	1.856	1.6264	2.1150377	16.88561127	42644.113	32792.06	60862.195	69356.44	18.46979561	9.225005685	3	High	5660	Unique	Used	92700	1	62.595	1	6.1255	-0.1078	1	16	29/68	22.13948	5660	2436.28	MS2	812.765	120	Glioma_grade 2_7rep.raw
29568111			dGFIDkEDLHDmLASLGk	Unambiguous	N-Term(iTRAQ4plex); K6(iTRAQ4plex); M12(Oxidation); K18(iTRAQ4plex)	HCD	2.4621496	1	1	3	1.9278	2.9257	2.1567	3.2730925	24.64287805	25613.12	16877.127	49376.953	55240.395	29.07622491	7.92617989	4	High	3929	Unique	Used	86649	1	53.322	1	5.3442	1.6125	1	6	21/102	12.93482	3929	2452.28	MS2	613.825	132	Glioma_grade 2_1rep.raw
29568111			dGFIDkEDLHDmLASLGk	Unambiguous	N-Term(iTRAQ4plex); K6(iTRAQ4plex); M12(Oxidation); K18(iTRAQ4plex)	HCD	2.2061694	1	1	3	2.25779	2.3339	2.0826	2.1528077	5.039694628	23207.535	22450.486	52397.75	48331.582	2.344886922	5.708793872	3	High	4008	Unique	Used	96084	1	55.189	1	5.9123	1.5682	1	17	26/68	8.300601	4008	2452.28	MS2	818.098	111	Glioma_grade 2_8rep.raw

29568111			dGFIDkEDLHDmLASLGk	Unambiguous	N-Term(iTRAQ4plex); K6(iTRAQ4plex); M12(Oxidation); K18(iTRAQ4plex)	HCD	1.5703878	1	1	3	1.56389	1.6725	1.4749	1.5773379	5.144055627	22906.682	21419.152	35823.51	33785.242	4.745957178	4.141068711	3	High	4571	Unique	Used	91894	1	51.18	1	6.3354	-0.8958	1	45	24/68	25.28307	4571	2452.27	MS2	818.096	136	Glioma_grade 2_7rep.raw
29568111			dGFIDkEDLHDMLASLGk	Unambiguous	N-Term(iTRAQ4plex); K6(iTRAQ4plex); K18(iTRAQ4plex)	HCD	2.4501772	1	1	3	2.13293	2.578	2.3444	2.8336196	12.2073375	21260.645	17590.316	45347.477	49844.266	13.36036205	6.680642449	2	High	4952	Unique	Used	87367	1	64.947	1	6.5241	-0.1023	1	56	22/34	23.21869	4952	2436.28	MS2	1218.64	168	Glioma_grade 2_1rep.raw
29568111			aTSNVFAMFDQSQIQEFk	Unambiguous	N-Term(iTRAQ4plex); K18(iTRAQ4plex)	HCD	2.1855838	0	1	4	2.10242	3.0023	1.6137	2.3043501	25.53785271	18959.629	13276.774	39861.023	30594.338	24.93073006	18.60053148	3	High	7624	Unique	Used	54930	1	68.117	1	6.2429	1.1964	1	11	24/68	1.786423	7624	2379.19	MS2	793.736	146	Glioma_grade 2_2.raw
29568111			dGFIDkEDLHDMLASLGk	Unambiguous	N-Term(iTRAQ4plex); K6(iTRAQ4plex); K18(iTRAQ4plex)	HCD	2.5605643	1	1	3	2.40138	2.0968	3.0917	2.6995547	16.5131183	18706.969	21424.479	44922.598	57836.55	9.576378849	17.77271067	4	High	4858	Unique	Used	96568	1	66.638	1	5.3629	1.2395	1	4	28/102	5.320328	4858	2436.28	MS2	609.826	184	Glioma_grade 2_8rep.raw
29568111			aTSNVFAmFDQSQIQEFk	Unambiguous	N-Term(iTRAQ4plex); M8(Oxidation); K18(iTRAQ4plex)	HCD	2.2043872	0	1	4	1.87861	2.3605	2.0802	2.6137302	14.39776491	14742.568	11733.016	27695.504	30666.938	16.07575212	7.200250912	2	High	6736	Unique	Used	54116	1	61.131	1	5.1317	0.3356	1	42	19/34	0	6736	2395.19	MS2	1198.1	139	Glioma_grade 2_2.raw
29568111			eDLHDMLASLGk	Unambiguous	N-Term(iTRAQ4plex); K12(iTRAQ4plex)	HCD	2.2715282	0	1	3	2.26792	2.2375	2.306	2.275092	1.23655239	11977.446	12140.026	27163.844	27619.676	0.953345529	1.176711165	3	High	5474	Unique	Used	75847	1	54.67	1	5.088	-3.7318	1	37	21/44	4.517314	5474	1616.85	MS2	539.621	165	Glioma_grade 2_5.raw
29568111			dGFIDkEDLHDMLASLGk	Unambiguous	N-Term(iTRAQ4plex); K6(iTRAQ4plex); K18(iTRAQ4plex)	HCD	1.8313814	1	1	3	1.56314	2.1436	1.6037	2.1992388	18.14508928	10930.546	7970.5244	17085.982	17529.088	22.14743717	1.810328608	3	High	4837	Unique	Used	96557	1	66.339	1	7.3266	0.268	1	100	29/68	29.58556	4837	2436.28	MS2	812.765	123	Glioma_grade 2_8rep.raw
29568111			dGFIDkEDLHDMLASLGk	Unambiguous	N-Term(iTRAQ4plex); K6(iTRAQ4plex); K18(iTRAQ4plex)	HCD	2.3048682	1	1	3	1.98599	2.419	2.2112	2.6932693	12.94533879	10820.446	8883.601	21489.305	23925.928	13.90126844	7.587553923	2	High	4854	Unique	Used	96566	1	66.578	1	5.7961	-0.4029	1	100	21/34	23.90792	4854	2436.28	MS2	1218.64	174	Glioma_grade 2_8rep.raw
29568111			dGFIDkEDLHDmLASLGk	Unambiguous	N-Term(iTRAQ4plex); K6(iTRAQ4plex); M12(Oxidation); K18(iTRAQ4plex)	HCD	2.2186027	1	1	3	1.82206	2.6481	1.9231	2.7949336	21.57232785	9210.913	6337.5845	16782.812	17713.127	26.13435886	3.813968045	4	High	3992	Unique	Used	96074	1	54.985	1	3.717	0.2187	1	21	23/102	9.661485	3992	2452.28	MS2	613.824	153	Glioma_grade 2_8rep.raw
29568111			dGFIDkEDLHDMLASLGk	Unambiguous	N-Term(iTRAQ4plex); K6(iTRAQ4plex); K18(iTRAQ4plex)	HCD	1.7052674	1	1	3	1.39219	1.6972	1.7119	2.0869398	16.51587693	5648.759	4633.534	7864.1514	9669.907	13.96327613	14.56436383	4	High	4908	Unique	Used	87333	1	64.49	1	5.3141	0.037	1	38	25/102	36.1903	4908	2436.28	MS2	609.826	171	Glioma_grade 2_1rep.raw
29725609	NP_005219.2	EGFR	epidermal growth factor receptor isoform a precursor [Homo sapiens]	2	4	2.035327435	16.66774717	35.82426	5.12	9.47E-05	1210	134.19	6.6831	Receptor tyrosine kinase	Cell communication,Signal transduction	Transmembrane receptor protein tyrosine kinase activity	Cytoplasm, Nucleus, Plasma membrane	Cytoplasm, Endosome, Nucleus, Clathrin-coated vesicle																								
29725609			nLQEILHGAVR	Unambiguous	N-Term(iTRAQ4plex)	HCD	2.1168177	0	1	4	2.10955	2.0412	2.195	2.1238506	2.977511707	24885.975	25719.193	52498.184	54623.723	2.328513554	2.806117036	3	High	4048	Unique	Used	67287	1	41.276	1	3.8347	-0.4433	1	24	26/40	2.143426	4048	1393.8	MS2	465.272	220	Glioma_grade 2_4.raw
29725609			lTQLGTFEDHFLSLQR	Unambiguous	N-Term(iTRAQ4plex)	HCD	1.9538373	0	1	4	1.94389	1.8719	2.039	1.9634175	3.514582309	16640.062	17280.363	32346.424	33928.57	2.669548976	3.37607323	3	High	6967	Unique	Used	69964	1	64.557	1	6.1641	0.6118	1	27	25/60	72.81283	6967	2049.09	MS2	683.702	167	Glioma_grade 2_4.raw
29725609			nLQEILHGAVR	Unambiguous	N-Term(iTRAQ4plex)	HCD	1.5483729	0	1	4	1.47051	1.4515	1.6465	1.6252276	6.561426903	12323.517	12484.578	18121.9	20290.28	0.918146478	7.983281356	3	High	3371	Unique	Used	73983	1	37.25	1	4.0311	-0.5746	1	45	24/40	0	3371	1393.8	MS2	465.272	194	Glioma_grade 2_5.raw
29725609			lTQLGTFEDHFLSLQR	Unambiguous	N-Term(iTRAQ4plex)	HCD	2.3318987	0	1	4	2.45842	2.3143	2.3506	2.2127907	4.343718541	6244.527	6633.388	15351.692	14678.3	4.270353548	3.171229946	3	High	6546	Unique	Used	76795	1	63.647	1	5.4379	-4.1243	1	57	28/60	20.56154	6546	2049.08	MS2	683.698	147	Glioma_grade 2_5.raw
29826291	NP_816929.1	CASC4	protein CASC4 isoform b [Homo sapiens]	3	5	2.326057673	34.35991109	28.294003	10.26	1.47E-04	380	43.306	6.6538	Unclassified	Biological_process unknown	Molecular function unknown	-	-																								
29826291			lADQFLEEQk	Unambiguous	N-Term(iTRAQ4plex); K10(iTRAQ4plex)	HCD	2.1993363	0	1	2	1.96531	2.3601	2.0654	2.4803734	10.92619349	90689.516	75518.7	178233.2	187314.56	12.90837135	3.513352804	2	High	4168	Unique	Used	51701	1	42.322	1	4.4449	-0.684	1	49	17/18	13.32732	4168	1508.82	MS2	754.913	267	Glioma_grade 2_2.raw
29826291			nSDLLLLVDTHk	Unambiguous	N-Term(iTRAQ4plex); K12(iTRAQ4plex)	HCD	2.3260577	0	1	2	2.17776	2.2302	2.4196	2.4779284	6.229220159	24425.035	23850.557	53191.844	59099.97	1.682909614	7.440748902	3	High	4424	Unique	Used	74916	1	45.997	1	5.3427	-0.1975	1	22	23/44	44.93069	4424	1655.96	MS2	552.657	215	Glioma_grade 2_5.raw
29826291			hVLLQEEVAELQGQVQR	Unambiguous	N-Term(iTRAQ4plex)	HCD	2.7127028	0	1	2	2.50097	2.9288	2.5282	2.9606485	9.119418089	14572.952	12444.271	36446.582	36843.113	11.14255725	0.765154662	2	High	5746	Unique	Used	68851	1	54.623	1	5.8745	-0.5114	1	100	22/32	29.13633	5746	2120.16	MS2	1060.58	159	Glioma_grade 2_4.raw
29826291			hVLLQEEVAELQGQVQR	Unambiguous	N-Term(iTRAQ4plex)	HCD	4.0418553	0	1	2	3.86189	3.7465	4.3463	4.2164426	7.03729487	7849.9624	8091.7944	30315.688	34118.586	2.145322492	8.346660238	3	High	5752	Unique	Used	68856	1	54.679	1	5.9217	0.3171	1	17	27/64	36.01066	5752	2120.16	MS2	707.391	136	Glioma_grade 2_4.raw
29826291			hVLLQEEVAELQGQVQR	Unambiguous	N-Term(iTRAQ4plex)	HCD	1.6802552	0	1	2	1.36821	1.7529	1.6235	2.0800383	17.35403382	6960.3105	5432.7046	9523.194	11300.233	17.43208545	12.06867945	3	High	5277	Unique	Used	75668	1	53.12	1	6.7102	-0.7193	1	57	22/64	59.46495	5277	2120.16	MS2	707.39	199	Glioma_grade 2_5.raw
30089972	NP_004026.2	ACOX1	peroxisomal acyl-coenzyme A oxidase 1 isoform a [Homo sapiens]	7	11	2.019799709	16.25120278	124.08102	27.12	5.76E-04	660	74.62	7.6206	Enzyme: Oxidase	Metabolism,Energy pathways	Catalytic activity	Nucleus, Peroxisome	Mitochondrion																								
30089972			yDGNVYENLFEWAk	Unambiguous	N-Term(iTRAQ4plex); K14(iTRAQ4plex)	HCD	2.6277635	0	1	3	2.41333	2.8067	2.4739	2.8771448	8.818830837	39820.887	34240.043	96101.05	98513.555	10.65677311	1.753104445	2	High	7889	Unique	Used	55174	1	70.212	1	6.3658	0.4229	1	59	22/26	3.248147	7889	2036	MS2	1018.5	116	Glioma_grade 2_2.raw
30089972			dSASFNPELLTHILDGSPEk	Unambiguous	N-Term(iTRAQ4plex); K20(iTRAQ4plex)	HCD	1.954999	0	1	2	1.92948	2.0242	1.8891	1.9817713	3.023378647	20731.264	19761.531	40000.535	39162.836	3.386798961	1.496506872	3	High	6622	Unique	Used	76863	1	64.275	1	6.8929	-1.9848	1	13	37/76	3.826514	6622	2458.27	MS2	820.093	161	Glioma_grade 2_5.raw
30089972			eFGIADPDEIMWFk	Unambiguous	N-Term(iTRAQ4plex); K14(iTRAQ4plex)	HCD	2.0197997	0	1	3	1.87978	2.0664	1.9774	2.173723	6.195062527	20712.146	18841.775	38934.2	40956.8	6.687337103	3.580363685	2	High	8052	Unique	Used	55322	1	71.549	1	5.3466	-0.9644	1	100	18/26	43.84676	8052	1985.99	MS2	993.499	147	Glioma_grade 2_2.raw
30089972			eIENMILNDPDFQHEDLNFLTR	Unambiguous	N-Term(iTRAQ4plex)	HCD	2.3673663	0	1	2	1.95282	2.6412	2.1649	2.9280427	18.31791275	11256.616	8322.705	21982.19	24369.234	21.19162726	7.283034063	3	High	7931	Unique	Used	55212	1	70.563	1	6.7653	-3.5498	1	27	29/84	24.27241	7931	2847.36	MS2	949.793	68	Glioma_grade 2_2.raw
30089972			eIGTHkPLPGITVGDIGPk	Unambiguous	N-Term(iTRAQ4plex); K6(iTRAQ4plex); K19(iTRAQ4plex)	HCD	1.5941374	0	1	3	1.50008	1.626	1.5647	1.6960902	5.251861865	7422.0356	6847.153	11133.659	11613.389	5.697638405	2.982543811	3	High	3252	Unique	Used	86251	1	44.433	1	6.2383	3.3392	1	100	30/72	32.09502	3252	2361.39	MS2	787.803	29	Glioma_grade 2_1rep.raw
30089972			cYGLHAFIVPIR	Unambiguous	N-Term(iTRAQ4plex); C1(Methylthio)	HCD	2.428841	0	1	3	2.05991	2.5308	2.3459	2.8821049	14.03689436	6827.515	5557.241	14064.054	16016.553	14.50524111	9.179504145	3	High	5646	Unique	Used	76002	1	56.068	1	4.036	-2.2973	1	35	16/44	19.41564	5646	1578.84	MS2	526.951	171	Glioma_grade 2_5.raw
30089972			eIGTHkPLPGITVGDIGPk	Unambiguous	N-Term(iTRAQ4plex); K6(iTRAQ4plex); K19(iTRAQ4plex)	HCD	2.018348	0	1	3	1.73149	2.0113	2.0244	2.3515592	12.49291903	5990.8022	5157.418	10373.015	12127.975	10.57194079	11.03012905	3	High	3359	Unique	Used	95709	1	46.507	1	6.1952	0.3926	1	100	32/72	18.82654	3359	2361.39	MS2	787.801	32	Glioma_grade 2_8rep.raw
30089972			eIGTHkPLPGITVGDIGPk	Unambiguous	N-Term(iTRAQ4plex); K6(iTRAQ4plex); K19(iTRAQ4plex)	HCD	1.6702363	0	1	3	1.72042	1.9509	1.4227	1.6133267	13.13963786	5776.604	5094.0933	9938.197	8218.437	8.879061405	13.39514756	4	High	3251	Unique	Used	86250	1	44.429	1	6.7367	-1.2673	1	46	25/108	39.38961	3251	2361.38	MS2	591.101	48	Glioma_grade 2_1rep.raw
30089972			iNEGIGQGDLSELPELHALTAGLk	Unambiguous	N-Term(iTRAQ4plex); K24(iTRAQ4plex)	HCD	2.0834122	0	1	3	1.60149	2.3209	1.9195	2.7818186	23.68497846	5400.3384	3726.3853	8648.598	10366.128	25.9384118	12.774069	3	High	6825	Unique	Used	77034	1	66.129	1	8.8763	-0.4564	1	73	36/92	41.99662	6825	2763.51	MS2	921.842	75	Glioma_grade 2_5.raw
30089972			eIGTHkPLPGITVGDIGPk	Unambiguous	N-Term(iTRAQ4plex); K6(iTRAQ4plex); K19(iTRAQ4plex)	HCD	1.8307893	0	1	3	1.90649	1.8837	1.7772	1.7559891	4.111214393	2494.7732	2524.9404	4756.2705	4433.7676	0.849906325	4.962851842	4	High	3339	Unique	Used	95699	1	46.219	1	6.0047	-1.8876	1	100	22/108	37.43219	3339	2361.38	MS2	591.101	54	Glioma_grade 2_8rep.raw
30089972			dSASFNPELLTHILDGSPEk	Unambiguous	N-Term(iTRAQ4plex); K20(iTRAQ4plex)	HCD	2.498041	0	1	2	2.16445	2.4993	2.4969	2.8832355	11.70132349	1085.151	939.76526	2348.7593	2709.5645	10.15382657	10.08744452	4	High	7019	Unique	Used	70012	1	64.966	1	5.3557	-0.8233	1	73	28/114	18.78924	7019	2458.27	MS2	615.323	208	Glioma_grade 2_4.raw
30181236	NP_689940.3	CPNE2	copine-2 [Homo sapiens]	2	4	2.142506123	29.18631543	26.224628	8.76	6.25E-05	548	61.151	6.0747	Transport/cargo protein	Transport	Transporter activity	Cytoplasm	-																								
30181236			fALFDQDk	Unambiguous	N-Term(iTRAQ4plex); K8(iTRAQ4plex)	HCD	1.6837895	0	1	1	1.63929	1.7546	1.6176	1.7314177	3.998839365	55195.39	51567.176	90481.375	89284.32	4.80605669	0.941721064	2	High	4273	Unique	Used	59824	1	43.635	1	3.4468	-2.8009	1	100	13/14	23.03419	4273	1271.68	MS2	636.346	233	Glioma_grade 2_3.raw
30181236			aVLAELPQQVVQYFk	Unambiguous	N-Term(iTRAQ4plex); K15(iTRAQ4plex)	HCD	3.0779212	0	1	1	3.198	2.8666	3.3136	2.9702847	6.635306035	9398.227	10484.655	30055.531	31142.412	7.727457278	2.511656071	2	High	8312	Unique	Used	55554	1	73.758	1	5.5638	-3.4531	1	100	17/28	18.6333	8312	2021.16	MS2	1011.08	155	Glioma_grade 2_2.raw
30181236			aVLAELPQQVVQYFk	Unambiguous	N-Term(iTRAQ4plex); K15(iTRAQ4plex)	HCD	2.515728	0	1	1	2.27961	2.7104	2.352	2.7964666	10.13192899	3701.6467	3113.3013	8438.3125	8706.242	12.20913269	2.210086781	3	High	8306	Unique	Used	55548	1	73.716	1	5.2388	-1.2855	1	47	23/56	41.68341	8306	2021.16	MS2	674.393	122	Glioma_grade 2_2.raw
30181236			aVLAELPQQVVQYFk	Unambiguous	N-Term(iTRAQ4plex); K15(iTRAQ4plex)	HCD	1.7692842	0	1	1	2.05422	1.4482	2.2247	1.5684065	20.51840501	1926.322	2732.4324	3957.0964	4285.5645	24.47032324	5.635607815	3	High	8008	Unique	Used	63257	1	73.557	1	3.8917	2.3382	1	100	18/56	35.6287	8008	2021.17	MS2	674.396	110	Glioma_grade 2_3.raw
30425563	NP_848665.1	RTN4RL2	reticulon-4 receptor-like 2 precursor [Homo sapiens]	3	3	0.454012841	23.44015918	11.047648	8.1	1.50E-04	420	46.077	7.6206	Cell surface receptor	Cell communication,Signal transduction	Receptor activity	Plasma membrane	-																								
30425563			lLTEHVFR	Unambiguous	N-Term(iTRAQ4plex)	HCD	0.44876358	0	1	1	0.42065	0.4344	0.4627	0.47779468	0.058019395	46375.715	44911.156	19507.91	21458.312	2.268890562	6.733071359	3	High	2316	Unique	Used	73002	1	29.365	1	3.9771	0.299	1	66	17/28	48.99658	2316	1158.68	MS2	386.897	218	Glioma_grade 2_5.raw
30425563			lQSLHLYR	Unambiguous	N-Term(iTRAQ4plex)	HCD	0.45401284	0	1	1	0.42731	0.4707	0.4388	0.4834268	0.057899436	11808.219	10719.302	5045.7856	5181.998	6.835910572	1.883432726	3	High	1963	Unique	Used	72673	1	26.767	1	3.286	6.7821	1	94	16/28	55.84997	1963	1173.69	MS2	391.903	202	Glioma_grade 2_5.raw
30425563			eADFQAcPPAAPTRPGSR	Unambiguous	N-Term(iTRAQ4plex); C7(Methylthio)	HCD	0.6632762	0	1	1	0.68836	0.6684	0.658	0.63891566	0.031103484	11578.026	11923.363	7969.888	7618.024	2.078091082	3.192286696	3	High	2448	Unique	Used	65800	1	28.988	1	3.7846	-2.1288	1	100	21/68	32.07164	2448	2060.97	MS2	687.662	58	Glioma_grade 2_4.raw
30795212	NP_006538.2	IGF2BP3	insulin-like growth factor 2 mRNA-binding protein 3 [Homo sapiens]	2	2	4.567387581	16.12142724	20.32199	7.77	1.49E-04	579	63.666	8.8657	Translation regulatory protein	Protein metabolism	Translation regulator activity	Cytoplasm	Nucleus																								
30795212			mVIITGPPEAQFk	Unambiguous	N-Term(iTRAQ4plex); K13(iTRAQ4plex)	HCD	5.0880504	0	1	5	5.02845	5.4082	4.7904	5.1521506	5.049629906	13806.658	12837.235	69426.1	66139.37	5.145536181	3.428703594	2	High	5209	Unique	Used	60692	1	50.963	1	4.6065	-0.3655	1	100	17/24	32.24855	5209	1718.97	MS2	859.991	238	Glioma_grade 2_3.raw
30795212			lLVPTQFVGAIIGk	Unambiguous	N-Term(iTRAQ4plex); K14(iTRAQ4plex)	HCD	4.0467253	0	1	1	3.48399	4.7282	3.5446	4.8104367	17.52402699	877.7887	646.7958	3058.204	3111.37	21.42703746	1.21869157	3	High	8018	Unique	Used	70874	1	73.976	1	4.606	-0.7693	1	100	23/52	0	8018	1744.1	MS2	582.037	30	Glioma_grade 2_4.raw
30795231	NP_006308.3	BASP1	brain acid soluble protein 1 [Homo sapiens]	3	4	0.399901271	39.14178197	82.772956	51.1	8.40E-05	227	22.68	4.6274	Transcription regulatory protein	Regulation of nucleobase, nucleoside, nucleotide and nucleic acid metabolism	Transcription regulator activity	Nucleus	Cytoplasm, Plasma membrane																								
30795231			aEPPkAPEQEQAAPGPAAGGEAPk	Unambiguous	N-Term(iTRAQ4plex); K5(iTRAQ4plex); K24(iTRAQ4plex)	HCD	0.4721419	1	1	1	0.4502	0.4846	0.4605	0.49576586	0.044510584	172332.61	160088.52	77583.516	79366.42	5.208982394	1.606504011	2	High	1077	Unique	Used	78592	1	19.744	1	6.7507	-3.3867	1	24	24/46	0	1077	2730.43	MS2	1365.72	110	Glioma_grade 2_6.raw
30795231			aQGPAASAEEPkPVEAPAANSDQTVTVkE	Unambiguous	N-Term(iTRAQ4plex); K12(iTRAQ4plex); K28(iTRAQ4plex)	HCD	0.32766068	1	1	1	0.33543	0.2748	0.3921	0.32129228	0.145990084	26317.37	32118.525	8827.711	10319.434	14.03943942	11.0179084	3	High	1961	Unique	Used	72671	1	26.754	1	6.1969	9.5486	1	13	34/112	10.56995	1961	3324.76	MS2	1108.92	37	Glioma_grade 2_5.raw
30795231			aQGPAASAEEPkPVEAPAANSDQTVTVkE	Unambiguous	N-Term(iTRAQ4plex); K12(iTRAQ4plex); K28(iTRAQ4plex)	HCD	0.24234651	1	1	1	0.26842	0.3061	0.1864	0.21261483	0.221954761	25172.086	22070.834	6756.57	4692.5864	9.283576542	25.49457355	3	High	2322	Unique	Used	58005	1	28.851	1	4.058	-5.7619	1	12	20/112	1.55043	2322	3324.71	MS2	1108.91	69	Glioma_grade 2_3.raw
30795231			kTEAPAAPAAQETk	Unambiguous	N-Term(iTRAQ4plex); K1(iTRAQ4plex); K14(iTRAQ4plex)	HCD	0.6089927	1	1	1	0.5249	0.5287	0.6887	0.6937036	0.155878676	3565.751	3539.8303	1871.6539	2455.593	0.515895941	19.08406462	3	High	874	Unique	Used	78437	1	17.623	1	6.0337	1.1103	1	100	28/52	22.11876	874	1845.05	MS2	615.687	149	Glioma_grade 2_6.raw
32171186	NP_005736.3	BCAP31	B-cell receptor-associated protein 31 isoform b [Homo sapiens]	3	8	2.049546242	12.08408787	62.268689	18.29	2.38E-04	246	27.974	8.4409	Transport/cargo protein	Transport	Transporter activity	Endoplasmic reticulum	Mitochondrion, Golgi apparatus																								
32171186			lkDELASTk	Unambiguous	N-Term(iTRAQ4plex); K2(iTRAQ4plex); K9(iTRAQ4plex)	HCD	1.6857711	1	1	2	1.44728	1.7401	1.6406	1.9725133	12.85396063	2432939	2023520.4	3521149.5	3991421	12.99249662	8.852686751	3	High	1382	Unique	Used	89278	1	21.685	1	3.386	-0.8829	1	0	19/32	9.196602	1382	1436.87	MS2	479.627	136	Glioma_grade 2_7rep.raw

32171186			lkDELASTk	Unambiguous	N-Term(iTRAQ4plex); K2(iTRAQ4plex); K9(iTRAQ4plex)	HCD	2.045427	1	1	2	1.94767	2.0041	2.0856	2.1460218	4.279236182	1976018.2	1920353.6	3848623.8	4121121	2.020382969	4.835402458	2	High	1371	Unique	Used	89268	1	21.603	1	3.9238	-2.1479	1	2	15/16	6.910071	1371	1436.87	MS2	718.936	162	Glioma_grade 2_7rep.raw
32171186			lQAAVDGPMDk	Unambiguous	N-Term(iTRAQ4plex); K11(iTRAQ4plex)	HCD	2.0536656	0	1	2	1.90535	2.1159	1.9976	2.2183728	6.635227215	489730.03	440998.66	933106	978299.44	7.404581497	3.343779108	2	High	2200	Unique	Used	49847	1	28.187	1	4.1883	-0.9132	1	5	18/20	11.93346	2200	1432.77	MS2	716.888	220	Glioma_grade 2_2.raw
32171186			lQAAVDGPMDk	Unambiguous	N-Term(iTRAQ4plex); K11(iTRAQ4plex)	HCD	2.3232276	0	1	2	2.46026	2.1073	2.5753	2.2058496	9.30621264	162075.5	189219.88	398748.53	417390.6	10.92754204	3.230315427	2	High	2171	Unique	Used	57865	1	27.708	1	4.3511	12.037	1	18	18/20	17.26593	2171	1432.79	MS2	716.898	262	Glioma_grade 2_3.raw
32171186			lQAAVDGPmDk	Unambiguous	N-Term(iTRAQ4plex); M9(Oxidation); K11(iTRAQ4plex)	HCD	2.2723255	0	1	2	2.31278	2.2462	2.2992	2.2330406	1.720173902	132229.23	136148.86	305816.5	304025.94	2.065442043	0.415227618	2	High	1403	Unique	Used	57145	1	22.172	1	3.9671	-0.3423	1	34	16/20	16.04763	1403	1448.77	MS2	724.886	179	Glioma_grade 2_3.raw
32171186			vNLQNNPGAMEHFHmk	Unambiguous	N-Term(iTRAQ4plex); M15(Oxidation); K16(iTRAQ4plex)	HCD	2.1306808	0	1	2	1.90922	2.6241	1.7717	2.4350564	18.72518626	60392.79	43940.34	115303.375	106997.21	22.30094882	5.284147675	4	High	1810	Unique	Used	89660	1	25.081	1	5.0553	-2.9309	1	4	20/90	8.198102	1810	2171.07	MS2	543.522	125	Glioma_grade 2_7rep.raw
32171186			vNLQNNPGAMEHFHMk	Unambiguous	N-Term(iTRAQ4plex); K16(iTRAQ4plex)	HCD	1.6558778	0	1	2	1.60118	1.6958	1.6182	1.713813	3.370197411	49718.594	46943.883	79608.23	80453.04	4.059521388	0.746427765	3	High	2706	Unique	Used	90431	1	32.801	1	6.9431	1.2363	1	33	31/60	36.36092	2706	2155.08	MS2	719.032	122	Glioma_grade 2_7rep.raw
32171186			vNLQNNPGAMEHFHMk	Unambiguous	N-Term(iTRAQ4plex); K16(iTRAQ4plex)	HCD	1.9733012	0	1	2	1.7554	1.9229	2.0193	2.2120023	9.641610049	35726.203	32613.676	62713.637	72141.52	6.441009204	9.886933729	2	High	2767	Unique	Used	90482	1	33.347	1	5.387	7.2183	1	63	18/30	0	2767	2155.09	MS2	1078.05	161	Glioma_grade 2_7rep.raw
33457308	NP_872353.2	TMED4	transmembrane emp24 domain-containing protein 4 precursor [Homo sapiens]	3	4	2.172850609	3.074467543	24.059198	23.79	1.49E-04	227	25.926	8.2798	Integral membrane protein	Biological_process unknown	Molecular function unknown	-	-																								
33457308			qLLDQVEQIQk	Unambiguous	N-Term(iTRAQ4plex); K11(iTRAQ4plex)	HCD	2.1757631	0	1	1	2.12409	2.1901	2.1619	2.2290456	2.038939178	153751.31	149117.6	326581.06	332389.9	2.163660683	1.246631613	2	High	4651	Unique	Used	52154	1	45.914	1	5.5999	-1.0383	1	25	19/20	16.65366	4651	1629.94	MS2	815.474	188	Glioma_grade 2_2.raw
33457308			dkLTELQLR	Unambiguous	N-Term(iTRAQ4plex); K2(iTRAQ4plex)	HCD	2.1699378	1	1	1	2.04172	2.1671	2.1726	2.3060348	4.970607842	47103.656	44377.613	96172.35	102336.32	4.214203655	4.391329594	2	High	2170	Unique	Used	79604	1	27.907	1	3.0654	2.4629	1	54	14/16	8.618976	2170	1403.85	MS2	702.428	213	Glioma_grade 2_6.raw
33457308			dkLTELQLR	Unambiguous	N-Term(iTRAQ4plex); K2(iTRAQ4plex)	HCD	2.044767	1	1	1	1.93397	2.2263	1.887	2.1723151	8.238111819	46690.7	40559.285	90298.33	88107.55	9.938259874	1.736619212	3	High	2158	Unique	Used	79593	1	27.813	1	3.9696	1.236	1	31	16/32	41.44216	2158	1403.85	MS2	468.621	186	Glioma_grade 2_6.raw
33457308			eVFLPSTPGLGMHVEVk	Unambiguous	N-Term(iTRAQ4plex); K17(iTRAQ4plex)	HCD	2.182724	0	1	1	2.04007	2.443	1.9654	2.3535557	10.60007175	33569.926	28033.54	68484.875	65978.49	12.7097267	2.636081329	3	High	5127	Unique	Used	75537	1	51.845	1	6.6786	1.4272	1	13	31/64	24.9008	5127	2128.17	MS2	710.063	157	Glioma_grade 2_5.raw
33942072	NP_006577.2	CNPY3	protein canopy homolog 3 precursor [Homo sapiens]	3	3	2.237290621	14.28112773	18.637994	12.95	1.04E-04	278	30.729	5.4907	Unclassified	Biological_process unknown	Molecular function unknown	-	-																								
33942072			lIEVTETIck	Unambiguous	N-Term(iTRAQ4plex); C9(Methylthio); K10(iTRAQ4plex)	HCD	2.2372906	0	1	1	2.17951	2.2871	2.1898	2.2979283	2.795205705	91713.836	87398.664	199890.88	200835.86	3.407118301	0.333494973	2	High	6616	Unique	Used	54007	1	60.173	1	3.8642	-1.8444	1	28	16/18	11.85776	6616	1482.81	MS2	741.91	225	Glioma_grade 2_2.raw
33942072			lLDYSLHk	Unambiguous	N-Term(iTRAQ4plex); K8(iTRAQ4plex)	HCD	2.6720326	0	1	1	2.69561	2.5738	2.775	2.649525	3.152467327	35088.617	36749.914	94585.09	97369.81	3.270429832	2.051621913	3	High	2501	Unique	Used	79914	1	30.35	1	4.2979	5.4596	1	43	19/28	29.30051	2501	1276.76	MS2	426.257	285	Glioma_grade 2_6.raw
33942072			nHQEEDLTEFLcANHVLk	Unambiguous	N-Term(iTRAQ4plex); C12(Methylthio); K18(iTRAQ4plex)	HCD	2.024436	0	1	1	2.17928	1.7104	2.4246	1.9029056	15.23675257	7118.857	9070.451	15514.006	17260.213	17.04810794	7.53491524	4	High	5180	Unique	Used	92355	1	57.412	1	7.2464	0.4045	1	14	35/102	10.09974	5180	2474.21	MS2	619.307	138	Glioma_grade 2_7rep.raw
33946291	NP_079106.3	LPCAT1	lysophosphatidylcholine acyltransferase 1 [Homo sapiens]	2	4	2.43517828	20.01400224	18.630963	5.81	1.00E-04	534	59.113	6.0239	Enzyme: Acyltransferase	Cell organization and biogenesis	Acyltransferase activity	Endoplasmic reticulum	Golgi apparatus																								
33946291			iTFADFHR	Unambiguous	N-Term(iTRAQ4plex)	HCD	2.2497551	0	1	1	2.1224	2.334	2.1732	2.389802	5.649688359	55622.023	50580.035	118052.36	120876.26	6.714039205	1.671460572	2	High	2947	Unique	Used	73591	1	34.014	1	2.9687	0.214	1	55	13/14	22.25168	2947	1150.61	MS2	575.81	302	Glioma_grade 2_5.raw
33946291			iTFADFHR	Unambiguous	N-Term(iTRAQ4plex)	HCD	2.7634757	0	1	1	2.45348	2.8048	2.7273	3.11786	9.845781984	49026.105	42884.8	120284.74	133708.8	9.449495489	7.474397858	3	High	2987	Unique	Used	73626	1	34.364	1	4.2575	0.4676	1	19	19/28	3.053982	2987	1150.61	MS2	384.209	261	Glioma_grade 2_5.raw
33946291			iTFADFHR	Unambiguous	N-Term(iTRAQ4plex)	HCD	2.6206014	0	1	1	2.55744	2.6236	2.6176	2.685395	1.995227745	25453.371	24811.223	65095.53	66627.93	1.80670794	1.645220117	3	High	2911	Unique	Used	73560	1	33.702	1	4.2354	-0.0894	1	63	18/28	0	2911	1150.61	MS2	384.209	261	Glioma_grade 2_5.raw
33946291			tcLITFkPGAFIPGAPVQPVVLR	Unambiguous	N-Term(iTRAQ4plex); C2(Methylthio); K7(iTRAQ4plex)	HCD	1.7152771	0	1	1	1.51471	1.6422	1.7827	1.9327155	10.48578498	12548.529	11574.61	19007.438	22370.43	5.709578088	11.49404048	3	High	6307	Unique	Used	93134	1	70.088	1	7.1694	4.0661	1	45	33/88	32.26826	6307	2758.59	MS2	920.2	56	Glioma_grade 2_7rep.raw
34452173	NP_000878.2	ITGAX	integrin alpha-X precursor [Homo sapiens]	3	3	2.783153057	28.4493594	23.030178	5.5	1.58E-04	1163	127.75	6.6392	Cell surface receptor	Cell communication,Signal transduction	Receptor activity	Plasma membrane	-																								
34452173			tTFQLELPVk	Unambiguous	N-Term(iTRAQ4plex); K10(iTRAQ4plex)	HCD	1.8393314	0	1	1	1.80852	1.9691	1.7201	1.8728747	5.698787219	38557.176	35412.69	69731.57	66323.54	6.011873467	3.542448532	2	High	5720	Unique	Used	53162	1	53.642	1	4.0611	-1.3095	1	100	16/18	39.22968	5720	1463.87	MS2	732.438	233	Glioma_grade 2_2.raw
34452173			yFTASLPFEk	Unambiguous	N-Term(iTRAQ4plex); K10(iTRAQ4plex)	HCD	2.783153	0	1	1	2.56012	2.6946	2.8673	3.0178993	7.174187748	33340.637	31676.84	85356.07	95597.51	3.618971992	8.004032496	2	High	5933	Unique	Used	61361	1	56.654	1	3.6352	1.5791	1	100	15/18	17.08948	5933	1490.82	MS2	745.912	262	Glioma_grade 2_3.raw
34452173			iAPPASDFLAHIQk	Unambiguous	N-Term(iTRAQ4plex); K14(iTRAQ4plex)	HCD	3.3301435	0	1	1	3.05014	3.2337	3.4211	3.6269948	7.432016573	20881.469	19696.32	63691.402	71438.445	4.130470956	8.1077375	3	High	4774	Unique	Used	81877	1	50.101	1	6.5203	-1.0982	1	45	27/52	28.77918	4774	1796.03	MS2	599.348	175	Glioma_grade 2_6.raw
34740329	NP_919223.1	HNRNPA3	heterogeneous nuclear ribonucleoprotein A3 [Homo sapiens]	4	9	2.212531328	22.77215333	47.906469	16.14	2.96E-04	378	39.571	9.0122	Ribonucleoprotein	Regulation of nucleobase, nucleoside, nucleotide and nucleic acid metabolism	RNA binding	Nucleus	Cytoplasm																								
34740329			eDSVkPGAHLTVk	Unambiguous	N-Term(iTRAQ4plex); K5(iTRAQ4plex); K13(iTRAQ4plex)	HCD	2.3740902	0	1	1	2.03329	2.6024	2.1957	2.8102784	14.86345974	280970.9	219525.75	571295.7	616928.5	17.36206715	5.43117407	3	High	1075	Unique	Used	89002	1	19.311	1	4.5549	-1.5788	1	2	26/48	15.41814	1075	1813.05	MS2	605.022	86	Glioma_grade 2_7rep.raw
34740329			gFAFVTFDDHDTVDk	Unambiguous	N-Term(iTRAQ4plex); K15(iTRAQ4plex)	HCD	2.4431276	0	1	1	1.93469	2.4503	2.4375	3.0870764	19.0555171	72607.15	57328.26	140472.05	176976.72	16.62950342	16.26259236	3	High	5010	Unique	Used	75430	1	50.947	1	5.1377	-1.6098	1	4	31/56	12.26959	5010	2001.98	MS2	667.997	108	Glioma_grade 2_5.raw
34740329			gFAFVTFDDHDTVDk	Unambiguous	N-Term(iTRAQ4plex); K15(iTRAQ4plex)	HCD	2.2125313	0	1	1	1.99942	2.3518	2.0941	2.4632115	9.72854508	54301.656	46164.64	108571.555	113713.28	11.45406852	3.271252053	2	High	4937	Unique	Used	75366	1	50.336	1	6.2965	-1.2372	1	39	23/28	29.08042	4937	2001.98	MS2	1001.49	121	Glioma_grade 2_5.raw
34740329			gFAFVTFDDHDTVDk	Unambiguous	N-Term(iTRAQ4plex); K15(iTRAQ4plex)	HCD	2.5391066	0	1	1	2.87032	2.3909	2.717	2.2632189	10.99237068	48412.77	58120.543	138959.94	131539.52	12.88692133	3.879511849	3	High	4925	Unique	Used	75355	1	50.244	1	5.4178	1.1341	1	9	30/56	17.93269	4925	2001.98	MS2	667.999	119	Glioma_grade 2_5.raw
34740329			eDSVkPGAHLTVk	Unambiguous	N-Term(iTRAQ4plex); K5(iTRAQ4plex); K13(iTRAQ4plex)	HCD	3.066981	0	1	1	3.27611	2.8891	3.2687	2.882555	7.249463399	39640.78	44950.75	129867.625	129573.01	8.877285456	0.160594939	4	High	1049	Unique	Used	88979	1	19.097	1	3.4499	-2.8655	1	27	19/72	54.34488	1049	1813.05	MS2	454.018	122	Glioma_grade 2_7rep.raw
34740329			iFVGGIkEDTEEYNLR	Unambiguous	N-Term(iTRAQ4plex); K7(iTRAQ4plex)	HCD	1.5789592	1	1	1	1.45793	1.588	1.5707	1.710778	6.543278376	32923.258	30227.314	47999.953	51712.223	6.037380894	5.265096793	3	High	4500	Unique	Used	81637	1	47.792	1	4.3642	0.6177	1	39	25/60	13.22706	4500	2171.16	MS2	724.392	165	Glioma_grade 2_6.raw
34740329			gFAFVTFDDHDTVDk	Unambiguous	N-Term(iTRAQ4plex); K15(iTRAQ4plex)	HCD	1.8482393	0	1	1	1.75045	1.7881	1.9071	1.9481328	5.096744759	31009.2	30356.438	54280.01	59138.37	1.504335168	6.057888151	2	High	5025	Unique	Used	75444	1	51.052	1	5.6148	-2.1518	1	49	21/28	24.05863	5025	2001.98	MS2	1001.49	117	Glioma_grade 2_5.raw
34740329			kLFIGGLSFETTDDSLR	Unambiguous	N-Term(iTRAQ4plex); K1(iTRAQ4plex)	HCD	1.546027	1	1	1	1.41022	1.9181	1.2725	1.7307357	18.61234509	18444.44	13560.777	26010.807	23470.121	21.57942703	7.261530339	3	High	5480	Unique	Used	82476	1	56.376	1	7.627	-0.2515	1	27	22/64	30.90648	5480	2187.19	MS2	729.735	169	Glioma_grade 2_6.raw
34740329			gFAFVTFDDHDTVDk	Unambiguous	N-Term(iTRAQ4plex); K15(iTRAQ4plex)	HCD	1.9349204	0	1	1	2.00603	1.9123	1.9586	1.867137	3.087465393	12784.922	13411.424	25646.879	25040.965	3.382180191	1.690527213	3	High	5226	Unique	Used	75623	1	52.695	1	5.4437	-0.5122	1	41	27/56	37.77982	5226	2001.98	MS2	667.998	111	Glioma_grade 2_5.raw
38327552	NP_938405.1	G3BP1	ras GTPase-activating protein-binding protein 1 [Homo sapiens]	2	2	2.442709208	34.14607341	19.79834	7.51	4.09E-04	466	52.132	5.5161	RNA binding protein,Ribonuclease	Signal transduction,Regulation of nucleobase, nucleoside, nucleotide and nucleic acid metabolism	RNA binding,Ribonuclease activity	Cytosol	Cytoplasm, Nucleus, Plasma membrane																								
38327552			lPNFGFVVFDDSEPVQk	Unambiguous	N-Term(iTRAQ4plex); K17(iTRAQ4plex)	HCD	1.852919	0	1	1	1.80786	1.9686	1.7467	1.9019839	5.301905577	8725.939	8013.4697	15775.278	15241.49	6.019231413	2.433813314	2	High	7609	Unique	Used	62900	1	70.123	1	4.5068	0.9544	1	100	19/32	30.25868	7609	2226.17	MS2	1113.59	212	Glioma_grade 2_3.raw
38327552			hPDSHQLFIGNLPHEVDk	Unambiguous	N-Term(iTRAQ4plex); K18(iTRAQ4plex)	HCD	3.0324993	0	1	1	2.66606	3.3326	2.7924	3.4905596	13.10944953	4079.0654	3263.22	10875.013	11390.463	15.71417572	3.273931268	4	High	2936	Unique	Used	86064	1	40.283	1	5.1413	-0.6694	1	70	26/102	38.66102	2936	2371.24	MS2	593.565	125	Glioma_grade 2_1rep.raw
38372935	NP_068767.3	BCAN	brevican core protein isoform 1 precursor [Homo sapiens]	6	8	2.185297012	22.0811141	118.32907	19.87	2.90E-04	911	99.056	4.6401	Adhesion molecule	Cell communication,Signal transduction	Cell adhesion molecule activity	Extracellular	Plasma membrane																								
38372935			cGGGLPGVk	Unambiguous	N-Term(iTRAQ4plex); C1(Methylthio); K9(iTRAQ4plex)	HCD	2.958082	0	1	2	2.84204	2.9608	2.9555	3.0789747	3.269762293	348884.88	334892.8	991544.06	1031126.5	2.893892252	2.767530442	2	High	2286	Unique	Used	65649	1	27.783	1	3.0454	12.225	1	9	16/16	27.97017	2286	1121.62	MS2	561.313	224	Glioma_grade 2_4.raw
38372935			cEVQHGIDDSSDAVEVk	Unambiguous	N-Term(iTRAQ4plex); C1(Methylthio); K17(iTRAQ4plex)	HCD	2.344179	0	1	2	2.21369	2.4362	2.2605	2.487783	5.656992511	23442.988	21301.684	51895.555	52993.97	6.767869833	1.480980479	3	High	2954	Unique	Used	66273	1	32.76	1	5.9515	0.9323	1	10	28/64	9.502435	2954	2165.01	MS2	722.342	69	Glioma_grade 2_4.raw
38372935			rSWEEAETQcR	Unambiguous	N-Term(iTRAQ4plex); C10(Methylthio)	HCD	1.5728387	1	1	1	1.44318	1.5501	1.594	1.7120969	7.066544121	21480.41	19999.143	31000.174	34240.473	5.050266286	7.023956694	2	High	1661	Unique	Used	72402	1	24.387	1	3.5224	4.2369	1	100	15/20	47.97895	1661	1584.71	MS2	792.858	23	Glioma_grade 2_5.raw
38372935			cEVQHGIDDSSDAVEVk	Unambiguous	N-Term(iTRAQ4plex); C1(Methylthio); K17(iTRAQ4plex)	HCD	2.2662613	0	1	2	2.30886	2.3161	2.2166	2.2235255	2.362177997	15171.909	15124.374	35029.86	33629.434	0.221890724	2.884535111	2	High	2965	Unique	Used	66284	1	32.83	1	6.4286	1.7842	1	69	23/32	0	2965	2165.01	MS2	1083.01	75	Glioma_grade 2_4.raw
38372935			cEVQHGIDDSSDAVEVk	Unambiguous	N-Term(iTRAQ4plex); C1(Methylthio); K17(iTRAQ4plex)	HCD	1.8990664	0	1	2	1.67698	1.902	1.8964	2.1509535	10.15927398	12306.728	10850.509	20638.191	23338.94	8.893136343	8.68505011	3	High	2758	Unique	Used	58414	1	32.026	1	6.6425	-0.0826	1	25	27/64	0	2758	2165.01	MS2	722.341	58	Glioma_grade 2_3.raw
38372935			mYGAHLASISTPEEQDFINNR	Unambiguous	N-Term(iTRAQ4plex)	HCD	2.104333	0	1	1	1.90996	2.4863	1.8109	2.3573587	15.45193812	9250.12	7105.897	17667.355	16751.148	18.53990032	3.764580828	3	High	5297	Unique	Used	68440	1	51.077	1	7.8093	-0.3485	1	17	27/80	1.339177	5297	2537.22	MS2	846.412	114	Glioma_grade 2_4.raw
38372935			fcNPGWDAFQGAcYk	Unambiguous	N-Term(iTRAQ4plex); C2(Methylthio); C13(Methylthio); K15(iTRAQ4plex)	HCD	1.7253041	0	1	1	1.64712	1.8035	1.6539	1.8109093	5.23740865	6168.841	5634.047	10160.823	10202.748	6.407863295	0.291161622	3	High	7413	Unique	Used	70362	1	68.343	1	5.4145	1.1678	1	33	23/56	24.46071	7413	2086.89	MS2	696.302	30	Glioma_grade 2_4.raw
38372935			gAEIATTGQLYAAWDGGLDHcSPGWLADGSVR	Unambiguous	N-Term(iTRAQ4plex); C21(Methylthio)	HCD	2.7872329	0	1	2	2.44219	3.571	2.2512	3.2917469	22.19637519	1152.6926	788.33136	2815.0989	2594.9873	26.54705031	5.75378651	3	High	7956	Unique	Used	63211	1	73.096	1	9.2572	-1.054	1	76	43/124	13.65074	7956	3464.61	MS2	1155.54	36	Glioma_grade 2_3.raw
38454326	NP_001766.2	CD38	ADP-ribosyl cyclase 1 [Homo sapiens]	5	10	3.617897987	30.76934495	54.676964	16	2.65E-05	300	34.306	7.6646	Enzyme: Hydrolase	Metabolism,Energy pathways	Hydrolase activity	Plasma membrane	Nucleus																								
38454326			hVDcQSVWDAFk	Unambiguous	N-Term(iTRAQ4plex); C4(Methylthio); K12(iTRAQ4plex)	HCD	3.8263807	0	1	1	3.74722	3.9406	3.7178	3.9096227	2.939372492	86879.55	82616.945	325557.03	323001.1	3.556553663	0.557333368	2	High	5832	Unique	Used	82781	1	59.406	1	4.9338	-0.6514	1	24	18/22	30.37603	5832	1768.84	MS2	884.923	100	Glioma_grade 2_6.raw
38454326			dLAHQFTQVQR	Unambiguous	N-Term(iTRAQ4plex)	HCD	3.6776059	0	1	1	3.89377	3.701	3.653	3.472147	4.708497775	85738.77	90204.46	333846.72	313203.16	3.58947563	4.511924572	2	High	1783	Unique	Used	57504	1	24.836	1	4.1851	-0.7098	1	19	16/20	6.374756	1783	1486.79	MS2	743.897	245	Glioma_grade 2_3.raw
38454326			lGTQTVPcNk	Unambiguous	N-Term(iTRAQ4plex); C8(Methylthio); K10(iTRAQ4plex)	HCD	3.55819	0	1	1	3.54375	3.4828	3.6349	3.5723858	1.776395281	78557.77	79933.17	278388.78	285552.12	1.22726847	1.796374865	2	High	2157	Unique	Used	57852	1	27.6	1	3.6339	11.659	1	33	15/18	29.96109	2157	1394.75	MS2	697.881	274	Glioma_grade 2_3.raw
38454326			dLAHQFTQVQR	Unambiguous	N-Term(iTRAQ4plex)	HCD	3.697384	0	1	1	3.6641	3.8126	3.5866	3.7320154	2.605022402	61918.473	59506.105	226875.61	222077.69	2.80964826	1.5113562	2	High	1925	Unique	Used	65312	1	25.138	1	4.1571	0.9323	1	22	17/20	3.374942	1925	1486.79	MS2	743.899	226	Glioma_grade 2_4.raw

38454326			dLAHQFTQVQR	Unambiguous	N-Term(iTRAQ4plex)	HCD	3.8180757	0	1	1	3.47463	3.7259	3.9041	4.186361	7.839078081	51438.39	47969.73	178729.12	200818.6	4.934633122	8.230649417	3	High	1922	Unique	Used	65309	1	25.121	1	3.7725	0.5128	1	6	20/40	11.88208	1922	1486.79	MS2	496.268	231	Glioma_grade 2_4.raw
38454326			ikDLAHQFTQVQR	Unambiguous	N-Term(iTRAQ4plex); K2(iTRAQ4plex)	HCD	2.4888015	1	1	1	2.30332	2.7138	2.2978	2.7073412	9.449282467	44087.516	37418.355	101547.69	101304.25	11.57170376	0.169717948	2	High	3030	Unique	Used	90703	1	35.705	1	4.9065	-0.42	1	44	20/24	6.181312	3030	1872.07	MS2	936.538	174	Glioma_grade 2_7rep.raw
38454326			hVDcQSVWDAFk	Unambiguous	N-Term(iTRAQ4plex); C4(Methylthio); K12(iTRAQ4plex)	HCD	4.9071236	0	1	1	4.81244	4.74	5.0768	5.0003867	3.208255115	35145.53	35682.39	169135.6	178425.77	1.071942665	3.780133681	3	High	5819	Unique	Used	82769	1	59.307	1	4.0834	0.0657	1	8	22/44	10.85583	5819	1768.84	MS2	590.285	115	Glioma_grade 2_6.raw
38454326			vQTLEAWVIHGGR	Unambiguous	N-Term(iTRAQ4plex)	HCD	1.7303206	0	1	1	1.66249	1.6761	1.7841	1.7987033	4.100746631	26292.887	26079.154	43711.758	46908.66	0.577147848	4.989054637	2	High	6170	Unique	Used	69242	1	57.989	1	5.1088	2.3255	1	100	19/24	49.02546	6170	1609.9	MS2	805.452	272	Glioma_grade 2_4.raw
38454326			vQTLEAWVIHGGR	Unambiguous	N-Term(iTRAQ4plex)	HCD	1.708322	0	1	1	1.73443	1.608	1.8165	1.6841127	5.118742292	15767.605	17007.092	27347.877	28641.857	5.34833114	3.26839214	3	High	6150	Unique	Used	69224	1	57.824	1	5.5878	-0.7158	1	28	29/48	14.50157	6150	1609.89	MS2	537.302	170	Glioma_grade 2_4.raw
38454326			vQTLEAWVIHGGR	Unambiguous	N-Term(iTRAQ4plex)	HCD	3.3545022	0	1	1	3.14223	3.663	3.0898	3.6019607	8.890499953	11314.635	9705.914	35553.156	34960.32	10.82310008	1.18898792	3	High	5595	Unique	Used	75955	1	55.67	1	5.8576	-2.4219	1	40	30/48	6.897499	5595	1609.89	MS2	537.301	182	Glioma_grade 2_5.raw
38679907	NP_877429.2	TPRG1L	tumor protein p63-regulated gene 1-like protein [Homo sapiens]	2	3	0.359139472	28.1866623	19.312857	19.49	1.52E-03	272	30.193	7.3423	Unclassified	Biological_process unknown	Molecular function unknown	-	-																								
38679907			tASLcQLESFk	Unambiguous	N-Term(iTRAQ4plex); C5(Methylthio); K11(iTRAQ4plex)	HCD	0.5074133	0	1	1	0.48585	0.4951	0.5195	0.5293869	0.040133122	145061.62	142345.52	70478.375	75355.84	1.336482266	4.729875738	2	High	6075	Unique	Used	53497	1	56.236	1	4.7155	-1.555	1	41	18/20	31.33201	6075	1560.8	MS2	780.903	156	Glioma_grade 2_2.raw
38679907			eYFVFRPGSIEQAVEEIR	Unambiguous	N-Term(iTRAQ4plex)	HCD	0.29464042	0	1	1	0.30663	0.324	0.2669	0.28196904	0.086096393	29547.77	27967.984	9060.361	7886.1055	3.884422322	9.799376488	3	High	7370	Unique	Used	62683	1	68.146	1	4.7729	0.6708	1	30	26/68	10.06683	7370	2313.2	MS2	771.739	105	Glioma_grade 2_3.raw
38679907			eYFVFRPGSIEQAVEEIR	Unambiguous	N-Term(iTRAQ4plex)	HCD	0.35913947	0	1	1	0.30866	0.2786	0.4483	0.40471143	0.221363584	29193.395	32340.088	9010.699	13088.402	7.231990943	26.09492072	3	High	7366	Unique	Used	70319	1	67.96	1	4.122	-0.5165	1	41	28/68	21.28243	7366	2313.2	MS2	771.738	126	Glioma_grade 2_4.raw
39930375	NP_060682.2	ENAH	protein enabled homolog isoform b [Homo sapiens]	2	3	2.22640276	23.0772704	13.270177	3.86	3.32E-04	570	63.886	6.4302	Cytoskeletal protein	Cell growth and/or maintenance	Structural constituent of cytoskeleton	Cytoplasm, Nucleus	Plasma membrane																								
39930375			lkEELIDAIR	Unambiguous	N-Term(iTRAQ4plex); K2(iTRAQ4plex)	HCD	1.5097154	1	1	2	1.37976	1.572	1.4551	1.6577736	8.123618433	45690.234	40103.09	63041.656	66481.84	9.209824787	3.756194837	2	High	4783	Unique	Used	81885	1	50.177	1	3.5152	-1.6376	1	54	16/18	40.84767	4783	1487.9	MS2	744.454	166	Glioma_grade 2_6.raw
39930375			yNQATQTFHQWR	Unambiguous	N-Term(iTRAQ4plex)	HCD	2.2264028	0	1	2	2.43751	2.2973	2.1512	2.027444	7.977742379	17515.365	18584.635	42693.836	37679.31	4.188853562	8.823358337	3	High	2424	Unique	Used	65777	1	28.815	1	5.8352	-1.1377	1	26	25/44	56.88109	2424	1723.84	MS2	575.285	129	Glioma_grade 2_4.raw
39930375			lkEELIDAIR	Unambiguous	N-Term(iTRAQ4plex); K2(iTRAQ4plex)	HCD	2.4002435	1	1	2	2.45995	2.3587	2.4436	2.342999	2.457052712	16566.053	17277.293	40751.59	40480.68	2.972062083	0.471640884	3	High	4784	Unique	Used	81886	1	50.183	1	3.9199	-5.6257	1	39	20/36	15.54728	4784	1487.9	MS2	496.637	157	Glioma_grade 2_6.raw
40217847	NP_054733.2	SNRNP200	U5 small nuclear ribonucleoprotein 200 kDa helicase [Homo sapiens]	2	2	2.01581955	23.83101605	13.854486	1.59	4.50E-04	2136	244.35	6.062	Ribonucleop rotein	Regulation of nucleobase, nucleoside, nucleotide and nucleic acid metabolism	Ribonucleopr otein	Nucleus	Mitochondrion, Nucleolus																								
40217847			lIILDEIHLLHDDR	Unambiguous	N-Term(iTRAQ4plex)	HCD	1.6761324	0	1	1	1.64517	1.8136	1.5514	1.7102628	6.564976406	9708.534	8807.004	15972.202	15062.292	6.885870413	4.146376811	3	High	5641	Unique	Used	92686	1	62.391	1	5.8736	2.0523	1	70	25/52	53.96486	5641	1859.05	MS2	620.356	204	Glioma_grade 2_7rep.raw
40217847			qVLDLEDLVFTQGSHFMANk	Unambiguous	N-Term(iTRAQ4plex); K20(iTRAQ4plex)	HCD	2.355507	0	1	1	2.32981	2.6336	2.1095	2.3845556	9.104384191	3809.011	3369.6848	8874.267	8035.201	8.654790336	7.017479893	3	High	7543	Unique	Used	77628	1	72.894	1	7.9809	-0.9446	1	100	37/76	42.90594	7543	2580.33	MS2	860.783	97	Glioma_grade 2_5.raw
40254924	NP_060979.2	LRRC59	leucine-rich repeat-containing protein 59 [Homo sapiens]	5	13	2.354281425	16.28967632	78.167323	24.43	6.25E-05	307	34.909	9.5688	Unclassified	Biological_process unknown	Molecular function unknown	Integral to membrane	Membrane fraction																								
40254924			vAGDcLDEk	Unambiguous	N-Term(iTRAQ4plex); C5(Methylthio); K9(iTRAQ4plex)	HCD	1.7434403	0	1	1	1.63655	1.742	1.7448	1.8572229	5.164082982	530086.1	497993.28	867510.44	924884.56	4.414649509	4.526862585	2	High	2416	Unique	Used	50052	1	29.679	1	3.3459	-0.1284	1	8	16/16	10.94686	2416	1283.62	MS2	642.314	76	Glioma_grade 2_2.raw
40254924			dNPLDPVLAk	Unambiguous	N-Term(iTRAQ4plex); K10(iTRAQ4plex)	HCD	1.7210921	0	1	1	1.62429	1.7317	1.7112	1.8242923	4.765007828	309491.78	290301.72	502704.66	529595.2	4.524697769	3.683906958	2	High	3662	Unique	Used	51223	1	38.646	1	4.4219	1.1797	1	15	17/18	20.50728	3662	1369.79	MS2	685.401	212	Glioma_grade 2_2.raw
40254924			lVTLPVSFAQLk	Unambiguous	N-Term(iTRAQ4plex); K12(iTRAQ4plex)	HCD	2.4899507	0	1	1	2.42324	2.4324	2.5473	2.5569174	2.890478001	65493.25	65247.09	158705.6	166831.4	0.266270388	3.530049292	2	High	7117	Unique	Used	54473	1	63.972	1	4.1942	1.2325	1	30	15/22	49.51936	7117	1604	MS2	802.506	87	Glioma_grade 2_2.raw
40254924			lVNLQHLDLLNNk	Unambiguous	N-Term(iTRAQ4plex); K13(iTRAQ4plex)	HCD	2.121639	0	1	1	1.91047	2.1651	2.0833	2.3609498	8.780904864	63704.594	56213.516	121705.664	132717.28	8.834348794	6.120822457	2	High	5056	Unique	Used	82120	1	52.549	1	5.68	1.5902	1	33	18/24	11.45961	5056	1822.08	MS2	911.545	108	Glioma_grade 2_6.raw
40254924			lVNLQHLDLLNNk	Unambiguous	N-Term(iTRAQ4plex); K13(iTRAQ4plex)	HCD	2.475962	0	1	1	2.52687	2.4002	2.5557	2.427604	3.040733584	56597.605	59585.035	143014.92	144648.86	3.63639871	0.803278087	3	High	5021	Unique	Used	82088	1	52.276	1	4.932	0.2429	1	11	23/48	15.98545	5021	1822.08	MS2	608.031	106	Glioma_grade 2_6.raw
40254924			lVTLPVSFAQLk	Unambiguous	N-Term(iTRAQ4plex); K12(iTRAQ4plex)	HCD	2.7216167	0	1	1	2.61122	2.6906	2.7518	2.8353705	3.484963527	53498.914	51921.375	139697.3	147216.34	2.116269145	3.706177351	2	High	6867	Unique	Used	62235	1	63.882	1	4.2023	0.852	1	100	15/22	41.56031	6867	1604	MS2	802.506	87	Glioma_grade 2_3.raw
40254924			lVTLPVSFAQLk	Unambiguous	N-Term(iTRAQ4plex); K12(iTRAQ4plex)	HCD	2.3542814	0	1	1	2.24989	2.3992	2.3122	2.465595	4.029046775	45170.055	42359.86	101627.8	104442.27	4.540408707	1.931506145	2	High	6970	Unique	Used	69967	1	64.573	1	4.6507	1.0042	1	75	15/22	42.36415	6970	1604	MS2	802.506	87	Glioma_grade 2_4.raw
40254924			lTTLPSDFcGLTHLVk	Unambiguous	N-Term(iTRAQ4plex); C9(Methylthio); K16(iTRAQ4plex)	HCD	2.7927337	0	1	1	2.77468	2.7674	2.8181	2.810737	0.909224137	17442.936	17488.68	48398.62	49156.08	0.185195512	1.098061093	3	High	6749	Unique	Used	83507	1	68.476	1	7.5083	-2.0419	1	33	33/60	7.399034	6749	2079.12	MS2	693.711	194	Glioma_grade 2_6.raw
40254924			lTTLPSDFcGLTHLVk	Unambiguous	N-Term(iTRAQ4plex); C9(Methylthio); K16(iTRAQ4plex)	HCD	2.9459379	0	1	1	2.47917	3.1902	2.7561	3.5465767	15.73588893	15832.224	12303.605	39250.723	43635.68	17.73617847	7.481644076	3	High	7157	Unique	Used	77314	1	69.168	1	6.7189	0.6883	1	29	31/60	19.40406	7157	2079.12	MS2	693.713	180	Glioma_grade 2_5.raw
40254924			lTTLPSDFcGLTHLVk	Unambiguous	N-Term(iTRAQ4plex); C9(Methylthio); K16(iTRAQ4plex)	HCD	2.107204	0	1	1	1.86487	2.0709	2.1399	2.376314	9.979653762	13621.219	12266.001	25401.783	29147.87	7.403528365	9.711825374	2	High	6762	Unique	Used	83517	1	68.612	1	4.1356	-0.0098	1	100	17/30	18.12623	6762	2079.12	MS2	1040.06	202	Glioma_grade 2_6.raw
40254924			lVTLPVSFAQLk	Unambiguous	N-Term(iTRAQ4plex); K12(iTRAQ4plex)	HCD	2.5097916	0	1	1	2.31706	2.6427	2.3933	2.7296038	7.808859417	8920.476	7821.298	20669.32	21349.043	9.284992349	2.287746158	3	High	6870	Unique	Used	62238	1	63.904	1	5.4317	-0.5643	1	100	21/44	0	6870	1604	MS2	535.339	80	Glioma_grade 2_3.raw
40254924			lVTLPVSFAQLk	Unambiguous	N-Term(iTRAQ4plex); K12(iTRAQ4plex)	HCD	2.0219343	0	1	1	1.94217	2.2488	1.826	2.1142898	9.172019219	6619.4316	5716.807	12856.076	12086.986	10.34759453	4.3605613	3	High	7111	Unique	Used	54468	1	63.916	1	5.0986	-1.021	1	81	23/44	15.91107	7111	1604	MS2	535.339	81	Glioma_grade 2_2.raw
40254924			lVTLPVSFAQLk	Unambiguous	N-Term(iTRAQ4plex); K12(iTRAQ4plex)	HCD	2.2991855	0	1	1	2.28373	1.8884	2.796	2.3119645	16.01629329	2740.4033	3314.109	6258.3447	7662.102	13.40062323	14.26112721	3	High	6964	Unique	Used	69962	1	64.517	1	5.078	-1.2493	1	100	21/44	13.55659	6964	1604	MS2	535.338	81	Glioma_grade 2_4.raw
40548389	NP_056965.3	DKK3	dickkopf-related protein 3 precursor [Homo sapiens]	2	5	0.447769105	19.89242243	36.655058	10	5.81E-04	350	38.365	4.6528	Ligand	Cell communication,Signal transduction	Lipid binding,Receptor binding	Extracellular	Cytoplasm																								
40548389			eVEELMEDTQHk	Unambiguous	N-Term(iTRAQ4plex); K12(iTRAQ4plex)	HCD	0.37655753	0	1	1	0.3317	0.3841	0.37	0.42850268	0.105589258	52550.836	45378.023	17431.246	19444.605	10.35842705	7.721366495	2	High	3596	Unique	Used	66868	1	37.747	1	5.0967	4.7636	1	100	19/22	0	3596	1775.88	MS2	888.444	154	Glioma_grade 2_4.raw
40548389			eVEELMEDTQHk	Unambiguous	N-Term(iTRAQ4plex); K12(iTRAQ4plex)	HCD	0.4330252	0	1	1	0.4221	0.4176	0.4487	0.44382885	0.035754556	43702.79	44177.742	18447.127	19607.355	0.764314399	4.311739608	3	High	3614	Unique	Used	66884	1	37.897	1	5.095	1.6972	1	28	26/44	18.56694	3614	1775.88	MS2	592.63	128	Glioma_grade 2_4.raw
40548389			eVEELMEDTQHk	Unambiguous	N-Term(iTRAQ4plex); K12(iTRAQ4plex)	HCD	0.47389433	0	1	1	0.42765	0.4849	0.4642	0.52633137	0.086572249	42513.15	37494.223	18180.654	19734.387	8.871475673	5.795352512	2	High	3248	Unique	Used	58871	1	35.721	1	5.0095	0.1582	1	100	20/22	0	3248	1775.87	MS2	888.44	146	Glioma_grade 2_3.raw
40548389			eVEELMEDTQHk	Unambiguous	N-Term(iTRAQ4plex); K12(iTRAQ4plex)	HCD	0.6268832	0	1	1	0.58119	0.6432	0.6122	0.677453	0.06571001	34879.625	31518.477	20271.568	21352.285	7.158911089	3.671848059	2	High	3045	Unique	Used	73680	1	34.8	1	4.8237	2.9077	1	100	19/22	0	3045	1775.88	MS2	888.442	178	Glioma_grade 2_5.raw
40548389			gLLFPVcTPLPVEGELcHDPASR	Unambiguous	N-Term(iTRAQ4plex); C7(Methylthio); C17(Methylthio)	HCD	0.4477691	0	1	1	0.36106	0.532	0.3906	0.5755237	0.225796272	13911.613	9441.594	5022.984	5433.8613	27.06935066	5.556821713	3	High	7853	Unique	Used	77879	1	75.885	1	7.6615	-0.9935	1	23	33/88	29.37649	7853	2686.3	MS2	896.103	66	Glioma_grade 2_5.raw
44917606	NP_071363.1	NAPB	beta-soluble NSF attachment protein [Homo sapiens]	3	8	0.388157606	16.26492358	124.95491	33.22	1.12E-04	298	33.535	5.4653	Unclassified	Transport	Molecular function unknown	Integral to membrane	-																								
44917606			lLEAHEEQNSEAYTEAVk	Unambiguous	N-Term(iTRAQ4plex); K18(iTRAQ4plex)	HCD	0.3098388	0	1	1	0.30863	0.3105	0.3092	0.31105384	0.003593369	148024.48	147149.3	45684.934	45771.355	0.419309407	0.133635151	2	High	2645	Unique	Used	73312	1	31.729	1	7.2803	12.737	1	20	24/34	0	2645	2349.21	MS2	1175.11	185	Glioma_grade 2_5.raw
44917606			hDSATSFVDAGNAYk	Unambiguous	N-Term(iTRAQ4plex); K15(iTRAQ4plex)	HCD	0.41435575	0	1	1	0.41459	0.3432	0.5004	0.41416314	0.153840253	111877.44	135161.92	46383.098	55979.082	13.32954692	13.25760229	2	High	1952	Unique	Used	72663	1	26.676	1	6.1427	9.1989	1	100	24/28	0	1952	1870.93	MS2	935.971	132	Glioma_grade 2_5.raw
44917606			aALcHFIVDELNAk	Unambiguous	N-Term(iTRAQ4plex); C4(Methylthio); K14(iTRAQ4plex)	HCD	0.36294594	0	1	1	0.33191	0.3661	0.3601	0.39717448	0.073573281	83493.03	75703.16	27712.252	30067.361	6.920102675	5.764363788	2	High	6704	Unique	Used	83474	1	67.983	1	5.9246	1.0373	1	59	20/26	15.49808	6704	1877.99	MS2	939.497	209	Glioma_grade 2_6.raw
44917606			aALcHFIVDELNAk	Unambiguous	N-Term(iTRAQ4plex); C4(Methylthio); K14(iTRAQ4plex)	HCD	0.28050786	0	1	1	0.22161	0.2699	0.2892	0.35223103	0.190926644	63701.36	52311.15	14116.81	18425.61	13.88487281	18.72498541	3	High	6703	Unique	Used	83473	1	67.977	1	5.1267	1.5827	1	20	24/52	12.16305	6703	1877.99	MS2	626.668	163	Glioma_grade 2_6.raw
44917606			aALcHFIVDELNAk	Unambiguous	N-Term(iTRAQ4plex); C4(Methylthio); K14(iTRAQ4plex)	HCD	0.41336927	0	1	1	0.40552	0.3727	0.4576	0.42058566	0.085080366	51869.207	56436.695	21033.865	23736.465	5.964036453	8.537023457	2	High	6127	Unique	Used	93032	1	67.697	1	5.5255	2.2724	1	78	20/26	28.52261	6127	1877.99	MS2	939.499	205	Glioma_grade 2_7rep.raw
44917606			lLEAHEEQNSEAYTEAVk	Unambiguous	N-Term(iTRAQ4plex); K18(iTRAQ4plex)	HCD	0.4308951	0	1	1	0.44785	0.5092	0.3621	0.41161615	0.143161267	29590.75	26027.932	13252.3	10713.517	9.059160258	14.98125998	3	High	3086	Unique	Used	66395	1	33.788	1	9.2563	1.1437	1	34	30/68	17.5807	3086	2349.18	MS2	783.733	162	Glioma_grade 2_4.raw
44917606			lLEAHEEQNSEAYTEAVk	Unambiguous	N-Term(iTRAQ4plex); K18(iTRAQ4plex)	HCD	0.3404259	0	1	1	0.27867	0.3568	0.3277	0.41949093	0.170225692	21121.793	16498.73	5885.933	6921.068	17.37880782	11.43048209	2	High	3133	Unique	Used	66440	1	34.123	1	7.2756	-3.2674	1	100	25/34	18.62107	3133	2349.17	MS2	1175.09	163	Glioma_grade 2_4.raw
44917606			aALcHFIVDELNAk	Unambiguous	N-Term(iTRAQ4plex); C4(Methylthio); K14(iTRAQ4plex)	HCD	0.44844642	0	1	1	0.5031	0.4469	0.4502	0.39989948	0.09374682	6794.7466	7649.5605	3418.4426	3059.0552	8.369313961	7.846402282	3	High	6102	Unique	Used	93016	1	67.391	1	4.4228	1.6802	1	97	21/52	13.96058	6102	1877.99	MS2	626.668	174	Glioma_grade 2_7rep.raw
45267837	NP_055769.2	RPH3A	rabphilin-3A isoform 2 [Homo sapiens]	3	4	0.501905203	20.75447532	18.862003	6.23	2.48E-04	690	76.338	8.4849	Membrane transport protein	Transport	Auxiliary transport protein activity	Cytoplasmic vesicle	-																								
45267837			nPIWNETLVYHGITDEDMQR	Unambiguous	N-Term(iTRAQ4plex)	HCD	0.40814582	0	1	2	0.40174	0.4447	0.3751	0.41524243	0.070777525	43651.664	39432.344	17536.408	16373.983	7.18191107	4.847827323	3	High	6079	Unique	Used	61496	1	57.811	1	5.8665	0.1446	1	33	32/76	10.65518	6079	2575.24	MS2	859.084	76	Glioma_grade 2_3.raw
45267837			nPIWNETLVYHGITDEDMQR	Unambiguous	N-Term(iTRAQ4plex)	HCD	0.61823815	0	1	2	0.53308	0.6054	0.6295	0.7149487	0.120803961	27147.352	23903.15	14471.843	17089.525	8.987168172	11.72940725	3	High	6248	Unique	Used	53661	1	57.494	1	5.326	1.5667	1	41	30/76	36.85009	6248	2575.24	MS2	859.085	88	Glioma_grade 2_2.raw
45267837			lHSVWLck	Unambiguous	N-Term(iTRAQ4plex); C7(Methylthio); K8(iTRAQ4plex)	HCD	0.4274332	0	1	2	0.41804	0.3778	0.4824	0.43591714	0.101194131	22110.605	24467.7	9243.224	10665.89	7.156627354	10.10569105	3	High	3728	Unique	Used	91271	1	42.276	1	3.219	4.5615	1	100	14/28	67.10311	3728	1319.73	MS2	440.58	245	Glioma_grade 2_7rep.raw
45267837			tLNPEFNEEFFYDIk	Unambiguous	N-Term(iTRAQ4plex); K15(iTRAQ4plex)	HCD	0.57637715	0	1	2	0.6796	0.6021	0.5473	0.48492402	0.142918315	8363.599	9439.985	5683.904	4577.676	8.550186746	15.24563119	3	High	7458	Unique	Used	54780	1	66.762	1	4.4505	-0.314	1	69	18/56	38.86617	7458	2194.09	MS2	732.036	129	Glioma_grade 2_2.raw
45827708	NP_996735.1	QKI	protein quaking isoform HQK-6 [Homo sapiens]	2	2	2.334057331	34.60363017	10.714495	9.4	3.72E-04	319	35.108	7.4302	RNA binding protein	RNA metabolism	RNA binding	Cytoplasm	-																								
45827708			lLVPAAEGEDSLk	Unambiguous	N-Term(iTRAQ4plex); K13(iTRAQ4plex)	HCD	2.9051652	0	1	4	2.66153	2.8241	2.9816	3.1636791	7.391210796	66105.016	62299.58	175940.67	197095.88	4.191204497	8.020121593	2	High	4264	Unique	Used	51792	1	43.017	1	3.9488	4.566	1	69	14/24	57.33933	4264	1629.94	MS2	815.473	176	Glioma_grade 2_2.raw
45827708			lmSSLPNFcGIFNHLER	Unambiguous	N-Term(iTRAQ4plex); M2(Oxidation); C9(Methylthio)	HCD	1.7629493	0	1	4	1.54244	1.6622	1.8564	2.0005884	11.51406457	3601.4114	3341.882	5554.944	6685.7305	5.286108136	13.0644239	3	High	7264	Unique	Used	83888	1	74.09	1	6.7657	0.4274	1	100	31/64	56.54365	7264	2184.05	MS2	728.689	131	Glioma_grade 2_6.raw

47132585	NP_002727.2	PRKAR2B	cAMP-dependent protein kinase type II-beta regulatory subunit [Homo sapiens]	5	9	0.395958334	12.80530436	67.64701	22.01	4.06E-04	418	46.273	4.9194	Serine/threonine kinase	Cell communication,Signal transduction	Protein serine/threonine kinase activity	Cytoplasm	Nucleus, Golgi apparatus																								
47132585			hQPADLLEFALQHFTR	Unambiguous	N-Term(iTRAQ4plex)	HCD	0.37942284	0	1	1	0.33303	0.3921	0.3686	0.43404472	0.11095072	92586.81	78632.74	30834.482	34130.125	11.52557348	7.174280339	3	High	6271	Unique	Used	93112	1	69.64	1	7.985	-0.4263	1	15	30/60	18.60086	6271	2067.09	MS2	689.701	193	Glioma_grade2_7rep.raw
47132585			hQPADLLEFALQHFTR	Unambiguous	N-Term(iTRAQ4plex)	HCD	0.4798062	0	1	1	0.49281	0.5231	0.439	0.46600494	0.075068654	40607.867	38255.28	20011.877	17827.15	4.218777171	8.165301273	3	High	6189	Unique	Used	93062	1	68.606	1	7.8637	1.6996	1	28	30/60	13.67771	6189	2067.09	MS2	689.702	204	Glioma_grade2_7rep.raw
47132585			hQPADLLEFALQHFTR	Unambiguous	N-Term(iTRAQ4plex)	HCD	0.3011525	0	1	1	0.27257	0.2578	0.347	0.32818493	0.142406915	20670.61	21854.371	5634.2285	7172.275	3.93672336	16.98454398	4	High	6263	Unique	Used	93107	1	69.539	1	6.5858	-0.9643	1	18	35/90	4.954077	6263	2067.09	MS2	517.527	221	Glioma_grade2_7rep.raw
47132585			dGEHVIDQGDDGDNFYVIDR	Unambiguous	N-Term(iTRAQ4plex)	HCD	0.3434494	0	1	1	0.30769	0.3758	0.317	0.38711947	0.116337057	20160.764	16507.145	6203.3345	6390.2373	14.09133404	2.098852326	2	High	4678	Unique	Used	52180	1	46.098	1	6.3527	0.0361	1	61	25/38	15.17824	4678	2423.09	MS2	1212.05	61	Glioma_grade2_2.raw
47132585			mYESFIESLPFLk	Unambiguous	N-Term(iTRAQ4plex); M1(Oxidation); K13(iTRAQ4plex)	HCD	0.42125064	0	1	1	0.4512	0.4325	0.4095	0.3925417	0.061033002	19159.146	19988.205	8644.643	7846.2046	2.995008479	6.847206653	2	High	8084	Unique	Used	63324	1	74.231	1	4.9487	-2.5543	1	100	20/24	11.30099	8084	1908	MS2	954.505	238	Glioma_grade2_3.raw
47132585			nLDPEQMSQVLDAMFEk	Unambiguous	N-Term(iTRAQ4plex); K17(iTRAQ4plex)	HCD	0.4100792	0	1	1	0.34015	0.4112	0.4091	0.49463016	0.152763574	12410.763	10265.141	4221.4683	5077.448	13.38146313	13.01805567	3	High	8578	Unique	Used	55797	1	75.911	1	5.2867	3.4886	1	23	25/64	19.69945	8578	2283.13	MS2	761.716	131	Glioma_grade2_2.raw
47132585			hQPADLLEFALQHFTR	Unambiguous	N-Term(iTRAQ4plex)	HCD	0.37635338	0	1	1	0.37475	0.4086	0.3467	0.3780976	0.06714309	6949.8906	6373.5635	2604.498	2409.829	6.117404654	5.490338783	4	High	6183	Unique	Used	93059	1	68.519	1	7.0642	-1.3186	1	59	34/90	8.150988	6183	2067.09	MS2	517.527	229	Glioma_grade2_7rep.raw
47132585			aASAHAIGTVk	Unambiguous	N-Term(iTRAQ4plex); K11(iTRAQ4plex)	HCD	0.39800712	0	1	1	0.30672	0.387	0.4067	0.5131987	0.21072935	6322.406	5010.304	1939.2177	2571.2815	16.37377506	19.81761128	2	High	978	Unique	Used	71789	1	18.858	1	2.9285	-6.3149	1	100	14/20	47.27716	978	1313.77	MS2	657.389	152	Glioma_grade2_5.raw
47132585			mYESFIESLPFLk	Unambiguous	N-Term(iTRAQ4plex); M1(Oxidation); K13(iTRAQ4plex)	HCD	0.39595833	0	1	1	0.35082	0.3655	0.4252	0.44298196	0.113214613	4460.6665	4281.785	1564.8928	1896.7535	2.893657956	13.55776593	3	High	8100	Unique	Used	63336	1	74.409	1	3.951	-1.1538	1	100	19/48	20.54716	8100	1908	MS2	636.673	210	Glioma_grade2_3.raw
47717100	NP_998784.1	ATP6V1H	V-type proton ATPase subunit H isoform 2 [Homo sapiens]	10	17	0.475229859	25.96616618	98.972519	25.81	3.15E-04	465	54.116	6.481	ATPase	Transport	ATPase activity	-	-																								
47717100			yNALLAVQk	Unambiguous	N-Term(iTRAQ4plex); K9(iTRAQ4plex)	HCD	0.4945672	0	1	2	0.4681	0.5103	0.4801	0.5234217	0.051914061	544634.2	499603.25	254942.39	261503.19	6.098553563	1.796582776	2	High	3600	Unique	Used	51165	1	38.196	1	4.3622	-7.684	1	18	15/16	42.66092	3600	1307.78	MS2	654.395	245	Glioma_grade2_2.raw
47717100			qLENLEQQk	Unambiguous	N-Term(iTRAQ4plex); K9(iTRAQ4plex)	HCD	0.4780856	0	1	2	0.44593	0.4728	0.4831	0.5121752	0.057206851	452757.7	427025.4	201899.83	218711.81	4.136356751	5.652656243	2	High	1495	Unique	Used	49180	1	23.303	1	4.295	1.8368	1	14	16/16	16.17942	1495	1417.79	MS2	709.399	236	Glioma_grade2_2.raw
47717100			yNALLAVQk	Unambiguous	N-Term(iTRAQ4plex); K9(iTRAQ4plex)	HCD	0.6294478	0	1	2	0.58341	0.6472	0.6135	0.6805227	0.066510629	334241	301308.75	194997.95	205047.45	7.32802343	3.552631575	2	High	3628	Unique	Used	51191	1	38.404	1	3.941	3.2369	1	26	15/16	46.52729	3628	1307.8	MS2	654.402	206	Glioma_grade2_2.raw
47717100			vIEQLGGk	Unambiguous	N-Term(iTRAQ4plex); K8(iTRAQ4plex)	HCD	0.47522986	0	1	2	0.43303	0.4537	0.4958	0.5194438	0.082578014	217222.3	207344.14	94063.03	107703.63	3.290374963	9.560903137	2	High	1679	Unique	Used	57405	1	24.116	1	3.1179	-8.7901	1	32	13/14	65.7208	1679	1131.69	MS2	566.348	264	Glioma_grade2_3.raw
47717100			lEWSPVHk	Unambiguous	N-Term(iTRAQ4plex); K8(iTRAQ4plex)	HCD	0.5649526	0	1	2	0.56411	0.5476	0.5828	0.5657756	0.025428146	206405.61	212612.92	116434.43	120291.19	2.095005676	2.304052387	3	High	2033	Unique	Used	79477	1	26.879	1	3.681	1.7535	1	24	21/28	33.82916	2033	1283.74	MS2	428.584	213	Glioma_grade2_6.raw
47717100			lLEVSDDPQVLAVAAHDVGEYVR	Unambiguous	N-Term(iTRAQ4plex)	HCD	0.3337698	0	1	2	0.26792	0.32	0.3453	0.41242787	0.178484783	122990.97	102962.75	32951.816	42464.71	12.53539015	17.83861499	3	High	8088	Unique	Used	55354	1	71.865	1	8.7526	-0.0805	1	5	38/88	6.654086	8088	2639.38	MS2	880.465	120	Glioma_grade2_2.raw
47717100			yNIIPVLSDILQESVk	Unambiguous	N-Term(iTRAQ4plex); K16(iTRAQ4plex)	HCD	0.42258245	0	1	2	0.40014	0.4478	0.4	0.4477055	0.064949919	91494.836	81750.13	36610.297	36599.984	7.954687345	0.019921771	2	High	8726	Unique	Used	55937	1	76.941	1	4.3649	0.9462	1	55	17/30	26.9558	8726	2119.23	MS2	1060.12	60	Glioma_grade2_2.raw
47717100			vSIFFDYAR	Unambiguous	N-Term(iTRAQ4plex)	HCD	0.3824941	0	1	2	0.35288	0.3765	0.3881	0.41408727	0.06644524	69264.07	64919.31	24442.09	26882.26	4.579120393	6.723751024	2	High	4504	Unique	Used	87033	1	60.121	1	3.5782	-0.1356	1	85	13/16	26.86239	4504	1261.67	MS2	631.338	235	Glioma_grade2_1rep.raw
47717100			lLEVSDDPQVLAVAAHDVGEYVR	Unambiguous	N-Term(iTRAQ4plex)	HCD	0.33980024	0	1	2	0.30228	0.3677	0.3169	0.385436	0.11599386	65563.336	53900.926	19818.629	20775.357	13.80591829	3.333049662	3	High	7763	Unique	Used	63040	1	71.394	1	8.4411	2.2782	1	10	37/88	5.542379	7763	2639.39	MS2	880.467	118	Glioma_grade2_3.raw
47717100			lmVHNWEYLGk	Unambiguous	N-Term(iTRAQ4plex); M2(Oxidation); K11(iTRAQ4plex)	HCD	0.51955926	0	1	2	0.45661	0.5245	0.5152	0.59187865	0.106142207	60191.043	52395.82	27483.584	31011.967	9.791648683	8.53037027	3	High	3361	Unique	Used	80667	1	37.63	1	4.2302	12.775	1	32	17/40	50.64831	3361	1693.92	MS2	565.311	229	Glioma_grade2_6.raw
47717100			lMVHNWEYLGk	Unambiguous	N-Term(iTRAQ4plex); K11(iTRAQ4plex)	HCD	0.4248806	0	1	2	0.38255	0.3572	0.4974	0.46440473	0.15588111	31548.867	33788.758	12069.028	15691.658	4.8481778	18.45477622	3	High	4032	Unique	Used	81241	1	43.566	1	4.4742	3.4639	1	32	18/40	27.33904	4032	1677.91	MS2	559.974	282	Glioma_grade2_6.raw
47717100			lLEVSDDPQVLAVAAHDVGEYVR	Unambiguous	N-Term(iTRAQ4plex)	HCD	0.51159513	0	1	2	0.43237	0.4653	0.5547	0.5968449	0.149271982	30658.824	28491.514	13256.011	17005.014	5.181777991	17.52052645	3	High	5647	Unique	Used	87879	1	72.645	1	8.6424	-1.6068	1	28	37/88	12.67748	5647	2639.38	MS2	880.463	134	Glioma_grade2_1rep.raw
47717100			tFINLMTHIck	Unambiguous	N-Term(iTRAQ4plex); C10(Methylthio); K11(iTRAQ4plex)	HCD	0.12720524	0	1	2	0.12799	0.1317	0.1229	0.12639734	0.02868463	25406.738	24697.629	3251.8232	3121.7146	2.00148535	2.886957801	3	High	6170	Unique	Used	83040	1	62.883	1	5.3234	-7.8268	1	100	26/40	26.43276	6170	1654.86	MS2	552.291	219	Glioma_grade2_6.raw
47717100			rYNIIPVLSDILQESVk	Unambiguous	N-Term(iTRAQ4plex); K17(iTRAQ4plex)	HCD	0.38469425	1	1	2	0.34513	0.3075	0.4713	0.41994283	0.190743184	12182.069	13672.324	4204.442	5741.594	8.15156957	21.85655879	3	High	7275	Unique	Used	83896	1	74.213	1	6.8527	1.8209	1	27	31/64	32.78322	7275	2275.33	MS2	759.115	50	Glioma_grade2_6.raw
47717100			yNIIPVLSDILQESVk	Unambiguous	N-Term(iTRAQ4plex); K16(iTRAQ4plex)	HCD	0.5630591	0	1	2	0.54154	0.5461	0.5799	0.584756	0.039761748	11757.549	11659.331	6367.2236	6817.864	0.593167099	4.833504219	3	High	6086	Unique	Used	88233	1	76.922	1	4.501	-0.7018	1	43	17/60	30.20964	6086	2119.22	MS2	707.079	110	Glioma_grade2_1rep.raw
47717100			lLEVSDDPQVLAVAAHDVGEYVR	Unambiguous	N-Term(iTRAQ4plex)	HCD	0.40787306	0	1	2	0.35112	0.4603	0.3679	0.48226324	0.157687351	8373.128	6387.5767	2940.0005	3080.4934	19.02343848	3.30017716	3	High	8192	Unique	Used	55447	1	72.736	1	7.9222	-0.2193	1	61	37/88	8.406735	8192	2639.38	MS2	880.465	124	Glioma_grade2_2.raw
47717100			lMVHNWEYLGk	Unambiguous	N-Term(iTRAQ4plex); K11(iTRAQ4plex)	HCD	0.47606152	0	1	2	0.43307	0.4522	0.4989	0.52095175	0.085313539	6605.8	6326.1587	2860.7847	3295.6235	3.058102243	9.9888589	3	High	3914	Unique	Used	81146	1	42.413	1	3.9889	1.7178	1	74	17/40	28.72857	3914	1677.91	MS2	559.973	281	Glioma_grade2_6.raw
47717125	NP_001001132.1	ITSN1	intersectin-1 isoform ITSN-s [Homo sapiens]	2	2	0.323841572	5.765795711	30.38595	4.51	8.82E-04	1220	137.56	7.3276	Adapter molecule	Signal transduction,Vesicle-mediated transport	Receptor signaling complex scaffold activity	Cytoplasm, Nucleus	Cytoplasmic vesicle, Endosome, Golgi apparatus, Cytosol, Axon, Dendrite, Caveola, Cell surface, Plasma membrane																								
47717125			iAEITHLQQQLQESQQMLGR	Unambiguous	N-Term(iTRAQ4plex)	HCD	0.3370447	0	1	2	0.25042	0.2848	0.383	0.4355442	0.253278877	26323.867	23149.072	6592.148	10082.443	9.075341464	29.60206056	3	High	6693	Unique	Used	69717	1	62.271	1	6.8389	2.399	1	18	30/76	9.136348	6693	2495.32	MS2	832.445	141	Glioma_grade2_4.raw
47717125			iPENEVPAPVkPVTDSTSAPAPk	Unambiguous	N-Term(iTRAQ4plex); K11(iTRAQ4plex); K23(iTRAQ4plex)	HCD	0.31063843	0	1	2	0.28299	0.3096	0.3116	0.34088734	0.07602749	23341.818	21336.947	6605.4463	7273.4946	6.346002982	6.807169027	3	High	2850	Unique	Used	80203	1	33.587	1	6.9992	5.3306	1	100	24/88	19.53651	2850	2776.56	MS2	926.192	46	Glioma_grade2_6.raw
48255891	NP_001001329.1	PRKCSH	glucosidase 2 subunit beta isoform 2 precursor [Homo sapiens]	5	7	2.059291124	15.4506833	45.597726	11.43	9.76E-05	525	59.141	4.4243	Regulatory/other subunit	Metabolism,Energy pathways	Glucosidase activity	Endoplasmic reticulum	-																								
48255891			lWEEQLAAAk	Unambiguous	N-Term(iTRAQ4plex); K10(iTRAQ4plex)	HCD	1.8576697	0	1	2	1.74388	1.8366	1.8777	1.9775121	5.210926876	524321.9	497852.97	914353	984510.3	3.662066138	5.225094674	2	High	4683	Unique	Used	52184	1	46.152	1	5.0443	-0.5281	1	7	17/18	20.96824	4683	1446.82	MS2	723.913	295	Glioma_grade2_2.raw
48255891			lIELQAGk	Unambiguous	N-Term(iTRAQ4plex); K8(iTRAQ4plex)	HCD	1.6102556	0	1	2	1.53991	1.6428	1.5798	1.6852992	4.013195018	487357.12	456842.66	750486.4	769916.56	4.570427159	1.807310069	2	High	2379	Unique	Used	58059	1	29.247	1	3.2881	-0.3053	1	19	13/14	52.57222	2379	1159.73	MS2	580.368	183	Glioma_grade2_3.raw
48255891			aQQEQELAADAFk	Unambiguous	N-Term(iTRAQ4plex); K13(iTRAQ4plex)	HCD	2.0592911	0	1	2	1.94006	2.101	2.0208	2.1884127	5.166138425	471141.97	435061.25	914042.7	952093.56	5.630728565	2.883607346	2	High	3240	Unique	Used	50825	1	35.623	1	5.6909	2.9587	1	6	21/24	17.98976	3240	1736.91	MS2	868.959	207	Glioma_grade2_2.raw
48255891			lWEEQLAAAk	Unambiguous	N-Term(iTRAQ4plex); K10(iTRAQ4plex)	HCD	1.8240014	0	1	2	1.72971	1.8826	1.7701	1.9266251	5.067414594	141450.38	129959.78	244668.75	250383.77	5.987308051	1.632606333	2	High	4529	Unique	Used	60059	1	45.688	1	5.0517	-1.4562	1	52	17/18	30.75013	4529	1446.82	MS2	723.912	299	Glioma_grade2_3.raw
48255891			iLIEDWk	Unambiguous	N-Term(iTRAQ4plex); K7(iTRAQ4plex)	HCD	2.463202	0	1	2	2.62976	2.2746	2.6812	2.3191342	8.43592967	124406.16	143828.77	327158.56	333558.22	10.24017211	1.36979811	2	High	5317	Unique	Used	68458	1	51.241	1	2.9609	-3.2027	1	30	12-Nov	33.23477	5317	1204.71	MS2	602.861	277	Glioma_grade2_4.raw
48255891			lGGSPTSLGTWGSWIGPDHDk	Unambiguous	N-Term(iTRAQ4plex); K21(iTRAQ4plex)	HCD	2.389216	0	1	2	2.24161	1.9946	2.8327	2.520557	15.06151177	70123.12	78806.41	157188.77	198636.05	8.24552826	16.47307951	3	High	5449	Unique	Used	82450	1	56.1	1	8.5875	0.1865	1	3	34/80	3.599874	5449	2456.25	MS2	819.42	149	Glioma_grade2_6.raw
48255891			lWEEQLAAAk	Unambiguous	N-Term(iTRAQ4plex); K10(iTRAQ4plex)	HCD	2.2662485	0	1	2	2.46835	2.023	2.5631	2.100611	11.65736588	8395.668	10244.111	20723.48	21518.895	14.02427121	2.662934271	3	High	4542	Unique	Used	60071	1	45.789	1	4.2219	-1.128	1	38	19/36	6.069922	4542	1446.82	MS2	482.944	290	Glioma_grade2_3.raw
48255945	NP_001673.2	ATP2B1	plasma membrane calcium-transporting ATPase 1 isoform 1b [Homo sapiens]	7	15	0.455431014	21.14513285	302.75487	20.57	2.35E-04	1220	134.6	5.9097	ATPase	Transport	ATPase activity	Plasma membrane	Mitochondrion, Cytoplasm, Nucleus																								
48255945			aQDGAAmEMQPLk	Unambiguous	N-Term(iTRAQ4plex); M7(Oxidation); K13(iTRAQ4plex)	HCD	0.455431	0	1	2	0.41516	0.4431	0.467	0.49841025	0.077595859	334477.06	313401.56	138861.47	156202.56	4.600438572	8.31141792	2	High	1497	Unique	Used	49182	1	23.315	1	3.9963	-0.542	1	16	19/24	22.99125	1497	1693.85	MS2	847.428	178	Glioma_grade2_2.raw
48255945			iQESYGDVYGIcTk	Unambiguous	N-Term(iTRAQ4plex); C12(Methylthio); K14(iTRAQ4plex)	HCD	0.47633725	0	1	2	0.42817	0.4554	0.496	0.5275711	0.091984043	249385.73	234472.6	106778.79	123700.97	4.358786321	10.38337443	2	High	6089	Unique	Used	53510	1	56.343	1	6.5326	-1.2386	1	24	23/26	19.26716	6089	1909.93	MS2	955.466	108	Glioma_grade2_2.raw
48255945			aQDGAAMEMQPLk	Unambiguous	N-Term(iTRAQ4plex); K13(iTRAQ4plex)	HCD	0.44464976	0	1	2	0.43786	0.4161	0.4747	0.45110047	0.055146589	221200.92	232774.62	96855.37	105004.75	3.605410879	5.70938119	2	High	2324	Unique	Used	49965	1	29.045	1	5.6263	-0.3767	1	83	20/24	16.49708	2324	1677.85	MS2	839.431	174	Glioma_grade2_2.raw
48255945			tEcALLGLLLDLk	Unambiguous	N-Term(iTRAQ4plex); C3(Methylthio); K13(iTRAQ4plex)	HCD	0.3747007	0	1	2	0.32739	0.3779	0.3719	0.42931202	0.110793434	104008.06	90104.31	34051.18	38682.863	10.12963358	9.005671409	2	High	8498	Unique	Used	63701	1	77.508	1	5.6116	0.3388	1	100	21/24	34.61374	8498	1735.99	MS2	868.501	180	Glioma_grade2_3.raw
48255945			tEcALLGLLLDLk	Unambiguous	N-Term(iTRAQ4plex); C3(Methylthio); K13(iTRAQ4plex)	HCD	0.39012408	0	1	2	0.37864	0.3701	0.4107	0.40135303	0.048670963	73560.336	75264.38	27852.518	30207.59	1.619275481	5.736425366	2	High	8424	Unique	Used	71232	1	77.408	1	5.5194	2.7296	1	96	21/24	22.85968	8424	1736	MS2	868.503	178	Glioma_grade2_4.raw
48255945			kIQESYGDVYGIcTk	Unambiguous	N-Term(iTRAQ4plex); K1(iTRAQ4plex); C13(Methylthio); K15(iTRAQ4plex)	HCD	0.49427822	1	1	2	0.42906	0.5886	0.4255	0.58374363	0.181068757	53270.242	38832.69	22856.137	22668.336	22.16843851	0.583402077	3	High	4524	Unique	Used	81657	1	48.01	1	5.848	-1.9626	1	28	27/56	75.98307	4524	2182.12	MS2	728.045	140	Glioma_grade2_6.raw
48255945			eANHDGDFGITLAELR	Unambiguous	N-Term(iTRAQ4plex)	HCD	0.5023697	0	1	2	0.42936	0.4697	0.5322	0.58224124	0.134065619	50736.64	46377.812	21784.283	27003.074	6.347473055	15.12786399	2	High	5119	Unique	Used	60607	1	50.291	1	5.7886	-1.2294	1	78	25/30	0	5119	1901.95	MS2	951.476	173	Glioma_grade2_3.raw
48255945			eANHDGDFGITLAELR	Unambiguous	N-Term(iTRAQ4plex)	HCD	0.6421835	0	1	2	0.64332	0.6061	0.6805	0.641111	0.047284413	45493.156	48287.516	29266.74	30957.658	4.213897945	3.97068173	3	High	5259	Unique	Used	68405	1	50.777	1	5.8916	-1.4612	1	8	27/60	13.1732	5259	1901.94	MS2	634.653	147	Glioma_grade2_4.raw
48255945			kIQESYGDVYGIcTk	Unambiguous	N-Term(iTRAQ4plex); K1(iTRAQ4plex); C13(Methylthio); K15(iTRAQ4plex)	HCD	0.66001296	1	1	2	0.65978	0.65	0.6702	0.66024595	0.012531949	43987.184	44651.465	29021.71	29480.95	1.059848283	1.11014343	3	High	4337	Unique	Used	91721	1	48.707	1	6.2809	-2.8018	1	29	26/56	17.77382	4337	2182.12	MS2	728.044	132	Glioma_grade2_7rep.raw
48255945			eANHDGDFGITLAELR	Unambiguous	N-Term(iTRAQ4plex)	HCD	0.5631042	0	1	2	0.47241	0.4579	0.6716	0.65101695	0.20192578	39485.508	40736.805	18653.172	26520.35	2.205871561	24.62918394	3	High	5108	Unique	Used	60596	1	50.223	1	5.6372	0.6568	1	13	26/60	6.46241	5108	1901.95	MS2	634.654	137	Glioma_grade2_3.raw
48255945			sLDkDPLLLSGTHVMEGSGR	Unambiguous	N-Term(iTRAQ4plex); K4(iTRAQ4plex)	HCD	0.51133776	1	1	2	0.52175	0.4642	0.5643	0.5020729	0.081246685	26749.918	30067.008	13956.809	15095.829	8.256472139	5.544479409	3	High	3846	Unique	Used	91347	1	43.716	1	7.1101	0.3639	1	25	32/76	24.24532	3846	2400.28	MS2	800.765	88	Glioma_grade2_7rep.raw
48255945			tEcALLGLLLDLk	Unambiguous	N-Term(iTRAQ4plex); C3(Methylthio); K13(iTRAQ4plex)	HCD	0.39240915	0	1	2	0.33558	0.3283	0.4579	0.44801235	0.178456817	15136.402	15470.333	5079.4624	6930.9004	1.542960231	21.80057983	3	High	8494	Unique	Used	63697	1	77.479	1	4.5495	-2.2707	1	82	21/48	38.7186	8494	1735.99	MS2	579.335	157	Glioma_grade2_3.raw
48255945			gGQVIQIPVADITVGDIAQVk	Unambiguous	N-Term(iTRAQ4plex); K21(iTRAQ4plex)	HCD	0.3745623	0	1	2	0.42483	0.4103	0.3375	0.32601416	0.133750133	13020.6875	13480.659	5531.5186	4394.8857	2.454584463	16.19359452	2	High	7448	Unique	Used	54771	1	66.679	1	5.9015	3.5125	1	85	19/40	0	7448	2409.4	MS2	1205.21	42	Glioma_grade2_2.raw
48255945			sLDkDPLLLSGTHVMEGSGR	Unambiguous	N-Term(iTRAQ4plex); K4(iTRAQ4plex)	HCD	0.33537188	1	1	2	0.28131	0.286	0.384	0.39033124	0.178477528	11298.682	11115.113	3178.3806	4338.576	1.158241027	21.82750489	4	High	3805	Unique	Used	91319	1	43.24	1	4.9142	-0.074	1	25	27/114	23.82123	3805	2400.28	MS2	600.825	98	Glioma_grade2_7rep.raw
48255945			tEcALLGLLLDLk	Unambiguous	N-Term(iTRAQ4plex); C3(Methylthio); K13(iTRAQ4plex)	HCD	0.37578171	0	1	2	0.4281	0.3169	0.4553	0.3370494	0.176034774	9568.929	12926.062	4096.485	4356.721	21.10560088	4.353724263	3	High	8437	Unique	Used	71243	1	77.53	1	4.6204	-1.6378	1	28	22/48	12.50147	8437	1735.99	MS2	579.335	161	Glioma_grade2_4.raw

48255949	NP_001674.2	ATP2B2	plasma membrane calcium-transporting ATPase 2 isoform 2 [Homo sapiens]	14	25	0.469054103	19.33317916	259.06875	25.54	1.30E-04	1198	132.51	5.9604	ATPase	Transport	ATPase activity	Plasma membrane	-																								
48255949			tSPVEGLPGTAPDLEk	Unambiguous	N-Term(iTRAQ4plex); K16(iTRAQ4plex)	HCD	0.48623222	0	1	2	0.41377	0.4302	0.5401	0.56157124	0.15462316	254145.17	244438.58	105157.805	137269.69	2.753236788	18.73263727	2	High	4097	Unique	Used	51634	1	41.816	1	5.3664	-0.4434	1	14	20/30	9.565476	4097	1899.03	MS2	950.019	224	Glioma_grade2_2.raw
48255949			qIFGQNFIPPk	Unambiguous	N-Term(iTRAQ4plex); K11(iTRAQ4plex)	HCD	0.50796294	0	1	2	0.47782	0.5321	0.4863	0.5415349	0.062900217	224010.95	201154.48	107036.11	108932.18	7.602671238	1.241593342	2	High	5091	Unique	Used	60580	1	50.09	1	3.987	2.3007	1	29	13/20	20.74258	5091	1576.91	MS2	788.96	214	Glioma_grade2_3.raw
48255949			qQDGAAAmEMQPLk	Unambiguous	N-Term(iTRAQ4plex); M8(Oxidation); K14(iTRAQ4plex)	HCD	0.4775547	0	1	2	0.45858	0.5009	0.4562	0.49828348	0.050986905	137205.02	125619.71	62918.953	62594.227	6.233851178	0.365883418	2	High	1506	Unique	Used	49191	1	23.367	1	4.5157	0.4416	1	34	21/26	0	1506	1821.91	MS2	911.458	144	Glioma_grade2_2.raw
48255949			eIPDPSSINTk	Unambiguous	N-Term(iTRAQ4plex); K11(iTRAQ4plex)	HCD	0.47428894	0	1	2	0.4662	0.4742	0.4744	0.4825142	0.014040907	128729.79	126561.02	60014.117	61067.492	1.20141573	1.230324922	2	High	2258	Unique	Used	49902	1	28.593	1	3.1617	4.0375	1	39	15/20	30.89409	2258	1488.82	MS2	744.914	236	Glioma_grade2_2.raw
48255949			ikETYGDTEAIcR	Unambiguous	N-Term(iTRAQ4plex); K2(iTRAQ4plex); C12(Methylthio)	HCD	0.41247657	1	1	2	0.41693	0.4131	0.4118	0.40806204	0.008873005	111234.57	112259.445	46377.227	45808.816	0.648514961	0.871991593	3	High	2918	Unique	Used	80262	1	34.184	1	5.1971	-0.1433	1	16	20/48	21.12051	2918	1832.91	MS2	611.642	118	Glioma_grade2_6.raw
48255949			ikETYGDTEAIcR	Unambiguous	N-Term(iTRAQ4plex); K2(iTRAQ4plex); C12(Methylthio)	HCD	0.42354813	1	1	2	0.43619	0.422	0.4251	0.4113177	0.024151795	105177.766	108706.766	45877.38	44713.016	2.33338971	1.817697497	2	High	2909	Unique	Used	80255	1	34.088	1	5.0944	2.6612	1	100	21/24	0	2909	1832.92	MS2	916.962	169	Glioma_grade2_6.raw
48255949			qIFGQNFIPPk	Unambiguous	N-Term(iTRAQ4plex); K11(iTRAQ4plex)	HCD	0.3674352	0	1	2	0.3709	0.3376	0.4003	0.3642792	0.0699188	78155.805	85874.66	28988.213	31282.352	6.654928051	5.383063006	2	High	5241	Unique	Used	68388	1	50.646	1	3.5465	2.4555	1	19	13/20	31.27239	5241	1576.91	MS2	788.96	221	Glioma_grade2_4.raw
48255949			mTVVQAYVGDVHYk	Unambiguous	N-Term(iTRAQ4plex); K14(iTRAQ4plex)	HCD	0.5230878	0	1	2	0.50793	0.5079	0.5382	0.5382426	0.033441956	75977.984	75976.484	38591.75	40893.777	0.001396024	4.095786903	3	High	4377	Unique	Used	74872	1	45.651	1	7.2486	1.1522	1	17	30/52	45.7177	4377	1898.01	MS2	633.342	189	Glioma_grade2_5.raw
48255949			yGDLLPADGLFIQGNDLk	Unambiguous	N-Term(iTRAQ4plex); K18(iTRAQ4plex)	HCD	0.50155735	0	1	2	0.51003	0.5256	0.4783	0.4928263	0.040980193	68459.16	66434.47	34916.234	32740.654	2.122668103	4.547555811	2	High	7481	Unique	Used	54801	1	66.948	1	5.1994	0.1855	1	39	22/34	21.28808	7481	2237.21	MS2	1119.11	125	Glioma_grade2_2.raw
48255949			nESSHGGEFGcTMEELR	Unambiguous	N-Term(iTRAQ4plex); C11(Methylthio)	HCD	0.4690541	0	1	2	0.46856	0.4167	0.528	0.46949434	0.096608113	61997.645	69717.43	29049.56	32731.938	8.288667524	8.429172289	3	High	3822	Unique	Used	59403	1	40.178	1	4.9068	-0.3375	1	14	28/64	16.27284	3822	2072.86	MS2	691.624	13	Glioma_grade2_3.raw
48255949			aGQVVQIPVAEIVVGDIAQVk	Unambiguous	N-Term(iTRAQ4plex); K21(iTRAQ4plex)	HCD	0.41781062	0	1	2	0.39012	0.4095	0.4257	0.4468811	0.057650227	58543.277	55768.547	22838.785	24921.908	3.432768948	6.168212004	2	High	8574	Unique	Used	55793	1	75.888	1	5.514	1.574	1	15	22/40	10.13803	8574	2421.44	MS2	1211.22	26	Glioma_grade2_2.raw
48255949			tEcGLLGFVLDLk	Unambiguous	N-Term(iTRAQ4plex); C3(Methylthio); K13(iTRAQ4plex)	HCD	0.4147617	0	1	2	0.393	0.4342	0.3972	0.43880036	0.057794837	55841.305	50546.46	21945.764	22179.807	7.038442447	0.750101987	2	High	8469	Unique	Used	63675	1	77.259	1	4.9465	-0.1799	1	43	21/24	20.09901	8469	1741.95	MS2	871.477	227	Glioma_grade2_3.raw
48255949			tSIHNFMAHPEFR	Unambiguous	N-Term(iTRAQ4plex)	HCD	0.57831633	0	1	2	0.53756	0.5477	0.6084	0.6198372	0.072182205	55704.72	54676.36	29944.738	33890.44	1.317545234	8.74136402	3	High	2517	Unique	Used	90267	1	31.192	1	5.1847	11.494	1	29	23/48	43.78071	2517	1730.88	MS2	577.63	209	Glioma_grade2_7rep.raw
48255949			tEcGLLGFVLDLk	Unambiguous	N-Term(iTRAQ4plex); C3(Methylthio); K13(iTRAQ4plex)	HCD	0.43751746	0	1	2	0.44041	0.4095	0.4677	0.43483204	0.054497032	54299.965	58401.418	23914.018	25394.809	5.146636451	4.247017913	2	High	8380	Unique	Used	71192	1	77.055	1	5.4399	-0.8807	1	72	22/24	33.47685	8380	1741.95	MS2	871.476	244	Glioma_grade2_4.raw
48255949			mTVVQAYVGDVHYk	Unambiguous	N-Term(iTRAQ4plex); K14(iTRAQ4plex)	HCD	0.55672956	0	1	2	0.54904	0.5354	0.5786	0.56422573	0.033548927	52817.758	54161.652	28999.2	30559.398	1.776559733	3.704676144	2	High	4405	Unique	Used	74899	1	45.847	1	6.8121	-1.6705	1	79	21/26	35.05343	4405	1898.01	MS2	949.506	205	Glioma_grade2_5.raw
48255949			ikETYGDTEAIcR	Unambiguous	N-Term(iTRAQ4plex); K2(iTRAQ4plex); C12(Methylthio)	HCD	0.41692162	1	1	2	0.38417	0.4745	0.3703	0.45737514	0.123283418	52335.336	42375.336	20105.504	19381.426	14.87220688	2.593265488	2	High	2969	Unique	Used	73611	1	34.193	1	4.7949	-2.0008	1	100	19/24	49.79498	2969	1832.91	MS2	916.958	148	Glioma_grade2_5.raw
48255949			nESSHGGEFGcTMEELR	Unambiguous	N-Term(iTRAQ4plex); C11(Methylthio)	HCD	0.46722436	0	1	2	0.40542	0.4446	0.4878	0.53500473	0.119212976	44066.027	40181.793	17865.184	21497.447	6.52021192	13.0499295	2	High	3812	Unique	Used	59394	1	40.094	1	6.2583	0.2873	1	100	25/32	25.72096	3812	2072.86	MS2	1036.93	16	Glioma_grade2_3.raw
48255949			cGIIHPGEDFLcLEGk	Unambiguous	N-Term(iTRAQ4plex); C1(Methylthio); C12(Methylthio); K16(iTRAQ4plex)	HCD	0.46875125	0	1	2	0.42962	0.4696	0.468	0.5115206	0.071234126	41032.63	37540.13	17628.531	19202.549	6.286072764	6.043802145	3	High	6186	Unique	Used	83052	1	63.055	1	6.6814	2.2538	1	29	24/60	29.96367	6186	2111.01	MS2	704.341	110	Glioma_grade2_6.raw
48255949			nESSHGGEFGcTMEELR	Unambiguous	N-Term(iTRAQ4plex); C11(Methylthio)	HCD	0.5696903	0	1	2	0.55534	0.6388	0.5096	0.5861995	0.094753566	40928.45	35582.27	22729.105	20858.309	9.881805142	6.069883099	3	High	3932	Unique	Used	51479	1	40.568	1	4.7413	5.0509	1	18	27/64	15.18486	3932	2072.87	MS2	691.628	19	Glioma_grade2_2.raw
48255949			cGIIHPGEDFLcLEGk	Unambiguous	N-Term(iTRAQ4plex); C1(Methylthio); C12(Methylthio); K16(iTRAQ4plex)	HCD	0.643924	0	1	2	0.60603	0.5941	0.6947	0.6810726	0.079565634	34634.004	35328.113	20989.275	24061.008	1.403071267	9.642750676	3	High	5954	Unique	Used	82890	1	60.391	1	5.9454	-5.7262	1	38	22/60	48.99012	5954	2110.99	MS2	704.335	95	Glioma_grade2_6.raw
48255949			qIFGQNFIPPk	Unambiguous	N-Term(iTRAQ4plex); K11(iTRAQ4plex)	HCD	0.6622862	0	1	2	0.66665	0.6587	0.6659	0.6579782	0.006947961	19548.22	19783.889	13031.747	13017.367	0.847364417	0.078069415	3	High	5082	Unique	Used	60572	1	50.016	1	3.5537	-4.0556	1	55	16/40	16.2491	5082	1576.9	MS2	526.306	209	Glioma_grade2_3.raw
48255949			sVDkDPMLLSGTHVMEGSGR	Unambiguous	N-Term(iTRAQ4plex); K4(iTRAQ4plex)	HCD	0.4347445	1	1	2	0.41254	0.4263	0.443	0.4576831	0.04519129	15805.683	15297.278	6520.535	7001.3057	2.311655299	5.028253619	3	High	2751	Unique	Used	80123	1	32.628	1	7.5391	1.8931	1	40	29/76	11.71461	2751	2404.22	MS2	802.08	108	Glioma_grade2_6.raw
48255949			iEDSQPHIPLIDDTDLEEDAALk	Unambiguous	N-Term(iTRAQ4plex); K23(iTRAQ4plex)	HCD	0.2671245	0	1	2	0.24906	0.2808	0.255	0.2874911	0.070494692	15194.914	13477.462	3784.4434	3874.6506	8.471024205	1.665631022	3	High	6544	Unique	Used	69583	1	61.014	1	8.3506	0.0507	1	25	34/88	7.424792	6544	2865.46	MS2	955.825	107	Glioma_grade2_4.raw
48255949			tSIHNFMAHPEFR	Unambiguous	N-Term(iTRAQ4plex)	HCD	0.33203813	0	1	2	0.31108	0.2811	0.3884	0.35097602	0.140528222	14935.3125	16527.77	4646.0923	5800.851	7.157833292	15.63208843	4	High	2506	Unique	Used	90258	1	31.092	1	4.5043	13.178	1	37	27/72	44.11275	2506	1730.88	MS2	433.475	198	Glioma_grade2_7rep.raw
48255949			sVDkDPmLLSGTHVMEGSGR	Unambiguous	N-Term(iTRAQ4plex); K4(iTRAQ4plex); M7(Oxidation)	HCD	0.59359676	1	1	2	0.44529	0.4925	0.685	0.7576248	0.251988139	9827.245	8885.178	4376.002	6731.6313	7.119783088	29.99165361	4	High	2143	Unique	Used	79579	1	27.704	1	3.7286	5.3126	1	35	21/114	24.87445	2143	2420.23	MS2	605.813	132	Glioma_grade2_6.raw
48255953	NP_068768.2	ATP2B3	plasma membrane calcium-transporting ATPase 3 isoform 3a [Homo sapiens]	4	7	0.480829775	18.13717095	160.32216	16.45	9.51E-05	1173	128.46	5.9224	ATPase,Transport/cargo protein	Transport	Transporter activity	Plasma membrane	-																								
48255953			mTVVQSYLGDTHYk	Unambiguous	N-Term(iTRAQ4plex); K14(iTRAQ4plex)	HCD	0.33895782	0	1	2	0.3523	0.3296	0.3489	0.32647514	0.038813495	92085.44	98423.95	32441.67	32132.973	4.705283455	0.676060236	3	High	4136	Unique	Used	74653	1	43.73	1	7.7459	-2.1941	1	17	33/52	10.89235	4136	1929.99	MS2	644.003	193	Glioma_grade2_5.raw
48255953			iDESSLTGESDHVR	Unambiguous	N-Term(iTRAQ4plex)	HCD	0.4832883	0	1	2	0.50081	0.4754	0.4916	0.46665555	0.031881088	82441.24	86840.35	41287.28	40524.53	3.675107863	1.318503276	2	High	1886	Unique	Used	49551	1	25.974	1	5.7724	0.8757	1	95	22/26	14.34841	1886	1688.82	MS2	844.915	111	Glioma_grade2_2.raw
48255953			iDESSLTGESDHVR	Unambiguous	N-Term(iTRAQ4plex)	HCD	0.48082978	0	1	2	0.49933	0.4799	0.4818	0.46304598	0.030844324	80506.734	83769.234	40199.77	38789.008	2.808610294	2.525825572	2	High	1843	Unique	Used	57561	1	25.251	1	5.6549	2.2491	1	100	21/26	13.24447	1843	1688.82	MS2	844.916	107	Glioma_grade2_3.raw
48255953			qIYGQNFIPPk	Unambiguous	N-Term(iTRAQ4plex); K11(iTRAQ4plex)	HCD	0.5819822	0	1	2	0.56383	0.5843	0.5798	0.60079384	0.026112722	67619.9	65255.6	38126.008	39205.164	2.516359393	1.973534102	2	High	3811	Unique	Used	59393	1	40.086	1	3.3663	2.7971	1	100	20-Dec	12.70135	3811	1592.91	MS2	796.958	255	Glioma_grade2_3.raw
48255953			mTVVQSYLGDTHYk	Unambiguous	N-Term(iTRAQ4plex); K14(iTRAQ4plex)	HCD	0.3759117	0	1	2	0.34308	0.339	0.4132	0.4083579	0.107348347	35867.4	36296.605	12305.289	14822.005	0.841122291	13.12026883	2	High	4143	Unique	Used	74660	1	43.775	1	5.5748	3.189	1	100	22/26	39.03692	4143	1930	MS2	965.506	216	Glioma_grade2_5.raw
48255953			iLDLLVHAISINSAYTTk	Unambiguous	N-Term(iTRAQ4plex); K18(iTRAQ4plex)	HCD	0.5328027	0	1	2	0.53822	0.5445	0.5213	0.52731913	0.019639001	25046.89	24759.639	13480.82	13056.231	0.815624514	2.262721364	3	High	7482	Unique	Used	84058	1	76.27	1	7.0098	1.3942	1	31	23/68	30.09973	7482	2260.32	MS2	754.111	75	Glioma_grade2_6.raw
48255953			mTVVQSYLGDTHYk	Unambiguous	N-Term(iTRAQ4plex); M1(Oxidation); K14(iTRAQ4plex)	HCD	0.46205732	0	1	2	0.47996	0.4274	0.501	0.44611076	0.071319419	19308.893	21683.219	9267.589	9673.116	8.191341863	3.027879814	3	High	2923	Unique	Used	73571	1	33.794	1	7.086	-0.253	1	60	33/52	41.47416	2923	1945.99	MS2	649.336	179	Glioma_grade2_5.raw
48255959	NP_001001396.1	ATP2B4	plasma membrane calcium-transporting ATPase 4 isoform 4a [Homo sapiens]	10	19	0.48650831	17.975432	296.94732	20.85	2.12E-04	1170	129.32	7.5181	ATPase	Transport	ATPase activity	Plasma membrane	Cytoplasm, Nucleus, Basolateral membrane																								
48255959			gIIDSTVGEHR	Unambiguous	N-Term(iTRAQ4plex)	HCD	0.44556823	0	1	2	0.3929	0.4872	0.412	0.5108731	0.126760718	1157085.6	933103.2	454624.4	476697.28	15.15456153	3.351770599	2	High	1328	Unique	Used	64763	1	20.642	1	3.8197	1.7254	1	3	19/20	6.678351	1328	1327.71	MS2	664.359	195	Glioma_grade2_4.raw
48255959			lVPSSSYVAVAPVk	Unambiguous	N-Term(iTRAQ4plex); K14(iTRAQ4plex)	HCD	0.3186013	0	1	1	0.30067	0.32	0.3173	0.33767918	0.047504255	449733.44	422601.78	135223.33	142703.83	4.398534034	3.806401848	2	High	4332	Unique	Used	51856	1	43.515	1	4.6575	4.8475	1	29	17/26	15.75732	4332	1705.02	MS2	853.015	114	Glioma_grade2_2.raw
48255959			lVPSSSYVAVAPVk	Unambiguous	N-Term(iTRAQ4plex); K14(iTRAQ4plex)	HCD	0.45136827	0	1	1	0.42681	0.4413	0.4611	0.47676018	0.048604686	399749.34	386638.06	170616.69	184333.64	2.357890017	5.465186277	2	High	4311	Unique	Used	51836	1	43.365	1	4.8987	-14.483	1	24	15/26	25.8657	4311	1704.99	MS2	852.998	174	Glioma_grade2_2.raw
48255959			qIPSPDVFLPk	Unambiguous	N-Term(iTRAQ4plex); K11(iTRAQ4plex)	HCD	0.46526197	0	1	2	0.42947	0.4912	0.4426	0.50619465	0.079341455	93472.89	81727.9	40144.04	41370.227	9.480507564	2.127345739	2	High	5533	Unique	Used	60991	1	53.515	1	4.3638	-0.9009	1	81	17/20	27.03466	5533	1528.9	MS2	764.952	173	Glioma_grade2_3.raw
48255959			qIPSPDVFLPk	Unambiguous	N-Term(iTRAQ4plex); K11(iTRAQ4plex)	HCD	0.6019336	0	1	2	0.5771	0.5857	0.6179	0.62713456	0.040339596	92135.55	90778.58	53171.78	56930.383	1.049150975	4.827759231	2	High	5604	Unique	Used	68727	1	53.391	1	4.2589	0.0572	1	81	15/20	47.15743	5604	1528.9	MS2	764.953	181	Glioma_grade2_4.raw
48255959			eGDFGcTVMELR	Unambiguous	N-Term(iTRAQ4plex); C6(Methylthio)	HCD	0.62084347	0	1	2	0.56672	0.6207	0.6209	0.68011993	0.074450277	76239.54	69606.695	43206.785	47340.9	6.431608849	6.456842603	2	High	4502	Unique	Used	87032	1	60.089	1	4.6443	1.0656	1	81	21/22	46.80539	4502	1546.68	MS2	773.846	42	Glioma_grade2_1rep.raw
48255959			tEcALLGFVTDLk	Unambiguous	N-Term(iTRAQ4plex); C3(Methylthio); K13(iTRAQ4plex)	HCD	0.37547937	0	1	2	0.3293	0.3967	0.3579	0.43110377	0.11750876	70837.91	58807.71	23327.03	25352.227	13.12290535	5.883534878	3	High	8618	Unique	Used	55835	1	76.187	1	5.0973	1.6257	1	15	20/48	17.17735	8618	1743.93	MS2	581.981	211	Glioma_grade2_2.raw
48255959			qIPSPDVFLPk	Unambiguous	N-Term(iTRAQ4plex); K11(iTRAQ4plex)	HCD	0.5920878	0	1	2	0.5721	0.5717	0.6125	0.6120596	0.039339634	69019.25	69064.195	39486.12	42271.402	0.046031462	4.817885233	2	High	5638	Unique	Used	61086	1	54.367	1	3.6231	-0.1025	1	100	14/20	58.12649	5638	1528.9	MS2	764.952	204	Glioma_grade2_3.raw
48255959			tEcALLGFVTDLk	Unambiguous	N-Term(iTRAQ4plex); C3(Methylthio); K13(iTRAQ4plex)	HCD	0.48423874	0	1	2	0.41496	0.4726	0.4945	0.56313425	0.125912528	68689.5	60314.082	28503.611	33964.926	9.181628558	12.36376921	2	High	5989	Unique	Used	88145	1	76.17	1	3.5941	1.7034	1	50	16/24	52.11655	5989	1743.93	MS2	872.468	288	Glioma_grade2_1rep.raw
48255959			qIPSPDVFLPk	Unambiguous	N-Term(iTRAQ4plex); K11(iTRAQ4plex)	HCD	0.6278863	0	1	2	0.62332	0.6501	0.6066	0.6326478	0.028915514	65477.793	62781.223	40813.676	39718.402	2.973300423	1.923396718	2	High	5686	Unique	Used	68797	1	54.126	1	3.9106	0.6161	1	83	15/20	49.50363	5686	1528.9	MS2	764.953	190	Glioma_grade2_4.raw
48255959			mTVVQAYIGGIHYR	Unambiguous	N-Term(iTRAQ4plex)	HCD	0.52795947	0	1	2	0.52384	0.4626	0.602	0.53159463	0.107621213	58886.61	66681.35	30847.344	35447.445	8.778853318	9.813026515	3	High	5710	Unique	Used	68818	1	54.335	1	4.7913	-0.2705	1	23	24/52	36.9149	5710	1751.94	MS2	584.651	228	Glioma_grade2_4.raw
48255959			dALTQINVHYGGVQNLcSR	Unambiguous	N-Term(iTRAQ4plex); C17(Methylthio)	HCD	0.44298637	0	1	2	0.4034	0.4205	0.4646	0.48424646	0.084827562	58534.008	56153.973	23612.85	27192.363	2.934813	9.963929944	3	High	6071	Unique	Used	53493	1	56.213	1	6.1788	1.0341	1	15	32/72	3.106322	6071	2278.12	MS2	760.045	81	Glioma_grade2_2.raw
48255959			mTVVQAYIGGIHYR	Unambiguous	N-Term(iTRAQ4plex)	HCD	0.57721806	0	1	2	0.57769	0.6181	0.539	0.57671255	0.055961139	44268.047	41370.605	25573.23	23858.947	4.784757439	4.904421402	3	High	5616	Unique	Used	61065	1	54.204	1	3.5506	-0.0615	1	56	20/52	44.74632	5616	1751.94	MS2	584.651	203	Glioma_grade2_3.raw
48255959			dAEGLDEIDHAEMELR	Unambiguous	N-Term(iTRAQ4plex)	HCD	0.55886495	0	1	2	0.51763	0.5939	0.5283	0.60617256	0.080124913	39058.41	34041.445	20217.957	20634.99	9.70598361	1.44365038	2	High	5419	Unique	Used	52878	1	51.473	1	5.3559	-1.4136	1	69	24/30	0	5419	1986.92	MS2	993.962	89	Glioma_grade2_2.raw
48255959			cGILTPGDDFLcLEGk	Unambiguous	N-Term(iTRAQ4plex); C1(Methylthio); C12(Methylthio); K16(iTRAQ4plex)	HCD	0.43490195	0	1	2	0.37093	0.3952	0.4722	0.50305736	0.143492757	33733.723	31664.295	12512.768	15928.956	4.475079266	16.9863803	2	High	8147	Unique	Used	63375	1	74.857	1	5.217	0.9646	1	100	20/30	0	8147	2060.98	MS2	1030.99	66	Glioma_grade2_3.raw
48255959			mTVVQAYIGGIHYR	Unambiguous	N-Term(iTRAQ4plex)	HCD	0.5736741	0	1	2	0.51804	0.5695	0.5774	0.63484335	0.083179265	28541.4	25960.209	14785.493	16480.666	6.697701933	7.667512492	2	High	5722	Unique	Used	68829	1	54.432	1	5.0485	0.7823	1	100	22/26	0	5722	1751.94	MS2	876.474	279	Glioma_grade2_4.raw
48255959			sLDkDPMLLSGTHVMEGSGR	Unambiguous	N-Term(iTRAQ4plex); K4(iTRAQ4plex)	HCD	0.6122224	1	1	2	0.53951	0.573	0.6492	0.6894468	0.112089263	19320.58	18191.678	10423.648	12542.193	4.255964861	13.04578862	4	High	3334	Unique	Used	90953	1	38.515	1	3.6098	2.1943	1	17	28/114	41.32561	3334	2418.24	MS2	605.316	119	Glioma_grade2_7rep.raw
48255959			dALTQINVHYGGVQNLcSR	Unambiguous	N-Term(iTRAQ4plex); C17(Methylthio)	HCD	0.4865083	0	1	2	0.46176	0.5713	0.4179	0.5171284	0.135449381	19041.41	15389.306	8792.583	7958.2466	15.00071915	7.04400845	2	High	5917	Unique	Used	61346	1	56.527	1	6.2983	-1.2909	1	90	25/36	22.6796	5917	2278.12	MS2	1139.56	157	Glioma_grade2_3.raw
48255959			cGILTPGDDFLcLEGk	Unambiguous	N-Term(iTRAQ4plex); C1(Methylthio); C12(Methylthio); K16(iTRAQ4plex)	HCD	0.4086574	0	1	2	0.39491	0.3681	0.4522	0.4214716	0.088047146	12689.475	13614.3125	5011.1914	5738.046	4.972355162	9.562795898	3	High	8493	Unique	Used	55718	1	75.271	1	4.0048	-2.4476	1	45	16/60	35.17114	8493	2060.97	MS2	687.662	89	Glioma_grade2_2.raw
48375173	NP_149052.1	MAP6	microtubule-associated protein 6 isoform 1 [Homo sapiens]	8	8	0.499171019	19.00223373	36.793984	14.51	5.05E-04	813	86.452	9.1587	Cytoskeletal associated protein	Cell growth and/or maintenance	Cytoskeletal protein binding	Cytoskeleton	Axon, Synaptic vesicle																								
48375173			fWNQLDk	Unambiguous	N-Term(iTRAQ4plex); K7(iTRAQ4plex)	HCD	0.45154428	0	1	2	0.43341	0.4572	0.4462	0.47067374	0.035108809	263788.06	250063.53	114328.4	117698.34	3.777241686	2.053993799	2	High	4166	Unique	Used	67398	1	42.16	1	3.006	10.122	1	49	12-Dec	65.03989	4166	1238.69	MS2	619.849	304	Glioma_grade2_4.raw

48375173			dQGPIVPAPVk	Unambiguous	N-Term(iTRAQ4plex); K11(iTRAQ4plex)	HCD	0.5574816	0	1	1	0.54781	0.5798	0.5364	0.56772095	0.03495229	222341.78	210071.19	121800.45	119261.82	4.013116164	1.4893102	2	High	1756	Unique	Used	57478	1	24.657	1	3.7504	-0.5339	1	42	15/20	33.83573	1756	1408.84	MS2	704.923	209	Glioma_grade 2_3.raw
48375173			dLGPmIPLPVk	Unambiguous	N-Term(iTRAQ4plex); M5(Oxidation); K11(iTRAQ4plex)	HCD	0.43561345	0	1	1	0.40014	0.4311	0.4398	0.47383288	0.069479113	95580.46	88716.91	38245.42	42036.99	5.266773745	6.67903432	2	High	5037	Unique	Used	68202	1	49.01	1	4.1878	-0.5111	1	79	15/20	26.29059	5037	1483.88	MS2	742.443	179	Glioma_grade 2_4.raw
48375173			sLYSEPFkEPPk	Unambiguous	N-Term(iTRAQ4plex); K8(iTRAQ4plex); K12(iTRAQ4plex)	HCD	0.29703176	1	1	2	0.27834	0.3005	0.2938	0.31720948	0.054128607	92941.875	86095.445	25869.48	27310.293	5.407986536	3.831564466	3	High	2183	Unique	Used	79616	1	28.01	1	3.3009	0.9748	1	58	18/44	48.9926	2183	1854.04	MS2	618.685	134	Glioma_grade 2_6.raw
48375173			tQGPVATEPDkDQGSVVPG LLk	Unambiguous	N-Term(iTRAQ4plex); K11(iTRAQ4plex); K22(iTRAQ4plex)	HCD	0.50881845	1	1	1	0.45895	0.5286	0.4916	0.5662581	0.090666781	86707.56	75277.984	39794.47	42626.766	9.978582651	4.859756551	3	High	3296	Unique	Used	80607	1	37.139	1	5.652	2.0563	1	22	26/84	14.95508	3296	2668.49	MS2	890.169	56	Glioma_grade 2_6.raw
48375173			aDIAVPLVFTk	Unambiguous	N-Term(iTRAQ4plex); K11(iTRAQ4plex)	HCD	0.48952362	0	1	2	0.39676	0.4667	0.509	0.5986292	0.171275683	77281.99	65706.65	30662.4	39333.918	11.44846389	17.5200335	2	High	5865	Unique	Used	61297	1	56.133	1	3.9732	1.4456	1	43	16/20	43.6491	5865	1461.89	MS2	731.451	132	Glioma_grade 2_3.raw
48375173			dQDVVVPEHAk	Unambiguous	N-Term(iTRAQ4plex); K11(iTRAQ4plex)	HCD	0.5573312	0	1	1	0.52575	0.544	0.5702	0.590004	0.050818745	13102.16	12662.602	6888.5186	7470.9863	2.412709596	5.736505031	3	High	1145	Unique	Used	64589	1	19.417	1	3.8221	-0.8562	1	50	20/40	48.24252	1145	1524.82	MS2	508.946	175	Glioma_grade 2_4.raw
48375173			nQAPMVPAPVkDEGPMVSA SVk	Unambiguous	N-Term(iTRAQ4plex); K11(iTRAQ4plex); K22(iTRAQ4plex)	HCD	0.5823829	1	1	1	0.63032	0.6748	0.496	0.5310642	0.143250207	5271.4526	4923.821	3322.688	2614.865	4.822090536	16.85901391	4	High	3230	Unique	Used	80547	1	36.615	1	4.9175	-0.8289	1	91	24/126	47.35551	3230	2684.45	MS2	671.867	92	Glioma_grade 2_6.raw
49533617	NP_003092.4	SOAT1	sterol O-acyltransferase 1 isoform 1 [Homo sapiens]	2	2	4.358660221	24.18561319	10.11039	5.45	9.51E-05	550	64.692	8.939	Enzyme: Acyltransferase	Metabolism,Energy pathways	Acyltransferase activity	Endoplasmic reticulum	Cytoplasm, Nucleus																								
49533617			ITAEAEELkPFFMk	Unambiguous	N-Term(iTRAQ4plex); K9(iTRAQ4plex); K14(iTRAQ4plex)	HCD	5.10407	0	1	1	4.98367	4.8153	5.4029	5.2204027	5.06599391	9251.205	9574.626	46104.957	49983.406	2.429567994	5.708240837	3	High	5305	Unique	Used	92451	1	58.664	1	4.1023	-1.3069	1	58	25/52	14.53176	5305	2086.16	MS2	696.058	121	Glioma_grade 2_7rep.raw
49533617			eVGSHFDDFVTNLIEk	Unambiguous	N-Term(iTRAQ4plex); K16(iTRAQ4plex)	HCD	3.6132505	0	1	3	3.30002	3.3237	3.9008	3.928722	9.642359634	4139.6265	4110.1787	13660.863	16147.749	0.504805594	11.79856314	3	High	6445	Unique	Used	76709	1	62.74	1	6.0081	-4.6878	1	43	26/60	24.1763	6445	2138.09	MS2	713.368	194	Glioma_grade 2_5.raw
51477708	NP_001003810 .1	HNRNPD	heterogeneous nuclear ribonucleoprotein D0 isoform d [Homo sapiens]	7	21	3.338884115	23.8954704	126.38553	27.87	1.00E-04	287	30.653	8.4116	RNA binding protein	Regulation of nucleobase, nucleoside, nucleotide and nucleic acid metabolism	MRNA binding	Cytoplasm, Nucleus	Mitochondrion, Cytoplasm																								
51477708			fGEVVDcTLk	Unambiguous	N-Term(iTRAQ4plex); C7(Methylthio); K10(iTRAQ4plex)	HCD	2.9290361	0	1	4	2.73882	3.1334	2.7504	3.146659	7.762693833	342337.25	299228.2	937599.4	941569.1	9.502600736	0.298749345	2	High	5782	Unique	Used	53219	1	54.132	1	3.9231	-2.4568	1	10	18/18	7.770513	5782	1444.74	MS2	722.873	145	Glioma_grade 2_2.raw
51477708			mFIGGLSWDTTk	Unambiguous	N-Term(iTRAQ4plex); K12(iTRAQ4plex)	HCD	3.6690626	0	1	4	3.35752	3.846	3.5146	4.025927	8.264916228	178258.48	155617.95	598507	626506.5	9.589938585	3.232394797	2	High	6293	Unique	Used	61698	1	59.437	1	4.5329	0.9993	1	18	19/22	12.52243	6293	1643.87	MS2	822.44	199	Glioma_grade 2_3.raw
51477708			iFVGGLSPDTPEEk	Unambiguous	N-Term(iTRAQ4plex); K14(iTRAQ4plex)	HCD	4.1500096	0	1	4	4.00695	4.1949	4.1071	4.299777	3.006049058	176750.12	168829.25	708229.6	725928.1	3.241455582	1.745237552	2	High	4548	Unique	Used	52059	1	45.124	1	5.1534	1.4823	1	9	21/26	6.523744	4548	1776.96	MS2	888.986	244	Glioma_grade 2_2.raw
51477708			mFIGGLSWDTTk	Unambiguous	N-Term(iTRAQ4plex); K12(iTRAQ4plex)	HCD	2.9091673	0	1	4	2.71962	3.0785	2.7595	3.1237319	7.197453523	161194.5	142401.08	438387.5	444822.8	8.754379575	1.03043279	2	High	6476	Unique	Used	53874	1	59.18	1	4.6726	-1.5998	1	16	18/22	39.29456	6476	1643.87	MS2	822.438	211	Glioma_grade 2_2.raw
51477708			gFcFITFk	Unambiguous	N-Term(iTRAQ4plex); C3(Methylthio); K8(iTRAQ4plex)	HCD	5.0440335	0	1	4	4.96575	4.8776	5.2135	5.120922	2.995144417	158138.62	160998.33	785277.6	824459.94	1.267243002	3.442312505	2	High	7245	Unique	Used	70211	1	66.931	1	3.0875	1.0051	1	10	13/14	7.585616	7245	1296.67	MS2	648.84	165	Glioma_grade 2_4.raw
51477708			mFIGGLSWDTTk	Unambiguous	N-Term(iTRAQ4plex); M1(Oxidation); K12(iTRAQ4plex)	HCD	3.338884	0	1	4	3.12508	3.3976	3.2849	3.571334	5.619266506	119714.875	110112.41	374118.53	393248.16	5.908757194	3.525482998	2	High	5502	Unique	Used	68631	1	52.643	1	4.4577	0.6702	1	20	20/22	25.41727	5502	1659.87	MS2	830.437	194	Glioma_grade 2_4.raw
51477708			mFIGGLSWDTTk	Unambiguous	N-Term(iTRAQ4plex); K12(iTRAQ4plex)	HCD	3.8815608	0	1	4	3.57047	3.9062	3.8591	4.221906	6.858606131	115868.11	105910.23	413703.06	447143	6.349839641	5.493574156	2	High	6354	Unique	Used	69412	1	59.435	1	4.4234	0.1824	1	17	19/22	9.739392	6354	1643.87	MS2	822.439	193	Glioma_grade 2_4.raw
51477708			iFVGGLSPDTPEEk	Unambiguous	N-Term(iTRAQ4plex); K14(iTRAQ4plex)	HCD	4.057941	0	1	4	3.64342	4.2484	3.8946	4.541297	9.656723612	94296.58	80867.89	343561.97	367245.12	10.84183084	4.711972123	2	High	4489	Unique	Used	60023	1	45.36	1	5.1972	-1.3342	1	16	22/26	3.466222	4489	1776.96	MS2	888.984	237	Glioma_grade 2_3.raw
51477708			iFVGGLSPDTPEEk	Unambiguous	N-Term(iTRAQ4plex); K14(iTRAQ4plex)	HCD	3.754844	0	1	4	3.36938	3.6102	3.8898	4.1678686	9.191814911	78098.63	72887.98	263143.66	303787.53	4.880546625	10.13863996	2	High	4641	Unique	Used	52145	1	45.838	1	5.1297	1.9632	1	17	21/26	3.215798	4641	1776.97	MS2	888.987	241	Glioma_grade 2_2.raw
51477708			gFcFITFk	Unambiguous	N-Term(iTRAQ4plex); C3(Methylthio); K8(iTRAQ4plex)	HCD	3.7929337	0	1	4	3.2879	4.2208	3.4596	4.4412622	14.6488244	67489.266	52572.41	221898.11	233487.86	17.57065266	3.599228504	2	High	7142	Unique	Used	62489	1	66.053	1	3.1349	-0.3129	1	40	13/14	12.59353	7142	1296.67	MS2	648.84	166	Glioma_grade 2_3.raw
51477708			fGEVVDcTLk	Unambiguous	N-Term(iTRAQ4plex); C7(Methylthio); K10(iTRAQ4plex)	HCD	2.3410854	0	1	4	2.30402	2.1262	2.5739	2.3752887	7.897133437	66120.41	71649.836	152342.83	170189.06	5.675963765	7.825080647	2	High	6032	Unique	Used	53456	1	55.938	1	3.2629	-2.2033	1	28	16/18	17.70086	6032	1444.74	MS2	722.873	174	Glioma_grade 2_2.raw
51477708			mFIGGLSWDTTk	Unambiguous	N-Term(iTRAQ4plex); M1(Oxidation); K12(iTRAQ4plex)	HCD	3.1038826	0	1	4	2.95606	3.082	3.1249	3.2580025	4.009996056	46979.645	45059.8	138874.69	146804.94	2.949899184	3.92574966	2	High	5308	Unique	Used	60785	1	51.712	1	4.6143	4.0531	1	100	20/22	53.6153	5308	1659.87	MS2	830.44	214	Glioma_grade 2_3.raw
51477708			gFcFITFk	Unambiguous	N-Term(iTRAQ4plex); C3(Methylthio); K8(iTRAQ4plex)	HCD	4.1927743	0	1	4	4.22919	4.0433	4.3491	4.1579623	3.055873779	46591.67	48733.527	197044.88	202632.16	3.177589256	1.976998016	2	High	6800	Unique	Used	77013	1	65.897	1	3.4249	-2.4781	1	43	13/14	24.46782	6800	1296.67	MS2	648.838	161	Glioma_grade 2_5.raw
51477708			kYHNVGLSk	Unambiguous	N-Term(iTRAQ4plex); K1(iTRAQ4plex); K9(iTRAQ4plex)	HCD	3.2715461	1	1	4	3.09164	3.526	3.0484	3.4767325	7.622953417	44046.24	38620.105	136174.98	134271.78	9.282754316	0.995216675	3	High	984	Unique	Used	94059	1	18.606	1	3.6418	-2.096	1	21	17/32	12.15321	984	1477.88	MS2	493.299	158	Glioma_grade 2_8rep.raw
51477708			fGEVVDcTLk	Unambiguous	N-Term(iTRAQ4plex); C7(Methylthio); K10(iTRAQ4plex)	HCD	1.8335707	0	1	4	1.74396	1.8895	1.782	1.9306587	4.788890278	39889.758	36818.117	69566.09	71083.22	5.662986181	1.525457766	2	High	5900	Unique	Used	53331	1	54.984	1	3.6058	-0.8514	1	100	17/18	20.53395	5900	1444.74	MS2	722.874	184	Glioma_grade 2_2.raw
51477708			iFVGGLSPDTPEEk	Unambiguous	N-Term(iTRAQ4plex); K14(iTRAQ4plex)	HCD	4.6102767	0	1	4	4.90398	4.2403	5.0382	4.3563223	8.525168398	38777.184	44846.535	190162.53	195365.95	10.26426306	1.908742808	2	High	4403	Unique	Used	59945	1	44.646	1	5.1619	-1.2655	1	34	22/26	14.84196	4403	1776.96	MS2	888.984	239	Glioma_grade 2_3.raw
51477708			kYHNVGLSk	Unambiguous	N-Term(iTRAQ4plex); K1(iTRAQ4plex); K9(iTRAQ4plex)	HCD	3.7063165	1	1	4	3.5816	3.685	3.7271	3.834638	2.825147578	35070.027	34086.14	125606.64	130708.02	2.012006159	2.814679129	2	High	992	Unique	Used	94066	1	18.673	1	3.8067	-0.2078	1	84	15/16	26.25956	992	1477.88	MS2	739.446	166	Glioma_grade 2_8rep.raw
51477708			mFIGGLSWDTTk	Unambiguous	N-Term(iTRAQ4plex); M1(Oxidation); K12(iTRAQ4plex)	HCD	2.5631385	0	1	4	2.34552	2.6533	2.4834	2.8093154	7.839966579	33093.31	29254.398	77620.97	82184.83	8.707684034	4.038822564	2	High	5404	Unique	Used	60875	1	52.441	1	4.2421	1.4792	1	74	19/22	15.05679	5404	1659.87	MS2	830.438	212	Glioma_grade 2_3.raw
51477708			yHNVGLSk	Unambiguous	N-Term(iTRAQ4plex); K8(iTRAQ4plex)	HCD	2.0777922	0	1	4	1.84654	2.0228	2.128	2.3311167	9.730029142	20741.43	18934.379	38299.836	44138.246	6.441093695	10.01570925	3	High	1207	Unique	Used	94243	1	20.791	1	3.3504	1.4902	1	100	15/28	53.81853	1207	1205.69	MS2	402.568	333	Glioma_grade 2_8rep.raw
51477708			kYHNVGLSk	Unambiguous	N-Term(iTRAQ4plex); K1(iTRAQ4plex); K9(iTRAQ4plex)	HCD	3.0181987	1	1	4	2.55032	3.0209	3.0159	3.5724196	13.75093118	17353.729	14650.289	44257.508	52336.984	11.94612974	11.82894004	4	High	976	Unique	Used	94052	1	18.534	1	4.2585	0.187	1	38	17/48	35.98895	976	1477.89	MS2	370.227	175	Glioma_grade 2_8rep.raw
51477708			eYFGGFGEVESIELPMDNk	Unambiguous	N-Term(iTRAQ4plex); K19(iTRAQ4plex)	HCD	3.2428293	0	1	4	3.0683	3.1938	3.2899	3.4244967	4.643589799	8656.77	8316.628	26561.566	28480.264	2.834043186	4.92979382	2	High	7640	Unique	Used	54945	1	68.235	1	5.7263	-4.4882	1	75	21/36	6.023501	7640	2449.17	MS2	1225.09	128	Glioma_grade 2_2.raw
52426787	NP_002535.3	OMG	oligodendrocyte-myelin glycoprotein precursor [Homo sapiens]	3	8	0.489998996	14.83941758	39.053164	10.68	9.95E-05	440	49.576	7.9429	Adhesion molecule	Cell communication,Signal transduction	Cell adhesion molecule activity	Plasma membrane	-																								
52426787			IESLPAHLPR	Unambiguous	N-Term(iTRAQ4plex)	HCD	0.45812035	0	1	1	0.43899	0.4345	0.482	0.47705755	0.054225645	1418942.2	1433576.5	622898.4	683898.5	0.72553514	6.601421286	2	High	2507	Unique	Used	73181	1	30.759	1	3.1294	12.431	1	3	16/18	13.16386	2507	1276.77	MS2	638.886	248	Glioma_grade 2_5.raw
52426787			IESLPAHLPR	Unambiguous	N-Term(iTRAQ4plex)	HCD	0.40600422	0	1	1	0.37475	0.4294	0.3856	0.44181302	0.080193472	1086885.5	948541.3	407313.97	419077.9	9.612148367	2.013174374	3	High	2488	Unique	Used	73163	1	30.619	1	3.519	12.134	1	1	20/36	8.17106	2488	1276.76	MS2	426.26	206	Glioma_grade 2_5.raw
52426787			IWTVPTNMPSk	Unambiguous	N-Term(iTRAQ4plex); K11(iTRAQ4plex)	HCD	0.5328414	0	1	1	0.50173	0.51	0.5554	0.564466	0.059327382	318817.44	313669.47	159959.45	177055.75	1.151063979	7.17410352	2	High	4970	Unique	Used	60466	1	49.175	1	4.1207	0.8934	1	25	14/20	33.20993	4970	1561.87	MS2	781.437	253	Glioma_grade 2_3.raw
52426787			IWTVPTNMPSk	Unambiguous	N-Term(iTRAQ4plex); K11(iTRAQ4plex)	HCD	0.59005874	0	1	1	0.56128	0.577	0.6027	0.6196458	0.044111999	270044.53	262667.78	151570.58	162760.97	1.958336554	5.034684335	2	High	5126	Unique	Used	52601	1	49.363	1	4.0377	-1.5295	1	26	14/20	35.0924	5126	1561.86	MS2	781.435	259	Glioma_grade 2_2.raw
52426787			IWTVPTNmPSk	Unambiguous	N-Term(iTRAQ4plex); M8(Oxidation); K11(iTRAQ4plex)	HCD	0.55243665	0	1	1	0.54389	0.5699	0.5358	0.5613968	0.028355324	136088.42	129872.35	74016.55	72909.92	3.305318487	1.065166239	2	High	3336	Unique	Used	58954	1	36.364	1	3.7157	0.7804	1	76	14/20	18.74953	3336	1577.86	MS2	789.434	249	Glioma_grade 2_3.raw
52426787			aHVIGTPcSTQISSLk	Unambiguous	N-Term(iTRAQ4plex); C8(Methylthio); K16(iTRAQ4plex)	HCD	0.46409336	0	1	1	0.46519	0.4904	0.4391	0.4629326	0.04514749	123067.266	116737.05	57250.207	54041.383	3.733159381	4.077542984	3	High	3628	Unique	Used	80900	1	39.894	1	6.5703	0.3729	1	6	27/60	17.70791	3628	1976.06	MS2	659.357	207	Glioma_grade 2_6.raw
52426787			IESLPAHLPR	Unambiguous	N-Term(iTRAQ4plex)	HCD	0.5159046	0	1	1	0.49596	0.5825	0.4592	0.53932226	0.102772806	70537.32	60062.25	34984.01	32392.91	11.34305883	5.438611265	3	High	3009	Unique	Used	66324	1	33.183	1	3.6735	2.5254	1	17	20/36	4.34626	3009	1276.75	MS2	426.256	230	Glioma_grade 2_4.raw
52426787			aHVIGTPcSTQISSLk	Unambiguous	N-Term(iTRAQ4plex); C8(Methylthio); K16(iTRAQ4plex)	HCD	0.3825188	0	1	1	0.37011	0.408	0.3594	0.39620242	0.058808155	19815.053	17972.89	7333.696	7120.903	6.894294031	2.081930786	2	High	3604	Unique	Used	80879	1	39.687	1	2.9792	-1.5662	1	97	15/30	0	3604	1976.05	MS2	988.53	203	Glioma_grade 2_6.raw
55770844	NP_001894.2	CTNNA1	catenin alpha-1 [Homo sapiens]	5	6	3.026203632	27.63813686	62.469306	16.67	1.04E-04	906	100.01	6.2905	Cytoskeletal protein	Cell growth and/or maintenance	Structural constituent of cytoskeleton	Cytoplasm	Cell junction, Plasma membrane																								
55770844			aAAGEFADDPcSSVk	Unambiguous	N-Term(iTRAQ4plex); C11(Methylthio); K15(iTRAQ4plex)	HCD	2.7184126	0	1	1	2.45166	2.7117	2.7245	3.0134544	8.423309174	38982.273	35244.3	95571.37	106207.09	7.121832383	7.454303829	2	High	4699	Unique	Used	52200	1	46.245	1	5.254	1.3225	1	65	20/28	30.87399	4699	1801.84	MS2	901.422	64	Glioma_grade 2_2.raw
55770844			ILILADMADVYk	Unambiguous	N-Term(iTRAQ4plex); K12(iTRAQ4plex)	HCD	3.6381507	0	1	1	3.35777	3.7068	3.576	3.9476697	6.765953748	31160.225	28226.482	104628.96	111428.83	6.986309477	4.450877877	2	High	7681	Unique	Used	62966	1	70.704	1	5.4257	1.5893	1	71	20/22	15.85859	7681	1652.96	MS2	826.982	176	Glioma_grade 2_3.raw
55770844			aHVLAASVEQATENFLEk	Unambiguous	N-Term(iTRAQ4plex); K18(iTRAQ4plex)	HCD	1.5262069	0	1	1	1.53711	1.4762	1.5783	1.5157317	2.789050374	23467.56	24436.014	36072.324	37038.44	2.859078492	1.868800537	3	High	6255	Unique	Used	76540	1	61.158	1	6.6946	0.4407	1	12	30/68	31.25286	6255	2245.21	MS2	749.074	181	Glioma_grade 2_5.raw
55770844			ILILADMADVYk	Unambiguous	N-Term(iTRAQ4plex); K12(iTRAQ4plex)	HCD	3.279731	0	1	1	3.05148	3.2653	3.2932	3.523977	5.886928941	21208.15	19819.547	64716.188	69843.625	4.786476792	5.388897906	2	High	8026	Unique	Used	55298	1	71.347	1	5.2974	-1.9555	1	100	19/22	46.52145	8026	1652.95	MS2	826.979	221	Glioma_grade 2_2.raw
55770844			mSASQLEALcPQVINAALAL AAkPQSk	Unambiguous	N-Term(iTRAQ4plex); C10(Methylthio); K23(iTRAQ4plex); K27(iTRAQ4plex)	HCD	3.7366328	0	1	1	3.01558	4.2213	3.3904	4.7459908	20.42117191	2827.61	2019.9673	8526.87	9586.747	23.56185759	8.274948222	3	High	7534	Unique	Used	84104	1	76.689	1	9.6743	-3.2041	1	97	40/104	27.64908	7534	3231.75	MS2	1077.92	72	Glioma_grade 2_6.raw
55770844			nVPILYTASQAcLQHPDVAA Yk	Unambiguous	N-Term(iTRAQ4plex); C12(Methylthio); K22(iTRAQ4plex)	HCD	2.7726765	0	1	1	2.93192	2.1564	3.6105	2.655553	21.36536878	2440.5522	3318.2156	7155.503	8811.697	21.55328234	14.66889634	3	High	6962	Unique	Used	69960	1	64.502	1	5.2738	-0.2693	1	100	30/84	33.57846	6962	2736.41	MS2	912.808	114	Glioma_grade 2_4.raw
55770878	NP_002513.2	NPTX1	neuronal pentraxin-1 precursor [Homo sapiens]	5	11	0.372553796	27.92302763	64.250815	12.5	9.90E-05	432	47.093	6.5513	Transport/cargo protein	Cell communication,Signal transduction	Transporter activity	Extracellular	Plasma membrane																								
55770878			iETALTSLHQR	Unambiguous	N-Term(iTRAQ4plex)	HCD	0.3650834	0	1	1	0.36021	0.3596	0.3705	0.36995238	0.016331564	599592.6	600523.06	215977.38	222164.94	0.109645195	1.99718924	2	High	2573	Unique	Used	65917	1	29.909	1	4.4496	14.907	1	22	17/20	33.79318	2573	1412.82	MS2	706.913	239	Glioma_grade 2_4.raw
55770878			IPFVINDGk	Unambiguous	N-Term(iTRAQ4plex); K9(iTRAQ4plex)	HCD	0.50165963	0	1	1	0.48076	0.4815	0.5218	0.5225884	0.047247715	491283.16	490521	236190.95	256340.58	0.1097833	5.785595091	2	High	4995	Unique	Used	68163	1	48.689	1	3.2768	-6.0515	1	35	16-Nov	61.73255	4995	1290.76	MS2	645.883	317	Glioma_grade 2_4.raw

55770878			iETALTSLHQR	Unambiguous	N-Term(iTRAQ4plex)	HCD	0.296922	0	1	1	0.29788	0.3168	0.2782	0.29589903	0.053152087	340961.66	320568.03	101566.94	94855.77	4.359736013	4.831940071	2	High	2671	Unique	Used	66007	1	30.674	1	4.4024	13.179	1	20	16/20	7.344769	2671	1412.82	MS2	706.912	181	Glioma_grade2_4.raw
55770878			iETALTSLHQR	Unambiguous	N-Term(iTRAQ4plex)	HCD	0.55516243	0	1	1	0.49766	0.5201	0.5887	0.6152619	0.100095591	211807.81	202666.23	105407.62	124692.81	3.119169157	11.85281455	3	High	2570	Unique	Used	65914	1	29.891	1	4.7335	12.176	1	6	21/40	17.39673	2570	1412.82	MS2	471.61	148	Glioma_grade2_4.raw
55770878			iETALTSLHQR	Unambiguous	N-Term(iTRAQ4plex)	HCD	0.4184593	0	1	1	0.40049	0.3979	0.4392	0.43630916	0.053372147	173959.48	175113.45	69669.21	76403.61	0.467512627	6.519953414	3	High	2334	Unique	Used	58016	1	28.937	1	4.5027	-0.8492	1	5	23/40	17.27527	2334	1412.8	MS2	471.604	183	Glioma_grade2_3.raw
55770878			iETALTSLHQR	Unambiguous	N-Term(iTRAQ4plex)	HCD	0.3167768	0	1	1	0.29505	0.2867	0.3477	0.33788612	0.096035407	148170.39	152477.25	43718.254	51519.945	2.025899762	11.58490746	2	High	2710	Unique	Used	66044	1	30.942	1	4.0055	-2.1142	1	34	17/20	11.55531	2710	1412.79	MS2	706.901	256	Glioma_grade2_4.raw
55770878			iETALTSLHQR	Unambiguous	N-Term(iTRAQ4plex)	HCD	0.5622177	0	1	1	0.52658	0.4863	0.6444	0.5951242	0.125053727	109393.68	118459.49	57604.984	70498.11	5.626865519	14.23356227	3	High	2700	Unique	Used	66034	1	30.884	1	4.5708	-1.8213	1	7	23/40	11.31047	2700	1412.8	MS2	471.603	179	Glioma_grade2_4.raw
55770878			lTPGEVYNLATcSTk	Unambiguous	N-Term(iTRAQ4plex); C12(Methylthio); K15(iTRAQ4plex)	HCD	0.29146567	0	1	1	0.27043	0.2739	0.3088	0.31277943	0.076800768	56908.297	56177.965	15389.449	17571.312	0.91332528	9.361495767	2	High	6476	Unique	Used	61870	1	60.851	1	5.8515	3.2639	1	100	22/28	31.13631	6476	1930.99	MS2	966	159	Glioma_grade2_3.raw
55770878			tPAAETLSQLGQTLQSLk	Unambiguous	N-Term(iTRAQ4plex); K18(iTRAQ4plex)	HCD	0.2794148	0	1	1	0.25473	0.2682	0.29	0.30540383	0.080582156	47235.83	44860.758	12032.502	13700.646	3.647104748	9.167599195	2	High	8240	Unique	Used	55489	1	73.152	1	7.1279	0.8804	1	53	24/34	9.552285	8240	2174.23	MS2	1087.62	146	Glioma_grade2_2.raw
55770878			kLTPGEVYNLATcSTk	Unambiguous	N-Term(iTRAQ4plex); K1(iTRAQ4plex); C13(Methylthio); K16(iTRAQ4plex)	HCD	0.5617613	1	1	1	0.48627	0.588	0.54	0.6530486	0.125116655	40966.594	33877.934	19920.824	22123.936	13.39427127	7.410366642	3	High	4635	Unique	Used	91943	1	51.826	1	5.6194	-4.2704	1	26	23/60	31.03492	4635	2203.17	MS2	735.063	172	Glioma_grade2_7rep.raw
55770878			lTPGEVYNLATcSTk	Unambiguous	N-Term(iTRAQ4plex); C12(Methylthio); K15(iTRAQ4plex)	HCD	0.3725538	0	1	1	0.35303	0.3563	0.3887	0.392257	0.055761615	33030.758	32730.133	11660.847	12838.624	0.646505766	6.798629272	2	High	6656	Unique	Used	54043	1	60.501	1	4.6313	-2.2991	1	100	21/28	38.2188	6656	1930.98	MS2	965.994	170	Glioma_grade2_2.raw
55956788	NP_005372.2	NCL	nucleolin [Homo sapiens]	9	25	2.122485161	37.15097276	144.18938	18.31	1.05E-04	710	76.568	4.7036	RNA binding protein	Regulation of nucleobase, nucleoside, nucleotide and nucleic acid metabolism	RNA binding	Nucleolus	Cytoplasm, Nucleus, Plasma membrane																									
55956788			gIAYIEFk	Unambiguous	N-Term(iTRAQ4plex); K8(iTRAQ4plex)	HCD	3.1316593	0	1	1	3.0197	3.3404	2.9429	3.2555118	6.010531243	859306.1	776796.3	2594845.8	2528869.5	7.131978915	1.821033621	2	High	4537	Unique	Used	60066	1	45.762	1	3.0125	0.2458	1	3	14/14	10.39858	4537	1228.72	MS2	614.863	154	Glioma_grade2_3.raw
55956788			tGISDVFAk	Unambiguous	N-Term(iTRAQ4plex); K9(iTRAQ4plex)	HCD	2.9529555	0	1	1	2.88107	2.7836	3.1283	3.0224075	5.149228718	642360.3	664856.1	1850685.6	2009466.1	2.433710704	5.817116891	2	High	2873	Unique	Used	50483	1	32.907	1	3.6204	-0.7353	1	4	16/16	17.28908	2873	1225.7	MS2	613.355	268	Glioma_grade2_2.raw
55956788			sISLYYTGEk	Unambiguous	N-Term(iTRAQ4plex); K10(iTRAQ4plex)	HCD	1.9951675	0	1	1	1.94753	2.05	1.9431	2.0453126	2.963367967	283729.72	269544.97	552571.94	551303.75	3.625733508	0.162472234	2	High	3201	Unique	Used	50788	1	35.34	1	4.4054	0.1657	1	17	16/18	37.88245	3201	1448.79	MS2	724.898	225	Glioma_grade2_2.raw
55956788			aLELTGLk	Unambiguous	N-Term(iTRAQ4plex); K8(iTRAQ4plex)	HCD	1.546894	0	1	1	1.44296	1.7	1.4169	1.6693512	9.504119148	252921.34	214672.67	364954.25	358364.1	11.56810966	1.288489295	2	High	3325	Unique	Used	50906	1	36.204	1	3.0754	0.6889	1	9	14/14	21.12246	3325	1132.72	MS2	566.863	225	Glioma_grade2_2.raw
55956788			aLELTGLk	Unambiguous	N-Term(iTRAQ4plex); K8(iTRAQ4plex)	HCD	3.2601402	0	1	1	2.87276	3.5088	3.0565	3.733292	12.05789759	232759.61	190566.69	668661.94	711441.1	14.09546246	4.383648648	2	High	3389	Unique	Used	59003	1	36.783	1	3.2632	0.3656	1	11	14/14	17.14159	3389	1132.72	MS2	566.863	159	Glioma_grade2_3.raw
55956788			aLELTGLk	Unambiguous	N-Term(iTRAQ4plex); K8(iTRAQ4plex)	HCD	2.505903	0	1	1	2.42429	2.5234	2.4891	2.590851	2.769761979	221751.4	213038.58	537590.25	551951.25	2.833963241	1.8640429	2	High	3295	Unique	Used	58915	1	36.073	1	3.4782	-0.4966	1	13	14/14	41.20895	3295	1132.72	MS2	566.862	184	Glioma_grade2_3.raw
55956788			vTLDWAkPk	Unambiguous	N-Term(iTRAQ4plex); K7(iTRAQ4plex); K9(iTRAQ4plex)	HCD	1.6668172	0	1	1	1.5968	1.6433	1.6897	1.7388731	3.660686142	149232.73	145013.89	238294.58	252160.77	2.027666705	3.998275063	3	High	2681	Unique	Used	90409	1	32.597	1	3.671	-1.9582	1	14	17/32	34.11611	2681	1489.91	MS2	497.307	127	Glioma_grade2_7rep.raw
55956788			gIAYIEFk	Unambiguous	N-Term(iTRAQ4plex); K8(iTRAQ4plex)	HCD	1.8134707	0	1	1	1.65134	1.8474	1.7831	1.9948549	7.846661168	108807.07	97259.25	179677.12	194018.1	7.925159075	5.427205736	2	High	4600	Unique	Used	52107	1	45.524	1	3.169	1.3386	1	43	14/14	36.72294	4600	1228.72	MS2	614.863	210	Glioma_grade2_2.raw
55956788			gIAYIEFk	Unambiguous	N-Term(iTRAQ4plex); K8(iTRAQ4plex)	HCD	1.9133812	0	1	1	1.83778	1.9539	1.8752	1.9937631	3.724689458	74288.71	69871.91	136526.19	139308.03	4.332874305	1.426260983	2	High	4456	Unique	Used	59994	1	45.069	1	3.1764	-2.9333	1	100	13/14	55.36073	4456	1228.71	MS2	614.861	232	Glioma_grade2_3.raw
55956788			kFGYVDFESAEDLEk	Unambiguous	N-Term(iTRAQ4plex); K1(iTRAQ4plex); K15(iTRAQ4plex)	HCD	2.1224852	1	1	1	2.03971	2.0868	2.1574	2.2071702	3.495375164	73705.51	72043.086	150337.86	159011.36	1.613067043	3.965156703	3	High	4545	Unique	Used	91874	1	50.925	1	6.6198	1.9661	1	6	24/56	26.12285	4545	2209.14	MS2	737.053	167	Glioma_grade2_7rep.raw
55956788			gFGFVDFNSEEDAk	Unambiguous	N-Term(iTRAQ4plex); K14(iTRAQ4plex)	HCD	4.39983	0	1	1	4.36892	4.2547	4.5489	4.429932	2.787958486	70536.055	72430.34	308166.3	320861.5	1.87381345	2.854201995	2	High	5771	Unique	Used	53209	1	54.044	1	5.3726	1.0309	1	17	24/26	8.14758	5771	1849.89	MS2	925.447	92	Glioma_grade2_2.raw
55956788			kFGYVDFESAEDLEk	Unambiguous	N-Term(iTRAQ4plex); K1(iTRAQ4plex); K15(iTRAQ4plex)	HCD	3.072196	1	1	1	2.94894	3.0619	3.0821	3.2001684	3.34833398	59319.316	57131.016	174929.38	182828.86	2.65754806	3.122653933	2	High	4568	Unique	Used	91891	1	51.161	1	5.7766	-1.8682	1	45	20/28	14.80167	4568	2209.13	MS2	1105.07	163	Glioma_grade2_7rep.raw
55956788			vTLDWAkPk	Unambiguous	N-Term(iTRAQ4plex); K7(iTRAQ4plex); K9(iTRAQ4plex)	HCD	1.5595958	0	1	1	1.40276	1.6252	1.5029	1.7413032	9.376120766	54628.39	47150.277	76630.664	82102.93	10.39083055	4.875434745	2	High	2736	Unique	Used	90454	1	33.106	1	3.3279	-2.3588	1	90	15/16	30.16665	2736	1489.91	MS2	745.457	135	Glioma_grade2_7rep.raw
55956788			gFGFVDFNSEEDAk	Unambiguous	N-Term(iTRAQ4plex); K14(iTRAQ4plex)	HCD	4.1919646	0	1	1	3.95559	4.3738	4.0275	4.4533362	5.891035158	40253	36403.695	159224.25	162117.89	7.101453222	1.273479081	2	High	5684	Unique	Used	53129	1	53.355	1	5.865	-0.2889	1	75	20/26	23.63266	5684	1849.88	MS2	925.446	91	Glioma_grade2_2.raw
55956788			gYAFIEFASFEDAk	Unambiguous	N-Term(iTRAQ4plex); K14(iTRAQ4plex)	HCD	2.3467867	0	1	1	2.19042	2.6261	2.1138	2.5342476	10.64572838	24803.441	20688.795	54330.05	52430.53	12.79116766	2.516216141	2	High	7439	Unique	Used	54763	1	66.601	1	5.5873	0.1857	1	81	20/26	33.60251	7439	1882.95	MS2	941.977	175	Glioma_grade2_2.raw
55956788			gYAFIEFASFEDAk	Unambiguous	N-Term(iTRAQ4plex); K14(iTRAQ4plex)	HCD	3.8979495	0	1	1	3.49887	3.7559	4.0303	4.326349	9.1235035	21638.043	20157.148	75708.69	87206.85	5.010867861	9.981155766	2	High	7075	Unique	Used	62428	1	65.504	1	5.7088	-0.9164	1	42	21/26	17.36486	7075	1882.94	MS2	941.976	220	Glioma_grade2_3.raw
55956788			gFGFVDFNSEEDAk	Unambiguous	N-Term(iTRAQ4plex); K14(iTRAQ4plex)	HCD	1.7676188	0	1	1	1.58595	1.6508	1.8799	1.9567192	10.06358318	17295.258	16615.854	27429.357	32512.557	2.833355483	11.99282756	2	High	5902	Unique	Used	53333	1	54.999	1	4.4966	-2.9284	1	100	20/26	17.701	5902	1849.88	MS2	925.443	96	Glioma_grade2_2.raw
55956788			gFGFVDFNSEEDAk	Unambiguous	N-Term(iTRAQ4plex); K14(iTRAQ4plex)	HCD	3.8612432	0	1	1	3.66505	4.213	3.5553	4.0867734	8.221867709	11920.874	10370.452	43690.543	42381.688	9.836237736	2.150519942	2	High	5581	Unique	Used	61034	1	53.908	1	5.3643	0.9649	1	100	21/26	37.52536	5581	1849.89	MS2	925.447	93	Glioma_grade2_3.raw
55956788			gYAFIEFASFEDAk	Unambiguous	N-Term(iTRAQ4plex); K14(iTRAQ4plex)	HCD	4.238321	0	1	1	3.80315	4.9586	3.6859	4.8057003	15.33583966	11416.037	8755.951	43416.883	42078.477	18.64927591	2.213911863	2	High	7340	Unique	Used	54680	1	65.673	1	5.4868	-0.5922	1	71	22/26	8.92652	7340	1882.95	MS2	941.976	158	Glioma_grade2_2.raw
55956788			gYAFIEFASFEDAk	Unambiguous	N-Term(iTRAQ4plex); K14(iTRAQ4plex)	HCD	1.5457846	0	1	1	1.69998	1.5151	1.5802	1.4083554	7.869190792	9306.066	10441.597	15820.154	14705.479	8.132017144	5.164146809	2	High	7198	Unique	Used	62538	1	66.55	1	4.6222	-2.3426	1	100	19/26	0	7198	1882.94	MS2	941.975	217	Glioma_grade2_3.raw
55956788			gYAFIEFASFEDAk	Unambiguous	N-Term(iTRAQ4plex); K14(iTRAQ4plex)	HCD	2.5936482	0	1	1	2.45991	2.9618	2.2879	2.7546802	11.48624293	6211.9497	5159.2354	15280.811	14212.043	13.09241585	5.124855671	3	High	7063	Unique	Used	62417	1	65.41	1	4.9994	-0.2104	1	29	22/52	13.1997	7063	1882.95	MS2	628.32	152	Glioma_grade2_3.raw
55956788			gYAFIEFASFEDAk	Unambiguous	N-Term(iTRAQ4plex); K14(iTRAQ4plex)	HCD	1.820013	0	1	1	2.2166	1.6037	2.1191	1.5330948	18.71256321	5141.1704	7106.2183	11395.9	10894.506	22.69052987	3.18109143	3	High	7521	Unique	Used	54839	1	67.241	1	4.6468	0.1786	1	39	22/52	4.806165	7521	1882.95	MS2	628.321	150	Glioma_grade2_2.raw
55956788			gYAFIEFASFEDAk	Unambiguous	N-Term(iTRAQ4plex); K14(iTRAQ4plex)	HCD	1.860363	0	1	1	1.74321	2.1559	1.6214	2.0052464	12.92417432	4312.994	3487.3984	7518.4688	6993.0933	14.96807384	5.120008117	3	High	7418	Unique	Used	54744	1	66.427	1	4.9694	1.4428	1	73	24/52	0	7418	1882.95	MS2	628.321	165	Glioma_grade2_2.raw
55956788			vEGTEPTTAFNLFVGNLNFNk	Unambiguous	N-Term(iTRAQ4plex); K21(iTRAQ4plex)	HCD	2.0372884	0	1	1	1.73175	1.8118	2.2528	2.3569586	15.32738825	2374.9058	2269.9517	4112.7324	5350.182	3.195523472	18.49343588	3	High	8286	Unique	Used	55530	1	73.545	1	5.3539	3.4704	1	77	23/80	15.89836	8286	2600.37	MS2	867.461	160	Glioma_grade2_2.raw
55956788			gYAFIEFASFEDAk	Unambiguous	N-Term(iTRAQ4plex); K14(iTRAQ4plex)	HCD	1.7311429	0	1	1	2.02892	1.9862	1.4706	1.4396313	18.46550019	2120.7688	2166.337	4302.8657	3118.7266	1.50318582	22.56423564	3	High	7258	Unique	Used	62590	1	67.086	1	4.2995	-2.1553	1	100	22/52	22.29865	7258	1882.94	MS2	628.319	155	Glioma_grade2_3.raw
55956921	NP_004490.2	HNRNPAB	heterogeneous nuclear ribonucleoprotein A/B isoform b [Homo sapiens]	4	7	4.267074108	28.60367513	29.627222	14.74	3.66E-05	285	30.569	7.9136	Ribonucleoprotein	Regulation of nucleobase, nucleoside, nucleotide and nucleic acid metabolism	MRNA binding	Nucleolus	Nucleus																									
55956921			iFVGGLNPEATEEk	Unambiguous	N-Term(iTRAQ4plex); K14(iTRAQ4plex)	HCD	3.6343157	0	1	2	3.40762	3.7597	3.5207	3.8844361	5.977582158	85451.83	77449.945	291187.12	300849.38	6.946747076	2.308050118	2	High	4649	Unique	Used	52152	1	45.902	1	5.4533	-0.5255	1	23	21/26	4.474094	4649	1791.97	MS2	896.49	222	Glioma_grade2_2.raw
55956921			gFVFITFk	Unambiguous	N-Term(iTRAQ4plex); K8(iTRAQ4plex)	HCD	4.267074	0	1	2	3.98703	4.5443	4.0238	4.58627	7.561885121	80250.63	70408.664	319961.28	322913.16	9.238488664	0.649362997	2	High	6601	Unique	Used	69635	1	61.48	1	3.5786	0.6731	1	34	13/14	5.70556	6601	1246.74	MS2	623.876	286	Glioma_grade2_4.raw
55956921			gFGFILFk	Unambiguous	N-Term(iTRAQ4plex); K8(iTRAQ4plex)	HCD	4.849362	0	1	2	4.63196	5.034	4.6795	5.0856323	4.83855899	73766.76	67875.78	341684.7	345191.25	5.881780863	0.721965963	2	High	7085	Unique	Used	70068	1	65.578	1	3.6204	-0.4633	1	29	14/14	21.88734	7085	1216.73	MS2	608.87	139	Glioma_grade2_4.raw
55956921			mFVGGLSWDTSk	Unambiguous	N-Term(iTRAQ4plex); K12(iTRAQ4plex)	HCD	2.0284147	0	1	2	1.88908	2.1425	1.9278	2.186436	7.352314292	70568.11	62221.598	133309.06	136043.53	8.889055219	1.435710924	2	High	5843	Unique	Used	61276	1	55.97	1	4.6355	1.5764	1	51	19/22	14.11268	5843	1615.84	MS2	808.425	253	Glioma_grade2_3.raw
55956921			gFVFITFk	Unambiguous	N-Term(iTRAQ4plex); K8(iTRAQ4plex)	HCD	2.8181844	0	1	2	2.64835	2.9001	2.7434	3.0041668	5.62168359	40161.863	36675.227	106362.58	110178.5	6.417275717	2.492148749	2	High	6500	Unique	Used	61892	1	61.047	1	3.3046	0.8689	1	100	13/14	19.00035	6500	1246.74	MS2	623.876	237	Glioma_grade2_3.raw
55956921			gFGFILFk	Unambiguous	N-Term(iTRAQ4plex); K8(iTRAQ4plex)	HCD	5.0253797	0	1	2	4.95757	5.0781	4.974	5.094834	1.399243876	39643.004	38702.473	196533.1	197182.66	1.697751736	0.233319733	2	High	6601	Unique	Used	76845	1	64.093	1	3.5691	-3.2724	1	47	14/14	24.93824	6601	1216.73	MS2	608.868	145	Glioma_grade2_5.raw
55956921			iFVGGLNPEATEEk	Unambiguous	N-Term(iTRAQ4plex); K14(iTRAQ4plex)	HCD	4.452313	0	1	2	4.70889	4.051	4.9188	4.231584	9.031570665	15355.444	17849.057	72307.04	75529.78	10.62055209	3.082887346	2	High	4485	Unique	Used	60020	1	45.313	1	5.4659	2.9486	1	100	21/26	0	4485	1791.98	MS2	896.493	214	Glioma_grade2_3.raw
56549117	NP_001005336.1	DNM1	dynamin-1 isoform 2 [Homo sapiens]	15	29	0.478008926	24.90837892	300.34736	31.26	1.12E-04	851	95.98	7.0054	GTPase	Cell communication,Signal transduction	GTPase activity	Mitochondrion	Endocytic vesicle, Cytoplasm, Cytoplasmic vesicle, Microtubule, Perinuclear region, Cell surface, Cytoskeleton																									
56549117			mGTPYLQk	Unambiguous	N-Term(iTRAQ4plex); K8(iTRAQ4plex)	HCD	0.5570699	0	1	2	0.52038	0.5597	0.5546	0.5965365	0.055881556	2718943	2527746.8	1414879.6	1507893.2	5.15357815	4.500558326	2	High	1851	Unique	Used	57568	1	25.32	1	3.1512	0.1184	1	5	14/14	48.59657	1851	1225.69	MS2	613.346	252	Glioma_grade2_3.raw
56549117			nLVDSYMAIVNk	Unambiguous	N-Term(iTRAQ4plex); K12(iTRAQ4plex)	HCD	0.39885658	0	1	2	0.38316	0.401	0.3968	0.41528347	0.033144431	629703.6	601687.56	241277.56	249870.9	3.217553042	2.474367521	2	High	6557	Unique	Used	53951	1	59.752	1	6.1492	0.7032	1	14	20/22	13.64957	6557	1654.91	MS2	827.958	252	Glioma_grade2_2.raw
56549117			dMLMQFVTk	Unambiguous	N-Term(iTRAQ4plex); K9(iTRAQ4plex)	HCD	0.32551605	0	1	2	0.29638	0.3361	0.3162	0.35855058	0.081562713	605120.9	533659.44	179347.34	191343.89	8.874561877	4.57676965	2	High	6172	Unique	Used	53589	1	56.936	1	4.2186	-4.6592	1	19	15/16	8.103612	6172	1400.75	MS2	700.876	187	Glioma_grade2_2.raw
56549117			kDITAALAAER	Unambiguous	N-Term(iTRAQ4plex); K1(iTRAQ4plex)	HCD	0.3399352	1	1	2	0.31805	0.3233	0.3563	0.36217675	0.066192991	581863.25	572449.44	185063.64	207327.88	1.153338943	8.024227986	2	High	1693	Unique	Used	79163	1	24.296	1	3.7013	15.993	1	16	18/20	8.504459	1693	1446.88	MS2	723.941	137	Glioma_grade2_6.raw
56549117			kFTDFEEVR	Unambiguous	N-Term(iTRAQ4plex); K1(iTRAQ4plex)	HCD	0.33384466	1	1	2	0.30465	0.3291	0.3383	0.36538053	0.075033287	517938.12	479488.84	157789.78	175195.89	5.451576448	7.39249735	2	High	2108	Unique	Used	79546	1	27.451	1	3.2189	4.6733	1	23	14/16	11.99404	2108	1458.79	MS2	729.899	244	Glioma_grade2_6.raw
56549117			kFTDFEEVR	Unambiguous	N-Term(iTRAQ4plex); K1(iTRAQ4plex)	HCD	0.45412818	1	1	2	0.44059	0.4783	0.4319	0.4688291	0.048778679	380038.9	350074.1	167439.95	164124.94	5.80411889	1.413941054	3	High	2094	Unique	Used	79533	1	27.351	1	3.924	6.5677	1	7	16/32	36.56055	2094	1458.79	MS2	486.936	225	Glioma_grade2_6.raw
56549117			fFLSHPSYR	Unambiguous	N-Term(iTRAQ4plex)	HCD	0.3018552	0	1	2	0.28258	0.2596	0.3479	0.31955844	0.129353723	273536.75	297784.75	77296.75	95159.63	6.002198493	14.64829956	3	High	2275	Unique	Used	72963	1	29.073	1	3.4503	-0.3693	1	7	19/32	21.07485	2275	1297.68	MS2	433.232	188	Glioma_grade2_5.raw
56549117			dmLMQFVTk	Unambiguous	N-Term(iTRAQ4plex); M2(Oxidation); K9(iTRAQ4plex)	HCD	0.43730462	0	1	2	0.39915	0.4351	0.4393	0.4788943	0.074442403	256665.83	235441.12	102449.23	112751.41	6.099542536	6.770188074	2	High	4834	Unique	Used	52328	1	47.198	1	3.7439	3.9801	1	27	14/16	32.33913	4834	1416.75	MS2	708.88	207	Glioma_grade2_2.raw
56549117			ymLSVDNLk	Unambiguous	N-Term(iTRAQ4plex); M2(Oxidation); K9(iTRAQ4plex)	HCD	0.46555132	0	1	2	0.44024	0.4462	0.4846	0.49120623	0.05595032	187465.58	184950.28	82529.97	90848.73	0.955161086	6.785437435	2	High	3791	Unique	Used	51346	1	39.556	1	3.2563	2.2408	1	44	14/16	24.89379	3791	1386.76	MS2	693.882	208	Glioma_grade2_2.raw
56549117			nLVDSYmAIVNk	Unambiguous	N-Term(iTRAQ4plex); M7(Oxidation); K12(iTRAQ4plex)	HCD	0.5025072	0	1	2	0.48064	0.5133	0.4924	0.525859	0.040423182	117070.32	109623.55	56268.77	57646.53	4.645614427	1.710434751	2	High	5235	Unique	Used	52704	1	50.138	1	4.6762	-0.2783	1	52	17/22	13.57688	5235	1670.9	MS2	835.955	252	Glioma_grade2_2.raw
56549117			kFFLSHPSYR	Unambiguous	N-Term(iTRAQ4plex); K1(iTRAQ4plex)	HCD	0.47800893	1	1	2	0.4847	0.4581	0.4991	0.47168642	0.036705194	106575.23	112765.46	51656.87	53189.938	3.991191612	2.067860337	2	High	2051	Unique	Used	85453	1	29.963	1	4.0221	-4.2308	1	100	15/18	38.53017	2051	1569.87	MS2	785.439	237	Glioma_grade2_1rep.raw
56549117			dMLMQFVTk	Unambiguous	N-Term(iTRAQ4plex); K9(iTRAQ4plex)	HCD	0.60283715	0	1	2	0.59286	0.6012	0.6044	0.61295336	0.013773968	92000.18	90718.04	54543.406	55605.926	0.992358494	1.364175494	2	High	6100	Unique	Used	61516	1	57.968	1	3.5154	4.3169	1	75	14/16	48.05372	6100	1400.76	MS2	700.883	276	Glioma_grade2_3.raw

56549117			kDITAALAAER	Unambiguous	N-Term(iTRAQ4plex); K1(iTRAQ4plex)	HCD	0.494976	1	1	2	0.42696	0.5286	0.4678	0.5791871	0.133773668	75868.06	61281.89	32392.256	35493.68	15.04044255	6.460949266	3	High	1707	Unique	Used	79176	1	24.403	1	3.9824	7.1887	1	6	23/40	22.94915	1707	1446.86	MS2	482.959	164	Glioma_grade 2_6.raw
56549117			dmLMQFVTk	Unambiguous	N-Term(iTRAQ4plex); M2(Oxidation); K9(iTRAQ4plex)	HCD	0.5534289	0	1	2	0.58546	0.5004	0.6154	0.5260484	0.094942733	73163.75	85597.28	42834.62	45028.316	11.07555598	3.530902535	2	High	4700	Unique	Used	60219	1	46.994	1	3.6359	-2.7406	1	100	14/16	56.87094	4700	1416.74	MS2	708.875	198	Glioma_grade 2_3.raw
56549117			vPVGDQPPDIEFQIR	Unambiguous	N-Term(iTRAQ4plex)	HCD	0.63662845	0	1	2	0.56351	0.6413	0.6326	0.71983975	0.100080638	72584.25	63782.953	40901.734	45913.504	9.127497895	8.164134856	2	High	4120	Unique	Used	86776	1	55.585	1	4.4104	3.1476	1	100	17/28	45.02733	4120	1853.99	MS2	927.501	252	Glioma_grade 2_1rep.raw
56549117			kFFLSHPSYR	Unambiguous	N-Term(iTRAQ4plex); K1(iTRAQ4plex)	HCD	0.62923235	1	1	2	0.62011	0.6181	0.6404	0.63832325	0.018717714	71103.164	71338.75	44091.88	45537.184	0.23389809	2.280475135	3	High	2038	Unique	Used	85442	1	29.845	1	5.6359	-5.0558	1	42	18/36	41.50053	2038	1569.87	MS2	523.962	207	Glioma_grade 2_1rep.raw
56549117			tGLFTPDMAFETIVk	Unambiguous	N-Term(iTRAQ4plex); K15(iTRAQ4plex)	HCD	0.3658097	0	1	2	0.36691	0.3893	0.3437	0.36464584	0.050864499	64674.38	60960.793	23729.465	22229.1	4.180202864	4.616846787	2	High	7585	Unique	Used	54896	1	67.776	1	5.919	0.2227	1	74	21/28	13.97345	7585	1958.06	MS2	979.531	219	Glioma_grade 2_2.raw
56549117			nLVDSYMAIVNk	Unambiguous	N-Term(iTRAQ4plex); K12(iTRAQ4plex)	HCD	0.4136744	0	1	2	0.38606	0.3894	0.4377	0.4415345	0.072577551	59078.203	58566.188	22807.48	25858.992	0.615497731	8.867479969	3	High	6580	Unique	Used	53973	1	59.911	1	4.3911	-1.4071	1	13	22/44	10.36354	6580	1654.91	MS2	552.307	245	Glioma_grade 2_2.raw
56549117			rIEGSGDQIDTYELSGGAR	Unambiguous	N-Term(iTRAQ4plex)	HCD	0.20450653	1	1	2	0.16857	0.1779	0.2298	0.24242681	0.180155954	57501.977	54499.395	9692.897	13212.115	3.791285866	21.72854493	3	High	2713	Unique	Used	66046	1	30.977	1	6.7944	-1.995	1	11	22/72	25.79895	2713	2168.07	MS2	723.36	133	Glioma_grade 2_4.raw
56549117			gWLTINNIGIMk	Unambiguous	N-Term(iTRAQ4plex); K12(iTRAQ4plex)	HCD	0.6301698	0	1	2	0.60948	0.6572	0.6051	0.65248346	0.043713663	45670.71	42353.57	27835.242	27635.004	5.329375459	0.510506651	2	High	6764	Unique	Used	69782	1	62.842	1	5.5749	-0.7449	1	100	19/22	47.13003	6764	1647.95	MS2	824.478	268	Glioma_grade 2_4.raw
56549117			kFFLSHPSYR	Unambiguous	N-Term(iTRAQ4plex); K1(iTRAQ4plex)	HCD	0.44606054	1	1	2	0.4311	0.4409	0.4511	0.46135876	0.029245855	27522.172	26912.818	11864.837	12416.465	1.583093321	3.212841712	2	High	2229	Unique	Used	94994	1	32.217	1	3.2369	-2.7534	1	100	13/18	56.81381	2229	1569.87	MS2	785.441	230	Glioma_grade 2_8rep.raw
56549117			kFFLSHPSYR	Unambiguous	N-Term(iTRAQ4plex); K1(iTRAQ4plex)	HCD	0.5260271	1	1	2	0.48476	0.498	0.5533	0.56842846	0.077814475	27176.225	26452.162	13173.824	15036.161	1.909398682	9.336205755	3	High	2231	Unique	Used	94995	1	32.25	1	5.5449	0.8927	1	85	20/36	31.63183	2231	1569.88	MS2	523.965	143	Glioma_grade 2_8rep.raw
56549117			rIEGSGDQIDTYELSGGAR	Unambiguous	N-Term(iTRAQ4plex)	HCD	0.46878412	1	1	2	0.4468	0.447	0.4906	0.49077755	0.053894384	25027.816	25016.22	11182.45	12277.399	0.03276958	6.600603976	2	High	2467	Unique	Used	58140	1	29.934	1	5.5805	0.4604	1	54	18/36	5.985883	2467	2168.07	MS2	1084.54	99	Glioma_grade 2_3.raw
56549117			rIEGSGDQIDTYELSGGAR	Unambiguous	N-Term(iTRAQ4plex)	HCD	0.29701477	1	1	2	0.32437	0.3693	0.2336	0.26587278	0.202624288	20752.102	18229.736	6731.391	4846.7905	9.150836362	23.01939719	2	High	2736	Unique	Used	66068	1	31.137	1	5.9502	0.2352	1	30	20/36	0	2736	2168.07	MS2	1084.54	82	Glioma_grade 2_4.raw
56549117			rPLVLQLVNATTEYAEFLHck	Unambiguous	N-Term(iTRAQ4plex); C20(Methylthio); K21(iTRAQ4plex)	HCD	0.52625597	0	1	2	0.47775	0.6407	0.4409	0.591306	0.174648607	15816.536	11793.352	7556.389	6973.479	20.60725989	5.673549324	3	High	5548	Unique	Used	96940	1	76.251	1	10.303	2.3967	1	25	37/80	28.58291	5548	2779.49	MS2	927.17	91	Glioma_grade 2_8rep.raw
56549117			kFFLSHPSYR	Unambiguous	N-Term(iTRAQ4plex); K1(iTRAQ4plex)	HCD	0.6213445	1	1	2	0.48968	0.5225	0.714	0.7618211	0.218536617	15513.545	14540.005	7596.726	11076.882	4.581134247	26.35636249	4	High	2034	Unique	Used	85439	1	29.805	1	4.1395	-7.3583	1	70	16/54	51.93604	2034	1569.87	MS2	393.222	197	Glioma_grade 2_1rep.raw
56549117			vYSPHVLNLTLVDLPGMTk	Unambiguous	N-Term(iTRAQ4plex); K19(iTRAQ4plex)	HCD	0.6101235	0	1	2	0.56191	0.574	0.6455	0.6593715	0.080850457	14792.808	14480.777	8312.291	9548.212	1.507428872	9.786153504	2	High	6178	Unique	Used	83046	1	62.968	1	6.323	-2.5507	1	100	22/36	14.57384	6178	2385.34	MS2	1193.17	102	Glioma_grade 2_6.raw
56549117			kFFLSHPSYR	Unambiguous	N-Term(iTRAQ4plex); K1(iTRAQ4plex)	HCD	0.56013906	1	1	2	0.52472	0.5804	0.5418	0.5993207	0.061060652	14264.779	12896.385	7484.9585	7729.071	7.124883725	2.269137234	4	High	2221	Unique	Used	94988	1	32.13	1	4.2967	-1.3709	1	100	17/54	29.69671	2221	1569.88	MS2	393.224	210	Glioma_grade 2_8rep.raw
56549117			kTSGNQDEILVIR	Unambiguous	N-Term(iTRAQ4plex); K1(iTRAQ4plex)	HCD	0.6027083	1	1	2	0.56434	0.6047	0.6008	0.6438184	0.053847546	14228.245	13278.423	8029.603	8548.893	4.883365569	4.429756238	3	High	1576	Unique	Used	72323	1	23.755	1	3.518	2.8256	1	27	17/48	31.66659	1576	1761.02	MS2	587.677	147	Glioma_grade 2_5.raw
56550065	NP_001008221.1	CPLX2	complexin-2 [Homo sapiens]	3	4	0.265957177	26.56949755	22.889782	29.85	1.49E-04	134	15.385	5.0845	Unclassified	Vesicle docking	Binding	Cytoplasm	Ribosome, Endoplasmic reticulum, Plasma membrane																								
56550065			yLPGPLQDMFk	Unambiguous	N-Term(iTRAQ4plex); K11(iTRAQ4plex)	HCD	0.26662976	0	1	1	0.261	0.2746	0.2591	0.27255037	0.029618489	163702.5	155599.64	42726.715	42408.734	3.588818574	0.528208928	2	High	7121	Unique	Used	70101	1	65.866	1	3.9483	5.2378	1	100	15/20	45.19974	7121	1596.88	MS2	798.943	198	Glioma_grade 2_4.raw
56550065			mLGGEEEkDPDAQk	Unambiguous	N-Term(iTRAQ4plex); K8(iTRAQ4plex); K14(iTRAQ4plex)	HCD	0.2285945	1	1	1	0.21746	0.2453	0.2138	0.24115893	0.070197237	162769.69	144288.31	35395.35	34796.414	8.511948312	1.206727636	2	High	1104	Unique	Used	78617	1	19.943	1	5.4707	-3.0872	1	84	19/26	0	1104	1979.01	MS2	990.006	173	Glioma_grade 2_6.raw
56550065			aALEQPcEGSLTRPk	Unambiguous	N-Term(iTRAQ4plex); C7(Methylthio); K15(iTRAQ4plex)	HCD	0.40415886	0	1	1	0.41381	0.4131	0.3952	0.39452577	0.02663316	48385.27	48463.04	20022.182	19119.918	0.113562528	3.259901706	3	High	2442	Unique	Used	73120	1	30.278	1	4.0544	13.664	1	27	28/56	36.92791	2442	1934.03	MS2	645.35	115	Glioma_grade 2_5.raw
56550065			mLGGEEEkDPDAQk	Unambiguous	N-Term(iTRAQ4plex); K8(iTRAQ4plex); K14(iTRAQ4plex)	HCD	0.26528463	1	1	1	0.2184	0.2673	0.2636	0.32267484	0.159316471	21548.586	17604.105	4706.2046	5680.402	14.24765038	13.2644205	4	High	1109	Unique	Used	78621	1	19.995	1	4.9032	7.8787	1	33	22/78	31.34526	1109	1979.03	MS2	495.512	231	Glioma_grade 2_6.raw
56676393	NP_001166.3	ARHGDIB	rho GDP-dissociation inhibitor 2 [Homo sapiens]	2	3	3.884270906	34.67415606	15.576274	15.42	4.86E-04	201	22.974	5.2114	Adapter molecule	Cell communication,Signal transduction	Receptor signaling complex scaffold activity	Cytoplasm	Mitochondrion																								
56676393			aPEPHVEEDDDDELDSk	Unambiguous	N-Term(iTRAQ4plex); K17(iTRAQ4plex)	HCD	3.884271	0	1	1	3.20101	4.447	3.4792	4.8334966	19.4330785	27141.94	19536.96	86881.664	94431.836	23.04052978	5.889002	3	High	1589	Unique	Used	72334	1	23.872	1	6.4806	4.7278	1	7	30/64	1.8177	1589	2228.02	MS2	743.344	53	Glioma_grade 2_5.raw
56676393			eLQEMDkDDESLIk	Unambiguous	N-Term(iTRAQ4plex); K7(iTRAQ4plex); K14(iTRAQ4plex)	HCD	3.3483973	1	1	1	3.5381	3.2986	3.4019	3.1715407	4.638254718	16694.447	17906.803	59066.58	56792.152	4.955110863	2.776249031	3	High	3010	Unique	Used	73647	1	34.541	1	3.3232	1.5462	1	18	25/52	7.61661	3010	2125.11	MS2	709.041	166	Glioma_grade 2_5.raw
56676393			aPEPHVEEDDDDELDSk	Unambiguous	N-Term(iTRAQ4plex); K17(iTRAQ4plex)	HCD	6.2816916	0	1	1	6.87652	5.5892	7.1337	5.7982187	12.11352544	3597.3406	4425.903	24737.186	25662.354	14.6046193	2.596025942	3	High	1722	Unique	Used	65128	1	23.534	1	5.7724	1.6048	1	32	31/64	20.45417	1722	2228.01	MS2	743.342	49	Glioma_grade 2_4.raw
57163987	NP_055878.1	SLC8A2	sodium/calcium exchanger 2 precursor [Homo sapiens]	10	34	0.372012556	25.25942818	236.74498	18.02	6.55E-05	921	100.3	5.1479	Membrane transport protein	Transport	Auxiliary transport protein activity	Plasma membrane	-																								
57163987			iVDDEEYEk	Unambiguous	N-Term(iTRAQ4plex); K9(iTRAQ4plex)	HCD	0.44198692	0	1	1	0.43496	0.4511	0.4332	0.44927007	0.021092997	837556.94	807680.5	364306.75	362866.66	2.56811969	0.280070033	2	High	1669	Unique	Used	49345	1	24.498	1	3.6654	-0.9715	1	8	15/16	58.67072	1669	1427.71	MS2	714.36	133	Glioma_grade 2_2.raw
57163987			vGDAQGMFEPDGGGRPk	Unambiguous	N-Term(iTRAQ4plex); K17(iTRAQ4plex)	HCD	0.30311468	0	1	1	0.29383	0.3243	0.2839	0.31336245	0.060310838	311337.56	282071.75	91480.38	88390.695	6.974630279	2.429225728	3	High	1971	Unique	Used	79419	1	26.425	1	4.7542	4.338	1	4	29/64	11.6271	1971	2006.01	MS2	669.341	114	Glioma_grade 2_6.raw
57163987			lVAPLLATVTILDDDHAGIFSFQDR	Unambiguous	N-Term(iTRAQ4plex)	HCD	0.25189102	0	1	1	0.23167	0.2668	0.239	0.27517822	0.083270158	270839.53	235206.52	62744.746	64723.71	9.958122588	2.195584552	3	High	7967	Unique	Used	77981	1	76.803	1	8.2634	-3.1303	1	4	40/96	4.611095	7967	2871.53	MS2	957.848	81	Glioma_grade 2_5.raw
57163987			iAEMGkPVLGENcR	Unambiguous	N-Term(iTRAQ4plex); K6(iTRAQ4plex); C13(Methylthio)	HCD	0.32919043	0	1	1	0.31885	0.3298	0.3286	0.3398864	0.02612347	166205.66	160696.94	52994.5	54618.703	2.383127738	2.134468491	2	High	3192	Unique	Used	80512	1	36.322	1	4.1384	2.6057	1	93	19/26	12.00742	3192	1850.96	MS2	925.983	237	Glioma_grade 2_6.raw
57163987			lVAPLLATVTILDDDHAGIFSFQDR	Unambiguous	N-Term(iTRAQ4plex)	HCD	0.31044543	0	1	1	0.30095	0.3196	0.3018	0.32053065	0.034818556	112210.15	105647.86	33769.65	33863.375	4.259875282	0.195979946	3	High	8345	Unique	Used	71160	1	76.785	1	8.2288	0.2493	1	16	35/96	13.09066	8345	2871.54	MS2	957.851	75	Glioma_grade 2_4.raw
57163987			tNLALVIGTHSWR	Unambiguous	N-Term(iTRAQ4plex)	HCD	0.3555986	0	1	1	0.3248	0.3245	0.3867	0.38637108	0.100519129	91720.34	91807.12	29790.494	35471.617	0.066870349	12.31085093	3	High	4465	Unique	Used	74953	1	46.329	1	5.3424	-0.7242	1	7	26/48	8.470034	4465	1611.91	MS2	537.974	154	Glioma_grade 2_5.raw
57163987			kDNFFIELGQPQWLk	Unambiguous	N-Term(iTRAQ4plex); K1(iTRAQ4plex); K15(iTRAQ4plex)	HCD	0.4863525	1	1	1	0.44065	0.5066	0.4688	0.5388973	0.087982565	82218.24	71516.305	36229.242	38539.94	9.84477602	4.370544605	3	High	5595	Unique	Used	92653	1	61.901	1	6.249	0.4554	1	10	25/56	22.03918	5595	2295.29	MS2	765.767	120	Glioma_grade 2_7rep.raw
57163987			vGDAQGMFEPDGGGRPk	Unambiguous	N-Term(iTRAQ4plex); K17(iTRAQ4plex)	HCD	0.28778708	0	1	1	0.28434	0.301	0.2753	0.29143292	0.03781438	80360.46	75910.59	22849.945	22122.846	4.027020043	2.286434184	2	High	1975	Unique	Used	79423	1	26.448	1	5.6327	3.6145	1	61	21/32	0	1975	2006.01	MS2	1003.51	108	Glioma_grade 2_6.raw
57163987			tNLALVIGTHSWR	Unambiguous	N-Term(iTRAQ4plex)	HCD	0.29323426	0	1	1	0.27484	0.3086	0.2795	0.31388566	0.067588849	77180.83	68729.41	21212.705	21573.174	8.191414657	1.191468215	2	High	4477	Unique	Used	74965	1	46.397	1	4.3044	0.8745	1	52	19/24	13.84541	4477	1611.91	MS2	806.459	263	Glioma_grade 2_5.raw
57163987			iGIIDDDIFEEDEHFFVR	Unambiguous	N-Term(iTRAQ4plex)	HCD	0.27352625	0	1	1	0.24789	0.2563	0.2902	0.30002964	0.092803991	65761.48	63605.22	16301.737	19083.451	2.357184759	11.11747001	2	High	7806	Unique	Used	70699	1	71.935	1	6.0035	-3.2963	1	48	25/34	2.386443	7806	2353.14	MS2	1177.07	125	Glioma_grade 2_4.raw
57163987			aGSDYEYSEGTLVFkPGETQk	Unambiguous	N-Term(iTRAQ4plex); K15(iTRAQ4plex); K21(iTRAQ4plex)	HCD	0.26033375	0	1	1	0.26001	0.2648	0.256	0.2606675	0.01384732	63273.28	62133.22	16451.434	16196.111	1.285649718	1.105998167	2	High	3437	Unique	Used	80735	1	38.24	1	6.0509	-1.4612	1	38	24/40	0	3437	2738.38	MS2	1369.7	121	Glioma_grade 2_6.raw
57163987			dNFFIELGQPQWLk	Unambiguous	N-Term(iTRAQ4plex); K14(iTRAQ4plex)	HCD	0.41391876	0	1	1	0.37043	0.3978	0.4289	0.46062845	0.094028729	62947.844	58612.65	23317.516	26998.654	5.043489006	10.3464061	2	High	7743	Unique	Used	63022	1	71.23	1	4.8519	0.0463	1	89	19/26	15.56573	7743	2023.09	MS2	1012.05	203	Glioma_grade 2_3.raw
57163987			dNFFIELGQPQWLk	Unambiguous	N-Term(iTRAQ4plex); K14(iTRAQ4plex)	HCD	0.4012438	0	1	1	0.3672	0.3905	0.4113	0.43745688	0.074497555	39914.684	37528.168	14656.509	16416.955	4.358108214	8.012130894	2	High	8123	Unique	Used	55385	1	72.156	1	4.6643	-2.0052	1	78	20/26	19.25352	8123	2023.09	MS2	1012.05	190	Glioma_grade 2_2.raw
57163987			iGIIDDDIFEEDEHFFVR	Unambiguous	N-Term(iTRAQ4plex)	HCD	0.27875182	0	1	1	0.25819	0.2762	0.2811	0.30075556	0.062705193	39259	36694.527	10136.101	11036.084	4.774909922	6.01151069	2	High	7796	Unique	Used	63070	1	71.673	1	6.2256	-3.2963	1	100	24/34	14.81041	7796	2353.14	MS2	1177.07	129	Glioma_grade 2_3.raw
57163987			iGIIDDDIFEEDEHFFVR	Unambiguous	N-Term(iTRAQ4plex)	HCD	0.27660966	0	1	1	0.24574	0.2665	0.2859	0.31008977	0.099073224	31822.404	29339.31	7820.101	9097.82	5.741541533	10.68078955	3	High	7662	Unique	Used	62948	1	70.561	1	7.883	-2.4979	1	45	30/68	19.35257	7662	2353.14	MS2	785.052	121	Glioma_grade 2_3.raw
57163987			vGDAQGMFEPDGGGRPk	Unambiguous	N-Term(iTRAQ4plex); K17(iTRAQ4plex)	HCD	0.4915484	0	1	1	0.47439	0.5307	0.4565	0.5107483	0.068388724	28500.463	25476.082	13520.218	13011.866	7.924035575	2.709618637	3	High	1916	Unique	Used	72635	1	26.311	1	4.9465	0.9607	1	56	24/64	56.47363	1916	2006	MS2	669.339	89	Glioma_grade 2_5.raw
57163987			iGIIDDDIFEEDEHFFVR	Unambiguous	N-Term(iTRAQ4plex)	HCD	0.29462743	0	1	1	0.26131	0.2867	0.3018	0.33118522	0.09891965	27946.656	25469.436	7302.745	8435.101	6.55854442	10.17542815	3	High	7752	Unique	Used	63030	1	71.304	1	7.2894	-1.6419	1	27	31/68	5.201613	7752	2353.14	MS2	785.053	117	Glioma_grade 2_3.raw
57163987			lVAPLLATVTILDDDHAGIFSFQDR	Unambiguous	N-Term(iTRAQ4plex)	HCD	0.32102677	0	1	1	0.31619	0.3541	0.2915	0.32644075	0.080459719	21924.836	19577.738	6932.466	6390.971	7.997811952	5.747687875	2	High	7974	Unique	Used	77988	1	76.845	1	5.1932	-4.9323	1	51	22/48	12.45573	7974	2871.52	MS2	1436.27	90	Glioma_grade 2_5.raw
57163987			gGGVHYEDAcGELEFGDDETMk	Unambiguous	N-Term(iTRAQ4plex); C10(Methylthio); K22(iTRAQ4plex)	HCD	0.41097856	0	1	1	0.33432	0.4081	0.4134	0.5045429	0.167943169	21783.781	17847.543	7282.774	9004.851	14.04616501	14.95236199	3	High	5408	Unique	Used	75786	1	54.171	1	7.5059	1.2747	1	14	30/84	6.408795	5408	2693.14	MS2	898.386	30	Glioma_grade 2_5.raw
57163987			iGIIDDDIFEEDEHFFVR	Unambiguous	N-Term(iTRAQ4plex)	HCD	0.4222107	0	1	1	0.42792	0.4334	0.4112	0.41643268	0.024156183	20011.328	19759.266	8563.166	8228.404	0.896314244	2.819420463	3	High	7865	Unique	Used	70750	1	72.463	1	6.5227	1.0815	1	28	30/68	9.497682	7865	2353.15	MS2	785.055	123	Glioma_grade 2_4.raw
57163987			iGIIDDDIFEEDEHFFVR	Unambiguous	N-Term(iTRAQ4plex)	HCD	0.3676661	0	1	1	0.37127	0.4163	0.3243	0.3636303	0.102301496	18213.998	16243.111	6762.2236	5906.4873	8.089056819	9.552620553	3	High	7699	Unique	Used	70606	1	70.971	1	7.8602	-1.2529	1	51	32/68	21.50779	7699	2353.14	MS2	785.053	120	Glioma_grade 2_4.raw
57163987			iGIIDDDIFEEDEHFFVR	Unambiguous	N-Term(iTRAQ4plex)	HCD	0.3751892	0	1	1	0.35738	0.3493	0.4016	0.39259696	0.068585226	16893.543	17282.105	6037.431	6784.902	1.607898262	8.244081835	3	High	8224	Unique	Used	63443	1	75.517	1	7.0048	0.5368	1	50	31/68	12.68865	8224	2353.15	MS2	785.054	110	Glioma_grade 2_3.raw
57163987			vGDAQGMFEPDGGGRPk	Unambiguous	N-Term(iTRAQ4plex); K17(iTRAQ4plex)	HCD	0.54017115	0	1	1	0.47155	0.5478	0.5336	0.6198837	0.112112941	15456.615	13305.534	7288.599	8247.884	10.57670595	8.731923803	3	High	2035	Unique	Used	72740	1	27.297	1	3.377	1.052	1	75	14/64	43.60605	2035	2006	MS2	669.339	117	Glioma_grade 2_5.raw
57163987			dNFFIELGQPQWLk	Unambiguous	N-Term(iTRAQ4plex); K14(iTRAQ4plex)	HCD	0.53914064	0	1	1	0.56313	0.5376	0.5408	0.5162355	0.035577977	15041.842	15756.362	8470.57	8133.993	3.28098312	2.866632252	3	High	8108	Unique	Used	55372	1	72.025	1	4.8438	1.3068	1	20	23/52	10.34385	8108	2023.09	MS2	675.035	198	Glioma_grade 2_2.raw
57163987			iGIIDDDIFEEDEHFFVR	Unambiguous	N-Term(iTRAQ4plex)	HCD	0.44364592	0	1	1	0.48746	0.5522	0.3478	0.3940088	0.206382049	14151.342	12491.801	6898.2427	4921.8794	8.808815797	23.646116	3	High	8090	Unique	Used	55356	1	71.874	1	6.9517	-0.2413	1	44	29/68	0	8090	2353.15	MS2	785.054	146	Glioma_grade 2_2.raw
57163987			iGIIDDDIFEEDEHFFVR	Unambiguous	N-Term(iTRAQ4plex)	HCD	0.44347432	0	1	1	0.39156	0.4998	0.3994	0.50973266	0.140640855	12992.232	10179.397	5087.252	5188.7715	17.16732736	1.397138239	3	High	7916	Unique	Used	63176	1	72.734	1	6.2765	1.8596	1	58	31/68	10.60748	7916	2353.15	MS2	785.055	126	Glioma_grade 2_3.raw
57163987			dNFFIELGQPQWLk	Unambiguous	N-Term(iTRAQ4plex); K14(iTRAQ4plex)	HCD	0.49286443	0	1	1	0.41037	0.451	0.5309	0.5835376	0.157765952	12436.44	11314.98	5103.514	6602.717	6.677427883	18.11166391	3	High	7739	Unique	Used	63018	1	71.204	1	4.9666	-0.5939	1	48	23/52	13.8278	7739	2023.09	MS2	675.034	200	Glioma_grade 2_3.raw
57163987			iGIIDDDIFEEDEHFFVR	Unambiguous	N-Term(iTRAQ4plex)	HCD	0.35586706	0	1	1	0.34411	0.381	0.3332	0.36888537	0.061680489	12280.008	11090.438	4225.671	4091.1003	7.198433557	2.288288353	3	High	8164	Unique	Used	70996	1	75.365	1	6.973	2.0931	1	100	27/68	41.90864	8164	2353.15	MS2	785.056	135	Glioma_grade 2_4.raw
57163987			gGGVHYEDAcGELEFGDDETMk	Unambiguous	N-Term(iTRAQ4plex); C10(Methylthio); K22(iTRAQ4plex)	HCD	0.6633969	0	1	1	0.59987	0.6908	0.6396	0.73655087	0.08939393	10951.378	9510.501	6569.379	7004.9683	9.958556568	4.538091461	3	High	5830	Unique	Used	68928	1	55.296	1	7.5733	3.3144	1	43	30/84	11.22154	5830	2693.15	MS2	898.388	27	Glioma_grade 2_4.raw
57163987			iGIIDDDIFEEDEHFFVR	Unambiguous	N-Term(iTRAQ4plex)	HCD	0.3454346	0	1	1	0.33895	0.3414	0.3495	0.3519682	0.018114256	10113.813	10041.834	3428.052	3534.4065	0.505038007	2.160271064	2	High	7718	Unique	Used	70623	1	71.135	1	4.7345	-0.91	1	100	21/34	15.93026	7718	2353.14	MS2	1177.08	120	Glioma_grade 2_4.raw
57163987			iGIIDDDIFEEDEHFFVR	Unambiguous	N-Term(iTRAQ4plex)	HCD	0.489816	0	1	1	0.38496	0.4037	0.5719	0.59978247	0.227346862	5918.95	5644.032	2278.5425	3385.1917	3.362391848	27.63262279	3	High	8052	Unique	Used	63298	1	73.908	1	6.7759	-1.4085	1	100	33/68	17.62904	8052	2353.14	MS2	785.053	110	Glioma_grade 2_3.raw
57163987			iGIIDDDIFEEDEHFFVR	Unambiguous	N-Term(iTRAQ4plex)	HCD	0.44842088	0	1	1	0.47001	0.4109	0.4913	0.42954728	0.081549387	4966.7183	5680.6025	2334.3936	2440.0874	9.482054092	3.130677563	3	High	8011	Unique	Used	70868	1	73.913	1	6.459	-3.4317	1	100	32/68	28.63343	8011	2353.14	MS2	785.051	123	Glioma_grade 2_4.raw

57163987			iGIIDDDIFEEDEHFFVR	Unambiguous	N-Term(iTRAQ4plex)	HCD	0.36883596	0	1	1	0.39598	0.3697	0.3679	0.34349072	0.058084937	3212.258	3440.3381	1271.9926	1181.7241	4.848542823	5.202676289	3	High	8457	Unique	Used	71258	1	77.749	1	5.3166	0.1478	1	100	30/68	38.54374	8457	2353.15	MS2	785.054	131	Glioma_grade 2_4.raw
57163987			iGIIDDDIFEEDEHFFVR	Unambiguous	N-Term(iTRAQ4plex)	HCD	0.5205199	0	1	1	0.47227	0.5526	0.4931	0.5769857	0.093923102	1067.2664	912.0638	504.033	526.24774	11.08908568	3.049303493	3	High	8516	Unique	Used	71281	1	78.705	1	4.3299	1.5484	1	100	25/68	8.70858	8516	2353.15	MS2	785.055	99	Glioma_grade 2_4.raw
58218968	NP_005175.2	CALM3	calmodulin [Homo sapiens]	3	11	0.460006177	24.73692673	66.545239	36.91	1.80E-04	149	16.827	4.2212	Calcium binding protein	Cell communication,Signal transduction	Calcium ion binding	Cytoplasm	Nucleus, Plasma membrane																								
58218968			eAFSLFDk	Unambiguous	N-Term(iTRAQ4plex); K8(iTRAQ4plex)	HCD	0.46419615	0	1	2	0.42595	0.4736	0.4557	0.50671786	0.072578761	1.04E+07	9370156	4437906.5	4748025.5	7.493739977	4.774414787	2	High	5071	Unique	Used	52550	1	48.951	1	3.7385	-0.283	1	1	14/14	7.948358	5071	1244.68	MS2	622.842	213	Glioma_grade 2_2.raw
58218968			eAFSLFDk	Unambiguous	N-Term(iTRAQ4plex); K8(iTRAQ4plex)	HCD	0.46000618	0	1	2	0.44004	0.4782	0.4433	0.481702	0.048170174	1.03E+07	9478554	4532650	4565838.5	5.876981008	0.515861803	2	High	4943	Unique	Used	60442	1	48.946	1	3.6689	-2.7349	1	1	14/14	6.172896	4943	1244.67	MS2	622.84	214	Glioma_grade 2_3.raw
58218968			eAFSLFDk	Unambiguous	N-Term(iTRAQ4plex); K8(iTRAQ4plex)	HCD	0.5323796	0	1	2	0.48197	0.5367	0.5285	0.58851206	0.081755399	888538.7	797899.56	428251.88	469573.53	7.600817896	6.508797501	2	High	5034	Unique	Used	60526	1	49.674	1	3.8834	2.7573	1	14	14/14	20.97913	5034	1244.68	MS2	622.844	202	Glioma_grade 2_3.raw
58218968			eAFSLFDk	Unambiguous	N-Term(iTRAQ4plex); K8(iTRAQ4plex)	HCD	0.5565127	0	1	2	0.5421	0.5384	0.5748	0.57082283	0.033997694	821931.6	827604.06	445572.25	472415.28	0.486322912	4.135326008	2	High	4979	Unique	Used	52464	1	48.263	1	3.7307	-0.6753	1	9	14/14	20.82604	4979	1244.68	MS2	622.842	242	Glioma_grade 2_2.raw
58218968			eAFSLFDk	Unambiguous	N-Term(iTRAQ4plex); K8(iTRAQ4plex)	HCD	0.5261639	0	1	2	0.46442	0.5164	0.5349	0.59482205	0.101991724	303949.5	273335.03	141160.58	162585.7	7.499836983	9.975330489	2	High	5180	Unique	Used	52652	1	49.746	1	3.6706	-5.7752	1	25	14/14	41.84543	5180	1244.67	MS2	622.838	256	Glioma_grade 2_2.raw
58218968			eAFSLFDkDGDGTITTk	Unambiguous	N-Term(iTRAQ4plex); K8(iTRAQ4plex); K17(iTRAQ4plex)	HCD	0.5028283	1	1	1	0.52237	0.4883	0.5184	0.48456118	0.039173559	187472.55	200551.36	97930	97179.41	4.766775965	0.544050929	2	High	4308	Unique	Used	81480	1	45.96	1	6.9509	0.8696	1	39	22/32	4.284129	4308	2277.2	MS2	1139.1	183	Glioma_grade 2_6.raw
58218968			eAFSLFDkDGDGTITTk	Unambiguous	N-Term(iTRAQ4plex); K8(iTRAQ4plex); K17(iTRAQ4plex)	HCD	0.3503548	1	1	1	0.33521	0.4058	0.3046	0.3686911	0.123255633	121772.086	100587.54	40819.03	37085.73	13.47343167	6.777100003	3	High	4535	Unique	Used	81666	1	48.113	1	5.5123	4.1068	1	18	26/64	11.62651	4535	2277.21	MS2	759.741	176	Glioma_grade 2_6.raw
58218968			eAFSLFDkDGDGTITTk	Unambiguous	N-Term(iTRAQ4plex); K8(iTRAQ4plex); K17(iTRAQ4plex)	HCD	0.44515213	1	1	1	0.41624	0.5038	0.3967	0.48014125	0.113323775	86669.73	71612.086	36075.57	34383.918	13.45367706	3.395365577	2	High	4440	Unique	Used	81585	1	47.266	1	6.1733	1.4056	1	48	21/32	0	4440	2277.2	MS2	1139.1	183	Glioma_grade 2_6.raw
58218968			vFDkDGNGYISAAELR	Unambiguous	N-Term(iTRAQ4plex); K4(iTRAQ4plex)	HCD	0.29179752	1	1	1	0.30045	0.3083	0.2757	0.28291547	0.051760825	82472.76	80374.61	24779.24	22739.22	1.822094017	6.071375107	3	High	3842	Unique	Used	81089	1	41.693	1	6.19	4.1466	1	25	25/60	13.23217	3842	2043.08	MS2	681.699	186	Glioma_grade 2_6.raw
58218968			vFDkDGNGYISAAELR	Unambiguous	N-Term(iTRAQ4plex); K4(iTRAQ4plex)	HCD	0.22139603	1	1	1	0.22951	0.2164	0.2267	0.21374628	0.034682486	77972.81	82695.49	17895.47	17675.854	4.156935815	0.873130069	2	High	3872	Unique	Used	81114	1	41.975	1	4.5236	1.5542	1	55	23/30	0	3872	2043.08	MS2	1022.04	184	Glioma_grade 2_6.raw
58218968			eAFSLFDkDGDGTITTk	Unambiguous	N-Term(iTRAQ4plex); K8(iTRAQ4plex); K17(iTRAQ4plex)	HCD	0.3917636	1	1	1	0.37917	0.3978	0.386	0.4049794	0.029558826	52687.074	50218.727	19977.197	20337.55	3.392199244	1.264093509	3	High	3972	Unique	Used	81192	1	42.999	1	5.1115	-1.361	1	42	25/64	15.53619	3972	2277.19	MS2	759.736	147	Glioma_grade 2_6.raw
58219014	NP_001010898.1	SLC6A17	sodium-dependent neutral amino acid transporter SLC6A17 [Homo sapiens]	4	8	0.398807287	35.26218879	39.613171	9.49	1.92E-04	727	80.948	5.9858	Membrane transport protein	Transport	Auxiliary transport protein activity	Integral to membrane	-																								
58219014			vPSEAPSPMPTHR	Unambiguous	N-Term(iTRAQ4plex)	HCD	0.31731182	0	1	1	0.30135	0.302	0.3326	0.33330265	0.056964207	772214.4	770638.6	232709.61	256855.89	0.144441352	6.97516403	2	High	1284	Unique	Used	64721	1	20.357	1	3.8499	1.9918	1	6	15/24	4.547563	1284	1549.79	MS2	775.401	170	Glioma_grade 2_4.raw
58219014			qSVLNVAGEAGGk	Unambiguous	N-Term(iTRAQ4plex); K13(iTRAQ4plex)	HCD	0.6602059	0	1	1	0.6577	0.6811	0.64	0.66280025	0.025573117	252565.69	243892.34	166112.6	161651.92	2.470696103	1.924660471	2	High	1935	Unique	Used	49597	1	26.325	1	5.7952	-1.0789	1	21	21/24	47.25602	1935	1517.85	MS2	759.429	253	Glioma_grade 2_2.raw
58219014			vPSEAPSPMPTHR	Unambiguous	N-Term(iTRAQ4plex)	HCD	0.312808	0	1	1	0.29084	0.3155	0.3103	0.33663732	0.060065737	217677.55	200674.25	63309.355	67554.445	5.747865185	4.587566502	2	High	1363	Unique	Used	57107	1	21.887	1	3.9575	1.1254	1	31	14/24	15.6471	1363	1549.79	MS2	775.4	155	Glioma_grade 2_3.raw
58219014			vPSEAPSPMPTHR	Unambiguous	N-Term(iTRAQ4plex)	HCD	0.41573197	0	1	1	0.38783	0.4352	0.3984	0.44704285	0.068223421	212803.38	189652.14	82530.984	84782.63	8.135258672	1.903197376	3	High	1279	Unique	Used	64717	1	20.312	1	4.6892	1.1167	1	10	22/48	33.43209	1279	1549.79	MS2	517.269	188	Glioma_grade 2_4.raw
58219014			vPSEAPSPMPTHR	Unambiguous	N-Term(iTRAQ4plex)	HCD	0.25777534	0	1	1	0.24882	0.2626	0.2532	0.26722628	0.032697546	162331.86	153817.31	40391.426	41104.027	3.80876916	1.236596597	3	High	1357	Unique	Used	57101	1	21.853	1	4.3806	0.2897	1	3	23/48	5.560855	1357	1549.79	MS2	517.269	148	Glioma_grade 2_3.raw
58219014			gAVDGILHMFTPk	Unambiguous	N-Term(iTRAQ4plex); K13(iTRAQ4plex)	HCD	0.38188258	0	1	1	0.39528	0.4201	0.3459	0.367647	0.084637963	36095.418	33959.71	14267.648	12485.186	4.31138634	9.422485613	3	High	5713	Unique	Used	82675	1	58.429	1	4.4807	3.9127	1	52	19/48	49.70101	5713	1673.94	MS2	558.65	158	Glioma_grade 2_6.raw
58219014			aVEEELDAEDRPAWNSk	Unambiguous	N-Term(iTRAQ4plex); K17(iTRAQ4plex)	HCD	0.42996192	0	1	1	0.38178	0.5004	0.3762	0.49310684	0.155491258	16503.225	12591.304	6300.681	6208.8584	19.01488673	1.038061931	3	High	3687	Unique	Used	66951	1	38.482	1	4.8563	-0.4631	1	88	28/64	7.015076	3687	2247.11	MS2	749.709	92	Glioma_grade 2_4.raw
58219014			vPSEAPSPmPTHR	Unambiguous	N-Term(iTRAQ4plex); M9(Oxidation)	HCD	0.64890367	0	1	1	0.63753	0.7198	0.5861	0.6617403	0.085182553	7586.481	6719.6797	4836.637	4446.683	8.568631236	5.940528125	3	High	1060	Unique	Used	64516	1	18.666	1	4.2254	-1.4162	1	100	19/48	43.0499	1060	1565.78	MS2	522.6	146	Glioma_grade 2_4.raw
61744483	NP_001013269.1	SLC3A2	4F2 cell-surface antigen heavy chain isoform f [Homo sapiens]	12	17	0.456078798	27.42679245	96.146268	32.51	3.90E-04	529	57.909	5.3511	Membrane transport protein	Transport	Auxiliary transport protein activity	Cytoplasm, Nucleus, Plasma membrane	Mitochondrion, Extracellular																								
61744483			lLTSFLPAQLLR	Unambiguous	N-Term(iTRAQ4plex)	HCD	0.2973217	0	1	4	0.26981	0.2851	0.3089	0.326389	0.084243751	1027283.7	972225	277173.78	317323.56	3.894194622	9.550986957	2	High	5920	Unique	Used	88089	1	75.53	1	4.1796	-1.0062	1	7	17/22	6.314698	5920	1515.94	MS2	758.472	84	Glioma_grade 2_1rep.raw
61744483			lEPHEGLLLR	Unambiguous	N-Term(iTRAQ4plex)	HCD	0.58976173	0	1	4	0.58626	0.5775	0.6022	0.59321463	0.017761711	557217.3	565660.94	326672.28	335558.34	1.063437671	1.897645048	2	High	2348	Unique	Used	73032	1	29.593	1	3.1137	12.221	1	11	17/18	25.11753	2348	1320.79	MS2	660.9	241	Glioma_grade 2_5.raw
61744483			lLTSFLPAQLLR	Unambiguous	N-Term(iTRAQ4plex)	HCD	0.29968512	0	1	4	0.31382	0.2969	0.3026	0.28631288	0.038230359	535202.7	565640	167956.17	161950.02	3.910171949	2.574664873	2	High	8553	Unique	Used	55773	1	75.744	1	4.1305	-0.362	1	9	17/22	13.00519	8553	1515.94	MS2	758.472	79	Glioma_grade 2_2.raw
61744483			wWHTGALYR	Unambiguous	N-Term(iTRAQ4plex)	HCD	0.45398787	0	1	4	0.44643	0.4503	0.4577	0.46161366	0.015203563	483417.12	479305.38	215810.44	221253.9	0.604003591	1.761345929	2	High	3326	Unique	Used	80634	1	37.379	1	3.0445	10.398	1	20	13/16	37.55993	3326	1333.71	MS2	667.357	262	Glioma_grade 2_6.raw
61744483			eDFDSLLQSAk	Unambiguous	N-Term(iTRAQ4plex); K11(iTRAQ4plex)	HCD	0.5606704	0	1	4	0.53439	0.5531	0.568	0.58786845	0.040396988	478742.97	462523.12	255837.23	271902.75	2.43696571	4.305164879	2	High	5505	Unique	Used	52959	1	52.097	1	5.2743	-0.7243	1	23	18/20	35.94535	5505	1540.81	MS2	770.908	224	Glioma_grade 2_2.raw
61744483			gLVLGPIHk	Unambiguous	N-Term(iTRAQ4plex); K9(iTRAQ4plex)	HCD	0.2875876	0	1	4	0.26229	0.2841	0.2908	0.3149857	0.075317553	434425.2	401066.44	113946.85	126330.2	5.646544927	7.28854246	3	High	2148	Unique	Used	79583	1	27.757	1	3.7027	-0.1567	1	5	23/32	32.65336	2148	1221.79	MS2	407.936	68	Glioma_grade 2_6.raw
61744483			lDYLSSLk	Unambiguous	N-Term(iTRAQ4plex); K8(iTRAQ4plex)	HCD	0.39166495	0	1	4	0.36669	0.3892	0.394	0.41816714	0.053858773	351010.5	330743.75	128713.08	138306.17	4.204082734	5.080786491	2	High	5046	Unique	Used	52527	1	48.754	1	3.2125	-9.9845	1	26	14/14	47.51423	5046	1226.71	MS2	613.859	236	Glioma_grade 2_2.raw
61744483			iGDLQAFQGHGAGNLAGLk	Unambiguous	N-Term(iTRAQ4plex); K19(iTRAQ4plex)	HCD	0.38127643	0	1	4	0.35776	0.3786	0.3839	0.40615544	0.052096316	298562.75	282168.03	106814.875	114604.08	3.992492937	4.975002863	3	High	4705	Unique	Used	81815	1	49.557	1	7.5348	-1.3519	1	5	33/72	9.735432	4705	2155.18	MS2	719.066	127	Glioma_grade 2_6.raw
61744483			iGDLQAFQGHGAGNLAGLk	Unambiguous	N-Term(iTRAQ4plex); K19(iTRAQ4plex)	HCD	0.37837496	0	1	4	0.35336	0.3991	0.36	0.40662915	0.071030535	170943.08	151347.95	60404.375	61542.49	8.598346222	1.319868017	2	High	4724	Unique	Used	81833	1	49.688	1	6.8503	-0.3261	1	25	23/36	10.31302	4724	2155.19	MS2	1078.1	162	Glioma_grade 2_6.raw
61744483			gLVLGPIHk	Unambiguous	N-Term(iTRAQ4plex); K9(iTRAQ4plex)	HCD	0.34949294	0	1	4	0.3402	0.378	0.3238	0.35981485	0.067210139	165416.17	148858.48	56275.273	53561.492	7.450842682	3.494154162	2	High	2096	Unique	Used	79535	1	27.36	1	3.6053	-1.0199	1	32	15/16	52.12732	2096	1221.79	MS2	611.399	66	Glioma_grade 2_6.raw
61744483			gENSWFSTQVDTVATk	Unambiguous	N-Term(iTRAQ4plex); K16(iTRAQ4plex)	HCD	0.6563167	0	1	4	0.58934	0.6764	0.6388	0.7331928	0.092112382	98334.03	85672.766	57952.074	62814.656	9.731016275	5.694225067	2	High	5463	Unique	Used	52919	1	51.803	1	5.3774	-0.1836	1	23	21/30	3.446866	5463	2058.04	MS2	1029.52	174	Glioma_grade 2_2.raw
61744483			lDYLSSLk	Unambiguous	N-Term(iTRAQ4plex); K8(iTRAQ4plex)	HCD	0.4560788	0	1	4	0.47211	0.4133	0.505	0.44204542	0.086028922	94961.195	108478.51	44832.11	47952.426	9.396577821	4.755957616	2	High	4917	Unique	Used	60419	1	48.716	1	3.3937	-9.2879	1	100	14/14	48.95721	4917	1226.71	MS2	613.86	222	Glioma_grade 2_3.raw
61744483			gQSEDPGSLLSLFR	Unambiguous	N-Term(iTRAQ4plex)	HCD	0.62392515	0	1	4	0.55285	0.5941	0.6516	0.7003108	0.103484522	77347.95	71972.1	42761.695	50402.84	5.091479664	11.59906062	2	High	5197	Unique	Used	87554	1	67.534	1	4.3523	0.8354	1	76	20/26	32.80911	5197	1649.86	MS2	825.435	199	Glioma_grade 2_1rep.raw
61744483			aDLLLSTQPGREEGSPLELER	Unambiguous	N-Term(iTRAQ4plex)	HCD	0.6001056	1	1	4	0.60972	0.6631	0.5422	0.58965176	0.083298992	59363.758	54583.87	36195.137	32185.475	5.932359063	8.292582086	3	High	5287	Unique	Used	60765	1	51.558	1	3.2982	1.9089	1	10	26/80	13.35064	5287	2454.3	MS2	818.772	101	Glioma_grade 2_3.raw
61744483			lkLEPHEGLLLR	Unambiguous	N-Term(iTRAQ4plex); K2(iTRAQ4plex)	HCD	0.6357088	1	1	4	0.58468	0.651	0.622	0.6925205	0.071536288	58410.62	52460.24	34151.793	36329.79	7.59000886	4.370152833	2	High	3073	Unique	Used	86153	1	41.968	1	4.1231	-0.8991	1	100	15/22	27.90807	3073	1706.06	MS2	853.531	54	Glioma_grade 2_1rep.raw
61744483			dLLLTSSYLSDSGSTGEHTk	Unambiguous	N-Term(iTRAQ4plex); K20(iTRAQ4plex)	HCD	0.5897875	0	1	4	0.51676	0.5533	0.6239	0.66797554	0.115442636	51775.184	48357.68	26755.361	32301.746	4.826667603	13.28167478	3	High	4527	Unique	Used	60057	1	45.677	1	8.0576	-0.7484	1	9	36/76	9.794912	4527	2399.22	MS2	800.41	141	Glioma_grade 2_3.raw
61744483			dLLLTSSYLSDSGSTGEHTk	Unambiguous	N-Term(iTRAQ4plex); K20(iTRAQ4plex)	HCD	0.4933873	0	1	4	0.47214	0.3495	0.6877	0.50911313	0.277095303	47705.793	64442.234	22524.025	32808.387	21.10505417	26.28528867	3	High	4209	Unique	Used	74720	1	44.303	1	8.6487	0.0148	1	8	35/76	8.401649	4209	2399.22	MS2	800.411	138	Glioma_grade 2_5.raw
62414289	NP_003371.2	VIM	vimentin [Homo sapiens]	12	34	4.46786085	40.18685268	233.72998	30.69	7.87E-05	466	53.619	5.1226	Cytoskeletal protein	Cell growth and/or maintenance	Structural constituent of cytoskeleton	Intermediate filament	Nucleolus, Golgi apparatus, Cytoplasm, Membrane fraction, Endoplasmic reticulum, Perinuclear region, Nucleus, Mitochondrion, Cytoskeleton, Extracellular																								
62414289			nLQEAEEWYk	Unambiguous	N-Term(iTRAQ4plex); K10(iTRAQ4plex)	HCD	4.1900234	0	1	2	3.69031	4.2444	4.1427	4.7647696	10.47468588	709086.75	616514.44	2616751	2937549	9.87604849	8.168029858	2	High	4107	Unique	Used	51643	1	41.897	1	4.6195	-1.6627	1	3	17/18	23.50348	4107	1597.81	MS2	799.407	156	Glioma_grade 2_2.raw
62414289			iLLAELEQLk	Unambiguous	N-Term(iTRAQ4plex); K10(iTRAQ4plex)	HCD	6.9587755	0	1	1	6.00167	7.9373	6.2188	8.224566	16.1683092	282447.2	213566.95	1695155.4	1756495.8	19.63883162	2.513244258	2	High	7325	Unique	Used	62645	1	67.735	1	4.3748	1.2224	1	4	17/18	7.551294	7325	1457.92	MS2	729.464	65	Glioma_grade 2_3.raw
62414289			lQDEIQNMk	Unambiguous	N-Term(iTRAQ4plex); K9(iTRAQ4plex)	HCD	3.2415266	0	1	1	2.977	3.4074	3.0966	3.5443017	8.11168979	229239.17	200280.33	682445	709853.9	9.534837016	2.784031368	2	High	3349	Unique	Used	50929	1	36.362	1	4.2021	0.9241	1	13	16/16	29.00071	3349	1406.76	MS2	703.882	247	Glioma_grade 2_2.raw
62414289			lQDEIQNMk	Unambiguous	N-Term(iTRAQ4plex); K9(iTRAQ4plex)	HCD	4.7046666	0	1	1	4.5295	4.5806	4.8274	4.88181	3.732463024	191192.56	189060.02	866006.44	922955.06	0.793122032	4.501914142	2	High	3446	Unique	Used	51021	1	37.057	1	3.9079	-2.0262	1	8	16/16	5.688572	3446	1406.75	MS2	703.88	233	Glioma_grade 2_2.raw
62414289			lQDEIQNmk	Unambiguous	N-Term(iTRAQ4plex); M8(Oxidation); K9(iTRAQ4plex)	HCD	5.0114446	0	1	1	4.79413	4.7472	5.2783	5.22663	5.57905155	150274.88	151759.36	720437.8	793190.06	0.695077402	6.797392906	2	High	1552	Unique	Used	49234	1	23.684	1	3.1486	-0.3169	1	8	15/16	3.214328	1552	1422.75	MS2	711.879	210	Glioma_grade 2_2.raw
62414289			dGQVINETSQHHDDLE	Unambiguous	N-Term(iTRAQ4plex)	HCD	1.8678174	0	1	1	1.73411	1.7872	1.946	2.0056157	6.87418625	137626.27	133537.64	238659.5	267825.2	2.132361935	8.143686965	3	High	1365	Unique	Used	49056	1	22.438	1	7.159	-0.8419	1	2	25/60	13.2596	1365	1980.9	MS2	660.972	43	Glioma_grade 2_2.raw
62414289			kVESLQEEIAFLk	Unambiguous	N-Term(iTRAQ4plex); K1(iTRAQ4plex); K13(iTRAQ4plex)	HCD	6.195336	1	1	1	6.06181	6.3534	6.0445	6.3352866	2.717442706	115141.78	109856.52	697967.44	695972.6	3.322019043	0.202385304	3	High	4837	Unique	Used	92092	1	53.948	1	5.9585	-1.5251	1	1	22/48	8.633769	4837	1966.16	MS2	656.057	73	Glioma_grade 2_7rep.raw
62414289			kVESLQEEIAFLk	Unambiguous	N-Term(iTRAQ4plex); K1(iTRAQ4plex); K13(iTRAQ4plex)	HCD	6.813737	1	1	1	6.72141	6.8429	6.7851	6.9077306	1.168891538	102641.22	100818.336	689894	696425.9	1.267056375	0.66633261	2	High	4831	Unique	Used	92087	1	53.895	1	5.4227	0.9031	1	13	22/24	24.70155	4831	1966.16	MS2	983.584	93	Glioma_grade 2_7rep.raw
62414289			iLLAELEQLk	Unambiguous	N-Term(iTRAQ4plex); K10(iTRAQ4plex)	HCD	7.5166364	0	1	1	6.86028	7.6633	7.3853	8.249826	7.676611403	100010.234	89530.29	686097.8	738609.4	7.819372145	5.21248274	2	High	7703	Unique	Used	55002	1	68.754	1	4.3221	1.055	1	8	18/18	16.97319	7703	1457.92	MS2	729.464	65	Glioma_grade 2_2.raw
62414289			iLLAELEQLk	Unambiguous	N-Term(iTRAQ4plex); K10(iTRAQ4plex)	HCD	3.410234	0	1	1	2.91999	3.4705	3.3595	3.992903	12.83570525	99738.484	83917.69	291235.34	335075.2	12.18253705	9.899070928	2	High	7617	Unique	Used	54924	1	68.057	1	4.023	-0.5359	1	25	17/18	45.43974	7617	1457.92	MS2	729.462	63	Glioma_grade 2_2.raw
62414289			iSLPLPNFSSLNLR	Unambiguous	N-Term(iTRAQ4plex)	HCD	4.4714417	0	1	1	4.40713	4.5362	4.4085	4.5376425	1.666703642	80666.4	78370.98	355506.88	355619.47	2.041164219	0.022390719	2	High	8031	Unique	Used	55302	1	71.396	1	4.3284	1.5401	1	16	18/26	21.33941	8031	1715	MS2	858.004	160	Glioma_grade 2_2.raw
62414289			vESLQEEIAFLk	Unambiguous	N-Term(iTRAQ4plex); K12(iTRAQ4plex)	HCD	3.013533	0	1	1	2.68376	3.3471	2.7461	3.424819	12.77083673	72851.55	58413.113	195516.05	200054.34	15.55562096	1.622495371	2	High	6929	Unique	Used	54296	1	62.582	1	5.7626	1.9918	1	37	19/22	31.46118	6929	1693.96	MS2	847.486	195	Glioma_grade 2_2.raw
62414289			iLLAELEQLk	Unambiguous	N-Term(iTRAQ4plex); K10(iTRAQ4plex)	HCD	7.5744586	0	1	1	7.09879	8.2136	7.0221	8.124831	8.430393678	67581.25	58408.47	479744.9	474558.97	10.29629233	0.768519627	2	High	7408	Unique	Used	62717	1	68.465	1	4.348	1.8085	1	15	17/18	18.91742	7408	1457.92	MS2	729.464	63	Glioma_grade 2_3.raw
62414289			iLLAELEQLk	Unambiguous	N-Term(iTRAQ4plex); K10(iTRAQ4plex)	HCD	5.0074286	0	1	1	4.72053	5.0756	4.9441	5.315903	4.965345549	61134.637	56858.45	288587.94	302254.03	5.125250813	3.271055681	2	High	7251	Unique	Used	62583	1	67.044	1	4.2311	-0.7034	1	25	17/18	4.264225	7251	1457.92	MS2	729.462	63	Glioma_grade 2_3.raw
62414289			qVQSLTcEVDALk	Unambiguous	N-Term(iTRAQ4plex); C7(Methylthio); K13(iTRAQ4plex)	HCD	2.4968183	0	1	1	2.37842	2.4594	2.533	2.6192455	4.116786854	54918.53	53110.375	130619.44	139109.11	2.367067706	4.451218254	2	High	5732	Unique	Used	53173	1	53.737	1	6.0502	2.6479	1	100	20/24	39.86578	5732	1767.93	MS2	884.467	233	Glioma_grade 2_2.raw

62414289			eTNLDSLPLVDTHSk	Unambiguous	N-Term(iTRAQ4plex); K15(iTRAQ4plex)	HCD	3.0530062	0	1	1	2.81537	2.9174	3.1839	3.2992525	7.385579997	54012.91	52124.1	152066.38	171970.58	2.516728819	8.68690707	3	High	3592	Unique	Used	59190	1	38.387	1	5.1057	0.2661	1	3	27/56	4.580158	3592	1957.05	MS2	653.021	198	Glioma_grade 2_3.raw
62414289			kVESLQEEIAFLk	Unambiguous	N-Term(iTRAQ4plex); K1(iTRAQ4plex); K13(iTRAQ4plex)	HCD	5.627781	1	1	1	4.74869	6.0876	5.2691	6.754725	15.49443008	46596.074	36347.965	221270.36	245520.5	17.4732445	7.346946956	2	High	5081	Unique	Used	82140	1	52.796	1	5.8425	-0.1524	1	39	20/24	30.87773	5081	1966.16	MS2	983.583	91	Glioma_grade 2_6.raw
62414289			ekLQEEMLQR	Unambiguous	N-Term(iTRAQ4plex); K2(iTRAQ4plex)	HCD	2.1302137	1	1	1	2.04751	2.2907	1.9868	2.2227426	6.702136955	44227.664	39532.812	90556.45	87871.26	7.926797565	2.128274871	2	High	1453	Unique	Used	78937	1	22.604	1	3.8427	-4.6184	1	80	16/18	0	1453	1591.86	MS2	796.436	281	Glioma_grade 2_6.raw
62414289			lQDEIQNMkEEMAR	Unambiguous	N-Term(iTRAQ4plex); K9(iTRAQ4plex)	HCD	4.1474276	1	1	1	4.07607	4.2564	4.043	4.2219396	2.543268496	31270.697	29945.61	127461.66	126428.55	3.061203948	0.575460619	3	High	5396	Unique	Used	75775	1	54.078	1	3.7099	-0.1	1	10	27/52	19.84013	5396	2023.02	MS2	675.011	139	Glioma_grade 2_5.raw
62414289			iSLPLPNFSSLNLR	Unambiguous	N-Term(iTRAQ4plex)	HCD	3.0281343	0	1	1	2.85735	3.0515	3.0062	3.2105296	4.796344504	30904.455	28937.9	88304.71	92905.984	4.64742531	3.590949271	2	High	5600	Unique	Used	87843	1	72.139	1	4.882	-1.7341	1	83	18/26	18.64329	5600	1714.99	MS2	858.001	161	Glioma_grade 2_1rep.raw
62414289			eTNLDSLPLVDTHSk	Unambiguous	N-Term(iTRAQ4plex); K15(iTRAQ4plex)	HCD	3.6814177	0	1	1	4.12824	2.7997	4.9816	3.3783925	24.73179562	27191.209	40094.844	112251.945	135456.12	27.12076992	13.2477152	3	High	3220	Unique	Used	73842	1	36.13	1	6.2246	-1.7922	1	5	30/56	13.79123	3220	1957.04	MS2	653.02	189	Glioma_grade 2_5.raw
62414289			lQDEIQNMkEEMAR	Unambiguous	N-Term(iTRAQ4plex); K9(iTRAQ4plex)	HCD	4.9677052	1	1	1	4.71798	4.975	4.9608	5.231033	4.215632315	23500.969	22286.74	110877.164	116582.67	3.750305829	3.547353317	2	High	5401	Unique	Used	75780	1	54.108	1	5.135	-1.3001	1	54	20/26	20.31017	5401	2023.02	MS2	1012.01	175	Glioma_grade 2_5.raw
62414289			eTNLDSLPLVDTHSk	Unambiguous	N-Term(iTRAQ4plex); K15(iTRAQ4plex)	HCD	3.1446512	0	1	1	3.38928	2.492	4.0323	2.9647844	20.31732291	21832.63	29694.021	73996.984	88036.37	21.57657359	12.25345869	3	High	3873	Unique	Used	67124	1	39.933	1	5.3009	-0.2017	1	16	26/56	8.0186	3873	1957.05	MS2	653.021	183	Glioma_grade 2_4.raw
62414289			lLQDSVDFSLADAINTEFk	Unambiguous	N-Term(iTRAQ4plex); K19(iTRAQ4plex)	HCD	4.46428	0	1	1	3.60057	5.0634	4.0382	5.6789117	20.61862316	20619.896	14662.655	74243.28	83267.92	23.87812326	8.102768745	2	High	8582	Unique	Used	55801	1	75.936	1	7.2613	-0.0342	1	37	25/36	22.7095	8582	2414.27	MS2	1207.64	153	Glioma_grade 2_2.raw
62414289			kVESLQEEIAFLk	Unambiguous	N-Term(iTRAQ4plex); K1(iTRAQ4plex); K13(iTRAQ4plex)	HCD	1.9448379	1	1	1	2.07402	2.0004	1.8872	1.8202391	5.832518231	18197.512	18866.832	37741.996	34342.145	2.553832928	6.670143152	3	High	5070	Unique	Used	82131	1	52.688	1	6.2513	0.5237	1	40	21/48	30.06196	5070	1966.16	MS2	656.058	87	Glioma_grade 2_6.raw
62414289			eTNLDSLPLVDTHSk	Unambiguous	N-Term(iTRAQ4plex); K15(iTRAQ4plex)	HCD	1.7810805	0	1	1	1.5914	1.7922	1.7712	1.9946929	9.231190955	17300.662	15362.333	27532.29	30643.137	8.392406024	7.562302925	3	High	3074	Unique	Used	73707	1	35.015	1	5.2186	0.8275	1	33	24/56	59.16742	3074	1957.05	MS2	653.021	215	Glioma_grade 2_5.raw
62414289			eTNLDSLPLVDTHSk	Unambiguous	N-Term(iTRAQ4plex); K15(iTRAQ4plex)	HCD	5.4125385	0	1	1	4.92189	5.447	5.3814	5.95554	7.796502838	10605.244	9582.846	52197.793	57071.02	7.162089716	6.307182742	2	High	3584	Unique	Used	59183	1	38.317	1	5.798	0.3354	1	62	22/28	0	3584	1957.05	MS2	979.028	216	Glioma_grade 2_3.raw
62414289			eTNLDSLPLVDTHSk	Unambiguous	N-Term(iTRAQ4plex); K15(iTRAQ4plex)	HCD	4.9101944	0	1	1	4.52886	5.0441	4.79	5.334916	7.008817494	10210.713	9167.712	46242.86	48908.973	7.611692693	3.962564929	2	High	3773	Unique	Used	67032	1	39.135	1	5.4197	1.271	1	100	21/28	0	3773	1957.05	MS2	979.029	219	Glioma_grade 2_4.raw
62414289			eTNLDSLPLVDTHSk	Unambiguous	N-Term(iTRAQ4plex); K15(iTRAQ4plex)	HCD	4.1957893	0	1	1	4.1301	4.0694	4.324	4.2605157	2.783023242	7558.1064	7670.7573	31215.723	32681.383	1.046121587	3.243896914	2	High	3204	Unique	Used	73828	1	35.99	1	5.5073	-0.6002	1	100	21/28	29.26188	3204	1957.05	MS2	979.027	212	Glioma_grade 2_5.raw
62414289			iLLAELEQLk	Unambiguous	N-Term(iTRAQ4plex); K10(iTRAQ4plex)	HCD	5.8918576	0	1	1	6.15098	5.4158	6.4325	5.6637063	7.782177581	5070.6367	5758.94	31189.379	32616.943	8.98842022	3.164075763	3	High	7628	Unique	Used	54934	1	68.14	1	3.9588	0.7736	1	36	22/36	3.374067	7628	1457.92	MS2	486.645	66	Glioma_grade 2_2.raw
62414289			iLLAELEQLk	Unambiguous	N-Term(iTRAQ4plex); K10(iTRAQ4plex)	HCD	6.7171154	0	1	1	7.79256	5.2835	8.8316	5.9879456	23.34366393	4700.562	6932.8203	36629.42	41513.348	27.13647571	8.838843814	3	High	7712	Unique	Used	55010	1	68.823	1	4.0832	-3.999	1	23	21/36	0	7712	1457.91	MS2	486.642	58	Glioma_grade 2_2.raw
62414289			kVESLQEEIAFLk	Unambiguous	N-Term(iTRAQ4plex); K1(iTRAQ4plex); K13(iTRAQ4plex)	HCD	5.7122226	1	1	1	5.29222	5.6298	5.7897	6.159016	6.295445598	3964.2397	3726.5266	20979.635	22951.736	4.371178071	6.348474717	4	High	4844	Unique	Used	92098	1	54.005	1	4.443	-3.2083	1	19	26/72	5.681725	4844	1966.15	MS2	492.294	90	Glioma_grade 2_7rep.raw
62414289			iSLPLPNFSSLNLR	Unambiguous	N-Term(iTRAQ4plex)	HCD	1.817139	0	1	1	1.6887	1.6128	2.0311	1.9398012	10.95843103	3143.089	3291.0435	5307.743	6383.97	3.252019764	13.01789412	3	High	8053	Unique	Used	55323	1	71.556	1	4.0454	-0.959	1	60	22/52	36.6222	8053	1715	MS2	572.337	145	Glioma_grade 2_2.raw
62414289			iLLAELEQLk	Unambiguous	N-Term(iTRAQ4plex); K10(iTRAQ4plex)	HCD	8.318296	0	1	1	9.02479	7.5372	9.2535	7.728256	10.47218771	2071.1064	2479.8623	18691.295	19165.01	12.70208994	1.769676617	3	High	7342	Unique	Used	70298	1	67.758	1	4.0912	0.334	1	100	20/36	11.83709	7342	1457.92	MS2	486.644	63	Glioma_grade 2_4.raw
63252888	NP_001017962 .1	P4HA1	prolyl 4-hydroxylase subunit alpha-1 isoform 2 precursor [Homo sapiens]	6	9	3.346628904	35.73758947	49.997348	19.1	1.26E-04	534	61.011	6.0112	Enzyme: Hydroxylase	Protein metabolism,Metabolism,Energy pathways	Oxidoreductase activity	Endoplasmic reticulum	-																								
63252888			dPEGFVGHPVNAFk	Unambiguous	N-Term(iTRAQ4plex); K14(iTRAQ4plex)	HCD	5.0492024	0	1	3	4.76947	5.1248	4.9789	5.349778	4.832628413	29193.627	27169.648	139237.98	145351.6	5.078375151	3.038046691	3	High	3119	Unique	Used	73749	1	35.352	1	4.6398	-0.5771	1	13	26/52	39.97609	3119	1801.95	MS2	601.32	146	Glioma_grade 2_5.raw
63252888			lLELDPEHQR	Unambiguous	N-Term(iTRAQ4plex)	HCD	6.429382	0	1	3	6.05385	6.7449	6.1461	6.847778	6.296072935	27064.06	24291.055	163841.89	166339.75	7.63628176	1.069867934	2	High	2859	Unique	Used	66183	1	32.074	1	3.4082	5.2683	1	49	15/18	0	2859	1393.76	MS2	697.385	245	Glioma_grade 2_4.raw
63252888			fHDIISDAEIEIVk	Unambiguous	N-Term(iTRAQ4plex); K14(iTRAQ4plex)	HCD	4.0252175	0	1	3	3.62805	4.1128	3.9479	4.475462	8.726987898	18615.926	16421.578	67539.43	73494.14	8.857014193	5.971083085	2	High	5832	Unique	Used	76168	1	57.6	1	6.0663	-3.3324	1	100	22/26	37.20169	5832	1917.05	MS2	959.029	218	Glioma_grade 2_5.raw
63252888			fHDIISDAEIEIVk	Unambiguous	N-Term(iTRAQ4plex); K14(iTRAQ4plex)	HCD	3.6417086	0	1	3	3.03596	3.8465	3.4801	4.409183	15.73935738	14578.612	11506.531	44260.086	50734.402	16.65537588	9.638522915	3	High	5853	Unique	Used	76187	1	57.77	1	6.1906	-1.0153	1	11	27/52	19.35802	5853	1917.06	MS2	639.69	213	Glioma_grade 2_5.raw
63252888			sFLTAEDcFELGk	Unambiguous	N-Term(iTRAQ4plex); C8(Methylthio); K13(iTRAQ4plex)	HCD	2.5958586	0	1	3	2.51668	2.4478	2.7481	2.6728654	5.323988005	14072.079	14468.368	35414.96	38672	1.963659779	6.217221143	2	High	7320	Unique	Used	54663	1	65.493	1	5.0675	-0.7987	1	100	21/24	25.62811	7320	1793.87	MS2	897.438	121	Glioma_grade 2_2.raw
63252888			lLELDPEHQR	Unambiguous	N-Term(iTRAQ4plex)	HCD	2.8597188	0	1	3	2.61422	2.7667	2.9476	3.1195378	7.658187954	13816.8545	13055.209	36120.324	40726.22	4.008361307	8.476270046	3	High	2437	Unique	Used	73116	1	30.231	1	3.5229	11.389	1	47	15/36	54.66223	2437	1393.77	MS2	465.262	323	Glioma_grade 2_5.raw
63252888			lNTEWSELENLVLk	Unambiguous	N-Term(iTRAQ4plex); K14(iTRAQ4plex)	HCD	2.4533262	0	1	3	2.56818	2.4037	2.5063	2.3458247	4.070984253	9299.796	9936.1045	23883.578	23308.36	4.678107534	1.723771329	2	High	8565	Unique	Used	55785	1	75.814	1	3.6493	-1.6888	1	64	19/26	20.86158	8565	1976.09	MS2	988.549	223	Glioma_grade 2_2.raw
63252888			vAYTEADYYHTELWMEQAL R	Unambiguous	N-Term(iTRAQ4plex)	HCD	3.346629	0	1	3	3.53253	3.0884	3.642	3.1841035	7.944677607	3418.6653	3910.3035	12076.527	12450.811	9.486756312	2.158071573	3	High	7441	Unique	Used	54765	1	66.614	1	7.6919	2.1506	1	80	30/76	24.53758	7441	2633.25	MS2	878.422	93	Glioma_grade 2_2.raw
63252888			lNTEWSELENLVLk	Unambiguous	N-Term(iTRAQ4plex); K14(iTRAQ4plex)	HCD	2.7576299	0	1	3	2.23131	2.9388	2.62	3.4508421	18.35526168	2345.2424	1780.6217	5232.962	6144.6445	19.35338228	11.33202977	3	High	8562	Unique	Used	55782	1	75.795	1	3.8623	-1.2015	1	34	19/52	15.05506	8562	1976.09	MS2	659.369	214	Glioma_grade 2_2.raw
66529396	NP_001018171 .1	PON2	serum paraoxonase/arylesterase 2 isoform 2 [Homo sapiens]	8	24	3.156114578	27.73947898	149.34526	40.35	1.05E-04	342	37.957	5.7192	Enzyme: Esterase	Metabolism,Energy pathways,Lipid metabolism	Hydrolase activity,Antioxidant activity	Plasma membrane	-																								
66529396			yIYVADILAHEIHVLEk	Unambiguous	N-Term(iTRAQ4plex); K17(iTRAQ4plex)	HCD	3.6476333	0	1	2	3.76982	3.5577	3.743	3.5323215	3.368481017	125023.46	132478.56	471316.06	467956.9	4.094377018	0.505770935	3	High	6709	Unique	Used	93383	1	75.104	1	7.7257	0.1533	1	4	35/64	10.04261	6709	2314.31	MS2	772.107	104	Glioma_grade 2_7rep.raw
66529396			fEEAENSLLHLk	Unambiguous	N-Term(iTRAQ4plex); K12(iTRAQ4plex)	HCD	3.9426584	0	1	2	3.66322	4.0496	3.8459	4.25157	6.432288658	119421.67	108027.25	437468	459285.44	7.084730628	3.440691518	2	High	5071	Unique	Used	75485	1	51.431	1	5.6789	0.3376	1	13	21/22	34.38072	5071	1717.94	MS2	859.472	202	Glioma_grade 2_5.raw
66529396			eVESVDLPHcHLIk	Unambiguous	N-Term(iTRAQ4plex); C10(Methylthio); K14(iTRAQ4plex)	HCD	2.6237762	0	1	2	2.53271	2.6035	2.6435	2.717385	2.939771643	77693.85	75581.69	196776.16	205384.55	1.948807564	3.027173363	3	High	4096	Unique	Used	91531	1	46.369	1	5.3976	-2.3101	1	8	30/52	23.35056	4096	1953.01	MS2	651.676	207	Glioma_grade 2_7rep.raw
66529396			eVESVDLPHcHLIk	Unambiguous	N-Term(iTRAQ4plex); C10(Methylthio); K14(iTRAQ4plex)	HCD	3.038193	0	1	2	2.82056	3.2471	2.8567	3.2887342	8.15099168	58767.812	51047.72	165758.4	167882.38	9.941998741	0.900298016	2	High	4107	Unique	Used	91541	1	46.45	1	6.3361	-2.7095	1	40	19/26	0	4107	1953.01	MS2	977.01	210	Glioma_grade 2_7rep.raw
66529396			yIYVADILAHEIHVLEk	Unambiguous	N-Term(iTRAQ4plex); K17(iTRAQ4plex)	HCD	4.249297	0	1	2	3.17072	4.0151	4.4343	5.615098	23.60597422	25669.139	20271.045	81389.586	113823.91	16.61738609	23.49686975	4	High	6703	Unique	Used	93378	1	75.05	1	7.1743	-3.7558	1	4	37/96	4.407796	6703	2314.3	MS2	579.33	107	Glioma_grade 2_7rep.raw
66529396			fEEAENSLLHLk	Unambiguous	N-Term(iTRAQ4plex); K12(iTRAQ4plex)	HCD	4.166003	0	1	2	3.45854	4.7013	3.7722	5.1276755	18.30154804	24006.318	17660.447	83026.84	90557.05	21.53854957	6.134973188	3	High	5040	Unique	Used	75457	1	51.183	1	4.6297	1.1469	1	13	30/44	4.934792	5040	1717.94	MS2	573.318	206	Glioma_grade 2_5.raw
66529396			lFVYDPNNPPSSEVLR	Unambiguous	N-Term(iTRAQ4plex)	HCD	2.4044151	0	1	2	2.23009	2.5688	2.2617	2.6052113	8.190539405	22702.846	19709.371	50629.496	51347.08	9.981588427	0.995147185	2	High	4330	Unique	Used	86916	1	58.069	1	4.9317	2.3528	1	100	18/30	13.99577	4330	1991.04	MS2	996.024	239	Glioma_grade 2_1rep.raw
66529396			yIYVADILAHEIHVLEk	Unambiguous	N-Term(iTRAQ4plex); K17(iTRAQ4plex)	HCD	3.5302272	0	1	2	3.3853	3.8189	3.2743	3.693713	7.211876457	17279.615	15317.749	58496.74	56579.363	8.511416766	2.356336795	2	High	6717	Unique	Used	93390	1	75.167	1	6.5169	0.7393	1	69	21/32	26.4646	6717	2314.31	MS2	1157.66	115	Glioma_grade 2_7rep.raw
66529396			fPGLHSFAPDkPGGILMMDL k	Unambiguous	N-Term(iTRAQ4plex); K11(iTRAQ4plex); K21(iTRAQ4plex)	HCD	2.9244792	0	1	2	2.67532	2.8834	2.9626	3.1930182	7.303940998	15391.733	14280.887	41177.832	45599.133	5.294353781	7.205441948	3	High	4761	Unique	Used	87226	1	62.863	1	5.4488	2.4313	1	53	26/80	15.92967	4761	2703.48	MS2	901.831	112	Glioma_grade 2_1rep.raw
66529396			lLIGTLYHR	Unambiguous	N-Term(iTRAQ4plex)	HCD	5.41329	0	1	2	5.64086	5.2234	5.6184	5.2025595	4.44300396	14672.588	15845.252	82766.055	82435.87	5.434189749	0.282655366	3	High	3372	Unique	Used	73984	1	37.257	1	4.7927	0.2043	1	42	19/32	0	3372	1229.75	MS2	410.588	161	Glioma_grade 2_5.raw
66529396			fPGLHSFAPDkPGGILMmDL k	Unambiguous	N-Term(iTRAQ4plex); K11(iTRAQ4plex); M18(Oxidation); K21(iTRAQ4plex)	HCD	3.5827165	0	1	2	3.50201	3.3764	3.7967	3.660534	5.116655598	14012.332	14533.397	49071.258	53199.996	2.581462151	5.709245801	3	High	4478	Unique	Used	87013	1	59.856	1	5.8821	1.2345	1	40	19/80	29.16819	4478	2719.47	MS2	907.162	131	Glioma_grade 2_1rep.raw
66529396			eVESVDLPHcHLIk	Unambiguous	N-Term(iTRAQ4plex); C10(Methylthio); K14(iTRAQ4plex)	HCD	3.8907914	0	1	2	4.81112	3.2408	4.8557	3.2708502	22.52551715	13387.466	19874.15	64408.645	65005.37	27.5800084	0.65209057	4	High	4082	Unique	Used	91519	1	46.246	1	5.8628	-0.2231	1	10	33/78	10.48648	4082	1953.02	MS2	489.01	217	Glioma_grade 2_7rep.raw
66529396			fPGLHSFAPDkPGGILMMDL k	Unambiguous	N-Term(iTRAQ4plex); K11(iTRAQ4plex); K21(iTRAQ4plex)	HCD	2.775461	0	1	2	2.53093	2.9692	2.6104	3.0623338	9.37270365	12580.83	10723.975	31841.186	32840.39	11.26801758	2.184683701	4	High	4758	Unique	Used	87224	1	62.827	1	6.354	1.0943	1	13	30/120	16.73257	4758	2703.48	MS2	676.624	119	Glioma_grade 2_1rep.raw
66529396			fPGLHSFAPDkPGGILmmDL k	Unambiguous	N-Term(iTRAQ4plex); K11(iTRAQ4plex); M17(Oxidation); M18(Oxidation); K21(iTRAQ4plex)	HCD	1.8939283	0	1	2	1.8487	2.0724	1.7347	1.9446329	7.54627729	10954.467	9771.866	20251.494	19002.693	8.069205359	4.499064803	4	High	4129	Unique	Used	86782	1	55.694	1	4.9056	3.1036	1	16	29/120	29.96078	4129	2735.47	MS2	684.623	146	Glioma_grade 2_1rep.raw
66529396			fPGLHSFAPDkPGGILmMDL k	Unambiguous	N-Term(iTRAQ4plex); K11(iTRAQ4plex); M17(Oxidation); K21(iTRAQ4plex)	HCD	2.9777822	0	1	2	2.40186	3.0378	2.9303	3.7061875	17.74793323	8368.396	6616.5854	20099.709	24522.305	16.53278201	14.01661351	3	High	4504	Unique	Used	96364	1	61.9	1	5.7652	2.3118	1	82	21/80	22.25271	4504	2719.47	MS2	907.163	131	Glioma_grade 2_8rep.raw
66529396			lLIGTLYHR	Unambiguous	N-Term(iTRAQ4plex)	HCD	2.8784626	0	1	2	2.39832	3.3598	2.5349	3.551101	19.55148202	7716.629	5508.34	18506.908	19560.672	23.61436351	3.914741469	3	High	3533	Unique	Used	86417	1	48.121	1	4.1247	0.5765	1	100	18/32	70.18159	3533	1229.75	MS2	410.588	146	Glioma_grade 2_1rep.raw
66529396			fEEAENSLLHLk	Unambiguous	N-Term(iTRAQ4plex); K12(iTRAQ4plex)	HCD	1.5741863	0	1	2	1.50748	1.5933	1.5561	1.6446855	3.683910662	7400.4414	7001.9326	11156.043	11515.978	3.913080925	2.245167992	3	High	5147	Unique	Used	75555	1	52.003	1	4.4692	1.9996	1	75	29/44	36.57278	5147	1717.94	MS2	573.318	235	Glioma_grade 2_5.raw
66529396			fPGLHSFAPDkPGGILMMDL k	Unambiguous	N-Term(iTRAQ4plex); K11(iTRAQ4plex); K21(iTRAQ4plex)	HCD	3.6883652	0	1	2	3.34887	3.6054	3.7654	4.053869	7.994889354	7300.4424	6781.0156	24448.215	27489.346	5.21665033	8.280729057	3	High	4757	Unique	Used	96511	1	65.263	1	5.936	2.2958	1	74	25/80	12.28981	4757	2703.48	MS2	901.831	113	Glioma_grade 2_8rep.raw
66529396			fPGLHSFAPDkPGGILMmDL k	Unambiguous	N-Term(iTRAQ4plex); K11(iTRAQ4plex); M18(Oxidation); K21(iTRAQ4plex)	HCD	5.1827974	0	1	2	6.77957	5.3821	4.9317	3.9151635	22.63555169	5459.6543	6877.2603	37014.13	26925.598	16.25039725	22.3137308	4	High	4512	Unique	Used	96369	1	62.005	1	5.577	1.3418	1	11	26/120	29.16039	4512	2719.47	MS2	680.623	132	Glioma_grade 2_8rep.raw
66529396			lLIGTLYHR	Unambiguous	N-Term(iTRAQ4plex)	HCD	2.572537	0	1	2	2.01174	2.8732	2.362	3.3734944	22.42464513	4780.5938	3347.19	9617.298	11291.728	24.94085896	11.32526027	3	High	3643	Unique	Used	95879	1	50.214	1	4.0343	-0.0935	1	100	19/32	50.16663	3643	1229.75	MS2	410.588	170	Glioma_grade 2_8rep.raw
66529396			fPGLHSFAPDkPGGILMMDL k	Unambiguous	N-Term(iTRAQ4plex); K11(iTRAQ4plex); K21(iTRAQ4plex)	HCD	3.0233502	0	1	2	3.47649	2.414	3.9008	2.708695	21.89323566	4196.716	6043.761	14589.844	16370.706	25.50775798	8.134607406	4	High	4737	Unique	Used	96497	1	65.033	1	5.6209	0.3719	1	31	30/120	14.39504	4737	2703.47	MS2	676.624	121	Glioma_grade 2_8rep.raw
66529396			fPGLHSFAPDkPGGILmmDL k	Unambiguous	N-Term(iTRAQ4plex); K11(iTRAQ4plex); M17(Oxidation); M18(Oxidation); K21(iTRAQ4plex)	HCD	2.4081802	0	1	2	2.1294	2.39	2.4244	2.721073	10.0232794	3250.5986	2896.218	6921.8223	7880.8203	8.153323637	9.162066629	4	High	4187	Unique	Used	96184	1	57.624	1	6.1423	1.3186	1	56	29/120	46.57216	4187	2735.47	MS2	684.622	148	Glioma_grade 2_8rep.raw
66529396			hELLPSVNDITAVGPAHFYA TNDHYFSDPFLk	Unambiguous	N-Term(iTRAQ4plex); K32(iTRAQ4plex)	HCD	3.274036	0	1	2	2.82393	4.0531	2.7313	3.9200606	20.73087679	1840.757	1282.5237	5198.1626	5027.5703	25.27666194	2.359282671	4	High	5230	Unique	Used	87577	1	67.919	1	6.659	6.4621	1	82	26/186	10.93085	5230	3903.98	MS2	976.751	60	Glioma_grade 2_1rep.raw
66529396			lVAVGLLGIALALLGER	Unambiguous	N-Term(iTRAQ4plex)	HCD	3.6181343	0	1	2	3.97153	3.3977	3.8758	3.3157983	9.101254166	1665.1261	1946.3232	6613.093	6453.615	11.01144498	1.726034978	2	High	8536	Unique	Used	71286	1	79.069	1	4.763	3.6154	1	100	20/32	30.77044	8536	1822.17	MS2	911.589	3	Glioma_grade 2_4.raw

66933005	NP_001019820.1	CANX	calnexin precursor [Homo sapiens]	11	32	2.18304348	18.74409204	221.405	31.25	1.80E-04	592	67.526	4.6021	Chaperone	Protein folding	Chaperone activity	Endoplasmic reticulum membrane	Golgi apparatus, Lysosome, Endoplasmic reticulum, Nuclear membrane, Mitochondrion, Plasma membrane																								
66933005			tPELNLDQFHDk	Unambiguous	N-Term(iTRAQ4plex); K12(iTRAQ4plex)	HCD	2.1572967	0	1	1	2.08308	2.2183	2.1	2.2363338	3.659467048	1926912	1809472.8	4013905.2	4046585	4.44504831	0.573367317	3	High	3262	Unique	Used	73880	1	36.468	1	5.623	-1.0525	1	0	28/44	7.010926	3262	1744.91	MS2	582.308	167	Glioma_grade 2_5.raw
66933005			wEVEEMk	Unambiguous	N-Term(iTRAQ4plex); K7(iTRAQ4plex)	HCD	1.8460064	0	1	1	1.70055	1.9133	1.7862	2.0096595	7.368168639	1526278.2	1356539.5	2595517.5	2726182.5	8.326810662	3.472353104	2	High	3060	Unique	Used	50654	1	34.349	1	3.2392	1.7779	1	4	12-Dec	7.225971	3060	1238.64	MS2	619.821	182	Glioma_grade 2_2.raw
66933005			wEVEEMk	Unambiguous	N-Term(iTRAQ4plex); K7(iTRAQ4plex)	HCD	2.005541	0	1	1	1.91777	1.9995	2.0114	2.0970461	3.656188436	1387570.9	1330887.4	2661047.5	2790932.2	2.948824871	3.369137715	2	High	2957	Unique	Used	50561	1	33.535	1	3.1009	-0.5874	1	4	12-Dec	8.181482	2957	1238.63	MS2	619.82	170	Glioma_grade 2_2.raw
66933005			gTLSGWILSk	Unambiguous	N-Term(iTRAQ4plex); K10(iTRAQ4plex)	HCD	2.4360013	0	1	1	2.33023	2.5195	2.3588	2.5503635	4.560145676	701854.9	649142	1635482	1655548	5.517947383	0.862271366	2	High	5608	Unique	Used	61057	1	54.158	1	4.4273	2.0807	1	3	17/18	6.174044	5608	1349.81	MS2	675.407	183	Glioma_grade 2_3.raw
66933005			kIPNPDFFEDLEPFR	Unambiguous	N-Term(iTRAQ4plex); K1(iTRAQ4plex)	HCD	2.62509	1	1	1	2.04689	2.4657	2.7574	3.3216085	20.22333752	667100.4	553780.3	1365480.2	1839441.4	13.12649322	20.91415768	3	High	5844	Unique	Used	92831	1	64.607	1	6.0791	0.7428	1	0	27/56	5.987562	5844	2152.13	MS2	718.049	149	Glioma_grade 2_7rep.raw
66933005			iVDDWANDGWGLk	Unambiguous	N-Term(iTRAQ4plex); K13(iTRAQ4plex)	HCD	2.476916	0	1	1	2.36276	2.6731	2.3035	2.606063	7.265285844	634309.3	560673.8	1498721.9	1461151.1	8.714459876	1.795115362	2	High	6542	Unique	Used	61930	1	61.399	1	5.5863	2.4528	1	3	21/24	6.271607	6542	1776.92	MS2	888.964	205	Glioma_grade 2_3.raw
66933005			gTLSGWILSk	Unambiguous	N-Term(iTRAQ4plex); K10(iTRAQ4plex)	HCD	2.9172194	0	1	1	2.8027	2.8261	3.0076	3.0326903	4.099769108	580446.06	575639.6	1626818.8	1745736.8	0.587963432	4.986587869	2	High	5520	Unique	Used	60979	1	53.414	1	4.2602	1.7189	1	2	18/18	6.044792	5520	1349.81	MS2	675.406	185	Glioma_grade 2_3.raw
66933005			iVDDWANDGWGLk	Unambiguous	N-Term(iTRAQ4plex); K13(iTRAQ4plex)	HCD	2.4326372	0	1	1	2.24646	2.4406	2.4253	2.6348977	6.515154578	334389.62	307794.72	751193.94	811007.6	5.856709036	5.414748803	2	High	6782	Unique	Used	54158	1	61.497	1	5.3243	1.9719	1	7	22/24	5.282023	6782	1776.92	MS2	888.963	200	Glioma_grade 2_2.raw
66933005			tPELNLDQFHDk	Unambiguous	N-Term(iTRAQ4plex); K12(iTRAQ4plex)	HCD	2.0127537	0	1	1	1.94116	1.9978	2.0273	2.0864348	3.006613545	256169.11	248911.62	497265.3	519337.84	2.032079265	3.070547807	2	High	3207	Unique	Used	73830	1	36.026	1	5.4575	-0.73	1	10	18/22	10.50029	3207	1744.91	MS2	872.958	187	Glioma_grade 2_5.raw
66933005			kIPNPDFFEDLEPFR	Unambiguous	N-Term(iTRAQ4plex); K1(iTRAQ4plex)	HCD	2.5517936	1	1	1	2.3732	2.5384	2.5643	2.742823	5.92326102	203469.53	190229.23	482872.9	521765.1	4.756075897	5.474795569	2	High	5868	Unique	Used	92849	1	64.854	1	4.5391	-0.754	1	13	19/28	5.23647	5868	2152.13	MS2	1076.57	175	Glioma_grade 2_7rep.raw
66933005			tPYTIMFGPDk	Unambiguous	N-Term(iTRAQ4plex); K11(iTRAQ4plex)	HCD	1.707985	0	1	1	1.57202	1.8103	1.6191	1.8645632	8.312226362	186788.6	162203.17	293634.62	302438.1	9.96271303	2.088671465	2	High	5228	Unique	Used	60709	1	51.126	1	3.6492	1.169	1	13	14/20	17.92344	5228	1557.82	MS2	779.416	259	Glioma_grade 2_3.raw
66933005			tPYTIMFGPDk	Unambiguous	N-Term(iTRAQ4plex); K11(iTRAQ4plex)	HCD	2.5378265	0	1	1	2.47862	2.731	2.3625	2.6030629	6.242218151	171504.11	155657.33	425093.2	405185.8	6.850052743	3.390825864	2	High	5315	Unique	Used	52779	1	50.739	1	4.452	-1.0251	1	18	16/20	6.5673	5315	1557.82	MS2	779.414	208	Glioma_grade 2_2.raw
66933005			tPELNLDQFHDk	Unambiguous	N-Term(iTRAQ4plex); K12(iTRAQ4plex)	HCD	1.897325	0	1	1	1.7778	1.8705	1.9228	2.0230799	5.394327798	162255.27	154215.81	288457.6	311990.9	3.592591578	5.54270883	2	High	3298	Unique	Used	73914	1	36.726	1	5.2608	0.3194	1	13	20/22	4.096525	3298	1744.91	MS2	872.959	187	Glioma_grade 2_5.raw
66933005			gTLSGWILSk	Unambiguous	N-Term(iTRAQ4plex); K10(iTRAQ4plex)	HCD	2.7127953	0	1	1	3.21717	2.5898	2.8656	2.3067753	14.16640343	155105.97	192678.19	499001.94	444465.28	15.27819527	8.174792148	2	High	5707	Unique	Used	68815	1	54.318	1	4.4757	0.8146	1	12	17/18	13.42007	5707	1349.8	MS2	675.406	204	Glioma_grade 2_4.raw
66933005			kIPNPDFFEDLEPFR	Unambiguous	N-Term(iTRAQ4plex); K1(iTRAQ4plex)	HCD	2.20879	1	1	1	2.13987	2.2565	2.1636	2.2814634	3.12813687	154383.73	146405.3	330361.5	334018.3	3.751201935	0.77839455	2	High	5772	Unique	Used	92780	1	63.815	1	4.4429	-1.0943	1	26	16/28	30.26942	5772	2152.13	MS2	1076.57	176	Glioma_grade 2_7rep.raw
66933005			tPYTImFGPDk	Unambiguous	N-Term(iTRAQ4plex); M6(Oxidation); K11(iTRAQ4plex)	HCD	1.9493798	0	1	1	1.93606	1.9327	1.9661	1.9626753	0.89358119	142462.14	142708.52	275815.44	280090.5	0.122184357	1.08756669	2	High	3975	Unique	Used	59545	1	41.355	1	4.0917	0.8978	1	26	15/20	21.60797	3975	1573.82	MS2	787.413	178	Glioma_grade 2_3.raw
66933005			tPELNLDQFHDk	Unambiguous	N-Term(iTRAQ4plex); K12(iTRAQ4plex)	HCD	1.95104	0	1	1	1.72204	2.0716	1.8509	2.22652	11.43061141	132145.28	109849.664	227559.34	244582.5	13.02951293	5.098972747	3	High	3175	Unique	Used	73801	1	35.777	1	5.5488	-1.997	1	5	30/44	11.63994	3175	1744.91	MS2	582.307	161	Glioma_grade 2_5.raw
66933005			yDGkWEVEEMk	Unambiguous	N-Term(iTRAQ4plex); K4(iTRAQ4plex); K11(iTRAQ4plex)	HCD	2.7945302	1	1	1	2.65667	3.1773	2.4745	2.959401	11.09572465	117158.87	97962.266	311252.88	289909.6	12.61991185	5.020931453	3	High	2891	Unique	Used	80238	1	33.961	1	3.5822	0.5595	1	8	19/40	8.735604	2891	1845.94	MS2	615.986	167	Glioma_grade 2_6.raw
66933005			kPEDWDERPk	Unambiguous	N-Term(iTRAQ4plex); K1(iTRAQ4plex); K10(iTRAQ4plex)	HCD	3.0925937	0	1	1	2.96458	3.0413	3.1426	3.2239203	3.674983446	72314.586	70490.25	214382.36	227254.95	1.806662005	4.122068256	2	High	1075	Unique	Used	84703	1	19.876	1	3.6367	0.2601	1	46	14/18	0	1075	1731.94	MS2	866.473	135	Glioma_grade 2_1rep.raw
66933005			tPYTIMFGPDk	Unambiguous	N-Term(iTRAQ4plex); K11(iTRAQ4plex)	HCD	2.6827908	0	1	1	2.56532	2.6661	2.6988	2.8048782	3.678369187	65317.77	62848.38	167560.9	176282.06	2.724779381	3.586981322	2	High	5141	Unique	Used	60628	1	50.451	1	4.2442	-0.4765	1	83	16/20	13.92854	5141	1557.82	MS2	779.415	197	Glioma_grade 2_3.raw
66933005			aPVPTGEVYFADSFDR	Unambiguous	N-Term(iTRAQ4plex)	HCD	2.3878617	0	1	1	2.40504	2.5065	2.274	2.3699632	4.018261665	56874.926	54572.68	136786.22	129335.24	2.921433339	3.959574312	2	High	4311	Unique	Used	86900	1	57.899	1	5.0795	-0.5799	1	65	18/30	39.07844	4311	1914.93	MS2	957.971	119	Glioma_grade 2_1rep.raw
66933005			yDGkWEVEEMk	Unambiguous	N-Term(iTRAQ4plex); K4(iTRAQ4plex); K11(iTRAQ4plex)	HCD	2.7731092	1	1	1	2.61457	2.8668	2.6876	2.9469433	5.547680878	56011.99	51083.285	146447.33	150539.55	6.508449094	1.948662858	2	High	2889	Unique	Used	80237	1	33.928	1	4.2648	1.691	1	71	17/20	7.027957	2889	1845.94	MS2	923.476	169	Glioma_grade 2_6.raw
66933005			kIPNPDFFEDLEPFR	Unambiguous	N-Term(iTRAQ4plex); K1(iTRAQ4plex)	HCD	1.9541494	1	1	1	1.60709	1.8834	2.0145	2.3608863	15.9172465	38347.242	32720.969	61627.41	77250.49	11.19593623	15.9092063	3	High	5949	Unique	Used	92908	1	65.707	1	6.302	-1.4693	1	12	25/56	20.86666	5949	2152.13	MS2	718.048	150	Glioma_grade 2_7rep.raw
66933005			gTLSGWILSk	Unambiguous	N-Term(iTRAQ4plex); K10(iTRAQ4plex)	HCD	1.6820601	0	1	1	1.8512	1.6789	1.6855	1.5286591	7.815856364	36172.9	39885.12	66963.41	60970.75	6.902456665	6.624423881	2	High	5664	Unique	Used	53110	1	53.214	1	3.8708	-0.0898	1	19	17/18	10.02095	5664	1349.8	MS2	675.405	215	Glioma_grade 2_2.raw
66933005			gTLSGWILSk	Unambiguous	N-Term(iTRAQ4plex); K10(iTRAQ4plex)	HCD	2.011962	0	1	1	2.02665	2.1556	1.8769	1.996337	5.685426005	34322.664	32269.064	69560.09	64419.93	4.361245847	5.425647783	2	High	5427	Unique	Used	60896	1	52.631	1	3.696	2.2615	1	17	17/18	14.90677	5427	1349.81	MS2	675.407	179	Glioma_grade 2_3.raw
66933005			kIPNPDFFEDLEPFR	Unambiguous	N-Term(iTRAQ4plex); K1(iTRAQ4plex)	HCD	1.7107112	1	1	1	1.67523	1.6359	1.7874	1.7453544	3.983494581	22631.25	23175.916	37912.65	40450.184	1.681557956	4.579485982	3	High	5749	Unique	Used	92762	1	63.589	1	5.2436	-2.235	1	37	23/56	58.94062	5749	2152.13	MS2	718.047	179	Glioma_grade 2_7rep.raw
66933005			kIPNPDFFEDLEPFR	Unambiguous	N-Term(iTRAQ4plex); K1(iTRAQ4plex)	HCD	1.5099562	1	1	1	1.4153	1.7754	1.2984	1.6286949	13.9606272	21171.195	16877.178	29963.605	27487.773	15.9603594	6.09446686	3	High	6310	Unique	Used	83154	1	64.213	1	5.2053	1.7638	1	26	24/56	36.8879	6310	2152.14	MS2	718.05	168	Glioma_grade 2_6.raw
66933005			iVDDWANDGWGLk	Unambiguous	N-Term(iTRAQ4plex); K13(iTRAQ4plex)	HCD	1.8655326	0	1	1	2.29321	1.7716	1.9871	1.5351261	16.99573248	16066.139	20796.037	36843.055	31924.541	18.14620466	10.11498091	3	High	6552	Unique	Used	61939	1	61.478	1	4.3007	1.1743	1	7	23/48	13.46471	6552	1776.92	MS2	592.978	228	Glioma_grade 2_3.raw
66933005			iPDPEAVkPDDWDEDAPAk	Unambiguous	N-Term(iTRAQ4plex); K8(iTRAQ4plex); K19(iTRAQ4plex)	HCD	3.0164707	0	1	1	2.78034	3.0446	2.9908	3.275037	6.72343838	10429.011	9523.92	28996.236	31191.191	6.415057354	5.1574478	2	High	4228	Unique	Used	81411	1	45.259	1	3.9378	-0.3028	1	93	17/36	9.451672	4228	2540.29	MS2	1270.65	162	Glioma_grade 2_6.raw
66933005			wkPPMIDNPSYQGIWkPR	Unambiguous	N-Term(iTRAQ4plex); K2(iTRAQ4plex); K16(iTRAQ4plex)	HCD	2.0295858	0	1	1	1.87315	2.0146	2.0436	2.19783	6.550741829	7604.988	7071.176	14245.293	15541.241	5.143879355	6.152938901	4	High	4021	Unique	Used	86708	1	54.444	1	5.4744	0.0503	1	24	26/102	42.6365	4021	2645.44	MS2	662.115	60	Glioma_grade 2_1rep.raw
66933005			iVDDWANDGWGLk	Unambiguous	N-Term(iTRAQ4plex); K13(iTRAQ4plex)	HCD	2.4359956	0	1	1	2.09194	2.4619	2.414	2.8409073	12.51266796	7423.795	6308.1157	15530.102	17920.773	11.49008927	10.10711783	3	High	6773	Unique	Used	54150	1	61.42	1	4.2586	0.9682	1	29	22/48	12.74754	6773	1776.92	MS2	592.977	230	Glioma_grade 2_2.raw
66933005			wkPPMIDNPSYQGIWkPR	Unambiguous	N-Term(iTRAQ4plex); K2(iTRAQ4plex); K16(iTRAQ4plex)	HCD	2.145129	0	1	1	1.78989	2.4908	1.8967	2.6394727	19.20833032	3324.261	2388.821	5950.0693	6305.2275	23.15583664	4.098387427	4	High	4090	Unique	Used	96130	1	56.3	1	5.1739	1.5269	1	65	30/102	27.54975	4090	2645.44	MS2	662.116	45	Glioma_grade 2_8rep.raw
67189747	NP_001019833.1	RPL6	60S ribosomal protein L6 [Homo sapiens]	3	3	2.09427762	22.54191784	13.53203	12.85	6.25E-05	288	32.708	10.58	Ribosomal subunit	Protein metabolism	Structural constituent of ribosome	Nucleolus	Mitochondrion, Ribosome																								
67189747			aVDSQILPk	Unambiguous	N-Term(iTRAQ4plex); K9(iTRAQ4plex)	HCD	1.6213335	0	1	1	1.44598	1.7441	1.5195	1.8328447	11.16869237	451186.88	374058.44	652406.8	685591.06	13.21741345	3.507451839	2	High	1753	Unique	Used	49424	1	25.081	1	3.1877	-0.2221	1	6	14/16	41.07792	1753	1258.76	MS2	629.884	209	Glioma_grade 2_2.raw
67189747			vLATVTkPVGGDk	Unambiguous	N-Term(iTRAQ4plex); K7(iTRAQ4plex); K13(iTRAQ4plex)	HCD	2.5652113	0	1	1	2.43436	2.6194	2.5149	2.706008	4.626310791	129355.4	120218.97	314897.5	325313.5	5.177159505	2.300874003	2	High	1511	Unique	Used	78992	1	23.007	1	5.3553	-7.6321	1	62	19/24	21.08248	1511	1717.05	MS2	859.026	102	Glioma_grade 2_6.raw
67189747			aSITPGTILIILTGR	Unambiguous	N-Term(iTRAQ4plex)	HCD	2.0942776	0	1	1	2.04246	2.1155	2.0738	2.1479454	2.21034661	34906.883	33701.94	71295.99	72389.93	2.483713695	1.076698945	2	High	6037	Unique	Used	88189	1	76.534	1	4.989	1.2597	1	72	21/28	29.01093	6037	1670.04	MS2	835.521	34	Glioma_grade 2_1rep.raw
68160915	NP_001020241.1	RPS14	40S ribosomal protein S14 [Homo sapiens]	2	3	2.174393892	20.74084089	37.109229	20.53	1.47E-04	151	16.263	10.052	Ribosomal subunit	Protein metabolism	Structural constituent of ribosome	Ribosome	Cytoplasm, Nucleolus																								
68160915			eLGITALHIk	Unambiguous	N-Term(iTRAQ4plex); K10(iTRAQ4plex)	HCD	2.174394	0	1	1	2.0419	2.2298	2.1237	2.319082	5.562692162	198664.53	181924.31	405652.66	421897.44	6.220425738	2.776096359	2	High	3200	Unique	Used	80519	1	36.394	1	4.606	-0.256	1	17	17/18	24.13119	3200	1382.86	MS2	691.934	112	Glioma_grade 2_6.raw
68160915			eLGITALHIk	Unambiguous	N-Term(iTRAQ4plex); K10(iTRAQ4plex)	HCD	2.4582448	0	1	1	2.65177	2.302	2.6383	2.2902453	8.158793947	85893.19	98944.734	227769.14	226607.72	9.985867692	0.361483178	3	High	3167	Unique	Used	80489	1	36.132	1	4.6524	1.7204	1	11	21/36	8.570771	3167	1382.86	MS2	461.626	107	Glioma_grade 2_6.raw
68160915			aDRDESSPYAAMLAAQDVAQR	Unambiguous	N-Term(iTRAQ4plex)	HCD	1.6103147	1	1	1	1.23298	1.6751	1.5626	2.1229653	22.313138	24216.232	17824.307	29858.111	37840.387	21.501977	16.67487952	3	High	4845	Unique	Used	60352	1	48.168	1	7.0516	-1.7779	1	11	29/80	15.31885	4845	2409.15	MS2	803.723	95	Glioma_grade 2_3.raw
68799814	NP_001020376.1	AP2M1	AP-2 complex subunit mu isoform b [Homo sapiens]	10	21	0.456290394	20.75322934	128.31945	34.64	1.34E-04	433	49.358	9.5396	Adapter molecule	Transport	Receptor signaling complex scaffold activity	Plasma membrane	-																								
68799814			tFITQQGIk	Unambiguous	N-Term(iTRAQ4plex); K9(iTRAQ4plex)	HCD	0.4562904	0	1	2	0.41589	0.4932	0.4251	0.50419813	0.099042885	6745167.5	5687636	2805273.5	2867695.5	12.0292691	1.55611707	2	High	2033	Unique	Used	49690	1	26.997	1	4.193	0.5804	1	1	16/16	12.3519	2033	1323.79	MS2	662.398	262	Glioma_grade 2_2.raw
68799814			aSENAIVWk	Unambiguous	N-Term(iTRAQ4plex); K9(iTRAQ4plex)	HCD	0.6309933	0	1	2	0.57965	0.6678	0.5991	0.6901426	0.083768343	871036.94	756096.9	504895.94	521814.66	9.989944246	2.330421375	2	High	2276	Unique	Used	49919	1	28.721	1	4.2497	-3.5688	1	9	15/16	62.96461	2276	1305.74	MS2	653.372	294	Glioma_grade 2_2.raw
68799814			tFITQQGIk	Unambiguous	N-Term(iTRAQ4plex); K9(iTRAQ4plex)	HCD	0.42555875	0	1	2	0.44129	0.3771	0.4823	0.412116	0.104179571	833138.06	975014.44	367656.03	401819.06	11.09660279	6.278802392	2	High	1975	Unique	Used	57686	1	26.171	1	3.8931	10.447	1	10	16/16	17.82489	1975	1323.8	MS2	662.404	222	Glioma_grade 2_3.raw
68799814			lNYSDHDVIk	Unambiguous	N-Term(iTRAQ4plex); K10(iTRAQ4plex)	HCD	0.41205236	0	1	2	0.36119	0.3399	0.4887	0.459919	0.176802915	730264.06	775939.3	263765.4	356869.22	4.288567241	21.21516923	3	High	1513	Unique	Used	72265	1	23.275	1	3.4009	4.5087	1	2	18/36	14.73919	1513	1491.81	MS2	497.942	260	Glioma_grade 2_5.raw
68799814			lNYSDHDVIk	Unambiguous	N-Term(iTRAQ4plex); K10(iTRAQ4plex)	HCD	0.4321593	0	1	2	0.41345	0.4191	0.4451	0.45112193	0.043236645	622316.94	613967.94	257297.61	276974.4	0.955060538	5.208429931	2	High	1514	Unique	Used	72266	1	23.278	1	4.6312	3.6176	1	12	18/18	10.50215	1514	1491.81	MS2	746.409	236	Glioma_grade 2_5.raw
68799814			sNFkPSLLAQk	Unambiguous	N-Term(iTRAQ4plex); K4(iTRAQ4plex); K11(iTRAQ4plex)	HCD	0.5020728	0	1	2	0.46944	0.507	0.4976	0.53731626	0.055652917	481050.47	445453.78	225823.05	239349.56	5.433469061	4.112317338	2	High	2148	Unique	Used	89958	1	27.846	1	4.6537	-6.1352	1	27	16/20	26.4626	2148	1665	MS2	833.002	106	Glioma_grade 2_7rep.raw
68799814			sNIWLAAVTk	Unambiguous	N-Term(iTRAQ4plex); K10(iTRAQ4plex)	HCD	0.37498516	0	1	2	0.3377	0.3574	0.3916	0.41444018	0.091448153	159149.03	150388.23	53744.953	62326.93	4.002633537	10.45623449	2	High	5232	Unique	Used	52701	1	50.12	1	4.4145	-2.5883	1	25	16/18	41.53785	5232	1390.83	MS2	695.917	188	Glioma_grade 2_2.raw
68799814			sNIWLAAVTk	Unambiguous	N-Term(iTRAQ4plex); K10(iTRAQ4plex)	HCD	0.3556081	0	1	2	0.3712	0.3691	0.3421	0.34010226	0.04730033	157332.72	158237.14	58402.39	53816.81	0.405312165	5.778859079	2	High	5090	Unique	Used	60579	1	50.084	1	4.3273	1.9756	1	26	16/18	32.4267	5090	1390.83	MS2	695.92	166	Glioma_grade 2_3.raw
68799814			sYLSGmPEck	Unambiguous	N-Term(iTRAQ4plex); M6(Oxidation); C9(Methylthio); K10(iTRAQ4plex)	HCD	0.49638164	0	1	2	0.49156	0.5024	0.4905	0.50131124	0.012622037	146137	142991.34	71834.836	71683.164	1.538636799	0.149456235	2	High	2448	Unique	Used	50083	1	29.889	1	3.2529	3.7399	1	43	18-Dec	54.27323	2448	1464.68	MS2	732.845	63	Glioma_grade 2_2.raw
68799814			iPTPLNTSGVQVIcMk	Unambiguous	N-Term(iTRAQ4plex); C14(Methylthio); K16(iTRAQ4plex)	HCD	0.4379869	0	1	2	0.40807	0.4586	0.4196	0.4716146	0.069257132	96579.87	85935.41	39410.906	40528.39	8.247824344	1.976951396	2	High	6721	Unique	Used	62098	1	62.76	1	5.9649	1.191	1	72	19/30	21.7006	6721	2035.1	MS2	1018.05	189	Glioma_grade 2_3.raw
68799814			wARPPISMNFEVPFAPSGLk	Unambiguous	N-Term(iTRAQ4plex); K20(iTRAQ4plex)	HCD	0.39281076	0	1	2	0.38273	0.3954	0.3903	0.40322956	0.021958536	60660.906	58714.64	23216.521	23675.479	2.305694814	1.384169215	2	High	5891	Unique	Used	92866	1	65.089	1	4.7238	-0.0703	1	43	15/38	34.01389	5891	2532.37	MS2	1266.69	134	Glioma_grade 2_7rep.raw
68799814			iPTPLNTSGVQVIcMk	Unambiguous	N-Term(iTRAQ4plex); C14(Methylthio); K16(iTRAQ4plex)	HCD	0.4085779	0	1	2	0.39537	0.4146	0.4028	0.42242622	0.029469883	59190.32	56443.9	23402.203	23843.383	3.358888409	1.320594774	2	High	6812	Unique	Used	69825	1	63.248	1	5.4799	0.0514	1	67	20/30	18.42984	6812	2035.1	MS2	1018.05	190	Glioma_grade 2_4.raw
68799814			sNFkPSLLAQk	Unambiguous	N-Term(iTRAQ4plex); K4(iTRAQ4plex); K11(iTRAQ4plex)	HCD	0.46164933	0	1	2	0.45104	0.5355	0.3994	0.47424	0.121305492	34545.125	29096.904	15581.383	13798.917	12.10669765	8.579856542	3	High	2130	Unique	Used	89944	1	27.669	1	3.2713	-8.7827	1	56	14/40	66.55754	2130	1664.99	MS2	555.669	97	Glioma_grade 2_7rep.raw

68799814			qSIAIDDcTFHQcVR	Unambiguous	N-Term(iTRAQ4plex); C8(Methylthio); C13(Methylthio)	HCD	0.64659417	0	1	2	0.57705	0.712	0.5936	0.7323958	0.121989377	30200.293	24476.316	17427.225	17926.352	14.80509866	1.996607508	3	High	5597	Unique	Used	61048	1	54.049	1	5.8117	1.3853	1	34	26/56	30.24751	5597	1971.87	MS2	657.961	28	Glioma_grade2_3.raw
68799814			mIGGLFIYNHk	Unambiguous	N-Term(iTRAQ4plex); M1(Oxidation); K11(iTRAQ4plex)	HCD	0.6209959	0	1	2	0.48731	0.6329	0.6118	0.7946255	0.199809567	29978.684	23081.5	14609.005	18341.148	18.38307073	16.01827842	3	High	3419	Unique	Used	86350	1	46.629	1	5.3604	2.7484	1	100	20/40	34.75181	3419	1596.89	MS2	532.967	213	Glioma_grade2_1rep.raw
68799814			qSIAIDDcTFHQcVR	Unambiguous	N-Term(iTRAQ4plex); C8(Methylthio); C13(Methylthio)	HCD	0.59656537	0	1	2	0.59256	0.5575	0.6381	0.60032964	0.055377071	21773.697	23143.428	12902.315	13893.685	4.312591594	5.232157409	3	High	5713	Unique	Used	68821	1	54.354	1	7.0942	-0.2862	1	51	30/56	9.770206	5713	1971.87	MS2	657.96	29	Glioma_grade2_4.raw
68799814			qSIAIDDcTFHQcVR	Unambiguous	N-Term(iTRAQ4plex); C8(Methylthio); C13(Methylthio)	HCD	0.6270142	0	1	2	0.53483	0.7249	0.5548	0.75196046	0.175532387	20273.36	14957.642	10842.783	11247.556	21.33791281	2.591338532	2	High	5622	Unique	Used	61071	1	54.245	1	4.9617	1.1754	1	100	21/28	23.93255	5622	1971.87	MS2	986.438	40	Glioma_grade2_3.raw
68799814			wARPPISmNFEVPFAPSGLk	Unambiguous	N-Term(iTRAQ4plex); M8(Oxidation); K20(iTRAQ4plex)	HCD	0.5978826	0	1	2	0.61379	0.5999	0.5958	0.582333	0.021662353	19616.195	20069.783	12040.259	11687.297	1.616365108	2.10372972	3	High	5376	Unique	Used	92501	1	59.455	1	5.5829	1.8244	1	38	23/76	26.41663	5376	2548.37	MS2	850.127	137	Glioma_grade2_7rep.raw
68799814			iPTPLNTSGVQVIcMk	Unambiguous	N-Term(iTRAQ4plex); C14(Methylthio); K16(iTRAQ4plex)	HCD	0.57713455	0	1	2	0.59189	0.6719	0.4936	0.5603837	0.127708849	19511.906	17188.234	11548.913	9632.006	8.954103327	12.79885862	3	High	6830	Unique	Used	69841	1	63.405	1	6.3793	0.1047	1	27	28/60	16.0596	6830	2035.1	MS2	679.038	187	Glioma_grade2_4.raw
68799814			iPTPLNTSGVQVIcmk	Unambiguous	N-Term(iTRAQ4plex); C14(Methylthio); M15(Oxidation); K16(iTRAQ4plex)	HCD	0.44564083	0	1	2	0.44231	0.4889	0.4065	0.4493263	0.075570593	18383.197	16631.936	8131.0063	7473.166	7.073104813	5.962037949	2	High	6329	Unique	Used	61733	1	59.692	1	4.0501	1.4589	1	100	16/30	32.45427	6329	2051.1	MS2	1026.05	175	Glioma_grade2_3.raw
68799814			wARPPISMNFEVPFAPSGLk	Unambiguous	N-Term(iTRAQ4plex); K20(iTRAQ4plex)	HCD	0.32917812	0	1	2	0.25994	0.3123	0.3432	0.41237742	0.19183842	15691.51	13059.3545	4078.7727	5385.3833	12.94719331	19.5244714	4	High	5878	Unique	Used	92856	1	64.963	1	4.2439	1.317	1	13	21/114	30.0523	5878	2532.37	MS2	633.848	138	Glioma_grade2_7rep.raw
71361682	NP_006176.2	NUMA1	nuclear mitotic apparatus protein 1 [Homo sapiens]	2	2	2.335913897	34.52664308	13.922998	1.84	1.04E-03	2115	238.12	5.7827	Structural protein	Cell growth and/or maintenance	Structural molecule activity	Nucleus	Cytosol, Microtubule, Mitochondrion, Nucleolus, Cytoplasm																								
71361682			vEFATLQEALAHALTEk	Unambiguous	N-Term(iTRAQ4plex); K17(iTRAQ4plex)	HCD	2.9062045	0	1	1	2.7634	2.5649	3.2739	3.038754	10.67468189	6131.754	6606.172	16944.488	20074.53	5.267171201	11.95749668	3	High	7429	Unique	Used	77540	1	71.714	1	7.4456	-0.9679	1	58	36/64	30.41321	7429	2159.19	MS2	720.403	128	Glioma_grade2_5.raw
71361682			eFASHLQQLQDALNELTEEHSk	Unambiguous	N-Term(iTRAQ4plex); K22(iTRAQ4plex)	HCD	1.7656233	0	1	1	1.62867	1.4397	2.1343	1.8866861	17.094563	1241.091	1403.9827	2021.3293	2648.8745	8.709158136	19.00308789	4	High	6001	Unique	Used	92941	1	66.341	1	6.4774	1.9131	1	67	34/126	53.65267	6001	2855.45	MS2	714.617	96	Glioma_grade2_7rep.raw
72534712	NP_001026881.1	ERGIC1	endoplasmic reticulum-Golgi intermediate compartment protein 1 [Homo sapiens]	4	5	2.477740526	11.45630923	32.769513	19.31	1.19E-04	290	32.571	7.064	Transport/cargo protein	Transport	Transporter activity	Golgi apparatus	Cytoplasm, Endoplasmic reticulum																								
72534712			fEGQFSINk	Unambiguous	N-Term(iTRAQ4plex); K9(iTRAQ4plex)	HCD	2.4777405	0	1	1	2.33938	2.4489	2.5053	2.6225812	4.756287953	141935.38	135588.11	332040.4	355590.8	3.234463259	4.843482244	2	High	3335	Unique	Used	58953	1	36.358	1	3.9962	2.115	1	47	16/16	63.35286	3335	1357.74	MS2	679.373	197	Glioma_grade2_3.raw
72534712			iVPTVYEDk	Unambiguous	N-Term(iTRAQ4plex); K9(iTRAQ4plex)	HCD	2.1622136	0	1	1	2.17921	2.038	2.295	2.1463206	4.887486543	51182.54	54728.39	111537.39	117464.67	4.734722998	3.660421118	2	High	2909	Unique	Used	58553	1	33.211	1	3.3527	0.8116	1	100	16-Oct	45.08307	2909	1351.77	MS2	676.39	321	Glioma_grade2_3.raw
72534712			ITSNPLASHDYILk	Unambiguous	N-Term(iTRAQ4plex); K14(iTRAQ4plex)	HCD	2.2129202	0	1	1	2.02913	2.2369	2.1912	2.415535	7.16465875	28197.04	25577.793	57215.36	61784.055	6.888305224	5.429531256	3	High	4092	Unique	Used	74613	1	43.378	1	5.8711	1.3195	1	21	27/52	17.82486	4092	1860.05	MS2	620.688	146	Glioma_grade2_5.raw
72534712			ITSNPLASHDYILk	Unambiguous	N-Term(iTRAQ4plex); K14(iTRAQ4plex)	HCD	2.837826	0	1	1	2.70799	2.8954	2.784	2.9766498	4.17945565	23715.693	22180.902	64221.777	66024.78	4.729157463	1.957695738	2	High	4116	Unique	Used	74635	1	43.562	1	6.4547	1.7533	1	82	23/26	0	4116	1860.05	MS2	930.529	178	Glioma_grade2_5.raw
72534712			ISFGDTLQVQNIHGAFNALGGADR	Unambiguous	N-Term(iTRAQ4plex)	HCD	2.6195648	0	1	1	2.55568	2.3668	2.8925	2.67873	8.404965906	9010.162	9729.033	23027.082	26061.453	5.425190985	8.741855143	3	High	6819	Unique	Used	69831	1	63.313	1	7.9362	1.6289	1	38	36/92	18.61065	6819	2645.36	MS2	882.458	149	Glioma_grade2_4.raw
73486658	NP_002071.2	GOT2	aspartate aminotransferase, mitochondrial precursor [Homo sapiens]	8	11	0.397020102	28.09896148	51.980201	20	6.56E-05	430	47.487	9.0122	Enzyme: Aminotransferase	Metabolism,Energy pathways	Transaminase activity	Mitochondrion	Nucleolus																								
73486658			eFSIYMTk	Unambiguous	N-Term(iTRAQ4plex); K8(iTRAQ4plex)	HCD	0.36105463	0	1	1	0.34106	0.3676	0.355	0.38260996	0.049046194	456496.72	423527	155691	162045.66	5.298291866	2.828394544	2	High	4127	Unique	Used	51662	1	42.037	1	3.8173	-2.4864	1	17	13/14	45.35259	4127	1306.69	MS2	653.85	221	Glioma_grade2_2.raw
73486658			vGAFTMVck	Unambiguous	N-Term(iTRAQ4plex); C8(Methylthio); K9(iTRAQ4plex)	HCD	0.35215345	0	1	1	0.3409	0.3744	0.3319	0.36451775	0.056228231	281142.12	255991.77	95840.016	93313.55	6.621806356	1.888921556	2	High	5275	Unique	Used	60754	1	51.462	1	3.7712	-5.2518	1	30	15/16	51.70634	5275	1289.66	MS2	645.333	148	Glioma_grade2_3.raw
73486658			kQWLQEVk	Unambiguous	N-Term(iTRAQ4plex); K1(iTRAQ4plex); K8(iTRAQ4plex)	HCD	0.4559833	1	1	1	0.42175	0.4682	0.445	0.4939876	0.067783714	262340.62	236298.75	110642.586	116728.65	7.385851972	3.785436716	2	High	1979	Unique	Used	89811	1	26.425	1	3.2564	7.9587	1	80	14/14	73.72025	1979	1490.92	MS2	745.962	159	Glioma_grade2_7rep.raw
73486658			nLDkEYLPIGGLAEFck	Unambiguous	N-Term(iTRAQ4plex); K4(iTRAQ4plex); C16(Methylthio); K17(iTRAQ4plex)	HCD	0.25313064	1	1	1	0.23138	0.2731	0.2362	0.27879938	0.096211245	172072.1	145793.62	39814.387	40647.17	11.69153529	1.463721381	3	High	6592	Unique	Used	83384	1	66.89	1	6.9513	0.6676	1	10	26/64	12.66323	6592	2388.27	MS2	796.761	151	Glioma_grade2_6.raw
73486658			iAAAILNTPDLR	Unambiguous	N-Term(iTRAQ4plex)	HCD	0.6270902	0	1	1	0.58275	0.6311	0.6234	0.67511034	0.060260828	89573.49	82714.45	52198.703	55841.383	5.630195238	4.76816305	2	High	4141	Unique	Used	86791	1	55.816	1	4.042	0.6354	1	60	17/22	52.16523	4141	1411.84	MS2	706.424	206	Glioma_grade2_1rep.raw
73486658			eYLPIGGLAEFck	Unambiguous	N-Term(iTRAQ4plex); C12(Methylthio); K13(iTRAQ4plex)	HCD	0.41139957	0	1	1	0.38401	0.4106	0.4121	0.44068873	0.056218591	63463.223	59349.348	24370.45	26154.588	4.737216859	4.993864936	2	High	8448	Unique	Used	55677	1	74.9	1	3.6827	2.3345	1	47	17/24	43.84836	8448	1773.92	MS2	887.464	233	Glioma_grade2_2.raw
73486658			tQLVSNLk	Unambiguous	N-Term(iTRAQ4plex); K8(iTRAQ4plex)	HCD	0.41346833	0	1	1	0.38244	0.4449	0.3864	0.44956657	0.087390852	60035.137	51606.008	22959.742	23200.336	10.67759431	0.737111618	3	High	1585	Unique	Used	49266	1	23.902	1	3.5628	-6.1279	1	62	13/28	81.97922	1585	1190.73	MS2	397.581	220	Glioma_grade2_2.raw
73486658			tcGFDFTGAVEDISk	Unambiguous	N-Term(iTRAQ4plex); C2(Methylthio); K15(iTRAQ4plex)	HCD	0.3970201	0	1	1	0.40119	0.4076	0.3866	0.3927795	0.023255735	58866.844	57941.688	23617.027	22758.307	1.120096401	2.618662478	2	High	7236	Unique	Used	54586	1	64.827	1	4.8542	1.7255	1	54	25/28	0	7236	1923.91	MS2	962.459	75	Glioma_grade2_2.raw
73486658			tcGFDFTGAVEDISk	Unambiguous	N-Term(iTRAQ4plex); C2(Methylthio); K15(iTRAQ4plex)	HCD	0.31077427	0	1	1	0.30549	0.3255	0.2969	0.3164035	0.040147121	33880.19	31795.96	10350.105	10060.354	4.488001098	2.007646148	2	High	7345	Unique	Used	54684	1	65.723	1	5.9148	2.6138	1	100	26/28	33.22572	7345	1923.91	MS2	962.46	82	Glioma_grade2_2.raw
73486658			nLDkEYLPIGGLAEFck	Unambiguous	N-Term(iTRAQ4plex); K4(iTRAQ4plex); C16(Methylthio); K17(iTRAQ4plex)	HCD	0.36757267	1	1	1	0.37584	0.517	0.2589	0.35620376	0.282316289	11418.06	8299.901	4291.327	2956.456	22.36409103	26.04648445	4	High	6602	Unique	Used	83392	1	66.984	1	4.9371	4.8787	1	15	21/96	11.4982	6602	2388.28	MS2	597.825	155	Glioma_grade2_6.raw
73486658			tcGFDFTGAVEDISk	Unambiguous	N-Term(iTRAQ4plex); C2(Methylthio); K15(iTRAQ4plex)	HCD	0.62300986	0	1	1	0.62605	0.69	0.5622	0.6196562	0.083717222	11012.852	9991.852	6894.624	6191.513	6.874231825	7.598492298	3	High	7217	Unique	Used	54568	1	64.691	1	3.933	-0.5022	1	29	22/56	41.14702	7217	1923.91	MS2	641.974	83	Glioma_grade2_2.raw
74136552	NP_055969.1	MESDC2	LDLR chaperone MESD precursor [Homo sapiens]	2	3	2.611838818	9.258679269	17.426901	9.83	5.20E-04	234	26.06	7.7817	Chaperone	Cell growth and/or maintenance	Chaperone activity	Endoplasmic reticulum	-																								
74136552			rPSAPVDFSk	Unambiguous	N-Term(iTRAQ4plex); K10(iTRAQ4plex)	HCD	2.6438298	0	1	1	2.36167	2.7588	2.5454	2.9734387	9.95038998	39366.03	33699.18	92969.54	100202.45	10.96847067	5.295215218	3	High	1196	Unique	Used	78695	1	20.757	1	4.6439	-2.0974	1	41	16/36	12.43863	1196	1391.79	MS2	464.6	211	Glioma_grade2_6.raw
74136552			dDDIEEGDLPEHk	Unambiguous	N-Term(iTRAQ4plex); K13(iTRAQ4plex)	HCD	2.2287169	0	1	1	2.12109	2.0421	2.4226	2.33233	7.970685255	9549.952	9919.528	20256.346	23135.615	2.684506168	9.383999192	2	High	1648	Unique	Used	65064	1	22.877	1	4.593	1.2097	1	100	20/24	26.71036	1648	1799.86	MS2	900.432	76	Glioma_grade2_4.raw
74136552			dDDIEEGDLPEHk	Unambiguous	N-Term(iTRAQ4plex); K13(iTRAQ4plex)	HCD	2.6118388	0	1	1	2.30957	2.9656	2.3363	2.9999623	14.37538519	6249.995	4867.4424	14434.811	14602.144	17.58700829	0.814977321	3	High	1630	Unique	Used	65049	1	22.71	1	3.9659	0.6258	1	78	28/48	34.84074	1630	1799.86	MS2	600.623	59	Glioma_grade2_4.raw
76496472	NP_001029025.1	RPL3	60S ribosomal protein L3 isoform b [Homo sapiens]	6	14	2.227170944	19.4645756	94.060803	26.27	6.25E-05	354	40.127	10.228	Ribosomal subunit	Protein metabolism	Structural constituent of ribosome	Cytoplasm	Ribosome, Nucleolus																								
76496472			iGQGYLIk	Unambiguous	N-Term(iTRAQ4plex); K8(iTRAQ4plex)	HCD	2.2266111	0	1	2	2.1572	2.3114	2.1475	2.3009865	3.993805785	441278.88	411835.22	951926.06	947627.25	4.880897326	0.320045527	2	High	2709	Unique	Used	58368	1	31.665	1	3.0434	0.369	1	9	14/14	14.85478	2709	1179.73	MS2	590.371	195	Glioma_grade2_3.raw
76496472			iGQGYLIk	Unambiguous	N-Term(iTRAQ4plex); K8(iTRAQ4plex)	HCD	2.560545	0	1	2	2.49854	2.3937	2.7347	2.6199431	5.770692672	281608.28	293947.34	703610.75	770125.4	3.031864409	6.38281962	2	High	3067	Unique	Used	66377	1	33.649	1	3.1789	-1.4935	1	12	14/14	12.91799	3067	1179.73	MS2	590.37	208	Glioma_grade2_4.raw
76496472			iGQGYLIk	Unambiguous	N-Term(iTRAQ4plex); K8(iTRAQ4plex)	HCD	2.1656413	0	1	2	2.04738	2.1359	2.1941	2.2890203	4.684241844	173282.4	166099.17	354774.34	380204.34	2.993274292	4.89312845	2	High	3168	Unique	Used	66472	1	34.401	1	3.0165	0.2655	1	22	14/14	15.08241	3168	1179.73	MS2	590.371	208	Glioma_grade2_4.raw
76496472			tVFAEHISDEck	Unambiguous	N-Term(iTRAQ4plex); C11(Methylthio); K12(iTRAQ4plex)	HCD	2.2844315	0	1	2	2.49286	1.9093	2.7743	2.1247954	16.52518619	60646.54	79184.65	151183.58	168251.19	18.74892618	7.556236142	3	High	3332	Unique	Used	73946	1	36.969	1	4.3306	2.2909	1	9	23/44	10.94174	3332	1712.83	MS2	571.614	88	Glioma_grade2_5.raw
76496472			dDPSkPVHLTAFLGYk	Unambiguous	N-Term(iTRAQ4plex); K5(iTRAQ4plex); K16(iTRAQ4plex)	HCD	2.2277305	0	1	2	2.43528	2.4597	1.9981	2.0180984	11.4022804	41867.05	41451.5	101958.08	83653.21	0.705336862	13.94688621	3	High	4028	Unique	Used	91479	1	45.681	1	5.1706	0.1674	1	15	25/60	28.81283	4028	2220.24	MS2	740.751	148	Glioma_grade2_7rep.raw
76496472			tVFAEHISDEck	Unambiguous	N-Term(iTRAQ4plex); C11(Methylthio); K12(iTRAQ4plex)	HCD	2.0661728	0	1	2	2.10182	1.9123	2.2353	2.0337434	6.510338624	38028.023	41797.043	79927.92	85004.46	6.677350196	4.352881901	2	High	3326	Unique	Used	73941	1	36.915	1	5.6462	4.8644	1	100	19/22	12.67703	3326	1712.83	MS2	856.919	112	Glioma_grade2_5.raw
76496472			vAcIGAWHPAR	Unambiguous	N-Term(iTRAQ4plex); C3(Methylthio)	HCD	1.7226552	0	1	3	1.64642	1.6512	1.7939	1.799108	4.954039721	33944.184	33846.707	55886.45	60893.883	0.203350765	6.064017356	3	High	2998	Unique	Used	73636	1	34.447	1	4.4472	-0.3838	1	21	25/40	0	2998	1370.69	MS2	457.569	135	Glioma_grade2_5.raw
76496472			sINPLGGFVHYGEVTNDFVMLk	Unambiguous	N-Term(iTRAQ4plex); K22(iTRAQ4plex)	HCD	2.8694677	0	1	2	2.72966	3.3079	2.5077	3.0388935	12.10204782	31223.873	25765.955	85230.48	78299.99	13.54392868	5.993496473	3	High	7153	Unique	Used	77310	1	69.147	1	7.8016	1.2414	1	8	35/84	4.481116	7153	2725.43	MS2	909.148	119	Glioma_grade2_5.raw
76496472			vAcIGAWHPAR	Unambiguous	N-Term(iTRAQ4plex); C3(Methylthio)	HCD	1.9967065	0	1	3	2.04908	1.8903	2.112	1.9483925	4.971214921	27574.594	29890.518	56502.496	58238.457	5.699477502	2.139619313	3	High	2896	Unique	Used	80243	1	33.989	1	4.6277	1.2192	1	30	23/40	22.89711	2896	1370.7	MS2	457.57	170	Glioma_grade2_6.raw
76496472			dDPSkPVHLTAFLGYk	Unambiguous	N-Term(iTRAQ4plex); K5(iTRAQ4plex); K16(iTRAQ4plex)	HCD	3.0658312	0	1	2	2.83227	3.1376	3.0011	3.3245754	6.787365646	23220.729	20961.146	65767.25	69686.914	7.232678387	4.092337825	4	High	4036	Unique	Used	91486	1	45.745	1	6.1104	-3.4124	1	5	23/90	23.74357	4036	2220.23	MS2	555.813	174	Glioma_grade2_7rep.raw
76496472			vAcIGAWHPAR	Unambiguous	N-Term(iTRAQ4plex); C3(Methylthio)	HCD	1.7812855	0	1	3	1.90515	1.8131	1.7479	1.663406	5.736560737	16220.58	17044.031	30902.627	28351.145	3.500824261	6.089638392	3	High	2778	Unique	Used	80144	1	32.902	1	4.8327	-0.2502	1	50	24/40	28.42854	2778	1370.69	MS2	457.57	170	Glioma_grade2_6.raw
76496472			sINPLGGFVHYGEVTNDFVMLk	Unambiguous	N-Term(iTRAQ4plex); K22(iTRAQ4plex)	HCD	2.3859565	0	1	2	2.20473	2.4307	2.3454	2.585757	6.66755665	9551.175	8663.168	21057.781	22400.848	6.894739727	4.370555653	3	High	6773	Unique	Used	83525	1	68.735	1	7.503	0.7711	1	57	34/84	17.79429	6773	2725.43	MS2	909.148	140	Glioma_grade2_6.raw
76496472			sINPLGGFVHYGEVTNDFVMLk	Unambiguous	N-Term(iTRAQ4plex); K22(iTRAQ4plex)	HCD	2.1016114	0	1	2	2.5356	2.1045	2.0981	1.7414091	15.32834689	1705.3842	2054.7202	4324.1694	3578.1082	13.13888277	13.35171859	4	High	6790	Unique	Used	83538	1	68.912	1	4.6275	1.0349	1	47	24/126	13.56641	6790	2725.43	MS2	682.113	166	Glioma_grade2_6.raw
76496472			IEQQVPVNQVFGQDEMIDVIGVTk	Unambiguous	N-Term(iTRAQ4plex); K24(iTRAQ4plex)	HCD	3.250064	0	1	2	3.18224	3.689	2.8714	3.3286877	10.38556532	1668.3473	1439.1677	5309.0825	4790.54	10.4298418	7.260962835	2	High	7668	Unique	Used	54970	1	68.466	1	4.1539	1.0583	1	77	22/46	0	7668	2974.58	MS2	1487.8	74	Glioma_grade2_2.raw
83776596	NP_076951.2	CAMKV	caM kinase-like vesicle-associated protein [Homo sapiens]	5	8	0.498045236	22.28814618	50.052919	13.97	2.49E-04	501	54.32	5.5542	Unclassified	Biological_process unknown	Molecular function unknown	Integral to membrane	Cytoskeleton, Synaptic vesicle																								
83776596			qVLEAVAYLHSLk	Unambiguous	N-Term(iTRAQ4plex); K13(iTRAQ4plex)	HCD	0.49903676	0	1	1	0.47026	0.5411	0.4625	0.53214866	0.081464735	142989.56	124267.56	67242.31	66128.82	9.906904001	1.180699796	3	High	5574	Unique	Used	82556	1	57.212	1	6.3503	1.2209	1	14	28/48	24.53637	5574	1759.04	MS2	587.017	118	Glioma_grade2_6.raw
83776596			iVISDFHLAk	Unambiguous	N-Term(iTRAQ4plex); K10(iTRAQ4plex)	HCD	0.37790853	0	1	1	0.3483	0.3867	0.37	0.41077802	0.069708445	41047.22	36969.062	14296.939	15186.078	7.392541922	4.264938126	3	High	4168	Unique	Used	81358	1	44.755	1	4.5055	7.7412	1	100	20/36	49.17221	4168	1430.87	MS2	477.629	145	Glioma_grade2_6.raw
83776596			hPNILQLVDVFVTR	Unambiguous	N-Term(iTRAQ4plex)	HCD	0.5280779	0	1	1	0.54122	0.5489	0.5075	0.5147459	0.038007712	28990.885	28583.416	15690.522	14713.195	1.000877433	4.54598725	2	High	7709	Unique	Used	77759	1	74.556	1	4.1541	-1.0305	1	100	18/26	49.31608	7709	1795.03	MS2	898.02	170	Glioma_grade2_5.raw
83776596			iTAEEAISHEWISGNAASDk	Unambiguous	N-Term(iTRAQ4plex); K20(iTRAQ4plex)	HCD	0.3586424	0	1	1	0.34544	0.3708	0.3473	0.37281016	0.041020163	22974.29	21402.334	7936.331	7979.008	5.009577778	0.379221531	2	High	5205	Unique	Used	68355	1	50.365	1	6.6014	-0.0926	1	96	26/38	7.864203	5205	2417.22	MS2	1209.11	163	Glioma_grade2_4.raw

83776596			iTAEEAISHEWISGNAASDk	Unambiguous	N-Term(iTRAQ4plex); K20(iTRAQ4plex)	HCD	0.5965012	0	1	1	0.5204	0.5365	0.6547	0.67495704	0.133143462	20256.162	19647.965	10541.328	13261.531	2.155467393	16.16170551	3	High	4785	Unique	Used	75227	1	49.158	1	5.7573	0.3311	1	36	29/76	36.7763	4785	2417.22	MS2	806.411	136	Glioma_grade 2_5.raw
83776596			iTAEEAISHEWISGNAASDk	Unambiguous	N-Term(iTRAQ4plex); K20(iTRAQ4plex)	HCD	0.4970537	0	1	1	0.46123	0.5072	0.4878	0.53644496	0.063671058	16458.654	14966.577	7591.283	8028.7446	6.71471764	3.960710849	2	High	4767	Unique	Used	75212	1	48.989	1	6.0391	1.8264	1	97	25/38	10.80262	4767	2417.22	MS2	1209.12	164	Glioma_grade 2_5.raw
83776596			kEYFIFLELATGR	Unambiguous	N-Term(iTRAQ4plex); K1(iTRAQ4plex)	HCD	0.6564783	1	1	1	0.71235	0.8263	0.5101	0.59167075	0.209838103	5422.56	4674.5654	3862.7405	2765.8035	10.47648777	23.40337761	3	High	6014	Unique	Used	92951	1	66.469	1	5.0709	-0.5067	1	100	22/48	30.77426	6014	1875.06	MS2	625.692	197	Glioma_grade 2_7rep.raw
83776596			hPNILQLVDVFVTR	Unambiguous	N-Term(iTRAQ4plex)	HCD	0.38038218	0	1	1	0.37021	0.4067	0.3565	0.39156088	0.058447668	4033.3667	3671.7166	1493.1759	1437.7006	6.637831888	2.676807489	3	High	7692	Unique	Used	77746	1	74.379	1	5.7156	1.5122	1	46	23/52	13.54556	7692	1795.04	MS2	599.017	107	Glioma_grade 2_5.raw
86792863	NP_001034439.1	DPP6	dipeptidyl aminopeptidase-like protein 6 isoform 3 [Homo sapiens]	8	12	0.387932152	29.99955851	83.643572	13.73	6.25E-05	801	91.188	6.5073	Membrane transport protein	Transport	Auxiliary transport protein activity	Plasma membrane	-																								
86792863			vTVEDLFSEDFk	Unambiguous	N-Term(iTRAQ4plex); K12(iTRAQ4plex)	HCD	0.40971196	0	1	3	0.37416	0.4005	0.4183	0.44777134	0.075399059	251684.98	235114.05	94170.055	105277.33	4.814067511	7.875790874	2	High	7262	Unique	Used	54610	1	65.029	1	4.8264	2.5496	1	27	19/22	0	7262	1716.9	MS2	858.953	193	Glioma_grade 2_2.raw
86792863			mFDLETNEHVk	Unambiguous	N-Term(iTRAQ4plex); K11(iTRAQ4plex)	HCD	0.38471332	0	1	3	0.35129	0.407	0.3655	0.4234349	0.087827721	211534.83	182601.14	74309.66	77319.695	10.3818022	2.807393278	3	High	2566	Unique	Used	73237	1	31.173	1	5.0255	14.889	1	10	24/40	11.93472	2566	1650.86	MS2	550.96	180	Glioma_grade 2_5.raw
86792863			mFDLETNEHVk	Unambiguous	N-Term(iTRAQ4plex); K11(iTRAQ4plex)	HCD	0.4158902	0	1	3	0.39676	0.4273	0.4053	0.43649954	0.044559378	159235.48	147843.56	63177.516	64533.645	5.246404237	1.501713718	2	High	2577	Unique	Used	73248	1	31.24	1	4.4458	15.156	1	72	19/20	0	2577	1650.87	MS2	825.936	165	Glioma_grade 2_5.raw
86792863			iHFQHTAELITQLIR	Unambiguous	N-Term(iTRAQ4plex)	HCD	0.39115098	0	1	3	0.37708	0.4108	0.3731	0.40648448	0.049795524	145654.5	133698.31	54922.836	54346.293	6.052778224	0.746189649	2	High	5141	Unique	Used	92323	1	57.053	1	5.2069	-1.5821	1	44	21/28	27.75469	5141	1964.12	MS2	982.562	183	Glioma_grade 2_7rep.raw
86792863			yLGLHGLDNR	Unambiguous	N-Term(iTRAQ4plex)	HCD	0.26509175	0	1	3	0.2457	0.2555	0.2743	0.28525448	0.067416066	132034.78	126964.36	32441.385	36217.152	2.768602525	7.777242471	3	High	2397	Unique	Used	73078	1	29.945	1	3.4723	11.395	1	17	20/36	18.99281	2397	1301.72	MS2	434.579	254	Glioma_grade 2_5.raw
86792863			iHFQHTAELITQLIR	Unambiguous	N-Term(iTRAQ4plex)	HCD	0.34489164	0	1	3	0.30762	0.3238	0.365	0.38411888	0.102825067	96846.86	92020.21	29791.973	35346.7	3.614136594	12.05976403	3	High	5129	Unique	Used	92313	1	56.945	1	5.5388	-1.1539	1	14	23/56	29.9047	5129	1964.12	MS2	655.377	158	Glioma_grade 2_7rep.raw
86792863			vSALEEQQFLIIHPTADEk	Unambiguous	N-Term(iTRAQ4plex); K19(iTRAQ4plex)	HCD	0.2383466	0	1	3	0.20501	0.2137	0.262	0.27309474	0.143000626	53954.14	51766.094	11061.011	14137.049	2.9269367	17.2639269	3	High	5522	Unique	Used	68650	1	52.784	1	8.3392	2.0923	1	23	31/72	29.4808	5522	2456.33	MS2	819.449	106	Glioma_grade 2_4.raw
86792863			vSALEEQQFLIIHPTADEk	Unambiguous	N-Term(iTRAQ4plex); K19(iTRAQ4plex)	HCD	0.35288405	0	1	3	0.33434	0.3531	0.3527	0.3724737	0.044099552	40164.938	38030.312	13428.565	14165.291	3.860614347	3.775796688	3	High	5128	Unique	Used	75538	1	51.851	1	7.8504	2.6887	1	18	32/72	21.89999	5128	2456.33	MS2	819.45	108	Glioma_grade 2_5.raw
86792863			kVTVEDLFSEDFk	Unambiguous	N-Term(iTRAQ4plex); K1(iTRAQ4plex); K13(iTRAQ4plex)	HCD	0.6117062	1	1	3	0.54093	0.5912	0.6304	0.6890579	0.102135445	32974.453	30169.426	17837.021	20788.48	6.28233059	10.8063151	3	High	5040	Unique	Used	92243	1	56.072	1	5.357	-2.6294	1	17	20/48	39.72226	5040	1989.09	MS2	663.7	161	Glioma_grade 2_7rep.raw
86792863			iPHGDPQSLDPPEVSNAk	Unambiguous	N-Term(iTRAQ4plex); K18(iTRAQ4plex)	HCD	0.65552944	0	1	3	0.56971	0.658	0.6534	0.7546458	0.114752184	14785.6875	12801.525	8423.612	9660.617	10.17148622	9.673562792	3	High	2436	Unique	Used	73115	1	30.224	1	4.4475	9.531	1	42	20/68	24.17136	2436	2189.17	MS2	730.393	172	Glioma_grade 2_5.raw
86792863			vPImELPTYTGSIYPTVkPYHYPk	Unambiguous	N-Term(iTRAQ4plex); M4(Oxidation); K18(iTRAQ4plex); K24(iTRAQ4plex)	HCD	0.36176616	0	1	3	0.37299	0.3012	0.4368	0.35270673	0.153274625	2903.6067	3595.7764	1083.0022	1268.2545	15.06105675	11.14239526	4	High	4176	Unique	Used	96179	1	57.457	1	8.5044	0.5855	1	75	27/138	26.69695	4176	3242.76	MS2	811.444	87	Glioma_grade 2_8rep.raw
86792863			vPIMELPTYTGSIYPTVkPYHYPk	Unambiguous	N-Term(iTRAQ4plex); K18(iTRAQ4plex); K24(iTRAQ4plex)	HCD	0.4529099	0	1	3	0.56901	0.3987	0.5303	0.37155914	0.207370837	2105.4248	3004.7522	1198.0066	1116.4431	24.8883944	4.983828678	4	High	4544	Unique	Used	96388	1	62.416	1	8.6032	-0.6849	1	100	22/138	45.95311	4544	3226.76	MS2	807.445	87	Glioma_grade 2_8rep.raw
87578396	NP_002365.3	MAP2	microtubule-associated protein 2 isoform 1 [Homo sapiens]	25	56	0.413426012	24.63866511	312.25236	23.54	1.02E-04	1827	199.4	4.9067	Cytoskeletal associated protein	Cell growth and/or maintenance	Cytoskeletal protein binding	Cytoplasm, Nucleus	Cytoskeleton																								
87578396			vDHGAEIITQSPGR	Unambiguous	N-Term(iTRAQ4plex)	HCD	0.26617074	0	1	4	0.25371	0.2692	0.2633	0.27938908	0.040379004	1418202	1336558.6	359816.78	373419.88	4.191333489	2.623667031	2	High	1307	Unique	Used	64743	1	20.506	1	4.9089	2.2176	1	3	20/26	4.533657	1307	1623.86	MS2	812.434	148	Glioma_grade 2_4.raw
87578396			vDHGAEIITQSPGR	Unambiguous	N-Term(iTRAQ4plex)	HCD	0.26005188	0	1	4	0.23807	0.2584	0.2616	0.2839123	0.072062147	1137858.5	1048418	270886.5	297658.78	5.785543051	6.659403007	2	High	1379	Unique	Used	57122	1	22.007	1	5.2324	0.1128	1	4	20/26	4.287057	1379	1623.86	MS2	812.432	146	Glioma_grade 2_3.raw
87578396			vkDEFSVDk	Unambiguous	N-Term(iTRAQ4plex); K2(iTRAQ4plex); K9(iTRAQ4plex)	HCD	0.40400308	1	1	1	0.37495	0.4007	0.4071	0.4350537	0.061020901	564572.1	528235.44	211686.84	229810.78	4.702364827	5.805494886	2	High	1567	Unique	Used	89445	1	23.105	1	4.0951	11.527	1	27	16/16	41.06863	1567	1498.87	MS2	749.936	207	Glioma_grade 2_7rep.raw
87578396			lINQPLPDLk	Unambiguous	N-Term(iTRAQ4plex); K10(iTRAQ4plex)	HCD	0.5676864	0	1	4	0.53324	0.6076	0.5327	0.6069369	0.075222038	433570.56	380537.72	231195.78	230962.39	9.212504457	0.071417823	2	High	4742	Unique	Used	52240	1	46.568	1	4.0771	-0.6527	1	14	15/18	45.30679	4742	1438.89	MS2	719.947	125	Glioma_grade 2_2.raw
87578396			iDLSHVTSk	Unambiguous	N-Term(iTRAQ4plex); K9(iTRAQ4plex)	HCD	0.2990733	0	1	4	0.25978	0.2756	0.3212	0.34075624	0.126868367	403051.78	379978.66	104703.414	129480.09	4.167184002	14.96241649	3	High	1755	Unique	Used	72491	1	25.029	1	3.4171	-0.4464	1	6	16/32	58.28183	1755	1287.75	MS2	429.922	181	Glioma_grade 2_5.raw
87578396			yFETSALk	Unambiguous	N-Term(iTRAQ4plex); K8(iTRAQ4plex)	HCD	0.59298813	0	1	1	0.589	0.5931	0.5929	0.5970068	0.005516727	393357.5	390628.88	231686.53	233208.11	0.492208986	0.462865967	2	High	2924	Unique	Used	50531	1	33.275	1	3.1069	-7.0428	1	33	13/14	64.62485	2924	1246.68	MS2	623.845	242	Glioma_grade 2_2.raw
87578396			lEATQDMTPSSk	Unambiguous	N-Term(iTRAQ4plex); K12(iTRAQ4plex)	HCD	0.41439268	0	1	1	0.39051	0.4426	0.3895	0.44146606	0.072267738	341775.6	301523.38	133466.05	133112.34	8.848954718	0.187645172	2	High	1522	Unique	Used	49206	1	23.475	1	4.5659	2.3387	1	14	19/22	22.19375	1522	1595.82	MS2	798.415	205	Glioma_grade 2_2.raw
87578396			vkDEFSVDk	Unambiguous	N-Term(iTRAQ4plex); K2(iTRAQ4plex); K9(iTRAQ4plex)	HCD	0.40013024	1	1	1	0.38188	0.4177	0.3841	0.4200957	0.051841087	341431.12	312147.84	130384.74	131131.97	6.336313477	0.404082324	3	High	1573	Unique	Used	89450	1	23.161	1	4.1418	7.4664	1	8	18/32	56.26348	1573	1498.86	MS2	500.291	208	Glioma_grade 2_7rep.raw
87578396			vkDEFSVDk	Unambiguous	N-Term(iTRAQ4plex); K2(iTRAQ4plex); K9(iTRAQ4plex)	HCD	0.44752222	1	1	1	0.41214	0.4456	0.4493	0.48578185	0.067189815	336847.47	311536.2	138827.48	151338.62	5.520734554	6.097688141	2	High	1576	Unique	Used	89453	1	23.176	1	4.0528	-9.8109	1	48	16/16	58.26218	1576	1498.83	MS2	749.92	308	Glioma_grade 2_7rep.raw
87578396			tGLPPVTDENHVIVk	Unambiguous	N-Term(iTRAQ4plex); K15(iTRAQ4plex)	HCD	0.45491147	0	1	1	0.46895	0.4923	0.4193	0.44017217	0.07046937	318363.25	303235.28	149296.58	133475.73	3.441800344	7.912394477	3	High	2431	Unique	Used	73110	1	30.194	1	4.3456	11.992	1	4	22/56	22.02045	2431	1907.11	MS2	636.374	127	Glioma_grade 2_5.raw
87578396			aDVQMEFIQGPk	Unambiguous	N-Term(iTRAQ4plex); K12(iTRAQ4plex)	HCD	0.5826713	0	1	1	0.54211	0.6082	0.5599	0.6281855	0.068959065	290517.84	258928.11	157491.5	162654.88	8.13084974	2.280869777	2	High	4199	Unique	Used	51730	1	42.559	1	5.2866	2.128	1	23	16/22	50.12909	4199	1650.88	MS2	825.944	231	Glioma_grade 2_2.raw
87578396			aPQEADAFMGVESGHMk	Unambiguous	N-Term(iTRAQ4plex); K17(iTRAQ4plex)	HCD	0.255641	0	1	1	0.22999	0.2247	0.2873	0.28070423	0.128717802	287291.53	294075.06	66073.016	82548.12	1.650139566	15.67698656	3	High	3277	Unique	Used	73894	1	36.579	1	8.7259	-1.2268	1	3	38/64	9.928063	3277	2093	MS2	698.339	81	Glioma_grade 2_5.raw
87578396			vDHGAEIITQSPGR	Unambiguous	N-Term(iTRAQ4plex)	HCD	0.61707467	0	1	4	0.65589	0.5675	0.6744	0.5834932	0.084978286	283058.88	327152.97	185654.73	190891.53	10.21914932	1.966811085	3	High	1306	Unique	Used	64742	1	20.5	1	4.2835	0.7434	1	1	23/52	17.92855	1306	1623.86	MS2	541.958	155	Glioma_grade 2_4.raw
87578396			vDHGAEIITQSPGR	Unambiguous	N-Term(iTRAQ4plex)	HCD	0.29053685	0	1	4	0.316	0.3189	0.2625	0.26484573	0.106996436	267167.12	264760.75	84423.89	70120.76	0.639771157	13.08856724	3	High	1377	Unique	Used	57120	1	21.998	1	4.7233	-0.3841	1	1	28/52	7.940735	1377	1623.86	MS2	541.957	203	Glioma_grade 2_3.raw
87578396			vkDEFSVDk	Unambiguous	N-Term(iTRAQ4plex); K2(iTRAQ4plex); K9(iTRAQ4plex)	HCD	0.4516309	1	1	1	0.45646	0.4442	0.4593	0.4469338	0.016150416	247443.64	254286.94	112947.61	113649.42	1.928901298	0.438006279	3	High	1538	Unique	Used	89420	1	22.863	1	3.9178	-8.3539	1	11	16/32	59.75538	1538	1498.84	MS2	500.283	252	Glioma_grade 2_7rep.raw
87578396			vkDEFSVDk	Unambiguous	N-Term(iTRAQ4plex); K2(iTRAQ4plex); K9(iTRAQ4plex)	HCD	0.5001054	1	1	1	0.48011	0.6437	0.393	0.5269169	0.204523458	215705.89	160873.66	103562.414	84767.055	20.59179351	14.11390992	3	High	1367	Unique	Used	78855	1	22.001	1	3.9222	1.3583	1	11	13/32	57.8985	1367	1498.85	MS2	500.288	265	Glioma_grade 2_6.raw
87578396			yFETSALkEEATk	Unambiguous	N-Term(iTRAQ4plex); K8(iTRAQ4plex); K13(iTRAQ4plex)	HCD	0.26019865	1	1	1	0.21837	0.2827	0.2428	0.31435335	0.16057628	207640.38	160395.6	45341.566	50420.89	18.15425998	7.501111802	3	High	3018	Unique	Used	80352	1	34.987	1	3.569	-6.5932	1	19	23/48	43.60313	3018	1949.05	MS2	650.354	133	Glioma_grade 2_6.raw
87578396			vGSLDNAHHVPGGGNVk	Unambiguous	N-Term(iTRAQ4plex); K17(iTRAQ4plex)	HCD	0.47327766	0	1	4	0.39438	0.4318	0.5112	0.55964905	0.157995649	200814.45	183431.6	79197.76	102657.31	6.397739735	18.24354624	3	High	1083	Unique	Used	89009	1	19.376	1	5.1119	-1.3098	1	8	20/64	7.558478	1083	1946.04	MS2	649.352	192	Glioma_grade 2_7rep.raw
87578396			vDHGAEIITQSPGR	Unambiguous	N-Term(iTRAQ4plex)	HCD	0.39715824	0	1	4	0.32503	0.4657	0.3493	0.50050265	0.209740684	183855.83	128313.16	59759.406	64221.074	25.16239592	5.089310347	3	High	1364	Unique	Used	49055	1	22.432	1	4.5004	-0.7224	1	2	28/52	9.229859	1364	1623.86	MS2	541.957	210	Glioma_grade 2_2.raw
87578396			eEFVETcPSEHk	Unambiguous	N-Term(iTRAQ4plex); C7(Methylthio); K12(iTRAQ4plex)	HCD	0.34911194	0	1	1	0.336	0.3908	0.3133	0.36436248	0.096084109	165477.8	142265.34	55600.656	51836.152	10.66713485	4.95529671	3	High	2324	Unique	Used	65685	1	28.052	1	5.0145	12.35	1	9	23/44	18.38616	2324	1768.83	MS2	590.283	86	Glioma_grade 2_4.raw
87578396			tTAAGGESALAPSVFk	Unambiguous	N-Term(iTRAQ4plex); K16(iTRAQ4plex)	HCD	0.5744746	0	1	1	0.5661	0.5403	0.6103	0.58246434	0.051057186	161155.52	168852.28	91230.66	98350.43	3.298365184	5.311117947	2	High	3417	Unique	Used	50994	1	36.836	1	6.1714	5.236	1	54	22/30	20.17482	3417	1794.99	MS2	898	209	Glioma_grade 2_2.raw
87578396			sImTEQLETIPk	Unambiguous	N-Term(iTRAQ4plex); M3(Oxidation); K12(iTRAQ4plex)	HCD	0.3911775	0	1	1	0.37264	0.3767	0.4055	0.4099191	0.049177578	153686.8	152033.48	57269.47	62321.426	0.764799629	5.97415433	2	High	3154	Unique	Used	50744	1	34.998	1	4.921	2.6014	1	46	18/22	0	3154	1693.93	MS2	847.47	195	Glioma_grade 2_2.raw
87578396			amTEPSALIEk	Unambiguous	N-Term(iTRAQ4plex); M2(Oxidation); K11(iTRAQ4plex)	HCD	0.46076813	0	1	1	0.40908	0.4586	0.4627	0.5187122	0.096973398	143813.44	128287.664	58831.133	66544.38	8.069339912	8.70040589	2	High	1864	Unique	Used	49530	1	25.823	1	3.4239	-2.8513	1	38	15/20	14.25222	1864	1493.81	MS2	747.408	293	Glioma_grade 2_2.raw
87578396			ekDVFDDIPk	Unambiguous	N-Term(iTRAQ4plex); K2(iTRAQ4plex); K10(iTRAQ4plex)	HCD	0.4387	1	1	1	0.38516	0.4529	0.4266	0.5016533	0.110500818	143680.33	122195.06	55339.973	61299.562	11.42819583	7.225793201	2	High	2900	Unique	Used	80247	1	34.014	1	3.3052	9.1412	1	53	14/18	43.94962	2900	1637.93	MS2	819.467	235	Glioma_grade 2_6.raw
87578396			vGSLDNAHHVPGGGNVk	Unambiguous	N-Term(iTRAQ4plex); K17(iTRAQ4plex)	HCD	0.39873782	0	1	4	0.37577	0.3404	0.4631	0.41954967	0.133180497	125837.38	138902.25	47285.312	58276.395	6.979127509	14.72478902	4	High	1112	Unique	Used	89035	1	19.602	1	6.4638	-1.0031	1	3	29/96	15.37587	1112	1946.04	MS2	487.266	239	Glioma_grade 2_7rep.raw
87578396			fILkPAIk	Unambiguous	N-Term(iTRAQ4plex); K4(iTRAQ4plex); K8(iTRAQ4plex)	HCD	0.32505515	0	1	1	0.29616	0.3367	0.3148	0.3579041	0.08195874	120430.43	105934.1	35666.703	37914.25	9.056589604	4.319747598	2	High	2689	Unique	Used	90416	1	32.661	1	3.2981	-7.9803	1	55	13/14	50.44143	2689	1361.91	MS2	681.46	29	Glioma_grade 2_7rep.raw
87578396			aPLALFGHTLVASLEDMk	Unambiguous	N-Term(iTRAQ4plex); K18(iTRAQ4plex)	HCD	0.36595342	0	1	1	0.34839	0.3733	0.3591	0.38477132	0.043501991	118195.67	110316.05	41178.19	42446.453	4.876540017	2.144816015	3	High	7415	Unique	Used	83999	1	75.735	1	7.3284	-0.3277	1	14	24/68	24.35215	7415	2201.22	MS2	734.413	117	Glioma_grade 2_6.raw
87578396			kIDLSHVTSk	Unambiguous	N-Term(iTRAQ4plex); K1(iTRAQ4plex); K10(iTRAQ4plex)	HCD	0.34481978	1	1	4	0.33595	0.3507	0.3392	0.35407627	0.025453764	117136.35	112199.2	39352.36	39727.08	3.044527763	0.670128805	3	High	1624	Unique	Used	85134	1	25.412	1	4.5253	-8.436	1	23	21/36	58.34987	1624	1559.93	MS2	520.65	108	Glioma_grade 2_1rep.raw
87578396			lINQPLPDLk	Unambiguous	N-Term(iTRAQ4plex); K10(iTRAQ4plex)	HCD	0.29897758	0	1	4	0.31235	0.2632	0.3414	0.2877092	0.11126398	104139.06	123584.59	32527.848	35556.426	12.07609849	6.29081553	2	High	4774	Unique	Used	67959	1	46.961	1	4.1529	-0.1437	1	31	15/18	10.01292	4774	1438.89	MS2	719.947	127	Glioma_grade 2_4.raw
87578396			aMTEPSALIEk	Unambiguous	N-Term(iTRAQ4plex); K11(iTRAQ4plex)	HCD	0.41245934	0	1	1	0.39415	0.4068	0.4179	0.43135494	0.038425658	102415.16	99227.48	40367.13	42802.26	2.235668155	4.140698726	2	High	3132	Unique	Used	50723	1	34.844	1	3.5746	-0.4583	1	34	17/20	51.00444	3132	1477.82	MS2	739.412	301	Glioma_grade 2_2.raw
87578396			mAEAPPSEAMTLPk	Unambiguous	N-Term(iTRAQ4plex); K14(iTRAQ4plex)	HCD	0.4579855	0	1	1	0.44333	0.4367	0.4796	0.47241974	0.046203338	102154.08	103706.414	45288.164	48992.957	1.066417239	5.557176719	2	High	3569	Unique	Used	51137	1	37.953	1	4.5085	0.257	1	47	18/26	0	3569	1760.92	MS2	880.962	194	Glioma_grade 2_2.raw
87578396			eEFVETcPSEHk	Unambiguous	N-Term(iTRAQ4plex); C7(Methylthio); K12(iTRAQ4plex)	HCD	0.36208737	0	1	1	0.35275	0.3539	0.3702	0.37145743	0.027964609	96501.055	96178.414	34040.613	35726.184	0.236809495	3.416750476	2	High	2336	Unique	Used	65696	1	28.149	1	4.4363	12.185	1	55	18/22	0	2336	1768.83	MS2	884.921	95	Glioma_grade 2_4.raw
87578396			eEFVETcPSEHk	Unambiguous	N-Term(iTRAQ4plex); C7(Methylthio); K12(iTRAQ4plex)	HCD	0.33897945	0	1	1	0.31349	0.3138	0.3642	0.3644892	0.086371004	92902.63	92823.83	29124.174	33833.28	0.060002236	10.57806685	3	High	2009	Unique	Used	72715	1	27.117	1	4.5604	5.2075	1	10	22/44	15.83322	2009	1768.82	MS2	590.279	70	Glioma_grade 2_5.raw
87578396			lINQPLPDLk	Unambiguous	N-Term(iTRAQ4plex); K10(iTRAQ4plex)	HCD	0.41642857	0	1	4	0.40298	0.3997	0.4332	0.4297728	0.042058792	90661.33	91394.445	36534.375	39278.844	0.569485464	5.119509938	2	High	4592	Unique	Used	60118	1	46.166	1	3.9179	-1.0769	1	100	16/18	18.64817	4592	1438.89	MS2	719.947	124	Glioma_grade 2_3.raw
87578396			kIDLSHVTSk	Unambiguous	N-Term(iTRAQ4plex); K1(iTRAQ4plex); K10(iTRAQ4plex)	HCD	0.2722224	1	1	4	0.24075	0.2504	0.2932	0.3049588	0.115651739	60230.7	57902.8	14500.617	17657.969	2.786802856	13.88484562	2	High	1772	Unique	Used	94664	1	27.026	1	3.5255	-6.7058	1	100	17/18	50.48549	1772	1559.94	MS2	780.473	123	Glioma_grade 2_8rep.raw
87578396			aPQEADAFmGVESGHMk	Unambiguous	N-Term(iTRAQ4plex); M9(Oxidation); K17(iTRAQ4plex)	HCD	0.37899557	0	1	1	0.39005	0.3497	0.4117	0.36908844	0.07031954	55849.57	62296.363	21783.918	22992.867	7.716848023	3.818300202	3	High	2394	Unique	Used	73075	1	29.927	1	6.4714	13.493	1	14	34/64	15.5037	2394	2109.03	MS2	703.681	87	Glioma_grade 2_5.raw
87578396			dAHIPVVEEHVMGk	Unambiguous	N-Term(iTRAQ4plex); K14(iTRAQ4plex)	HCD	0.57953537	0	1	1	0.56543	0.6134	0.5483	0.5948366	0.050202382	51124.668	47128.35	28907.43	28033.668	5.752135894	2.170112826	3	High	2270	Unique	Used	90061	1	28.937	1	5.6833	1.1413	1	27	28/52	36.28851	2270	1848.99	MS2	617.002	205	Glioma_grade 2_7rep.raw
87578396			eEFVETcPSEHk	Unambiguous	N-Term(iTRAQ4plex); C7(Methylthio); K12(iTRAQ4plex)	HCD	0.50084156	0	1	1	0.51948	0.5101	0.4914	0.48253483	0.033769661	50855.066	51787.85	26418.398	24989.441	1.28518931	3.931015987	3	High	2272	Unique	Used	65636	1	27.676	1	4.7304	2.1019	1	31	22/44	33.71601	2272	1768.82	MS2	590.277	62	Glioma_grade 2_4.raw

87578396			tGLPPVTDENHVIVk	Unambiguous	N-Term(iTRAQ4plex); K15(iTRAQ4plex)	HCD	0.44856668	0	1	1	0.40493	0.4544	0.4433	0.49753323	0.084651716	49600.242	44196.938		20084.846	21989.443	8.146754304	6.401788295	3	High	2817	Unique	Used	66144	1	31.747	1	4.9833	1.2389	1	37	25/56	27.0289	2817	1907.09	MS2	636.367	147	Glioma_grade 2_4.raw
87578396			vGSLDNAHHVPGGGNVk	Unambiguous	N-Term(iTRAQ4plex); K17(iTRAQ4plex)	HCD	0.5461716	0	1	4	0.47977	0.5386	0.5529	0.62072027	0.105654755	47356.65	42184.016		22720.094	26184.473	8.169705993	10.01822952	2	High	1120	Unique	Used	89043	1	19.646	1	4.2074	0.2026	1	49	19/32	0	1120	1946.05	MS2	973.526	201	Glioma_grade 2_7rep.raw
87578396			aPQEADAFMGVESGHMk	Unambiguous	N-Term(iTRAQ4plex); K17(iTRAQ4plex)	HCD	0.2816345	0	1	1	0.27015	0.2812	0.282	0.2935935	0.033994815	44491.402	42736.008		12019.234	12547.013	2.846011365	3.038283462	2	High	3259	Unique	Used	73878	1	36.427	1	6.1405	4.4078	1	97	24/32	0	3259	2093.01	MS2	1047.01	59	Glioma_grade 2_5.raw
87578396			kIDLSHVTSk	Unambiguous	N-Term(iTRAQ4plex); K1(iTRAQ4plex); K10(iTRAQ4plex)	HCD	0.35437503	1	1	4	0.32447	0.3493	0.359	0.38657862	0.072355569	41604.383	38640.92		13499.178	14937.755	5.222697665	7.154270483	3	High	1766	Unique	Used	94659	1	26.965	1	4.0038	-5.5015	1	82	20/36	57.39006	1766	1559.94	MS2	520.651	97	Glioma_grade 2_8rep.raw
87578396			kIDLSHVTSk	Unambiguous	N-Term(iTRAQ4plex); K1(iTRAQ4plex); K10(iTRAQ4plex)	HCD	0.3611389	1	1	4	0.35725	0.3709	0.3518	0.36517772	0.023342829	40444.34	38960.562		14448.672	14227.529	2.642631523	1.090602726	3	High	1762	Unique	Used	94656	1	26.923	1	4.1259	10.345	1	86	21/36	63.12024	1762	1559.96	MS2	520.66	75	Glioma_grade 2_8rep.raw
87578396			mAEAPPSEAmTLPk	Unambiguous	N-Term(iTRAQ4plex); M10(Oxidation); K14(iTRAQ4plex)	HCD	0.64689505	0	1	1	0.57803	0.6491	0.645	0.7242157	0.092052771	36512.51	32517.502		21105.49	23549.686	8.184548042	7.740681914	2	High	2068	Unique	Used	49724	1	27.224	1	3.1017	0.6432	1	77	15/26	10.94428	2068	1776.91	MS2	888.96	221	Glioma_grade 2_2.raw
87578396			eEFVETcPSEHk	Unambiguous	N-Term(iTRAQ4plex); C7(Methylthio); K12(iTRAQ4plex)	HCD	0.40257552	0	1	1	0.38767	0.4067	0.3986	0.41821235	0.032033263	36252.93	34553.56		14054.235	14450.726	3.394140991	1.967106531	2	High	2108	Unique	Used	57809	1	27.181	1	4.1649	16.602	1	99	18/22	0	2108	1768.84	MS2	884.925	109	Glioma_grade 2_3.raw
87578396			dAHIPVVEEHVMGk	Unambiguous	N-Term(iTRAQ4plex); K14(iTRAQ4plex)	HCD	0.44346792	0	1	1	0.46207	0.4973	0.3935	0.4234501	0.101858133	32399.889	30106.852		14970.983	12748.75	5.187990883	11.33745425	4	High	2276	Unique	Used	90066	1	28.992	1	3.946	3.8738	1	16	23/78	30.30974	2276	1849	MS2	463.004	215	Glioma_grade 2_7rep.raw
87578396			kIDLSHVTSk	Unambiguous	N-Term(iTRAQ4plex); K1(iTRAQ4plex); K10(iTRAQ4plex)	HCD	0.4900178	1	1	4	0.4794	0.5318	0.4524	0.5017947	0.068538395	29218.645	26341.973		14007.429	13218.262	7.322144179	4.099255642	3	High	1656	Unique	Used	85161	1	25.7	1	4.4266	9.7577	1	48	19/36	68.3596	1656	1559.96	MS2	520.659	63	Glioma_grade 2_1rep.raw
87578396			tGLPPVTDENHVIVk	Unambiguous	N-Term(iTRAQ4plex); K15(iTRAQ4plex)	HCD	0.5148454	0	1	1	0.47739	0.5567	0.479	0.5585152	0.088550155	28879.602	24766.607		13786.975	13832.527	10.84261762	0.233241918	2	High	2866	Unique	Used	66190	1	32.121	1	3.8078	5.2144	1	100	18/28	22.42434	2866	1907.09	MS2	954.051	195	Glioma_grade 2_4.raw
87578396			aPLALFGHTLVASLEDmk	Unambiguous	N-Term(iTRAQ4plex); M17(Oxidation); K18(iTRAQ4plex)	HCD	0.53193945	0	1	1	0.48805	0.5594	0.508	0.5822491	0.082013727	28553.162	24910.145		13935.335	14503.909	9.636523325	2.82737847	3	High	6645	Unique	Used	83426	1	67.414	1	6.6498	-0.2066	1	44	26/68	28.29463	6645	2217.22	MS2	739.744	106	Glioma_grade 2_6.raw
87578396			iVQVVTAEAVAVLk	Unambiguous	N-Term(iTRAQ4plex); K14(iTRAQ4plex)	HCD	0.29628733	0	1	4	0.29502	0.373	0.2356	0.29789183	0.187349875	28308.984	22391.166		8351.666	6670.145	16.50696985	15.8305134	3	High	8530	Unique	Used	55752	1	75.564	1	6.2768	-0.1539	1	19	24/52	15.36583	8530	1728.09	MS2	576.701	29	Glioma_grade 2_2.raw
87578396			ekDVFDDIPk	Unambiguous	N-Term(iTRAQ4plex); K2(iTRAQ4plex); K10(iTRAQ4plex)	HCD	0.48403394	1	1	1	0.44953	0.4775	0.4902	0.5206881	0.060924658	25297.19	23814.709		11371.791	12400.035	4.268914008	6.117143085	3	High	2786	Unique	Used	80149	1	33.001	1	4.3509	-6.2197	1	79	19/36	68.1722	2786	1637.9	MS2	546.639	230	Glioma_grade 2_6.raw
87578396			yTRPTHLScVk	Unambiguous	N-Term(iTRAQ4plex); C9(Methylthio); K11(iTRAQ4plex)	HCD	0.34999436	0	1	1	0.31991	0.2949	0.4097	0.37772876	0.149796445	25125.248	27253.67		8037.8315	10294.495	5.746669412	17.40861493	4	High	1482	Unique	Used	89370	1	22.424	1	3.9563	6.5078	1	26	16/60	47.81328	1482	1638.88	MS2	410.476	276	Glioma_grade 2_7rep.raw
87578396			yTRPTHLScVk	Unambiguous	N-Term(iTRAQ4plex); C9(Methylthio); K11(iTRAQ4plex)	HCD	0.38052562	0	1	1	0.38804	0.3746	0.3866	0.37326786	0.020411494	20654.807	21394.121		8014.9556	7985.7383	2.48650307	0.258235688	4	High	1460	Unique	Used	89350	1	22.255	1	3.77	-4.0689	1	34	16/60	36.04343	1460	1638.86	MS2	410.471	273	Glioma_grade 2_7rep.raw
87578396			dAHIPVVEEHVMGk	Unambiguous	N-Term(iTRAQ4plex); K14(iTRAQ4plex)	HCD	0.51698565	0	1	1	0.55897	0.5272	0.5061	0.47738674	0.066509208	16138.095	17109.426		9020.678	8167.8135	4.131644804	7.017093634	4	High	2049	Unique	Used	79492	1	26.998	1	3.4769	3.4777	1	25	18/78	76.81135	2049	1848.99	MS2	463.004	222	Glioma_grade 2_6.raw
87578396			kDEWGLVAPISPGPLTPMR	Unambiguous	N-Term(iTRAQ4plex); K1(iTRAQ4plex)	HCD	0.5013736	1	1	1	0.53882	0.5829	0.426	0.46086246	0.142478085	13972.528	12916.87		7528.7334	5952.9004	5.552098492	16.53037335	3	High	5375	Unique	Used	82390	1	55.392	1	5.8873	-0.4343	1	33	24/72	21.23424	5375	2352.3	MS2	784.771	140	Glioma_grade 2_6.raw
87578396			rPSPHDEEEFEVEEAAEAQAEPk	Unambiguous	N-Term(iTRAQ4plex); K23(iTRAQ4plex)	HCD	0.63016886	0	1	1	0.5439	0.6154	0.6432	0.7277884	0.120168558	12156.594	10743.383		6611.943	7818.909	8.72744179	11.82818372	3	High	2932	Unique	Used	90621	1	34.822	1	8.7069	-5.5595	1	32	27/88	6.506643	2932	2912.36	MS2	971.459	70	Glioma_grade 2_7rep.raw
89191865	NP_000202.2	ITGB2	integrin beta-2 precursor [Homo sapiens]	5	6	2.948132992	32.74518555	30.42318	10.66	1.77E-04	769	84.726	6.9468	Adhesion molecule	Cell communication,Signal transduction	Cell adhesion molecule activity	Plasma membrane	Mitochondrion																									
89191865			tVLPFVNTHPDk	Unambiguous	N-Term(iTRAQ4plex); K12(iTRAQ4plex)	HCD	4.136379	0	1	1	4.11243	3.8765	4.4121	4.158952	5.303617283	80476.15	85375.16		330952.62	355071.2	4.177384179	4.971958983	3	High	2584	Unique	Used	79991	1	30.983	1	5.1717	5.4858	1	6	30/44	13.11899	2584	1655.95	MS2	552.653	263	Glioma_grade 2_6.raw
89191865			lLVFATDDGFHFAGDGk	Unambiguous	N-Term(iTRAQ4plex); K17(iTRAQ4plex)	HCD	4.429073	0	1	1	4.44607	3.9317	4.9916	4.414044	9.745788929	67440.22	76263.83		299843.78	336631.9	8.683449723	8.174115661	3	High	5869	Unique	Used	82813	1	59.721	1	5.8131	1.9323	1	7	31/64	6.355571	5869	2098.09	MS2	700.035	213	Glioma_grade 2_6.raw
89191865			vFLDHNALPDTLk	Unambiguous	N-Term(iTRAQ4plex); K13(iTRAQ4plex)	HCD	2.8217015	0	1	1	2.63404	2.9075	2.744	3.0288508	6.169077437	13656.53	12371.983		35971.805	37472.89	6.97936063	2.890412664	2	High	3958	Unique	Used	81181	1	42.855	1	3.9172	2.6983	1	100	18/24	15.66748	3958	1771	MS2	886.006	191	Glioma_grade 2_6.raw
89191865			sNEFDYPSVGQLAHk	Unambiguous	N-Term(iTRAQ4plex); K15(iTRAQ4plex)	HCD	3.0745642	0	1	1	2.70357	2.9272	3.2107	3.476245	10.91855488	12479.126	11525.8545		33738.16	40066.695	5.616040738	12.12643806	3	High	3989	Unique	Used	67232	1	40.824	1	6.2758	-0.2736	1	46	26/56	21.55695	3989	1980.01	MS2	660.674	133	Glioma_grade 2_4.raw
89191865			sNEFDYPSVGQLAHk	Unambiguous	N-Term(iTRAQ4plex); K15(iTRAQ4plex)	HCD	1.8228321	0	1	1	1.66654	1.9757	1.6939	2.008116	9.847429764	10201.66	8605.144		17001.52	17280.127	12.00530712	1.149331588	3	High	3355	Unique	Used	73968	1	37.131	1	4.9283	0.7437	1	32	28/56	30.21991	3355	1980.01	MS2	660.674	159	Glioma_grade 2_5.raw
89191865			gcAADDIMDPTSLAETQEDHNGGQk	Unambiguous	N-Term(iTRAQ4plex); C2(Methylthio); K25(iTRAQ4plex)	HCD	2.3216546	0	1	1	2.35674	2.4875	2.1645	2.2846234	5.816396284	1138.5094	1078.6613		2683.1697	2464.3345	3.817387389	6.012228368	3	High	5172	Unique	Used	68324	1	50.12	1	4.3172	-5.9981	1	100	13/96	31.04202	5172	2937.27	MS2	979.762	17	Glioma_grade 2_4.raw
89903008	NP_055905.2	NFASC	neurofascin isoform 4 precursor [Homo sapiens]	8	27	0.402391523	20.89097605	152.44492	10.01	1.49E-04	1169	131.59	6.8735	Adhesion molecule	Cell communication,Signal transduction	Cell adhesion molecule activity	Plasma membrane	-																									
89903008			lTVSWLk	Unambiguous	N-Term(iTRAQ4plex); K7(iTRAQ4plex)	HCD	0.38543049	0	1	6	0.38049	0.3652	0.4065	0.3901696	0.044882967	731071.8	761644.8		278167.8	297170.66	2.896514398	4.671007451	2	High	5212	Unique	Used	68361	1	50.427	1	3.0097	-4.2637	1	23	12-Dec	41.78936	5212	1134.71	MS2	567.858	241	Glioma_grade 2_4.raw
89903008			lTVSWLk	Unambiguous	N-Term(iTRAQ4plex); K7(iTRAQ4plex)	HCD	0.35602885	0	1	6	0.3213	0.3593	0.3531	0.39487168	0.084458459	596807.25	533672.75		191751.23	210732.27	7.898031469	6.669402397	2	High	5163	Unique	Used	52636	1	49.627	1	3.0682	-9.1048	1	20	12-Dec	41.92338	5163	1134.7	MS2	567.855	251	Glioma_grade 2_2.raw
89903008			aAPYWLDEPk	Unambiguous	N-Term(iTRAQ4plex); K10(iTRAQ4plex)	HCD	0.36108738	0	1	6	0.33791	0.3778	0.3462	0.38700268	0.06581878	561046.25	501838.66		189581.42	194212.9	7.877821577	1.706617709	2	High	3863	Unique	Used	59442	1	40.463	1	4.2272	0.0264	1	18	17/18	15.49817	3863	1477.79	MS2	739.4	250	Glioma_grade 2_3.raw
89903008			lDcPFFGSPIPTLR	Unambiguous	N-Term(iTRAQ4plex); C3(Methylthio)	HCD	0.32497245	0	1	6	0.31416	0.3129	0.3371	0.33573854	0.040769943	477157.2	479118.38		149904.7	160858.52	0.290034318	4.984837267	2	High	8573	Unique	Used	55792	1	75.882	1	5.1212	2.4687	1	14	19/26	34.43506	8573	1752.9	MS2	876.953	205	Glioma_grade 2_2.raw
89903008			lTVSWLk	Unambiguous	N-Term(iTRAQ4plex); K7(iTRAQ4plex)	HCD	0.395455	0	1	6	0.36142	0.3839	0.4063	0.43160123	0.076017549	387514.47	364829.28		140057.34	157460.77	4.264234715	8.272493643	2	High	4984	Unique	Used	60479	1	49.285	1	2.9376	-4.4789	1	36	12-Dec	58.31627	4984	1134.71	MS2	567.857	242	Glioma_grade 2_3.raw
89903008			lDcPFFGSPIPTLR	Unambiguous	N-Term(iTRAQ4plex); C3(Methylthio)	HCD	0.35730034	0	1	6	0.3623	0.362	0.3526	0.35230452	0.015752971	193476.14	193611.25		70096.34	68210.12	0.049362082	1.928700876	2	High	5953	Unique	Used	88115	1	75.849	1	4.6764	-0.2472	1	44	20/26	41.733	5953	1752.89	MS2	876.95	207	Glioma_grade 2_1rep.raw
89903008			fHFTHTIQQk	Unambiguous	N-Term(iTRAQ4plex); K10(iTRAQ4plex)	HCD	0.3919086	0	1	6	0.37999	0.386	0.3977	0.40401602	0.027858466	157629.78	155179.52		59897.633	62695.008	1.107764674	3.227017243	2	High	1602	Unique	Used	85116	1	25.198	1	4.8698	-5.3328	1	90	16/18	14.82792	1602	1574.86	MS2	787.934	253	Glioma_grade 2_1rep.raw
89903008			fHFTHTIQQk	Unambiguous	N-Term(iTRAQ4plex); K10(iTRAQ4plex)	HCD	0.40239152	0	1	6	0.38727	0.378	0.4274	0.4171501	0.058585991	150370.81	154065.97		58234.137	64268.637	1.716528925	6.966431423	2	High	1616	Unique	Used	85128	1	25.326	1	4.8281	5.2087	1	69	17/18	20.03677	1616	1574.88	MS2	787.942	231	Glioma_grade 2_1rep.raw
89903008			aAPYWLDEPk	Unambiguous	N-Term(iTRAQ4plex); K10(iTRAQ4plex)	HCD	0.5750355	0	1	6	0.54376	0.5866	0.5643	0.6087744	0.048716084	133168.4	123449.81		72411.516	75153.086	5.355879376	2.627436	2	High	3938	Unique	Used	51485	1	40.604	1	4.18	-0.0562	1	41	16/18	12.68358	3938	1477.79	MS2	739.4	263	Glioma_grade 2_2.raw
89903008			fHFTHTIQQk	Unambiguous	N-Term(iTRAQ4plex); K10(iTRAQ4plex)	HCD	0.38704565	0	1	6	0.36613	0.3567	0.4182	0.4074241	0.078064212	100861.96	103519.34		36928.62	42176.273	1.838770395	9.381596715	4	High	1599	Unique	Used	85113	1	25.179	1	4.8764	-9.148	1	24	15/54	49.85246	1599	1574.85	MS2	394.469	260	Glioma_grade 2_1rep.raw
89903008			fHFTHTIQQk	Unambiguous	N-Term(iTRAQ4plex); K10(iTRAQ4plex)	HCD	0.44714996	0	1	6	0.45542	0.433	0.462	0.4392889	0.030330803	100592.266	105804.29		45811.562	46478.652	3.571239352	1.022218591	3	High	1586	Unique	Used	85102	1	25.062	1	5.5356	-1.5433	1	44	18/36	42.11746	1586	1574.87	MS2	525.627	242	Glioma_grade 2_1rep.raw
89903008			vIAINEVGSSHPSLPSER	Unambiguous	N-Term(iTRAQ4plex)	HCD	0.6440281	0	1	4	0.63057	0.6533	0.6351	0.657968	0.020861745	96533.7	93172.99		60871.598	61304.848	2.505321062	0.501494393	3	High	3766	Unique	Used	67025	1	39.092	1	4.692	2.6204	1	14	28/68	26.59705	3766	2036.09	MS2	679.37	124	Glioma_grade 2_4.raw
89903008			vIAINEVGSSHPSLPSER	Unambiguous	N-Term(iTRAQ4plex)	HCD	0.5062047	0	1	4	0.49461	0.4773	0.5362	0.51739347	0.050906382	76941.38	79733.41		38055.977	41253.543	2.520205511	5.701763425	3	High	3552	Unique	Used	59154	1	38.054	1	5.7528	1.6312	1	10	34/68	4.783393	3552	2036.09	MS2	679.369	118	Glioma_grade 2_3.raw
89903008			kEDQGIYTcVATNILGk	Unambiguous	N-Term(iTRAQ4plex); K1(iTRAQ4plex); C9(Methylthio); K17(iTRAQ4plex)	HCD	0.30223796	1	1	6	0.27206	0.304	0.3006	0.33597037	0.086250682	76072.16	68067.26		20695.82	22868.582	7.853950117	7.053349403	2	High	5508	Unique	Used	82500	1	56.618	1	5.4232	-2.7455	1	89	18/32	54.39992	5508	2331.23	MS2	1166.12	162	Glioma_grade 2_6.raw
89903008			fHFTHTIQQk	Unambiguous	N-Term(iTRAQ4plex); K10(iTRAQ4plex)	HCD	0.34740806	0	1	6	0.33443	0.3422	0.3525	0.36069182	0.033122021	61382.31	59982.465		20527.914	21635.184	1.631181522	3.713949699	2	High	1770	Unique	Used	94663	1	26.994	1	4.5459	-1.6123	1	100	18/18	9.53175	1770	1574.87	MS2	787.936	224	Glioma_grade 2_8rep.raw
89903008			vIAINEVGSSHPSLPSER	Unambiguous	N-Term(iTRAQ4plex)	HCD	0.5439994	0	1	4	0.53535	0.5187	0.5702	0.55237633	0.040699709	58742.98	60633.953		31448.217	33492.76	2.24016449	4.452382106	2	High	3793	Unique	Used	67050	1	39.298	1	6.018	1.0382	1	57	25/34	6.563583	3793	2036.09	MS2	1018.55	141	Glioma_grade 2_4.raw
89903008			kEDDSLTIFGVAER	Unambiguous	N-Term(iTRAQ4plex); K1(iTRAQ4plex)	HCD	0.3961241	1	1	6	0.3808	0.3932	0.399	0.41194546	0.032650417	58014.168	56189.96		22091.807	23147.201	2.258954855	3.299260029	2	High	3771	Unique	Used	81029	1	41.048	1	4.6391	2.732	1	100	21/26	11.00034	3771	1868.01	MS2	934.506	204	Glioma_grade 2_6.raw
89903008			vIAINEVGSSHPSLPSER	Unambiguous	N-Term(iTRAQ4plex)	HCD	0.42170587	0	1	4	0.38427	0.4065	0.4361	0.46130875	0.079847482	54764.297	51768.418		21044.25	23881.225	3.97700623	8.930542239	2	High	3564	Unique	Used	59165	1	38.148	1	6.1642	0.079	1	64	25/34	17.44302	3564	2036.09	MS2	1018.55	133	Glioma_grade 2_3.raw
89903008			sGGRPEEYEGEYQcFAR	Unambiguous	N-Term(iTRAQ4plex); C14(Methylthio)	HCD	0.33053866	0	1	6	0.35581	0.3114	0.3524	0.30842417	0.077018344	53709.105	61364.227		19109.975	18926.21	9.407894224	0.683251896	3	High	3698	Unique	Used	66961	1	38.574	1	3.7706	1.0658	1	17	22/64	23.97895	3698	2167.93	MS2	723.315	17	Glioma_grade 2_4.raw
89903008			fHFTHTIQQk	Unambiguous	N-Term(iTRAQ4plex); K10(iTRAQ4plex)	HCD	0.4339282	0	1	6	0.38732	0.3425	0.5373	0.47514477	0.200644675	53491.324	60492.332		20718.105	28742.617	8.686263287	22.9442136	4	High	1588	Unique	Used	85104	1	25.075	1	5.1384	5.3467	1	39	13/54	55.39494	1588	1574.88	MS2	394.475	261	Glioma_grade 2_1rep.raw
89903008			kEDDSLTIFGVAER	Unambiguous	N-Term(iTRAQ4plex); K1(iTRAQ4plex)	HCD	0.3228182	1	1	6	0.2821	0.3202	0.3252	0.36903015	0.109797274	49299.3	43437.83		13907.363	16029.868	8.93856686	10.0265631	2	High	4006	Unique	Used	74536	1	42.67	1	4.5649	0.3795	1	83	22/26	0	4006	1868	MS2	934.504	169	Glioma_grade 2_5.raw
89903008			vIAINEVGSSHPSLPSER	Unambiguous	N-Term(iTRAQ4plex)	HCD	0.44101632	0	1	4	0.39659	0.4077	0.4734	0.4866928	0.103196155	48961.688	47623.56		19417.627	23178.041	1.959304143	12.48490452	2	High	3614	Unique	Used	51178	1	38.299	1	6.0064	-2.6189	1	51	26/34	0	3614	2036.08	MS2	1018.55	149	Glioma_grade 2_2.raw
89903008			kEDQGIYTcVATNILGk	Unambiguous	N-Term(iTRAQ4plex); K1(iTRAQ4plex); C9(Methylthio); K17(iTRAQ4plex)	HCD	0.5721845	1	1	6	0.5351	0.588	0.5578	0.6129344	0.059432533	45260.53	41190.98		24218.846	25247.371	6.657157062	2.940499784	3	High	5526	Unique	Used	82515	1	56.785	1	7.66	-1.2589	1	20	29/64	44.58586	5526	2331.24	MS2	777.751	168	Glioma_grade 2_6.raw
89903008			fHFTHTIQQk	Unambiguous	N-Term(iTRAQ4plex); K10(iTRAQ4plex)	HCD	0.48554608	0	1	6	0.46278	0.4788	0.492	0.50910115	0.040490144	35454.92	34266.375		16407.852	17445.05	2.410822201	4.332920937	3	High	1759	Unique	Used	94654	1	26.886	1	5.523	4.3863	1	100	18/36	28.53643	1759	1574.88	MS2	525.63	221	Glioma_grade 2_8rep.raw
89903008			fHFTHTIQQk	Unambiguous	N-Term(iTRAQ4plex); K10(iTRAQ4plex)	HCD	0.42619222	0	1	6	0.4239	0.4004	0.4535	0.42835513	0.050947355	24175.324	25592.322		10247.981	10962.603	4.026587453	4.764735023	4	High	1763	Unique	Used	94657	1	26.928	1	4.3468	-2.482	1	100	13/54	52.98636	1763	1574.86	MS2	394.472	252	Glioma_grade 2_8rep.raw
89903008			kEDDSLTIFGVAER	Unambiguous	N-Term(iTRAQ4plex); K1(iTRAQ4plex)	HCD	0.56816244	1	1	6	0.5919	0.5724	0.5638	0.5452076	0.034111569	23937.71	24752.951		14168.708	13495.498	2.367856289	3.441496612	3	High	3761	Unique	Used	81020	1	40.968	1	6.0934	2.6268	1	23	22/52	35.39552	3761	1868.01	MS2	623.34	209	Glioma_grade 2_6.raw
89903008			sGGRPEEYEGEYQcFAR	Unambiguous	N-Term(iTRAQ4plex); C14(Methylthio)	HCD	0.46227628	0	1	6	0.42588	0.4673	0.4577	0.5022206	0.067843575	21488.629	19581.795		9151.502	9834.381	6.565967042	5.086604312	3	High	3441	Unique	Used	59053	1	37.152	1	3.2654	1.1502	1	85	22/64	0	3441	2167.93	MS2	723.316	21	Glioma_grade 2_3.raw
90903247	NP_005935.4	CD200	OX-2 membrane glycoprotein isoform a precursor [Homo sapiens]	4	10	0.421630979	28.68043045	52.064329	24.16	6.82E-05	269	30.312	8.353	Unclassified, Cell surface receptor	Immune response	Molecular function unknown,Immunoglobulin receptor activity	Plasma membrane	-																									
90903247			eQLYTPASLk	Unambiguous	N-Term(iTRAQ4plex); K10(iTRAQ4plex)	HCD	0.6310158	0	1	2	0.59394	0.6222	0.6394	0.6698576	0.05034344	211566.78	201951.28		125657.81	135278.6	3.288458673	5.214240396	2	High	2722	Unique	Used	50342	1	31.791	1	3.6615	5.7468	1	38	14/18	37.38435	2722	1437.83	MS2	719.417	256	Glioma_grade 2_2.raw
90903247			eQLYTPASLk	Unambiguous	N-Term(iTRAQ4plex); K10(iTRAQ4plex)	HCD	0.5481266	0	1	2	0.54267	0.5727	0.5248	0.5538886	0.036653398	191438.58	181384.61		103887.43	100466.87	3.813727555	2.367164451	2	High	2635	Unique	Used	50261	1	31.154	1	3.3883	3.9639	1	30	14/18	27.60424	2635	1437.82	MS2	719.416	249	Glioma_grade 2_2.raw

90903247			eVIcQVLHLGTVTDFk	Unambiguous	N-Term(iTRAQ4plex); C4(Methylthio); K16(iTRAQ4plex)	HCD	0.29898232	0	1	2	0.27271	0.3022	0.296	0.32810244	0.075950309	152266.03	137385.78	41524.16	45076.61	7.265223498	5.80124515	3	High	7011	Unique	Used	77189	1	67.862	1	4.8344	-2.3521	1	5	29/60	9.557055	7011	2136.14	MS2	712.718	158	Glioma_grade 2_5.raw
90903247			eVIcQVLHLGTVTDFk	Unambiguous	N-Term(iTRAQ4plex); C4(Methylthio); K16(iTRAQ4plex)	HCD	0.3544367	0	1	2	0.30293	0.3019	0.4072	0.40575933	0.16952371	69307.305	69549.97	20995.564	28220.55	0.247145952	20.76082884	3	High	7091	Unique	Used	77256	1	68.601	1	5.063	-0.1234	1	10	29/60	16.98817	7091	2136.14	MS2	712.719	163	Glioma_grade 2_5.raw
90903247			eVIcQVLHLGTVTDFk	Unambiguous	N-Term(iTRAQ4plex); C4(Methylthio); K16(iTRAQ4plex)	HCD	0.43264124	0	1	2	0.42972	0.4013	0.4662	0.43537125	0.061432166	51330.484	54966.004	22057.627	23930.617	4.83685001	5.759749949	2	High	7022	Unique	Used	77199	1	67.945	1	6.1951	-4.1173	1	48	22/30	19.00437	7022	2136.13	MS2	1068.57	201	Glioma_grade 2_5.raw
90903247			cSLQNAQEALIVTWQk	Unambiguous	N-Term(iTRAQ4plex); C1(Methylthio); K16(iTRAQ4plex)	HCD	0.41062075	0	1	2	0.40055	0.4161	0.4054	0.42107832	0.023025106	44265.73	42612.36	17730.805	17943.14	2.691378549	0.841754498	2	High	7455	Unique	Used	54777	1	66.742	1	6.8795	0.6465	1	58	23/30	13.15583	7455	2166.13	MS2	1083.57	179	Glioma_grade 2_2.raw
90903247			aVSPENMVTFSENHGVVIQPAYk	Unambiguous	N-Term(iTRAQ4plex); K23(iTRAQ4plex)	HCD	0.3321633	0	1	2	0.30071	0.3544	0.3133	0.36923867	0.097624592	19661.707	16680.914	5912.4463	6159.2383	11.59926767	2.8912004	3	High	5865	Unique	Used	68961	1	55.565	1	6.4229	1.4766	1	48	28/88	9.810098	5865	2805.45	MS2	935.822	80	Glioma_grade 2_4.raw
90903247			eVIcQVLHLGTVTDFk	Unambiguous	N-Term(iTRAQ4plex); C4(Methylthio); K16(iTRAQ4plex)	HCD	0.45635334	0	1	2	0.44909	0.4178	0.4978	0.4631147	0.072572798	13775.358	14806.559	6186.3184	6857.1353	5.102311506	7.273214439	3	High	6639	Unique	Used	83421	1	67.359	1	5.0853	0.3909	1	79	26/60	21.49451	6639	2136.14	MS2	712.72	163	Glioma_grade 2_6.raw
90903247			cSLQNAQEALIVTWQk	Unambiguous	N-Term(iTRAQ4plex); C1(Methylthio); K16(iTRAQ4plex)	HCD	0.60801077	0	1	2	0.62513	0.6549	0.5632	0.590076	0.065898736	13340.795	12733.761	8339.725	7513.8867	3.292388624	7.366849563	3	High	7466	Unique	Used	54787	1	66.833	1	5.9617	2.0491	1	17	28/60	18.54641	7466	2166.13	MS2	722.716	172	Glioma_grade 2_2.raw
90903247			eVIcQVLHLGTVTDFk	Unambiguous	N-Term(iTRAQ4plex); C4(Methylthio); K16(iTRAQ4plex)	HCD	0.2842107	0	1	2	0.30559	0.2746	0.2949	0.26500237	0.06505241	3747.2327	4170.5557	1145.113	1105.2072	7.561065005	2.507879704	4	High	7023	Unique	Used	77200	1	67.952	1	4.5726	-3.6156	1	57	20/90	13.17791	7023	2136.14	MS2	534.789	187	Glioma_grade 2_5.raw
91932795	NP_001035191.1	OPALIN	opalin isoform b [Homo sapiens]	3	4	0.360132754	19.99370201	20.528798	44.92	8.90E-05	118	13.336	6.1763	Integral membrane protein	Biological_process unknown	Molecular function unknown	Integral to membrane	-																								
91932795			yRPTIEMER	Unambiguous	N-Term(iTRAQ4plex)	HCD	0.4703748	0	1	3	0.45491	0.4813	0.4601	0.48673338	0.033179831	182020.86	172043.48	82803.64	83739.305	3.985192667	0.794527882	2	High	1419	Unique	Used	64850	1	21.235	1	3.1026	1.0344	1	24	16-Sep	30.13545	1419	1338.7	MS2	669.852	212	Glioma_grade 2_4.raw
91932795			gLWWLVPR	Unambiguous	N-Term(iTRAQ4plex)	HCD	0.30969822	0	1	3	0.28529	0.3011	0.3179	0.3354555	0.069715241	168224.86	159411.48	47992.934	53475.465	3.804218276	7.641265431	2	High	7429	Unique	Used	54754	1	66.518	1	3.4487	1.888	1	28	13/14	31.61762	7429	1170.69	MS2	585.85	215	Glioma_grade 2_2.raw
91932795			gLWWLVPR	Unambiguous	N-Term(iTRAQ4plex)	HCD	0.32095832	0	1	3	0.30258	0.3236	0.3185	0.3406146	0.048778968	117170.95	109548.49	35453.68	37313.816	4.754681077	3.615116231	2	High	7352	Unique	Used	54689	1	65.807	1	3.4106	-1.8658	1	45	13/14	19.95471	7352	1170.69	MS2	585.848	134	Glioma_grade 2_2.raw
91932795			nTMGAQEAHIYVk	Unambiguous	N-Term(iTRAQ4plex); K13(iTRAQ4plex)	HCD	0.39930716	0	1	3	0.38135	0.4064	0.3927	0.41843936	0.040382865	115479.53	108369.55	44038.453	45346.082	4.491879147	2.068888837	3	High	1322	Unique	Used	72094	1	21.751	1	5.1223	3.5888	1	19	24/48	43.60932	1322	1749.93	MS2	583.98	174	Glioma_grade 2_5.raw
93141210	NP_066287.2	SCN2A	sodium channel protein type 2 subunit alpha isoform 1 [Homo sapiens]	4	5	0.443229169	23.68307199	33.794084	4.49	2.69E-04	2005	227.83	5.7319	Voltage gated channel	Transport	Voltage-gated ion channel activity	Plasma membrane	-																								
93141210			fDPDATQFIEFAk	Unambiguous	N-Term(iTRAQ4plex); K13(iTRAQ4plex)	HCD	0.41512173	0	1	2	0.37926	0.4196	0.4111	0.4547948	0.074594967	184330.23	166623.83	69909.016	75779.65	7.135016766	5.698679554	2	High	6865	Unique	Used	54236	1	62.113	1	5.2521	2.914	1	29	20/24	12.97519	6865	1816.94	MS2	908.974	198	Glioma_grade 2_2.raw
93141210			tVSIFNWDEYIEDk	Unambiguous	N-Term(iTRAQ4plex); K14(iTRAQ4plex)	HCD	0.44322917	0	1	2	0.42242	0.4241	0.4622	0.46412748	0.052034925	121797.1	121301.984	51449.023	56299.586	0.288030605	6.366422772	2	High	7789	Unique	Used	55080	1	69.451	1	6.1379	-2.9243	1	38	19/26	35.78801	7789	2047.02	MS2	1024.01	166	Glioma_grade 2_2.raw
93141210			kQEEVSAIIIQR	Unambiguous	N-Term(iTRAQ4plex); K1(iTRAQ4plex)	HCD	0.5715997	1	1	2	0.53072	0.5713	0.5719	0.615602	0.060565506	59326.504	55117.14	31485.734	33930.223	5.20163414	5.284688408	2	High	2001	Unique	Used	72708	1	27.051	1	3.3151	8.5243	1	100	17/22	18.03146	2001	1702.02	MS2	851.516	94	Glioma_grade 2_5.raw
93141210			fDPDATQFIEFAk	Unambiguous	N-Term(iTRAQ4plex); K13(iTRAQ4plex)	HCD	0.47686315	0	1	2	0.39624	0.4252	0.525	0.5633896	0.166536153	24546.902	22873.314	9726.366	12886.587	4.991143118	19.76401489	3	High	6864	Unique	Used	54235	1	62.107	1	4.2675	2.2683	1	19	19/48	11.82004	6864	1816.94	MS2	606.318	193	Glioma_grade 2_2.raw
93141210			tDMTPSTTSPPSYDSVTkPEk	Unambiguous	N-Term(iTRAQ4plex); K18(iTRAQ4plex); K21(iTRAQ4plex)	HCD	0.28559554	0	1	2	0.27586	0.2689	0.3027	0.29508796	0.055646746	19450.072	19954.268	5365.44	5888.264	1.809548951	6.570146074	3	High	2230	Unique	Used	72921	1	28.745	1	5.4672	-1.034	1	34	24/80	11.42442	2230	2701.36	MS2	901.124	94	Glioma_grade 2_5.raw
94818891	NP_001035548.1	ERAP1	endoplasmic reticulum aminopeptidase 1 isoform b precursor [Homo sapiens]	6	6	2.013364315	15.78113535	45.030374	9.99	2.65E-05	941	107.17	6.4556	Aminopeptidase	Protein metabolism	Aminopeptidase activity	Endoplasmic reticulum	Mitochondrion, Cytoplasm																								
94818891			sGIVQYLQk	Unambiguous	N-Term(iTRAQ4plex); K9(iTRAQ4plex)	HCD	1.6441954	0	1	2	1.51075	1.5966	1.6893	1.7852211	7.189960766	173733.4	164395.33	262467.72	293482.03	3.905620572	7.889356516	2	High	3652	Unique	Used	51214	1	38.567	1	3.2701	3.3543	1	23	14/16	51.39451	3652	1323.79	MS2	662.4	249	Glioma_grade 2_2.raw
94818891			eMFDDVSYDk	Unambiguous	N-Term(iTRAQ4plex); K10(iTRAQ4plex)	HCD	1.9167428	0	1	2	1.88122	1.9628	1.8726	1.9538037	2.465059745	46102.523	44186.25	86729.09	86331.26	3.001501932	0.325098488	2	High	3617	Unique	Used	51181	1	38.319	1	4.2775	-1.2026	1	85	17/18	14.91225	3617	1536.71	MS2	768.859	86	Glioma_grade 2_2.raw
94818891			vGDYFFGk	Unambiguous	N-Term(iTRAQ4plex); K8(iTRAQ4plex)	HCD	2.1047897	0	1	2	2.18019	2.0532	2.1596	2.033785	3.506677013	45619.82	48441.098	99459.67	98518.79	4.241814449	0.672095973	2	High	4793	Unique	Used	67977	1	47.099	1	2.915	0.7294	1	91	14-Dec	33.07586	4793	1220.66	MS2	610.832	292	Glioma_grade 2_4.raw
94818891			lQWLLDESFk	Unambiguous	N-Term(iTRAQ4plex); K10(iTRAQ4plex)	HCD	2.5679789	0	1	2	2.48137	2.4075	2.7334	2.6520138	5.846650398	26965.15	27792.22	66910.42	73705.35	2.136066088	6.833858081	2	High	7323	Unique	Used	70281	1	67.593	1	4.489	1.4505	1	100	16/18	37.75615	7323	1566.88	MS2	783.943	275	Glioma_grade 2_4.raw
94818891			mAFPcFDEPAFk	Unambiguous	N-Term(iTRAQ4plex); C5(Methylthio); K12(iTRAQ4plex)	HCD	2.3211613	0	1	2	2.43629	2.1501	2.515	2.2195544	7.435543449	10742.042	12171.74	26170.746	27015.842	8.823939678	2.247081961	2	High	7466	Unique	Used	70408	1	68.811	1	3.6453	0.5319	1	100	15/22	26.72629	7466	1736.81	MS2	868.909	114	Glioma_grade 2_4.raw
94818891			gSDGAPDTGYLWHVPLTFITSk	Unambiguous	N-Term(iTRAQ4plex); K22(iTRAQ4plex)	HCD	1.9219389	0	1	2	1.87259	2.0208	1.8303	1.9751986	4.589900785	3764.6165	3488.4727	7049.5674	6890.427	5.384275532	1.614480649	3	High	7130	Unique	Used	77290	1	68.941	1	5.834	0.5667	1	67	29/84	31.70997	7130	2650.38	MS2	884.131	111	Glioma_grade 2_5.raw
95147555	NP_002364.5	MAP1A	microtubule-associated protein 1A [Homo sapiens]	20	34	0.494077444	17.48224171	225.24335	13.31	5.95E-04	2803	305.3	4.9194	Cytoskeletal associated protein	Cell growth and/or maintenance	Cytoskeletal protein binding	Cytoplasm	Perinuclear region, Nucleus, Plasma membrane																								
95147555			lEALLEk	Unambiguous	N-Term(iTRAQ4plex); K7(iTRAQ4plex)	HCD	0.38207325	0	1	1	0.35251	0.3856	0.3788	0.4144208	0.066450704	502930.4	459715.88	177285.56	190515.83	6.348599849	5.08710075	2	High	4324	Unique	Used	59872	1	44.025	1	3.0988	9.9419	1	25	12-Dec	69.40717	4324	1103.7	MS2	552.355	182	Glioma_grade 2_3.raw
95147555			dLASGAVPTNLkPSk	Unambiguous	N-Term(iTRAQ4plex); K12(iTRAQ4plex); K15(iTRAQ4plex)	HCD	0.50692123	0	1	1	0.51271	0.5562	0.4615	0.50064343	0.076804381	354569.7	326841.94	181790.75	163631.27	5.754667509	7.434784529	3	High	2106	Unique	Used	79544	1	27.442	1	4.7703	2.5592	1	8	25/56	21.00468	2106	1930.14	MS2	644.051	88	Glioma_grade 2_6.raw
95147555			lEALLEk	Unambiguous	N-Term(iTRAQ4plex); K7(iTRAQ4plex)	HCD	0.4880924	0	1	1	0.4375	0.5162	0.4643	0.54779124	0.101313056	243412.22	206298.61	106491.875	113008.57	11.67118226	4.19862404	2	High	4481	Unique	Used	51996	1	44.626	1	2.9724	-6.7589	1	47	12-Dec	67.78453	4481	1103.68	MS2	552.346	254	Glioma_grade 2_2.raw
95147555			vLFPGNAPQNk	Unambiguous	N-Term(iTRAQ4plex); K11(iTRAQ4plex)	HCD	0.54714847	0	1	1	0.5038	0.5836	0.5157	0.59736854	0.085822773	168120.03	145127.19	84698.32	86694.414	10.3805506	1.647037853	2	High	2077	Unique	Used	57780	1	26.953	1	3.8116	8.6642	1	58	16/20	6.869566	2077	1472.86	MS2	736.933	245	Glioma_grade 2_3.raw
95147555			tLPQEPGkPQk	Unambiguous	N-Term(iTRAQ4plex); K8(iTRAQ4plex); K11(iTRAQ4plex)	HCD	0.43293625	0	1	1	0.41581	0.4391	0.4271	0.45102668	0.035072477	162294.95	153680.02	67483.234	69313.78	3.855796187	1.892426526	2	High	1080	Unique	Used	89007	1	19.337	1	3.8429	10.853	1	63	20-Dec	35.14758	1080	1655	MS2	828.005	111	Glioma_grade 2_7rep.raw
95147555			sIEEAcLTLQHLNR	Unambiguous	N-Term(iTRAQ4plex); C6(Methylthio)	HCD	0.3421796	0	1	1	0.31347	0.2833	0.4073	0.36812764	0.161504509	155632.97	172212.58	48785.844	63396.21	7.151876645	18.41843415	3	High	6506	Unique	Used	61897	1	61.109	1	5.8804	0.6354	1	4	30/52	14.39572	6506	1816.92	MS2	606.311	143	Glioma_grade 2_3.raw
95147555			sIEEAcLTLQHLNR	Unambiguous	N-Term(iTRAQ4plex); C6(Methylthio)	HCD	0.47372574	0	1	1	0.44608	0.4852	0.4632	0.5037891	0.053116441	116676.16	107279.71	52047.305	54046.35	5.93357389	2.664698988	2	High	6529	Unique	Used	61918	1	61.289	1	5.628	2.5576	1	47	21/26	45.52621	6529	1816.92	MS2	908.964	216	Glioma_grade 2_3.raw
95147555			sIEEAcLTLQHLNR	Unambiguous	N-Term(iTRAQ4plex); C6(Methylthio)	HCD	0.44704866	0	1	1	0.43547	0.4458	0.4483	0.45890337	0.021522894	103804.86	101405.23	45203.695	46535.2	1.653714635	2.052599859	2	High	6607	Unique	Used	69640	1	61.537	1	4.7778	1.9529	1	47	21/26	16.24805	6607	1816.92	MS2	908.964	168	Glioma_grade 2_4.raw
95147555			sPESLPGPALEDIAIk	Unambiguous	N-Term(iTRAQ4plex); K16(iTRAQ4plex)	HCD	0.43829605	0	1	1	0.39189	0.4412	0.4357	0.4905421	0.091780359	98553.695	87533.125	38622.438	42938.684	8.375359179	7.484072659	2	High	6035	Unique	Used	53459	1	55.955	1	5.8693	-2.8034	1	35	18/30	16.67142	6035	1925.08	MS2	963.043	191	Glioma_grade 2_2.raw
95147555			sPcGLTEQYLHk	Unambiguous	N-Term(iTRAQ4plex); C3(Methylthio); K12(iTRAQ4plex)	HCD	0.454454	0	1	1	0.42921	0.4494	0.4593	0.48088637	0.047265839	93203.445	89024.94	40003.54	42810.88	3.242798009	4.794066422	3	High	4313	Unique	Used	74814	1	45.138	1	4.8443	1.6062	1	25	22/44	65.55296	4313	1709.86	MS2	570.626	144	Glioma_grade 2_5.raw
95147555			yLPGAITSPDEHILTPDSSFSk	Unambiguous	N-Term(iTRAQ4plex); K22(iTRAQ4plex)	HCD	0.30202165	0	1	1	0.29074	0.3139	0.291	0.31419903	0.044229429	71369.94	66104.55	20750.285	20769.984	5.416558337	0.067096369	3	High	5481	Unique	Used	75853	1	54.731	1	5.7122	0.5361	1	20	29/84	5.32044	5481	2663.38	MS2	888.466	86	Glioma_grade 2_5.raw
95147555			vVSNTIEPLTLFHk	Unambiguous	N-Term(iTRAQ4plex); K14(iTRAQ4plex)	HCD	0.58605343	0	1	1	0.54221	0.6142	0.5612	0.63571113	0.074558464	56094.523	49523.555	30415.164	31482.676	8.798448371	2.439002635	2	High	5327	Unique	Used	75714	1	53.508	1	5.9885	-3.2799	1	70	21/26	22.61782	5327	1886.09	MS2	943.55	117	Glioma_grade 2_5.raw
95147555			vVSNTIEPLTLFHk	Unambiguous	N-Term(iTRAQ4plex); K14(iTRAQ4plex)	HCD	0.46526334	0	1	1	0.38861	0.4892	0.4462	0.5617641	0.154758688	54741.43	43484.484	21272.893	24428.023	16.20725637	9.763540926	3	High	5299	Unique	Used	75688	1	53.296	1	6.8949	-3.4489	1	21	27/52	26.69828	5299	1886.09	MS2	629.369	96	Glioma_grade 2_5.raw
95147555			vVSNTIEPLTLFHk	Unambiguous	N-Term(iTRAQ4plex); K14(iTRAQ4plex)	HCD	0.64483446	0	1	1	0.629	0.6133	0.6772	0.66027534	0.045081039	52141.457	53480.18	32796.83	35311.645	1.79247385	5.22179579	2	High	5085	Unique	Used	82144	1	52.819	1	5.7682	1.6389	1	100	20/26	38.66927	5085	1886.1	MS2	943.555	108	Glioma_grade 2_6.raw
95147555			vVSNTIEPLTLFHk	Unambiguous	N-Term(iTRAQ4plex); K14(iTRAQ4plex)	HCD	0.6559307	0	1	1	0.64106	0.6955	0.6195	0.67206645	0.051042544	49699.945	45808.99	31860.559	30786.686	5.761389059	2.424186029	3	High	5053	Unique	Used	82118	1	52.514	1	7.0359	0.0461	1	42	24/52	60.44018	5053	1886.1	MS2	629.371	113	Glioma_grade 2_6.raw
95147555			tLPQEPGkPQk	Unambiguous	N-Term(iTRAQ4plex); K8(iTRAQ4plex); K11(iTRAQ4plex)	HCD	0.53761107	0	1	1	0.53838	0.6054	0.4773	0.53674895	0.097075552	45756.504	40687.887	24634.283	21839.18	8.292159643	8.505655304	3	High	1110	Unique	Used	89034	1	19.571	1	3.5269	-6.8945	1	45	13/40	70.37798	1110	1654.97	MS2	552.329	109	Glioma_grade 2_7rep.raw
95147555			vPSAPGQESPIPDPk	Unambiguous	N-Term(iTRAQ4plex); K15(iTRAQ4plex)	HCD	0.38368666	0	1	1	0.40446	0.4653	0.3127	0.35978913	0.168699315	40766.344	35432.46	16488.25	12748.215	9.899435026	18.09113455	2	High	2748	Unique	Used	58405	1	31.944	1	3.8825	-0.9463	1	74	16/28	0	2748	1806.98	MS2	903.995	146	Glioma_grade 2_3.raw
95147555			sIEEAcLTLQHLNR	Unambiguous	N-Term(iTRAQ4plex); C6(Methylthio)	HCD	0.50080013	0	1	1	0.47285	0.5387	0.4675	0.53264695	0.0754661	36545.625	32077.623	17280.482	17086.047	9.207825641	0.800117504	3	High	6658	Unique	Used	69686	1	61.962	1	5.5973	0.1315	1	24	31/52	13.04427	6658	1816.92	MS2	606.311	139	Glioma_grade 2_4.raw
95147555			nLISPELGVVFFNVPEk	Unambiguous	N-Term(iTRAQ4plex); K17(iTRAQ4plex)	HCD	0.5438559	0	1	1	0.56098	0.5487	0.5389	0.5271093	0.026464657	34399.562	35169.03	19297.365	18537.924	1.564200238	2.838650874	2	High	7846	Unique	Used	63115	1	72.1	1	6.0358	-0.9323	1	100	20/32	27.36013	7846	2190.24	MS2	1095.62	145	Glioma_grade 2_3.raw
95147555			sIEEAcLTLQHLNR	Unambiguous	N-Term(iTRAQ4plex); C6(Methylthio)	HCD	0.5851697	0	1	1	0.55855	0.5736	0.5964	0.61250454	0.040866043	33004.027	32137.893	18434.469	19684.605	1.880353618	4.637991169	3	High	6573	Unique	Used	69609	1	61.261	1	5.6163	0.9377	1	29	30/52	42.68897	6573	1816.92	MS2	606.311	138	Glioma_grade 2_4.raw
95147555			nLISPELGVVFFNVPEk	Unambiguous	N-Term(iTRAQ4plex); K17(iTRAQ4plex)	HCD	0.509394	0	1	1	0.44444	0.4697	0.547	0.57803684	0.123646024	31671.908	29969.771	14076.269	17323.63	3.905125995	14.6257221	2	High	8225	Unique	Used	55476	1	73.02	1	5.6422	-1.9355	1	77	20/32	16.36871	8225	2190.24	MS2	1095.62	149	Glioma_grade 2_2.raw
95147555			gTVPLPTISGHR	Unambiguous	N-Term(iTRAQ4plex)	HCD	0.5673574	0	1	1	0.5666	0.5528	0.5823	0.56810105	0.021288838	26342.217	27001.32	14925.374	15339.479	1.747376447	1.935026439	2	High	1681	Unique	Used	65093	1	23.16	1	2.9799	0.4729	1	100	16/22	0	1681	1378.79	MS2	689.9	185	Glioma_grade 2_4.raw
95147555			iSkPDLkPFTPEVR	Unambiguous	N-Term(iTRAQ4plex); K3(iTRAQ4plex); K7(iTRAQ4plex)	HCD	0.41005105	0	1	1	0.3992	0.4377	0.3848	0.4219439	0.057198349	25576.875	23326.92	10210.401	9842.651	6.506482525	2.593505655	4	High	3169	Unique	Used	86206	1	43.286	1	3.4164	-4.248	1	33	20/78	38.19361	3169	2059.22	MS2	515.56	53	Glioma_grade 2_1rep.raw
95147555			iSkPDLkPFTPEVR	Unambiguous	N-Term(iTRAQ4plex); K3(iTRAQ4plex); K7(iTRAQ4plex)	HCD	0.55194736	0	1	1	0.56731	0.5892	0.516	0.5359905	0.058887715	25117.805	24182.629	14249.586	12961.658	2.682610426	6.693575806	3	High	3178	Unique	Used	86212	1	43.391	1	4.7663	-0.0622	1	100	19/52	41.76515	3178	2059.23	MS2	687.081	57	Glioma_grade 2_1rep.raw
95147555			iSkPDLkPFTPEVR	Unambiguous	N-Term(iTRAQ4plex); K3(iTRAQ4plex); K7(iTRAQ4plex)	HCD	0.48590925	0	1	1	0.44471	0.5348	0.4453	0.5354493	0.106172201	23280.904	19360.238	10353.3	10366.427	13.00307349	0.089597616	3	High	3287	Unique	Used	95671	1	45.497	1	4.2143	2.8721	1	96	20/52	27.91659	3287	2059.23	MS2	687.083	44	Glioma_grade 2_8rep.raw
95147555			yLPGAITSPDEHILTPDSSFSk	Unambiguous	N-Term(iTRAQ4plex); K22(iTRAQ4plex)	HCD	0.3895923	0	1	1	0.36371	0.403	0.3775	0.41827622	0.062991057	23151.408	20892.475	8420.331	8738.825	7.253251683	2.624945739	3	High	5924	Unique	Used	69015	1	56.044	1	5.4438	3.7673	1	68	26/84	37.83429	5924	2663.39	MS2	888.468	99	Glioma_grade 2_4.raw
95147555			aQWGENLQVTLIPTHDTEVTR	Unambiguous	N-Term(iTRAQ4plex)	HCD	0.577703	0	1	1	0.5364	0.5438	0.6111	0.6195817	0.075620918	20862.61	20578.045	11190.611	12749.779	0.971113228	9.210361784	3	High	5627	Unique	Used	61075	1	54.295	1	7.0761	-0.0554	1	22	29/80	1.963759	5627	2552.32	MS2	851.446	90	Glioma_grade 2_3.raw
95147555			gREDVALEQDTYWR	Unambiguous	N-Term(iTRAQ4plex)	HCD	0.49184343	1	1	1	0.42042	0.536	0.4572	0.5828916	0.147780359	19752.629	15494.566	8304.45	9031.652	17.08450969	5.932238579	2	High	3488	Unique	Used	66768	1	36.887	1	5.142	0.3062	1	100	19/26	0	3488	1881.92	MS2	941.465	111	Glioma_grade 2_4.raw
95147555			eSSEkPPEkPAkPER	Unambiguous	N-Term(iTRAQ4plex); K5(iTRAQ4plex); K9(iTRAQ4plex); K12(iTRAQ4plex)	HCD	0.41725644	0	1	1	0.35937	0.4294	0.4071	0.48641476	0.125357341	14857.78	12436.091	5339.446	6049.098	12.54781862	8.812359885	3	High	846	Unique	Used	84546	1	17.226	1	3.9124	3.6277	1	100	20/56	15.81328	846	2285.3	MS2	762.439	45	Glioma_grade 2_1rep.raw

95147555			qLSPESLGTLQFGELNLGk	Unambiguous	N-Term(iTRAQ4plex); K19(iTRAQ4plex)	HCD	0.4963115	0	1	1	0.47346	0.6538	0.3823	0.52787393	0.222842002	13298.2705	9630.01	6296.1377	5083.431	22.62578631	15.07110074	3	High	7430	Unique	Used	54755	1	66.524	1	5.9589	4.3526	1	30	27/72	6.85484	7430	2319.29	MS2	773.768	116	Glioma_grade2_2.raw
95147555			lDMYVLNPVk	Unambiguous	N-Term(iTRAQ4plex); K10(iTRAQ4plex)	HCD	0.59810156	0	1	1	0.58705	0.5645	0.6331	0.60872805	0.049067038	11280.467	11731.203	6622.202	7141.1123	2.770059558	5.331927819	3	High	5593	Unique	Used	61045	1	54.007	1	3.5728	-1.2958	1	100	15/36	36.62449	5593	1479.85	MS2	493.954	242	Glioma_grade2_3.raw
95147555			nEPTTPSWLADIPPWVPk	Unambiguous	N-Term(iTRAQ4plex); K18(iTRAQ4plex)	HCD	0.45091748	0	1	1	0.42047	0.4856	0.4209	0.48608112	0.083021891	10881.927	9422.117	4575.535	4579.9136	10.16784194	0.067634867	2	High	7820	Unique	Used	63091	1	71.892	1	4.2394	-0.836	1	100	19/34	24.38399	7820	2336.25	MS2	1168.63	149	Glioma_grade2_3.raw
95147555			nLISPELGVVFFNVPEk	Unambiguous	N-Term(iTRAQ4plex); K17(iTRAQ4plex)	HCD	0.6453009	0	1	1	0.58123	0.5555	0.7393	0.7065291	0.140710667	4595.7104	4808.8906	2671.177	3397.621	3.205689748	16.92834326	3	High	8190	Unique	Used	55445	1	72.723	1	5.4044	-0.4368	1	40	27/64	9.090455	8190	2190.24	MS2	730.752	129	Glioma_grade2_2.raw
95147555			dRWPEVSPEDTQSLSLSEESPSk	Unambiguous	N-Term(iTRAQ4plex); K23(iTRAQ4plex)	HCD	0.54801106	1	1	1	0.49145	0.5005	0.5946	0.6056215	0.110221803	2599.0725	2551.9243	1277.3029	1545.5004	1.294460596	13.43659127	3	High	4587	Unique	Used	75059	1	47.379	1	6.7983	-4.5721	1	94	23/88	10.02979	4587	2891.4	MS2	964.472	79	Glioma_grade2_5.raw
98986464	NP_006818.3	TMED10	transmembrane emp24 domain-containing protein 10 precursor [Homo sapiens]	4	9	2.054940701	17.01694729	53.763656	22.37	3.09E-04	219	24.96	7.4448	Integral membrane protein	Transport	Transporter activity	Integral to membrane	Zymogen granule, Golgi apparatus, Microsome, Endoplasmic reticulum, Mitochondrion, Plasma membrane																								
98986464			lkPLEVELR	Unambiguous	N-Term(iTRAQ4plex); K2(iTRAQ4plex)	HCD	2.6403646	0	1	1	2.43509	2.7456	2.5471	2.871812	7.392175977	610245.3	541239.8	1486001.8	1554338.9	8.475013179	3.178698152	2	High	2569	Unique	Used	79977	1	30.875	1	3.0747	6.5063	1	4	14/16	5.725253	2569	1384.89	MS2	692.947	38	Glioma_grade2_6.raw
98986464			iTDSAGHILYSk	Unambiguous	N-Term(iTRAQ4plex); K12(iTRAQ4plex)	HCD	1.8850645	0	1	1	1.71577	1.8213	1.9451	2.0647674	8.015698483	246698.94	232405.62	423279.44	479863.6	4.219080485	8.860400063	2	High	2277	Unique	Used	72965	1	29.084	1	5.5015	0.039	1	15	20/22	20.73378	2277	1592.89	MS2	796.948	257	Glioma_grade2_5.raw
98986464			iTDSAGHILYSk	Unambiguous	N-Term(iTRAQ4plex); K12(iTRAQ4plex)	HCD	1.7639648	0	1	1	1.6287	1.8342	1.7016	1.9163053	7.306299473	177010.17	157173.97	288295.75	301193.3	8.394360985	3.09418642	2	High	2352	Unique	Used	73036	1	29.616	1	4.8045	12.76	1	15	20/22	33.95748	2352	1592.91	MS2	796.958	293	Glioma_grade2_5.raw
98986464			iPDQLVILDMk	Unambiguous	N-Term(iTRAQ4plex); K11(iTRAQ4plex)	HCD	1.9874629	0	1	1	1.87072	2.0216	1.9559	2.1136253	5.162163009	169229.78	156599.94	316580.9	330993.56	5.481786934	3.147526733	2	High	6860	Unique	Used	54231	1	62.083	1	4.5601	2.1135	1	21	17/20	12.78404	6860	1572.93	MS2	786.969	151	Glioma_grade2_2.raw
98986464			iPDQLVILDMk	Unambiguous	N-Term(iTRAQ4plex); K11(iTRAQ4plex)	HCD	2.2522733	0	1	1	2.22134	2.2906	2.2151	2.2841713	1.780027853	125199.49	121413.68	278110.62	277329.66	2.170988616	0.198841219	2	High	6613	Unique	Used	61997	1	61.928	1	4.5499	0.8718	1	30	17/20	15.92995	6613	1572.93	MS2	786.968	161	Glioma_grade2_3.raw
98986464			lkPLEVELR	Unambiguous	N-Term(iTRAQ4plex); K2(iTRAQ4plex)	HCD	1.699047	0	1	1	1.69589	1.6629	1.7359	1.7021446	1.759950873	38236.062	38994.25	64844.074	66373.85	1.388366465	1.648730524	3	High	2556	Unique	Used	79965	1	30.771	1	3.3291	6.3203	1	26	19/32	18.67752	2556	1384.89	MS2	462.3	47	Glioma_grade2_6.raw
98986464			iPDQLVILDMk	Unambiguous	N-Term(iTRAQ4plex); K11(iTRAQ4plex)	HCD	2.0549407	0	1	1	1.8674	2.1777	1.9497	2.273651	9.203216422	15744.257	13500.716	29400.768	30695.916	10.84920171	3.047781916	3	High	6619	Unique	Used	62003	1	61.966	1	4.7176	-0.5725	1	55	18/40	12.74315	6619	1572.93	MS2	524.98	158	Glioma_grade2_3.raw
98986464			iPDQLVILDMk	Unambiguous	N-Term(iTRAQ4plex); K11(iTRAQ4plex)	HCD	2.0840633	0	1	1	2.42032	2.1664	1.9921	1.7830905	12.91626217	13782.204	15397.769	33357.297	27455.615	7.829869938	13.72445168	3	High	6866	Unique	Used	54237	1	62.119	1	4.0766	-0.9217	1	27	18/40	8.695889	6866	1572.93	MS2	524.98	167	Glioma_grade2_2.raw
98986464			rLEDLSESIVNDFAYMk	Unambiguous	N-Term(iTRAQ4plex); K17(iTRAQ4plex)	HCD	2.7304187	1	1	1	2.46522	2.9036	2.5834	3.0427792	9.810955032	12712.777	10793.347	31339.79	32841.773	11.54798612	3.309555938	2	High	6975	Unique	Used	83671	1	70.978	1	5.1375	-0.9469	1	80	18/32	0	6975	2318.19	MS2	1159.6	195	Glioma_grade2_6.raw
112799847	NP_001026.2	RYR2	ryanodine receptor 2 [Homo sapiens]	9	11	0.390976101	17.88515111	70.033376	3.6	9.96E-05	4967	564.21	6.0747	Intracellular ligand gated channel	Transport	Intracellular ligand-gated ion channel activity	Sarcoplasmic reticulum	Endoplasmic reticulum																								
112799847			fFDMFLk	Unambiguous	N-Term(iTRAQ4plex); K7(iTRAQ4plex)	HCD	0.36407906	0	1	5	0.32582	0.3488	0.3784	0.40503278	0.094773855	191198.12	178623.5	62295.94	72348.375	4.808588028	10.55840338	2	High	7228	Unique	Used	70196	1	66.781	1	3.0874	-6.787	1	95	12-Dec	46.95766	7228	1235.67	MS2	618.336	181	Glioma_grade2_4.raw
112799847			fFDMFLk	Unambiguous	N-Term(iTRAQ4plex); K7(iTRAQ4plex)	HCD	0.36486584	0	1	5	0.33259	0.3629	0.3666	0.4000801	0.075521243	129738.625	118897.5	43150.305	47568.527	6.166306689	6.887555027	2	High	7253	Unique	Used	70219	1	66.982	1	3.0813	10.501	1	100	12-Dec	37.17725	7253	1235.69	MS2	618.347	231	Glioma_grade2_4.raw
112799847			fFDMFLk	Unambiguous	N-Term(iTRAQ4plex); K7(iTRAQ4plex)	HCD	0.38299835	0	1	5	0.34424	0.3781	0.3875	0.4255621	0.087126692	125003.62	113819.15	43031.61	48437.117	6.622998788	8.357546411	2	High	7129	Unique	Used	62478	1	65.931	1	3.132	-5.3052	1	100	12-Dec	40.54617	7129	1235.67	MS2	618.337	213	Glioma_grade2_3.raw
112799847			lLSLVEk	Unambiguous	N-Term(iTRAQ4plex); K7(iTRAQ4plex)	HCD	0.35064006	0	1	1	0.3297	0.3658	0.3369	0.37387472	0.061307863	90401.85	81469.53	29805.594	30459.4	7.349803142	1.534259362	2	High	4581	Unique	Used	60108	1	46.074	1	2.9464	-6.9375	1	100	12-Dec	48.38832	4581	1089.7	MS2	545.356	162	Glioma_grade2_3.raw
112799847			tVAGGLPGAGLFGPk	Unambiguous	N-Term(iTRAQ4plex); K15(iTRAQ4plex)	HCD	0.479462	0	1	1	0.46153	0.482	0.477	0.49819112	0.031495808	83469.73	79920.195	38523.73	39815.53	3.072282784	2.332012174	2	High	5438	Unique	Used	68571	1	52.164	1	5.5939	-0.6081	1	71	21/28	29.79984	5438	1629.96	MS2	815.482	148	Glioma_grade2_4.raw
112799847			lVFDVPLLNEHAk	Unambiguous	N-Term(iTRAQ4plex); K13(iTRAQ4plex)	HCD	0.399082	0	1	1	0.37227	0.4372	0.3667	0.4305659	0.092988359	71572.44	60948.457	26644.377	26242.328	11.33751822	1.075096565	3	High	5529	Unique	Used	82518	1	56.803	1	6.6063	-0.0391	1	43	27/48	28.72826	5529	1783.04	MS2	595.017	121	Glioma_grade2_6.raw
112799847			hGLPGIGLSTSLRPR	Unambiguous	N-Term(iTRAQ4plex)	HCD	0.483749	0	1	1	0.49762	0.4664	0.5023	0.47074962	0.037843054	33663.684	35920.664	16751.719	16909.639	4.587025413	0.663468793	4	High	2664	Unique	Used	90394	1	32.452	1	4.848	-1.6351	1	32	17/84	35.15816	2664	1705	MS2	427.005	121	Glioma_grade2_7rep.raw
112799847			yVDEAHQYILEFDGGSR	Unambiguous	N-Term(iTRAQ4plex)	HCD	0.5529688	0	1	1	0.48243	0.554	0.5521	0.63397163	0.111487736	19570.516	17043.104	9441.345	10804.844	9.762215067	9.524156759	3	High	6340	Unique	Used	61743	1	59.781	1	4.7364	0.4324	1	68	21/64	14.81486	6340	2143.02	MS2	715.012	62	Glioma_grade2_3.raw
112799847			aALSLPTNVEDVcPNIPSLEk	Unambiguous	N-Term(iTRAQ4plex); C13(Methylthio); K21(iTRAQ4plex)	HCD	0.46520203	0	1	1	0.50644	0.634	0.3304	0.4135819	0.276460927	4110.585	3283.661	2081.7556	1358.0627	15.8156374	29.7532086	2	High	8012	Unique	Used	55285	1	71.237	1	3.4011	-5.0365	1	100	14/40	17.22056	8012	2544.32	MS2	1272.66	118	Glioma_grade2_2.raw
112799847			yYcLPGGWGNFGAASEEELHLSR	Unambiguous	N-Term(iTRAQ4plex); C3(Methylthio)	HCD	0.3909761	0	1	1	0.39141	0.3484	0.4388	0.39058736	0.094233741	2808.9382	3155.989	1099.4545	1232.6895	8.228163257	8.079378631	3	High	7500	Unique	Used	62802	1	69.194	1	6.2946	1.5399	1	100	32/88	39.93833	7500	2746.26	MS2	916.09	78	Glioma_grade2_3.raw
112799847			dLLGPTVSLTQAAFTPIPVDTSQIVLPPHLER	Unambiguous	N-Term(iTRAQ4plex)	HCD	0.3013452	0	1	1	0.3191	0.3736	0.2396	0.28055564	0.188087906	1842.9248	1574.1906	588.0833	441.64804	11.12188223	20.11114187	3	High	8119	Unique	Used	70958	1	74.949	1	9.1303	-5.174	1	92	36/124	42.74957	8119	3569.95	MS2	1190.66	37	Glioma_grade2_4.raw
115334682	NP_079524.2	SRCIN1	SRC kinase signaling inhibitor 1 [Homo sapiens]	3	3	0.428654045	37.15021705	18.56287	4.9	3.57E-05	1183	127.03	9.3784	Unclassified	Cell communication,Signal transduction	Molecular function unknown	Cytoplasm	Plasma membrane																								
115334682			dVkPDEDLASk	Unambiguous	N-Term(iTRAQ4plex); K3(iTRAQ4plex); K11(iTRAQ4plex)	HCD	0.58457375	0	1	1	0.52197	0.6038	0.568	0.65698296	0.097089848	41585.46	35952.258	21706.492	23620.021	10.27441982	5.970321749	3	High	1068	Unique	Used	78584	1	19.674	1	3.8803	-8.4717	1	49	19/40	66.6464	1068	1648.9	MS2	550.304	223	Glioma_grade2_6.raw
115334682			aHPGPAPTPDHkPPk	Unambiguous	N-Term(iTRAQ4plex); K12(iTRAQ4plex); K15(iTRAQ4plex)	HCD	0.42865404	0	1	1	0.43662	0.4305	0.4268	0.42080227	0.015472172	22872.896	23197.953	9986.705	9761.751	0.997811041	1.610925926	5	High	821	Unique	Used	93927	1	16.977	1	5.1606	0.3242	1	100	30/112	22.49684	821	1979.12	MS2	396.63	159	Glioma_grade2_8rep.raw
115334682			eMSGSNETSSPVSEkPSASR	Unambiguous	N-Term(iTRAQ4plex); K15(iTRAQ4plex)	HCD	0.2674144	0	1	1	0.27885	0.3365	0.2102	0.25361192	0.19550742	5262.2393	4360.958	1467.3904	1105.9911	13.24512216	19.86086367	3	High	1190	Unique	Used	56948	1	20.491	1	5.3173	-1.0477	1	100	30/76	0	1190	2355.13	MS2	785.715	69	Glioma_grade2_3.raw
115387104	NP_000687.3	ALDH9A1	4-trimethylaminobutyraldehyde dehydrogenase [Homo sapiens]	5	9	2.119815588	16.15510832	41.942762	11.97	3.16E-04	518	56.255	6.5659	Enzyme: Dehydrogenase	Metabolism,Energy pathways	Catalytic activity	Cytoplasm	-																								
115387104			eVNLAVQNAk	Unambiguous	N-Term(iTRAQ4plex); K10(iTRAQ4plex)	HCD	1.9668393	0	1	1	1.8817	1.9525	1.9807	2.0551836	3.647258869	165600	159595.19	311609.28	327997.4	2.611380489	3.623524002	2	High	1480	Unique	Used	49166	1	23.198	1	4.7494	-1.516	1	29	17/18	23.25414	1480	1373.8	MS2	687.402	260	Glioma_grade2_2.raw
115387104			mGPLINRPHLER	Unambiguous	N-Term(iTRAQ4plex)	HCD	2.201962	0	1	1	1.80873	2.2205	2.1869	2.68471	16.12182252	134839.75	109837.02	243888.38	294880.56	14.45139228	13.3849276	4	High	1707	Unique	Used	89568	1	24.258	1	3.8214	-1.8142	1	5	25/66	3.982007	1707	1576.88	MS2	394.976	230	Glioma_grade2_7rep.raw
115387104			vSFTGSVPTGMk	Unambiguous	N-Term(iTRAQ4plex); K12(iTRAQ4plex)	HCD	2.1973183	0	1	1	2.15741	2.1483	2.2465	2.2370634	2.349420592	123437.44	123959.586	266304.53	277305.47	0.298478105	2.861919123	2	High	3613	Unique	Used	59209	1	38.558	1	4.3508	0.5496	1	25	20/22	2.298303	3613	1498.82	MS2	749.913	245	Glioma_grade2_3.raw
115387104			vSFTGSVPTGmk	Unambiguous	N-Term(iTRAQ4plex); M11(Oxidation); K12(iTRAQ4plex)	HCD	1.811076	0	1	1	1.76355	1.8865	1.7406	1.8619164	3.961143444	120757.64	112889.95	212961.9	210191.66	4.762126544	0.925836705	2	High	2382	Unique	Used	50020	1	29.444	1	3.821	0.9191	1	26	15/22	17.26979	2382	1514.81	MS2	757.911	271	Glioma_grade2_2.raw
115387104			gIkPVTLELGGk	Unambiguous	N-Term(iTRAQ4plex); K3(iTRAQ4plex); K12(iTRAQ4plex)	HCD	2.365357	0	1	1	2.23808	2.4447	2.2927	2.5043921	5.283039391	119244.875	109164.42	266879	273390.5	6.24139056	1.704455205	2	High	2747	Unique	Used	90464	1	33.19	1	4.9005	13.427	1	42	19/22	24.7516	2747	1644.06	MS2	822.536	31	Glioma_grade2_7rep.raw
115387104			gIkPVTLELGGk	Unambiguous	N-Term(iTRAQ4plex); K3(iTRAQ4plex); K12(iTRAQ4plex)	HCD	2.1198156	0	1	1	1.93945	2.0833	2.1538	2.3135517	7.325466053	89628.87	83441.24	173831.1	193045.62	5.05611874	7.406693665	2	High	2735	Unique	Used	90453	1	33.1	1	5.0312	-1.646	1	44	19/22	6.366404	2735	1644.04	MS2	822.523	40	Glioma_grade2_7rep.raw
115387104			mGPLINRPHLER	Unambiguous	N-Term(iTRAQ4plex)	HCD	1.5414895	0	1	1	1.42015	1.5118	1.5694	1.6706545	6.802901875	56190.215	52783.76	79798.805	88183.43	4.42073886	7.058871666	2	High	1702	Unique	Used	89564	1	24.207	1	3.635	-2.2616	1	75	13/22	0	1702	1576.88	MS2	788.945	208	Glioma_grade2_7rep.raw
115387104			vSFTGSVPTGmk	Unambiguous	N-Term(iTRAQ4plex); M11(Oxidation); K12(iTRAQ4plex)	HCD	2.381281	0	1	1	2.5715	2.2016	2.5911	2.2184198	8.958528907	36674.03	42835.164	94307.34	95026.38	10.95868141	0.537081361	2	High	2350	Unique	Used	58032	1	29.032	1	3.8638	-0.9344	1	69	18/22	0	2350	1514.81	MS2	757.909	250	Glioma_grade2_3.raw
115387104			sPLIIFSDcDMNNAVk	Unambiguous	N-Term(iTRAQ4plex); C9(Methylthio); K16(iTRAQ4plex)	HCD	1.5239046	0	1	1	1.85287	1.4049	1.6808	1.27447	16.86063122	2242.3235	2957.2524	4154.728	3768.9297	19.44508871	6.885723852	3	High	7894	Unique	Used	55179	1	70.246	1	3.83	1.2078	1	53	20/60	33.56483	7894	2101.04	MS2	701.018	145	Glioma_grade2_2.raw
116063573	NP_001447.2	FLNA	filamin-A isoform 1 [Homo sapiens]	11	13	2.278694868	37.91944614	121.19275	11.44	2.61E-04	2639	279.84	6.0493	Structural protein,Anchor protein	Cell growth and/or maintenance	Cytoskeletal anchoring activity	Cytoplasm	Cytosol, Cell projection, Mitochondrion, Nucleus, Plasma membrane																								
116063573			lTVSSLQESGLk	Unambiguous	N-Term(iTRAQ4plex); K12(iTRAQ4plex)	HCD	2.2972217	0	1	2	2.14679	2.3516	2.2475	2.4620075	5.888455917	104900.336	95762.34	225199.02	235767.62	6.440200108	3.242372909	2	High	4046	Unique	Used	51586	1	41.431	1	4.3543	2.9083	1	69	18/22	62.27443	4046	1549.91	MS2	775.458	223	Glioma_grade2_2.raw
116063573			vDVGkDQEFTVk	Unambiguous	N-Term(iTRAQ4plex); K5(iTRAQ4plex); K12(iTRAQ4plex)	HCD	1.62269	1	1	2	1.53182	1.672	1.5775	1.7218789	5.32978958	55927.49	51239.18	85670.62	88227.664	6.186878426	2.079495106	3	High	1942	Unique	Used	79392	1	26.207	1	3.4805	3.6652	1	34	19/44	54.13968	1942	1797.02	MS2	599.678	159	Glioma_grade2_6.raw
116063573			dVDIIDHHDNTYTVk	Unambiguous	N-Term(iTRAQ4plex); K15(iTRAQ4plex)	HCD	2.127778	0	1	2	2.01828	2.1039	2.1507	2.2419138	4.382666705	28344.686	27191.771	57207.64	60961.605	2.935851722	4.49263107	3	High	2018	Unique	Used	79463	1	26.766	1	4.8148	6.9286	1	30	25/56	30.97142	2018	2073.06	MS2	691.693	163	Glioma_grade2_6.raw
116063573			aWGPGLEGGVVGk	Unambiguous	N-Term(iTRAQ4plex); K13(iTRAQ4plex)	HCD	3.5072427	0	1	2	3.3828	3.3738	3.6411	3.6313524	4.248912044	28170.486	28245.984	95295.125	102571.12	0.189253769	5.200387165	2	High	3789	Unique	Used	59373	1	39.904	1	4.915	0.3857	1	100	21/24	0	3789	1514.86	MS2	757.932	210	Glioma_grade2_3.raw
116063573			tGVAVNkPAEFTVDAk	Unambiguous	N-Term(iTRAQ4plex); K7(iTRAQ4plex); K16(iTRAQ4plex)	HCD	2.6999223	0	1	2	2.34087	2.7553	2.6529	3.1225395	11.85605989	23502.955	19968.14	55017.27	62351.31	11.49955692	8.837031883	3	High	2294	Unique	Used	79719	1	28.846	1	4.5128	4.1266	1	25	25/60	11.7076	2294	2079.19	MS2	693.735	79	Glioma_grade2_6.raw
116063573			iVGPSGAAVPck	Unambiguous	N-Term(iTRAQ4plex); C11(Methylthio); K12(iTRAQ4plex)	HCD	1.9733667	0	1	2	2.00674	1.9376	2.0104	1.9411386	2.026588884	15932.929	16501.412	31973.322	32031.53	2.478719603	0.128612973	2	High	3382	Unique	Used	58997	1	36.716	1	3.8587	-0.3631	1	100	16/22	0	3382	1432.79	MS2	716.898	301	Glioma_grade2_3.raw
116063573			lVSNHSLHETSSVFVDSLTk	Unambiguous	N-Term(iTRAQ4plex); K20(iTRAQ4plex)	HCD	1.9194177	0	1	2	1.76511	1.7662	2.0726	2.0738215	9.251329982	15258.402	15249.233	26932.709	31624.188	0.042503865	11.33043855	3	High	4208	Unique	Used	91619	1	47.459	1	7.7568	-2.3016	1	47	28/76	26.74338	4208	2488.32	MS2	830.113	139	Glioma_grade2_7rep.raw
116063573			fVPAEMGTHTVSVk	Unambiguous	N-Term(iTRAQ4plex); K14(iTRAQ4plex)	HCD	2.2786949	0	1	2	2.10583	2.0755	2.4849	2.449072	9.571310244	12365.662	12546.457	26039.943	30727.176	1.026338791	11.67709159	3	High	2704	Unique	Used	73368	1	32.149	1	3.9003	12.49	1	56	19/52	52.19736	2704	1790.99	MS2	597.669	220	Glioma_grade2_5.raw
116063573			vHSPSGALEEcYVTEIDQDk	Unambiguous	N-Term(iTRAQ4plex); C11(Methylthio); K20(iTRAQ4plex)	HCD	1.5014136	0	1	2	1.51437	1.5068	1.496	1.4885175	0.761418563	11599.91	11658.416	17566.611	17353.756	0.355743482	0.862025384	3	High	5421	Unique	Used	75798	1	54.27	1	6.9639	1.3631	1	27	25/76	38.68876	5421	2554.21	MS2	852.074	98	Glioma_grade2_5.raw
116063573			tGVAVNkPAEFTVDAk	Unambiguous	N-Term(iTRAQ4plex); K7(iTRAQ4plex); K16(iTRAQ4plex)	HCD	4.661784	0	1	2	4.55727	4.9385	4.4064	4.775044	5.027888136	11447.644	10563.837	52169.99	50442.785	5.678363241	2.380441164	2	High	2270	Unique	Used	79697	1	28.666	1	5.2813	2.4012	1	100	21/30	17.75681	2270	2079.19	MS2	1040.1	130	Glioma_grade2_6.raw
116063573			tFSVWYVPEVTGTHk	Unambiguous	N-Term(iTRAQ4plex); K15(iTRAQ4plex)	HCD	1.6004915	0	1	2	1.56242	1.2768	1.9966	1.6315991	18.31471811	9886.678	12098.602	15447.185	19740.068	14.22830603	17.25355873	3	High	5332	Unique	Used	75718	1	53.556	1	4.5948	1.8869	1	39	22/56	35.6605	5332	2039.09	MS2	680.368	191	Glioma_grade2_5.raw
116063573			vHGPGIQSGTTNkPNk	Unambiguous	N-Term(iTRAQ4plex); K13(iTRAQ4plex); K16(iTRAQ4plex)	HCD	3.7285247	0	1	2	3.31542	3.4944	3.9507	4.1639314	10.54478366	7206.086	6837.015	23891.19	28468.861	3.71673759	12.36401472	4	High	962	Unique	Used	94040	1	18.405	1	5.2692	-0.8341	1	34	20/90	25.35309	962	2067.17	MS2	517.547	140	Glioma_grade2_8rep.raw

116063573			vHGPGIQSGTTNkPNk	Unambiguous	N-Term(iTRAQ4plex); K13(iTRAQ4plex); K16(iTRAQ4plex)	HCD	3.3119924	0	1	2	2.86365	3.1639	3.4461	3.807339	12.12278085	6088.562	5510.831	17435.502	20981.602	7.043773891	13.05393221	3	High	914	Unique	Used	84579	1	18.224	1	3.3631	-6.3786	1	100	19/60	41.96627	914	2067.15	MS2	689.723	124	Glioma_grade 2_1rep.raw
116235472	NP_115667.2	SGIP1	SH3-containing GRB2-like protein 3-interacting protein 1 [Homo sapiens]	4	7	0.384812146	22.73151087	29.172692	9.54	3.37E-04	828	89.054	8.2212	Unclassified	Biological_process unknown	Molecular function unknown	Plasma membrane	-																								
116235472			aTPPPPPPPTYR	Unambiguous	N-Term(iTRAQ4plex)	HCD	0.29154265	0	1	1	0.27903	0.2853	0.2977	0.30433658	0.039485991	413658.25	404628.72	115422.24	123143.31	1.560538571	4.577040528	2	High	1314	Unique	Used	64750	1	20.545	1	3.2021	1.8481	1	8	13/22	0	1314	1434.79	MS2	717.898	239	Glioma_grade 2_4.raw
116235472			aTPPPPPPPTYR	Unambiguous	N-Term(iTRAQ4plex)	HCD	0.37030518	0	1	1	0.35456	0.3835	0.3581	0.38733208	0.045639792	285108	263592.62	101088.8	102097.88	5.545344963	0.702336699	2	High	1228	Unique	Used	72005	1	21.104	1	3.1269	0.4018	1	23	13/22	21.97137	1228	1434.79	MS2	717.897	314	Glioma_grade 2_5.raw
116235472			aTPPPPPPPTYR	Unambiguous	N-Term(iTRAQ4plex)	HCD	0.47064966	0	1	1	0.41832	0.4872	0.4564	0.53160375	0.101315582	115743.9	99375.64	48417.473	52828.465	10.76062885	6.161318501	2	High	1387	Unique	Used	57130	1	22.054	1	3.2632	0.1465	1	43	13/22	20.77269	1387	1434.79	MS2	717.897	296	Glioma_grade 2_3.raw
116235472			nVLSPLNLEEVQk	Unambiguous	N-Term(iTRAQ4plex); K13(iTRAQ4plex)	HCD	0.5610938	0	1	1	0.50277	0.5394	0.5813	0.62367404	0.093020754	33781.02	31484.676	16984.004	19636.174	4.975846467	10.24226257	2	High	5559	Unique	Used	53011	1	52.466	1	4.2005	4.139	1	77	17/24	25.85123	5559	1771.03	MS2	886.018	159	Glioma_grade 2_2.raw
116235472			sTTPPPPPPRPPSRPk	Unambiguous	N-Term(iTRAQ4plex); K16(iTRAQ4plex)	HCD	0.38481215	0	1	1	0.32772	0.428	0.3518	0.45937818	0.158657256	25018.082	19156.695	8198.823	8800.168	18.76467422	5.002827842	4	High	955	Unique	Used	84609	1	18.67	1	3.2427	-5.4109	1	48	19/90	45.62478	955	1997.14	MS2	500.041	93	Glioma_grade 2_1rep.raw
116235472			yNSPELDEEGYSIRPEEPGSTk	Unambiguous	N-Term(iTRAQ4plex); K22(iTRAQ4plex)	HCD	0.3227079	0	1	1	0.3236	0.2896	0.3598	0.3219107	0.088626536	15026.322	16792.951	4862.4995	5405.831	7.851815758	7.483074061	3	High	3932	Unique	Used	67179	1	40.386	1	4.0207	2.9346	1	41	26/84	4.596539	3932	2785.35	MS2	929.121	68	Glioma_grade 2_4.raw
116235472			sTTPPPPPPRPPSRPk	Unambiguous	N-Term(iTRAQ4plex); K16(iTRAQ4plex)	HCD	0.4046279	0	1	1	0.37177	0.3794	0.4293	0.43816113	0.083793913	8547.992	8375.633	3177.8938	3669.8767	1.440308654	10.16051706	4	High	1006	Unique	Used	94078	1	18.801	1	3.4867	6.569	1	100	16/90	62.07414	1006	1997.17	MS2	500.047	65	Glioma_grade 2_8rep.raw
116284394	NP_079005.3	MYH14	myosin-14 isoform 2 [Homo sapiens]	13	15	2.069345236	22.22393054	181.34993	14.34	4.86E-04	1995	227.73	5.605	Structural protein	Cell growth and/or maintenance	Structural molecule activity	-	-																								
116284394			iHEAAVQELR	Unambiguous	N-Term(iTRAQ4plex)	HCD	2.283808	0	1	3	2.1617	2.3135	2.2561	2.4144866	4.629187945	282658	264113.56	611022.56	637698.7	4.796481835	3.021151332	2	High	1286	Unique	Used	64723	1	20.368	1	3.0207	1.0484	1	11	17/18	22.0319	1286	1309.74	MS2	655.372	295	Glioma_grade 2_4.raw
116284394			iHEAAVQELR	Unambiguous	N-Term(iTRAQ4plex)	HCD	2.2097492	0	1	3	2.17009	2.251	2.1699	2.2508883	2.114265251	138508.39	133527.4	300575.66	300555.25	2.589432667	0.004801633	3	High	1285	Unique	Used	64722	1	20.363	1	4.8219	0.8517	1	4	21/36	8.551814	1285	1309.74	MS2	437.25	227	Glioma_grade 2_4.raw
116284394			kLEGELEELk	Unambiguous	N-Term(iTRAQ4plex); K1(iTRAQ4plex); K10(iTRAQ4plex)	HCD	2.0245855	1	1	3	1.91675	2.0431	2.0072	2.1395316	4.546577551	77047.08	72280.984	147680	154647.44	4.513738022	3.259197426	2	High	3231	Unique	Used	90866	1	37.597	1	4.5922	-0.2229	1	90	17/18	17.63449	3231	1619.96	MS2	810.483	171	Glioma_grade 2_7rep.raw
116284394			kQELELVVSELEAR	Unambiguous	N-Term(iTRAQ4plex); K1(iTRAQ4plex)	HCD	2.434231	1	1	3	2.23478	2.4502	2.4197	2.6529093	7.019494563	56660.016	51678.6	126622.58	137098.64	6.502562362	5.61782102	2	High	5763	Unique	Used	82719	1	58.857	1	5.0137	-0.7405	1	55	21/26	17.0149	5763	1931.1	MS2	966.056	107	Glioma_grade 2_6.raw
116284394			lEVTVQALk	Unambiguous	N-Term(iTRAQ4plex); K9(iTRAQ4plex)	HCD	1.9188448	0	1	3	1.86528	1.9454	1.8933	1.9747133	2.577575256	55694.832	53399.934	103886.37	105449.56	2.974914375	1.056046374	2	High	3990	Unique	Used	51534	1	40.992	1	3.8508	1.0509	1	78	16/16	56.57505	3990	1288.81	MS2	644.908	122	Glioma_grade 2_2.raw
116284394			rQEEEAGALEAGEEAR	Unambiguous	N-Term(iTRAQ4plex)	HCD	1.5924172	1	1	3	1.46345	1.4277	1.7613	1.7182304	10.76172434	23114.389	23693.898	33826.75	40711.574	1.750864088	13.06255756	3	High	1247	Unique	Used	64686	1	20.112	1	5.0597	0.9498	1	7	25/60	15.21289	1247	1888.91	MS2	630.309	112	Glioma_grade 2_4.raw
116284394			hGQALGELAEQLEQAR	Unambiguous	N-Term(iTRAQ4plex)	HCD	2.0693452	0	1	3	1.87658	2.3047	1.8777	2.306096	11.82394275	21038.965	17130.494	39481.23	39504.562	14.48124454	0.041775147	2	High	4808	Unique	Used	75248	1	49.335	1	4.9695	-1.003	1	79	24/30	21.85066	4808	1893.99	MS2	947.498	215	Glioma_grade 2_5.raw
116284394			lQQHIQELEAHLEAEEGAR	Unambiguous	N-Term(iTRAQ4plex)	HCD	2.0868993	0	1	3	1.93794	2.1428	2.0364	2.251605	6.464571369	20657.129	18682.629	40032.21	42065.902	7.098072843	3.503216746	3	High	5136	Unique	Used	82189	1	53.245	1	7.2033	-0.8609	1	35	29/72	17.28747	5136	2345.19	MS2	782.403	175	Glioma_grade 2_6.raw
116284394			vLGFSHEEIISMLR	Unambiguous	N-Term(iTRAQ4plex)	HCD	2.2534442	0	1	3	2.01802	2.5798	1.9982	2.5544088	14.11965615	20468.389	16011.348	41305.52	40899.53	17.27865481	0.698444395	3	High	6068	Unique	Used	76378	1	59.534	1	4.8759	-3.173	1	16	23/52	10.27297	6068	1774.96	MS2	592.324	192	Glioma_grade 2_5.raw
116284394			lVWVPSELHGFEAAALR	Unambiguous	N-Term(iTRAQ4plex)	HCD	3.3809445	0	1	3	3.16385	3.5026	3.2711	3.6212845	6.175663586	19481.18	17597.05	61635.516	63723.926	7.186325235	2.355991458	3	High	6722	Unique	Used	76948	1	65.172	1	5.4509	-2.5807	1	16	27/64	15.15271	6722	2039.11	MS2	680.376	152	Glioma_grade 2_5.raw
116284394			lQQHIQELEAHLEAEEGAR	Unambiguous	N-Term(iTRAQ4plex)	HCD	1.6577958	0	1	3	1.50836	1.7293	1.5955	1.8291216	8.521703245	15692.948	13688.32	23670.543	25037.602	9.648909996	3.969178827	3	High	4879	Unique	Used	92122	1	54.399	1	8.0339	2.106	1	37	39/72	18.68136	4879	2345.2	MS2	782.405	163	Glioma_grade 2_7rep.raw
116284394			eLSSTEAQLHDAQELLQEETR	Unambiguous	N-Term(iTRAQ4plex)	HCD	2.2062356	0	1	3	1.94323	2.6815	1.8618	2.5691614	18.60174	14584.575	10569.21	28341.168	27154.008	22.57546386	3.025303988	3	High	6663	Unique	Used	54048	1	60.581	1	7.4945	-1.1737	1	14	36/80	10.47477	6663	2571.26	MS2	857.759	87	Glioma_grade 2_2.raw
116284394			kFEEDLLLLEDQNSk	Unambiguous	N-Term(iTRAQ4plex); K1(iTRAQ4plex); K15(iTRAQ4plex)	HCD	1.7406249	1	1	3	1.74217	1.7587	1.7228	1.7390684	0.845486715	13105.697	12982.733	22832.31	22577.861	0.666568883	0.792433102	3	High	4883	Unique	Used	92125	1	54.44	1	5.8914	-1.2684	1	33	22/56	30.83232	4883	2253.23	MS2	751.749	158	Glioma_grade 2_7rep.raw
116284394			aDLLLEPcSHYR	Unambiguous	N-Term(iTRAQ4plex); C8(Methylthio)	HCD	1.6732359	0	1	3	1.74095	1.7468	1.5999	1.6052954	4.879935361	10399.069	10363.9375	18104.227	16637.182	0.239288293	5.971879078	3	High	5494	Unique	Used	68624	1	52.577	1	3.8741	1.103	1	70	19/44	25.81904	5494	1606.79	MS2	536.267	123	Glioma_grade 2_4.raw
116284394			dVEGIVGLEQVSSLGDGPPGGRPR	Unambiguous	N-Term(iTRAQ4plex)	HCD	1.5177039	0	1	2	1.37636	1.6317	1.4216	1.6852692	9.978307238	5302.278	4472.588	7297.8364	7537.5146	12.00383566	2.284786933	3	High	5850	Unique	Used	68947	1	55.445	1	4.1417	-0.6579	1	94	20/92	23.66293	5850	2535.33	MS2	845.78	102	Glioma_grade 2_4.raw
117190254	NP_001070911.1	HNRNPC	heterogeneous nuclear ribonucleoproteins C1/C2 isoform b [Homo sapiens]	5	8	2.500688076	19.49092647	57.915144	20.48	7.32E-05	293	32.318	5.0845	RNA binding protein	Regulation of nucleobase, nucleoside, nucleotide and nucleic acid metabolism	RNA binding	Nucleus	Mitochondrion, Cytoplasm, Nucleolus, Nucleoplasm																								
117190254			vDSLLENLEk	Unambiguous	N-Term(iTRAQ4plex); K10(iTRAQ4plex)	HCD	3.4027162	0	1	5	3.15331	3.4508	3.3587	3.67565	6.352730715	734918.1	671553.5	2317428.2	2468395.5	6.371339222	4.461092103	2	High	5967	Unique	Used	53394	1	55.481	1	4.5538	-2.476	1	3	17/18	16.55758	5967	1447.82	MS2	724.414	270	Glioma_grade 2_2.raw
117190254			lkGDDLQAlk	Unambiguous	N-Term(iTRAQ4plex); K2(iTRAQ4plex); K10(iTRAQ4plex)	HCD	2.4292617	1	1	5	2.30765	2.4004	2.457	2.555761	4.275974926	484846.94	466118.88	1118856.2	1191288.5	2.785113396	4.434126616	2	High	1824	Unique	Used	89673	1	25.185	1	3.6203	-1.7969	1	9	17/18	9.433258	1824	1532.93	MS2	766.971	132	Glioma_grade 2_7rep.raw
117190254			lkGDDLQAlk	Unambiguous	N-Term(iTRAQ4plex); K2(iTRAQ4plex); K10(iTRAQ4plex)	HCD	1.7857617	1	1	5	1.75431	1.8294	1.7439	1.818559	2.444666094	225391.22	216136.53	395406.34	393057.06	2.964277582	0.421374491	3	High	1809	Unique	Used	89659	1	25.078	1	4.1527	-2.5024	1	5	19/36	20.32903	1809	1532.93	MS2	511.649	133	Glioma_grade 2_7rep.raw
117190254			vDSLLENLEk	Unambiguous	N-Term(iTRAQ4plex); K10(iTRAQ4plex)	HCD	2.8791091	0	1	5	2.94692	2.7211	3.0503	2.816496	5.013968454	63374.32	68634.79	186759.05	193309.61	5.635541379	2.437425594	2	High	5773	Unique	Used	61211	1	55.418	1	4.7328	0.3907	1	34	18/18	16.91572	5773	1447.83	MS2	724.416	289	Glioma_grade 2_3.raw
117190254			vFIGNLNTLVVk	Unambiguous	N-Term(iTRAQ4plex); K12(iTRAQ4plex)	HCD	2.46656	0	1	5	2.33987	2.5207	2.4163	2.6030378	4.680475813	59315.273	55059.2	138790.3	143321.19	5.262534551	2.271316949	2	High	6201	Unique	Used	61612	1	58.721	1	5.9455	-1.0567	1	92	21/22	0	6201	1605	MS2	803.002	81	Glioma_grade 2_3.raw
117190254			mIAGQVLDINLAAEPk	Unambiguous	N-Term(iTRAQ4plex); K16(iTRAQ4plex)	HCD	3.02989	0	1	2	2.83183	3.2147	2.8671	3.2547288	7.345968465	49832.477	43897.98	141116.95	142876.02	8.95402243	0.875972617	2	High	6437	Unique	Used	53837	1	58.903	1	6.3457	-2.6504	1	22	25/30	10.01018	6437	1971.11	MS2	986.06	131	Glioma_grade 2_2.raw
117190254			qkVDSLLENLEk	Unambiguous	N-Term(iTRAQ4plex); K2(iTRAQ4plex); K12(iTRAQ4plex)	HCD	2.1951392	1	1	5	1.99421	2.2533	2.1437	2.4221702	8.189266248	30407.85	26911.863	60639.64	65185.113	8.625430887	5.10890696	2	High	4194	Unique	Used	81380	1	44.991	1	5.5791	2.4209	1	100	21/22	16.6604	4194	1848.08	MS2	924.546	111	Glioma_grade 2_6.raw
117190254			mIAGQVLDINLAAEPk	Unambiguous	N-Term(iTRAQ4plex); M1(Oxidation); K16(iTRAQ4plex)	HCD	2.5348163	0	1	2	2.46731	2.5739	2.4973	2.6052363	2.540047094	18912.65	18129.186	46663.434	47230.812	2.991173047	0.854571709	2	High	5735	Unique	Used	53176	1	53.758	1	6.2698	2.1414	1	100	21/30	0	5735	1987.12	MS2	994.063	140	Glioma_grade 2_2.raw
119395742	NP_000201.2	ITGA6	integrin alpha-6 isoform b precursor [Homo sapiens]	3	3	3.196633577	36.99963718	20.2311	3.26	6.25E-05	1073	119.4	6.771	Cell surface receptor	Cell communication,Signal transduction	Receptor activity	Plasma membrane	-																								
119395742			wLLYLVk	Unambiguous	N-Term(iTRAQ4plex); K7(iTRAQ4plex)	HCD	3.1966336	0	1	2	3.14279	3.0786	3.3172	3.2493796	3.337256274	41451.754	42316.48	130274.05	137502.31	1.459869904	3.817477885	2	High	7381	Unique	Used	70333	1	68.078	1	3.1238	-0.1066	1	56	12-Dec	7.499496	7381	1222.78	MS2	611.894	87	Glioma_grade 2_4.raw
119395742			lRPIPITASVEIQEPSSR	Unambiguous	N-Term(iTRAQ4plex)	HCD	1.6020377	0	1	2	1.39113	1.7318	1.4978	1.8646036	13.30729909	13246.537	10640.525	18427.611	19840.361	15.42867647	5.220893885	3	High	4233	Unique	Used	74741	1	44.507	1	5.8153	0.0514	1	26	26/68	41.95906	4233	2137.21	MS2	713.075	114	Glioma_grade 2_5.raw
119395742			tAHIDVHFLk	Unambiguous	N-Term(iTRAQ4plex); K10(iTRAQ4plex)	HCD	3.5165086	0	1	2	3.98741	3.2075	3.9007	3.1377146	12.58160079	12411.107	15428.872	49488.13	48411.4	15.32962432	1.555396812	4	High	2251	Unique	Used	90045	1	28.771	1	3.9283	0.4808	1	22	24/54	11.48393	2251	1468.85	MS2	367.969	149	Glioma_grade 2_7rep.raw
119395750	NP_006112.3	KRT1	keratin, type II cytoskeletal 1 [Homo sapiens]	2	4	0.387304068	19.8503843	27.61392	6.06	9.96E-05	644	65.999	8.1187	Structural protein	Cell growth and/or maintenance	Structural molecule activity	Plasma membrane	Cytoskeleton																								
119395750			sLDLDSIIAEVk	Unambiguous	N-Term(iTRAQ4plex); K12(iTRAQ4plex)	HCD	0.36040568	0	1	1	0.35328	0.3711	0.3502	0.36789373	0.028836684	45593.688	43404.773	16107.208	15968.343	3.478254843	0.612256875	2	High	7713	Unique	Used	55011	1	68.829	1	5.8486	-3.0429	1	84	21/22	27.51613	7713	1590.91	MS2	795.961	220	Glioma_grade 2_2.raw
119395750			tHNLEPYFESFINNLR	Unambiguous	N-Term(iTRAQ4plex)	HCD	0.37545124	0	1	1	0.2835	0.3094	0.436	0.4757939	0.250079795	19953.242	18283.701	5656.834	8699.273	6.174885699	29.97092803	3	High	7128	Unique	Used	77288	1	68.931	1	4.6281	0.3403	1	48	18/60	55.44984	7128	2138.08	MS2	713.365	106	Glioma_grade 2_5.raw
119395750			sLDLDSIIAEVk	Unambiguous	N-Term(iTRAQ4plex); K12(iTRAQ4plex)	HCD	0.3991569	0	1	1	0.41562	0.4279	0.3712	0.3822118	0.067270824	9571.78	9296.906	3978.1792	3553.3872	2.06018871	7.97638334	3	High	7723	Unique	Used	55020	1	68.913	1	4.1996	-6.7728	1	77	18/44	32.57924	7723	1590.91	MS2	530.974	209	Glioma_grade 2_2.raw
119395750			tHNLEPYFESFINNLR	Unambiguous	N-Term(iTRAQ4plex)	HCD	0.54166496	0	1	1	0.4157	0.4764	0.5986	0.68602544	0.222999767	2622.7307	2288.4868	1090.2742	1569.9602	9.62474695	25.500702	3	High	7586	Unique	Used	70511	1	69.909	1	4.5942	1.7962	1	100	27/60	38.19895	7586	2138.08	MS2	713.366	221	Glioma_grade 2_4.raw
120433590	NP_001073331.1	DBI	acyl-CoA-binding protein isoform 3 [Homo sapiens]	3	21	2.935091257	37.91832666	113.8448	50.57	2.11E-04	87	10.038	6.5659	Ligand	Metabolism,Energy pathways	Receptor binding	Mitochondrion	Cytoplasm, Extracellular																								
120433590			wDAWNELk	Unambiguous	N-Term(iTRAQ4plex); K8(iTRAQ4plex)	HCD	3.3175232	0	1	6	2.98682	3.3883	3.2552	3.6926708	8.809575657	1519795.5	1339734.5	4539358.5	4947198.5	8.905124557	6.079896629	2	High	5311	Unique	Used	60787	1	51.753	1	3.5216	2.3728	1	2	14/14	10.41353	5311	1349.71	MS2	675.36	210	Glioma_grade 2_3.raw
120433590			wDAWNELk	Unambiguous	N-Term(iTRAQ4plex); K8(iTRAQ4plex)	HCD	3.4200943	0	1	6	3.21322	3.5807	3.276	3.6506312	6.345068435	1143525.4	1026167.4	3674395.2	3746158.5	7.649436605	1.367669264	2	High	5521	Unique	Used	52974	1	52.217	1	3.6187	-0.6117	1	3	14/14	11.9603	5521	1349.71	MS2	675.358	202	Glioma_grade 2_2.raw
120433590			wDAWNELk	Unambiguous	N-Term(iTRAQ4plex); K8(iTRAQ4plex)	HCD	2.4450495	0	1	6	2.46414	2.3349	2.5613	2.4269645	3.828429181	693436.4	731817.75	1708721.1	1776095.8	3.808403274	2.734210067	2	High	5497	Unique	Used	68626	1	52.617	1	3.7444	0.8353	1	6	14/14	24.85138	5497	1349.71	MS2	675.359	207	Glioma_grade 2_4.raw
120433590			qATVGDINTERPGMLDFTGk	Unambiguous	N-Term(iTRAQ4plex); K20(iTRAQ4plex)	HCD	5.6157894	0	1	6	5.93422	4.9943	6.3542	5.3477936	10.69182267	76899.625	91371.76	456339.38	488637.3	12.16290553	4.833574994	3	High	4376	Unique	Used	74871	1	45.648	1	3.7666	1.9763	1	3	25/76	3.6391	4376	2438.26	MS2	813.426	75	Glioma_grade 2_5.raw
120433590			qATVGDINTERPGmLDFTGk	Unambiguous	N-Term(iTRAQ4plex); M14(Oxidation); K20(iTRAQ4plex)	HCD	4.7160587	0	1	6	3.93267	4.6479	4.7737	5.6419215	14.76245109	63581.75	53797.47	250046.22	303521.1	11.78833994	13.66137375	3	High	3421	Unique	Used	74030	1	37.619	1	3.5349	1.623	1	3	16/76	2.543937	3421	2454.26	MS2	818.757	78	Glioma_grade 2_5.raw
120433590			tkPSDEEMLFIYGHYk	Unambiguous	N-Term(iTRAQ4plex); K2(iTRAQ4plex); K16(iTRAQ4plex)	HCD	4.0479045	0	1	6	3.75344	4.0824	4.0162	4.3681827	6.227070717	55472.44	51001.945	208212.33	222785.8	5.93779871	4.78192305	2	High	4126	Unique	Used	91557	1	46.62	1	5.9462	-0.2884	1	24	20/30	0	4126	2390.24	MS2	1195.62	175	Glioma_grade 2_7rep.raw
120433590			qATVGDINTERPGMLDFTGk	Unambiguous	N-Term(iTRAQ4plex); K20(iTRAQ4plex)	HCD	4.674399	0	1	6	4.47986	4.7034	4.6468	4.878642	3.516014783	41907.28	39915.707	187738.83	194734.45	3.442198397	2.586664533	2	High	4397	Unique	Used	74891	1	45.796	1	5.253	-1.0221	1	17	21/38	0	4397	2438.26	MS2	1219.63	131	Glioma_grade 2_5.raw
120433590			tkPSDEEMLFIYGHYk	Unambiguous	N-Term(iTRAQ4plex); K2(iTRAQ4plex); K16(iTRAQ4plex)	HCD	3.0260177	0	1	6	2.69921	3.2725	2.8227	3.4222372	11.38508196	40537.45	33435.715	109419.16	114424.95	13.57704507	3.162580063	3	High	4102	Unique	Used	91536	1	46.423	1	7.1055	-0.243	1	11	28/60	12.56611	4102	2390.24	MS2	797.419	143	Glioma_grade 2_7rep.raw
120433590			tkPSDEEmLFIYGHYk	Unambiguous	N-Term(iTRAQ4plex); K2(iTRAQ4plex); M8(Oxidation); K16(iTRAQ4plex)	HCD	2.70207	0	1	6	2.71704	3.5467	2.055	2.6825304	22.24982328	30988.994	23739.748	84198.3	63682.598	18.73235458	19.61956169	3	High	3121	Unique	Used	90776	1	36.572	1	6.6602	-2.6431	1	12	26/60	5.510092	3121	2406.23	MS2	802.749	135	Glioma_grade 2_7rep.raw
120433590			qATVGDINTERPGMLDFTGk	Unambiguous	N-Term(iTRAQ4plex); K20(iTRAQ4plex)	HCD	3.9681444	0	1	6	3.45947	4.6529	3.459	4.652294	16.98688802	27401.664	20373.434	94795.28	94783.2	20.80460031	0.009011413	3	High	4850	Unique	Used	68029	1	47.56	1	3.4633	0.9249	1	12	17/76	28.97418	4850	2438.26	MS2	813.425	70	Glioma_grade 2_4.raw
120433590			tkPSDEEMLFIYGHYk	Unambiguous	N-Term(iTRAQ4plex); K2(iTRAQ4plex); K16(iTRAQ4plex)	HCD	2.1020863	0	1	6	1.62859	2.1837	2.0412	2.7369823	21.32718863	18018.557	13438.077	29344.775	36779.78	20.5927212	15.90133182	4	High	3744	Unique	Used	86538	1	50.94	1	4.6517	0.7728	1	9	28/90	16.0133	3744	2390.24	MS2	598.317	122	Glioma_grade 2_1rep.raw
120433590			tkPSDEEmLFIYGHYk	Unambiguous	N-Term(iTRAQ4plex); K2(iTRAQ4plex); M8(Oxidation); K16(iTRAQ4plex)	HCD	3.3038595	0	1	6	3.30763	3.3111	3.2966	3.3000894	0.201441974	16109.901	16093.04	53285.523	53108.473	0.074046202	0.235338948	4	High	3110	Unique	Used	90766	1	36.492	1	5.1304	-1.4819	1	6	27/90	21.53269	3110	2406.23	MS2	602.314	121	Glioma_grade 2_7rep.raw

120433590			tkPSDEEMLFIYGHYk	Unambiguous	N-Term(iTRAQ4plex); K2(iTRAQ4plex); K16(iTRAQ4plex)	HCD	1.7775389	0	1	6	1.55756	1.8585	1.7097	2.040025	11.51564205	14534.454	12180.977	22638.22	24849.498	12.45841436	6.585322415	3	High	3741	Unique	Used	86536	1	50.904	1	6.814	-1.4686	1	45	27/60	23.41248	3741	2390.24	MS2	797.418	134	Glioma_grade 2_1rep.raw
120433590			qATVGDINTERPGMLDFTGk	Unambiguous	N-Term(iTRAQ4plex); K20(iTRAQ4plex)	HCD	2.9350913	0	1	6	2.73717	3.1844	2.7208	3.1653523	8.728018392	13940.333	11982.464	38157.055	37928.72	10.68111938	0.424408444	3	High	4490	Unique	Used	74977	1	46.497	1	3.9899	0.0238	1	25	22/76	21.54744	4490	2438.26	MS2	813.425	85	Glioma_grade 2_5.raw
120433590			tkPSDEEmLFIYGHYk	Unambiguous	N-Term(iTRAQ4plex); K2(iTRAQ4plex); M8(Oxidation); K16(iTRAQ4plex)	HCD	2.674432	0	1	6	2.38451	2.488	2.8531	2.9769325	10.6204521	11277.511	10808.497	26891.361	32176.166	3.003195325	12.65304861	4	High	4069	Unique	Used	91510	1	46.097	1	4.4044	-3.1053	1	28	21/90	42.52838	4069	2406.23	MS2	602.313	145	Glioma_grade 2_7rep.raw
120433590			qATVGDINTERPGmLDFTGk	Unambiguous	N-Term(iTRAQ4plex); M14(Oxidation); K20(iTRAQ4plex)	HCD	4.1463404	0	1	6	4.41165	3.9158	4.406	3.9108496	6.875455089	10582.233	11922.14	46685.094	46625.69	8.420206383	0.090032404	3	High	3959	Unique	Used	67204	1	40.598	1	4.5382	2.2199	1	26	16/76	18.88874	3959	2454.26	MS2	818.758	85	Glioma_grade 2_4.raw
120433590			tkPSDEEMLFIYGHYk	Unambiguous	N-Term(iTRAQ4plex); K2(iTRAQ4plex); K16(iTRAQ4plex)	HCD	1.6491553	0	1	6	1.35602	1.7057	1.6042	2.017878	16.39679204	8399.323	6677.5356	11389.627	13474.453	16.15041407	11.85802654	3	High	3855	Unique	Used	95998	1	53.11	1	6.6968	1.3658	1	71	27/60	28.14941	3855	2390.25	MS2	797.42	148	Glioma_grade 2_8rep.raw
120433590			wDAWNELk	Unambiguous	N-Term(iTRAQ4plex); K8(iTRAQ4plex)	HCD	2.2187617	0	1	6	2.06977	2.2644	2.177	2.3817713	5.951462672	7860.369	7184.586	16269.132	17112.041	6.352305366	3.571034905	3	High	5346	Unique	Used	60820	1	52.011	1	3.2902	-0.0338	1	82	28-Dec	8.449774	5346	1349.71	MS2	450.575	235	Glioma_grade 2_3.raw
120433590			tkPSDEEmLFIYGHYk	Unambiguous	N-Term(iTRAQ4plex); K2(iTRAQ4plex); M8(Oxidation); K16(iTRAQ4plex)	HCD	1.764153	0	1	6	1.58083	1.8766	1.6695	1.9817667	10.37000445	6398.708	5390.322	10115.292	10682.36	12.09661149	3.855989399	3	High	3245	Unique	Used	86247	1	44.342	1	6.6163	-0.8929	1	100	24/60	20.81694	3245	2406.24	MS2	802.75	127	Glioma_grade 2_1rep.raw
120433590			tkPSDEEmLFIYGHYk	Unambiguous	N-Term(iTRAQ4plex); K2(iTRAQ4plex); M8(Oxidation); K16(iTRAQ4plex)	HCD	1.7763971	0	1	6	1.64249	1.72	1.8303	1.9166222	6.788509557	3651.2827	3486.7551	5997.1973	6682.7925	3.259679618	7.646520584	3	High	3369	Unique	Used	95715	1	46.642	1	6.1456	0.7812	1	100	23/60	26.03437	3369	2406.24	MS2	802.751	148	Glioma_grade 2_8rep.raw
120433590			tkPSDEEmLFIYGHYk	Unambiguous	N-Term(iTRAQ4plex); K2(iTRAQ4plex); M8(Oxidation); K16(iTRAQ4plex)	HCD	1.5710903	0	1	6	1.52139	1.4484	1.6999	1.618406	7.002430638	2218.7183	2330.49	3375.5376	3771.6792	3.474649733	7.838419332	4	High	3355	Unique	Used	95707	1	46.453	1	5.4174	-2.1921	1	89	25/90	23.20459	3355	2406.23	MS2	602.314	129	Glioma_grade 2_8rep.raw
122937327	NP_001073935.1	BTBD17	BTB/POZ domain-containing protein 17 precursor [Homo sapiens]	2	2	2.957950115	36.2850825	25.229961	13.18	6.99E-05	478	52.438	8.7778	Unclassified	Biological_process unknown	Molecular function unknown	-	-																								
122937327			aDVGGEAAGTSINHSQAVLQR	Unambiguous	N-Term(iTRAQ4plex)	HCD	2.199016	0	1	1	2.52673	2.2701	2.1199	1.9045846	11.86041298	39238.76	43674.57	99145.836	83181.92	7.565951895	12.38230921	3	High	1814	Unique	Used	49482	1	25.497	1	8.1061	0.4159	1	7	41/80	11.49814	1814	2225.14	MS2	742.385	102	Glioma_grade 2_2.raw
122937327			sTSFQTQLGPSGHDAGR	Unambiguous	N-Term(iTRAQ4plex)	HCD	3.716884	0	1	1	3.56144	3.4185	4.0278	3.866091	7.480222458	12765.755	13299.574	45464.438	51417.363	2.896315138	8.689668425	3	High	1385	Unique	Used	57128	1	22.042	1	6.5837	-0.5537	1	27	31/64	7.272804	1385	1889.92	MS2	630.645	79	Glioma_grade 2_3.raw
149363689	NP_001092281.1	SEPT8	septin-8 isoform a [Homo sapiens]	3	6	0.478566051	19.46176197	65.923856	21.95	7.24E-05	483	55.721	6.2778	GTPase	Regulation of exocytosis,Cell communication,Signal transduction,Vesicle-mediated transport	GTPase activity	Cytoplasm	Cytoplasmic vesicle, Cell surface																								
149363689			aAVEALQSQALHATSQQPLR	Unambiguous	N-Term(iTRAQ4plex)	HCD	0.43638983	0	1	4	0.43296	0.5168	0.369	0.44048244	0.137538047	259343.36	217283.77	112285.55	95709.69	12.47961747	11.27035697	3	High	4596	Unique	Used	52103	1	45.503	1	7.1132	2.5527	1	2	38/76	14.8116	4596	2263.23	MS2	755.083	96	Glioma_grade 2_2.raw
149363689			aAVEALQSQALHATSQQPLR	Unambiguous	N-Term(iTRAQ4plex)	HCD	0.39059523	0	1	4	0.48944	0.4131	0.364	0.30717546	0.196406766	127047.83	150534.33	62181.816	46240.45	11.96580026	20.79323448	3	High	4460	Unique	Used	59997	1	45.117	1	6.4983	-2.5443	1	7	33/76	32.81871	4460	2263.22	MS2	755.079	105	Glioma_grade 2_3.raw
149363689			sDIGAHQPGMSLSSSk	Unambiguous	N-Term(iTRAQ4plex); K16(iTRAQ4plex)	HCD	0.5408486	0	1	1	0.5334	0.5032	0.5808	0.54787195	0.059509815	63014.137	66795.7	33611.977	36595.492	4.119824664	6.00979844	3	High	1265	Unique	Used	72040	1	21.363	1	4.3467	1.8347	1	7	23/60	13.2854	1265	1889.97	MS2	630.66	166	Glioma_grade 2_5.raw
149363689			aAVEALQSQALHATSQQPLR	Unambiguous	N-Term(iTRAQ4plex)	HCD	0.4012671	0	1	4	0.42795	0.4071	0.3951	0.37587988	0.05434768	27829.514	29253.617	11909.737	10995.847	3.528162772	5.642447853	2	High	4492	Unique	Used	60026	1	45.376	1	7.2891	-1.2979	1	61	28/38	17.62219	4492	2263.22	MS2	1132.12	152	Glioma_grade 2_3.raw
149363689			aAVEALQSQALHATSQQPLR	Unambiguous	N-Term(iTRAQ4plex)	HCD	0.5207423	0	1	4	0.44969	0.4846	0.5543	0.5973068	0.127904462	20325.615	18862.967	9140.175	11266.978	5.278314583	14.73872248	2	High	4634	Unique	Used	52139	1	45.77	1	7.2303	-1.6215	1	62	29/38	0	4634	2263.22	MS2	1132.12	174	Glioma_grade 2_2.raw
149363689			iHVcLYFITPTGHSLk	Unambiguous	N-Term(iTRAQ4plex); C4(Methylthio); K16(iTRAQ4plex)	HCD	0.6344552	0	1	4	0.58224	0.5268	0.7534	0.68170047	0.158991842	6299.714	6962.2964	3667.9507	4746.2007	7.065542765	18.12275179	4	High	4505	Unique	Used	96365	1	61.907	1	6.01	0.038	1	91	28/90	17.67354	4505	2163.17	MS2	541.548	219	Glioma_grade 2_8rep.raw
150010589	NP_003632.3	IFITM1	interferon-induced transmembrane protein 1 [Homo sapiens]	2	2	2.395404339	1.120223743	10.906013	27.2	2.65E-05	125	13.929	7.9282	Unclassified	Cell communication,Signal transduction	Molecular function unknown	Plasma membrane	-																								
150010589			mVGDVTGAQAYASTAk	Unambiguous	N-Term(iTRAQ4plex); K16(iTRAQ4plex)	HCD	2.37643	0	1	3	2.16285	2.3185	2.4305	2.6053824	7.835742294	20856.79	19456.44	45110.105	50691.465	4.912516219	8.239149952	2	High	3194	Unique	Used	50782	1	35.278	1	5.2631	1.9841	1	100	22/30	33.00496	3194	1857.97	MS2	929.486	218	Glioma_grade 2_2.raw
150010589			eEHEVAVLGAPPSTILPR	Unambiguous	N-Term(iTRAQ4plex)	HCD	2.414379	0	1	1	2.58681	2.4171	2.4114	2.2532597	5.636003387	4272.992	4572.951	11053.413	10304.047	4.795487445	4.962029944	2	High	4567	Unique	Used	60095	1	45.971	1	5.6429	-1.9435	1	100	24/34	0	4567	2059.13	MS2	1030.07	143	Glioma_grade 2_3.raw
151101292	NP_110382.3	TMX1	thioredoxin-related transmembrane protein 1 precursor [Homo sapiens]	4	6	2.531194925	17.41789885	37.486524	15.36	5.21E-05	280	31.771	4.9829	Enzyme: Oxidoreductase	Metabolism,Energy pathways	Oxidoreductase activity	Endoplasmic reticulum	Mitochondrion, Microsome, Plasma membrane																								
151101292			lLSESAQPLk	Unambiguous	N-Term(iTRAQ4plex); K10(iTRAQ4plex)	HCD	2.9991326	0	1	1	2.79908	3.07	2.9345	3.2185493	5.988687763	237755.66	216776.11	665496.5	697704.6	6.527500628	3.3413362	2	High	2458	Unique	Used	50092	1	29.967	1	3.7412	0.5252	1	12	17/18	23.13166	2458	1373.83	MS2	687.416	230	Glioma_grade 2_2.raw
151101292			dFINFISDk	Unambiguous	N-Term(iTRAQ4plex); K9(iTRAQ4plex)	HCD	2.8638687	0	1	1	2.68517	3.0257	2.7202	3.0652287	6.926790139	184438.95	163678.88	495250.6	501713.16	8.433688257	0.916727404	2	High	5979	Unique	Used	61404	1	57.013	1	4.0404	0.3541	1	24	15/16	51.35744	5979	1386.75	MS2	693.879	221	Glioma_grade 2_3.raw
151101292			dFINFISDk	Unambiguous	N-Term(iTRAQ4plex); K9(iTRAQ4plex)	HCD	2.9503274	0	1	1	2.92171	2.9158	2.9849	2.9788847	1.240244606	111286.35	111510.17	325146.72	332175.94	0.142071016	1.512319423	2	High	6149	Unique	Used	53567	1	56.775	1	4.0868	-1.2304	1	24	16/16	35.24456	6149	1386.75	MS2	693.878	226	Glioma_grade 2_2.raw
151101292			sLGPSLATDk	Unambiguous	N-Term(iTRAQ4plex); K10(iTRAQ4plex)	HCD	2.1894443	0	1	1	2.23048	2.0015	2.3989	2.1526194	7.533143362	67713.2	75461	151033.12	162438.81	7.652945739	5.145622285	2	High	1622	Unique	Used	57353	1	23.664	1	3.542	0.691	1	49	17/18	33.83461	1622	1276.74	MS2	638.872	285	Glioma_grade 2_3.raw
151101292			lLSESAQPLkk	Unambiguous	N-Term(iTRAQ4plex); K10(iTRAQ4plex); K11(iTRAQ4plex)	HCD	2.0353558	1	1	1	1.89504	1.9878	2.0807	2.1825378	6.05785288	37648.008	35891.027	71344.58	78333.53	3.378812843	6.603415741	2	High	1841	Unique	Used	89689	1	25.304	1	4.7517	-1.0519	1	100	17/20	0	1841	1646.02	MS2	823.513	82	Glioma_grade 2_7rep.raw
151101292			lLSESAQPLk	Unambiguous	N-Term(iTRAQ4plex); K10(iTRAQ4plex)	HCD	2.1985211	0	1	1	2.22407	2.2254	2.1717	2.1729555	1.376929075	27529.059	27512.777	61226.6	59784.047	0.041834043	1.685866548	2	High	2428	Unique	Used	58104	1	29.634	1	3.8398	0.7917	1	93	16/18	40.66703	2428	1373.83	MS2	687.416	252	Glioma_grade 2_3.raw
153946395	NP_002151.2	TNC	tenascin precursor [Homo sapiens]	12	17	2.8111508	39.76031731	180.05611	15.36	2.65E-05	2201	240.7	4.894	Adhesion molecule	Cell communication,Signal transduction	Cell adhesion molecule activity	Extracellular	Plasma membrane																								
153946395			vFAILENk	Unambiguous	N-Term(iTRAQ4plex); K8(iTRAQ4plex)	HCD	3.505688	0	1	1	3.52387	3.3634	3.6548	3.4883301	3.416395514	220157.78	230661.08	775808.25	804622	3.294873092	2.578335617	2	High	5211	Unique	Used	68360	1	50.421	1	3.4129	0.589	1	16	13/14	36.00337	5211	1221.75	MS2	611.376	240	Glioma_grade 2_4.raw
153946395			lDAPSQIEVk	Unambiguous	N-Term(iTRAQ4plex); K10(iTRAQ4plex)	HCD	2.164269	0	1	1	2.23853	2.2334	2.095	2.0901785	3.826974817	139204.98	139525.61	311614.56	291633.44	0.162680133	4.684237809	2	High	2549	Unique	Used	50179	1	30.574	1	4.0067	0.1522	1	19	16/18	16.02283	2549	1387.8	MS2	694.406	231	Glioma_grade 2_2.raw
153946395			tPVLSAEASTAk	Unambiguous	N-Term(iTRAQ4plex); K12(iTRAQ4plex)	HCD	2.0467381	0	1	1	1.94233	2.0882	2.0082	2.1589801	4.600627662	115486.62	107421.73	224313.66	231921.36	5.116666476	2.358195239	2	High	1524	Unique	Used	49208	1	23.487	1	3.9373	0.696	1	21	17/22	23.11241	1524	1462.84	MS2	731.922	284	Glioma_grade 2_2.raw
153946395			vFAILENk	Unambiguous	N-Term(iTRAQ4plex); K8(iTRAQ4plex)	HCD	2.8111508	0	1	1	2.46852	2.965	2.6831	3.2226958	11.6034306	105817.38	88098.82	261211.97	283915.7	12.92198788	5.889982228	2	High	5182	Unique	Used	52654	1	49.758	1	3.2489	-2.9081	1	33	13/14	36.42253	5182	1221.74	MS2	611.374	239	Glioma_grade 2_2.raw
153946395			aATHYTITIR	Unambiguous	N-Term(iTRAQ4plex)	HCD	1.9020628	0	1	1	1.87119	2.0245	1.7889	1.9354638	5.233118732	99747.81	92195.86	186647.17	178441.75	5.564168963	3.178463003	2	High	1421	Unique	Used	64852	1	21.247	1	3.1316	0.8597	1	32	18-Dec	13.22767	1421	1290.73	MS2	645.869	359	Glioma_grade 2_4.raw
153946395			vATYLPAPEGLk	Unambiguous	N-Term(iTRAQ4plex); K12(iTRAQ4plex)	HCD	3.341157	0	1	1	3.10663	3.282	3.3971	3.588922	6.055531911	89930.04	85123.97	279379.62	305503.28	3.882692762	6.3165523	2	High	4664	Unique	Used	52166	1	46.012	1	3.6687	-0.6148	1	20	16/22	24.50518	4664	1546.91	MS2	773.957	202	Glioma_grade 2_2.raw
153946395			vFAILENk	Unambiguous	N-Term(iTRAQ4plex); K8(iTRAQ4plex)	HCD	3.4536176	0	1	1	3.4403	3.4665	3.4409	3.467036	0.437400327	46499.4	46148.535	159971.89	159998.64	0.535573774	0.01182303	2	High	5038	Unique	Used	60530	1	49.695	1	3.4174	1.8878	1	54	13/14	38.14734	5038	1221.75	MS2	611.377	239	Glioma_grade 2_3.raw
153946395			aATHYTITIR	Unambiguous	N-Term(iTRAQ4plex)	HCD	3.6216478	0	1	1	4.10225	3.4677	3.8037	3.2153814	10.62161684	42807.77	50640.395	175608.17	162828.19	11.85363512	5.340330762	3	High	1392	Unique	Used	64824	1	21.061	1	3.3538	0.7547	1	5	16/36	14.02776	1392	1290.73	MS2	430.915	294	Glioma_grade 2_4.raw
153946395			yAPISGGDHAEVDVPk	Unambiguous	N-Term(iTRAQ4plex); K16(iTRAQ4plex)	HCD	5.198805	0	1	1	5.85769	4.483	6.134	4.6945434	15.58295481	32725.99	42760.82	191698.67	200742.52	18.79983097	3.25907057	3	High	2162	Unique	Used	72856	1	28.277	1	4.9368	0.596	1	10	22/60	7.11291	2162	1943.01	MS2	648.342	173	Glioma_grade 2_5.raw
153946395			gLEPGQEYNVLLTAEk	Unambiguous	N-Term(iTRAQ4plex); K16(iTRAQ4plex)	HCD	2.0765357	0	1	1	2.04121	2.1524	2.0046	2.113786	3.234056294	30084.408	28530.727	61408.566	60307.85	3.748582583	1.278913352	2	High	5673	Unique	Used	53119	1	53.269	1	5.9508	-1.3418	1	84	21/30	44.35963	5673	2049.11	MS2	1025.06	235	Glioma_grade 2_2.raw
153946395			lEELENLVSSLR	Unambiguous	N-Term(iTRAQ4plex)	HCD	5.165381	0	1	1	4.66063	5.1547	5.1751	5.723636	8.387296855	29146.576	26353.158	135841.39	150835.88	7.118033432	7.396962835	2	High	5924	Unique	Used	88092	1	75.571	1	4.4063	-1.101	1	41	19/22	21.16835	5924	1545.86	MS2	773.433	244	Glioma_grade 2_1rep.raw
153946395			aGTPYTVTLHGEVR	Unambiguous	N-Term(iTRAQ4plex)	HCD	2.7462115	0	1	1	2.66293	2.7474	2.745	2.8321393	2.515281055	16980.725	16458.445	45218.42	46612.605	2.208833112	2.147068853	2	High	1762	Unique	Used	57484	1	24.696	1	4.7909	0.5825	1	69	19/26	11.25527	1762	1644.88	MS2	822.945	190	Glioma_grade 2_3.raw
153946395			aGTPYTVTLHGEVR	Unambiguous	N-Term(iTRAQ4plex)	HCD	1.928816	0	1	1	1.89148	1.9262	1.9313	1.9668388	1.598590438	11048.035	10848.642	20897.129	21337.531	1.287794878	1.47467147	3	High	1865	Unique	Used	65257	1	24.674	1	4.066	0.0549	1	54	24/52	39.45308	1865	1644.88	MS2	548.966	217	Glioma_grade 2_4.raw
153946395			lIPGVEYLVSIIAmk	Unambiguous	N-Term(iTRAQ4plex); M14(Oxidation); K15(iTRAQ4plex)	HCD	5.3833575	0	1	1	5.22354	5.5549	5.2221	5.553316	3.549738844	10493.188	9867.28	54811.562	54796.13	4.347481514	0.019911142	2	High	8025	Unique	Used	78033	1	77.275	1	4.6866	-3.5634	1	100	17/28	57.26211	8025	1950.15	MS2	975.58	81	Glioma_grade 2_5.raw
153946395			vTEYLVVYTPTHEGGLEMQFR	Unambiguous	N-Term(iTRAQ4plex)	HCD	2.3769808	0	1	1	2.14311	2.0877	2.674	2.6048007	12.81555622	5514.2744	5660.675	11817.711	14744.929	1.852730663	15.5847137	3	High	6583	Unique	Used	61969	1	61.699	1	6.884	0.761	1	79	23/80	29.79998	6583	2613.32	MS2	871.777	121	Glioma_grade 2_3.raw
153946395			lIPGVEYLVSIIAmk	Unambiguous	N-Term(iTRAQ4plex); M14(Oxidation); K15(iTRAQ4plex)	HCD	5.173627	0	1	1	6.61479	4.7948	5.6963	4.12899	20.3808353	3776.86	5210.483	24983.127	21514.033	22.5589375	10.55126761	3	High	8403	Unique	Used	71213	1	77.236	1	5.7216	-2.694	1	39	25/56	26.61237	8403	1950.15	MS2	650.723	71	Glioma_grade 2_4.raw
153946395			wQPAIATVDSYVISYTGEk	Unambiguous	N-Term(iTRAQ4plex); K19(iTRAQ4plex)	HCD	1.9783303	0	1	1	1.80442	2.3202	1.7124	2.2019508	14.75696546	3351.0413	2606.0908	6046.691	5738.4834	17.68500484	3.698471937	3	High	8114	Unique	Used	55377	1	72.079	1	5.9757	0.8513	1	59	25/72	12.46434	8114	2416.27	MS2	806.094	156	Glioma_grade 2_2.raw
155722998	NP_071341.2	SLC4A10	sodium-driven chloride bicarbonate exchanger isoform 2 [Homo sapiens]	4	7	0.400102913	20.28729348	64.851737	10.39	3.12E-04	1088	122.56	6.3413	Membrane transport protein	Transport	Auxiliary transport protein activity	Plasma membrane	-																								
155722998			fLFILLGPLGk	Unambiguous	N-Term(iTRAQ4plex); K11(iTRAQ4plex)	HCD	0.34774852	0	1	3	0.31964	0.358	0.3386	0.3792346	0.073362251	602148.7	537640.75	192468.11	203891.98	8.00393597	4.07603901	2	High	7547	Unique	Used	84115	1	76.804	1	4.3229	1.7413	1	16	16/20	39.94135	7547	1505.97	MS2	753.49	29	Glioma_grade 2_6.raw
155722998			sIATLMTDEVFHDVAYk	Unambiguous	N-Term(iTRAQ4plex); K17(iTRAQ4plex)	HCD	0.39561078	0	1	3	0.41604	0.3697	0.4248	0.37746236	0.069233476	41079.285	46233.938	17090.479	17451.572	8.348999076	1.478379549	3	High	6795	Unique	Used	77009	1	65.851	1	7.5945	-0.1276	1	24	33/64	17.69406	6795	2228.15	MS2	743.389	143	Glioma_grade 2_5.raw
155722998			fLFILLGPLGk	Unambiguous	N-Term(iTRAQ4plex); K11(iTRAQ4plex)	HCD	0.4001029	0	1	3	0.36233	0.4135	0.3884	0.44320405	0.086058669	33271.684	29156.928	12055.401	12922.469	9.321276823	4.909222945	3	High	7550	Unique	Used	84118	1	76.821	1	3.8637	-0.0509	1	39	21/40	22.57984	7550	1505.97	MS2	502.661	29	Glioma_grade 2_6.raw
155722998			tLFIGVHVPLGGR	Unambiguous	N-Term(iTRAQ4plex)	HCD	0.31926146	0	1	3	0.35078	0.3678	0.273	0.28621426	0.146641763	24768.14	23622.559	8688.172	6761.113	3.347949545	17.64012363	3	High	4918	Unique	Used	75349	1	50.181	1	4.3006	0.9443	1	41	24/48	7.779632	4918	1509.9	MS2	503.973	84	Glioma_grade 2_5.raw
155722998			kIPPGAEASNILVGELEFLDR	Unambiguous	N-Term(iTRAQ4plex); K1(iTRAQ4plex)	HCD	0.55582213	1	1	3	0.6042	0.5869	0.5238	0.50882876	0.083964349	16204.091	16682.145	9790.541	8488.3545	2.055785437	10.07484183	3	High	6943	Unique	Used	83647	1	70.636	1	6.2298	0.7281	1	46	27/80	34.5898	6943	2556.43	MS2	852.815	99	Glioma_grade 2_6.raw
155722998			sIATLMTDEVFHDVAYk	Unambiguous	N-Term(iTRAQ4plex); K17(iTRAQ4plex)	HCD	0.51740164	0	1	3	0.40798	0.469	0.5595	0.64319414	0.198300237	14028.722	12203.492	5723.3755	7849.215	9.840057802	22.15045869	3	High	7262	Unique	Used	70227	1	67.059	1	7.2414	1.9269	1	71	33/64	20.20023	7262	2228.16	MS2	743.39	139	Glioma_grade 2_4.raw

155722998			tLFIGVHVPLGGR	Unambiguous	N-Term(iTRAQ4plex)	HCD	0.4521907	0	1	3	0.35933	0.3994	0.4997	0.5554157	0.198510977	11591.518	10427.783	4165.169	5791.754	7.474214645	23.10290606	3	High	5007	Unique	Used	75428	1	50.91	1	4.5173	-1.1779	1	72	23/48	21.16293	5007	1509.9	MS2	503.972	114	Glioma_grade 2_5.raw
156104878	NP_055720.3	GLS	glutaminase kidney isoform, mitochondrial isoform 1 [Homo sapiens]	6	9	0.424461573	33.24569928	54.906857	15.1	3.40E-04	669	73.414	7.7671	Enzyme: Deaminase	Metabolism,Energy pathways	Deaminase activity	Mitochondrion	Golgi apparatus																								
156104878			fDYVMQFLNk	Unambiguous	N-Term(iTRAQ4plex); K10(iTRAQ4plex)	HCD	0.21193303	0	1	2	0.19831	0.2138	0.2102	0.22661877	0.054894829	219578.73	203697.66	43544.54	46161.71	5.306042366	4.12595255	2	High	7876	Unique	Used	55162	1	70.106	1	4.6727	-0.7656	1	57	18/18	34.75886	7876	1592.84	MS2	796.922	186	Glioma_grade 2_2.raw
156104878			fDYVMQFLNk	Unambiguous	N-Term(iTRAQ4plex); K10(iTRAQ4plex)	HCD	0.20695256	0	1	2	0.19397	0.1989	0.2148	0.22026515	0.060599002	191050.78	186307.58	37058.21	41037.066	1.777593524	7.205240068	2	High	7539	Unique	Used	62837	1	69.527	1	4.697	1.6868	1	71	17/18	35.95977	7539	1592.84	MS2	796.924	182	Glioma_grade 2_3.raw
156104878			yAIAVNDLGTEYVHR	Unambiguous	N-Term(iTRAQ4plex)	HCD	0.54181427	0	1	2	0.48018	0.6017	0.4941	0.61903924	0.130711828	121928.016	97309.87	58547.58	60238.625	15.88015493	2.013279887	3	High	4823	Unique	Used	52317	1	47.134	1	6.136	-2.3472	1	7	26/56	16.14239	4823	1864.96	MS2	622.326	190	Glioma_grade 2_2.raw
156104878			gSTHPQPGVSPPAAPAAPGPk	Unambiguous	N-Term(iTRAQ4plex); K21(iTRAQ4plex)	HCD	0.42446157	0	1	2	0.42678	0.4907	0.3669	0.42179695	0.118753281	79788.03	69397.664	34051.87	29271.723	9.849601608	10.67556087	3	High	1314	Unique	Used	89215	1	21.186	1	6.9813	-3.9484	1	15	28/80	13.12204	1314	2209.19	MS2	737.068	125	Glioma_grade 2_7rep.raw
156104878			tALHVAAAEGHVEVVk	Unambiguous	N-Term(iTRAQ4plex); K16(iTRAQ4plex)	HCD	0.36157084	0	1	1	0.32218	0.3798	0.3461	0.40799826	0.103436438	61818.297	52442.547	19916.924	21396.467	11.604424	5.064676915	3	High	1594	Unique	Used	79070	1	23.597	1	6.9063	-7.202	1	18	39/60	17.82853	1594	1919.08	MS2	640.365	137	Glioma_grade 2_6.raw
156104878			fVIPDFmSFTSHIDELYESAk	Unambiguous	N-Term(iTRAQ4plex); M7(Oxidation); K21(iTRAQ4plex)	HCD	0.26937822	0	1	2	0.26488	0.271	0.2678	0.273977	0.014635893	53090.16	51887.242	14062.713	14215.912	1.620523006	0.766147942	3	High	7456	Unique	Used	84035	1	76.062	1	6.1776	3.2823	1	25	26/80	20.5822	7456	2780.38	MS2	927.466	118	Glioma_grade 2_6.raw
156104878			lTLQTTSDGVMLDkDLFk	Unambiguous	N-Term(iTRAQ4plex); K14(iTRAQ4plex); K18(iTRAQ4plex)	HCD	0.48883668	1	1	2	0.44422	0.4521	0.5249	0.5342486	0.096654093	35037.664	34423.406	15564.464	18390.656	1.250617064	11.77094664	3	High	5570	Unique	Used	82553	1	57.169	1	6.2554	-0.8696	1	76	22/68	35.34507	5570	2457.36	MS2	819.792	120	Glioma_grade 2_6.raw
156104878			fDYVMQFLNk	Unambiguous	N-Term(iTRAQ4plex); K10(iTRAQ4plex)	HCD	0.48587283	0	1	2	0.43991	0.4742	0.4967	0.5354194	0.082388577	22006.867	20416.725	9680.961	10931.511	5.300825028	8.579974156	3	High	7867	Unique	Used	55154	1	70.03	1	3.6421	3.1728	1	100	17/36	42.39172	7867	1592.84	MS2	531.619	209	Glioma_grade 2_2.raw
156104878			tALHVAAAEGHVEVVk	Unambiguous	N-Term(iTRAQ4plex); K16(iTRAQ4plex)	HCD	0.47827122	0	1	1	0.44962	0.5037	0.4556	0.51036876	0.065953808	17708.62	15807.017	7962.1567	8067.4077	8.023934478	0.92858039	2	High	1598	Unique	Used	79074	1	23.622	1	5.7704	-10.662	1	100	23/30	0	1598	1919.07	MS2	960.041	197	Glioma_grade 2_6.raw
156564401	NP_006169.2	NSF	vesicle-fusing ATPase [Homo sapiens]	25	61	0.477741838	18.17531305	317.63959	39.78	9.95E-05	744	82.542	6.9468	ATPase	Metabolism,Energy pathways	ATPase activity	Cytoplasm	Cytosol, Plasma membrane, Golgi apparatus																								
156564401			mEIGLPDEk	Unambiguous	N-Term(iTRAQ4plex); K9(iTRAQ4plex)	HCD	0.45459792	0	1	2	0.42191	0.4626	0.4473	0.49044195	0.062947654	4279516.5	3903076.5	1805557.2	1914232.4	6.506086193	4.131683731	2	High	3527	Unique	Used	51098	1	37.636	1	3.1633	-19.984	1	3	15/16	18.06011	3527	1319.69	MS2	660.346	143	Glioma_grade 2_2.raw
156564401			mGIGGLDk	Unambiguous	N-Term(iTRAQ4plex); K8(iTRAQ4plex)	HCD	0.46917388	0	1	2	0.42514	0.4652	0.4728	0.5173574	0.080396348	3546860	3241337	1507915	1676929.6	6.365088848	7.505004783	2	High	1908	Unique	Used	57622	1	25.716	1	2.9978	-1.4215	1	4	14/14	27.37661	1908	1078.62	MS2	539.811	353	Glioma_grade 2_3.raw
156564401			mIGFSETAk	Unambiguous	N-Term(iTRAQ4plex); K9(iTRAQ4plex)	HCD	0.4723752	0	1	1	0.44625	0.4846	0.4611	0.5007415	0.05120468	1891187.2	1741493.2	843950.3	872037.94	5.827633089	2.314813149	2	High	2372	Unique	Used	50010	1	29.387	1	3.1809	-0.9472	1	6	16/16	26.10704	2372	1271.69	MS2	636.348	180	Glioma_grade 2_2.raw
156564401			vEVDMEk	Unambiguous	N-Term(iTRAQ4plex); K7(iTRAQ4plex)	HCD	0.45353103	0	1	3	0.43645	0.445	0.4619	0.47094336	0.034539684	1876945	1840744.5	819201.06	866886.4	1.377071379	3.999629684	2	High	1470	Unique	Used	49156	1	23.14	1	2.9881	-6.166	1	7	12-Dec	52.56169	1470	1137.6	MS2	569.303	228	Glioma_grade 2_2.raw
156564401			dFQSGQHVIVR	Unambiguous	N-Term(iTRAQ4plex)	HCD	0.46332404	0	1	2	0.38708	0.4561	0.4694	0.5531701	0.146109413	1747243.9	1482688.9	676326.3	820179.2	11.58343818	13.59425155	2	High	1263	Unique	Used	64701	1	20.221	1	4.9147	1.8916	1	2	19/20	6.787251	1263	1429.77	MS2	715.388	207	Glioma_grade 2_4.raw
156564401			mEIGLPDEk	Unambiguous	N-Term(iTRAQ4plex); K9(iTRAQ4plex)	HCD	0.466231	0	1	2	0.40191	0.456	0.4752	0.5392226	0.12107408	1542094.8	1359012.4	619775.75	732810.2	8.924786134	11.81846168	2	High	3582	Unique	Used	51148	1	38.069	1	3.8536	-3.6117	1	8	16/16	40.73522	3582	1319.71	MS2	660.357	191	Glioma_grade 2_2.raw
156564401			mIGFSETAk	Unambiguous	N-Term(iTRAQ4plex); K9(iTRAQ4plex)	HCD	0.45771623	0	1	1	0.44591	0.4394	0.4763	0.46935263	0.038961502	1253176.5	1271675.2	558801.5	596864.2	1.036144516	4.657816405	2	High	2321	Unique	Used	58004	1	28.845	1	3.0305	-6.7066	1	15	16/16	36.27795	2321	1271.68	MS2	636.345	186	Glioma_grade 2_3.raw
156564401			kLFADAEEEQR	Unambiguous	N-Term(iTRAQ4plex); K1(iTRAQ4plex)	HCD	0.5042721	1	1	2	0.48892	0.5136	0.4954	0.52039474	0.029398453	978609.44	931636.44	478465.03	484818.7	3.477555134	0.93279332	2	High	1296	Unique	Used	78788	1	21.496	1	4.3201	-4.2328	1	11	19/20	16.81117	1296	1623.85	MS2	812.429	182	Glioma_grade 2_6.raw
156564401			qSIINPDWNFEk	Unambiguous	N-Term(iTRAQ4plex); K12(iTRAQ4plex)	HCD	0.44923288	0	1	2	0.39575	0.4671	0.4341	0.5123651	0.109485931	958485.7	812034.06	379317.78	416057.94	11.69791494	6.532564579	2	High	5586	Unique	Used	53036	1	52.663	1	5.0309	0.0455	1	7	17/22	10.17441	5586	1778.93	MS2	889.97	206	Glioma_grade 2_2.raw
156564401			lFGLLVk	Unambiguous	N-Term(iTRAQ4plex); K7(iTRAQ4plex)	HCD	0.29334915	0	1	2	0.28583	0.2864	0.3003	0.30087852	0.028586017	887414.5	885811.44	253652.69	266521.62	0.12784999	3.498714756	2	High	6159	Unique	Used	69232	1	57.902	1	3.0359	-5.2035	1	18	12-Dec	51.86715	6159	1077.72	MS2	539.365	120	Glioma_grade 2_4.raw
156564401			lFGLLVk	Unambiguous	N-Term(iTRAQ4plex); K7(iTRAQ4plex)	HCD	0.26335564	0	1	2	0.23955	0.2661	0.2609	0.28979036	0.078108442	792228.6	713309.75	189781.73	206710.28	7.413169382	6.038100238	2	High	6231	Unique	Used	69298	1	58.485	1	2.9612	9.068	1	19	12-Dec	57.89453	6231	1077.74	MS2	539.372	88	Glioma_grade 2_4.raw
156564401			iAEESNFPFlk	Unambiguous	N-Term(iTRAQ4plex); K11(iTRAQ4plex)	HCD	0.4171691	0	1	1	0.38774	0.3984	0.4354	0.44740373	0.068691149	738023.5	718248.5	286164.62	321347.06	1.920388032	8.190045631	2	High	5587	Unique	Used	61039	1	53.97	1	4.6841	1.6375	1	10	18/20	33.71851	5587	1582.87	MS2	791.941	238	Glioma_grade 2_3.raw
156564401			mGIGGLDk	Unambiguous	N-Term(iTRAQ4plex); K8(iTRAQ4plex)	HCD	0.47774184	0	1	2	0.48253	0.4555	0.5013	0.47321907	0.039842438	700753.4	742275.1	338136.34	351258.75	4.069257903	2.691909249	2	High	2091	Unique	Used	65467	1	26.357	1	3.299	-2.4401	1	16	14/14	39.80382	2091	1078.61	MS2	539.811	356	Glioma_grade 2_4.raw
156564401			lQILHIHTAR	Unambiguous	N-Term(iTRAQ4plex)	HCD	0.47852492	0	1	2	0.42196	0.4724	0.484	0.5418573	0.102537843	663280.4	592442.2	279875.44	321019.12	7.977903971	9.68322134	2	High	1780	Unique	Used	89634	1	24.83	1	4.2992	-1.1608	1	13	15/18	3.229111	1780	1345.82	MS2	673.412	237	Glioma_grade 2_7rep.raw
156564401			dFQSGQHVIVR	Unambiguous	N-Term(iTRAQ4plex)	HCD	0.41159794	0	1	2	0.41499	0.4043	0.419	0.40829515	0.016024479	658837.44	676187.3	273409.44	276084	1.837899073	0.68834289	3	High	1241	Unique	Used	64680	1	20.078	1	5.2863	0.879	1	2	25/40	9.206581	1241	1429.77	MS2	477.261	216	Glioma_grade 2_4.raw
156564401			kIFDDAYk	Unambiguous	N-Term(iTRAQ4plex); K1(iTRAQ4plex); K8(iTRAQ4plex)	HCD	0.64920795	1	1	1	0.59891	0.6461	0.6521	0.7034726	0.065789822	515355.53	477684.88	308651.5	336038.22	5.36477102	6.007645174	2	High	1642	Unique	Used	89511	1	23.72	1	3.3722	-9.0036	1	41	14/14	50.08717	1642	1431.81	MS2	716.408	212	Glioma_grade 2_7rep.raw
156564401			gILLYGPPGcGk	Unambiguous	N-Term(iTRAQ4plex); C10(Methylthio); K12(iTRAQ4plex)	HCD	0.3935228	0	1	2	0.36927	0.4119	0.3771	0.42057708	0.064115446	384308.7	344566.3	141912.23	144916.7	7.711094643	1.481357624	2	High	5706	Unique	Used	68814	1	54.314	1	3.9402	0.5995	1	11	16/22	8.003162	5706	1508.82	MS2	754.915	199	Glioma_grade 2_4.raw
156564401			iAEESNFPFlk	Unambiguous	N-Term(iTRAQ4plex); K11(iTRAQ4plex)	HCD	0.46267718	0	1	1	0.4175	0.4891	0.4401	0.51559937	0.096174638	359204.16	306619.5	149968.6	158092.81	11.16901423	3.729570661	2	High	5821	Unique	Used	53256	1	54.41	1	5.1191	-0.3676	1	24	19/20	37.23528	5821	1582.87	MS2	791.939	253	Glioma_grade 2_2.raw
156564401			vLDDGELLVQQTk	Unambiguous	N-Term(iTRAQ4plex); K13(iTRAQ4plex)	HCD	0.5610684	0	1	1	0.52586	0.5508	0.5708	0.59794766	0.054409479	297675.97	284172.8	156536.5	169920.47	3.282015385	5.797943874	2	High	5320	Unique	Used	52784	1	50.769	1	5.7976	0.9902	1	43	21/24	27.05441	5320	1745.99	MS2	873.499	193	Glioma_grade 2_2.raw
156564401			lQILHIHTAR	Unambiguous	N-Term(iTRAQ4plex)	HCD	0.5614814	0	1	2	0.52644	0.5053	0.6201	0.59511626	0.097239992	281802.12	293611.3	148351.1	174732.86	2.902383215	11.54790934	3	High	1773	Unique	Used	89628	1	24.772	1	4.5741	-1.3748	1	5	23/36	13.5698	1773	1345.82	MS2	449.277	166	Glioma_grade 2_7rep.raw
156564401			gHQLLSADVDlk	Unambiguous	N-Term(iTRAQ4plex); K12(iTRAQ4plex)	HCD	0.4540849	0	1	2	0.42222	0.5035	0.4127	0.49208593	0.102219709	264118.94	221493.2	111515.46	108993.7	12.41358991	1.617308934	2	High	2145	Unique	Used	72840	1	28.158	1	4.8316	0.4665	1	21	20/22	15.20705	2145	1583.9	MS2	792.454	172	Glioma_grade 2_5.raw
156564401			vFPPEIVEQMGck	Unambiguous	N-Term(iTRAQ4plex); C12(Methylthio); K13(iTRAQ4plex)	HCD	0.39330176	0	1	2	0.35523	0.3815	0.4043	0.43419823	0.085230497	254404.48	236859.8	90371	102844.11	5.0506266	9.129535121	2	High	6876	Unique	Used	69883	1	63.774	1	4.2854	1.9593	1	28	17/24	24.13298	6876	1810.92	MS2	905.963	160	Glioma_grade 2_4.raw
156564401			tPLVSVLLEGPPHSGk	Unambiguous	N-Term(iTRAQ4plex); K16(iTRAQ4plex)	HCD	0.4750969	0	1	1	0.46491	0.4459	0.5055	0.48486614	0.053991239	250653.22	261328.47	116531.586	126709.33	2.948754541	5.917385872	3	High	4931	Unique	Used	82013	1	51.451	1	6.8003	1.5423	1	5	33/60	11.3762	4931	1919.12	MS2	640.379	71	Glioma_grade 2_6.raw
156564401			kFLALLR	Unambiguous	N-Term(iTRAQ4plex); K1(iTRAQ4plex)	HCD	0.4370775	1	1	1	0.39083	0.4572	0.4198	0.49118713	0.099480323	237698.17	203169.61	92899.086	99794.29	11.07605501	5.06052217	3	High	3366	Unique	Used	90980	1	38.804	1	3.2779	-4.2647	1	21	15/24	62.18155	3366	1148.77	MS2	383.595	82	Glioma_grade 2_7rep.raw
156564401			kIFDDAYk	Unambiguous	N-Term(iTRAQ4plex); K1(iTRAQ4plex); K8(iTRAQ4plex)	HCD	0.4138823	1	1	1	0.41417	0.4168	0.411	0.41359127	0.005691809	236097.6	234630.17	97784.9	97040.99	0.440861904	0.539993741	3	High	1641	Unique	Used	89510	1	23.717	1	3.73	-8.1604	1	9	16/28	58.74818	1641	1431.81	MS2	477.941	182	Glioma_grade 2_7rep.raw
156564401			mGIGGLDkEFSDIFR	Unambiguous	N-Term(iTRAQ4plex); K8(iTRAQ4plex)	HCD	0.2516467	1	1	2	0.23584	0.2669	0.2382	0.26953053	0.071573256	222683.27	196765.25	52518.586	53034.246	8.738525384	0.690889436	3	High	6390	Unique	Used	83224	1	64.892	1	3.5605	-1.9726	1	15	25/56	12.50613	6390	1973.04	MS2	658.35	166	Glioma_grade 2_6.raw
156564401			lFGLLVk	Unambiguous	N-Term(iTRAQ4plex); K7(iTRAQ4plex)	HCD	0.33988532	0	1	2	0.27776	0.3513	0.3309	0.41844636	0.168823171	221189.53	174905.4	61438.33	73188.53	16.52524166	12.343222	2	High	6085	Unique	Used	61502	1	57.85	1	2.9377	-8.9414	1	100	12-Dec	67.98281	6085	1077.72	MS2	539.363	204	Glioma_grade 2_3.raw
156564401			iAEESNFPFlk	Unambiguous	N-Term(iTRAQ4plex); K11(iTRAQ4plex)	HCD	0.63630176	0	1	1	0.58594	0.6194	0.6523	0.6895392	0.06969569	219696.38	207821.4	128729.305	143301	3.928201014	7.575438588	2	High	5714	Unique	Used	53156	1	53.602	1	4.7617	-0.9074	1	36	17/20	29.97498	5714	1582.87	MS2	791.939	246	Glioma_grade 2_2.raw
156564401			iAEESNFPFlk	Unambiguous	N-Term(iTRAQ4plex); K11(iTRAQ4plex)	HCD	0.45573565	0	1	1	0.42422	0.4576	0.454	0.48973697	0.058718865	185397.11	171854.52	78648.8	84163.51	5.36095929	4.790164622	2	High	5704	Unique	Used	61147	1	54.876	1	4.6514	3.6426	1	44	18/20	37.88234	5704	1582.88	MS2	791.943	248	Glioma_grade 2_3.raw
156564401			aENSSLNLIGk	Unambiguous	N-Term(iTRAQ4plex); K11(iTRAQ4plex)	HCD	0.49407563	0	1	2	0.51731	0.5267	0.462	0.47041643	0.066006305	182295.5	179045.67	94303.836	84226.02	1.27191531	7.98308159	2	High	2460	Unique	Used	50094	1	29.978	1	4.0754	-1.631	1	12	19/20	46.18696	2460	1433.82	MS2	717.413	206	Glioma_grade 2_2.raw
156564401			qSIINPDWNFEk	Unambiguous	N-Term(iTRAQ4plex); K12(iTRAQ4plex)	HCD	0.57126737	0	1	2	0.6175	0.5335	0.615	0.5313239	0.084332485	154576.66	178924.22	95451.45	95066.71	10.32460531	0.285591949	2	High	5382	Unique	Used	60854	1	52.277	1	5.1077	0.6631	1	59	17/22	0	5382	1778.93	MS2	889.97	211	Glioma_grade 2_3.raw
156564401			kFLALLR	Unambiguous	N-Term(iTRAQ4plex); K1(iTRAQ4plex)	HCD	0.5007601	1	1	1	0.45077	0.5023	0.4993	0.55646366	0.086005541	143855.08	129088.56	64846.188	71833.09	7.65103479	7.229312089	3	High	3383	Unique	Used	90994	1	38.959	1	3.336	8.3272	1	30	15/24	63.19897	3383	1148.79	MS2	383.6	73	Glioma_grade 2_7rep.raw
156564401			tPLVSVLLEGPPHSGk	Unambiguous	N-Term(iTRAQ4plex); K16(iTRAQ4plex)	HCD	0.47814348	0	1	1	0.46118	0.4871	0.4697	0.4960645	0.033279657	134117.19	126978.695	61851.664	62989.625	3.866532191	1.289092649	3	High	4792	Unique	Used	81892	1	50.27	1	7.4242	-3.8961	1	14	33/60	17.91359	4792	1919.11	MS2	640.376	85	Glioma_grade 2_6.raw
156564401			lFGLLVk	Unambiguous	N-Term(iTRAQ4plex); K7(iTRAQ4plex)	HCD	0.32651928	0	1	2	0.29412	0.3716	0.2908	0.3674622	0.134604495	132769.5	105076.49	39049.664	38611.637	16.46604609	0.797647884	2	High	6066	Unique	Used	61485	1	57.693	1	2.9983	4.5374	1	100	12-Dec	63.74196	6066	1077.73	MS2	539.37	99	Glioma_grade 2_3.raw
156564401			gHQLLSADVDlk	Unambiguous	N-Term(iTRAQ4plex); K12(iTRAQ4plex)	HCD	0.56897795	0	1	2	0.58679	0.6503	0.4956	0.54923856	0.113986318	128794.55	116222.875	75575.42	63834.086	7.256232199	11.91077586	3	High	2120	Unique	Used	72817	1	27.969	1	5.9564	0.6121	1	6	20/44	17.69778	2120	1583.9	MS2	528.638	189	Glioma_grade 2_5.raw
156564401			dFQSGQHVIVR	Unambiguous	N-Term(iTRAQ4plex)	HCD	0.5627586	0	1	2	0.61273	0.6207	0.5056	0.5121369	0.110900527	123041.836	121464.445	75391.586	62206.426	0.912356008	13.55152725	3	High	1340	Unique	Used	57085	1	21.725	1	4.7618	1.1351	1	7	24/40	18.07608	1340	1429.77	MS2	477.261	193	Glioma_grade 2_3.raw
156564401			gILLYGPPGcGk	Unambiguous	N-Term(iTRAQ4plex); C10(Methylthio); K12(iTRAQ4plex)	HCD	0.54697466	0	1	2	0.52034	0.5539	0.5405	0.57532156	0.042194431	122135.3	114742.75	63552.312	66013.98	4.413513397	2.686905844	2	High	5199	Unique	Used	75598	1	52.49	1	3.8892	0.5186	1	33	16/22	22.09813	5199	1508.82	MS2	754.914	231	Glioma_grade 2_5.raw
156564401			vFPPEIVEQMGck	Unambiguous	N-Term(iTRAQ4plex); C12(Methylthio); K13(iTRAQ4plex)	HCD	0.46568188	0	1	2	0.45828	0.4739	0.4577	0.47333893	0.01937803	110409.29	106767.02	50598.082	50536.99	2.371781541	0.085427472	2	High	6836	Unique	Used	62206	1	63.639	1	4.5181	0.2741	1	63	16/24	40.33773	6836	1810.91	MS2	905.961	156	Glioma_grade 2_3.raw
156564401			iEVGLVVGNSQVAFEk	Unambiguous	N-Term(iTRAQ4plex); K16(iTRAQ4plex)	HCD	0.6185451	0	1	2	0.60113	0.6444	0.5944	0.63721406	0.040609808	84356.85	78695.375	50709.355	50145.8	4.910411206	0.790229438	2	High	6233	Unique	Used	53647	1	57.382	1	6.4534	-0.7789	1	68	24/30	0	6233	1977.12	MS2	989.066	146	Glioma_grade 2_2.raw
156564401			sIDSNPYDTDk	Unambiguous	N-Term(iTRAQ4plex); K11(iTRAQ4plex)	HCD	0.6229406	0	1	2	0.63926	0.6074	0.6393	0.60743105	0.029534954	81933.55	86234.95	52377.195	52381.79	3.617263767	0.006203106	2	High	1422	Unique	Used	49111	1	22.807	1	3.1467	-1.27	1	29	16/20	29.86743	1422	1542.75	MS2	771.879	128	Glioma_grade 2_2.raw
156564401			gILLYGPPGcGk	Unambiguous	N-Term(iTRAQ4plex); C10(Methylthio); K12(iTRAQ4plex)	HCD	0.45914522	0	1	2	0.43579	0.4761	0.4436	0.48465982	0.052054642	72528.79	66390.52	31607.318	32176.818	6.248825079	1.262687988	2	High	5627	Unique	Used	68746	1	53.61	1	3.8942	5.6965	1	64	16/22	8.891254	5627	1508.83	MS2	754.918	220	Glioma_grade 2_4.raw
156564401			lLDYVPIGPR	Unambiguous	N-Term(iTRAQ4plex)	HCD	0.48719838	0	1	1	0.46315	0.4696	0.5045	0.51158595	0.050025965	70585.586	69611.65	32691.525	35612.344	0.982439839	6.047478574	2	High	4358	Unique	Used	86937	1	58.368	1	3.4139	2.9598	1	78	13/18	49.69186	4358	1286.76	MS2	643.885	221	Glioma_grade 2_1rep.raw
156564401			vFPPEIVEQMGck	Unambiguous	N-Term(iTRAQ4plex); C12(Methylthio); K13(iTRAQ4plex)	HCD	0.5562766	0	1	2	0.50643	0.5491	0.5629	0.610315	0.076829334	70172.71	64725.004	35537.805	39502.645	5.711156609	7.472144025	2	High	7005	Unique	Used	69999	1	64.858	1	4.4444	0.1392	1	93	16/24	22.58368	7005	1810.91	MS2	905.961	182	Glioma_grade 2_4.raw
156564401			kLFADAEEEQR	Unambiguous	N-Term(iTRAQ4plex); K1(iTRAQ4plex)	HCD	0.5074178	1	1	2	0.49149	0.522	0.4937	0.52433467	0.03483609	65273.15	61460.406	32081.04	32225.822	4.25462241	0.318399408	3	High	1286	Unique	Used	78779	1	21.418	1	5.4213	-3.4517	1	21	18/40	28.82896	1286	1623.85	MS2	541.956	180	Glioma_grade 2_6.raw
156564401			vFPPEIVEQmGck	Unambiguous	N-Term(iTRAQ4plex); M10(Oxidation); C12(Methylthio); K13(iTRAQ4plex)	HCD	0.45397592	0	1	2	0.43615	0.4645	0.4441	0.4729593	0.037794822	60053.69	56390.88	26192.44	26670.59	4.448464689	1.279166584	2	High	5356	Unique	Used	68495	1	51.531	1	3.6194	0.7164	1	100	17/24	0	5356	1826.91	MS2	913.959	140	Glioma_grade 2_4.raw
156564401			tPLVSVLLEGPPHSGk	Unambiguous	N-Term(iTRAQ4plex); K16(iTRAQ4plex)	HCD	0.53113574	0	1	1	0.49133	0.522	0.5397	0.5734238	0.06451933	49595.727	46682.734	24367.94	26768.99	4.278832633	6.640205961	2	High	4804	Unique	Used	81903	1	50.359	1	6.2449	-0.2953	1	100	21/30	17.86793	4804	1919.12	MS2	960.064	135	Glioma_grade 2_6.raw
156564401			gSMAGSTGVHDTVVNQLLSk	Unambiguous	N-Term(iTRAQ4plex); K20(iTRAQ4plex)	HCD	0.47138694	0	1	2	0.45104	0.4472	0.4958	0.49156478	0.054805717	48331.703	48747.277	21799.318	23962.443	0.605394069	6.684884159	2	High	4775	Unique	Used	67960	1	46.967	1	7.4853	2.7912	1	32	24/38	2.358141	4775	2289.22	MS2	1145.11	153	Glioma_grade 2_4.raw
156564401			vFPPEIVEQMGck	Unambiguous	N-Term(iTRAQ4plex); C12(Methylthio); K13(iTRAQ4plex)	HCD	0.38056144	0	1	2	0.33624	0.3294	0.4328	0.42397606	0.145474163	46286.92	47250.39	15563.66	20033.035	1.456693956	17.75628535	3	High	6905	Unique	Used	69909	1	64.024	1	4.5868	0.6374	1	14	23/48	24.98194	6905	1810.92	MS2	604.31	122	Glioma_grade 2_4.raw

156564401			gSMAGSTGVHDTVVNQLLSk	Unambiguous	N-Term(iTRAQ4plex); K20(iTRAQ4plex)	HCD	0.3769769	0	1	2	0.37422	0.3747	0.3793	0.37973422	0.007774631	44813.51	44759.41	16770.244	16996.68	0.085415272	0.948350706	3	High	4737	Unique	Used	67925	1	46.675	1	6.1416	-1.4006	1	13	26/76	5.29102	4737	2289.21	MS2	763.741	132	Glioma_grade2_4.raw
156564401			gILLYGPPGcGk	Unambiguous	N-Term(iTRAQ4plex); C10(Methylthio); K12(iTRAQ4plex)	HCD	0.57472163	0	1	2	0.54948	0.5591	0.5901	0.6004089	0.042362025	40897.85	40195.62	22472.363	24133.809	1.224640147	5.041477052	2	High	5546	Unique	Used	61002	1	53.631	1	3.9974	-1.8276	1	73	16/22	39.30077	5546	1508.82	MS2	754.913	271	Glioma_grade2_3.raw
156564401			iAEESNFPFIk	Unambiguous	N-Term(iTRAQ4plex); K11(iTRAQ4plex)	HCD	0.4854736	0	1	1	0.46571	0.4912	0.4801	0.5063206	0.035382584	35103.883	33284.117	16348.133	16852.436	3.763142302	2.148132287	3	High	5615	Unique	Used	61064	1	54.198	1	3.742	-0.1833	1	41	17/40	28.23475	5615	1582.87	MS2	528.295	252	Glioma_grade2_3.raw
156564401			iDGVEQLNNILVIGMTNRPDLIDEALLRPGR	Unambiguous	N-Term(iTRAQ4plex)	HCD	0.6008684	0	1	2	0.63636	0.5226	0.6962	0.5717207	0.124765613	33437.293	40717.66	21278.238	23279.129	13.88443163	6.350660687	3	High	7429	Unique	Used	84011	1	75.852	1	7.4118	-1.7623	1	9	25/120	13.80549	7429	3588.96	MS2	1196.99	22	Glioma_grade2_6.raw
156564401			gSmAGSTGVHDTVVNQLLSk	Unambiguous	N-Term(iTRAQ4plex); M3(Oxidation); K20(iTRAQ4plex)	HCD	0.49111703	0	1	2	0.54987	0.4392	0.5561	0.44418383	0.129309507	29542.152	36982.535	16244.363	16427.043	15.81712147	0.790748135	3	High	3405	Unique	Used	74015	1	37.5	1	6.2514	1.7416	1	18	23/76	15.61068	3405	2305.21	MS2	769.075	145	Glioma_grade2_5.raw
156564401			gILLYGPPGcGk	Unambiguous	N-Term(iTRAQ4plex); C10(Methylthio); K12(iTRAQ4plex)	HCD	0.5830665	0	1	2	0.50205	0.6222	0.5515	0.68347913	0.134921321	22962.068	18526.898	11528.079	12662.749	15.1179414	6.633364111	3	High	5604	Unique	Used	61054	1	54.111	1	3.3178	2.0671	1	48	18/44	29.51313	5604	1508.82	MS2	503.613	253	Glioma_grade2_3.raw
156564401			gSmAGSTGVHDTVVNQLLSk	Unambiguous	N-Term(iTRAQ4plex); M3(Oxidation); K20(iTRAQ4plex)	HCD	0.49546036	0	1	2	0.50768	0.4485	0.5486	0.48466164	0.084366707	22288.191	25229.273	11315.359	12227.661	8.753240814	5.480137473	2	High	3416	Unique	Used	74026	1	37.57	1	6.5491	-2.7536	1	100	22/38	0	3416	2305.2	MS2	1153.1	163	Glioma_grade2_5.raw
156564401			gSmAGSTGVHDTVVNQLLSk	Unambiguous	N-Term(iTRAQ4plex); M3(Oxidation); K20(iTRAQ4plex)	HCD	0.5515367	0	1	2	0.57757	0.52	0.5866	0.52809733	0.061277923	18493.729	20542.975	10681.483	10848.69	7.423965624	1.098302402	2	High	4130	Unique	Used	67365	1	41.871	1	5.1644	-2.7536	1	58	22/38	0	4130	2305.2	MS2	1153.1	153	Glioma_grade2_4.raw
156564401			gSMAGSTGVHDTVVNQLLSk	Unambiguous	N-Term(iTRAQ4plex); K20(iTRAQ4plex)	HCD	0.52365816	0	1	2	0.55158	0.5298	0.5173	0.49684128	0.043767131	18128.236	18873.28	9999.121	9377.024	2.847589621	4.540521422	2	High	4237	Unique	Used	74745	1	44.533	1	7.5776	-1.3681	1	100	25/38	11.94379	4237	2289.21	MS2	1145.11	164	Glioma_grade2_5.raw
156564401			vFPPEIVEQMGck	Unambiguous	N-Term(iTRAQ4plex); C12(Methylthio); K13(iTRAQ4plex)	HCD	0.590932	0	1	2	0.58996	0.5772	0.605	0.5918848	0.019211663	15241.808	15578.398	8992.03	9220.617	1.544474242	1.774979966	3	High	6821	Unique	Used	62192	1	63.526	1	3.8404	-1.7893	1	39	16/48	43.11182	6821	1810.91	MS2	604.309	170	Glioma_grade2_3.raw
156564401			gSMAGSTGVHDTVVNQLLSk	Unambiguous	N-Term(iTRAQ4plex); K20(iTRAQ4plex)	HCD	0.5884262	0	1	2	0.68866	0.5469	0.6407	0.5088175	0.138927121	13386.934	16855.459	9219.0625	8576.353	16.219732	5.107655348	3	High	4214	Unique	Used	74725	1	44.334	1	5.9163	-2.8404	1	53	25/76	17.49826	4214	2289.2	MS2	763.74	138	Glioma_grade2_5.raw
156564401			vFPPEIVEQMGck	Unambiguous	N-Term(iTRAQ4plex); C12(Methylthio); K13(iTRAQ4plex)	HCD	0.5941205	0	1	2	0.63532	0.7194	0.4835	0.54746723	0.172603672	10794.047	9532.375	6857.6816	5218.663	8.77810002	19.19390685	3	High	7029	Unique	Used	70020	1	65.068	1	4.8941	2.1541	1	79	20/48	19.24395	7029	1810.92	MS2	604.311	130	Glioma_grade2_4.raw
156564401			fSNLVLQALLVLLk	Unambiguous	N-Term(iTRAQ4plex); K14(iTRAQ4plex)	HCD	0.39714912	0	1	1	0.34887	0.4043	0.391	0.4531004	0.107542019	10316.026	8901.2705	3598.963	4033.1694	10.41127932	8.045727558	2	High	8101	Unique	Used	78077	1	78.299	1	4.7303	-4.3141	1	100	20/26	12.88041	8101	1859.19	MS2	930.098	20	Glioma_grade2_5.raw
156602653	NP_061979.3	SEPT3	neuronal-specific septin-3 isoform B [Homo sapiens]	6	9	0.404268175	31.61445853	42.84784	18.29	9.96E-05	350	40.075	6.8149	GTPase	Cell communication,Signal transduction	GTPase activity	Plasma membrane	Cytoplasm																								
156602653			yINEQYEk	Unambiguous	N-Term(iTRAQ4plex); K8(iTRAQ4plex)	HCD	0.40426818	0	1	2	0.38373	0.3958	0.4125	0.42545864	0.04536349	1689946.2	1638306.2	648475.06	697031.56	2.194244294	5.103598884	2	High	1534	Unique	Used	49217	1	23.562	1	3.4503	1.9868	1	8	14/14	16.61483	1534	1374.72	MS2	687.862	149	Glioma_grade2_2.raw
156602653			aIGHVIEEGGVk	Unambiguous	N-Term(iTRAQ4plex); K12(iTRAQ4plex)	HCD	0.31942907	0	1	2	0.30813	0.3041	0.3349	0.33058107	0.048700417	686221.06	695237.44	211445.66	229832.33	0.923016282	5.892584418	2	High	1405	Unique	Used	78891	1	22.271	1	5.2754	-5.353	1	14	21/22	11.98476	1405	1496.86	MS2	748.933	216	Glioma_grade2_6.raw
156602653			vVNIIPVIAk	Unambiguous	N-Term(iTRAQ4plex); K10(iTRAQ4plex)	HCD	0.44568256	0	1	2	0.40172	0.408	0.4828	0.49033317	0.106176598	466328.44	459122.84	187334.36	225123.16	1.101112232	12.95683334	2	High	5393	Unique	Used	60864	1	52.376	1	3.6959	0.9362	1	8	14/18	18.25456	5393	1353.91	MS2	677.458	18	Glioma_grade2_3.raw
156602653			vVNIIPVIAk	Unambiguous	N-Term(iTRAQ4plex); K10(iTRAQ4plex)	HCD	0.31087545	0	1	2	0.29927	0.3133	0.3085	0.32303116	0.031832374	389471	371971.25	116555.414	120158.3	3.250198395	2.152494741	2	High	5627	Unique	Used	53075	1	52.943	1	3.6593	-3.3915	1	9	14/18	14.53647	5627	1353.9	MS2	677.455	16	Glioma_grade2_2.raw
156602653			aIGHVIEEGGVk	Unambiguous	N-Term(iTRAQ4plex); K12(iTRAQ4plex)	HCD	0.37011534	0	1	2	0.37494	0.38	0.3604	0.36522177	0.024162302	264642.4	261123.77	99226.04	95368.086	0.946446263	2.80376956	3	High	1420	Unique	Used	78905	1	22.381	1	4.3964	-5.6678	1	5	19/44	19.72447	1420	1496.86	MS2	499.625	222	Glioma_grade2_6.raw
156602653			aIGHVIEEGGVk	Unambiguous	N-Term(iTRAQ4plex); K12(iTRAQ4plex)	HCD	0.31081346	0	1	2	0.29727	0.3289	0.2945	0.32579657	0.058579399	155779.22	140799.34	46308.66	45871.945	7.143048189	0.669998072	2	High	1361	Unique	Used	72131	1	22.025	1	4.8664	-3.7219	1	31	21/22	33.45994	1361	1496.86	MS2	748.935	200	Glioma_grade2_5.raw
156602653			sTLVNTLFk	Unambiguous	N-Term(iTRAQ4plex); K9(iTRAQ4plex)	HCD	0.6332116	0	1	2	0.54866	0.6377	0.6294	0.7314827	0.117479158	144240.14	124107.06	79138.4	90782.164	10.61031186	9.690862941	2	High	4934	Unique	Used	52422	1	47.916	1	3.6447	-7.9734	1	58	15/16	55.249	4934	1310.78	MS2	655.895	211	Glioma_grade2_2.raw
156602653			qESMPFAVVGSDk	Unambiguous	N-Term(iTRAQ4plex); K13(iTRAQ4plex)	HCD	0.5986118	0	1	2	0.58748	0.58	0.6175	0.60960543	0.029707287	90659.46	91832.445	53260.25	55981.562	0.908999934	3.522933451	2	High	3900	Unique	Used	51449	1	40.335	1	5.5908	2.9807	1	100	20/24	34.77274	3900	1682.87	MS2	841.939	182	Glioma_grade2_2.raw
156602653			eVTHNIHYETYR	Unambiguous	N-Term(iTRAQ4plex)	HCD	0.6544245	0	1	2	0.59043	0.7121	0.6066	0.7316087	0.108988032	11200.604	9286.976	6613.141	6794.432	13.20936231	1.91223416	4	High	1248	Unique	Used	84847	1	21.478	1	4.0358	-4.3728	1	68	23/66	37.78438	1248	1705.83	MS2	427.214	146	Glioma_grade2_1rep.raw
156627571	NP_003095.2	SORD	sorbitol dehydrogenase [Homo sapiens]	2	7	2.563909054	31.54794904	39.356998	6.72	9.33E-05	357	38.3	7.9722	Enzyme: Dehydrogenase	Metabolism,Energy pathways	Catalytic activity	Plasma membrane	Cytoplasm																								
156627571			aLEAFETFk	Unambiguous	N-Term(iTRAQ4plex); K9(iTRAQ4plex)	HCD	1.7423412	0	1	1	1.66468	1.8057	1.684	1.8265849	4.738119438	325324.62	299923.1	541559.8	547835	5.745430641	0.814624133	2	High	5506	Unique	Used	52960	1	52.103	1	3.7571	-2.4875	1	18	15/16	34.454	5506	1343.74	MS2	672.374	234	Glioma_grade2_2.raw
156627571			aLEAFETFk	Unambiguous	N-Term(iTRAQ4plex); K9(iTRAQ4plex)	HCD	2.8570673	0	1	1	2.69841	2.9536	2.7689	3.0307295	5.421875786	177253.28	161939.97	478301.72	490796.25	6.384646713	1.823338231	2	High	5288	Unique	Used	60766	1	51.564	1	3.988	0.692	1	21	16/16	32.08126	5288	1343.75	MS2	672.377	229	Glioma_grade2_3.raw
156627571			kPMVLGHEASGTVEk	Unambiguous	N-Term(iTRAQ4plex); K1(iTRAQ4plex); K15(iTRAQ4plex)	HCD	4.42776	0	1	1	4.8674	3.8274	5.1912	4.082055	14.30942855	12080.158	15362.492	58798.934	62710.54	16.91498911	4.552604891	3	High	1445	Unique	Used	85000	1	23.5	1	5.7895	-4.0776	1	33	27/56	35.07599	1445	2015.12	MS2	672.379	149	Glioma_grade2_1rep.raw
156627571			kPMVLGHEASGTVEk	Unambiguous	N-Term(iTRAQ4plex); K1(iTRAQ4plex); K15(iTRAQ4plex)	HCD	2.563909	0	1	1	2.67052	2.4399	2.6997	2.4665043	5.249573236	10879.603	11908.16	29054.22	29371.53	6.383247268	0.768058785	4	High	1440	Unique	Used	84997	1	23.435	1	6.1302	-3.3573	1	38	23/84	41.45149	1440	2015.13	MS2	504.537	150	Glioma_grade2_1rep.raw
156627571			kPMVLGHEASGTVEk	Unambiguous	N-Term(iTRAQ4plex); K1(iTRAQ4plex); K15(iTRAQ4plex)	HCD	2.0841134	0	1	1	1.89965	2.04	2.1251	2.2822084	7.670625176	7756.616	7222.8003	14734.866	16483.936	5.039778504	7.923297363	4	High	1592	Unique	Used	94541	1	24.877	1	6.2516	1.3677	1	75	25/84	41.17192	1592	2015.13	MS2	504.539	140	Glioma_grade2_8rep.raw
156627571			kPmVLGHEASGTVEk	Unambiguous	N-Term(iTRAQ4plex); K1(iTRAQ4plex); M3(Oxidation); K15(iTRAQ4plex)	HCD	2.552681	0	1	1	2.37511	3.0019	2.1972	2.777113	14.19994024	5763.348	4559.942	13688.593	12663.474	16.48576264	5.501417376	3	High	1316	Unique	Used	94329	1	21.928	1	4.2146	1.0424	1	100	22/56	5.206929	1316	2031.13	MS2	677.715	107	Glioma_grade2_8rep.raw
156627571			kPMVLGHEASGTVEk	Unambiguous	N-Term(iTRAQ4plex); K1(iTRAQ4plex); K15(iTRAQ4plex)	HCD	3.2948408	0	1	1	3.00596	3.3343	3.2593	3.6152706	7.590055775	5129.0806	4624.0435	15417.823	16717.168	7.323092673	5.718225722	3	High	1577	Unique	Used	94529	1	24.722	1	5.3678	0.0114	1	100	28/56	26.72822	1577	2015.13	MS2	672.382	142	Glioma_grade2_8rep.raw
157738645	NP_065962.1	PLXNA4	plexin-A4 isoform 1 precursor [Homo sapiens]	6	7	0.378348649	22.2246017	76.027807	9.77	1.07E-04	1894	212.32	6.8589	Unclassified	Biological_process unknown	Molecular function unknown	-	-																								
157738645			iLVPVEVIkPITLk	Unambiguous	N-Term(iTRAQ4plex); K9(iTRAQ4plex); K14(iTRAQ4plex)	HCD	0.3663411	0	1	1	0.35077	0.3713	0.3617	0.3828238	0.03726668	193013.34	182348.39	67703.13	69807.305	4.018128575	2.164019642	3	High	5325	Unique	Used	92464	1	58.909	1	5.7214	-3.6309	1	21	21/52	40.27809	5325	1994.33	MS2	665.448	1	Glioma_grade2_7rep.raw
157738645			iLDAIFk	Unambiguous	N-Term(iTRAQ4plex); K7(iTRAQ4plex)	HCD	0.37834865	0	1	1	0.35651	0.3885	0.369	0.4021431	0.053431887	150916.61	138493.6	53803.72	55694.24	6.070549214	2.441688433	2	High	6204	Unique	Used	61615	1	58.742	1	2.9178	8.6592	1	77	12-Dec	61.42718	6204	1107.71	MS2	554.359	139	Glioma_grade2_3.raw
157738645			vFLLSFIR	Unambiguous	N-Term(iTRAQ4plex)	HCD	0.30172646	0	1	1	0.2809	0.2944	0.3087	0.32355085	0.060843909	74740.05	71312.125	20994.709	23073.1	3.319237133	6.669922573	2	High	8115	Unique	Used	55378	1	72.086	1	2.941	1.0479	1	100	14-Dec	16.29224	8115	1138.71	MS2	569.86	137	Glioma_grade2_2.raw
157738645			vLFPGIEDHPVLR	Unambiguous	N-Term(iTRAQ4plex)	HCD	0.44255787	0	1	2	0.41955	0.4057	0.4807	0.46480948	0.080750689	58516.527	60511.656	24550.469	28126.393	2.370479343	9.600268556	3	High	4926	Unique	Used	75356	1	50.25	1	5.4683	1.5852	1	18	24/48	34.31857	4926	1635.94	MS2	545.984	147	Glioma_grade2_5.raw
157738645			nLIPPVAGGNVk	Unambiguous	N-Term(iTRAQ4plex); K12(iTRAQ4plex)	HCD	0.50563663	0	1	1	0.48475	0.4999	0.5112	0.52717066	0.035445005	54393.574	52747.87	26367.51	27807.13	2.172247106	3.758087047	2	High	2793	Unique	Used	58446	1	32.303	1	3.4305	-4.3404	1	100	13/22	31.93214	2793	1466.89	MS2	733.947	163	Glioma_grade2_3.raw
157738645			vLFPGIEDHPVLR	Unambiguous	N-Term(iTRAQ4plex)	HCD	0.32291028	0	1	2	0.30668	0.3607	0.2908	0.3419996	0.098402824	17568.17	14937.405	5387.7983	5108.5864	11.44561677	3.761916765	3	High	4845	Unique	Used	75282	1	49.618	1	5.0663	-1.5488	1	74	26/48	26.56193	4845	1635.93	MS2	545.982	134	Glioma_grade2_5.raw
157738645			ySEEILGPLDHDDQcGk	Unambiguous	N-Term(iTRAQ4plex); C15(Methylthio); K17(iTRAQ4plex)	HCD	0.5403749	0	1	1	0.48659	0.5692	0.5157	0.60329676	0.096437585	12369.348	10573.728	6018.7666	6379.095	11.06822013	4.110235512	3	High	5428	Unique	Used	68562	1	52.083	1	5.0597	0.3905	1	78	27/64	27.45563	5428	2253.04	MS2	751.685	49	Glioma_grade2_4.raw
167614506	NP_002289.2	LCP1	plastin-2 [Homo sapiens]	3	3	3.259919882	26.77152171	20.017689	4.63	9.95E-05	627	70.244	5.4272	Calcium binding protein	Cell communication,Signal transduction	Calcium ion binding	Cytoplasm	Mitochondrion																								
167614506			iSFDEFIk	Unambiguous	N-Term(iTRAQ4plex); K8(iTRAQ4plex)	HCD	3.25992	0	1	1	3.04113	3.3039	3.2195	3.4976106	5.805075735	80865.03	74434.66	245921.11	260343.45	5.855720939	4.028776739	2	High	6083	Unique	Used	61500	1	57.836	1	3.4365	-0.2372	1	75	14/14	37.92836	6083	1286.72	MS2	643.865	216	Glioma_grade2_3.raw
167614506			lNLAFIANLFNR	Unambiguous	N-Term(iTRAQ4plex)	HCD	4.090966	0	1	1	3.84985	4.0554	4.1247	4.3449554	4.986663375	53965.49	51230.16	207759.11	222592.75	3.677282077	4.87460072	2	High	8711	Unique	Used	55923	1	76.831	1	4.2629	-0.0994	1	24	19/22	24.37433	8711	1549.9	MS2	775.452	216	Glioma_grade2_2.raw
167614506			yAFVNWINk	Unambiguous	N-Term(iTRAQ4plex); K9(iTRAQ4plex)	HCD	2.3585355	0	1	3	2.39963	2.3075	2.4116	2.319015	2.27917739	38022.367	39541.043	91239.78	91696.27	2.76900177	0.352896189	2	High	6218	Unique	Used	69286	1	58.379	1	4.2119	0.7095	1	84	15/16	26.81237	6218	1442.8	MS2	721.906	238	Glioma_grade2_4.raw
167857790	NP_000598.2	ORM1	alpha-1-acid glycoprotein 1 precursor [Homo sapiens]	3	5	2.43031311	20.32406047	29.041213	23.88	3.77E-04	201	23.525	5.1099	Secreted polypeptide	Immune response	Defense/immunity protein activity	Extracellular	-																								
167857790			nWGLSVYADkPETTk	Unambiguous	N-Term(iTRAQ4plex); K10(iTRAQ4plex); K15(iTRAQ4plex)	HCD	2.7192051	0	1	1	2.43009	2.9217	2.5508	3.0668077	10.97286332	131412.83	109300.88	319345.2	335204.8	12.99095909	3.426607809	3	High	3105	Unique	Used	80432	1	35.662	1	4.248	1.3314	1	7	27/56	13.29656	3105	2141.16	MS2	714.393	123	Glioma_grade2_6.raw
167857790			sDVVYTDWk	Unambiguous	N-Term(iTRAQ4plex); K9(iTRAQ4plex)	HCD	2.5180655	0	1	1	2.41902	2.508	2.5277	2.6207588	3.285615012	128158.914	123610.234	310018.56	323952.6	2.555040996	3.108297284	2	High	3210	Unique	Used	50797	1	35.394	1	3.8351	0.6106	1	46	15/16	8.424752	3210	1400.73	MS2	700.869	185	Glioma_grade2_2.raw
167857790			yVGGQEHFAHLLILR	Unambiguous	N-Term(iTRAQ4plex)	HCD	1.6733062	0	1	1	1.63614	1.6661	1.6803	1.7111529	1.862503526	87928.99	86345.66	143864.23	147750.61	1.28484938	1.884736492	3	High	4242	Unique	Used	91645	1	47.805	1	5.4382	-0.1137	1	15	24/56	39.93797	4242	1897.06	MS2	633.024	185	Glioma_grade2_7rep.raw
167857790			yVGGQEHFAHLLILR	Unambiguous	N-Term(iTRAQ4plex)	HCD	1.837908	0	1	1	1.6882	1.9235	1.7628	2.008483	7.927756861	78116.836	68560.66	131876.83	137702.9	9.21373358	3.056352645	2	High	4250	Unique	Used	91652	1	47.872	1	6.4834	-3.131	1	49	23/28	18.35753	4250	1897.05	MS2	949.029	211	Glioma_grade2_7rep.raw
167857790			yVGGQEHFAHLLILR	Unambiguous	N-Term(iTRAQ4plex)	HCD	2.430313	0	1	1	2.70946	2.0734	2.8968	2.2166982	15.84253833	6532.446	8536.565	17699.426	18922.988	18.80848232	4.724915116	4	High	4236	Unique	Used	91641	1	47.734	1	5.566	-1.8582	1	45	28/84	33.5737	4236	1897.05	MS2	475.019	145	Glioma_grade2_7rep.raw
169404009	NP_003135.2	SSR1	translocon-associated protein subunit alpha precursor [Homo sapiens]	2	2	2.047345638	27.92108944	9.4901242	9.09	7.32E-05	286	32.215	4.4878	Membrane transport protein	Transport	Auxiliary transport protein activity	Endoplasmic reticulum	Mitochondrion, Nucleus, Microsome																								
169404009			gEDFPANNIVk	Unambiguous	N-Term(iTRAQ4plex); K11(iTRAQ4plex)	HCD	2.451557	0	1	1	2.37617	2.3603	2.5434	2.5264373	3.944692619	118691.66	119489.74	282031.9	301883.34	0.473863853	4.80791967	2	High	2379	Unique	Used	50017	1	29.427	1	4.4255	-0.7393	1	18	19/20	8.751359	2379	1491.8	MS2	746.405	218	Glioma_grade2_2.raw
169404009			gTEDFIVESLDASFR	Unambiguous	N-Term(iTRAQ4plex)	HCD	1.6431341	0	1	1	1.58775	1.7126	1.5787	1.702874	4.380742233	30769.979	28526.72	48855.02	48577.414	5.350124636	0.402939919	2	High	5586	Unique	Used	87832	1	71.992	1	5.0646	1.9356	1	100	20/28	39.67622	5586	1829.91	MS2	915.457	187	Glioma_grade2_1rep.raw
170016077	NP_065143.2	ATP13A1	probable cation-transporting ATPase 13A1 [Homo sapiens]	3	4	2.058669806	11.97308013	28.16872	7.14	6.25E-05	1204	132.87	8.1333	ATPase	Metabolism,Energy pathways	ATPase activity	Integral to membrane	Nucleus																								
170016077			hADVGVALLANAPER	Unambiguous	N-Term(iTRAQ4plex)	HCD	1.6333549	0	1	1	1.5443	1.6014	1.6642	1.7257077	4.79699919	46312.434	44660.79	71520.09	77071.47	2.56754377	5.28350122	2	High	3315	Unique	Used	66610	1	35.497	1	4.8923	1.2239	1	95	21/28	31.3518	3315	1676.92	MS2	838.965	195	Glioma_grade2_4.raw
170016077			lIPHVQVFAR	Unambiguous	N-Term(iTRAQ4plex)	HCD	2.1473303	0	1	1	1.8997	2.4626	1.9041	2.4683392	14.90098851	23967.826	18489.137	45531.664	45637.465	18.24915336	0.164118283	3	High	3416	Unique	Used	80716	1	38.08	1	3.9377	-3.6056	1	35	20/36	6.985103	3416	1323.8	MS2	441.937	182	Glioma_grade2_6.raw

170016077			lIPHVQVFAR	Unambiguous	N-Term(iTRAQ4plex)	HCD	2.1188717	0	1	1	2.02399	2.1668	2.0741	2.2204487	4.181188586	23310.252	21773.693	47179.73	48347.367	4.819947716	1.728606995	3	High	3387	Unique	Used	80689	1	37.87	1	3.9362	9.2579	1	32	20/36	8.139145	3387	1323.81	MS2	441.943	142	Glioma_grade 2_6.raw
170016077			aLALEYALcLTGDGLAHLQA TDPQQLLR	Unambiguous	N-Term(iTRAQ4plex); C9(Methylthio)	HCD	1.9984679	0	1	1	2.38447	1.6371	2.5248	1.7334522	21.71957433	2609.794	3801.1895	6222.9644	6589.18	26.28126674	4.042313699	3	High	8383	Unique	Used	71195	1	77.075	1	7.3304	-2.6961	1	48	32/108	18.62437	8383	3184.65	MS2	1062.22	61	Glioma_grade 2_4.raw
170016081	NP_008846.2	RP2	protein XRP2 [Homo sapiens]	3	4	2.267562389	14.77032537	21.13844	9.14	1.87E-04	350	39.615	5.1226	Structural protein	Cell growth and/or maintenance	Structural molecule activity	Plasma membrane	Nucleus																								
170016081			dYMFSGLk	Unambiguous	N-Term(iTRAQ4plex); K8(iTRAQ4plex)	HCD	2.1161885	0	1	1	1.97585	2.1287	2.1046	2.267384	5.637146945	128459.914	119237.98	253817.17	270358.28	5.265197828	4.462754236	2	High	4319	Unique	Used	51844	1	43.415	1	3.5264	3.4511	1	55	13/14	64.41541	4319	1248.66	MS2	624.833	234	Glioma_grade 2_2.raw
170016081			aPDFLPLLNk	Unambiguous	N-Term(iTRAQ4plex); K10(iTRAQ4plex)	HCD	2.5531027	0	1	1	2.45372	2.6451	2.4677	2.6602404	4.347441017	45452.992	42163.68	111528.836	112165.53	5.309251693	0.402523008	2	High	6555	Unique	Used	61942	1	61.496	1	3.8772	2.5271	1	61	16/18	43.62175	6555	1415.85	MS2	708.431	149	Glioma_grade 2_3.raw
170016081			dYMFSGLkDETVGR	Unambiguous	N-Term(iTRAQ4plex); K8(iTRAQ4plex)	HCD	1.8160661	1	1	1	1.86585	1.9788	1.6626	1.7632672	7.467831067	12516.432	11802.1045	23353.807	20810.264	4.15408073	8.144885482	2	High	4360	Unique	Used	74857	1	45.506	1	4.2137	-1.9086	1	100	21/26	0	4360	1905.96	MS2	953.483	193	Glioma_grade 2_5.raw
170016081			aPDFLPLLNk	Unambiguous	N-Term(iTRAQ4plex); K10(iTRAQ4plex)	HCD	2.4189363	0	1	1	2.24959	2.5487	2.3044	2.6107976	7.331219368	2328.717	2055.4355	5238.6597	5366.326	8.81535037	1.702476722	3	High	6643	Unique	Used	69673	1	61.831	1	3.231	-4.466	1	100	15/36	17.02035	6643	1415.84	MS2	472.619	177	Glioma_grade 2_4.raw
171906582	NP_064583.2	CCDC47	coiled-coil domain-containing protein 47 precursor [Homo sapiens]	5	14	2.151060104	15.63950074	97.148632	24.02	2.61E-04	483	55.838	4.8687	Unclassified	Biological_process unknown	Molecular function unknown	Mitochondrion	-																								
171906582			iESVHFSDQFSGPk	Unambiguous	N-Term(iTRAQ4plex); K14(iTRAQ4plex)	HCD	1.7716271	0	1	1	1.70481	1.7457	1.7969	1.8400447	3.33177345	53132.707	51887.44	90581.33	95475.22	1.676891083	3.719839807	2	High	3248	Unique	Used	80563	1	36.761	1	5.5331	2.1113	1	55	18/26	6.426672	3248	1865.97	MS2	933.487	217	Glioma_grade 2_6.raw
171906582			eMQDLSEFcSDkPk	Unambiguous	N-Term(iTRAQ4plex); C9(Methylthio); K12(iTRAQ4plex); K14(iTRAQ4plex)	HCD	1.8620373	0	1	1	1.62521	2.065	1.7023	2.1629553	14.0224322	33738.133	26552.977	54831.39	57432.902	16.85380326	3.277171652	3	High	4829	Unique	Used	81922	1	50.621	1	6.4715	-2.4185	1	45	29/52	18.45889	4829	2135.01	MS2	712.343	76	Glioma_grade 2_6.raw
171906582			mVHFLTHYADk	Unambiguous	N-Term(iTRAQ4plex); M1(Oxidation); K11(iTRAQ4plex)	HCD	1.7651706	0	1	1	1.35335	1.9889	1.6129	2.370385	24.24698895	28482.861	19381.4	38547.207	45941.38	26.89148295	12.37674828	3	High	1905	Unique	Used	85341	1	28.452	1	4.9447	0.7152	1	42	23/40	36.68952	1905	1665.87	MS2	555.961	197	Glioma_grade 2_1rep.raw
171906582			lAQAWFNTHR	Unambiguous	N-Term(iTRAQ4plex)	HCD	2.292021	0	1	1	2.20594	2.0005	2.6135	2.3700855	11.28709699	23587.719	26010.387	52033.06	61646.84	6.907864471	11.95984344	3	High	2798	Unique	Used	73455	1	32.853	1	4.4169	-0.9252	1	17	19/36	7.623941	2798	1387.73	MS2	463.249	162	Glioma_grade 2_5.raw
171906582			lAQAWFNTHR	Unambiguous	N-Term(iTRAQ4plex)	HCD	2.216294	0	1	1	2.02138	1.9233	2.5242	2.40175	13.09608594	22435.48	23579.57	45350.65	56632.234	3.516213923	15.64435959	3	High	2771	Unique	Used	73430	1	32.655	1	4.5025	11.873	1	19	21/36	0	2771	1387.75	MS2	463.255	173	Glioma_grade 2_5.raw
171906582			mVHFLTHYADk	Unambiguous	N-Term(iTRAQ4plex); K11(iTRAQ4plex)	HCD	2.0858264	0	1	1	2.00992	2.0865	2.0851	2.164626	3.02729595	16762.635	16147.085	33691.562	34952.4	2.645173397	2.597598023	3	High	3156	Unique	Used	95590	1	43.821	1	5.5577	0.4516	1	81	26/40	37.762	3156	1649.87	MS2	550.629	199	Glioma_grade 2_8rep.raw
171906582			mVHFLTHYADk	Unambiguous	N-Term(iTRAQ4plex); K11(iTRAQ4plex)	HCD	1.7640024	0	1	1	1.54863	1.899	1.6539	2.0280952	12.33929235	15696.825	12800.769	24308.648	25961.178	14.37188583	4.648972404	2	High	3146	Unique	Used	95583	1	43.7	1	3.9516	1.6436	1	100	16/20	61.7957	3146	1649.87	MS2	825.441	227	Glioma_grade 2_8rep.raw
171906582			mVHFLTHYADk	Unambiguous	N-Term(iTRAQ4plex); M1(Oxidation); K11(iTRAQ4plex)	HCD	1.7609496	0	1	1	1.59449	1.9653	1.5952	1.9661183	12.02694652	12284.513	9966.892	19587.516	19596.088	14.72990605	0.030938039	3	High	2077	Unique	Used	94884	1	30.488	1	4.9567	0.3854	1	100	23/40	31.94051	2077	1665.87	MS2	555.96	195	Glioma_grade 2_8rep.raw
171906582			mVHFLTHYADk	Unambiguous	N-Term(iTRAQ4plex); M1(Oxidation); K11(iTRAQ4plex)	HCD	2.070767	0	1	1	1.86051	2.2108	1.9529	2.3206034	10.32355581	9132.105	7685.289	16990.398	17834.508	12.16661041	3.427868004	4	High	1908	Unique	Used	85344	1	28.469	1	4.2517	1.0002	1	83	25/60	50.9682	1908	1665.87	MS2	417.222	232	Glioma_grade 2_1rep.raw
171906582			tLLFTFNVPGSGNTYPk	Unambiguous	N-Term(iTRAQ4plex); K17(iTRAQ4plex)	HCD	2.5877485	0	1	1	2.35711	2.5192	2.6519	2.8342493	7.807462875	9049.287	8466.946	21330.174	23997.436	4.701664679	8.321811132	2	High	7066	Unique	Used	54426	1	63.576	1	4.5391	-0.5384	1	90	21/32	0	7066	2144.16	MS2	1072.58	181	Glioma_grade 2_2.raw
171906582			tLLFTFNVPGSGNTYPk	Unambiguous	N-Term(iTRAQ4plex); K17(iTRAQ4plex)	HCD	2.8390698	0	1	1	2.80622	2.7969	2.8813	2.871816	1.537035598	6831.013	6853.657	19169.293	19682.441	0.234009676	1.867872515	2	High	6822	Unique	Used	62193	1	63.532	1	4.7695	0.6002	1	100	21/32	0	6822	2144.16	MS2	1072.59	183	Glioma_grade 2_3.raw
171906582			mVHFLTHYADk	Unambiguous	N-Term(iTRAQ4plex); M1(Oxidation); K11(iTRAQ4plex)	HCD	2.3379009	0	1	1	2.1117	2.5279	2.1792	2.6086857	10.51781314	6745.111	5634.5537	14243.649	14698.78	12.68665375	2.223906061	4	High	2086	Unique	Used	94891	1	30.582	1	4.2613	1.2201	1	100	24/60	37.81924	2086	1665.87	MS2	417.223	229	Glioma_grade 2_8rep.raw
171906582			mVHFLTHYADk	Unambiguous	N-Term(iTRAQ4plex); K11(iTRAQ4plex)	HCD	2.3338025	0	1	1	2.04059	2.6469	2.0924	2.7141266	15.0060652	5600.119	4317.397	11427.561	11717.961	18.29130247	1.774371814	4	High	2942	Unique	Used	86069	1	40.338	1	5.9091	######	1	100	25/60	27.04779	2942	1649.87	MS2	413.223	211	Glioma_grade 2_1rep.raw
171906582			mVHFLTHYADk	Unambiguous	N-Term(iTRAQ4plex); K11(iTRAQ4plex)	HCD	2.2563295	0	1	1	2.18305	2.2916	2.2227	2.3332512	2.987823726	3535.7185	3368.261	7718.653	7858.9995	3.430205255	1.274132439	4	High	3129	Unique	Used	95572	1	43.491	1	4.9753	-1.4062	1	100	26/60	10.31245	3129	1649.87	MS2	413.223	175	Glioma_grade 2_8rep.raw
172072620	NP_055047.2	VAMP2	vesicle-associated membrane protein 2 [Homo sapiens]	4	9	0.409398556	20.80425051	88.799653	35.34	9.95E-05	116	12.655	8.1333	Membrane transport protein	Transport	Auxiliary transport protein activity	Plasma membrane	Endosome, Zymogen granule, Golgi apparatus																								
172072620			aDALQAGASQFETSAAk	Unambiguous	N-Term(iTRAQ4plex); K17(iTRAQ4plex)	HCD	0.38654232	0	1	2	0.41599	0.3533	0.4257	0.36152935	0.095011937	361139.53	425203.9	150231.33	153723.69	11.52177248	1.624892682	2	High	3871	Unique	Used	51421	1	40.139	1	6.8427	0.4707	1	12	25/32	8.215196	3871	1954.01	MS2	977.51	201	Glioma_grade 2_2.raw
172072620			lQQTQAQVDEVVDIMR	Unambiguous	N-Term(iTRAQ4plex)	HCD	0.26322943	0	1	1	0.25064	0.2528	0.2736	0.27592444	0.05077368	284693.62	282279.25	71356.2	77887.74	0.602221901	6.189190966	2	High	4670	Unique	Used	87158	1	61.889	1	6.4308	0.3831	1	20	24/30	7.530696	4670	2017.05	MS2	1009.03	174	Glioma_grade 2_1rep.raw
172072620			aDALQAGASQFETSAAk	Unambiguous	N-Term(iTRAQ4plex); K17(iTRAQ4plex)	HCD	0.36513293	0	1	2	0.3576	0.3558	0.3745	0.3726334	0.026764647	60018.67	60314.22	21462.408	22475.094	0.34734545	3.259525941	2	High	3020	Unique	Used	50618	1	34.03	1	6.374	0.7206	1	54	25/32	0	3020	1954.01	MS2	977.51	195	Glioma_grade 2_2.raw
172072620			lQQTQAQVDEVVDImR	Unambiguous	N-Term(iTRAQ4plex); M15(Oxidation)	HCD	0.34353885	0	1	1	0.32436	0.3482	0.3392	0.36412093	0.048380872	56684.066	52806.312	18386.314	19227.883	5.008633999	3.164119903	2	High	3735	Unique	Used	86533	1	50.813	1	4.9864	-0.6612	1	100	18/30	35.13801	3735	2033.04	MS2	1017.03	173	Glioma_grade 2_1rep.raw
172072620			aDALQAGASQFETSAAk	Unambiguous	N-Term(iTRAQ4plex); K17(iTRAQ4plex)	HCD	0.40939856	0	1	2	0.39093	0.4261	0.3941	0.42953223	0.049877847	55637.137	51049.207	21749.955	21927.28	6.081671361	0.574155896	2	High	4075	Unique	Used	51614	1	41.636	1	7.1817	2.8446	1	97	25/32	18.72961	4075	1954.02	MS2	977.512	200	Glioma_grade 2_2.raw
172072620			lQQTQAQVDEVVDIMR	Unambiguous	N-Term(iTRAQ4plex)	HCD	0.48890403	0	1	1	0.49114	0.601	0.3973	0.48617187	0.168933658	37864.332	30940.307	18596.559	15042.307	14.2316713	14.94245193	3	High	4675	Unique	Used	87162	1	61.941	1	5.959	0.8606	1	12	24/60	12.26351	4675	2017.05	MS2	673.022	174	Glioma_grade 2_1rep.raw
172072620			aDALQAGASQFETSAAk	Unambiguous	N-Term(iTRAQ4plex); K17(iTRAQ4plex)	HCD	0.45157132	0	1	2	0.4181	0.4618	0.4423	0.48853695	0.065932888	22641.033	20497.94	9466.321	10014.001	7.0256452	3.975994256	2	High	3558	Unique	Used	51127	1	37.868	1	5.8178	-2.2156	1	100	25/32	0	3558	1954.01	MS2	977.508	176	Glioma_grade 2_2.raw
172072620			rLQQTQAQVDEVVDIMR	Unambiguous	N-Term(iTRAQ4plex)	HCD	0.44934815	1	1	1	0.37627	0.393	0.5033	0.5256664	0.168700843	11429.967	10944.186	4300.7935	5752.991	3.070498707	20.42730675	2	High	5119	Unique	Used	75530	1	51.779	1	3.6456	0.2222	1	42	20/32	8.864519	5119	2173.15	MS2	1087.08	127	Glioma_grade 2_5.raw
172072620			lSELDDRADALQAGASQFE TSAAk	Unambiguous	N-Term(iTRAQ4plex); K24(iTRAQ4plex)	HCD	0.5547228	1	1	2	0.55312	0.7603	0.4052	0.5569207	0.256410137	3231.4443	2350.9302	1787.3887	1309.2817	22.30654683	21.83459382	3	High	5812	Unique	Used	68912	1	55.141	1	5.339	1.9843	1	100	30/92	10.12806	5812	2782.42	MS2	928.143	76	Glioma_grade 2_4.raw
181337167	NP_066000.2	SLC7A14	probable cationic amino acid transporter [Homo sapiens]	5	5	0.385196775	15.44564858	20.944354	8.3	1.71E-04	771	83.997	5.2368	Integral membrane protein	Biological_process unknown	Molecular function unknown	-	-																								
181337167			eGILADcEk	Unambiguous	N-Term(iTRAQ4plex); C7(Methylthio); K9(iTRAQ4plex)	HCD	0.35647553	0	1	1	0.35448	0.3612	0.3518	0.35851043	0.011684976	498169.34	488890.34	176590.33	175272.3	1.329452303	0.529745345	2	High	4279	Unique	Used	51806	1	43.128	1	3.5687	11.19	1	22	15/16	67.75445	4279	1311.67	MS2	656.337	185	Glioma_grade 2_2.raw
181337167			nLPSLGDNEMLIGk	Unambiguous	N-Term(iTRAQ4plex); K14(iTRAQ4plex)	HCD	0.33909407	0	1	1	0.33151	0.3569	0.3226	0.3472567	0.045369208	242478.16	225247.7	80384.3	78218.77	5.209793236	1.930934815	2	High	5705	Unique	Used	53148	1	53.525	1	5.9382	2.0397	1	43	22/26	18.85465	5705	1788.98	MS2	894.994	216	Glioma_grade 2_2.raw
181337167			gFYYQQmSDAk	Unambiguous	N-Term(iTRAQ4plex); M7(Oxidation); K11(iTRAQ4plex)	HCD	0.49287996	0	1	1	0.43155	0.4605	0.5232	0.5583171	0.117087456	71436.84	66948.164	30828.889	37378.31	4.58716356	13.5796223	2	High	1564	Unique	Used	49246	1	23.756	1	3.2672	-2.0759	1	61	14/20	34.6314	1564	1641.78	MS2	821.393	97	Glioma_grade 2_2.raw
181337167			lIGPHYYTMR	Unambiguous	N-Term(iTRAQ4plex)	HCD	0.42946294	0	1	1	0.43788	0.47	0.3917	0.42043078	0.076121075	55961.84	52142.31	24504.494	21922.232	4.996691735	7.865878679	3	High	2269	Unique	Used	72958	1	29.015	1	3.5954	0.2561	1	38	20/36	35.71508	2269	1394.74	MS2	465.584	207	Glioma_grade 2_5.raw
181337167			tkPVESMLEGTGTTTAHGTk	Unambiguous	N-Term(iTRAQ4plex); K2(iTRAQ4plex); K20(iTRAQ4plex)	HCD	0.38519678	0	1	1	0.26568	0.3838	0.3862	0.5578511	0.301923436	32565.004	22541.938	8651.973	12575.044	25.72226903	26.13678697	4	High	1853	Unique	Used	89699	1	25.414	1	4.5749	-2.7968	1	13	28/114	32.24191	1853	2478.32	MS2	620.335	121	Glioma_grade 2_7rep.raw
187608363	NP_001120645 .1	CADM3	cell adhesion molecule 3 isoform 2 precursor [Homo sapiens]	5	12	0.382140756	15.81783559	90.840898	25.88	1.19E-04	398	43.273	6.0874	Immunoglob ulin	Immune response	Antigen binding	Plasma membrane	-																								
187608363			gNPVPQQYLWEk	Unambiguous	N-Term(iTRAQ4plex); K12(iTRAQ4plex)	HCD	0.36512586	0	1	2	0.32236	0.3905	0.3442	0.41692442	0.116691303	554574.6	457839.8	178774.08	190884.58	13.5126156	4.633148145	2	High	4391	Unique	Used	51911	1	43.964	1	4.3028	1.5675	1	10	18/22	2.189908	4391	1746.94	MS2	873.976	233	Glioma_grade 2_2.raw
187608363			gNPVPQQYLWEk	Unambiguous	N-Term(iTRAQ4plex); K12(iTRAQ4plex)	HCD	0.43692672	0	1	2	0.44518	0.4326	0.4414	0.4289071	0.017281939	184351.66	189716.9	82069.62	81370.92	2.028396926	0.604569109	2	High	4300	Unique	Used	51826	1	43.276	1	4.6546	4.9215	1	41	18/22	6.584926	4300	1746.95	MS2	873.979	221	Glioma_grade 2_2.raw
187608363			sLVTVLGIPQkPIITGYk	Unambiguous	N-Term(iTRAQ4plex); K11(iTRAQ4plex); K18(iTRAQ4plex)	HCD	0.4447393	0	1	2	0.43605	0.4868	0.407	0.45444033	0.074950307	176250.64	157870.06	76854.086	71742.52	7.779842889	4.864745001	3	High	5286	Unique	Used	92437	1	58.462	1	5.3754	3.2233	1	13	25/68	30.7206	5286	2359.48	MS2	787.164	5	Glioma_grade 2_7rep.raw
187608363			sLVTVLGIPQkPIITGYk	Unambiguous	N-Term(iTRAQ4plex); K11(iTRAQ4plex); K18(iTRAQ4plex)	HCD	0.30590758	0	1	2	0.26641	0.3047	0.307	0.3510791	0.112656193	172355.75	150695.52	45917.777	52906.05	9.482145367	10.00053404	2	High	5238	Unique	Used	92399	1	57.998	1	5.0761	-1.2979	1	20	18/34	6.602737	5238	2359.47	MS2	1180.24	6	Glioma_grade 2_7rep.raw
187608363			gNPVPQQYLWEk	Unambiguous	N-Term(iTRAQ4plex); K12(iTRAQ4plex)	HCD	0.39384735	0	1	2	0.35568	0.4054	0.3837	0.43735957	0.087298945	141993.81	124563.4	50503.855	54478.992	9.247666653	5.354867789	2	High	4267	Unique	Used	59818	1	43.596	1	4.8179	-2.4155	1	34	17/22	20.06378	4267	1746.94	MS2	873.972	227	Glioma_grade 2_3.raw
187608363			gNPVPQQYLWEk	Unambiguous	N-Term(iTRAQ4plex); K12(iTRAQ4plex)	HCD	0.46555707	0	1	2	0.46838	0.4675	0.4636	0.4627353	0.006046027	110496.93	110698.28	51754.99	51224	0.128733303	0.729210162	2	High	4167	Unique	Used	59725	1	42.817	1	4.7262	3.9433	1	72	16/22	32.04275	4167	1746.95	MS2	873.978	226	Glioma_grade 2_3.raw
187608363			lLLHcEGR	Unambiguous	N-Term(iTRAQ4plex); C5(Methylthio)	HCD	0.3579346	0	1	2	0.34339	0.3471	0.3687	0.37263697	0.041459216	98787.055	97735.12	33922.363	36419.72	0.756993832	5.020886486	3	High	2254	Unique	Used	79682	1	28.548	1	3.4478	7.4388	1	100	17/28	45.86281	2254	1130.6	MS2	377.539	145	Glioma_grade 2_6.raw
187608363			eDDGASIVcSVNHESLk	Unambiguous	N-Term(iTRAQ4plex); C9(Methylthio); K17(iTRAQ4plex)	HCD	0.3704342	0	1	2	0.31639	0.3321	0.4069	0.4271693	0.147250701	62988.06	60001.043	19928.764	25630.604	3.434678235	17.69914688	3	High	4257	Unique	Used	67481	1	42.903	1	6.7411	1.2521	1	13	33/64	7.457664	4257	2137.02	MS2	713.011	90	Glioma_grade 2_4.raw
187608363			eDDGASIVcSVNHESLk	Unambiguous	N-Term(iTRAQ4plex); C9(Methylthio); K17(iTRAQ4plex)	HCD	0.46205643	0	1	2	0.54951	0.4057	0.5384	0.39749146	0.174275528	28020.754	37954.996	15397.733	15086.786	21.29439949	1.442520594	3	High	3840	Unique	Used	74386	1	41.341	1	4.1876	1.2521	1	22	30/64	26.94325	3840	2137.02	MS2	713.011	85	Glioma_grade 2_5.raw
187608363			eDDGASIVcSVNHESLk	Unambiguous	N-Term(iTRAQ4plex); C9(Methylthio); K17(iTRAQ4plex)	HCD	0.35914645	0	1	2	0.36961	0.3304	0.3913	0.34978887	0.072537429	22601.174	25281.541	8353.705	8843.201	7.916450359	4.025444356	2	High	4028	Unique	Used	59595	1	41.751	1	5.7016	1.6011	1	100	24/32	10.09392	4028	2137.02	MS2	1069.01	114	Glioma_grade 2_3.raw
187608363			eDDGASIVcSVNHESLk	Unambiguous	N-Term(iTRAQ4plex); C9(Methylthio); K17(iTRAQ4plex)	HCD	0.46358117	0	1	2	0.52195	0.5031	0.4226	0.40732267	0.12332463	19993.09	20742.729	10435.374	8448.983	2.60249988	14.87570423	3	High	4096	Unique	Used	59659	1	42.263	1	6.0065	2.88	1	46	33/64	10.53907	4096	2137.02	MS2	713.012	84	Glioma_grade 2_3.raw
187608363			dHEDSSLQWSNPAQQTLYF GEk	Unambiguous	N-Term(iTRAQ4plex); K22(iTRAQ4plex)	HCD	0.28554082	0	1	2	0.25925	0.318	0.2591	0.3177884	0.117551367	9030.825	7362.006	2341.2622	2339.56	14.39694256	0.05142845	2	High	5236	Unique	Used	75632	1	52.776	1	5.0981	-1.0143	1	59	23/42	23.45555	5236	2868.37	MS2	1434.69	58	Glioma_grade 2_5.raw
188497750	NP_277035.2	HK1	hexokinase-1 isoform HKI-td [Homo sapiens]	13	22	0.50147295	17.86288423	122.38018	20.33	1.19E-04	905	101.02	6.9028	Enzyme: Sugar phosphotran sferase	Metabolism,Energy pathways	Catalytic activity	Cytoplasm	Mitochondrion, Mitochondrial membrane, Plasma membrane																								
188497750			hIDLVEGDEGR	Unambiguous	N-Term(iTRAQ4plex)	HCD	0.3679682	0	1	4	0.34905	0.3648	0.371	0.38773784	0.04343662	2524939.8	2415665.2	881340.6	936644.9	3.127908856	4.302129534	2	High	1308	Unique	Used	64744	1	20.511	1	3.6871	1.7641	1	3	17/20	6.918747	1308	1383.7	MS2	692.354	161	Glioma_grade 2_4.raw
188497750			dFNPTATVk	Unambiguous	N-Term(iTRAQ4plex); K9(iTRAQ4plex)	HCD	0.4678221	0	1	4	0.43928	0.4444	0.491	0.49669215	0.064546923	1700231.5	1680773.5	746881.9	834827	0.813893132	7.863213842	2	High	1589	Unique	Used	49269	1	23.944	1	3.6662	-1.4277	1	5	15/16	18.58702	1589	1280.71	MS2	640.857	239	Glioma_grade 2_2.raw
188497750			fLLSESGSGk	Unambiguous	N-Term(iTRAQ4plex); K10(iTRAQ4plex)	HCD	0.5664913	0	1	4	0.54207	0.5582	0.5746	0.5916361	0.037619124	1336341.6	1297798.6	724393	767824.56	2.069291275	4.116122396	2	High	2375	Unique	Used	50013	1	29.404	1	3.6516	2.2529	1	11	17/18	30.54774	2375	1312.74	MS2	656.873	194	Glioma_grade 2_2.raw
188497750			fLLSESGSGk	Unambiguous	N-Term(iTRAQ4plex); K10(iTRAQ4plex)	HCD	0.493043	0	1	4	0.4892	0.4954	0.4907	0.4969304	0.007457982	931143.1	919548.44	455518.97	456951.53	0.886010719	0.222028633	2	High	2340	Unique	Used	58022	1	28.972	1	3.6337	-3.0475	1	19	17/18	14.76775	2340	1312.73	MS2	656.869	176	Glioma_grade 2_3.raw

188497750			fNTSDVSAIEk	Unambiguous	N-Term(iTRAQ4plex); K11(iTRAQ4plex)	HCD	0.47659758	0	1	4	0.44976	0.4766	0.4766	0.50504166	0.047326318	831130.1	784274.44	373806.62	396091.28	4.102000967	4.093434779	2	High	3053	Unique	Used	50648	1	34.286	1	4.8712	2.4798	1	11	20/20	11.5645	3053	1498.8	MS2	749.905	226	Glioma_grade2_2.raw
188497750			fLLSESGSGk	Unambiguous	N-Term(iTRAQ4plex); K10(iTRAQ4plex)	HCD	0.50990295	0	1	4	0.47385	0.5671	0.4621	0.55305904	0.104490416	273455.9	228478.19	129575.766	126361.92	12.67259761	1.775848126	2	High	2438	Unique	Used	58113	1	29.715	1	3.6094	7.5532	1	40	17/18	20.75386	2438	1312.75	MS2	656.876	183	Glioma_grade2_3.raw
188497750			tTVGVDGSLYk	Unambiguous	N-Term(iTRAQ4plex); K11(iTRAQ4plex)	HCD	0.51184374	0	1	4	0.49896	0.4892	0.535	0.52446944	0.041828541	233360.69	238039.22	116438.78	124844.3	1.403572728	4.926661407	2	High	1999	Unique	Used	49658	1	26.759	1	3.9982	-0.5549	1	18	18/20	13.4989	1999	1427.8	MS2	714.402	251	Glioma_grade2_2.raw
188497750			aTDcVGHDVVTLLR	Unambiguous	N-Term(iTRAQ4plex); C4(Methylthio)	HCD	0.45095477	0	1	4	0.4253	0.4341	0.4675	0.4771417	0.055810252	231852.25	227151	98606.49	108383.22	1.448480269	6.67974469	2	High	5313	Unique	Used	60789	1	51.762	1	4.5653	2.6652	1	23	23/26	17.00388	5313	1688.86	MS2	844.935	173	Glioma_grade2_3.raw
188497750			lVDEYSLNAGk	Unambiguous	N-Term(iTRAQ4plex); K11(iTRAQ4plex)	HCD	0.5568134	0	1	4	0.54454	0.514	0.6022	0.5683948	0.067003869	230113.56	243808.97	125306.67	138579.73	4.086793376	7.113265961	2	High	3224	Unique	Used	50810	1	35.499	1	5.0262	-2.0692	1	20	17/20	18.23741	3224	1496.82	MS2	748.912	255	Glioma_grade2_2.raw
188497750			aTDcVGHDVVTLLR	Unambiguous	N-Term(iTRAQ4plex); C4(Methylthio)	HCD	0.6111585	0	1	4	0.53287	0.5839	0.6361	0.6969384	0.114856414	224126.94	204546.73	119431.086	142556.48	6.459598635	12.483129	2	High	5527	Unique	Used	52980	1	52.249	1	4.8652	0.3522	1	22	23/26	17.09072	5527	1688.86	MS2	844.933	217	Glioma_grade2_2.raw
188497750			qIEETLAHFHLTk	Unambiguous	N-Term(iTRAQ4plex); K13(iTRAQ4plex)	HCD	0.60351604	0	1	4	0.55982	0.5996	0.6072	0.65031534	0.061383017	185908.5	173584.86	104075.12	112884.9	4.848005767	5.742491339	3	High	4458	Unique	Used	91808	1	50.036	1	5.5781	-0.4261	1	11	26/48	35.11969	4458	1855.03	MS2	619.015	176	Glioma_grade2_7rep.raw
188497750			iDEAILITWTk	Unambiguous	N-Term(iTRAQ4plex); K11(iTRAQ4plex)	HCD	0.35814226	0	1	4	0.32871	0.3693	0.3482	0.39121598	0.074992985	165866.53	147621.47	54521.414	57751.88	8.230749279	4.069150078	2	High	7031	Unique	Used	54393	1	63.317	1	4.9343	1.4897	1	30	16/20	31.47107	7031	1590.94	MS2	795.972	176	Glioma_grade2_2.raw
188497750			aTDcVGHDVVTLLR	Unambiguous	N-Term(iTRAQ4plex); C4(Methylthio)	HCD	0.5313137	0	1	4	0.51752	0.523	0.5395	0.5452589	0.02480473	143631.55	142120.67	74331.52	77492.555	0.747748167	2.944446438	2	High	5493	Unique	Used	68623	1	52.57	1	4.7754	3.7494	1	72	22/26	9.871124	5493	1688.86	MS2	844.936	175	Glioma_grade2_4.raw
188497750			sANLVAATLGAILNR	Unambiguous	N-Term(iTRAQ4plex)	HCD	0.3653784	0	1	4	0.34508	0.3502	0.3803	0.385981	0.056697818	111252.72	109610.98	38391.074	42307.75	1.051223435	6.863812933	2	High	6004	Unique	Used	88158	1	76.297	1	4.1517	3.7399	1	29	20/28	36.86342	6004	1627.97	MS2	814.487	129	Glioma_grade2_1rep.raw
188497750			aTDcVGHDVVTLLR	Unambiguous	N-Term(iTRAQ4plex); C4(Methylthio)	HCD	0.3504913	0	1	4	0.32956	0.3604	0.3415	0.3733754	0.055475522	110140.48	100726.58	36298.24	37608.824	6.313582147	2.50780584	3	High	5523	Unique	Used	52976	1	52.226	1	4.0719	-1.535	1	6	22/52	8.923395	5523	1688.86	MS2	563.623	141	Glioma_grade2_2.raw
188497750			lSDETLIDIMTR	Unambiguous	N-Term(iTRAQ4plex)	HCD	0.44860575	0	1	4	0.40773	0.4501	0.4473	0.49373063	0.078161888	106186.5	96194.85	43295.09	47494.344	6.982030182	6.541115741	2	High	5247	Unique	Used	87589	1	68.118	1	5.271	0.9501	1	63	19/22	27.05113	5247	1550.82	MS2	775.915	269	Glioma_grade2_1rep.raw
188497750			fNTSDVSAIEk	Unambiguous	N-Term(iTRAQ4plex); K11(iTRAQ4plex)	HCD	0.46937996	0	1	4	0.46668	0.4614	0.4775	0.47204775	0.014800366	100253.21	101411.3	46786.305	47870.973	0.812134264	1.620532756	2	High	2966	Unique	Used	50570	1	33.589	1	4.2933	-0.778	1	66	19/20	0	2966	1498.8	MS2	749.903	215	Glioma_grade2_2.raw
188497750			iDEAILITWTk	Unambiguous	N-Term(iTRAQ4plex); K11(iTRAQ4plex)	HCD	0.46158445	0	1	4	0.43269	0.3993	0.5291	0.48824486	0.12476234	53290.867	57749.31	23058.615	28195.803	5.678296566	14.17454578	2	High	6773	Unique	Used	62147	1	63.158	1	4.7313	2.6406	1	100	18/20	35.32098	6773	1590.94	MS2	795.973	175	Glioma_grade2_3.raw
188497750			cNVSFLLSEDGSGk	Unambiguous	N-Term(iTRAQ4plex); C1(Methylthio); K14(iTRAQ4plex)	HCD	0.5342975	0	1	4	0.4645	0.5334	0.5351	0.6144485	0.114170254	34458.406	30006.068	16006.024	18437.184	9.76748338	9.982169618	2	High	6449	Unique	Used	53849	1	58.978	1	4.526	0.3805	1	87	18/26	10.41107	6449	1789.87	MS2	895.439	112	Glioma_grade2_2.raw
188497750			aTDcVGHDVVTLLR	Unambiguous	N-Term(iTRAQ4plex); C4(Methylthio)	HCD	0.64212847	0	1	4	0.56456	0.667	0.6211	0.73377144	0.110800357	29560.67	25019.863	16688.854	18358.861	11.76549676	6.738660562	3	High	5296	Unique	Used	60774	1	51.616	1	4.2652	1.3923	1	56	24/52	20.27441	5296	1688.86	MS2	563.625	149	Glioma_grade2_3.raw
188497750			kLPVGFTFSFPcQQSk	Unambiguous	N-Term(iTRAQ4plex); K1(iTRAQ4plex); C12(Methylthio); K16(iTRAQ4plex)	HCD	0.6589853	1	1	4	0.66464	0.735	0.5902	0.6527359	0.089814995	14850.6875	13429.017	9870.308	8765.602	7.109500392	8.383224686	3	High	4793	Unique	Used	96530	1	65.773	1	5.9811	-0.6743	1	100	24/60	35.73499	4793	2292.22	MS2	764.746	149	Glioma_grade2_8rep.raw
188497750			qIEETLAHFHLTk	Unambiguous	N-Term(iTRAQ4plex); K13(iTRAQ4plex)	HCD	0.52778447	0	1	4	0.48399	0.5146	0.5402	0.5743455	0.072585781	14827.096	13944.745	7176.2285	8009.1025	4.336993072	7.756575781	4	High	4431	Unique	Used	91788	1	49.745	1	7.0742	-6.981	1	49	32/72	27.88588	4431	1855.02	MS2	464.51	197	Glioma_grade2_7rep.raw
189083772	NP_001121134.1	GSN	gelsolin isoform b [Homo sapiens]	11	19	2.773602247	20.18412318	126.79429	24.62	1.19E-04	731	80.591	5.8462	Cytoskeletal protein	Cell growth and/or maintenance	Structural constituent of cytoskeleton	Extracellular	Mitochondrion, Cytoplasm, Cytoskeleton																								
189083772			tASDFITk	Unambiguous	N-Term(iTRAQ4plex); K8(iTRAQ4plex)	HCD	2.5384583	0	1	4	2.62177	2.2923	2.82	2.4656193	8.81618723	1027522.44	1175221.1	2693924.8	2897647.8	9.482604049	5.152536687	2	High	1663	Unique	Used	49339	1	24.464	1	3.346	-1.0009	1	2	14/14	11.81856	1663	1170.66	MS2	585.834	164	Glioma_grade2_2.raw
189083772			hVVPNEVVVQR	Unambiguous	N-Term(iTRAQ4plex)	HCD	2.7551706	0	1	4	2.79856	2.741	2.7696	2.7126703	1.341066349	742723.25	758309.3	2078557.5	2057043.2	1.46845605	0.735704848	2	High	1242	Unique	Used	64681	1	20.084	1	3.1864	1.3622	1	3	16/20	2.316662	1242	1419.82	MS2	710.414	163	Glioma_grade2_4.raw
189083772			sEDcFILDHGk	Unambiguous	N-Term(iTRAQ4plex); C4(Methylthio); K11(iTRAQ4plex)	HCD	2.9021332	0	1	4	2.55628	3.7222	2.339	3.40572	22.06711903	556299.1	382051.38	1422058	1301160	26.26134841	6.278439378	3	High	3440	Unique	Used	74048	1	37.756	1	3.8881	1.6766	1	1	24/40	5.836908	3440	1597.76	MS2	533.259	69	Glioma_grade2_5.raw
189083772			hVVPNEVVVQR	Unambiguous	N-Term(iTRAQ4plex)	HCD	2.6705728	0	1	4	2.59697	3.3415	2.1492	2.7652822	18.16915115	445333.78	346112	1156516.2	957097.4	17.72967783	13.34306193	3	High	1240	Unique	Used	64679	1	20.075	1	4.8133	0.9658	1	1	21/40	7.110042	1240	1419.82	MS2	473.945	201	Glioma_grade2_4.raw
189083772			qTQVSVLPEGGETPLFk	Unambiguous	N-Term(iTRAQ4plex); K17(iTRAQ4plex)	HCD	3.3423765	0	1	4	3.06056	3.4841	3.2179	3.6631868	8.014974405	104683.23	91958.82	320389.4	336862.34	9.151162325	3.544495015	2	High	5587	Unique	Used	53037	1	52.668	1	5.0831	-2.1043	1	8	19/32	10.02172	5587	2118.16	MS2	1059.59	147	Glioma_grade2_2.raw
189083772			aGALNSNDAFVLk	Unambiguous	N-Term(iTRAQ4plex); K13(iTRAQ4plex)	HCD	2.7736022	0	1	4	2.68162	2.8547	2.6974	2.8715205	3.625755277	97703.14	91779.52	262002.8	263546.78	4.421124203	0.415473162	2	High	3935	Unique	Used	51482	1	40.586	1	6.0997	3.9456	1	41	22/24	25.16874	3935	1607.91	MS2	804.457	272	Glioma_grade2_2.raw
189083772			aGALNSNDAFVLk	Unambiguous	N-Term(iTRAQ4plex); K13(iTRAQ4plex)	HCD	3.316667	0	1	4	3.46124	3.1892	3.455	3.1834612	4.724880837	80151.3	86988.805	277422.6	276925.47	5.785381255	0.126824287	2	High	3850	Unique	Used	59430	1	40.367	1	6.0883	0.757	1	30	22/24	0	3850	1607.9	MS2	804.454	253	Glioma_grade2_3.raw
189083772			sEDcFILDHGk	Unambiguous	N-Term(iTRAQ4plex); C4(Methylthio); K11(iTRAQ4plex)	HCD	3.0949113	0	1	4	3.00019	3.0386	3.1506	3.1908457	2.916798162	61911.37	61129.727	185745.77	195055.53	0.898407246	3.457443253	2	High	3413	Unique	Used	74023	1	37.55	1	5.1266	0.921	1	97	18/20	41.46464	3413	1597.76	MS2	799.384	89	Glioma_grade2_5.raw
189083772			dSQEEEkTEALTSAk	Unambiguous	N-Term(iTRAQ4plex); K7(iTRAQ4plex); K15(iTRAQ4plex)	HCD	1.7068154	1	1	4	1.48677	1.8335	1.6041	1.978172	12.83797483	49071.477	39791.938	72958.14	78715.29	14.76788834	5.368006519	2	High	1436	Unique	Used	72194	1	22.701	1	5.7304	-0.5514	1	26	20/28	5.593384	1436	2098.09	MS2	1049.55	158	Glioma_grade2_5.raw
189083772			aQPVQVAEGSEPDGFWEALGGk	Unambiguous	N-Term(iTRAQ4plex); K22(iTRAQ4plex)	HCD	3.4692464	0	1	4	2.86347	3.73	3.2691	4.2583365	17.01827455	46627.965	35795.668	133517.89	152430	18.58590888	9.353369404	2	High	6908	Unique	Used	54276	1	62.435	1	5.7399	-0.4859	1	10	26/42	2.195566	6908	2560.29	MS2	1280.65	145	Glioma_grade2_2.raw
189083772			aGALNSNDAFVLk	Unambiguous	N-Term(iTRAQ4plex); K13(iTRAQ4plex)	HCD	2.0683498	0	1	4	1.92307	2.039	2.096	2.2223914	6.010587348	45740.27	43138.812	87961.72	95871.33	4.139351018	6.084802344	2	High	4020	Unique	Used	51561	1	41.243	1	5.8551	-0.0022	1	41	22/24	9.996595	4020	1607.9	MS2	804.453	251	Glioma_grade2_2.raw
189083772			aQPVQVAEGSEPDGFWEALGGk	Unambiguous	N-Term(iTRAQ4plex); K22(iTRAQ4plex)	HCD	1.8530129	0	1	4	1.9458	1.7312	1.9899	1.7704592	6.861031607	39810.484	44744.227	77463.15	79217.83	8.251895348	1.583786528	3	High	6878	Unique	Used	54248	1	62.215	1	7	0.7246	1	2	28/84	4.421028	6878	2560.29	MS2	854.103	150	Glioma_grade2_2.raw
189083772			tPSAAYLWVGTGASEAEk	Unambiguous	N-Term(iTRAQ4plex); K18(iTRAQ4plex)	HCD	2.7983499	0	1	4	2.80829	2.541	3.0828	2.7893598	7.893259608	24108.965	26644.893	67704.86	74322.195	7.066150066	6.589114239	2	High	5434	Unique	Used	52892	1	51.585	1	5.8646	-2.6409	1	83	22/34	22.44895	5434	2126.1	MS2	1063.55	198	Glioma_grade2_2.raw
189083772			eVQGFESATFLGYFk	Unambiguous	N-Term(iTRAQ4plex); K15(iTRAQ4plex)	HCD	2.6372914	0	1	4	2.52845	2.8054	2.4857	2.7580624	6.076784627	23224.127	20931.098	58720.926	57729.273	7.344165296	1.204299463	2	High	7610	Unique	Used	54918	1	67.989	1	6.6814	-0.0029	1	69	23/28	16.98146	7610	2011.04	MS2	1006.02	198	Glioma_grade2_2.raw
189083772			dPDQTDGLGLSYLSSHIANVER	Unambiguous	N-Term(iTRAQ4plex)	HCD	3.0908139	0	1	4	2.44971	3.0497	3.1238	3.8889463	18.87021287	18737.182	15050.684	45900.703	58531.3	15.43008212	17.10429855	3	High	6398	Unique	Used	53801	1	58.604	1	8.4875	0.8323	1	21	39/84	25.22164	6398	2531.25	MS2	844.422	105	Glioma_grade2_2.raw
189083772			tPSAAYLWVGTGASEAEk	Unambiguous	N-Term(iTRAQ4plex); K18(iTRAQ4plex)	HCD	1.9398996	0	1	4	1.76343	1.8676	2.0082	2.1267881	8.195296489	18235.836	17218.865	32157.682	36620.875	4.056483739	9.177145243	2	High	5538	Unique	Used	52991	1	52.318	1	5.8342	-2.0668	1	73	22/34	0	5538	2126.1	MS2	1063.55	206	Glioma_grade2_2.raw
189083772			vPFDAATLHTSTAmAAQHGmDDDGTGQk	Unambiguous	N-Term(iTRAQ4plex); M14(Oxidation); M20(Oxidation); K28(iTRAQ4plex)	HCD	2.6363142	0	1	4	2.61713	3.1962	2.1779	2.6597462	15.66163457	4928.123	4035.295	12897.524	10732.86	14.08669624	12.95491934	4	High	2481	Unique	Used	79896	1	30.19	1	4.0282	5.8747	1	22	22/162	16.75288	2481	3193.5	MS2	799.129	46	Glioma_grade2_6.raw
189083772			vPFDAATLHTSTAMAAQHGmDDDGTGQk	Unambiguous	N-Term(iTRAQ4plex); M20(Oxidation); K28(iTRAQ4plex)	HCD	3.7353835	0	1	4	4.07584	4.1134	3.3608	3.391785	11.11520391	4784.4067	4740.713	19500.496	16079.478	0.648729098	13.59767731	3	High	3137	Unique	Used	80462	1	35.893	1	7.2346	3.2902	1	40	29/108	0	3137	3177.49	MS2	1059.84	62	Glioma_grade2_6.raw
189083772			vPFDAATLHTSTAMAAQHGMDDDGTGQk	Unambiguous	N-Term(iTRAQ4plex); K28(iTRAQ4plex)	HCD	2.9504347	0	1	4	3.17847	3.0285	2.8685	2.7331605	6.545213731	4142.701	4347.8135	13167.437	11883.272	3.41643465	7.249629379	4	High	3860	Unique	Used	81104	1	41.861	1	5.9514	4.4163	1	29	26/162	8.478571	3860	3161.5	MS2	791.131	47	Glioma_grade2_6.raw
189217897	NP_640337.3	STXBP5	syntaxin-binding protein 5 isoform a [Homo sapiens]	3	4	0.433350623	5.876572782	28.923722	7.53	1.49E-04	1115	123.41	7.1079	Transport/cargo protein	Cell communication,Signal transduction	Transporter activity	Cytoplasm	Cytoplasmic vesicle, Plasma membrane																								
189217897			sPAkPVQTITPHGk	Unambiguous	N-Term(iTRAQ4plex); K4(iTRAQ4plex); K14(iTRAQ4plex)	HCD	0.42702842	0	1	2	0.38835	0.4619	0.3977	0.47302794	0.10082442	54476.547	45802.926	21156.121	21666.062	12.23216685	1.684093215	4	High	1034	Unique	Used	84671	1	19.467	1	4.0357	-8.2133	1	44	19/78	64.88095	1034	1893.11	MS2	474.034	109	Glioma_grade2_1rep.raw
189217897			ePEIQETLQSEHFQLck	Unambiguous	N-Term(iTRAQ4plex); C16(Methylthio); K17(iTRAQ4plex)	HCD	0.47998962	0	1	2	0.45932	0.4551	0.5051	0.50047076	0.055105768	24301.3	24527.367	11162.023	12275.231	0.654752704	6.717142936	3	High	5578	Unique	Used	75940	1	55.529	1	7.0195	-2.6173	1	61	25/64	49.16945	5578	2393.17	MS2	798.393	146	Glioma_grade2_5.raw
189217897			ePEIQETLQSEHFQLck	Unambiguous	N-Term(iTRAQ4plex); C16(Methylthio); K17(iTRAQ4plex)	HCD	0.42312598	0	1	2	0.44357	0.4319	0.4141	0.40321535	0.042581387	17812.938	18292.518	7901.332	7375.824	1.878465516	4.864652431	3	High	6098	Unique	Used	69178	1	57.379	1	6.3972	3.0445	1	100	26/64	34.40284	6098	2393.18	MS2	798.398	141	Glioma_grade2_4.raw
189217897			gLFGGGAQSLDREELFGESSSGk	Unambiguous	N-Term(iTRAQ4plex); K23(iTRAQ4plex)	HCD	0.43967283	1	1	2	0.44695	0.5284	0.3646	0.43107206	0.152002158	12784.875	10814.529	5714.176	4661.842	11.80745936	14.34293016	3	High	5258	Unique	Used	75652	1	52.949	1	3.7349	-0.4704	1	51	26/88	16.54424	5258	2616.31	MS2	872.776	57	Glioma_grade2_5.raw
189491660	NP_001121646.1	UGT8	2-hydroxyacylsphingosine 1-beta-galactosyltransferase precursor [Homo sapiens]	2	3	3.097439051	17.10013455	24.285757	5.55	2.65E-05	541	61.416	9.5249	Enzyme: Glycosyltransferase	Metabolism,Energy pathways	Transferase activity	Endoplasmic reticulum	Nucleus																								
189491660			lIEWLPQNDLLGHSk	Unambiguous	N-Term(iTRAQ4plex); K15(iTRAQ4plex)	HCD	2.3014808	0	1	1	1.97515	2.5307	2.1226	2.7196043	14.84017088	16174.849	12623.873	31947.764	34331.94	17.43771275	5.087128986	3	High	5711	Unique	Used	82673	1	58.416	1	6.0392	-1.925	1	34	29/56	17.9849	5711	2051.15	MS2	684.388	141	Glioma_grade2_6.raw
189491660			lTAIELFDILDHYTk	Unambiguous	N-Term(iTRAQ4plex); K15(iTRAQ4plex)	HCD	3.097439	0	1	1	2.99762	3.1651	3.0334	3.2028382	3.211563334	12460.308	11801.056	37351.223	37796.87	3.842830599	0.838664038	3	High	7393	Unique	Used	83981	1	75.53	1	7.287	-2.9451	1	52	32/56	35.6199	7393	2080.15	MS2	694.055	146	Glioma_grade2_6.raw
189491660			lTAIELFDILDHYTk	Unambiguous	N-Term(iTRAQ4plex); K15(iTRAQ4plex)	HCD	3.1939769	0	1	1	3.35165	2.9921	3.4202	3.053221	6.649154533	4406.2583	4935.81	14768.231	15070.12	8.016417479	1.430828125	3	High	7834	Unique	Used	77863	1	75.714	1	6.7275	-1.8008	1	81	34/56	68.48839	7834	2080.15	MS2	694.056	133	Glioma_grade2_5.raw
189571583	NP_003250.3	ICAM5	intercellular adhesion molecule 5 precursor [Homo sapiens]	4	5	0.284076929	23.26058308	33.34052	6.6	3.65E-04	924	97.056	5.9478	Adhesion molecule	Cell communication,Signal transduction	Cell adhesion molecule activity	Plasma membrane	-																								
189571583			lFScEVDGkPQPSVk	Unambiguous	N-Term(iTRAQ4plex); C4(Methylthio); K9(iTRAQ4plex); K15(iTRAQ4plex)	HCD	0.2771332	0	1	1	0.2395	0.2731	0.2806	0.32005888	0.1188287	359206.28	314957.3	86028.234	100804.88	9.282243879	11.18502643	3	High	3968	Unique	Used	91432	1	45.088	1	4.249	-0.3721	1	14	23/56	24.13516	3968	2112.12	MS2	704.711	164	Glioma_grade2_7rep.raw
189571583			lFScEVDGkPQPSVk	Unambiguous	N-Term(iTRAQ4plex); C4(Methylthio); K9(iTRAQ4plex); K15(iTRAQ4plex)	HCD	0.26561713	0	1	1	0.24796	0.2509	0.2801	0.2834851	0.070668453	83942.516	82947.84	20814.46	23514.479	0.842879315	8.613794001	2	High	3962	Unique	Used	91428	1	45.016	1	5.4876	-1.1459	1	100	18/28	14.96156	3962	2112.12	MS2	1056.56	171	Glioma_grade2_7rep.raw
189571583			sGELGAVIEGLLR	Unambiguous	N-Term(iTRAQ4plex)	HCD	0.3926623	0	1	1	0.40924	0.4009	0.3843	0.37642092	0.038224571	54504.395	55644.113	22305.559	20945.607	1.463301394	4.446729974	2	High	5854	Unique	Used	88041	1	74.802	1	4.9657	1.0362	1	100	19/24	47.47738	5854	1457.85	MS2	729.427	238	Glioma_grade2_1rep.raw
189571583			aSLTLTLLR	Unambiguous	N-Term(iTRAQ4plex)	HCD	0.43331826	0	1	1	0.41737	0.4461	0.4214	0.4503621	0.038817493	49361.23	46181.555	20602.012	20798.422	4.706519188	0.670924575	2	High	4027	Unique	Used	86713	1	54.496	1	3.3016	-0.0769	1	100	14/16	43.2881	4027	1131.72	MS2	566.365	185	Glioma_grade2_1rep.raw
189571583			lLEVGSERPVScTLDGLFPASEAR	Unambiguous	N-Term(iTRAQ4plex); C12(Methylthio)	HCD	0.28407693	0	1	1	0.2388	0.2591	0.3071	0.333205	0.152040901	24194.24	22297.545	5777.5894	7429.654	5.769474742	17.69008182	3	High	7398	Unique	Used	62708	1	68.377	1	4.9355	3.3139	1	33	28/92	11.07955	7398	2736.39	MS2	912.802	88	Glioma_grade2_3.raw
190194386	NP_064508.3	TM9SF3	transmembrane 9 superfamily member 3 precursor [Homo sapiens]	2	4	2.591403246	34.33920801	40.27687	10.53	3.09E-04	589	67.843	7.2104	Membrane transport protein	Transport	Auxiliary transport protein activity	Plasma membrane	-																								
190194386			yLDPSFFQHR	Unambiguous	N-Term(iTRAQ4plex)	HCD	3.3271687	0	1	1	3.11401	3.6614	3.0429	3.5777953	9.425014213	29700.62	25260.037	92488.14	90375.24	11.42623296	1.634056986	3	High	4470	Unique	Used	74958	1	46.356	1	4.7482	-1.801	1	16	21/36	0	4470	1453.73	MS2	485.249	118	Glioma_grade2_5.raw
190194386			yLDPSFFQHR	Unambiguous	N-Term(iTRAQ4plex)	HCD	3.212343	0	1	1	2.93946	3.5136	2.9603	3.5385246	10.28111069	19850.986	16607.426	58351.145	58765.785	12.58169594	0.500687229	3	High	5008	Unique	Used	68175	1	48.789	1	4.7748	0.4033	1	34	20/36	3.934569	5008	1453.74	MS2	485.25	130	Glioma_grade2_4.raw

190194386			sISHYHETLGEALQGVELEF SGLDIk	Unambiguous	N-Term(iTRAQ4plex); K26(iTRAQ4plex)	HCD	1.9704634	0	1	1	1.75648	1.9566	1.9829	2.2088263	9.368036394	4587.715	4118.487	8058.23	9097.021	7.622021651	8.563397415	3	High	6553	Unique	Used	93283	1	73.206	1	8.8203	-0.222	1	44	33/100	12.4937	6553	3160.64	MS2	1054.22	133	Glioma_grade 2_7rep.raw
190194386			sISHYHETLGEALQGVELEF SGLDIk	Unambiguous	N-Term(iTRAQ4plex); K26(iTRAQ4plex)	HCD	1.6083468	0	1	1	1.48078	1.6366	1.5828	1.7493366	6.937398549	2651.4968	2399.0862	3926.28	4196.8096	7.067748294	4.709865928	4	High	6527	Unique	Used	93268	1	72.865	1	6.474	1.2221	1	16	28/150	14.67182	6527	3160.64	MS2	790.917	137	Glioma_grade 2_7rep.raw
191252797	NP_001122103 .1	DCAKD	dephospho-CoA kinase domain-containing protein [Homo sapiens]	3	6	2.069394112	19.9421144	26.954086	14.29	1.05E-04	231	26.533	9.5835	Enzyme: Phosphoryla se	Biological_process unknown	Kinase activity	-	-																								
191252797			iNAQLPLTDk	Unambiguous	N-Term(iTRAQ4plex); K10(iTRAQ4plex)	HCD	2.0865746	0	1	1	1.90565	2.1503	2.0301	2.29073	7.867639892	105102.695	93142.67	200289.02	213364.72	8.531866337	4.470365064	2	High	3292	Unique	Used	50875	1	35.966	1	3.2309	1.7966	1	31	14/18	51.0448	3292	1400.84	MS2	700.923	150	Glioma_grade 2_2.raw
191252797			iNAQLPLTDk	Unambiguous	N-Term(iTRAQ4plex); K10(iTRAQ4plex)	HCD	2.717023	0	1	1	2.5565	2.7904	2.6498	2.89224	5.460026341	104061.25	95336.83	266032.2	275736.97	6.187719103	2.533296118	2	High	3257	Unique	Used	58879	1	35.797	1	3.7769	0.1409	1	30	15/18	11.78269	3257	1400.84	MS2	700.921	163	Glioma_grade 2_3.raw
191252797			qLLNAITHPEIR	Unambiguous	N-Term(iTRAQ4plex)	HCD	1.675157	0	1	1	1.53453	1.4437	1.9212	1.8074638	13.39653849	37240.324	39583.09	57146.277	71545	4.312710511	15.82303776	3	High	4371	Unique	Used	67587	1	43.786	1	5.8085	1.3185	1	25	28/44	28.75006	4371	1548.9	MS2	516.972	140	Glioma_grade 2_4.raw
191252797			qLLNAITHPEIR	Unambiguous	N-Term(iTRAQ4plex)	HCD	2.431088	0	1	1	2.1623	2.5721	2.3125	2.7508235	10.7261658	30520.238	25657.264	65993.84	70578.61	12.2420605	4.747548949	2	High	4402	Unique	Used	67617	1	44.007	1	5.0502	1.8	1	70	19/22	10.72475	4402	1548.9	MS2	774.954	247	Glioma_grade 2_4.raw
191252797			qVILLHTELER	Unambiguous	N-Term(iTRAQ4plex)	HCD	1.6473095	0	1	1	1.43888	1.6815	1.6181	1.8908768	11.25523843	20727.07	17736.746	29823.81	33538.004	10.99463651	8.289951307	3	High	3272	Unique	Used	66570	1	35.165	1	3.4367	-1.6279	1	43	23/40	22.23474	3272	1494.87	MS2	498.963	222	Glioma_grade 2_4.raw
191252797			qLLNAITHPEIR	Unambiguous	N-Term(iTRAQ4plex)	HCD	2.052214	0	1	1	2.09426	2.1233	1.9821	2.009582	3.27578429	9896.449	9761.03	20725.76	19615.592	0.974241847	3.891824553	3	High	3568	Unique	Used	74164	1	38.763	1	5.6509	-1.8734	1	69	26/44	12.40199	3568	1548.9	MS2	516.97	208	Glioma_grade 2_5.raw
192448443	NP_068758.3	FKBP10	peptidyl-prolyl cis-trans isomerase FKBP10 precursor [Homo sapiens]	2	2	4.443282604	24.56413975	24.817616	7.22	3.08E-04	582	64.204	5.6177	Unclassified	Protein folding	Isomerase activity	Endoplasmic reticulum	-																								
192448443			eDGLPTGYLFVWHk	Unambiguous	N-Term(iTRAQ4plex); K14(iTRAQ4plex)	HCD	3.671508	0	1	1	3.48239	3.5175	3.824	3.8625288	5.429280071	9993.311	9893.539	34800.645	38214.08	0.709508623	6.611441831	3	High	5726	Unique	Used	82686	1	58.547	1	5.972	-1.5943	1	25	27/52	30.31	5726	1950.03	MS2	650.683	234	Glioma_grade 2_6.raw
192448443			iIIPPFLAYGEk	Unambiguous	N-Term(iTRAQ4plex); K12(iTRAQ4plex)	HCD	5.2150574	0	1	1	4.30485	6.4858	4.3716	6.586385	23.35538821	1677.6884	1113.5486	7222.201	7334.2593	28.58281673	1.088687527	3	High	6949	Unique	Used	77139	1	67.256	1	4.3085	-6.2644	1	82	22/44	8.097734	6949	1648.98	MS2	550.332	160	Glioma_grade 2_5.raw
194018518	NP_001123475 .1	SYNPR	synaptoporin isoform 1 [Homo sapiens]	4	4	0.335022986	24.29671798	20.3635	14.74	6.41E-05	285	31.309	6.5366	Membrane transport protein	Transport	Auxiliary transport protein activity	Cytoplasmic vesicle	-																								
194018518			yLSDPMEk	Unambiguous	N-Term(iTRAQ4plex); K8(iTRAQ4plex)	HCD	0.47769502	0	1	2	0.46326	0.4742	0.4811	0.49247742	0.025643954	781088.94	762992.7	361843.4	375756.66	1.657421951	2.6676138	2	High	2565	Unique	Used	50194	1	30.686	1	3.3323	-5.8209	1	13	13/14	46.10839	2565	1270.65	MS2	635.83	155	Glioma_grade 2_2.raw
194018518			lHQVTFEVPTcEGk	Unambiguous	N-Term(iTRAQ4plex); C11(Methylthio); K14(iTRAQ4plex)	HCD	0.31874686	0	1	2	0.32798	0.3145	0.3231	0.30989128	0.025654557	168707.27	175923.73	55332.816	54517.23	2.961316772	1.049988438	3	High	4478	Unique	Used	81618	1	47.601	1	4.8344	1.9208	1	5	22/52	6.411048	4478	1921.98	MS2	641.331	108	Glioma_grade 2_6.raw
194018518			eVLLLMSAck	Unambiguous	N-Term(iTRAQ4plex); C9(Methylthio); K10(iTRAQ4plex)	HCD	0.2769591	0	1	2	0.27658	0.2802	0.2738	0.27734473	0.009507476	134848.67	133114.28	37296.234	36918.543	0.915349626	0.719716148	2	High	7205	Unique	Used	62544	1	66.615	1	4.0338	-4.1082	1	96	16/18	68.83447	7205	1440.78	MS2	720.894	241	Glioma_grade 2_3.raw
194018518			vLkEPLAFLR	Unambiguous	N-Term(iTRAQ4plex); K3(iTRAQ4plex)	HCD	0.35129914	1	1	1	0.36697	0.3399	0.3636	0.33678296	0.044515455	34031.836	36740.43	12488.687	12373.551	5.412473821	0.654916475	3	High	4672	Unique	Used	91971	1	52.202	1	3.3472	5.702	1	100	16/36	61.62521	4672	1473.95	MS2	491.988	51	Glioma_grade 2_7rep.raw
194688133	NP_001124160 .1	HMGB2	high mobility group protein B2 [Homo sapiens]	2	3	2.18075037	17.96232915	16.407472	11.96	1.59E-04	209	24.019	7.811	Transcription regulatory protein	Regulation of nucleobase, nucleoside, nucleotide and nucleic acid metabolism	Transcription regulator activity	Nucleus	Mitochondrion, Cytoplasm, Nucleolus																								
194688133			lGEMWSEQSAk	Unambiguous	N-Term(iTRAQ4plex); K11(iTRAQ4plex)	HCD	2.6639264	0	1	1	2.60611	2.5825	2.7461	2.721219	3.064798501	37141.465	37480.62	96794.76	101992.98	0.642755561	3.698114749	2	High	3249	Unique	Used	58872	1	35.728	1	4.2299	3.3136	1	100	16/20	54.88147	3249	1553.79	MS2	777.4	206	Glioma_grade 2_3.raw
194688133			kHPDSSVNFAEFSk	Unambiguous	N-Term(iTRAQ4plex); K1(iTRAQ4plex); K14(iTRAQ4plex)	HCD	2.1807504	1	1	1	2.06379	2.2668	2.1024	2.309214	5.518598377	24238.541	22067.506	50023.324	50958.598	6.630466948	1.309815806	3	High	2289	Unique	Used	85618	1	32.74	1	6.7491	-0.6769	1	33	23/52	10.03141	2289	2025.08	MS2	675.697	154	Glioma_grade 2_1rep.raw
194688133			kHPDSSVNFAEFSk	Unambiguous	N-Term(iTRAQ4plex); K1(iTRAQ4plex); K14(iTRAQ4plex)	HCD	1.8662243	1	1	1	1.82782	2.0785	1.6796	1.9098895	8.876965593	6602.9995	5806.789	12069.123	11090.325	9.073576779	5.976953364	3	High	2426	Unique	Used	95121	1	34.68	1	5.4285	1.8548	1	100	22/52	40.98367	2426	2025.08	MS2	675.698	146	Glioma_grade 2_8rep.raw
194688149	NP_001124164 .1	PPP3CA	serine/threonine-protein phosphatase 2B catalytic subunit alpha isoform isoform 3 [Homo sapiens]	3	6	0.477651656	18.52700591	37.880167	14.29	3.64E-04	469	52.638	6.2271	Serine/threo nine phosphatase	Cell communication,Sig nal transduction	Protein serine/threoni ne phosphatase activity	Cytoplasm	Nucleus																								
194688149			gLTPTGMLPSGVLSGGk	Unambiguous	N-Term(iTRAQ4plex); K17(iTRAQ4plex)	HCD	0.3622044	0	1	3	0.33691	0.3585	0.3656	0.3891227	0.05935044	127518.06	119822.6	42962.184	46625.69	4.400014094	5.783126264	2	High	5642	Unique	Used	61089	1	54.409	1	5.5047	2.2116	1	34	22/32	16.61076	5642	1860.05	MS2	930.531	139	Glioma_grade 2_3.raw
194688149			gLTPTGMLPSGVLSGGk	Unambiguous	N-Term(iTRAQ4plex); K17(iTRAQ4plex)	HCD	0.5292739	0	1	3	0.43273	0.5236	0.5339	0.6460961	0.163682083	119580.81	98821.48	51746.47	63848.17	13.44222445	14.80552063	2	High	5839	Unique	Used	53273	1	54.541	1	5.4523	0.1771	1	28	22/32	9.917717	5839	1860.05	MS2	930.529	171	Glioma_grade 2_2.raw
194688149			gLTPTGmLPSGVLSGGk	Unambiguous	N-Term(iTRAQ4plex); M7(Oxidation); K17(iTRAQ4plex)	HCD	0.42602938	0	1	3	0.38579	0.4567	0.4001	0.4736691	0.099469337	37593.562	31755.9	14503.12	15041.789	11.90449145	2.578423936	2	High	4434	Unique	Used	59974	1	44.884	1	4.9589	1.3245	1	81	19/32	11.67469	4434	1876.05	MS2	938.528	199	Glioma_grade 2_3.raw
194688149			nLLDIDAPVTVcGDIHGQFF DLmk	Unambiguous	N-Term(iTRAQ4plex); C12(Methylthio); M23(Oxidation); K24(iTRAQ4plex)	HCD	0.4259927	0	1	3	0.42095	0.4315	0.4206	0.43116143	0.01435057	24667.441	24062.205	10383.779	10374.695	1.756493285	0.061886611	3	High	7829	Unique	Used	77858	1	75.681	1	7.0394	-3.3999	1	26	30/92	10.52534	7829	3011.48	MS2	1004.5	68	Glioma_grade 2_5.raw
194688149			gLTPTGmLPSGVLSGGk	Unambiguous	N-Term(iTRAQ4plex); M7(Oxidation); K17(iTRAQ4plex)	HCD	0.5587707	0	1	3	0.52234	0.5716	0.5471	0.59863186	0.058431724	21828.998	19949.225	11402.206	11942.242	6.363124805	3.271554056	2	High	4529	Unique	Used	52042	1	44.97	1	4.669	-0.6926	1	100	19/32	28.52026	4529	1876.04	MS2	938.526	218	Glioma_grade 2_2.raw
194688149			fkEPPAYGPMcDILWSDPLE DFGNEk	Unambiguous	N-Term(iTRAQ4plex); K2(iTRAQ4plex); C11(Methylthio); K26(iTRAQ4plex)	HCD	0.5871338	1	1	3	0.63301	0.4467	0.7861	0.5547594	0.235967073	6267.9907	8882.105	3967.7024	4927.431	24.40193098	15.25846934	3	High	6826	Unique	Used	93466	1	76.362	1	6.2681	-3.9708	1	34	30/100	29.63373	6826	3476.64	MS2	1159.55	72	Glioma_grade 2_7rep.raw
208022622	NP_001129064 .1	RPS27A	ubiquitin-40S ribosomal protein S27a precursor [Homo sapiens]	4	13	0.490942895	16.27898921	60.48774	42.31	1.00E-04	156	17.953	9.6421	Ubiquitin proteasome system protein	Protein metabolism	Ubiquitin-specific protease activity	Ribosome	Cytoplasm																								
208022622			eSTLHLVLR	Unambiguous	N-Term(iTRAQ4plex)	HCD	0.4909429	0	1	4	0.49437	0.545	0.4419	0.48716336	0.085862014	3853387.2	3495113.8	1905003	1702691.4	6.894944987	7.930599902	2	High	2320	Unique	Used	65681	1	28.03	1	3.3027	12.351	1	1	16/16	4.354391	2320	1211.74	MS2	606.373	130	Glioma_grade 2_4.raw
208022622			eSTLHLVLR	Unambiguous	N-Term(iTRAQ4plex)	HCD	0.48729306	0	1	4	0.45951	0.4781	0.4961	0.5162009	0.049820467	2465913.2	2369967.2	1133111.9	1223379.2	2.805862082	5.417259581	2	High	1970	Unique	Used	57681	1	26.143	1	3.4483	13.258	1	2	16/16	5.972155	1970	1211.74	MS2	606.373	121	Glioma_grade 2_3.raw
208022622			eSTLHLVLR	Unambiguous	N-Term(iTRAQ4plex)	HCD	0.45866236	0	1	4	0.43817	0.448	0.4691	0.4796106	0.041412261	2324152.8	2273347.2	1018378.9	1090321.4	1.562805189	4.824870524	2	High	2371	Unique	Used	65728	1	28.415	1	3.4461	-2.7599	1	2	16/16	6.062802	2371	1211.72	MS2	606.363	190	Glioma_grade 2_4.raw
208022622			tLSDYNIQk	Unambiguous	N-Term(iTRAQ4plex); K9(iTRAQ4plex)	HCD	0.53081244	0	1	4	0.53134	0.5339	0.5278	0.5302835	0.00475112	2224515.5	2213998	1181971.5	1174046.5	0.335111995	0.475702753	2	High	2055	Unique	Used	49711	1	27.144	1	4.339	-0.393	1	5	16/16	5.84075	2055	1369.76	MS2	685.382	240	Glioma_grade 2_2.raw
208022622			eSTLHLVLR	Unambiguous	N-Term(iTRAQ4plex)	HCD	0.4792418	0	1	4	0.45558	0.472	0.4862	0.5037613	0.04278336	1503436.9	1451070.4	684930.3	730993.06	2.506591015	4.600713693	2	High	2250	Unique	Used	65615	1	27.53	1	3.4542	1.0682	1	4	16/16	7.087992	2250	1211.72	MS2	606.366	168	Glioma_grade 2_4.raw
208022622			tLSDYNIQk	Unambiguous	N-Term(iTRAQ4plex); K9(iTRAQ4plex)	HCD	0.48303112	0	1	4	0.43728	0.4716	0.4937	0.5323686	0.082432765	791196.2	733672.6	345974.8	390584.28	5.334928177	8.565142069	2	High	2162	Unique	Used	49811	1	27.928	1	3.9849	-1.1059	1	13	16/16	25.9109	2162	1369.76	MS2	685.381	239	Glioma_grade 2_2.raw
208022622			eSTLHLVLR	Unambiguous	N-Term(iTRAQ4plex)	HCD	0.6131474	0	1	4	0.60688	0.6129	0.6134	0.61947185	0.008389309	467518.66	462959.28	283729.88	286790.22	0.692970435	0.758601552	2	High	1958	Unique	Used	57670	1	26.055	1	3.6852	0.1616	1	40	15/16	35.01193	1958	1211.72	MS2	606.365	257	Glioma_grade 2_3.raw
208022622			eSTLHLVLR	Unambiguous	N-Term(iTRAQ4plex)	HCD	0.6506376	0	1	4	0.60531	0.6149	0.6858	0.69668955	0.072541494	179456.23	176643.67	108626.2	123065.79	1.11697883	8.813711692	2	High	2233	Unique	Used	65600	1	27.388	1	3.0707	12.15	1	64	15/16	57.45452	2233	1211.74	MS2	606.372	205	Glioma_grade 2_4.raw
208022622			eSTLHLVLR	Unambiguous	N-Term(iTRAQ4plex)	HCD	0.45441288	0	1	4	0.34877	0.3927	0.5092	0.5733549	0.22688647	160802	142820.55	56083.13	81886.86	8.37540244	26.44921908	3	High	2468	Unique	Used	65818	1	29.153	1	3.2654	3.3742	1	5	18/32	5.423175	2468	1211.73	MS2	404.581	170	Glioma_grade 2_4.raw
208022622			iQDkEGIPPDQQR	Unambiguous	N-Term(iTRAQ4plex); K4(iTRAQ4plex)	HCD	0.36636728	1	1	4	0.34719	0.351	0.3815	0.38575497	0.054798422	61744.94	61070.586	21437.309	23558.281	0.776514667	6.666225219	2	High	1133	Unique	Used	71916	1	20.4	1	4.3363	2.1966	1	50	20/24	0	1133	1811.99	MS2	906.498	167	Glioma_grade 2_5.raw
208022622			tITLEVEPSDTIENVk	Unambiguous	N-Term(iTRAQ4plex); K16(iTRAQ4plex)	HCD	0.6455992	0	1	4	0.59065	0.6766	0.6185	0.70854753	0.082758681	46498.508	40589.65	27464.348	28759.695	9.595319632	3.258209829	2	High	3926	Unique	Used	86647	1	53.283	1	5.4986	3.2387	1	81	19/30	14.78027	3926	2076.14	MS2	1038.57	211	Glioma_grade 2_1rep.raw
208022622			tITLEVEPSDTIENVk	Unambiguous	N-Term(iTRAQ4plex); K16(iTRAQ4plex)	HCD	0.6040265	0	1	4	0.53525	0.6722	0.5498	0.6904028	0.131868217	38102.21	30340.492	20394.049	20947.16	16.0378339	1.89210015	3	High	3920	Unique	Used	86643	1	53.214	1	4.237	-1.6186	1	32	18/60	42.27206	3920	2076.13	MS2	692.714	217	Glioma_grade 2_1rep.raw
208022622			iQDkEGIPPDQQR	Unambiguous	N-Term(iTRAQ4plex); K4(iTRAQ4plex)	HCD	0.49448624	1	1	4	0.49111	0.5121	0.4776	0.49800646	0.028975965	15268.475	14643.834	7498.501	7292.724	2.953218268	1.967468037	3	High	1108	Unique	Used	71893	1	20.196	1	3.8173	2.3576	1	100	17/48	48.40885	1108	1811.99	MS2	604.668	150	Glioma_grade 2_5.raw
209413738	NP_001129243 .1	RPN2	dolichyl-diphosphooligosaccharide--protein glycosyltransferase subunit 2 isoform 2 precursor [Homo sapiens]	7	7	2.058004379	26.11512648	105.41187	28.29	7.16E-04	615	67.681	6.062	Enzyme: Glycosyltran sferase	Protein metabolism	Transferase activity	Endoplasmic reticulum	Mitochondrion																								
209413738			sIVEEIEDLVAR	Unambiguous	N-Term(iTRAQ4plex)	HCD	2.0580044	0	1	2	2.01368	2.3095	1.8387	2.1088407	9.48889765	76380.13	66597.555	153805.06	140443.64	9.676090531	6.421745067	2	High	6060	Unique	Used	88210	1	76.711	1	3.7134	-2.2153	1	24	20/22	34.49464	6060	1516.83	MS2	758.919	245	Glioma_grade 2_1rep.raw
209413738			nPILWNVADVVIk	Unambiguous	N-Term(iTRAQ4plex); K13(iTRAQ4plex)	HCD	1.6335841	0	1	2	1.5711	1.7353	1.5415	1.7025982	5.838288265	34643.113	31365.342	54427.793	53402.574	7.022537041	1.344592117	2	High	7219	Unique	Used	62556	1	66.74	1	4.7748	2.4246	1	100	19/24	50.71413	7219	1769.06	MS2	885.034	75	Glioma_grade 2_3.raw
209413738			fPEEEAPSTVLSQNLFTPk	Unambiguous	N-Term(iTRAQ4plex); K19(iTRAQ4plex)	HCD	1.7747167	0	1	2	1.96673	1.6116	1.9737	1.6173697	11.45996403	16319.033	19914.494	32095.146	32209.098	14.03327286	0.250609375	2	High	6836	Unique	Used	54209	1	61.895	1	5.3836	0.8924	1	100	20/36	29.86013	6836	2422.28	MS2	1211.64	157	Glioma_grade 2_2.raw
209413738			yHVPVVVVPEGSASDTHEQ AILR	Unambiguous	N-Term(iTRAQ4plex)	HCD	2.620045	0	1	2	2.71759	2.4169	2.8485	2.5332901	7.288407319	12960.875	14573.313	35222.38	36918.434	8.281819272	3.324862081	3	High	4094	Unique	Used	74615	1	43.39	1	6.2543	0.1489	1	43	31/88	11.90972	4094	2647.4	MS2	883.137	96	Glioma_grade 2_5.raw
209413738			eETVLATVQALQTASHLSQ QADLR	Unambiguous	N-Term(iTRAQ4plex)	HCD	3.0992968	0	1	2	2.8203	2.3868	3.9412	3.3354046	21.48021689	6388.402	7548.754	18017.232	25178.148	11.77417786	23.44478629	3	High	7556	Unique	Used	54869	1	67.556	1	8.5316	1.9757	1	17	45/92	22.88482	7556	2753.46	MS2	918.492	125	Glioma_grade 2_2.raw
209413738			nFESLSEAFSVASAAAVLSH NR	Unambiguous	N-Term(iTRAQ4plex)	HCD	1.5969037	0	1	2	1.52241	1.5132	1.6811	1.6709486	5.731030771	5483.103	5516.442	8347.522	9217.691	0.428640148	7.005920175	3	High	8037	Unique	Used	55308	1	71.434	1	7.6691	1.9402	1	37	37/84	11.03634	8037	2451.24	MS2	817.753	150	Glioma_grade 2_2.raw
209413738			aSLDRPFTNLESAFYSIVGL SSLGAQVPDAk	Unambiguous	N-Term(iTRAQ4plex); K31(iTRAQ4plex)	HCD	2.0775867	0	1	2	1.85335	2.5754	1.7194	2.389175	19.32942839	2439.7375	1755.7542	4521.694	4194.8037	23.05566376	5.303651898	3	High	7540	Unique	Used	84109	1	76.744	1	5.7498	-3.498	1	17	26/120	9.914374	7540	3541.87	MS2	1181.29	100	Glioma_grade 2_6.raw
209447070	NP_001129277 .1	SYT1	synaptotagmin-1 [Homo sapiens]	8	34	0.267372161	33.32406448	229.24812	38.15	6.25E-05	422	47.543	8.1187	Calcium binding protein	Cell communication,Sig nal transduction	Calcium ion binding	Cytoplasmic vesicle	Chromaffin granule																								
209447070			vPYSELGGk	Unambiguous	N-Term(iTRAQ4plex); K9(iTRAQ4plex)	HCD	0.22122647	0	1	1	0.20543	0.2164	0.2258	0.23786622	0.06224556	3022475.5	2868688.5	620917.3	682364.1	3.691760425	6.667700308	2	High	1986	Unique	Used	57696	1	26.251	1	3.1423	12.298	1	3	13/16	11.21424	1986	1237.72	MS2	619.363	236	Glioma_grade 2_3.raw
209447070			vPYSELGGk	Unambiguous	N-Term(iTRAQ4plex); K9(iTRAQ4plex)	HCD	0.26505512	0	1	1	0.23018	0.2743	0.2573	0.3066044	0.119773755	2635518.5	2211931.2	606653.56	678187.9	12.35789831	7.873721152	2	High	2054	Unique	Used	49710	1	27.139	1	3.1812	3.225	1	4	13/16	26.47077	2054	1237.71	MS2	619.357	257	Glioma_grade 2_2.raw
209447070			vPYSELGGk	Unambiguous	N-Term(iTRAQ4plex); K9(iTRAQ4plex)	HCD	0.24726386	0	1	1	0.25596	0.2288	0.268	0.23949091	0.070024963	1978660.1	2213983	506460.3	530228.8	7.937638115	3.242412316	2	High	1924	Unique	Used	57637	1	25.833	1	3.1834	-3.383	1	5	13/16	24.30763	1924	1237.7	MS2	619.353	267	Glioma_grade 2_3.raw

209447070			ITVVILEAk	Unambiguous	N-Term(iTRAQ4plex); K9(iTRAQ4plex)	HCD	0.2060164	0	1	1	0.18754	0.204	0.2078	0.22612122	0.07673323	1265547.6	1163212.2	237337.42	263026.97	5.958765893	7.260810471	2	High	5312	Unique	Used	52776	1	50.724	1	4.0325	5.9683	1	7	15/16	52.99374	5312	1273.84	MS2	637.424	57	Glioma_grade2_2.raw
209447070			vPMNTVDFGHVTEEWR	Unambiguous	N-Term(iTRAQ4plex)	HCD	0.23900567	0	1	1	0.22235	0.2231	0.2549	0.25571722	0.078748546	894505.2	891596.44	198891.86	227996.56	0.23031208	9.641925042	3	High	5677	Unique	Used	61121	1	54.695	1	4.8063	1.7903	1	1	28/60	17.64236	5677	2061	MS2	687.672	107	Glioma_grade2_3.raw
209447070			vPMNTVDFGHVTEEWR	Unambiguous	N-Term(iTRAQ4plex)	HCD	0.23561811	0	1	1	0.20208	0.2115	0.2586	0.27071762	0.144210946	822555.7	785864.1	166225.58	212747.27	3.226132776	17.36050615	3	High	5841	Unique	Used	68938	1	55.392	1	5.0657	0.6354	1	1	27/60	5.026716	5841	2061	MS2	687.671	112	Glioma_grade2_4.raw
209447070			hDIIGEFk	Unambiguous	N-Term(iTRAQ4plex); K8(iTRAQ4plex)	HCD	0.23804432	0	1	1	0.2232	0.2424	0.2341	0.25416282	0.054889809	740187.2	681673.2	165209.8	173255.97	5.819930873	3.36193605	3	High	1984	Unique	Used	79431	1	26.522	1	3.4363	-1.0474	1	6	18/28	57.24944	1984	1246.7	MS2	416.239	227	Glioma_grade2_6.raw
209447070			ITVVILEAk	Unambiguous	N-Term(iTRAQ4plex); K9(iTRAQ4plex)	HCD	0.3012528	0	1	1	0.29772	0.2949	0.3077	0.3047507	0.019711225	732253.2	739219.75	218007.61	225277.73	0.669546083	2.319386945	2	High	5170	Unique	Used	60655	1	50.678	1	3.934	-4.1896	1	14	15/16	51.97832	5170	1273.83	MS2	637.418	72	Glioma_grade2_3.raw
209447070			ITVVILEAk	Unambiguous	N-Term(iTRAQ4plex); K9(iTRAQ4plex)	HCD	0.21772605	0	1	1	0.19718	0.2244	0.2118	0.24110793	0.085354762	727652.3	639342.9	143479.84	154150.62	9.135975837	5.07030154	2	High	5230	Unique	Used	52699	1	50.111	1	4.3151	-5.3395	1	14	16/16	53.96898	5230	1273.83	MS2	637.417	70	Glioma_grade2_2.raw
209447070			ITVVILEAk	Unambiguous	N-Term(iTRAQ4plex); K9(iTRAQ4plex)	HCD	0.22491544	0	1	1	0.20289	0.2172	0.2321	0.24849606	0.086993868	683213	638098.3	138618.4	158564.92	4.828674409	9.491999452	2	High	5216	Unique	Used	52686	1	50.002	1	4.1708	6.3516	1	14	15/16	29.11159	5216	1273.84	MS2	637.424	58	Glioma_grade2_2.raw
209447070			tLVMAVYDFDR	Unambiguous	N-Term(iTRAQ4plex)	HCD	0.3643712	0	1	1	0.32785	0.3561	0.372	0.4040413	0.08711592	496985.75	457575.53	162935.23	184879.42	5.838752188	8.922502578	2	High	4788	Unique	Used	87247	1	63.143	1	4.0444	1.1467	1	21	16/20	32.08223	4788	1473.75	MS2	737.381	195	Glioma_grade2_1rep.raw
209447070			tLNPVFNEQFTFk	Unambiguous	N-Term(iTRAQ4plex); K13(iTRAQ4plex)	HCD	0.2379546	0	1	1	0.22997	0.2206	0.256	0.24561802	0.066439346	385114.34	401452.88	88563.234	98604.06	2.937598246	7.586727362	2	High	6361	Unique	Used	53767	1	58.32	1	5.8869	0.9541	1	21	21/24	8.422	6361	1873.01	MS2	937.009	188	Glioma_grade2_2.raw
209447070			vPYSELGGk	Unambiguous	N-Term(iTRAQ4plex); K9(iTRAQ4plex)	HCD	0.34375665	0	1	1	0.30763	0.3383	0.3487	0.3834808	0.090780599	375272.6	341249.94	115446.57	130862.81	6.715114252	8.851408363	2	High	2155	Unique	Used	89964	1	27.907	1	3.0366	7.2686	1	24	16-Oct	53.01279	2155	1237.71	MS2	619.36	310	Glioma_grade2_7rep.raw
209447070			tLNPVFNEQFTFk	Unambiguous	N-Term(iTRAQ4plex); K13(iTRAQ4plex)	HCD	0.26626688	0	1	1	0.24884	0.3084	0.2323	0.28786644	0.129694552	327481.3	264250.88	81489.72	76068.96	15.11178884	4.865560127	2	High	6457	Unique	Used	53856	1	59.046	1	5.4694	0.4327	1	14	21/24	0	6457	1873.01	MS2	937.009	189	Glioma_grade2_2.raw
209447070			vPMNTVDFGHVTEEWR	Unambiguous	N-Term(iTRAQ4plex)	HCD	0.20669605	0	1	1	0.19568	0.1878	0.2264	0.21726799	0.087414733	312684.88	325853.28	61185.824	70797.484	2.916494431	10.29898412	2	High	5789	Unique	Used	68890	1	54.977	1	5.2299	0.4642	1	16	19/30	6.595182	5789	2061	MS2	1031	129	Glioma_grade2_4.raw
209447070			tLNPVFNEQFTFk	Unambiguous	N-Term(iTRAQ4plex); K13(iTRAQ4plex)	HCD	0.2847901	0	1	1	0.28574	0.2696	0.3009	0.2838945	0.044922633	304560.38	322800.7	87024.875	91641.35	4.111780081	3.654121844	2	High	6197	Unique	Used	61608	1	58.694	1	6.3237	-0.0887	1	66	20/24	6.980914	6197	1873.01	MS2	937.009	201	Glioma_grade2_3.raw
209447070			vPMNTVDFGHVTEEWR	Unambiguous	N-Term(iTRAQ4plex)	HCD	0.32680058	0	1	1	0.26355	0.301	0.3494	0.3990245	0.179278016	301265.4	263825.38	79399.21	105272.79	9.369854532	19.81392292	3	High	5768	Unique	Used	61206	1	55.392	1	5.1056	1.435	1	4	27/60	10.20189	5768	2061	MS2	687.672	115	Glioma_grade2_3.raw
209447070			tLNPVFNEQFTFk	Unambiguous	N-Term(iTRAQ4plex); K13(iTRAQ4plex)	HCD	0.2472497	0	1	1	0.22665	0.2531	0.242	0.2702435	0.074036503	273316.97	244800.6	61948.65	66155.766	7.783607339	4.644461675	2	High	6296	Unique	Used	61701	1	59.455	1	5.5508	1.4103	1	20	20/24	2.644146	6296	1873.01	MS2	937.01	184	Glioma_grade2_3.raw
209447070			vPmNTVDFGHVTEEWR	Unambiguous	N-Term(iTRAQ4plex); M3(Oxidation)	HCD	0.28292212	0	1	1	0.27798	0.2952	0.2714	0.28817034	0.037268198	238811.12	224913.64	66384.55	64813.438	4.238290985	1.693538089	2	High	5134	Unique	Used	68289	1	49.822	1	4.0174	0.6755	1	15	18/30	10.76118	5134	2076.99	MS2	1039	112	Glioma_grade2_4.raw
209447070			vPMNTVDFGHVTEEWR	Unambiguous	N-Term(iTRAQ4plex)	HCD	0.25469473	0	1	1	0.24567	0.2292	0.2821	0.26311588	0.089246932	193407.38	207342.11	47513.785	54555.004	4.917457076	9.755957235	3	High	5749	Unique	Used	68853	1	54.664	1	5.6413	1.435	1	9	27/60	22.67973	5749	2061	MS2	687.672	116	Glioma_grade2_4.raw
209447070			ITVVILEAk	Unambiguous	N-Term(iTRAQ4plex); K9(iTRAQ4plex)	HCD	0.26847744	0	1	1	0.28892	0.2441	0.2973	0.2512057	0.098393282	184006.56	217754.56	53162.605	54701.19	11.87941713	2.017254978	2	High	5076	Unique	Used	60566	1	49.976	1	4.2724	-4.2854	1	51	15/16	45.76937	5076	1273.83	MS2	637.418	72	Glioma_grade2_3.raw
209447070			tLNPVFNEQFTFk	Unambiguous	N-Term(iTRAQ4plex); K13(iTRAQ4plex)	HCD	0.3514498	0	1	1	0.32734	0.3588	0.3447	0.37787443	0.060817736	174965.88	159623.42	57273.727	60317.61	6.484820349	3.660729379	2	High	4520	Unique	Used	87045	1	60.287	1	5.4005	1.0192	1	45	19/24	19.6953	4520	1873.01	MS2	937.01	208	Glioma_grade2_1rep.raw
209447070			vPmNTVDFGHVTEEWR	Unambiguous	N-Term(iTRAQ4plex); M3(Oxidation)	HCD	0.25377816	0	1	1	0.25167	0.2481	0.2595	0.2558539	0.019596759	153195.25	155402.45	38555.035	39760.32	1.011495606	2.176495776	2	High	4981	Unique	Used	60476	1	49.27	1	4.1891	0.558	1	24	19/30	1.493221	4981	2076.99	MS2	1039	114	Glioma_grade2_3.raw
209447070			vPMNTVDFGHVTEEWR	Unambiguous	N-Term(iTRAQ4plex)	HCD	0.22696216	0	1	1	0.21102	0.2273	0.2267	0.24412982	0.059501007	145065.08	134699.3	30611.812	32884.117	5.239918913	5.060993042	2	High	5743	Unique	Used	61183	1	55.191	1	5.0665	-0.0096	1	28	19/30	0	5743	2061	MS2	1031	129	Glioma_grade2_3.raw
209447070			tLVMAVYDFDR	Unambiguous	N-Term(iTRAQ4plex)	HCD	0.3829476	0	1	1	0.34041	0.3924	0.3747	0.43198344	0.099000305	120048.586	104142.24	40865.613	44987.727	10.03384957	6.790125491	2	High	6247	Unique	Used	76533	1	61.086	1	3.9526	-0.0958	1	58	17/20	36.1033	6247	1473.75	MS2	737.38	215	Glioma_grade2_5.raw
209447070			kTLNPVFNEQFTFk	Unambiguous	N-Term(iTRAQ4plex); K1(iTRAQ4plex); K14(iTRAQ4plex)	HCD	0.49465168	1	1	1	0.47688	0.5315	0.4616	0.5144578	0.065317277	97229.945	87238.63	46367.094	44880.598	7.659761658	2.303864084	3	High	4509	Unique	Used	91848	1	50.537	1	6.7476	-1.8816	1	15	25/52	29.05746	4509	2145.2	MS2	715.739	129	Glioma_grade2_7rep.raw
209447070			kTLNPVFNEQFTFk	Unambiguous	N-Term(iTRAQ4plex); K1(iTRAQ4plex); K14(iTRAQ4plex)	HCD	0.37918356	1	1	1	0.35193	0.3716	0.3864	0.40795532	0.062363627	93277.914	88347.875	32827.527	36041.984	3.838732404	6.600785484	2	High	4506	Unique	Used	91846	1	50.497	1	4.8524	1.4536	1	67	19/26	0	4506	2145.21	MS2	1073.11	120	Glioma_grade2_7rep.raw
209447070			vPMNTVDFGHVTEEWR	Unambiguous	N-Term(iTRAQ4plex)	HCD	0.25414887	0	1	1	0.25334	0.2567	0.2516	0.25496617	0.008520513	71179.03	70250.61	18032.652	17911.53	0.928365621	0.476551045	2	High	5660	Unique	Used	61106	1	54.544	1	5.8606	-0.0096	1	100	19/30	0	5660	2061	MS2	1031	131	Glioma_grade2_3.raw
209447070			tLNPVFNEQFTFk	Unambiguous	N-Term(iTRAQ4plex); K13(iTRAQ4plex)	HCD	0.33759257	0	1	1	0.31407	0.349	0.3274	0.3637268	0.065319178	61299.293	55169.594	19252.371	20066.66	7.442934918	2.928807039	3	High	6199	Unique	Used	61610	1	58.708	1	5.9096	0.1648	1	53	22/48	17.02938	6199	1873.01	MS2	625.008	195	Glioma_grade2_3.raw
209447070			vPmNTVDFGHVTEEWR	Unambiguous	N-Term(iTRAQ4plex); M3(Oxidation)	HCD	0.33465567	0	1	1	0.32061	0.3192	0.3502	0.34863457	0.050970032	53171.84	53408.605	17047.662	18620.088	0.314162954	6.234612991	3	High	5102	Unique	Used	68260	1	49.561	1	5.7311	2.6967	1	28	26/60	21.55725	5102	2077	MS2	693.004	111	Glioma_grade2_4.raw
209447070			vPmNTVDFGHVTEEWR	Unambiguous	N-Term(iTRAQ4plex); M3(Oxidation)	HCD	0.37279016	0	1	1	0.35024	0.3849	0.3617	0.39757183	0.057605213	49973.465	45468.754	17502.822	18077.096	6.674848361	2.282596827	3	High	4956	Unique	Used	60454	1	49.046	1	5.5996	-0.3889	1	37	26/60	16.77491	4956	2076.99	MS2	693.002	105	Glioma_grade2_3.raw
209447070			vPMNTVDFGHVTEEWR	Unambiguous	N-Term(iTRAQ4plex)	HCD	0.6119274	0	1	1	0.60102	0.636	0.5892	0.6234713	0.034614637	27011.502	25525.87	16234.408	15914.647	3.999059799	1.406605398	3	High	5942	Unique	Used	69032	1	56.181	1	6.1761	0.9019	1	66	22/60	47.34834	5942	2061	MS2	687.672	123	Glioma_grade2_4.raw
209447070			tLNPVFNEQFTFk	Unambiguous	N-Term(iTRAQ4plex); K13(iTRAQ4plex)	HCD	0.54585135	0	1	1	0.52212	0.6031	0.4963	0.5732674	0.088115142	23529.426	20370.578	12285.087	11677.789	10.17604846	3.58408176	3	High	6363	Unique	Used	53769	1	58.332	1	4.1835	-1.9859	1	21	24/48	22.31991	6363	1873.01	MS2	625.007	205	Glioma_grade2_2.raw
209447070			dQALkDDDAETGLTDGEEk	Unambiguous	N-Term(iTRAQ4plex); K5(iTRAQ4plex); K19(iTRAQ4plex)	HCD	0.543459	1	1	1	0.49166	0.6481	0.4641	0.6117482	0.162058042	17098.531	12970.486	8406.605	7934.6724	19.41512496	4.084218553	3	High	1598	Unique	Used	72343	1	23.926	1	5.1917	4.21	1	42	25/72	11.09576	1598	2482.23	MS2	828.08	100	Glioma_grade2_5.raw
209693439	NP_003078.2	SNCG	gamma-synuclein [Homo sapiens]	2	3	0.467280447	29.48317108	22.230143	22.83	3.42E-04	127	13.323	4.856	Chaperone	Protein metabolism	Chaperone activity	Cytoplasm	Nucleus, Centrosome																								
209693439			ekEEVAEEAQSGGD	Unambiguous	N-Term(iTRAQ4plex); K2(iTRAQ4plex)	HCD	0.49433717	1	1	1	0.48169	0.5748	0.4269	0.50943255	0.123457278	160970.6	134894.16	77537.47	68719.48	12.46436213	8.526446812	2	High	1269	Unique	Used	48965	1	21.784	1	3.4129	2.1884	1	6	13/26	7.798946	1269	1765.84	MS2	883.422	78	Glioma_grade2_2.raw
209693439			tkENVVQSVTSVAEk	Unambiguous	N-Term(iTRAQ4plex); K2(iTRAQ4plex); K15(iTRAQ4plex)	HCD	0.27211305	1	1	1	0.25178	0.2812	0.264	0.29482016	0.069356964	78709.57	70474.234	19817.637	20777.225	7.80682859	3.342941193	2	High	2871	Unique	Used	80221	1	33.778	1	6.8531	-5.3339	1	100	20/28	24.54221	2871	2051.16	MS2	1026.08	135	Glioma_grade2_6.raw
209693439			ekEEVAEEAQSGGD	Unambiguous	N-Term(iTRAQ4plex); K2(iTRAQ4plex)	HCD	0.46728045	1	1	1	0.4301	0.4603	0.4738	0.5070747	0.068234048	11109.437	10380.872	4778.123	5263.878	4.794470401	6.840880707	3	High	1262	Unique	Used	57011	1	21.144	1	5.6472	0.0724	1	30	20/52	15.87924	1262	1765.83	MS2	589.283	71	Glioma_grade2_3.raw
209862831	NP_001129487.1	ANXA2	annexin A2 isoform 2 [Homo sapiens]	7	10	2.779760361	26.34113967	81.540224	37.76	6.82E-05	339	38.58	7.7524	Calcium binding protein	Cell communication,Signal transduction	Calcium ion binding	Nucleus	Endosome, Nucleolus, Cytoplasm, Caveola, Cytoskeleton, Apical membrane, Extracellular, Plasma membrane																								
209862831			IMVALAk	Unambiguous	N-Term(iTRAQ4plex); K7(iTRAQ4plex)	HCD	1.9458332	0	1	2	1.88382	2.0175	1.8789	2.012243	3.959348293	283123.28	264364.1	533354.3	531964.8	4.845680055	0.184456445	2	High	3394	Unique	Used	66683	1	36.111	1	2.9721	0.4948	1	26	12-Nov	38.48636	3394	1033.67	MS2	517.338	240	Glioma_grade2_4.raw
209862831			IMVALAk	Unambiguous	N-Term(iTRAQ4plex); K7(iTRAQ4plex)	HCD	2.3783088	0	1	2	2.322	2.3383	2.418	2.435019	2.37337762	146894.03	145866.78	341087.28	355188.38	0.496224506	2.864090763	2	High	3058	Unique	Used	58693	1	34.298	1	3.0076	-3.1661	1	38	12-Nov	53.32486	3058	1033.66	MS2	517.336	223	Glioma_grade2_3.raw
209862831			dLYDAGVk	Unambiguous	N-Term(iTRAQ4plex); K8(iTRAQ4plex)	HCD	1.7798322	0	1	2	1.74862	1.7503	1.8094	1.8110722	1.972144419	109046.21	108943.51	190680.58	197304.56	0.066626873	2.414453902	2	High	1575	Unique	Used	49256	1	23.84	1	2.9486	-2.1303	1	16	14/14	30.65975	1575	1168.64	MS2	584.825	255	Glioma_grade2_2.raw
209862831			tPAQYDASELk	Unambiguous	N-Term(iTRAQ4plex); K11(iTRAQ4plex)	HCD	3.0519314	0	1	2	2.97877	3.0823	3.0226	3.1276352	2.144616415	97263.19	93997.46	289724.62	293989.75	2.414735941	1.033348672	2	High	1823	Unique	Used	49491	1	25.549	1	3.8651	-0.038	1	23	17/20	19.81329	1823	1510.8	MS2	755.903	216	Glioma_grade2_2.raw
209862831			gLGTDEDSLIEIIcSR	Unambiguous	N-Term(iTRAQ4plex); C14(Methylthio)	HCD	3.4428062	0	1	2	3.27163	3.5924	3.3066	3.6307666	5.430772263	41462.06	37760.004	135648.45	137097.77	6.608635953	0.751485392	2	High	6035	Unique	Used	88187	1	76.521	1	6.6465	0.1221	1	41	26/30	26.17689	6035	1910.93	MS2	955.97	180	Glioma_grade2_1rep.raw
209862831			lSLEGDHSTPPSAYGSVk	Unambiguous	N-Term(iTRAQ4plex); K18(iTRAQ4plex)	HCD	4.129178	0	1	2	3.7981	4.3868	3.9061	4.51157	8.462430961	13029.818	11281.219	49488.6	50896.004	10.17189197	1.982744111	3	High	2935	Unique	Used	66255	1	32.625	1	4.8927	-1.2659	1	8	26/68	3.606726	2935	2133.1	MS2	711.706	122	Glioma_grade2_4.raw
209862831			lSLEGDHSTPPSAYGSVk	Unambiguous	N-Term(iTRAQ4plex); K18(iTRAQ4plex)	HCD	3.4674351	0	1	2	3.38007	4.3239	2.7979	3.5791924	17.89642557	12982.041	10148.327	43880.242	36322.812	17.32560749	13.32595141	3	High	2459	Unique	Used	73136	1	30.401	1	5.1162	11.524	1	22	22/68	31.03372	2459	2133.13	MS2	711.715	142	Glioma_grade2_5.raw
209862831			sALSGHLETVILGLLk	Unambiguous	N-Term(iTRAQ4plex); K16(iTRAQ4plex)	HCD	3.181048	0	1	2	2.89781	3.3196	3.0601	3.505516	8.444488684	11536.689	10070.71	33431.137	35303.035	9.594895637	3.85145185	2	High	7239	Unique	Used	83868	1	73.838	1	7.3832	-0.1549	1	100	21/30	32.52275	7239	1939.18	MS2	970.095	41	Glioma_grade2_6.raw
209862831			sALSGHLETVILGLLk	Unambiguous	N-Term(iTRAQ4plex); K16(iTRAQ4plex)	HCD	2.420139	0	1	2	1.99972	2.1298	2.6928	2.8678927	17.44619311	5251.946	4931.294	10502.436	14142.422	4.453105369	20.88759273	3	High	7199	Unique	Used	83836	1	73.444	1	6.0226	-0.5081	1	66	23/60	7.584676	7199	1939.18	MS2	647.066	40	Glioma_grade2_6.raw
209862831			rAEDGSVIDYELIDQDAR	Unambiguous	N-Term(iTRAQ4plex)	HCD	2.507589	1	1	2	2.78258	2.6166	2.3916	2.2489965	9.422505769	3021.8237	3213.4958	8408.479	7227.141	4.347255716	10.68498864	2	High	4446	Unique	Used	59985	1	44.983	1	5.8662	-0.6257	1	76	22/34	0	4446	2209.08	MS2	1105.05	96	Glioma_grade2_3.raw
209915588	NP_001129609.1	RPL28	60S ribosomal protein L28 isoform 5 [Homo sapiens]	2	4	2.318916798	16.4004612	12.719725	21.74	6.25E-05	69	7.8832	10.697	Ribosomal subunit	Protein metabolism	Structural constituent of ribosome	Ribosome	Nucleolus																								
209915588			gVVVVIk	Unambiguous	N-Term(iTRAQ4plex); K7(iTRAQ4plex)	HCD	2.2973979	0	1	5	2.45902	2.2099	2.3947	2.152147	6.347599829	443021.28	492951.94	1089397.8	1060905.1	7.544298816	1.873911009	2	High	1771	Unique	Used	57492	1	24.768	1	2.9935	-1.527	1	5	12-Dec	16.36068	1771	1001.69	MS2	501.351	42	Glioma_grade2_3.raw
209915588			gVVVVIk	Unambiguous	N-Term(iTRAQ4plex); K7(iTRAQ4plex)	HCD	1.7357775	0	1	5	1.61679	1.766	1.7081	1.8657415	6.001188098	224043.06	205115.78	362231.22	382693.03	6.237134967	3.884605611	2	High	1719	Unique	Used	49392	1	24.841	1	3.0455	-2.1973	1	10	12-Dec	31.85444	1719	1001.69	MS2	501.351	38	Glioma_grade2_2.raw
209915588			ncSSFLIk	Unambiguous	N-Term(iTRAQ4plex); C2(Methylthio); K8(iTRAQ4plex)	HCD	2.6141157	0	1	5	2.32991	2.6895	2.5488	2.94219	9.768114032	100877.04	87388.57	235034.33	257113.77	10.13226856	6.344643716	2	High	4480	Unique	Used	60015	1	45.279	1	3.3187	1.3703	1	52	14-Dec	47.53868	4480	1245.66	MS2	623.333	240	Glioma_grade2_3.raw
209915588			ncSSFLIk	Unambiguous	N-Term(iTRAQ4plex); C2(Methylthio); K8(iTRAQ4plex)	HCD	2.340436	0	1	5	2.20095	2.3203	2.3596	2.4874797	5.042251528	89269.07	84678.11	196477.17	210635.06	3.732511154	4.918122959	2	High	4604	Unique	Used	52111	1	45.549	1	3.362	0.9783	1	56	14/14	70.12177	4604	1245.66	MS2	623.333	185	Glioma_grade2_2.raw
215277000	NP_001135840.1	GSTM2	glutathione S-transferase Mu 2 isoform 2 [Homo sapiens]	7	12	2.234464884	14.18027101	81.388503	59.16	1.59E-04	191	22.629	5.313	Enzyme: Glutathione transferase	Metabolism,Energy pathways	Glutathione transferase activity	Cytoplasm	-																								
215277000			lYSQFLGk	Unambiguous	N-Term(iTRAQ4plex); K8(iTRAQ4plex)	HCD	2.4939547	0	1	2	2.29403	2.5236	2.467	2.7138817	6.92198644	159676.39	145151.34	366302.97	393923.56	6.738731645	5.138128102	2	High	4520	Unique	Used	52033	1	44.911	1	2.9919	0.5166	1	25	13/14	24.05553	4520	1243.73	MS2	622.368	237	Glioma_grade2_2.raw
215277000			lYSQFLGk	Unambiguous	N-Term(iTRAQ4plex); K8(iTRAQ4plex)	HCD	1.6815486	0	1	2	1.65574	1.6486	1.7146	1.7072482	2.031775751	108998.73	109467.984	180473.38	186889.03	0.30376498	2.46979522	2	High	4326	Unique	Used	59874	1	44.037	1	3.0892	2.087	1	48	13/14	33.95881	4326	1243.73	MS2	622.369	230	Glioma_grade2_3.raw
215277000			lYSQFLGk	Unambiguous	N-Term(iTRAQ4plex); K8(iTRAQ4plex)	HCD	2.2855144	0	1	2	2.06774	2.3523	2.2268	2.5332599	8.580568351	100189.74	88071.25	207165.84	223107.36	9.103390412	5.239627704	2	High	4414	Unique	Used	59955	1	44.737	1	3.2904	-0.1705	1	52	13/14	45.51488	4414	1243.73	MS2	622.368	251	Glioma_grade2_3.raw
215277000			lcYDPDFEk	Unambiguous	N-Term(iTRAQ4plex); C2(Methylthio); K9(iTRAQ4plex)	HCD	1.7868941	0	1	3	1.68536	1.7616	1.8111	1.8930187	4.875497834	72041.39	68923.1	121415.9	130472.72	3.128396385	5.084897315	2	High	4939	Unique	Used	60439	1	48.899	1	3.6136	-0.6164	1	100	13/16	49.01614	4939	1463.68	MS2	732.343	64	Glioma_grade2_3.raw
215277000			lkPEYLQALPEMLk	Unambiguous	N-Term(iTRAQ4plex); K2(iTRAQ4plex); K14(iTRAQ4plex)	HCD	2.398604	0	1	2	2.2043	2.5343	2.2805	2.6219966	8.279583104	58217.766	50636.18	128329.6	132767.89	9.849878796	2.403964095	2	High	5248	Unique	Used	92407	1	58.095	1	4.5272	-1.732	1	49	18/26	22.3307	5248	2105.24	MS2	1053.12	69	Glioma_grade2_7rep.raw
215277000			qPWFLGDk	Unambiguous	N-Term(iTRAQ4plex); K8(iTRAQ4plex)	HCD	2.4374676	0	1	2	2.30898	2.5081	2.3725	2.5770288	5.022980813	46906.57	43183.305	108306.52	111284.62	5.844709918	1.917959627	2	High	4238	Unique	Used	74746	1	44.54	1	3.1033	-1.6977	1	100	14-Nov	41.94028	4238	1278.71	MS2	639.857	332	Glioma_grade2_5.raw
215277000			lGLDFPNLPYLIDGTHk	Unambiguous	N-Term(iTRAQ4plex); K17(iTRAQ4plex)	HCD	2.0089874	0	1	2	2.15324	1.723	2.3664	1.8935611	13.93938291	42275.297	52832.16	91028.766	100040.93	15.6976743	6.670406047	3	High	6693	Unique	Used	83464	1	67.9	1	6.5065	1.2169	1	4	29/64	4.710283	6693	2201.22	MS2	734.413	113	Glioma_grade2_6.raw

215277000			nQVFEPScLDAFPNLk	Unambiguous	N-Term(iTRAQ4plex); C8(Methylthio); K16(iTRAQ4plex)	HCD	2.4144342	0	1	2	2.15058	2.2687	2.5526	2.6927862	10.34406465	22788.432	21601.65	49008.305	58168.625	3.780941878	12.08716165	2	High	7667	Unique	Used	54969	1	68.46	1	5.4306	-1.1105	1	61	23/30	0	7667	2156.07	MS2	1078.54	156	Glioma_grade2_2.raw
215277000			lkPEYLQALPEMLk	Unambiguous	N-Term(iTRAQ4plex); K2(iTRAQ4plex); K14(iTRAQ4plex)	HCD	2.1834154	0	1	2	1.92504	2.1749	2.1909	2.4753351	10.26552232	22087.494	19549.77	42519.21	48392.234	8.619403279	9.136044735	3	High	5217	Unique	Used	92383	1	57.787	1	4.4244	0.407	1	17	23/52	22.85573	5217	2105.24	MS2	702.419	57	Glioma_grade2_7rep.raw
215277000			lGLDFPNLPYLIDGTHk	Unambiguous	N-Term(iTRAQ4plex); K17(iTRAQ4plex)	HCD	2.0399554	0	1	2	2.13179	2.0531	2.0263	1.9515111	3.646456217	14288.474	14836.32	30460.037	28953.244	2.660177591	3.586617437	2	High	6689	Unique	Used	83461	1	67.852	1	4.6738	-1.9657	1	100	19/32	12.60706	6689	2201.22	MS2	1101.11	161	Glioma_grade2_6.raw
215277000			eQIREDILENQFMDSR	Unambiguous	N-Term(iTRAQ4plex)	HCD	2.3890433	1	1	2	2.34579	2.4426	2.3376	2.4340801	2.34259182	12628.442	12128.053	29623.691	29520.652	2.858469708	0.246378849	3	High	5298	Unique	Used	68441	1	51.081	1	5.0485	0.2289	1	33	26/60	12.19023	5298	2167.06	MS2	723.024	118	Glioma_grade2_4.raw
215277000			eQIREDILENQFMDSR	Unambiguous	N-Term(iTRAQ4plex)	HCD	1.6712903	1	1	2	1.73688	1.4874	1.8861	1.6151204	10.12962787	11301.353	13197.361	19629.135	21315.328	10.94490196	5.824076895	3	High	5151	Unique	Used	60637	1	50.535	1	4.6069	0.2289	1	47	25/60	27.51107	5151	2167.06	MS2	723.024	132	Glioma_grade2_3.raw
216548223	NP_055664.3	SV2A	synaptic vesicle glycoprotein 2A [Homo sapiens]	7	22	0.322234273	36.59725114	186.47449	10.38	3.52E-04	742	82.642	5.5669	Integral membrane protein	Biological_process unknown	Neurotransmitter transporter activity	-	-																								
216548223			vFSVTHIk	Unambiguous	N-Term(iTRAQ4plex); K8(iTRAQ4plex)	HCD	0.24193195	0	1	1	0.22646	0.2253	0.2587	0.25732642	0.07678727	3223664	3240775	730017.3	833937	0.374334235	9.396991276	2	High	1616	Unique	Used	79090	1	23.767	1	3.3713	9.5425	1	5	13/14	38.05689	1616	1218.76	MS2	609.882	184	Glioma_grade2_6.raw
216548223			vFSVTHIk	Unambiguous	N-Term(iTRAQ4plex); K8(iTRAQ4plex)	HCD	0.2601237	0	1	1	0.24821	0.2485	0.2717	0.27205294	0.052223356	2336712.8	2333867.2	579993.1	634935.44	0.086162449	6.395454532	2	High	1589	Unique	Used	79065	1	23.571	1	3.7172	-13.194	1	6	14/14	52.13434	1589	1218.73	MS2	609.868	297	Glioma_grade2_6.raw
216548223			vFSVTHIk	Unambiguous	N-Term(iTRAQ4plex); K8(iTRAQ4plex)	HCD	0.2454837	0	1	1	0.24457	0.2453	0.2457	0.24640022	0.003140936	844122	841777.8	206446.67	207414.23	0.196642732	0.330627144	3	High	1579	Unique	Used	79056	1	23.487	1	4.3554	-13.556	1	8	17/28	41.29472	1579	1218.73	MS2	406.914	269	Glioma_grade2_6.raw
216548223			hDEAWMVLk	Unambiguous	N-Term(iTRAQ4plex); K9(iTRAQ4plex)	HCD	0.22853576	0	1	1	0.22008	0.2229	0.2341	0.23709689	0.036343865	535609.8	528791.9	117878.945	125374.91	0.905857877	4.357958006	2	High	2607	Unique	Used	80013	1	31.139	1	4.4421	4.8117	1	19	15/16	28.28698	2607	1416.76	MS2	708.884	215	Glioma_grade2_6.raw
216548223			vFSVTHIk	Unambiguous	N-Term(iTRAQ4plex); K8(iTRAQ4plex)	HCD	0.6145201	0	1	1	0.53609	0.5443	0.6837	0.6941461	0.13998766	533006.75	524967.44	285742.25	364404.12	1.074629357	17.11071361	3	High	1658	Unique	Used	79130	1	24.05	1	3.4901	6.8022	1	6	15/28	55.3468	1658	1218.75	MS2	406.923	212	Glioma_grade2_6.raw
216548223			hDEAWmVLk	Unambiguous	N-Term(iTRAQ4plex); M6(Oxidation); K9(iTRAQ4plex)	HCD	0.5371845	0	1	1	0.49982	0.5569	0.5195	0.57882005	0.06627237	205339.75	184279.17	102632.76	106664.48	7.644433147	2.724218009	2	High	1907	Unique	Used	79359	1	25.956	1	3.3087	6.7734	1	62	13/16	10.33357	1907	1432.76	MS2	716.883	263	Glioma_grade2_6.raw
216548223			eELAQQYEAILR	Unambiguous	N-Term(iTRAQ4plex)	HCD	0.5167764	0	1	1	0.4714	0.5389	0.4974	0.5686553	0.08320114	114008.23	99720.68	53743.355	56706.695	9.453867043	3.794290376	2	High	4104	Unique	Used	86764	1	55.418	1	4.6499	2.2069	1	49	20/22	43.37269	4104	1606.86	MS2	803.933	295	Glioma_grade2_1rep.raw
216548223			eREELAQQYEAILR	Unambiguous	N-Term(iTRAQ4plex)	HCD	0.2429759	1	1	1	0.20772	0.2124	0.2729	0.27903172	0.157069529	92342.28	90301.016	19181.072	25196.848	1.580558004	19.17077684	2	High	4847	Unique	Used	68026	1	47.542	1	5.1221	1.1418	1	8	24/26	1.101829	4847	1892	MS2	946.504	157	Glioma_grade2_4.raw
216548223			aAPILFASAALALGSSLALk	Unambiguous	N-Term(iTRAQ4plex); K20(iTRAQ4plex)	HCD	0.29070666	0	1	1	0.25779	0.2672	0.3134	0.32481882	0.114372665	71255.65	68758.63	18368.99	22334.096	2.522113851	13.77661311	2	High	8431	Unique	Used	71238	1	77.477	1	6.8261	0.4146	1	34	28/38	3.705411	8431	2173.32	MS2	1087.16	22	Glioma_grade2_4.raw
216548223			eREELAQQYEAILR	Unambiguous	N-Term(iTRAQ4plex)	HCD	0.62315184	1	1	1	0.54426	0.6173	0.6283	0.712636	0.110233105	47809.11	42152.03	26020.395	30039.055	8.893083457	10.13788661	3	High	4846	Unique	Used	68025	1	47.539	1	5.0039	0.9734	1	3	24/52	7.000039	4846	1892	MS2	631.339	198	Glioma_grade2_4.raw
216548223			eREELAQQYEAILR	Unambiguous	N-Term(iTRAQ4plex)	HCD	0.42748478	1	1	1	0.38272	0.4012	0.4526	0.47440603	0.100351179	47393.824	45213.543	18138.664	21449.578	3.329522326	11.82760144	2	High	4761	Unique	Used	67947	1	46.863	1	5.1133	-0.7293	1	30	22/26	0	4761	1892	MS2	946.503	158	Glioma_grade2_4.raw
216548223			eREELAQQYEAILR	Unambiguous	N-Term(iTRAQ4plex)	HCD	0.29514232	1	1	1	0.23275	0.2931	0.2968	0.37369663	0.193126338	40663.023	32295.604	9464.42	12068.759	16.21921618	17.10426284	2	High	4613	Unique	Used	60137	1	46.342	1	5.281	-2.7294	1	29	23/26	2.070751	4613	1891.99	MS2	946.501	157	Glioma_grade2_3.raw
216548223			vFSVTHIk	Unambiguous	N-Term(iTRAQ4plex); K8(iTRAQ4plex)	HCD	0.3051022	0	1	1	0.28534	0.3517	0.2673	0.3294555	0.126133214	23394.088	18979.674	6675.3706	6252.9575	14.73299479	4.620723811	3	High	2402	Unique	Used	95104	1	34.405	1	3.7483	7.328	1	100	16/28	80.00519	2402	1218.75	MS2	406.923	218	Glioma_grade2_8rep.raw
216548223			aAPILFASAALALGSSLALk	Unambiguous	N-Term(iTRAQ4plex); K20(iTRAQ4plex)	HCD	0.28028297	0	1	1	0.27869	0.3101	0.2535	0.28205186	0.082393571	22079.783	19844.281	6153.4854	5597.116	7.540960836	6.696041542	2	High	8485	Unique	Used	71270	1	78.188	1	7.226	-2.5061	1	100	28/38	0	8485	2173.31	MS2	1087.16	27	Glioma_grade2_4.raw
216548223			aAPILFASAALALGSSLALk	Unambiguous	N-Term(iTRAQ4plex); K20(iTRAQ4plex)	HCD	0.34968773	0	1	1	0.3305	0.3686	0.3327	0.37109324	0.063060699	18210.824	16328.176	6018.589	6059.276	7.708579677	0.476409591	2	High	8524	Unique	Used	63724	1	77.735	1	6.969	1.875	1	100	28/38	29.8089	8524	2173.32	MS2	1087.17	22	Glioma_grade2_3.raw
216548223			tIHQEDELIEIQSDTGTWYQR	Unambiguous	N-Term(iTRAQ4plex)	HCD	0.2079052	0	1	1	0.18864	0.2367	0.185	0.23207211	0.130866135	16755.28	13355.622	3160.7454	3099.4675	15.96711534	1.384298564	2	High	5807	Unique	Used	68907	1	55.107	1	4.9091	1.157	1	37	25/40	3.628529	5807	2706.32	MS2	1353.66	107	Glioma_grade2_4.raw
216548223			aAPILFASAALALGSSLALkLPETR	Unambiguous	N-Term(iTRAQ4plex); K20(iTRAQ4plex)	HCD	0.4122405	1	1	1	0.42341	0.4149	0.4096	0.40129837	0.022509231	14474.033	14772.564	6128.426	5928.206	1.443540898	2.348531824	3	High	7609	Unique	Used	84169	1	77.335	1	9.8189	2.703	1	62	36/96	26.70594	7609	2769.66	MS2	923.89	13	Glioma_grade2_6.raw
216548223			tIHQEDELIEIQSDTGTWYQR	Unambiguous	N-Term(iTRAQ4plex)	HCD	0.1854598	0	1	1	0.178	0.2275	0.1525	0.19499803	0.16709077	14327.049	11207.889	2550.1882	2185.516	17.27499152	10.89013058	2	High	5707	Unique	Used	61150	1	54.894	1	5.2929	-0.7374	1	40	26/40	5.404195	5707	2706.31	MS2	1353.66	106	Glioma_grade2_3.raw
216548223			tIHQEDELIEIQSDTGTWYQR	Unambiguous	N-Term(iTRAQ4plex)	HCD	0.4826993	0	1	1	0.36926	0.5244	0.4534	0.6437934	0.233506405	9389.54	6612.2095	3467.1367	4256.897	24.54568146	14.4599282	3	High	5753	Unique	Used	68857	1	54.685	1	6.9987	1.8512	1	17	30/80	10.41709	5753	2706.32	MS2	902.777	105	Glioma_grade2_4.raw
216548223			aAPILFASAALALGSSLALk	Unambiguous	N-Term(iTRAQ4plex); K20(iTRAQ4plex)	HCD	0.33936638	0	1	1	0.27031	0.3781	0.3116	0.4359726	0.209250381	4072.1836	2910.9248	1100.7488	1269.0835	23.51772091	10.04548785	2	High	8530	Unique	Used	71285	1	78.953	1	5.0059	-1.4951	1	100	23/38	11.08497	8530	2173.32	MS2	1087.16	24	Glioma_grade2_4.raw
216548223			aAPILFASAALALGSSLALk	Unambiguous	N-Term(iTRAQ4plex); K20(iTRAQ4plex)	HCD	0.35868067	0	1	1	0.39128	0.4577	0.274	0.3205537	0.223178368	4031.4324	3446.5	1577.4015	1104.7883	11.06213976	24.91904179	2	High	8574	Unique	Used	63752	1	78.415	1	5.4691	1.875	1	100	27/38	26.36755	8574	2173.32	MS2	1087.17	18	Glioma_grade2_3.raw
216548223			aAPILFASAALALGSSLALk	Unambiguous	N-Term(iTRAQ4plex); K20(iTRAQ4plex)	HCD	0.46298784	0	1	1	0.4622	0.4318	0.4964	0.46371987	0.056897645	894.9226	957.92883	413.63705	444.2106	4.809034526	5.040234016	3	High	8522	Unique	Used	71282	1	78.822	1	5.3721	3.5538	1	100	28/76	10.67585	8522	2173.33	MS2	725.114	15	Glioma_grade2_4.raw
221139815	NP_001137416.1	LEMD2	LEM domain-containing protein 2 isoform 2 [Homo sapiens]	2	3	3.160921574	11.05516463	14.260005	11.44	3.88E-05	201	23.505	6.2271	Integral membrane protein	Biological_process unknown	Molecular function unknown	Integral to membrane	Nucleus																								
221139815			yPYVGILHVR	Unambiguous	N-Term(iTRAQ4plex)	HCD	3.200268	0	1	2	3.0848	3.6343	2.8318	3.3363035	10.66436466	9335.161	7923.603	28797.129	26435.545	11.56655522	6.046754357	3	High	3994	Unique	Used	74525	1	42.577	1	5.0371	1.0139	1	100	23/36	0	3994	1360.79	MS2	454.267	180	Glioma_grade2_5.raw
221139815			yPYVGILHVR	Unambiguous	N-Term(iTRAQ4plex)	HCD	3.1609216	0	1	2	3.08902	3.0987	3.2229	3.2330525	2.456093819	8747.682	8720.231	27021.738	28192.967	0.222245076	2.999867402	3	High	4083	Unique	Used	74605	1	43.303	1	4.956	-0.3989	1	55	24/36	0	4083	1360.79	MS2	454.267	222	Glioma_grade2_5.raw
221139815			fEAALTWILSSNk	Unambiguous	N-Term(iTRAQ4plex); K13(iTRAQ4plex)	HCD	2.609065	0	1	2	2.71136	2.1518	3.1853	2.527883	16.24170835	2738.0874	3450.1313	7423.9375	8721.528	16.27256872	11.36585429	3	High	8242	Unique	Used	63460	1	75.648	1	4.2668	-2.601	1	77	18/48	8.841878	8242	1767.98	MS2	589.999	222	Glioma_grade2_3.raw
221316661	NP_060715.2	OGDHL	2-oxoglutarate dehydrogenase-like, mitochondrial isoform a [Homo sapiens]	2	2	0.502398193	7.077612847	42.589801	4.85	1.28E-03	1010	114.41	6.6538	Unclassified	Biological_process unknown	Molecular function unknown	-	-																								
221316661			lEQISPFPFDLIk	Unambiguous	N-Term(iTRAQ4plex); K13(iTRAQ4plex)	HCD	0.47725502	0	1	3	0.47327	0.4187	0.5434	0.48078406	0.106534592	10487.497	11853.164	4963.38	5698.8125	8.644976051	9.754641137	2	High	8033	Unique	Used	70887	1	74.112	1	3.0257	1.0395	1	100	15/24	56.20199	8033	1835.06	MS2	918.033	163	Glioma_grade2_4.raw
221316661			fLDTAFNLQAFEGk	Unambiguous	N-Term(iTRAQ4plex); K14(iTRAQ4plex)	HCD	0.52754134	0	1	3	0.46586	0.4634	0.592	0.58890057	0.137764334	9271.481	9320.875	4319.1675	5489.0684	0.375711743	16.86837201	3	High	7389	Unique	Used	62700	1	68.302	1	4.5666	-0.9653	1	84	22/52	26.88308	7389	1889	MS2	630.339	173	Glioma_grade2_3.raw
221316760	NP_001137435.1	L1CAM	neural cell adhesion molecule L1 isoform 3 precursor [Homo sapiens]	6	14	0.371818304	23.75772995	68.472965	8.73	1.27E-04	1248	138.82	6.2144	Adhesion molecule	Biological_process unknown	Cell adhesion molecule activity	Cytoplasm, Nucleus, Plasma membrane	-																								
221316760			lGTAMSHEIR	Unambiguous	N-Term(iTRAQ4plex)	HCD	0.35279858	0	1	3	0.32868	0.3625	0.344	0.3793968	0.062347015	761485.75	690404.44	250287.55	261937.22	6.923674623	3.216385125	2	High	1349	Unique	Used	64783	1	20.778	1	3.5358	0.7813	1	10	16/18	9.49533	1349	1258.67	MS2	629.839	178	Glioma_grade2_4.raw
221316760			lVVFPTDDISLk	Unambiguous	N-Term(iTRAQ4plex); K12(iTRAQ4plex)	HCD	0.27153432	0	1	3	0.22678	0.2419	0.2993	0.31926972	0.163560175	721756.1	676622.94	163682.72	216025.22	4.564422461	19.49484475	2	High	6578	Unique	Used	53971	1	59.9	1	4.5071	-0.103	1	10	16/22	10.17642	6578	1634.96	MS2	817.984	207	Glioma_grade2_2.raw
221316760			lGTAMSHEIR	Unambiguous	N-Term(iTRAQ4plex)	HCD	0.44216558	0	1	3	0.37915	0.424	0.4584	0.51263005	0.127036864	540765.1	483604.03	205030.83	247909.97	7.891487362	13.38812077	3	High	1347	Unique	Used	64781	1	20.767	1	4.0498	0.8434	1	3	19/36	19.21348	1347	1258.67	MS2	420.228	183	Glioma_grade2_4.raw
221316760			wRPVDLAQVk	Unambiguous	N-Term(iTRAQ4plex); K10(iTRAQ4plex)	HCD	0.3768386	0	1	3	0.34891	0.3756	0.378	0.4069007	0.062820845	217261.23	201848.61	75804.42	82132.336	5.200721662	5.666207699	2	High	3169	Unique	Used	80491	1	36.143	1	4.0313	-2.6744	1	44	15/18	6.031139	3169	1499.89	MS2	750.449	167	Glioma_grade2_6.raw
221316760			lSPYVHYTFR	Unambiguous	N-Term(iTRAQ4plex)	HCD	0.47256848	0	1	3	0.46209	0.4926	0.4538	0.48373687	0.038375941	177797.61	166782.61	82158.84	80678.91	4.520736097	1.285289853	3	High	3102	Unique	Used	73733	1	35.23	1	3.481	-0.5003	1	10	15/36	29.54021	3102	1426.76	MS2	476.258	195	Glioma_grade2_5.raw
221316760			lVLSDLHLLTQSQVR	Unambiguous	N-Term(iTRAQ4plex)	HCD	0.36248305	0	1	3	0.31907	0.4184	0.3198	0.41941828	0.155578082	162229.56	123705.7	51762.25	51884.434	19.0536016	0.166714711	3	High	6353	Unique	Used	69411	1	59.432	1	3.8014	0.0088	1	4	22/56	5.319174	6353	1866.09	MS2	622.703	106	Glioma_grade2_4.raw
221316760			lVVFPTDDISLk	Unambiguous	N-Term(iTRAQ4plex); K12(iTRAQ4plex)	HCD	0.36679798	0	1	3	0.36166	0.3528	0.3811	0.37181097	0.033487341	121994.945	125054.68	44120.6	46496.703	1.751518034	3.708251048	2	High	6441	Unique	Used	61837	1	60.58	1	4.4129	1.8382	1	51	16/22	24.41344	6441	1634.96	MS2	817.986	210	Glioma_grade2_3.raw
221316760			lVLSDLHLLTQSQVR	Unambiguous	N-Term(iTRAQ4plex)	HCD	0.30139756	0	1	3	0.28656	0.2866	0.3162	0.3162402	0.056762676	80182.05	80168.57	22976.76	25352.523	0.011888697	6.951967931	2	High	6346	Unique	Used	69405	1	59.365	1	5.7455	1.8149	1	59	22/28	0	6346	1866.1	MS2	933.552	89	Glioma_grade2_4.raw
221316760			yDIEFEDkEMAPEk	Unambiguous	N-Term(iTRAQ4plex); K8(iTRAQ4plex); K14(iTRAQ4plex)	HCD	0.20723934	1	1	3	0.16373	0.1992	0.2138	0.26018426	0.190938319	54416.566	44722.77	8909.41	11636.16	13.82811135	18.76904282	3	High	3718	Unique	Used	74282	1	40.257	1	3.4392	7.9263	1	41	24/52	18.24904	3718	2176.1	MS2	726.039	123	Glioma_grade2_5.raw
221316760			lVLSDLHLLTQSQVR	Unambiguous	N-Term(iTRAQ4plex)	HCD	0.3386435	0	1	3	0.32332	0.3479	0.3301	0.35513008	0.043874061	38436.15	35724.94	12427.168	12687.002	5.170136998	1.463161103	2	High	6428	Unique	Used	69477	1	60.072	1	5.5923	1.0953	1	100	23/28	32.24669	6428	1866.1	MS2	933.551	97	Glioma_grade2_4.raw
221316760			lVLSDLHLLTQSQVR	Unambiguous	N-Term(iTRAQ4plex)	HCD	0.5164019	0	1	3	0.4629	0.6725	0.4089	0.5941362	0.225237671	36161.76	24889.85	16739.205	14787.959	26.11051207	8.752701501	3	High	6315	Unique	Used	61719	1	59.593	1	4.0786	0.3032	1	34	22/56	19.23832	6315	1866.09	MS2	622.703	112	Glioma_grade2_3.raw
221316760			lVLSDLHLLTQSQVR	Unambiguous	N-Term(iTRAQ4plex)	HCD	0.4765422	0	1	3	0.39716	0.4696	0.4825	0.5704007	0.148084444	33633.98	28447.922	13357.932	16226.714	11.8137385	13.71343234	2	High	6327	Unique	Used	61731	1	59.677	1	5.5597	0.5066	1	100	23/28	0	6327	1866.09	MS2	933.551	109	Glioma_grade2_3.raw
221316760			lVLSDLHLLTQSQVR	Unambiguous	N-Term(iTRAQ4plex)	HCD	0.42038685	0	1	3	0.43079	0.405	0.4368	0.41060346	0.036577011	29365.465	31238.8	12650.477	12826.76	4.371467526	0.978527654	3	High	5877	Unique	Used	76209	1	57.952	1	4.0214	0.0088	1	26	19/56	33.57735	5877	1866.09	MS2	622.703	81	Glioma_grade2_5.raw
221316760			lVLSDLHLLTQSQVR	Unambiguous	N-Term(iTRAQ4plex)	HCD	0.5140065	0	1	3	0.41014	0.5716	0.4727	0.65875727	0.207209205	17375.182	12467.148	7126.326	8212.824	23.25893537	10.01711442	3	High	6457	Unique	Used	69503	1	60.319	1	4.092	0.5975	1	53	20/56	25.03239	6457	1866.09	MS2	622.703	85	Glioma_grade2_4.raw
221554543	NP_001138349.1	SCAI	protein SCAI isoform 2 [Homo sapiens]	3	5	0.458987027	4.829663689	26.390944	5.45	1.00E-04	606	70.355	8.6021	Unclassified	Biological_process unknown	Molecular function unknown	-	-																								
221554543			tVTDFcYLLDk	Unambiguous	N-Term(iTRAQ4plex); C6(Methylthio); K11(iTRAQ4plex)	HCD	0.4141963	0	1	2	0.38732	0.4127	0.4156	0.44283888	0.054757503	62589.598	58737.195	24241.996	26011.115	4.490451314	4.97862129	2	High	8159	Unique	Used	55418	1	72.446	1	5.1649	-4.9149	1	100	18/20	19.83073	8159	1651.82	MS2	826.415	164	Glioma_grade2_2.raw
221554543			dIAQLLTHSR	Unambiguous	N-Term(iTRAQ4plex)	HCD	0.43946922	0	1	2	0.40398	0.4623	0.4195	0.4800833	0.080774722	15774.775	13783.767	6372.7134	6617.356	9.52587755	2.663395184	3	High	3231	Unique	Used	58855	1	35.597	1	5.3316	-1.4608	1	96	20/36	53.6795	3231	1297.73	MS2	433.249	200	Glioma_grade2_3.raw
221554543			dIAQLLTHSR	Unambiguous	N-Term(iTRAQ4plex)	HCD	0.46277386	0	1	2	0.47746	0.5394	0.3949	0.44617724	0.130089075	14155.988	12530.341	6758.9824	5590.753	8.614943011	13.37782396	3	High	3619	Unique	Used	66889	1	37.929	1	5.1811	2.4194	1	51	22/36	10.00591	3619	1297.74	MS2	433.251	152	Glioma_grade2_4.raw
221554543			dIAQLLTHSR	Unambiguous	N-Term(iTRAQ4plex)	HCD	0.46591	0	1	2	0.40098	0.3881	0.5463	0.52875435	0.178175417	11483.407	11864.675	4604.607	6273.4985	2.30937332	21.69650766	3	High	3522	Unique	Used	66799	1	37.169	1	5.5211	0.1618	1	100	20/36	41.31208	3522	1297.73	MS2	433.25	207	Glioma_grade2_4.raw
221554543			hILELASILDVR	Unambiguous	N-Term(iTRAQ4plex)	HCD	0.45898703	0	1	2	0.4921	0.4358	0.4852	0.42966378	0.070519259	7660.8184	8650.161	3769.8672	3716.661	8.577913615	1.005071077	3	High	6252	Unique	Used	76538	1	61.119	1	5.1922	-1.7575	1	100	23/44	17.48602	6252	1522.9	MS2	508.306	153	Glioma_grade2_5.raw
222136619	NP_001138397.1	MX1	interferon-induced GTP-binding protein Mx1 [Homo sapiens]	3	3	2.690617085	33.66822668	12.791914	4.98	9.96E-05	662	75.473	5.8335	GTPase	Cell communication,Signal transduction	GTPase activity	Cytoplasm	Nucleus, Endoplasmic reticulum																								
222136619			lTSELITHIck	Unambiguous	N-Term(iTRAQ4plex); C10(Methylthio); K11(iTRAQ4plex)	HCD	2.690617	0	1	1	2.53711	2.7262	2.6575	2.855566	4.937800969	8605.583	8008.624	21833.314	22869.156	5.081359068	3.277004167	3	High	5309	Unique	Used	82341	1	54.695	1	4.082	-2.5474	1	71	23/40	8.972806	5309	1591.88	MS2	531.297	204	Glioma_grade2_6.raw
222136619			nFEEFFNLHR	Unambiguous	N-Term(iTRAQ4plex)	HCD	3.9271681	0	1	1	3.41759	4.0243	3.8446	4.5272202	11.62098349	3804.317	3230.7324	13001.584	14626.237	11.53042537	8.316277664	3	High	5371	Unique	Used	75753	1	53.872	1	4.6458	0.187	1	83	22/36	64.21064	5371	1496.74	MS2	499.585	128	Glioma_grade2_5.raw
222136619			vRPcIDLIDSLR	Unambiguous	N-Term(iTRAQ4plex); C4(Methylthio)	HCD	2.016552	0	1	2	2.15949	1.7951	2.2829	1.8977315	11.11656152	1844.8922	2219.3704	3984.0276	4211.769	13.03046091	3.929758049	3	High	6179	Unique	Used	83047	1	62.975	1	4.0641	-6.4461	1	100	23/44	59.89418	6179	1589.85	MS2	530.622	209	Glioma_grade2_6.raw

223468595	NP_001138471.1	ITGAV	integrin alpha-V isoform 2 precursor [Homo sapiens]	12	15	2.194468975	26.2827388	76.157426	15.67	9.51E-05	1002	111.08	5.6177	Cell surface receptor	Cell adhesion,Cell communication,Signal transduction	Receptor activity	Plasma membrane	Cell surface, Nucleus, Cytoplasm																								
223468595			yDPNVYSIk	Unambiguous	N-Term(iTRAQ4plex); K9(iTRAQ4plex)	HCD	1.891793	0	1	3	1.80774	1.9556	1.8328	1.9827236	4.606252175	147636.02	136472.9	266887.22	270588.06	5.556684282	0.973770946	2	High	3092	Unique	Used	50685	1	34.56	1	3.0294	-1.7369	1	38	16-Dec	51.09169	3092	1386.75	MS2	693.878	234	Glioma_grade2_2.raw
223468595			mFLLVGAPk	Unambiguous	N-Term(iTRAQ4plex); K9(iTRAQ4plex)	HCD	1.7803237	0	1	3	1.6983	1.8866	1.6847	1.8714453	6.082965125	106503.266	95873.41	180874.17	179421.83	7.428171476	0.570064315	2	High	5377	Unique	Used	60849	1	52.246	1	3.7521	-0.2312	1	37	15/16	12.17108	5377	1263.77	MS2	632.39	218	Glioma_grade2_3.raw
223468595			mFLLVGAPk	Unambiguous	N-Term(iTRAQ4plex); M1(Oxidation); K9(iTRAQ4plex)	HCD	1.8672156	0	1	3	1.65331	1.8508	1.8819	2.1066647	9.912868453	87756.32	78394.02	145088.6	165149.92	7.968862197	9.144896263	2	High	3855	Unique	Used	59435	1	40.397	1	3.9921	-1.215	1	44	16/16	17.2035	3855	1279.77	MS2	640.387	209	Glioma_grade2_3.raw
223468595			yDPNVYSIk	Unambiguous	N-Term(iTRAQ4plex); K9(iTRAQ4plex)	HCD	2.5593655	0	1	3	2.39056	2.4917	2.6243	2.7353098	5.890391966	85117.08	81662.445	203477.67	223372.08	2.929371378	6.591299266	2	High	2988	Unique	Used	50590	1	33.76	1	3.116	1.432	1	50	13/16	33.13105	2988	1386.75	MS2	693.88	230	Glioma_grade2_2.raw
223468595			mFLLVGAPk	Unambiguous	N-Term(iTRAQ4plex); K9(iTRAQ4plex)	HCD	2.5686893	0	1	3	2.42933	2.6142	2.5264	2.7186496	4.799226693	76844.77	71411.56	186681.44	194143.02	5.182725938	2.770900701	2	High	5568	Unique	Used	68694	1	53.108	1	3.513	0.7347	1	50	15/16	40.44556	5568	1263.77	MS2	632.391	287	Glioma_grade2_4.raw
223468595			tLGMVYIYDGk	Unambiguous	N-Term(iTRAQ4plex); K11(iTRAQ4plex)	HCD	1.5976002	0	1	3	1.61793	1.4734	1.734	1.5790844	6.722010482	76531.78	84040.81	123823.27	132707.55	6.613440106	4.897762097	2	High	5303	Unique	Used	52768	1	50.646	1	4.5787	-0.8048	1	49	18/20	60.678	5303	1547.84	MS2	774.422	254	Glioma_grade2_2.raw
223468595			fDLQIQSSNLFDk	Unambiguous	N-Term(iTRAQ4plex); K13(iTRAQ4plex)	HCD	2.5276635	0	1	3	2.34256	2.5582	2.4997	2.7298083	6.315572401	67813.555	62096.277	158857.39	169510.94	6.223895423	4.588260658	2	High	6522	Unique	Used	53918	1	59.501	1	4.7971	1.8649	1	24	21/24	3.456546	6522	1842.99	MS2	921.997	187	Glioma_grade2_2.raw
223468595			tcSLPGTALk	Unambiguous	N-Term(iTRAQ4plex); C2(Methylthio); K10(iTRAQ4plex)	HCD	2.8280065	0	1	3	2.74566	2.7328	2.9237	2.9099674	3.636316043	59201.992	59481.273	162548.47	173088.56	0.332787422	4.441088704	2	High	3616	Unique	Used	59212	1	38.578	1	3.6813	0.1937	1	78	14/18	51.9515	3616	1324.72	MS2	662.864	358	Glioma_grade2_3.raw
223468595			ePVGTcFLQDGTk	Unambiguous	N-Term(iTRAQ4plex); C6(Methylthio); K13(iTRAQ4plex)	HCD	3.0988631	0	1	3	3.08903	3.0175	3.1822	3.1084638	2.184959611	58249.92	59631.52	179936.03	185362.4	1.657493714	2.100760753	2	High	4812	Unique	Used	52307	1	47.05	1	5.2481	-2.4174	1	48	22/24	22.97255	4812	1728.85	MS2	864.929	121	Glioma_grade2_2.raw
223468595			sSASFNVIEFPYk	Unambiguous	N-Term(iTRAQ4plex); K13(iTRAQ4plex)	HCD	1.918431	0	1	3	1.77969	1.8928	1.9425	2.0659902	6.179761157	52384.21	49253.492	93227.7	101757.23	4.356162889	6.186415025	2	High	6080	Unique	Used	53502	1	56.267	1	5.0166	0.6128	1	57	19/24	36.50403	6080	1776.94	MS2	888.975	229	Glioma_grade2_2.raw
223468595			iYIGDDNPLTLIVk	Unambiguous	N-Term(iTRAQ4plex); K14(iTRAQ4plex)	HCD	2.194469	0	1	3	2.11685	2.2515	2.1408	2.2770228	3.61892295	49507.445	46546.047	104800	105986.41	4.36012177	0.795991894	2	High	7762	Unique	Used	55055	1	69.241	1	6.2604	1.0698	1	69	20/26	48.8011	7762	1862.09	MS2	931.549	148	Glioma_grade2_2.raw
223468595			aMLHLQWPYk	Unambiguous	N-Term(iTRAQ4plex); K10(iTRAQ4plex)	HCD	3.0541296	0	1	3	3.0623	2.8234	3.3044	3.046597	6.425039422	20848.154	22612.316	63843.297	68890.62	5.740623207	5.377670457	3	High	4499	Unique	Used	81636	1	47.788	1	4.4817	0.6113	1	20	16/36	33.27025	4499	1574.88	MS2	525.63	233	Glioma_grade2_6.raw
223468595			fSVHQQSEmDTSVk	Unambiguous	N-Term(iTRAQ4plex); M9(Oxidation); K14(iTRAQ4plex)	HCD	1.5130906	0	1	3	1.59892	1.531	1.4944	1.4309114	4.637859199	13939.667	14558.086	22288.352	20831.33	3.068931565	4.778653686	3	High	1177	Unique	Used	71957	1	20.736	1	4.6447	-0.9542	1	30	19/52	26.32945	1177	1926.95	MS2	642.987	113	Glioma_grade2_5.raw
223468595			iLAcAPLYHWR	Unambiguous	N-Term(iTRAQ4plex); C4(Methylthio)	HCD	3.2046995	0	1	3	2.84764	3.6119	2.8836	3.657593	13.68166248	12782.841	10078.001	36400.906	36861.227	16.73263571	0.888579372	3	High	5683	Unique	Used	82648	1	58.187	1	5.1467	0.0228	1	42	25/40	14.35037	5683	1532.8	MS2	511.604	157	Glioma_grade2_6.raw
223468595			kLNFQVELLLDk	Unambiguous	N-Term(iTRAQ4plex); K1(iTRAQ4plex); K12(iTRAQ4plex)	HCD	1.5104237	1	1	3	1.29868	1.5549	1.4732	1.7639549	12.68211743	11424.863	9541.992	14837.192	16831.645	12.69995764	8.906492152	3	High	5144	Unique	Used	92326	1	57.069	1	5.2575	-2.7604	1	74	20/44	40.10625	5144	1892.15	MS2	631.389	49	Glioma_grade2_7rep.raw
223890243	NP_006004.2	RPL10	60S ribosomal protein L10 isoform a [Homo sapiens]	4	9	2.295339346	18.7467271	54.272406	27.57	1.07E-04	214	24.588	10.082	Ribosomal subunit	Protein metabolism	Structural constituent of ribosome	Ribosome	Cytoplasm, Nucleolus, Endoplasmic reticulum																								
223890243			lHPFHVIR	Unambiguous	N-Term(iTRAQ4plex)	HCD	2.7975862	0	1	4	2.66293	3.0104	2.6093	2.9498205	7.170161548	323651.94	286290.72	861860.94	844506.25	8.662575599	1.438332741	3	High	1669	Unique	Used	85171	1	25.834	1	3.494	4.0451	1	6	19/28	39.10403	1669	1162.7	MS2	388.239	269	Glioma_grade2_1rep.raw
223890243			fNADEFEDMVAEk	Unambiguous	N-Term(iTRAQ4plex); K13(iTRAQ4plex)	HCD	2.1805098	0	1	2	2.15097	2.2067	2.1549	2.2108161	1.481647556	133855.3	130472.375	287918.66	288450.44	1.809942306	0.130480709	2	High	6455	Unique	Used	53854	1	59.035	1	5.3449	-0.6373	1	18	23/24	6.674957	6455	1832.86	MS2	916.934	66	Glioma_grade2_2.raw
223890243			vHIGQVIMSIR	Unambiguous	N-Term(iTRAQ4plex)	HCD	2.1332328	0	1	3	2.01709	2.2579	2.0219	2.2632377	6.505475468	133050.45	118861.125	268375.1	269010.97	7.965785556	0.167338907	2	High	3983	Unique	Used	74514	1	42.505	1	4.2437	0.0118	1	25	17/20	24.71261	3983	1396.82	MS2	698.914	215	Glioma_grade2_5.raw
223890243			fNADEFEDmVAEk	Unambiguous	N-Term(iTRAQ4plex); M9(Oxidation); K13(iTRAQ4plex)	HCD	1.8984852	0	1	2	1.7412	2.0833	1.744	2.0866702	10.3294399	54623.17	45653.25	95110.02	95263.28	12.65041424	0.113851244	2	High	5117	Unique	Used	52593	1	49.291	1	3.8758	-0.9186	1	45	17/24	17.55202	5117	1848.85	MS2	924.931	55	Glioma_grade2_2.raw
223890243			vHIGQVIMSIR	Unambiguous	N-Term(iTRAQ4plex)	HCD	2.307975	0	1	3	2.13414	2.2947	2.3203	2.4948874	6.391212793	34175.414	31784.348	72935.11	79298.37	5.126576966	5.911320291	3	High	4057	Unique	Used	74582	1	43.087	1	4.9006	0.024	1	16	22/40	7.293164	4057	1396.82	MS2	466.279	141	Glioma_grade2_5.raw
223890243			lHPFHVIR	Unambiguous	N-Term(iTRAQ4plex)	HCD	2.2101996	0	1	4	2.06276	2.3952	2.0509	2.381401	8.616330964	23716.932	20425.508	48922.273	48641.324	10.54490069	0.407243991	3	High	1834	Unique	Used	94713	1	27.66	1	3.8734	-1.1518	1	100	18/28	36.74595	1834	1162.7	MS2	388.237	279	Glioma_grade2_8rep.raw
223890243			vDEFPLcGHMVSDEYEQLSSEALEAAR	Unambiguous	N-Term(iTRAQ4plex); C7(Methylthio)	HCD	2.4857464	0	1	2	2.63456	2.6329	2.3385	2.3370273	6.873648396	15729.378	15739.489	41440.008	36783.617	0.04543892	8.418340756	3	High	8306	Unique	Used	63520	1	76.115	1	7.9423	-1.0691	1	6	31/104	3.581934	8306	3215.44	MS2	1072.49	38	Glioma_grade2_3.raw
223890243			vHIGQVIMSIR	Unambiguous	N-Term(iTRAQ4plex)	HCD	2.2953393	0	1	3	2.25173	2.3687	2.2256	2.3412187	2.999449172	12889.806	12253.363	29024.299	28687.803	3.579764835	0.824570914	3	High	3962	Unique	Used	74495	1	42.334	1	4.8176	-2.6633	1	69	22/40	15.90836	3962	1396.82	MS2	466.277	136	Glioma_grade2_5.raw
223890243			vDEFPLcGHMVSDEYEQLSSEALEAAR	Unambiguous	N-Term(iTRAQ4plex); C7(Methylthio)	HCD	3.4245944	0	1	2	3.07005	3.46	3.3932	3.8241699	8.999561935	10426.979	9251.774	32011.387	35380.36	8.445610601	7.0697786	3	High	8611	Unique	Used	55828	1	76.147	1	4.7852	-1.183	1	11	21/104	46.69525	8611	3215.44	MS2	1072.49	21	Glioma_grade2_2.raw
224809323	NP_001139246.1	GPR56	G-protein coupled receptor 56 isoform b precursor [Homo sapiens]	5	6	2.495543003	19.92842089	30.270347	10.33	1.05E-04	687	77.023	8.4409	G protein coupled receptor	Signal transduction	G-protein coupled receptor activity	Plasma membrane	-																								
224809323			sSLHYkPTPDLR	Unambiguous	N-Term(iTRAQ4plex); K6(iTRAQ4plex)	HCD	2.061344	0	1	3	1.95456	2.0974	2.0277	2.1759374	4.591398939	83917.5	78201.83	164021.4	170162.3	4.985943399	2.59873359	3	High	1177	Unique	Used	89095	1	20.066	1	3.2892	-3.2196	1	13	15/44	62.05973	1177	1701.95	MS2	567.987	248	Glioma_grade2_7rep.raw
224809323			vLGIVVQNTk	Unambiguous	N-Term(iTRAQ4plex); K10(iTRAQ4plex)	HCD	2.059245	0	1	3	1.86447	2.0049	2.1097	2.2686946	8.271936995	76027.52	70700.93	141751.03	160398.83	5.133929936	8.728109842	2	High	3451	Unique	Used	59062	1	37.232	1	4.1252	-1.7877	1	55	16/18	21.21953	3451	1358.86	MS2	679.933	117	Glioma_grade2_3.raw
224809323			lQPTAGLQDLHIHSR	Unambiguous	N-Term(iTRAQ4plex)	HCD	2.3950315	0	1	3	2.28678	2.36	2.429	2.5067532	3.92927898	46548.645	45104.74	106446.29	113066.45	2.227948301	4.265043204	3	High	2390	Unique	Used	79810	1	29.54	1	6.6982	3.7599	1	23	28/56	22.94788	2390	1830.02	MS2	610.677	197	Glioma_grade2_6.raw
224809323			dLQLLSQFLk	Unambiguous	N-Term(iTRAQ4plex); K10(iTRAQ4plex)	HCD	3.412753	0	1	3	3.12552	3.7149	3.1585	3.754156	9.968264834	44276.117	37251.18	138385.78	139846.75	12.18580959	0.742588794	2	High	7443	Unique	Used	62749	1	68.746	1	4.6324	1.1282	1	47	17/18	34.26276	7443	1492.9	MS2	746.953	188	Glioma_grade2_3.raw
224809323			iSIENSEEALTVHAPFPAAHPASR	Unambiguous	N-Term(iTRAQ4plex)	HCD	2.736451	0	1	3	2.29772	2.7969	2.6868	3.2704945	14.49208408	7711.47	6335.2026	17718.785	20719.246	13.85620693	11.03930802	4	High	4658	Unique	Used	81772	1	49.194	1	7.2377	0.3889	1	9	42/138	22.09473	4658	2688.39	MS2	672.852	97	Glioma_grade2_6.raw
224809323			dLQLLSQFLk	Unambiguous	N-Term(iTRAQ4plex); K10(iTRAQ4plex)	HCD	2.5960546	0	1	3	2.5887	2.595	2.5971	2.6034305	0.234208487	4358.113	4347.5806	11281.831	11318.623	0.171095649	0.230224337	3	High	7450	Unique	Used	62756	1	68.791	1	4.2876	-1.0476	1	71	20/36	0	7450	1492.9	MS2	498.304	187	Glioma_grade2_3.raw
226371772	NP_001139508.2	SLC14A1	urea transporter 1 isoform 2 [Homo sapiens]	2	3	2.838840485	25.01791397	13.694092	4.37	8.90E-05	389	42.5	7.1519	Transport/cargo protein	Transport	Transporter activity	Plasma membrane	-																								
226371772			iFYLQAk	Unambiguous	N-Term(iTRAQ4plex); K7(iTRAQ4plex)	HCD	1.8701385	0	1	2	1.74783	2.0513	1.7157	2.0136926	9.287318784	126579.414	107850.37	221238.67	217177.5	11.29842274	1.31002506	2	High	3725	Unique	Used	51283	1	39.091	1	2.9448	2.5492	1	20	12-Nov	17.5124	3725	1170.72	MS2	585.861	118	Glioma_grade2_2.raw
226371772			iFYLQAk	Unambiguous	N-Term(iTRAQ4plex); K7(iTRAQ4plex)	HCD	3.1107743	0	1	2	3.01221	3.0937	3.1274	3.2120082	2.658961452	96520.51	93978.695	290739.7	301860.34	1.886973358	2.653891132	2	High	3679	Unique	Used	59269	1	39.092	1	3.183	-0.0575	1	40	12-Dec	35.81585	3679	1170.71	MS2	585.86	114	Glioma_grade2_3.raw
226371772			aLGYVTGDMk	Unambiguous	N-Term(iTRAQ4plex); K10(iTRAQ4plex)	HCD	2.8388405	0	1	2	2.6872	2.8226	2.8543	2.9981174	4.492592409	70890.06	67490.32	190495.92	202343.89	3.474450942	4.265239783	2	High	2969	Unique	Used	58609	1	33.656	1	4.2234	-3.4431	1	51	16/18	28.959	2969	1342.72	MS2	671.865	242	Glioma_grade2_3.raw
226443017	NP_001139792.1	GRAMD3	GRAM domain-containing protein 3 isoform 3 [Homo sapiens]	2	2	2.594815254	32.37024052	11.800538	9.45	7.21E-04	328	36.626	7.7817	Integral membrane protein	Biological_process unknown	Molecular function unknown	Cytoplasm, Nucleus	-																								
226443017			sLPVQGLSETVGILHk	Unambiguous	N-Term(iTRAQ4plex); K16(iTRAQ4plex)	HCD	3.1887481	0	1	5	2.64628	3.772	2.7796	3.9619865	20.45644484	24301.79	17049.1	64309.26	67548.305	24.80442999	3.473976915	3	High	5301	Unique	Used	75690	1	53.308	1	8.2591	-2.4373	1	16	38/60	44.61699	5301	1966.15	MS2	656.056	68	Glioma_grade2_5.raw
226443017			dFHATESQTVLNVSk	Unambiguous	N-Term(iTRAQ4plex); K15(iTRAQ4plex)	HCD	2.0008824	0	1	5	1.92074	1.9282	2.0733	2.081338	4.415714573	10062.82	10023.85	19328.047	20863.018	0.274370528	5.401142781	3	High	2668	Unique	Used	66005	1	30.638	1	3.5414	15.586	1	100	21/56	38.29557	2668	1964.06	MS2	655.359	198	Glioma_grade2_4.raw
238624147	NP_001154835.1	NPTN	neuroplastin isoform c precursor [Homo sapiens]	8	22	0.462926835	20.20484786	138.47898	20.05	1.59E-04	394	43.903	8.5288	Immunoglobulin	Immune response	Antigen binding	Plasma membrane	-																								
238624147			sVGYPHPDWIWR	Unambiguous	N-Term(iTRAQ4plex)	HCD	0.37104207	0	1	4	0.38588	0.3372	0.4097	0.35807553	0.085062139	400982.94	458817.2	154730.81	164291.2	9.51267522	4.238087899	3	High	4970	Unique	Used	75395	1	50.613	1	4.0712	-2.8415	1	2	25/44	7.681915	4970	1656.84	MS2	552.95	156	Glioma_grade2_5.raw
238624147			aQATISVLQkPR	Unambiguous	N-Term(iTRAQ4plex); K10(iTRAQ4plex)	HCD	0.31951553	0	1	2	0.28369	0.3277	0.3124	0.360899	0.100115795	357764.34	309730.12	101493.555	111781.3	10.17696018	6.82174582	3	High	1480	Unique	Used	72234	1	23.038	1	4.9143	4.8012	1	6	25/44	41.80817	1480	1599.99	MS2	534	51	Glioma_grade2_5.raw
238624147			sVGYPHPDWIWR	Unambiguous	N-Term(iTRAQ4plex)	HCD	0.45101944	0	1	4	0.39312	0.4264	0.4738	0.5138078	0.117251502	350545.47	323223.34	137808.16	166074.67	5.73480491	13.15470236	2	High	4943	Unique	Used	75371	1	50.391	1	4.2057	1.4767	1	12	18/22	23.44987	4943	1656.84	MS2	828.925	238	Glioma_grade2_5.raw
238624147			sENkNEGQDATMYck	Unambiguous	N-Term(iTRAQ4plex); K4(iTRAQ4plex); C14(Methylthio); K15(iTRAQ4plex)	HCD	0.37254226	1	1	4	0.34303	0.3704	0.3746	0.40440518	0.06734316	326504.5	302409.34	112001.07	122295.91	5.418182888	6.213952203	2	High	1354	Unique	Used	78843	1	21.904	1	5.9608	-6.7918	1	26	18/28	0	1354	2195.99	MS2	1098.5	41	Glioma_grade2_6.raw
238624147			sVGYPHPDWIWR	Unambiguous	N-Term(iTRAQ4plex)	HCD	0.37570712	0	1	4	0.32752	0.3692	0.3815	0.43003058	0.11191094	314351.2	278860.22	102955.32	119918.42	8.461034897	10.76369342	3	High	4882	Unique	Used	75316	1	49.906	1	4.3098	0.474	1	3	26/44	7.717639	4882	1656.84	MS2	552.952	147	Glioma_grade2_5.raw
238624147			aQATISVLQkPR	Unambiguous	N-Term(iTRAQ4plex); K10(iTRAQ4plex)	HCD	0.35825887	0	1	2	0.33967	0.3872	0.3329	0.3794526	0.076440027	193979.42	170158.86	65888.555	64567.223	9.2512545	1.432397755	2	High	1516	Unique	Used	72268	1	23.29	1	4.4961	7.1366	1	38	18/22	22.45786	1516	1599.99	MS2	800.499	43	Glioma_grade2_5.raw
238624147			nEGQDATMYck	Unambiguous	N-Term(iTRAQ4plex); C10(Methylthio); K11(iTRAQ4plex)	HCD	0.5729149	0	1	4	0.58641	0.5559	0.5909	0.56012577	0.031169388	155174	163695.44	90995.016	91690.02	3.779332387	0.538021123	2	High	2091	Unique	Used	49745	1	27.4	1	4.8991	-0.0794	1	54	17/20	0	2091	1593.69	MS2	797.351	25	Glioma_grade2_2.raw
238624147			sVGYPHPDWIWR	Unambiguous	N-Term(iTRAQ4plex)	HCD	0.55908394	0	1	4	0.53738	0.5909	0.5301	0.5829495	0.05535055	147503.17	134139.06	79265.34	78196.3	6.710536844	0.960139159	2	High	4857	Unique	Used	75293	1	49.713	1	4.0106	-1.8387	1	24	18/22	14.17851	4857	1656.84	MS2	828.922	240	Glioma_grade2_5.raw
238624147			aEDSGEYHcVYHFVSAPk	Unambiguous	N-Term(iTRAQ4plex); C9(Methylthio); K18(iTRAQ4plex)	HCD	0.5485742	0	1	4	0.62456	0.5057	0.6015	0.48704663	0.123384438	74303.27	91769.41	46407.21	44695.98	14.87351927	2.656377537	3	High	3492	Unique	Used	80785	1	38.669	1	7.6718	1.8399	1	15	33/68	7.322482	3492	2373.09	MS2	791.702	44	Glioma_grade2_6.raw
238624147			aEDSGEYHcVYHFVSAPk	Unambiguous	N-Term(iTRAQ4plex); C9(Methylthio); K18(iTRAQ4plex)	HCD	0.45389405	0	1	4	0.36538	0.487	0.429	0.57187176	0.189441295	51948.6	38973.203	18981.191	22287.674	20.18215852	11.33075286	4	High	3485	Unique	Used	80779	1	38.614	1	4.4752	1.8599	1	5	24/102	8.631803	3485	2373.09	MS2	594.029	55	Glioma_grade2_6.raw
238624147			aEDSGEYHcVYHFVSAPk	Unambiguous	N-Term(iTRAQ4plex); C9(Methylthio); K18(iTRAQ4plex)	HCD	0.46125072	0	1	4	0.48379	0.4277	0.4992	0.44132054	0.073302221	47405.805	53620.457	22934.627	23663.809	8.699564816	2.21299074	2	High	3528	Unique	Used	80816	1	38.987	1	6.0389	0.2766	1	48	21/34	5.941936	3528	2373.09	MS2	1187.05	45	Glioma_grade2_6.raw
238624147			sVGYPHPDWIWR	Unambiguous	N-Term(iTRAQ4plex)	HCD	0.3532196	0	1	4	0.37569	0.339	0.369	0.33294746	0.06022901	46012.805	50997.45	17286.451	16979.47	7.266605538	1.266963446	3	High	4784	Unique	Used	75226	1	49.154	1	4.3536	-2.2889	1	26	25/44	37.97303	4784	1656.84	MS2	552.95	163	Glioma_grade2_5.raw
238624147			kRPDEVPDAGPMk	Unambiguous	N-Term(iTRAQ4plex); K1(iTRAQ4plex); K13(iTRAQ4plex)	HCD	0.52615994	1	1	2	0.46777	0.548	0.5075	0.5945661	0.102700866	43420.246	37062.66	20310.68	22036.201	11.17129685	5.762538215	3	High	1142	Unique	Used	84759	1	20.495	1	4.1458	-3.0768	1	38	22/48	22.70985	1142	1872.03	MS2	624.682	174	Glioma_grade2_1rep.raw
238624147			aEDSGEYHcVYHFVSAPk	Unambiguous	N-Term(iTRAQ4plex); C9(Methylthio); K18(iTRAQ4plex)	HCD	0.4844152	0	1	4	0.51981	0.5079	0.4603	0.4498302	0.071328725	29153.973	29834.568	15154.456	13420.489	1.63168416	8.581642583	4	High	3486	Unique	Used	86393	1	47.452	1	5.5939	-0.712	1	15	22/102	22.3671	3486	2373.09	MS2	594.027	50	Glioma_grade2_1rep.raw
238624147			kRPDEVPDAGPmk	Unambiguous	N-Term(iTRAQ4plex); K1(iTRAQ4plex); M12(Oxidation); K13(iTRAQ4plex)	HCD	0.34800538	1	1	2	0.3196	0.3539	0.3427	0.37945482	0.071264029	26579.988	24007.088	8494.97	9109.6045	7.192805678	4.937491933	3	High	965	Unique	Used	94043	1	18.421	1	4.0795	-6.9846	1	81	17/48	41.4062	965	1888.02	MS2	630.011	107	Glioma_grade2_8rep.raw

238624147			aEDSGEYHcVYHFVSAPk	Unambiguous	N-Term(iTRAQ4plex); C9(Methylthio); K18(iTRAQ4plex)	HCD	0.51390064	0	1	4	0.49827	0.4911	0.537	0.5293106	0.043985373	25664.984	26037.355	12788.018	13781.848	1.018546024	5.289781532	3	High	3483	Unique	Used	86391	1	47.413	1	7.8336	2.0713	1	69	29/68	27.73041	3483	2373.09	MS2	791.703	60	Glioma_grade 2_1rep.raw
238624147			kRPDEVPDAGPMk	Unambiguous	N-Term(iTRAQ4plex); K1(iTRAQ4plex); K13(iTRAQ4plex)	HCD	0.3794532	1	1	2	0.38546	0.4071	0.3532	0.3731088	0.05946699	21248.656	20117.084	8190.5015	7505.8613	3.868622849	6.168482906	3	High	1225	Unique	Used	94255	1	21.012	1	4.4105	-5.0331	1	89	24/48	43.14562	1225	1872.03	MS2	624.681	185	Glioma_grade 2_8rep.raw
238624147			kRPDEVPDAGPMk	Unambiguous	N-Term(iTRAQ4plex); K1(iTRAQ4plex); K13(iTRAQ4plex)	HCD	0.49743277	1	1	2	0.45767	0.4848	0.5094	0.53954756	0.070114604	20160.45	19032.307	9226.927	10268.834	4.070739731	7.557945597	4	High	1230	Unique	Used	94259	1	21.067	1	3.8252	-4.8446	1	45	18/72	53.52655	1230	1872.03	MS2	468.763	211	Glioma_grade 2_8rep.raw
238624147			rPDEVPDAGPMk	Unambiguous	N-Term(iTRAQ4plex); K12(iTRAQ4plex)	HCD	0.5479279	0	1	2	0.63121	0.6106	0.4832	0.46736446	0.15462905	17232.549	17814.922	10877.426	8326.062	2.349954994	18.78915732	3	High	1135	Unique	Used	78641	1	20.261	1	6.2058	-0.4266	1	43	23/44	27.68264	1135	1599.84	MS2	533.951	261	Glioma_grade 2_6.raw
238624147			aEDSGEYHcVYHFVSAPk	Unambiguous	N-Term(iTRAQ4plex); C9(Methylthio); K18(iTRAQ4plex)	HCD	0.46460295	0	1	4	0.38755	0.4126	0.5135	0.5466245	0.165446319	8563.1	8043.936	3318.6658	4397.012	4.421070502	19.76510503	2	High	3587	Unique	Used	95847	1	49.463	1	4.5248	3.1572	1	100	19/34	0	3587	2373.1	MS2	1187.05	49	Glioma_grade 2_8rep.raw
238624147			aEDSGEYHcVYHFVSAPk	Unambiguous	N-Term(iTRAQ4plex); C9(Methylthio); K18(iTRAQ4plex)	HCD	0.6381344	0	1	4	0.61796	0.5888	0.6899	0.65735567	0.069423388	7062.7856	7412.808	4364.523	4872.8516	3.419593275	7.782354093	3	High	3563	Unique	Used	95833	1	49.142	1	7.6264	-1.9409	1	100	31/68	25.0493	3563	2373.08	MS2	791.699	54	Glioma_grade 2_8rep.raw
238624147			aAPDITGHk	Unambiguous	N-Term(iTRAQ4plex); K9(iTRAQ4plex)	HCD	0.60960376	0	1	4	0.58963	0.5868	0.6325	0.6294841	0.040632386	5742.804	5770.608	3386.113	3632.5063	0.341521644	4.964690798	3	High	932	Unique	Used	78475	1	18.348	1	3.2171	-1.8173	1	100	17/32	10.14225	932	1197.68	MS2	399.898	206	Glioma_grade 2_6.raw
255652944	NP_000262.2	NPC1	Niemann-Pick C1 protein precursor [Homo sapiens]	2	3	2.357508898	23.99211945	16.622955	3.6	2.55E-04	1278	142.07	5.3638	Cell surface receptor	Cell communication,Signal transduction	Receptor activity	Cytoplasm	Plasma membrane, Endoplasmic reticulum, Lysosome																								
255652944			aLGLLcGk	Unambiguous	N-Term(iTRAQ4plex); C6(Methylthio); K8(iTRAQ4plex)	HCD	3.1577206	0	1	1	3.0348	3.0859	3.2284	3.282709	3.69682418	67234.64	66121.98	204043.73	217059.2	1.179948069	4.37105821	2	High	5392	Unique	Used	68528	1	51.822	1	3.4058	0.263	1	56	14/14	31.93316	5392	1108.65	MS2	554.827	335	Glioma_grade 2_4.raw
255652944			aLGLLcGk	Unambiguous	N-Term(iTRAQ4plex); C6(Methylthio); K8(iTRAQ4plex)	HCD	2.357509	0	1	1	2.17283	2.4619	2.2654	2.5667598	7.595023186	46443.57	40990.574	100913.83	105212.95	8.820010749	2.949579773	2	High	5242	Unique	Used	60723	1	51.217	1	3.1411	-0.9482	1	100	14/14	0	5242	1108.65	MS2	554.826	334	Glioma_grade 2_3.raw
255652944			eYFDQHFGPFFR	Unambiguous	N-Term(iTRAQ4plex)	HCD	1.9838173	0	1	1	1.89458	2.0563	1.917	2.0806725	4.775103965	13548.975	12483.447	25669.604	25973.967	5.788490017	0.833471184	3	High	5685	Unique	Used	76036	1	56.406	1	4.0608	-2.8839	1	25	23/44	10.09782	5685	1733.81	MS2	578.61	54	Glioma_grade 2_5.raw
256985102	NP_001157941.1	TMED7-TICAM2	TRAM adaptor with GOLD domain isoform 2 precursor [Homo sapiens]	2	6	2.006566525	12.99788869	51.689275	22.34	1.34E-04	188	21.219	6.2017	-	-	-	-	-																								
256985102			sVIDYQTHFR	Unambiguous	N-Term(iTRAQ4plex)	HCD	2.1451144	0	1	3	2.10755	2.1517	2.1386	2.1834648	1.465878654	130140.23	127469.01	274277.2	278324.06	1.466436356	1.035669788	2	High	2210	Unique	Used	65578	1	27.229	1	3.0891	15.509	1	18	16/18	12.18642	2210	1409.75	MS2	705.379	236	Glioma_grade 2_4.raw
256985102			sVIDYQTHFR	Unambiguous	N-Term(iTRAQ4plex)	HCD	1.8465136	0	1	3	1.6152	1.8911	1.8084	2.1173475	11.19382062	115913.73	99000.94	187223.47	209619.38	11.1292063	7.981144089	2	High	2257	Unique	Used	65622	1	27.57	1	3.4523	1.395	1	16	16/18	3.447037	2257	1409.73	MS2	705.369	212	Glioma_grade 2_4.raw
256985102			sVIDYQTHFR	Unambiguous	N-Term(iTRAQ4plex)	HCD	2.020026	0	1	3	1.86732	2.0053	2.0337	2.1840186	6.420314338	107385.11	99993.92	200522.42	218388.6	5.04039446	6.031494245	2	High	1934	Unique	Used	57647	1	25.893	1	3.6846	0.4425	1	40	16/18	0	1934	1409.73	MS2	705.369	270	Glioma_grade 2_3.raw
256985102			sVIDYQTHFR	Unambiguous	N-Term(iTRAQ4plex)	HCD	1.5989074	0	1	3	1.52007	1.6392	1.5616	1.6839242	4.622674342	50309.824	46654.74	76474.305	78563.05	5.330884966	1.905303085	3	High	2176	Unique	Used	65546	1	26.997	1	4.9466	0.1515	1	16	16/36	19.76998	2176	1409.73	MS2	470.581	244	Glioma_grade 2_4.raw
256985102			vSALTQMESAcVSIHEALk	Unambiguous	N-Term(iTRAQ4plex); C11(Methylthio); K19(iTRAQ4plex)	HCD	1.9931072	0	1	3	2.22267	1.6683	2.4259	1.8208046	17.22455746	27444.984	36565.39	61001.105	66578.43	20.15017419	6.182440355	3	High	7487	Unique	Used	77587	1	72.279	1	8.9433	-1.9262	1	7	39/72	3.410463	7487	2351.2	MS2	784.404	159	Glioma_grade 2_5.raw
256985102			vSALTQMESAcVSIHEALk	Unambiguous	N-Term(iTRAQ4plex); C11(Methylthio); K19(iTRAQ4plex)	HCD	2.360779	0	1	3	2.10716	2.2986	2.4178	2.6374395	9.387663106	7616.6753	6982.3936	16049.537	18415.64	6.144294466	9.708857589	3	High	7559	Unique	Used	77641	1	73.046	1	9.5209	0.3322	1	35	41/72	7.640047	7559	2351.2	MS2	784.406	143	Glioma_grade 2_5.raw
258613943	NP_001158222.1	ASPH	aspartyl/asparaginyl beta-hydroxylase isoform f [Homo sapiens]	4	4	2.626475096	26.61076329	26.149893	10.29	1.49E-04	729	83.216	4.9194	Enzyme: Hydroxylase	Protein metabolism	Catalytic activity	Endoplasmic reticulum	-																								
258613943			lGIYDADGDGDFDVDDAk	Unambiguous	N-Term(iTRAQ4plex); K18(iTRAQ4plex)	HCD	2.6739306	0	1	12	2.63345	2.7356	2.6145	2.7159777	2.236507727	64274.45	61873.82	169263.81	168047.92	2.691280272	0.509774187	2	High	5744	Unique	Used	53184	1	53.833	1	6.2229	0.0939	1	28	27/34	0	5744	2189.01	MS2	1095.01	74	Glioma_grade 2_2.raw
258613943			fYFHLGDAMQR	Unambiguous	N-Term(iTRAQ4plex)	HCD	1.5986625	0	1	2	1.6403	1.593	1.6045	1.5582256	2.116355532	22318.23	22981.58	36608.617	35810.49	2.070910599	1.558596998	2	High	4624	Unique	Used	75089	1	47.747	1	3.2037	0.4271	1	100	20-Dec	41.66917	4624	1528.75	MS2	764.878	142	Glioma_grade 2_5.raw
258613943			vLIFDDSFEHEVWQDASSFR	Unambiguous	N-Term(iTRAQ4plex)	HCD	3.20293	0	1	2	3.49793	2.836	3.6554	2.963751	12.33403162	19144.328	23612.244	66965.5	69980.81	14.77804957	3.113842422	3	High	8196	Unique	Used	71024	1	75.636	1	6.4666	1.446	1	23	28/76	18.25643	8196	2571.23	MS2	857.749	65	Glioma_grade 2_4.raw
258613943			lIFIVDVWHPELTPQQR	Unambiguous	N-Term(iTRAQ4plex)	HCD	2.5790195	0	1	2	2.27344	2.7095	2.4695	2.9432151	11.18383352	10070.103	8449.414	22893.752	24868.443	12.37613467	5.84695656	3	High	7288	Unique	Used	77425	1	70.365	1	5.9555	-2.6068	1	39	28/64	20.66258	7288	2235.23	MS2	745.75	108	Glioma_grade 2_5.raw
262118282	NP_115618.3	PLXNA1	plexin-A1 precursor [Homo sapiens]	6	6	0.477390826	15.46519124	62.513228	8.28	1.74E-04	1896	210.93	6.9175	Cell surface receptor	Cell communication,Signal transduction	Receptor activity	Plasma membrane	-																								
262118282			vLFPGIEDHPVLk	Unambiguous	N-Term(iTRAQ4plex); K13(iTRAQ4plex)	HCD	0.4715217	0	1	1	0.43747	0.454	0.4884	0.5068623	0.067069174	116674.39	112431.51	51041.113	56987.29	2.619023971	7.784215932	3	High	4731	Unique	Used	81839	1	49.747	1	5.3623	-1.6008	1	20	21/48	30.96383	4731	1752.03	MS2	584.681	117	Glioma_grade 2_6.raw
262118282			lSLPWLLNk	Unambiguous	N-Term(iTRAQ4plex); K9(iTRAQ4plex)	HCD	0.37505862	0	1	1	0.35454	0.3991	0.3537	0.39815328	0.068329626	108544.48	96418.39	38483.797	38389.297	8.366823189	0.173849099	2	High	7201	Unique	Used	70172	1	66.549	1	4.1676	2.1427	1	98	14/16	31.57071	7201	1371.86	MS2	686.435	81	Glioma_grade 2_4.raw
262118282			sPPELGERPDELGFVMDNVR	Unambiguous	N-Term(iTRAQ4plex)	HCD	0.3816874	0	1	1	0.38399	0.4171	0.3491	0.37918836	0.072864407	69668.63	64135.88	26751.918	24319.578	5.847702807	6.735358244	3	High	5967	Unique	Used	61393	1	56.913	1	3.6347	0.7832	1	22	20/76	6.783602	5967	2401.2	MS2	801.07	89	Glioma_grade 2_3.raw
262118282			lNTLAHYQVTDGSSVALVPk	Unambiguous	N-Term(iTRAQ4plex); K20(iTRAQ4plex)	HCD	0.48325998	0	1	1	0.45624	0.4861	0.4806	0.5120543	0.04735135	20595.537	19328.436	9396.449	9897.209	4.488409556	3.670540773	3	High	4727	Unique	Used	75175	1	48.692	1	6.7204	1.747	1	24	27/76	24.13047	4727	2401.34	MS2	801.117	110	Glioma_grade 2_5.raw
262118282			kHFLLTFIR	Unambiguous	N-Term(iTRAQ4plex); K1(iTRAQ4plex)	HCD	0.5352773	1	1	1	0.4948	0.5557	0.5171	0.58072937	0.071606057	15914.005	14170.657	7874.3193	8229.317	8.195094183	3.117572656	3	High	3701	Unique	Used	95915	1	50.959	1	3.4012	-4.5667	1	100	15/32	24.5122	3701	1462.91	MS2	488.307	104	Glioma_grade 2_8rep.raw
262118282			aLAHQLGLAEDEDVLFTVFAQGQk	Unambiguous	N-Term(iTRAQ4plex); K24(iTRAQ4plex)	HCD	0.5387048	0	1	1	0.54531	0.5257	0.5522	0.5323381	0.02235743	6287.966	6522.5913	3428.885	3472.2239	2.590131511	0.888124808	3	High	7116	Unique	Used	83779	1	72.458	1	7.3785	-2.5707	1	58	24/92	25.44632	7116	2888.53	MS2	963.516	135	Glioma_grade 2_6.raw
283135173	NP_001164408.1	FUS	RNA-binding protein FUS isoform 3 [Homo sapiens]	2	6	2.103490829	23.8786984	37.850828	9.39	3.99E-04	522	53.166	9.3638	RNA binding protein	RNA localization	Nucleocytoplasmic transporter activity	Nucleus	Mitochondrion, Cytoplasm, Nucleolus																								
283135173			lkGEATVSFDDPPSAk	Unambiguous	N-Term(iTRAQ4plex); K2(iTRAQ4plex); K16(iTRAQ4plex)	HCD	1.5698588	1	1	3	1.80971	1.7888	1.3484	1.3327795	16.88137657	39098.15	39555.895	70756.45	52719.285	0.823033561	20.65863659	3	High	2241	Unique	Used	79669	1	28.47	1	5.9962	3.7946	1	8	25/60	7.003011	2241	2094.15	MS2	698.722	175	Glioma_grade 2_6.raw
283135173			lkGEATVSFDDPPSAk	Unambiguous	N-Term(iTRAQ4plex); K2(iTRAQ4plex); K16(iTRAQ4plex)	HCD	2.703092	1	1	3	2.50943	3.0053	2.4507	2.9350235	10.47388824	28883.568	24117.479	72481.34	70785.37	12.71723501	1.674124977	2	High	2234	Unique	Used	79663	1	28.403	1	4.4166	4.0634	1	44	19/30	4.7768	2234	2094.15	MS2	1047.58	170	Glioma_grade 2_6.raw
283135173			aPkPDGPGGGPGGSHMGGNYGDDR	Unambiguous	N-Term(iTRAQ4plex); K3(iTRAQ4plex)	HCD	2.162673	0	1	3	2.08532	2.5658	1.835	2.2578511	14.04381948	8014.1904	6513.3794	16712.14	14706.24	14.60992651	9.029017361	4	High	1090	Unique	Used	84716	1	20.006	1	4.9231	0.3252	1	33	28/138	8.888106	1090	2541.18	MS2	636.05	59	Glioma_grade 2_1rep.raw
283135173			aPkPDGPGGGPGGSHMGGNYGDDR	Unambiguous	N-Term(iTRAQ4plex); K3(iTRAQ4plex)	HCD	2.0443087	0	1	3	2.00446	2.3469	1.7858	2.0909617	11.27901732	7215.777	6162.854	14463.761	12886.292	11.13012226	8.156759528	3	High	1106	Unique	Used	84729	1	20.158	1	6.3334	0.2584	1	47	27/92	7.614469	1106	2541.18	MS2	847.731	45	Glioma_grade 2_1rep.raw
283135173			aPkPDGPGGGPGGSHmGGNYGDDR	Unambiguous	N-Term(iTRAQ4plex); K3(iTRAQ4plex); M16(Oxidation)	HCD	3.0440714	0	1	3	2.98949	3.0948	2.9951	3.100581	2.00127742	3010.445	2908.0186	8999.68	9016.547	2.447473091	0.132400309	3	High	897	Unique	Used	84568	1	18.012	1	6.6385	1.8635	1	100	21/92	4.209218	897	2557.18	MS2	853.064	51	Glioma_grade 2_1rep.raw
283135173			aPkPDGPGGGPGGSHMGGNYGDDR	Unambiguous	N-Term(iTRAQ4plex); K3(iTRAQ4plex)	HCD	1.9506346	0	1	3	1.58198	1.5845	2.3162	2.3198726	21.74933715	2575.959	2571.898	4075.1118	5966.4756	0.111563341	26.63714641	4	High	1224	Unique	Used	94254	1	21.008	1	5.5826	-0.1552	1	54	28/138	11.29223	1224	2541.18	MS2	636.05	61	Glioma_grade 2_8rep.raw
284807150	NP_001165282.1	GBA	glucosylceramidase isoform 2 [Homo sapiens]	3	4	2.31124258	18.83766678	20.055125	8.91	2.48E-04	449	50.285	7.1519	Enzyme: Glucosidase	Lipid metabolism	Glucosidase activity	Lysosome	Endosome, Vesicle																								
284807150			nFVDSPIIVDITk	Unambiguous	N-Term(iTRAQ4plex); K13(iTRAQ4plex)	HCD	1.9811704	0	1	3	1.78642	2.0266	1.9412	2.202099	8.715133676	75177.69	66269.22	134299.03	145931.39	8.906860598	5.870398108	2	High	6567	Unique	Used	53961	1	59.812	1	5.2678	1.1822	1	53	22/24	49.53155	6567	1749.01	MS2	875.007	199	Glioma_grade 2_2.raw
284807150			fLDAYAEHk	Unambiguous	N-Term(iTRAQ4plex); K9(iTRAQ4plex)	HCD	1.9111288	0	1	3	1.62533	1.9838	1.8516	2.2599564	13.73112778	39742.812	32562.055	64595.09	73588.82	14.04486912	9.204439896	3	High	2133	Unique	Used	72829	1	28.066	1	4.1725	-0.3317	1	20	21/32	40.78876	2133	1381.74	MS2	461.25	194	Glioma_grade 2_5.raw
284807150			lLLPHWAk	Unambiguous	N-Term(iTRAQ4plex); K8(iTRAQ4plex)	HCD	2.6413145	0	1	3	2.48528	2.7317	2.559	2.812824	5.710980982	27285.105	24823.389	67811.1	69823.83	6.681045424	2.068101508	3	High	3693	Unique	Used	91248	1	41.853	1	3.536	-3.1761	1	41	19/28	0	3693	1265.79	MS2	422.603	187	Glioma_grade 2_7rep.raw
284807150			lLLPHWAk	Unambiguous	N-Term(iTRAQ4plex); K8(iTRAQ4plex)	HCD	2.7545004	0	1	3	2.65966	2.7015	2.8067	2.8508358	3.234106541	15666.232	15423.663	41666.8	43970.33	1.103395073	3.804054815	3	High	3780	Unique	Used	91305	1	42.896	1	3.6008	-0.2829	1	77	19/28	15.91983	3780	1265.8	MS2	422.604	153	Glioma_grade 2_7rep.raw
296010971	NP_001171582.1	SCAMP5	secretory carrier-associated membrane protein 5 [Homo sapiens]	3	13	0.45443821	13.62024441	52.034683	19.15	3.68E-04	235	26.087	8.5435	Integral membrane protein	Transport	Transporter activity	Cytoplasmic vesicle	-																								
296010971			vNNFPPLPk	Unambiguous	N-Term(iTRAQ4plex); K9(iTRAQ4plex)	HCD	0.4544382	0	1	1	0.4143	0.4705	0.4403	0.5000204	0.081340855	878581.6	773664.94	363995.72	386848.22	8.980170931	4.304265336	2	High	3505	Unique	Used	66783	1	37.038	1	3.7305	1.1247	1	12	14/16	28.3572	3505	1313.78	MS2	657.395	161	Glioma_grade 2_4.raw
296010971			vNNFPPLPk	Unambiguous	N-Term(iTRAQ4plex); K9(iTRAQ4plex)	HCD	0.44783774	0	1	1	0.39768	0.446	0.4495	0.5040955	0.096829525	771253.25	687682.6	306708.8	346657.72	8.10088714	8.646954311	2	High	2986	Unique	Used	73625	1	34.358	1	3.7903	1.5892	1	10	14/16	32.40614	2986	1313.78	MS2	657.396	157	Glioma_grade 2_5.raw
296010971			aQEEWTTGAWk	Unambiguous	N-Term(iTRAQ4plex); K11(iTRAQ4plex)	HCD	0.3911576	0	1	1	0.37895	0.3869	0.3954	0.40362167	0.027243553	648015.44	634777	245564.23	256209.75	1.459470824	3.000362586	2	High	3663	Unique	Used	51224	1	38.651	1	4.6606	2.4011	1	17	19/20	19.02102	3663	1594.81	MS2	797.911	170	Glioma_grade 2_2.raw
296010971			aQEEWTTGAWk	Unambiguous	N-Term(iTRAQ4plex); K11(iTRAQ4plex)	HCD	0.38236257	0	1	1	0.40688	0.3686	0.3975	0.36015356	0.058518784	538222.5	594072.06	218989.9	213957.17	6.975499839	1.643931906	2	High	3620	Unique	Used	59215	1	38.621	1	4.8181	1.406	1	19	19/20	10.1183	3620	1594.81	MS2	797.91	153	Glioma_grade 2_3.raw
296010971			aQEEWTTGAWk	Unambiguous	N-Term(iTRAQ4plex); K11(iTRAQ4plex)	HCD	0.43638402	0	1	1	0.38397	0.4763	0.4042	0.5014017	0.127430764	469312.66	378370.94	180199.75	189715.83	15.17205403	3.638065041	2	High	3761	Unique	Used	51317	1	39.353	1	4.3292	4.6208	1	16	19/20	11.16332	3761	1594.82	MS2	797.913	155	Glioma_grade 2_2.raw
296010971			vNNFPPLPk	Unambiguous	N-Term(iTRAQ4plex); K9(iTRAQ4plex)	HCD	0.49982443	0	1	1	0.4356	0.5091	0.4919	0.5748847	0.11406743	405425.3	346888.2	176603.97	199420.72	11.00391801	8.581286856	2	High	3239	Unique	Used	58862	1	35.664	1	3.404	-4.2644	1	33	13/16	52.3017	3239	1313.78	MS2	657.392	173	Glioma_grade 2_3.raw
296010971			vNNFPPLPk	Unambiguous	N-Term(iTRAQ4plex); K9(iTRAQ4plex)	HCD	0.5045138	0	1	1	0.48308	0.5185	0.4915	0.5275211	0.042006692	246977.03	230120.33	119308.88	121393.32	4.996668554	1.224684825	2	High	3088	Unique	Used	73720	1	35.12	1	3.6666	4.6554	1	27	14/16	35.56971	3088	1313.79	MS2	657.398	165	Glioma_grade 2_5.raw
296010971			vNNFPPLPk	Unambiguous	N-Term(iTRAQ4plex); K9(iTRAQ4plex)	HCD	0.57102203	0	1	1	0.55645	0.5894	0.5537	0.58645123	0.033280945	222278.97	209869.44	123688.06	123078.2	4.061041351	0.349512681	2	High	3407	Unique	Used	66695	1	36.21	1	3.6927	-2.0345	1	45	14/16	32.15538	3407	1313.78	MS2	657.393	160	Glioma_grade 2_4.raw
296010971			vNNFPPLPk	Unambiguous	N-Term(iTRAQ4plex); K9(iTRAQ4plex)	HCD	0.5008303	0	1	1	0.47519	0.4886	0.5127	0.52718884	0.046730426	147330.14	143292.98	70010.4	75542.46	1.96453965	5.375033015	2	High	2907	Unique	Used	73556	1	33.677	1	3.9103	0.8459	1	40	13/16	27.45179	2907	1313.78	MS2	657.395	175	Glioma_grade 2_5.raw
296010971			vNNFPPLPk	Unambiguous	N-Term(iTRAQ4plex); K9(iTRAQ4plex)	HCD	0.44961336	0	1	1	0.4087	0.4414	0.4572	0.49380448	0.078508243	51012.383	47228.875	20848.75	23321.83	5.44647782	7.918083206	3	High	3476	Unique	Used	66757	1	36.788	1	3.5303	-3.5081	1	51	16/32	68.82169	3476	1313.78	MS2	438.597	167	Glioma_grade 2_4.raw
296010971			vNNFPPLPk	Unambiguous	N-Term(iTRAQ4plex); K9(iTRAQ4plex)	HCD	0.5067412	0	1	1	0.44256	0.5117	0.5024	0.58095413	0.11128638	24488.732	21179.52	10837.656	12304.33	10.2476716	8.962887897	3	High	3015	Unique	Used	73652	1	34.573	1	3.2606	-1.9053	1	97	13/32	74.18102	3015	1313.78	MS2	438.598	161	Glioma_grade 2_5.raw
296010971			fIPLkPcFYQDFEADIPPQHVSMTk	Unambiguous	N-Term(iTRAQ4plex); K5(iTRAQ4plex); C7(Methylthio); K25(iTRAQ4plex)	HCD	0.5342498	0	1	1	0.50832	0.4617	0.6142	0.5577988	0.122328939	12972.239	14282.919	6594.0684	7966.995	6.800846401	13.33426938	4	High	5272	Unique	Used	87608	1	68.384	1	4.7844	-1.8396	1	39	24/144	58.70889	5272	3429.73	MS2	858.189	81	Glioma_grade 2_1rep.raw

296010971			fIPLkPcFYQDFEADIPPQHVSmTk	Unambiguous	N-Term(iTRAQ4plex); K5(iTRAQ4plex); C7(Methylthio); M23(Oxidation); K25(iTRAQ4plex)	HCD	0.3537732	0	1	1	0.40156	0.3591	0.3478	0.3110377	0.105044793	4377.445	4895.1475	1757.8192	1522.5754	7.895762666	10.14161444	4	High	4984	Unique	Used	96643	1	68.298	1	4.457	3.8958	1	100	28/144	30.40436	4984	3445.75	MS2	862.193	96	Glioma_grade2_8rep.raw
296040505	NP_001171649.1	GYG1	glycogenin-1 isoform 2 [Homo sapiens]	3	6	1.999952435	24.30339692	25.862375	14.71	4.06E-04	333	37.455	5.4019	Unclassified	Metabolism,Energy pathways	Molecular function unknown	-	-																								
296040505			gALVLGSSLk	Unambiguous	N-Term(iTRAQ4plex); K10(iTRAQ4plex)	HCD	2.2533345	0	1	3	2.16259	2.0494	2.4685	2.339329	8.238182919	161078.47	169975.94	348346.7	397629.66	3.800862446	9.343007924	2	High	2960	Unique	Used	58600	1	33.602	1	3.3131	-2.9897	1	17	15/18	38.51852	2960	1232.78	MS2	616.893	146	Glioma_grade2_3.raw
296040505			rPELGVTLTk	Unambiguous	N-Term(iTRAQ4plex); K10(iTRAQ4plex)	HCD	3.0735135	0	1	3	2.89171	3.1506	3.0028	3.2715905	5.405000596	102915.695	94460.2	297602.4	309035.1	6.058427604	2.665229135	2	High	1580	Unique	Used	79057	1	23.493	1	3.7112	-8.3227	1	24	15/18	20.79545	1580	1401.86	MS2	701.431	127	Glioma_grade2_6.raw
296040505			rPELGVTLTk	Unambiguous	N-Term(iTRAQ4plex); K10(iTRAQ4plex)	HCD	1.9362875	0	1	3	1.8232	1.8556	2.0156	2.05138	5.875278648	87476.11	85949.18	159486.8	176314.42	1.245150067	7.086885636	3	High	1622	Unique	Used	79096	1	23.8	1	3.8904	11.739	1	11	14/36	20.46484	1622	1401.88	MS2	467.966	62	Glioma_grade2_6.raw
296040505			gALVLGSSLk	Unambiguous	N-Term(iTRAQ4plex); K10(iTRAQ4plex)	HCD	2.0486338	0	1	3	1.91476	1.9884	2.1067	2.1876564	5.925912101	72231.85	69557.484	138306.55	152167.89	2.667425371	6.748578299	2	High	2858	Unique	Used	58505	1	32.826	1	3.5888	0.8721	1	52	16/18	54.59956	2858	1232.78	MS2	616.895	109	Glioma_grade2_3.raw
296040505			rPELGVTLTk	Unambiguous	N-Term(iTRAQ4plex); K10(iTRAQ4plex)	HCD	1.951271	0	1	3	1.64046	2.0733	1.8547	2.344092	15.22856884	31986.775	25308.78	52473.055	59326.1	16.48314795	8.668821497	3	High	1571	Unique	Used	79049	1	23.422	1	4.3855	-9.1595	1	30	19/36	60.67572	1571	1401.85	MS2	467.956	119	Glioma_grade2_6.raw
296040505			wEQGQADYMGADSFDNIk	Unambiguous	N-Term(iTRAQ4plex); K18(iTRAQ4plex)	HCD	1.5292102	0	1	2	1.62599	1.3306	1.7719	1.4500159	12.56745975	8804.264	10758.857	14315.61	15600.515	14.12970829	6.07408238	3	High	5826	Unique	Used	53261	1	54.44	1	3.2318	-0.6788	1	38	17/68	24.78835	5826	2363.08	MS2	788.366	60	Glioma_grade2_2.raw
299523086	NP_006001.3	MANF	mesencephalic astrocyte-derived neurotrophic factor precursor [Homo sapiens]	3	8	2.177510262	19.77971861	47.375515	28.57	3.72E-04	182	20.687	8.6899	Unclassified	Biological_process unknown	Molecular function unknown	Cytoplasm	Nucleolus, Nucleus																								
299523086			kINELMPk	Unambiguous	N-Term(iTRAQ4plex); K1(iTRAQ4plex); K8(iTRAQ4plex)	HCD	2.156555	1	1	1	1.9915	2.1492	2.1634	2.3346786	6.495282486	95930.12	88892.266	191044.75	207534.88	5.385185633	5.850917542	2	High	1685	Unique	Used	89548	1	24.087	1	3.2	-3.0575	1	42	13/14	22.57561	1685	1404.86	MS2	702.932	186	Glioma_grade2_7rep.raw
299523086			iLDDWGETck	Unambiguous	N-Term(iTRAQ4plex); C9(Methylthio); K10(iTRAQ4plex)	HCD	1.8975853	0	1	1	1.75334	1.9708	1.8324	2.059725	7.210070447	62341.465	55461.387	109305.766	114235.2	8.259477129	3.118565936	2	High	6110	Unique	Used	53530	1	56.492	1	3.4082	-0.7139	1	43	17/18	17.66915	6110	1513.73	MS2	757.367	82	Glioma_grade2_2.raw
299523086			iLDDWGETck	Unambiguous	N-Term(iTRAQ4plex); C9(Methylthio); K10(iTRAQ4plex)	HCD	2.1984658	0	1	1	2.17964	2.1383	2.2597	2.216935	2.358139168	48276.414	49208.64	105225.22	109092.35	1.352378235	2.55179624	2	High	5912	Unique	Used	61341	1	56.492	1	4.3801	-0.0687	1	69	17/18	28.5061	5912	1513.73	MS2	757.367	82	Glioma_grade2_3.raw
299523086			iINEVSkPLAHHIPVEk	Unambiguous	N-Term(iTRAQ4plex); K7(iTRAQ4plex); K17(iTRAQ4plex)	HCD	1.8417027	0	1	1	1.61998	1.8469	1.8372	2.0944827	10.48618321	10047.021	8812.678	16275.957	18458	9.255845553	8.884316878	3	High	3028	Unique	Used	86122	1	41.443	1	5.0227	-0.018	1	88	30/64	0	3028	2356.41	MS2	786.141	32	Glioma_grade2_1rep.raw
299523086			iINEVSkPLAHHIPVEk	Unambiguous	N-Term(iTRAQ4plex); K7(iTRAQ4plex); K17(iTRAQ4plex)	HCD	2.4754558	0	1	1	1.80491	2.6737	2.3416	3.4687834	27.08428332	6614.8706	4465.3857	11939.229	15489.455	27.43466049	18.30484379	4	High	3023	Unique	Used	86120	1	41.363	1	4.2336	1.7117	1	31	24/96	37.88867	3023	2356.41	MS2	589.858	28	Glioma_grade2_1rep.raw
299523086			iINEVSkPLAHHIPVEk	Unambiguous	N-Term(iTRAQ4plex); K7(iTRAQ4plex); K17(iTRAQ4plex)	HCD	1.8117542	0	1	1	1.62353	2.0698	1.6093	2.0517128	13.96229471	6439.1016	5050.718	10454.105	10362.623	17.08878803	0.621495775	3	High	3169	Unique	Used	95599	1	43.975	1	5.8807	1.2253	1	100	27/64	30.63902	3169	2356.41	MS2	786.142	30	Glioma_grade2_8rep.raw
299523086			iINEVSkPLAHHIPVEk	Unambiguous	N-Term(iTRAQ4plex); K7(iTRAQ4plex); K17(iTRAQ4plex)	HCD	3.0457664	0	1	1	2.57665	3.4654	2.7337	3.6766903	17.33244314	4183.7153	3110.737	10779.98	11437.218	20.80239066	4.183582886	5	High	3036	Unique	Used	86128	1	41.528	1	5.2637	-0.6381	1	96	21/128	14.9284	3036	2356.41	MS2	472.087	41	Glioma_grade2_1rep.raw
299523086			iINEVSkPLAHHIPVEk	Unambiguous	N-Term(iTRAQ4plex); K7(iTRAQ4plex); K17(iTRAQ4plex)	HCD	2.7365508	0	1	1	2.42192	2.6592	2.807	3.0820098	10.07681161	2455.8235	2236.6567	5947.812	6893.3984	6.605220433	10.41382447	5	High	3157	Unique	Used	95591	1	43.826	1	6.012	-1.1561	1	100	22/128	60.73848	3157	2356.41	MS2	472.087	41	Glioma_grade2_8rep.raw
302344767	NP_001180546.1	SCP2	non-specific lipid-transfer protein isoform 8 proprotein [Homo sapiens]	5	12	2.682611227	21.80128566	76.289082	17.6	2.65E-05	466	50.309	7.02	Membrane transport protein	Transport,Lipid metabolism	Auxiliary transport protein activity,Sterol transporter activity	Cytoplasm	Soluble fraction, Lysosome, Microsome, Endoplasmic reticulum, Peroxisome, Mitochondrion, Plasma membrane																								
302344767			iGGIFAFk	Unambiguous	N-Term(iTRAQ4plex); K8(iTRAQ4plex)	HCD	3.257171	0	1	6	3.32087	3.2284	3.2867	3.1952448	1.735393263	270107.6	277840.28	896992.1	887767.75	1.995748378	0.730921915	2	High	5297	Unique	Used	75686	1	53.284	1	3.4707	-3.1257	1	12	14/14	33.83057	5297	1140.7	MS2	570.853	156	Glioma_grade2_5.raw
302344767			iGGIFAFk	Unambiguous	N-Term(iTRAQ4plex); K8(iTRAQ4plex)	HCD	2.725303	0	1	6	2.65102	2.7162	2.7342	2.8014061	2.268671753	182010.22	177645.5	482513.6	497657.22	1.716265272	2.184957188	2	High	5790	Unique	Used	68891	1	54.981	1	3.4795	-0.1293	1	23	14/14	17.5789	5790	1140.7	MS2	570.855	124	Glioma_grade2_4.raw
302344767			iTGNMGLAMk	Unambiguous	N-Term(iTRAQ4plex); K10(iTRAQ4plex)	HCD	2.21513	0	1	6	1.92435	2.3006	2.1437	2.5627587	12.03266032	163887.14	137086.75	315376.06	351320.25	12.59294453	7.624575121	2	High	3148	Unique	Used	58776	1	34.994	1	4.5979	-0.0963	1	21	17/18	33.68569	3148	1323.74	MS2	662.372	291	Glioma_grade2_3.raw
302344767			lQNLQLQPGNAk	Unambiguous	N-Term(iTRAQ4plex); K12(iTRAQ4plex)	HCD	1.7150772	0	1	6	1.63762	1.6949	1.7346	1.7952493	3.868299529	145290.38	140378.22	237929.78	252013.9	2.431783995	4.06535574	2	High	2517	Unique	Used	58187	1	30.299	1	5.0419	3.1539	1	24	17/22	34.92224	2517	1611.95	MS2	806.477	171	Glioma_grade2_3.raw
302344767			kLEEEGEQFVk	Unambiguous	N-Term(iTRAQ4plex); K1(iTRAQ4plex); K11(iTRAQ4plex)	HCD	2.1443672	1	1	6	1.77054	2.3775	1.9707	2.646356	18.01387179	134405.36	100091.12	237969.84	264876.72	20.69441025	7.567333188	2	High	2167	Unique	Used	79601	1	27.889	1	4.9147	5.2863	1	33	20/20	10.61429	2167	1767.99	MS2	884.501	216	Glioma_grade2_6.raw
302344767			kLEEEGEQFVk	Unambiguous	N-Term(iTRAQ4plex); K1(iTRAQ4plex); K11(iTRAQ4plex)	HCD	2.6399195	1	1	6	2.63771	2.5782	2.7031	2.6420822	1.932446307	101016.94	103349.125	266453.06	273056.88	1.613872468	1.731054632	2	High	2320	Unique	Used	90102	1	29.404	1	5.0095	1.8341	1	40	19/20	0	2320	1767.99	MS2	884.498	223	Glioma_grade2_7rep.raw
302344767			mNPQSAFFQGk	Unambiguous	N-Term(iTRAQ4plex); K11(iTRAQ4plex)	HCD	2.2111459	0	1	6	2.12573	2.2624	2.163	2.3020515	3.734069033	99760.89	93733.64	212064.95	215779.67	4.40519879	1.227877402	2	High	3706	Unique	Used	51265	1	38.952	1	4.2279	3.2568	1	35	18/20	18.12996	3706	1542.8	MS2	771.905	219	Glioma_grade2_2.raw
302344767			mNPQSAFFQGk	Unambiguous	N-Term(iTRAQ4plex); K11(iTRAQ4plex)	HCD	3.1892567	0	1	6	3.11582	3.1197	3.2587	3.2627842	2.589201377	55061.78	54992.895	171562.66	179429.95	0.088517913	3.169875348	2	High	3861	Unique	Used	67113	1	39.837	1	4.3846	1.1997	1	73	17/20	24.52098	3861	1542.8	MS2	771.903	211	Glioma_grade2_4.raw
302344767			kLEEEGEQFVk	Unambiguous	N-Term(iTRAQ4plex); K1(iTRAQ4plex); K11(iTRAQ4plex)	HCD	1.6153214	1	1	6	1.51274	1.6365	1.5958	1.7262931	5.487114408	46590.293	43068.082	70478.95	74348.13	5.555709175	3.778193161	3	High	2300	Unique	Used	90085	1	29.225	1	6.0058	1.4122	1	29	21/40	48.61224	2300	1767.99	MS2	590.001	227	Glioma_grade2_7rep.raw
302344767			mNPQSAFFQGk	Unambiguous	N-Term(iTRAQ4plex); M1(Oxidation); K11(iTRAQ4plex)	HCD	3.0437343	0	1	6	2.75643	3.1845	2.9219	3.3756661	8.978507805	44300.426	38344.918	122110.83	129439.64	10.19096754	4.120247717	2	High	2266	Unique	Used	57953	1	28.434	1	3.9238	13.22	1	62	17/20	23.61181	2266	1558.81	MS2	779.91	185	Glioma_grade2_3.raw
302344767			mNPQSAFFQGk	Unambiguous	N-Term(iTRAQ4plex); K11(iTRAQ4plex)	HCD	2.7608724	0	1	6	2.67867	2.9088	2.6247	2.8501325	4.898824798	41109.047	37857.02	110117.7	107897.52	5.824097443	1.440181278	2	High	3627	Unique	Used	59222	1	38.664	1	4.7081	1.8326	1	100	18/20	8.798506	3627	1542.8	MS2	771.904	196	Glioma_grade2_3.raw
302344767			mNPQSAFFQGk	Unambiguous	N-Term(iTRAQ4plex); M1(Oxidation); K11(iTRAQ4plex)	HCD	2.9720657	0	1	6	2.73079	3.0361	2.9144	3.2403173	7.178372726	39639.55	35653.016	108247.37	115527.08	7.487871312	4.600643466	2	High	2483	Unique	Used	65833	1	29.242	1	4.0101	5.6241	1	77	18/20	22.11784	2483	1558.8	MS2	779.904	185	Glioma_grade2_4.raw
307746865	NP_001182657.1	SLC1A2	excitatory amino acid transporter 2 isoform 2 [Homo sapiens]	12	44	0.406614244	26.81800798	349.13497	28.85	9.47E-05	565	61.188	6.6685	Membrane transport protein	Transport	Auxiliary transport protein activity	Plasma membrane	-																								
307746865			cLEENLGIDk	Unambiguous	N-Term(iTRAQ4plex); C1(Methylthio); K10(iTRAQ4plex)	HCD	0.36168623	0	1	2	0.31198	0.3378	0.3837	0.41550723	0.127703733	1.05E+07	9711186	3280711	4035067.8	5.624827522	14.58247504	2	High	4584	Unique	Used	52092	1	45.409	1	4.4851	1.4923	1	1	18/18	6.125533	4584	1467.74	MS2	734.376	160	Glioma_grade2_2.raw
307746865			vHEDIEMTk	Unambiguous	N-Term(iTRAQ4plex); K9(iTRAQ4plex)	HCD	0.32466716	0	1	2	0.29491	0.3191	0.3298	0.35686103	0.078988293	1977070.6	1827402.2	583058.8	652128.6	5.563532512	7.90806706	2	High	1185	Unique	Used	71964	1	20.807	1	3.7609	1.594	1	4	15/16	8.554607	1185	1389.73	MS2	695.369	155	Glioma_grade2_5.raw
307746865			nDEVSSLDAFLDLIR	Unambiguous	N-Term(iTRAQ4plex)	HCD	0.2421757	0	1	2	0.20838	0.2182	0.265	0.27756298	0.140773375	1696751.1	1620219.8	353576.72	449713.03	3.262965087	16.92506016	2	High	6041	Unique	Used	88192	1	76.581	1	5.7103	-1.9714	1	4	23/28	5.136071	6041	1850.96	MS2	925.983	195	Glioma_grade2_1rep.raw
307746865			cLEENLGIDk	Unambiguous	N-Term(iTRAQ4plex); C1(Methylthio); K10(iTRAQ4plex)	HCD	0.4649259	0	1	2	0.46012	0.4541	0.4759	0.46966368	0.020936333	1440284.6	1459484	662710.9	685466.6	0.936352365	2.387031349	2	High	4492	Unique	Used	52006	1	44.72	1	4.6999	0.4942	1	6	18/18	15.2466	4492	1467.74	MS2	734.375	165	Glioma_grade2_2.raw
307746865			sADcSVEEEPWk	Unambiguous	N-Term(iTRAQ4plex); C4(Methylthio); K12(iTRAQ4plex)	HCD	0.38832623	0	1	2	0.35691	0.3878	0.3888	0.42246148	0.068834306	722234.3	664701.56	257772.75	280810.8	5.866427103	6.049334919	2	High	4141	Unique	Used	51675	1	42.142	1	5.2948	2.1503	1	10	19/22	28.67814	4141	1713.77	MS2	857.391	35	Glioma_grade2_2.raw
307746865			tQSIYDDMk	Unambiguous	N-Term(iTRAQ4plex); K9(iTRAQ4plex)	HCD	0.34477776	0	1	2	0.33407	0.3856	0.3094	0.35713518	0.093880417	473509.12	410206.3	158186.34	146499.1	10.13037733	5.424694175	2	High	1953	Unique	Used	49614	1	26.455	1	3.874	-2.5119	1	14	15/16	26.61632	1953	1388.69	MS2	694.85	117	Glioma_grade2_2.raw
307746865			cLEENLGIDk	Unambiguous	N-Term(iTRAQ4plex); C1(Methylthio); K10(iTRAQ4plex)	HCD	0.6015794	0	1	2	0.54248	0.5929	0.6096	0.6661643	0.084582069	321751.28	294412.6	174544.36	196127.16	6.27474821	8.23443044	2	High	4692	Unique	Used	52193	1	46.202	1	4.455	1.9081	1	27	18/18	34.84599	4692	1467.75	MS2	734.376	234	Glioma_grade2_2.raw
307746865			sADcSVEEEPWk	Unambiguous	N-Term(iTRAQ4plex); C4(Methylthio); K12(iTRAQ4plex)	HCD	0.34277624	0	1	2	0.34075	0.3338	0.3519	0.3447615	0.02209413	283223.97	289109.62	96508.5	99673.87	1.454320732	2.281810126	2	High	4045	Unique	Used	51585	1	41.424	1	5.3567	2.9338	1	53	18/22	39.4773	4045	1713.78	MS2	857.391	30	Glioma_grade2_2.raw
307746865			cLEENLGIDkR	Unambiguous	N-Term(iTRAQ4plex); C1(Methylthio); K10(iTRAQ4plex)	HCD	0.35481414	1	1	2	0.31739	0.349	0.3601	0.3959716	0.091162866	210364.83	191289.66	66767.414	75745.27	6.716310857	8.909105744	3	High	2747	Unique	Used	73408	1	32.469	1	3.6004	14.994	1	11	26/40	14.06746	2747	1623.87	MS2	541.961	162	Glioma_grade2_5.raw
307746865			kNDEVSSLDAFLDLIR	Unambiguous	N-Term(iTRAQ4plex); K1(iTRAQ4plex)	HCD	0.32405162	1	1	2	0.2998	0.3167	0.3311	0.3496627	0.065353871	191284.7	181107.1	57347.77	63326.39	3.865095835	7.006508674	2	High	7055	Unique	Used	83733	1	71.811	1	5.098	-0.5741	1	20	25/30	8.456946	7055	2123.16	MS2	1062.08	130	Glioma_grade2_6.raw
307746865			tSVNVVGDSFGAGIVYHLSk	Unambiguous	N-Term(iTRAQ4plex); K20(iTRAQ4plex)	HCD	0.3314207	0	1	2	0.33819	0.3235	0.3397	0.324943	0.025856097	164117.89	171595.64	55503.6	55758.805	3.150047443	0.324381243	3	High	5603	Unique	Used	75962	1	55.742	1	9.5084	-0.1942	1	4	44/76	5.443023	5603	2338.26	MS2	780.093	99	Glioma_grade2_5.raw
307746865			tSVNVVGDSFGAGIVYHLSk	Unambiguous	N-Term(iTRAQ4plex); K20(iTRAQ4plex)	HCD	0.34365648	0	1	2	0.29794	0.2854	0.4045	0.38744906	0.176953707	158748.73	165731.34	47297.23	64212.453	3.043299936	21.45261033	2	High	5644	Unique	Used	76000	1	56.057	1	6.9204	-2.0417	1	11	27/38	3.672168	5644	2338.26	MS2	1169.63	130	Glioma_grade2_5.raw
307746865			cLEENLGIDkR	Unambiguous	N-Term(iTRAQ4plex); C1(Methylthio); K10(iTRAQ4plex)	HCD	0.38261414	1	1	2	0.35684	0.4017	0.3657	0.41162622	0.069733531	151755.3	134807.55	54152.67	55490.32	8.363867787	1.725347669	3	High	2794	Unique	Used	73451	1	32.83	1	3.5061	-2.5967	1	13	21/40	22.55202	2794	1623.84	MS2	541.951	149	Glioma_grade2_5.raw
307746865			nLFPENLVQAcFQQIQTVTk	Unambiguous	N-Term(iTRAQ4plex); C11(Methylthio); K20(iTRAQ4plex)	HCD	0.26819563	0	1	2	0.25248	0.2795	0.258	0.28559706	0.059938326	135045.81	121997.37	34095.77	34842.09	7.179058715	1.531024992	2	High	8514	Unique	Used	63715	1	77.653	1	6.0749	-2.4	1	17	22/38	5.535697	8514	2655.38	MS2	1328.19	131	Glioma_grade2_3.raw
307746865			tSVNVVGDSFGAGIVYHLSk	Unambiguous	N-Term(iTRAQ4plex); K20(iTRAQ4plex)	HCD	0.34743187	0	1	2	0.27188	0.2777	0.4157	0.42460454	0.241818978	132303.92	129522.41	35971.008	54995.8	1.502388689	29.57685277	3	High	5724	Unique	Used	76070	1	56.744	1	9.0669	-0.0376	1	4	43/76	6.472593	5724	2338.26	MS2	780.093	99	Glioma_grade2_5.raw
307746865			cLEENLGIDk	Unambiguous	N-Term(iTRAQ4plex); C1(Methylthio); K10(iTRAQ4plex)	HCD	0.497375	0	1	2	0.46312	0.5058	0.4896	0.53478914	0.060208334	107476.16	98402	49774.324	52624.32	6.233201297	3.936090204	3	High	4613	Unique	Used	52119	1	45.626	1	3.3636	-2.9664	1	7	18/36	14.12018	4613	1467.74	MS2	489.918	172	Glioma_grade2_2.raw
307746865			cLEENLGIDkR	Unambiguous	N-Term(iTRAQ4plex); C1(Methylthio); K10(iTRAQ4plex)	HCD	0.44705835	1	1	2	0.4036	0.4597	0.436	0.4965538	0.087286125	101882.65	89449.1	41120.164	44416.293	9.190160566	5.44964159	2	High	2735	Unique	Used	73397	1	32.374	1	3.962	13.874	1	81	19/20	18.10697	2735	1623.87	MS2	812.437	267	Glioma_grade2_5.raw
307746865			tSVNVVGDSFGAGIVYHLSk	Unambiguous	N-Term(iTRAQ4plex); K20(iTRAQ4plex)	HCD	0.37025312	0	1	2	0.3797	0.4244	0.3218	0.35969242	0.115044459	77975.164	69760.195	29607.266	25092.213	7.863872707	11.67332725	3	High	6132	Unique	Used	69208	1	57.671	1	9.8291	2.8598	1	9	44/76	18.35998	6132	2338.27	MS2	780.095	108	Glioma_grade2_4.raw
307746865			tSVNVVGDSFGAGIVYHLSk	Unambiguous	N-Term(iTRAQ4plex); K20(iTRAQ4plex)	HCD	0.34463385	0	1	2	0.35167	0.3666	0.3236	0.33729607	0.053712408	53884.992	51692.61	18949.902	17435.713	2.936698977	5.885256082	2	High	6162	Unique	Used	69235	1	57.918	1	8.1613	1.6127	1	53	30/38	4.519883	6162	2338.27	MS2	1169.64	125	Glioma_grade2_4.raw

307746865			cLEENLGIDkR	Unambiguous	N-Term(iTRAQ4plex); C1(Methylthio); K10(iTRAQ4plex)	HCD	0.4596097	1	1	2	0.43313	0.4131	0.5084	0.48486096	0.096316882	52027.508	54554.273	22534.781	26451.238	3.352716851	11.30670897	3	High	2657	Unique	Used	80053	1	31.627	1	3.9583	-5.9795	1	66	22/40	11.62493	2657	1623.83	MS2	541.95	139	Glioma_grade2_6.raw
307746865			cLEENLGIDkR	Unambiguous	N-Term(iTRAQ4plex); C1(Methylthio); K10(iTRAQ4plex)	HCD	0.44502145	1	1	2	0.44207	0.4207	0.4706	0.4478283	0.046010351	41865.434	43990.59	18507.541	19700.23	3.500539971	4.414591365	3	High	2760	Unique	Used	80131	1	32.704	1	4.0223	-2.2584	1	56	23/40	22.55807	2760	1623.84	MS2	541.952	136	Glioma_grade2_6.raw
307746865			tSVNVVGDSFGAGIVYHLSk	Unambiguous	N-Term(iTRAQ4plex); K20(iTRAQ4plex)	HCD	0.50980204	0	1	2	0.47955	0.4803	0.5392	0.5401023	0.067654835	38523.316	38461.543	18473.87	20773.168	0.113477138	8.285207193	3	High	5067	Unique	Used	92264	1	56.342	1	9.6694	0.2757	1	34	40/76	29.6949	5067	2338.27	MS2	780.093	132	Glioma_grade2_7rep.raw
307746865			nLFPENLVQAcFQQIQTVTk	Unambiguous	N-Term(iTRAQ4plex); C11(Methylthio); K20(iTRAQ4plex)	HCD	0.23010875	0	1	2	0.27373	0.2814	0.1802	0.18526012	0.238513496	38463.73	37410.992	10528.673	6930.7646	1.962177018	29.14303984	3	High	8502	Unique	Used	63704	1	77.555	1	7.4031	2.6976	1	7	28/76	10.8151	8502	2655.4	MS2	885.803	166	Glioma_grade2_3.raw
307746865			tSVNVVGDSFGAGIVYHLSk	Unambiguous	N-Term(iTRAQ4plex); K20(iTRAQ4plex)	HCD	0.49963164	0	1	2	0.50726	0.4863	0.5135	0.4923219	0.025298828	37696.723	39317.223	19121.873	19356.73	2.975737768	0.86317571	3	High	5839	Unique	Used	76174	1	57.663	1	9.657	-0.2725	1	15	43/76	17.56089	5839	2338.26	MS2	780.093	102	Glioma_grade2_5.raw
307746865			kNDEVSSLDAFLDLIR	Unambiguous	N-Term(iTRAQ4plex); K1(iTRAQ4plex)	HCD	0.46650118	1	1	2	0.46464	0.5269	0.4133	0.46860927	0.099169173	36613.926	32289.953	17012.367	15131.372	8.874712641	8.275728719	3	High	7043	Unique	Used	83723	1	71.693	1	7.7537	-0.5805	1	19	30/60	11.36476	7043	2123.16	MS2	708.391	164	Glioma_grade2_6.raw
307746865			cLEENLGIDkR	Unambiguous	N-Term(iTRAQ4plex); C1(Methylthio); K10(iTRAQ4plex)	HCD	0.483943	1	1	2	0.48525	0.4559	0.5138	0.4827125	0.048889413	34909.24	37158.777	16939.805	17937.006	4.414337825	4.043532474	3	High	2630	Unique	Used	80033	1	31.333	1	3.5319	4.3944	1	71	21/40	38.75687	2630	1623.85	MS2	541.955	167	Glioma_grade2_6.raw
307746865			tSVNVVGDSFGAGIVYHLSk	Unambiguous	N-Term(iTRAQ4plex); K20(iTRAQ4plex)	HCD	0.56104356	0	1	2	0.61549	0.5573	0.5652	0.51174545	0.075559424	30569.523	33761.04	18815.19	17277.057	7.016084448	6.026913618	3	High	5924	Unique	Used	76250	1	58.354	1	9.293	-0.1942	1	21	41/76	29.35684	5924	2338.26	MS2	780.093	107	Glioma_grade2_5.raw
307746865			vHEDIEMTk	Unambiguous	N-Term(iTRAQ4plex); K9(iTRAQ4plex)	HCD	0.5228977	0	1	2	0.44725	0.5779	0.4803	0.62065333	0.152938501	28215.93	21835.17	12619.535	13552.072	18.02908893	5.039073347	3	High	1237	Unique	Used	72014	1	21.159	1	3.8173	-5.5525	1	43	17/32	22.9141	1237	1389.72	MS2	463.912	173	Glioma_grade2_5.raw
307746865			nLFPENLVQAcFQQIQTVTk	Unambiguous	N-Term(iTRAQ4plex); C11(Methylthio); K20(iTRAQ4plex)	HCD	0.31940013	0	1	2	0.28907	0.3099	0.3283	0.3519191	0.083646932	24885.553	23213.951	7193.5557	8169.433	4.91481621	8.983271028	2	High	6162	Unique	Used	88289	1	77.75	1	5.1396	1.0018	1	100	20/38	8.66723	6162	2655.39	MS2	1328.2	130	Glioma_grade2_1rep.raw
307746865			lASPIHPDVVMLIAFPGDILMR	Unambiguous	N-Term(iTRAQ4plex)	HCD	0.5927056	0	1	2	0.50197	0.5696	0.6131	0.6956568	0.136383683	24387.219	21492.94	12241.719	14951.71	8.92134793	14.0935004	3	High	7492	Unique	Used	84067	1	76.35	1	6.8283	2.3671	1	22	24/84	16.90699	7492	2549.42	MS2	850.477	102	Glioma_grade2_6.raw
307746865			nLFPENLVQAcFQQIQTVTk	Unambiguous	N-Term(iTRAQ4plex); C11(Methylthio); K20(iTRAQ4plex)	HCD	0.3787952	0	1	2	0.27113	0.388	0.3724	0.53284967	0.275574507	18093.922	12644.983	4905.8745	6737.8755	25.06908894	22.25091281	3	High	8809	Unique	Used	56011	1	77.631	1	6.7377	2.3529	1	11	25/76	18.09117	8809	2655.39	MS2	885.803	171	Glioma_grade2_2.raw
307746865			tSVNVVGDSFGAGIVYHLSk	Unambiguous	N-Term(iTRAQ4plex); K20(iTRAQ4plex)	HCD	0.5816737	0	1	2	0.54063	0.5616	0.601	0.6243145	0.064888272	16528.451	15910.364	8935.733	9933.07	2.694633013	7.475023782	3	High	6280	Unique	Used	69344	1	58.85	1	9.1317	1.0588	1	51	37/76	31.85844	6280	2338.27	MS2	780.094	114	Glioma_grade2_4.raw
307746865			kNDEVSSLDAFLDLIR	Unambiguous	N-Term(iTRAQ4plex); K1(iTRAQ4plex)	HCD	0.42490226	1	1	2	0.41497	0.5253	0.3456	0.43748102	0.17212499	15276.404	12066.812	6339.193	5279.001	16.60027312	12.90508581	3	High	7145	Unique	Used	83801	1	72.771	1	7.6564	-0.753	1	26	29/60	12.01422	7145	2123.16	MS2	708.391	167	Glioma_grade2_6.raw
307746865			nLFPENLVQAcFQQIQTVTk	Unambiguous	N-Term(iTRAQ4plex); C11(Methylthio); K20(iTRAQ4plex)	HCD	0.36370528	0	1	2	0.30059	0.3291	0.3954	0.4327913	0.165819343	15200.884	13886.181	4569.3003	6009.8184	6.392088075	19.25680476	2	High	8801	Unique	Used	56005	1	77.543	1	5.3943	-1.1129	1	100	22/38	5.419766	8801	2655.38	MS2	1328.2	159	Glioma_grade2_2.raw
307746865			tSVNVVGDSFGAGIVYHLSk	Unambiguous	N-Term(iTRAQ4plex); K20(iTRAQ4plex)	HCD	0.59716403	0	1	2	0.678	0.5804	0.6168	0.527972	0.104998883	14596.774	17052.756	9896.583	9003.377	10.97420105	6.68352758	3	High	6352	Unique	Used	76627	1	61.947	1	9.242	-5.2842	1	61	40/76	22.61745	6352	2338.25	MS2	780.089	120	Glioma_grade2_5.raw
307746865			tSVNVVGDSFGAGIVYHLSk	Unambiguous	N-Term(iTRAQ4plex); K20(iTRAQ4plex)	HCD	0.65684336	0	1	2	0.70954	0.6683	0.6447	0.6072117	0.06523308	14246.392	15125.524	10108.354	9184.3955	4.232888306	6.772879323	3	High	6220	Unique	Used	76510	1	60.847	1	8.9116	-0.0376	1	41	45/76	32.12298	6220	2338.26	MS2	780.093	104	Glioma_grade2_5.raw
307746865			mHDSHLGSEEPk	Unambiguous	N-Term(iTRAQ4plex); K12(iTRAQ4plex)	HCD	0.5499134	0	1	2	0.51128	0.5239	0.5753	0.58949965	0.069511187	12580.389	12278.171	6432.0776	7237.9775	1.719330462	8.337307788	4	High	919	Unique	Used	94006	1	17.96	1	3.5878	7.2641	1	100	15/66	61.97256	919	1654.82	MS2	414.461	147	Glioma_grade2_8rep.raw
307746865			kNDEVSSLDAFLDLIR	Unambiguous	N-Term(iTRAQ4plex); K1(iTRAQ4plex)	HCD	0.649093	1	1	2	0.76056	0.6418	0.6578	0.5550376	0.128920948	12076.913	14312.637	9185.22	7944.052	11.9812244	10.2472342	3	High	7244	Unique	Used	83872	1	73.893	1	6.166	-15.156	1	29	21/60	35.08373	7244	2123.13	MS2	708.38	192	Glioma_grade2_6.raw
307746865			nLFPENLVQAcFQQIQTVTk	Unambiguous	N-Term(iTRAQ4plex); C11(Methylthio); K20(iTRAQ4plex)	HCD	0.35315844	0	1	2	0.28746	0.3588	0.3486	0.4351728	0.169521676	5878.7617	4709.3438	1689.9004	2049.3784	15.61947654	13.59563408	3	High	8560	Unique	Used	63743	1	78.242	1	5.8477	2.146	1	33	28/76	7.036247	8560	2655.39	MS2	885.803	169	Glioma_grade2_3.raw
307746865			tSVNVVGDSFGAGIVYHLSk	Unambiguous	N-Term(iTRAQ4plex); K20(iTRAQ4plex)	HCD	0.5247175	0	1	2	0.53821	0.5001	0.5512	0.5121836	0.044572225	5161.5884	5554.935	2778.0007	2845.1465	5.190826126	1.688707367	3	High	7497	Unique	Used	77594	1	72.388	1	8.9866	1.2154	1	100	41/76	26.54907	7497	2338.27	MS2	780.094	98	Glioma_grade2_5.raw
307746865			tSVNVVGDSFGAGIVYHLSk	Unambiguous	N-Term(iTRAQ4plex); K20(iTRAQ4plex)	HCD	0.57498264	0	1	2	0.60164	0.5381	0.6163	0.5511391	0.065882963	5055.924	5653.2495	3041.862	3115.727	7.888058058	1.696457571	3	High	7301	Unique	Used	77436	1	70.483	1	8.5281	-1.2905	1	100	43/76	45.79521	7301	2338.26	MS2	780.092	109	Glioma_grade2_5.raw
307746865			tSVNVVGDSFGAGIVYHLSk	Unambiguous	N-Term(iTRAQ4plex); K20(iTRAQ4plex)	HCD	0.66322005	0	1	2	0.61525	0.5865	0.7437	0.7089416	0.11265503	4964.8276	5208.555	3054.63	3692.5615	3.388082491	13.37106527	3	High	7886	Unique	Used	77909	1	76.142	1	8.4878	-0.4291	1	83	41/76	7.251715	7886	2338.26	MS2	780.093	107	Glioma_grade2_5.raw
307746865			vHEDIEmTk	Unambiguous	N-Term(iTRAQ4plex); M7(Oxidation); K9(iTRAQ4plex)	HCD	0.3637848	0	1	2	0.30309	0.2849	0.4477	0.42083484	0.225354367	3913.269	4163.069	1186.0845	1751.9645	4.374142685	27.2383194	3	High	998	Unique	Used	71805	1	19.066	1	3.6012	5.4442	1	100	17/32	30.25529	998	1405.73	MS2	469.249	168	Glioma_grade2_5.raw
307746865			mLILPLIISSLITGLSGLDAk	Unambiguous	N-Term(iTRAQ4plex); M1(Oxidation); K21(iTRAQ4plex)	HCD	0.3010499	0	1	2	0.4018	0.2763	0.337	0.23177119	0.237197169	1600.6539	2327.7024	643.13696	539.4943	26.17384399	12.39379174	3	High	7720	Unique	Used	84224	1	78.965	1	5.2563	3.3875	1	100	25/80	14.65428	7720	2472.51	MS2	824.84	13	Glioma_grade2_6.raw
308818200	NP_001184223.1	DPYSL3	dihydropyrimidinase-related protein 3 isoform 1 [Homo sapiens]	15	39	2.205678701	23.95442977	305.20475	45.03	6.50E-05	684	73.865	6.354	Enzyme: Hydrolase	Metabolism,Energy pathways	Hydrolase activity	Cytoplasm	Nucleus																								
308818200			mADLHAVPR	Unambiguous	N-Term(iTRAQ4plex)	HCD	2.0575554	0	1	2	1.89573	2.1809	1.9504	2.24372	8.24212698	744054.5	646774.56	1410526.9	1451181	9.891554214	2.009065271	2	High	1346	Unique	Used	64780	1	20.761	1	3.0195	0.7358	1	3	15/16	12.31363	1346	1153.63	MS2	577.318	214	Glioma_grade2_4.raw
308818200			mADLHAVPR	Unambiguous	N-Term(iTRAQ4plex)	HCD	2.4933562	0	1	2	2.3613	2.6281	2.3723	2.6403353	6.180858043	250910.25	225436.45	592474.1	595227.8	7.562851479	0.32788698	3	High	1329	Unique	Used	64764	1	20.647	1	3.5117	1.1739	1	4	15/32	3.450359	1329	1153.63	MS2	385.214	283	Glioma_grade2_4.raw
308818200			gAPLVVIcQGk	Unambiguous	N-Term(iTRAQ4plex); C8(Methylthio); K11(iTRAQ4plex)	HCD	1.7665006	0	1	2	1.65837	1.8367	1.7031	1.8862538	6.088858649	180264.81	162764.22	298946.38	307014.62	7.215007933	1.882994852	2	High	5520	Unique	Used	68648	1	52.772	1	4.273	0.4231	1	30	18/20	42.88701	5520	1418.81	MS2	709.909	224	Glioma_grade2_4.raw
308818200			gMTTVDDFFQGTk	Unambiguous	N-Term(iTRAQ4plex); K13(iTRAQ4plex)	HCD	1.6496564	0	1	2	1.57737	1.6054	1.6931	1.7232293	4.211020681	169055.22	166098.72	266662.7	286226.2	1.247522973	5.004073518	2	High	5743	Unique	Used	53183	1	53.827	1	5.7558	2.3775	1	23	20/24	45.26645	5743	1734.87	MS2	867.936	162	Glioma_grade2_2.raw
308818200			iMLEDGNLHVTQGAGR	Unambiguous	N-Term(iTRAQ4plex)	HCD	1.617712	0	1	2	1.47981	1.5763	1.6566	1.7646114	7.462890593	149502.22	140350.05	221234.19	247663.3	4.465420588	7.971125161	3	High	3219	Unique	Used	58843	1	35.525	1	4.7669	-2.3013	1	4	26/60	10.14783	3219	1854.96	MS2	618.991	169	Glioma_grade2_3.raw
308818200			gmTTVDDFFQGTk	Unambiguous	N-Term(iTRAQ4plex); M2(Oxidation); K13(iTRAQ4plex)	HCD	1.5922462	0	1	2	1.5701	1.5963	1.5883	1.6147575	1.163198061	103777.055	102074.44	162940.78	164825.47	1.169707912	0.813187495	2	High	4354	Unique	Used	51877	1	43.674	1	4.3453	0.9374	1	23	18/24	30.04843	4354	1750.86	MS2	875.933	109	Glioma_grade2_2.raw
308818200			gAPLVVIcQGk	Unambiguous	N-Term(iTRAQ4plex); C8(Methylthio); K11(iTRAQ4plex)	HCD	2.558449	0	1	2	2.42433	2.5154	2.5999	2.6976047	4.563429915	86600.305	83464.664	209947.88	225154.69	2.607512914	4.942668333	2	High	5305	Unique	Used	60782	1	51.692	1	4.0769	-0.1791	1	42	17/20	46.98219	5305	1418.81	MS2	709.909	245	Glioma_grade2_3.raw
308818200			gmYDGPVFDLTTTPk	Unambiguous	N-Term(iTRAQ4plex); M2(Oxidation); K15(iTRAQ4plex)	HCD	2.872802	0	1	2	2.72262	2.7385	3.0063	3.0238674	5.727334589	80336.1	79868.76	218724.31	241512.52	0.41254589	7.00235043	2	High	5213	Unique	Used	52683	1	49.985	1	4.4301	-1.5164	1	12	20/28	2.576741	5213	1945.98	MS2	973.493	153	Glioma_grade2_2.raw
308818200			qIGDNLIVPGGVk	Unambiguous	N-Term(iTRAQ4plex); K13(iTRAQ4plex)	HCD	1.8542138	0	1	2	1.61766	2.0611	1.6918	2.1556056	14.16059639	71105.84	55807.844	115025.41	120299.7	17.0467303	3.169645793	2	High	4042	Unique	Used	59608	1	41.857	1	4.0807	0.6615	1	32	18/24	5.687949	4042	1597.95	MS2	799.48	119	Glioma_grade2_3.raw
308818200			aITIASQTNcPLYVTk	Unambiguous	N-Term(iTRAQ4plex); C10(Methylthio); K16(iTRAQ4plex)	HCD	2.3656948	0	1	2	2.2759	2.4374	2.2988	2.461863	3.997831408	63592.06	59379.195	144729.02	146183.44	4.844945934	0.707037605	2	High	6031	Unique	Used	53455	1	55.932	1	6.8506	-3.2153	1	25	21/30	7.663436	6031	2057.09	MS2	1029.05	213	Glioma_grade2_2.raw
308818200			gMYDGPVFDLTTTPk	Unambiguous	N-Term(iTRAQ4plex); K15(iTRAQ4plex)	HCD	3.0883791	0	1	2	3.00555	2.8875	3.2975	3.1679535	5.827615606	53230.125	55406.305	159985.89	175524.6	2.832920108	6.549736917	2	High	5953	Unique	Used	53381	1	55.371	1	5.6366	-2.3294	1	26	20/28	4.97484	5953	1929.98	MS2	965.495	164	Glioma_grade2_2.raw
308818200			mVIPGGIDVHTHFQmPYk	Unambiguous	N-Term(iTRAQ4plex); M15(Oxidation); K18(iTRAQ4plex)	HCD	2.250603	0	1	2	2.17649	2.1448	2.358	2.3236413	4.697970573	50158.46	50899.062	109169.2	118271.16	1.036409138	5.659556323	3	High	4177	Unique	Used	91593	1	47.186	1	6.6829	0.9582	1	17	27/68	16.9675	4177	2374.23	MS2	792.082	185	Glioma_grade2_7rep.raw
308818200			mVIPGGIDVHTHFQMPYk	Unambiguous	N-Term(iTRAQ4plex); K18(iTRAQ4plex)	HCD	2.0598702	0	1	2	1.87658	2.3327	1.8404	2.2877085	12.56409094	45095.07	36277.1	84624.69	82991.44	15.32525526	1.37800837	3	High	4760	Unique	Used	92035	1	53.146	1	5.9609	1.2414	1	27	26/68	32.53377	4760	2358.24	MS2	786.751	191	Glioma_grade2_7rep.raw
308818200			qIGDNLIVPGGVk	Unambiguous	N-Term(iTRAQ4plex); K13(iTRAQ4plex)	HCD	2.785663	0	1	2	2.73822	2.8101	2.7618	2.8343554	1.576197854	42857.44	41761.11	117352.93	118365.83	1.832275257	0.60769746	2	High	3947	Unique	Used	59520	1	41.119	1	4.734	-0.1024	1	100	19/24	0	3947	1597.95	MS2	799.479	117	Glioma_grade2_3.raw
308818200			fIPcSPFSDYVYk	Unambiguous	N-Term(iTRAQ4plex); C4(Methylthio); K13(iTRAQ4plex)	HCD	3.005187	0	1	2	2.88809	3.0433	2.969	3.1285806	3.417177076	39363.28	37355.84	113684.55	116870.75	3.700445044	1.954397601	2	High	7892	Unique	Used	55177	1	70.232	1	4.3164	0.7441	1	72	16/24	12.58949	7892	1899.93	MS2	950.468	152	Glioma_grade2_2.raw
308818200			iSVGSDSDLVIWDPDAVk	Unambiguous	N-Term(iTRAQ4plex); K18(iTRAQ4plex)	HCD	2.3071904	0	1	2	2.31865	2.2845	2.3302	2.2959015	0.903551827	36200	36741.11	83935.055	84353.98	1.049127304	0.352042792	2	High	6934	Unique	Used	54301	1	62.612	1	6.0291	-0.436	1	50	23/34	9.708157	6934	2204.17	MS2	1102.59	181	Glioma_grade2_2.raw
308818200			iMLEDGNLHVTQGAGR	Unambiguous	N-Term(iTRAQ4plex)	HCD	2.2267838	0	1	2	2.08053	2.2201	2.233	2.3828537	5.543551704	34141.855	31995.342	71033.016	76240.22	4.589894846	5.000296536	2	High	3211	Unique	Used	58836	1	35.448	1	5.9337	-1.5702	1	100	24/30	18.59254	3211	1854.96	MS2	927.983	212	Glioma_grade2_3.raw
308818200			fIPcSPFSDYVYk	Unambiguous	N-Term(iTRAQ4plex); C4(Methylthio); K13(iTRAQ4plex)	HCD	3.2302198	0	1	2	3.26364	3.1467	3.3169	3.197999	2.30445884	33091.52	34321.43	107998.76	109759.9	2.580150257	1.143756153	2	High	7550	Unique	Used	62847	1	69.621	1	4.1087	-0.1554	1	50	16/24	15.97339	7550	1899.93	MS2	950.467	117	Glioma_grade2_3.raw
308818200			fIPcSPFSDYVYk	Unambiguous	N-Term(iTRAQ4plex); C4(Methylthio); K13(iTRAQ4plex)	HCD	3.6320586	0	1	2	3.68434	3.6685	3.5954	3.580004	1.432026634	26780.246	26895.576	98667.46	96286.27	0.303863535	1.727338683	2	High	7570	Unique	Used	70497	1	69.766	1	3.9568	-0.0912	1	57	16/24	0	7570	1899.93	MS2	950.467	118	Glioma_grade2_4.raw
308818200			mLEMGITGPEGHVLSRPEELEAEAVFR	Unambiguous	N-Term(iTRAQ4plex)	HCD	2.1812577	0	1	2	2.03473	2.104	2.256	2.332766	6.261052282	22308.523	21574.506	45391.92	50328.273	2.365508535	7.293191899	3	High	5863	Unique	Used	82808	1	59.665	1	4.561	0.7799	1	14	26/104	4.24282	5863	3141.59	MS2	1047.87	107	Glioma_grade2_6.raw
308818200			imLEDGNLHVTQGAGR	Unambiguous	N-Term(iTRAQ4plex); M2(Oxidation)	HCD	1.5558269	0	1	2	1.51244	1.7015	1.4263	1.6046371	7.592421326	21705.592	19293.447	32828.426	30958.98	8.320410079	4.144698857	3	High	2647	Unique	Used	58310	1	31.193	1	3.6169	1.0886	1	12	27/60	15.52309	2647	1870.96	MS2	624.324	121	Glioma_grade2_3.raw
308818200			mVIPGGIDVHTHFQmPYk	Unambiguous	N-Term(iTRAQ4plex); M15(Oxidation); K18(iTRAQ4plex)	HCD	1.9257421	0	1	2	1.82624	2.0528	1.8127	2.0375865	6.757753992	21678.014	19285.531	39589.293	39295.938	8.25973901	0.52591165	4	High	4164	Unique	Used	91583	1	47.057	1	7.1632	-0.3842	1	10	32/102	20.1954	4164	2374.23	MS2	594.313	193	Glioma_grade2_7rep.raw
308818200			gNVVFGEPITASLGIDGTHYWSk	Unambiguous	N-Term(iTRAQ4plex); K23(iTRAQ4plex)	HCD	2.1396842	0	1	2	2.49857	1.8934	2.4647	1.8677174	15.93005412	21410.26	28253.172	53495.113	52768.94	19.48584414	0.966426253	3	High	6456	Unique	Used	76718	1	62.846	1	7.6939	-3.126	1	8	36/88	6.519561	6456	2736.42	MS2	912.81	101	Glioma_grade2_5.raw
308818200			iMLEDGNLHVTQGAGR	Unambiguous	N-Term(iTRAQ4plex)	HCD	1.5755183	0	1	2	1.63164	1.5959	1.5546	1.5206207	3.0692564	20316.193	20770.676	33148.797	31584.318	1.564334392	3.417891167	3	High	3474	Unique	Used	66755	1	36.775	1	5.6702	0.7587	1	50	24/60	11.28168	3474	1854.96	MS2	618.992	172	Glioma_grade2_4.raw
308818200			mVIPGGIDVHTHFQMPYk	Unambiguous	N-Term(iTRAQ4plex); M1(Oxidation); K18(iTRAQ4plex)	HCD	1.937542	0	1	2	1.76441	2.342	1.6328	2.167347	16.8585659	19105.744	14393.682	33710.445	31196.1	19.89246618	5.47837017	3	High	4369	Unique	Used	91746	1	49.026	1	5.2087	1.421	1	37	25/68	21.56366	4369	2374.23	MS2	792.082	199	Glioma_grade2_7rep.raw
308818200			iMLEDGNLHVTQGAGR	Unambiguous	N-Term(iTRAQ4plex)	HCD	2.2056787	0	1	2	2.15187	2.2461	2.1669	2.2618494	2.507026472	18163.1	17401.008	39084.54	39358.457	3.030473426	0.493832657	2	High	3491	Unique	Used	66771	1	36.909	1	5.5827	0.6672	1	100	23/30	16.41292	3491	1854.96	MS2	927.985	204	Glioma_grade2_4.raw
308818200			mVIPGGIDVHTHFQMPYk	Unambiguous	N-Term(iTRAQ4plex); K18(iTRAQ4plex)	HCD	2.4472816	0	1	2	2.44504	2.3547	2.5434	2.4494443	3.147986582	16846.568	17492.842	41190.46	42847.74	2.661575886	2.788907726	2	High	4776	Unique	Used	92046	1	53.325	1	4.4855	-0.0988	1	100	16/34	0	4776	2358.23	MS2	1179.62	161	Glioma_grade2_7rep.raw
308818200			gAPLVVIcQGk	Unambiguous	N-Term(iTRAQ4plex); C8(Methylthio); K11(iTRAQ4plex)	HCD	2.2136858	0	1	2	2.34193	1.9703	2.503	2.1057873	10.67052778	9825.153	11678.279	23009.87	24591.973	12.18743093	4.700304397	3	High	5470	Unique	Used	68601	1	52.407	1	4.43	-0.6188	1	74	19/40	68.81023	5470	1418.81	MS2	473.608	165	Glioma_grade2_4.raw
308818200			fIPcSPFSDYVYk	Unambiguous	N-Term(iTRAQ4plex); C4(Methylthio); K13(iTRAQ4plex)	HCD	3.1916587	0	1	2	3.07922	3.3527	3.0437	3.3140826	4.955191097	9022.901	8286.895	27783.506	27463.451	6.01318275	0.819277923	2	High	7648	Unique	Used	70563	1	70.492	1	3.5828	-2.6612	1	100	18/24	9.77133	7648	1899.92	MS2	950.464	129	Glioma_grade2_4.raw
308818200			nHQSAAEYNIFEGMELR	Unambiguous	N-Term(iTRAQ4plex)	HCD	1.5215325	0	1	2	1.38805	2.0744	1.1516	1.7210088	25.37640105	8770.596	5868.8413	12174.053	10100.328	28.03182095	13.1662021	3	High	5709	Unique	Used	68817	1	54.329	1	5.3983	0.03	1	24	28/64	18.43095	5709	2153.02	MS2	718.345	130	Glioma_grade2_4.raw

308818200			aALAGGTTMIIDHVVPEPESSLTEAYEk	Unambiguous	N-Term(iTRAQ4plex); K28(iTRAQ4plex)	HCD	1.8398229	0	1	2	1.75803	2.0229	1.6807	1.9339384	8.499108105	8709.686	7569.109	15311.907	14638.19	9.908715371	3.18122415	3	High	6725	Unique	Used	69747	1	62.513	1	6.7822	-0.5844	1	20	35/108	10.99204	6725	3217.65	MS2	1073.22	99	Glioma_grade2_4.raw
308818200			nLHQSGFSLSGTQVDEGVR	Unambiguous	N-Term(iTRAQ4plex)	HCD	1.7385738	0	1	2	1.63314	1.752	1.726	1.8516803	5.162759919	8675.728	8086.946	14168.707	14974.438	4.96736672	3.909927044	3	High	2904	Unique	Used	58548	1	33.177	1	8.9067	0.5917	1	50	28/72	22.21343	2904	2175.09	MS2	725.702	184	Glioma_grade2_3.raw
308818200			gAPLVVIcQGk	Unambiguous	N-Term(iTRAQ4plex); C8(Methylthio); K11(iTRAQ4plex)	HCD	2.3682773	0	1	2	2.45259	2.2827	2.4603	2.289809	4.147824975	8668.892	9314.254	21261.21	21327.861	5.075194813	0.221321447	3	High	5318	Unique	Used	60794	1	51.792	1	4.1368	-0.6188	1	57	18/40	20.12934	5318	1418.81	MS2	473.608	194	Glioma_grade2_3.raw
308818200			mVIPGGIDVHTHFQMPYk	Unambiguous	N-Term(iTRAQ4plex); K18(iTRAQ4plex)	HCD	1.8593557	0	1	2	1.64011	2.1414	1.6433	2.145607	15.30727932	8282.902	6343.949	13584.898	13611.621	18.74698545	0.138959067	4	High	4748	Unique	Used	92025	1	53.037	1	6.5773	-2.9572	1	29	31/102	23.346	4748	2358.23	MS2	590.312	179	Glioma_grade2_7rep.raw
308818200			mVIPGGIDVHTHFQMPYk	Unambiguous	N-Term(iTRAQ4plex); M1(Oxidation); K18(iTRAQ4plex)	HCD	1.791172	0	1	2	1.90764	1.7014	1.8919	1.687298	6.617377802	7984.474	8952.547	15231.503	15105.615	8.083251275	0.586847165	4	High	4309	Unique	Used	91699	1	48.436	1	7.0514	-3.3663	1	24	24/102	29.60445	4309	2374.22	MS2	594.311	178	Glioma_grade2_7rep.raw
308818200			mVIPGGIDVHTHFQMPYk	Unambiguous	N-Term(iTRAQ4plex); K18(iTRAQ4plex)	HCD	2.7313871	0	1	2	2.28064	2.4984	2.9441	3.2251654	15.59199766	7375.4585	6732.6533	16820.795	21713.92	6.44355421	17.9576357	4	High	4149	Unique	Used	86795	1	55.933	1	4.7995	-3.7854	1	30	29/102	38.66931	4149	2358.23	MS2	590.312	161	Glioma_grade2_1rep.raw
308818200			gAPLVVIcQGk	Unambiguous	N-Term(iTRAQ4plex); C8(Methylthio); K11(iTRAQ4plex)	HCD	1.880477	0	1	2	1.78899	1.9463	1.82	1.9800029	4.962251731	6871.759	6316.468	12293.531	12506.624	5.954553734	1.215149706	3	High	5540	Unique	Used	52993	1	52.333	1	4.1341	-2.103	1	80	19/40	12.91274	5540	1418.81	MS2	473.607	189	Glioma_grade2_2.raw
308818200			aALAGGTTMIIDHVVPEPESSLTEAYEk	Unambiguous	N-Term(iTRAQ4plex); K28(iTRAQ4plex)	HCD	2.5866535	0	1	2	2.6817	2.401	2.794	2.501555	6.803104502	4532.632	5062.488	12155.158	12664.092	7.809485877	2.899931969	3	High	6573	Unique	Used	61959	1	61.633	1	7.1357	-0.9259	1	33	34/108	0	6573	3217.65	MS2	1073.22	93	Glioma_grade2_3.raw
308818200			fIPcSPFSDYVYk	Unambiguous	N-Term(iTRAQ4plex); C4(Methylthio); K13(iTRAQ4plex)	HCD	3.014116	0	1	2	3.07251	2.9893	3.0396	2.9573019	1.702357745	3619.0454	3719.7676	11119.56	11000.476	1.940950141	0.761346898	3	High	7571	Unique	Used	70498	1	69.772	1	5.0307	0.2551	1	94	21/48	17.50096	7571	1899.93	MS2	633.98	142	Glioma_grade2_4.raw
309384267	NP_001185462.1	OXR1	oxidation resistance protein 1 isoform 4 [Homo sapiens]	3	3	0.500457823	14.68572966	14.441484	6.53	1.07E-04	873	97.855	5.4272	Unclassified	Biological_process unknown	Molecular function unknown	Mitochondrion	Cytoplasm, Nucleus																								
309384267			eWEVVSVAEYHR	Unambiguous	N-Term(iTRAQ4plex)	HCD	0.5004578	0	1	2	0.49176	0.481	0.5204	0.5089632	0.035049042	28459.69	29097.152	13995.391	14809.382	1.5662906	3.996410983	3	High	4153	Unique	Used	59712	1	42.709	1	4.5917	4.2704	1	36	19/44	41.0189	4153	1647.83	MS2	549.949	149	Glioma_grade2_3.raw
309384267			sQSVDINAPGFNPLAGAGk	Unambiguous	N-Term(iTRAQ4plex); K19(iTRAQ4plex)	HCD	0.53920674	0	1	3	0.48393	0.4908	0.5869	0.5952779	0.111328286	12221.478	12049.133	5914.2944	7172.5825	1.004229504	13.59750008	2	High	5283	Unique	Used	52750	1	50.484	1	4.211	-3.7607	1	100	22/36	27.573	5283	2131.13	MS2	1066.07	241	Glioma_grade2_2.raw
309384267			aDLESESFRPNLSDPSELLLPDQIEk	Unambiguous	N-Term(iTRAQ4plex); K26(iTRAQ4plex)	HCD	0.40226907	0	1	6	0.34326	0.4306	0.3797	0.47629544	0.142862355	4867.1694	3880.1147	1670.6809	1848.0809	15.9581663	7.129822938	3	High	6802	Unique	Used	69816	1	63.165	1	5.6389	2.3433	1	51	32/100	16.39443	6802	3230.67	MS2	1077.56	104	Glioma_grade2_4.raw
313569778	NP_001186274.1	RPL17	60S ribosomal protein L17 isoform b [Homo sapiens]	4	15	2.111802578	21.16583046	128.81481	40.41	6.18E-04	146	17.083	10.096	Ribosomal subunit	Protein metabolism	Structural constituent of ribosome	Ribosome	Nucleolus																								
313569778			eQIVPkPEEEVAQk	Unambiguous	N-Term(iTRAQ4plex); K6(iTRAQ4plex); K14(iTRAQ4plex)	HCD	2.8733997	0	1	4	2.9983	2.7268	3.0346	2.7598057	5.519157724	504924.53	555192.2	1513916.5	1532222.6	6.705791792	0.849886825	3	High	1556	Unique	Used	79034	1	23.338	1	3.6456	-6.7812	1	1	27/52	5.660579	1556	2056.15	MS2	686.055	138	Glioma_grade2_6.raw
313569778			eQIVPkPEEEVAQk	Unambiguous	N-Term(iTRAQ4plex); K6(iTRAQ4plex); K14(iTRAQ4plex)	HCD	2.9927754	0	1	4	2.98651	2.907	3.0809	2.9988744	2.378261827	137027.3	140775.89	409233.34	422169.22	1.908295876	2.200389777	2	High	1577	Unique	Used	79054	1	23.478	1	3.3848	-8.7932	1	11	15/26	3.521149	1577	2056.15	MS2	1028.58	173	Glioma_grade2_6.raw
313569778			iNPYmSSPcHIEMILTEk	Unambiguous	N-Term(iTRAQ4plex); M5(Oxidation); C9(Methylthio); K18(iTRAQ4plex)	HCD	1.8163484	0	1	4	1.67557	1.8591	1.7778	1.9725443	6.904052097	80203.375	72285.51	134386.58	142586.36	7.343192304	4.186777266	3	High	5771	Unique	Used	82726	1	58.927	1	5.0377	2.5658	1	14	20/68	31.01829	5771	2456.2	MS2	819.405	109	Glioma_grade2_6.raw
313569778			eQIVPkPEEEVAQk	Unambiguous	N-Term(iTRAQ4plex); K6(iTRAQ4plex); K14(iTRAQ4plex)	HCD	2.3664536	0	1	4	2.23572	2.4035	2.332	2.5069976	4.834411213	44274.64	41183.652	98985.77	103247.32	5.115146873	2.980096782	2	High	1641	Unique	Used	72383	1	24.246	1	4.053	3.5554	1	20	15/26	3.217704	1641	2056.17	MS2	1028.59	136	Glioma_grade2_5.raw
313569778			gLDVDSLVIEHIQVNk	Unambiguous	N-Term(iTRAQ4plex); K16(iTRAQ4plex)	HCD	2.279839	0	1	4	1.964	2.4515	2.1423	2.6740856	13.71103299	43189.168	34600.188	84823.35	92523.86	15.61479954	6.140590359	3	High	5777	Unique	Used	76117	1	57.188	1	6.4649	-0.6689	1	6	30/60	24.0516	5777	2067.17	MS2	689.727	116	Glioma_grade2_5.raw
313569778			sAEFLLHMLk	Unambiguous	N-Term(iTRAQ4plex); K10(iTRAQ4plex)	HCD	2.1118026	0	1	4	1.80763	2.117	2.1073	2.468045	12.70816227	31614.602	26993.908	57147.426	66622.18	11.14965749	10.82602267	3	High	5394	Unique	Used	82404	1	55.595	1	4.0816	-0.7848	1	23	21/36	8.306961	5394	1476.85	MS2	492.954	276	Glioma_grade2_6.raw
313569778			gLDVDSLVIEHIQVNk	Unambiguous	N-Term(iTRAQ4plex); K16(iTRAQ4plex)	HCD	2.3069217	0	1	4	1.82007	2.4442	2.2047	2.960719	20.24852124	18743.951	13957.624	34115.367	41324.605	20.69896804	13.514589	2	High	6199	Unique	Used	69268	1	58.236	1	6.1996	-1.2529	1	50	24/30	16.51348	6199	2067.17	MS2	1034.09	154	Glioma_grade2_4.raw
313569778			iNPYMSSPcHIEMILTEk	Unambiguous	N-Term(iTRAQ4plex); C9(Methylthio); K18(iTRAQ4plex)	HCD	2.6318264	0	1	4	2.95517	2.2729	3.0985	2.38314	15.31999895	18483.254	24031.787	54621.1	57271.113	18.45655193	3.349370099	3	High	6343	Unique	Used	83183	1	64.491	1	5.2746	0.6738	1	22	29/68	22.10425	6343	2440.2	MS2	814.072	123	Glioma_grade2_6.raw
313569778			gLDVDSLVIEHIQVNk	Unambiguous	N-Term(iTRAQ4plex); K16(iTRAQ4plex)	HCD	2.2427835	0	1	4	2.20887	2.4335	2.0697	2.280145	6.734643862	16730.832	15186.567	36956.25	34627.574	6.842413778	4.600543807	2	High	5693	Unique	Used	76043	1	56.475	1	6.6433	-1.9615	1	89	24/30	14.98206	5693	2067.16	MS2	1034.09	152	Glioma_grade2_5.raw
313569778			gLDVDSLVIEHIQVNk	Unambiguous	N-Term(iTRAQ4plex); K16(iTRAQ4plex)	HCD	1.9665225	0	1	4	2.09765	2.022	1.909	1.8401284	5.836857428	16002.03	16600.883	33566.605	30547.758	2.597639157	6.658873566	3	High	5876	Unique	Used	76208	1	57.946	1	5.6919	-0.9346	1	20	31/60	24.52143	5876	2067.17	MS2	689.727	106	Glioma_grade2_5.raw
313569778			gLDVDSLVIEHIQVNk	Unambiguous	N-Term(iTRAQ4plex); K16(iTRAQ4plex)	HCD	1.7293581	0	1	4	1.55807	1.8636	1.6171	1.9342352	10.53373512	14941.991	12492.289	23280.668	24163.023	12.62800333	2.630146141	3	High	5970	Unique	Used	76291	1	58.73	1	6.76	-1.2003	1	38	30/60	43.79425	5970	2067.17	MS2	689.727	117	Glioma_grade2_5.raw
313569778			gLDVDSLVIEHIQVNk	Unambiguous	N-Term(iTRAQ4plex); K16(iTRAQ4plex)	HCD	1.709221	0	1	4	1.64631	1.5002	1.9386	1.7665449	10.84501672	11959.643	13124.754	19689.307	23185.469	6.568688009	11.53200128	2	High	5790	Unique	Used	76130	1	57.266	1	6.9151	-1.2529	1	100	24/30	24.33382	5790	2067.17	MS2	1034.09	161	Glioma_grade2_5.raw
313569778			gLDVDSLVIEHIQVNk	Unambiguous	N-Term(iTRAQ4plex); K16(iTRAQ4plex)	HCD	1.7363578	0	1	4	1.72268	1.8103	1.666	1.7507318	3.456246376	9499.236	9039.277	16364.144	15825.351	3.508804918	2.36713365	2	High	5893	Unique	Used	76224	1	58.074	1	6.3022	-5.1503	1	100	25/30	39.38824	5893	2067.16	MS2	1034.08	176	Glioma_grade2_5.raw
313569778			sAEFLLHMLk	Unambiguous	N-Term(iTRAQ4plex); K10(iTRAQ4plex)	HCD	1.6250534	0	1	4	1.74407	1.4677	1.8121	1.5248954	10.18062469	5558.9863	6605.8125	9695.274	10073.174	12.16983391	2.703456059	3	High	5517	Unique	Used	82508	1	56.694	1	3.559	-0.2268	1	99	19/36	0	5517	1476.85	MS2	492.954	305	Glioma_grade2_6.raw
313569778			gLDVDSLVIEHIQVNk	Unambiguous	N-Term(iTRAQ4plex); K16(iTRAQ4plex)	HCD	1.5783011	0	1	4	1.41271	1.6611	1.5079	1.7730076	10.06496457	4989.7456	4243.7197	7049.04	7524.1475	11.4262621	4.610545703	3	High	6558	Unique	Used	76806	1	63.745	1	5.3526	-1.5547	1	86	28/60	51.08811	6558	2067.17	MS2	689.727	116	Glioma_grade2_5.raw
313760653	NP_001186512.1	PACSIN1	protein kinase C and casein kinase substrate in neurons protein 1 [Homo sapiens]	7	12	0.490227729	12.33475065	93.96866	27.03	4.86E-04	444	50.934	5.2368	Adapter molecule	Cell communication,Signal transduction	Receptor signaling complex scaffold activity	Cytoplasm	-																								
313760653			aYGQQLTDWAk	Unambiguous	N-Term(iTRAQ4plex); K11(iTRAQ4plex)	HCD	0.4635888	0	1	1	0.42378	0.4833	0.4463	0.50899345	0.081478937	809962.2	710214.25	343242.4	361494.38	9.279508554	3.662672134	2	High	4080	Unique	Used	51618	1	41.691	1	4.8291	1.3719	1	16	18/20	28.28296	4080	1568.83	MS2	784.92	245	Glioma_grade2_2.raw
313760653			nNLLNEDLEk	Unambiguous	N-Term(iTRAQ4plex); K10(iTRAQ4plex)	HCD	0.49094376	0	1	1	0.44107	0.495	0.4874	0.54691386	0.088013588	415986.12	370688.7	183478.31	202734.78	8.143164631	7.051226834	2	High	2792	Unique	Used	50407	1	32.317	1	4.6859	-1.8719	1	25	18/18	19.89652	2792	1489.81	MS2	745.407	225	Glioma_grade2_2.raw
313760653			aWGAIMTEADk	Unambiguous	N-Term(iTRAQ4plex); K11(iTRAQ4plex)	HCD	0.4797505	0	1	1	0.48987	0.4837	0.4758	0.46975896	0.01839357	243221.08	246329.94	119146.664	115715.7	0.898086572	2.065943182	2	High	4589	Unique	Used	60115	1	46.147	1	4.6992	1.0949	1	46	20/20	7.615851	4589	1480.77	MS2	740.89	217	Glioma_grade2_3.raw
313760653			aWGAIMTEADk	Unambiguous	N-Term(iTRAQ4plex); K11(iTRAQ4plex)	HCD	0.536122	0	1	1	0.5204	0.5357	0.5366	0.5523008	0.024294289	165461.44	160745.75	86106.86	88780.01	2.044403973	2.16162882	2	High	4728	Unique	Used	52227	1	46.459	1	4.0765	0.4354	1	28	19/20	51.26425	4728	1480.77	MS2	740.889	213	Glioma_grade2_2.raw
313760653			aWGAImTEADk	Unambiguous	N-Term(iTRAQ4plex); M6(Oxidation); K11(iTRAQ4plex)	HCD	0.4467859	0	1	1	0.42758	0.4847	0.4133	0.46856192	0.074977632	138618.34	122269.97	59270.164	57291.05	8.862063071	2.401218865	2	High	2856	Unique	Used	58503	1	32.814	1	3.9531	1.2182	1	47	17/20	4.811196	2856	1496.77	MS2	748.887	200	Glioma_grade2_3.raw
313760653			hLNLAENSSYIHVYR	Unambiguous	N-Term(iTRAQ4plex)	HCD	0.5347183	0	1	1	0.5186	0.5192	0.5502	0.5508556	0.034141333	122101.19	121954.805	63321.72	67179.484	0.084824654	4.180576119	3	High	2478	Unique	Used	79893	1	30.172	1	6.6243	6.2106	1	13	29/56	34.74851	2478	1960.03	MS2	654.014	178	Glioma_grade2_6.raw
313760653			aLYDYDGQEQDELSFk	Unambiguous	N-Term(iTRAQ4plex); K16(iTRAQ4plex)	HCD	0.47167552	0	1	1	0.45272	0.4434	0.5005	0.49024633	0.059126806	120871.81	123403.234	54720.574	60497.98	1.46555051	7.091293578	2	High	5638	Unique	Used	53086	1	53.008	1	6.1157	-3.6311	1	38	23/30	6.432156	5638	2209.05	MS2	1105.03	94	Glioma_grade2_2.raw
313760653			hLNLAENSSYIHVYR	Unambiguous	N-Term(iTRAQ4plex)	HCD	0.56804144	0	1	1	0.59429	0.5472	0.5907	0.5438784	0.047793075	51136.613	55540.234	30389.736	30207.133	5.837874587	0.426160037	2	High	2500	Unique	Used	79913	1	30.343	1	4.6205	5.003	1	76	22/28	0	2500	1960.03	MS2	980.516	98	Glioma_grade2_6.raw
313760653			hLNLAENSSYIHVYR	Unambiguous	N-Term(iTRAQ4plex)	HCD	0.5363744	0	1	1	0.49917	0.5342	0.5384	0.5761905	0.058661214	26378.246	24648.418	13167.213	14202.185	4.794250743	5.347839361	4	High	2894	Unique	Used	86031	1	39.858	1	5.962	-8.3388	1	28	23/84	20.73436	2894	1960	MS2	490.755	173	Glioma_grade2_1rep.raw
313760653			kAEGVALTNATGAVESTSQAGDR	Unambiguous	N-Term(iTRAQ4plex); K1(iTRAQ4plex)	HCD	0.33375484	1	1	1	0.33218	0.292	0.3812	0.33514023	0.108809117	16047.957	18254.01	5330.7944	6117.6533	9.095192914	9.719977391	2	High	2406	Unique	Used	73087	1	30.001	1	4.9136	11.475	1	81	26/44	0	2406	2521.34	MS2	1261.17	125	Glioma_grade2_5.raw
313760653			hLNLAENSSYIHVYR	Unambiguous	N-Term(iTRAQ4plex)	HCD	0.4895117	0	1	1	0.39469	0.4639	0.5113	0.60097605	0.175777045	10993.19	9352.289	4338.848	5620.502	11.40589734	18.19930486	4	High	3026	Unique	Used	95502	1	42.254	1	6.7495	-0.4914	1	100	26/84	18.98754	3026	1960.01	MS2	490.759	169	Glioma_grade2_8rep.raw
313760653			sTSGPGMPMNWPQFEEWNPDLPHTTTk	Unambiguous	N-Term(iTRAQ4plex); K27(iTRAQ4plex)	HCD	0.49112916	0	1	1	0.43199	0.4479	0.5328	0.5524566	0.122464481	10179.238	9816.767	4397.283	5423.338	2.563569094	14.77565316	3	High	6495	Unique	Used	76752	1	63.181	1	6.4776	-3.2876	1	24	30/104	0	6495	3373.58	MS2	1125.2	53	Glioma_grade2_5.raw
315113881	NP_001186693.1	PTPRN	receptor-type tyrosine-protein phosphatase-like N isoform 3 [Homo sapiens]	6	9	0.4367899	21.62440902	71.565947	15.86	9.90E-05	889	96.201	6.5659	Receptor tyrosine phosphatase	Cell communication,Signal transduction	Receptor signaling protein tyrosine phosphatase activity	Plasma membrane	-																								
315113881			lAALGPEGAHGDTTFEYQDLcR	Unambiguous	N-Term(iTRAQ4plex); C21(Methylthio)	HCD	0.4527057	0	1	3	0.48702	0.5046	0.4026	0.41715264	0.111527594	126319.34	121905.51	61519.605	50853.203	2.514695144	13.42368375	3	High	6372	Unique	Used	61772	1	60.049	1	7.5833	1.9003	1	11	30/84	26.22319	6372	2554.19	MS2	852.067	39	Glioma_grade2_3.raw
315113881			eIDIAATLEHVR	Unambiguous	N-Term(iTRAQ4plex)	HCD	0.2935962	0	1	3	0.29849	0.3119	0.2761	0.28848177	0.0517771	44166.344	42263.426	13183.225	12192.229	3.11366378	5.522975011	3	High	5131	Unique	Used	60618	1	50.386	1	5.7217	-0.9933	1	23	26/44	26.53331	5131	1510.83	MS2	504.283	283	Glioma_grade2_3.raw
315113881			eIDIAATLEHVR	Unambiguous	N-Term(iTRAQ4plex)	HCD	0.36045855	0	1	3	0.34035	0.3574	0.3633	0.3815789	0.047157085	26236.867	24983.102	8929.651	9533.024	3.461719133	4.621747822	3	High	5288	Unique	Used	52754	1	50.532	1	5.9489	-1.1145	1	25	26/44	11.38606	5288	1510.83	MS2	504.283	263	Glioma_grade2_2.raw
315113881			lAALGPEGAHGDTTFEYQDLcR	Unambiguous	N-Term(iTRAQ4plex); C21(Methylthio)	HCD	0.4367899	0	1	3	0.4183	0.4537	0.4212	0.45684478	0.047013189	21104.854	19459.375	8828.132	8889.914	5.736726115	0.493129673	3	High	6431	Unique	Used	69480	1	60.094	1	7.6063	-1.3974	1	100	33/84	9.319251	6431	2554.18	MS2	852.064	43	Glioma_grade2_4.raw
315113881			hPDFLPYDHAR	Unambiguous	N-Term(iTRAQ4plex)	HCD	0.5004434	0	1	3	0.55487	0.5529	0.4478	0.4462155	0.123286133	20841.002	20916.219	11563.983	9333.142	0.254741333	15.09722317	4	High	1687	Unique	Used	89550	1	24.1	1	4.5323	-1.3548	1	23	23/60	32.90565	1687	1511.75	MS2	378.693	151	Glioma_grade2_7rep.raw
315113881			lAALGPEGAHGDTTFEYQDLcR	Unambiguous	N-Term(iTRAQ4plex); C21(Methylthio)	HCD	0.42423794	0	1	3	0.39365	0.3829	0.4668	0.45398435	0.0994085	19820.736	20378.219	7802.5293	9251.393	1.961245061	12.01484713	3	High	6602	Unique	Used	53994	1	60.065	1	7.204	-2.9745	1	49	33/84	30.92732	6602	2554.17	MS2	852.063	40	Glioma_grade2_2.raw
315113881			sDYINASPIIEHDPR	Unambiguous	N-Term(iTRAQ4plex)	HCD	0.6370972	0	1	3	0.66132	0.6492	0.6248	0.61331964	0.034484788	18803.816	19156.266	12435.349	11748.913	1.313062417	4.014044757	2	High	3026	Unique	Used	58663	1	34.07	1	4.0395	-2.4998	1	100	17/28	64.53657	3026	1870.94	MS2	935.972	186	Glioma_grade2_3.raw
315113881			qLTPEQLSTLLTLLQLLPk	Unambiguous	N-Term(iTRAQ4plex); K19(iTRAQ4plex)	HCD	0.519892	0	1	3	0.42571	0.5113	0.527	0.633026	0.162288707	6689.4404	5569.1167	2847.7302	3525.3955	12.92466118	15.03757345	3	High	8414	Unique	Used	71223	1	77.326	1	6.2489	0.1462	1	39	29/72	5.464304	8414	2437.49	MS2	813.168	14	Glioma_grade2_4.raw
315113881			skDQFEFALTAVAEEVNAILk	Unambiguous	N-Term(iTRAQ4plex); K2(iTRAQ4plex); K21(iTRAQ4plex)	HCD	0.43272945	1	1	3	0.31698	0.3935	0.4644	0.5764194	0.251889384	2417.084	1947.1642	766.15546	1122.3833	15.22752428	26.67576929	4	High	6912	Unique	Used	93534	1	77.197	1	6.4836	2.7475	1	45	29/120	24.63405	6912	2755.53	MS2	689.638	80	Glioma_grade2_7rep.raw
315221152	NP_001186731.1	RPL11	60S ribosomal protein L11 isoform 2 [Homo sapiens]	2	6	2.291549683	18.64295945	31.755749	12.99	5.13E-05	177	20.112	9.5981	Ribosomal subunit	Protein metabolism	Structural constituent of ribosome	Ribosome	Nucleoplasm, Mitochondrion, Nucleolus																								
315221152			yDGIILPGk	Unambiguous	N-Term(iTRAQ4plex); K9(iTRAQ4plex)	HCD	2.3484428	0	1	2	2.33316	2.3312	2.3657	2.363711	0.799952347	664486.6	665033.7	1550355	1571947.5	0.058195143	0.978009221	2	High	4095	Unique	Used	51632	1	41.807	1	3.6504	0.5204	1	4	13/16	18.00236	4095	1263.76	MS2	632.382	289	Glioma_grade2_2.raw
315221152			yDGIILPGk	Unambiguous	N-Term(iTRAQ4plex); K9(iTRAQ4plex)	HCD	2.8816466	0	1	2	2.81309	2.7855	2.9787	2.9495354	3.351120231	417018.47	421146.53	1173108.6	1242186.6	0.696516609	4.044683418	2	High	3923	Unique	Used	59497	1	40.946	1	3.6394	0.6169	1	7	13/16	9.337143	3923	1263.76	MS2	632.382	246	Glioma_grade2_3.raw
315221152			yDGIILPGk	Unambiguous	N-Term(iTRAQ4plex); K9(iTRAQ4plex)	HCD	1.9413842	0	1	2	1.85307	2.0934	1.8068	2.0411477	7.181429539	268833.66	237973.34	498168.34	485738.75	8.611381272	1.786560432	2	High	3997	Unique	Used	51540	1	41.058	1	3.7108	-0.1558	1	14	13/16	32.60877	3997	1263.75	MS2	632.381	271	Glioma_grade2_2.raw
315221152			vLEQLTGQTPVFSk	Unambiguous	N-Term(iTRAQ4plex); K14(iTRAQ4plex)	HCD	2.2346566	0	1	2	2.16205	2.313	2.1614	2.3123388	3.895978087	73201.875	68423.46	158265.77	158218.23	4.771532791	0.021243321	2	High	6007	Unique	Used	53432	1	55.77	1	4.8256	-0.8887	1	24	22/26	14.50709	6007	1835.05	MS2	918.029	191	Glioma_grade2_2.raw

315221152			vLEQLTGQTPVFSk	Unambiguous	N-Term(iTRAQ4plex); K14(iTRAQ4plex)	HCD	2.4902585	0	1	2	2.36754	2.3476	2.6342	2.6119428	6.174542827	28221.031	28461.006	66814.4	74338.52	0.598736244	7.538428924	2	High	5789	Unique	Used	61226	1	55.543	1	5.7186	4.0338	1	80	22/26	27.27774	5789	1835.06	MS2	918.033	173	Glioma_grade 2_3.raw
315221152			vLEQLTGQTPVFSk	Unambiguous	N-Term(iTRAQ4plex); K14(iTRAQ4plex)	HCD	1.6769623	0	1	2	1.41992	1.6257	1.7218	1.9712498	13.58289629	14810.613	12936.257	21029.86	25500.594	9.553292591	13.58803131	3	High	5970	Unique	Used	53397	1	55.496	1	5.3781	-2.6256	1	19	23/52	24.83052	5970	1835.05	MS2	612.354	173	Glioma_grade 2_2.raw
319803020	NP_001188357 .1	GORASP2	Golgi reassembly-stacking protein 2 isoform 2 [Homo sapiens]	2	2	2.222519636	25.0326113	14.662366	6.77	1.73E-04	384	39.743	4.6274	Transport/cargo protein	Transport	Transporter activity	Golgi apparatus	Cytoplasm, Nucleus																								
319803020			iPTRPFEEGk	Unambiguous	N-Term(iTRAQ4plex); K10(iTRAQ4plex)	HCD	1.8291174	0	1	2	1.6294	1.8736	1.7904	2.058771	9.721374531	207108.55	180115.48	337461.7	370816.53	9.858366921	6.659932631	2	High	1500	Unique	Used	78981	1	22.939	1	3.5704	-6.3091	1	20	16/18	0	1500	1461.82	MS2	731.414	269	Glioma_grade 2_6.raw
319803020			iSLPGQMAGTPITPLk	Unambiguous	N-Term(iTRAQ4plex); K16(iTRAQ4plex)	HCD	2.6159217	0	1	2	2.53093	2.691	2.5453	2.706288	3.554894194	22209.674	20888.465	56211.15	56530.203	4.335388325	0.40021613	2	High	5914	Unique	Used	69006	1	55.961	1	6.3607	0.0807	1	83	19/30	6.096276	5914	1912.12	MS2	956.563	127	Glioma_grade 2_4.raw
331284207	NP_001193594 .1	IGSF8	immunoglobulin superfamily member 8 precursor [Homo sapiens]	3	12	0.445630699	27.75017197	85.982544	12.89	3.71E-04	613	64.994	8.0015	Immunoglobulin	Immune response	Antigen binding	Plasma membrane	-																								
331284207			hAAYSVGWEMAPAGAPGPGR	Unambiguous	N-Term(iTRAQ4plex)	HCD	0.27345577	0	1	1	0.25387	0.2681	0.2785	0.29413822	0.062045821	150890.78	142868.33	38307.082	42023.04	3.86216366	6.541952222	2	High	3222	Unique	Used	73844	1	36.139	1	5.9995	-1.9418	1	23	29/38	0	3222	2126.03	MS2	1063.52	75	Glioma_grade 2_5.raw
331284207			mTVHEGQELALGcLAR	Unambiguous	N-Term(iTRAQ4plex); M1(Oxidation); C13(Methylthio)	HCD	0.6115381	0	1	1	0.52078	0.5728	0.6468	0.71135336	0.136302493	143283.94	130279.72	74619.69	92674.92	6.722656179	15.2628654	3	High	5179	Unique	Used	68330	1	50.186	1	6.2784	0.4269	1	7	20/60	32.9831	5179	1933.94	MS2	645.319	120	Glioma_grade 2_4.raw
331284207			lQAQDAGIYEcHTPSTDTR	Unambiguous	N-Term(iTRAQ4plex); C11(Methylthio)	HCD	0.44740885	0	1	1	0.40922	0.4128	0.4817	0.48593202	0.094058574	126415.016	125310.71	51731.836	60892.484	0.620407198	11.50294416	3	High	3608	Unique	Used	51172	1	38.263	1	6.1849	-1.4398	1	8	28/72	29.52439	3608	2296.04	MS2	766.019	58	Glioma_grade 2_2.raw
331284207			mTVHEGQELALGcLAR	Unambiguous	N-Term(iTRAQ4plex); C13(Methylthio)	HCD	0.24695823	0	1	1	0.1876	0.2195	0.2704	0.31641504	0.228161334	89709.58	76669.48	16829.303	24259.375	11.08401879	25.57324573	3	High	6256	Unique	Used	69321	1	58.682	1	5.8549	1.4389	1	4	23/60	7.360448	6256	1917.95	MS2	639.988	125	Glioma_grade 2_4.raw
331284207			mTVHEGQELALGcLAR	Unambiguous	N-Term(iTRAQ4plex); C13(Methylthio)	HCD	0.27069595	0	1	1	0.24635	0.2503	0.2908	0.29543212	0.096027041	69083.83	68004.31	17018.549	20090.656	1.113642526	11.70764877	2	High	6247	Unique	Used	69313	1	58.602	1	5.7462	0.8094	1	51	25/30	3.523262	6247	1917.95	MS2	959.478	152	Glioma_grade 2_4.raw
331284207			mTVHEGQELALGcLAR	Unambiguous	N-Term(iTRAQ4plex); M1(Oxidation); C13(Methylthio)	HCD	0.3843155	0	1	1	0.3543	0.4037	0.3673	0.41851875	0.078102341	49397.344	43351.41	17501.406	18143.379	9.21871345	2.547039976	2	High	5159	Unique	Used	68312	1	50.02	1	5.074	-1.4282	1	93	20/30	7.858075	5159	1933.94	MS2	967.473	137	Glioma_grade 2_4.raw
331284207			hAAYSVGWEmAPAGAPGPGR	Unambiguous	N-Term(iTRAQ4plex); M10(Oxidation)	HCD	0.39935452	0	1	1	0.39619	0.5812	0.2754	0.40399247	0.30400124	39630.633	27017.6	15701.366	10914.907	26.76368379	25.43209274	3	High	2349	Unique	Used	73033	1	29.599	1	5.4094	12.436	1	8	29/76	3.075261	2349	2142.06	MS2	714.691	102	Glioma_grade 2_5.raw
331284207			lQAQDAGIYEcHTPSTDTR	Unambiguous	N-Term(iTRAQ4plex); C11(Methylthio)	HCD	0.47197294	0	1	1	0.39564	0.3571	0.5992	0.54087555	0.243832028	35224.156	39021.992	13936.129	21106.043	7.233979571	28.93596213	3	High	3572	Unique	Used	59172	1	38.219	1	7.3113	1.2716	1	29	32/72	20.19584	3572	2296.05	MS2	766.021	53	Glioma_grade 2_3.raw
331284207			hAAYSVGWEmAPAGAPGPGR	Unambiguous	N-Term(iTRAQ4plex); M10(Oxidation)	HCD	0.5610731	0	1	1	0.53138	0.5197	0.6034	0.5901172	0.074367791	26662.805	27261.22	14168.004	16087.316	1.56940549	8.971371186	3	High	2313	Unique	Used	72999	1	29.346	1	5.8361	1.6655	1	17	27/76	0	2313	2142.03	MS2	714.683	72	Glioma_grade 2_5.raw
331284207			hAAYSVGWEMAPAGAPGPGR	Unambiguous	N-Term(iTRAQ4plex)	HCD	0.49792495	0	1	1	0.47845	0.5041	0.4921	0.5184407	0.034208031	18074.67	17155.865	8647.863	8894.299	3.688239455	1.986717107	3	High	3184	Unique	Used	73809	1	35.849	1	4.3554	0.4346	1	11	26/76	11.1499	3184	2126.04	MS2	709.35	78	Glioma_grade 2_5.raw
331284207			mTVHEGQELALGcLAR	Unambiguous	N-Term(iTRAQ4plex); C13(Methylthio)	HCD	0.44385254	0	1	1	0.42337	0.4141	0.4743	0.46388212	0.066710312	17836.805	18236.21	7551.6475	8459.452	1.565835204	8.01837148	3	High	5773	Unique	Used	76114	1	57.141	1	5.5797	-1.1388	1	39	19/60	29.40719	5773	1917.94	MS2	639.986	128	Glioma_grade 2_5.raw
331284207			mTVHEGQELALGcLAR	Unambiguous	N-Term(iTRAQ4plex); C13(Methylthio)	HCD	0.5425687	0	1	1	0.54962	0.625	0.4701	0.5345473	0.116733373	11187.813	9839.086	6149.0796	5259.457	9.071180754	11.02785038	2	High	6342	Unique	Used	69402	1	59.322	1	4.6255	-1.8001	1	100	21/30	32.32669	6342	1917.94	MS2	959.475	173	Glioma_grade 2_4.raw
332801090	NP_001193929 .1	HNRPDL	heterogeneous nuclear ribonucleoprotein D-like isoform b [Homo sapiens]	3	6	2.307659626	28.75055926	46.233708	13.77	9.67E-04	363	40.016	9.9644	Ribonucleoprotein	Regulation of nucleobase, nucleoside, nucleotide and nucleic acid metabolism	RNA binding	Nucleus	Mitochondrion																								
332801090			vFVGGLSPDTSEEQIk	Unambiguous	N-Term(iTRAQ4plex); K16(iTRAQ4plex)	HCD	3.5229647	0	1	2	3.54857	3.4747	3.5722	3.4978886	1.273014739	67731.58	69170.805	240350.5	241951.78	1.486731962	0.469529585	2	High	4768	Unique	Used	52265	1	46.747	1	6.2956	-0.3002	1	36	23/30	11.27776	4768	1994.07	MS2	997.538	216	Glioma_grade 2_2.raw
332801090			gFcFITYTDEEPVk	Unambiguous	N-Term(iTRAQ4plex); C3(Methylthio); K14(iTRAQ4plex)	HCD	2.1376684	0	1	2	1.95821	2.3199	1.9838	2.3502774	9.791440756	57041.523	48148.28	111699.086	113161.82	11.95642972	0.919954606	2	High	7045	Unique	Used	54406	1	63.427	1	5.0287	-0.0406	1	33	18/26	33.537	7045	1982.95	MS2	991.978	111	Glioma_grade 2_2.raw
332801090			mFIGGLSWDTSk	Unambiguous	N-Term(iTRAQ4plex); M1(Oxidation); K12(iTRAQ4plex)	HCD	1.6177324	0	1	2	1.58545	1.6608	1.5766	1.651546	2.701063561	25608.273	24445.875	40600.734	40373.49	3.284201374	0.396881292	2	High	5495	Unique	Used	68625	1	52.584	1	3.5891	3.0645	1	100	17/22	52.26067	5495	1645.85	MS2	823.431	251	Glioma_grade 2_4.raw
332801090			mFIGGLSWDTSk	Unambiguous	N-Term(iTRAQ4plex); K12(iTRAQ4plex)	HCD	2.7563977	0	1	2	2.47081	3.2212	2.3998	3.128721	15.31776688	11019.496	8452.347	27227.11	26445.035	18.6448552	2.060698472	3	High	6324	Unique	Used	69385	1	59.186	1	4.2913	-0.7077	1	34	18/44	6.402082	6324	1629.85	MS2	543.956	225	Glioma_grade 2_4.raw
332801090			mFIGGLSWDTSk	Unambiguous	N-Term(iTRAQ4plex); K12(iTRAQ4plex)	HCD	1.8563328	0	1	2	1.85438	1.7525	1.9662	1.8581737	4.697090474	8497.566	8991.517	15757.758	16707.8	3.994218584	4.138423499	3	High	6416	Unique	Used	53818	1	58.737	1	4.2618	-1.8311	1	54	17/44	8.148794	6416	1629.85	MS2	543.956	229	Glioma_grade 2_2.raw
332801090			mFIGGLSWDTSk	Unambiguous	N-Term(iTRAQ4plex); K12(iTRAQ4plex)	HCD	2.4776511	0	1	2	2.0841	2.6637	2.3321	2.9806545	15.53632723	7660.8745	5993.9277	15966.01	17865.828	17.26439342	7.941479212	3	High	6236	Unique	Used	61645	1	58.987	1	4.8612	-1.6064	1	100	17/44	31.5867	6236	1629.85	MS2	543.956	230	Glioma_grade 2_3.raw
333360851	NP_001193943 .1	SERPINH1	serpin H1 precursor [Homo sapiens]	5	7	4.164913654	37.84552698	41.888165	16.03	7.32E-05	418	46.411	8.6899	Heat shock protein	Protein metabolism	Heat shock protein activity	Endoplasmic reticulum	Nucleolus, Plasma membrane																								
333360851			gVVEVTHDLQk	Unambiguous	N-Term(iTRAQ4plex); K11(iTRAQ4plex)	HCD	2.4274864	0	1	1	2.32572	2.6009	2.2724	2.5412924	6.588649551	41741.332	37324.41	97078.78	94852.24	7.90035082	1.640591013	2	High	1563	Unique	Used	72311	1	23.657	1	4.5953	3.8036	1	53	18/20	8.657027	1563	1512.87	MS2	756.938	167	Glioma_grade 2_5.raw
333360851			hLAGLGLTEAIDk	Unambiguous	N-Term(iTRAQ4plex); K13(iTRAQ4plex)	HCD	5.4752655	0	1	1	5.0668	5.5567	5.401	5.9232273	6.478748291	29808.26	27180.4	151032.34	160995.7	6.521218874	4.515721997	2	High	3139	Unique	Used	80464	1	35.907	1	5.8439	1.6784	1	100	21/24	54.07439	3139	1625.95	MS2	813.478	162	Glioma_grade 2_6.raw
333360851			hLAGLGLTEAIDk	Unambiguous	N-Term(iTRAQ4plex); K13(iTRAQ4plex)	HCD	2.087653	0	1	1	2.08247	2.3046	1.8916	2.0933874	8.064436787	23484.062	21220.191	48904.887	44422.08	7.161728151	6.792941698	3	High	3131	Unique	Used	80456	1	35.86	1	4.6084	1.3323	1	19	21/48	20.50303	3131	1625.95	MS2	542.654	164	Glioma_grade 2_6.raw
333360851			dEEVHAGLGELLR	Unambiguous	N-Term(iTRAQ4plex)	HCD	2.0449915	0	1	1	1.98893	1.96	2.1313	2.1002383	4.077958451	20611.75	20916.234	40995.297	43929.074	1.036904181	4.885508334	2	High	4026	Unique	Used	59593	1	41.736	1	4.5138	0.8492	1	100	19/24	14.78858	4026	1581.84	MS2	791.422	291	Glioma_grade 2_3.raw
333360851			lFYADHPFIFLVR	Unambiguous	N-Term(iTRAQ4plex)	HCD	4.2026434	0	1	1	4.14697	4.0628	4.3454	4.257188	2.946877904	20016.865	20431.713	83009.305	86981.65	1.450443247	3.304731227	2	High	6569	Unique	Used	83365	1	66.67	1	4.5665	0.0827	1	86	18/24	28.26568	6569	1781.99	MS2	891.497	237	Glioma_grade 2_6.raw
333360851			lSSLIILMPHHVEPLER	Unambiguous	N-Term(iTRAQ4plex)	HCD	4.250726	0	1	1	3.88785	4.5503	3.9948	4.6754327	9.199922936	13548.316	11575.99	52673.79	54122.76	11.10195911	1.91874459	3	High	5205	Unique	Used	92375	1	57.649	1	6.451	-1.1091	1	29	30/64	29.53297	5205	2128.2	MS2	710.073	109	Glioma_grade 2_7rep.raw
333360851			lFYADHPFIFLVR	Unambiguous	N-Term(iTRAQ4plex)	HCD	4.1649137	0	1	1	3.7549	4.2853	4.0594	4.6328487	8.852285191	11328.193	9926.085	42536.184	45986.05	9.329322546	5.511437144	3	High	6546	Unique	Used	83346	1	66.453	1	4.7413	-2.117	1	57	21/48	31.26519	6546	1781.98	MS2	594.666	184	Glioma_grade 2_6.raw
333470703	NP_001193992 .1	CXADR	coxsackievirus and adenovirus receptor isoform 2 precursor [Homo sapiens]	2	4	0.361742973	19.58354936	19.570244	9.13	1.17E-04	252	28.183	8.0894	Cell surface receptor	Cell communication,Signal transduction	Receptor activity	Plasma membrane	Mitochondrion, Cytoplasm																								
333470703			iYDDYYPDLk	Unambiguous	N-Term(iTRAQ4plex); K10(iTRAQ4plex)	HCD	0.510783	0	1	4	0.48979	0.5221	0.5002	0.5331599	0.038775849	257373.58	241466.98	126058.766	128740.52	4.509522933	1.488452868	2	High	4942	Unique	Used	52429	1	47.983	1	4.4154	-2.0793	1	37	16/18	26.82463	4942	1592.81	MS2	796.906	137	Glioma_grade 2_2.raw
333470703			iHLVVLVkPSGAR	Unambiguous	N-Term(iTRAQ4plex); K8(iTRAQ4plex)	HCD	0.3520209	0	1	3	0.34854	0.3915	0.3169	0.355932	0.08668056	39441.5	35115.754	13746.891	12498.821	8.205142132	6.725051013	4	High	2840	Unique	Used	85994	1	39.218	1	5.0228	7.1046	1	100	22/72	21.34414	2840	1677.09	MS2	420.028	6	Glioma_grade 2_1rep.raw
333470703			iHLVVLVkPSGAR	Unambiguous	N-Term(iTRAQ4plex); K8(iTRAQ4plex)	HCD	0.34809783	0	1	3	0.31665	0.341	0.3547	0.38195783	0.078182957	36429.277	33828.707	11535.513	12921.139	5.234652568	8.012425746	4	High	2838	Unique	Used	85993	1	39.188	1	5.0382	-6.7978	1	100	20/72	60.43361	2838	1677.07	MS2	420.022	10	Glioma_grade 2_1rep.raw
333470703			iHLVVLVkPSGAR	Unambiguous	N-Term(iTRAQ4plex); K8(iTRAQ4plex)	HCD	0.37146503	0	1	3	0.40221	0.4048	0.3384	0.34052438	0.099582932	22926.246	22782.158	9221.193	7757.881	0.445806867	12.18815394	4	High	2987	Unique	Used	95480	1	41.721	1	5.0938	0.1171	1	100	22/72	35.0441	2987	1677.08	MS2	420.025	9	Glioma_grade 2_8rep.raw
341916196	XP_003403644 .1	C4B	PREDICTED: complement C4-A isoform 2 [Homo sapiens]	15	17	2.522236824	32.86520957	129.39906	16.02	3.89E-04	1698	187.65	7.1226	Complement protein	Immune response	Complement activity	Extracellular	-																								
341916196			aEFQDALEk	Unambiguous	N-Term(iTRAQ4plex); K9(iTRAQ4plex)	HCD	1.6295538	0	1	6	1.66403	1.6454	1.6135	1.595464	1.894844924	539235	545330.44	897302.7	870055.06	0.794811783	2.180315888	2	High	2499	Unique	Used	50131	1	30.243	1	3.205	-0.3604	1	8	16/16	39.92896	2499	1338.71	MS2	669.861	251	Glioma_grade 2_2.raw
341916196			cSVFYGAPSk	Unambiguous	N-Term(iTRAQ4plex); C1(Methylthio); K10(iTRAQ4plex)	HCD	1.9646815	0	1	6	1.89408	1.9531	1.9759	2.0374777	3.01683466	119119.53	115521.99	225622.36	235373.48	2.168282007	2.991386246	2	High	3834	Unique	Used	59415	1	40.252	1	3.6412	1.0162	1	57	15/18	39.83	3834	1392.69	MS2	696.849	106	Glioma_grade 2_3.raw
341916196			yVLPNFEVk	Unambiguous	N-Term(iTRAQ4plex); K9(iTRAQ4plex)	HCD	3.7258139	0	1	6	3.60538	3.6493	3.8014	3.84772	3.134931837	96726.695	95562.086	348736.03	367696.16	0.856527268	3.742667257	2	High	5569	Unique	Used	53020	1	52.544	1	3.1259	-2.6937	1	21	14/16	29.2432	5569	1396.8	MS2	698.906	227	Glioma_grade 2_2.raw
341916196			lGQYASPTAk	Unambiguous	N-Term(iTRAQ4plex); K10(iTRAQ4plex)	HCD	4.623385	0	1	6	4.21978	4.6457	4.6031	5.0677247	7.483410166	94675.15	85995.555	399508.4	435801.8	6.794018413	6.144617713	2	High	1432	Unique	Used	57173	1	22.364	1	4.3136	-0.6085	1	35	17/18	35.98476	1432	1323.75	MS2	662.379	276	Glioma_grade 2_3.raw
341916196			fGLLDEDGkk	Unambiguous	N-Term(iTRAQ4plex); K9(iTRAQ4plex); K10(iTRAQ4plex)	HCD	3.4358835	1	1	6	3.14562	3.5631	3.3236	3.7646706	7.85878675	83373.625	73605.11	262261.62	277099	8.800406244	3.890388591	2	High	2352	Unique	Used	90127	1	29.72	1	4.2083	-1.5905	1	36	17/18	11.98336	2352	1553.89	MS2	777.447	193	Glioma_grade 2_7rep.raw
341916196			kYVLPNFEVk	Unambiguous	N-Term(iTRAQ4plex); K1(iTRAQ4plex); K10(iTRAQ4plex)	HCD	2.1603096	1	1	6	2.04008	2.222	2.1036	2.291257	5.238315845	60368.07	55425.094	123155.945	126993.14	6.036991698	2.169351613	2	High	3544	Unique	Used	91126	1	40.462	1	4.1972	2.2734	1	91	15/18	13.18549	3544	1669.01	MS2	835.008	103	Glioma_grade 2_7rep.raw
341916196			gPEVQLVAHSPWLk	Unambiguous	N-Term(iTRAQ4plex); K14(iTRAQ4plex)	HCD	2.1127343	0	1	6	2.33029	2.2202	2	1.9054629	9.243206461	33570.52	35235.555	78228.91	67140.04	3.422248804	10.78774411	3	High	4364	Unique	Used	81524	1	46.521	1	5.4801	0.3528	1	15	24/52	25.28816	4364	1849.06	MS2	617.024	148	Glioma_grade 2_6.raw
341916196			vLSLAQEQVGGSPEk	Unambiguous	N-Term(iTRAQ4plex); K15(iTRAQ4plex)	HCD	3.2858057	0	1	6	2.95008	3.485	3.1172	3.6824136	10.11536774	26969.87	22830.096	79563.15	84069.85	11.75608139	3.894957778	2	High	3658	Unique	Used	51220	1	38.605	1	6.5875	2.6006	1	43	23/28	0	3658	1830.03	MS2	915.517	190	Glioma_grade 2_2.raw
341916196			lTVAAPPSGGPGFLSIERPDSRPPR	Unambiguous	N-Term(iTRAQ4plex)	HCD	2.5222368	0	1	6	2.26394	2.1749	2.8837	2.7703757	14.09700819	15621.737	16260.956	35366.75	45048.957	2.835369582	17.02740542	4	High	4256	Unique	Used	91657	1	47.927	1	5.1153	1.1496	1	8	25/144	32.92192	4256	2718.48	MS2	680.376	61	Glioma_grade 2_7rep.raw
341916196			eELVYELNPLDHR	Unambiguous	N-Term(iTRAQ4plex)	HCD	1.9496976	0	1	6	2.03075	2.0085	1.8903	1.8695369	4.186110798	14829.234	14993.555	30114.416	28031.004	0.779219498	5.067277021	3	High	6009	Unique	Used	53434	1	55.782	1	5.6308	-2.9604	1	16	22/48	2.384164	6009	1770.91	MS2	590.975	189	Glioma_grade 2_2.raw
341916196			lTVAAPPSGGPGFLSIERPDSRPPR	Unambiguous	N-Term(iTRAQ4plex)	HCD	2.556649	0	1	6	2.40132	2.4635	2.6475	2.7160008	5.820135504	13711.857	13365.834	32926.535	36301.617	1.807208818	6.894719273	3	High	4272	Unique	Used	91669	1	48.091	1	3.781	1.2444	1	35	15/96	28.65387	4272	2718.48	MS2	906.833	84	Glioma_grade 2_7rep.raw
341916196			yVSHFETEGPHVLLYFDSVPTSR	Unambiguous	N-Term(iTRAQ4plex)	HCD	2.083123	0	1	6	2.18254	2.0589	2.1088	1.9893409	3.908025979	9542.852	10116.101	20827.64	20124.373	4.123803086	2.428622323	3	High	5627	Unique	Used	82604	1	57.628	1	5.9052	0.4144	1	48	29/88	26.05556	5627	2824.41	MS2	942.141	74	Glioma_grade 2_6.raw
341916196			lLLFSPSVVHLGVPLSVGVQLQDVPR	Unambiguous	N-Term(iTRAQ4plex)	HCD	4.150492	0	1	6	3.28142	4.4106	3.957	5.3186245	20.14875868	6878.86	5117.7925	22572.396	27219.615	20.76017075	13.19922615	3	High	7813	Unique	Used	77843	1	75.561	1	7.2586	-2.576	1	25	34/100	14.02606	7813	2913.7	MS2	971.904	17	Glioma_grade 2_5.raw
341916196			hLVPGAPFLLQALVR	Unambiguous	N-Term(iTRAQ4plex)	HCD	2.2556865	0	1	6	2.19148	2.2035	2.3076	2.32025	2.996098058	4267.7026	4244.3574	9352.567	9847.971	0.387862614	3.648892837	3	High	6207	Unique	Used	83070	1	63.235	1	5.4367	2.4918	1	97	26/56	31.1366	6207	1775.09	MS2	592.367	69	Glioma_grade 2_6.raw
341916196			hLVPGAPFLLQALVR	Unambiguous	N-Term(iTRAQ4plex)	HCD	3.0573318	0	1	6	2.84487	2.5285	3.6523	3.2461655	15.90058692	3765.2969	4236.3506	10711.796	13751.896	8.325417124	17.57441204	3	High	6334	Unique	Used	83175	1	64.415	1	5.5052	-1.7375	1	68	28/56	19.55577	6334	1775.08	MS2	592.364	77	Glioma_grade 2_6.raw
341916196			iTPGkPYILTVPGHLDEMQLDIQAR	Unambiguous	N-Term(iTRAQ4plex); K5(iTRAQ4plex)	HCD	3.2877734	0	1	6	2.97734	3.2523	3.3202	3.6268797	8.098438717	3524.8572	3226.8474	10494.687	11703.388	6.242119966	7.700493611	4	High	5327	Unique	Used	92466	1	58.919	1	6.2513	-0.8509	1	30	32/144	24.72472	5327	3093.7	MS2	774.18	56	Glioma_grade 2_7rep.raw
341916196			lHLETDSLALVALGALDTALYAAGSk	Unambiguous	N-Term(iTRAQ4plex); K26(iTRAQ4plex)	HCD	1.6176262	0	1	6	1.51355	1.7613	1.4942	1.7387371	8.766203086	1257.0806	1080.2766	1902.6531	1878.3171	10.69749265	0.910250529	4	High	7606	Unique	Used	84167	1	77.3	1	7.5168	-1.1224	1	69	27/150	22.35492	7606	2901.61	MS2	726.159	81	Glioma_grade 2_6.raw
344313196	NP_001230702 .1	GNG2	guanine nucleotide-binding protein G(I)/G(S)/G(O) subunit gamma-2 precursor [Homo sapiens]	2	23	0.394868284	25.78748964	154.58327	42.25	9.95E-05	71	7.845	7.9868	G protein	Cell communication,Signal transduction	GTPase activity	-	-																								
344313196			aAADLMAYcEAHAk	Unambiguous	N-Term(iTRAQ4plex); C9(Methylthio); K14(iTRAQ4plex)	HCD	0.2974006	0	1	1	0.2645	0.2759	0.318	0.33172005	0.108821005	757148	725800.25	200267.2	240762.5	2.989478102	12.98529384	2	High	5963	Unique	Used	76284	1	58.689	1	6.1127	0.0192	1	8	23/26	9.762761	5963	1798.85	MS2	899.93	78	Glioma_grade 2_5.raw

344313196			aAADLMAYcEAHAk	Unambiguous	N-Term(iTRAQ4plex); C9(Methylthio); K14(iTRAQ4plex)	HCD	0.3731638	0	1	1	0.37305	0.36	0.3868	0.3732784	0.029379589	121241.305	125643.96	45228.465	46900.176	2.521938444	2.566147012	3	High	5624	Unique	Used	82601	1	57.61	1	6.6721	-2.3973	1	15	32/52	9.745476	5624	1798.85	MS2	600.288	72	Glioma_grade 2_6.raw
344313196			aAADLmAYcEAHAk	Unambiguous	N-Term(iTRAQ4plex); M6(Oxidation); C9(Methylthio); K14(iTRAQ4plex)	HCD	0.66112006	0	1	1	0.65735	0.608	0.7185	0.66460603	0.068301325	121187.03	131017.68	79662.445	87075.14	5.512442078	6.287205013	3	High	4408	Unique	Used	74901	1	45.887	1	6.9962	0.1903	1	9	26/52	32.29718	4408	1814.85	MS2	605.621	59	Glioma_grade 2_5.raw
344313196			aAADLMAYcEAHAk	Unambiguous	N-Term(iTRAQ4plex); C9(Methylthio); K14(iTRAQ4plex)	HCD	0.2569134	0	1	1	0.24483	0.2978	0.2233	0.27161652	0.124811932	112076.12	92127.94	27439.287	25023.47	13.81509589	6.512202867	3	High	4648	Unique	Used	87140	1	61.679	1	6.5792	1.9797	1	20	32/52	24.91846	4648	1798.86	MS2	600.29	83	Glioma_grade 2_1rep.raw
344313196			aAADLMAYcEAHAk	Unambiguous	N-Term(iTRAQ4plex); C9(Methylthio); K14(iTRAQ4plex)	HCD	0.4399992	0	1	1	0.4695	0.3924	0.4969	0.4153406	0.108397346	104373.375	124878.45	49003.523	51867.094	12.64921453	4.014747868	2	High	5636	Unique	Used	82612	1	57.703	1	6.0171	1.6479	1	71	21/26	27.48677	5636	1798.86	MS2	899.932	87	Glioma_grade 2_6.raw
344313196			aAADLMAYcEAHAk	Unambiguous	N-Term(iTRAQ4plex); C9(Methylthio); K14(iTRAQ4plex)	HCD	0.31347588	0	1	1	0.30961	0.3501	0.2811	0.31784928	0.090323143	102767.445	90872.25	31817.697	28883.678	8.687447115	6.835643282	3	High	6050	Unique	Used	76362	1	59.39	1	6.6944	-2.2955	1	12	36/52	19.82901	6050	1798.85	MS2	600.288	74	Glioma_grade 2_5.raw
344313196			eDPLLTPVPASENPFR	Unambiguous	N-Term(iTRAQ4plex)	HCD	0.4730221	0	1	1	0.47389	0.4909	0.4558	0.47211885	0.030355679	88323.625	85264.016	41856.043	40254.75	2.49265473	2.757944717	2	High	4352	Unique	Used	86932	1	58.312	1	4.064	-1.4128	1	51	18/30	12.74789	4352	1926.01	MS2	963.507	240	Glioma_grade 2_1rep.raw
344313196			aAADLmAYcEAHAk	Unambiguous	N-Term(iTRAQ4plex); M6(Oxidation); C9(Methylthio); K14(iTRAQ4plex)	HCD	0.46012768	0	1	1	0.43846	0.4654	0.4552	0.48312417	0.040626457	75932.52	71536.17	33293.574	34560.85	4.216066336	2.641241058	2	High	4415	Unique	Used	74908	1	45.926	1	4.9346	0.8031	1	71	18/26	0	4415	1814.85	MS2	907.928	79	Glioma_grade 2_5.raw
344313196			aAADLMAYcEAHAk	Unambiguous	N-Term(iTRAQ4plex); C9(Methylthio); K14(iTRAQ4plex)	HCD	0.39486828	0	1	1	0.37105	0.4399	0.3569	0.42310488	0.10060785	65732.37	55443.43	24390.094	23458.387	12.00797394	2.753760721	2	High	6057	Unique	Used	76369	1	59.432	1	5.8266	-3.4417	1	66	24/26	0	6057	1798.85	MS2	899.927	83	Glioma_grade 2_5.raw
344313196			aAADLMAYcEAHAk	Unambiguous	N-Term(iTRAQ4plex); C9(Methylthio); K14(iTRAQ4plex)	HCD	0.33974767	0	1	1	0.3046	0.2883	0.3941	0.37301958	0.151347957	64204.28	67833.414	19556.307	25303.191	3.887049499	18.11728097	3	High	6132	Unique	Used	76434	1	60.078	1	6.7595	-1.9901	1	20	34/52	20.79241	6132	1798.85	MS2	600.288	78	Glioma_grade 2_5.raw
344313196			aAADLMAYcEAHAk	Unambiguous	N-Term(iTRAQ4plex); C9(Methylthio); K14(iTRAQ4plex)	HCD	0.3749286	0	1	1	0.36182	0.4046	0.3484	0.38958764	0.067981016	58682.72	52483.52	21232.469	20446.932	7.886380538	2.665386384	2	High	6148	Unique	Used	76448	1	60.216	1	5.4595	1.7836	1	100	23/26	36.59109	6148	1798.86	MS2	899.932	125	Glioma_grade 2_5.raw
344313196			aAADLMAYcEAHAk	Unambiguous	N-Term(iTRAQ4plex); C9(Methylthio); K14(iTRAQ4plex)	HCD	0.41837904	0	1	1	0.3835	0.3701	0.4684	0.4520344	0.116929595	42696.758	44244.12	16374.377	19999.865	2.516998191	14.09572823	3	High	5738	Unique	Used	82697	1	58.644	1	6.3014	-2.8045	1	34	31/52	14.15944	5738	1798.85	MS2	600.288	62	Glioma_grade 2_6.raw
344313196			aAADLMAYcEAHAk	Unambiguous	N-Term(iTRAQ4plex); C9(Methylthio); K14(iTRAQ4plex)	HCD	0.25421438	0	1	1	0.21337	0.2461	0.2612	0.30133113	0.142845944	28234.162	24477.02	6024.252	7375.688	10.08021632	14.26289312	3	High	4650	Unique	Used	96446	1	63.877	1	6.5954	0.6564	1	100	32/52	36.87027	4650	1798.85	MS2	600.29	82	Glioma_grade 2_8rep.raw
344313196			aAADLMAYcEAHAk	Unambiguous	N-Term(iTRAQ4plex); C9(Methylthio); K14(iTRAQ4plex)	HCD	0.41477358	0	1	1	0.3995	0.4795	0.3608	0.43311316	0.120550686	27979.441	23309.404	11177.648	10095.609	12.87693194	7.193229644	3	High	6305	Unique	Used	76584	1	61.579	1	6.4013	1.8779	1	43	30/52	36.06274	6305	1798.86	MS2	600.29	67	Glioma_grade 2_5.raw
344313196			aAADLmAYcEAHAk	Unambiguous	N-Term(iTRAQ4plex); M6(Oxidation); C9(Methylthio); K14(iTRAQ4plex)	HCD	0.37682295	0	1	1	0.35703	0.3927	0.3624	0.39859262	0.055602289	24317.416	22108.846	8682.058	8812.423	6.727635422	1.053840643	3	High	3946	Unique	Used	96044	1	54.43	1	6.5867	0.5939	1	100	24/52	54.66521	3946	1814.85	MS2	605.621	71	Glioma_grade 2_8rep.raw
344313196			aAADLMAYcEAHAk	Unambiguous	N-Term(iTRAQ4plex); C9(Methylthio); K14(iTRAQ4plex)	HCD	0.37548947	0	1	1	0.3471	0.3712	0.3795	0.40584755	0.06445719	17573.373	16432.717	6099.75	6669.178	4.743653814	6.306659419	3	High	6437	Unique	Used	76702	1	62.672	1	6.3993	-0.8704	1	78	34/52	18.62706	6437	1798.85	MS2	600.289	84	Glioma_grade 2_5.raw
344313196			aAADLMAYcEAHAk	Unambiguous	N-Term(iTRAQ4plex); C9(Methylthio); K14(iTRAQ4plex)	HCD	0.4020195	0	1	1	0.32428	0.3388	0.4626	0.48322603	0.204544934	8495.613	8132.499	2754.9902	3929.8352	3.088268491	24.85452698	3	High	6851	Unique	Used	77057	1	66.35	1	6.0833	-5.96	1	100	32/52	31.8885	6851	1798.84	MS2	600.286	51	Glioma_grade 2_5.raw
344313196			aAADLMAYcEAHAk	Unambiguous	N-Term(iTRAQ4plex); C9(Methylthio); K14(iTRAQ4plex)	HCD	0.5321285	0	1	1	0.55604	0.5641	0.5006	0.50787306	0.061162771	6431.5864	6339.3823	3576.1956	3219.6013	1.021036789	7.420770555	3	High	7357	Unique	Used	77480	1	71.049	1	5.9694	0.6564	1	100	34/52	16.17763	7357	1798.85	MS2	600.29	83	Glioma_grade 2_5.raw
344313196			aAADLMAYcEAHAk	Unambiguous	N-Term(iTRAQ4plex); C9(Methylthio); K14(iTRAQ4plex)	HCD	0.5160569	0	1	1	0.52538	0.5652	0.4704	0.5060314	0.076521324	5977.5283	5556.15	3140.4478	2811.5862	5.166772397	7.813808437	3	High	6973	Unique	Used	77158	1	67.496	1	6.154	-5.7564	1	100	34/52	15.8038	6973	1798.84	MS2	600.286	72	Glioma_grade 2_5.raw
344313196			aAADLMAYcEAHAk	Unambiguous	N-Term(iTRAQ4plex); C9(Methylthio); K14(iTRAQ4plex)	HCD	0.2867578	0	1	1	0.29998	0.3697	0.2195	0.27046037	0.216432046	5965.825	4840.7617	1789.6389	1309.2341	14.72324076	21.92393762	4	High	5995	Unique	Used	76313	1	58.941	1	3.9305	4.6187	1	61	24/78	26.49651	5995	1798.86	MS2	450.471	139	Glioma_grade 2_5.raw
344313196			aAADLMAYcEAHAk	Unambiguous	N-Term(iTRAQ4plex); C9(Methylthio); K14(iTRAQ4plex)	HCD	0.51161426	0	1	1	0.59572	0.4857	0.5434	0.44303724	0.129007256	5117.8965	6277.136	3048.856	2781.0051	14.38707808	6.497554041	3	High	7178	Unique	Used	77334	1	69.326	1	6.1565	2.8958	1	100	32/52	75.29665	7178	1798.86	MS2	600.291	102	Glioma_grade 2_5.raw
344313196			aAADLMAYcEAHAk	Unambiguous	N-Term(iTRAQ4plex); C9(Methylthio); K14(iTRAQ4plex)	HCD	0.2696507	0	1	1	0.20604	0.2645	0.2737	0.35129768	0.217903007	5091.5327	3966.7397	1049.0631	1393.5065	17.56071627	19.94279007	4	High	5973	Unique	Used	76294	1	58.751	1	4.8461	-5.696	1	73	23/78	0	5973	1798.84	MS2	450.466	89	Glioma_grade 2_5.raw
344313196			aAADLMAYcEAHAk	Unambiguous	N-Term(iTRAQ4plex); C9(Methylthio); K14(iTRAQ4plex)	HCD	0.5427956	0	1	1	0.49983	0.5102	0.5747	0.5866536	0.08134193	4479.6133	4388.42	2239.0474	2574.4822	1.454288649	9.85506443	3	High	7964	Unique	Used	77979	1	76.767	1	5.5786	0.351	1	100	32/52	15.56311	7964	1798.85	MS2	600.289	85	Glioma_grade 2_5.raw
344313201	NP_001230704 .1	LIMA1	LIM domain and actin-binding protein 1 isoform 4 [Homo sapiens]	4	4	2.405107975	24.57923188	23.527646	14.22	3.64E-04	457	51.187	5.5669	Cytoskeletal protein	Cell growth and/or maintenance	Structural constituent of cytoskeleton	Cytoplasm	Cytoskeleton, Nucleus																								
344313201			vGVLAASMEAk	Unambiguous	N-Term(iTRAQ4plex); K11(iTRAQ4plex)	HCD	1.7066418	0	1	4	1.67415	1.7433	1.6714	1.74048	2.339512613	69769.97	67001.15	116805.16	116614.15	2.862960248	0.1157269	2	High	3262	Unique	Used	58884	1	35.828	1	4.4384	1.4372	1	60	18/20	28.78906	3262	1363.79	MS2	682.398	211	Glioma_grade 2_3.raw
344313201			lSLGTYASLHGR	Unambiguous	N-Term(iTRAQ4plex)	HCD	2.151668	0	1	4	1.83914	2.5748	1.8494	2.5892198	19.24869484	16380.038	11699.836	30125.121	30293.445	23.57134916	0.393994925	3	High	2627	Unique	Used	73295	1	31.6	1	4.464	11.896	1	32	24/44	29.85997	2627	1418.8	MS2	473.606	236	Glioma_grade 2_5.raw
344313201			lLANQQVFHIScFR	Unambiguous	N-Term(iTRAQ4plex); C12(Methylthio)	HCD	3.0547755	0	1	4	2.79467	2.8572	3.248	3.3207083	8.75720592	4598.411	4497.733	12851.019	14935.659	1.565280772	10.6098547	3	High	6108	Unique	Used	76413	1	59.877	1	5.7198	0.7742	1	88	24/52	56.0562	6108	1865.97	MS2	622.66	193	Glioma_grade 2_5.raw
344313201			gNYDEGFGHRPHk	Unambiguous	N-Term(iTRAQ4plex); K13(iTRAQ4plex)	HCD	2.658548	0	1	4	2.7204	2.7494	2.5687	2.5960402	3.368328054	4456.799	4409.827	12124.264	11448.088	0.749196362	4.056690099	4	High	987	Unique	Used	94062	1	18.623	1	5.3155	-2.3932	1	100	21/72	54.31597	987	1801.89	MS2	451.229	147	Glioma_grade 2_8rep.raw
344925834	NP_001230710 .1	FAM213A	redox-regulatory protein FAM213A isoform 2 precursor [Homo sapiens]	4	16	2.01052475	18.65358178	95.890688	22.48	1.80E-04	218	24.447	9.0122	-	-	-	-	-																								
344925834			vNLLSVLEAAk	Unambiguous	N-Term(iTRAQ4plex); K11(iTRAQ4plex)	HCD	1.762423	0	1	3	1.59308	1.8698	1.6709	1.961186	9.632849153	240985.28	205316.95	383908.6	402664.75	11.30234909	3.3722477	2	High	7336	Unique	Used	62654	1	67.838	1	4.6298	0.6519	1	13	19/20	26.48256	7336	1444.9	MS2	722.953	110	Glioma_grade 2_3.raw
344925834			vNLLSVLEAAk	Unambiguous	N-Term(iTRAQ4plex); K11(iTRAQ4plex)	HCD	1.5072204	0	1	3	1.50196	1.5805	1.4376	1.5127559	3.881763219	162512.12	154433.31	244086.73	233619.89	3.604772806	3.098627162	2	High	7261	Unique	Used	62592	1	67.122	1	4.4344	-2.8119	1	22	18/20	25.62305	7261	1444.89	MS2	722.951	125	Glioma_grade 2_3.raw
344925834			vNLLSVLEAAk	Unambiguous	N-Term(iTRAQ4plex); K11(iTRAQ4plex)	HCD	1.8479341	0	1	3	1.71551	1.8342	1.8608	1.9895222	6.077397134	118838.73	111150.625	203868.53	221136.64	4.727445912	5.745999598	2	High	7618	Unique	Used	54925	1	68.061	1	4.7465	-0.4464	1	39	18/20	33.68815	7618	1444.9	MS2	722.952	112	Glioma_grade 2_2.raw
344925834			aALEYLEDIDLk	Unambiguous	N-Term(iTRAQ4plex); K12(iTRAQ4plex)	HCD	2.6567388	0	1	3	2.61797	2.6792	2.6348	2.6964104	1.384332873	91675.29	89581.55	240003.45	241548.62	1.633591044	0.45378278	2	High	7105	Unique	Used	54462	1	63.877	1	5.6929	2.3119	1	44	20/22	16.60581	7105	1680.93	MS2	840.971	248	Glioma_grade 2_2.raw
344925834			mIkPQTLASEk	Unambiguous	N-Term(iTRAQ4plex); M1(Oxidation); K3(iTRAQ4plex); K11(iTRAQ4plex)	HCD	2.371465	0	1	3	2.25216	2.2788	2.463	2.49218	5.208256153	79684.445	78752.016	179462.3	196264.22	0.832291841	6.324148516	2	High	1222	Unique	Used	89136	1	20.407	1	3.0219	-1.6435	1	36	13/20	8.341477	1222	1693.99	MS2	847.496	149	Glioma_grade 2_7rep.raw
344925834			vNLLSVLEAAk	Unambiguous	N-Term(iTRAQ4plex); K11(iTRAQ4plex)	HCD	1.6942959	0	1	3	1.56668	1.78	1.6189	1.8392899	7.600014878	59943.617	52760.2	93912.31	97041.305	9.013790319	2.317351869	2	High	7720	Unique	Used	55017	1	68.892	1	4.4328	-0.1084	1	50	19/20	38.87272	7720	1444.9	MS2	722.952	105	Glioma_grade 2_2.raw
344925834			sMLDQLGVPLYAVVk	Unambiguous	N-Term(iTRAQ4plex); K15(iTRAQ4plex)	HCD	1.9822594	0	1	3	1.91111	1.8568	2.1114	2.051384	5.985155239	57335.03	59011.617	109573.75	121055.49	2.037920417	7.040578388	2	High	7718	Unique	Used	55015	1	68.883	1	5.0698	0.2312	1	22	20/28	16.76992	7718	1921.11	MS2	961.057	145	Glioma_grade 2_2.raw
344925834			vNLLSVLEAAk	Unambiguous	N-Term(iTRAQ4plex); K11(iTRAQ4plex)	HCD	2.5029962	0	1	3	2.44761	2.4598	2.546	2.558656	2.29332323	38917.16	38724.88	95254.086	99083.64	0.350229056	2.786801779	2	High	7455	Unique	Used	70398	1	68.719	1	4.8465	0.2295	1	52	19/20	19.03626	7455	1444.9	MS2	722.953	110	Glioma_grade 2_4.raw
344925834			smLDQLGVPLYAVVk	Unambiguous	N-Term(iTRAQ4plex); M2(Oxidation); K15(iTRAQ4plex)	HCD	1.8036634	0	1	3	1.58383	1.8016	1.8055	2.0537248	10.60327364	33111.54	29109.021	52443.03	59781.918	9.097341076	9.248170863	2	High	6592	Unique	Used	53985	1	59.985	1	4.7085	-3.4473	1	79	19/28	29.23383	6592	1937.1	MS2	969.051	186	Glioma_grade 2_2.raw
344925834			aALEYLEDIDLk	Unambiguous	N-Term(iTRAQ4plex); K12(iTRAQ4plex)	HCD	2.0442772	0	1	3	1.94468	1.9937	2.0936	2.1463878	4.494106512	31017.645	30254.344	60319.375	64937.56	1.761768541	5.214162282	2	High	5024	Unique	Used	87424	1	65.68	1	5.396	0.4964	1	100	19/22	54.06964	5024	1680.93	MS2	840.969	299	Glioma_grade 2_1rep.raw
344925834			vNLLSVLEAAk	Unambiguous	N-Term(iTRAQ4plex); K11(iTRAQ4plex)	HCD	1.6442034	0	1	3	1.70304	1.5708	1.7238	1.5899335	4.715861077	23520.951	25500.912	40057.16	40544.754	5.711916128	0.855515724	2	High	7368	Unique	Used	70321	1	67.973	1	4.08	0.9898	1	100	18/20	50.75248	7368	1444.9	MS2	722.953	117	Glioma_grade 2_4.raw
344925834			vNLLSVLEAAk	Unambiguous	N-Term(iTRAQ4plex); K11(iTRAQ4plex)	HCD	2.0387902	0	1	3	1.58047	2.2359	1.8994	2.6871808	22.55253684	5751.2935	4065.3303	9089.759	10924.277	24.28851377	12.96290381	3	High	7629	Unique	Used	54935	1	68.146	1	3.765	1.3185	1	34	23/40	10.31941	7629	1444.9	MS2	482.305	105	Glioma_grade 2_2.raw
344925834			vNLLSVLEAAk	Unambiguous	N-Term(iTRAQ4plex); K11(iTRAQ4plex)	HCD	2.830427	0	1	3	2.92138	2.545	3.158	2.7511892	9.13727973	5184.8047	5951.4795	15146.793	16373.646	9.736119163	5.504464808	3	High	7351	Unique	Used	62667	1	67.969	1	4.2066	1.762	1	35	23/40	6.070358	7351	1444.9	MS2	482.305	110	Glioma_grade 2_3.raw
344925834			vNLLSVLEAAk	Unambiguous	N-Term(iTRAQ4plex); K11(iTRAQ4plex)	HCD	1.9507635	0	1	3	1.80943	2.5343	1.5341	2.148722	21.5451047	4035.5781	2881.3223	7302.0757	6191.1606	23.59964887	11.64339797	3	High	7714	Unique	Used	55012	1	68.836	1	3.7441	-3.307	1	52	23/40	5.159017	7714	1444.89	MS2	482.303	111	Glioma_grade 2_2.raw
344925834			vNLLSVLEAAk	Unambiguous	N-Term(iTRAQ4plex); K11(iTRAQ4plex)	HCD	2.1263897	0	1	3	2.28958	1.7885	2.5589	1.9989134	15.58934433	3433.4546	4395.312	7861.1606	8785.849	17.37530124	7.85550623	3	High	5422	Unique	Used	87718	1	70.062	1	4.401	0.4314	1	100	22/40	8.558901	5422	1444.9	MS2	482.304	106	Glioma_grade 2_1rep.raw
344925834			vNLLSVLEAAk	Unambiguous	N-Term(iTRAQ4plex); K11(iTRAQ4plex)	HCD	2.4865365	0	1	3	2.74433	2.2327	2.7985	2.2768059	11.92408232	3293.6465	4048.3816	9038.842	9217.379	14.53762639	1.383032374	3	High	7374	Unique	Used	70327	1	68.016	1	4.3793	-0.5824	1	100	23/40	15.13797	7374	1444.9	MS2	482.304	108	Glioma_grade 2_4.raw
344925852	NP_001230713 .1	PDE2A	cGMP-dependent 3',5'-cyclic phosphodiesterase isoform 4 [Homo sapiens]	7	11	0.313111991	39.15893887	79.593019	15.54	1.39E-04	920	103.41	5.3892	Enzyme: Phosphodie sterase	Metabolism,Energy pathways	Phosphoric diester hydrolase activity	Cytoplasm	Nucleus																								
344925852			eFFSQGDLEk	Unambiguous	N-Term(iTRAQ4plex); K10(iTRAQ4plex)	HCD	0.33899072	0	1	4	0.32318	0.3497	0.3291	0.35609454	0.046665717	434435.44	401518.94	140402.06	142978.69	5.568600613	1.285868956	2	High	3766	Unique	Used	51322	1	39.383	1	4.2605	3.6473	1	28	18/18	12.06623	3766	1487.77	MS2	744.387	177	Glioma_grade 2_2.raw
344925852			lLQDLFPk	Unambiguous	N-Term(iTRAQ4plex); K8(iTRAQ4plex)	HCD	0.22545399	0	1	4	0.19766	0.2296	0.2219	0.25774068	0.109196571	292403.62	251690.48	57797.31	64870.875	10.58219061	8.15495196	2	High	6514	Unique	Used	69555	1	60.785	1	3.6995	-6.6285	1	40	13/14	52.77181	6514	1261.77	MS2	631.387	248	Glioma_grade 2_4.raw
344925852			lLQDLFPk	Unambiguous	N-Term(iTRAQ4plex); K8(iTRAQ4plex)	HCD	0.24384192	0	1	4	0.23008	0.2561	0.2328	0.25916046	0.062144002	230165.34	206794.81	52956.164	53593.035	7.563829444	0.845310536	2	High	6485	Unique	Used	69528	1	60.559	1	3.6118	7.1092	1	59	13/14	43.36739	6485	1261.78	MS2	631.396	165	Glioma_grade 2_4.raw
344925852			lLQDLFPk	Unambiguous	N-Term(iTRAQ4plex); K8(iTRAQ4plex)	HCD	0.2267977	0	1	4	0.21218	0.2282	0.2255	0.24251826	0.05476587	143708.92	133628.19	30492.145	32407.275	5.140424621	4.305926541	2	High	6427	Unique	Used	61824	1	60.467	1	3.7965	-5.2741	1	71	13/14	45.76683	6427	1261.77	MS2	631.388	241	Glioma_grade 2_3.raw
344925852			nILcFPIk	Unambiguous	N-Term(iTRAQ4plex); C4(Methylthio); K8(iTRAQ4plex)	HCD	0.30991432	0	1	4	0.28049	0.3141	0.3062	0.34287062	0.08262603	133919.08	119585.25	37562.35	41002.266	7.9963513	6.1920698	2	High	6471	Unique	Used	61865	1	60.816	1	3.6386	-4.9744	1	77	14-Dec	39.90421	6471	1281.72	MS2	641.366	278	Glioma_grade 2_3.raw
344925852			nILcFPIk	Unambiguous	N-Term(iTRAQ4plex); C4(Methylthio); K8(iTRAQ4plex)	HCD	0.313112	0	1	4	0.29786	0.317	0.3095	0.32934728	0.042184034	93506.31	87859.57	27851.52	28936.312	4.403086342	2.701507532	2	High	6460	Unique	Used	61855	1	60.726	1	3.5282	10.74	1	71	14-Dec	62.60213	6460	1281.74	MS2	641.376	256	Glioma_grade 2_3.raw

344925852			lLQDLFPk	Unambiguous	N-Term(iTRAQ4plex); K8(iTRAQ4plex)	HCD	0.29741654	0	1	4	0.2621	0.2741	0.3197	0.33435163	0.117287748	87324.72	83506.484	22887.496	27920.53	3.1608986	14.00917434	2	High	6429	Unique	Used	69478	1	60.079	1	3.7392	-4.9838	1	100	13/14	39.87337	6429	1261.77	MS2	631.388	265	Glioma_grade 2_4.raw
344925852			aYIPELQISFMEHIAMPIYk	Unambiguous	N-Term(iTRAQ4plex); K20(iTRAQ4plex)	HCD	0.31681922	0	1	4	0.32703	0.3144	0.3193	0.30699974	0.026574409	41159	42812.125	13460.3545	13143.312	2.784137756	1.685353421	3	High	7417	Unique	Used	84001	1	75.747	1	6.6118	2.4053	1	42	30/76	30.04806	7417	2682.43	MS2	894.816	123	Glioma_grade 2_6.raw
344925852			dIILATDLAHHLR	Unambiguous	N-Term(iTRAQ4plex)	HCD	0.6049579	0	1	4	0.58014	0.6417	0.5717	0.63240355	0.058775893	24905.248	22516.385	14448.65	14239.442	7.124095565	1.03131568	3	High	3828	Unique	Used	81077	1	41.569	1	5.8689	-0.2511	1	52	31/48	40.6604	3828	1631.93	MS2	544.65	217	Glioma_grade 2_6.raw
344925852			vETVYTYLLDGESQLVcEDPPHELPQEGk	Unambiguous	N-Term(iTRAQ4plex); C17(Methylthio); K29(iTRAQ4plex)	HCD	0.32474366	0	1	4	0.23573	0.3262	0.3237	0.44791964	0.261498393	4548.6353	3287.1726	1072.2396	1472.3892	22.76699074	22.23888181	3	High	7985	Unique	Used	63236	1	73.363	1	6.8722	1.4481	1	70	32/112	24.09717	7985	3622.76	MS2	1208.26	56	Glioma_grade 2_3.raw
344925852			lLHDGIQPVAAIDSNFASFTYTPR	Unambiguous	N-Term(iTRAQ4plex)	HCD	0.6149089	0	1	4	0.54795	0.7027	0.5465	0.7007707	0.142846196	3535.982	2757.4746	1937.5449	1932.3573	17.49397499	0.189575186	3	High	7093	Unique	Used	70075	1	65.649	1	6.6662	0.6035	1	24	31/92	8.070801	7093	2777.44	MS2	926.485	116	Glioma_grade 2_4.raw
346644849	NP_001231178.1	APEX1	DNA-(apurinic or apyrimidinic site) lyase [Homo sapiens]	2	2	2.753936291	1.599431954	11.65697	12.89	1.49E-04	318	35.532	8.1187	DNA repair protein	Regulation of nucleobase, nucleoside, nucleotide and nucleic acid metabolism	DNA repair protein,Transcription regulator activity	Cytoplasm	Mitochondrion, Nucleolus, Nucleus, Endoplasmic reticulum																								
346644849			kPLVLcGDLNVAHEEIDLR	Unambiguous	N-Term(iTRAQ4plex); K1(iTRAQ4plex); C6(Methylthio)	HCD	2.7227902	0	1	1	2.8821	3.2272	2.2723	2.5444033	15.15679063	11711.569	10459.289	33753.95	26612.648	7.987924328	16.72999055	4	High	5211	Unique	Used	92379	1	57.716	1	5.2109	-0.0392	1	11	37/108	14.14193	5211	2468.32	MS2	617.837	118	Glioma_grade 2_7rep.raw
346644849			lPAELQELPGLSHQYWSAPSDk	Unambiguous	N-Term(iTRAQ4plex); K22(iTRAQ4plex)	HCD	2.7850826	0	1	1	2.78682	2.7408	2.8302	2.7833748	1.311258251	10836.886	11019.008	30200.44	30670.03	1.178443684	1.09100611	3	High	6351	Unique	Used	76626	1	61.941	1	6.446	-2.6849	1	32	28/84	18.53738	6351	2754.43	MS2	918.814	122	Glioma_grade 2_5.raw
351721324	NP_001234930.1	SET	protein SET isoform 4 [Homo sapiens]	2	3	1.996933818	7.473712939	23.971485	19.92	1.91E-04	266	31.105	4.2339	MHC complex protein	Regulation of nucleobase, nucleoside, nucleotide and nucleic acid metabolism	MHC class I receptor activity,MHC class II receptor activity	Nucleus	Cytosol, Cytoplasm, Plasma membrane, Endoplasmic reticulum																								
351721324			lNEQASEEILk	Unambiguous	N-Term(iTRAQ4plex); K11(iTRAQ4plex)	HCD	1.8459791	0	1	4	1.82295	1.8399	1.852	1.8692216	1.058179319	141790.94	140487.8	258477.81	262602.8	0.65287179	1.119522909	2	High	2820	Unique	Used	50433	1	32.53	1	4.8666	-0.1601	1	33	19/20	25.28187	2820	1561.87	MS2	781.437	257	Glioma_grade 2_2.raw
351721324			iDFYFDENPYFENk	Unambiguous	N-Term(iTRAQ4plex); K14(iTRAQ4plex)	HCD	1.9969338	0	1	4	2.11526	1.88	2.1285	1.8917701	6.809879621	16224.779	18255.385	34319.613	34534.992	8.328587257	0.44236969	2	High	7018	Unique	Used	62375	1	65.067	1	5.2943	-3.7126	1	95	19/26	14.55086	7018	2129	MS2	1065.01	74	Glioma_grade 2_3.raw
351721324			iDFYFDENPYFENk	Unambiguous	N-Term(iTRAQ4plex); K14(iTRAQ4plex)	HCD	2.1442823	0	1	4	2.03258	2.1997	2.0931	2.2651665	4.863824896	5597.6396	5172.424	11377.656	11716.402	5.583492269	2.074382888	3	High	6997	Unique	Used	62355	1	64.914	1	3.4655	2.7601	1	67	16/52	45.80821	6997	2129.02	MS2	710.344	85	Glioma_grade 2_3.raw
354983504	NP_001238994.1	SYT7	synaptotagmin-7 isoform 1 [Homo sapiens]	3	5	0.305012405	37.58678683	43.603989	17.36	4.69E-04	478	52.788	9.3931	Calcium binding protein	Regulation of exocytosis	Calcium ion binding	Endosome, Chromaffin granule	Cytosol, Membrane fraction, Lysosome																								
354983504			vDLTQMQTFWk	Unambiguous	N-Term(iTRAQ4plex); K11(iTRAQ4plex)	HCD	0.5126242	0	1	2	0.47813	0.5114	0.5138	0.5495228	0.05684557	105475.086	98616.02	50430.277	54191.758	4.752869614	5.084528747	2	High	6660	Unique	Used	62042	1	62.267	1	5.5742	0.655	1	96	18/20	26.0656	6660	1684.9	MS2	842.953	225	Glioma_grade 2_3.raw
354983504			aVNTAPVPGQTPHDESDR	Unambiguous	N-Term(iTRAQ4plex)	HCD	0.3050124	0	1	2	0.29191	0.3841	0.2449	0.32225966	0.187644104	29326.873	22287.541	8560.661	7182.3755	19.28747808	12.38128392	3	High	1151	Unique	Used	48858	1	20.815	1	5.6068	-0.8724	1	19	30/68	6.693626	1151	2034.99	MS2	679.003	104	Glioma_grade 2_2.raw
354983504			aVNTAPVPGQTPHDESDR	Unambiguous	N-Term(iTRAQ4plex)	HCD	0.29876477	0	1	2	0.2645	0.2345	0.3712	0.32914445	0.206139371	24456.703	27580.715	6468.907	9078.039	8.490083308	23.7337279	3	High	1188	Unique	Used	56946	1	20.479	1	5.9744	-0.6925	1	18	29/68	13.37826	1188	2034.99	MS2	679.003	107	Glioma_grade 2_3.raw
354983504			nLNPIFNESFAFDIPTEk	Unambiguous	N-Term(iTRAQ4plex); K18(iTRAQ4plex)	HCD	0.5766106	0	1	2	0.46662	0.6193	0.5444	0.722605	0.185531375	11668.782	8791.446	5444.8423	6352.7427	19.88818304	10.88328721	3	High	7847	Unique	Used	55135	1	69.882	1	4.9614	1.3772	1	39	25/68	16.67002	7847	2384.24	MS2	795.419	142	Glioma_grade 2_2.raw
354983504			aVNTAPVPGQTPHDESDR	Unambiguous	N-Term(iTRAQ4plex)	HCD	0.2490689	0	1	2	0.21315	0.2278	0.269	0.28745413	0.139154972	11233.0205	10510.78	2394.3374	3021.3674	4.697441509	16.37375675	2	High	1168	Unique	Used	56928	1	20.307	1	4.7666	1.2937	1	100	21/34	0	1168	2035	MS2	1018	113	Glioma_grade 2_3.raw
358356396	NP_001240308.1	RPL15	60S ribosomal protein L15 isoform 1 [Homo sapiens]	3	7	2.436270714	23.98883233	44.389924	20.59	2.10E-04	204	24.131	11.62	Ribosomal subunit	Protein metabolism	Structural constituent of ribosome	Ribosome	Nucleolus																								
358356396			fFEVILIDPFHk	Unambiguous	N-Term(iTRAQ4plex); K12(iTRAQ4plex)	HCD	2.162885	0	1	1	2.05665	2.1172	2.2072	2.2722495	4.409299367	42091.688	40887.3	86567.86	92906.15	2.052642344	4.994425477	2	High	6506	Unique	Used	93255	1	72.602	1	4.7509	4.038	1	79	16/22	29.87314	6506	1793.03	MS2	897.019	166	Glioma_grade 2_7rep.raw
358356396			vLNSYWVGEDSTYk	Unambiguous	N-Term(iTRAQ4plex); K14(iTRAQ4plex)	HCD	2.8140633	0	1	1	2.59626	2.543	3.0908	3.0274007	10.11201145	30023.492	30651.908	77948.81	92795.61	1.464703043	12.29706126	2	High	5319	Unique	Used	52783	1	50.763	1	5.3366	-2.3532	1	34	20/26	12.11258	5319	1948.98	MS2	974.996	155	Glioma_grade 2_2.raw
358356396			gATYGkPVHHGVNQLk	Unambiguous	N-Term(iTRAQ4plex); K6(iTRAQ4plex); K16(iTRAQ4plex)	HCD	2.294602	0	1	2	2.16666	2.5467	2.0801	2.444991	9.606142742	28720.027	24433.758	62226.453	59740.32	11.40407922	2.882689211	5	High	935	Unique	Used	84594	1	18.461	1	3.2506	-5.7472	1	14	12/120	36.93624	935	2138.21	MS2	428.447	155	Glioma_grade 2_1rep.raw
358356396			vLNSYWVGEDSTYk	Unambiguous	N-Term(iTRAQ4plex); K14(iTRAQ4plex)	HCD	3.7153707	0	1	1	3.57576	3.5655	3.8657	3.8545754	4.501856776	26904.818	26982.383	96205.29	104005.63	0.203561278	5.509862608	2	High	5102	Unique	Used	60591	1	50.161	1	6.1095	-0.5995	1	70	21/26	0	5102	1948.99	MS2	974.998	173	Glioma_grade 2_3.raw
358356396			fFEVILIDPFHk	Unambiguous	N-Term(iTRAQ4plex); K12(iTRAQ4plex)	HCD	2.6482017	0	1	1	2.41378	2.8741	2.4585	2.9273233	10.10731103	19126.166	16063.101	46166.387	47021.887	12.31008326	1.298296074	3	High	6496	Unique	Used	93248	1	72.486	1	5.6265	0.0819	1	31	25/44	6.147471	6496	1793.02	MS2	598.346	173	Glioma_grade 2_7rep.raw
358356396			fFEVILIDPFHk	Unambiguous	N-Term(iTRAQ4plex); K12(iTRAQ4plex)	HCD	2.4362707	0	1	1	2.25441	2.5375	2.3464	2.6409707	7.202253823	11060.986	9827.065	24935.955	25952.992	8.354191653	2.826365259	3	High	7032	Unique	Used	83714	1	71.585	1	5.4206	0.5925	1	72	25/44	14.65327	7032	1793.03	MS2	598.347	177	Glioma_grade 2_6.raw
358356396			fFEVILIDPFHk	Unambiguous	N-Term(iTRAQ4plex); K12(iTRAQ4plex)	HCD	1.7872003	0	1	1	1.66373	2.0293	1.5887	1.9377955	11.73876079	2936.4666	2407.4993	4885.496	4665.241	13.99845627	3.261398656	3	High	7135	Unique	Used	83794	1	72.653	1	5.1825	-0.9394	1	100	26/44	33.58786	7135	1793.02	MS2	598.346	194	Glioma_grade 2_6.raw
379317149	NP_001243821.1	SRI	sorcin isoform D [Homo sapiens]	7	15	3.299853086	30.6527693	64.653562	41.11	6.25E-05	180	19.994	5.6304	Calcium binding protein	Cell communication,Signal transduction	Calcium ion binding	Cytoplasm	Plasma membrane																								
379317149			lSPQAVNSIAk	Unambiguous	N-Term(iTRAQ4plex); K11(iTRAQ4plex)	HCD	3.5728278	0	1	4	3.19988	3.7863	3.3924	4.0141335	10.26687082	728984.25	616073.06	2332659	2472999.2	11.87165298	4.129944451	2	High	2371	Unique	Used	50009	1	29.383	1	4.3026	-0.4031	1	3	18/20	41.42563	2371	1415.85	MS2	708.426	203	Glioma_grade 2_2.raw
379317149			lSPQAVNSIAk	Unambiguous	N-Term(iTRAQ4plex); K11(iTRAQ4plex)	HCD	4.4830427	0	1	4	4.58854	4.3088	4.6686	4.383978	3.765993521	396078.06	421795.88	1817420	1849143.9	4.446955461	1.223608012	2	High	2313	Unique	Used	57996	1	28.799	1	5.0232	-1.8688	1	4	18/20	3.545258	2313	1415.84	MS2	708.425	172	Glioma_grade 2_3.raw
379317149			sGTVDPQELQk	Unambiguous	N-Term(iTRAQ4plex); K11(iTRAQ4plex)	HCD	3.0513625	0	1	4	3.15501	2.9704	3.1374	2.9537818	3.495254555	340428.84	361587.3	1074055.8	1068050	4.262377941	0.396501602	2	High	1383	Unique	Used	49073	1	22.564	1	4.3846	-0.7248	1	6	17/20	20.05034	1383	1489.81	MS2	745.408	232	Glioma_grade 2_2.raw
379317149			dMSGTMGFNEFk	Unambiguous	N-Term(iTRAQ4plex); K12(iTRAQ4plex)	HCD	2.776765	0	1	4	2.62411	2.7021	2.8493	2.9339564	5.042308423	327059.62	317617.22	858241.06	931875.06	2.071358751	5.817175785	2	High	4841	Unique	Used	52334	1	47.26	1	5.7048	1.7965	1	9	21/22	28.7649	4841	1651.77	MS2	826.39	62	Glioma_grade 2_2.raw
379317149			dMSGTmGFNEFk	Unambiguous	N-Term(iTRAQ4plex); M6(Oxidation); K12(iTRAQ4plex)	HCD	1.8702621	0	1	4	1.86313	1.828	1.9133	1.8772639	1.886785086	301259.75	307043.84	561284.9	576402.3	1.344713176	1.879183673	2	High	3352	Unique	Used	50932	1	36.38	1	3.4441	1.3882	1	19	22-Dec	47.17768	3352	1667.77	MS2	834.387	53	Glioma_grade 2_2.raw
379317149			dmSGTMGFNEFk	Unambiguous	N-Term(iTRAQ4plex); M2(Oxidation); K12(iTRAQ4plex)	HCD	2.4239805	0	1	4	2.36638	2.3794	2.4684	2.481892	2.455990147	160813.75	159937.23	380547.16	396946.97	0.386463814	2.983023643	2	High	3624	Unique	Used	51187	1	38.38	1	4.4217	-2.1251	1	17	20/22	8.857627	3624	1667.76	MS2	834.384	39	Glioma_grade 2_2.raw
379317149			qHFISFDTDR	Unambiguous	N-Term(iTRAQ4plex)	HCD	3.687519	0	1	4	3.51042	3.7579	3.6218	3.8771002	4.325569307	136314.2	127337.586	478520.44	493700.56	4.81500598	2.208132882	2	High	2065	Unique	Used	72767	1	27.538	1	3.0846	-4.0653	1	9	15/18	3.464946	2065	1409.69	MS2	705.347	157	Glioma_grade 2_5.raw
379317149			qHFISFDTDR	Unambiguous	N-Term(iTRAQ4plex)	HCD	3.794284	0	1	4	3.66512	4.0402	3.5712	3.936664	5.817863059	108520.73	98445.77	397741.78	387547.94	6.884275993	1.835789571	3	High	2064	Unique	Used	72766	1	27.535	1	3.8606	-4.0532	1	4	20/36	4.726335	2064	1409.69	MS2	470.567	106	Glioma_grade 2_5.raw
379317149			lSPQAVNSIAk	Unambiguous	N-Term(iTRAQ4plex); K11(iTRAQ4plex)	HCD	3.1490269	0	1	4	3.00895	3.0493	3.2474	3.2909818	4.467590528	90632.38	89431.78	272708.53	294318.38	0.942944339	5.389681232	2	High	2265	Unique	Used	57952	1	28.427	1	4.2576	14.512	1	64	18/20	41.28262	2265	1415.87	MS2	708.437	175	Glioma_grade 2_3.raw
379317149			dmSGTmGFNEFk	Unambiguous	N-Term(iTRAQ4plex); M2(Oxidation); M6(Oxidation); K12(iTRAQ4plex)	HCD	2.6494133	0	1	4	2.57043	2.6853	2.615	2.7319276	2.713063813	64891.29	62114.27	166798.53	169691.7	3.092226314	1.21595217	2	High	2243	Unique	Used	49888	1	28.482	1	3.6507	-0.8249	1	63	13/22	25.1388	2243	1683.76	MS2	842.383	38	Glioma_grade 2_2.raw
379317149			lSPQAVNSIAk	Unambiguous	N-Term(iTRAQ4plex); K11(iTRAQ4plex)	HCD	1.8102983	0	1	4	1.7797	1.8143	1.8064	1.8414913	1.404609479	58024.785	56919.29	103266.69	104816.38	1.360144942	1.053229662	2	High	2416	Unique	Used	58093	1	29.537	1	4.5313	0.8902	1	75	18/20	39.5053	2416	1415.85	MS2	708.427	196	Glioma_grade 2_3.raw
379317149			qHFISFDTDR	Unambiguous	N-Term(iTRAQ4plex)	HCD	3.299853	0	1	4	3.20711	3.4311	3.1772	3.399069	3.934055979	50570.086	47268.535	162184.06	160669.03	4.772244491	0.663638057	3	High	2161	Unique	Used	72855	1	28.272	1	3.5861	0.2981	1	8	20/36	10.72571	2161	1409.69	MS2	470.569	131	Glioma_grade 2_5.raw
379317149			eLWAVLNGWR	Unambiguous	N-Term(iTRAQ4plex)	HCD	4.547462	0	1	4	4.67348	4.2262	4.9028	4.43351	6.430114139	38955.957	43079.176	182059.69	190991.95	7.108067016	3.386159416	2	High	8035	Unique	Used	55306	1	71.421	1	3.838	1.1127	1	48	16/18	26.1505	8035	1387.76	MS2	694.385	280	Glioma_grade 2_2.raw
379317149			cLTQSGIAGGYkPFNLETcR	Unambiguous	N-Term(iTRAQ4plex); C1(Methylthio); K12(iTRAQ4plex); C19(Methylthio)	HCD	4.4969926	0	1	4	3.68114	4.7961	4.2674	5.5599585	17.44835921	28254.219	21685.795	104007.74	120572.12	18.60062415	10.43084222	3	High	5575	Unique	Used	82557	1	57.217	1	6.3153	1.7646	1	25	24/76	31.72166	5575	2538.23	MS2	846.747	100	Glioma_grade 2_6.raw
379317149			dmSGTmGFNEFkELWAVLNGWR	Unambiguous	N-Term(iTRAQ4plex); M2(Oxidation); M6(Oxidation); K12(iTRAQ4plex)	HCD	5.4824867	1	1	4	5.18703	5.3396	5.6213	5.7866282	4.93249106	6412.152	6228.954	33260.05	36044.64	2.049512884	5.682162266	3	High	7509	Unique	Used	84082	1	76.487	1	4.2482	-2.4542	1	21	17/84	18.086	7509	2908.39	MS2	970.136	79	Glioma_grade 2_6.raw

Supplementary Table S2: Comparison of 340 differentially expressed proteins with differential transcript data in Grade II diffuse astrocytoma. Out of 340 differentially expressed proteins listed in Supplementary Table S1, a total of 195 proteins were found to be common with the transcript data (Oncomine data resource; www.oncomine.org, Ref. 11) and are listed in the Table along with their fold change values.

Gene Symbol	Protein fold change	Transcript fold change
AMPH	0.349	0.266
ANXA1	3.202	2.026
ANXA2	2.780	3.290
ANXA5	4.009	2.422
APOE	2.516	2.733
ARHGDIB	3.884	3.208
ASPH	2.626	3.473
ATP1B1	0.464	0.517
ATP2B1	0.455	0.550
ATP2B3	0.481	0.294
ATP6V1A	0.486	0.520
ATP6V1H	0.475	0.432
BASP1	0.400	0.599
BCAN	2.185	1.698
BTBD17	2.958	2.339
C4B	2.522	2.481
CALU	2.462	1.541
CAMK2A	0.372	0.185
CAMK2B	0.496	0.141
CAMKV	0.498	0.260
CD200	0.422	0.385
CD38	3.618	2.934
CKAP4	3.030	1.544
CLIC4	2.028	1.669
CNN3	3.198	2.024
CNPY3	2.237	1.566
CPLX2	0.266	0.259
CPNE3	3.976	1.639
CRYAB	2.923	1.593
CRYM	0.319	0.215
CXADR	0.362	0.540
CYBB	2.209	2.299
DBI	2.935	1.517
DDX39B	2.127	1.548
DKK3	0.448	0.578
DNM1	0.478	0.634
DPP6	0.388	0.348
DPYSL3	2.206	2.697
EEF1A1	2.092	1.529
EEF1A2	0.425	0.260
EGFR	2.035	2.976
ENAH	2.226	2.336
ENO2	0.466	0.319
ENPP6	2.049	0.416
ERAP1	2.013	2.273
ERGIC1	2.478	1.729
FADS2	3.057	2.137
FAM213A	2.011	1.566
FKBP10	4.443	5.342
FLNA	2.279	2.962
GAP43	0.498	0.470

GBA	2.311	1.780
GDA	0.420	0.107
GFAP	4.757	4.914
GLIPR2	4.413	1.821
GLS	0.424	2.044
GNAO1	0.356	0.461
GNAZ	0.474	0.431
GNB2L1	1.997	2.208
GOT2	0.397	0.478
GPR56	2.496	1.588
GRAMD3	2.595	1.617
GYG1	2.000	1.802
HK1	0.501	0.522
HMGB2	2.181	1.635
HNRNPD	3.339	1.618
HSP90B1	2.431	1.603
HSPB1	2.400	2.107
ICAM5	0.284	0.184
IDH1	2.622	3.191
IFITM1	2.395	1.704
IGF2BP3	4.567	4.380
IGSF8	0.446	0.336
ILF3	2.030	1.615
IQGAP1	3.012	1.743
ITGA6	3.197	1.606
ITGAV	2.194	1.518
ITGAX	2.783	2.559
ITGB1	2.335	2.565
ITGB2	2.948	3.614
ITSN1	0.324	1.902
KIAA0513	0.469	0.516
L1CAM	0.372	0.273
LCP1	3.260	2.054
LIMA1	2.405	1.862
LMAN1	2.118	1.745
MAP2K4	0.477	0.532
MAPT	0.457	0.615
MGST1	3.231	3.096
MSI2	2.245	2.031
MSN	2.918	1.910
MYH14	2.069	1.624
NFASC	0.402	0.355
NPC1	2.358	0.648
NPTN	0.463	0.557
NPTX1	0.373	0.229
NRXN3	0.447	0.284
NUMA1	2.336	1.874
OGDHL	0.502	0.197
OMG	0.490	0.411
OPALIN	0.360	0.130
ORM1	2.430	1.981
OXR1	0.500	0.483
P4HA1	3.347	1.806
P4HB	2.577	1.828
PACSIN1	0.490	0.533
PBXIP1	2.036	4.500
PDE2A	0.313	0.283
PDIA4	3.151	1.522
PON2	3.156	2.032

PPIB	2.970	1.836
PPP3CA	0.478	0.541
PPP3R1	0.429	0.473
PRDX4	2.424	1.885
PRKAR2B	0.396	0.317
PTPRC	2.222	2.616
PTPRN	0.437	0.286
QKI	2.334	1.527
RAB3A	0.258	0.217
RCN1	3.079	1.846
RDX	2.297	1.570
RP2	2.268	3.150
RPH3A	0.502	0.249
RPL10	2.295	2.029
RPL10A	2.322	1.509
RPL11	2.292	1.625
RPL12	2.260	2.601
RPL15	2.436	1.617
RPL17	2.112	1.663
RPL18A	2.212	2.471
RPL23	2.056	1.999
RPL28	2.319	1.817
RPL3	2.227	1.516
RPL32	2.363	1.511
RPL4	1.999	1.558
RPL5	2.211	1.631
RPL8	3.317	1.650
RPLP0	1.999	2.190
RPN2	2.058	1.658
RPS11	2.431	1.681
RPS14	2.174	1.753
RPS16	2.110	1.957
RPS19	2.491	2.331
RPS2	2.203	1.845
RPS20	2.801	1.684
RPS3	2.473	1.939
RPS5	3.024	1.789
RPS8	2.358	1.706
RPS9	2.217	1.713
RTN4RL2	0.454	0.153
RYR2	0.391	0.235
S100A11	2.175	4.362
SCN2A	0.443	0.430
SCP2	2.683	1.566
SEPT3	0.404	0.373
SEPT5	0.271	0.546
SEPT6	0.384	1.505
SGIP1	0.385	0.419
SH3GL2	0.446	0.349
SIRPA	0.379	0.615
SLC14A1	2.839	2.395
SLC17A7	0.380	0.149
SLC1A2	0.407	0.605
SLC2A3	0.317	0.414
SLC6A17	0.399	0.266
SLC7A14	0.385	0.652
SLC8A2	0.372	0.203
SNAP25	0.502	0.572
SNAP91	0.441	0.222

SNCA	0.307	0.250
SNCG	0.467	0.237
SNRNP200	2.016	1.558
SNTB1	2.561	2.861
SOAT1	4.359	2.732
SORD	2.564	1.740
SPARC	2.446	3.292
SRI	3.300	1.616
SSR1	2.047	1.895
STX1A	0.394	0.192
STX1B	0.389	0.296
SV2A	0.322	0.293
SV2B	0.280	0.639
SYN1	0.224	0.211
SYNPR	0.335	0.180
SYT1	0.267	0.183
TAPBP	3.298	1.514
THY1	0.481	0.387
TMX1	2.531	1.593
TNC	2.811	4.698
TPRG1L	0.359	0.494
UGT8	3.097	0.280
VAMP2	0.409	0.445
VAT1	2.968	1.867
VIM	4.468	2.402
YWHAG	0.445	0.559

Supplementary Table S3: Immunohistochemistry scoring details for four differentially expressed proteins - BCAN, EGFR, ENPP6 and HNRNP K. Immunohistochemistry was performed using tissue microarrays consisting of 4 control subjects and 13 diffuse astrocytoma samples. Scoring was based on both staining intensities and distributions. Scores +1, +2 and +3 indicate low, medium and strong staining, respectively.

Protein	iTRAQ result	Samples (n)	Staining intensity			
			**0**	**1+**	**2+**	**3+**
**EGFR**	Upreg	Case (13)	2	4	6	1
		Control (4)	4	-	-	-
**BCAN**	Upreg	Case (13)	3	5	5	0
		Control (4)	4	-	-	-
**ENPP6**	Upreg	Case (13)	9	3	1	0
		Control (4)	4	-	-	-
**HNRNPK**	Upreg	Case (13)	0	2	5	6
		Control (4)	4	-	-	-

Supplementary Table S4: Ingenuity Pathway Analysis of the differentially expressed proteins associated with major molecular and cellular functions (A), networks and processes (B) and those associated canonical pathways (C). Differentially expressed proteins from Supplementary Table S1 were used for the analysis. The Table includes only top 5 molecular and cellular functions, networks and processes or pathways and proteins from the dataset mapping to these respective groups are shown in bold.

A. **Molecular and cellular functions**

**Category**	**p-value**	**Molecules**
Protein Synthesis	8.26E-09-8.54E-03	**APOE, IGF2BP3, HNRNPK, RPS7, RPS3A, GBA, FLNA, SRCIN1, RPS9, ITGAV, RPS20, ASPH, RPS5, APEX1, EGFR, AGT, ITGB1, CALR, ERAP1, NCBP1, RPL23, EEF1A1, RPS4X, CIRBP, FBXO2, TPP1, MYH9, RPS14, HSPB1**
RNA Post-Transcriptional Modification	2.63E-07-2.72E-03	**RPL11, HNRNPU, NCBP1, RPS19, HNRNPD, HNRNPK, RPL7, FUS, RPS7, RPS16, DDX39B, RPL5, CIRBP, RPS15, SNRNP200, APEX1, RPS24, EGFR**
Cellular Movement	5.52E-07-1.93E-02	**CRYAB, ILF3, GNB2L1, HSPA5, BCAN, SRCIN1, ITGAV, PDE2A, LCP1, PON2, MYH14, ITGA6, THY1, ANXA2, SCAI, L1CAM, GNAZ, ARHGDIB, ORM1, GNAO1, SIRPA, HMGB2, HSPB1, APOE, MX1, PPIB, IQGAP1, CLIC4, HNRNPK, C4A/C4B, TNC, CD47, DKK3, FLNA, ANXA1, CD38, IGSF8, NCL, PPP3CA, EGFR, AGT, ITGB1, PLXNA1, AP2M1, RPS19, VIM, SNCG, DNM1, GLIPR2, ITGB2, SPARC, MYH9, CXADR, MSN, ITGAX**
Cell-To-Cell Signaling and Interaction	8.21E-07-1.93E-02	**KCND2, CRYAB, CADM3, GNB2L1, NPTX1, PTPRC, NSF, BCAN, COMT, AMPH, ITGAV, KRT1, CD200, YWHAG, DDOST, ITGA6, THY1, L1CAM, ANXA2, CORO1C, ORM1, MAPT, SYT1, SNCA, SYN1, SIRPA, RPSA, SRI, APOE, PPIB, RAB27B, IQGAP1, C4A/C4B, TNC, CD47, DKK3, FLNA, ANXA1, ANXA5, CD38, BCAP31, NCL, PPP3CA, EGFR, AGT, ITGB1, CALR, VIM, G3BP1, ITGB2, LGALS3BP, SPARC, SLC1A2, MYH9, SYN2, CXADR, ITGAX, MSN**
Cellular Function and Maintenance	2.74E-06-1.93E-02	**APOE, MAP2, ITSN1, RAB27B, GNB2L1, HSPA5, GAP1, AP2A2, NSF, HSP90B1, CD47, CPLX2, FLNA, ICAM5, ANXA1, AMPH, ITGAV, MAP1A, NCL, PPP3CA, EGFR, ITGB1, CALR, AP2M1, LCP1, ABCD3, SEPT5, NUMA1, ITGA6, VIM, VAMP2, LMAN1, AP2S1, RHGDIB, SLC3A2, DNM1, SCAMP5, ITGB2, CORO1C, ATP6V1H, MAPT, MYH9, GAP43, SYT1, SNCA, CXADR, HSPB1**

B. Networks and processes

Top Functions	Molecules in Network	Score	Focus Molecules
Cell-To-Cell Signaling and Interaction, Tissue Development, Cellular Movement	Actin, **AMPH, ANXA1, ARHGDIB, BCAP31, CALM1 (includes others), CAMK2D, Cg, DNM1, ERAP1**, Fibrinogen, **FLNA**, Focal adhesion kinase, **GBA**, HDL, **HSP90B1, IGSF8, ITGA6, ITGAV, ITGAX, ITGB1, ITGB2,** Laminin, **MAPT,** NFkBcomplex), **PACSIN1,** Pkc(s), Rac, **RDX, RPSA, RYR2, SH3GLB2, SNCA, SOAT1, TNC**	35	26
Cell-To-Cell Signaling and Interaction, Hematological System Development and Function, Immune Cell Trafficking	**ABCD3, AGT, ANXA2, APOD, BCAN**, CD3, **CD38, CD47, CD200, EGFR,** FActin, **GSN, HADHB,** Hsp90, **IQGAP1, LCP1**, mediator, **NCL,** P38 **MAPK,** p85 (pik3r), PI3K (complex), **PRKAR2B, PTPRC, RPL6, RPL8, RPLP0, RPS2, RPS3, RPS25, S100A11, SIRPA, SLC2A3, SLC3A2**, STAT5a/b, TCR	35	26
Protein Synthesis, Gene Expression, Developmental Disorder	**ANXA5,** Ap1, **C4A/C4B, CALR, CANX,** ERK1/2, **GLIPR2,** HLA-B27, **HMGB2, HSPA5, HSPB1, ITSN1,** LDL, MAP2K1/2, MHC Class I (complex), **NPC1, PDIA3**, PI3K (family), **PPIB,** Ras, Rnr, **RPL12, RPS5, RPS8, RPS9, RPS12, RPS14, RPS20, RPS24, RPS27A, SET, SNCG, SPARC,** Tap, **TAPBP**	33	25

C. Canonical pathways

Pathway	Molecules	-log (P-value)	Ratio
EIF2 Signaling	**RPL11, RPS8, RPL7, RPS11, RPS7, RPL6, RPS3A, RPL18A, RPL7A, RPS9, RPS20, RPL12, RPL8, RPS5, RPS3, RPL18, RPS24, RPL4, RPL3, RPL27, RPS2, RPL17, RPS19, RPL23A, RPL21, RPL23, RPLP0, RPS12, RPL10A, RPL15, RPS4X, RPS16, RPL5, RPL28, RPL26L1, RPL10, RPL32, RPS15, RPS25, RPL38, RPS14, RPS27A, RPSA**	3.5E01	2.38E-01
Regulation of eIF4 and p70S6K Signaling	**ITGB1, RPS2, RPS19, RPS8, RPS11, RPS12, RPS7, RPS4X, RPS3A, RPS16, RPS9, RPS15, RPS20, RPS25, RPS3, RPS5, RPS14, RPSA, RPS27A, RPS24**	1.14E01	1.27E-01

Calcium Signaling	**CALR, MYL6, ATP2B1, MYH14, RYR2, SLC8A2, ATP2B2, MYL9, CALM1(includes thers), CAMK2A, CAMK2D, PRKAR2B, ATP2B3, PPP3R1, MYH9, ASPH, PPP3CA, ATP2B4, CAMK2B**	9.11E00	1.01E-01
mTOR Signaling	**RPS2, RPS19, RPS8, RPS11, RPS12, RPS7, RPS4X, RPS3A, RPS16, RPS9, RPS15, RPS20, RPS25, RPS3, RPS5, RPS14, RPSA, RPS27A, RPS24**	8.6E00	1.01E-01
Lipid Antigen Presentation by CD1	**AP2B1, CALR, AP2M1, AP2A1, PDIA3, CANX, AP2A2, AP2S1**	8E00	2.96E-01

Supplementary Table S5: List of proteins with secretory potential. Proteins with secretory potential were derived from the differentially expressed protein dataset (n=340) on the basis of mapping to Signal peptide, transmembrane domain or Exocarta database or detectability in cerebrospinal fluid (CSF) or plasma.

Gene Symbol	Protein name	Peptides	PSM	Fold change	Signal	TM	Exocarta	Plasma	CSF
A1BG	alpha-1B-glycoprotein precursor	4	4	2.67	+		+	+	+
ABCD3	ATP-binding cassette sub-family D member 3 isoform a	7	8	2.09		+			
AGT	angiotensinogen preproprotein	2	2	3.17	+		+	+	+
ALDH3A2	fatty aldehyde dehydrogenase isoform 2	5	13	2.44		+	+		
ALDH9A1	4-trimethylaminobutyraldehyde dehydrogenase	5	9	2.12			+	+	
AMPH	amphiphysin isoform 2	8	18	0.35		+			
ANP32B	acidic leucine-rich nuclear phosphoprotein 32 family member B	2	2	2.92			+	+	
ANXA1	annexin A1	4	5	3.20			+	+	
ANXA2	annexin A2 isoform 2	7	10	2.78			+	+	
ANXA5	annexin A5	10	29	4.01			+	+	+
AP2A1	AP-2 complex subunit alpha-1 isoform 2	14	25	0.46			+		
AP2A2	AP-2 complex subunit alpha-2 isoform 2	12	21	0.44			+		
AP2M1	AP-2 complex subunit mu isoform b	10	21	0.46			+		
APEX1	DNA-(apurinic or apyrimidinic site) lyase	2	2	2.75				+	
APOD	apolipoprotein D precursor	4	6	2.61	+		+	+	+
APOE	apolipoprotein E precursor	2	3	2.52	+		+	+	+
ARHGDIB	rho GDP-dissociation inhibitor 2	2	3	3.88			+	+	
ASPH	aspartyl/asparaginyl beta-hydroxylase isoform f	4	4	2.63		+			
ATP13A1	probable cation-transporting ATPase 13A1	3	4	2.06		+			
ATP1A3	sodium/potassium-transporting ATPase subunit alpha-3 isoform 1	17	89	0.44		+	+		+
ATP1B1	sodium/potassium-transporting ATPase subunit beta-1	9	35	0.46		+	+		
ATP2B1	plasma membrane calcium-transporting ATPase 1 isoform 1b	7	15	0.46		+	+		
ATP2B2	plasma membrane calcium-transporting ATPase 2 isoform 2	14	25	0.47		+			
ATP2B3	plasma membrane calcium-transporting ATPase 3 isoform 3a	4	7	0.48		+			

ATP2B4	plasma membrane calcium-transporting ATPase 4 isoform 4a	10	19	0.49		+	+		
ATP6V1A	V-type proton ATPase catalytic subunit A	13	18	0.49			+	+	
ATP6V1H	V-type proton ATPase subunit H isoform 2	10	17	0.48			+		
ATP8A1	probable phospholipid-transporting ATPase IA isoform a	9	14	0.37		+			
BANF1	barrier-to-autointegration factor	2	2	2.79				+	
BASP1	brain acid soluble protein 1	3	4	0.40			+	+	
BCAN	brevican core protein isoform 1 precursor	6	8	2.19	+	+		+	+
BCAP31	B-cell receptor-associated protein 31 isoform b	3	8	2.05		+			
BTBD17	BTB/POZ domain-containing protein 17 precursor	2	2	2.96	+				
C4B	PREDICTED: complement C4-A isoform 2	15	17	2.52			+		
CADM3	cell adhesion molecule 3 isoform 2 precursor	5	12	0.38	+	+	+	+	+
CALM3	calmodulin	3	11	0.46				+	+
CALR	calreticulin precursor	11	28	2.66	+		+	+	+
CALU	calumenin isoform a precursor	3	5	2.46	+		+	+	
CAMK2A	calcium/calmodulin-dependent protein kinase type II subunit alpha isoform 2	8	17	0.37				+	+
CAMK2B	calcium/calmodulin-dependent protein kinase type II subunit beta isoform 4	5	11	0.50				+	
CAMK2D	calcium/calmodulin-dependent protein kinase type II subunit delta isoform 1	5	11	0.48				+	+
CANX	calnexin precursor	11	32	2.18	+	+	+	+	
CASC4	protein CASC4 isoform b	3	5	2.33		+			
CCDC47	coiled-coil domain-containing protein 47 precursor	5	14	2.15	+				
CD200	OX-2 membrane glycoprotein isoform a precursor	4	10	0.42	+	+			+
CD38	ADP-ribosyl cyclase 1	5	10	3.62		+			
CD47	leukocyte surface antigen CD47 isoform 1 precursor	4	7	0.37	+	+	+		
CKAP4	cytoskeleton-associated protein 4	13	21	3.03		+	+		
CLIC4	chloride intracellular channel protein 4	3	4	2.03			+	+	
CNPY2	protein canopy homolog 2 isoform 1 precursor	3	5	2.02	+			+	
CNPY3	protein canopy homolog 3 precursor	3	3	2.24	+	+			

COMT	catechol O-methyltransferase isoform S-COMT	8	10	2.09		+	+		
CORO1C	coronin-1C isoform 1	3	5	2.34			+	+	
CPNE3	copine-3	3	6	3.98			+	+	
CRYAB	alpha-crystallin B chain	5	17	2.92			+		
CRYM	thiomorpholine-carboxylate dehydrogenase isoform 1	2	6	0.32			+		+
CTNNA1	catenin alpha-1	5	6	3.03			+		
CXADR	coxsackievirus and adenovirus receptor isoform 2 precursor	2	4	0.36	+	+			
CYB5A	cytochrome b5 isoform 2	2	7	2.19		+		+	
CYBB	cytochrome b-245 heavy chain	3	4	2.21		+			
DBI	acyl-CoA-binding protein isoform 3	3	21	2.94			+	+	
DCAKD	dephospho-CoA kinase domain-containing protein	3	6	2.07		+			
DDOST	dolichyl-diphosphooligosaccharide--protein glycosyltransferase 48 kDa subunit precursor	4	7	2.19		+			
DKK3	dickkopf-related protein 3 precursor	2	5	0.45	+		+	+	+
DNM1	dynamin-1 isoform 2	15	29	0.48				+	
DPP6	dipeptidyl aminopeptidase-like protein 6 isoform 3	8	12	0.39		+			+
EEF1A1	elongation factor 1-alpha 1	3	9	2.09			+	+	
EEF1A2	elongation factor 1-alpha 2	2	5	0.42			+		
EGFR	epidermal growth factor receptor isoform a precursor	2	4	2.04	+	+	+	+	+
ENO2	gamma-enolase	6	15	0.47			+	+	+
ENPP6	ectonucleotide pyrophosphatase/phosphodiesterase family member 6 precursor	9	22	2.05	+		+		
ERAP1	endoplasmic reticulum aminopeptidase 1 isoform b precursor	6	6	2.01	+		+	+	+
ERGIC1	endoplasmic reticulum-Golgi intermediate compartment protein 1	4	5	2.48		+			
ERLIN2	erlin-2 isoform 1	5	7	2.25	+				
FADS2	fatty acid desaturase 2	2	3	3.06		+			
FAM213A	redox-regulatory protein FAM213A isoform 2 precursor	4	16	2.01		+			
FKBP10	peptidyl-prolyl cis-trans isomerase FKBP10 precursor	2	2	4.44	+				
FLNA	filamin-A isoform 1	11	13	2.28			+	+	+
FUS	RNA-binding protein FUS isoform 3	2	6	2.10			+	+	
G3BP1	ras GTPase-activating protein-binding protein 1	2	2	2.44				+	

GAP43	neuromodulin isoform 2	3	3	0.50				+	
GDA	guanine deaminase isoform b	3	5	0.42				+	
GFAP	glial fibrillary acidic protein isoform 1	15	46	4.76				+	
GGH	gamma-glutamyl hydrolase precursor	3	6	2.10	+		+	+	+
GLIPR2	Golgi-associated plant pathogenesis-related protein 1	2	2	4.41			+	+	
GNB1	guanine nucleotide-binding protein G(I)/G(S)/G(T) subunit beta-1	3	17	0.50			+		
GNB2L1	guanine nucleotide-binding protein subunit beta-2-like 1	6	10	2.00			+	+	
GNG2	guanine nucleotide-binding protein G(I)/G(S)/G(O) subunit gamma-2 precursor	2	23	0.39			+		
GOLM1	Golgi membrane protein 1	2	2	2.55		+		+	+
GOT2	aspartate aminotransferase, mitochondrial precursor	8	11	0.40			+	+	+
GPR56	G-protein coupled receptor 56 isoform b precursor	5	6	2.50	+	+			
GRAMD3	GRAM domain-containing protein 3 isoform 3	2	2	2.59		+			
GSN	gelsolin isoform b	11	19	2.77	+		+	+	+
GSTM2	glutathione S-transferase Mu 2 isoform 2	7	12	2.23			+		
HIBADH	3-hydroxyisobutyrate dehydrogenase, mitochondrial precursor	2	2	2.19				+	
HIST1H1B	histone H1.5	2	2	2.13			+	+	
HIST1H1C	histone H1.2	4	7	2.04				+	
HK1	hexokinase-1 isoform HKI-td	13	22	0.50				+	
HMGB2	high mobility group protein B2	2	3	2.18				+	
HNRNPA3	heterogeneous nuclear ribonucleoprotein A3	4	9	2.21				+	
HNRNPC	heterogeneous nuclear ribonucleoproteins C1/C2 isoform b	5	8	2.50			+	+	
HNRNPD	heterogeneous nuclear ribonucleoprotein D0 isoform d	7	21	3.34				+	
HNRNPK	heterogeneous nuclear ribonucleoprotein K isoform b	6	13	2.06			+	+	
HNRNPU	heterogeneous nuclear ribonucleoprotein U isoform b	7	8	2.05				+	
HP	haptoglobin isoform 1 preproprotein	3	5	2.91	+		+	+	+
HSP90B1	endoplasmin precursor	14	58	2.43	+		+	+	+
HSPA5	78 kDa glucose-regulated protein precursor	18	39	2.36	+		+	+	+
HSPB1	heat shock protein beta-1	2	3	2.40			+	+	

ICAM5	intercellular adhesion molecule 5 precursor	4	5	0.28	+	+	+		+
IDH1	isocitrate dehydrogenase [NADP] cytoplasmic	5	6	2.62			+	+	
IFITM1	interferon-induced transmembrane protein 1	2	2	2.40		+	+		
IGSF8	immunoglobulin superfamily member 8 precursor	3	12	0.45	+	+	+	+	+
ILF3	interleukin enhancer-binding factor 3 isoform c	9	19	2.03				+	
IQGAP1	ras GTPase-activating-like protein IQGAP1	3	4	3.01			+		
ITGA6	integrin alpha-6 isoform b precursor	3	3	3.20	+	+	+	+	
ITGAV	integrin alpha-V isoform 2 precursor	12	15	2.19	+	+	+		
ITGAX	integrin alpha-X precursor	3	3	2.78	+	+			
ITGB1	integrin beta-1 isoform 1A precursor	3	5	2.34	+	+	+	+	+
ITGB2	integrin beta-2 precursor	5	6	2.95	+	+	+		
KCND2	potassium voltage-gated channel subfamily D member 2 precursor	2	2	2.07	+	+			
KRT1	keratin, type II cytoskeletal 1	2	4	0.39			+	+	
L1CAM	neural cell adhesion molecule L1 isoform 3 precursor	6	14	0.37	+	+	+	+	
LCP1	plastin-2	3	3	3.26			+	+	+
LEMD2	LEM domain-containing protein 2 isoform 2	2	3	3.16		+			
LGALS3BP	galectin-3-binding protein precursor	2	2	3.13			+	+	+
LGALS3BP	galectin-3-binding protein precursor	2	2	3.13	+				
LIMA1	LIM domain and actin-binding protein 1 isoform 4	4	4	2.41			+		
LMAN1	protein ERGIC-53 precursor	6	8	2.12	+	+	+		
LMAN2	vesicular integral-membrane protein VIP36 precursor	4	8	2.37	+	+	+	+	+
LPCAT1	lysophosphatidylcholine acyltransferase 1	2	4	2.44		+			
LRRC59	leucine-rich repeat-containing protein 59	5	13	2.35		+			
MANF	mesencephalic astrocyte-derived neurotrophic factor precursor	3	8	2.18	+			+	
MAP1A	microtubule-associated protein 1A	20	34	0.49			+		
MDH2	malate dehydrogenase, mitochondrial precursor	12	25	0.49			+	+	+
MESDC2	LDLR chaperone MESD precursor	2	3	2.61	+				
MGST1	microsomal glutathione S-transferase 1	2	4	3.23		+			
MMGT1	membrane magnesium transporter 1 precursor	2	2	2.41	+	+			
MSN	moesin	5	10	2.92			+	+	+

MX1	interferon-induced GTP-binding protein Mx1	3	3	2.69			+		
MYH14	myosin-14 isoform 2	13	15	2.07			+		
MYH9	myosin-9	39	79	2.79			+	+	
MYL6	myosin light polypeptide 6 isoform 1	3	8	2.31			+		
NCL	nucleolin	9	25	2.12			+		
NFASC	neurofascin isoform 4 precursor	8	27	0.40	+	+			+
NPC1	Niemann-Pick C1 protein precursor	2	3	2.36	+	+	+		
NPTN	neuroplastin isoform c precursor	8	22	0.46	+	+			
NPTX1	neuronal pentraxin-1 precursor	5	11	0.37	+				+
NPTXR	neuronal pentraxin receptor	5	6	0.44		+		+	+
NRXN3	neurexin-3-beta isoform 1 precursor	2	5	0.45	+	+			
NUCB2	nucleobindin-2 precursor	4	4	2.24	+	+	+	+	
OMG	oligodendrocyte-myelin glycoprotein precursor	3	8	0.49					+
OMGP	oligodendrocyte-myelin glycoprotein precursor	3	8	0.49	+	+			
OPALIN	opalin isoform b	3	4	0.36		+			
ORM1	alpha-1-acid glycoprotein 1 precursor	3	5	2.43	+			+	
P4HA1	prolyl 4-hydroxylase subunit alpha-1 isoform 2 precursor	6	9	3.35	+				
P4HB	protein disulfide-isomerase precursor	15	42	2.58	+		+	+	+
PAM	peptidyl-glycine alpha-amidating monooxygenase isoform d preproprotein	4	4	0.48	+		+	+	+
PDCD6	programmed cell death protein 6	2	8	2.12			+	+	
PDIA3	protein disulfide-isomerase A3 precursor	13	44	2.24	+		+	+	
PDIA4	protein disulfide-isomerase A4 precursor	13	28	3.15	+	+	+	+	
PDIA6	protein disulfide-isomerase A6 precursor	5	9	2.49	+		+	+	
PHGDH	D-3-phosphoglycerate dehydrogenase	8	16	2.34			+	+	
PLXNA1	plexin-A1 precursor	6	6	0.48	+	+	+		
PLXNA4	plexin-A4 isoform 1 precursor	6	7	0.38	+	+			
PPIB	peptidyl-prolyl cis-trans isomerase B precursor	6	12	2.97		+	+	+	+
PPIB	peptidyl-prolyl cis-trans isomerase B precursor	6	12	2.97					+
PPP3CA	serine/threonine-protein phosphatase 2B catalytic subunit alpha isoform isoform 3	3	6	0.48				+	
PRDX4	peroxiredoxin-4 precursor	4	8	2.42	+		+		
PRKCSH	glucosidase 2 subunit beta isoform 2 precursor	5	7	2.06	+		+	+	+
PSME1	proteasome activator complex subunit 1 isoform 1	8	12	2.04			+	+	

PTPRC	receptor-type tyrosine-protein phosphatase C isoform 2 precursor	4	4	2.22			+	+	
PTPRN	receptor-type tyrosine-protein phosphatase-like N isoform 3	6	9	0.44	+	+			+
RAB27B	ras-related protein Rab-27B	2	6	0.28			+		
RCN1	reticulocalbin-1 precursor	3	5	3.08	+				
RCN2	reticulocalbin-2 precursor	4	5	2.18	+				
RDX	radixin	5	7	2.30			+		
RP2	protein XRP2	3	4	2.27			+		
RPL10	60S ribosomal protein L10 isoform a	4	9	2.30			+	+	
RPL10A	60S ribosomal protein L10a	6	18	2.32			+	+	
RPL11	60S ribosomal protein L11 isoform 2	2	6	2.29			+	+	
RPL12	60S ribosomal protein L12	4	19	2.26			+	+	
RPL15	60S ribosomal protein L15 isoform 1	3	7	2.44			+	+	
RPL17	60S ribosomal protein L17 isoform b	4	15	2.11				+	
RPL18	60S ribosomal protein L18	3	7	2.47			+		
RPL23	60S ribosomal protein L23	4	10	2.06			+		
RPL23A	60S ribosomal protein L23a	5	12	3.03				+	
RPL26L1	60S ribosomal protein L26-like 1	2	2	2.58			+		
RPL27	60S ribosomal protein L27	2	3	2.18			+		
RPL28	60S ribosomal protein L28 isoform 5	2	4	2.32			+		
RPL3	60S ribosomal protein L3 isoform b	6	14	2.23			+	+	
RPL32	60S ribosomal protein L32	3	7	2.36				+	
RPL4	60S ribosomal protein L4	10	27	2.00			+	+	
RPL5	60S ribosomal protein L5	7	12	2.21			+	+	
RPL6	60S ribosomal protein L6	3	3	2.09			+		
RPL7	60S ribosomal protein L7	5	10	2.42				+	
RPL7A	60S ribosomal protein L7a	4	9	2.09				+	
RPL8	60S ribosomal protein L8	2	6	3.32			+		
RPLP0	60S acidic ribosomal protein P0	6	11	2.00			+	+	
RPN1	dolichyl-diphosphooligosaccharide--protein glycosyltransferase subunit 1 precursor	12	25	2.32	+	+	+		
RPN2	dolichyl-diphosphooligosaccharide--protein glycosyltransferase subunit 2 isoform 2 precursor	7	7	2.06	+	+			
RPS11	40S ribosomal protein S11	5	6	2.43			+	+	

RPS12	40S ribosomal protein S12	4	10	2.52				+	
RPS14	40S ribosomal protein S14	2	3	2.17			+	+	
RPS16	40S ribosomal protein S16	3	5	2.11			+	+	
RPS19	40S ribosomal protein S19	4	15	2.49			+	+	
RPS2	40S ribosomal protein S2	10	20	2.20			+	+	
RPS20	40S ribosomal protein S20 isoform 2	2	11	2.80			+	+	
RPS24	40S ribosomal protein S24 isoform a	3	5	2.58			+	+	
RPS25	40S ribosomal protein S25	3	5	2.56			+	+	
RPS27A	ubiquitin-40S ribosomal protein S27a precursor	4	13	0.49			+	+	
RPS3	40S ribosomal protein S3	7	23	2.47			+	+	
RPS3A	40S ribosomal protein S3a	4	10	2.21			+	+	
RPS4X	40S ribosomal protein S4, X isoform X isoform	6	15	2.19			+	+	
RPS5	40S ribosomal protein S5	3	8	3.02			+	+	
RPS7	40S ribosomal protein S7	4	9	2.15			+	+	
RPS8	40S ribosomal protein S8	3	7	2.36			+	+	
RPS9	40S ribosomal protein S9	3	4	2.22			+	+	
RPSA	40S ribosomal protein SA	6	11	2.37			+	+	
RTN4RL2	reticulon-4 receptor-like 2 precursor	3	3	0.45	+			+	+
RYR2	ryanodine receptor 2	9	11	0.39		+			
S100A11	protein S100-A11	2	2	2.17			+	+	
SCAMP5	secretory carrier-associated membrane protein 5	3	13	0.45		+			
SCARB2	lysosome membrane protein 2 isoform 1 precursor	8	9	2.40	+	+	+		
SCN2A	sodium channel protein type 2 subunit alpha isoform 1	4	5	0.44		+			
SCP2	non-specific lipid-transfer protein isoform 8 proprotein	5	12	2.68				+	
SEPT6	septin-6 isoform A	2	2	0.38				+	
SERPINH1	serpin H1 precursor	5	7	4.16	+			+	
SET	protein SET isoform 4	2	3	2.00			+		
SIRPA	tyrosine-protein phosphatase non-receptor type substrate 1 precursor	8	26	0.38	+	+	+		+
SLC14A1	urea transporter 1 isoform 2	2	3	2.84		+			
SLC17A7	vesicular glutamate transporter 1	3	4	0.38		+			
SLC1A2	excitatory amino acid transporter 2 isoform 2	12	44	0.41		+			
SLC2A3	solute carrier family 2, facilitated glucose transporter member 3	3	3	0.32		+	+		
SLC3A2	4F2 cell-surface antigen heavy chain isoform f	12	17	0.46		+	+	+	+
SLC44A1	choline transporter-like protein 1	3	4	2.14		+	+		

SLC4A10	sodium-driven chloride bicarbonate exchanger isoform 2	4	7	0.40		+			
SLC6A17	sodium-dependent neutral amino acid transporter SLC6A17	4	8	0.40		+			
SLC7A14	probable cationic amino acid transporter	5	5	0.39		+			
SLC8A2	sodium/calcium exchanger 2 precursor	10	34	0.37	+	+			
SNAP91	clathrin coat assembly protein AP180 isoform a	7	16	0.44				+	
SNCA	alpha-synuclein isoform NACP112	2	5	0.31				+	
SNCG	gamma-synuclein	2	3	0.47			+		
SNTB1	beta-1-syntrophin	2	3	2.56				+	
SOAT1	sterol O-acyltransferase 1 isoform 1	2	2	4.36		+			
SORD	sorbitol dehydrogenase	2	7	2.56			+	+	
SPARC	SPARC precursor	5	8	2.45	+		+		+
SRI	sorcin isoform D	7	15	3.30			+		
SSR1	translocon-associated protein subunit alpha precursor	2	2	2.05	+	+			
STT3A	dolichyl-diphosphooligosaccharide--protein glycosyltransferase subunit STT3A	3	4	2.28		+			
STX1A	syntaxin-1A isoform 1	6	19	0.39		+			
STX1B	syntaxin-1B	10	32	0.39		+			
SV2A	synaptic vesicle glycoprotein 2A	7	22	0.32		+			
SV2B	synaptic vesicle glycoprotein 2B isoform 1	6	16	0.28		+			
SYN1	synapsin-1 isoform Ia	13	36	0.22				+	
SYNPR	synaptoporin isoform 1	4	4	0.34	+	+			
SYPH	synaptophysin	3	14	0.35		+			
SYT1	synaptotagmin-1	8	34	0.27		+	+		
SYT7	synaptotagmin-7 isoform 1	3	5	0.31		+			
TAPBP	tapasin isoform 1 precursor	2	2	3.30	+	+			
THY1	thy-1 membrane glycoprotein preproprotein	4	22	0.48	+		+	+	+
TM9SF3	transmembrane 9 superfamily member 3 precursor	2	4	2.59	+	+			
TMED10	transmembrane emp24 domain-containing protein 10 precursor	4	9	2.05	+	+	+		
TMED4	transmembrane emp24 domain-containing protein 4 precursor	3	4	2.17	+	+			+
TMED7-TICAM2	TRAM adaptor with GOLD domain isoform 2 precursor	2	6	2.01	+	+			
TMEM30A	cell cycle control protein 50A isoform 1	4	5	0.44		+			
TMEM43	transmembrane protein 43	8	10	2.29		+			

TMX1	thioredoxin-related transmembrane protein 1 precursor	4	6	2.53	+	+			
TNC	tenascin precursor	12	17	2.81	+	+	+	+	
TPP1	tripeptidyl-peptidase 1 preproprotein	4	10	2.22	+		+	+	+
TPRG1L	tumor protein p63-regulated gene 1-like protein	2	3	0.36			+		
TXNDC12	thioredoxin domain-containing protein 12 precursor	3	7	2.15	+			+	
UGT8	2-hydroxyacylsphingosine 1-beta-galactosyltransferase precursor	2	3	3.10		+			
VAMP2	vesicle-associated membrane protein 2	4	9	0.41		+			
VAT1	synaptic vesicle membrane protein VAT-1 homolog	4	7	2.97			+		
VIM	vimentin	12	34	4.47			+	+	
XRCC6	X-ray repair cross-complementing protein 6	8	10	2.06			+	+	
YWHAG	14-3-3 protein gamma	2	2	0.45			+	+	+

Supplementary Table S6: List of proteins with secretory potential and their proteotypic peptides. Proteins listed in Supplementary Table S5 were first screened for mapping to at least two of the three criteria namely Signal peptide, transmembrane domain or Exocarta database. The filtered list was further screened for experimental detection in cerebrospinal fluid (CSF) or plasma. The proteotypic peptides for these proteins were derived from the GPMdb's MRM database. The same strategy was also applied to differentially expressed proteins from Grade III – anaplastic astrocytoma and proteins along with their proteotypic peptides are

Gene symbol	Protein name	Peptides	Protein Fold change	Architecture	Exocarta	CSF	Plasma	Tumor grade	Top 10 most common proteotypic peptides in GPMdb's MRM database
A1BG	alpha 1B-glycoprotein precursor	5	2.67	SP	+	+	+	Gr II and Gr III	CLAPLEGAR;ATWSGAVLAGR;HQFLLTGDTQGR;SGLSTGWTQLSK;NGVAQEPVHLDSPAIK;IFFHLNAVALGDGGHYTCR;CEGPIPDVTFELLR;VTLTCVAPLSGVDFQLR;SWVPHTFESELSDPVELLVAES;SLPAPWLSMAPVSWITPGLK
A2M	alpha-2-macroglobulin precursor	22	2.13	SP	+	+	+	Gr III only	YGAATFTR;AAQVTIQSSGTFSSK;SSGSLLNNAIK;FSGQLNSHGCFYQQVK;MVSGFIPLKPTVK;IAQWQSFQLEGGLK;NQGNTWLTAFVLK;AGAFCLSEDAGLGISSTASLR;GCVLLSYLNETVTVSASLESVR;AFQPFFVELTMPYSVIR
ALB	albumin preproprotein	41	2.99	SP	+	+	+	Gr III only	CCTESLVNR;YICENQDSISSK;LVNEVTEFAK;KVPQVSTPTLVEVSR;VPQVSTPTLVEVSR;QNCELFEQLGEYK;AVMDDFAAFVEK;EFNAETFTFHADICTLSEK;VFDEFKPLVEEPQNLIK;DVFLGMFLYEYAR
ANXA5	annexin A5	10	4.01	SP	+	+	+	Gr II and Gr III	GAGTDDHTLIR;LYDAYELK;VLTEIIASR;GTVTDFPGFDER;DLLDDLKSELTGK;SEIDLFNIR;NFATSLYSMIK;GLGTDEESILTLLTSR;ETSGNLEQLLLAVVK;SIPAYLAETLYYAMK
APOA1	apolipoprotein A-I preproprotein	10	2.53	SP	+	+	+	Gr III only	ATEHLSTLSEK;THLAPYSDELR;DYVSQFEGSALGK;WQEEMELYR;DLATVYVDVLK;VKDLATVYVDVLK;LLDNWDSVTSTFSK;VSFLSALEEYTK;QGLLPVLESFK;EQLGPVTQEFWDNLEK
APOB	apolipoprotein B precursor	7	2.25	SP	+	+	+	Gr III only	YGMVAQVTQTLK;ALYWVNGQVPDGVSK;TGISPLALIK;SVSDGIAALDLNAVANK;LAAYLMLMR;VIGNMGQTMEQLTPELK;GFEPTLEALFGK;IEGNLIFDPNNYLPK;QVLFLDTVYGNCSTHFTVK;TTLTAFGFASADLIEIGLEGK
APOD	apolipoprotein D precursor	4	2.61	SP	+	+	+	Gr II only	MTVTDQVNCPK;KMTVTDQVNCPK;IPTTFENGR;CPNPPVQENFDVNK;NILTSNNIDVK;CIQANYSLMENGK;NPNLPPETVDSLK;ADGTVNQIEGEATPVNLTEPAK;CPNPPVQENFDVNKYLGR;WYEIEKIPTTFENGR
APOE	apolipoprotein E precursor	7	2.52	SP	+	+	+	Gr II and Gr III	LAVYQAGAR;LGADMEDVCGR;ALMDETMK;LGPLVEQGR;VQAAVGTSAAPVPSDNH;AATVGSLAGQPLQER;WELALGR;SELEEQLTPVAEETR;GEVQAMLGQSTEELR;SWFEPLVEDMQR
ATP1A3	sodium/potassium-transporting ATPase subunit alpha-3	19	0.44	TM	+	+		Gr II and Gr III	VDNSSLTGESEPQTR;VAEIPFNSTNK;LIIVEGCQR;LNIPVSQVNPR;VIMVTGDHPITAK;NLEAVETLGSTSTICSDK;NMVPQQALVIR;GVGIISEGNETVEDIAAR;QGAIVAVTGDGVNDSPALK;QAADMILLDDNFASIVTGVEEGR
BCAN	brevican core protein isoform 1 precursor	6	2.19	SP, TM		+	+	Gr II only	CLCLPGYGGDLCDVGLR;FCNPGWDAFQGACYK;SWEEAETQCR;WEAPQISCVPR;CEVQHGIDDSSDAVEVK;LRYEVDTVLR;EACYGDMDGFPGVR;ELEAPSEDNSGR;YAFSFSGAQEACAR;ALHPEEDPEGR
C3	complement component 3 precursor	26	2.22	SP	+		+	Gr III only	HQQTVTIPPK;VPVAVQGEDTVQSLTQGDGVAK;TIYTPGSTVLYR;IPIEDGSGEVVLSR;LVAYYTLIGASGQR;IHWESASLLR;AGDFLEANYMNLQR;SNLDEDIIAEENIVSR;ILLQGTPVAQMTEDAVDAER;VQLSNDFDEYIMAIEQTIK
CADM3	cell adhesion molecule 3 isoform 2	5	0.38	TM, SP		+	+	Gr II and Gr III	SDSGTYGCTATSNMGSYK;EDDGASIVCSVNHESLK;GNPVPQQYLWEK;GSDDAPDADTAIINAEGGQSGGDDKKEYFI;DHEDSSLQWSNPAQQTLY;IEVLYTPTAMIRPD;DHEDSSLQWSNPAQQTLYFGEK;SLVTVLGIPQKPIITGYK;IQLVTSTPHELSISISNVALADEGEYTCSIFTMPVR
CALR	calreticulin precursor	11	2.66	SP	+	+	+	Gr II only	HEQNIDCGGGYVK;IKDPDASKPEDWDER;KVHVIFNYK;VHVIFNYK;EQFLDGDGWTSR;GQTLVVQFTVK;DMHGDSEYNIMFGPDICGPGTK;FYALSASFEPFSNK;IDNSQVESGSLEDDWDFLPPKK;IDNSQVESGSLEDDWDFLPPK
CALU	calumenin isoform a precursor	3	2.46	SP	+		+	Gr II only	VHHEPQLSDK;HLVYESDQNKDGK;HLVYESDQNK;MADKDGDLIATK;TFDQLTPEESK;WIYEDVER;DIVVQETMEDIDK;DWILPSDYDHAEAEAR;EEFTAFLHPEEYDYMK;EEIVDKYDLFVGSQATDFGEALVR
CANX	calnexin precursor	11	2.18	SP, TM	+		+	Gr II only	KPEDWDERPK;AEEDEILNR;TPELNLDQFHDK;IPDPEAVKPDDWDEDAPAK;TPYTIMFGPDK;GTLSGWILSK;IVDDWANDGWGLK;APVPTGEVYFADSFDR;IPNPDFFEDLEPFR;KIPNPDFFEDLEPFR
CD14	CD14 antigen precursor	3	3.76	SP	+	+	+	Gr III only	ATVNPSAPR;GLMAALCPHK;TTPEPCELDDEDFR;LKELTLEDLK;FPAIQNLALR;VLSIAQAHSPAFSCEQVR;LTVGAAQVPAQLLVGALR;AFPALTSLDLSDNPGLGER;SWLAELQQWLKPGLK;ITGTMPPLPLEATGLALSSLR
CD200	CD200 antigen isoform a precursor	3	0.42	TM, SP		+		Gr II and Gr III	CSLQNAQEALIVTWQK;AVSPENMVTFSENHGVVIQPAYK;EVICQVLHLGTVTDFK;PENMVTFSENHGVVIQPAYK

CDH13	cadherin 13 preproprotein	2	0.45	SP	+		+	Gr III only	QQTPDKPSPN;INENTGSVSVTR;TLFVHAR;EDLDCTPGFQQK;YEVSSPYFK;VNSDGGLVALR;DIQGSLQDIFK;MTAFDADDPATDNALLR;SIVVSPILIPENQR;GDIVTVVSPALLDR
CFB	complement factor B preproprotein	2	2.40	SP	+	+		Gr III only	QLNEINYEDHK;VSEADSSNADWVTK;FLCTGGVSPYADPNTCR;YGLVTYATYPK;AIHCPRPHDFENGEYWPR;SPYYNVSDEISFHCYDGYTLR;EAGIPEFYDYDVALIK;FIQVGVISWGVVDVCK;IVLDPSGSMNIYLVLDGSDSIGASNFTGAK;DFHINLFQVLPWLK
CP	ceruloplasmin precursor	6	2.25	SP	+	+	+	Gr III only	EVGPTNADPVCLAK;QSEDSTFYLGER;ETFTYEWTVPK;GAYPLSIEPIGVR;ALYLQYTDETFR;DIFTGLIGPMK;ENLTAPGSDSAVFFEQGTTR;DIASGLIGPLILCK;DLYSGLIGPLIVCR;DIASGLIGPLIICK
DKK3	dickkopf-related protein 3 precursor	2	0.45	SP	+	+	+	Gr II only	VGNNTIHVHR;EVEELMEDTQHK;SAVEEMEAEEAAAK;DCQPGLCCAFQR;SLTEEMALR;DQDGEILLPR;EPAAAAAALLGGEEI;EVPDEYEVGSFMEEVR;ITNNQTGQMVFSETVITSVGDEEGR;LLDLITWELEPDGALDR
EGFR	epidermal growth factor receptor isoform a precursor	7	2.04	TM, SP	+	+	+	Gr II and Gr III	IICAQQCSGR;NYVVTDHGSCVR;NLQEILHGAVR;VLGSGAFGTVYK;NLCYANTINWK;IPLENLQIIR;FSNNPALCNVESIQWR;TDLHAFENLEIIR;LTQLGTFEDHFLSLQR;TIQEVAGYVLIALNTVER
ERAP1	endoplasmic reticulum aminopeptidase 1 isoform b precursor	6	2.01	SP	+	+	+	Gr II only	QTWTDEGSVSER;VSVYAVPDK;DMNEVETQFK;ILASTQFEPTAAR;HLAISNMPLVK;GFPLITITVR;ASLINNAFQLVSIGK;TQEFPQILTLIGR;NPVGYPLAWQFLR;TDVLILPEEVEWIK
FN1	fibronectin 1 isoform 6 preproprotein	4	2.01	SP	+	+		Gr III only	FTNIGPDTMR;VPGTSTSATLTGLTR;SYTITGLQPGTDYK;IYLYTLNDNAR;DLQFVEVTDVK;SSPVVIDASTAIDAPSNLR;NLQPASEYTVSLVAIK;LDAPTNLQFVNETDSTVLVR;NTFAEVTGLSPGVTYYFK;VTWAPPPSIDLTNFLVR
GGH	gamma-glutamyl hydrolase precursor	3	2.10	SP	+	+	+	Gr II only	YLESAGAR;NLDGISHAPNAVK;YYIAASYVK;YPVYGVQWHPEK;FFNVLTTNTDGK;IEFISTMEGYK;KPIIGILMQK;LDLTEKDYEILFK;SINGILFPGGSVDLR;TAFYLAEFFVNEAR
GSN	gelsolin isoform b	10	2.77	SP	+	+	+	Gr II and Gr III	DSQEEEKTEALTSAK;YIETDPANR;TGAQELLR;HVVPNEVVVQR;AGALNSNDAFVLK;TPSAAYLWVGTGASEAEK;QTQVSVLPEGGETPLFK;AQPVQVAEGSEPDGFWEALGGK;VSNGAGTMSVSLVADENPFAQGALK;EVQGFESATFLGYFK
HLA-DRA	major histocompatibility complex, class II, DR alpha precursor	4	2.44	TM, SP	+		+	Gr III only	NOT PRESENT IN THE DATABASE
HP	haptoglobin isoform 1 preproprotein	10	2.91	SP	+	+	+	Gr II and Gr III	TEGDGVYTLNNEK;ILGGHLDAK;VGYVSGWGR;GSFPWQAK;VTSIQDWVQK;SCAVAEYGVYVK;NLFLNHSENATAK;DIAPTLTLYVGK;YVMLPVADQDQCIR;MVSHHNLTTGATLINEQWLLTTAK
HPX	hemopexin precursor	6	2.29	SP	+	+	+	Gr III only	GGYTLVSGYPK;QGHNSVFLIK;NFPSPVDAAFR;GECQAEGVLFFQGDR;YYCFQGNQFLR;SGAQATWTELPWPHEK;EVGTPHGIILDSVDAAFICPGSSR;EWFWDLATGTMK;LYLVQGTQVYVFLTK;CSDGWSFDATTLDDNGTMLFFK
HSP90B1	endoplasmin precursor	14	2.43	SP	+	+	+	Gr II only	LGVIEDHSNR;GLFDEYGSK;ELISNASDALDK;DDEVDVDGTVEEDLGK;GVVDSDDLPLNVSR;ELISNASDALDKIR;SILFVPTSAPR;VFITDDFHDMMPK;EFEPLLNWMK;YSQFINFPIYVWSSK
HSPA5	78 kDa glucose-regulated protein precursor	18	2.36	SP	+	+	+	Gr II only	VEIIANDQGNR;VEILANDQGNR;NQLTSNPENTVFDAK;IINEPTAAAIAY;ITPSYVAFTPEGER;SQIFSTASDNQPTVTIK;IINEPTAAAIAYGLDK;FEELNMDLFR;TFAPEEISAMVLTK;GVPQIEVTFEIDVNGILR
ICAM5	intercellular adhesion molecule 5 precursor	6	0.28	TM, SP	+	+		Gr II and Gr III	TFSLSPDAPR;LFSCEVDGKPQPSVK;GAVLTATVLAR;TVTVGVEYRPVVAE;ASLTLTLLR;CVGSGGATEGVLLPLAPPDPSPR;SGELGAVIEGLLR;SDGGAVLALGLLGPVTR;LLEVGSERPVSCTLDGLFPASEAR
IGSF8	immunoglobulin superfamily, member 8	7	0.45	TM, SP	+	+	+	Gr II and Gr III	VVAGEVQVQR;LQGDAVVLK;STLQEVVGIR;SDLAVEAGAPYAER;DTQFSYAVFK;PDVLQVSAAPPGPR;VLPDVLQVSAAPPGPR;REVLVPEGPLYR;HAAYSVGWEMAPAGAPGPGR;LVAQLDTEGVGSLGPGYEGR
ITGA6	integrin alpha-6 isoform b precursor	3	3.20	SP, TM	+		+	Gr II only	SRPVINIQK;LETTSNQDNLAPITAK;FSLFAER;AFIDVTAAAENIR;LNYLDILMR;NIGDINQDGYPDIAVGAPYDDLGK;LIATFPDTLTYSAYR;VNSLPEVLPILNSDEPK;DGWQDIVIGAPQYFDR;NSYPDVAVGSLSDSVTIFR
ITGB1	integrin beta-1 isoform 1A precursor	3	2.34	SP, TM	+	+	+	Gr II only	SAVTTVVNPK;FCECDNFNCDR;LSEGVTISYK;SGEPQTFTLK;TVMPYISTTPAK;GEVFNELVGK;SLGTDLMNEMR;IGFGSFVEK;LKPEDITQIQPQQLVLR;LSENNIQTIFAVTEEFQPVYK
ITIH2	inter-alpha globulin inhibitor H2 polypeptide	2	4.62	SP	+	+	+	Gr III only	LSNENHGIAQR;MATTMIQSK;TEVNVLPGAK;SSALDMENFR;VQFELHYQEVK;FYNQVSTPLLR;GAFISNFSMTVDGK;VVNNSPQPQNVVFDVQIPK;ETAVDGELVVLYDVK;HLEVDVWVIEPQGLR
L1CAM	L1 cell adhesion molecule isoform 3 precursor	14	0.37	TM, SP	+		+	Gr II and Gr III	VKDATQITQGPR;DETFGEYSDNEEK;CEASGKPEVQFR;CLAENSLGSAR;EAAGGNDSSGATSPINPAVALE;YGPGEPSPVSETVVTPEAAPEK;AQLLVVGSPGPVPR;LVVFPTDDISLK;LVLSDLHLLTQSQVR;RLVVFPTDDISLK
LGALS3BP	galectin-3-binding protein	4	3.13	SP	+	+	+	Gr II and Gr III	LADGGATNQGR;ASHEEVEGLVEK;AVDTWSWGER;YSSDYFQAPSDYR;ELSEALGQIFDSQR;TVIRPFYLTNSSGVD;SDLAVPSELALLK;IYTSPTWSAFVTDSSWSAR;GQWGTVCDNLWDLTDASVVCR;TLQALEFHTVPFQLLAR

LMAN2	vesicular integral-membrane protein VIP36 precursor	4	2.37	SP, TM	+	+	+	Gr II only	DITDGNSEHLK;DNVDDPTGNFR;NCIDITGVR;NRDHDTFLAVR;DHDTFLAVR;DRLVPGPVFGSK;WTELAGCTADFR;NLHGDGIALWYTR;DNFHGLAIFLDTYPNDETTER;LFQLMVEHTPDEESIDWTK
NFASC	neurofascin isoform 4 precursor	8	0.40	SP, TM		+		Gr II only	DDEPLYIGNR;GNPAPSFHWTR;DLELTDLAER;DQGSYTCVASTELDQDLAK;SGTLVIDFR;VIAINEVGSSHPSLPSER;ITNVSEEDSGEYFCLASNK;YVVGQTPVYVPYEIR;LDCPFFGSPIPTLR;TRLDCPFFGSPIPTLR
NUCB2	nucleobindin-2 precursor	4	2.24	SP, TM	+		+	Gr II only	AATSDLEHYDK;VQNIHPVESAK;VPIDIDKTK;NEEDDMVEMEEER;ELDLVSHHVR;LEYHQVIQQMEQK;QVIDVLETDKHFR;LVTLEEFLK;EVWEETDGLDPNDFDPK;LHDVNSDGFLDEQELEALFTK
P4HB	protein disulfide-isomerase precursor	15	2.58	SP	+	+	+	Gr II only	MDSTANEVEAVK;YKPESEELTAER;VDATEESDLAQQYGVR;NFEDVAFDEK;LITLEEEMTK;DAPEEEDHVLVLR;EADDIVNWLK;THILLFLPK;ILFIFIDSDHTDNQR;HNQLPLVIEFTEQTAPK
PAM	peptidylglycine alpha-amidating monooxygenase isoform c preproprotein	2	0.48	TM, SP	+	+	+	Gr II and Gr III	GDHVWDGNSFDSK;NGQWTLIGR;TIPPEANIPIPVK;ANILYAWAR;NLFYLPHGLSIDK;REEEEVLDQGDFYSLLSK;IPVDEEAFVIDFKPR;YFVLQVHYGDISAFR;QSPQLPQAFYPVGHPVDVSFGDLLAAR;NLFYLPHGLSIDKDGNYWVTDVALHQVFK
PCDH1	protocadherin 1 isoform 1 precursor	2	0.50	TM, SP		+	+	Gr III only	VTVLDTNDNAPK;DMNDNAPTIEIR;FNLMSDAPGDSPR;NTGLITVQGPVDR;TGDIFTTETSIDR;SNSPLPSIQLQPQSPSASK;WDSYDLTIK;IHLPLNYPPGSPDLGR;YFLQTTTPLDYEK;GLFTISPETGEIQVK
PDIA3	protein disulfide-isomerase A3 precursor	13	2.24	SP	+		+	Gr II only	DGEEAGAYDGPR;LAPEYEAAATR;YGVSGYPTLK;MDATANDVPSPYEVR;FVMQEEFSR;GFPTIYFSPANK;SDVLELTDDNFESR;ELSDFISYLQR;DLLIAYYDVDYEK;DASIVGFFDDSFSEAHSEFLK
PDIA4	protein disulfide-isomerase A4 precursor	9	3.15	SP	+		+	Gr II and Gr III	MDATANDVPSDR;VDATAETDLAK;IDATSASVLASR;FDVSGYPTIK;FDVSGYPTLK;RFDVSGYPTLK;YGIVDYMIEQSGPPSK;VEGFPTIYFAPSGDK;FAMEPEEFDSDTLR;EVSQPDWTPPPEVTLVLTK
PDIA6	protein disulfide-isomerase A6 precursor	5	2.49	SP	+		+	Gr II only	GESPVDYDGGR;LAAVDATVNQVLASR;NLEPEWAAAASEVK;GSTAPVGGGAFPTIVER;NSYLEVLLK;TGEAIVDAALSALR;GSFSEQGINEFLR;LYSSSDDVIELTPSNFNR;DGELPVEDDIDLSDVELDDLGKDEL;ALDLFSDNAPPPELLEIINEDIAK
PLG	plasminogen isoform 1 precursor	2	2.03	SP	+	+	+	Gr III only	QLGAGSIEECAAK;EQQCVIMAENRK;EQQCVIMAENR;EAQLPVIENK;HSIFTPETNPR;NPDGDVGGPWCYTTNPR;LDDYVNTQGASLFSVTK;FVTWIEGVMR;YILQGVTSWGLGCAR;EPLDDYVNTQGASLFSVTK
PPIB	peptidylprolyl isomerase B precursor	7	2.97	SP	+	+	+	Gr II and Gr III	HVVFGK;VLEGMEVVR;HYGPGWVSMANAGK;DKPLKDVIIADCGK;IEVEKPFAIAK;TVDNFVALATGEK;IEVEKPFAIAKE;DTNGSQFFITTVK;DFMIQGGDFTR;VIKDFMIQGGDFTR
PRCP	prolylcarboxypeptidase isoform 1 preproprotein	3	2.87	SP	+		+	Gr III only	SGPHCSESIHR;VDHFGFNTVK;AMLVFAEHR;YLVADKYWK;ELSDDCFQQWGVR;PSWITTMYGGK;YYGESLPFGDNSFK;NALDPMSVLLAR;DITDTLVAVTISEGAHHLDLR;HLNFLTSEQALADFAELIK
PRKCSH	glucosidase 2 subunit beta isoform 2 precursor	5	2.06	SP	+	+	+	Gr II only	ETMVTSTTEPSR;YEQGTGCWQGPNR;ESLQQMAEVTR;SLKDMEESIR;AQQEQELAADAFK;LWEEQLAAAK;SLEDQVEMLR;MPPYDEQTQAFIDAAQEAR;SEALPTDLPAPSAPDLTEPK;LGGSPTSLGTWGSWIGPDHDK
PRRT3	proline-rich transmembrane protein 3 precursor	2	0.33	TM, SP			+	Gr III only	AELDDAGSSLLR;AVAGALGLLASGLQLAAALWLYPG
PTPRN	protein tyrosine phosphatase, receptor type, N precursor	3	0.44	TM, SP		+		Gr II and Gr III	LILSSR;SCPIIVHCSDGAGR;SELEAQTGLQILQTGVGQR
RNF170	ring finger protein 170 isoform c	16	0.50	TM, SP		+		Gr III only	EQLQTEQDAPAATR;YQGEVQSLK;NVHQNIHPENQELVR;VLREQLQTEQDAPAATR;YGSWLGAISCPICR
S100A10	protein S100-A10	2	2.75	TM	+		+	Gr III only	DPLAVDK;FAGDKGYLTK;PSQMEHAMETM;EFPGFLENQK;EFPGFLENQKD;PGFLENQKDPLAVDK;PSQMEHAMETMMF;EFPGFLENQKDPLAVDK;PSQMEHAMETMMFTFHK;PSQMEHAMETMMFTF
SERPINA1	serine proteinase inhibitor, clade A, member 1 precursor	11	2.47	SP	+	+	+	Gr III only	DTEEEDFHVDQVTTVK;LSITGTYDLK;SVLGQLGITK;LYHSEAFTVNFGDTEEAK;VFSNGADLSGVTEEAPLK;LSSWVLLMK;ITPNLAEFAFSLYR;YLGNATAIFFLPDEGK;TLNQPDSQLQLTTGNGLFLSEGLK;GTEAAGAMFLEAIPMSIPPEVK
SIRPA	tyrosine-protein phosphatase non-receptor type substrate 1 precursor	8	0.38	SP, TM	+	+		Gr II only	MEPAGPAPGR;LTCQVEHDGQPAVSK;SGAGTELSVR;AKPSAPVVSGPAAR;KGSPDDVEFK;EEELQVIQPDK;NNMDFSIR;VPPTLEVTQQPVR;SVLVAAGETATLR;EITQDTNDITYADLNLPK
SLC3A2	4F2 cell-surface antigen heavy chain isoform f	12	0.46	SP, TM	+	+	+	Gr II only	VAEDEAEAAAAAK;GLVLGPIHK;EDFDSLLQSAK;GENSWFSTQVDTVATK;VILDLTPNYR;GQSEDPGSLLSLFR;IGDLQAFQGHGAGNLAGLK;LKLEPHEGLLLR;DDVAQTDLLQIDPNFGSK;LLTSFLPAQLLR
SPARC	SPARC precursor	5	2.45	SP	+	+	+	Gr II and Gr III	DEDNNLLTEK;APLIPMEHCTTR;FFETCDLDNDK;LHLDYIGPCK;TFDSSCHFFATK;LEAGDHPVELLAR;NVLVTLYER;RLEAGDHPVELLAR;NVLVTLYERDEDNNLLTEK;YIALDEWAGCFGIK
TF	transferrin precursor	23	2.23	SP	+	+	+	Gr III only	HSTIFENLANK;SASDLTWDNLK;CSTSSLLEACTFR;FDEFFSEGCAPGSK;EDPQTFYYAVAVVK;MYLGYEYVTAIR;TAGWNIPMGLLYNK;QQQHLFGSNVTDCSGNFCLFR;IMNGEADAMSLDGGFVYIAGK;SAGWNIPIGLLYCDLPEPR
THY1	thy-1 membrane glycoprotein preproprotein	4	0.48	SP	+	+	+	Gr II only	SLTACLVDQSLR;HENTSSSPIQYEFSLTR;VLYLSAFTSK;TSLTACLVDQSLR;VTSLTACLVDQSLR;QKVTSLTACLVDQSLR;HVLFGTVGVPEHTYR;HVLFGTVGVPEHTY;KHVLFGTVGVPEHTYR;YNMKVLYLSAFTSK

TMED4	transmembrane emp24 domain-containing protein 4 precursor	3	2.17	SP, TM		+		Gr II only	LTSESTNQR;QLLDQVEQIQK;LYFHIGETEK;DKLTELQLR;QLLDQVEQIQKEQDYQR;VHLDIQVGEHANNYPEIAAK;EVFLPSTPGLGMHVEVKDPDGK;EVFLPSTPGLGMHVEVK;CFIEEIPDETMVIGNYR;RCFIEEIPDETMVIGNYR
TNC	tenascin precursor	9	2.81	SP	+		+	Gr II and Gr III	APTAQVESFR;FTTDLDSPR;LDAPSQIEVK;DHGETAFAVYDK;ETFTTGLDAPR;VSQTDNSITLEWR;LEELENLVSSLR;GLEPGQEYNVLLTAEK;ELEPGVEYFIR;WQPAIATVDSYVISYTGEK
TPP1	tripeptidyl-peptidase 1 preproprotein	4	2.22	SP	+	+	+	Gr II only	PSYQEEAVTK;HLGVTPSVIR;LFGGNFAHQASVAR;LYQQHGAGLFDVTR;LSELVQAVSDPSSPQYGK;LHLGVTPSVIR;ILSGRPPLGFLNPR;AYPDVAALSDGYWVVSNR;GLTLLFASGDSGAGCWSVSGR;LITNEIVDYISGGGFSNVFPR
